# Supplementary material for: Mining and quantitative evaluation of COVID-19 policy tools in China
Source: PLoS One. 2023 Apr 7;18(4):e0284143. doi: 10.1371/journal.pone.0284143 (PMC10081750; doi:10.1371/journal.pone.0284143)
Supplement: S1 Appendix — (DOCX) [file pone.0284143.s001.docx]

**新冠肺炎疫情相关政策**

1. 国务院：应对新型冠状病毒感染肺炎疫情联防联控机制关于加强口岸城市新冠肺炎疫情防控工作的通知

国办发明电〔2021〕14号

各省、自治区、直辖市及新疆生产建设兵团应对新型冠状病毒感染肺炎疫情联防联控机制（领导小组、指挥部），国务院应对新型冠状病毒感染肺炎疫情联防联控机制各成员单位，国家疾控局：

近期我国发生多起本土聚集性疫情，均是境外疫情经口岸城市输入，暴露出一些地方疫情监测预警不及时、跨境货车司机和冷链等高风险岗位人员闭环管理不落实、定期核酸检测流于形式、冷链物品各环节消杀不到位、集中隔离场所管理不规范、疫情应急处置不够科学精准、民生保障存在薄弱环节等问题。为深入贯彻党的十九届六中全会精神和习近平总书记重要指示，落实党中央、国务院决策部署，加快补齐口岸城市防控短板弱项，毫不放松、科学精准做好防控工作，织密扎牢外防输入防线，统筹好疫情防控和经济社会发展，现通知如下。

一、完善口岸城市疫情防控机制。各口岸城市要学习借鉴上海市建立空港管理委员会的经验，在本地联防联控机制（领导小组、指挥部）框架下，建立由1名负责同志牵头，卫生健康、疾控、海关、移民边检、民航、交通运输等部门和单位参与的口岸防控专班，落实属地责任，明确各环节职责分工和责任人，统筹各方力量做好疫情防控工作。有条件的口岸城市可研究在辖区内设置疫情防控缓冲区，缓冲区内实行相对严格的管控措施，缓冲区外落实好疫情常态化防控要求。

二、健全疫情监测预警体系。各口岸城市要密切跟踪相关国家和地区疫情走势，整合各方面对入境人员、入境物品、重点场所环境开展核酸检测的数据，加强阳性结果分析。对来自疫情严重国家和地区的人员和物品，相关方面要及时按规定采取加密核酸检测、限制入境流量、航班熔断等措施。对入境口岸通道、隔离场所、定点医院、冷链相关企业等高风险岗位人员每隔1天开展1次核酸检测，对其他工作人员每周开展2次核酸检测，对工作人员家属每周开展1次核酸检测，均纳入当地应检尽检、免费检测范围；可根据当地疫情风险，适当扩大重点区域和人员核酸筛查范围。坚持人员、物品、环境同监测，建立健全多点触发的监测预警网络，重点加强药店、基层诊所、民营医院管理，强化首诊报告责任。对有发热、干咳、乏力、嗅觉味觉减退等症状患者和呼吸科就诊患者，陆地边境口岸城市医疗卫生机构要将核酸检测列为必查项目。

三、落实边境管控措施。陆地边境口岸城市要督促跨境运输企业落实“人货分离、分段运输”的要求，实行甩挂、接驳、吊装等非接触式货物交接模式；确需入境的驾驶员应全程不下车、当日返回，不得与境内人员接触；严格做好入境人员“点对点、一站式”转运工作。加大对非法出入境活动的打击力度，加快推进陆地边境、口岸两翼物理拦阻设施建设，强化东北方向界江界河封冻期联防联控，严防非法越境输入疫情。坚持非必要不登轮、不登陆、不搭靠，减少国际航行船舶登轮人员数量，确需登轮的人员要避免与船员接触并做好个人防护。对船舶上发现的核酸检测阳性人员，各口岸城市要按规定接收并及时转运至定点医疗机构，未转运或实施终末消毒前，不得采取登轮作业的方式装卸货物。海关、公安、海警、市场监管等部门要强化协调联动，按职责加大主动打击冻品走私的力度。

四、加强高风险岗位人员防控。各口岸城市要排查明确本地直接接触入境人员、物品、环境的高风险岗位人员范围，督促相关单位登记造册，落实规范防护、闭环管理、高频次核酸检测、每日健康监测零报告等措施；相关人员闭环管理期间，不得与非闭环管理人员接触。推广“14+7+7”（14天封闭管理作业+7天集中隔离医学观察+7天居家健康监测）的做法，做好轮班工作，各地可结合实际对封闭管理作业时间作适当调整。对实行轮班制后一线工作人员不足的地区，有关省份要统筹力量予以支持。

五、严格人员流动管控。从各省（自治区、直辖市）确定口岸城市范围并公告之日起至2022年3月15日，离开陆地边境口岸城市（与香港、澳门有口岸相连的除外）人员需持有48小时内核酸检测阴性证明，前往陆地边境口岸城市人员抵达后至少进行1次核酸检测。加强社会责任和法律义务提醒，发热患者、健康码“黄码”等人员核酸检测未出结果前不流动、不聚集。陆地边境口岸城市要做好旅游限流、风险提示等工作。文化和旅游部门要按照“谁组织、谁负责”的原则，督促旅行社加强对团队游人员的核酸检测。

六、加强进口冷链食品等风险防范。进口冷链食品入境量较大的口岸城市要发挥政府主导作用，建设集中监管仓，对进口冷链食品入库统一消杀、统一检测。海关、交通运输、市场监管、疾控等部门要按职责督促相关企业落实进口冷链食品入境、仓储、生产、加工、运输、销售等各环节疫情防控要求，依托信息化手段推动全链条追溯。各口岸城市口岸防控专班协调推动有关方面对入境航班乘客托运和手提行李做好消毒工作。引导公众提高防护意识，尽可能减少从疫情严重国家和地区邮购、快递物品，邮政、快递企业要切实做好物品消毒工作。各省（自治区、直辖市）要抓紧组织本辖区口岸城市开展一次冷链相关企业风险排查，全面评估和整改各环节风险漏洞，并于2021年12月15日前将排查整改情况报送国务院联防联控机制综合组。

七、提升疫情防控和处置能力。各省（自治区、直辖市）要统筹资源和力量，支持防控任务重的口岸城市加强疾控机构、定点医院、发热门诊、基层医疗卫生机构、集中隔离点等建设，提高流调溯源、基因测序、核酸检测、集中隔离、医疗救治等能力，确保落实“应检尽检、应隔尽隔、应治尽治”的要求；对口岸通道、货场、道路、机场等基础设施及时进行升级改造，满足口岸卫生检疫、边防检查、人员闭环管理、入境货物分段运输等防疫要求。口岸城市要加强组织，尽可能提高新冠病毒疫苗全程接种和加强免疫接种覆盖率。卫生健康、疾控部门要会同药监等部门建立对核酸检测机构的评价、监管机制，开展检查抽查，依法严惩弄虚作假等违法违规行为，确保核酸检测质量可靠、结果真实。科学精准做好集中隔离、协查管控、赋码管理、区域封控等工作，避免“一刀切”。加强各省（自治区、直辖市）和各口岸城市的疫情防控信息化支撑，通过建立完善口岸防控、边境管控、冷链防控等数据闭环，支撑对入境人员、货物等的“人”、“物”同防和闭环管理，提升区域协查的及时性和精准度。

八、做好民生保障工作。各口岸城市要将疫情防控与民生保障工作同步研究、同步部署，公布并畅通民生保障热线，定期排查基本生活物资供应、价格等方面的苗头隐患，及时解决和主动回应群众反映的突出问题。加强市场监管，严厉打击哄抬物价、囤积居奇等行为。做好困难群众救助工作，保障群众基本生活。加强宣传引导，争取广大群众对防疫措施的理解和支持，动员群众积极参与创建无疫小区、无疫村。有关省份要结合实际加大对口岸城市的支持力度，及时帮助解决民生保障方面的实际困难。

九、加强组织实施和监督检查。做好口岸城市疫情防控工作关键在落实，落实的关键在细节。各省（自治区、直辖市）、各有关部门要把加强口岸城市疫情防控作为当前一项重要政治任务，对照本通知要求，抓紧制定本省（自治区、直辖市）、本部门实施方案，周密组织实施；要将防控措施落实情况融入本地区本行业日常监督和执法检查，发现问题限期整改。要指导口岸城市按照“一地一方案”的要求，制定具体工作方案，落实各项政策措施。口岸防控专班每周将口岸相关人员、物品、环境核酸检测以及高风险岗位人员闭环管理等各项重点防控措施的落实情况，上报所在地联防联控机制（领导小组、指挥部）。卫生健康、疾控部门要强化卫生监督执法，加强对重点场所、重要环节的监督。国务院联防联控机制综合组要会同有关部门，指导有关省份针对边境口岸防控、高风险岗位人员闭环管理、核酸检测质量和效率、隔离点管理等开展专项督查。要健全疫情防控责任制，对履职不力、失职渎职的，依法依规追究责任。

本通知所指的口岸城市包括开放的陆路、水路、航空口岸所在县（市、区、旗）、所在市（地、州、盟）及所在直辖市，具体范围由各省（自治区、直辖市）根据疫情防控需要确定并及时公告。

国务院应对新型冠状病毒感染肺炎

疫情联防联控机制

2021年11月25日

1. 教育部 发展改革委 财政部 卫生健康委：市场监管总局关于全面加强和改进新时代学校卫生与健康教育工作的意见 教体艺〔2021〕7号

各省、自治区、直辖市教育厅（教委）、发展改革委、财政厅（局）、卫生健康委、市场监管局（厅、委），新疆生产建设兵团教育局、发展改革委、财政局、卫生健康委、市场监管局：

加强新时代学校和幼儿园（以下统称学校）卫生与健康教育工作，是全面推进健康中国建设的重要基础，是加快推进教育现代化、建设高质量教育体系和建成教育强国的重要任务，是大力发展素质教育、促进学生全面发展的重要举措。为深入贯彻落实习近平总书记关于教育、卫生健康的重要论述和全国教育大会精神，把新时代学校卫生与健康教育工作摆在更加突出位置，提升学生健康素养，为学生健康成长和终身发展奠定基础，现就全面加强和改进新时代学校卫生与健康教育工作提出如下意见。

一、总体要求

1．指导思想。以习近平新时代中国特色社会主义思想为指导，全面落实党的十九大和十九届二中、三中、四中、五中全会精神，全面贯彻党的教育方针，牢记为党育人、为国育才使命，落实立德树人根本任务，坚持健康第一的教育理念，把全面提升学生健康素养纳入高质量教育体系，作为学校教育重要目标和评价标准，深化学校健康教育改革，夯实学校卫生条件保障，构建高质量学校卫生与健康教育体系，促进学生身心健康、养成健康生活方式，培养德智体美劳全面发展的社会主义建设者和接班人。

2．基本原则

——坚持健康第一。教育学生树牢“每个人是自己健康第一责任人”理念，学会和掌握健康知识与技能，为人人终身健康、建成健康中国奠定基础。

——坚持面向全体。将健康教育与德育、智育、体育、美育、劳动教育相结合，融入教育教学、管理服务全过程，发挥学校卫生专业技术人员、体育与健康课教师和教职员工等全员育人作用，构建面向人人、人人有责的健康教育体系。

——坚持预防为主。树立大卫生、大健康观念，普及健康知识，优化健康服务，完善健康保障，引导树立正确健康观，以防病为中心向以健康促进为中心转变。

——坚持问题导向。着力破解制约学校卫生与健康教育发展的突出问题、影响学生健康的重点问题，因地制宜，深化改革，综合施策。

3．工作目标

2025年，政府主导、部门协作、学校实施、社会参与的新时代学校卫生与健康教育工作格局更加完善。学校健康教育时间切实保证，健康教育教学效果明显提升。办学条件达到国家学校卫生基本标准。学校应对突发公共卫生事件预测研判、精准管控、应急处置等能力显著增强。学生健康素养普遍提高，防病意识和健康管理能力显著增强，体质健康水平明显提升。

2035年，学校卫生条件、体育设施、健康教育和健康素养水平基本实现现代化，达到建成教育强国和健康中国要求，形成高质量的新时代学校卫生与健康教育体系。

二、深化教育教学改革

4．提升学生健康素养。聚焦以健康观念、健康知识、健康方法、健康管理能力等为主要内涵的学生健康素养，促使学生养成良好卫生行为和习惯，保持文明健康、绿色环保生活方式，形成健康文明校园文化。以中小学为重点，注重大中小幼相衔接，完善以课堂教学为主渠道、以主题教育为重要载体、以日常教育为基础的学校健康教育推进机制，健全学生健康素养评价机制，纳入教育评价改革，形成学校全员促进、学生人人健康的良好氛围。

5．明确健康教育内容。构建分学段、一体化健康教育内容体系。修订《中小学健康教育指导纲要》，落实《普通高等学校健康教育指导纲要》。崇尚科学、尊重生命，引导学生主动学习掌握日常锻炼、传染病预防、食品卫生安全、合理膳食、体格检查、心理健康、生长发育、性与生殖健康、心肺复苏、安全避险与应急救护等方面知识和技能。把预防新型毒品等毒品教育纳入健康教育课程。落实预防艾滋病专题教育任务，加强青春期、性道德和性责任教育。开发健康教育教学资源。开展全国学校健康教育示范课与教研交流。

6．落实课程课时要求。完善课程安排，系统设计教学标准、师资配备、评价体系、制度保障，确保各级各类学校将健康教育贯穿教育全过程。鼓励普通高校开设健康教育必修课或选修课，师范类、体育类普通高校应开设健康教育必修课和教法课。落实各学段健康教育教学时间，中小学校每学期应在体育与健康课程总课时中安排4个健康教育课时。

7．拓展健康教育渠道。构建学科教学与实践活动相结合、课内教育与课外教育相结合、经常性宣传教育与集中式宣传教育相结合的健康教育模式。依托“师生健康中国健康”主题健康教育、“中国学生营养日”等重要活动和时间节点，多渠道、多形式向学生、教师和家长开展健康教育。鼓励开展健康知识竞赛、健康技能展示等，每年举办全国学校健康教育成果展示。创新健康教育形式，深化“互联网+健康教育”。广泛开展传染病防治法、突发公共卫生事件应急条例等卫生防疫法律法规教育。鼓励学校建设健康教育体验室、健康教育校长（名师）工作室。支持学生社团、志愿者开展卫生健康知识宣传教育。

8．保障食品营养健康。倡导营养均衡、膳食平衡。学校配备有资质的专（兼）职营养指导人员和食品安全管理人员，开展学生膳食营养监测，实施学生营养干预措施。根据年龄和生长发育特点，为学生提供均衡营养膳食。引导家长科学安排家庭膳食。加强饮食教育，引导学生珍惜粮食、尊重劳动、践行“光盘行动”、读懂食品标签标识，形成健康饮食新风尚。落实《学校食品安全与营养健康管理规定》，严格学校食品安全管理，全面落实学校食品安全校长（园长）负责制，完善学校负责人陪餐制度和家长委员会代表参与学校食品安全监督检查机制。建立学校安全饮用水管理制度，定期开展水质监测。加强校园及周边食品安全综合治理。

9．增加体育锻炼时间。按照教会、勤练、常赛要求，开齐开足体育与健康课，强化学校体育教学、训练，健全体育竞赛和人才培养体系。推广中华传统体育项目，开展全员运动会、亲子运动会。严格落实眼保健操、课间操制度，提倡中小学生到校后先进行20分钟左右的身体活动。保障学生每天校内、校外各1个小时体育活动时间。

10．强化心理健康教育。开展生命教育、亲情教育，增强学生尊重生命、珍爱生命意识。培育学生积极心理品质，保持乐观向上心态，引导学生树立健康理念，自觉维护心理健康，掌握正确应对学业、人际关系等方面不良情绪和心理压力的技能，提高心理适应能力，做到自尊自信、理性平和。加强重大疫情、重大灾害等特殊时期心理危机干预，强化人文关怀和心理疏导。加大学校心理健康人才队伍建设，2022年配备专（兼）职心理健康工作人员的中小学校比例达到80%，2030年达到90%。

11．养成健康行为习惯。保持勤洗手、常通风、分餐制、使用公勺公筷、科学就医用药、不滥食野生动物等日常健康行为和习惯，生活中做好自我防护、保持手卫生。保持规律作息和充足睡眠。健康足量饮水，减少饮料摄入。践行绿色环保理念，减少污染和浪费。

三、夯实卫生工作基础

12．开展爱国卫生运动。弘扬爱国卫生运动精神和伟大抗疫精神，推动新时代校园爱国卫生运动从环境卫生治理向师生健康管理转变。宣传和落实生活垃圾分类要求。推进学校厕所革命，建设、改造学校卫生厕所。加大学校控烟宣传教育力度，建设无烟学校。

13．健全疾病预防体系。巩固深化拓展教育系统新冠肺炎疫情防控成果与经验，全面提升应对突发公共卫生事件能力、应急管理能力、健康管理能力。坚持多病共防，完善疫情防控制度，健全学校突发公共卫生事件信息报告制度、疫情调查制度、健康检查制度、留观隔离制度、医校联防制度，做到早发现、早报告、早隔离、早治疗。预防、控制学生近视、肥胖、脊柱弯曲异常等发生、发展，定期对学生课桌椅高度进行个性化调整。修订《中小学生健康体检管理办法》，将脊柱健康检查纳入中小学生体检项目。加强学生健康体检管理和数据分析应用。

14．实施体质健康监测。每年开展学生体质健康测试，每3年开展一次国家义务教育阶段学生体质健康监测，每5年开展一次全国学生体质健康监测与调研。全面开展学生常见病及健康影响因素监测与干预评价。建设全国学生健康管理信息系统，建立健全学生健康电子档案，与卫生健康系统有关数据互通共享。加强学生健康管理，开展健康评价，为学生制定个性化健康指导方案、发放健康处方。探索建立特异性体质和特殊疾病学生健康管理制度。

15．加强学校急救教育。实施青少年急救教育行动计划，完善学校急救教育标准，加强学校急救设施建设和师生急救教育培训，纳入教育“十四五”规划，从人、财、物等方面予以保障。鼓励开发和应用优质急救技能教育培训课程资源，建设高水平急救培训讲师队伍，加强适龄学生急救培训，在师生中逐步普及急救知识和技能。

16．推进卫生设施建设。按照国家学校体育卫生条件试行基本标准，加强质量认证管理，高水平推进教学卫生、生活设施建设，高质量改善学校办学条件。新建学校的饮水、教室采光和照明、通风换气、采暖、厕所和其他卫生设备，应严格执行最新国家标准。制定高校校医院、中小学卫生室（保健室）配备标准。加强学校卫生标准宣贯及应用。

17．优化组织机构设置。鼓励学校成立健康教育中心，整合校内外资源提升健康教育能力，提供高质量健康教育服务。加强疾控机构学校卫生科所、区域性中小学卫生保健机构、高校校医院和中小学卫生室（保健室）建设，配齐卫生专业技术人员。拓展现有疾控机构、区域性中小学卫生保健机构职能，加强业务指导和技术培训。2022年，中小学配备专职卫生技术人员、专（兼）职保健教师或卫生专业技术人员比例达到70%，2030年达到90%。

18．加大人才培养力度。鼓励具备条件的高校开设健康教育等相关专业，支持高校设立健康教育学院，培养健康教育师资。加大全科医学人才培养力度，探索订单定向免费医学生培养、医疗机构派驻等方式解决校医和健康教育师资配备问题，或通过政府购买服务提供相关服务。实施学校健康教育教师培训计划，加强健康教育师资培训，建立定期轮训制度。把健康教育作为教师继续教育培训重要内容，纳入“国培计划”。把健康教育作为学校卫生专业技术人员、专（兼）职保健教师、健康教育教师、体育教师职前教育和职后培训重要内容。利用大数据、云平台提高校医医疗服务能力和健康教育水平。

19．完善激励保障机制。各地和高校要坚持成果、贡献、业绩导向，完善职称评审制度，实行分类评价，中小学和高校健康教育教师、校医和保健教师按规定参加职称评审。完善校医等学校卫生专业技术人员培养、准入、职称晋升、待遇、评价和激励机制。校医参与学校健康教育、传染病防控、值班值守等计入工作量，依标准核发薪酬。

20．建设专业研究平台。切实发挥全国中小学和高校健康教育教学指导委员会等智库作用。各地和相关高校要加强健康教育教学研究和学科建设，成立健康教育教学等专家组织，服务科学决策，提高学校卫生与健康教育工作专业化、科学化水平。

四、加强组织实施

21．强化组织领导。实施中国青少年健康教育行动计划（2021-2025年）和儿童青少年近视防控光明行动（2021-2025年）。各地要把新时代学校卫生与健康教育工作纳入规划，加强统筹协调，落实工作责任，制定本地区实施方案和五年行动计划。各校要建立校长负总责、分管校领导牵头抓、相关部门保落实的新时代学校卫生与健康教育工作机制。

22．健全协作机制。构建各级教育、卫生健康等部门密切协作的新时代学校卫生与健康教育工作机制，疾病预防控制机构、区域性中小学卫生保健机构等要为学校提供专业指导和技术支持，鼓励选聘医务工作者担任健康副校长。将符合要求的高校校医院纳入当地社区卫生服务网络，提供公共卫生服务与基本医疗保障。将具备医疗机构执业许可证的中小学校医室建设纳入政府公共卫生体系。推广医务托管、医校协同等经验做法，或通过政府购买服务提供学校医务服务。

23．优化发展环境。持续推进健康中国行动中小学健康促进专项行动，实施健康学校建设计划，遴选建设全国健康学校、健康教育改革试验区和试点县（市、区），将健康学校建设相关内容纳入文明城市、健康促进县（区）评价指标体系。大力宣传地方和学校加强学校卫生与健康教育工作典型经验做法，健全学校、家庭、社会协同健康促进机制，营造健康教育环境，培育健康促进文化。

24．完善投入机制。加强新时代学校卫生与健康教育工作经费保障，纳入学校年度预算。鼓励社会资金、公益机构支持学校卫生与健康教育，多渠道增加投入。

25．纳入评价体系。各地要强化考核，把新时代学校卫生与健康教育工作列入政府政绩考核指标、教育部门和学校负责人业绩考核评价指标，纳入学校督导评价体系，加强督导检查。

教　 育　 部　　发展改革委

财　 政　 部　　卫生健康委

市场监管总局

2021年8月2日

1. 国务院办公厅关于进一步加大对中小企业纾困帮扶力度的通知

国办发〔2021〕45号

各省、自治区、直辖市人民政府，国务院各部委、各直属机构：

中小企业是国民经济和社会发展的主力军，在促进增长、保障就业、活跃市场、改善民生等方面发挥着重要作用。近期，受原材料价格上涨、订单不足、用工难用工贵、应收账款回款慢、物流成本高以及新冠肺炎疫情散发、部分地区停电限电等影响，中小企业成本压力加大、经营困难加剧。为贯彻落实党中央、国务院决策部署，进一步加大助企纾困力度，减轻企业负担，帮助渡过难关，经国务院同意，现就有关事项通知如下：

一、加大纾困资金支持力度。鼓励地方安排中小企业纾困资金，对生产经营暂时面临困难但产品有市场、项目有前景、技术有竞争力的中小企业，以及劳动力密集、社会效益高的民生领域服务型中小企业（如养老托育机构等）给予专项资金支持，减轻房屋租金、水电费等负担，给予社保补贴等，帮助企业应对原材料价格上涨、物流及人力成本上升等压力。落实创业担保贷款贴息及奖补政策。用好小微企业融资担保降费奖补资金，支持扩大小微企业融资担保业务规模，降低融资担保成本。有条件的地方要发挥好贷款风险补偿机制作用。（财政部、工业和信息化部、人力资源社会保障部、人民银行等国务院相关部门及各地区按职责分工负责）

二、进一步推进减税降费。深入落实月销售额15万元以下的小规模纳税人免征增值税、小型微利企业减征所得税、研发费用加计扣除、固定资产加速折旧、支持科技创新进口等税收优惠政策。制造业中小微企业按规定延缓缴纳2021年第四季度部分税费。研究适时出台部分惠企政策到期后的接续政策。持续清理规范涉企收费，确保政策红利落地。（财政部、税务总局、海关总署、市场监管总局等国务院相关部门及各地区按职责分工负责）

三、灵活精准运用多种金融政策工具。加强再贷款再贴现政策工具精准“滴灌”中小企业，用好新增3000亿元支小再贷款额度。加大信用贷款投放，按规定实施普惠小微企业信用贷款支持政策。对于受新冠肺炎疫情、洪涝灾害及原材料价格上涨等影响严重的小微企业，加强流动资金贷款支持，按规定实施普惠小微企业贷款延期还本付息政策。（人民银行、银保监会按职责分工负责）

四、推动缓解成本上涨压力。加强大宗商品监测预警，强化市场供需调节，严厉打击囤积居奇、哄抬价格等违法行为。支持行业协会、大型企业搭建重点行业产业链供需对接平台，加强原材料保供对接服务。推动期货公司为中小企业提供风险管理服务，助力中小企业运用期货套期保值工具应对原材料价格大幅波动风险。稳定班轮公司在中国主要出口航线的运力供给。发挥行业协会、商会及地方政府作用，引导外贸企业与班轮公司签订长约合同，鼓励班轮公司推出中小企业专线服务。（国家发展改革委、工业和信息化部、市场监管总局、中国证监会、交通运输部、商务部等国务院相关部门及各地区按职责分工负责）

五、加强用电保障。加强电力产供储销体系建设，科学实施有序用电，合理安排错峰用电，保障对中小企业尤其是制造业中小企业的能源安全稳定供应。推动产业链龙头企业梳理上下游重点企业名单，保障产业链关键环节中小企业用电需求，维护产业链供应链安全稳定，确保企业已有订单正常生产，防范订单违约风险。加快推进电力市场化改革，充分考虑改革进程和中小企业承受能力，平稳有序推动中小企业进入电力市场。鼓励有条件的地方对小微企业用电实行阶段性优惠。（国家发展改革委、工业和信息化部及各地区按职责分工负责）

六、支持企业稳岗扩岗。落实失业保险稳岗返还及社保补贴、培训补贴等减负稳岗扩就业政策，支持中小企业稳定岗位，更多吸纳高校毕业生等重点群体就业。推动各级政府公共服务平台、人力资源服务机构为中小企业发布实时有效的岗位信息，加强用工供需信息对接。（人力资源社会保障部、财政部及各地区按职责分工负责）

七、保障中小企业款项支付。进一步落实《保障中小企业款项支付条例》，制定保障中小企业款项支付投诉处理办法，加强大型企业应付账款管理，对滥用市场优势地位逾期占用、恶意拖欠中小企业账款行为，加大联合惩戒力度。继续开展清理拖欠中小企业账款专项行动。推动各级政府部门、事业单位、大型企业及时支付采购中小企业货物、工程、服务的账款，从源头防范层层拖欠形成“三角债”。严禁以不签合同、在合同中不约定具体付款时限和付款方式等方法规避及时支付义务的行为。（工业和信息化部、国务院国资委、财政部、人民银行等国务院相关部门及各地区按职责分工负责）

八、着力扩大市场需求。加大民生领域和新型基础设施建设投资力度，进一步落实《政府采购促进中小企业发展管理办法》，鼓励各地因地制宜细化预留采购份额、价格评审优惠、降低投标成本、优先采购等支持措施。组织开展供需对接活动，促进大型企业扩大向中小企业采购规模。搭建政银合作平台，开展中小企业跨境撮合服务。依托跨境电商等外贸新业态，为中小企业提供远程网上交流、供需信息对接等服务。加快海外仓发展，保障外贸产业链供应链畅通运转。充分发挥境外经贸合作区作为中小企业“抱团出海”平台载体的作用，不断提升合作区建设质量和服务水平，引导和支持有合作需求的中小企业入区开展投资合作。（国家发展改革委、财政部、商务部、工业和信息化部按职责分工负责）

九、全面压实责任。各有关部门、各地区要进一步把思想认识行动统一到党中央、国务院决策部署上来，强化责任担当，勇于开拓创新，进一步细化纾困举措，积极采取针对性措施，帮助中小企业应对困难，推动中小企业向“专精特新”方向发展，不断提升市场竞争力。各有关部门要加强对中小企业面临困难和问题的调研，总结经验做法，加强政策储备，适时推动出台；要加大对地方的指导支持力度，扎实推动各项政策措施落地见效。落实情况要及时报送国务院促进中小企业发展工作领导小组办公室。（国务院相关部门及各地区按职责分工负责）

国务院办公厅

2021年11月10日

1. 国务院：应对新型冠状病毒感染肺炎疫情联防联控机制关于进一步做好当前新冠肺炎疫情防控工作的通知 国办发明电〔2021〕1号

各省、自治区、直辖市人民政府，国务院各部委、各直属机构：

在以习近平同志为核心的党中央坚强领导下，我国疫情防控取得重大战略成果，疫情总体得到有效控制，但反弹风险丝毫不能忽视。近期，境外疫情持续扩散蔓延，我国多地接连发生局部聚集性疫情，甚至在同一省份或城市出现多个源头导致的多条传播链。为贯彻党中央、国务院决策部署，进一步压实“四方责任”，落实“四早”要求，毫不放松抓好“外防输入、内防反弹”各项工作，巩固来之不易的防控成果，现就有关事项通知如下。

一、激活疫情应急指挥体系

1．强化应急指挥体系建设。各地党政主要负责同志要亲自抓，相关负责同志分兵把守，整合各部门力量，扁平化运行，细化完善防控工作目标、方案和措施。

2．建立健全工作专班。各地要参照国务院联防联控机制有关做法，结合实际设立核酸检测力量调度、流调溯源、转运隔离、区域协查、交通管控等工作专班，提升应急处置能力。

3．保持24小时运行状态。各地应急指挥体系要加强值班值守，落实岗位责任，严格执行“日报告”和“零报告”制度，密切跟踪疫情形势，全时待命，确保发生疫情后第一时间启动应急处置。

4．加强前线指挥力量。发生聚集性疫情的地区，所在省份要在启动应急指挥体系的同时，成立前线指挥中心，省委或省政府主要负责同志坐镇指挥，各有关方面配合协作。

二、提升核酸检测能力

5．明确核酸检测能力要求。常住人口500万以下的城市，通过统筹省内资源，具备在2天内完成全员检测的能力。常住人口500万以上的城市，通过统筹省内资源和国家支持，具备在3—5天内完成全员检测的能力。

6．补齐核酸检测能力短板。各地要对核酸检测能力再摸底，统筹疾控机构、医疗机构、第三方检测机构等力量，迅速补齐人员、物资设备等缺口。国家卫生健康委要统筹调配全国机动检测队伍，及时对检测能力薄弱地区予以支持。

7．提升核酸检测质量。要加强质量控制，做好核酸检测试剂质量监督评估，组建专业采样队伍，推进采样环节和流程标准化、信息化，加强第三方检测机构管理，制定核酸检测组织工作预案，加强人员统一培训，提高检测效率和质量。

8．加强重点人员筛查。要对冷链食品从业人员等高风险人群至少每周开展一次核酸检测，对服务业从业人员定期开展核酸抽样检测，对一些重点场所定期开展环境核酸检测，力争主动发现疫情。

三、加强流调溯源力量

9．加强各方协作。强化疾控、公安、工业和信息化、通信、交通运输等方面协作，“平战结合”组建流调队伍，科学配置专业结构，综合运用信息化等技术手段，加强演练“备战”，规范开展流调溯源。发挥好医务人员优势，把流调溯源工作延伸到医疗机构和急救机构。

10．拓宽流调溯源思路。要解放思想，针对“人传人、人传物、物传人”开展多链条追溯分析，“人”、“物”同查，摸清疫情传播的脉络。

11．提升病毒基因测序能力。针对全国疫情多点散发防控需要，完善病毒基因测序能力布局，在中国疾控中心做好病毒基因测序工作的基础上，发挥中国医学科学院、军事科学院军事医学研究院及有条件省份相关机构的病毒基因测序优势，加强技术人员培训，做好数据分析比对，提高病毒基因测序准确性。

四、严格重点人员隔离管控

12．备足隔离房间。各地要按照密切接触者和密切接触者的密切接触者（以下称次密切接触者）“应隔尽隔、一人一间、集中隔离”的要求，根据自身实际情况，提前准备好一定数量、可随时转换的隔离房间。制定调用征用后备隔离场所方案，以及农村地区大规模集中隔离预案和建设临时集中隔离场所方案。专班专人负责隔离场所的调用征用和管理工作，做到隔离人员12小时内转运到集中隔离场所。

13．加强隔离场所管理。严格按照“三区两通道”标准进行改造，隔离区内配齐送餐人员、垃圾清运人员、保安人员和医务人员，分工明确，各司其职。隔离场所启用后不得提供与隔离无关的服务。加强管理人员、医务人员和相关服务人员培训，规范送餐、消毒和垃圾处理等服务保障工作，避免交叉感染。加强隔离人员日常健康监测，发现确诊病例、疑似病例和无症状感染者后及时转运。

14．加强重点地区、重点人员隔离管控。对高风险地区要进行入户“拉网式”排查，确保每一位感染者追踪到位，每一位密切接触者、次密切接触者应隔尽隔。对省内跨地区的密切接触者、次密切接触者，疫情发生地要第一时间向流入地通报协查；对跨省份的，疫情发生省份要第一时间报告国务院联防联控机制综合组，同时通报有关省份协查，综合组加强统筹协调和指导支持。

五、全力以赴加强医疗救治

15．加强救治力量。要选择综合救治力量强的传染病专科医院或者符合呼吸道传染病防控条件的综合性医院作为定点收治医院，并按照不低于床位总数的10%配置重症监护床位，提前做好供氧等基础设施安排，建立健全多学科综合诊疗制度。发现感染者后，确保2小时内转往定点收治医院。严格落实新冠肺炎诊疗方案（试行第八版），坚持中西医结合，关口前移、“一人一策”，实行规范化同质化治疗。组织好有援鄂经验的医务人员，优先调派其参与一线救治工作。

16．强化院感防控。实行严格的院内感染防控制度，明确专人监督负责。地方各级卫生健康行政部门主要负责同志亲自抓，医疗机构主要负责同志负总责，每个业务科室有专人督促检查。加强对民营医疗机构、个体诊所监管。对发生院内感染的医疗机构要在全国范围内通报，情节严重的要依法依规从重处理直至吊销执业许可证，对有关责任人要严肃问责。

六、突出抓好农村地区疫情防控

17．完善落实农村地区疫情防控工作方案。各地要把农村地区疫情防控作为重中之重，加强机场周边、城乡结合部、务工返乡人员较多的农村地区疫情防控，强化网格化、精细化管理。对机场入境物品和周边遗弃垃圾等进行集中管理，开展必要的预防性消毒。对城乡结合部服务业从业人员特别是从事冷链工作人员加大核酸检测频次。对春节返乡人员做好信息登记和日常健康监测，督促减少外出、落实个人防护措施。

18．提升“早发现”能力。加强农村地区疫情监测，扩大“应检尽检”范围，将基层医疗卫生机构和个体诊所工作人员、农村需排查和协查人员等纳入定期核酸检测范围，落实“村报告、乡采样、县检测”规定和相应的处置流程。严格落实首诊报告制度，充分发挥村卫生室、个体诊所、药店等的“哨点”作用，做好退烧药、抗病毒药、抗菌素等药品处方或销售的实名登记，发现有发热、咳嗽、咽痛、嗅（味）觉减退、腹泻等症状的可疑患者，2小时内必须向乡镇卫生院报告，对缓报、迟报甚至瞒报的严肃处理。接到报告后，乡镇卫生院要立即组织核酸采样，尽快送到县级医疗卫生机构检测。县域内要加强统筹协调，加大对采样力量薄弱乡镇卫生院的人员培训和采样物资保障力度，县级医疗卫生机构和专家组要加强对基层监测排查工作的巡回指导和督查。

19．加强重点环节防控。压实乡镇党委政府、村两委等责任，做好重点人员摸排和网格化管理，组织开展居家健康监测。加强集贸市场、村民活动室、棋牌室等场所疫情防控管理，对养老机构、福利机构、监所等人员集中场所严格落实防控指南要求，必要时实行封闭管理，相关行业主管部门要切实担负起责任。要引导农村地区减少聚集性活动，推动移风易俗，倡导“喜事缓办，丧事简办，宴会不办”，严格控制庙会等民俗活动。暂停宗教活动场所聚集性活动，依法制止非法宗教活动。

七、做好疫情发布和宣传等工作

20．及时报告和发布疫情信息。严格按照新冠肺炎防控方案（第七版）和诊疗方案（试行第八版）要求诊断报告确诊病例、疑似病例和无症状感染者，医疗机构接到核酸检测阳性报告后应在2小时内完成网络直报，辖区内的疾控机构应立即启动流行病学调查并在2小时内完成审核，任何地方不得擅自增加会诊、复核等程序。要完善疫情信息发布机制，发生疫情后及时发布权威信息，不得晚于次日召开新闻发布会。发生疫情的市（地、州、盟）每天都要召开发布会，所在省份在疫情初期就要及时主动发声，加强正面宣传和政策解读。

21．加强健康教育宣传。灵活运用群众喜闻乐见、通俗易懂的宣传手段，引导群众养成良好的卫生习惯和生活方式，持之以恒落实好戴口罩、勤洗手、不聚集等常态化防控要求，做到群防群控。

22．做好物资保障。加强生活物资、家庭取暖等方面保障，对留在当地过春节的群众做好关心关爱，做好困难群众生活兜底保障工作。全力做好疫情防控、生产生活物资等应急物资运输保障，按照“非必要不阻断”原则，保障运输车辆快速便捷通行。

当前，疫情防控正处于关键时期，各地要把疫情防控作为头等大事，将每项工作落实到部门、机构、个人，细化实化各项措施，领导干部带头学习掌握疫情防控政策规定和部署要求。要强化督导检查，建立通报机制，对疫情防控中的突出问题进行通报，问题严重的依法依规严肃问责。要切实履行防控投入责任，确保所需经费、物资及时到位，关心关爱一线疾控人员、医务工作者、社区防控人员等，按规定落实相关补助政策。春节前，各省份要开展疫情防控应急演练，国务院联防联控机制综合组对演练情况进行检查指导。

国务院应对新型冠状病毒感染肺炎

疫情联防联控机制

2021年1月18日

1. 商务部 卫生健康委 市场监管总局：关于餐饮服务 新冠肺炎疫情常态化防控工作的指导意见 商服贸发〔2020〕224号

各省、自治区、直辖市人民政府，新疆生产建设兵团：

为贯彻党中央关于抓紧抓实抓细疫情常态化防控工作的决策部署，统筹推进疫情防控和经济社会发展，落实全国深化“放管服”改革优化营商环境电视电话会议精神，按照“外防输入、内防反弹”的总体防控策略，在疫情常态化防控下规范餐饮服务经营活动，保障消费者与从业人员健康安全，促进餐饮业恢复发展，经国务院同意，现提出以下意见。

一、总体要求

依据《中华人民共和国传染病防治法》、《突发公共卫生事件应急条例》等法律法规，贯彻“安全第一、预防为主”的方针，依法科学开展餐饮业疫情防控工作，落实落细防控举措，充分发挥餐饮业在保障民生、增加就业、拉动消费等方面的重要作用，扎实做好“六稳”工作，全面落实“六保”任务，努力克服新冠肺炎疫情带来的不利影响，支持餐饮业加快恢复发展。

二、基本原则

（一）属地管理原则。各地人民政府要落实属地责任，加强组织领导，完善细化各项举措，加大宣贯力度，督促餐饮服务单位严格落实主体责任，结合实际做好防控工作。

（二）群防群控原则。坚持底线思维，牢固树立群防群控意识，餐饮服务单位及其从业人员、消费者均应克服疫情防控麻痹思想，自觉执行防控要求，严格履行防控职责，确保全员参与、全面覆盖、全过程防控。

（三）动态调整原则。根据疫情形势变化和当地突发公共卫生事件应急响应级别调整，按照当地疫情防控总体要求，因地制宜、因时制宜，动态调整和完善餐饮服务疫情防控标准和措施。

三、严格落实各方防控责任

（一）各地人民政府要落实属地责任。各地要按照分区分级精准防控的原则，依据当地疫情形势、风险等级和应急响应级别，提出餐饮业防控原则并严格落实疫情防控责任。商务、卫生健康、市场监管等有关部门要按照当地人民政府的要求做好餐饮领域疫情防控工作，指导餐饮服务单位制定并严格落实各项防控措施。

（二）餐饮服务单位要落实疫情防控主体责任。餐饮服务单位要按照当地疫情防控部门要求，落实疫情防控主体责任，科学制定具有针对性和可操作性的疫情防控工作方案和应急处置预案；确保口罩、洗手液、消毒剂和测温仪等疫情防控物资配备到位；对员工开展疫情防控知识培训，确保所有员工应知应会。

（三）顾客及其他进店人员要落实群防群控责任。顾客及其他进店人员要按当地防控要求，积极配合餐饮服务单位，科学佩戴口罩，做好体温检测、信息登记、出示“防疫健康码”、“行程卡”信息等相关工作，做好个人防护。

四、压实压紧餐饮服务单位疫情防控责任

（一）在员工卫生管理方面的防控责任。

1．在高、中风险地区运营的餐饮服务单位应建立员工健康档案，记录每日体温、外出情况等信息。

2．员工上岗期间要保持工作服整洁，及时进行手部清洁消毒，佩戴一次性使用医用口罩或医用外科口罩并及时更换，避免用未清洁的手触摸口、眼、鼻，打喷嚏、咳嗽时用纸巾遮住口鼻或采用肘臂遮挡等。

3．员工一旦有发热、乏力、干咳等疑似新冠肺炎典型症状，应上报单位或所在社区，及时到定点医疗机构就诊并通报相关人员，按规定进行隔离，就诊途中尽量避免乘坐公共交通工具，防范交叉感染。如员工发现共同居住人或密切接触者出现疑似新冠肺炎典型症状，要及时上报单位并做好个人防护和隔离，必要时应到定点医疗机构就诊。

（二）在食品采购和加工方面的防控责任。

1．严格落实进货查验及索证索票制度，确保食材来源可追溯。外出采购人员要做好个人防护。严禁采购和制售野生动物及其制品。

2．原料供应商选择、食品加工制作、餐饮具和加工用具的清洗要符合保障食品安全的有关规定。密切关注原料供货商所在地的疫情变化情况，如原料供货商有员工确诊，根据相关防控规定对已采购原料封存待查。

（三）在环境卫生方面的防控责任。

1．根据当地防控要求，定时对食品处理区域、就餐区域、人员通道、食品货梯、员工更衣室、集体宿舍区域进行清洁、消毒工作。加强环境卫生消毒处理时，要避免污染食物。当场所出现新冠肺炎确诊病例，餐饮服务单位应立即停业，在疾控机构指导下对场所环境和空调（系统）进行终末消毒，直到卫生学评价合格并经相关部门同意后，方可恢复正常营业。

2．温度适宜时，尽量采用自然通风加强室内空气流通。按照空调运行管理与使用的有关指引做好空调的运行管理和使用。如使用集中空调，运行过程中以最大新风量运行，每月至少一次清洗、消毒或更换空调关键部件。在高、中风险地区运营的餐饮服务单位，每周清洗、消毒空调通风系统空气处理机组、送风口和冷凝水盘等部位，必要时更换空调关键部件。

3．按照全面精准开展环境卫生和消毒工作的有关规定做好餐饮服务场所消毒工作，制定就餐及公共区域清洁消毒制度，做好清洁消毒记录并在公共区域展示；做好收银台、电梯、公共卫生间等公用设备设施和门把手等高频接触物体表面的定时清洁消毒；有条件的餐饮服务单位可在电梯口、收银台等处配备手消毒剂或感应式手消毒设施；就餐区无洗手设施的，应配备免洗手消毒液等手消毒用品或其他手消毒设施。

4．确保卫生间通风良好，洗手设备正常运行，洗手盆、地漏等水封隔离有效。每日定时对卫生间进行清洁、消毒和杀虫，保持地面、墙壁、洗手池无污垢，便池无粪便污物累积，室内无蚊蝇。

5．加强垃圾分类管理，及时收集并清运。废弃口罩应设置专门垃圾桶。每天对垃圾存放设施进行清洁消毒。餐厨垃圾处置应当符合法律、法规、规章的要求。

（四）在顾客服务方面的防控责任。

1．在高、中风险地区运营的餐饮服务单位应要求进店人员佩戴一次性使用医用口罩或医用外科口罩，在其进店时按照当地防控要求，检查进店人员“防疫健康码”或“行程卡”等，上述信息符合要求且体温检测正常，方允许进店；通过扫描进店人员“防疫健康码”等方式，记录其姓名、联系方式和到店时段等，同时依法做好信息保护工作。低风险地区餐饮服务单位无需对进店人员进行体温检测，在餐厅（馆）可不戴口罩。

2．在高、中风险地区运营的餐饮服务单位在店内外候餐区、取餐区、结账区等人员易聚集区域划设“一米线”，严格控制人流密度，进店人数要与餐位数相匹配，不得造成点餐、等餐、等位等人员聚集。提倡建立顾客预约制度，合理安排顾客到店时间，避免人员聚集。提倡非接触式点餐、结账。

3．在高、中风险地区运营的餐饮服务单位应控制餐厅（馆）就餐人数，拉开桌位间距，确保间隔在1米以上；如桌椅固定无法移动，要明确标识出非使用桌位；不安排非同行顾客同桌就餐。

4．在高、中风险地区运营的餐饮服务单位每个包间限开一桌，就餐座位间要保持1米以上距离，提倡就餐人员在用餐前后戴好口罩。每餐次顾客离开后，须对包间进行清洁消毒和通风处理。

5．对于合餐顾客，餐饮服务单位应提供“一菜一公筷、一汤一公勺”，或者“一人一公筷、一人一公勺”服务，公勺公筷宜采用不同颜色、材质或突出标识等醒目的方式进行区分。鼓励提供密封包装的牙签。有条件的餐厅（馆）要积极推广分餐制。提倡就餐时间不超过两小时。

6．用于顾客自取或外送的餐食，宜采用密封方式盛放，提倡每份餐食使用自制或订制的专用食安封签。如无食安封签，可选用一次性使用、不可复原的材料封闭外包装，防止运送过程中污染餐食。餐饮外卖服务应按照外卖配送和快递从业人员疫情健康防护的有关要求严格执行。

各地要根据本指导意见，结合当地疫情常态化防控工作实际，进一步细化完善各项措施和要求，指导餐饮服务单位做好疫情防控工作。

商　 务　 部

卫 生 健 康 委

市场监管总局

2020年10月27日

1. 国务院：进一步做好稳外贸稳外资 国办发〔2020〕28号

各省、自治区、直辖市人民政府，国务院各部委、各直属机构：

当前国际疫情持续蔓延，世界经济严重衰退，我国外贸外资面临复杂严峻形势。为深入贯彻习近平总书记关于稳住外贸外资基本盘的重要指示批示精神，落实党中央、国务院决策部署，做好“六稳”工作，落实“六保”任务，进一步加强稳外贸稳外资工作，稳住外贸主体，稳住产业链供应链，经国务院同意，现提出以下意见：

一、更好发挥出口信用保险作用。中国出口信用保险公司在风险可控前提下，积极保障出运前订单被取消的风险。2020年底前，中国出口信用保险公司根据外贸企业申请，可合理变更短期险支付期限或延长付款宽限期、报损期限等。（财政部、商务部、银保监会、中国出口信用保险公司按职责分工负责）

二、支持有条件的地方复制或扩大“信保+担保”的融资模式。鼓励有条件的地方支持政府性融资担保机构参与风险分担，对出口信用保险赔付额以外的贷款本金进行一定比例的担保，商业银行在“信保+担保”条件下，合理确定贷款利率。（各地方人民政府，财政部、商务部、银保监会、中国出口信用保险公司按职责分工负责）

三、以多种方式为外贸企业融资提供增信支持。充分发挥国家融资担保基金和地方政府性融资担保机构作用，参与外贸领域融资风险分担，支持、引导各类金融机构加大对小微外贸企业融资支持。（各地方人民政府，财政部、商务部、人民银行、银保监会按职责分工负责）鼓励银行机构结合内部风险管理要求，与资质较好的外贸类服务平台进行合作，获取贸易相关信息和资信评估服务，优化贸易背景真实性审核，更好服务外贸企业。（各地方人民政府，商务部、银保监会按职责分工负责）

四、进一步扩大对中小微外贸企业出口信贷投放。更好发挥金融支持作用，进一步加大对中小微外贸企业的信贷投放，缓解融资难、融资贵问题。（各地方人民政府，财政部、商务部、人民银行、银保监会、进出口银行按职责分工负责）

五、支持贸易新业态发展。尽快推动在有条件的地方新增一批市场采购贸易方式试点，力争将全国试点总量扩大至30个左右，带动中小微企业出口。（商务部牵头，各地方人民政府，发展改革委、财政部、海关总署、税务总局、市场监管总局、外汇局按职责分工负责）充分利用外经贸发展专项资金、服务贸易创新发展引导基金等现有渠道，支持跨境电商平台、跨境物流发展和海外仓建设等。鼓励进出口银行、中国出口信用保险公司等各类金融机构在风险可控前提下积极支持海外仓建设。（商务部牵头，财政部、银保监会、进出口银行、中国出口信用保险公司按职责分工负责）深入落实外贸综合服务企业代办退税管理办法，不断优化退税服务，持续加快退税进度。加大对外贸综合服务企业的信用培育力度，使更多符合认证标准的外贸综合服务企业成为海关“经认证的经营者”（AEO）。（商务部、海关总署、税务总局按职责分工负责）

六、引导加工贸易梯度转移。鼓励有条件的地方结合当地实际，通过基金等方式，支持加工贸易梯度转移。培育一批东部与中西部、东北地区共建的加工贸易产业园区。借助中国加工贸易产品博览会等平台，完善产业转移对接机制。鼓励中西部、东北地区发挥优势，承接劳动密集型外贸产业。（各地方人民政府，财政部、商务部按职责分工负责）

七、加大对劳动密集型企业支持力度。对纺织品、服装、家具、鞋靴、塑料制品、箱包、玩具、石材、农产品、消费电子类产品等劳动密集型产品出口企业，在落实减税降费、出口信贷、出口信保、稳岗就业、用电用水等各项普惠性政策基础上进一步加大支持力度。（各地方人民政府，发展改革委、工业和信息化部、财政部、人力资源社会保障部、商务部、人民银行、税务总局、银保监会、进出口银行、中国出口信用保险公司按职责分工负责）

八、助力大型骨干外贸企业破解难题。研究确定大型骨干外贸企业名单，梳理大型骨干外贸企业及其核心配套企业需求，建立问题批办制度，推动解决生产经营中遇到的矛盾问题，在进出口各环节予以支持，“一企一策”做好服务。研究在风险可控前提下，对大型骨干外贸企业进一步加快出口退税进度的支持措施。（商务部牵头，工业和信息化部、海关总署、税务总局、进出口银行、中国出口信用保险公司按职责分工负责）

九、拓展对外贸易线上渠道。推进“线上一国一展”，支持和鼓励有能力、有意愿的地方政府、重点行业协会举办线上展会。用好外经贸发展专项资金，在规定范围内，支持中小外贸企业开拓市场，参加线上线下展会。发挥好国内商协会、驻外机构、海外中资企业协会作用，积极对接国外商协会，帮助出口企业对接更多海外买家。（各地方人民政府，外交部、工业和信息化部、财政部、商务部按职责分工负责）

十、进一步提升通关便利化水平。持续优化口岸营商环境，继续巩固压缩货物整体通关时间成效，进一步推动规范和降低进出口环节合规成本，在有条件的口岸推广口岸收费“一站式阳光价格”，提升口岸收费透明度和可比性。加大对出口企业提供技术贸易措施咨询服务力度，助力企业开拓海外市场。推进扩大油脂油料、肉类、乳品市场准入，促进进口，保障市场供应。（海关总署负责）

十一、提高外籍商务人员来华便利度。在严格落实好防疫要求前提下，继续与有关国家商谈建立“快捷通道”，为外贸外资企业重要商务、物流、生产和技术服务急需人员往来提供便利。继续对符合条件的来华复工复产外国人全面实施“快捷通道”。参照“快捷通道”有关做法，本着“防疫为先、确保必需、压实责任、体现便利”原则，对来华从事必要经贸、科技等活动的外国人作出便利性安排。支持地方结合当地市场采购贸易方式特点，开通专有通道，便利外商入市采购，优先安排在华常驻外商尽快返华入市。在做好疫情防控的前提下，逐步有序恢复中外人员往来。按照国务院联防联控机制部署，分阶段增加国际客运航班总量，在防疫证明齐全的情况下，适度增加与我主要投资来源地民航班次，便利外籍商务人员来华。（各地方人民政府，外交部、发展改革委、商务部、移民局、民航局按职责分工负责）

十二、给予重点外资企业金融支持。外资企业同等适用现有1.5万亿元再贷款再贴现专项额度支持。加大对重点外资企业的金融支持力度，进出口银行5700亿元新增贷款规模可用于积极支持符合条件的重点外资企业。各省区市商务主管部门摸清辖区内重点外资企业融资需求及经营情况，及时与银行业金融机构共享重点外资企业信息，加强各地外资企业协会等机构与银行业金融机构的合作，推动开展“银企对接”，银行业金融机构按市场化原则积极保障重点外资企业融资需求。（各地方人民政府，人民银行、商务部、银保监会、进出口银行按职责分工负责）

十三、加大重点外资项目支持服务力度。对全国范围内投资额1亿美元以上的重点外资项目，梳理形成清单，在前期、在建和投产等环节，内外资一视同仁加大用海、用地、能耗、环保等方面服务保障力度。（各地方人民政府，商务部、发展改革委、自然资源部、生态环境部按职责分工负责）

十四、鼓励外资更多投向高新技术产业。推动高新技术企业认定管理和服务的便利化，进一步加强对外商投资企业申请高新技术企业认定的培训和宣传解读，着重加强对疫情防控等应急领域企业的政策服务，吸引更多外资投向高新技术和民生健康领域。（科技部牵头，财政部、税务总局按职责分工负责）

十五、降低外资研发中心享受优惠政策门槛。降低适用支持科技创新进口税收政策的外资研发中心专职研究与试验发展人员数量要求，鼓励外商来华投资设立研发中心，提升引资质量。（财政部牵头，商务部、税务总局按职责分工负责）

各地区、各部门要以习近平新时代中国特色社会主义思想为指导，增强“四个意识”、坚定“四个自信”、做到“两个维护”，坚决贯彻党中央、国务院决策部署，提高站位、积极作为、狠抓落实。各地区要结合实际，完善配套措施，认真组织实施，推动各项政策在本地区落地见效。各部门要按职责分工，加强协作、形成合力，确保各项政策落实到位。

国务院办公厅

2020年8月5日

1. 国务院：支持多渠道灵活就业 国办发〔2020〕27号

各省、自治区、直辖市人民政府，国务院各部委、各直属机构：

个体经营、非全日制以及新就业形态等灵活多样的就业方式，是劳动者就业增收的重要途径，对拓宽就业新渠道、培育发展新动能具有重要作用。为全面强化稳就业举措，落实保居民就业任务，经国务院同意，现就支持多渠道灵活就业提出以下意见。

一、总体要求

以习近平新时代中国特色社会主义思想为指导，全面贯彻党的十九大和十九届二中、三中、四中全会精神，坚持以人民为中心的发展思想，把支持灵活就业作为稳就业和保居民就业的重要举措，坚持市场引领和政府引导并重、放开搞活和规范有序并举，顺势而为、补齐短板，因地制宜、因城施策，清理取消对灵活就业的不合理限制，强化政策服务供给，创造更多灵活就业机会，激发劳动者创业活力和创新潜能，鼓励自谋职业、自主创业，全力以赴稳定就业大局。

二、拓宽灵活就业发展渠道

（一）鼓励个体经营发展。持续深化商事制度改革，提供便捷高效的咨询、注册服务。引导劳动者以市场为导向，依法自主选择经营范围。鼓励劳动者创办投资小、见效快、易转型、风险小的小规模经济实体。支持发展各类特色小店，完善基础设施，增加商业资源供给。对下岗失业人员、高校毕业生、农民工、就业困难人员等重点群体从事个体经营的，按规定给予创业担保贷款、税收优惠、创业补贴等政策支持。（财政部、人力资源社会保障部、商务部、人民银行、税务总局、市场监管总局等按职责分工负责）

（二）增加非全日制就业机会。落实财政、金融等针对性扶持政策，推动非全日制劳动者较为集中的保洁绿化、批发零售、建筑装修等行业提质扩容。增强养老、托幼、心理疏导和社会工作等社区服务业的吸纳就业能力。加强对非全日制劳动者的政策支持，对就业困难人员、离校2年内未就业高校毕业生从事非全日制等工作的，按规定给予社会保险补贴。（民政部、财政部、人力资源社会保障部、住房城乡建设部、商务部、人民银行等按职责分工负责）

（三）支持发展新就业形态。实施包容审慎监管，促进数字经济、平台经济健康发展，加快推动网络零售、移动出行、线上教育培训、互联网医疗、在线娱乐等行业发展，为劳动者居家就业、远程办公、兼职就业创造条件。合理设定互联网平台经济及其他新业态新模式监管规则，鼓励互联网平台企业、中介服务机构等降低服务费、加盟管理费等费用，创造更多灵活就业岗位，吸纳更多劳动者就业。（国家发展改革委、教育部、工业和信息化部、人力资源社会保障部、交通运输部、商务部、文化和旅游部、国家卫生健康委、市场监管总局等按职责分工负责）

三、优化自主创业环境

（四）加强审批管理服务。开通行业准入办理绿色通道，对需要办理相关行业准入许可的，实行多部门联合办公、一站式审批。在政府指定的场所和时间内销售农副产品、日常生活用品，或者个人利用自己的技能从事依法无须取得许可的便民劳务活动，无须办理营业执照。加大“放管服”改革力度，引导劳动者规范有序经营。（市场监管总局和地方各级人民政府按职责分工负责）

（五）取消部分收费。取消涉及灵活就业的行政事业性收费，对经批准占道经营的免征城市道路占用费。建立公开投诉举报渠道，依法查处违规收费行为。（财政部、住房城乡建设部、市场监管总局和地方各级人民政府按职责分工负责）

（六）提供低成本场地支持。落实阶段性减免国有房产租金政策，鼓励各类业主减免或缓收房租，帮助个体经营者等灵活就业人员减轻房租负担。有条件的地方可将社区综合服务设施闲置空间、非必要办公空间改造为免费经营场地，优先向下岗失业人员、高校毕业生、农民工、就业困难人员提供。（国家发展改革委、民政部、住房城乡建设部和地方各级人民政府按职责分工负责）

四、加大对灵活就业保障支持

（七）推动新职业发布和应用。密切跟踪经济社会发展、互联网技术应用和职业活动新变化，广泛征求社会各方面对新职业的意见建议，动态发布社会需要的新职业、更新职业分类，引导直播销售、网约配送、社群健康等更多新就业形态发展。及时制定新职业标准，推出新职业培训课程。完善统计监测制度，探索建立新就业形态统计监测指标。（人力资源社会保障部、国家统计局等负责。列第一位者为牵头单位，下同）

（八）开展针对性培训。将有创业意愿的灵活就业人员纳入创业培训范围，组织开展开办店铺、市场分析、经营策略等方面的创业培训，促进提升创业能力和创业成功率。支持各类院校、培训机构、互联网平台企业，更多组织开展养老、托幼、家政、餐饮、维修、美容美发等技能培训和新兴产业、先进制造业、现代服务业等领域新职业技能培训，推进线上线下结合，灵活安排培训时间和培训方式，按规定落实职业培训补贴和培训期间生活费补贴，增强劳动者就业能力。（人力资源社会保障部、教育部、财政部等负责）

（九）优化人力资源服务。把灵活就业岗位供求信息纳入公共就业服务范围，开设灵活就业专区专栏，免费发布供求信息，按需组织专场招聘，送岗位进基层进社区，提供职业指导等服务。指导企业规范开展用工余缺调剂，帮助有“共享用工”需求的企业精准、高效匹配人力资源。有条件的城市可选择交通便利、人员求职集中的地点设立劳务市场或零工市场，组织劳务对接洽谈，加强疫情防控、秩序维护和安全管理。鼓励各类人力资源服务机构为灵活就业人员提供规范有序的求职招聘、技能培训、人力资源外包等专业化服务，按规定给予就业创业服务补助。（人力资源社会保障部、财政部等负责）

（十）维护劳动保障权益。研究制定平台就业劳动保障政策，明确互联网平台企业在劳动者权益保护方面的责任，引导互联网平台企业、关联企业与劳动者协商确定劳动报酬、休息休假、职业安全保障等事项，引导产业（行业、地方）工会与行业协会或行业企业代表协商制定行业劳动定额标准、工时标准、奖惩办法等行业规范。依法纠正拖欠劳动报酬等违法违规行为。持续深入推进工程建设领域农民工按项目参加工伤保险，有针对性地做好工伤预防工作。（人力资源社会保障部、应急部、全国总工会等按职责分工负责）

（十一）加大对困难灵活就业人员帮扶力度。2020年缴纳基本养老保险费确有困难的灵活就业人员，可按规定自愿暂缓缴费。对符合条件的灵活就业人员，及时按规定纳入最低生活保障、临时救助范围。（民政部、财政部、人力资源社会保障部、税务总局等按职责分工负责）

五、切实加强组织实施

（十二）强化组织领导。地方各级人民政府特别是市、县级人民政府要切实履行稳就业主体责任，把支持多渠道灵活就业作为就业工作重要内容，结合实际创新工作举措，加强规范引导，完善监督管理，促进灵活就业健康发展。各级人民政府要统筹用好就业补助资金和其他稳就业、保就业的资金，保障灵活就业扶持政策落实。各有关部门要同向发力、分工合作，坚持问题导向，完善政策措施，共同破解工作难题。（各有关部门、单位和地方各级人民政府按职责分工负责）

（十三）加强激励督导。各地区各有关部门要加强督促检查和政策实施情况评估，狠抓政策落实，简化手续，提高效率，确保灵活就业人员便捷享受各项支持政策和就业创业服务。将支持多渠道灵活就业有关工作纳入文明城市创建和测评内容。对灵活就业政策落实好、发展环境优、工作成效显著的城市，优先纳入创业型城市创建范围。（中央文明办、人力资源社会保障部和地方各级人民政府按职责分工负责）

（十四）注重舆论引导。充分利用各种宣传渠道和媒介，大力宣传支持灵活就业的政策措施和典型做法，宣传自主就业创业和灵活就业的典型事迹。建立舆情监测和处置机制，积极主动回应社会关切，营造良好舆论氛围。（各有关部门、单位和地方各级人民政府按职责分工负责）

国务院办公厅

2020年7月28日

1. 国务院：印发《应急救援领域中央与地方财政事权和支出责任划分改革方案》 国办发〔2020〕22号

各省、自治区、直辖市人民政府，国务院各部委、各直属机构：

《应急救援领域中央与地方财政事权和支出责任划分改革方案》已经党中央、国务院同意，现印发给你们，请结合实际认真贯彻落实。

国务院办公厅

2020年7月4日

（此件公开发布）

应急救援领域中央与地方财政事权和支出责任划分改革方案

按照党中央、国务院有关决策部署，现就应急救援领域中央与地方财政事权和支出责任划分改革制定如下方案。

一、总体要求

以习近平新时代中国特色社会主义思想为指导，全面贯彻党的十九大和十九届二中、三中、四中全会以及中央经济工作会议精神，健全充分发挥中央和地方两个积极性体制机制，优化政府间事权和财权划分，建立权责清晰、财力协调、区域均衡的中央和地方财政关系，形成稳定的各级政府事权、支出责任和财力相适应的制度，充分发挥我国应急管理体系特色和优势，积极推进我国应急管理体系和能力现代化。

二、主要内容

（一）预防与应急准备。

1.应急管理制度建设。

将研究制定应急救援领域法律法规和国家政策、标准、技术规范，国家级规划编制，国家总体应急预案和安全生产类、自然灾害类专项预案编制，应急预案综合协调衔接，中央部门直接组织的全国性应急预案演练等，确认为中央财政事权，由中央承担支出责任。

将研究制定应急救援领域地方性法规和政策、标准、技术规范，地区性规划编制，地方应急预案编制，地方应急预案演练等，确认为地方财政事权，由地方承担支出责任。

2.应急救援能力建设。

将国家应对特别重大灾害和事故协调联动机制建设、国家综合性消防救援队伍管理、国家应急指挥总部建设与运行维护、国家应急物资储备，确认为中央财政事权，由中央承担支出责任。

将国家区域应急救援中心建设与运行维护、国家综合性消防救援队伍建设、国家级专业应急救援队伍建设，确认为中央与地方共同财政事权，由中央与地方共同承担支出责任。

将地方应急救援队伍建设、应急避难设施建设、地方应急物资储备，确认为地方财政事权，由地方承担支出责任。

3.应急管理信息系统建设。

将全国统一的应急管理信息系统建设，确认为中央与地方共同财政事权，由中央与地方按照相关职责分工分别承担支出责任，其中中央主要负责信息系统的规划设计、中央部门信息系统软硬件配备及维护支出，地方主要负责地方各级信息系统软硬件配备及维护支出。

4.安全生产监督管理。

将中央部门负责的国家安全生产综合监督管理，对各省、自治区、直辖市和中央企业总部安全生产工作的指导协调服务和监督检查，中央部门直接组织的安全生产巡查、安全生产和消防考核、安全生产标准化建设以及危险化学品、烟花爆竹、工矿商贸企业安全生产监督管理，国家煤矿安全监察，国家煤矿安全生产准入制度的组织实施和监督，海洋石油安全生产综合监督管理等，确认为中央财政事权，由中央承担支出责任。

将地方性的安全生产监督管理事项，确认为地方财政事权，由地方承担支出责任。

5.应急宣传教育培训。

将中央部门直接组织开展的全国性应急宣传教育培训工作，确认为中央财政事权，由中央承担支出责任。

将地方组织开展的应急宣传教育培训工作，确认为地方财政事权，由地方承担支出责任。

（二）灾害事故风险隐患调查及监测预警。

1.灾害事故风险隐患调查。

将全国灾害事故风险调查和重点隐患排查，确认为中央与地方共同财政事权，由中央与地方按照相关职责分工分别承担支出责任，其中中央主要负责国家灾害风险事故隐患基础数据库建设、支持开展综合风险评估相关支出，灾害风险事故隐患基础数据库纳入全国统一的应急管理信息系统；地方主要负责本行政区内灾害事故风险调查评估和隐患排查、为国家灾害风险事故隐患基础数据库提供数据支撑相关支出。

2.灾害事故监测预警。

将国家自然灾害、安全生产及火灾监测预警体系建设，确认为中央与地方共同财政事权，由中央与地方按照相关职责分工分别承担支出责任，其中中央主要负责全国统一的灾害事故监测预警和报告制度建设、中央部门监测预警系统建设支出；地方主要负责当地监测预警系统建设、应急信息员队伍建设支出。监测预警系统建设中相关信息化工作纳入全国统一的应急管理信息系统建设。

（三）应急处置与救援救灾。

将特别重大事故调查处理，特别重大自然灾害调查评估，安全生产类、自然灾害类等突发事件的国际救援，确认为中央财政事权，由中央承担支出责任。

将煤矿生产安全事故调查处理、国家启动应急响应的特别重大灾害事故应急救援救灾，确认为中央与地方共同财政事权，由中央与地方共同承担支出责任。

将其他事故调查处理、自然灾害调查评估、灾害事故应急救援救灾等，确认为地方财政事权，由地方承担支出责任。

中央预算内投资支出按国家有关规定执行，主要用于中央财政事权或中央与地方共同财政事权事项。中央与新疆生产建设兵团财政事权和支出责任划分，参照中央与地方划分原则执行；财政支持政策原则上参照新疆维吾尔自治区有关政策执行，并适当考虑新疆生产建设兵团的特殊因素。应急救援领域其他未列事项，按照改革的总体要求和事项特点具体确定财政事权和支出责任。

三、配套措施

（一）加强组织领导。应急救援领域中央与地方财政事权和支出责任划分改革是推进应急管理体系和能力现代化的重要举措。各地区各有关部门要增强“四个意识”、坚定“四个自信”、做到“两个维护”，加强组织领导，切实履行职责，密切协调配合，确保改革工作落实到位。

（二）落实支出责任。各地区各有关部门要践行安全发展理念，筑牢安全生产防线，根据改革确定的中央与地方财政事权和支出责任划分，合理安排预算，及时下达资金，切实落实支出责任。跨区域调动救援队伍按照“谁调动，谁补偿”的原则承担相应支出责任。要全面实施预算绩效管理，优化支出结构，着力提高应急救援领域财政资源配置效率和使用效益。

（三）推进省以下改革。各省级人民政府要参照本方案精神，结合省以下财政体制等实际，合理划分省以下应急救援领域财政事权和支出责任。要明确省级人民政府推进本区域内应急救援工作的职责，加强省级统筹，加大对区域内财力困难地区的资金支持力度。要将适宜由地方更高一级政府承担的应急救援领域支出责任上移，避免基层政府承担过多支出责任。

（四）协同推进改革。应急救援领域财政事权和支出责任划分改革，要同其他改革紧密结合、协同推进、良性互动、形成合力。应急处置和应急救援阶段地质灾害调查监测等事项由相关部门共同研究确定。国家综合性消防救援队伍完成整合前，总体维持原公安消防部队和武警森林部队财政管理模式，中央与地方承担的支出责任不变；队伍整合完成后，根据队伍管理体制、事权职责调整中央与地方支出责任。

本方案自2020年1月1日起实施。

2020.7.27

1. 国务院联防联控机制综合组：做好精准健康管理推进人员有序流动 联防联控机制综发﹝2020﹞203号

各省、自治区、直辖市及新疆生产建设兵团应对新型冠状病毒肺炎疫情联防联控机制（领导小组、指挥部）：

为指导各地精准做好不同疫情风险等级地区人员健康管理服务，推进人员有序流动，有效遏制疫情传播和扩散，统筹疫情防控和正常生产生活秩序恢复，提出以下工作要求。

一、各地要根据疫情情况科学划分疫情风险等级，依法依规、精准划定防控区域范围至最小单元（如楼栋、病区、居民小区、自然村组等），及时采取限制人员流动、核酸检测、健康监测等综合防控措施。

二、加强个人防护，保持“一米线”、勤洗手、戴口罩、公筷制等卫生习惯和生活方式，降低疫情传播风险。

三、中高风险等级地区要尽量减少不必要人员流动，避免人员聚集。有中高风险等级地区旅居史的人员，跨地区流动时须持有到达目的地前7日内核酸检测阴性证明或能够出示包含核酸检测阴性信息的健康通行码“绿码”，到达目的地后，在测温正常且做好个人防护的前提下可自由有序流动。如无法提供上述核酸检测阴性信息，到达目的地后应当立即接受核酸检测或接受14天隔离医学观察。瞒报、谎报人员将承担法律责任。

四、低风险等级地区人员持健康通行码“绿码”，在测温正常且做好个人防护的前提下可自由有序流动。如无必要，尽量避免前往中高风险地区。

五、各地要按照依法、科学、精准防控要求，规范人员健康管理措施。对在常态化防控措施之外附加其他不合理限制要求的，要立即予以纠正。对造成恶劣影响的典型案例，坚决依法查处，并通过媒体予以曝光。

六、离京人员健康管理工作按照《关于做好离京人员新冠肺炎健康管理服务工作的通知》（联防联控机制综发〔2020〕198号）执行。

国务院应对新型冠状病毒肺炎疫情联防联控机制综合组

（代章）

2020年6月25日

1. 国务院联防联控机制综合组：全面精准开展环境卫生和消毒工作 联防联控机制综发〔2020〕195号

各省、自治区、直辖市及新疆生产建设兵团应对新冠肺炎疫情联防联控机制（领导小组、指挥部）：

　　本次新冠肺炎疫情防控过程中，环境卫生和消毒工作对于切断传播途径、保护易感人群发挥了重要作用。近期，部分地区出现零星散发病例和聚集性疫情,为强化防控措施，巩固防控成果，各地要进一步全面精准开展环境卫生和消毒工作，坚决防止疫情反弹。现就有关工作通知如下：

　　一、严格落实主体责任

　　要落实属地、部门、单位、个人“四方责任”，建立日常环境卫生和消毒管理监督制度。针对复工复产后的生产、生活、购物、交通等环境，采取网格化管理、包片包干，责任到人。要强化主体责任落实，加大检查力度，对各单位、各场所环境卫生和消毒进行全面评估，开展查漏洞补短板大排查，即查即改，将环境卫生和消毒措施布置到位、落实到位、效果到位。

　　二、全面开展环境卫生治理

　　（一）规范市场环境卫生管理。以农集贸市场为重点，发动商户对所有摊位进行全面彻底环境卫生清理和卫生排查，对市场内公厕、水池、垃圾桶、活禽宰杀点等基础设施进行清扫保洁。及时清运积存垃圾，清除卫生死角，对操作台面、下水道、运输车辆等重点部位严格落实消毒措施。加强对运输车辆、存储库房、物品的环境和包装消毒，落实实名登记、验视和可疑物品报告制度。

　　（二）开展企事业单位环境卫生大扫除。要对车间、厂房、办公室、职工宿舍、食堂等人员较集中的场所环境进行大扫除，清理积存杂物、废弃物，彻底清理卫生死角，加强场所内自然通风换气，保持室内空气流通，规范空调运行管理与使用。要针对仓库、车间、食堂、宿舍、地下车库等重点区域采取措施，清除隐患。

　　（三）强化重点场所环境卫生整治。要对机场、铁路、长途客运、公交、地铁、出租车场站和所有公共交通工具开展环境卫生清扫，经常换气通风，科学规范做好日常消毒。超市、商场、宾馆等人员密集、流动性大的场所做好公共区域的物体表面清洁消毒，加强洗手、卫生间等设施的卫生管理，及时清理下水明沟的污水污物，对下水管道、空气处理装置水封、卫生间地漏等U型管定期检查与维护。要抓好小熟食店、流动摊贩、早夜市餐饮点等食品重点场所卫生管理，及时清理垃圾，确保卫生整洁。

　　（四）落实社区自主保洁制度。充分发挥物业、居（村）委会等的作用，落实街道社区、乡村的环境卫生保洁制度，确保有专人负责，定时定点清理垃圾和废弃物，消除卫生死角。社区要组织群众自己动手净化绿化美化家庭和公共空间，倡导群众保持良好的生活习惯，提升居民文明意识和健康卫生意识，合理膳食、适度运动、充分休息，不随地吐痰，妥善处理废弃口罩。

　　三、科学规范消毒

　　（一）采取科学消毒措施。应当合理使用消毒剂，遵循“五要”，即：隔离病区、病人住所要进行随时消毒和终末消毒；医院、机场、车站等人员密集场所的环境物体表面要增加消毒频次；高频接触的门把手、电梯按钮等要加强清洁消毒；垃圾、粪便和污水要进行收集和无害化处理；要做好个人手卫生。遵循“七不”，即：不对室外环境开展大规模的消毒；不对外环境进行空气消毒；不直接使用消毒剂对人员进行消毒；不对水塘、水库、人工湖等环境中投加消毒剂进行消毒；不在有人条件下对空气使用化学消毒剂消毒；不用戊二醛对环境进行擦拭和喷雾消毒；不使用高浓度的含氯消毒剂做预防性消毒。

　　（二）做好消毒质量控制和效果评价。各地要确保消毒效果，做好消毒质量控制。所用消毒产品要合法有效，并严格遵循产品说明书使用。要根据消毒对象的特点，选择可靠的消毒方法及消毒剂量，采取必要的检测手段，确保消毒效果。对消毒范围广、持续时间长的预防性消毒和影响大的终末消毒，各级疾控部门要做好消毒效果评价。

　　各地要严格落实常态化防控各项措施要求，强化对各行各业环境卫生和消毒工作落实情况的监督指导，加强培训与科普宣传，有效改善生产生活环境，消除疫情传播隐患。

附件：常用消毒剂使用指南

国务院应对新型冠状病毒肺炎疫情

联防联控机制综合组

（代章）

2020年6月17日

1. 国务院：支持出口产品转内销 国办发〔2020〕16号

各省、自治区、直辖市人民政府，国务院各部委、各直属机构：

为深入贯彻落实党中央、国务院关于统筹推进新冠肺炎疫情防控和经济社会发展工作的决策部署，做好“六稳”工作、落实“六保”任务，在鼓励企业拓展国际市场的同时，支持适销对路的出口产品开拓国内市场，着力帮扶外贸企业渡过难关，促进外贸基本稳定，经国务院同意，现提出以下意见：

一、工作原则

发挥政府引导作用，支持出口产品转内销，帮助外贸企业纾困，确保产业链供应链畅通运转，稳住外贸外资基本盘。发挥企业主体作用，坚持市场化运作，鼓励外贸企业拓展销售渠道，促进国内消费提质升级。落实地方属地责任，因地制宜推动出口产品转内销工作，重点帮扶本地区重要产业链供应链外贸企业和中小微外贸企业。

二、支持出口产品进入国内市场

（一）加快转内销市场准入。在2020年底前，对依据出口目的国标准生产且相关标准技术指标达到我国强制性标准要求的出口产品，因疫情影响转内销的，允许企业作出相关书面承诺，通过自我符合性声明的方式进行销售，法律法规另有规定的从其规定。外贸企业要对出口转内销产品加贴的中文和外文标签、标识的一致性负责。（市场监管总局、生态环境部、交通运输部、农业农村部、卫生健康委、应急部、铁路局、民航局、煤矿安监局等部门按职责分工负责）出口转内销产品涉及强制性产品认证（CCC认证）的，应当依法获得强制性产品认证证书。继续深化强制性产品认证制度改革，简化出口转内销产品认证程序，缩短办理时间。（市场监管总局负责）简化企业办税程序。（税务总局负责）

（二）促进“同线同标同质”发展。支持企业发展“同线同标同质”（以下称“三同”）产品，即在同一生产线上按照相同标准、相同质量要求生产既能出口又可内销的产品，帮助企业降低成本、实现内外销转型。扩大“三同”适用范围至一般消费品、工业品领域。（市场监管总局、工业和信息化部、商务部等部门按职责分工负责）开通国内生产销售审批快速通道。加快完善“三同”公共信息服务平台功能。（市场监管总局负责）开展“三同”产品宣传推广活动，提升知名度和影响力。（中央宣传部、商务部、市场监管总局按职责分工负责）

（三）加强知识产权保障。支持外贸企业与品牌商协商出口转内销产品涉及的知识产权授权，做好专利申请、商标注册和著作权登记。加强对外贸企业知识产权运用的指导和服务。（中央宣传部、知识产权局按职责分工负责）

三、多渠道支持转内销

（四）搭建转内销平台。鼓励外贸企业对接电商平台，依托各类网上购物节，设置外贸产品专区。在符合国内疫情防控要求的前提下，引导主要步行街组织开展出口产品转内销专题活动。组织各地大型商业企业与外贸企业开展订单直采，设立外贸产品销售专区、专柜。组织国内采购商在中国进出口商品交易会（广交会）、中国加工贸易产品博览会等采购外贸产品。（商务部，各省、自治区、直辖市人民政府负责）

（五）发挥有效投资带动作用。重点结合各地新型基础设施、新型城镇化和重大工程（“两新一重”）建设需要，组织对接一批符合条件的出口产品转内销，帮助企业融入投资项目产业链供应链。（各省、自治区、直辖市人民政府负责）引导外贸企业积极补链固链强链，推动产业链协同创新和产业结构调整、加大技术和工艺升级改造力度，参与工业和通信业重大项目建设。（工业和信息化部，各省、自治区、直辖市人民政府负责）

（六）精准对接消费需求。引导外贸企业精准对接国内市场消费升级需求，发挥质量、研发等优势，应用大数据、工业互联网等技术，通过个性化定制、柔性化生产，研发适销对路的内销产品，创建自有品牌，培育和发展新的消费热点，推动消费回升。鼓励外贸企业充分利用网上销售、直播带货、场景体验等新业态新模式，促进线上线下融合发展。（工业和信息化部、商务部按职责分工负责，各省、自治区、直辖市人民政府负责）

四、加大支持力度

（七）提升转内销便利化水平。对符合条件可集中办理内销征税手续的加工贸易企业，在不超过手（账）册有效期或核销截止日期的前提下，由每月15日前申报，调整为最迟可在季度结束后15天内申报。（海关总署负责）

（八）做好融资服务和支持。鼓励各类金融机构对出口产品转内销提供金融支持，加强供应链金融服务，结合实际开展内销保险项下的保单融资业务，加大流动性资金贷款等经营周转类信贷支持，积极开展应收账款、存货、机器设备、仓单、订单等质押融资，依托大型电商平台加强对中小微外贸企业直贷业务。（财政部、人民银行、银保监会按职责分工负责）

（九）加大保险支持力度。支持保险公司加大对出口产品转内销的保障力度，提供多元化的保险服务。（银保监会负责）

（十）加强资金支持。用足用好外经贸发展专项资金，支持出口转内销相关业务培训、宣传推介、信息服务等，支持外贸企业参加线上线下内销展会。（财政部、商务部负责）

五、加强组织实施

各地方人民政府、各有关部门要高度重视支持出口产品转内销工作。各地方要根据本地区实际出台针对性配套措施，商务部要会同相关部门制定具体工作方案并组织实施，加强政策指导和业务培训，及时总结推广好经验好做法；组织引导媒体集中开展宣传报道，营造支持出口产品转内销的良好环境，引导拓展国内市场空间，促进公平竞争。（中央宣传部、发展改革委、工业和信息化部、财政部、商务部、人民银行、海关总署、税务总局、市场监管总局、银保监会按职责分工负责，各省、自治区、直辖市人民政府负责）

国务院办公厅

2020年6月17日

1. 国务院联防联控机制综合组 ：做好离京人员新冠肺炎健康管理服务工作 联防联控机制综发﹝2020﹞198 号

各省、自治区、直辖市及新疆生产建设兵团应对新型冠状病毒肺炎疫情联防联控机制（领导小组、指挥部）：

　　近日，北京市发生新冠肺炎聚集性疫情，并在多省引发相关病例，防范疫情扩散形势严峻。为指导各地做好离京人员健康管理服务工作，坚决遏制疫情传播和扩散，统筹疫情防控和正常生产生活秩序恢复，现将有关事项通知如下。

　　一、各地要充分发挥大数据等优势，对5月30日至6月16日来自北京市中高风险街道（乡镇）人员、北京新发地批发市场等相关人员加强追踪管理，做到核酸检测应检尽检。对核酸检测阳性者及时进行隔离治疗，对疑似或确诊病例、无症状感染者的密切接触者但核酸检测阴性人员实施14天集中隔离医学观察，对其他核酸检测阴性者，单位和社区要加强健康管理，督促做好14天健康监测和个人防护，如出现发热、呼吸道及其他可疑症状应当及时就医。

　　二、根据北京市新冠肺炎疫情防控规定，自6月16日起，北京市中高风险街道（乡镇）人员、新发地批发市场等相关人员禁止离京，其他人员坚持“非必要不出京”，确需离京的须持7日内核酸检测阴性证明。

　　三、持有离京前7日内核酸检测阴性证明或能够出示包含核酸检测阴性信息的健康通行码“绿码”的离京人员，到达目的地后，在测温正常且做好个人防护的前提下可自由有序流动，各地、各部门不得另行设置其他限制条件。对瞒报、谎报人员依法追究有关责任。

　　四、无法提供离京前7日内核酸检测阴性证明的离京人员，到达目的地后应当立即接受核酸检测。核酸检测阳性者及时进行隔离治疗，核酸检测阴性者在测温正常且做好个人防护的前提下可自由有序流动。

　　五、上述要求根据北京市应急响应级别及时进行调整。

国务院应对新型冠状病毒肺炎

疫情联防联控机制综合组

（代章）

2020年6月19日

1. 国务院联防联控机制综合组：印发《低风险地区夏季重点场所重点单位重点人群新冠肺炎疫情常态化防控相关防护指南（修订版）》 联防联控机制综发〔2020〕192号 发改委

各省、自治区、直辖市及新疆生产建设兵团应对新型冠状病毒肺炎疫情联防联控机制（领导小组、指挥部）：

为做好新冠肺炎疫情常态化防控工作，指导重点场所、重点单位、重点人群做好防护，结合夏季防控特点，我们对《重点场所重点单位重点人群新冠肺炎疫情防控相关防控技术指南》进行了修订调整，形成《低风险地区夏季重点场所重点单位重点人群新冠肺炎疫情常态化防控相关防护指南（修订版）》。本指南只适用于新冠肺炎疫情低风险地区，中、高风险地区仍参照原版指南实施。有关措施如下：

一、落实日常重点防护措施

1.减少人员聚集。娱乐、休闲等活动场所，通过限量、错峰等方式，减少大范围人员聚集活动，人员接触时尽量保持1米以上的社交距离。餐饮场所，分散错峰就餐，减少人员聚集。在人员流动性较大、相对密闭的公共场所以及公共交通工具，做好个人防护，佩戴口罩。

2.加强环境卫生和消毒。室内经常开窗通风换气，保持空气流通。超市、商场、农集贸市场等人员密集、流动性大的场所做好公共区域的物体表面清洁消毒，落实日常保洁、环境卫生与消毒等措施，工作人员戴手套。加强洗手、卫生间等设施的卫生管理，及时清理下水明沟的污水污物，对下水管道、空气处理装置水封、卫生间地漏等U型管定期检查与维护。

3.规范空调管理和使用。办公场所、公共场所、公共交通工具和住宅等空调系统，开启前检查设备是否正常。对冷却塔等进行清洗，保持新风口清洁，定期对送风口等设备和部件进行清洗、消毒或更换。新风口和排风口要保持一定距离。集中空调系统运行过程中，尽可能减小回风、增大新风量。

4.提高公众健康素养。开展新时代爱国卫生运动，将卫生创建与防控工作有机结合。强化公众手卫生、一米线、开窗通风、清洁消毒、生病时减少去人员聚集场所和佩戴口罩等健康防护和意识，养成勤洗手、咳嗽和打喷嚏时注意遮挡等良好卫生习惯和行为。

二、强化重点环节防护

5.重点场所防护。在落实常态化防控措施前提下，商场、超市、农集贸市场、宾馆、餐馆等生活服务类场所，图书馆、博物馆、美术馆等室内场馆，加强室内通风，正确使用空调，做好环境清洁消毒和工作人员健康监测；公园、旅游景点等开放式活动场所，采取限量、预约、错峰等方式，减少人员聚集，做好环境卫生；影剧院、游艺厅、网吧等密闭式娱乐休闲场所，强化人员健康监测、限制人员数量和停留时间、通风消毒等措施。

6.重点单位防护。企业、邮政快递业、机关事业单位、建筑业等单位，做好办公场所、工区及公共区域、职工宿舍等通风换气、环境清洁消毒、人员健康监测；保持分区作业、分散错峰就餐、减少人员聚集等措施。进口物资、食品加工等相关单位做好运输工具和储存场所清洁消毒以及环境监测。养老机构、儿童福利院、监狱、精神卫生医疗机构等特殊单位做好风险防范，开展预防性卫生措施，落实人员进出管理、人员防护、健康监测、日常消毒等防护措施。学校和托幼机构做好应急预案、防护物资储备、教室宿舍环境卫生和消毒，加强因病缺勤管理，严格实施“晨午检”“日报告”“零报告”制度。

7.重点人群防护。指导老年人、儿童、孕产妇、残疾人等重点人群做好个人防护、关爱帮扶等措施。在密闭公共场所工作的售货员、保洁员、服务员、司乘人员、食品从业人员、保安、客运场站服务人员，以及就医人员、教师、警察、环卫工人、快递员、海关人员、理发师等，加强健康管理和监测，做好戴口罩、勤洗手、戴手套等个人防护措施。

三、有效应对风险等级调整

各地按照分区分级标准，依据本地疫情形势，及时调整风险等级和应急响应级别。一旦从低风险调整为中高风险地区，要严格执行疫情防控规定，采取应急处置措施，实施精准防控。在划定防控区域范围内重点场所、重点单位、重点人群要从严从紧落实防控措施，及时调整转换卫生防护要求。

8.密闭式娱乐、休闲场所，建议暂停营业；生活服务类场所，应缩短营业时间、限制人员数量和停留时间，减少人群聚集；开放式活动场所，在做好环境清洁消毒、人员健康监测、减少人群聚集的前提下正常营业；客运场站和公共交通工具，要按照指南严格落实体温测量、戴口罩、通风消毒、分区分级客座率（满载率、人员聚集度）控制等措施。

9.鼓励具备条件的企事业单位采取错时上下班、弹性工作制或居家办公方式，实施分区作业、分散错峰就餐，控制会议频次和规模，尽量减少人员聚集。监狱、养老机构、儿童福利院等特殊单位实行全封闭管理，严格落实体温测量和健康监测等措施，加强个人防护，禁止外来人员探视。学校建议暂时停课。

10.重点人群要强化卫生防护措施，减少外出，做好健康监测、科学佩戴口罩、加强手卫生、避免到人群聚集尤其是通风不良的场所等。

各地要压实属地单位主体责任，严格落实常态化防控各项措施要求，加强统筹调度，因地制宜、因时制宜，要强化监督，安排专人负责监督各行各业防护指南的落实情况，要开展重点场所专职防疫培训，保证防护效果，要加强指导与科普宣传，指导联防联控、精准防护。

国务院应对新型冠状病毒肺炎

疫情联防联控机制综合组

（代章）

2020年6月17日

1. 国务院联防联控机制综合组：发挥医疗机构哨点作用做好常态化疫情防控工作 联防联控机制综发〔2020〕186号

据国家卫健委官网消息，11日国务院应对新型冠状病毒肺炎疫情联防联控机制综合组发布了《关于发挥医疗机构哨点作用做好常态化疫情防控工作的通知》，通知称，对于所有到发热门诊就诊的患者，必须扫“健康码”，并进行核酸检测。

关于发挥医疗机构哨点作用做好常态化疫情防控工作的通知

联防联控机制综发〔2020〕186号

各省、自治区、直辖市及新疆生产建设兵团联防联控机制（领导小组、指挥部）：

　　为有效落实“四早”要求，充分发挥医疗机构“哨点”作用，实现及时发现、快速处置、精准管控、有效救治，做好常态化疫情防控工作，现将有关要求通知如下：

　　一、充分发挥发热门诊的“哨点”作用

　　对于所有到发热门诊就诊的患者，必须扫“健康码”，并进行核酸检测。对于待排除和疑似病例要第一时间进行隔离医学观察，提供基本医疗服务。发热门诊不得拒绝接收发热患者就诊。各类医疗机构要全面落实预检分诊制，对有发热、咳嗽、乏力等症状的患者，在做好防护条件下由专人陪同按规定路径前往发热门诊就医。严格落实首诊负责制，医务人员要做好“守门人”，发现发热等可疑病例，要详细登记相关信息，并按相关程序及时报告、收治和转运。各地要加强宣传，引导发热患者首选设置有发热门诊的医疗机构就诊。

　　二、充分发挥基层医疗机构的“哨点”作用

　　乡镇卫生院、村卫生室、社区卫生服务中心（站）、诊所、门诊部等基层医疗卫生机构，要落实首诊负责制，对发热患者进行登记，在1小时内上报到所在地卫生健康部门，引导发热原因不明的患者到发热门诊进一步检查治疗。乡镇卫生院应当在相对独立区域设立临时隔离病房留观发热患者。各区县每天应对乡镇卫生院留观发热患者进行核酸检测。发热患者要提供核酸检测报告，如不能提供，由发热门诊报告本区县领导小组（指挥部），由领导小组（指挥部）组织人员对患者进行核酸检测。

　　三、充分发挥急救中心“哨点”作用

　　严格落实救护车信息传递与反馈制度。因发热拨打“120”急救电话，经120救护车转运至发热门诊就医的，急救中心要做好交接和登记，同时将患者转运信息报告本区县领导小组（指挥部），信息应包括患者姓名、地址、年龄、症状、就诊医院等。因发热拨打“120”未实施转运的，急救中心核实后，将患者准确信息报告本区县领导小组（指挥部），由领导小组（指挥部）组织做好防护后引导其去发热门诊就诊。

　　四、加强“哨点”监管，严格责任倒查制度

各地要加强对医疗机构的指导和监督，特别是针对具有“哨点”性质的医疗机构要开展定期检查和随机暗访，督促落实各项措施，确保“哨点”的敏感性，坚决防止因职责落实不力导致的疫情扩散。各地要进一步加强新冠病毒核酸检测管理，将上述通过“哨点”发现的人员纳入“应检尽检”范围。在确认核酸检测结果为阴性后，要及时解除管控措施，将“健康码”调整回正常状态。各“哨点”落实检测、登记、报告、引导等措施不力，导致“应检未检”的，开展责任倒查，依法依规对相关机构和责任人追究责任。

2020年6月9日 卫健委

1. 国务院联防联控机制综合组：加快推进新冠病毒核酸检测 联防联控机制综发﹝2020﹞181号

各省、自治区、直辖市及新疆生产建设兵团应对新冠肺炎疫情联防联控机制（领导小组、指挥部），国务院应对新冠肺炎疫情联防联控机制各成员单位：

党中央、国务院高度重视新冠病毒核酸检测工作，中央应对新型冠状病毒感染肺炎疫情工作领导小组会议作出部署，要求着力提升检测能力，尽力扩大检测范围。加强核酸检测工作，既有利于巩固防控成果，维护群众健康，又有助于人员合理流动，推动全面复工复产复学，是“外防输入、内防反弹”的重要措施。为指导各地在常态化防控工作中做好核酸检测工作，提出如下意见：

一、总体要求

坚持预防为主，将加快提升核酸检测能力、尽力扩大核酸检测范围作为常态化防控落实“四早”措施的首要举措。按照依法依规、分类指导、因地制宜的原则，做好对重点人群重点行业的应检尽检工作，全力排查风险隐患。落实加快提升检测能力所需的人员、设备和资金，压实属地、部门、单位和个人责任，完善工作机制，加强组织管理，做到及早发现、精准防控，为加快推进生产生活秩序全面恢复创造条件。

二、积极扩大检测范围，全力排查风险隐患

各地可根据疫情防控工作需要和检测能力，确定并动态调整检测策略和人群范围。

（一）重点人群“应检尽检”。

1.密切接触者；

2.境外入境人员；

3.发热门诊患者；

4.新住院患者及陪护人员；

5.医疗机构工作人员；

6.口岸检疫和边防检查人员；

7.监所工作人员；

8.社会福利养老机构工作人员。

（二）其他人群“愿检尽检”。各地可根据本地实际确定和动态调整优先检测人群。

（三）加强公众监测预警。疾控机构定期对普通人群进行抽样监测和流行病学调查，做好信息收集、分析研判和监测预警工作。各地根据疫情发展变化动态调整常态化防控措施和动态推进应检尽检。

三、加快提升检测能力，全力满足检测需求

（一）加强实验室建设。各地要加强三级医院、传染病专科医院、县（区)级及以上疾控机构、海关及有条件的县医院实验室建设，使其具备开展新冠病毒检测能力。对医疗资源相对缺乏、检测能力相对薄弱、疫情防控压力较大的陆路边境口岸城市，要对疾控机构、海关和1家综合实力较强的医疗机构予以重点支持，必要时可采用移动方舱实验室。同步加强其他二级以上医院、专科医院、妇幼保健院实验室建设，使其逐步达到新冠病毒核酸检测条件。

（二）强化质量控制和生物安全。加强实验室能力建设标准体系认证和实验室室内质控，常态化接受国家级或省级临床检验质量控制。各省级卫生健康行政部门组织辖区内检测机构分批参加室间质评，保证每家机构每年至少参加1次室间质评。检测机构加强生物安全管理，严格操作流程，妥善处置剩余生物样本，做好样本使用、保存、上送与销毁工作。

（三）加强人员规范培训。制定培训计划，省市级疾控机构、临检中心、海关全面加强对检验检测人员的技术培训和指导，确保实验室人员数量质量需求。做到采样人员熟练掌握鼻咽拭子、咽拭子等规范采集方法，实验室检测人员熟练掌握标本处理、相关试剂使用和检测方法，减少技术操作问题对检测结果准确性、可靠性的影响，实现标本采集、保存、运输、实验室检测等各个环节全流程规范化操作。

（四）加快设备产能提升。加强检测设备、检测方法和检测试剂以及移动方舱实验室等方面研究、审批和市场转化，抓紧增加更简便、更高效准确的检测设备生产，特别是核酸快速检测设备的生产扩能，扩大商业化应用，积极组织提升移动方舱实验室等新型检测设备产能应用规模。属地政府组织有关部门科学评估当地检测需求和设备、试剂和耗材生产能力，制定供求计划。工业和信息化部门协调生产企业，根据需求尽快扩大设备、试剂和耗材等产能。发展改革部门做好检测所需物资统一调配。

（五）确保信息安全畅通。加强信息安全管理，通过信息化手段，确保受检对象的信息准确、检测结果的规范管理和隐私保护。检测机构向受检对象提供检测证明并做好信息登记工作，发现检测结果阳性要严格履行报告责任。各地要及时将检测结果信息共享到“健康码”数据库，并依托全国一体化政务服务平台推动各地区核酸检测相关信息互认共享，在全国一体化平台“防疫健康信息码”及入境人员版统一标注。对伪造、篡改新冠病毒检测结果等行为依法依规严厉打击。

四、健全完善工作机制，全力提高检测效率

（一）落实相关部门责任。卫生健康部门公布具有检测资质的机构名单，指定医疗机构、疾控机构、海关、社会检测机构为“应检尽检”人群提供检测服务，指定医疗机构、社会检测机构为“愿检尽检”人群提供检测服务。进行较大规模人群检测时，可采用将5至10份标本混检进行初筛的方法，提高检测效率，降低检测成本。要加强对实验室质量控制和生物安全的监管，将检测工作纳入医疗卫生机构业务考核内容，收集检测相关信息并上报上级卫生健康部门。卫生健康部门负责组织密切接触者、发热门诊患者、新住院患者及陪护人员、医疗机构工作人员的检测工作，民政部门协调组织养老机构、儿童福利机构、未成人救助保护机构工作人员和城乡社区工作者的检测工作，公安、司法行政部门负责组织监所工作人员和新收被监管人员检测工作，海关负责组织境外入境人员、口岸检疫人员检测工作，移民管理部门负责组织边防检查人员检测工作。教育、交通运输、人力资源社会保障、商务等部门根据各地实际，优先组织、有序引导做好学校（幼儿园）工作人员、来自疫情重点地区复学学生、公共交通工具和客运场站服务人员、复工复产单位新到岗（返岗）人员检测工作。

（二）发挥社区单位作用。社区要加强对重点人群的管理，主动加强信息沟通，加大健康教育力度，引导社区群众协助、配合政府部门组织开展检测工作，依法接受样本采集、检测、隔离、治疗等防控措施。各机关企事业单位、学校、社会团体和其他组织在复工复产复学过程中，要建立健全核酸检测的管理制度，切实履行主体责任，主动联系卫生健康部门，有序组织开展检测工作。

（三）完善检测后续工作。检测机构及时将检测结果通知到检测对象。对检测结果阳性者按规定进行网络直报、流行病学调查、疫情处置等，社会检测机构要及时将核酸检测结果阳性者信息报告所在县（区）疾控机构。确诊患者立即按规定转运至定点医疗机构，无症状感染者按规定对其实施集中隔离医学观察，观察期间如出现临床表现应及时转运至定点医疗机构，确诊后及时订正。各地以县（区）为单位，合理设置隔离点，做到应隔尽隔、应收尽收。

五、强化组织管理，加大保障力度

（一）加强组织领导。地方各级党委和政府要将加强检测工作作为当前常态化疫情防控工作的重要内容，加强领导，精心组织。各级联防联控机制（领导小组、指挥部）要制定工作方案，落实部门职责，定期召开协调会议，研究解决扩大核酸检测工作的重点难点问题，做好舆情评估和风险应对，并及时根据疫情防控形势作动态调整。

（二）加强政策扶持。各地党委政府要研究制订快速提升核酸检测能力的相关政策措施，调动社会检测服务机构开展核酸检测工作的积极性。省级医保部门及时按程序将核酸检测项目及相关耗材纳入本省医疗保障诊疗项目目录。省级物价部门制定本省域内统一执行的收费标准。

（三）加大经费保障。各级政府加强资金统筹安排，对疾控机构、医疗机构、海关实验室建设和检测设备的配备予以经费支持，特别要加大对人群相对密集、流动性较大地区和边境口岸等重点地区支持力度。具备条件的地区将检测试剂和耗材纳入省级集中采购平台采购范围并简化招标采购程序。“应检尽检”所需费用由各地政府承担，“愿检尽检”所需费用由企事业单位或个人承担。

1. 加大宣传倡导。各地宣传部门与卫生健康部门共同加强新冠肺炎防控知识的科普宣传工作，利用多种媒体广泛传播防控知识，提高群众主动参与核酸检测的意识，促进愿检尽检。

2020年6月8日 卫健委

1. 国务院：发布《抗击新冠肺炎疫情的中国行动》白皮书

新华社北京6月7日电　国务院新闻办公室7日发布《抗击新冠肺炎疫情的中国行动》白皮书。全文如下：

抗击新冠肺炎疫情的中国行动

（2020年6月）

中华人民共和国

国务院新闻办公室

　　目录

　　前言

　　一、中国抗击疫情的艰辛历程

　　（一）第一阶段：迅即应对突发疫情

　　（二）第二阶段：初步遏制疫情蔓延势头

　　（三）第三阶段：本土新增病例数逐步下降至个位数

　　（四）第四阶段：取得武汉保卫战、湖北保卫战决定性成果

　　（五）第五阶段：全国疫情防控进入常态化

　　二、防控和救治两个战场协同作战

　　（一）建立统一高效的指挥体系

　　（二）构建全民参与严密防控体系

　　（三）全力救治患者、拯救生命

　　（四）依法及时公开透明发布疫情信息

　　（五）充分发挥科技支撑作用

　　三、凝聚抗击疫情的强大力量

　　（一）人的生命高于一切

　　（二）举全国之力抗击疫情

　　（三）平衡疫情防控与经济社会民生

　　（四）14亿中国人民坚韧奉献守望相助

　　四、共同构建人类卫生健康共同体

　　（一）中国感谢和铭记国际社会宝贵支持和帮助

　　（二）中国积极开展国际交流合作

　　（三）国际社会团结合作共同抗疫

　　结束语

　　前言

　　新型冠状病毒肺炎是近百年来人类遭遇的影响范围最广的全球性大流行病，对全世界是一次严重危机和严峻考验。人类生命安全和健康面临重大威胁。

　　这是一场全人类与病毒的战争。面对前所未知、突如其来、来势汹汹的疫情天灾，中国果断打响疫情防控阻击战。中国把人民生命安全和身体健康放在第一位，以坚定果敢的勇气和决心，采取最全面最严格最彻底的防控措施，有效阻断病毒传播链条。14亿中国人民坚韧奉献、团结协作，构筑起同心战疫的坚固防线，彰显了人民的伟大力量。

　　中国始终秉持人类命运共同体理念，肩负大国担当，同其他国家并肩作战、共克时艰。中国本着依法、公开、透明、负责任态度，第一时间向国际社会通报疫情信息，毫无保留同各方分享防控和救治经验。中国对疫情给各国人民带来的苦难感同身受，尽己所能向国际社会提供人道主义援助，支持全球抗击疫情。

　　当前，疫情在全球持续蔓延。中国为被病毒夺去生命和在抗击疫情中牺牲的人们深感痛惜，向争分夺秒抢救生命、遏制疫情的人们深表敬意，向不幸感染病毒、正在进行治疗的人们表达祝愿。中国坚信，国际社会同舟共济、守望相助，就一定能够战胜疫情，走出人类历史上这段艰难时刻，迎来人类发展更加美好的明天。

　　为记录中国人民抗击疫情的伟大历程，与国际社会分享中国抗疫的经验做法，阐明全球抗疫的中国理念、中国主张，中国政府特发布此白皮书。

　　一、中国抗击疫情的艰辛历程

　　新冠肺炎疫情是新中国成立以来发生的传播速度最快、感染范围最广、防控难度最大的一次重大突发公共卫生事件，对中国是一次危机，也是一次大考。中国共产党和中国政府高度重视、迅速行动，习近平总书记亲自指挥、亲自部署，统揽全局、果断决策，为中国人民抗击疫情坚定了信心、凝聚了力量、指明了方向。在中国共产党领导下，全国上下贯彻“坚定信心、同舟共济、科学防治、精准施策”总要求，打响抗击疫情的人民战争、总体战、阻击战。经过艰苦卓绝的努力，中国付出巨大代价和牺牲，有力扭转了疫情局势，用一个多月的时间初步遏制了疫情蔓延势头，用两个月左右的时间将本土每日新增病例控制在个位数以内，用3个月左右的时间取得了武汉保卫战、湖北保卫战的决定性成果，疫情防控阻击战取得重大战略成果，维护了人民生命安全和身体健康，为维护地区和世界公共卫生安全作出了重要贡献。

　　截至2020年5月31日24时，31个省、自治区、直辖市和新疆生产建设兵团累计报告确诊病例83017例，累计治愈出院病例78307例，累计死亡病例4634例，治愈率94.3%，病亡率5.6%。回顾前一阶段中国抗疫历程，大体分为五个阶段。

　　（一）第一阶段：迅即应对突发疫情

　　（2019年12月27日至2020年1月19日）

　　湖北省武汉市监测发现不明原因肺炎病例，中国第一时间报告疫情，迅速采取行动，开展病因学和流行病学调查，阻断疫情蔓延。及时主动向世界卫生组织以及美国等国家通报疫情信息，向世界公布新型冠状病毒基因组序列。武汉地区出现局部社区传播和聚集性病例，其他地区开始出现武汉关联确诊病例，中国全面展开疫情防控。

　　（1）2019年12月27日，湖北省中西医结合医院向武汉市江汉区疾控中心报告不明原因肺炎病例。武汉市组织专家从病情、治疗转归、流行病学调查、实验室初步检测等方面情况分析，认为上述病例系病毒性肺炎。

　　（2）12月30日，武汉市卫生健康委向辖区医疗机构发布《关于做好不明原因肺炎救治工作的紧急通知》。国家卫生健康委获悉有关信息后立即组织研究，迅速开展行动。

　　（3）12月31日凌晨，国家卫生健康委作出安排部署，派出工作组、专家组赶赴武汉市，指导做好疫情处置工作，开展现场调查。武汉市卫生健康委在官方网站发布《关于当前我市肺炎疫情的情况通报》，发现27例病例，提示公众尽量避免到封闭、空气不流通的公众场合和人多集中地方，外出可佩戴口罩。当日起，武汉市卫生健康委依法发布疫情信息。

　　（4）2020年1月1日，国家卫生健康委成立疫情应对处置领导小组。1月2日，国家卫生健康委制定《不明原因的病毒性肺炎防控“三早”方案》；中国疾控中心、中国医学科学院收到湖北省送检的第一批4例病例标本，即开展病原鉴定。

　　（5）1月3日，武汉市卫生健康委在官方网站发布《关于不明原因的病毒性肺炎情况通报》，共发现44例不明原因的病毒性肺炎病例。国家卫生健康委组织中国疾控中心等4家科研单位对病例样本进行实验室平行检测，进一步开展病原鉴定。国家卫生健康委会同湖北省卫生健康委制定《不明原因的病毒性肺炎诊疗方案（试行）》等9个文件。当日起，中国有关方面定期向世界卫生组织、有关国家和地区组织以及中国港澳台地区及时主动通报疫情信息。

　　（6）1月4日，中国疾控中心负责人与美国疾控中心负责人通电话，介绍疫情有关情况，双方同意就信息沟通和技术协作保持密切联系。国家卫生健康委会同湖北省卫生健康部门制定《不明原因的病毒性肺炎医疗救治工作手册》。

　　（7）1月5日，武汉市卫生健康委在官方网站发布《关于不明原因的病毒性肺炎情况通报》，共发现59例不明原因的病毒性肺炎病例，根据实验室检测结果，排除流感、禽流感、腺病毒、传染性非典型性肺炎和中东呼吸综合征等呼吸道病原。中国向世界卫生组织通报疫情信息。世界卫生组织首次就中国武汉出现的不明原因肺炎病例进行通报。

　　（8）1月6日，国家卫生健康委在全国卫生健康工作会议上通报武汉市不明原因肺炎有关情况，要求加强监测、分析和研判，及时做好疫情处置。

　　（9）1月7日，中共中央总书记习近平在主持召开中共中央政治局常务委员会会议时，对做好不明原因肺炎疫情防控工作提出要求。

　　（10）1月7日，中国疾控中心成功分离新型冠状病毒毒株。

　　（11）1月8日，国家卫生健康委专家评估组初步确认新冠病毒为疫情病原。中美两国疾控中心负责人通电话，讨论双方技术交流合作事宜。

　　（12）1月9日，国家卫生健康委专家评估组对外发布武汉市不明原因的病毒性肺炎病原信息，病原体初步判断为新型冠状病毒。中国向世界卫生组织通报疫情信息，将病原学鉴定取得的初步进展分享给世界卫生组织。世界卫生组织网站发布关于中国武汉聚集性肺炎病例的声明，表示在短时间内初步鉴定出新型冠状病毒是一项显著成就。

　　（13）1月10日，中国疾控中心、中国科学院武汉病毒研究所等专业机构初步研发出检测试剂盒，武汉市立即组织对在院收治的所有相关病例进行排查。国家卫生健康委、中国疾控中心负责人分别与世界卫生组织负责人就疫情应对处置工作通话，交流有关信息。

　　（14）1月11日起，中国每日向世界卫生组织等通报疫情信息。

　　（15）1月12日，武汉市卫生健康委在情况通报中首次将“不明原因的病毒性肺炎”更名为“新型冠状病毒感染的肺炎”。中国疾控中心、中国医学科学院、中国科学院武汉病毒研究所作为国家卫生健康委指定机构，向世界卫生组织提交新型冠状病毒基因组序列信息，在全球流感共享数据库（GISAID）发布，全球共享。国家卫生健康委与世界卫生组织分享新冠病毒基因组序列信息。

　　（16）1月13日，国务院总理李克强在主持召开国务院全体会议时，对做好疫情防控提出要求。

　　（17）1月13日，国家卫生健康委召开会议，部署指导湖北省、武汉市进一步强化管控措施，加强口岸、车站等人员体温监测，减少人群聚集。世界卫生组织官方网站发表关于在泰国发现新冠病毒病例的声明指出，中国共享了基因组测序结果，使更多国家能够快速诊断患者。香港、澳门、台湾考察团赴武汉市考察疫情防控工作。

　　（18）1月14日，国家卫生健康委召开全国电视电话会议，部署加强湖北省、武汉市疫情防控工作，做好全国疫情防范应对准备工作。会议指出，新冠病毒导致的新发传染病存在很大不确定性，人与人之间的传播能力和传播方式仍需要深入研究，不排除疫情进一步扩散蔓延的可能性。

　　（19）1月15日，国家卫生健康委发布新型冠状病毒感染的肺炎第一版诊疗方案、防控方案。

　　（20）1月16日，聚合酶链式反应（PCR）诊断试剂优化完成，武汉市对全部69所二级以上医院发热门诊就医和留观治疗的患者进行主动筛查。

　　（21）1月17日，国家卫生健康委派出7个督导组赴地方指导疫情防控工作。

　　（22）1月18日，国家卫生健康委发布新型冠状病毒感染的肺炎第二版诊疗方案。

　　（23）1月18日至19日，国家卫生健康委组织国家医疗与防控高级别专家组赶赴武汉市实地考察疫情防控工作。19日深夜，高级别专家组经认真研判，明确新冠病毒出现人传人现象。

　　（二）第二阶段：初步遏制疫情蔓延势头

　　（1月20日至2月20日）

　　全国新增确诊病例快速增加，防控形势异常严峻。中国采取阻断病毒传播的关键一招，坚决果断关闭离汉离鄂通道，武汉保卫战、湖北保卫战全面打响。中共中央成立应对疫情工作领导小组，并向湖北等疫情严重地区派出中央指导组。国务院先后建立联防联控机制、复工复产推进工作机制。全国集中资源和力量驰援湖北省和武汉市。各地启动重大突发公共卫生事件应急响应。最全面最严格最彻底的全国疫情防控正式展开，疫情蔓延势头初步遏制。

　　（1）1月20日，中共中央总书记、国家主席、中央军委主席习近平对新型冠状病毒感染的肺炎疫情作出重要指示，指出要把人民生命安全和身体健康放在第一位，坚决遏制疫情蔓延势头；强调要及时发布疫情信息，深化国际合作。

　　（2）1月20日，国务院总理李克强主持召开国务院常务会议，进一步部署疫情防控工作，并根据《中华人民共和国传染病防治法》将新冠肺炎纳入乙类传染病，采取甲类传染病管理措施。

　　（3）1月20日，国务院联防联控机制召开电视电话会议，部署全国疫情防控工作。

　　（4）1月20日，国家卫生健康委组织召开记者会，高级别专家组通报新冠病毒已出现人传人现象。

　　（5）1月20日，国家卫生健康委发布公告，将新冠肺炎纳入传染病防治法规定的乙类传染病并采取甲类传染病的防控措施；将新冠肺炎纳入《中华人民共和国国境卫生检疫法》规定的检疫传染病管理。国家卫生健康委发布《新型冠状病毒感染的肺炎防控方案（第二版）》。

　　（6）1月22日，中共中央总书记、国家主席、中央军委主席习近平作出重要指示，要求立即对湖北省、武汉市人员流动和对外通道实行严格封闭的交通管控。

　　（7）1月22日，国家卫生健康委发布《新型冠状病毒感染的肺炎诊疗方案（试行第三版）》。国务院新闻办公室就疫情举行第一场新闻发布会，介绍疫情有关情况。国家卫生健康委收到美方通报，美国国内发现首例确诊病例。国家生物信息中心开发的2019新型冠状病毒信息库正式上线，发布全球新冠病毒基因组和变异分析信息。

　　（8）1月23日凌晨2时许，武汉市疫情防控指挥部发布1号通告，23日10时起机场、火车站离汉通道暂时关闭。交通运输部发出紧急通知，全国暂停进入武汉市道路水路客运班线发班。国家卫生健康委等6部门发布《关于严格预防通过交通工具传播新型冠状病毒感染的肺炎的通知》。1月23日至29日，全国各省份陆续启动重大突发公共卫生事件省级一级应急响应。

　　（9）1月23日，中国科学院武汉病毒研究所、武汉市金银潭医院、湖北省疾病预防控制中心研究团队发现新冠病毒的全基因组序列与SARS－CoV的序列一致性有79.5%。国家微生物科学数据中心和国家病原微生物资源库共同建成“新型冠状病毒国家科技资源服务系统”，发布新冠病毒第一张电子显微镜照片和毒株信息。

　　（10）1月24日开始，从各地和军队调集346支国家医疗队、4.26万名医务人员和965名公共卫生人员驰援湖北省和武汉市。

　　（11）1月25日，中共中央总书记习近平主持召开中共中央政治局常务委员会会议，明确提出“坚定信心、同舟共济、科学防治、精准施策”总要求，强调坚决打赢疫情防控阻击战；指出湖北省要把疫情防控工作作为当前头等大事，采取更严格的措施，内防扩散、外防输出；强调要按照集中患者、集中专家、集中资源、集中救治“四集中”原则，将重症病例集中到综合力量强的定点医疗机构进行救治，及时收治所有确诊病人。会议决定，中共中央成立应对疫情工作领导小组，在中央政治局常务委员会领导下开展工作；中共中央向湖北等疫情严重地区派出指导组，推动有关地方全面加强防控一线工作。

　　（12）1月25日，国家卫生健康委发布通用、旅游、家庭、公共场所、公共交通工具、居家观察等6个公众预防指南。

　　（13）1月26日，中共中央政治局常委、国务院总理、中央应对疫情工作领导小组组长李克强主持召开领导小组第一次全体会议。国务院办公厅印发通知，决定延长2020年春节假期，各地大专院校、中小学、幼儿园推迟开学。国家药监局应急审批通过4家企业4个新型冠状病毒检测产品，进一步扩大新型冠状病毒核酸检测试剂供给能力。

　　（14）1月27日，中共中央总书记习近平作出指示，要求中国共产党各级组织和广大党员、干部，牢记人民利益高于一切，不忘初心、牢记使命，团结带领广大人民群众坚决贯彻落实党中央决策部署，全面贯彻“坚定信心、同舟共济、科学防治、精准施策”的要求，让党旗在防控疫情斗争第一线高高飘扬。

　　（15）1月27日，受中共中央总书记习近平委托，中共中央政治局常委、国务院总理、中央应对疫情工作领导小组组长李克强赴武汉市考察指导疫情防控工作，代表中共中央、国务院慰问疫情防控一线的医护人员。同日，中央指导组进驻武汉市，全面加强对一线疫情防控的指导督导。

　　（16）1月27日，国家卫生健康委发布《新型冠状病毒感染的肺炎诊疗方案（试行第四版）》。国家卫生健康委负责人应约与美国卫生与公众服务部负责人通话，就当前新型冠状病毒感染的肺炎疫情防控工作进行交流。

　　（17）1月28日，国家主席习近平在北京会见世界卫生组织总干事谭德塞时指出，疫情是魔鬼，我们不能让魔鬼藏匿；指出中国政府始终本着公开、透明、负责任的态度及时向国内外发布疫情信息，积极回应各方关切，加强与国际社会合作；强调中方愿同世界卫生组织和国际社会一道，共同维护好地区和全球的公共卫生安全。

　　（18）1月28日，国家卫生健康委发布《新型冠状病毒感染的肺炎防控方案（第三版）》。

　　（19）1月30日，国家卫生健康委通过官方渠道告知美方，欢迎美国加入世界卫生组织联合专家组。美方当天即回复表示感谢。

　　（20）1月31日，世界卫生组织宣布新冠肺炎疫情构成“国际关注的突发公共卫生事件”。国家卫生健康委发布《新型冠状病毒感染的肺炎重症患者集中救治方案》。

　　（21）2月2日开始，在中央指导组指导下，武汉市部署实施确诊患者、疑似患者、发热患者、确诊患者的密切接触者“四类人员”分类集中管理，按照应收尽收、应治尽治、应检尽检、应隔尽隔“四应”要求，持续开展拉网排查、集中收治、清底排查三场攻坚战。

　　（22）2月2日，国家卫生健康委负责人致函美国卫生与公众服务部负责人，就双方卫生和疫情防控合作再次交换意见。

　　（23）2月3日，中共中央总书记习近平主持召开中共中央政治局常务委员会会议，指出要进一步完善和加强防控，严格落实早发现、早报告、早隔离、早治疗“四早”措施；强调要全力以赴救治患者，努力“提高收治率和治愈率”“降低感染率和病亡率”。

　　（24）2月3日，中央指导组从全国调集22支国家紧急医学救援队，在武汉市建设方舱医院。

　　（25）2月4日，中国疾控中心负责人应约与美国国家过敏症和传染病研究所负责人通电话，交流疫情信息。

　　（26）2月5日，中共中央总书记、国家主席、中央军委主席、中央全面依法治国委员会主任习近平主持召开中央全面依法治国委员会第三次会议，强调要始终把人民生命安全和身体健康放在第一位，从立法、执法、司法、守法各环节发力，全面提高依法防控、依法治理能力，为疫情防控工作提供有力法治保障。

　　（27）2月5日，国务院联防联控机制加强协调调度，供应湖北省医用N95口罩首次实现供大于需。

　　（28）2月5日，国家卫生健康委发布《新型冠状病毒感染肺炎诊疗方案（试行第五版）》。

　　（29）2月7日，国务院联防联控机制印发《关于进一步强化责任落实做好防治工作的通知》，国家卫生健康委发布《新型冠状病毒感染肺炎防控方案（第四版）》。

　　（30）2月8日，国家卫生健康委在亚太经合组织卫生工作组会议上介绍中国防疫努力和措施。国家卫生健康委向中国驻外使领馆通报新型冠状病毒防控、诊疗、监测、流行病学调查、实验室检测等方案。中美两国卫生部门负责人再次就美方专家参加中国－世界卫生组织联合专家考察组的安排进行沟通。

　　（31）2月10日，中共中央总书记、国家主席、中央军委主席习近平在北京调研指导新冠肺炎疫情防控工作，并通过视频连线武汉市收治新冠肺炎患者的金银潭医院、协和医院、火神山医院，强调要以更坚定的信心、更顽强的意志、更果断的措施，紧紧依靠人民群众，坚决打赢疫情防控的人民战争、总体战、阻击战；指出湖北和武汉是疫情防控的重中之重，是打赢疫情防控阻击战的决胜之地，武汉胜则湖北胜，湖北胜则全国胜，要打好武汉保卫战、湖北保卫战；强调要按照集中患者、集中专家、集中资源、集中救治“四集中”原则，全力做好救治工作；强调要坚决抓好“外防输入、内防扩散”两大环节，尽最大可能切断传染源，尽最大可能控制疫情波及范围。

　　（32）2月10日，建立省际对口支援湖北省除武汉市以外地市新冠肺炎医疗救治工作机制，统筹安排19个省份对口支援湖北省武汉市以外16个市州及县级市。

　　（33）2月11日，国务院联防联控机制加强协调调度，供应湖北省医用防护服首次实现供大于求。

　　（34）2月11日，中国疾控中心专家应约与美国疾控中心流感部门专家召开电话会议，沟通和分享疫情防控信息。

　　（35）2月12日，中共中央总书记习近平主持召开中共中央政治局常务委员会会议，指出疫情防控工作到了最吃劲的关键阶段，要毫不放松做好疫情防控重点工作，加强疫情特别严重或风险较大的地区防控；强调要围绕“提高收治率和治愈率”“降低感染率和病亡率”，抓好疫情防控重点环节；强调要全面增强收治能力，坚决做到“应收尽收、应治尽治”，提高收治率；强调要提高患者特别是重症患者救治水平，集中优势医疗资源和技术力量救治患者；强调人口流入大省大市要按照“联防联控、群防群控”要求，切实做好防控工作。

　　（36）2月13日，美国卫生与公众服务部相关负责人致函中国国家卫生健康委负责人，沟通双方卫生和疫情防控合作等有关安排。

　　（37）2月14日，中共中央总书记、国家主席、中央军委主席、中央全面深化改革委员会主任习近平主持召开中央全面深化改革委员会第十二次会议，指出确保人民生命安全和身体健康，是中国共产党治国理政的一项重大任务；强调既要立足当前，科学精准打赢疫情防控阻击战，更要放眼长远，总结经验、吸取教训，针对这次疫情暴露出来的短板和不足，抓紧补短板、堵漏洞、强弱项，完善重大疫情防控体制机制，健全国家公共卫生应急管理体系。

　　（38）2月14日，全国除湖北省以外其他省份新增确诊病例数实现“十连降”。

　　（39）2月15日，国务院新闻办公室首次在湖北省武汉市举行疫情防控新闻发布会。至2月15日，已有7个诊断检测试剂获批上市，部分药物筛选与治疗方案、疫苗研发、动物模型构建等取得阶段性进展。

　　（40）2月16日开始，由中国、德国、日本、韩国、尼日利亚、俄罗斯、新加坡、美国和世界卫生组织25名专家组成的中国－世界卫生组织联合专家考察组，利用9天时间，对北京、成都、广州、深圳和武汉等地进行实地考察调研。

　　（41）2月17日，国务院联防联控机制印发《关于科学防治精准施策分区分级做好新冠肺炎疫情防控工作的指导意见》，部署各地区各部门做好分区分级精准防控，有序恢复生产生活秩序。

　　（42）2月18日，全国新增治愈出院病例数超过新增确诊病例数，确诊病例数开始下降。中国国家卫生健康委复函美国卫生与公众服务部，就双方卫生与疫情合作有关安排进一步沟通。

　　（43）2月19日，中共中央总书记习近平主持召开中共中央政治局常务委员会会议，听取疫情防控工作汇报，研究统筹做好疫情防控和经济社会发展工作。

　　（44）2月19日，国家卫生健康委发布《新型冠状病毒肺炎诊疗方案（试行第六版）》。

　　（45）2月19日，武汉市新增治愈出院病例数首次大于新增确诊病例数。

　　（三）第三阶段：本土新增病例数逐步下降至个位数

　　（2月21日至3月17日）

　　湖北省和武汉市疫情快速上升势头均得到遏制，全国除湖北省以外疫情形势总体平稳，3月中旬每日新增病例控制在个位数以内，疫情防控取得阶段性重要成效。根据疫情防控形势发展，中共中央作出统筹疫情防控和经济社会发展、有序复工复产重大决策。

　　（1）2月21日，中共中央总书记习近平主持召开中共中央政治局会议，指出疫情防控工作取得阶段性成效，同时，全国疫情发展拐点尚未到来，湖北省和武汉市防控形势依然严峻复杂；强调要针对不同区域情况，完善差异化防控策略，坚决打好湖北保卫战、武汉保卫战，加强力量薄弱地区防控，全力做好北京疫情防控工作；强调要建立与疫情防控相适应的经济社会运行秩序，有序推动复工复产。

　　（2）2月21日，国务院联防联控机制印发《企事业单位复工复产疫情防控措施指南》，国家卫生健康委发布《新型冠状病毒肺炎防控方案（第五版）》。

　　（3）2月21日起，各地因地制宜，陆续调低省级重大突发公共卫生事件响应级别，逐步取消通行限制。至2月24日，除湖北省、北京市外，其他省份主干公路卡点全部打通，运输秩序逐步恢复。

　　（4）2月23日，中共中央总书记、国家主席、中央军委主席习近平出席统筹推进新冠肺炎疫情防控和经济社会发展工作部署会议，通过视频直接面向全国17万名干部进行动员部署，指出新冠肺炎疫情是新中国成立以来在我国发生的传播速度最快、感染范围最广、防控难度最大的一次重大突发公共卫生事件，这是一次危机，也是一次大考，经过艰苦努力，疫情防控形势积极向好的态势正在拓展；强调疫情形势依然严峻复杂，防控正处在最吃劲的关键阶段，要坚定必胜信念，咬紧牙关，继续毫不放松抓紧抓实抓细各项防控工作；强调要变压力为动力、善于化危为机，有序恢复生产生活秩序，强化“六稳”举措，加大政策调节力度，把发展巨大潜力和强大动能充分释放出来，努力实现今年经济社会发展目标任务。

　　（5）2月24日，中国－世界卫生组织联合专家考察组在北京举行新闻发布会，认为中国在减缓疫情扩散蔓延、阻断病毒人际传播方面取得明显效果，已经避免或至少推迟了数十万人感染新冠肺炎。至2月24日，全国新增确诊病例数已连续5天在1000例以下，现有确诊病例数近一周以来呈现下降趋势，所有省份新增出院病例数均大于或等于新增确诊病例数。

　　（6）2月25日起，全面加强出入境卫生检疫工作，对出入境人员严格健康核验、体温监测、医学巡查、流行病学调查、医学排查、采样监测，防止疫情跨境传播。

　　（7）2月26日，中共中央总书记习近平主持召开中共中央政治局常务委员会会议，指出全国疫情防控形势积极向好的态势正在拓展，经济社会发展加快恢复，同时湖北省和武汉市疫情形势依然复杂严峻，其他有关地区疫情反弹风险不可忽视；强调要继续集中力量和资源，全面加强湖北省和武汉市疫情防控；强调要准确分析把握疫情和经济社会发展形势，紧紧抓住主要矛盾和矛盾的主要方面，确保打赢疫情防控的人民战争、总体战、阻击战，努力实现决胜全面建成小康社会、决战脱贫攻坚目标任务。

　　（8）2月27日，全国除湖北省以外其他省份，湖北省除武汉市以外其他地市，新增确诊病例数首次双双降至个位数。

　　（9）2月28日，国务院联防联控机制印发《关于进一步落实分区分级差异化防控策略的通知》。

　　（10）2月29日，中国－世界卫生组织新型冠状病毒肺炎联合考察报告发布。报告认为，面对前所未知的病毒，中国采取了历史上最勇敢、最灵活、最积极的防控措施，尽可能迅速地遏制病毒传播；令人瞩目的是，在所考察的每一个机构都能够强有力地落实防控措施；面对共同威胁时，中国人民凝聚共识、团结行动，才使防控措施得以全面有效的实施；每个省、每个城市在社区层面都团结一致，帮助和支持脆弱人群及社区。

　　（11）3月2日，中共中央总书记、国家主席、中央军委主席习近平在北京考察新冠肺炎防控科研攻关工作，强调要把新冠肺炎防控科研攻关作为一项重大而紧迫任务，在坚持科学性、确保安全性的基础上加快研发进度，为打赢疫情防控的人民战争、总体战、阻击战提供强大科技支撑；指出尽最大努力挽救更多患者生命是当务之急、重中之重，要加强药物、医疗装备研发和临床救治相结合，切实提高治愈率、降低病亡率；强调要加快推进已有的多种技术路线疫苗研发，争取早日推动疫苗的临床试验和上市使用；指出要把生物安全作为国家总体安全的重要组成部分，加强疫病防控和公共卫生科研攻关体系和能力建设。

　　（12）3月3日，国家卫生健康委发布《新型冠状病毒肺炎诊疗方案（试行第七版）》，在传播途径、临床表现、诊断标准等多个方面作出修改和完善，强调加强中西医结合。

　　（13）3月4日，中共中央总书记习近平主持召开中共中央政治局常务委员会会议，指出要加快建立同疫情防控相适应的经济社会运行秩序，完善相关举措，巩固和拓展来之不易的良好势头；强调要持续用力加强湖北省和武汉市疫情防控工作，继续保持“内防扩散、外防输出”的防控策略。

　　（14）3月6日，中共中央总书记、国家主席、中央军委主席习近平出席决战决胜脱贫攻坚座谈会，指出到2020年现行标准下的农村贫困人口全部脱贫，是中共中央向全国人民作出的郑重承诺，必须如期实现；强调要以更大决心、更强力度推进脱贫攻坚，坚决克服新冠肺炎疫情影响，坚决夺取脱贫攻坚战全面胜利，坚决完成这项对中华民族、对人类都具有重大意义的伟业。

　　（15）3月6日，全国新增本土确诊病例数降至100例以下，11日降至个位数。

　　（16）3月7日，国家卫生健康委发布《新型冠状病毒肺炎防控方案（第六版）》。

　　（17）3月10日，中共中央总书记、国家主席、中央军委主席习近平赴湖北省武汉市考察疫情防控工作，指出经过艰苦努力，湖北和武汉疫情防控形势发生积极向好变化，取得阶段性重要成果，但疫情防控任务依然艰巨繁重，要慎终如始、再接再厉、善作善成，坚决打赢湖北保卫战、武汉保卫战；指出武汉人民识大体、顾大局，不畏艰险、顽强不屈，自觉服从疫情防控大局需要，主动投身疫情防控斗争，作出了重大贡献；指出抗击疫情有两个阵地，一个是医院救死扶伤阵地，一个是社区防控阵地，要充分发挥社区在疫情防控中的重要作用，使所有社区成为疫情防控的坚强堡垒；强调打赢疫情防控人民战争要紧紧依靠人民，把群众发动起来，构筑起群防群控的人民防线。

　　（18）3月11日，世界卫生组织总干事谭德塞表示，新冠肺炎疫情已具有大流行特征。

　　（19）3月11日至17日，全国每日新增本土确诊病例数维持在个位数。总体上，中国本轮疫情流行高峰已经过去，新增发病数持续下降，疫情总体保持在较低水平。

　　（20）3月17日，首批42支国家援鄂医疗队撤离武汉。

　　（四）第四阶段：取得武汉保卫战、湖北保卫战决定性成果

　　（3月18日至4月28日）

　　以武汉市为主战场的全国本土疫情传播基本阻断，离汉离鄂通道管控措施解除，武汉市在院新冠肺炎患者清零，武汉保卫战、湖北保卫战取得决定性成果，全国疫情防控阻击战取得重大战略成果。境内疫情零星散发，境外疫情快速扩散蔓延，境外输入病例造成关联病例传播。中共中央把握疫情形势发展变化，确定了“外防输入、内防反弹”的防控策略，巩固深化国内疫情防控成效，及时处置聚集性疫情，分类推动复工复产，关心关爱境外中国公民。

　　（1）3月18日，中共中央总书记习近平主持召开中共中央政治局常务委员会会议，强调要落实外防输入重点任务，完善应对输入性风险的防控策略和政策举措，决不能让来之不易的疫情防控持续向好形势发生逆转；指出要加强对境外中国公民疫情防控的指导和支持，保护他们的生命安全和身体健康。

　　（2）3月18日，国务院办公厅印发《关于应对新冠肺炎疫情影响强化稳就业举措的实施意见》。

　　（3）3月18日，全国新增本土确诊病例首次实现零报告。至19日，湖北省以外省份连续7日无新增本土确诊病例。

　　（4）3月25日，中共中央总书记习近平主持召开中共中央政治局常务委员会会议，听取疫情防控工作和当前经济形势的汇报，研究当前疫情防控和经济工作。

　　（5）3月25日起，湖北省有序解除离鄂通道管控措施，撤除除武汉市以外地区所有通道（市际、省界通道）检疫站点。湖北省除武汉市以外地区逐步恢复正常生产生活秩序，离鄂人员凭湖北健康码“绿码”安全有序流动。

　　（6）3月25日，23个省份报告了境外输入确诊病例，防止疫情扩散压力依然很大。

　　（7）3月26日，国家主席习近平出席二十国集团领导人特别峰会，发表题为《携手抗疫　共克时艰》的讲话。

　　（8）3月27日，中共中央总书记习近平主持召开中共中央政治局会议，指出要因应国内外疫情防控新形势，及时完善疫情防控策略和应对举措，把重点放在“外防输入、内防反弹”上来，保持疫情防控形势持续向好态势；强调要在疫情防控常态化条件下加快恢复生产生活秩序，力争把疫情造成的损失降到最低限度，努力完成全年经济社会发展目标任务；强调要在做好疫情防控的前提下，支持湖北有序复工复产，做好援企、稳岗、促就业、保民生等工作。

　　（9）3月29日至4月1日，中共中央总书记、国家主席、中央军委主席习近平前往浙江，就统筹推进新冠肺炎疫情防控和经济社会发展工作进行调研，指出要把严防境外疫情输入作为当前乃至较长一段时间疫情防控的重中之重，增强防控措施的针对性和实效性，筑起应对境外疫情输入风险的坚固防线；强调要准确识变、科学应变、主动求变，善于从眼前的危机、眼前的困难中捕捉和创造机遇；强调要在严格做好疫情防控工作的前提下，有力有序推动复工复产提速扩面，积极破解复工复产中的难点、堵点，推动全产业链联动复工。

　　（10）4月1日，中国海关在所有航空、水运、陆路口岸对全部入境人员实施核酸检测。

　　（11）4月4日清明节，举行全国性哀悼活动，全国各地各族人民深切悼念抗击新冠肺炎疫情斗争牺牲烈士和逝世同胞。

　　（12）4月6日，国务院联防联控机制印发《关于进一步做好重点场所重点单位重点人群新冠肺炎疫情防控相关工作的通知》和《新冠病毒无症状感染者管理规范》。

　　（13）4月7日，中央应对疫情工作领导小组印发《关于在有效防控疫情的同时积极有序推进复工复产的指导意见》，国务院联防联控机制印发《全国不同风险地区企事业单位复工复产疫情防控措施指南》。各地做好复工复产相关疫情防控，分区分级恢复生产秩序。

　　（14）4月8日，中共中央总书记习近平主持召开中共中央政治局常务委员会会议，指出要坚持底线思维，做好较长时间应对外部环境变化的思想准备和工作准备；强调“外防输入、内防反弹”防控工作决不能放松；强调要抓好无症状感染者精准防控，把疫情防控网扎得更密更牢，堵住所有可能导致疫情反弹的漏洞；强调要加强陆海口岸疫情防控，最大限度减少境外输入关联本地病例。

　　（15）4月8日起，武汉市解除持续76天的离汉离鄂通道管控措施，有序恢复对外交通，逐步恢复正常生产生活秩序。

　　（16）4月10日，湖北省在院治疗的重症、危重症患者首次降至两位数。

　　（17）4月14日，国务院总理李克强在北京出席东盟与中日韩（10+3）抗击新冠肺炎疫情领导人特别会议并发表讲话，介绍中国统筹推进疫情防控和经济社会发展的经验，提出全力加强防控合作、努力恢复经济发展、着力密切政策协调等合作倡议。

　　（18）4月15日，中共中央总书记习近平主持召开中共中央政治局常务委员会会议，听取疫情防控工作和当前经济形势汇报，研究疫情防控和经济工作。

　　（19）4月17日，中共中央总书记习近平主持召开中共中央政治局会议，强调要抓紧抓实抓细常态化疫情防控，因时因势完善“外防输入、内防反弹”各项措施并切实抓好落实，不断巩固疫情持续向好形势；强调要坚持稳中求进工作总基调，在稳的基础上积极进取，在常态化疫情防控中全面推进复工复产达产，恢复正常经济社会秩序，培育壮大新的增长点增长极，牢牢把握发展主动权。

　　（20）4月17日，武汉市新冠肺炎疫情防控指挥部发布《关于武汉市新冠肺炎确诊病例数确诊病例死亡数订正情况的通报》，对确诊和死亡病例数进行订正。截至4月16日24时，确诊病例核增325例，累计确诊病例数订正为50333例；确诊病例的死亡病例核增1290例，累计确诊病例的死亡数订正为3869例。

　　（21）4月20日至23日，中共中央总书记、国家主席、中央军委主席习近平在陕西考察，指出要坚持稳中求进工作总基调，坚持新发展理念，扎实做好稳就业、稳金融、稳外贸、稳外资、稳投资、稳预期工作，全面落实保居民就业、保基本民生、保市场主体、保粮食能源安全、保产业链供应链稳定、保基层运转任务，努力克服新冠肺炎疫情带来的不利影响，确保完成决战决胜脱贫攻坚目标任务，全面建成小康社会。

　　（22）4月23日，国务院总理李克强主持召开部分省市经济形势视频座谈会，推动做好当前经济社会发展工作。

　　（23）4月26日，武汉市所有新冠肺炎住院病例清零。

　　（24）4月27日，中共中央总书记、国家主席、中央军委主席、中央全面深化改革委员会主任习近平主持召开中央全面深化改革委员会第十三次会议，强调中国疫情防控和复工复产之所以能够有力推进，根本原因是中国共产党的领导和中国社会主义制度的优势发挥了无可比拟的重要作用；强调发展环境越是严峻复杂，越要坚定不移深化改革，健全各方面制度，完善治理体系，促进制度建设和治理效能更好转化融合，善于运用制度优势应对风险挑战冲击。

　　（25）4月27日，经中共中央总书记习近平和中共中央批准，中央指导组离鄂返京。

　　（五）第五阶段：全国疫情防控进入常态化

　　（4月29日以来）

　　境内疫情总体呈零星散发状态，局部地区出现散发病例引起的聚集性疫情，境外输入病例基本得到控制，疫情积极向好态势持续巩固，全国疫情防控进入常态化。加大力度推进复工复产复学，常态化防控措施经受“五一”假期考验。经中共中央批准，国务院联防联控机制派出联络组，继续加强湖北省疫情防控。

　　（1）4月29日，中共中央总书记习近平主持召开中共中央政治局常务委员会会议，指出经过艰苦卓绝的努力，湖北保卫战、武汉保卫战取得决定性成果，全国疫情防控阻击战取得重大战略成果；强调要抓好重点地区、重点群体疫情防控工作，有针对性加强输入性风险防控工作。

　　（2）4月30日，京津冀地区突发公共卫生事件应急响应级别由一级响应调整为二级响应。

　　（3）5月1日，世界卫生组织宣布，鉴于当前国际疫情形势，新冠肺炎疫情仍然构成“国际关注的突发公共卫生事件”。

　　（4）5月2日，湖北省突发公共卫生事件应急响应级别由一级响应调整为二级响应。

　　（5）5月4日，经中共中央批准，国务院联防联控机制设立联络组，赴湖北省武汉市开展工作。

　　（6）5月6日，中共中央总书记习近平主持召开中共中央政治局常务委员会会议，指出在党中央坚强领导和全国各族人民大力支持下，中央指导组同湖北人民和武汉人民并肩作战，下最大气力控制疫情流行，努力守住全国疫情防控第一道防线，为打赢疫情防控的人民战争、总体战、阻击战作出了重要贡献；指出中共中央决定继续派出联络组，加强对湖北省和武汉市疫情防控后续工作指导支持，继续指导做好治愈患者康复和心理疏导工作，巩固疫情防控成果，决不能前功尽弃。

　　（7）5月7日，国务院联防联控机制印发《关于做好新冠肺炎疫情常态化防控工作的指导意见》。

　　（8）5月8日，中共中央召开党外人士座谈会，就新冠肺炎疫情防控工作听取各民主党派中央、全国工商联和无党派人士代表的意见和建议，中共中央总书记习近平主持座谈会并发表重要讲话，强调面对突如其来的疫情，中共中央高度重视，坚持把人民生命安全和身体健康放在第一位，果断采取一系列防控和救治举措，用一个多月的时间初步遏制了疫情蔓延势头，用两个月左右的时间将本土每日新增病例控制在个位数以内，用3个月左右的时间取得了武汉保卫战、湖北保卫战的决定性成果；指出对一个拥有14亿人口的大国来说，这样的成绩来之不易。

　　（9）5月11日至12日，中共中央总书记、国家主席、中央军委主席习近平赴山西，就统筹推进常态化疫情防控和经济社会发展工作、巩固脱贫攻坚成果进行调研，强调要坚持稳中求进工作总基调，坚持新发展理念，坚持以供给侧结构性改革为主线，扎实做好“六稳”工作，全面落实“六保”任务，努力克服新冠肺炎疫情带来的不利影响，在高质量转型发展上迈出更大步伐，确保完成决战决胜脱贫攻坚目标任务，全面建成小康社会。

　　（10）5月14日，中共中央总书记习近平主持召开中共中央政治局常务委员会会议，指出要加强重点地区、重点场所内防反弹工作，近期发生聚集性疫情的地区要有针对性加强防控措施；强调要针对境外疫情的新情况新趋势，采取更加灵活管用的措施，强化外防输入重点领域和薄弱环节。

　　（11）5月15日，中共中央总书记习近平主持召开中共中央政治局会议，讨论国务院拟提请第十三届全国人民代表大会第三次会议审议的《政府工作报告》稿，指出做好今年工作，要紧扣全面建成小康社会目标任务，统筹推进疫情防控和经济社会发展工作，在常态化疫情防控前提下，坚持稳中求进工作总基调，坚持新发展理念，坚持以供给侧结构性改革为主线，坚持以改革开放为动力推动高质量发展，坚决打好三大攻坚战，扎实做好“六稳”工作，全面落实“六保”任务，坚定实施扩大内需战略，维护经济发展和社会稳定大局，确保完成决战决胜脱贫攻坚目标任务，全面建成小康社会。

　　（12）5月18日，国家主席习近平在第73届世界卫生大会视频会议开幕式上发表题为《团结合作战胜疫情　共同构建人类卫生健康共同体》的致辞。

　　（13）5月21日至27日，全国政协十三届三次会议在北京举行。5月22日至28日，十三届全国人大三次会议在北京举行。

　　二、防控和救治两个战场协同作战

　　面对突发疫情侵袭，中国把人民生命安全和身体健康放在第一位，统筹疫情防控和医疗救治，采取最全面最严格最彻底的防控措施，前所未有地采取大规模隔离措施，前所未有地调集全国资源开展大规模医疗救治，不遗漏一个感染者，不放弃每一位病患，实现“应收尽收、应治尽治、应检尽检、应隔尽隔”，遏制了疫情大面积蔓延，改变了病毒传播的危险进程。“通过全面执行（中国）这些措施可以争取到一些时间，即使只有几天或数周，但这对最终减少新冠肺炎感染人数和死亡人数的价值不可估量。”（注1）

　　（一）建立统一高效的指挥体系

　　在以习近平同志为核心的中共中央坚强领导下，建立中央统一指挥、统一协调、统一调度，各地方各方面各负其责、协调配合，集中统一、上下协同、运行高效的指挥体系，为打赢疫情防控的人民战争、总体战、阻击战提供了有力保证。

　　习近平总书记亲自指挥、亲自部署。习近平总书记高度重视疫情防控工作，全面加强集中统一领导，强调把人民生命安全和身体健康放在第一位，提出“坚定信心、同舟共济、科学防治、精准施策”的总要求，明确坚决打赢疫情防控的人民战争、总体战、阻击战。习近平总书记主持召开14次中央政治局常委会会议、4次中央政治局会议以及中央全面依法治国委员会会议、中央网络安全和信息化委员会会议、中央全面深化改革委员会会议、中央外事工作委员会会议、党外人士座谈会等会议，听取中央应对疫情工作领导小组和中央指导组汇报，因时因势调整防控策略，对加强疫情防控、开展国际合作等进行全面部署；在北京就社区防控、防疫科研攻关等进行考察，亲临武汉一线视察指导，赴浙江、陕西、山西就统筹推进常态化疫情防控和经济社会发展工作、巩固脱贫攻坚成果进行考察调研；时刻关注疫情动态和防控进展，及时作出决策部署。

　　加强统筹协调、协同联动。中共中央政治局常委、国务院总理、中央应对疫情工作领导小组组长李克强主持召开30余次领导小组会议，研究部署疫情防控和统筹推进经济社会发展的重大问题和重要工作，赴北京、武汉等地和中国疾控中心、中国医学科学院病原生物学研究所、北京西站、首都机场及疫情防控国家重点医疗物资保障调度等平台考察调研。中央指导组指导湖北省、武汉市加强防控工作，以争分夺秒的战时状态开展工作，有力控制了疫情流行，守住了第一道防线。国务院联防联控机制发挥协调作用，持续召开例会跟踪分析研判疫情形势，加强医务人员和医疗物资调度，根据疫情发展变化相应调整防控策略和重点工作。国务院复工复产推进工作机制，加强复工复产统筹指导和协调服务，打通产业链、供应链堵点，增强协同复工复产动能。

　　各地方各方面守土有责、守土尽责。全国各省、市、县成立由党政主要负责人挂帅的应急指挥机制，自上而下构建统一指挥、一线指导、统筹协调的应急决策指挥体系。在中共中央统一领导下，各地方各方面坚决贯彻中央决策部署，有令必行、有禁必止，严格高效落实各项防控措施，全国形成了全面动员、全面部署、全面加强，横向到边、纵向到底的疫情防控局面。

　　（二）构建全民参与严密防控体系

　　针对春节期间人员密集、流动性大的特点，中国迅速开展社会动员、发动全民参与，坚持依法、科学、精准防控，在全国范围内实施史无前例的大规模公共卫生应对举措，通过超常规的社会隔离和灵活、人性化的社会管控措施，构建联防联控、群防群控防控体系，打响抗击疫情人民战争，通过非药物手段有效阻断了病毒传播链条。

　　采取有力措施坚决控制传染源。以确诊患者、疑似患者、发热患者、确诊患者的密切接触者等“四类人员”为重点，实行“早发现、早报告、早隔离、早治疗”和“应收尽收、应治尽治、应检尽检、应隔尽隔”的防治方针，最大限度降低传染率。关闭离汉通道期间，武汉对全市421万户居民集中开展两轮拉网式排查，以“不落一户、不漏一人”标准实现“存量清零”，确保没有新的潜在感染源发生。持续提升核酸检测能力，增强试剂盒供应能力，扩充检测机构，缩短检测周期，确保检测质量，实现“应检尽检”“即收即检”。湖北省检测周期从2天缩短到4－6小时，日检测量由疫情初期的300人份提升到4月中旬的5万人份以上，缩短了患者确诊时间，降低了传播风险。在全国范围内排查“四类人员”，以社区网格为基础单元，采取上门排查与自查自报相结合的方式展开地毯式排查。全面实行各类场所体温筛查，强化医疗机构发热门诊病例监测和传染病网络直报，实行2小时网络直报、12小时反馈检测结果、24小时内完成现场流行病学调查，及时发现和报告确诊病例和无症状感染者。加强流行病学追踪调查，精准追踪和切断病毒传播途径，截至5月31日，全国累计追踪管理密切接触者74万余人。

　　第一时间切断病毒传播链。对湖北省、武汉市对外通道实施最严格的封闭和交通管控，暂停武汉及湖北国际客运航班、多地轮渡、长途客运、机场、火车站运营，全国暂停入汉道路水路客运班线发班，武汉市及湖北省多地暂停市内公共交通，阻断疫情向全国以及湖北省内卫生基础设施薄弱的农村地区扩散。对湖北以外地区实施差异化交通管控，湖北省周边省份筑牢环鄂交通管控“隔离带”，防止湖北省疫情外溢蔓延。全国其他地区实行分区分级精准防控，对城乡道路运输服务进行动态管控，加强国内交通卫生检疫。采取有效措施避免人员聚集和交叉感染，延长春节假期，取消或延缓各种人员聚集性活动，各类学校有序推迟开学；关闭影院、剧院、网吧以及健身房等场所；对车站、机场、码头、农贸市场、商场、超市、餐馆、酒店、宾馆等需要开放的公共服务类场所，以及汽车、火车、飞机等密闭交通工具，落实环境卫生整治、消毒、通风、“进出检”、限流等措施，进入人员必须测量体温、佩戴口罩；推行政务服务网上办、预约办，推广无接触快递等“不见面”服务，鼓励民众居家和企业远程办公，有效减少人员流动和聚集；在公共场所设置“一米线”并配以明显标识，避免近距离接触。全国口岸实施严格的出入境卫生检疫，防范疫情通过口岸扩散蔓延。实施最严边境管控，取消非紧急非必要出国出境活动。

　　牢牢守住社区基础防线。城乡社区是疫情联防联控的第一线，是外防输入、内防扩散的关键防线。充分发挥基层主体作用，加强群众自治，实施社区封闭式、网格化管理，把防控力量、资源、措施向社区下沉，组建专兼结合工作队伍，充分发挥街道（乡镇）和社区（村）干部、基层医疗卫生机构医务人员、家庭医生团队作用，将一个个社区、村庄打造成为严密安全的“抗疫堡垒”，把防控有效落实到终端和末梢。按照“追踪到人、登记在册、社区管理、上门观察、规范运转、异常就医”的原则，依法对重点人群进行有效管理，开展主动追踪、人员管理、环境整治和健康教育。武汉市全面实施社区24小时封闭管理，除就医和防疫相关活动外一律禁止出入，由社区承担居民生活保障。其他地方对城市社区、农村村落普遍实施封闭式管理，人员出入检查登记、测量体温。加强居民个人防护，广泛开展社会宣传，强化个体责任意识，自觉落实居家隔离以及跨地区旅行后隔离14天等防控要求，严格执行外出佩戴口罩、保持社交距离、减少聚集等防护措施，养成勤洗手、常通风等良好生活习惯。大力开展爱国卫生运动，提倡文明健康、绿色环保的生活方式。

　　实施分级、分类、动态精准防控。全国推行分区分级精准施策防控策略，以县域为单位，依据人口、发病情况综合研判，划分低、中、高疫情风险等级，分区分级实施差异化防控，并根据疫情形势及时动态调整名单，采取对应防控措施。低风险区严防输入，全面恢复生产生活秩序；中风险区外防输入、内防扩散，尽快全面恢复生产生活秩序；高风险区内防扩散、外防输出、严格管控，集中精力抓疫情防控。本土疫情形势稳定后，以省域为单元在疫情防控常态化条件下加快恢复生产生活秩序，健全及时发现、快速处置、精准管控、有效救治的常态化防控机制。全力做好北京市疫情防控，确保首都安全。做好重点场所、重点单位、重点人群聚集性疫情防控和处置，加强老年人、儿童、孕产妇、学生、医务人员等重点人群健康管理，加强医疗机构、社区、办公场所、商场超市、客运场站、交通运输工具，托幼机构、中小学校、大专院校以及养老机构、福利院、精神卫生医疗机构、救助站等特殊场所的管控，覆盖全人群、全场所、全社区，不留死角、不留空白、不留隐患。针对输入性疫情，严格落实国境卫生检疫措施，强化从“国门”到“家门”的全链条、闭环式管理，持续抓紧抓实抓细外防输入、内防反弹工作。

　　为疫情防控提供有力法治保障。依法将新冠肺炎纳入《中华人民共和国传染病防治法》规定的乙类传染病并采取甲类传染病的预防、控制措施，纳入《中华人民共和国国境卫生检疫法》规定的检疫传染病管理，同时做好国际国内法律衔接。一些地方人大常委会紧急立法，在国家法律和法规框架下授权地方政府在医疗卫生、防疫管理等方面，规定临时性应急行政管理措施。严格执行传染病防治法及其实施办法等法律法规，出台依法防控疫情、依法惩治违法犯罪、保障人民生命健康安全的意见，加强治安管理、市场监管，依法惩处哄抬物价、囤积居奇、制假售假等破坏疫情防控的违法犯罪行为，强化防疫物资质量和价格监管，加大打击虚假违法广告力度，保障社会稳定有序。加强疫情防控期间行政执法监督，严格规范执法，公正文明执法，依法化解与疫情相关的法律纠纷，为疫情防控和企业复工复产提供法律保障和服务。加强普法宣传，引导公众依法行事。

　　遵循科学规律开展防控。新冠病毒是新病毒，对其认识需要有个过程。积极借鉴以往经验，紧密结合中国国情，遵循流行病学规律，探索行之有效的方法手段，用中国办法破解疫情防控难题。注重发挥病毒学、流行病学、临床医学等领域专家作用，及时开展疫情形势分析研判，提出防控策略建议，充分尊重专家意见，增强疫情防控的科学性专业性。秉持科学态度，加强病毒感染、致病机理、传播途径、传播能力等研究，与世界卫生组织及其他国家和地区保持沟通交流。随着对病毒认识的不断深化，及时调整和优化工作措施，不断提升防控水平。根据疫情形势变化和评估结果，先后制修订6版新冠肺炎防控方案，科学规范开展病例监测、流行病学调查、可疑暴露者和密切接触者管理以及实验室检测等工作。针对重点人群、重点场所、重点单位发布15项防控技术方案、6项心理疏导工作方案，并细化形成50项防控技术指南，进一步提高疫情防控的科学性精准性。

　　（三）全力救治患者、拯救生命

　　医疗救治始终以提高收治率和治愈率、降低感染率和病亡率的“两提高”“两降低”为目标，坚持集中患者、集中专家、集中资源、集中救治“四集中”原则，坚持中西医结合，实施分类救治、分级管理。对重症患者，调集最优秀的医生、最先进的设备、最急需的资源，不惜一切代价进行救治，大幅度降低病亡率；对轻症患者及早干预，尽可能在初期得以治愈，大幅度降低转重率。

　　集中优势资源加强重症救治。疫情突发导致武汉市医疗资源挤兑。针对疫情初期患者数量激增与床位资源不足的突出矛盾，集中资源和力量在武汉市建设扩充重症定点医院和救治床位，将全部重症危重症患者集中到综合实力最强且具备呼吸道传染性疾病收治条件的综合医院集中开展救治。建成火神山、雷神山两座各可容纳1000多张床位的传染病专科医院，改扩建一批定点医院，改造一批综合医院，使重症床位从1000张左右迅速增加至9100多张，解决了重症患者大规模收治难题。优化重症救治策略，制定个体化医疗救治方案。建立专家巡查制度，定期组织专家团队对武汉市定点医院重症患者救治进行巡诊，评估患者病情和治疗方案。针对超过80%的重症患者合并严重基础性疾病情况，实行“一人一策”，建立感染、呼吸、重症、心脏、肾脏等多学科会诊制度，并制定重症、危重症护理规范，推出高流量吸氧、无创和有创机械通气、俯卧位通气等措施。严格落实疑难危重症患者会诊制度、死亡病例讨论制度等医疗质量安全核心制度，强化对治愈出院患者健康监测，确保重症患者救治质量。开展康复者恢复期血浆采集和临床治疗工作，建立应急储备库，截至5月31日，全国共采集恢复期血浆2765人次，1689人次患者接受恢复期血浆治疗，取得较好治疗效果。

　　对轻症患者及早干预治疗。及时收治轻症患者，及早实施医疗干预，尽量减少轻症转为重症。完善临床救治体系，全国共指定1万余家定点医院，对新冠肺炎患者实行定点集中治疗。建立全国医疗救治协作网络，通过远程会诊方式提供技术支持。武汉市针对患者数量急剧增长、80%左右是轻症的情况，集中力量将一批体育场馆、会展中心等改造成16家方舱医院，床位达到1.4万余张，使轻症患者应收尽收、应治尽治，减少了社区感染传播，减少了轻症向重症转化。16家方舱医院累计收治患者1.2万余人，累计治愈出院8000余人、转院3500余人，实现“零感染、零死亡、零回头”。方舱医院是阻击重大传染病的重大创新，使“应收尽收”“床位等人”成为现实，有力扭转了防控形势。英国《柳叶刀》社论认为，“中国建造的方舱庇护医院对于缓解医疗卫生系统所承受的巨大压力有着至关重要的作用”。（注2）

　　及时总结推广行之有效的诊疗方案。坚持边实践、边研究、边探索、边总结、边完善，在基于科学认知和证据积累的基础上，将行之有效的诊疗技术和科技研究成果纳入诊疗方案。先后制修订7版新冠肺炎诊疗方案，3版重型、危重型病例诊疗方案，2版轻型、普通型管理规范，2版康复者恢复期血浆治疗方案，1版新冠肺炎出院患者主要功能障碍康复治疗方案，提高了医疗救治工作的科学性和规范性。最新的第7版新冠肺炎诊疗方案增加病理改变内容，增补和调整临床表现、诊断标准、治疗方法和出院标准等，并纳入无症状感染者可能具有感染性、康复者恢复期血浆治疗等新发现。目前，第7版诊疗方案已被多个国家借鉴和采用。强化治愈出院患者隔离管理和健康监测，加强复诊复检和康复，实现治疗、康复和健康监测一体化全方位医疗服务。注重孕产妇、儿童等患者差异性诊疗策略，实现不同人群诊疗方案的全覆盖。

　　充分发挥中医药特色优势。坚持中西医结合、中西药并用，发挥中医药治未病、辨证施治、多靶点干预的独特优势，全程参与深度介入疫情防控，从中医角度研究确定病因病基、治则治法，形成了覆盖医学观察期、轻型、普通型、重型、危重型、恢复期发病全过程的中医诊疗规范和技术方案，在全国范围内全面推广使用。中医医院、中医团队参与救治，中医医疗队整建制接管定点医院若干重症病区和方舱医院，其他方舱医院派驻中医专家。中医药早期介入、全程参与、分类救治，对轻症患者实施中医药早介入早使用；对重症和危重症患者实行中西医结合；对医学观察发热病人和密切接触者服用中药提高免疫力；对出院患者实施中医康复方案，建立全国新冠肺炎康复协作网络，提供康复指导。中医药参与救治确诊病例的占比达到92%。湖北省确诊病例中医药使用率和总有效率超过90%。筛选金花清感颗粒、连花清瘟胶囊/颗粒、血必净注射液和清肺排毒汤、化湿败毒方、宣肺败毒方等“三药三方”为代表的针对不同类型新冠肺炎的治疗中成药和方药，临床疗效确切，有效降低了发病率、转重率、病亡率，促进了核酸转阴，提高了治愈率，加快了恢复期康复。

　　实施患者免费救治。及时预拨疫情防控资金，确保患者不因费用问题影响就医，确保各地不因资金问题影响医疗救治和疫情防控。截至5月31日，全国各级财政共安排疫情防控资金1624亿元。及时调整医保政策，明确确诊和疑似患者医疗保障政策，对确诊和疑似患者实行“先救治，后结算”。对新冠肺炎患者（包括确诊和疑似患者）发生的医疗费用，在基本医保、大病保险、医疗救助等按规定支付后，个人负担部分由财政给予补助。异地就医医保支付的费用由就医地医保部门先行垫付。截至5月31日，全国确诊住院患者结算人数5.8万人次，总医疗费用13.5亿元，确诊患者人均医疗费用约2.3万元。其中，重症患者人均治疗费用超过15万元，一些危重症患者治疗费用几十万元甚至上百万元，全部由国家承担。

　　加强医疗机构感染控制和医务人员防护。制定感染控制技术指南和制度文件，明确医疗机构重点区域、就诊流程“三区两通道”建筑布局要求。加强对医务人员的感染控制培训，开展全国督导，确保感染控制措施落实。对疫情严重、院内感染风险高、医疗救治压力大的重点地区重点医院，有针对性地开展指导。加强医疗废物分类收集、运送贮存，做好病亡者遗体处置。在援鄂援汉医疗队中配置感染控制专家，全国支援湖北省和武汉市的医务人员没有感染病例。2月份以后，全国医务人员感染病例报告数明显减少。关心关爱医务人员，制定一系列保障政策，开展心理疏导，妥善安排轮换休整，缓解身体和心理压力，保持一线医务人员战斗力。

　　（四）依法及时公开透明发布疫情信息

　　在全力做好疫情防控的同时，中国以对生命负责、对人民负责、对历史负责、对国际社会负责的态度，建立最严格且专业高效的信息发布制度，第一时间发布权威信息，速度、密度、力度前所未有。持续、权威、清晰的疫情信息，有效回应了公众关切、凝聚了社会共识，为其他国家提供了参考和借鉴。

　　建立严格的疫情发布机制。依法、及时、公开、透明发布疫情信息，制定严格规定，坚决防止瞒报、迟报、漏报。武汉市从2019年12月31日起依法发布疫情信息，并逐步增加信息发布频次。2020年1月21日起，国家卫生健康委每日在官方网站、政务新媒体平台发布前一天全国疫情信息，各省级卫生健康部门每日统一发布前一天本省份疫情信息。2月3日起，国家卫生健康委英文网站同步发布相关数据。

　　建立分级分层新闻发布制度。坚持国家和地方相结合、现场发布与网上发布相结合，建立多层次多渠道多平台信息发布机制，持续发布权威信息，及时回应国内外关注的疫情形势、疫情防控、医疗救治、科研攻关等热点问题。截至5月31日，国务院联防联控机制、国务院新闻办公室共举行新闻发布会161场，邀请50多个部门490余人次出席发布会，回答中外媒体1400多个提问；湖北省举行103场新闻发布会，其他省份共举行1050场新闻发布会。

　　依法适时订正病例数据。本土疫情得到控制后，为确保公开透明、数据准确，武汉市针对疫情早期因收治能力不足导致患者在家中病亡、医院超负荷运转、死亡病例信息登记不全等原因，客观上存在迟报、漏报、误报现象，根据相关法律规定，在深入开展涉疫大数据与流行病学调查的基础上，对确诊和死亡病例数进行了订正，并向社会公开发布。

　　多渠道多平台传播信息。国家卫生健康委中、英文官方网站和政务新媒体平台设置疫情防控专题页面，发布每日疫情信息，解读政策措施，介绍中国抗疫进展，普及科学防控知识，澄清谣言传言。各省（自治区、直辖市）政府网站及政务新媒体平台及时发布本地疫情信息和防控举措。大力开展应急科普，通过科普专业平台、媒体和互联网面向公众普及科学认知、科学防治知识，组织权威专家介绍日常防控常识，引导公众理性认识新冠肺炎疫情，做好个人防护，消除恐慌恐惧。加强社会舆论引导，各类媒体充分传递抗击疫情正能量，同时发挥舆论监督作用，推动解决疫情防控中出现的问题。

　　（五）充分发挥科技支撑作用

　　科学技术是人类同疾病较量的锐利武器，人类战胜大灾大疫离不开科学发展和技术创新。面对人类未知的新冠病毒，中国坚持以科学为先导，充分运用近年来科技创新成果，组织协调全国优势科研力量，以武汉市为主战场，统筹全国和疫情重灾区，根据疫情发展不同阶段确定科研攻关重点，坚持科研、临床、防控一线相互协同和产学研各方紧密配合，为疫情防控提供了有力科技支撑。

　　实施科研应急攻关。遵循安全、有效、可供的原则，加快推进药物、疫苗、新型检测试剂等研发和应用。适应疫情防控一线的紧迫需求，围绕“可溯、可诊、可治、可防、可控”，坚持产学研用相结合，聚焦临床救治和药物、疫苗研发、检测技术和产品、病毒病原学和流行病学、动物模型构建5大主攻方向，组织全国优势力量开展疫情防控科技攻关，加速推进科技研发和应用，部署启动83个应急攻关项目。按照灭活疫苗、重组蛋白疫苗、减毒流感病毒载体疫苗、腺病毒载体疫苗、核酸疫苗等5条技术路线开展疫苗研发。目前，已有4种灭活疫苗和1种腺病毒载体疫苗获批开展临床试验，总体研发进度与国外持平，部分技术路线进展处于国际领先。组织科研团队开展科学溯源研究。

　　坚持科研攻关和临床救治、防控实践相结合。第一时间研发出核酸检测试剂盒，推出一批灵敏度高、操作便捷的检测设备和试剂，检测试剂研发布局涵盖核酸检测、基因测序、免疫法检测等多个技术路径。坚持“老药新用”基本思路，积极筛选有效治疗药物，探索新的治疗手段，在严谨的体外研究和机制研究基础上，不断总结救治经验，推动磷酸氯喹、恢复期血浆、托珠单抗和中医药方剂、中成药等10种药物或治疗手段进入诊疗方案，获得4项临床批件，形成5项指导意见或专家共识。开展试验性临床治疗，加快推广应用临床验证有效的诊疗方法和药物。强化实验室生物安全监管，加强新冠病毒临床检测血液样本和实验室检测生物样本管理。

　　运用大数据、人工智能等新技术开展防控。充分利用大数据、人工智能等新技术，进行疫情趋势研判，开展流行病学调查，努力找到每一个感染者、穷尽式地追踪密切接触者并进行隔离。建立数据库，依法开展疫情防控风险数据服务，对不同风险人群进行精准识别，预判不同地区疫情风险，为促进人员有序流动和复工复产提供服务。通过5G视频实时对话平台，偏远山区的流行病学调查团队可以与几千公里之外的高级别专家实时互动交流。经公民个人授权，推广个人“健康码”“通信大数据行程卡”作为出行、复工复产复学、日常生活及出入公共场所的凭证，根据查询结果进行管控通行和分类处置，实现分区分级的精准识别、精准施策和精准防控。利用大数据技术绘制“疫情地图”，通过社区名称、地址和位置，标明疫情传播具体地点、距离、人数等，为公众防范传染提供方便。

　　此次新冠肺炎疫情防控，为应对重大突发公共卫生事件积累了宝贵经验，同时也暴露出国家公共卫生应急管理体系存在的不足。中国将认真总结疫情防控和医疗救治经验教训，研究采取一系列重要举措，补短板、强弱项。改革完善疾病预防控制体系，建设平战结合的重大疫情防控救治体系，健全应急物资保障体系，加强构建关键核心技术攻关新型举国体制，深入开展爱国卫生运动，不断完善公共卫生体系，切实提高应对突发重大公共卫生事件的能力和水平，更好维护人民生命安全和身体健康。

　　三、凝聚抗击疫情的强大力量

　　面对未知病毒突然袭击，中国坚持人民至上、生命至上，举全国之力，快速有效调动全国资源和力量，不惜一切代价维护人民生命安全和身体健康。中国共产党以人民为中心的执政理念，中国集中力量办大事的制度特点，改革开放40多年来特别是中共十八大以来积累的雄厚综合国力和国家治理现代化建设的显著成效，中华民族同舟共济、守望相助的文化底色，中国人民深厚的家国情怀、天下情怀，汇聚成抗击疫情的强大合力。

　　（一）人的生命高于一切

　　在新冠肺炎疫情突袭，人民生命安全和身体健康受到严重威胁的重大时刻，中国共产党和中国政府始终以对人民负责、对生命负责的鲜明态度，准确分析和把握形势，既多方考量、慎之又慎，又及时出手、坚决果敢，以非常之举应对非常之事，全力保障人民生命权、健康权。

　　在人民生命和经济利益之间果断抉择生命至上。疫情暴发后，以宁可一段时间内经济下滑甚至短期“停摆”，也要对人民生命安全和身体健康负责的巨大勇气，对湖北省和武汉市果断采取史无前例的全面严格管控措施。同时，在全国范围内严控人员流动，延长春节假期，停止人员聚集性活动，决定全国企业和学校延期开工开学，迅速遏制疫情的传播蔓延，避免更多人受到感染。英国《柳叶刀》社论认为，“中国的成功也伴随着巨大的社会和经济代价，中国必须做出艰难的决定，从而在国民健康与经济保护之间获得最佳平衡”。（注3）在疫情防控的关键阶段，准确把握疫情形势变化，作出统筹推进疫情防控和经济社会发展的重大决策，有序恢复生产生活秩序，推动落实分区分级精准复工复产，最大限度保障民生和人民正常生产生活。随着本土疫情防控取得重大战略成果，及时采取“外防输入、内防反弹”的防控策略，坚决防止来之不易的持续向好形势发生逆转，坚决防止人民生命安全再次面临病毒威胁。

　　不惜一切代价抢救生命。疫情初期，病毒感染者急剧增多，中国把提高治愈率、降低病亡率作为首要任务，快速充实医疗救治力量，把优质资源集中到救治一线。采取积极、科学、灵活的救治策略，慎终如始、全力以赴救治每一位患者，从出生仅30个小时的婴儿至100多岁的老人，不计代价抢救每一位患者的生命。为了抢救病患，医务人员冒着被感染的风险采集病毒样本，没有人畏难退缩。为满足重症患者救治需要，想尽一切办法筹措人工膜肺（ECMO）设备，能买尽买，能调尽调。武汉市重症定点医院累计收治重症病例9600多例，转归为治愈的占比从14%提高到89%，超过一般病毒性肺炎救治平均水平。对伴有基础性疾病的老年患者，一人一案、精准施策，只要有一丝希望绝不轻易放弃，只要有抢救需要，人员、药品、设备、经费全力保障。疫情发生以来，湖北省成功治愈3000余位80岁以上、7位百岁以上新冠肺炎患者，多位重症老年患者是从死亡线上抢救回来的。一位70岁老人身患新冠肺炎，10多名医护人员精心救护几十天，终于挽回了老人生命，治疗费用近150万元全部由国家承担。

　　关心关爱海外中国公民。国家时刻挂念海外中国公民的安危，敦促、支持有关国家政府采取有效措施保障当地华侨、留学生、中资机构人员等安全。派出医疗专家组、工作组，开设远程医疗服务平台，为海外中国公民提供科学专业的疫情防控指导。协调外方全力救治在国外确诊感染的中国公民，充分调动国内专家、援外医疗队等资源，积极支持配合外方开展救治。驻外使领馆尽力履行领事保护职能，通过各种渠道宣介疫情防护知识，向留学生发放100多万份“健康包”。协助在海外确有困难的中国公民有序回国。

　　以国之名悼念逝者。4月4日清明节，中国举行全国性哀悼活动，深切悼念抗击疫情斗争牺牲烈士和逝世同胞，为没有等来春天的生命默哀，向所有用生命守护生命的英雄致敬。从最高领导人到普通民众，14亿中国人民以最深的怀念为牺牲烈士和逝世同胞送行。中国以国家之名和最高仪式祭奠逝者，是国家对人民个体尊严与生命的尊重与敬畏，是14亿中国人民集体情感背后的团结和力量。

　　（二）举全国之力抗击疫情

　　一方有难，八方支援。疫情发生后，全国上下紧急行动，依托强大综合国力，开展全方位的人力组织战、物资保障战、科技突击战、资源运动战，全力支援湖北省和武汉市抗击疫情，在最短时间集中最大力量阻断疫情传播。“中方行动速度之快、规模之大，世所罕见，展现出中国速度、中国规模、中国效率”。（注4）

　　开展新中国成立以来规模最大的医疗支援行动。调动全国医疗资源和力量，全力支持湖北省和武汉市医疗救治。自1月24日除夕至3月8日，全国共调集346支国家医疗队、4.26万名医务人员、900多名公共卫生人员驰援湖北。19个省份以对口支援、以省包市的方式支援湖北省除武汉市以外16个地市，各省在发生疫情、防控救治任务十分繁重的情况下，集中优质医疗资源支援湖北省和武汉市。人民解放军派出4000多名医务人员支援湖北，承担火神山医院等3家医疗机构的医疗救治任务，空军出动运输机紧急运送医疗物资。各医疗队从接受指令到组建2小时内完成，24小时内抵达，并自带7天防护物资，抵达后迅速开展救治。在全国紧急调配全自动测温仪、负压救护车、呼吸机、心电监护仪等重点医疗物资支援湖北省和武汉市。从全国调集4万名建设者和几千台机械设备，仅用10天建成有1000张病床的火神山医院，仅用12天建成有1600张病床的雷神山医院。短短10多天建成16座方舱医院，共有1.4万余张床位。加强临床血液供应，10个省份无偿支援湖北省红细胞4.5万单位，血小板1762个治疗量，新鲜冰冻血浆137万毫升（不含恢复期血浆）。大规模、强有力的医疗支援行动，有力保障了湖北省和武汉市救治，极大缓解了重灾区医疗资源严重不足的压力。

　　大力加强医疗物资生产供应和医疗支持服务。疫情防控阻击战，也是后勤保障战。疫情初期，武汉市医疗防护物资极度短缺，为了节省防护用品、争分夺秒抢救病患，一线医护人员克服困难，最大限度地延长防护用品使用时间。为尽快解决医疗资源短缺和病患急剧增多的突出矛盾，中国充分发挥制造业门类全、韧性强和产业链完整配套的优势，克服春节假期停工减产等不利因素，开足马力，深挖潜力，全力保障上下游原料供应和物流运输，保证疫情防控物资的大规模生产与配送。医疗企业克服工人返岗不足等困难，以最快速度恢复医疗用品生产，最大限度扩大产能。其他行业企业迅速调整转产，生产口罩、防护服、消毒液、测温仪等防疫物资，有效扩大了疫情防控物资的生产供应。快速启动防控医疗物资应急审批程序，全面加强质量安全监管，确保以最快的速度批准上市、促产保供，截至5月31日，共应急批准17个药物和疫苗的19件临床试验申请，附条件批准2个疫情防控用药上市。在各方共同努力下，医用物资产能不断提升，医用物资保供实现从“紧缺”到“紧平衡”“动态平衡”“动态足额供应”的跨越式提升。2月初，医用非N95口罩、医用N95口罩日产量分别为586万只、13万只，到4月底分别超过2亿只、500万只。畅通供应链条和物流渠道，建立联保联供协作机制，源源不断地把全国支援物资运送到疫情防控重点地区。

　　统筹协调生活物资保障。离汉通道关闭后，武汉市近千万人居家隔离，每天需要消耗大量的粮食、蔬菜、肉蛋奶。加强联动协调，建立央地协同、政企联动的9省联保联供协作和500家应急保供企业调运机制，加大粮油供应力度，投放中央冻猪肉储备，提升蔬菜大省产品供应能力，组织紧急物资运输队伍，全力保障湖北省特别是武汉市居民生活必需品的生产、库存、供应和价格稳定。1月27日至3月19日，全国通过铁路、公路、水运、民航、邮政快递等运输方式向湖北地区运送防疫物资和生活物资92.88万吨，运送电煤、燃油等生产物资148.7万吨，煤、电、油、气、热等能源供应充足，保障了湖北省、武汉市社会正常运转和隔离措施顺利实施。武汉市将生活物资配送纳入社区服务，打通生活物资配送从商场、超市到小区的最后环节，通过无接触配送方式将经过检疫、符合防疫标准的蔬菜直送社区，保障了隔离期间居民生活需要和防疫安全。

　　社会力量广泛参与。工会、共青团、妇联等人民团体和群众组织，组织动员所联系群众积极投身疫情防控。城乡居民、企业、社会组织等纷纷捐款捐物、献出爱心。各级慈善组织、红十字会加强捐赠资金和物资的调配和拨付，将捐赠款物重点投向湖北省和武汉市等疫情严重地区。截至5月31日，累计接受社会捐赠资金约389.3亿元、物资约9.9亿件，累计拨付捐款资金约328.3亿元、物资约9.4亿件。

　　疫情发生后，港澳台同胞和海外侨胞通过各种方式和渠道伸出援手，积极捐款和捐赠各类防疫物资，体现了浓浓的同胞亲情，体现了海内外中华儿女守望相助、共克时艰的凝聚力向心力。

　　（三）平衡疫情防控与经济社会民生

　　在毫不放松加强疫情防控的同时，稳妥有序放开经济和社会活动，做好“六稳”工作，落实“六保”任务，形成同疫情防控相适应的经济社会运行秩序，努力将疫情对经济社会发展的冲击和影响降到最低，为抗击疫情提供有力的物资保障和社会保障。

　　保持社会稳定、有序运转。着力加强社会安全稳定工作，加强社会治安管理，强化防疫物资质量和价格监管，维护市场秩序和社会稳定。及时出台受疫情影响困难群众兜底保障政策，有效保障基本生活。将心理危机干预纳入疫情防控，妥善处理疫情防控中思想和心理问题，加强思想引导和心理疏导，培育理性平和、积极健康的心态，及时预防化解涉疫矛盾纠纷。疫情大考下，在交通管制、全民居家隔离等严格管控措施的情况下，不论是城市还是农村，水、电、燃气、通信不停，生活物资供应不断，社会秩序不乱，食品、药品、能源、基础工业品、基本公共服务等关系国计民生的重点行业有序运转，14亿人民的基本民生得到有效保障，经济社会大局保持了稳定有序。

　　有序推动复工复产。密集制定出台多项政策，为企业特别是中小企业和个体工商户减负纾困，实施减费降税，增加财政补贴，加大金融支持，减负稳岗扩就业，优化政府服务。各地方及时制定实施细则，将疫情防控、公共事业运行、群众生活必需等领域的1万多家企业列为重点，通过租用专车、专列、包机等方式“点对点”“一站式”帮助农民工返岗，并从个人防护物资、人流、物流等方面为企业复工提供全方位服务。针对公共交通运输、餐饮、住宿、旅游、体育、娱乐等受疫情影响较大的行业，采取免征增值税等税收优惠政策。阶段性减免企业社保费，缓缴住房公积金，免收公路通行费，降低企业用电用气价格，减轻小微企业和个体工商户房租负担。对中小微企业贷款实施临时性延期还本付息、新增优惠利率贷款。支持大学生、农民工等重点群体创业就业，扩大中小微企业稳岗返还政策受益面，发力稳就业，促进中小企业发展。用好用足出口退税、出口信用保险政策，扩大出口信贷投放，开拓多元化市场，加快压减外资准入负面清单，持续扩大外资市场准入，为企业“补血”“减负”“拓空间”。国有企业发挥主力军作用，带动上下游产业和中小企业全面复工复产。截至4月底，全国规模以上工业企业复工率超过99%，中小微企业复工率达到88.4%，重大项目复工率超过95%；湖北全省规模以上工业企业复工率、员工到岗率分别达到98.2%、92.1%，整体接近全国平均水平。一批国家重点科技专项、超级民生工程、重大标志性外资项目重现往日繁忙景象。中国经济运行加快回归常态，经济活力正在快速释放。

　　公众生活逐步恢复。随着疫情防控形势积极向好，公众日常生活逐步恢复。公共交通全面恢复运行，餐饮门店有序开放堂食。“五一”假期重新绽放活力，全国铁路、道路、水路、民航累计发送旅客1.21亿人次，全国累计接待国内游客1.15亿人次，实现国内旅游收入475.6亿元，经受住了疫情和假期的双重考验。在落实防控措施前提下，全面开放商场、超市、宾馆、餐馆等生活场所。全国分批分次复学复课，截至5月31日，各省（自治区、直辖市）和新疆生产建设兵团中小学部分学段均已开学，共有1.63亿学生（含幼儿园）返校。中国社会正在恢复往常热闹景象，人气日益回暖，消费逐步复苏。

　　（四）14亿中国人民坚韧奉献守望相助

　　国家兴亡，匹夫有责。14亿中国人民，不分男女老幼，不论岗位分工，都自觉投入抗击疫情的人民战争，坚韧团结、和衷共济，凝聚起抗击疫情的磅礴力量。14亿中国人民都是抗击疫情的伟大战士。

　　医务工作者白衣执甲、逆行出征。从年逾古稀的院士专家，到90后、00后的年轻医护人员，面对疫情义无反顾、坚定前行。54万名湖北省和武汉市医务人员冲锋在前，4万多名军地医务人员第一时间驰援湖北省和武汉市，数百万名医务人员战斗在全国抗疫一线。他们以对人民的赤诚和对生命的敬佑，争分夺秒、舍生忘死、连续作战，挽救了一个又一个垂危生命，用血肉之躯构筑起阻击病毒的钢铁长城，为病毒肆虐的漫漫黑夜带来了光明，守护了国家和民族生生不息的希望。他们与病毒直面战斗，承受难以想象的身体和心理压力，付出巨大牺牲，2000多人确诊感染，几十人以身殉职。没有人生而英勇，只是选择了无畏。中国医生的医者仁心和大爱无疆，永远铭刻在中华民族历史上，永远铭刻在中国人民心中。

　　武汉人民和湖北人民顾全大局、顽强不屈，为阻击病毒作出巨大牺牲。武汉人民、湖北人民面对离汉离鄂通道关闭后与外隔绝、交通停滞、城市“停摆”，克服了近距离接触病毒、医疗资源和生活物资紧张以及长时间隔离带来的困难，忍住失去至爱亲朋的痛苦，服从大局，咬紧牙关，团结坚守。在伟大的抗疫战争中，英雄的武汉人民、湖北人民将载入史册为人们所铭记。

　　社区工作者、公安民警、海关关员、基层干部、下沉干部不辞辛苦、日夜值守，为保护人民生命安全牺牲奉献。400万名社区工作者奋战在全国65万个城乡社区中，监测疫情、测量体温、排查人员、站岗值守、宣传政策、防疫消杀，认真细致，尽职尽责，守好疫情防控“第一关口”。公安民警及辅警驻守医院、转运病人、街道巡逻、维护秩序，面对急难险重任务勇挑重担，130多人牺牲在工作岗位。海关关员依法履行卫生检疫职责，筑牢口岸检疫防线。社区防控一线广大党员、干部及时将党和政府的声音传导到基层，组织动员群众做好防控，积极为群众排忧解难，抓实抓细网格服务管理。

　　快递小哥、环卫工人、道路运输从业人员、新闻工作者、志愿者等各行各业工作者不惧风雨、敬业坚守。疫情期间，千家万户关门闭户，数百万快递员顶风冒雪、冒疫前行，在城市乡村奔波，给人们送来温暖。全国180万环卫工人起早贪黑、不辞辛劳，高标准做好卫生清扫、消毒杀菌、医疗废物集中处理、垃圾清理清运。数千万道路运输从业人员坚守岗位，许多城市出租车司机没有停工，有力保障疫情防控、生产生活物资运输和复工复产。新闻工作者不惧风险、深入一线，记录中国抗疫的点点滴滴，传递中国人民抗击疫情的温情和力量。许多普通人投入一线志愿服务，社区值守、排查患者、清洁消杀、买药送菜，缓解居民燃眉之急。据不完全统计，截至5月31日，全国参与疫情防控的注册志愿者达到881万人，志愿服务项目超过46万个，记录志愿服务时间超过2.9亿小时。

　　广大民众扛起责任、众志成城，自觉参与抗击疫情。危难面前，中国人民对中国共产党和中国政府高度信任，勇敢承担起社会责任，为取得抗疫胜利约束自我乃至牺牲自我。疫情暴发正值春节假期，国家一声令下，全民响应，一致行动，整个社会紧急停下脚步。人们取消了春节期间的走亲访友和各种聚会，克服困难就地隔离，外出自觉佩戴口罩、测量体温、保持社交距离。保护自己就是保护别人、就是为国家作贡献成为社会共识和每个人的自觉行动。人们长时间在家隔离，上网课、学美食、陪家人，用各种方式缓解压力，以积极乐观的态度抗击疫情。“所有好的做法如果想要奏效，必须要有公众的集体意愿。正因如此，中国有能力通过传统公共卫生干预方法应对一种新型的未知病毒”（注5）。

　　重大危机是考验执政党执政理念、执政效能的试金石。中国在较短时间内遏制疫情蔓延，根本在于中国共产党的坚强领导。中国共产党有坚强有力的领导核心，有以人民为中心的执政理念，面对疫情危机，迅速科学作出决策，实行高效有力的危机应对。中国共产党严密的组织体系和高效的运行机制，在短时间内建立横向到边、纵向到底的危机应对机制，有效调动各方积极性，全国上下令行禁止、统一行动。中国共产党460多万个基层组织，广泛动员群众、组织群众、凝聚群众、服务群众，筑起一座座抗击疫情的坚强堡垒。在疫情危及人民生命安全的危难关头，共产党员冲在最前面，全国3900多万名党员、干部战斗在抗疫一线，1300多万名党员参加志愿服务，近400名党员、干部为保卫人民生命安全献出了宝贵生命。广大党员自觉捐款，为疫情防控斗争真情奉献。注重在疫情考验中锤炼党员干部，检验为民初心和责任担当，对湖北省委和武汉市委领导班子作出调整补充，对不担当、不作为、失职渎职的党员干部严肃问责，对敢于担当、认真负责的党员干部大力褒奖、大胆使用，立起了鲜明导向。历经疫情磨砺，中国人民更加深切地认识到，风雨来袭，中国共产党的领导是最重要的保障、最可靠的依托，对中国共产党更加拥护和信赖，对中国制度更加充满信心。

　　四、共同构建人类卫生健康共同体

　　当前，新冠肺炎疫情仍在全球肆虐，每天都有许多生命逝去。面对严重危机，人类又一次站在了何去何从的十字路口。坚持科学理性还是制造政治分歧？加强团结合作还是寻求脱钩孤立？推进多边协调还是奉行单边主义？迫切需要各个国家作出回答。中国主张，各国应为全人类前途命运和子孙后代福祉作出正确选择，秉持人类命运共同体理念，齐心协力、守望相助、携手应对，坚决遏制疫情蔓延势头，打赢疫情防控全球阻击战，护佑世界和人民康宁。

　　（一）中国感谢和铭记国际社会宝贵支持和帮助

　　在中国疫情防控形势最艰难的时候，国际社会给予了中国和中国人民宝贵的支持和帮助。全球170多个国家领导人、50个国际和地区组织负责人以及300多个外国政党和政治组织向中国领导人来函致电、发表声明表示慰问支持。77个国家和12个国际组织为中国人民抗疫斗争提供捐赠，包括医用口罩、防护服、护目镜、呼吸机等急用医疗物资和设备。84个国家的地方政府、企业、民间机构、人士向中国提供了物资捐赠。金砖国家新开发银行、亚洲基础设施投资银行分别向中国提供70亿、24.85亿元人民币的紧急贷款，世界银行、亚洲开发银行向中国提供国家公共卫生应急管理体系建设等贷款支持。中国感谢国际社会给予的宝贵理解和支持，中国人民永远铭记在心。中华民族是懂得感恩、投桃报李的民族，中国始终在力所能及的范围内为国际社会抗击疫情提供支持。

　　（二）中国积极开展国际交流合作

　　疫情发生以来，中国始终同国际社会开展交流合作，加强高层沟通，分享疫情信息，开展科研合作，力所能及为国际组织和其他国家提供援助，为全球抗疫贡献中国智慧、中国力量。中国共产党同110多个国家的240个政党发出共同呼吁，呼吁各方以人类安全健康为重，秉持人类命运共同体理念，携手加强国际抗疫合作。

　　习近平主席亲自推动开展国际合作。疫情发生以来，习近平主席同近50位外国领导人和国际组织负责人通话或见面，介绍中国抗疫努力和成效，阐明中国始终本着公开、透明、负责任的态度，及时发布疫情信息，分享防控和救治经验，阐明中国对其他国家遭受的疫情和困难感同身受，积极提供力所能及的帮助，呼吁各方树立人类命运共同体意识，加强双多边合作，支持国际组织发挥作用，携手应对疫情挑战。习近平主席出席二十国集团领导人特别峰会并发表讲话，介绍中国抗疫经验，提出坚决打好新冠肺炎疫情防控全球阻击战、有效开展国际联防联控、积极支持国际组织发挥作用、加强国际宏观经济政策协调等4点主张和系列合作倡议，呼吁国际社会直面挑战、迅速行动。5月18日，习近平主席在第73届世界卫生大会视频会议开幕式上发表致辞，呼吁各国团结合作战胜疫情，共同构建人类卫生健康共同体，提出全力搞好疫情防控、发挥世界卫生组织作用、加大对非洲国家支持、加强全球公共卫生治理、恢复经济社会发展、加强国际合作等6点建议，并宣布两年内提供20亿美元国际援助、与联合国合作在华设立全球人道主义应急仓库和枢纽、建立30个中非对口医院合作机制、中国新冠疫苗研发完成并投入使用后将作为全球公共产品、同二十国集团成员一道落实“暂缓最贫困国家债务偿付倡议”等中国支持全球抗疫的一系列重大举措。

　　同国际社会分享疫情信息和抗疫经验。中国及时向国际社会通报疫情信息，交流防控经验，为全球防疫提供了基础性支持。疫情发生后，中国第一时间向世界卫生组织、有关国家和地区组织主动通报疫情信息，分享新冠病毒全基因组序列信息和新冠病毒核酸检测引物探针序列信息，定期向世界卫生组织和有关国家通报疫情信息。中国与东盟、欧盟、非盟、亚太经合组织、加共体、上海合作组织等国际和地区组织，以及韩国、日本、俄罗斯、美国、德国等国家，开展70多次疫情防控交流活动。国家卫生健康委汇编诊疗和防控方案并翻译成3个语种，分享给全球180多个国家、10多个国际和地区组织参照使用，并与世界卫生组织联合举办“新冠肺炎防治中国经验国际通报会”。国务院新闻办公室在武汉举行两场英文专题发布会，邀请相关专家和一线医护人员介绍中国抗疫经验和做法。中国媒体开设“全球疫情会诊室”“全球抗疫中国方案”等栏目，为各国开展交流搭建平台。中国智库和专家通过多种方式开展对外交流。中国－世界卫生组织联合专家考察组实地考察调研北京、成都、广州、深圳和武汉等地一线疫情防控工作，高度评价中国抗疫的努力和成效。

　　向国际社会提供人道主义援助。在自身疫情防控仍然面临巨大压力的情况下，中国迅速展开行动，力所能及地为国际社会提供援助。向世界卫生组织提供两批共5000万美元现汇援助，积极协助世界卫生组织在华采购个人防护用品和建立物资储备库，积极协助世界卫生组织“团结应对基金”在中国筹资，参与世界卫生组织发起的“全球合作加速开发、生产、公平获取新冠肺炎防控新工具”倡议。积极开展对外医疗援助，截至5月31日，中国共向27个国家派出29支医疗专家组，已经或正在向150个国家和4个国际组织提供抗疫援助；指导长期派驻在56个国家的援外医疗队协助驻在国开展疫情防控工作，向驻在国民众和华侨华人提供技术咨询和健康教育，举办线上线下培训400余场；地方政府、企业和民间机构、个人通过各种渠道，向150多个国家、地区和国际组织捐赠抗疫物资。中国政府始终关心在华外国人士的生命安全和身体健康，对于感染新冠肺炎的外国人士一视同仁及时进行救治。

　　有序开展防疫物资出口。中国在满足国内疫情防控需要的基础上，想方设法为各国采购防疫物资提供力所能及的支持和便利，打通需求对接、货源组织、物流运输、出口通关等方面堵点，畅通出口环节，有序开展防疫物资出口。采取有力措施严控质量、规范秩序，发布防疫用品国外市场准入信息指南，加强防疫物资市场和出口质量监管，保质保量向国际社会提供抗击疫情急需的防疫物资。3月1日至5月31日，中国向200个国家和地区出口防疫物资，其中，口罩706亿只，防护服3.4亿套，护目镜1.15亿个，呼吸机9.67万台，检测试剂盒2.25亿人份，红外线测温仪4029万台，出口规模呈明显增长态势，有力支持了相关国家疫情防控。1月至4月，中欧班列开行数量和发送货物量同比分别增长24%和27%，累计运送抗疫物资66万件，为维持国际产业链和供应链畅通、保障抗疫物资运输发挥了重要作用。

　　开展国际科研交流合作。加强同世界卫生组织沟通交流，同有关国家在溯源、药物、疫苗、检测等方面开展科研交流与合作，共享科研数据信息，共同研究防控和救治策略。科技部、国家卫生健康委、中国科协、中华医学会联合搭建“新型冠状病毒肺炎科研成果学术交流平台”，供全球科研人员发布成果、参与研讨，截至5月31日，共上线104种期刊、970篇论文和报告。国家中医药管理局联合上合组织睦邻友好合作委员会召开“中国中西医结合专家组同上海合作组织国家医院新冠肺炎视频诊断会议”，指导世界中医药学会联合会和世界针灸学会联合会开展“中医药抗疫全球直播”“国际抗疫专家大讲堂”等活动。中国科学院发布“2019新型冠状病毒资源库”，建成“新型冠状病毒国家科技资源服务系统”“新型冠状病毒肺炎科研文献共享平台”，截至5月31日，3个平台为全球超过37万用户提供近4800万次下载、浏览和检索服务。建立国际合作专家库，同有关国家开展疫苗研发、药品研发等合作。充分发挥“一带一路”国际科学组织联盟作用，推动成员之间就新冠病毒研究和新冠肺炎治疗开展科技合作。中国医疗机构、疾控机构和科学家在《柳叶刀》《科学》《自然》《新英格兰医学杂志》等国际知名学术期刊上发表数十篇高水平论文，及时发布新冠肺炎首批患者临床特征描述、人际传播风险、方舱医院经验、药物研发进展、疫苗动物实验结果等研究成果。同有关国家、世界卫生组织以及流行病防范创新联盟（CEPI）、全球疫苗免疫联盟（GAVI）等开展科研合作，加快推进疫苗研发和药物临床试验。

　　（三）国际社会团结合作共同抗疫

　　疫情在全球传播蔓延的形势令人担忧。无论是阻击病毒的传播蔓延，还是抵御不断恶化的全球经济衰退，都需要国际社会团结合作，都需要坚持多边主义、推动构建人类命运共同体。团结合作是国际社会战胜疫情最有力武器。未来的成败取决于今天的作为。中国呼吁各国紧急行动起来，更好团结起来，全面加强合作，联合抗疫，共克时艰。

　　有效开展联防联控国际合作。应对疫情必须各国协同作战，建立起严密的联防联控网络。疫情发生以来，世界卫生组织秉持客观公正立场，积极履行职责，采取一系列专业、科学、有效措施，为领导和推进国际抗疫合作作出了重大贡献。中国坚定支持世界卫生组织发挥全球抗疫领导作用，呼吁国际社会加大对世界卫生组织政治支持和资金投入，调动全球资源打赢疫情阻击战。中国主张，各国在世界卫生组织的指导和协调下，采取科学合理、协同联动的防控措施，科学调配医疗力量和重要物资，在防护、隔离、检测、救治、追踪等重要领域采取有力举措，同时，加强信息共享和经验交流，开展检测方法、临床救治、疫苗药物研发国际合作，继续支持各国科学家开展病毒源头和传播途径的全球科学研究。中国呼吁，二十国集团、亚太经合组织、金砖国家、上海合作组织等多边机制加大机制内对话交流与政策协调力度，二十国集团成员切实落实二十国集团领导人特别峰会达成的共识。开展联防联控国际合作，大国的负责任、担当和主动作为至关重要。中国愿同各国包括美国加强交流合作，共同应对疫情挑战，特别是在疫苗和特效药的研发、生产和分发上开展合作，为阻断病毒传播作出应有贡献。

　　合作应对疫情给世界经济带来的影响。疫情在全球传播蔓延，人员流动、跨境商贸活动受阻，金融市场剧烈震荡，全球产业链供应链受到双重打击，世界经济深度衰退不可避免，国际社会联手稳定和恢复世界经济势在必行。中国愿同各国一道，在加强疫情防控的同时，一齐应对日益上升的全球经济衰退，加强国际宏观经济政策协调，共同维护全球产业链供应链的稳定、安全与畅通。新冠肺炎疫情改变了经济全球化形态，但全球化发展大势没有改变，搞“脱钩”“筑墙”“去全球化”，既割裂全球也难以自保。中国主张，各国继续推进全球化，维护以世界贸易组织为基石的多边贸易体制，减免关税、取消壁垒、畅通贸易，使全球产业链供应链安全顺畅运行，同时，实施有力有效的财政和货币政策，加强金融监管协调，维护金融市场稳定，防止引发全球性金融危机导致世界经济陷入大规模、长周期衰退。中国将继续向国际市场供应防疫物资、原料药、生活必需品等产品，坚定不移扩大改革开放，积极扩大进口，扩大对外投资，为各国抗击疫情、稳定世界经济作出更大贡献。

　　向应对疫情能力薄弱的国家和地区提供帮助。亚洲、非洲和拉美地区发展中国家特别是非洲国家，公共卫生体系薄弱，难以独立应对疫情带来的严峻挑战，帮助他们提升疫情防控能力和水平是全球抗疫的重中之重。中国呼吁，联合国、世界卫生组织、国际货币基金组织、世界银行等多边机构向非洲国家提供必要的紧急援助；发达国家向发展中国家特别是非洲国家提供更多物资、技术、人力支持，在全球抗疫中担负更多责任、发挥更大作用。中国积极参与并落实二十国集团缓债倡议，已宣布77个有关发展中国家暂停债务偿还。在向50多个非洲国家和非盟交付医疗援助物资、派出7个医疗专家组的基础上，中国将进一步加大援非抗疫力度，继续向非洲国家提供力所能及的支持，援助急需医疗物资，开展医疗技术合作，派遣更多医疗专家组和工作组，帮助非洲国家提升疫情防控能力和水平。中国将向联合国人道应对计划提供支持。

　　坚决反对污名化和疫情政治化。面对新冠病毒对人类生命安全和健康的严重威胁，当务之急是团结合作、战胜疫情。人类的共同敌人是病毒，而不是某个国家、某个种族。中国呼吁国际社会更加团结起来，摒弃偏见和傲慢，抵制自私自利、“甩锅”推责，反对污名化和疫情政治化，让团结、合作、担当、作为的精神引领全世界人民取得全球抗疫胜利。中国是病毒受害国，也是全球抗疫贡献国，应该得到公正对待而不是责难。中国在疫情初期就向国际社会发出清晰而明确的信息，个别国家无视这些信息耽误疫情应对和拯救生命，却反称被中国“延误”，真是“欲加之罪，何患无辞”。中国始终坚持公开、透明、负责任原则及时向国际社会公布疫情信息，无端指责中国隐瞒疫情信息和死亡病例数据，是对14亿中国人民、对被病毒夺去生命的逝者、对数百万中国医护人员的极不尊重，中国对此坚决反对。新冠病毒是人类未知的新病毒，病毒溯源是科学问题，需要科学家和医学专家进行研究，基于事实和证据得出科学结论。通过转嫁责任掩盖自身问题，既不负责任也不道德，中国绝不接受任何滥诉和索赔要求。面对疫情在全球传播蔓延，中国向国际社会提供力所能及的援助，源于中国人民的古道热肠，源于对其他国家人民遭受疫情苦难的感同身受，源于面对灾难同舟共济的人道主义精神，源于大国的责任和担当，绝非输出中国模式，更不是为谋求所谓地缘政治利益。

　　健全完善惠及全人类、高效可持续的全球公共卫生体系。人类发展史也是同病毒的斗争史。当前，全球公共卫生治理存在诸多短板，全球传染病联防联控机制远未形成，国际公共卫生资源十分匮乏，逆全球化兴起使得全球公共卫生体系更加脆弱。人类终将战胜疫情，但重大公共卫生突发事件对人类来说不会是最后一次。中国呼吁，各国以此次疫情为鉴，反思教训，化危为机，以卓越的政治远见和高度负责的精神，坚持生命至上、全球一体、平等尊重、合作互助，建立健全全球公共卫生安全长效融资机制、威胁监测预警与联合响应机制、资源储备和资源配置体系等合作机制，建设惠及全人类、高效可持续的全球公共卫生体系，筑牢保障全人类生命安全和健康的坚固防线，构建人类卫生健康共同体。中国支持在全球疫情得到控制之后，坚持客观公正原则和科学专业态度，全面评估全球应对疫情工作，总结经验，弥补不足。中国主张，为人类发展计、为子孙后代谋，各国应立即行动起来，采取断然措施，最大限度消除病毒对人类的现实和潜在威胁。中国作为负责任大国，始终秉持人类命运共同体理念，积极推进和参与卫生健康领域国际合作，认真落实习近平主席在第73届世界卫生大会视频会议开幕式上提出的6点建议和5项举措，为维护地区和世界公共卫生安全，推动构建人类卫生健康共同体作出更大贡献。

　　结束语

　　中华民族历经磨难，但从未被压垮过，而是愈挫愈勇，不断在磨难中成长、从磨难中奋起。面对疫情，中国人民万众一心、众志成城，取得了抗击疫情重大战略成果。中国始终同各国紧紧站在一起，休戚与共，并肩战斗。

　　当前，新冠病毒仍在全球传播蔓延，国际社会将会面对更加严峻的困难和挑战。全球疫情防控战，已经成为维护全球公共卫生安全之战、维护人类健康福祉之战、维护世界繁荣发展之战、维护国际道义良知之战，事关人类前途命运。人类唯有战而胜之，别无他路。国际社会要坚定信心，团结合作。团结就是力量，胜利一定属于全人类！

新冠肺炎疫情深刻影响人类发展进程，但人们对美好生活的向往和追求没有改变，和平发展、合作共赢的历史车轮依然滚滚向前。阳光总在风雨后。全世界人民心怀希望和梦想，秉持人类命运共同体理念，目标一致、团结前行，就一定能够战胜各种困难和挑战，建设更加繁荣美好的世界。

2020年6月7日

1. 国务院联防联控机制：做好疫情常态化防控下新冠病毒核酸检测质量控制工作 联防联控机制医疗发〔2020〕242号

各省、自治区、直辖市及新疆生产建设兵团卫生健康委：

为落实《国务院应对新型冠状病毒感染肺炎疫情联防联控机制关于做好新冠肺炎疫情常态化防控工作的指导意见》（国发明电〔2020〕14号）要求，扩大新冠病毒核酸检测（以下简称核酸检测）范围，保证核酸检测质量，现提出以下工作要求：

一、高度重视核酸检测质量控制工作

当前，全国疫情防控进入常态化阶段，对核酸检测能力提出了更高要求。按照扩大核酸检测范围，对重点人群实行应检尽检，对其他人群实行愿检尽检的要求，各地着力加强核酸检测能力建设，核酸检测量大幅度增长，效率明显提升。在保障核酸检测量增加的同时，必须加强质量控制，保证检测结果准确可靠。各地要高度重视核酸检测质量控制工作，使核酸检测既保“量”又保“质”，为做好常态化疫情防控工作提供有力技术支撑。

二、加强实验室备案或准入管理

开展核酸检测的实验室，应当符合《病原微生物实验室生物安全管理条例》（国务院令第424号）和《医疗机构临床基因扩增检验实验室管理办法》（卫办医政发〔2010〕194号）有关规定，并在相应的卫生健康行政部门进行登记备案，具备生物安全二级及以上实验室条件以及PCR实验室条件。医学检验实验室（常称为第三方实验室）还应当符合《医学检验实验室基本标准（试行）》《医学检验实验室管理规范（试行）》等要求。

三、加强实验室检测质量控制

各地应当加强核酸检测质量控制，将开展核酸检测的医疗机构（含医学检验实验室，下同）和疾控机构实验室统一纳入质量控制体系。实验室要做好日常室内质控，并常态化接受国家级或省级检验质量控制。各省级卫生健康行政部门要加强对核酸检测实验室的日常质量控制工作，并组织实验室分批参加室间质评，保证短期内每个实验室至少参加1次室间质评并合格。检测结果质量问题突出的，或室间质量评价不合格的，不得开展核酸检测。

四、加强核酸检测人员培训

各地要按照《新型冠状病毒肺炎实验室检测技术指南》要求，加强医疗机构、疾控机构核酸检测相关人员的技术培训和指导，覆盖标本的采集、保存、运输、处理和检测等各个环节，最大限度减少产生假阴性的因素。对标本采集和处理等重点环节，加大人员培训力度，确保采样人员规范采集各类标本，实验室检测人员要熟练掌握标本处理和检测方法，开展检测时做好生物安全防护。

国务院应对新型冠状病毒肺炎疫情联防联控机制医疗救治组

2020年6月1日 卫健委

1. 国务院：做好新冠肺炎疫情 常态化防控工作 国发明电〔2020〕14号

各省、自治区、直辖市人民政府，国务院各部委、各直属机构：

在以习近平同志为核心的党中央坚强领导下，经过全国上下艰苦努力，我国新冠肺炎疫情防控向好态势进一步巩固，防控工作已从应急状态转为常态化。按照党中央关于抓紧抓实抓细常态化疫情防控工作的决策部署，为全面落实“外防输入、内防反弹”的总体防控策略，坚持及时发现、快速处置、精准管控、有效救治，有力保障人民群众生命安全和身体健康，有力保障经济社会秩序全面恢复，经中央应对新型冠状病毒感染肺炎疫情工作领导小组同意，现提出以下意见。

一、坚持预防为主

1.科学佩戴口罩。在人员密集的封闭场所、与他人小于1米距离接触时佩戴口罩。医疗机构工作人员，在密闭公共场所工作的营业员、保安员、保洁员、司乘人员、客运场站服务人员、警察等人员以及就医人员等要佩戴口罩。

2.减少人员聚集。注意保持1米以上的社交距离。减少非必要的聚集性活动，减少参加聚集性活动的人员。尽量不前往人员聚集场所尤其是密闭式场所。

3.加强通风消毒。室内经常开窗通风，保持空气流通。公共场所、场站码头、公共交通工具要落实日常清洁、消毒等卫生措施。

4.提高健康素养。养成“一米线”、勤洗手、戴口罩、公筷制等卫生习惯和生活方式。咳嗽、打喷嚏时注意遮挡。

二、落实“四早”措施

5.及时发现。落实公共场所体温检测措施，加强预检分诊和发热门诊排查，做到对确诊病例、疑似病例、无症状感染者的“早发现”，并按要求“早报告”，不得瞒报、漏报、迟报。

6.快速处置。24小时内完成流行病学调查，充分发挥大数据等优势，尽快彻底查明可能的感染源，做好对密切接触者的判定和追踪管理。落实“早隔离”措施，及时对确诊病例、疑似病例进行隔离治疗，对无症状感染者、密切接触者实行14天集中隔离医学观察。对可能的污染场所全面终末消毒。

7.精准管控。依法依规、科学划定防控区域范围至最小单元（如楼栋、病区、居民小区、自然村组等），果断采取限制人员聚集性活动、封锁等措施，切断传播途径，尽最大可能降低感染风险。及时公布防控区域相关信息。

8.有效救治。指定定点收治医院，落实“早治疗”措施，加强中西医结合治疗。及时有效全面收治轻症患者，减少向重症转化。坚持“四集中”，对重症患者实施多学科救治，最大限度提高治愈率、降低病亡率。患者治愈出院后，继续集中或居家隔离医学观察14天。

三、突出重点环节

9.重点场所防控。按照相关技术指南，在落实防控措施前提下，全面开放商场、超市、宾馆、餐馆等生活场所；采取预约、限流等方式，开放公园、旅游景点、运动场所，图书馆、博物馆、美术馆等室内场馆，以及影剧院、游艺厅等密闭式娱乐休闲场所，可举办各类必要的会议、会展活动等。

10.重点机构防控。做好养老机构、福利院、监所、精神卫生医疗机构等风险防范，落实人员进出管理、人员防护、健康监测、消毒等防控措施。养老机构内设医务室、护理站等医疗服务机构的，不得超出医疗许可服务范围对外服务。医疗机构举办养老机构或与养老机构毗邻的，应按照医疗机构分区管理要求开展交叉感染评估，评估有风险的应采取必要的控制措施。

11.重点人群防控。指导老年人、儿童、孕产妇、残疾人、严重慢性病患者等重点人群做好个人防护，并开展心理疏导和关爱帮扶等工作。

12.医疗机构防控。加强院内感染防控，推广分时段预约诊疗，严格落实医疗机构分区管理要求，及时排查风险并采取处置措施，严格探视和陪护管理，避免交叉感染。严格预检分诊和发热门诊工作流程，强化防控措施。落实医务人员防护措施，加强对医务人员的健康管理和监测。

13.校园防控。实行教职员工和学生健康情况“日报告”、“零报告”制度。做好健康提示、健康管理和教室通风、消毒等工作，落实入学入托晨（午）检、因病缺课（勤）病因追查和登记等防控措施。

14.社区防控。加强基层社区网格化管理，发挥社区志愿者作用。做好健康教育、环境卫生治理、出租房屋和集体宿舍管理、外来人员管理等工作。出现疫情的社区要加强密切接触者排查和隔离管理、终末消毒等工作，必要时采取限制人员聚集性活动、封闭式管理等措施。

四、强化支撑保障

15.扩大检测范围。各地可根据疫情防控工作需要和检测能力，进行科学评估，对密切接触者、境外入境人员、发热门诊患者、新住院患者及陪护人员、医疗机构工作人员、口岸检疫和边防检查人员、监所工作人员、社会福利养老机构工作人员等重点人群“应检尽检”。对其他人群实施“愿检尽检”。人群相对密集、流动性较大地区和边境口岸等重点地区县区级及以上疾控机构、二级及以上医院要着力加强核酸检测能力建设；鼓励有资质的社会检测机构提供检测服务，扩大商业化应用。“应检尽检”所需费用由各地政府承担，“愿检尽检”所需费用由企事业单位或个人承担；检测收费标准由各地物价部门确定并公示。各地要及时公布检测机构名单。

16.发挥大数据作用。依托全国一体化政务服务平台，全面推动各地落实“健康码”互通互认“一码通行”，及时将核酸和血清抗体检测结果、重点人员等信息共享到“健康码”数据库，推进人员安全有序流动。做好全国一体化政务服务平台“防疫健康信息码”入境人员版的推广应用，加强入境人员闭环管理。

17.强化科研与国际合作。推进疫苗、药物科技攻关和病毒变异、免疫策略等研究。加快检测试剂和设备研发，提高灵敏度、特异性、简便性，进一步提升检测能力、缩短检测时间。加强与世界卫生组织等国际组织、有关国家的信息共享、技术交流和防控合作。

五、加强组织领导

18.落实党委和政府责任。各地党委和政府要落实属地责任，加强组织领导，坚持依法防控、科学防控、联防联控，加大经费投入，加强医疗物资动态储备，提升防控和应急处置能力，严格落实常态化防控各项措施要求。国务院各有关部门要落实主管责任，继续加强联防联控、统筹调度，强化对各地常态化防控工作的指导和支持。

19.落实企事业单位责任。各企事业单位要落实主体责任，严格执行疫情防控规定，健全防控工作责任制和管理制度，制定完善应急预案。

20.动态调整风险等级和应急响应级别。各地要按照分区分级标准，依据本地疫情形势，动态调整风险等级和应急响应级别。要因地制宜、因时制宜，不断完善疫情防控应急预案和各项配套工作方案，一旦发生疫情，及时采取应急处置措施，实施精准防控。

境外疫情输入防控在落实常态化防控工作的同时，按照中央关于做好防控境外疫情输入工作的指导意见实施。

国务院应对新型冠状病毒感染肺炎

疫情联防联控机制

2020年5月7日

1. 国务院联防联控机制：开展企事业单位复工复产疫情防控措施落实情况专项检查工作 联防联控机制综发〔2020〕159号

各省、自治区、直辖市及新疆生产建设兵团应对新型冠状病毒肺炎疫情联防联控机制（领导小组、指挥部）：

为进一步推动企事业单位贯彻落实复工复产疫情防控各项措施要求，按照科学防控、精准施策原则，在积极有序推进复工复产的同时防止疫情反弹，经研究，决定对企事业单位复工复产疫情防控措施落实情况开展专项检查工作。现就有关事项通知如下：

一、组织实施

省级应对新型冠状病毒肺炎疫情联防联控机制（领导小组、指挥部）负责本地区具体检查方案的制定和组织实施。

地市级应对新型冠状病毒肺炎疫情联防联控机制（领导小组、指挥部）负责组织协调和指导各县（区）开展检查工作，并对工矿企业、建筑施工及其他存在人员密集作业场所的重点单位开展抽查。

县（区）级应对新型冠状病毒肺炎疫情联防联控机制（领导小组、指挥部）负责组织开展现场检查工作（附件1、附件2），对20人以上复工复产的企事业单位要进行全覆盖。

二、检查重点

（一）高风险和中风险地区企事业单位。将高风险和中风险地区的企事业单位作为重点检查对象，对其员工健康监测、工作场所以及个人防护等各项防控措施落实情况进行检查。

（二）外来人员较多的企事业单位。对建筑施工等外来人员较多的单位，重点检查人员健康管理、宣传教育以及员工就餐和集体宿舍等防控措施落实情况。

（三）存在人员密集作业场所的企事业单位。对存在流水线作业等人员密集作业场所的企事业单位，重点检查作业场所人员登记、工作场所通风以及场所清洁消毒和个人防护等防控措施落实情况。

三、工作要求

（一）加强组织领导。各级应对新型冠状病毒肺炎疫情联防联控机制（领导小组、指挥部）要加强对专项检查工作的组织领导，明确工作职责，对检查中发现的问题要及时指导用人单位予以改正。以专项检查推动疫情防控措施落地见效，进一步督促用人单位在做好复工复产的同时切实落实疫情防控的各项措施。

（二）加强舆论宣传。各级应对新型冠状病毒肺炎疫情联防联控机制（领导小组、指挥部）要充分利用各类媒体，加强对企事业单位复工复产疫情防控措施的宣传，充分发挥社会监督作用，营造群防群控的社会氛围，确保取得防控效果。

（三）及时总结报告。各省级应对新型冠状病毒肺炎疫情联防联控机制（领导小组、指挥部）请于2020年5月5日前将检查工作情况报送至国家卫生健康委，报告内容包括专项检查工作情况、发现的典型问题（案例）。

国务院应对新型冠状病毒感染

肺炎疫情联防联控机制综合组

2020年4月26日

1. 人社部等15部门：做好当前农民工就业创业工作 人社部发〔2020〕61号

各省、自治区、直辖市人民政府，国务院有关部委、有关直属机构：

促进农民工就业创业，事关农民增收致富，事关就业大局稳定，事关打赢脱贫攻坚战。今年以来，受新冠肺炎疫情、经济下行压力等多重因素叠加影响，部分农民工就业创业面临一些困难。为进一步做好当前农民工就业创业工作，经国务院同意，现提出以下意见：

一、拓宽外出就业渠道

（一）稳定现有就业岗位。全面落实减税降费、失业保险稳岗返还、以工代训等援企稳岗政策，引导企业特别是中小微企业不裁员或少裁员，督促企业将补贴资金用于职工生活补助、缴纳社会保险费、开展在岗转岗培训等。帮助外贸企业纾困解难，支持出口产品转内销，加大对住宿餐饮、批发零售、文化旅游、家政服务等行业的针对性政策扶持，最大限度稳定农民工就业岗位。（国家发展改革委、工业和信息化部、财政部、人力资源社会保障部、商务部、文化和旅游部、国家卫生健康委、税务总局等按职责分工负责）

（二）创造更多就业机会。推动重大投资项目加速落地，强化促消费、扩内需政策扶持，释放经济发展潜力，提升吸纳就业能力。各类基础设施建设要优先考虑带动就业能力强的项目。大力发展生活服务业、劳动密集型产业，对吸纳农民工就业多的给予更大政策激励。培育经济发展新动能，加快信息网络等新型基础设施建设，促进共享出行、社区团购等新业态发展，支持农业、林业生产端电子商务发展，促进产销对接，拓展农民工就业新领域。（国家发展改革委、科技部、工业和信息化部、民政部、财政部、交通运输部、农业农村部、商务部、国家林草局等按职责分工负责）

（三）支持多渠道灵活就业。支持农民工通过临时性、非全日制、季节性、弹性工作等多种形式实现灵活就业，灵活就业支持政策对城镇户籍居民和农民工一视同仁。因地制宜发展零工市场或劳务市场，搭建企业用工余缺调剂平台。鼓励农民工从事个体经营，开办特色小店，符合条件的按规定给予税收优惠、场地支持等政策。鼓励互联网平台企业降低平台服务费、信息中介费、加盟管理费等费用标准，支持农民工从事直播销售、网约配送等新就业形态增加收入。（财政部、人力资源社会保障部、商务部、税务总局、市场监管总局等按职责分工负责）

二、促进就地就近就业

（四）发展乡村产业吸纳就业。结合农业生产特点创新开发“惠农”产品包等金融产品，支持发展特色种植业、林草特色产业、规模养殖业和种养结合循环农林业。大力发展农林产品加工业、农林产品物流冷链和产销对接等相关产业，推动休闲观光、健康养生、农事体验等乡村休闲旅游业健康发展。将带动就业情况作为创建现代农林业产业园的重要考量。支持返乡留乡农民工成立农民合作社、发展现代种养业和农产品初加工，鼓励发展新产业新业态，增加就业岗位。（农业农村部、国家林草局牵头，财政部、人民银行等按职责分工负责）

（五）推动项目建设促进就业。大力发展县域经济，建设一批卫星城镇，发展一批当地优势特色产业项目，提高就业承载力。加强小型水利、高标准农田、林下经济、木本粮油等特色经济林基地、乡村绿化、人居环境整治等领域的农村中小型基础设施建设，加快灾后恢复重建，积极采取以工代赈方式实施项目。加大以工代赈投入力度，加快推进项目开工建设，将发放劳务报酬的资金占比由10%提高至15%以上，吸纳更多返乡留乡农民工就业。（国家发展改革委、住房城乡建设部、水利部、农业农村部、国家林草局等按职责分工负责）

（六）支持返乡入乡创业带动就业。加强创业服务能力建设，组织协调企业家、科技人员、创业成功人士等成立创业服务专家团队和农村创新创业导师队伍，为返乡入乡创业农民工提供政策咨询、开业指导等专业服务。对符合条件的返乡入乡创业农民工，按规定给予税费减免、创业补贴、创业担保贷款及贴息等创业扶持政策，对其中首次创业且正常经营1年以上的，按规定给予一次性创业补贴，正常经营6个月以上的可先行申领补贴资金的50%。加强创业载体建设，政府投资开发的孵化基地等创业载体可安排一定比例的场地，免费向返乡入乡创业农民工提供，支持高质量建设一批返乡入乡创业园（基地）、集聚区，吸引农民工等就地就近创业就业。（国家发展改革委、财政部、人力资源社会保障部、农业农村部、人民银行、税务总局等按职责分工负责）

三、强化平等就业服务和权益保障

（七）加强就业服务。提供便捷高效求职服务，广泛收集跨区域岗位信息，通过线上线下多渠道发布，举办农民工专场招聘会，送岗位下乡进村入户，对有集中外出需求的农民工开展有组织劳务输出。畅通就业求助渠道，建立健全动态更新的岗位储备机制和多方联动的快速响应机制，及时帮助农民工解决求职困难。全面放开失业登记，失业农民工可在户籍地、常住地、就业地、参保地进行登记，免费享受职业介绍、培训项目推介等基本公共就业服务，对其中大龄、身有残疾、长期失业等特殊困难的，按规定纳入就业援助范围，实施重点帮扶。（人力资源社会保障部、农业农村部等按职责分工负责）

（八）强化教育培训。用好职业技能提升行动专账资金，实施农民工稳就业职业技能培训计划。支持企业面向新吸纳失业农民工开展以工代训，实现以训稳岗。面向失业农民工开展定向定岗培训、急需紧缺职业专项培训，面向返乡农民工就近开展职业转换培训和创业培训。农民工可按规定在培训地申领职业培训补贴、培训期间生活费补贴和职业技能鉴定补贴等。落实高职扩招任务要求，针对农民工单列招生计划，做好考试测试、招生录取、分类教育管理等工作。（教育部、财政部、人力资源社会保障部、农业农村部等按职责分工负责）

（九）维护劳动权益。指导督促企业依法招工用工，加强农民工劳动保障权益维护，依法严厉打击恶意欠薪等违法行为。加大涉劳动报酬等劳动争议处理力度，依法为农民工提供法律援助服务，支持农民工与用人单位协商化解矛盾纠纷。加大日常监察执法力度，坚决纠正针对湖北等受疫情影响严重地区农民工的就业歧视。科学合理界定互联网平台企业责任，维护平台就业农民工劳动保障权益。（司法部、人力资源社会保障部等按职责分工负责）

（十）做好生活保障。扩大失业保险保障范围，畅通线上线下申领渠道，为符合条件的农民工及时发放失业保险金、失业补助金、一次性生活补助或临时生活补助。对受疫情影响无法返岗复工、连续3个月无收入来源，生活困难且失业保险政策无法覆盖的农民工等未参保失业人员，未纳入低保范围的，经本人申请，由务工地或经常居住地发放一次性临时救助金。（民政部、财政部、人力资源社会保障部等按职责分工负责）

四、优先保障贫困劳动力稳岗就业

（十一）稳定贫困劳动力外出务工规模。优先组织贫困劳动力有序外出务工，加大岗位归集发布和劳务对接力度，按规定落实各项扶持政策，力争有就业意愿和就业能力的贫困劳动力都能实现就业，确保今年贫困劳动力外出务工规模不低于去年。千方百计稳定已就业贫困劳动力就业岗位，对企业确需裁员的，提前介入指导，鼓励同等条件下优先留用贫困劳动力。加大对失业贫困劳动力就业帮扶力度，优先提供转岗就业机会，对通过市场渠道难以就业的，纳入当地就业困难人员范围，符合条件的提供公益性岗位等托底安置。对有培训意愿的贫困劳动力实行技能培训全覆盖。将贫困劳动力外出务工情况作为年度脱贫攻坚成效考核的重要内容。（人力资源社会保障部、农业农村部、国务院扶贫办等按职责分工负责）

（十二）拓宽贫困劳动力就地就近就业渠道。积极发展农业生产，加强农业农村、交通、水利、林草等领域工程项目建设，积极采取以工代赈方式实施项目，为返乡留乡贫困劳动力提供更多就业机会。促进扶贫龙头企业和扶贫车间健康发展，坚持扶贫性质，更多招收贫困劳动力就业。加强乡村公益性岗位开发和管理，充分考虑当地收入水平和岗位职责等情况，合理确定岗位待遇水平，统筹用好各类乡村公益性岗位托底安置就业困难贫困劳动力。（国家发展改革委、人力资源社会保障部、自然资源部、交通运输部、水利部、农业农村部、国家林草局、国务院扶贫办等按职责分工负责）

（十三）聚焦聚力重点地区攻坚。将52个未摘帽贫困县、“三区三州”等深度贫困地区、易地扶贫搬迁大型安置区以及湖北等受疫情影响严重地区作为重中之重，充分发挥对口支援、省际省内协作机制等作用，加大劳务协作、项目建设等各类资源倾斜支持力度，定向投放岗位，开展点对点劳务输出，及时解决贫困劳动力就业面临的突出困难和问题。（国家发展改革委、人力资源社会保障部、国务院扶贫办等按职责分工负责）

五、加强组织保障

（十四）加强组织领导。地方各级人民政府要高度重视农民工就业创业工作，将其作为稳就业和保居民就业重点，坚持市场就业和政府促进相结合，层层压实责任，健全机制，综合施策，稳定城镇常住农民工就业，确保农民工就业形势总体平稳，困难农民工及时得到救助。各有关部门要各司其职，协同配合，形成合力。人力资源社会保障部门要发挥统筹协调作用，做好农民工就业创业服务、职业技能培训和权益维护等工作；发展改革、住房城乡建设、交通运输、水利等部门要抓好项目投资带动就业、以工代赈项目实施；工业和信息化、商务、文化和旅游等部门要完善行业产业发展规划，助力稳企稳岗；民政、人力资源社会保障等部门要做好生活困难农民工的兜底保障工作；财政部门要做好农民工就业创业各项工作资金保障；农业农村、林草等部门要加大乡村地区一二三产业岗位开发力度，拓宽农民工就地就近就业渠道；统计部门要做好农民工就业情况调查监测；扶贫部门要配合做好贫困劳动力就业稳岗工作。（各有关部门、单位和地方各级人民政府按职责分工负责）

（十五）加强工作保障。健全公共就业服务体系，推进城镇公共就业服务向农村延伸，运用购买服务等多种方式，充分发挥各类人力资源服务机构、劳务经纪人作用，为农民工提供便捷高效的就业服务。加大农民工就业创业政策落实力度，优化申领流程，精简证明材料，确保政策便捷惠及享受对象。统筹用好各类资金，将吸纳农民工就业数量作为城镇建设用地增加规模的重要因素，保障农民工平等享受就业服务政策。（财政部、人力资源社会保障部、自然资源部、农业农村部和地方各级人民政府按职责分工负责）

（十六）加强宣传引导。大力宣传促进农民工就业创业的优惠政策和服务举措，充分利用各种受众面广、宣传效果好的新媒体，提高政策知晓度。广泛挖掘农民工就业创业典型案例，讲好就业故事，营造有利于农民工就业创业的良好氛围。（各有关部门、单位和地方各级人民政府按职责分工负责）

人力资源社会保障部 国家发展改革委 工业和信息化部

民政部 财政部 自然资源部 住房城乡建设部 交通运输部

水利部 农业农村部 商务部 文化和旅游部

国家统计局 国家林业和草原局 国务院扶贫办

2020年8月6日

1. 人社部等3部门：进一步加强贫困家庭高校毕业生就业帮扶工作

各省、自治区、直辖市及新疆生产建设兵团人力资源社会保障厅（局）、教育厅（教委、教育局）、扶贫办（局）：

　　今年是决战决胜脱贫攻坚和全面建成小康社会的收官之年，促进贫困家庭高校毕业生尽早就业，是稳就业保民生的重要内容。受新冠肺炎疫情影响，一些贫困家庭高校毕业生求职面临更多困难。为进一步加强对贫困家庭高校毕业生的就业帮扶工作，现就有关事项通知如下。

　　一、明确目标任务。各地要将贫困家庭高校毕业生及时纳入就业帮扶，坚持重点关注、重点推荐、重点服务，建立健全覆盖就业创业全过程的帮扶机制，统筹调动资源，突出精准施策，加强关爱指导，使建档立卡贫困家庭、零就业家庭毕业生全面就业到位，使有需求的其他贫困家庭毕业生全面帮扶到位，有就业意愿的都能实现就业或组织到就业准备活动中。

　　二、摸清就业需求。各地要不断完善求职创业补贴政策数据库，将受疫情影响而导致家庭经济困难的高校毕业生及时纳入政策范围。依托现有就业信息平台，建立贫困家庭毕业生就业帮扶机制，做实专门台账，实施动态管理，做到人员底数清、就业需求清、帮扶举措清、求职进展清。教育部门和高校要摸清每名贫困家庭毕业生服务需求，实施“一生一策”针对性帮扶。对离校未就业贫困家庭毕业生，教育部门与人力资源社会保障部门要在实名信息交接中，同步交接其帮扶台账，记录就业意向、求职区域、存在困难等情况，做到就业服务不断线。

　　三、加强招聘服务。各地各高校要在“百日冲刺”行动中对贫困家庭毕业生实施专项服务，组织其参加24365校园招聘等专项活动，积极帮助他们解决网上求职遇到的困难和问题。严格遵守疫情防控要求，有序恢复线下招聘，举办针对贫困家庭毕业生的小型供需对接活动。完善信息精准推送机制，通过短信息、微信群等，向每名贫困家庭毕业生推送3-5个符合其需求的针对性岗位，并向用人单位重点推荐。对有异地求职意愿的贫困家庭毕业生，依托大中城市联合招聘，组织跨地区服务协作和岗位共享，为其求职提供便利。加强人力资源市场监管，依法查处虚假招聘、违规收费、求职贷等侵害毕业生就业权益的行为。

　　四、提升就业能力。各地要为每名贫困家庭毕业生确定一名职业指导师，讲解就业形势政策、职业规划、求职技巧等，加强心理疏导和人文关怀。各类就业创业服务项目要向贫困家庭毕业生倾斜，对有培训需求的全部纳入培训计划，支持参加“互联网+”培训、专项能力培训、以工代训，按规定给予职业培训补贴、生活费补贴，使之至少掌握一种专项技能；对有创业意愿的全面纳入创业培训，指定创业导师全程跟踪指导，落实创业担保贷款、免费场地等支持政策，提升创业能力和创业成功率；对有见习需求的全员纳入见习安排，提供能够发挥其专长的见习岗位，增强就业竞争力，并优先推荐见习单位留用。

　　五、突出重点帮扶。各地要将建档立卡贫困家庭毕业生、52个未摘帽贫困县毕业生作为就业援助重点，实施结对帮扶、包干到人，优先提供岗位、优先推荐录用。对建档立卡贫困家庭学生实施“普通高校专升本专项计划”，单独进行录取。国有企业招聘、科研助理岗位吸纳、“三支一扶”“特岗教师”等基层服务项目招募，要在同等条件下优先录用建档立卡贫困家庭毕业生，事业单位可拿出一定数量的岗位招聘建档立卡贫困家庭毕业生。对符合条件的贫困家庭毕业生，可运用公益性岗位进行临时性安置。将离校未就业贫困家庭毕业生全部纳入实名制服务，列出就业需求清单、帮扶清单，量身定制求职计划，实施“一对一”帮扶。充分发挥东西部扶贫协作、对口支援机制作用，受援地要将贫困家庭毕业生信息提供给支援地，支援地要将其纳入就业政策扶持范围，项目建设、企业吸纳等岗位要优先录用。

　　六、加强组织领导。各地要坚持以人民为中心的发展思想，把做好贫困家庭高校毕业生就业帮扶工作作为重要政治任务，纳入就业工作目标责任体系和高校重点督促考核内容，加强领导，精心实施，层层压实工作责任。要结合实际细化工作措施，建立教育、人力资源社会保障和扶贫部门数据共享、工作协同、情况互通机制，强化学籍地、求职地、户籍地政策服务对接，做到摸排到位、帮扶到位、保障到位。加快就业创业政策落地实施，亮出政策清单，简化经办手续，推进网上办理，确保对贫困家庭高校毕业生就业补贴政策及时兑现。加强舆论宣传引导，选树一批贫困家庭毕业生自强不息、干事创业的优秀典型，营造全社会关心关爱贫困家庭毕业生就业的良好氛围。

　　人力资源社会保障部

　　教育部

　　国务院扶贫办

　　2020年7月15日

1. 最高法等3部门：依法妥善处理涉疫情旅游合同纠纷有关问题 法〔2020〕182号

各省、自治区、直辖市高级人民法院、司法厅（局）、文化和旅游厅（局），解放军军事法院，新疆维吾尔自治区高级人民法院生产建设兵团分院、新疆生产建设兵团司法局、新疆生产建设兵团文化体育广电和旅游局：

　　为贯彻落实党中央关于统筹推进疫情防控和经济社会发展工作部署，扎实做好“六稳”工作，落实“六保”任务，依法妥善化解涉疫情旅游合同纠纷，切实保障在常态化疫情防控中加快推进生产生活秩序全面恢复，抓紧解决复工复产面临的困难和问题，力争把疫情造成的损失降到最低限度，保障人民群众生命安全和身体健康，现将有关事项通知如下。

　　一、处理涉疫情旅游合同纠纷的基本要求

　　1.增强大局意识。旅游业是国民经济的重要支柱产业，推动旅游业平稳健康发展，对于促进经济平稳增长、持续改善民生具有重大意义。新冠肺炎疫情给旅游行业造成巨大冲击，由此导致旅游合同纠纷数量激增。文化和旅游部门、司法行政部门、人民法院要充分认识妥善处理旅游合同纠纷的重要意义，增强责任意识，发挥好行政机关与审判机关化解纠纷的职能作用，协同处理涉疫情旅游合同纠纷，为促进旅游业与经济社会持续发展、维护社会稳定提供服务和保障。

　　2.妥善化解纠纷。文化和旅游部门、司法行政部门、人民法院应当始终以法律为准绳，客观、全面、公平认定疫情在具体案件中对旅游经营者、旅游者造成的影响，在明确法律关系性质和合同双方争议焦点的基础上，平衡各方利益，兼顾旅游者权益保护与文化旅游产业发展，积极、正面引导旅游经营者和旅游者协商和解、互谅互让、共担风险、共渡难关，妥善化解纠纷，争取让绝大多数涉疫情旅游合同纠纷以非诉讼方式解决，维护良好的旅游市场秩序。

　　二、建立健全多元化解和联动机制

　　3.建立旅游合同纠纷多元化解机制。文化和旅游部门、司法行政部门、人民法院应当充分发挥矛盾纠纷多元化解机制作用，坚持把非诉讼纠纷解决机制挺在前面，强化诉源治理、综合治理，形成人民调解、行政调解、司法调解优势互补、对接顺畅的调解联动工作机制。文化和旅游部门、人民调解组织应当充分发挥调解职能作用，及时组织调解。司法行政部门应当组织律师积极参与旅游合同纠纷调解，充分发挥律师调解专业优势。当事人起诉的，人民法院可以征得当事人同意后，通过人民法院调解平台，委派或者委托特邀调解组织、特邀调解员进行调解。对调解不成的简易案件，人民法院应当速裁快审，努力做到能调则调，当判则判，及时定分止争。

　　4.畅通矛盾纠纷化解的协作对接渠道。文化和旅游部门、司法行政部门、人民法院应当发挥主观能动性，在兼顾法、理、情的基础上主动服务、创新服务。各部门、各单位之间主动加强沟通协调，共享信息，相互支持配合，形成工作合力。文化和旅游部门、司法行政部门对投诉、调解中反映出的新问题应及时与人民法院沟通。人民法院与当地文化和旅游部门、司法行政部门共同研判纠纷化解思路，确保纠纷处理的社会效果和法律效果统一。

　　5.充分发挥非诉讼纠纷化解机制作用。文化和旅游部门指导旅游经营者通过网络、电话、面谈等多种沟通方式加速涉疫情旅游合同纠纷的处理，简化流程、缩短时间；指导旅游经营者对员工进行培训，有效提升处理投诉人员业务水平，做好解释和安抚工作；做好涉疫情旅游合同纠纷的投诉处理工作，引导投诉人与被投诉人达成和解。人民调解组织可引导当事人选择人民调解调处矛盾纠纷并安排业务精通的调解员进行调解；律师调解工作室（中心）接到人民法院委派、委托调解或者接到当事人调解申请后，积极组织具有相应专业特长的律师调解员进行调解。当事人达成调解协议后，能够即时履行的即时履行，不能即时履行的明确履行时间，并引导当事人对调解协议申请司法确认。人民法院通过司法审查、司法确认等方式为非诉纠纷解决提供支持。

　　6.提供便捷高效的诉讼服务。人民法院开辟旅游合同纠纷诉讼绿色通道。有条件的地方可以充分发挥“旅游巡回法庭”在基层一线的作用，及时调处旅游合同纠纷。充分运用在线诉讼平台，开展线上调解、线上审判活动，切实将“智慧法院”用于解决群众实际困难。充分发挥小额速裁程序优势，通过快捷高效的法律服务，实现涉疫情旅游合同案件的快立、快审、快结。

　　三、依法妥善处理涉疫情旅游合同纠纷

　　7.严格执行法律政策。依据民法总则、合同法、旅游法，最高人民法院关于审理旅游纠纷案件适用法律若干问题的规定、关于依法妥善审理涉新冠肺炎疫情民事案件若干问题的指导意见（一），以及文化和旅游部办公厅印发的关于全力做好新型冠状病毒感染的肺炎疫情防控工作暂停旅游企业经营活动的紧急通知等相关法律、司法解释、政策，妥善处理涉疫情旅游合同的解除、费用负担等纠纷。

　　8.积极引导变更旅游合同。结合纠纷产生的实际情况，准确把握疫情或者疫情防控措施与旅游合同不能履行之间的因果关系，积极引导当事人在合理范围内调整合同中约定的权利义务关系，包括延期履行合同、替换为其他旅游产品，或者将旅游合同中的权利义务转让给第三人等合同变更和转让行为，助力旅游企业复工复产。旅游经营者与旅游者均同意变更旅游合同的，除双方对旅游费用分担协商一致的以外，因合同变更增加的费用由旅游者承担，减少的费用退还给旅游者。

　　9.慎重解除旅游合同。疫情或者疫情防控措施直接导致合同不能履行的，旅游经营者、旅游者应尽可能协商变更旅游合同。旅游经营者、旅游者未就旅游合同变更达成一致且请求解除旅游合同的，请求解除旅游合同的一方当事人应当举证证明疫情或者疫情防控措施对其履行合同造成的障碍，并已在合同约定的或合理的期间内通知合同相对人。旅游合同对解除条件另有约定的遵循合同约定。

　　10.妥善处理合同解除后的费用退还。因疫情或者疫情防控措施导致旅游合同解除的，旅游经营者与旅游者应就旅游费用的退还进行协商。若双方不能协商一致，旅游经营者应当在扣除已向地接社或者履行辅助人支付且不可退还的费用后，将余款退还旅游者。旅游经营者应协调地接社和履行辅助人退费，并提供其已支付相关费用且不能退回的证据，尽力减少旅游者因疫情或者疫情防控措施受到的损失。旅游经营者主张旅游者承担其他经营成本或者经营利润的，不予支持。旅游经营者应及时安排退费，因客观原因导致不能及时退费的，应当及时向旅游者作出说明并出具退款期限书面承诺。

　　11.妥善处理安全措施和安置费用的负担。因疫情影响旅游者人身安全，旅游经营者应当采取相应的安全措施，因此支出的费用，由旅游经营者与旅游者分担。因疫情或者疫情防控措施造成旅游者滞留的，旅游经营者应当采取相应的合理安置措施，因此增加的食宿费用由旅游者承担，增加的返程费用由旅游经营者与旅游者分担。

　　12.妥善认定减损和通知义务。旅游经营者、履行辅助人与旅游者均应当采取措施减轻疫情或疫情防控措施对合同当事人造成的损失，为防止扩大损失而支出的合理费用，可依公平原则予以分担。旅游经营者和旅游者应将受疫情或者疫情防控措施影响不能履行合同的情况及时通知对方，以减轻对方的损失。旅游经营者或旅游者未履行或未及时履行减损和通知义务的，应承担相应责任。

　　四、做好法律政策宣传工作

　　13.主动宣传法律、政策和典型案例。文化和旅游部门、司法行政部门、人民法院应当加大对涉疫情法律法规、政策文件等的解释和宣传力度，通过报纸、电视台、电台及各类新媒体解答涉疫情旅游合同纠纷热点问题，增强民众依法处理纠纷的自觉性，倡导旅游者理性维权。不断总结经验，宣传典型案例，提升涉疫情旅游合同矛盾纠纷多元化解机制在全社会的影响力和公信力。

　　14.共同维护社会稳定。涉疫情旅游合同纠纷牵涉面广、群体效应强，文化和旅游部门、司法行政部门、人民法院应密切关注各类媒体报道及投诉过程中的特殊情况，预防发生负面舆情和群体性事件，努力为统筹推进疫情防控和经济社会发展工作提供更加有力的服务和保障。

最高人民法院

司法部

文化和旅游部

　　2020年7月13日

1. 卫健委：印发《肉类加工企业新冠肺炎疫情防控指南》 联防联控机制综发〔2020〕216号

肉类加工企业新冠肺炎疫情防控指南

为科学指导肉类加工企业落实好新冠肺炎疫情防控工作要求，特制定本指南。本指南重点对存在生鲜、低温的肉类屠宰、分割、存储等工艺过程作出疫情防控要求，肉类加工企业的其他工艺过程可参照《关于依法科学精准做好新冠肺炎疫情防控工作的通知》(联防联控机制发〔2020〕28号)中《工业企业和建筑施工企业新冠肺炎防控技术方案》相关要求开展防疫工作。

一、组织保障和制度要求

（一）加强组织领导。肉类加工企业应当成立由主要负责人牵头的新冠肺炎疫情防控工作领导小组，全面领导疫情防控工作，确保各项措施落实。

（二）落实企业主体责任。肉类加工企业应当围绕关键环节和重点岗位，制订专门的疫情防控工作方案、应急处置预案和工作制度，包括组织领导体系、责任分工、排查制度、日常管控、后勤保障、应急处置等，并按要求严格落实。

（三）做好物资保障工作。肉类加工企业应当设立企业测温点和临时隔离点，结合应急方案储备足够数量的疫情防控物资，包括口罩、手套、消毒设备及用品、非接触式体温计等。

二、人员管控要求

（一）建立上岗员工健康卡制度。企业要做好新进员工近期行程及健康状况登记，建立上岗员工健康卡，切实掌握企业员工流动及健康情况。鼓励新进员工上岗前自愿接受核酸检测。

（二）加强日常健康监测。肉类加工企业应当加强人员出入管理和健康监测，建立全体员工健康状况台账和风险接触信息报告制度，设置测温点，落实登记、测温、消毒、查验健康码等防控措施，实行“绿码”上岗制。

（三）加强外来人员登记与管理。尽可能减少不必要的外来人员进入，确需进入厂区的，需询问单位、健康状况、接触疫情发生地区人员等情况，通过登记、测温、消毒、查验健康码等措施并佩戴口罩后方可入厂。车辆进出时，门卫值班员、工作人员和司机应当避免不必要的接触。

（四）加强防控知识宣传。开展多种形式的健康宣教，引导员工掌握新冠肺炎和其他呼吸道传染病防治相关知识和技能，养成良好卫生习惯，加强自我防护意识。

三、重点环节和重点场所防控要求

（一）源头管控。肉类加工企业应当把好禽畜肉类来源的“追溯关”“自查关”“检测关”“贮存关”，建立健全全程追溯机制，严禁加工不符合动物检疫规定或不符合食品安全标准的畜禽肉。除了索票索证和进货查验记录外，进口畜禽肉类食品应当具备《核酸检测合格证明》方可入厂生产。做好畜禽肉类食品转运存放区域、运输工具、货物外包装及其他相关用品用具的清洁和消毒。

（二）通风换气。普通厂房优先选择自然通风，如条件不具备可辅以机械通风。密闭厂房应当保持室内空气流通和空调系统供风安全，采用全新风模式，关闭回风系统，确保人均新风量≥30m3/h。空调通风系统应当定期进行检查、清洗、消毒，确保运行清洁安全。

（三）生产过程防护。

1.饲养车间。

（1）作业人员进入饲养车间需更换工作衣、鞋，走过消毒池，用消毒液洗手后入场。

（2）动物运输车辆进厂应当使用500～1000mg/L的含氯消毒液进行充分喷洒消毒，消毒作用30分钟。动物卸车台应当进行清洗消毒；卸车完毕，车辆经冲洗、喷雾消毒后方可离场。

（3）圈舍的通道、地面、墙面应当定期用1000mg/L的含氯消毒剂充分喷洒或擦拭消毒，消毒作用30分钟，后用清水冲洗干净。圈舍附近应有粪便集中储存设施，并及时清除。

（4）生病畜禽的运送应当备有不渗水密闭的专用运输工具，车间进出口处应有消毒池，排出的污水应当加入消毒药物处理。

2.屠宰车间。

（1）供宰动物应来自非疫区，并具有动物检疫合格证明和运载工具消毒证明，按国家有关规定、程序和标准进行宰前检验。

（2）作业人员进入屠宰车间需更换工作衣、鞋，走过消毒池，用消毒液洗手后入场。

（3）动物宰杀后放血处应当装有冷、热水刀具消毒器和洗手池，进行刀具消毒并洗手。

（4）屠宰车间每天屠宰完毕清洗后，用无腐蚀性的消毒剂对作业环境和设备进行消毒。

（5）兽医卫检员专用刀（钩）具，生产前后必须用500～1000mg/L的含氯消毒剂充分喷洒或擦拭消毒。

3.分割肉车间。

（1）作业人员进入分割肉车间需更换工作衣、鞋，走过消毒池，用消毒液洗手后入场。

（2）每班工作结束后必须彻底清洗加工场地的地面、墙壁、排水沟，然后使用1000mg/L的含氯消毒剂充分喷洒或擦拭消毒，再用清水冲洗干净。

（3）分割肉车间内的设备、工器具、操作台等物体表面应当每班用1000mg/L的含氯消毒剂充分喷洒或擦拭消毒，消毒作用30分钟。可移动的设备，如工作台、砧板，清洗时要将表面的肉屑用钢刷清除；不可移动的设备，如绞肉机、切片机、搅拌机、包装机、锯骨机等要将表面的肉屑清除干净；然后用消毒液喷洒消毒，再彻底洗净。

4.包装车间。

（1）作业人员进入包装车间需更换工作衣、鞋，走过消毒池，用消毒液洗手后入场。

（2）包装车间每天工作完毕后，用无腐蚀性的消毒剂对作业环境进行消毒。

5.冷库。

冷冻产品必须堆放在清洁平滑的垫板上，垫板应当经常清洗消毒。冷冻分割肉应当采用托盘式货架堆垛，人工进入冷库堆放时要穿库房用鞋，穿防寒服。

6.化验室。

（1）化验室应当有防潮、防蝇、防尘的设备。墙面、地面应当定期清扫消毒。

（2）化验室的空调应当定期进行检查、清洗、消毒，确保运行清洁安全。

（四）公共区域防控。

1.会议管理。应当控制会议频次、规模、时间，提倡采取网络视频、电话等线上方式召开会议和培训，确需开展现场活动的，需按规定向企业相关部门申请。参会人员需做好个人防护。会议过程中至少每小时进行一次开窗通风，确保人员之间的间隔在1米以上并按要求佩戴口罩。

2.就餐管理。员工食堂应当设置洗手设施和配备消毒用品，采取错峰用餐、分散就餐等措施，减少人员聚集，用餐时避免面对面就坐，避免与人交谈。

3.宿舍管理。员工集体宿舍应当严控入住人数，原则上每间不超过6人。宿舍应当设置可开启窗户，定时通风，每日至少开窗通风2次，每次不少于30分钟。对通风不畅的宿舍应当安装排风扇等机械通风设备。盥洗室配设洗手池和消毒用品，定时清洁。

4.公共区域消毒。安排专人对办公区域、会议场所、宿舍及其他人员活动场所和相关物品定时清洁消毒，如门把手、楼梯扶手、电梯按键等频繁接触部位应适当增加消毒次数。用500mg/L含氯消毒液进行喷洒或擦拭消毒，对不耐腐蚀的表面和用品用具用清水冲洗干净。

（五）环境定期监测。加强重点场所风险排查，开展环境中核酸的定期监测工作，建立环境定期核酸监测档案。中风险和高风险地区肉类加工企业应当每天在屠宰车间、分割肉车间、包装车间采集各5份环境标本进行核酸监测。低风险地区肉类加工企业应当每周至少进行一次采样监测。

四、个体防护

从业人员在岗时应当根据岗位需要正确佩戴口罩、手套等，做好个人防护。

（一）检验检疫人员。检验检疫人员上岗前应当穿戴好工作服、手套、一次性使用医用口罩或一次性医用外科口罩、防护镜、胶靴等。

（二）待宰区工作人员。待宰区工作人员上岗前应当穿戴好工作服、手套、一次性使用医用口罩或一次性医用外科口罩、面屏或护目镜、胶靴等。工作过程中应当避免直接接触动物的血液和体液。参与急宰的工作人员完成急宰工作后应当彻底洗手、消毒。工作结束后，应当做好衣服和器械的清洗、消毒。

（三）屠宰工作人员。屠宰工作人员进入屠宰车间前应当穿戴好工作帽、一次性使用医用口罩或一次性医用外科口罩、面屏、手套、工作服、胶靴等防护用品。放血工人和与水接触较多的工人应当穿防水的衣裤。屠宰技术人员不得徒手操作。工作结束后，人员应当采用有效浓度的消毒液对设备、工器具、场地等进行消毒。工衣统一收集、清洗、消毒。

（四）分割加工人员。分割加工人员进入车间前应当穿戴工作帽、一次性使用医用口罩或一次性医用外科口罩、面屏、手套、工作服和胶靴，根据岗位需求穿戴相应的围裙和雨衣。生产结束后，人员应当采用有效浓度的消毒液对设备、工器具、场地等进行清洗、消毒；人员应当洗澡后离开车间。分割车间员工工衣应当和其他区域员工工衣分开清洗、消毒。

（五）包装和副产品处理人员。包装和副产品处理人员进入车间应当穿戴工作帽、一次性使用医用口罩或一次性医用外科口罩、面屏、手套、工作服、防水围裙、防水套袖和防潮防滑的胶靴。进入车间和生产结束均需按照流程进行洗手消毒和鞋靴的清洗、消毒，生产结束后洗澡离开车间。

（六）低温环境工作人员的防护。低温环境工作人员进入车间前应当穿戴工作帽、一次性使用医用口罩或一次性医用外科口罩、面屏、手套、工作服和胶靴，根据岗位需求穿戴相应的围裙和雨衣。冷冻、冷藏车间的人员进入工作区时应当穿戴有防护耳朵的棉帽、棉服和棉鞋，并佩戴手套。

（七）外围工作人员的防护。维修、制冷、配电、污水处理及无害化处理等岗位工作人员进入工作岗位前应当穿戴适宜的工作服、工帽、工鞋及其他要求佩戴的个体防护用品，并保持佩戴一次性使用医用口罩或一次性医用外科口罩。

（八）减少员工的密切接触。合理安排员工的作业班次，降低加工车间分割、切片、挑拣、清洗、包装等作业工序的员工密度，2名员工之间应当保持至少1米的安全距离。

五、异常情况处置与报告

（一）核酸检测阳性样品的处置措施。一旦接到当地疾病预防控制机构通知有新冠病毒核酸检测阳性的样品，企业应当根据当地要求对相关物品和环境采取及时应急处置，迅速启动本单位应急预案，在专业人员指导下采取对相关物品临时封存、工作场所进行消毒处理和对可能接触人员及时开展核酸检测和健康筛查等措施。相关物品按照医疗废弃物集中转运处理，应当使用双层黄色垃圾袋盛装，封口严密，避免运输过程溢洒或泄露。参与相关物品清运工作的人员应当做好个人防护，建议穿戴工作服、一次性工作帽、一次性手套、防护服、医用防护口罩、护目镜或防护面屏、工作鞋或胶靴、防水靴套等。

（二）员工出现异常状况的应急处置。

1.企业应当设立隔离观察区域，员工出现可疑症状或排查发现为密切接触者时，企业应当及时报告当地疾病预防控制机构，配合实施集中隔离医学观察或居家医学观察，并按要求封闭其所在工作场所以及员工宿舍等生活场所，严禁无关人员进入，同时在专业人员指导下对其活动场所及使用物品进行消毒。

2.企业一旦发现病例，必须实施内防扩散、外防输出的防控措施，配合有关部门开展流行病学调查、密切接触者追踪管理、疫点消毒等工作，并对该员工作业的工作场所及其加工的禽肉类进行采样和核酸检测。根据疫情严重程度，暂时关闭工作场所，待疫情得到控制后再恢复生产。

国务院应对新型冠状病毒肺炎疫情

联防联控机制综合组

（代  章）

2020年7月21日

1. 发改委：全力做好下半年稳就业保就业有关工作 发改办就业〔2020〕557号

各省、自治区、直辖市、新疆生产建设兵团发展改革委：

  今年以来，突如其来的新冠肺炎疫情对我国经济运行和就业造成巨大冲击。在以习近平同志为核心的党中央坚强领导下，各地方各有关部门履职尽责，统筹推进疫情防控和经济社会发展，集中精力抓好“六稳”工作、落实“六保”任务，在做好常态化疫情防控的前提下，不失时机推进复工复产，推出减税降费、金融支持等一系列援企稳岗政策，加大对高校毕业生、农民工等重点群体的就业帮扶力度，促进经济回升和就业大局稳定。经过全国上下和广大人民群众的共同努力，我国经济下滑的困难局面逐步得到扭转，就业形势保持总体稳定。但同时也要看到，就业领域仍面临诸多困难和挑战，重点群体就业压力持续攀升，就业形势依然复杂严峻。为进一步做好下半年稳就业保就业工作，现将有关事项通知如下：

  一、提高认识、勇于担当，坚决扛起稳就业保就业的政治责任。就业是最大的民生。各地发展改革部门要提高政治站位、主动担当作为，认真履行职责，着力攻坚克难，切实增强紧迫感、责任感，不折不扣贯彻落实党中央、国务院决策部署，把就业摆在“六稳”、“六保”首要位置，在补短板、强弱项上下功夫，在想办法、拓思路上下功夫，系统谋划、精心部署下半年稳就业保就业工作。

  二、立足职能、发挥优势，认真做好就业形势分析研判。各地发展改革部门要保持战略定力、把握宏观大势，深入开展调查研究，综合运用实地调研、问卷调查、大数据分析等多种方式，在全面掌握实际情况基础上，科学研判下半年就业走势，重大情况及时上报。对受疫情影响较大的重点行业、重点企业、重点群体，要持续开展跟踪监测和动态分析，完善监测分析机制，扩大监测范围，紧盯关键性指标，加强分类监测。进一步强化规模性失业风险预警和防范，及时开展就业影响程度评估，结合实际制定稳就业预案。强化政策储备，在援企稳岗、就业帮扶等方面，提出更多有针对性、可操作的政策建议，更好发挥参谋助手作用。

  三、统筹资源、凝聚合力，加大各方面对就业的支持力度。要正视当前困难、树立底线思维，充分发挥综合部门的优势，集中政策资源，强化要素支撑，加强项目资金投入，促就业举措要应出尽出，拓岗位办法要能用尽用。更好地发挥投资对就业的拉动作用，合理调配各类资金流向，优先支持具有民生效益和就业效应的项目。用好用足抗疫特别国债和地方政府专项债券，重点支持带动就业能力强的补短板项目。扎实推进新型城镇化建设，大力提升县城公共设施和服务能力，开发更多就业岗位。因地制宜适当扩大今年以工代赈规模，将更多返乡留乡贫困劳动力纳入政策覆盖范围。进一步优化营商环境，激发市场主体活力和社会创造力。大力发展数字经济，充分发挥双创示范基地带动作用，加快双创支撑平台项目建设，鼓励创业带动就业。支持养老、托育、医疗、家政等社区家庭服务业发展壮大，吸纳更多就业。实施适当政策倾斜，扩大省级资金投入，撬动更多社会资源，支持返乡入乡创业和长江流域渔民退捕安置，带动更多农民工等人员就地就近就业创业。

  四、创新方式、完善机制，坚持就业优先导向的宏观调控。全面强化就业优先政策，注重财政、货币、金融、产业、区域、贸易等经济政策与就业政策的衔接配套、协调联动，努力实现经济发展与扩大就业良性互动。宏观政策酝酿出台要评估其对就业的影响，政策措施的操作落实要统筹考虑其给就业带来的变化。充分发挥劳动力市场信号和就业目标的引导作用，根据就业形势变化，及时调整宏观政策取向，统筹考虑宏观调控的重点和节奏，千方百计稳定和扩大就业。

  五、上下联动、协作配合，切实抓好政策落实落地。要把各级发展改革部门的力量充分调动起来，瞄准已出台的稳就业政策措施，加大抓落实力度，级级传导压力、层层压实责任，确保“件件有着落、事事有回音”。积极主动推动教育、财政、人社、商务等有关部门和地方政府落实好稳定中小微企业、个体工商户和外贸行业生产经营及用工，促进高校毕业生和农民工等重点群体就业的各项任务举措，务求尽快见实效。不断完善部门联动的政策协调机制，推动数据和信息资源共享，会商重大问题，协同制定措施，增进工作合力。

  当前，全球疫情和经贸形势的不确定性依然很大，就业领域仍面临一些难以预料的影响因素。全国发展改革系统要增强“四个意识”，坚定“四个自信”，做到“两个维护”，以习近平新时代中国特色社会主义思想为指导，深入贯彻落实党中央、国务院关于稳就业保就业的决策部署，坚定信心、主动作为，为实现“十三五”规划目标、全面建成小康社会、打赢脱贫攻坚战作出积极贡献。

国家发展改革委办公厅

2020年7月21日

1. 最高检：充分发挥检察职能服务保障“六稳”“六保”

为贯彻落实党中央关于做好“六稳”工作、落实“六保”任务的重大决策部署，主动服务统筹推进疫情防控和经济社会发展工作，最高人民检察院日前下发《关于充分发挥检察职能服务保障“六稳”“六保”的意见》（下称《意见》），提出了11条具体举措，对充分发挥检察职能服务保障“六稳”“六保”作出细化要求。

《意见》指出，要落实在疫情防控常态化条件下加快恢复生产生活秩序的要求，重点惩治妨害复工复产、妨害疫情防控、网络犯罪、非法放贷、“套路贷”等破坏复工复产和经济社会发展的犯罪，为“六稳”“六保”营造稳定的社会环境。要以学习贯彻民法典为契机，坚持运用法治思维和法治方式，积极推进涉疫矛盾纠纷化解，维护社会和谐稳定。

《意见》要求，依法保护企业正常生产经营活动，加大知识产权司法保护力度。依法严格追诉职务侵占、非国家工作人员受贿和挪用资金犯罪，综合考虑其犯罪行为对民营企业经营发展、商业信誉、内部治理、外部环境的影响程度，精准提出量刑建议。依法慎重处理贷款类犯罪案件，充分考虑企业“融资难”“融资贵”的实际情况，合理判断借款人的行为危害性。依法慎重处理拒不支付劳动报酬犯罪案件，注意把握企业因资金周转困难拖欠劳动报酬与恶意欠薪的界限。严格把握涉企业生产经营、创新创业的新类型案件的法律政策界限，对于企业创新产品与现有国家标准难以对应的，应当进行实质性评估，防止简单化“对号入座”。依法着力保护与疫情防控相关的诊断检测技术、医用呼吸防护产品、疫苗研制等领域的知识产权。重点打击涉及高新技术、关键核心技术，以及网络侵权、链条式产业化有组织侵权等严重侵权假冒犯罪。加大对采用盗窃、利诱、欺诈、胁迫、电子侵入或者其他不正当手段侵犯商业秘密犯罪的打击力度。依法妥善办理科研人员涉嫌职务犯罪案件。

《意见》要求，依法惩治破坏金融管理秩序犯罪，维护有利于对外开放的法治化营商环境。依法“全链条”从严追诉欺诈发行股票、债券，违规披露、不披露重要信息和提供虚假证明文件等犯罪，全面落实对资本市场违法犯罪“零容忍”要求。严惩不法分子借互联网金融名义实施的非法吸收公众存款、集资诈骗等犯罪，从严追诉组织者、领导者。加大惩治洗钱犯罪的力度。依法惩治侵害外国投资者和外商投资企业合法权益，以及扰乱投资秩序、妨害项目推进的各类犯罪，保障外商投资法顺利施行。依法惩治利用外贸合同诈骗，虚开出口退税、抵扣税款发票，骗取出口退税以及对外贸易经营活动中的走私、逃汇骗汇等犯罪。依法慎重处理企业涉税案件。

《意见》强调，努力为决战决胜脱贫攻坚提供司法保障，积极促进基层依法治理。突出对重点领域和弱势群体的司法保护，依法严惩侵害群众切身利益的腐败犯罪，加强扶贫领域涉案财物依法快速返还工作，符合快速返还条件的，依法作出决定并于五日内将涉案财物返还给被侵害的个人或单位。突出对困难群体的司法救助，对严重暴力犯罪造成被害人重伤、死亡的，或者被害人家庭因案致贫、因案返贫的，要结合具体案情及时、主动给予司法救助。突出对未成年人的司法保护，持续推进“一号检察建议”落实，加大对侵害农村留守儿童、困境儿童等犯罪打击力度。严惩“蝇贪”“蚁贪”，从严追诉发生在基层的、影响恶劣的贪污贿赂犯罪尤其是吃拿卡要型索贿犯罪，严惩“村霸”和宗族恶势力，依法查办司法工作人员利用职权实施的侵害公民权利、损害司法公正的犯罪。深入推进行政争议实质性化解，通过促进和解、公开听证、司法救助、释法说理等方式，促进基层依法行政，提高公共服务能力。

《意见》特别指出，落实“少捕”“少押”“慎诉”的司法理念，依法合理采取更加灵活务实的司法措施。坚持依法能不捕的不捕，注重将犯罪嫌疑人认罪认罚积极复工复产、开展生产自救、努力保就业岗位作为审查判断有无社会危险性的重要考量因素。积极探索总结非羁押强制措施适用经验，认真履行羁押必要性审查职责，减少不必要的羁押。坚持依法能不诉的不诉，逐步扩大酌定不起诉在认罪认罚案件中的适用，同时防止不起诉后一放了之。慎重适用涉财产强制性措施，对涉嫌犯罪但仍在正常生产经营的各类企业，原则上不采取查封、扣押、冻结措施。优化刑罚执行环节司法措施，扩大涉企服刑人员假释的适用，为接受社区矫正的民营企业人员从事相关生产经营活动提供必要便利，简化批准流程。妥善采取公益诉讼案件司法措施，慎用关停涉案企业等影响企业生存和正常生产经营的措施，探索通过分期支付、替代性修复等方法促使相关企业接受惩罚、守法经营、健康发展。

《意见》强调，加大对涉民营企业各类案件的法律监督力度。加强立案监督，着重纠正涉及企业犯罪案件不应当立案而立和应立不立等突出问题，坚决防止和纠正以刑事案件名义插手民事纠纷、经济纠纷等各类违法行为。加大清理涉民营企业刑事诉讼“挂案”力度，推动建立长效机制，维护企业和当事人合法权益。加强涉企行政非诉执行监督，防止企业因不当强制执行措施陷入生产经营困境。加强控告申诉案件办理答复工作，对涉及民营企业的控告申诉案件进行集中清理和统一管理，做到件件有回音，事事有着落。

2020年7月24日 最高人民检察院

1. 中央政法委：依法保障和服务疫情防控常态化条件下经济社会发展

为认真贯彻落实以习近平同志为核心的党中央关于统筹推进常态化疫情防控和经济社会发展工作的重大决策部署，中央政法委近日印发《关于依法保障和服务疫情防控常态化条件下经济社会发展的指导意见》，要求政法机关忠实履行职能，充分发挥法治固根本、稳预期、利长远的重要作用，推动实现高质量发展、高效能治理。

《意见》指出，要运用好执法司法政策，以稳市场主体推动做好“六稳”“六保”工作。依法严厉打击合同诈骗、非法经营等破坏市场经济秩序的犯罪和侵害经营主体人身、财产权利的违法犯罪活动。落实“少捕慎诉”的司法政策，依法减少羁押性强制措施适用，减少社会对抗，促进社会和谐稳定。依法慎重适用财产强制措施，依法妥善处理涉疫情民事纠纷，依法合理采取民事执行措施，鼓励和引导当事人互谅互让、共担风险，通过和解、调解等方式解决纠纷。

《意见》强调，要强化营商环境执法司法保障，充分激发各类市场主体活力。坚持各类市场主体法律地位平等、权利保护平等和发展机会平等，推动健全以公平为原则的产权保护制度，建立完善公平公正的司法审判机制和司法监督机制，营造支持民营经济健康发展的执法司法环境。准确界定企业生产自救过程中的行为性质，严格区分经济纠纷和经济犯罪。落实和完善对新技术、新产业、新业态、新商业模式的依法包容审慎监管，推动企业提升科技创新力和市场竞争力。切实保障科研人员依法享有的职务科技成果所有权和长期使用权，促进更多科技成果转化为现实生产力。尊重和保护市场主体意思自治，提高运用法治思维和法治方式推动企业发展的能力和水平。

《意见》要求，要服务国家重大战略，推动形成高质量发展新格局。加大户籍制度改革和农业转移人口市民化落实力度，推进市域社会治理现代化，创新加强基层社会治理体制机制，推动新型城镇化建设。围绕重大区域发展战略，推动立法授权，加强区域执法司法协作和法律服务。全面实施外商投资法及配套法规、司法解释，平等保护中外投资者合法权益，完善国际商事纠纷多元化解决机制，助力高水平对外开放。

《意见》强调，要用法治护航人民群众美好生活，不断充实提升人民群众获得感、幸福感、安全感。积极推进平安中国建设，加快完善社会治安防控体系，深入推进扫黑除恶深挖根治和长效常治，严格依法公正办理涉黑涉恶案件，完善矛盾纠纷源头预防、排查预警、多元化解机制，依法严厉打击影响社会安全稳定的各类违法犯罪活动，保障人民群众生命财产安全。依法防范打击脱贫攻坚领域违法犯罪活动，加大环境公益诉讼工作力度，依法严惩非法捕杀、交易野生动物犯罪行为。抓好民法典贯彻实施，加强民事审判工作和民事检察工作，增强尊重民事权利主体、保护公民私权的意识，严格落实人格权保护制度，让人民群众生活更有尊严、更为体面。推动民法典普法工作制度化、常态化、长效化，努力把民法典的文本规定变成人民群众的行为准则。

《意见》要求，要创新政法公共服务体制机制，提供更多优质政法公共产品。持续推进“互联网+公安政务服务”平台建设，优化行政审批服务，在交管服务、移民和出入境管理服务等领域持续研究探索新的便利政策措施。探索互联网审判机制和审理规则，健全立体化诉讼服务渠道。完善“12309检察服务中心”，构建融检察服务、检察宣传、监督评议于一体的检察服务公共平台。整合律师、公证、司法鉴定、仲裁、司法所、人民调解等法律服务资源，加快建成覆盖全业务、全时空的法律服务网络。

2020年7月21日

1. 民航局等3部门：印发《关于来华航班乘客凭新冠病毒核酸检测阴性证明登机的公告》 民航公告【2020】9 号

为确保国际旅行健康安全，降低疫情跨境传播风险，对来华航班乘客实行凭新冠病毒核酸检测阴性证明登机的做法。安排如下：

　　一、搭乘航班来华的中、外籍乘客在登机前5天内完成核酸检测。检测应在中国驻外使领馆指定或认可的机构进行。

　　二、中国籍乘客通过防疫健康码国际版小程序拍照上传核酸检测阴性证明。

　　三、外国籍乘客凭核酸检测阴性证明向中国使领馆申办健康状况声明书。

　　四、有关航空公司负责在登机前查验健康码状态和健康状况声明书。不符合相关要求的乘客不能登机。各航空公司应严格履行查验手续。

　　五、乘客提供虚假证明和信息，须承担相应法律责任。

　　六、中国大使馆将认真评估驻在国核酸检测能力，并在具备条件的时候发布具体实施办法。

　　特此公告。

　　民航局

　　海关总署

　　外交部

　　2020年7月20日

1. 交通运输部：稳妥有序恢复省际旅游客运切实做好旅游客运常态化疫情防控有关工作 交运明电〔2020〕220号

各省、自治区、直辖市、新疆生产建设兵团交通运输厅（局、委）：

　　根据国家关于旅游领域复工复产的有关部署，旅行社跨省（区、市）团队旅游业务将逐步恢复。为稳妥有序恢复省际旅游客运，切实做好旅游客运常态化疫情防控，现就有关工作通知如下：

　　一、准确把握形势，统一思想认识

　　当前，北京市已连续14天以上无新增本地新冠肺炎确诊病例，突发公共卫生事件应急响应级别已调整为三级，首都疫情防控总体形势持续稳定向好，但仍需谨防输入性风险；同时，新疆乌鲁木齐近日接连新增本地确诊病例，初期病例与一起聚集性活动关联，表明我国疫情防控形势依然严峻复杂。当前，我国已经全面进入“七下八上”主汛期，防汛抗洪形势也十分严峻。各地交通运输主管部门要进一步提高政治站位，坚持底线思维，紧密结合夏季疫情防控、汛期安全生产等形势，分区分级统筹精准做好旅游客运复工复产、疫情防控、安全生产等各方面工作，严密防范疫情通过旅游客运环节传播，坚决防止发生重特大旅游客运安全生产事故，为常态化疫情防控条件下加快恢复生产生活秩序提供安全可靠的运输保障。

　　二、稳妥有序恢复省际旅游客运

　　各省级交通运输主管部门要加强与文化和旅游部门沟通对接，密切跟进本地跨省（区、市）团队旅游业务恢复运营安排，在做好疫情防控工作的前提下，可与跨省（区、市）团队旅游业务同步恢复，国内游轮运输要在充分、科学、综合评估分析的基础上，严格按照《国内游轮常态化疫情防控工作指南》（见附件）审慎有序复航。进出北京和新疆的省际旅游客运业务恢复安排，按照北京市和新疆维吾尔自治区疫情防控领导机构的有关部署执行。

　　各地交通运输主管部门要督促指导本地省际旅游客运经营者（含开展非定线旅游客运业务的包车客运经营者和定线旅游客运业务的班车客运经营者、水路旅游客运经营者，下同）做好复工复产相关准备；在本省（区、市）省际旅游客运业务恢复后，依托省级包车客运管理信息系统，督促省际旅游客运经营者严格执行省际旅游客运业务备案要求，及时如实备案经营者、车牌号码、驾驶员、起讫地、主要途经地、有效期、包车合同等信息，规范省际包车客运标志牌发放管理；要配合执行“中、高风险地区不可开展团队旅游业务及‘机票+酒店’业务”要求，督促旅游客运企业不得承接出入中、高风险地区的团队旅游用车、乘船业务。省际旅游客运经营者要会同旅行社查验游客“健康码”，确保所有游客全部来自低风险地区。游轮运输相关企业应认真落实实名制管理规定，加强旅客信息登记和留存，确保相关信息可以追溯。

　　三、抓紧抓实抓细常态化旅游客运疫情防控工作

　　各地交通运输主管部门要加强督导检查，指导旅游客运经营者严格落实国务院联防联控机制综合组下发的《低风险地区夏季重点场所重点单位重点人群新冠肺炎疫情常态化防控相关防护指南（修订版）》和交通运输部下发的最新版《客运场站和交通运输工具新冠肺炎分区分级防控指南》等疫情防控指南，做好旅游客运车辆、国内游轮及相关客运场站消毒、通风、人员防护、体温检测、发热人员移交、客座率控制等防疫措施。要督促旅游客运经营者配合执行旅游景区“限量、预约、错峰”等限制客流措施，强化运力供给，提高疏运能力，减少游客聚集；要会同有关部门加强宣传，引导游客落实戴口罩、手卫生、“一米线”等防护举措，阻断疫情传播途径。

　　四、坚决守住旅游客运安全生产底线

　　各地交通运输主管部门要深入贯彻落实习近平总书记关于安全生产工作的重要指示批示精神，坚持人民至上、生命至上，始终把安全生产工作放在首要位置，保持高度警醒、高度戒备、高度负责，从严从深、从细从实全力做好旅游客运安全生产工作。要切实压实企业主体责任，督促指导省际旅游客运经营者落实全员安全生产责任制，严格安全生产隐患排查治理和风险防控；加强旅游客运车辆、游轮和从业人员管理，保障车辆（船舶）技术状况良好、车载卫星定位装置完备有效，严防车辆（船舶）带病运营、脱离动态监控投入运营。要以交通运输安全专项整治三年行动为契机，会同公安、文化和旅游等部门形成工作合力，建立健全联合执法、信息共享等工作机制，强化暑期、节假日等重点时段，旅游景区（点）、旅游集散中心等重点区域的旅游客运监督管理，严厉打击旅游客运领域非法营运等各类违法违规行为。

　　五、突出抓好汛期旅游客运安全和应急管理

　　各地交通运输主管部门要深入贯彻习近平总书记关于防汛救灾工作的重要指示精神，认真落实党中央、国务院关于防汛防台风工作有关部署，坚持预防为主，全力做好汛期旅游客运安全生产工作，全力维护行业安全形势稳定。要会同气象、应急、水利等部门加强汛情预警通报，及时发布极端天气信息，依托客运站、航行通告、公路情报板、广播、新媒体等渠道加强汛情严重地区运行的客运车辆安全预警。要督促旅游客运经营者针对复工初期、夏季汛期等特点，加强驾驶员、船员教育培训，提升安全意识、恶劣天气条件下安全驾驶技能和应急处置能力；会同旅行社强化旅游线路安全风险评估，避开汛情严重区域；依托企业动态监控平台加强出行前和行程中预警提示，及时纠正从业人员不安全从业行为，杜绝冒险行车（航行），行程中一旦遭遇突发汛情，安全通行条件不足的，要会同旅行社采取有效措施应对，并暂停旅游经营活动，切实降低途中事故风险。要按照相关预案要求，加强汛期和台风防御期间应急值守，及时核报突发事件信息，强化应急信息调度，发生重大以上突发事件要立即开展部、省、现场三级视频调度，统筹协调应急资源，全力高效做好交通运输突发事件应急处置和信息报送工作。

　　各地交通运输主管部门要加强与文化和旅游、卫生健康等部门沟通协作，结合当地疫情形势变化和旅游客运的实际，及时调整策略，确保省际旅游客运恢复安全有序。

　　铁路、民航领域相关工作由国家铁路局、中国民用航空局进行部署。

　　附件：国内游轮常态化疫情防控工作指南

交通运输部

2020年7月21日

1. 卫健委：做好托育机构复托相关工作 国卫办人口函〔2020〕469号

各省、自治区、直辖市及新疆生产建设兵团卫生健康委：

　　为全面贯彻党中央关于抓紧抓实抓细常态化疫情防控、做好当前经济社会发展各项工作的决策部署，安全有序推进托育机构恢复入托，现就有关事项通知如下：

　　一、坚持属地管理的原则，按照当地党委政府的统一部署，认真评估中小学开学复课情况，参照幼儿园复园时间，确定托育机构复托时间。

　　二、认真组织检查评估托育机构复托前各项准备工作，未达到《托幼机构新冠肺炎疫情防控技术方案（修订版）》（国卫办疾控函﹝2020﹞363号）各项要求的，不得复托。

　　三、加强托育机构日常健康管理，严格实行工作人员和婴幼儿健康状况“日报告”、“零报告”制度，落实入托婴幼儿晨（午）检、全日观察、因病缺勤病因追查及登记等防控措施，做好场所通风换气、清洁灭菌等工作，切实保障婴幼儿生命安全和身体健康。

　　四、主动协调编制、民政、市场监管等部门，加快推进托育机构登记和备案工作。县级卫生健康行政部门应在2020年7月31日前，完成托育机构备案信息系统管理用户注册。

　　五、加强调查研究，及时了解托育机构面临的现实困难和突出问题，积极协调发展改革、财政、人力资源社会保障、金融、税务等部门，在落实好国家各项优惠政策的同时，结合本地实际，研究制订促进托育机构发展的支持政策。

国家卫生健康委办公厅

2020年6月15日

1. 国家电影局：在疫情防控常态化条件下有序推进电影院恢复开放 国影发【2020】1号

各省、自治区、直辖市电影主管部门，各电影院线公司，各制片单位，中国电影发行放映协会，中国电影制片人协会：

为深入贯彻习近平总书记关于统筹推进新冠肺炎疫情防控和经济社会发展工作的重要指示精神，经国务院应对新型冠状病毒感染肺炎疫情联防联控机制同意，现就在疫情防控常态化条件下有序推进电影院恢复开放通知如下。

一、坚持分区分级原则。低风险地区在电影院各项防控措施有效落实到位的前提下，可于7月20日有序恢复开放营业。中高风险地区暂不开放营业。一旦从低风险地区调整为中高风险地区，要严格执行疫情防控规定，从严从紧落实防控措施，电影院及时按要求暂停营业。

二、严格落实属地管理和行业管理责任。各地电影主管部门将关于恢复开放电影院的工作安排报当地党委和政府同意后，商当地疫情防控部门有序推进恢复营业。要制定疫情防控方案和应急预案，对复工电影院加强指导和巡查，密切关注电影院疫情防控及复工经营情况。各地具体复工时间及有关安排向国家电影局报备。

三、精准科学落实防控措施。电影院恢复开放要把防疫安全放在第一位，夯实院线、影院的主体责任，严格遵守当地疫情防控部门要求，健全制度，完善措施，切实做到常态防控到位、预约限流到位、排片间隔到位、人员排查到位、环境消毒到位、应急处置到位。

各地区各单位要认真执行本通知要求，按照《中国电影发行放映协会电影放映场所恢复开放疫情防控指南》，抓紧抓实抓细各项防控工作，稳妥有序推进电影院恢复开放，执行过程中遇有情况及时上报。

国家电影局

2020年7月16日

1. 文旅部：印发《关于推进旅游企业扩大复工复业有关事项的通知》

各省、自治区、直辖市文化和旅游厅（局），新疆生产建设兵团文化体育广电和旅游局：

　　按照党中央、国务院统筹推进新冠肺炎疫情防控和经济社会发展的决策部署，结合当前疫情防控总体形势，为扎实做好“六稳”工作，全面落实“六保”任务，推进旅游企业扩大复工复业，现就有关事项通知如下：

　　一、工作事项

　　(一)恢复跨省（区、市）团队旅游。各省（区、市）文化和旅游行政部门在做好疫情防控工作的前提下，经当地省（区、市）党委、政府同意后，可恢复旅行社及在线旅游企业经营跨省（区、市）团队旅游及“机票+酒店”业务。中、高风险地区不得开展团队旅游及“机票+酒店”业务。出入境旅游业务暂不恢复。

　　（二）调整旅游景区限量措施。旅游景区要继续贯彻落实“限量、预约、错峰”要求，接待游客量由不得超过最大承载量的30%调至50%。在严格落实各项防控措施的前提下，采取预约、限流等方式，开放旅游景区室内场所。

　　二、工作要求

　　（一）强化疫情防控管理。各地要坚持把疫情防控摆在首位，统筹做好旅游安全等各项工作。要按照“谁组织、谁管理、谁负责”的原则，进一步压实旅游企业主体责任，指导旅游企业制定应急预案，明确疫情防控和安全突发事件应急措施和处置流程。要督促旅行社控制团队规模，做好行程管理，将防控措施贯穿游客招徕、组织、接待各环节。在线旅游企业参照执行《旅行社有序恢复经营疫情防控措施指南》。

　　（二）规范旅游市场秩序。各地要创新发展理念，支持旅游企业综合运用科技创新成果，推动线上线下融合发展，扩大复工复业，加快转型升级，着力提升服务质量。行业组织要倡导诚信经营，强化行业自律，加强自我监督。旅游企业要落实质量主体责任，严格执行产品质量标准和服务规范，不断增强游客满意度。

　　（三）加强旅游景区开放管理。各地旅游景区要按照《旅游景区恢复开放疫情防控措施指南》要求，加强清洁消毒和垃圾分类处理。要在重要游览点、观景平台、交通接驳点、狭窄通道、购票餐饮等容易形成游客拥堵的区域，采取有针对性的措施，加强疏导，指导游客做好安全防护。

　　（四）树立文明旅游新风尚。各地要通过多种形式开展文明旅游宣传，引导游客遵守旅游活动中的疫情防护要求、安全警示和文明旅游规定；鼓励和支持旅游企业创新服务模式，推广“分餐制”“公筷制”等健康旅游新方式；加强对游客的宣传教育，引导游客讲究卫生、拒绝野味、理性消费，树立文明、健康、绿色旅游新风尚。

　　（五）提升应急处置能力。各地要严格落实属地管理责任，加强与当地卫生健康部门之间的联动，强化旅游目的地和客源地主管部门之间的协作，提升防控和应急处置能力。按照防控方案和应急预案，开展应急演练、隐患排查、风险评估等工作，及时发现苗头性问题并有效处置。发生异常情况要及时上报并暂停有关经营活动。

　　（六）做好汛期旅游安全工作。各地要始终绷紧汛期旅游安全这根弦，进一步压实责任，根据当地防汛抗旱指挥部的安排，加强对索道、缆车、大型游乐设施等设备的安全检查，达不到安全要求的坚决停止运营或使用；加大水上旅游项目检查指导，督促企业及时关注雨情预报和水情变化，密切关注汛情预报，避免组织旅游团到汛情严重的区域旅游；正在行程中的旅游团一旦遭遇突发汛情，要采取有效措施应对，必要时暂停旅游经营活动。

　　特此通知。

附件：旅行社有序恢复经营疫情防控措施指南

文化和旅游部办公厅

2020年7月14日

旅行社有序恢复经营疫情防控措施指南

　　按照党中央、国务院统筹推进新冠肺炎疫情防控和经济社会发展的决策部署，结合当前疫情防控总体形势，为指导全国旅行社做好疫情常态化防控和有序恢复经营工作，制定本指南。

　　一、总体要求

　　（一）坚持常态防控。各地文化和旅游行政部门应当按照属地原则，把疫情防控摆在首位，结合团队旅游涉及范围广、流动性大、链条长等特点，制定疫情防控方案和应急预案，及时动态调整。指导旅行社严格执行疫情防控规定，按照相关技术指南，制定企业经营疫情防控方案和应急预案，建立应对机制，开展应急演练，提高处置能力。要按照“谁组织、谁管理、谁负责”的原则，压实企业主体责任，把防控责任落实到具体部门和个人，从严做好游客招徕、组织、接待等环节的疫情防控工作。要提醒游客增强安全意识，积极配合旅行社做好各项防控措施。

　　（二）坚持有序恢复。各省（区、市）文化和旅游行政部门在做好疫情防控工作的前提下，经当地省（区、市）党委、政府同意后，可恢复旅行社及在线旅游企业经营跨省（区、市）团队旅游及“机票+酒店”业务。中、高风险地区不得开展团队旅游及“机票+酒店”业务。出入境旅游业务暂不恢复。

　　（三）坚持高质量发展。各地文化和旅游行政部门要创新发展理念，支持旅行社综合运用科技创新成果，推动线上线下融合,扩大复工复业，加快转型升级，着力提升服务质量。行业组织要倡导诚信经营，强化行业自律，加强自我监督。旅行社要落实质量主体责任，严格执行产品质量标准和服务规范，不断增强游客满意度。

　　二、行前管理

　　（四）加强风险研判。旅行社要对旅游产品进行安全评估，选择具有相应资质且符合当地疫情防控要求的供应商、合作商，明确各方权责。要加强沟通联系，及时了解和掌握旅游目的地和客源地卫生健康部门疫情防控情况，做好线路设计、产品对接和预订等工作。要强化数据分析，提高产品防疫标准。组团社和地接社应明确双方疫情防控相关责任，加强协作配合，实现信息共享，确保团队旅游平稳、有序、安全。

　　（五）控制组团规模。旅行社要严格落实各地防控要求，根据自身运营能力和供应商、合作商接待能力，提前发布组团人数等产品防疫要求，从严控制团队人数，提倡小规模旅游团队。要合理安排团队旅游线路、规模和出游时间，分时段、分批次、分区域开展旅游活动，避免游客聚集。

　　（六）配备防护用品。旅行社应当配备数量充足且符合一次性使用医用口罩标准或相当防护级别的口罩、体温检测设备、洗手液、一次性手套、消毒用品等防护用品，为司机、导游和游客提供必要的防护保障。要正确储存和使用消毒物品，远离火源和电源，不得混用、混放,定期检查并及时补充更换。要督促供应商、合作商对旅游包车、酒店客房、餐厅等接待设施和场所进行全面消毒清洁。

　　（七）加强宣传引导。旅行社要严格落实团队旅游各项制度和规范，依法签订旅游合同，明确各方权责。要主动宣传疫情防控知识，及时发布游客出游防控注意事项,提醒游客落实“戴口罩、勤洗手、保距离”要求，增强游客自我防控意识。

　　（八）加强行前排查。旅行社要做好游客信息采集、健康档案、检测登记，要求游客报名时出示健康码并在出行前再次核验。对没有通过健康码检核的游客要做好解释说明和劝阻工作。要严格落实体温检测制度，体温异常的游客不允许参加行程，劝导其就医检查并做好登记。

　　三、行程管理

　　（九）落实防控措施。旅行社要严格落实各地在交通、住宿、餐饮、游览、购物等方面的疫情防控要求，督促供应商、合作商落实通风、消毒等措施。要加强对游客的体温检测，游客乘坐汽车等交通工具应全程佩戴口罩，严格执行景区和文化娱乐场所“限量、预约、错峰”等措施，主动配合接待单位做好疫情防控相关工作。

　　（十）加强服务规范。旅行社要进一步落实《旅行社服务通则》《导游服务规范》等行业标准，加强对游客的安全提示和行程管理。导游要做好游客在乘车、入住、购票、游览、就餐等环节的防控提醒，引导游客科学佩戴口罩，保持安全距离。提醒游客配合开展健康检疫，做好个人防护。行程结束后，旅行社要做好旅游团队档案整理并妥善保管。

　　（十一）倡导文明旅游。旅行社应当结合疫情防控工作，加强文明旅游宣传，推广“分餐制”“公筷制”等健康旅游新方式。要加强对游客的宣传引导，倡导讲究卫生、拒绝野味、理性消费，提醒游客规范处理垃圾，树立文明、健康、绿色旅游新风尚。

　　四、企业内部管理

　　（十二）加强办公及经营场所管理。旅行社要落实防控责任，制定企业内部疫情防控方案和应急预案，做好办公场所和旅行社服务网点卫生清洁、消杀和通风等工作。要减少开会频次和会议时长，尽可能使用网络会议工具。办公场所和旅行社服务网点应当将日常值守、清洁消毒、检测登记、垃圾清理、场地巡查、安全管理等各个防疫环节的责任落实到人，并根据当地疫情防控要求，及时动态调整。

　　（十三）做好员工监测。旅行社应当按照当地要求做好员工健康管理，建立《员工健康记录表》，每日进行体温检测，及时掌握员工健康状态、出行轨迹等情况。发现员工出现发热、咳嗽、乏力、鼻塞、流涕、咽痛、腹泻等相关症状，及时安排到就近定点医疗机构就诊，并跟踪相关情况。要在导游上岗前进行健康码检核，要求导游科学佩戴口罩。

　　（十四）加强教育培训。旅行社应当开展常态化疫情防控措施和应急处置等方面的专项培训，督促员工掌握疫情防控、个人防护、卫生健康及应急处置等方面的知识，提高员工疫情防控和应急处置能力。要压实导游责任，细化岗位职责，做好全陪、地陪等各项服务工作。

　　五、应急处置

　　（十五）建立协同机制。旅行社应当预先掌握旅游目的地和客源地卫生健康部门、定点医疗机构等联系方式，并确保导游等服务人员知晓。要加强与合作商、供应商的协调联动，畅通疫情上报通道，发现疑似疫情应及时向当地卫生健康部门、文化和旅游行政部门报告。

　　（十六）做好应急处置。旅游团队如发现疑似症状人员，旅行社要立即停止该团旅游活动并第一时间报告，配合相关部门做好疫情排查和防控措施。旅游团队中一旦出现确诊病例，旅行社要立即落实应急处置预案，配合相关部门和单位做好患者隔离、密切接触者追踪等工作，妥善处理善后事宜。

　　六、保障措施

　　（十七）加强组织领导。各地文化和旅游行政部门要严格落实属地管理责任，加强与当地卫生健康部门之间的联动，强化旅游目的地和客源地主管部门之间的协作，提升防控和应急处置能力，确保有序恢复经营工作平稳有序。

　　（十八）加强监督检查。各地文化和旅游行政部门要督促旅行社按照“一团一报”制度，在全国旅游监管服务平台填报旅游团队信息，上传电子合同。要加强对旅行社的监督检查，对疫情防控措施落实不力的要及时纠正，依法依规查处违法经营行为，维护市场秩序。

1. 加强日常调度。各地文化和旅游行政部门要按照防控方案和应急预案，明确疫情防控、安全突发事件应急措施和处置流程，开展应急演练、隐患排查、风险评估等工作，及时发现苗头性问题并有效处置。发生异常情况要及时上报并暂停有关经营活动。
2. 卫健委：发布《关于疫情常态化防控下规范医疗机构诊疗流程的通知》 联防联控机制医疗发〔2020〕272号

各省、自治区、直辖市及新疆生产建设兵团应对新冠肺炎疫情联防联控机制（领导小组、指挥部）：

为进一步落实常态化疫情防控工作有关要求，规范医疗机构诊疗流程，保障群众医疗服务需求，现就有关要求通知如下：

一、落实门急诊预检分诊制度

医疗机构要落实预检分诊制度，在门诊、急诊入口对所有人员进行体温检测；在预检分诊点由有经验的医务人员询问症状体征和流行病学史；门诊出诊医师要加强对患者有关新冠肺炎症状和流行病学史的问诊。对于预检分诊中发现的不能排除新冠肺炎的患者，要安排专人按照指定路线引导至发热门诊就诊；对于经预检分诊排除新冠肺炎，需要门诊检查和治疗的患者，不再进行针对新冠肺炎的核酸检测和CT检查。

二、加强急危重症患者救治

医疗机构要建立急危重症患者救治的绿色通道，对不能排除新冠肺炎的患者，在积极抢救的同时进行核酸检测。不得以疫情防控为由延误治疗或推诿急危重症患者。要在急诊抢救室、手术室、病房设置缓冲区，用于暂未取得核酸检测结果等情况下，急危重症患者的紧急救治，同时严格按照院感防控要求做好人员防护。医疗机构对急危重症患者的核酸检测要加急出具检测结果，提供检测结果的时间不得超过4—6小时，有条件的可采用快速检测技术，最大程度缩短急危重症患者检测时间。对于推诿患者的医疗机构，要严厉追究医疗机构主要负责人责任。

三、做好定期复诊和长期治疗患者的诊疗管理

各地要做好血液透析、肿瘤放化疗、孕妇产检等需要定期复诊和长期治疗患者的诊疗管理。对于来自低风险地区的上述患者，首次核酸检测阴性后，再次到医疗机构复诊和治疗的，经过预检分诊和医师问诊排除新冠肺炎可能的，不需反复进行核酸检测和CT检查。在中高风险地区以及对于来自中高风险地区的上述患者，地方卫生健康部门要确定定点医疗机构并公布，引导患者就医。相关医疗机构要做好患者诊疗信息对接，保证医疗安全。定点医疗机构要设置专门区域，提前与患者预约时间，明确专人进行对接安排，实现专门时段专门区域诊疗。医务人员按照院感防控要求做好个人防护，诊疗结束后，对相关区域和设备进行终末消毒。上述患者经预检分诊和医师问诊排除新冠肺炎可能的，不需反复进行核酸检测和CT检查。

四、科学做好个人防护

医疗机构要严格执行标准预防措施。进入医疗机构的人员均要求正确佩戴口罩。医疗机构的全体工作人员按照标准预防要求，佩戴外科口罩、穿工作服/白大衣、正确实施手卫生。加强诊疗环境的通风，做好诊疗环境、医疗器械、生活区域及设施等清洁消毒。医务人员对患者实施近距离操作、存在感染风险时，佩戴医用防护口罩，必要时佩戴护目镜或防护面屏等。

五、增强医务人员疫情防控意识和能力

医疗机构要开展疫情防控知识培训，确保所有接触患者的医务人员熟练掌握新冠病毒感染的防控知识与技能，具备排查新冠肺炎的意识和能力。通过体温检测、询问相关流行病学史和症状体征等，对就诊患者进行初筛。发现高度怀疑的患者时，依法采取隔离或者控制传播措施，并对其陪同人员采取必要的预防措施。

国务院应对新冠肺炎疫情

联防联控机制医疗救治组

（代章）

2020年7月10日

1. 卫健委：新冠肺炎疫情防控常态化下进一步提高院前医疗急救应对能力 国卫办医函〔2020〕557号

各省、自治区、直辖市及新疆生产建设兵团卫生健康委：

　　当前新冠肺炎疫情已进入常态化防控阶段，为深入贯彻落实党中央、国务院决策部署，做好应对新发突发传染病医疗服务保障，确保院前医疗急救转运安全高效，医疗机构接收迅速顺利，有效提升院前医疗急救服务能力，现就有关工作要求通知如下：

　　一、高度重视，加强院前医疗急救能力建设

　　各地卫生健康行政部门要高度重视院前医疗急救工作，强化政策协调衔接，统筹推进院前医疗急救事业健康发展。结合城乡功能布局、人口规模、服务需求，科学规划院前医疗急救网络布局。整合资源加大投入，提高救护车配置水平，特别是提高负压监护型救护车比例。加强院前医疗急救人才培养，提高院前医疗急救质量与效率。加强质量控制，确保院前医疗急救服务质量和安全。

　　二、加强管理，确保转运工作高效安全

　　各地卫生健康行政部门要根据《新型冠状病毒肺炎诊疗方案（试行第七版）》《新型冠状病毒肺炎防控方案（第六版）》《新型冠状病毒感染的肺炎病例转运工作方案（试行）》等文件要求，结合新冠肺炎疫情防控整体安排和实际情况，制订辖区内新冠肺炎疫情防控期间院前医疗急救工作方案，针对工作任务、转运流程、信息上报、个人防护要求、车辆装备洗消、医疗废物管理等方面提出明确要求，并监督急救中心和网络医院切实落实，在及时高效完成转运任务的同时，最大程度降低转运过程中的传播风险和医患交叉感染风险。

　　三、统筹安排，专车执行高风险任务

　　各地卫生健康行政部门统筹负责辖区内新型冠状病毒肺炎病例的转运指挥调度工作，指导辖区急救中心根据实际情况，划拨一定数量的负压救护车辆和专业人员，成立新冠肺炎疫情转运车组，采取平战结合管理，“战时”全力承担新冠肺炎疑似病例、确诊病例、无症状感染者以及发热相关病例的转运任务，平时承担日常院前医疗急救任务。在急救中心和定点收治医疗机构，设置专门区域停放新冠肺炎疫情转运救护车车辆，建立标准化洗消中心，严格按照《医院感染管理办法》《医疗机构消毒技术规范》对转运车辆、医疗设备等进行终末消毒。

　　四、加强信息化建设，提高调度水平

　　国家卫生健康委建立院前急救工作信息上报机制，依托国家卫生健康委医疗管理服务指导中心建立全国院前急救工作信息管理平台，加强急救相关信息管理，健全急救系统监测预警机制，提高智能化预警多点触发能力。各地要加强急救中心信息化建设，推动与通信、公安、交通、应急管理等部门及消防救援机构的急救调度信息共享与联动，提高调度效率，探索居民健康档案与调度平台有效对接，提高调度水平，指导辖区急救中心制定相关调度原则和具体要求，有效提高指挥调度和信息分析处理能力。

　　各急救中心要加强调度人员培训，在调度环节加强问询，准确掌握有关信息，根据实际情况分类调派。受理有发热、呼吸系统症状患者急救电话时，必须主动询问两周内旅行史、居住史、密切接触史、疫区暴露史等相关情况，自述有相关流行病史且患者病情稳定的，派遣新冠肺炎疫情转运车组执行任务，有相关流行病史且病情危急或不明的，或无相关流行病史的，就近派遣日常急救车组执行任务，并加强个人防护；受理无接诊高度疑似发热病人条件的医疗机构请求转运相关病人的急救电话时，派遣新冠肺炎疫情转运车组执行任务，迅速将患者转运至辖区指定定点医院。

　　五、院前院内有效衔接，提高救治效率

　　各地卫生健康行政部门要按照有关要求加强医疗机构急诊、发热门诊管理，确保医疗机构与院前急救网络有效衔接，要求医疗机构间转接病人前必须做好信息对接，缩短院前院内交接时间。对于新冠肺炎疑似病例、确诊病例、无症状感染者及发热相关病例等，提前预留接诊区域妥善安置，做好医护人员防护，入院立即进行相关检查尽快明确诊断，完善院内流程，整合呼吸、重症等相关科室，迅速开展救治，提高救治效率。

　　各地卫生健康行政部门要切实履行监督指导职责，加强对辖区内应对新冠肺炎疫情院前医疗急救服务实施情况的政策指导和督促检查，及时发现问题、解决问题，综合评价院前医疗急救工作开展情况，及时总结经验并定期通报工作进展。

国家卫生健康委办公厅

2020年7月9日

1. 农业农村部、财政部：发布2020年重点强农惠农政策

2020年，贯彻落实中央农村工作会议、中央1号文件、国务院政府工作报告，围绕实施乡村振兴战略，打赢脱贫攻坚战，如期实现全面小康目标，应对新冠肺炎疫情新形势，扎实做好“六稳”工作、落实“六保”任务，国家将继续加大支农投入，强化项目统筹整合，加快推进农业农村现代化。为便于广大农民和社会各界了解国家强农惠农政策，发挥政策引导的作用，现将2020年农业农村部、财政部实施的重点支农政策发布如下。

　　一、农业生产发展与流通

　　1. 耕地地力保护补贴。补贴对象原则上为拥有耕地承包权的种地农民。补贴资金通过“一卡（折）通”等形式直接兑现到户，严禁任何方式统筹集中使用。各省（自治区、直辖市）继续按照《财政部、农业部关于全面推开农业“三项补贴”改革工作的通知》（财农〔2016〕26号）要求，并结合本地实际具体确定补贴对象、补贴方式、补贴标准。鼓励各地逐步将补贴发放与土地确权面积挂钩。鼓励各地创新方式方法，以绿色生态为导向，探索将补贴发放与耕地保护责任落实挂钩的机制，引导农民自觉提升耕地地力。

　　2. 农机购置补贴。各省（自治区、直辖市）在中央财政农机购置补贴机具种类范围内选取确定本省补贴机具品目，优先保证粮食等主要农产品生产所需机具和支持农业绿色发展机具的补贴需要，增加畜禽粪污资源化利用机具品目。将支持生猪等畜产品生产的自动饲喂等机具装备全部纳入各省补贴范围。将果园轨道运输机等助力丘陵山区等贫困地区产业发展所需机具纳入全国补贴范围，由各省从中选取品目进行补贴。补贴额依据同档产品上年市场销售均价测算，原则上测算比例不超过30%。 实施农机报废更新补贴政策，对报废老旧农机给予适当补助。

　　3. 优势特色产业集群。坚持市场主导、政府扶持的原则，按照全产业链开发、全价值链提升的思路，支持选择省域内基础好、规模大、有特色、比较优势显著的主导产业，打造一批结构合理、链条完整的优势特色产业集群。着力解决好产业发展中的瓶颈制约和关键环节，推动产业形态由“小特产”升级为“大产业”，空间布局由“平面分布”转型为“集群发展”，主体关系由“同质竞争”转变为“合作共赢”。2020年首批启动支持建设50个产业集群，原则上连续支持，中央财政对批准建设的产业集群进行适当补助，支持各省围绕基地建设、机收机种、仓储保鲜、产地初加工、精深加工、现代流通、品牌培育等全产业链建设，鼓励创新资金使用方式，采取先建后补、以奖代补、贷款贴息、政府购买服务等方式对相关主体给予支持。

　　4. 国家现代农业产业园。立足优势特色产业，突出一二三产业融合和联农增收机制创新两大任务，2020年继续创建31个国家现代农业产业园，择优认定一批国家现代农业产业园，着力改善产业园基础设施条件，提升公共服务能力。创建工作由各省（自治区、直辖市）负责，中央财政对符合创建条件的安排部分补助资金，通过农业农村部、财政部中期评估和认定后，再视情况安排部分奖补资金。

　　5. 农业产业强镇。继续以乡镇为平台载体，聚焦主导产业，发展壮大乡村产业，加快培育一批产业生产经营市场主体，创新农民利益联结共享机制，重点支持全产业链开发中的仓储保鲜、加工营销等关键领域、薄弱环节，推动主导产业转型升级、由大变强，建设一批主导产业突出、产村融合发展、宜业宜居的农业产业强镇。中央财政通过安排奖补资金予以支持，引导企业与农户建立订单收购、保底分红、二次返利等紧密型利益联结机制，促进农民分享全产业链增值收益。2020年共支持259个镇（乡）开展农业产业强镇建设。

　　6. 农产品地理标志保护工程。支持区域特色品种繁育基地和核心生产基地建设，改善生产及配套仓储保鲜设施设备条件。健全生产技术标准体系，强化特色品质保持技术集成，推动全产业链标准化生产。挖掘传统农耕文化，讲好地标历史故事，强化产品推介，叫响特色品牌。支持利用信息化技术，实施产品可追溯管理，推动地理标志农产品身份化、标识化和数字化。

　　7. 推进信息进村入户。支持开展益农信息社整省推进建设。严格按照《农业部关于全面推进信息进村入户工程的实施意见》（农市发〔2016〕7号）要求组织实施，依据“六有”标准建设益农信息社，提升便民服务、电子商务、培训体验服务水平，推进“互联网+”农产品出村进城，将益农信息社打造成为农服务的一站式窗口。

　　8. 奶业振兴和畜牧业转型升级。支持建设优质苜蓿生产基地，降低奶牛养殖饲喂成本，提高生鲜乳质量水平。以北方农牧交错带为重点，支持牛羊养殖场（户）和饲草专业化服务组织收储青贮玉米、苜蓿、燕麦草等优质饲草，通过以养带种的方式加快推动种植结构调整和现代饲草产业发展。在内蒙古、四川等8个主要草原牧区省份，对项目区内使用良种精液开展人工授精的肉牛养殖场（小区、户），以及存栏能繁母羊、牦牛能繁母牛养殖户进行补助。鼓励和支持推广应用优良种猪和精液，加快生猪品种改良。在黑龙江、江苏等10个蜂业主产省，实施蜂业质量提升行动，支持建设高效优质蜂产业发展示范区。

　　9. 重点作物绿色高质高效行动。建设一批绿色高质高效生产示范片，集成组装耕种管收全过程绿色高质高效新技术，示范推广优质高产、多抗耐逆新品种，集中打造优良食味稻米、优质专用小麦、高油高蛋白大豆、双低双高油菜、高品质棉花、高产高糖甘蔗、优质果菜茶、道地中药材等生产基地，带动大面积区域性均衡发展，促进种植业稳产高产、节本增效和提质增效。南方早稻主产省要集中支持早稻生产，促进双季稻恢复。

　　10. 推广旱作节水农业技术。以巩固提升旱区农业综合生产能力和资源利用效率为目标，实施区域向黄河流域倾斜，示范水肥一体化、集雨补灌、垄作（膜）沟灌、测墒节灌等高效节水技术，提高水肥利用效率；示范蓄水保墒、抗旱抗逆等现代旱作雨养技术，提高天然降水利用效率；立足干旱半干旱地区水资源条件，开展旱作节水农业新技术、新产品、新材料试验示范，创新集成旱作节水农业技术模式，促进旱区农业绿色高质量发展。

　　11. 有机肥替代化肥行动。以减少化肥投入、增加有机肥投入为目标，支持重点县实施果菜茶有机肥替代化肥，实施区域重点向长江经济带和黄河流域倾斜，试点作物向其他节肥潜力大的园艺作物和大田作物拓展。集成推广堆肥还田、商品有机肥施用、沼渣沼液还田等技术模式，配套完善设施设备，促进果菜茶提质增效和资源循环利用。工作推进要与畜禽粪污资源化利用相结合，采取政府购买服务、技术补贴、物化补贴等方式，支持农民和新型农业经营主体积造施用有机肥，培育一批生产性服务组织，加快有机肥应用，促进种养结合。

　　12. 农业生产社会化服务。围绕粮棉油糖等重要农产品和当地特色主导产业，集中连片开展社会化服务，服务方式进一步聚焦农业生产托管，服务对象进一步聚焦服务小农户，服务环节进一步聚焦农业生产的关键薄弱环节和农民急需的生产环节。采取先服务后补助的方式，支持专业服务公司、供销合作社、农村集体经济组织、服务型农民合作社和家庭农场等服务主体，并优先支持安装机械作业监测传感器的服务主体，提升农业生产社会化服务的市场化、专业化、规模化、信息化水平。江西、湖南、广东、广西等早稻主产省要重点推广早稻生产托管等社会化服务。

　　13. 农机深松整地。支持适宜地区开展农机深松整地作业，全国作业面积达到1.4亿亩以上，作业深度一般要求达到或超过25厘米，打破犁底层。

　　14. 产粮大县奖励。对符合规定的常规产粮大县、超级产粮大县、产油大县、商品粮大省、制种大县、“优质粮食工程”实施省份给予奖励。常规产粮大县奖励资金作为财力补助，由县级人民政府统筹安排；其他奖励资金按照有关规定用于扶持粮油产业发展。

　　15. 生猪（牛羊）调出大县奖励。包括生猪调出大县奖励、牛羊调出大县奖励和省级统筹奖励资金。生猪调出大县奖励资金和牛羊调出大县奖励资金由县级人民政府统筹安排用于支持本县生猪（牛羊）生产流通和产业发展，省级统筹奖励资金由省级人民政府统筹安排用于支持本省（自治区、直辖市）生猪（牛羊）生产流通和产业发展。

　　16. 玉米、大豆生产者补贴和稻谷补贴。为巩固农业供给侧结构性改革成效，在玉米和大豆价格由市场形成的基础上，国家继续在东北三省和内蒙古自治区实施玉米和大豆生产者补贴政策。中央财政对有关省（区）玉米补贴不超过2014年基期播种面积，大豆补贴面积不超过2019年基期播种面积，2020-2022年保持不变。为支持深化稻谷收储制度和价格形成机制改革，国家在有关稻谷主产省份继续实施稻谷补贴政策。中央财政对稻谷补贴数量上限为基期（2016-2018年）稻谷年平均产量的85%。

　　二、农业绿色生产与农业资源保护利用

　　17. 草原生态保护补助奖励。在内蒙古、四川、云南、西藏、甘肃、宁夏、青海、新疆等8个省（自治区）和新疆生产建设兵团实施禁牧补助、草畜平衡奖励；在河北、山西、辽宁、吉林、黑龙江和黑龙江省农垦总局实施“一揽子”政策。

　　18. 长江流域重点水域禁捕补偿。中央财政采取一次性补助与过渡期补助相结合的方式，对长江流域重点水域禁捕工作给予支持，促进水生生物资源恢复和水域生态环境修复。其中，一次性补助由地方结合实际统筹用于收回渔民捕捞权和专用生产设备报废，直接发放到符合条件的退捕渔民。过渡期补助由各地统筹用于禁捕宣传动员、提前退捕奖励、加强执法管理、突发事件应急处置等与禁捕直接相关的工作。

　　19. 渔业发展与船舶报废拆解更新补助。按照海洋捕捞强度与资源再生能力平衡协调发展的要求，支持渔民减船转产和人工鱼礁建设，促进渔业生态环境修复。适应渔业发展现代化、专业化的新形势，在严控海洋捕捞渔船数和功率数“双控”指标、不增加捕捞强度的前提下，有计划升级改造选择性好、高效节能、安全环保的标准化捕捞渔船。同时，支持深水网箱推广、渔港航标等公共基础设施，改善渔业发展基础条件。

　　20. 渔业增殖放流。在流域性大江大湖、界江界河、资源退化严重海域等重点水域开展渔业增殖放流，促进恢复或增加渔业种群的数量，改善和优化水域的渔业种群结构，实现渔业可持续发展。

　　21. 农作物秸秆综合利用试点。在全国范围内整县推进，坚持农用优先、多元利用，培育一批产业化利用主体，打造一批全量利用样板县。激发秸秆还田、离田、加工利用等各环节市场主体活力，探索可推广、可持续的秸秆综合利用技术路线、模式和机制。

　　22. 畜禽粪污资源化处理。支持畜牧大县开展畜禽粪污资源化利用工作，探索在非畜牧养殖大县推广粪污资源化利用。按照政府支持、企业主体、市场化运作的原则，以就地就近用于农村能源和农用有机肥为主要利用方式，新（扩）建畜禽粪污收集、利用等处理设施，以及区域性粪污集中处理中心、大型沼气工程，实现规模养殖场全部实现粪污处理和资源化利用，形成农牧结合、种养循环发展的产业格局。

　　23. 地膜回收利用。在内蒙古、甘肃和新疆支持100个县整县推进废旧地膜回收利用，鼓励其他地区自主开展探索。支持建立健全废旧地膜回收加工体系，建立经营主体上交、专业化组织回收、加工企业回收、以旧换新等多种方式的回收利用机制，并探索“谁生产、谁回收”的地膜生产者责任延伸制度。

　　24. 耕地轮作休耕制度试点。2020年，中央财政继续支持轮作休耕试点。其中，轮作试点主要在东北冷凉区、北方农牧交错区、黄淮海地区、华南双季稻区和长江流域的大豆、花生、油菜产区实施；休耕试点主要在地下水超采区、西北生态严重退化地区实施。

　　三、农田建设

　　25. 高标准农田建设。2020年，按照“统一规划布局、统一建设标准、统一组织实施、统一验收考核、统一上图入库”五个统一的要求，在全国建设高标准农田8000万亩，并向粮食生产功能区、重要农产品生产保护区倾斜。在建设内容上，按照《高标准农田建设通则》，以土地平整、土壤改良、农田水利、机耕道路、农田输配电设备等为重点，加强农业基础设施建设，提高农业综合生产能力，落实好“藏粮于地、藏粮于技”战略。

　　26. 东北黑土地保护利用和保护性耕作。继续在辽宁、吉林、黑龙江和内蒙古四省（区）实施东北黑土地保护利用工程，建立集中连片示范区，集中展示一批黑土地保护利用模式和保护性耕作示范；支持开展控制黑土流失、增加土壤有机质含量、保水保肥、黑土养育、耕地质量监测评价、保护性耕作等技术措施和工程措施。2020年启动东北黑土地保护性耕作行动计划，支持在适宜区域推广应用秸秆覆盖免（少）耕播种等关键技术，有效减轻风蚀水蚀、增加土壤有机质、增强保墒抗旱能力、提高农业生态效益和经济效益，中央财政支持实施面积4000万亩。鼓励新型农业经营主体和社会化服务组织承担实施任务。

　　27. 耕地质量保护与提升。选择一批节肥潜力大的重点县开展化肥减量增效示范，引导企业和社会化服务组织开展科学施肥技术服务，支持农户和新型农业经营主体应用化肥减量增效新技术新产品，着力解决限制化肥使用过量、利用率不高的突出问题。选择部分县开展肥料包装废弃物回收处理工作试点。继续支持做好耕地质量等级调查评价与监测、取土化验、田间肥效试验、肥料配方制定发布、测土配方施肥数据成果开发应用等工作。开展退化耕地治理。在土壤酸化区域，集成示范施用石灰质物质和酸性土壤调理剂、种植绿肥还田、水肥调控、生物修复等治理模式；在土壤盐碱化区域，结合排灌工程措施，集成示范施用碱性土壤调理剂、耕作压盐、增施堆沤有机肥等治理模式，改善耕地土壤质量。

　　四、新型经营主体培育

　　28. 高素质农民培育。重点实施新型农业经营服务主体经营者、产业扶贫带头人、农村实用人才带头人、返乡入乡创新创业者、专业种养加能手等培养计划，加快培养有文化、懂技术、善经营、会管理的高素质农民。加大产业精准扶贫培训力度，深入开展“三区三州”等产业扶贫带头人培训行动。

　　29. 新型农业经营主体高质量发展。一是支持新型农业经营主体建设农产品仓储保鲜设施。聚焦鲜活农产品产地“最先一公里”问题，支持新型农业经营主体建设农产品产地仓储保鲜设施。采取“先建后补、以奖代补”的方式，重点在河北、山西等16个省份，聚焦重点县（市），并向“三区三州”深度贫困地区和未摘帽贫困县、新冠疫情防控重点地区和鲜活农产品主产区、特色农产品优势区倾斜；重点支持建设节能型通风贮藏设施、节能型机械冷库、节能型气调贮藏库；支持对象限定为县级以上示范家庭农场、农民合作社示范社（包括联合社），贫困地区可适当放宽条件，并支持多个家庭农场（农民合作社）联合建设，避免设施闲置浪费；补助采取“双限”，补贴比例上限不超过仓储保鲜设施造价的30％（湖北省和贫困地区放宽至50％），有条件的地方可安排地方财政资金适当叠加补贴，同时实行定额补贴并限定上限，单个主体补贴上限为100万元，具体定额补贴标准由地方制定。二是支持新型农业经营主体提升技术应用和生产经营能力。支持县级以上农民合作社示范社（联合社）和示范家庭农场改善生产条件，应用先进技术，提升规模化、绿色化、标准化、集约化生产能力，建设清选包装、烘干等产地初加工设施，提高产品质量水平和市场竞争力。鼓励各地为农民合作社和家庭农场提供财务管理、技术指导等服务。鼓励有条件的地方依托龙头企业，带动农民合作社和家庭农场，形成农业产业化联合体。对具有种畜禽生产经营许可证的种猪场（含地方猪保种场）及年出栏500头以上的规模猪场给予贷款贴息支持。优先重点支持发展奶牛家庭牧场和奶农合作社。

　　30. 农业信贷担保服务。重点服务家庭农场、农民合作社、农业社会化服务组织、小微农业企业等农业适度规模经营主体。服务范围限定为农业生产（包括农林牧渔生产和农田建设，下同）及与农业生产直接相关的产业融合项目（指县域范围内，向农业生产者提供农资、农技、农机，农产品收购、仓储保鲜、销售、初加工，以及农业新业态等服务的项目），突出对粮食、生猪等重要农产品生产的支持。中央财政对政策性农担业务实行担保费用补助和业务奖补，支持省级农担公司降低担保费用和应对代偿风险，确保政策性农担业务贷款主体实际负担的担保费率不超过0.8%（政策性扶贫项目不超过0.5%） 。

　　31. 基层农技推广体系改革与建设。通过政府购买服务等方式，支持市场化服务力量开展农技服务，完善公益性和经营性农技服务融合发展机制，构建多元互补、高效协同的农技推广体系。应用信息化手段大力推行在线指导服务和绩效考评，提高中国农技推广信息平台覆盖面和使用率。建设科技示范展示基地，培育科技示范主体，推广应用绿色增产、节本增效的主推技术，打造智慧农场、生态循环农场等科技示范样板。在内蒙古、吉林等8个省份开展重大技术协同推广试点。在贫困地区、生猪大县实施农技推广服务特聘计划。

　　五、农业防灾减灾

　　32. 农业生产救灾。中央财政对各地农业重大自然灾害及生物灾害的预防控制、应急救灾和灾后恢复生产工作给予适当补助。支持范围包括农业重大自然灾害预防及生物灾害防控所需的物资材料补助，恢复农业生产措施所需的物资材料补助，牧区抗灾保畜所需的储草棚（库）、牲畜暖棚和应急调运饲草料补助等。

　　33. 动物疫病防控。中央财政对动物疫病强制免疫、强制扑杀和养殖环节无害化处理工作给予补助。强制免疫补助经费主要用于开展口蹄疫、高致病性禽流感、小反刍兽疫、布病、包虫病等动物强制免疫疫苗（驱虫药物）采购、储存、注射（投喂）以及免疫效果监测评价、人员防护等相关防控工作，以及对实施和购买动物防疫服务等予以补助。国家在预防、控制和扑灭动物疫病过程中，对被强制扑杀动物的所有者给予补偿，补助经费由中央财政和地方财政共同承担。国家对养殖环节病死猪无害化处理予以支持，由各地根据有关要求，结合当地实际，完善无害化处理补助政策，切实做好养殖环节无害化处理工作。

　　34. 农业保险保费补贴。在地方财政自主开展、自愿承担一定补贴比例基础上，中央财政对水稻、小麦、玉米、棉花、马铃薯、油料作物、糖料作物、能繁母猪、奶牛、育肥猪、森林、青稞、牦牛、藏系羊和天然橡胶，以及水稻、小麦、玉米制种保险给予保费补贴支持。继续在13个粮食主产省面向适度规模经营农户开展农业大灾保险试点，保障水平覆盖“直接物化成本+地租”；继续在内蒙古、辽宁、安徽、山东、河南、湖北等6个省（自治区），面向规模经营农户和小农户开展三大粮食作物完全成本保险和收入保险试点，保障水平覆盖农业生产总成本或农业生产产值；在20个省份开展中央财政对地方优势特色农产品保险奖补试点。

　　六、乡村建设

　　35. 农村人居环境整治先进县激励。贯彻落实《农村人居环境整治三年行动方案》和《国务院办公厅关于对真抓实干成效明显地方进一步加大激励支持力度的通知》（国办发〔2018〕117号）精神，按照《农村人居环境整治激励措施实施办法》对各省开展农村人居环境整治工作进行评价，确定20个农村人居环境整治激励县（市、区、旗）名单。2020年，中央财政对农村人居环境整治成效明显的县予以激励支持，主要用于农村厕所革命整村推进、村容村貌整治提升等农村人居环境整治相关建设。

　　36. 农村厕所革命整村推进。中央财政安排专项奖补资金，支持和引导各地以行政村为单元，整体规划设计，整体组织发动，同步实施户厕改造、公共设施配套建设，并建立健全后期管护机制。卫生厕所普及率达到85%左右。奖补资金主要支持粪污收集、储存、运输、资源化利用及后期管护能力提升等方面的设施设备建设。奖补标准、方式等由各地结合实际确定。

2020.7.13

1. 体育总局：科学有序恢复体育赛事和活动推动体育行业复工复产工作 体办字﹝2020﹞102号

各省、自治区、直辖市、计划单列市、新疆生产建设兵团体育局，中央军委训练管理部军事体育训练中心，各行业体协，各厅、司、局，各直属单位，中国足球协会、中国篮球协会、中国田径协会，各改革试点项目协会：

　　《科学有序恢复体育赛事和活动推动体育行业复工复产工作方案》已经国务院应对新型冠状病毒感染肺炎疫情联防联控机制审批同意，现印发你们。

　　请结合实际，认真贯彻落实，在疫情防控常态化条件下，科学有序推动体育行业复工复产，加快恢复经济社会正常秩序。

体育总局

2020年7月6日

科学有序恢复体育赛事和活动推动体育行业复工复产工作方案

科学有序恢复体育赛事和活动，是在疫情防控常态化情况下发展体育事业、体育产业的必然要求。按照“外防输入、内防反弹”的总体防控策略，根据党中央、国务院关于复工复产的有关要求，为科学、安全、有序、积极恢复体育赛事和活动，制定以下方案。

　　一、基本原则

　　（一）坚持防控为先。把人民群众生命安全和身体健康放在首位，全面落实疫情防控各项要求。

　　（二）坚持属地管理。结合各地疫情风险等级和防控工作要求，积极稳妥有序恢复体育赛事和活动。明确赛事和活动承办地主体责任，实施属地管理。

　　（三）坚持科学施策。充分考虑项目特点、规模、场地和人群密度等因素，科学制定工作方案和疫情防控方案。

　　（四）坚持循序渐进。先开放户外场所，后开放室内场所；先开放无直接身体接触类项目，后有序开放有接触和身体对抗类项目。

　　（五）坚持动态调整。根据各地疫情风险等级变化和防控要求，制定体育赛事和活动疫情防控指南，动态调整完善赛事规模、观众数量等方面政策。建立熔断机制，确保遇到突发疫情风险时能够立刻中止相关赛事和活动，做好人员筛查隔离，有效防范疫情扩散，做到收放有序。

　　二、分类推进，有序恢复体育赛事和活动

　　疫情风险处于中、高等级地区原则上暂不举办体育赛事和群体性全民健身活动。低风险地区在科学评估疫情风险，坚决贯彻防控要求的前提下，周密制定具体工作方案，经属地政府和疫情防控领导机制审核评估后组织实施，积极有序恢复体育赛事和活动。

　　（一）采取预约、限流等方式，有序、全面开放各类体育设施，开展群众健身活动和小型分散的商业性、群众性体育赛事。

　　（二）采取改变赛事方式、空场比赛等措施，按照“一赛事一方案”要求，重启中国足球协会超级联赛等职业体育赛事。不邀请境外裁判员参加；对尚处境外的运动员、教练员不要求近期返回，如返回须制定专门的防控方案，严格执行入境防控措施。

　　比赛地点的选择应综合考虑当地疫情风险、防控压力、接待能力、组织水平等因素，并征得属地政府同意，及时总结经验，形成具体化、可操作、可复制的工作方案，有序推广。

　　（三）采取线上、线下等多种形式，在封闭训练基地组织开展专业运动队赛事。密切关注国际奥委会和各单项国际体育组织有关安排，积极、稳妥参与各项目奥运积分赛、资格赛等赛事，全面做好东京奥运会备战参赛工作。

　　（四）科学应对西方国家“带疫解封”产生的影响，切实做好北京冬奥会测试赛及相关筹备工作。审慎调整第十四届全国冬运会、亚洲沙滩运动会等大型综合性运动会举办方案。除北京冬奥会测试赛等重要赛事外，今年内原则上不举办其他国际性体育赛事和活动。

　　三、抓紧抓细抓实疫情防控工作

　　（一）加强联防联控。地方各级人民政府要切实负起领导责任，协调体育、卫生健康、公安等部门，建立体育赛事和活动相关风险研判、方案审核、应急响应等工作机制，完善相关场馆、酒店、餐厅、交通工具和人员的疫情防控措施，赛前要组织专门培训和演练。

　　（二）加强人员管控。对所有参与人员进行风险评估和实名制管理，实行“健康码”绿码准入制度，做到核酸检测全覆盖，加强每日健康状态监测，并根据属地相关要求进行必要的申报或者登记。

　　（三）加强防控力量。根据赛事和活动规模、项目特点、人群密度，配备足够数量的医护人员和防护物资，并对相关人员进行个人防护、消毒程序、测温登记、异常情况处置等培训，确保各项防控措施落到实处。

　　（四）原则上实行空场比赛。在落实防疫措施前提下，逐步探索持有48小时内核酸检测结果阴性证明并严格自我隔离的观众现场观赛，间隔就坐，总数不超过观众席容量的50%，视疫情风险等级和防控工作情况，动态调整比例。

　　（五）优化赛事组织。简化开闭幕式、颁奖仪式等赛事环节，减少人群聚集，合理规划人员流线，采取观众区与运动队隔离措施，避免人员交叉。

　　（六）强化环境卫生。突出关键环节和重点领域，落实场地、器材防控措施，对赛场环境和空调系统进行彻底清洁，保证空气流通，保障洗手设施正常运行，规范垃圾收集处理，公共区域设置口罩回收箱，定期开展预防性消毒。

　　（七）加强应急处置。赛事和活动组织者要制定应急处置预案，与卫生健康、疾控等部门保持密切联系，一旦发现有发热等症状的人员要立即启动应急预案。

　　四、组织保障

　　（一）压实各方主体责任。各级体育主管部门要按照“谁主管、谁负责”原则，落实管理责任。根据属地疫情防控工作要求，制定细化本地区实施方案。强化事中监管，开展必要的督查检查，严控数量和频次，力戒形式主义、官僚主义，坚决杜绝防控措施"一刀切"、防控要求“层层加码”。体育赛事和活动的主办方、承办方及场地设施经营单位要按照各自职责，认真落实疫情防控工作主体责任，科学制定防控工作方案和应急预案，做到“一赛一方案”，确保办赛安全。体育赛事和活动的参加者要服从组织者的管理，严格执行疫情防控措施，加强个人防护，主动配合做好身体健康检测、信息登记等工作。

　　（二）加大政策扶持力度。地方各级人民政府要积极研究出台产业扶持政策，适当减免税费、降低安保费用，通过政府购买服务等形式帮助相关企业克服疫情带来的不利影响。

　　（三）加强宣传教育科学引导。统筹做好科学有序恢复体育赛事和活动的宣传报道，做好政策解读，避免误读、误解。利用场馆大屏、广播、新媒体等媒介，广泛宣传普及疫情防控知识，提高办赛、参赛和观赛等各类人员的防护意识和能力，引导公众安全依规参与体育活动；鼓励群众积极参加体育锻炼，科学健身，养成健康生活习惯；积极推广各地好经验、好做法，营造良好舆论氛围。

1. 商务部等8部门：加快家政服务业信用体系建设有序推动家政服务企业复工营业 商办服贸函﹝2020﹞237号

为深入贯彻习近平总书记关于统筹推进疫情防控和经济社会发展工作的系列重要讲话精神，落实好《国务院办公厅关于促进家政服务业提质扩容的意见》（国办发〔2019〕30号，以下简称《意见》）的要求，加快推进家政服务业信用体系建设，有序推动家政服务企业复工营业，保居民就业、保基本民生、保市场主体，现就有关事项通知如下：

　　一、强化应用和宣传推广

　　（一）强化信用平台应用。商务部拟正式启用“家政服务信用信息平台”（以下简称信用平台）。各地商务主管部门要强化信用信息应用，主动公布登录信用平台的家政服务企业名单，引导消费者积极使用信用平台“家政信用查”手机App或国务院客户端小程序家政服务员信用信息查询服务，优先选择信用记录良好的家政服务企业和家政服务员。鼓励家政服务员将相关培训证书数据上传至职业技能等级证书信息管理服务平台及家政服务信用信息平台，供消费者查询。

　　（二）加大宣传力度。各地宣传部、文明办要加强家政服务业信用体系建设宣传工作，通过“诚信建设万里行”等多种渠道宣传信用平台。各地商务主管部门要在《商务部办公厅关于做好“家政服务信用信息平台”启用相关工作的通知》（商办服贸函〔2019〕293号）要求基础上，进一步加强信用平台宣传推广工作，推进宣传深入社区、深入乡村、深入企业，实现宣传海报家政服务企业门店全覆盖。

　　二、支持家政服务员返岗复工

　　（一）支持家政服务员进社区。在做好疫情防控基础上，低风险地区要支持家政服务员在其服务对象向社区组织报备后，凭相关健康证明（国家政务服务平台防疫健康信息码“未见异常”，各地防疫健康信息码、健康通行码绿码，隔离医学观察期满证明等）进入社区，并自觉接受出入登记和体温检测，不得再设置超出社区防控要求的措施。高风险、中风险地区按本地区要求做好家政服务员健康管理，支持家政服务企业对集中居住的家政服务员做好生活保障。

　　（二）支持家政服务员健康上岗。各地商务主管部门要指导家政服务企业加强家政服务员健康管理，做好个人安全防护，引导家政服务员使用“家政信用查”手机App或国务院客户端小程序家政服务员信用信息查询服务，积极向消费者查询展示防疫健康信息和个人信用记录。

　　三、提高项目建设绩效

　　（一）加快执行进度。各地要严格按照《服务业发展资金管理办法》（财建〔2019〕50号）、《财政部关于下达2019年服务业发展资金（第二批）预算的通知》（财建〔2019〕306号）和《商务部办公厅 财政部办公厅关于支持家政服务业信用体系建设有关工作的通知》（商办服贸函〔2019〕254号）等文件要求，安全、规范、有效使用服务业发展资金，加快家政服务业信用体系建设进度。对体系建设工作开展不力，导致财政资金闲置浪费的，将收回支持资金并统筹用于其他亟需支持的领域。

　　（二）加强绩效管理。各级财政和商务主管部门要开展全过程绩效管理，密切跟踪预算执行进度、项目建设实施、项目产出情况，发现问题及时整改。请于2020年7月底前，向财政部（经济建设司）、商务部（服贸司）报送项目实施及绩效情况总结。绩效情况将作为以后年度服务业发展资金安排参考因素。

　　各地相关部门要加强横向协作、纵向联动，力戒形式主义、官僚主义，强化主动作为、责任担当，加快推动信用平台建设、宣传等各项工作，依法依规保障家政服务员信息安全，促进家政服务业提质扩容，推动家政服务消费加快回补。

商务部办公厅

中央宣传部办公厅

中央文明办秘书局

发展改革委办公厅

教育部办公厅

民政部办公厅

财政部办公厅

卫生健康委办公厅

2020年6月19日

1. 商务部等3部门：印发《关于展览活动新冠肺炎疫情常态化防控工作的指导意见》

各省、自治区、直辖市及计划单列市人民政府，新疆生产建设兵团：

为认真贯彻党中央关于抓紧抓实抓细常态化疫情防控工作的决策部署，统筹推进疫情防控和经济社会发展，全面落实“外防输入、内防反弹”的总体防控策略，规范展览活动新冠肺炎疫情常态化防控工作，安全有序推进展览活动复展复业，经国务院应对新型冠状病毒感染肺炎疫情联防联控机制同意，现提出以下意见。

一、指导思想

依据《中华人民共和国传染病防治法》、《突发公共卫生事件应急条例》等法律法规，贯彻“安全第一、预防为主”的方针，依法科学开展展览活动新冠肺炎疫情常态化防控工作，充分发挥展览业在扩大开放、增加就业、稳住外贸外资基本盘、拉动消费等方面的重要作用，扎实做好“六稳”工作，全面落实“六保”任务，努力克服新冠肺炎疫情带来的不利影响，增强展览业防控和应变能力，确保防控常态化条件下各项展览活动科学稳妥、安全有序开展，推动经济社会持续向好发展。

二、基本原则

（一）属地管理原则。各地人民政府要对属地举办的展览活动疫情防控工作负责，加强组织领导，坚持依法防控、科学防控、联防联控。展览活动疫情防控按照分区分级标准，根据举办地疫情应急响应级别相应的疫情防控标准和要求组织实施。

（二）群防群控原则。坚持底线思维，牢固树立群防群控意识，所有展览活动参与者（包含展览场所单位、展览举办单位、参展商、服务商、观众、现场工作人员等）都必须始终绷紧疫情防控这根弦，自觉执行防控要求，严格履行防控职责，确保全员参与、全面覆盖，全方位、全过程、全领域防控，做到不留死角。

（三）科学专业原则。严格执行国家卫生健康委和疾控部门的专业防控要求，在卫健、疾控部门的指导下，科学防控、专业防控；制订防控方案和应急预案，确保防控到位，处置及时，信息收集要完整和可追溯。

（四）分级分类原则。根据疫情响应级别，结合展览活动特点和各地实际情况，针对来自市内、省（区）内、跨省（区）、境外不同区域和低、中、高不同等级风险地区的展览活动参与人员，实行分级分类管理。审慎举办大型涉外展览活动，要按照国家和各地防控疫情境外输入的要求做好防控并落实管理责任。鼓励境外参展商和观众通过在线方式参展参会，或委托其在华分支机构、代表处或合作伙伴等参加线下展会。

（五）动态调整原则。根据疫情形势发展变化和本地区疫情响应级别调整变化，按照国务院联防联控机制和本地区疫情防控总体要求，各地要因地制宜、因时制宜，合理调整展览活动防控措施。

（六）风险可控原则。中、高风险地区原则上暂不举办展览活动。在严格落实各项防控措施的前提下，低风险地区可举办必要的展览活动。举办展览活动要做好疫情风险评估，有效管控并防范重大风险隐患，制定应急处置预案并组织开展演练，确保展览活动风险总体可控。

三、严格落实展览活动举办地防控责任

（一）举办地人民政府要落实属地责任。根据分区分级精准防控的原则，按照展览活动举办地疫情应急响应级别，分别由相应的省级、地市级、区县级疫情防控领导机构对展览活动出具举办必要性和已落实防控举措、具备举办条件的评估意见。商务、公安、卫生健康等有关部门要强化协同联动、加强信息沟通，根据各级疫情防控领导机构出具的意见和职责分工做好展览活动的审批、备案和监督管理工作，指导各有关单位制订并严格落实各项防控措施。

（二）督促展览活动有关单位落实疫情防控责任。按照“谁举办、谁负责，谁组织、谁负责”的原则，展览活动举办单位（主、承办单位，下同）承担疫情防控主体责任，负责制定展览活动期间具有针对性和可操作性的疫情防控工作方案和应急处置预案，指定专人负责疫情防控工作。

展览场所单位承担展览活动疫情防控现场管理责任，负责展览场所防疫消杀、通风保洁、现场疫情防控设备、相关防疫物资、应急处置场地的安排配置。

其他展览活动参与者承担联防联控责任，要按照展览活动疫情防控要求自觉接受体温检测，出示健康码，科学戴口罩，做好自我防护。如在现场出现发热、咳嗽等疑似症状，要立即自我隔离并及时通知疾控工作人员。

（三）鼓励展览活动创新服务。地方政府要支持和鼓励运用互联网大数据等手段做好展览活动，采取线上预登记、错峰观展、人员限流、实名入场等方式，做好展商观众注册、安检、门禁、自助取证、顺序入场、精准对接等服务工作。

（四）加强工作人员防疫培训。地方政府要指导督促展览活动举办单位、展览场所单位、相关服务企业等提前做好本单位工作人员健康排查，开展防疫知识培训，加强疫情防控信息宣传，确保防疫措施落到实处。

（五）加大疫情防控宣传工作。地方政府要引导和支持展览活动举办单位采取线上线下多样化宣传方式积极开展展览活动期间防疫宣传，确保所有展览活动参与人员知悉防疫要求，主动配合疫情防控工作。

四、压实压紧展览活动举办单位、场所单位等疫情防控责任

展览活动举办单位和展览场所单位要根据展览活动实际情况，因地制宜，有针对性地做好现场应急医疗、人员隔离、消防安全、防疫物资配备等服务保障安排；要依据国务院联防联控机制和国家卫生健康委有关规定，全面落实早发现、早报告、早隔离、早治疗的“四早”措施；严格遵循信息必验、身份必录、体温必测、消毒必做、突发必处的“五必”要求。

（一）展览活动举办单位的防控责任

1.提前通知相关人员参加展览活动的安全防护要求及健康查验程序，做好注册人员健康状况信息核验和登记，对展览活动参与人员实施健康排查，确保人员信息可追溯。

2.按防控要求合理规划展览活动人员动线，提出流量管控方案。根据场地规模控制入馆人员数量，实行预约分流、分批错时入馆等管控措施，引导参展观展人员保持合理间距。

3.合理规划场地分区及展位布局，通道宽度和展位间距要符合防控要求。加强现场人流管控，引导人员有序观展，有序进出。

4.加强防疫物资保障，展前做好口罩、消毒用品等必要的防疫物资储备。

5.对所有展览活动参与人员实施健康排查，并做好疫情防控宣传和培训工作。

6．一旦发现新冠肺炎疑似病例，须第一时间启动应急处置预案并组织实施。

7.主动配合展览活动举办地相关管理部门，认真落实好各项疫情防控要求。

（二）展览场所的防控责任

8.根据展览活动规模情况，展览场所应划定明确的功能分区，如落客区、测温区、安检区、登录区、展览展示区等，做好观展线路的指引，有效控制人流和人员活动间距。

9.展览场所单位应配备必要的门禁、安检、测温设备，设置临时隔离区，并配备适量应急防疫物资。

10.应建立现场工作人员健康档案，做好日常健康监测。工作人员须戴口罩，人员入场须进行体温检测。

11.展览场所单位在展览活动举办前，应对展览场所和设备，特别是空调、通风系统进行全面检查、清洁消杀。

在展览活动布展、开展和撤展期间，应加强展馆通风换气，每日定时高频做好公共区域、高频接触点位的清洁消毒，并在相关区域更新公示消毒情况。

12.展览场所单位要协助展览活动举办单位做好展览活动参与人员的健康排查、安全检查、秩序维护、现场巡视、流量管控、应急处置等现场疫情防控工作。

13.通过海报、广播、短信、电子屏、宣传视频等形式加强疫情防控知识宣传，倡导良好卫生习惯，增强健康防护意识，营造文明参展参观良好氛围。

（三）服务商、参展商的联防联控责任

14.预先做好本单位工作人员健康排查，按要求向展览活动举办单位如实报备。

15.负责做好本单位展位和服务区域的日常消毒、人员防护工作。

16.如本单位工作人员出现发热、咳嗽等疑似症状，要主动自我隔离并及时告知展览活动举办单位，配合做好早期排查等工作。

（四）餐饮管理防控措施

17.餐饮服务商必须具备法定经营资质，做好服务人员的卫生防护，严格按照国家食品安全相关法律、法规和相关防控措施的规定开展工作。

18.设立专用就餐区，间隔安全距离取餐用餐。

19.加强就餐区卫生管理，定时做好防疫消毒工作。

（五）垃圾处置防控措施

20.加强垃圾密闭化、分类化管理，及时收集并清运，做到日产日清。

21.展览场所内应设置“废弃口罩垃圾桶”并作好标识。安排专人每日及时收集、集中消毒，并按有毒有害垃圾进行处置。

各地要根据本指导意见，结合本地疫情常态化防控工作实际，指导展览活动有关单位进一步细化、优化和完善各项疫情防控措施，确保展览活动疫情防控工作不折不扣落细落实落地。

商务部 公安部 卫生健康委

2020年7月3日

1. 文旅部：印发《剧院等演出场所恢复开放疫情防控措施指南》（第二版）等的通知

剧院等演出场所恢复开放疫情防控措施指南

（第二版）

为贯彻落实习近平总书记关于统筹推进新冠肺炎疫情防控和经济社会发展工作的重要指示精神及《国务院应对新型冠状病毒感染肺炎疫情联防联控机制关于做好新冠肺炎疫情常态化防控工作的指导意见》（国发明电〔2020〕14号）要求，加强剧院等演出场所常态化疫情防控工作，制定本指南。

一、总体要求

（一）坚持常态防控。各地文化和旅游行政部门应当按照“属地原则”，严格遵守当地疫情防控指挥部门的要求，结合本地区实际情况，制定包含演出场所在内的文化市场疫情防控方案和应急预案，并及时进行动态调整。要时刻绷紧疫情防控这根弦，坚决克服麻痹思想、厌战情绪、侥幸心理、松劲心态，全面排查防控漏洞、紧盯防控重点环节、切实落实防控要求，抓紧抓实抓细常态化疫情防控，确保安全。

（二）坚持有序开放。在充分做好防疫措施的情况下，在低风险地区，经当地党委、政府同意，可以举办营业性演出活动，但暂缓举办中大型营业性演出活动，暂缓新批涉外、涉港澳台营业性演出活动（演职人员已在境内的除外），暂时取消演出前后的现场互动环节。中高风险地区，暂缓举办营业性演出活动。

（三）坚持预约限流。恢复开放的演出场所应当严格执行人员预约限流措施。剧院等演出场所观众人数不得超过剧院座位数的30%，应当间隔就坐，保持1米以上距离。含有多个剧场的综合性演出场所，同时只能开一个剧场。在歌舞娱乐场所、旅游景区、主题公园、游乐园、宾馆、饭店、酒吧、餐饮场所、现场音乐厅（LiveHouse）等场所举办营业性演出，演出主办方应当安排工作人员在现场做好人员疏导。

二、场所防控管理

（四）落实防控主体责任。演出场所应当严格执行疫情防控规定，按照相关技术指南，制定本场所防控具体措施和应急预案，并开展应急演练，提高处置能力，将日常值守、清洁消毒、检测登记、垃圾清理、场地巡查、安全管理等各个防疫环节的责任落实到具体岗位和个人，并根据当地疫情防控的要求，及时动态调整。

（五）加强清洁消毒。演出场所应当建立《清洁消毒记录表》，明确消毒范围和频次，记录消毒时间、责任人等信息。每场演出前后，应当对场所舞台区、观众区、化妆间、通道、出入口、行政办公场地等公共区域进行全面清洁消毒（建议使用有效氯500mg/L的含氯消毒液消毒）。

（六）加强通风换气。演出场所应当在条件允许情况下首选自然通风。如采用集中空调通风系统应在开启前检查设备是否正常，对冷却塔等进行清洗，保持新风口清洁；运行过程中以最大新风量运行，加强对冷却水、冷凝水的卫生管理，定期对送风口等设备和部件进行清洗、消毒或更换；出现确诊病例和疑似病例时，应关闭集中空调通风系统并在疾控部门指导下进行清洗消毒。

（七）配备防护用品。演出场所应当配备充足的口罩（建议配备符合一次性使用医用口罩标准或相当防护级别的口罩）、体温检测设备、一次性手套、洗手液、消毒剂等消毒防护用品。在公共休息区、洗手间等区域配备洗手液、速干手消毒剂等清洁消毒物品，便于消费者和员工随时清洁消毒。消毒物品应当严格按照说明书正确储存和使用，远离火源和电源，不得混用、混放。应当定期检查防护用品，及时补充更换。

（八）规范垃圾处理。演出场所应当设置专用垃圾桶等垃圾收集容器，引导将废弃口罩、消毒纸巾等用品投入专用垃圾收集容器，有条件的用塑料袋密闭扎紧后投放。垃圾收集容器应当做到干净整洁无异味，防止满冒，日产日清，并定时定点对垃圾收集容器及周边区域地面进行消毒。

（九）设置临时隔离区。演出场所应当设立（临时）隔离区，位置相对独立，设立提醒标识，配备安全有效的防护用品，并指定专人负责，以备人员出现发热等症状时立即进行暂时隔离。

（十）加强防疫宣传。演出场所应当在醒目位置通过设置提示牌、摆放宣传品、电子显示屏等多种方式，加强疫情防控知识科普宣传，提升员工及消费者的疫情防控意识。

（十一）排查安全隐患。演出场所应当及时排查消毒用品存放、电源管理等安全隐患，并对照《文化部关于落实安全生产责任加强文化市场安全生产工作的通知》（文市发〔2017〕5号）有关规定，加强自查自检，不符合安全条件的立整立改。

（十二）鼓励线上服务。鼓励推广在线实名制购票及电子票，鼓励使用在线支付，鼓励采取二维码闸机验票，尽量减少直接接触。对剧院等专业演出场所，实行实名制购票和实名制入场。鼓励云音乐会、空中剧场等线上演出活动，丰富文化产品供给。

三、演职人员和观众管理

（十三）做好演职人员管理。演出主办方应当与参演单位和个人签订安全协议或者健康承诺书，提前做好对演职人员（含行政、后勤等工作人员）的体温检测等防控措施，并建立《演职人员健康记录表》。每场演出尽量压缩不必要的演职人员，并注意保持一定距离。演员人均化妆间面积不低于5平米。

（十四）做好入场检测登记。演出场所应当配备测量体温设施设备，并安排专人值守。观众进入演出场所必须佩戴口罩，测量体温，出示健康码。观众拒绝佩戴口罩或者体温异常的，应当拒绝其进入。

（十五）加强现场巡查。演出场所应当安排专人做好演出现场管理，提醒观众在入场、退场及观演期间，科学佩戴口罩，对号入座，保持1米以上距离。

四、员工健康管理

（十六）做好员工健康监测。演出场所应当按照要求做好员工健康管理，建立《员工健康记录表》，每日对员工进行两次体温检测，随时掌握员工健康状态、出行轨迹等情况。发现员工出现发热、咳嗽、乏力、鼻塞、流涕、咽痛、腹泻等症状，及时安排去定点医疗机构就诊，并跟踪相关情况。

（十七）指导员工做好个人防护。演出场所应当及时对员工进行常态化疫情防控措施、应急处置等方面的培训，督促员工掌握疫情防控、个人防护、卫生健康及应急处置等方面的知识，并做好个人防护。

（十八）减少员工聚集。根据实际采取错时上下班、弹性工作制或者居家办公方式，员工上岗应佩戴口罩，打喷嚏时用纸巾遮住口鼻或采用肘臂遮挡，督促上班员工不串岗、不扎堆。加强员工用餐管理，鼓励实行错峰就餐、分散用餐。科学管理工作会议，减少开会频次和会议时长。提醒员工减少不必要外出，尽量避开密集人群，避免在公共场所长时间停留。

五、异常情况处置

（十九）建立沟通机制。演出场所应当建立疫情应急沟通机制，了解卫生健康行政部门、疾控机构、就近定点医疗机构、社区卫生服务中心等联系方式，并确保全体员工知晓。畅通疫情上报通道，发现疫情应当及时向属地卫生健康行政部门、文化和旅游行政部门报告。

（二十）做好发现疫情时的应对处置。演出场所如出现疑似疫情，应当立即启动应急预案，做好现场管理，避免恐慌，在专业机构指导下采取相应疫情防控处置措施，配合相关部门做好密切接触者的排查管理并暂时关闭场所。

六、保障措施

（二十一）加强组织领导。各地文化和旅游行政部门应当严格落实属地管理责任，提升防控和应急处置能力，加强与当地卫生健康行政部门之间的联动，确保开放管理工作平稳有序。

（二十二）加强外籍等演职人员信息核验。文化和旅游行政部门在审批含有外国或者港澳台地区演职人员（演职人员已在境内）的营业性演出活动时，应当核验相关演职人员最近一次在境内演出批准文件、出入境记录等信息，演出主办方应配合做好演职人员身体健康检测，并作出书面承诺。

（二十三）加强监督检查。各地文化和旅游行政部门应当加强对恢复开放的演出场所的巡查和监管力度，依法依规查处违法经营行为，及时发现隐患苗头并有效处置，切实保障消费者合法权益，维护市场经营秩序。

（二十四）加强应急管理。各地文化和旅游行政部门应当按照应急预案，明确疫情防控、安全突发事件应急措施和处置流程，开展排查、评估和宣传培训工作，发生异常情况及时上报，并督促指导暂时关闭场所。

互联网上网服务营业场所恢复开放

疫情防控措施指南

（第二版）

为贯彻落实习近平总书记关于统筹推进新冠肺炎疫情防控和经济社会发展工作的重要指示精神和《国务院应对新型冠状病毒感染肺炎疫情联防联控机制关于做好新冠肺炎疫情常态化防控工作的指导意见》（国发明电〔2020〕14号）要求，加强互联网上网服务营业场所（以下简称“上网服务场所”）常态化疫情防控工作，制定本指南。

一、总体要求

（一）坚持常态防控。各地文化和旅游行政部门应当按照“属地原则”，严格遵守当地疫情防控指挥部门的要求，结合本地区实际情况，制定包含上网服务场所在内的疫情防控方案和应急预案，并及时进行动态调整。要时刻绷紧疫情防控这根弦，坚决克服麻痹思想、厌战情绪、侥幸心理、松劲心态，全面排查防控漏洞、紧盯防控重点环节、切实落实防控要求，抓紧抓实抓细常态化疫情防控，确保安全。

（二）坚持有序开放。在充分做好防疫措施的情况下，各地应当严格按照属地党委、政府统一部署，以县域为单位，分区分级，确定上网服务场所恢复开放时间和具体要求。疫情中高风险地区，建议暂停营业。

（三）坚持预约限流。恢复开放的上网服务场所应当严格执行人员限流限量措施，应当采取预约消费、错峰入场、间隔就坐、限制停留时间等措施，并对场所内消费者数量实行动态管理，防范聚集性风险。

二、消费者保护

（四）落实戴口罩、检测登记制度。上网服务场所应当配备测量体温设施设备，并安排专人值守。消费者进入场所必须科学佩戴口罩、测量体温、出示健康码，严格落实实名登记制度。消费者不戴口罩或者体温异常的，场所应当拒绝其进入。

（五）实施预约限流措施。上网服务场所应当采取预约消费、错峰入场、间隔就坐等措施，限制上网人流。上网服务场所接纳消费者人数不得超过核定人数的50%，每个包间也不得超过核定人数的50%（建议在包间门口明示限额人数）。消费者上网时间不超过两小时。

（六）建立值守制度。上网服务场所应当安排专人监督进入场所的员工和消费者遵守相关防疫要求，对消费者未按要求科学佩戴口罩行为进行劝导，及时对消费者进行疏导、分流，避免人群聚集，保持1米以上距离。

三、场所防控管理

（七）落实防控主体责任。上网服务场所应当落实防控主体责任，严格执行疫情防控规定，制定本场所防控工作制度和应急预案，并开展应急演练，提高处置能力。应当将日常值守、清洁消毒、检测登记、垃圾清理、场地巡查、安全管理等各个防疫环节的责任落实到具体岗位和个人，并根据当地疫情防控指挥要求，及时动态调整。

（八）加强清洁消毒。上网服务场所应当建立《清洁消毒记录表》，明确消毒范围和频次，记录消毒时间、责任人等信息。每日营业前，应当对场所内外公共部位（门厅、前台、独用楼道、独用电梯、楼梯、场所内卫生间、门把手等）进行全面清洁消毒（建议使用有效氯500mg/L的含氯消毒液喷洒或者擦拭消毒），并对显示器、键盘、耳机、鼠标进行专门清洁消毒（建议使用有效氯250-500mg/L的含氯消毒剂进行擦拭消毒，也可采用消毒湿巾进行擦拭），软质材料的座位可以增加布套并提高更换频次。营业期间，应当及时对消费者使用过的显示器、键盘、耳机、鼠标以及座位、扶手等进行清洁消毒，做到“一客一消毒”。

（九）加强通风换气。上网服务场所应当在条件允许情况下首选自然通风。如采用集中空调通风系统，应当在开启前检查设备是否正常，对冷却塔等进行清洗，保持新风口清洁；运行过程中以最大新风量运行，加强对冷却水、冷凝水的卫生管理，定期对送风口等设备和部件进行清洗、消毒或更换；出现确诊病例和疑似病例时，应关闭集中空调通风系统并在疾控部门指导下进行清洗消毒。

（十）规范垃圾处理。上网服务场所应当设置专用垃圾桶等垃圾收集容器，引导将废弃口罩、消毒纸巾等用品投入专用垃圾收集容器，有条件的用塑料袋密闭扎紧后投放。垃圾收集容器应当做到干净整洁无异味，防止满冒，日产日清，并定时定点对垃圾收集容器及周边区域地面进行消毒。

（十一）设置临时隔离区。上网服务场所应当设立（临时）隔离区，位置相对独立，设立提醒标识，配备安全有效的防护用品，并指定专人负责，以备人员出现发热等症状时立即进行暂时隔离。

（十二）配备防护用品。上网服务场所应当配备充足的口罩（建议配备符合一次性使用医用口罩标准或者相当防护级别的口罩）、体温检测设备、一次性手套、洗手液、消毒剂等消毒防护用品。在公共休息区、洗手间等区域配备洗手液、速干手消毒剂等清洁消毒物品，便于消费者和员工随时清洁消毒。消毒物品应当严格按照说明书正确储存和使用，远离火源和电源，不得混用、混放。应当定期检查防护用品，及时补充更换。

（十三）加强疫情防控知识宣传。上网服务场所应在醒目位置张贴健康提示，利用官方网站、微信公众号、显示屏等宣传宣传疫情期间场所运营规则以及新冠肺炎等传染病防控知识，提升员工及消费者的疫情防控意识。

（十四）排查安全隐患。上网服务场所应当及时排查安全隐患，并按照《文化部关于落实安全生产责任加强文化市场安全生产工作的通知》（文市发〔2017〕5号）有关规定，加强自查自检，不符合安全条件的立整立改。

四、员工健康管理

（十五）做好员工健康检测。上网服务场所应当按照要求做好员工健康管理，建立《员工健康记录表》，每日对员工进行两次体温检测，随时掌握员工健康状态、出行轨迹等情况。发现员工出现发热、咳嗽、乏力、鼻塞、流涕、咽痛、腹泻等相关症状，及时安排去就近定点医疗机构就诊，并跟踪相关情况。

（十六）减少员工聚集。加强员工用餐管理，鼓励实行错峰就餐、分散用餐。科学管理工作会议，减少开会频次和会议时长。提醒员工减少不必要外出，尽量避开密集人群，避免在公共场所长时间停留。

（十七）做好员工个人防护。上网服务场所应当及时对员工进行常态化疫情防控措施、应急处置等方面的培训，督促员工掌握疫情防控、个人防护、卫生健康及应急处置等方面的知识。

五、异常情况处置

（十八）建立沟通机制。上网服务场所应当建立疫情应急沟通机制，了解卫生健康行政部门、疾控机构、就近定点医疗机构、社区卫生服务中心等联系方式，并确保全体员工知晓。畅通疫情上报通道，发现疫情应当及时向当地卫生健康行政部门、文化和旅游行政部门报告。

（十九）做好应急处置。上网服务场所如出现疑似疫情，应当立即启动应急预案，做好现场管理，避免恐慌，在专业机构指导下采取相应疫情防控处置措施，暂时关闭场所，并配合相关部门做好密切接触者的排查管理。

六、保障措施

（二十）加强组织领导。各地文化和旅游行政部门应当严格落实属地管理责任，提升防控和应急处置能力，加强与当地卫生健康行政部门之间的联动，确保开放管理工作平稳有序。

（二十一）加强监督检查。各地文化和旅游行政部门应当加强对恢复开放的上网服务场所的巡查和监管力度，依法依规查处违法经营行为，切实保障消费者合法权益，维护市场经营秩序。

（二十二）加强应急管理。各地文化和旅游行政部门应当按照应急预案，明确疫情防控、安全突发事件应急措施和处置流程，开展排查、评估和宣传培训工作，及时发现隐患苗头并有效处置，发生异常情况及时上报并暂时关闭场所。

娱乐场所恢复开放疫情防控措施指南

（第二版）

为贯彻落实习近平总书记关于统筹推进新冠肺炎疫情防控和经济社会发展工作的重要指示精神和《国务院应对新型冠状病毒感染肺炎疫情联防联控机制关于做好新冠肺炎疫情常态化防控工作的指导意见》（国发明电〔2020〕14号）要求，加强娱乐场所常态化疫情防控工作，制定本指南。

一、总体要求

（一）坚持常态防控。各地文化和旅游行政部门应当按照“属地原则”，严格遵守当地疫情防控指挥部门的要求，结合本地区实际情况，制定包含娱乐场所在内的疫情防控方案和应急预案，并及时进行动态调整。要时刻绷紧疫情防控这根弦，坚决克服麻痹思想、厌战情绪、侥幸心理、松劲心态，全面排查防控漏洞、紧盯防控重点环节、切实落实防控要求，抓紧抓实抓细常态化疫情防控，确保安全。

（二）坚持有序开放。在充分做好防疫措施的情况下，各地应当严格按照属地党委、政府统一部署，以县域为单位，分区分级，确定娱乐场所恢复开放时间和具体要求。疫情中高风险地区，建议暂停营业。

（三）坚持预约限流。恢复开放的娱乐场所应当严格执行人员限流限量措施，实行预约消费、错峰入场、限制停留时间等措施，并对场所内消费者数量实行动态监控，防范聚集性风险。

二、消费者保护

（四）落实戴口罩、检测登记制度。娱乐场所应当配备测量体温设施设备，并安排专人值守。消费者进入场所必须佩戴口罩、测量体温、出示健康码，并进行实名登记。消费者不戴口罩或者体温异常的，场所应当拒绝其进入。

（五）实施预约限流措施。娱乐场所应当采取预约消费、错峰入场等措施，对消费者进行限流限量。歌舞娱乐场所接纳消费者人数不得超过核定人数的50%，每个包间接纳消费者人数也不得超过核定人数的50%（建议在包间门口明示限额人数）；游艺娱乐场所接纳消费者人数不得超过核定人数的50%。消费者娱乐时间不超过两小时。

（六）建立值守制度。娱乐场所应当安排专人监督进入场所的员工和消费者遵守相关防疫要求，提醒消费者科学佩戴口罩，及时对消费者进行疏导、分流，避免聚集，保持1米以上距离。

三、场所防控管理

（七）落实防控主体责任。娱乐场所应当落实防控主体责任，严格执行疫情防控规定，制定本场所防控工作制度和应急预案，并开展应急演练，提高处置能力。应当将日常值守、清洁消毒、检测登记、垃圾清理、场地巡查、安全管理等各个防疫环节的责任落实到具体岗位和个人，并根据当地疫情防控要求，及时动态调整。

（八）加强清洁消毒。娱乐场所应当建立《清洁消毒记录表》，明确消毒范围和频次，记录消毒时间、责任人等信息。每日营业前，应当对场所内外公共部位（门厅、前台、独用楼道、独用电梯、楼梯、场所内卫生间、门把手等）进行全面消毒清洁（建议使用有效氯500mg/L的含氯消毒液喷洒或者擦拭消毒），并对娱乐设备及附属设施进行专门清洁消毒（建议使用有效氯250-500mg/L的含氯消毒剂进行擦拭，也可采用消毒湿巾进行擦拭），软质材料的座位可以增加布套并提高更换频次。歌舞娱乐场所应当及时对消费者使用过的麦克风、点歌按钮、屏幕、座位、扶手、桌台等设备消毒或者更换。游艺娱乐场所应当及时对消费者使用过的设备按键、摇杆、代币及座位、扶手等相关附属设备进行消毒或者更换，做到“使用一次消毒一次”。

（九）加强通风换气。娱乐场所应当在条件允许情况下首选自然通风。如采用集中空调通风系统应当在开启前检查设备是否正常，对冷却塔等进行清洗，保持新风口清洁；运行过程中以最大新风量运行，加强对冷却水、冷凝水的卫生管理，定期对送风口等设备和部件进行清洗、消毒或更换；出现确诊病例和疑似病例时，应当关闭集中空调通风系统并在疾控部门指导下进行清洗消毒。

（十）规范垃圾处理。娱乐场所应当设置专用垃圾桶等垃圾收集容器，引导将废弃口罩、消毒纸巾等用品投入专用垃圾收集容器，有条件的用塑料袋密闭扎紧后投放。垃圾收集容器应当做到干净整洁无异味，防止满冒，日产日清，并定时定点对垃圾收集容器及周边区域地面进行消毒。

（十一）设置临时隔离区。娱乐场所应当设立（临时）隔离区，位置相对独立，设立提醒标识，配备安全有效的防护用品，并指定专人负责，以备人员出现发热等症状时立即进行暂时隔离。

（十二）配备防护物品。娱乐场所应当配备充足的口罩（建议配备符合一次性使用医用口罩标准或者相当防护级别的口罩）、体温检测设备、一次性手套、洗手液、消毒物品等防护用品。在公共休息区、洗手间等区域配备洗手液、速干手消毒剂等清洁物品，便于消费者和员工随时清洁。应当严格按照说明书正确储存和使用消毒物品，远离火源和电源，不得混用、混放。定期检查防护用品，及时补充更换。

（十三）加强疫情防控知识宣传。娱乐场所应当在醒目位置通过设置提示牌，张贴健康提示、显示屏宣传等多种方式，宣传疫情期间场所运营规则及防控知识，提升员工及消费者的疫情防控意识。

（十四）排查安全隐患。娱乐场所应当及时排查安全隐患，并按照《文化部关于落实安全生产责任加强文化市场安全生产工作的通知》（文市发〔2017〕5号）有关规定，加强自查自检，不符合安全条件的立整立改。

四、员工健康管理

（十五）做好员工健康检测。娱乐场所应当按照要求做好员工健康管理，建立《员工健康记录表》，每日对员工进行两次体温检测，随时掌握员工健康状态、出行轨迹等情况。发现员工出现发热、咳嗽、乏力、鼻塞、流涕、咽痛、腹泻等相关症状，及时安排去就近定点医疗机构就诊，并跟踪相关情况。

（十六）减少员工聚集。加强员工用餐管理，鼓励实行错峰就餐、分散用餐。科学管理工作会议，减少开会频次和会议时长。提醒员工减少不必要外出，尽量避开密集人群，避免在公共场所长时间停留。

（十七）指导员工做好个人防护。娱乐场所应当及时对员工进行常态化疫情防控措施、应急处置等方面的培训，督促员工掌握疫情防控、个人防护、卫生健康及应急处置等方面的知识，养成戴口罩、勤洗手、常通风、公筷制等卫生习惯和健康生活方式。

五、异常情况处置

（十八）建立沟通机制。娱乐场所应当建立疫情应急沟通机制，了解卫生健康行政部门、疾控机构、就近定点医疗机构、社区卫生服务中心等联系方式，并确保全体员工知晓。畅通疫情上报通道，发现疫情应当及时向当地卫生健康行政部门、文化和旅游行政部门报告。

（十九）做好发现疫情时的应对处置。娱乐场所如出现疑似疫情，应当立即启动应急预案，做好现场管理，避免恐慌，在专业机构指导下采取相应疫情防控处置措施，暂时关闭场所，并配合相关部门做好密切接触者的排查管理。

六、保障措施

（二十）加强组织领导。各地文化和旅游行政部门应当严格落实属地管理责任，提升防控和应急处置能力，加强与当地卫生健康行政部门之间的联动，确保恢复开放工作平稳有序。

（二十一）加强监督检查。各地文化和旅游行政部门应当加强对恢复开放的娱乐场所的巡查和监管力度，依法依规查处违法经营行为，切实保障消费者合法权益，维护市场经营秩序。

（二十二）加强应急管理。各地文化和旅游行政部门应当按照应急预案，明确疫情防控、安全突发事件应急措施和处置流程，开展排查、评估和宣传培训工作，及时发现隐患苗头并有效处置，发生异常情况及时上报并暂时关闭场所。

　　文化和旅游部市场管理司

　　 2020年6月22日

1. 人社部等3部门：进一步做好就业扶贫工作

为贯彻落实习近平总书记关于就业扶贫的有关指示要求，贯彻落实党中央、国务院关于决战决胜脱贫攻坚和应对疫情影响强化稳就业举措决策部署，日前，人力资源社会保障部、财政部、国务院扶贫办印发《关于进一步做好就业扶贫工作的通知》，明确要求围绕贫困劳动力出得去、稳得住、留得下，帮助有劳动能力和就业意愿的贫困劳动力外出务工，帮助已外出贫困劳动力稳定务工，力争今年外出务工规模不降低、有提高。

通知强调，要优先组织贫困劳动力外出务工，加强贫困劳动力就业岗位信息的归集发布，加强输出地和输入地劳务对接，扩大劳务输出规模。优先留用贫困劳动力，建立企业定期联系专人帮扶机制，对生产经营遇到困难、确实需要裁员的企业，提前介入指导，同等条件下不裁贫困劳动力。优先帮助转岗就业，将就业转失业的贫困劳动力纳入当地就业困难人员范围，加强就业帮扶，及时提供职业介绍、岗位推荐等服务。优先落实扶持政策，对企业、服务机构以及贫困劳动力，优先落实失业保险稳岗返还、困难企业培训补贴、一次性吸纳就业补贴、社会保险补贴、就业创业服务补助、一次性求职创业补贴、交通费补贴等政策，全力帮助贫困劳动力稳在企业、稳在当地。同时，拓宽就地就近就业渠道，充分尊重贫困劳动力意愿，多渠道帮助返乡留乡贫困劳动力就业创业。

通知提出，要始终聚焦重点，把52个未摘帽贫困县、“三区三州”等深度贫困地区、易地扶贫搬迁大型安置区以及湖北省的贫困劳动力作为重点对象，优先组织输出、优先稳岗转岗、优先托底安置、优先实施救助。

通知要求，要强化考核评估，将贫困劳动力就业情况作为年度脱贫攻坚成效考核的重要内容，东部地区今年吸纳中西部地区贫困劳动力务工总数不少于去年，中西部地区外出务工贫困劳动力总数不少于去年。

2020.6.23

1. 国家能源局：印发《2020年能源工作指导意见》

2020年能源工作指导意见

2020年是全面建成小康社会和“十三五”规划收官之年，也是为“十四五”良好开局打下坚实基础的关键之年。为深入贯彻落实党中央、国务院关于统筹做好新冠肺炎疫情防控和经济社会发展的决策部署，顺应国内外发展环境和形势变化，妥善应对面临的问题和挑战，在常态化疫情防控中做好能源发展改革工作，保障能源安全，推动能源高质量发展，制定本指导意见。

一、总体要求

（一）指导思想

以习近平新时代中国特色社会主义思想为指导，坚持稳中求进工作总基调，深入践行新发展理念，遵循“四个革命、一个合作”能源安全新战略，坚决贯彻落实党中央、国务院决策部署，紧紧围绕“六稳”工作和“六保”任务，着力加强煤电油气产供储销衔接，保持能源产业链和供应链稳定；着力加大补短板、强弱项力度，提升能源安全底线保障能力；着力深化改革、扩大开放，增强创新驱动能力，培育壮大新产业新业态新模式，持续推动能源发展质量、效率和动力变革，为全面建成小康社会和建设现代化经济体系提供坚强能源保障。

（二）主要目标

2020年主要预期目标如下：

能源消费。全国能源消费总量不超过50亿吨标准煤。煤炭消费比重下降到57.5%左右

供应保障。石油产量约1.93亿吨，天然气产量约1810亿立方米，非化石能源发电装机达到9亿千瓦左右。

质量效率。能源系统效率和风电、光伏发电等清洁能源利用率进一步提高。西部地区具备条件的煤电机组年底前完成超低排放改造。

惠民利民。新增清洁取暖面积15亿平方米左右，新增电能替代电量1500亿千瓦时左右，电能占终端能源消费比重达到27%左右。光伏扶贫等能源扶贫工程持续推进，完成“三区三州”和抵边村寨农网改造升级。

改革创新。深入推进电力现货市场连续结算试运行，具备条件的地区正式运行。电网主辅分离改革进一步深化。完善油气勘查开采管理体制，健全油气管网运营机制。能源革命试点深入推进。稳妥有序推进能源关键技术装备攻关，推动储能、氢能技术进步与产业发展。

（三）政策取向

——坚持以保障能源安全为首要任务。增强忧患意识，强化底线思维，着眼能源发展面临的内外部环境变化和风险挑战，建立健全能源供需联动等工作机制，着力补强能源供应链的短板和弱项，抓紧抓实抓细保供措施，切实提高能源安全保障能力和风险管控应对能力。积极推动能源领域军民融合发展。

——坚持以惠民利民为根本宗旨。牢记初心使命，以满足人民群众美好生活需要为出发点和落脚点，深入推进贫困地区能源资源开发利用，加大民生用能基础设施投入，加快能源惠民利民工程建设，推动能源公共服务向农村延伸、向贫困地区延伸，统筹做好能源领域脱贫攻坚和乡村振兴各项工作。严格执行阶段性降电价、气价政策，降低社会用能成本。

——坚持以清洁低碳为发展目标。牢固树立绿水青山就是金山银山的理念，坚持清洁低碳战略方向不动摇，加快化石能源清洁高效利用，大力推动非化石能源发展，持续扩大清洁能源消费占比，推动能源绿色低碳转型。

——坚持以质量效益为主攻方向。持续深化能源供给侧结构性改革，优化存量资源配置，扩大优质增量供给，集中力量突破能源技术装备短板，推动能源改革不断取得新进展，持续提升能源系统质量和运行效率。

二、全力以赴，做好疫情防控能源供应保障

抓紧抓实抓细保供措施。建立健全重点突出、涵盖全国、运转高效的能源供给保障体系，支撑经济社会秩序全面恢复。密切关注煤炭供需变化，及时组织产运调度，确保港口、电厂库存保持在合理水平。优化电网调度运行，重点满足民生、医疗和应急物资生产企业等用电需求。妥善应对油气市场形势变化，保持油气产供储销衔接有序、供应稳定。

密切跟踪供需形势变化。进一步完善能源统计、信息共享、分析会商等工作机制。密切关注境外疫情对全球和我国能源供应链产业链的影响，加强能源监测预测预警，早做预案，适时启动分级动用和应急响应机制。建立能源形势监测日报制度，监测全国和重点地区煤电油气供需变化，重点关注苗头性、倾向性、潜在性问题，及早应对，妥善解决。

压实保供和安全生产责任。严格落实能源保供属地责任，加强组织协调，推动解决能源供应问题，保障可供能源资源充足稳定。密切关注能源企业经营情况，针对行业反映集中、影响较大的问题，适时研究出台支持政策措施予以有效解决。落实企业安全生产主体责任，积极采取针对性防控措施，避免发生影响能源安全的重大事故。组织能源企业做好高峰时段用能供应保障预案。

三、多措并举，增强油气安全保障能力

加大油气勘探开发力度。大力提升油气勘探开发力度保障能源安全，狠抓主要目标任务落地，进一步巩固增储上产良好态势。重点做大渤海湾、四川、新疆、鄂尔多斯四大油气上产基地，推动常规天然气产量稳步增加，页岩气、煤层气较快发展。探索湖北宜昌等地区页岩气商业化开发。加快推进煤层气（煤矿瓦斯）规模化开发利用，落实低产井改造方案。推动吉木萨尔等页岩油项目开发取得突破。

加快天然气产供储销体系建设。加快管网和储气设施建设，补强天然气互联互通和重点地区输送能力短板，加快形成“全国一张网”。压实上游供气企业和国家管网公司储气责任，加快储气库基地及储气设施重点项目建设。有序推进液化天然气（LNG）长期协议落实和现货采购。健全项目用海、用地、环评等协调机制，积极创造条件推动项目建设。

专栏一：油气储运重点工程

在建油气管道重点工程：中俄东线中段、新疆煤制气外输管道（潜江-郴州段）、神木-安平煤层气管道、青宁天然气管道等；日照-濮阳-洛阳原油管道等；唐山LNG外输管线（宝坻-永清）、粤东LNG项目一期配套管线等；部分区域管网及互联互通管道等。

在建储气库重点工程：雷61、双台子储气库群、吉林双坨子储气库、大庆四站、西南相国寺储气库（扩容达产）、呼图壁储气库（扩容达产）、苏桥储气库（扩容达产）、驴驹河储气库、中石油文23储气库、苏东39-61储气库、中原文23储气库等。

在建LNG接收站重点工程：广西北海、浙江二期、唐山三期等。

增强油气替代能力。有序推进国家规划内的内蒙古、新疆、陕西、贵州等地区煤制油气示范项目建设，做好相关项目前期工作。健全燃料乙醇政策体系，稳妥扩大生物燃料乙醇生产和车用乙醇汽油推广使用。启动生物天然气项目建设，研究加大政策支持力度，推动生物天然气产业化发展。

专栏二：煤制油气重点项目

在建项目：伊泰鄂尔多斯200万吨/年煤炭间接液化示范项目、伊泰伊犁100万吨/年煤炭间接液化示范项目、内蒙古汇能16亿立方米/年煤制天然气项目（二期工程）。

前期工作项目：兖矿集团榆林400万吨/年煤炭间接液化项目、贵州渝富毕节（纳雍）200万吨/年煤炭间接液化示范项目、内蒙古华星40亿立方米/年煤制天然气示范项目。

四、深化供给侧结构性改革，不断提升能源发展质量

优化煤炭煤电产能结构。坚持煤炭产能置换长效机制，不断完善产能置换政策。推动煤炭行业“上大压小、增优减劣”，落实年产能30万吨以下煤矿分类处置工作方案，加快淘汰落后煤矿产能。按照煤炭发展规划和产业政策，有序建设大型现代化煤矿，推动释放煤炭先进产能。统筹推进现役煤电机组超低排放和节能改造，西部地区具备条件的机组年底前完成超低排放改造。在确保电力、热力供应的基础上，继续淘汰关停不达标的落后煤电机组。从严控制、按需推动煤电项目建设。

推动煤炭绿色开发利用。继续实施煤矿安全技术改造，推进煤矿智能化发展，加强矿区瓦斯、煤矸石、煤泥等煤炭开采伴生资源综合利用，发展矿区循环经济。推进散煤治理和煤炭清洁化利用，着力提高电煤消费比重。研究完善煤炭绿色开发政策措施，推广应用绿色开采技术，进一步提高原煤入选比例，促进资源开发与生态环境保护协调发展。

提升电网安全和智能化水平。有序建设跨省跨区输电通道重点工程，合理配套送出电源，优化调度，提高通道运行效率和非化石能源发电输送占比。调整优化区域主网架建设规划，加快重点工程建设，提升电力供应保障能力。推进长三角、粤港澳大湾区、深圳社会主义先行示范区、海南自贸区（港）等区域智能电网建设。加强充电基础设施建设，提升新能源汽车充电保障能力。增强系统储备调节能力。积极推进抽水蓄能电站建设和煤电灵活性改造。加强需求侧管理，充分挖掘用户端调节潜力。完善电力系统调峰、调频等辅助服务市场机制和煤电机组深度调峰补偿机制。

提高炼油行业发展质量。研究建立炼油行业“能效领跑者”机制，促进节能技术创新及推广应用。建立健全全国炼油行业综合信息监测系统，加强数据分析评估，引导行业健康发展。着力化解炼油产能过剩风险，稳步提升产能利用率。

五、壮大清洁能源产业，推进能源结构转型

持续发展非化石能源。落实《关于2020年风电、光伏项目建设有关事项的通知》，保持风电、光伏发电合理规模和发展节奏。有序推进集中式风电、光伏和海上风电建设，加快中东部和南方地区分布式光伏、分散式风电发展。积极推进风电、光伏发电平价上网。积极稳妥发展水电，启动雅砻江、黄河上游、乌江及红水河等水电规划调整，加快龙头水库建设。安全发展核电，稳妥推进项目建设和核能综合利用等。

提高清洁能源利用水平。明确2020年各省（区、市）可再生能源电力消纳责任权重。鼓励可再生能源就近开发利用，进一步提高利用率。完善流域水电综合监测体系，开展重点流域水能利用情况预测预警。继续落实好保障核电安全消纳暂行办法，促进核电满发多发。

专栏三：水电、核电重大工程

在建水电项目：金沙江乌东德，白鹤滩，雅砻江两河口，大渡河双江口等水电站。

前期工作水电项目：金沙江旭龙等水电站。

在建核电项目：高温气冷堆示范工程，红沿河（5、6号），福清（5、6号），田湾（5、6号），防城港红沙（3、4号），漳州（1、2号），惠州（1、2号）等。

六、突出重点，强化能源民生服务保障

助力打赢脱贫攻坚战。坚决落实能源行业扶贫责任，大力支持贫困地区能源资源开发，促进资源优势尽快转化为经济发展优势。狠抓民生工程，集中力量推进“三区三州”、抵边村寨农村电网改造攻坚，确保上半年完成。加强对农村电网建设的监测评价，提高农村电力服务水平。配合国务院扶贫办做好光伏扶贫“收口”工作，开展集中式光伏扶贫电站验收评估。

稳妥推进北方地区清洁取暖。提前部署2020-2021年取暖季清洁取暖工作，统筹能源供应和配套设施建设，明确各地年度目标任务和实施进度，确保按期完成。坚持“以气定改、先立后破”，压实地方政府和企业责任，稳步推进北方重点地区“煤改气”工作。量力而行、尽力而为，因地制宜选择清洁供暖技术路线，支持发展背压式热电联产供暖，积极推动风电、地热能、生物质能技术应用。研究完善清洁取暖支持政策，建立健全长效机制。探索推动分户计量供热，加强建筑保暖和节能，营造节约用能社会新风尚。

降低社会用能成本。落实阶段性降电价政策，采取“支持性两部制电价”，降低企业用电成本。阶段性降低非居民用气成本，以基准门站价格为基础适当下浮。加快推广北京、上海等地“获得电力”典型经验，持续优化我国用电营商环境。

七、促创新补短板，培育增强新动能

加大能源技术装备短板攻关力度。加强能源技术创新平台建设，组织评选一批首台（套）重大技术装备并推进示范。加强组织协调、资源配置、政策保障和评估调整，确保各项技术装备短板“攻关有主体、落地有项目、进度可追踪”。

专栏四：科技创新重点任务

实施燃气轮机自主创新发展示范项目。

依托示范项目建设，推动火电DCS控制系统、特高压交直流套管、超超临界发电机组高温材料、大功率电力电子器件等自主创新示范应用。结合大力提升油气勘探开发力度、天然气互联互通重点工程设备保障等工作，推动深水和非常规油气、天然气长输管线和LNG接收站等领域技术装备短板攻关和示范应用。

加大储能发展力度。研究实施促进储能技术与产业发展的政策，开展储能示范项目征集与评选，积极探索储能应用于可再生能源消纳、电力辅助服务、分布式电力和微电网等技术模式和商业模式，建立健全储能标准体系和信息化平台。

推动新技术产业化发展。制定实施氢能产业发展规划，组织开展关键技术装备攻关，积极推动应用示范。继续做好“互联网+”智慧能源试点验收工作。加强国家能源研发中心日常管理和考核评价。积极探索区块链等新兴技术在能源领域的融合应用。

八、完善能源治理体系，提高治理效能

抓好能源规划编制实施。深入推进能源“十三五”规划实施，确保各项约束性指标如期完成，预期性指标保持在合理区间。按照“十四五”能源规划工作安排，结合疫情对能源供需形势的影响，尽快研究编制“十四五”综合能源规划、分领域能源规划和重点区域发展规划，做好国家规划和省级规划的统筹衔接。

深入推进能源体制机制改革。抓好山西能源革命综合改革试点和海南能源综合改革。有序推动电网企业辅业市场化改革。推动电力交易机构独立规范运行，优化电力调度、交易机构间的工作协调机制。稳步推进电力中长期、现货、辅助服务市场建设，健全清洁能源消纳市场化机制。深化输配电价改革，完善增量配电价格形成机制。健全油气管网运营机制，完善主干管网与地方管网统筹协调发展长效机制，推动管网设施公平开放。进一步推进非居民用气价格市场化。

持续推进能源法治建设。加快《能源法》立法进程。研究制修订《电力法》《煤炭法》，以及《核电管理条例》《能源监管条例》《天然气管理条例》《国家石油储备条例》等一批法律法规。推进能源普法工作，全面提升依法行政水平。

健全完善能源行业标准体系。围绕能源转型变革需求，聚焦关键技术标准，强化标准制修订及实施。加强标准化管理工作，研究组建页岩油、电力气象应用、综合能源服务等领域标准化技术组织，稳步推进标准国际化。研究推动标准化信息管理平台建设，着力提升标准化工作质量和效率。

强化能源行业和市场监管。加强对国家能源规划、政策、重大项目落实情况的监管。加大电力市场准入、电力调度、市场交易、价格成本、油气管网设施公平开放监管力度，持续优化营商环境。加大能源行政执法力度，推进能源行业信用体系建设，探索构建以信用为基础的新型能源监管机制，严肃查处违法违规行为，有效维护市场秩序。

加强电力安全生产监管。巩固“齐抓共管”既有成果，建立电力企业安全生产情况排序公布制度和典型事故分析通报制度。制定实施《电力建设工程施工安全五年行动计划》，进一步加强施工安全和工程质量监督管理。制定实施《电力安全文化建设指导意见》，推动建立教育培训体系。强化发电机组并网安全性评价，推进安全生产标准化建设。加强水电站大坝重大工程缺陷和事故隐患治理。防范化解特高压输电安全隐患，开展电力行业网络安全责任暨电力监控系统安全防护落实情况专项监管。做好重大活动保电工作，加强突发事件应对处置。

九、加强能源国际合作，拓展开放发展新空间

推进产能合作和设施联通。坚持“开放、绿色、廉洁”和“高标准、惠民生、可持续”发展理念，推动“一带一路”能源合作。深化能源基础设施互联互通。

深化技术合作与政策协调。深化高效低成本新能源发电、先进核电、清洁高效燃煤发电等先进技术合作。发挥各地区位优势，深化区域和次区域合作，加强与能源国际组织交流合作，加强联合研究，拓展对外培训交流。

防范化解重大风险。根据疫情防控和全球能源供需形势变化，加强国际能源市场分析和预测，适时研究提出应对举措，化解重大风险，维护投资整体安全。

各省（区、市）能源主管部门和有关能源企业，要依据本指导意见，结合本地区和企业的实际情况，采取有力有效措施，全力抓好各项任务落实，推动能源高质量发展，为全面建成小康社会提供稳定可靠的能源保障。

　国家能源局

　　2020年6月5日

1. 医保局：配合做好进一步提升新冠病毒检测能力有关工作 医保办发〔2020〕30 号

各省、自治区、直辖市及新疆生产建设兵团医疗保障局：

　　为贯彻落实中央应对疫情工作领导小组会议部署和《国务院应对新冠肺炎疫情联防联控机制关于做好新冠肺炎疫情常态化防控工作的指导意见》，进一步提升新冠病毒检测能力，有序引导降低偏高的检测费用，支持实现“应检尽检、愿检尽检”，助力常态化防控和复工复产复学复市，现就做好新冠病毒检测的挂网采购、价格管理和医保支付工作通知如下。

　　一、畅通试剂采购渠道

　　(一)普遍开展公开挂网采购。各地医药集中采购机构要做好新冠病毒检测试剂挂网工作，完善省级集中采购平台之间信息共享、价格联动机制。公立医疗机构应从所在省份的省级药品耗材集中采购平台阳光采购新冠病毒检测试剂。

　　(二)鼓励开展集中采购。鼓励省级和统筹地区医保部门积极探索开展新冠病毒检测试剂集中采购，选择产品质量较高、生产能力较强、供应稳定、诚信较好的企业，通过竞争促进价格回归合理水平。新冠病毒检测试剂集中采购原则上应多家中选，保证供应稳定性。对于有大规模人群检测需求的地区，应优先开展集中采购。有条件的地区积极开展集中带量采购。

　　二、完善检测项目价格政策

　　(一)检测项目不“按病立项”。原则上不区分病原体或操作步骤新设核酸、抗体检测价格项目；甲类传染病或依法按甲类管理传染病的相关检查，如风险难度大、防护要求高，可在通行价格项目基础上制定统一的加收政策。公立医疗机构针对新冠病毒开展核酸、抗体检测，可直接执行已有收费政策，无需申请新增价格项目。

　　(二)单设临时项目需体现“技耗分离”。确需单设临时项目满足公立医疗机构新冠病毒检测收费需求的，鼓励各地按“技耗分离”的方式立项。核酸、抗体检测的样本采集、处理、标记、回收、出具诊断结果，以及鼻咽拭子等消耗品应合并作为医疗服务价格项目定价；体外诊断试剂盒在医疗服务价格项目外，按“零差率”收费。

　　三、做好医保支付工作

　　要在综合考虑新型冠状病毒肺炎防控工作需要、本地区医保基金支付能力等因素的基础上，按程序将针对新冠病毒开展的核酸、抗体检测项目和相关耗材纳入省级医保诊疗项目目录，并同步确定支付条件。

　　各地医保部门要高度重视此项工作，在工作中遇到重大问题和情况，请及时向国家医疗保障局报告。

国家医疗保障局办公室

2020年6月16日

1. 交通运输部：进一步强化交通运输疫情防控措施坚决防止疫情反弹 交运明电〔2020〕202号

各省、自治区、直辖市、新疆生产建设兵团交通运输厅(局、委)：

　　当前全国新冠肺炎疫情形势总体稳定，但近日北京市发生新发地批发市场聚集性疫情，辽宁、河北、四川、浙江等省份先后出现关联病例，疫情防控形势十分严峻。北京及各地交通运输主管部门要紧密结合疫情防控新形势，牢固树立底线思维，进一步强化防控措施，严密防范疫情通过交通运输环节传播蔓延，坚决防止因局部疫情防控不力造成疫情反弹。现就有关工作通知如下：

　　一、因时因势调整北京交通运输疫情防控措施

　　(一)从严执行防疫措施。对处在高风险街道(乡镇)的北京市省际客运站及进站客车、途经高风险街道(乡镇)的公共交通线路和出租汽车按照《客运场站和交通运输工具新冠肺炎疫情分区分级防控指南(第四版)》高风险地区标准执行防控措施，对其他北京市省际客运站及进站客车、公共交通线路和出租汽车按照中风险地区标准执行防控措施。

　　(二)严格出京旅客信息核查。北京市省际客运站和进出京省际道路客运经营者，要严格落实北京市对确诊病例、疑似病例、密切接触者、无症状感染者和有发热症状人员、5月30日以来进新发地批发市场相关人员和与市场工作人员有过密切接触的人员、中高风险街道(乡镇)人员等高风险人员严禁出京的部署，认真核验出京旅客健康码和7日内核酸检测阴性证明。对健康码异常或者未持7日内核酸检测阴性证明的人员，禁止进站乘车。

　　(三)强化从业人员防疫管理。北京市相关运输经营者和客运场站经营者建立司乘人员和客运场站服务人员健康监测制度，每日登记人员健康状况，如出现可疑症状要立即暂停工作、及时就医。要按照有关规定加强对交通运输从业人员的核酸检测。

　　(四)做好城市交通保障。北京市交通委要督促指导城市公共交通经营者切实强化运力供给，优化运输组织，提高疏运能力，控制满载率，减少公共交通人员聚集，降低疫情传播风险。

　　(五)实施特殊时段进出京客票免费退票。自2020年6月19日零时起，旅客办理2020年6月18日24时前购买的进出京省际道路客运班线客票退票的，售票单位应当予以免费办理，购买人身意外伤害保险的一同办理。

　　(六)及时发布进出京客运停运信息。因疫情防控需要，进出京道路客运班线暂停运营的，相关地区交通运输主管部门、客运站应当及时向社会公布停运信息，售票单位应当免费办理退票，购买人身意外伤害保险的一同办理。

　　二、加强环京“护城河”地区交通运输疫情联防联控

　　(七)强化进京省际客运属地管理。相关地区交通运输主管部门要动态掌握进京省际客运班线、车辆和相关客运站运营情况，督促进京省际客运班线经营者严格落实疫情防控措施，严格按照批准的客运站点运行，严禁站外揽客，进京后统一进站落客测温。根据疫情防控需要北京市省际客运站暂停运营的，北京市交通委要及时告知相关地区交通运输主管部门和省际客运班线经营者。进出京省际客运班线尚未恢复运营的，暂不恢复。

　　(八)继续暂停省际旅游客运。各省级交通运输主管部门不得开放包车客运管理信息系统省际旅游客运业务备案。因人员返岗、通勤等确需发送进出京省际包车的，严格执行“点对点”运输和相关疫情防控措施。

　　(九)继续暂停出租汽车、顺风车进出京业务。北京市和天津、河北、内蒙古、辽宁、山西、山东等环京“护城河”地区交通运输主管部门要督促指导出租汽车(含巡游车、网约车)经营者和相关平台公司暂停出租汽车、顺风车进出京业务。

　　(十)严厉打击非法营运。北京市及环京“护城河”地区交通运输主管部门要增派执法力量，加强汽车客运站、城市轨道交通站周边区域等相关场所执法检查，强化安全秩序管控，严格查处非法营运行为，坚决防止旅客乘坐非法车辆进出北京。

　　三、加强物资运输保障和防疫管理

　　(十一)强化道路货运行业疫情防控。各地交通运输主管部门要按照《交通运输部关于印发〈道路货运车辆、从业人员及场站新冠肺炎疫情防控工作指南〉的通知》(交运明电〔2020〕199号)要求，做好货运车辆、场站消毒及从业人员防护等工作，降低通过道路货物运输传播新冠病毒的风险。

　　(十二)严格落实进出京道路货运从业人员封闭管理措施。北京市交通委要积极会同相关部门按照“封闭式管理、人员不接触、车辆严消毒”的总体要求，做好进出京道路货运疫情防控工作，避免交叉感染。各地交通运输主管部门要加强与相关部门的沟通对接，认真落实国务院有关疫情防控的决策部署，对短期向北京地区运送物资的司机、装卸工(包括邮政快递车辆司机、装卸工)等从业人员，在体温检测正常和封闭式管理的前提下，原则上不需采取隔离14天的措施。

　　(十三)做好北京地区生活必需品等重点物资运输保障。各地交通运输主管部门要在当地疫情防控领导机构领导下，重点围绕保障北京地区生活必需品、疫情防控物资运输需求，统筹做好物资中转调运和保通保畅工作，指导道路货运企业加强运力组织和司机调配，避免出现因运力不足、人员短缺影响北京地区生活必需品和疫情防控物资供应。

　　四、切实做好交通运输“外防输入、内防反弹”工作

　　(十四)严格做好公路水路口岸出入境运输疫情防控工作。相关地区交通运输主管部门要继续按照当地疫情防控领导机构统一部署和部有关要求，严格执行公路水路口岸“货开客关”措施，强化对入境货运车辆和驾驶员的封闭管理，以最严措施组织做好“点对点、一站式”入境人员接运工作，并组织做好相关从业人员和一线检查人员的自身防护。

　　(十五)切实做好常态化交通运输疫情防控工作。各地交通运输主管部门要坚持分区分级、精准防控，全面落实运输场站、交通运输工具、公路服务区、公路水运工程建设等领域常态化防控工作各项要求。要继续严格落实“一断三不断”的要求，做好公路水路保通保畅保运保供。要进一步完善突发公共卫生事件交通运输应急预案，加强培训演练，确保一旦出现散发病例或发生局部疫情后的防控和应急处置工作及时有效。

　　五、加强疫情防控工作督导检查

　　各地交通运输主管部门要在当地疫情防控领导机构的领导下，进一步增强做好行业疫情常态化防控工作的责任感使命感，强化落实“属地、部门、单位、个人”四方责任，加强对交通运输疫情防控措施落实情况的督促检查，对发现的问题及时整改，堵塞防控漏洞，确保各项措施落地落细。

　　铁路、民航、邮政领域有关疫情防控要求，由国家铁路局、中国民用航空局、国家邮政局分别负责部署。

交通运输部

2020年6月18日

1. 交通运输部：印发《道路货运车辆、从业人员及场站新冠肺炎疫情防控工作指南》 交运明电〔2020〕199号

各省、自治区、直辖市、新疆生产建设兵团交通运输厅（局、委）：

　　为深入贯彻落实中央应对新冠肺炎疫情工作领导小组会议精神，以及《国务院应对新型冠状病毒感染肺炎疫情联防联控机制关于做好新冠肺炎疫情常态化防控工作的指导意见》（国发明电〔2020〕14号）部署安排，切实做好道路货运行业新冠肺炎疫情防控工作，坚决遏制疫情通过道路货物运输途径传播，部制定了《道路货运车辆、从业人员及场站新冠肺炎疫情防控工作指南》（以下简称《指南》）。各地交通运输主管部门要组织开展广泛宣传，督促指导道路货运企业和货运场站经营者严格按照《指南》表1的要求，全面做好货运车辆、场站消毒及从业人员防护等工作。冷藏保鲜货物运输车辆进出中高风险区域的，经营者参照《指南》表2要求进行信息登记。遇有突发情况立即按照有关规定程序报告属地疫情防控工作机制或有关部门。

　　交通运输部

　　2020年6月17日

1. 最高法：印发《关于依法妥善审理涉新冠肺炎疫情民事案件若干问题的指导意见（三）》 法发〔2020〕20号

最高人民法院

关于依法妥善审理涉新冠肺炎疫情民事案件

若干问题的指导意见（三）

　　为依法妥善审理涉新冠肺炎疫情涉外商事海事纠纷等案件，平等保护中外当事人合法权益，营造更加稳定公平透明、可预期的法治化营商环境，依照法律、司法解释相关规定，结合审判实践经验，提出如下指导意见。

　　一、关于诉讼当事人

　　1.外国企业或者组织向人民法院提交身份证明文件、代表人参加诉讼的证明，因疫情或者疫情防控措施无法及时办理公证、认证或者相关证明手续，申请延期提交的，人民法院应当依法准许，并结合案件实际情况酌情确定延长的合理期限。

　　在我国领域内没有住所的外国人、无国籍人、外国企业和组织从我国领域外寄交或者托交的授权委托书，因疫情或者疫情防控措施无法及时办理公证、认证或者相关证明手续，申请延期提交的，人民法院依照前款规定处理。

　　二、关于诉讼证据

　　2.对于在我国领域外形成的证据，当事人以受疫情或者疫情防控措施影响无法在原定的举证期限内提供为由，申请延长举证期限的，人民法院应当要求其说明拟收集、提供证据的形式、内容、证明对象等基本信息。经审查理由成立的，应当准许，适当延长举证期限，并通知其他当事人。延长的举证期限适用于其他当事人。

　　3.对于一方当事人提供的在我国领域外形成的公文书证，因疫情或者疫情防控措施无法及时办理公证或者相关证明手续，对方当事人仅以该公文书证未办理公证或者相关证明手续为由提出异议的，人民法院可以告知其在保留对证明手续异议的前提下，对证据的关联性、证明力等发表意见。

　　经质证，上述公文书证与待证事实无关联，或者即使符合证明手续要求也无法证明待证事实的，对提供证据一方的当事人延长举证期限的申请，人民法院不予准许。

　　三、关于时效、期间

　　4.在我国领域内没有住所的当事人因疫情或者疫情防控措施不能在法定期间提出答辩状或者提起上诉，分别依据《中华人民共和国民事诉讼法》第二百六十八条、第二百六十九条的规定申请延期的，人民法院应当依法准许，并结合案件实际情况酌情确定延长的合理期限。但有证据证明当事人存在恶意拖延诉讼情形的，对其延期申请，不予准许。

　　5.根据《中华人民共和国民事诉讼法》第二百三十九条和《最高人民法院关于适用〈中华人民共和国民事诉讼法〉的解释》第五百四十七条的规定，当事人申请承认和执行外国法院作出的发生法律效力的判决、裁定或者外国仲裁裁决的期间为二年。在时效期间的最后六个月内，当事人因疫情或者疫情防控措施不能提出承认和执行申请，依据《中华人民共和国民法总则》第一百九十四条第一款第一项规定主张时效中止的，人民法院应予支持。

　　四、关于适用法律

　　6.对于与疫情相关的涉外商事海事纠纷等案件的适用法律问题，人民法院应当依照《中华人民共和国涉外民事关系法律适用法》等法律以及相关司法解释的规定，确定应当适用的法律。

　　应当适用我国法律的，关于不可抗力规则的具体适用，按照《最高人民法院关于依法妥善审理涉新冠肺炎疫情民事案件若干问题的指导意见（一）》执行。

　　应当适用域外法律的，人民法院应当准确理解该域外法中与不可抗力规则类似的成文法规定或者判例法的内容，正确适用，不能以我国法律中关于不可抗力的规定当然理解域外法的类似规定。

　　7.人民法院根据《最高人民法院关于适用〈中华人民共和国涉外民事关系法律适用法〉若干问题的解释（一）》第四条的规定，确定国际条约的适用。对于条约不调整的事项，应当通过我国法律有关冲突规范的指引，确定应当适用的法律。

　　人民法院在适用《联合国国际货物销售合同公约》时，要注意，我国已于2013年撤回了关于不受公约第11条以及公约中有关第11条内容约束的声明，仍然保留了不受公约第1条第1款（b）项约束的声明。关于某一国家是否属于公约缔约国以及该国是否已作出相应保留，可查阅联合国国际贸易法委员会官方网站刊载的公约缔约国状况予以确定。此外，根据公约第4条的规定，公约不调整合同的效力以及合同对所售货物所有权可能产生的影响。对于这两类事项，应当通过我国法律有关冲突规范的指引，确定应当适用的法律，并根据该法律作出认定。

　　当事人以受疫情或者疫情防控措施影响为由，主张部分或者全部免除合同责任的，人民法院应当依据公约第79条相关条款的规定进行审查，严格把握该条所规定的适用条件。对公约条款的解释，应当依据其用语按其上下文并参照公约的目的及宗旨所具有的通常意义，进行善意解释。同时要注意，《〈联合国国际货物销售合同公约〉判例法摘要汇编》并非公约的组成部分，审理案件过程中可以作为参考，但不能作为法律依据。

　　五、关于涉外商事案件的审理

　　8.在审理信用证纠纷案件时，人民法院应当遵循信用证的独立抽象性原则与严格相符原则。准确区分恶意不交付货物与因疫情或者疫情防控措施导致不能交付货物的情形，严格依据《最高人民法院关于审理信用证纠纷案件若干问题的规定》第十一条的规定，审查当事人以存在信用证欺诈为由，提出中止支付信用证项下款项的申请应否得到支持。

　　适用国际商会《跟单信用证统一惯例》（UCP600）的，人民法院要正确适用该惯例第36条关于银行不再进行承付或者议付的具体规定。当事人主张因疫情或者疫情防控措施导致银行营业中断的，人民法院应当依法对是否构成该条规定的不可抗力作出认定。当事人关于不可抗力及其责任另有约定的除外。

　　9.在审理独立保函纠纷案件时，人民法院应当遵循保函独立性原则与严格相符原则。依据《最高人民法院关于审理独立保函纠纷案件若干问题的规定》第十二条的规定，严格认定构成独立保函欺诈的情形，并依据该司法解释第十四条的规定，审查当事人以独立保函欺诈为由，提出中止支付独立保函项下款项的申请应否得到支持。

　　独立保函载明适用国际商会《见索即付保函统一规则》（URDG758）的，人民法院要正确适用该规则第26条因不可抗力导致独立保函或者反担保函项下的交单或者付款无法履行的规定以及相应的展期制度的规定。当事人主张因疫情或者疫情防控措施导致相关营业中断的，人民法院应当依法对是否构成该条规定的不可抗力作出认定。当事人关于不可抗力及其责任另有约定的除外。

　　六、关于运输合同案件的审理

　　10.根据《中华人民共和国合同法》第二百九十一条的规定，承运人应当按照约定的或者通常的运输路线将货物运输到约定地点。承运人提供证据证明因运输途中运输工具上发生疫情需要及时确诊、采取隔离等措施而变更运输路线，承运人已及时通知托运人，托运人主张承运人违反该条规定的义务的，人民法院不予支持。

　　承运人提供证据证明因疫情或者疫情防控，起运地或者到达地采取禁行、限行防控措施等而发生运输路线变更、装卸作业受限等导致迟延交付，并已及时通知托运人，承运人主张免除相应责任的，人民法院依法予以支持。

　　七、关于海事海商案件的审理

　　11.承运人在船舶开航前和开航当时，负有谨慎处理使船舶处于适航状态的义务。承运人未谨慎处理，导致船舶因采取消毒、熏蒸等疫情防控措施不适合运载特定货物，或者持证健康船员的数量不能达到适航要求，托运人主张船舶不适航的，人民法院依法予以支持。

　　托运人仅以船舶曾经停靠过受疫情影响的地区或者船员中有人感染新冠肺炎为由，主张船舶不适航的，人民法院不予支持。

　　12.船舶开航前，因疫情或者疫情防控措施出现以下情形，导致运输合同不能履行，承运人或者托运人请求依据《中华人民共和国海商法》第九十条的规定解除合同的，人民法院依法予以支持：（1）无法在合理期间内配备必要的船员、物料；（2）船舶无法到达装货港、目的港；（3）船舶一旦进入装货港或者目的港，无法再继续正常航行、靠泊；（4）货物被装货港或者目的港所在国家或者地区列入暂时禁止进出口的范围；（5）托运人因陆路运输受阻，无法在合理期间内将货物运至装货港码头；（6）因其他不能归责于承运人和托运人的原因致使合同不能履行的情形。

　　13.目的港具有因疫情或者疫情防控措施被限制靠泊卸货等情形，导致承运人在目的港邻近的安全港口或者地点卸货，除合同另有约定外，托运人或者收货人请求承运人承担违约责任的，人民法院不予支持。

　　承运人卸货后未就货物保管作出妥善安排并及时通知托运人或者收货人，托运人或者收货人请求承运人承担相应责任的，人民法院依法予以支持。

　　14.因疫情或者疫情防控措施导致集装箱超期使用，收货人或者托运人请求调减集装箱超期使用费的，人民法院应尽可能引导当事人协商解决。协商不成的，人民法院可以结合案件实际情况酌情予以调减，一般应以一个同类集装箱重置价格作为认定滞箱费数额的上限。

　　15.货运代理企业以托运人名义向承运人订舱后，承运人因疫情或者疫情防控措施取消航次或者变更航期，托运人主张由货运代理企业赔偿损失的，人民法院不予支持。但货运代理企业未尽到勤勉和谨慎义务，未及时就航次取消、航期变更通知托运人，或者在配合托运人处理相关后续事宜中存在过错，托运人请求货运代理企业承担相应责任的，人民法院依法予以支持。

　　16.除合同另有约定外，船舶修造企业以疫情或者疫情防控措施导致劳动力不足、设备物资交付延期，无法及时复工为由，请求延展交船期限的，人民法院可根据疫情或者疫情防控措施对船舶修造进度的影响程度，酌情予以支持。

　　因受疫情或者疫情防控措施影响，船舶延期交付导致适用新的船舶建造标准的，除合同另有约定外，当事人请求分担因此增加的成本与费用，人民法院应当综合考虑疫情或者疫情防控措施对迟延交船的影响以及当事人履行合同是否存在可归责事由等因素，酌情予以支持。

　　17.2020年1月29日《交通运输部关于统筹做好疫情防控与水路运输保障有关工作的紧急通知》规定，严禁港口经营企业以疫情防控为名随意采取禁限货运船舶靠港作业、锚地隔离14天等措施。在港口经营企业所在地的海事部门、港口管理部门没有明确要求的情况下，港口经营企业擅自以检疫隔离为由限制船舶停泊期限，船舶所有人或者经营人请求其承担赔偿责任的，人民法院依法予以支持。

　　八、关于诉讼绿色通道

　　18.在审理与疫情相关的涉外商事海事纠纷等案件中，人民法院要积极开辟诉讼绿色通道，充分运用智慧法院建设成果，坚持线上与线下服务有机结合，优化跨域诉讼服务，健全在线诉讼服务规程和操作指南，确保在线诉讼各环节合法规范、指引清晰、简便易行。

　　九、关于涉港澳台案件的审理

　　19.人民法院审理涉及香港特别行政区、澳门特别行政区和台湾地区的与疫情相关的商事海事纠纷等案件，可以参照本意见执行。

　最高人民法院

　　2020年6月8日

1. 交通部：印发《客运场站和交通运输工具新冠肺炎疫情分区分级防控指南（第四版）》 交运明电〔2020〕196号

各省、自治区、直辖市、新疆生产建设兵团交通运输厅（局、委）：

　　为深入贯彻落实中央应对新冠肺炎疫情工作领导小组会议精神和《国务院应对新型冠状病毒感染肺炎疫情联防联控机制关于做好新冠肺炎疫情常态化防控工作的指导意见》（国发明电〔2020〕14号）有关要求，针对国内生产生活秩序恢复情况，阶段性动态调整、精准优化防控措施，强化对各地常态化防控工作的指导，部修订形成了《客运场站和交通运输工具新冠肺炎疫情分区分级防控指南（第四版）》，重点调整了客运场站和交通运输工具消毒、通风、留观区设置、客运车辆空调使用等要求。请各地交通运输主管部门指导客运场站运营单位、客运企业按照最新版指南要求，认真贯彻执行。

　　铁路、民航、邮政领域场站及交通运输工具分区分级疫情防控要求，由国家铁路局、中国民用航空局、国家邮政局分别负责制修订。

交通运输部

2020年6月12日

1. 农业农村部：进一步强化动物检疫工作 农牧发〔2020〕22号

各省、自治区、直辖市及计划单列市农业农村（农牧、畜牧兽医）厅（局、委），新疆生产建设兵团畜牧兽医局：

　　按照《全国人民代表大会常务委员会关于全面禁止非法野生动物交易、革除滥食野生动物陋习、切实保障人民群众生命健康安全的决定》要求，我部近日公布了《国家畜禽遗传资源目录》。为规范做好《国家畜禽遗传资源目录》所列畜禽的检疫工作，严格监督管理，现将有关事项通知如下。

　　一、按照《畜禽遗传资源目录》明确检疫范围

　　《国家畜禽遗传资源目录》共列入33种畜禽，包括传统畜禽17种、特种畜禽16种。各地要按照《动物检疫管理办法》和动物检疫规程要求，规范做好检疫工作。对于水貂、银狐、北极狐、貉等非食用动物，我部经过充分调研论证，制定了《水貂等非食用动物检疫规程（试行）》（见附件），请各地严格贯彻执行。羊驼的产地检疫，依照《反刍动物产地检疫规程》执行，检疫对象暂定为口蹄疫、布鲁氏菌病、结核病、炭疽、小反刍兽疫。马、驴、骆驼、梅花鹿、马鹿、羊驼的屠宰检疫，依照《畜禽屠宰卫生检疫规范》（NY467-2001）执行。其他畜禽的产地检疫、屠宰检疫要按照现行规程规定严格实施。跨省调运乳用种用动物的检疫继续执行现行规程规定，暂不调整范围。

　　二、规范开展动物检疫工作

　　各地要严格动物检疫申报工作，对于畜禽收购贩运单位和个人代为申报检疫的，严格查验畜禽养殖场（户）的委托书；要严格动物检疫操作，按照检疫规程要求，认真查验相关资料和畜禽标识，规范开展临床检查和实验室检测，规范填写动物检疫证明；要坚决查处“隔山开证”、买卖动物检疫证明、开“人情证”等违法违规行为，对检疫失职渎职情况要严肃问责；鼓励有条件的地区利用信息化手段，积极探索建立从养殖到屠宰全链条的动物检疫信息化监督管理模式，不断提升动物检疫监管效能。

　　三、积极做好动物检疫新要求的培训和宣传

　　各地要及时组织基层动物检疫人员学习和掌握动物检疫新的要求，确保有关工作有序落实到位；要做好动物检疫相关法律法规的宣传解读，加大普法宣传力度，提高从事养殖、贩运、交易、屠宰等各环节生产经营者的防疫主体责任意识；要积极发挥社会监督作用，畅通各种监督举报渠道，推动形成动物检疫监管合力。

农 业 农 村 部

2020年6月8日

1. 教育部、卫健委：印发《疫情防控常态化下复学复课工作20问》

一、疫情防控常态化条件下，进一步做好学校复学复课和疫情防控的总体要求是什么？

　　教育系统要按照“确保一方净土、确保师生安全”的总目标，建立与疫情防控常态化相适应的保障机制，最大程度确保师生健康安全，有序推动大中小学全学段全面复学复课，最大限度复课，最严标准防控。一是突出“四严”，积极稳妥、有序推进学校复学复课。严格复学标准、把握复课进度，严格把好校门、确保复学有序，严格活动管控、确保校园安全，严格落实责任、确保万无一失。二是突出“四重”，做好对重点地区、重点人群的检测工作，抓住重点节点、重点事，精准施策、强化关键环节管理。三是突出“四防”，落实“防输入、防反弹、防突发、防松懈”，压实责任、织好“防护网”，筑牢“安全墙”。四是突出“四预”，建立健全“预判、预警、预防、预演”机制。

　　二、目前发布了哪些防控标准和手册？

　　教育部已印发高等学校、中小学校、幼儿园3个新冠肺炎疫情防控指南以及《教育系统应对学校突发新冠肺炎疫情处置预案》，国家卫生健康委、教育部印发大专院校、中小学校、托幼机构3个新冠肺炎疫情防控技术方案。这些指南和方案的核心要求和标准概括起来是“53421”。

　　“5”是“五个一律”，即未经学校批准学生一律不准返校、校外无关人员一律不准进校门、师生进入校门一律核验身份和检测体温、对发烧咳嗽者一律实行医学隔离观察、不服从管理者一律严肃处理。

　　“3”是开学“三不”标准，即区域疫情未达到低风险的不开学、学校疫情防控条件不具备不开学、师生管理不到位和校园公共卫生安全未有效落实不开学。

　　“4”是“四错”开学原则，即错区域、错层次、错时、错峰开学返校原则。

　　“2”是“两个咨询”，即复学复课要咨询疾控专家意见、咨询疫情联防联控机制专家指导组意见。

　　“1”是坚持“属地原则”。复学复课具体安排由当地教育部门按照地方党委和政府统一部署确定。

　　各地要压实责任，明确地方教育部门领导干部、学校书记校长、相关部门负责人和班主任、教师的责任和工作要求，指导各地各校按照防控指南、技术方案和应急处置预案落细落实各项常态化防控举措，精确划分重点人群，分类做好精准防控。

　　三、在疫情防控常态化形势下如何做好学校管理？

　　除按照疫情防控指南、技术方案和应急处置预案精准做好各项防控工作、加强应急演练外，还要重视以下几个环节：一是要关注所在区域疫情信息，主动与家长、社区及疾控中心等专业机构建立信息通报渠道，及早识别区域疫情变化，为调整防控策略提供依据。各类学校特别是中小学、幼儿园，要坚持每日“晨午晚检”、“日报告”、“零报告”制度，了解学生健康状况，家庭成员发病情况等。二是要有效切断传播途径，加强规范消毒、教室通风换气，减少人员聚集，做好个人防护，养成勤洗手、讲卫生等个人卫生习惯，保持安全社交距离，出现咳嗽等感冒症状及时戴口罩，师生随身备用口罩。三是养成健康的生活方式，包括合理的膳食、适度锻炼、保证睡眠，增强体质，提高免疫力。

　　四、各地各校首批学生复学两周后是否可以全面复学？

　　按照教育部、国家卫生健康委“最大限度返校，最严标准防控”的总体要求，在符合属地疫情防控要求、各校制定精细化防控方案、实行“一校一策”情况下，应该加快全面复学，恢复正常的教育教学秩序。但是，如学校是多校区办学，校区人口密度大、学生流动性强、疫情防控条件不达标的，不建议学校复学。

　　五、全面复学后是否需要严格执行教室、食堂、宿舍人员间隔相关标准要求？

　　全面复学后要严格执行相关标准要求。低风险地区的学校师生可不戴口罩，但在教室、食堂等人员相对密集场所要落实保持一定的社交距离等要求。

　　六、全面复学后学校是否要严格做到相对闭环管理？

　　复学前后要严格地执行封闭管理；中小学、幼儿园复学后需适当提高防控要求，需落实闭环管理；大学复学后因毕业班学生要外出应聘求职，做到单独闭环管理，并加大症状、体温检测力度，利用大数据追踪旅行史，设置专门的观察隔离区，发现异常要及时按程序处置。

　　七、全面复学后是否需要对每个师生做核酸检测？

　　不建议对低风险地区师生进行核酸检测普查。加强症状监测，早期识别、早诊断、及时隔离。对确诊为新冠肺炎学生，经治疗康复并符合出院标准，出院后建议继续进行14天的隔离管理和健康状况监测，学校要对其加强心理疏导和人文关怀。对于来自高、中风险地区的师生，或者是体温异常的师生要采取核酸检测。

　　八、全面复学后教室、食堂、图书馆、实验室、宿舍等重点场所是否仍需要戴口罩？

　　具备开学条件的学校，在校园内中小学校学生和授课老师无需戴口罩，幼儿园幼儿不建议戴口罩，在大学校园人群密集、通风不能保证的地方仍然鼓励大家戴口罩。除了在教室上课外，鼓励学生尽量去阅览室、图书馆或者人少的地方学习。食堂用餐，需要坚持实行分餐分座，减少不必要聚餐，如有必要的聚餐，一定要坚持使用公筷公勺。在宿舍休息时，可以采取通风、拉帘子等防护措施，睡觉时无需戴口罩。

　　九、如何结合常态化疫情防控工作，上好复学后“开学第一课”？

　　各地各校可结合工作实际，面向学生上好爱国主义教育课、思想政治理论课和公共卫生知识普及课；面向教师做好卫生防疫、疫情防控、应急心理辅导等工作培训；面向家长加强法律法规宣传和家庭教育指导，配合学校共同做好学生返校工作。营造校园疫情防控良好氛围，提高师生、家长传染病预防意识和防护能力。

　　十、全面复学后，体育课该怎么上？

　　教育部组织专家制定印发了《关于在常态化疫情防控下做好学校体育工作的指导意见》，对统筹推进疫情防控和体育教育教学活动、优化体育课程内容和教学方式方法、切实落实每天锻炼1小时要求、创新形式加强学校健康教育教学、适当调整各类体育考试工作方案、家校协同营造良好体育学习环境等提出了要求。各地各校要按照意见要求，认真贯彻落实。

　　十一、学校全面复学后中央空调、公共浴室能否使用？

　　复学后国内部分地区如若在夏季不开空调，可能会导致其他疾病的发生。因此，低风险地区严格按照《夏季空调运行管理与使用指引（修订版）》（联防联控机制综发〔2020〕174号）要求运行管理，学校中央空调可以使用。在人员密集的场所使用空调系统时，空调每运行2至3小时须通风换气约20至30分钟。公共浴室要加强通风，定时清洁消毒，延时长、分时段、限人数使用并减少学生公共浴室淋浴时间，加强工作人员个人防护。

　　十二、高校复学后毕业班学生外出面试、签约等如何管理？

　　毕业生就业工作是“六稳”之首，做好毕业生求职就业工作十分重要。要通过线上线下、校内校外相结合的方式进行。毕业班学生外出求职要做好个人防护，尽量做到“两点一线”，不进入疫情高、中风险地区。在符合疫情防控要求的情况下，支持用人单位采用网络招聘或者进校开展招聘，招聘过程中要避免人员聚集，在校内开阔场所、多批次、小范围开展面试工作，面试过程中做好考官和学生个人防护，缓解学生就业压力。

　　十三、如何加强学校医务室管理防止交叉感染？

　　要着力加强学校医务室管理，可参照医院的防感染措施来执行，规范工作流程，强化防控措施，防止交叉感染和医源性感染。各地教育、卫生健康部门要积极会同财政、人力资源社会保障等部门研究加强学校医务室设置、校医配备等问题。

　　十四、如何加强对学校管理人员、医护人员的防疫培训？

　　各地各校要积极配合卫生健康部门，强化对学校管理人员、医护人员的防疫知识和技能培训，切实提高防控意识和能力。现阶段可以线上培训为主，突出应急演练，适时邀请专家现场指导。

　　十五、如何建立健全医校联防联控机制？

　　要建立学校与医院、疾控等医疗机构点对点的“绿色通道”，具体责任到人。完善应急预案，开展必要的演练，及时按应急预案处置新冠肺炎确诊、无症状感染者、疑似以及核酸检测为阳性的师生，及时治疗非新冠肺炎引起发热的师生。

　　十六、如何保障学校防疫物资、场所储备充足？

　　各地各校要做好口罩、隔离服、消毒液、洗手液等物资储备，开学前至少准备可供两周使用的口罩等防控物资，并建立供应渠道，保障持续供应。必要时，可在地方党委和政府领导下，通过联防联控机制协调解决。加大对条件相对薄弱学校改善卫生基础设施和防疫物资配备支持力度。

　　十七、如何加强高校实验室等场所管理？

　　一是在学生返校前，对所有实验场所开展一次全面安全检查，重点检查危化品安全、特种设备安全、电气消防安全、废弃物安全、实验动物安全，以及应急处置器材设施检查维护等。二是对所属实验场所开展全面的卫生清洁和环境消毒工作。三是开展实验安全及疫情防控安全教育。四是制定并落实实验场所开放及运行方案。五是对实验室所有的仪器设备进行清查盘点，做好仪器设备使用登记。六是确定实验室管理人员及其职责，从严落实管控措施与安全管理。七是做好实验室个人防护用品和消毒剂等配备。

　　十八、如何做好疫情防控常态下的高校后勤工作？

　　一是落实联防联控机制的要求，并建立落实机制。二是结合本校情况，制定预案，并组织师生培训和演练。三是严格校园公共场所管理，强化公共场所通风消毒。四是统筹利用校医院、校内宾馆，按照防疫要求提前准备临时隔离观察场所，储备足量防疫必备品，做好吃、住、用等服务保障工作。五是强化食堂食品卫生安全管理，严格执行加工、售卖、储存食品安全操作规范。六是摸清开学后校园工作必需品供应底数，采取有效措施确保准备充足、供应有序、价格稳定。七是加大后勤疫情防控工作宣传引导力度，规范信息报送发布机制。

　　十九、如何做好学生的心理疏导？

　　疫情的发生对学生的学习、生活、心理都产生了影响，要关注每个学生的情况，全面掌握心理健康状况，做好心理疏导和心理干预，让他们保持积极向上的态度。一是学校要营造包容团结互助的校园氛围，抓住学生返校、毕业离校等节点积极组织开展“共抗疫情爱国力行”等系列主题教育活动，做好学生思想教育。二是要充分发挥学校辅导员、班主任、心理咨询室的作用，加强对学生心理状况的排查，进一步健全学生心理健康状况监测报告制度。三是依托心理教师并借助教育部华中师范大学心理援助热线平台以及1630余所高校心理支持热线或网络平台资源，为学生提供及时、专业的心理咨询服务，对学生心理问题做到早干预、早疏导。

　　二十、如何做好学校夏季传染病的防治？

　　夏季可能会有其他传染病的发生，因此在学校的管控过程中，要将食堂的卫生安全问题提上重要日程，对食堂餐饮服务人员的健康防护要严格标准。同时，可向学生提供一些调整体内内环境的预防方，对于易感体质、易感人群采取一些预防措施，尽量避免其受到夏季传染病的病原微生物感染。

2020.6.15

1. 文旅部：印发《恢复开展社会艺术水平考级现场考级活动疫情防控措施指南》 科教函〔2020〕4号

各省、自治区、直辖市文化和旅游厅（局），新疆生产建设兵团文化体育广电和旅游局：

　　现将《恢复开展社会艺术水平考级现场考级活动疫情防控措施指南》印发给你们，请结合实际抓好贯彻落实。

　　特此通知。

文化和旅游部科技教育司

2020年6月3日

恢复开展社会艺术水平考级现场考级活动疫情防控措施指南

　　为贯彻落实习近平总书记关于统筹推进新冠肺炎疫情防控和经济社会发展工作的重要指示精神和《国务院应对新型冠状病毒感染肺炎疫情联防联控机制关于做好新冠肺炎疫情常态化防控工作的指导意见》（国发明电〔2020〕14号）要求，有序恢复社会艺术水平考级现场考级活动，制定本指南。

　　一、总体要求

　　（一）坚持有序恢复。恢复工作按照“属地原则”，由各地文化和旅游行政部门根据属地党委、政府统一部署和当地疫情防控指挥部门要求，研判是否在疫情低风险地区启动社会艺术水平考级现场考级活动恢复工作，恢复现场考级活动时间应当安排在省级教育部门批准校外培训机构开展线下培训活动之后。疫情中高风险地区、疫情风险具有较大不确定性地区和境外输入压力较大地区，应当暂缓恢复现场考级活动。

　　（二）坚持防控为先。各地文化和旅游行政部门应当结合本地疫情防控工作的实际情况，分区分级，确定恢复社会艺术水平考级现场考级活动的具体条件和管理要求，指导社会艺术水平考级机构制定疫情防控方案和应急预案，落实落细各项防控工作，确保现场考级活动安全有序开展。

　　（三）坚持预约限流。恢复现场考级活动的机构应当严格执行人员限流限量措施，错峰安排报到和考试时间，减少人员聚集和等待。

　　二、考生和家长防护

　　（四）做好入场检测工作。考点应当配备测量体温设施设备，并安排专人值守。考生检录时必须佩戴口罩、测量体温、出示健康码或核酸检测等有效健康证明，按照考场安排表持准考证进行检录登记。考生不戴口罩或体温异常的，禁止入内。

　　（五）实施预约限流措施。考级机构应当通过不见面方式开展现场考级活动考试报名、考场安排、证书发放等工作。严格按照疫情防控要求及相关参数标准，确定日均人数上限，并实现学生和家长错峰入场。鼓励考生在保障安全前提下通过步行、自行车、私家车前往，原则上一名考生由一名家长陪同。

　　（六）建立巡查制度。考点应当安排专人做好考试现场管理，监督进入考试区的考生、考官及工作人员遵守相关防疫要求，及时对考生进行疏导、分流，保持安全社交距离。

　　（七）提供医务保障。考级机构应当有专人负责医务保障工作，应急处理考生、考官和工作人员临时出现身体不适等情况。对发热、咳嗽等疑似症状要及时向就近医疗机构反馈和沟通。

　　三、场所防控管理

　　（八）严格选择考场。考场选择应符合《社会艺术水平考级考点、考场设置及环境要求》（GB/T 36725—2018）要求，并避免在人员密集和流动性大的车站、医院、商业区等地点附近安排考场。

　　（九）降低人员密度。各专业考生考场内人均面积最低标准按照《社会艺术水平考级考点、考场设置及环境要求》（GB/T 36725—2018）规定上浮2㎡执行，且集体性考试中音乐基础知识和美术专业每个考场人数上限50人、舞蹈专业每个考场人数上限10人。考生在候考区内人均面积不低于2㎡，并保持安全距离。严格控制家长等候休息区人数，考生进入考场后，鼓励家长在户外活动，根据时间安排在规定出口处等候。

　　（十）加强消毒通风。考级机构应当在考试前一天以及考试期间对考点公共部分如地面、电梯、门把手、楼梯扶手和考场内部的桌椅、乐器、考试道具等高频接触物体，每日至少进行三次（早、中、晚）清洁消毒。建立《考点清洁消毒记录表》，记录消毒时间、责任人等信息。集体性考试相邻场次间应有充足的间隔期做好考场的换气通风。

　　（十一）配备充足防护物资。考级机构在各考点应当配备体温检测设备、口罩、免洗手消毒剂或消毒湿巾等防护物资，在考场、候考区、洗手间等区域配备洗手液、医用酒精等消毒物品，便于考生、考官和工作人员随时消毒清洁。

　　（十二）加强宣传教育。考级机构要积极通过多种形式向考生和家长宣传、培训疫情防控知识和技能，讲解现场考级活动期间的防疫措施和安排，提高考生和家长预防控制意识和应对能力。考点应通过设置提示牌、摆放宣传品、微信公众号、电子显示屏等多种方式，加强疫情防控知识科普宣传。

　　四、考官和工作人员健康管理

　　（十三）开展每日健康监测。考级机构应当在考试前14天对直接接触考生的工作人员、考官等群体进行健康监测，重点监测有无发热、咳嗽、乏力、鼻塞、流涕、咽痛、腹泻等症状，实行“日报告”“零报告”制度，对有疑似症状的禁止上岗并密切跟踪病情进展。

　　（十四）减少人员聚集。考官及工作人员应当减少不必要外出，对因参加考级活动出行和流动时，均须严格遵守所在地疫情防控相关规定，要尽量避开密集人群，避免在公共场所长时间停留。要加强考官及工作人员用餐管理，实行错峰就餐、分散用餐。

　　五、应急处置措施

　　（十五）做好疫情防控应急处置。考级机构应健全应急机制，完善应急预案，加强与卫生健康部门、疾控机构、就近定点医疗机构、社区卫生服务中心的沟通协调，接受专业指导，明确疫情防控应急措施和处置流程，严防恢复现场考级活动引发各类安全事故。在现场考级活动中如出现疑似症状或病例，考点应当立即启动应急预案，在专业机构的指导下做好现场管理，避免恐慌，配合卫生健康部门做好密切接触者排查管理和隔离措施，及时做好清洁消毒等工作并暂停考试。

1. 国资委：进一步做好服务业小微企业和个体工商户房租减免工作 国资厅财评〔2020〕158号

各中央企业：

为贯彻国务院第91次常务会议关于减轻服务业小微企业和个体工商户房租负担的有关精神，落实发展改革委等8部门印发的《关于应对新冠肺炎疫情进一步帮扶服务业小微企业和个体工商户缓解房屋租金压力的指导意见》（发改投资规〔2020〕734号，以下简称734号文件）要求，推动中央企业进一步做好房租减免工作，切实帮助服务业小微企业和个体工商户减轻经营负担，现将有关事项通知如下：

一、高度重视减租工作

服务业小微企业和个体工商户是我国社会主义市场经济的有机组成部分，在繁荣市场、促进就业、维护稳定方面发挥着重要作用。各中央企业要充分认识应对疫情冲击积极帮扶服务业小微企业和个体工商户缓解房租压力的重要意义，进一步把思想认识行动统一到党中央、国务院决策部署上来，明确职责分工，完善工作机制，落实工作要求，带头履行社会责任，主动帮扶服务业小微企业，为全社会有力应对疫情冲击，实现国民经济平稳运行积极作出贡献。

二、全面落实减免要求

各中央企业要按照734号文件要求，严格执行房屋所在地对经营出现困难的服务业小微企业和个体工商户的房租减免政策，对承租本企业房屋用于经营、出现困难的服务业小微企业和个体工商户至少免除上半年3个月房屋租金。各中央企业要抓紧组织落实，加快内部决策，对符合减免条件的小微企业和个体工商户，不得以任何理由拖延减免。上半年减免期限不足的，根据房屋所在地要求在下半年进行补足或顺延。对于转租、分租中央企业房屋的，各中央企业要通过与中间承租人加强沟通、签订合同等方式，积极协调减免租金惠及最终承租人。

三、积极争取支持政策

各中央企业要对实际减免出租人有关支持政策加强跟踪研究，主动对接有关部门和地方政府，争取支持政策落地见效。业绩考核方面，认真统计分析减免房租对企业业绩的影响并做好申报工作。财税政策方面，积极争取享受地方政府对出租人的财政补贴和税收优惠。金融支持方面，加强与金融机构协商，争取通过展期、续贷和优惠利率质押贷款支持等方式解决到期还款困难问题。中央企业所属子企业因落实减免政策导致资金紧张、难以周转的，上级企业或集团公司应给予资金支持。

四、加大监督检查力度

各中央企业要切实承担起减免房租主体责任，加强对各级子企业的跟踪督导和监督检查，及时发现问题并督促整改，对减免要求落实不到位、违规操作造成国有资产流失的企业和有关负责人进行严肃追责。要建立完善减租问题来信来访处理机制，企业集团要在官方网站主动公开受理减免房租问题投诉的联系人和联系方式，依法合规妥善处理群众反映问题。国资委将加强对各中央企业减免工作的督促指导，对有关方面反映的重大问题进行核实检查。

除734号文件和本通知有明确规定外，各中央企业应继续执行《关于支持中小微企业和个体工商户发展 积极减免经营用房租金的通知》（国资厅财评〔2020〕42号）的工作要求，并认真做好房租减免情况每月统计工作，按时报送统计表。

国资委办公厅

2020年5月20日

1. 银保监会：积极推动落实中小微企业贷款临时性延期还本付息政策

经国务院同意，2020年3月1日，银保监会与人民银行等五部委联合出台了对中小微企业贷款实施临时性延期还本付息政策，引导各银行业金融机构主动对接企业需求，通过展期、续贷等方式，对受疫情影响、暂时遇到困难的中小微企业（含小微企业主、个体工商户等）贷款给予临时性延期还本付息安排，相关工作已取得积极进展。

一是中小微企业到期贷款本息延期规模达1.3万亿元。自2020年1月25日至5月15日，各银行业金融机构已对1.28万亿元中小微企业到期贷款本金实行延期安排，涉及贷款户数75万户，共计157.8万笔。同时，各银行业金融机构已对559.9亿元到期贷款利息实行延期安排，涉及贷款户数57.9万户，对应贷款余额3.44万亿元。

二是小微企业、小微企业主和个体工商户合计有73.9万户贷款本金、57.1万户贷款利息得到延期。截至2020年5月15日，小型企业、微型企业、小微企业主到期贷款延期本金分别为5437.5亿元、1070.9亿元和1010.8亿元，涉及贷款户数合计43.2万户，占全部延期还本户数的57.6%。个体工商户到期贷款延期本金为1006.4亿元，涉及贷款户数30.7万户，延期户数在各类企业中居于首位。同时，有57.1万户小型企业、微型企业、小微企业主和个体工商户享受了贷款延期付息安排，合计延期付息金额300亿元。

三是超过40%的中小微企业到期贷款得到各种形式的接续融资支持。为切实缓解企业资金周转压力，银行业金融机构还通过还贷后再给予融资等多种方式，对中小微企业提供了1.9万亿元的再融资支持，加上前述已实行临时性延期还本安排的到期贷款，中小微企业已有超过四成的到期贷款本金享受了接续融资支持。

下一步，银保监会将深入贯彻党中央、国务院的决策部署，继续完善中小微企业贷款延期还本付息政策，引导银行业金融机构根据疫情发展趋势和中小微企业经营状况，进一步延长贷款延期还本付息期限，重点满足普惠型小微企业的贷款延期需求，为企业提供更全面、更精准的金融服务。

2020.6.4

1. 央行等8部门：进一步强化中小微企业金融服务 银发〔2020〕120号

面对新冠肺炎疫情对中小微企业造成的重大影响，金融及相关部门坚决贯彻党中央、国务院的决策部署，迅速行动，主动作为，出台了一系列措施，支持扩内需、助复产、保就业，为疫情防控、复工复产、实体经济发展提供了精准金融服务。为推动金融支持政策更好适应市场主体的需要，进一步疏通内外部传导机制，促进中小微企业（含个体工商户和小微企业主，不含地方政府融资平台，下同）融资规模明显增长、融资结构更加优化，实现“增量、降价、提质、扩面”，推动加快恢复正常生产生活秩序，支持实体经济高质量发展，提出以下意见。

一、不折不扣落实中小微企业复工复产信贷支持政策

（一）安排好中小微企业贷款延期还本付息。完善延期还本付息政策，加大对普惠小微企业延期还本付息的支持力度。银行业金融机构要加大政策落实力度，提高受惠企业占比，对于疫情前经营正常、受疫情冲击经营困难的企业，贷款期限要能延尽延。要结合企业实际，提供分期还本、利息平摊至后续还款日等差异化支持。提高响应效率、简化办理手续，鼓励通过线上办理。

（二）发挥好全国性银行带头作用。全国性银行要用好全面降准和定向降准政策，实现中小微企业贷款“量增价降”，出台细化方案，按月跟进落实。五家大型国有商业银行普惠型小微企业贷款增速高于40%。全国性银行要合理让利，确保中小微企业贷款覆盖面明显扩大，综合融资成本明显下降。

（三）用好再贷款再贴现政策。人民银行分支机构要用好再贷款再贴现政策，引导金融机构重点支持中小微企业，以及支持脱贫攻坚、春耕备耕、禽畜养殖、外贸、旅游娱乐、住宿餐饮、交通运输等行业领域。加强监督管理，确保资金发放依法合规，防止“跑冒滴漏”。中小银行要运用好再贷款再贴现资金，鼓励中小银行加大自有资金支持力度，促进加大中小微企业信贷投放，降低融资成本。

（四）落实好开发性、政策性银行专项信贷额度。开发性、政策性银行要在2020年6月底前将3500亿元专项信贷额度落实到位，以优惠利率支持中小微企业复工复产，制定本银行专项信贷额度实施方案，按月报送落实情况。

（五）加大保险保障支持力度。鼓励保险机构根据中小微企业受疫情影响程度的具体情况，提供针对性较强的相关贷款保证保险产品。鼓励保险公司区分国别风险类型，进一步提高出口信用保险覆盖面，加大出口中小微企业的风险保障。鼓励保险公司在疫情防控期间，探索创新有效的理赔方式，确保出险客户得到及时、便捷的理赔服务。

二、开展商业银行中小微企业金融服务能力提升工程

（六）提高政治站位，转变经营理念。要高度重视对受疫情影响的中小微企业等实体经济的金融支持工作，强化社会责任担当。按照金融供给侧结构性改革要求，把经营重心和信贷资源从偏好房地产、地方政府融资平台，转移到中小微企业等实体经济领域，实现信贷资源增量优化、存量重组。

（七）改进内部资源配置和政策安排。大中型商业银行要做实普惠金融事业部“五专”机制，单列小微企业、民营企业、制造业等专项信贷计划，适当下放审批权限。改革小微信贷业务条线的成本分摊和收益分享机制，全国性商业银行内部转移定价优惠力度要不低于50个基点，中小银行可结合自身实际，实施内部转移定价优惠或经济利润补贴。

（八）完善内部绩效考核评价。商业银行要提升普惠金融在分支行和领导班子绩效考核中的权重，将普惠金融在分支行综合绩效考核中的权重提升至10%以上。要降低小微金融利润考核权重，增加小微企业客户服务情况考核权重。改进贷款尽职免责内部认定标准和流程，如无明显证据表明失职的均认定为尽职，逐步提高小微信贷从业人员免责比例，激发其开展小微信贷业务的积极性。

（九）大幅增加小微企业信用贷款、首贷、无还本续贷。商业银行要优化风险评估机制，注重审核第一还款来源，减少对抵押担保的依赖。在风险可控的前提下，力争实现新发放信用贷款占比显著提高。督促商业银行提高首次从银行体系获得贷款的户数。允许将符合条件的小微企业续贷贷款纳入正常类贷款，鼓励商业银行加大中长期贷款投放力度，力争2020年小微企业续贷比例高于上年。

（十）运用金融科技手段赋能小微企业金融服务。鼓励商业银行运用大数据、云计算等技术建立风险定价和管控模型，改造信贷审批发放流程。深入挖掘整合银行内部小微企业客户信用信息，加强与征信、税务、市场监管等外部信用信息平台的对接，提高客户识别和信贷投放能力。打通企业融资“最后一公里”堵点，切实满足中小微企业融资需求。

三、改革完善外部政策环境和激励约束机制

（十一）强化货币政策逆周期调节和结构调整功能。实施稳健的货币政策，综合运用公开市场操作、中期借贷便利等货币政策工具，保持银行体系流动性合理充裕，引导金融机构加大对中小微企业的信贷支持力度。

（十二）发挥贷款市场报价利率改革作用。将主要银行贷款利率与贷款市场报价利率的点差纳入宏观审慎评估考核，密切监测中小银行贷款点差变化。督促银行业金融机构将贷款市场报价利率内嵌到内部定价和传导相关环节，疏通银行内部利率传导机制。按照市场化、法治化原则，有序推进存量浮动利率贷款定价基准转换。

（十三）优化监管政策外部激励。推动修订商业银行法，研究修改商业银行贷款应当提供担保的规定，便利小微企业获得信贷。开展商业银行小微企业金融服务监管评价，继续实施普惠型小微企业贷款增速和户数“两增”要求。进一步放宽普惠型小微企业不良贷款容忍度。

（十四）研究完善金融企业绩效评价制度。修改完善金融企业绩效评价管理办法，弱化国有金融企业绩效考核中对利润增长的要求。将金融机构绩效考核与普惠型小微企业贷款情况挂钩。引导金融企业更好地落实国家宏观战略、服务实体经济，加大对小微企业融资支持力度。鼓励期货公司风险管理子公司通过场外期权、仓单服务等方式，为小微企业提供更加优质、便捷的风险管理服务。

（十五）更好落实财税政策优惠措施。加大小微企业金融服务税收优惠和奖补措施的宣传力度，力争做到应享尽享。加强普惠金融发展专项资金保障，做好财政支持小微企业金融服务综合改革试点。

（十六）发挥地方政府性融资担保机构作用。建立政府性融资担保考核评价体系，突出其准公共产品属性和政策性，逐步取消盈利考核要求，重点考核其支小支农成效（包括新增户数、金额、占比、费率水平等）、降低反担保要求、及时履行代偿责任和首次贷款支持率等指标，落实考核结果与资金补充、风险补偿、薪酬待遇等直接挂钩的激励约束机制。逐步提高担保放大倍数，并将政府性融资担保和再担保机构平均担保费率降至1%以下。

（十七）推动国家融资担保基金加快运作。2020年力争新增再担保业务规模4000亿元。与银行业金融机构开展批量担保贷款业务合作，提高批量合作业务中风险责任分担比例至30%。对合作机构单户100万元及以下担保业务免收再担保费，2020年全年对100万元以上担保业务减半收取再担保费。

（十八）清理规范不合理和违规融资收费。对银行业金融机构小微贷款中违规收费及借贷搭售、转嫁成本、存贷挂钩等变相抬高中小微企业实际融资成本的乱象加强监管检查，从严问责处罚。

四、发挥多层次资本市场融资支持作用

（十九）加大债券市场融资支持力度。引导公司信用类债券净融资比上年多增1万亿元，支持大型企业更多发债融资，释放信贷资源用于支持小微企业贷款。优化小微企业专项金融债券审批流程，疏通审批堵点，加强后续管理，2020年支持金融机构发行小微企业专项金融债券3000亿元。进一步发挥民营企业债券融资工具支持作用。推动信用风险缓释工具和信用保护工具发展，推广非公开发行可转换公司债融资工具。

（二十）提升中小微企业使用商业汇票融资效率。对于确需延时支付中小微企业货款的，促进企业使用更有利于保护中小微企业合法权益的商业汇票结算，推动供应链信息平台与商业汇票基础设施互联，加快商业汇票产品规范创新，提升中小微企业应收账款融资效率。

（二十一）支持优质中小微企业上市或挂牌融资。支持符合条件的中小企业在主板、科创板、中小板、创业板上市融资，加快推进创业板改革并试点注册制。优化新三板发行融资制度，引入向不特定合格投资者公开发行机制，取消定向发行单次融资新增股东35人限制，允许内部小额融资实施自办发行，降低企业融资成本。设立精选层，建立转板上市制度，允许在精选层挂牌一年并符合相关条件的企业直接转板上市，打通挂牌公司持续发展壮大的上升通道。对基础层、创新层、精选层建立差异化的投资者适当性标准，引入公募基金等长期资金，优化投资者结构。

（二十二）引导私募股权投资和创业投资投早投小。修订《私募投资基金监督管理暂行办法》（中国证券监督管理委员会令第105号），强化对创业投资基金的差异化监管和自律。制定《创业投资企业标准》，引导和鼓励创业投资企业和天使投资专注投资中小微企业创新创造企业。鼓励资管产品加大对创业投资的支持力度，并逐步提高股权投资类资管产品比例，完善银行、保险等金融机构与创业投资企业的投贷联动、投保联动机制，加强创业投资企业与金融机构的市场化合作。推动完善保险资金投资创业投资基金政策。

（二十三）推进区域性股权市场创新试点。选择具备条件的区域性股权市场开展制度和业务创新试点，推动修改区域性股权市场交易制度、融资产品、公司治理有关政策规定。推动有关部门和地方政府加大政策扶持力度，将区域性股权市场作为地方中小微企业扶持政策措施综合运用平台。加强与征信、税务、市场监管、地方信用平台等对接，鼓励商业银行、证券公司、私募股权投资机构等参与，推动商业银行提供相关金融服务。

五、加强中小微企业信用体系建设

（二十四）加大对地方征信平台和中小企业融资综合信用服务平台建设指导力度。研究制定相关数据目录、运行管理等标准，推动地方政府充分利用现有的信用信息平台，建立地方征信平台和中小企业融资综合信用服务平台，支持有条件的地区设立市场化征信机构运维地方平台。以地方服务平台为基础，加快实现互联互通，服务区域经济一体化发展。探索建立制造业单项冠军、专精特新“小巨人”企业、专精特新中小企业以及纳入产业部门先进制造业集群和工业企业技术改造升级导向计划等优质中小微企业信息库，搭建产融合作平台，加强信息共享和比对，促进金融机构与中小微企业对接，提供高质量融资服务。完善和推广“信易贷”模式。

（二十五）建立动产和权利担保统一登记公示系统。推动动产和权利担保登记改革，建立统一的动产和权利担保登记公示系统，逐步实现市场主体在一个平台上办理动产和权利担保登记。

六、优化地方融资环境

（二十六）建立健全贷款风险奖补机制。有条件的地方政府可因地制宜建立风险补偿“资金池”，提供中小微企业贷款贴息和奖励、政府性融资担保机构资本补充等，以出资额为限承担有限责任。完善风险补偿金管理制度，合理设置托管对象、补偿条件，提高风险补偿金使用效率。

（二十七）支持对中小微企业开展供应链金融服务。支持产融合作，推动全产业链金融服务，鼓励发展订单、仓单、存货、应收账款融资等供应链金融产品，发挥应收账款融资服务平台作用，促进中小微企业2020年应收账款融资8000亿元。加强金融、财政、工信、国资等部门政策联动，加快推动核心企业、财政部门与应收账款融资服务平台完成系统对接，力争实现国有商业银行、主要股份制商业银行全部接入应收账款融资服务平台。

（二十八）推动地方政府深化放管服改革。推动地方政府夯实风险分担、信息共享、账款清欠等主体责任，继续组织清理拖欠民营企业、中小微企业账款，督促政府部门和大型企业依法依规及时支付各类应付未付账款。支持有条件的地方探索建立续贷中心、首次贷款中心、确权中心等平台，提供便民利企服务。继续清理地方政府部门、中介机构在中小微企业融资环节不合理和违规收费。

七、强化组织实施

（二十九）加强组织推动。人民银行分支机构、银保监会派出机构可通过建立专项小组等形式，加强与当地发展改革、财税、工信、商务、国资等部门的联动，从强化内部激励、加强首贷户支持、改进服务效率、降低融资成本、强化银企对接、优化融资环境等方面，因地制宜开展商业银行中小微企业金融服务能力提升专项行动。

（三十）完善监测评价。探索建立科学客观的全国性中小微企业融资状况调查统计制度和评价体系，开发中小微企业金融条件指数，适时向社会发布。人民银行副省级城市中心支行以上分支机构会同各银保监局探索建立地市级和县级中小微金融区域环境评价体系，重点评价辖区内金融服务中小微企业水平、融资担保、政府部门信息公开和共享、账款清欠等，并视情将金融机构和市县政府评价结果告知金融机构上级部门和副省级以上地方政府，营造良好金融生态环境。

中国人民银行

银保监会

发展改革委

工业和信息化部

财 政 部

市场监管总局

证 监 会

外 汇 局

2020年5月26日

1. 民政部等5部门：扎实做好疫情防控常态化背景下残疾人基本民生保障工作

各省、自治区、直辖市残联、民政、财政、人力资源社会保障厅（局）、扶贫办（局），新疆生产建设兵团残联、民政、财政、人力资源社会保障局、扶贫办：

我国新冠肺炎疫情防控工作已从应急状态转为常态化。为贯彻习近平总书记统筹推进新冠肺炎疫情防控和经济社会发展重要讲话精神，落实党中央、国务院关于疫情防控常态化下的一系列决策部署，扎实做好残疾人基本民生保障工作，现提出如下指导意见。

一、充分认识做好残疾人基本民生保障工作重要性

习近平总书记指出：“全面建成小康社会，残疾人一个也不能少”。2020年是全面建成小康社会和脱贫攻坚决战决胜之年，残疾人群体能否如期摆脱贫困，事关全面建成小康社会和打赢脱贫攻坚战全局。新冠肺炎疫情发生以来，各地深入贯彻落实党中央、国务院关于做好困难群众基本民生保障、稳就业和脱贫攻坚的一系列决策部署，保障残疾人生产生活，但作为弱势群体，残疾人面对疫情冲击时较其他人群更为脆弱，受疫情影响更大，部分残疾人家庭生活水平明显下滑，残疾人就业和脱贫压力进一步加大。各地各部门要充分认识做好疫情防控常态化背景下残疾人基本民生保障工作的重要性，结合本地实际，进一步加大工作力度，采取更加有力的措施，扎实做好残疾人基本民生保障各项工作。

二、采取切实措施保障残疾人基本民生

（一）坚持应保尽保和分类施策

各地要密切关注、准确掌握受疫情影响残疾人的基本生活情况，对于符合条件的要尽快落实低保、临时救助、价格临时补贴以及困难残疾人生活补贴、重度残疾人护理补贴等各项生活保障措施。对因疫情影响无法经营、就业，收入下降导致基本生活困难，同时又不符合社会救助条件的困难残疾人，要甄别不同情况，做到一户一策、一事一议，给予及时帮扶。对因监护缺失或托养照护服务机构暂时无法开放导致重度失能残疾人家庭照护困难的，要协调村（社区）安排人员上门探视，并提供监护照料。有条件的地区可阶段性适当提高相关补贴标准，适当增加对特殊困难残疾人家庭的生活补助，对残疾人供养、托养、照护等机构恢复服务给予一定的支持。

（二）千方百计稳定残疾人就业

各地要主动协商辖区内安置残疾人就业的各类企业，努力使残疾人职工不因疫情影响而被裁员。对经营确有困难的，鼓励企业通过协商调整薪酬、缩短工时等方式，保留与残疾人职工的劳动关系。要主动帮助失业残疾人做好失业登记，申领失业保险金、失业补助金。主动协助残疾人集中就业企业、残疾人开办企业、残疾人个体工商户、盲人按摩机构、辅助性就业机构以及居家就业、灵活就业的残疾人，落实国家和地方失业保险返还、税收减免、担保贷款贴息、就业补贴、社会保险费减免或缓缴等各项就业帮扶政策。要主动抓好线上线下残疾人就业服务，充分发挥全国和省级残疾人就业创业网络服务平台作用，为用人单位招用残疾人和残疾人求职牵线搭桥。要通过“一对一”帮扶的形式，重点做好应届高校残疾毕业生就业服务工作。要认真落实发展改革委等部门《关于印发〈关于完善残疾人就业保障金制度更好促进残疾人就业的总体方案〉通知》（发改价格规【2019】2015号）要求，进一步加强残疾人就业资金保障，完善残疾人就业各项支持政策。疫情防控期间，各地可结合实际进一步研究出台支持残疾人就业和保障残疾人生活的政策措施。对租用私有产权不能减免房租等原因经营确有困难的盲人按摩机构、残疾人集中就业单位、残疾人个体工商户等，可按照发展改革委等部门《关于应对新冠肺炎疫情进一步帮扶服务业小微企业和个体工商户缓解房屋租金压力的指导意见》（发改投资规【2020】734号）规定，给予阶段性房租补贴；对疫情期间超比例安排残疾人就业的用人单位，可加大奖励力度。

（三）加大对农村贫困残疾人帮扶力度

各地要准确掌握疫情对建档立卡贫困残疾人生产生活造成的实际困难和问题，做好低保、临时救助、监护照料、生产增收等相关兜底保障政策和帮扶措施的落实，确保贫困残疾人家庭稳定持续脱贫。各地可结合实际，对受疫情影响较大的符合条件的种植、养殖贫困残疾人家庭给予农业保险投保费用补贴；对受疫情影响较重的残疾人扶贫基地、残疾人农业生产合作社，按规定给予资金扶持；对残疾人开展免费职业技能培训，贫困残疾人在培训期间可按规定给予生活费补贴；对残疾人返贫监测户、脱贫边缘户等农村困难残疾人家庭，采取针对性帮扶措施，巩固脱贫，减少返贫。

三、强化部门协作和组织实施

残疾人基本民生保障工作涉及面广，需要各地各部门完善工作机制，明确责任分工，共同推动实施。各地相关部门要在本地党委、政府统一领导下，在确保疫情防控到位前提下，根据各自职责，同向发力，结合残疾人实际，围绕残疾人需求，细化实化政策措施，密切配合，形成工作合力。地方各级残联组织要切实承担起牵头组织责任，加强与各有关部门的密切协作，依托二代残疾人证数据库，做好各类别残疾人状况摸底排查工作，准确掌握残疾人信息，及时反映残疾人需求，为精准施策奠定基础；要重点督促政策措施落地和资金保障落实。财政部门要结合本地实际，充分发挥残疾人就业保障金制度作用，并合理安排资金，支持做好残疾人基本生活保障、稳定残疾人就业、贫困残疾人脱贫攻坚等各项工作。民政部门要将符合条件的残疾人及其家庭纳入相应的社会救助和福利补贴范围，采取多种措施保障困难残疾群众基本生活，充分发挥临时救助的兜底作用。人力资源社会保障部门在组织开展线上线下职业技能培训和各项就业服务活动时，要将残疾人列入服务对象；要将符合就业困难人员条件的残疾人纳入就业援助范围，落实就业帮扶政策，对其中特别困难的，利用公益性岗位托底安置。扶贫部门要认真研究因疫情给贫困残疾人脱贫工作带来的新情况新问题，切实将贫困残疾人纳入帮扶。

中国残联 民政部 财政部

人力资源和社会保障部 国务院扶贫办

2020年5月22日

1. 财政部、民航局：对民航运输企业在疫情防控期间稳定和提升国际货运能力实施资金支持政策

各省、自治区、直辖市、计划单列市财政厅(局)，新疆生产建设兵团财政局，民航各地区管理局，各运输航空公司：

为积极应对新冠肺炎疫情对民航业影响，稳定和提升国际航空货运能力，保障全球产业链、供应链畅通运转，在疫情防控期间，中央财政安排资金对中外航空运输企业予以支持。现将有关事项通知如下：

一、支持对象

中央财政在疫情防控期间，对按照经中国民航适航审定部门批准的设计方案实施的航空器客舱内装货改装项目(以下简称客舱内装货改装项目)，以及对中外航空公司从2020年4月1日起使用客运航权执飞往返我国内航点(不含港澳台地区)与国外航点间的不载客国际货运航班(以下简称不载客国际货运航班)给予资金支持。

二、支持标准

(一)客舱内装货改装项目。

对航空公司疫情防控期间实施客舱内装货改装项目发生的费用给予补助。补助标准按照改造成本的80%予以补助，按飞机类型分成两档：单通道飞机每架最高补助80万元，双通道飞机每架最高补助145万元。具体补助金额根据民航局核定的实际改造成本确定。

(二)不载客国际货运航班。

1。对疫情防控期间执飞的不载客国际货运航班给予奖励，奖励标准按航班飞行里程和最大起飞全重分为八档，具体标准为：

2。奖励金额按照疫情防控期间航空公司实际执行不载客国际货运航班的航班数量和本通知规定的标准进行核定。

三、申报程序

(一)中国航空集团有限公司、中国东方航空集团有限公司、中国南方航空集团有限公司(以下简称三大航)及外国航空公司每月定期向民航局、财政部报送资金申请文件和相关证明材料。其他航空公司向企业注册所在地的民航地区管理局和省级财政部门报送资金申请和相关证明材料，经民航地区管理局和省级财政部门审核后报至民航局、财政部。

(二)民航局根据有关数据，对航空公司申请文件及相关材料进行审核，审核结果报送财政部。

(三)财政部根据民航局审核情况和相关标准向有关企业和地方拨付资金，其中：三大航的资金由财政部直接拨付；其他国内航空公司通过中央对地方转移支付方式下达，由地方财政部门负责拨付；外国航空公司的资金纳入民航局部门预算，由民航局负责转拨。资金支付按照国库集中支付有关规定执行。

(四)各航空公司应对申报材料的真实性和准确性负责，任意单位不得截留、挪用支持资金。审核中发现虚报、瞒报的，将取消公司申请资格；对于违反国家法律、行政法规和有关规定的单位和个人，将严格按照《中华人民共和国预算法》《财政违法行为处罚处分条例》等予以处理。

四、其他事项

(一)港澳台地区航线航班参照执行。

(二)政策执行期限为2020年4月1日至2020年6月30日

2020.7.27

1. 教育部：常态化疫情防控下做好学校体育工作 教体艺厅函〔2020〕12号

各省、自治区、直辖市教育厅（教委），新疆生产建设兵团教育局，部属各高等学校、部省合建各高等学校：

　　当前，部分地区的大中小学已陆续复学复课。为贯彻落实《国务院应对新型冠状病毒感染肺炎疫情联防联控机制关于做好新冠肺炎疫情常态化防控工作的指导意见》（国发明电〔2020〕14号）精神，做好常态化疫情防控下学校体育工作，提出以下意见。

　　一、充分认识常态化疫情防控下做好学校体育工作的重要意义。新时代学校体育工作的重要使命和目标是帮助学生在体育锻炼中享受乐趣、增强体质、健全人格、锤炼意志，培养德智体美劳全面发展的社会主义建设者和接班人。在常态化疫情防控下，积极有序开展学校体育工作，对树立健康第一理念，增强学生体质、提高免疫力、疏导心理焦虑，实现体育与健康教育深度融合，培养学生良好体育卫生习惯，保障学生生命安全，维护教育系统安全稳定具有重要意义。各地各校要充分发挥体育在抗击疫情中的重要作用，在常态化疫情防控下切实做好学校体育工作。

　　二、综合研判学校体育工作面临的风险。经过较长期的居家学习，学生体质、体力和运动技能有所下降。此外，夏季到来气温逐渐升高，这给复学学校开展体育工作带来风险、给学生参加体育活动带来影响和限制。各地要高度重视疫情防控形势变化，对学校开展体育工作作出综合研判，纾解学生参加体育活动的迫切心情和急切愿望，围绕课程内容设置、教学计划调整、课堂组织变化、场地器材设施使用、分时错峰活动、应急安全防范等方面制定切实可行的工作方案，积极稳妥设计和安排好体育教育教学活动。

　　三、统筹推进疫情防控和体育教育教学活动。根据本地疫情防控总体要求，要为师生配备好参加体育活动必要的防疫物资，在课前课后及时对体育场地、器材设施进行适度消毒防护，合理安排使用运动器材，避免交叉使用。师生体育活动应安排在户外或通风较好的体育场馆进行，一般保持1.5米间距，或对间隔距离进行最大化安排，尽量减少接触。不得佩戴N95口罩进行体育运动。低风险地区学生在校参加体育活动时不需戴口罩。体育课前教师应带领学生充分做好热身运动，课中要关注学生的机体和情绪变化，合理调控运动强度和运动密度，注意学生学练动向，防止扎堆聚集运动，要提醒学生课后及时休整。

　　四、优化体育课程内容和教学方式方法。要对延期开学期间学生居家学习体育情况进行摸底和诊断，在评估的基础上确定教学起点，加强居家学习和复课后体育教学的衔接。体育课程内容要根据学生实际在教学计划中作出调整，复课初期可以适当降低课程标准规定的运动强度及密度要求，以中等或中上等运动强度为宜。要多安排非身体接触性的体能练习和以发展心肺功能为主的单人项目，如跳绳、踢毽、慢跑、武术、健身操等，并根据学生体能的恢复情况循序渐进提高学习强度和难度。有条件的学校可为班级或学生制定“一班一案”“一生一策”的教学计划。

　　五、切实落实每天锻炼一小时要求。在确保师生安全和身体健康前提下，学校要开齐开足体育课，条件不具备的学校，要错时分段上好体育课。要充分利用课间休息时间，让学生多到户外参加个人体育活动，通过学生“少吃多餐”式的体育活动安排，努力保证学生每天一小时体育锻炼。要根据疫情防控要求调整大课间形式和内容，场地空间不足的学校可分时段、分区域、分批次开展大课间活动，鼓励安排以单人自主完成的练习动作。复学初期，不提倡组织统一距离、统一速度的集体跑步。根据有关要求，当前暂停学校大型聚集性活动和运动会，鼓励学校开展网络远程学生体育“云”竞赛和“云”上运动会。

　　六、创新形式加强学校健康教育教学。学校要加强健康教育教学和实践，做好健康教育科普工作，引导学生掌握健康教育知识，树立健康第一理念，养成健康生活方式。广大体育教师要增强健康教育的意识，加强自身学习，创新健康教育的形式，利用多种渠道传播健康的理念与知识，促进学生养成健康习惯。

　　七、适当调整各类体育考试工作方案。各地要根据实际，科学研制并及时发布初中升学体育考试、体育教育专业高考运动技能测试、运动训练专业和武术民族传统体育专业单招单考、普通高校高水平运动队招生等工作方案。可适当调整有关体育考试内容，并精心设计和组织考场，确保学生安全有序参加考试。在确保安全的情况下，学校可为参加体育考试的学生和学校运动代表队制定专门的运动训练计划，增强学生体能，提高学生运动水平。

　　八、家校协同营造良好体育学习环境。学校、教师要积极主动与学生家长沟通，布置体育家庭作业，动员家长对学生体育锻炼进行经常指导和监督，引导学生在常态化疫情防控下积极参加体育锻炼。家长要密切关注学生的身体和心理健康，及时向学校、教师反馈学生在家体育锻炼情况、体质健康变化情况等，为学生积极营造家校协同共进的良好体育学习环境。

　　九、加强组织领导压实主体责任。各地教育部门要始终把师生生命安全和身体健康放在第一位，强化学校疫情防控的主体责任，及时研究制定本地本校体育复学复课工作方案，积极指导、督促和检查学校的体育教学活动，确保万无一失。各地各校要把疫情防控、健康教育、安全教育和体育教育有机结合起来，制定应急预案，加强应急演练，积极稳妥推进学校体育工作。

教育部应对新冠肺炎疫情工作领导小组办公室

（教育部办公厅代章）

2020年5月12日

1. 最高法：印发《关于依法妥善审理涉新冠肺炎疫情民事案件若干问题的指导意见（二）》 法发〔2020〕17号

各省、自治区、直辖市高级人民法院，解放军军事法院，新疆维吾尔自治区高级人民法院生产建设兵团分院：

　　现将《最高人民法院关于依法妥善审理涉新冠肺炎疫情民事案件若干问题的指导意见（二）》印发给你们，请认真贯彻执行。

最高人民法院

2020年5月15日

最高人民法院

关于依法妥善审理涉新冠肺炎疫情民事案件若干问题的指导意见（二）

为进一步贯彻落实党中央关于统筹推进新冠肺炎疫情防控和经济社会发展工作部署，扎实做好“六稳”工作，落实“六保”任务，指导各级人民法院依法妥善审理涉新冠肺炎疫情合同、金融、破产等民事案件，提出如下指导意见。

　　一、关于合同案件的审理

　　1.疫情或者疫情防控措施导致当事人不能按照约定的期限履行买卖合同或者履行成本增加，继续履行不影响合同目的实现，当事人请求解除合同的，人民法院不予支持。

　　疫情或者疫情防控措施导致出卖人不能按照约定的期限完成订单或者交付货物，继续履行不能实现买受人的合同目的，买受人请求解除合同，返还已经支付的预付款或者定金的，人民法院应予支持；买受人请求出卖人承担违约责任的，人民法院不予支持。

　　2.买卖合同能够继续履行，但疫情或者疫情防控措施导致人工、原材料、物流等履约成本显著增加，或者导致产品大幅降价，继续履行合同对一方当事人明显不公平，受不利影响的当事人请求调整价款的，人民法院应当结合案件的实际情况，根据公平原则调整价款。疫情或者疫情防控措施导致出卖人不能按照约定的期限交货，或者导致买受人不能按照约定的期限付款，当事人请求变更履行期限的，人民法院应当结合案件的实际情况，根据公平原则变更履行期限。

　　已经通过调整价款、变更履行期限等方式变更合同，当事人请求对方承担违约责任的，人民法院不予支持。

　　3.出卖人与买受人订立防疫物资买卖合同后，将防疫物资高价转卖他人致使合同不能履行，买受人请求将出卖人所得利润作为损失赔偿数额的，人民法院应予支持。因政府依法调用或者临时征用防疫物资，致使出卖人不能履行买卖合同，买受人请求出卖人承担违约责任的，人民法院不予支持。

　　4.疫情或者疫情防控措施导致出卖人不能按照商品房买卖合同约定的期限交付房屋，或者导致买受人不能按照约定的期限支付购房款，当事人请求解除合同，由对方当事人承担违约责任的，人民法院不予支持。但是，当事人请求变更履行期限的，人民法院应当结合案件的实际情况，根据公平原则进行变更。

　　5.承租房屋用于经营，疫情或者疫情防控措施导致承租人资金周转困难或者营业收入明显减少，出租人以承租人没有按照约定的期限支付租金为由请求解除租赁合同，由承租人承担违约责任的，人民法院不予支持。

　　为展览、会议、庙会等特定目的而预订的临时场地租赁合同，疫情或者疫情防控措施导致该活动取消，承租人请求解除租赁合同，返还预付款或者定金的，人民法院应予支持。

　　6.承租国有企业房屋以及政府部门、高校、研究院所等行政事业单位房屋用于经营，受疫情或者疫情防控措施影响出现经营困难的服务业小微企业、个体工商户等承租人，请求出租人按照国家有关政策免除一定期限内的租金的，人民法院应予支持。

　　承租非国有房屋用于经营，疫情或者疫情防控措施导致承租人没有营业收入或者营业收入明显减少，继续按照原租赁合同支付租金对其明显不公平，承租人请求减免租金、延长租期或者延期支付租金的，人民法院可以引导当事人参照有关租金减免的政策进行调解；调解不成的，应当结合案件的实际情况，根据公平原则变更合同。

　　7.疫情或者疫情防控措施导致承包方未能按照约定的工期完成施工，发包方请求承包方承担违约责任的，人民法院不予支持；承包方请求延长工期的，人民法院应当视疫情或者疫情防控措施对合同履行的影响程度酌情予以支持。

　　疫情或者疫情防控措施导致人工、建材等成本大幅上涨，或者使承包方遭受人工费、设备租赁费等损失，继续履行合同对承包方明显不公平，承包方请求调整价款的，人民法院应当结合案件的实际情况，根据公平原则进行调整。

　　8.当事人订立的线下培训合同，受疫情或者疫情防控措施影响不能进行线下培训，能够通过线上培训、变更培训期限等方式实现合同目的，接受培训方请求解除的，人民法院不予支持；当事人请求通过线上培训、变更培训期限、调整培训费用等方式继续履行合同的，人民法院应当结合案件的实际情况，根据公平原则变更合同。

　　受疫情或者疫情防控措施影响不能进行线下培训，通过线上培训方式不能实现合同目的，或者案件实际情况表明不宜进行线上培训，接受培训方请求解除合同的，人民法院应予支持。具有时限性要求的培训合同，变更培训期限不能实现合同目的，接受培训方请求解除合同的，人民法院应予支持。培训合同解除后，已经预交的培训费，应当根据接受培训的课时等情况全部或者部分予以返还。

　　9.限制民事行为能力人未经其监护人同意，参与网络付费游戏或者网络直播平台“打赏”等方式支出与其年龄、智力不相适应的款项，监护人请求网络服务提供者返还该款项的，人民法院应予支持。

　　二、关于金融案件的审理

　　10.对于受疫情或者疫情防控措施影响较大的行业，以及具有发展前景但受疫情或者疫情防控措施影响暂遇困难的企业特别是中小微企业所涉金融借款纠纷，人民法院在审理中要充分考虑中国人民银行等五部门发布的《关于进一步强化金融支持防控新型冠状病毒感染肺炎疫情的通知》等系列金融支持政策：对金融机构违反金融支持政策提出的借款提前到期、单方解除合同等诉讼主张，人民法院不予支持；对金融机构收取的利息以及以咨询费、担保费等其他费用为名收取的变相利息，要严格依据国家再贷款再贴现等专项信贷优惠利率政策的规定，对超出部分不予支持；对因感染新冠肺炎住院治疗或者隔离人员、疫情防控需要隔离观察人员、参加疫情防控工作人员以及受疫情或者疫情防控措施影响暂时失去收入来源的人员所涉住房按揭、信用卡等个人还贷纠纷，人民法院应当结合案件的实际情况，根据公平原则变更还款期限。

　　11.防疫物资生产经营企业以其生产设备、原材料、半成品、产品等动产设定浮动抵押，抵押权人依照《中华人民共和国民事诉讼法》第一百九十六条的规定申请实现担保物权的，人民法院受理申请后，被申请人或者利害关系人能够证明实现抵押权将危及企业防疫物资生产经营的，可待疫情或者疫情防控措施影响因素消除后再行处理。

　　12.对于因疫情防控期间证券市场价格波动引发的股票质押和融资融券纠纷，应当区分不同情形处理：对于债权人为证券公司的场内股票质押和融资融券纠纷，人民法院可以参照中国证监会发布的有关政策，引导证券公司按照政策与不同客户群体协商解决纠纷；协商不成的，对于客户要求证券公司就违规强行平仓导致损失扩大部分承担赔偿责任的诉讼请求，依法予以支持。对于债权人为其他金融机构的场外股票质押纠纷，人民法院应当充分考虑股票质权实现对上市公司正常经营的影响，加强政策引导和各方利益协调，努力降低对证券市场的影响。

　　13.人民法院审理因上市公司虚假陈述侵权民事赔偿案件，在认定投资者损失数额时，应当根据《最高人民法院关于审理证券市场因虚假陈述引发的民事赔偿案件的若干规定》第十九条第四项的规定，区分疫情或者疫情防控措施影响因素和虚假陈述因素所导致的股价下跌损失，依法公平、合理确定损失赔偿范围。

　　14.对于批发零售、住宿餐饮、物流运输、文化旅游等受疫情或者疫情防控措施影响严重的公司或者其股东、实际控制人与投资方因履行“业绩对赌协议”引发的纠纷，人民法院应当充分考虑疫情或者疫情防控措施对目标公司业绩影响的实际情况，引导双方当事人协商变更或者解除合同。当事人协商不成，按约定的业绩标准或者业绩补偿数额继续履行对一方当事人明显不公平的，人民法院应当结合案件的实际情况，根据公平原则变更或者解除合同；解除合同的，应当依法合理分配因合同解除造成的损失。

　　“业绩对赌协议”未明确约定公司中小股东与控股股东或者实际控制人就业绩补偿承担连带责任的，对投资方要求中小股东与公司、控制股东或实际控制人共同向其承担连带责任的诉讼请求，人民法院不予支持。

　　15.在审理与疫情或者疫情防控措施相关的医疗保险合同纠纷案件时，对于保险人提出的该疾病不属于商业医疗保险合同约定的重大疾病范围或者保险事故的抗辩，人民法院不予支持。感染新冠肺炎的被保险人因疫情或者疫情防控措施未在保险合同约定的医疗服务机构接受治疗发生的约定费用，被保险人、受益人依据保险合同的约定向保险人请求赔付的，人民法院应予支持。被保险人因其他疾病在非保险合同约定的医疗服务机构接受治疗发生的约定费用，确系疫情或者疫情防控措施等客观原因造成，被保险人、受益人请求赔付的，人民法院应予支持。被保险人、受益人根据疫情防控期间保险公司赠与的医疗保险合同的约定请求赔付的，人民法院应予支持。

　　16.在审理融资租赁公司与医疗服务机构之间开展的医疗设备融资租赁业务所引发的民事纠纷案件时，对于医疗服务机构以融资租赁公司未取得医疗器械销售行政许可为由主张融资租赁合同无效的抗辩，人民法院不予支持。

　　三、关于破产案件的审理

　　17.企业受疫情或者疫情防控措施影响不能清偿到期债务，债权人提出破产申请的，人民法院应当积极引导债务人与债权人进行协商，通过采取分期付款、延长债务履行期限、变更合同价款等方式消除破产申请原因，或者引导债务人通过庭外调解、庭外重组、预重整等方式化解债务危机，实现对企业尽早挽救。

　　18.人民法院在审查企业是否符合破产受理条件时，要注意审查企业陷入困境是否因疫情或者疫情防控措施所致而进行区别对待。对于疫情爆发前经营状况良好，因疫情或者疫情防控措施影响而导致经营、资金周转困难无法清偿到期债务的企业，要结合企业持续经营能力、所在行业的发展前景等因素全面判定企业清偿能力，防止简单依据特定时期的企业资金流和资产负债情况，裁定原本具备生存能力的企业进入破产程序。对于疫情爆发前已经陷入困境，因疫情或者疫情防控措施导致生产经营进一步恶化，确已具备破产原因的企业，应当依法及时受理破产申请，实现市场优胜劣汰和资源重新配置。

　　19.要进一步推进执行与破产程序的衔接。在执行程序中发现被执行人因疫情影响具备破产原因但具有挽救价值的，应当通过释明等方式引导债权人或者被执行人将案件转入破产审查，合理运用企业破产法规定的执行中止、保全解除、停息止付等制度，有效保全企业营运价值，为企业再生赢得空间。同时积极引导企业适用破产重整、和解程序，全面解决企业债务危机，公平有序清偿全体债权人，实现对困境企业的保护和拯救。

　　执行法院作出移送决定前已经启动的司法拍卖程序，在移送决定作出后可以继续进行。拍卖成交的，拍卖标的不再纳入破产程序中债务人财产范围，但是拍卖所得价款应当按照破产程序依法进行分配。执行程序中已经作出资产评估报告或者审计报告，且评估结论在有效期内或者审计结论满足破产案件需要的，可以在破产程序中继续使用。

　　20.在破产重整程序中，对于因疫情或者疫情防控措施影响而无法招募投资人、开展尽职调查以及协商谈判等原因不能按期提出重整计划草案的，人民法院可以依债务人或者管理人的申请，根据疫情或者疫情防控措施对重整工作的实际影响程度，合理确定不应当计入企业破产法第七十九条规定期限的期间，但一般不得超过六个月。

　　对于重整计划或者和解协议已经进入执行阶段，但债务人因疫情或者疫情防控措施影响而难以执行的，人民法院要积极引导当事人充分协商予以变更。协商变更重整计划或者和解协议的，按照《全国法院破产审判工作会议纪要》第19条、第20条的规定进行表决并提交法院批准。但是，仅涉及执行期限变更的，人民法院可以依债务人或债权人的申请直接作出裁定，延长的期限一般不得超过六个月。

　　21.要切实保障债权人的实体权利和程序权利，减少疫情或者疫情防控措施对债权人权利行使造成的不利影响。受疫情或者疫情防控措施影响案件的债权申报期限，可以根据具体情况采取法定最长期限。债权人确因疫情或者疫情防控措施影响无法按时申报债权或者提供有关证据资料，应当在障碍消除后十日内补充申报，补充申报人可以不承担审查和确认补充申报债权的费用。因疫情或者疫情防控措施影响，确有必要延期组织听证、召开债权人会议的，应当依法办理有关延期手续，管理人应当提前十五日告知债权人等相关主体，并做好解释说明工作。

　　22.要最大限度维护债务人的持续经营能力，充分发挥共益债务融资的制度功能，为持续经营提供资金支持。债务人企业具有继续经营的能力或者具备生产经营防疫物资条件的，人民法院应当积极引导和支持管理人或者债务人根据企业破产法第二十六条、第六十一条的规定继续债务人的营业，在保障债权人利益的基础上，选择适当的经营管理模式，充分运用府院协调机制，发掘、释放企业产能。

　　坚持财产处置的价值最大化原则，积极引导管理人充分评估疫情或者疫情防控措施对资产处置价格的影响，准确把握处置时机和处置方式，避免因资产价值的不当贬损而影响债权人利益。

　　23.疫情防控期间，要根据《最高人民法院关于推进破产案件依法高效审理的意见》的要求，进一步推进信息化手段在破产公告通知、债权申报、债权人会议召开、债务人财产查询和处置、引进投资人等方面的深度应用，在加大信息公开和信息披露力度、依法保障债权人的知情权和参与权的基础上，助力疫情防控工作，进一步降低破产程序成本，提升破产程序效率。

1. 最高法：印发《关于依法妥善办理涉新冠肺炎疫情 执行案件若干问题的指导意见》 法发〔2020〕16号

各省、自治区、直辖市高级人民法院，解放军军事法院，新疆维吾尔自治区高级人民法院生产建设兵团分院：

现将《最高人民法院关于依法妥善办理涉新冠肺炎疫情执行案件若干问题的指导意见》印发给你们，请认真贯彻执行。

最高人民法院

2020年5月13日

最高人民法院

关于依法妥善办理涉新冠肺炎疫情执行案件若干问题的指导意见

为贯彻落实党中央关于统筹推进新冠肺炎疫情防控和经济社会发展工作部署会议精神，依法妥善办理涉新冠肺炎疫情执行案件，维护人民群众合法权益，维护社会和经济秩序，维护社会公平正义，依照法律、司法解释相关规定，结合执行工作实际，提出如下指导意见。

一、充分发挥执行工作的服务保障作用。各级人民法院要充分认识此次疫情对经济社会产生的重大影响，立足统筹推进疫情防控和经济社会发展工作大局，充分利用“基本解决执行难”工作成果，有效发挥“统一管理、统一指挥、统一协调”工作机制作用，平稳有序推进执行工作。被执行人有履行能力且具备强制执行条件的，要持续加大执行力度，依法保障胜诉当事人尤其是受疫情影响导致生产生活困难当事人的合法权益。在涉疫情执行案件办理过程中，要准确理解和适用法律，进一步突出强化善意文明执行理念，依法审慎采取强制执行措施，平衡协调各方利益，在依法保障胜诉当事人合法权益的同时，最大限度降低对被执行人权益的影响，积极引导当事人以和解方式化解矛盾纠纷，为统筹推进经济社会发展各项工作提供有力司法服务和保障。

二、依法中止申请执行时效。在申请执行时效期间的最后六个月内，因疫情或者疫情防控措施不能行使请求权，债权人依据《最高人民法院关于适用〈中华人民共和国民事诉讼法〉执行程序若干问题的解释》第二十七条规定主张申请执行时效中止的，人民法院应予支持。

三、准确把握查封措施的法律界限。坚决禁止超标的查封，严禁违法查封案外人财产，畅通财产查控的救济渠道，加大监督力度，切实防止违法执行或采取过度执行措施影响企业财产效用发挥和企业正常运营。做好审判程序与执行程序的衔接，人民法院审理涉疫情民事案件，要加大对财产保全申请的审查力度，对明显超出诉讼请求范围的超标的部分保全申请，依法不予支持。当事人通过恶意提高诉讼标的等方式超标的申请保全，给对方当事人造成损失的，对方当事人可以就所受损失依法提起诉讼。

对受疫情影响导致生产生活困难的被执行人，在不影响债权实现的前提下，人民法院应当选择适当的查封措施。被执行人有多项财产可供执行的，应当选择对其生产生活影响较小且方便执行的财产执行。对能“活封”的财产，不进行“死封”。查封厂房、机器设备等生产性资料，被执行人继续使用对该财产价值无重大影响的，应当允许其继续使用。被执行人申请利用查封财产融资清偿债务，经执行债权人同意或者融资款足以清偿所有执行债务的，可以监督其在指定期限内进行融资。查封被执行人在建工程的，原则上应当允许其继续建设。查封被执行人在建商品房或现房的，在确保能够控制相应价款的前提下，可以监督其在指定期限内按照合理价格自行销售房屋。冻结被执行人银行账户内存款的，应当明确具体数额，不得影响冻结之外资金的流转和账户的使用。

四、有效防止执行财产被低价处置。充分发挥网络司法拍卖公开透明、成本低、效率高、受疫情影响小的优势，加快财产变价流程，降低变价成本，为执行债权人及时回笼资金、减轻资金周转压力、恢复生产经营活动提供有力保障。在疫情期间进行网络司法拍卖，也要适当考虑疫情影响和财产实际情况，把握好拍卖时机，有效实现财产变现价值最大化。被执行人有充分证据证明疫情期间进行拍卖将严重贬损其财产价值，申请暂缓或中止拍卖的，人民法院可以准许。拍卖过程中，应当及时全面客观披露财产现状，充分发挥网拍平台、拍卖辅助机构作用，做好拍卖财产在线推介，吸引更多市场主体参与竞买。对财产价值较大、竞拍参与度可能较低的财产，可以确定适当宽松的拍卖价款支付期限。

对于一些专业化程度高、市场受众面较窄的财产，在不影响债权实现的前提下，可以允许被执行人通过其自身专业优势和渠道，灵活采取自行变卖、融资等方式偿还债务。被执行人认为网络询价或评估价过低，申请以不低于网络询价或评估价自行变卖查封财产清偿债务，人民法院经审查认为不损害执行债权人权益的，可以监督其在指定期限内变卖。网络司法拍卖第二次流拍后，被执行人提出以流拍价融资的，人民法院可以结合拍卖财产基本情况、流拍价与市场价差异程度等因素，酌情予以考虑；准许融资的，暂不启动以物抵债或者强制变卖程序。

五、依法执行疫情期间减免租金的政策规定。人民法院对被执行人的租金债权，可以强制执行。冻结被执行人的租金债权后，承租人在法定期限内提出异议的，依照有关司法解释的规定，人民法院不得对异议部分的租金强制执行。承租人对原租金债权的数额没有异议，但超过法定期限后依照疫情期间对承租国有经营性房屋的中小微企业、个体工商户减免租金的有关政策规定，主张减免租金提出异议，人民法院经审查属实的，应予支持；承租非国有经营性房屋的中小微企业、个体工商户以其与被执行人就疫情期间的租金减免已达成协议为由提出异议，请求对异议部分的租金不予强制执行，人民法院经审查认为租金减免协议真实有效的，应予支持。

对受疫情影响较大的中小微企业、个体工商户欠缴租金的涉众型执行案件，人民法院要充分发挥多元化纠纷解决机制作用，根据双方当事人实际情况，制定合理工作方案，依法妥善处理此类案件产生的矛盾纠纷。

六、强化应用执行和解制度。被执行人受疫情影响导致生产生活困难，无法及时履行生效法律文书确定义务的，人民法院要积极引导当事人协商和解，为被执行人缓解债务压力、恢复正常生产生活创造便利条件。当事人在疫情发生前已经达成和解，确因疫情或者疫情防控措施直接导致无法按照和解协议约定期限履行，申请执行人据此申请恢复执行原生效法律文书的，人民法院不予支持，但当事人另有约定的除外；和解协议已经履行不能或者因迟延履行导致和解协议目的不能实现的，应当及时恢复执行。

七、精准适用失信惩戒和限制消费措施。有效发挥失信惩戒和限制消费措施的惩戒作用，重点打击规避执行、抗拒执行等违法失信行为，进一步推动国家信用体系建设和营商环境改善。建立健全惩戒分级分类机制，准确把握失信惩戒和限制消费措施的适用条件，持续推动惩戒措施向精细化、精准化方向转变。疫情期间，对已纳入发展改革、工业和信息化部门确定的全国性或地方性疫情防控重点保障企业名单的企业，原则上不得采取失信惩戒和限制消费措施；已经采取并妨碍疫情防控工作的，要及时解除并向申请执行人说明有关情况。对未纳入重点保障企业名单的疫情防控企业采取失信惩戒和限制消费措施的，可以根据具体情况参照前述规定办理。对受疫情影响较大、暂时经营困难的企业尤其是中小微企业，人民法院在依法采取失信惩戒或者限制消费措施前，原则上要给予三个月的宽限期。

健全完善信用修复机制，失信名单信息依法应当删除或撤销的，应当及时采取删除或撤销措施。失信名单信息被依法删除或撤销，被执行人因求职、借贷等被有关单位要求提供信用修复证明的，经被执行人申请，人民法院可以就删除或撤销情况出具相关证明材料。受疫情影响较大的被执行企业尤其是中小微企业确因复工复产需要，申请暂时解除失信惩戒措施的，人民法院应当积极与申请执行人沟通，在征得其同意后及时予以解除。

八、合理减免被执行人加倍部分债务利息。被执行人以疫情或者疫情防控措施直接导致其无法及时履行义务为由，申请减免《中华人民共和国民事诉讼法》第二百五十三条规定的相应期间的加倍部分债务利息，人民法院经审查属实的，应予支持；被执行人申请减免生效法律文书确定的一般债务利息的，不予支持，但申请执行人同意的除外。

九、充分发挥破产和解、重整制度的保护功能。对执行债权人人数众多，特别是多个执行债权人正在申请分配案款的案件，被执行企业因疫情影响导致生产经营困难不能清偿所有执行债务的，人民法院要积极引导各方当事人进行协商，依法为被执行企业缓解债务压力、恢复生产经营创造条件；多个案件由不同法院管辖的，上级法院要加强统筹协调，通过提级执行、指定执行等方式协调案件进行集中办理，力争促成各方当事人达成解决债务的“一揽子”协议。当事人未能达成协议且案件符合移送破产审查条件，通过破产和解或重整能够帮助被执行企业恢复经营的，人民法院要进一步加强立审执协调配合，畅通执行移送破产工作渠道，充分发挥破产和解和破产重整制度的保护功能，帮助企业及时走出困境。人民法院在执行过程中，要严格防止被执行企业通过破产程序逃避债务，依法保障执行债权人合法权益。

十、充分利用信息化手段推动执行工作。充分利用“智慧法院”建设成果，特别是以现代信息技术为支撑的执行信息化系统，强化上级法院对下级法院执行工作的监督管理，提高执行效率，降低执行成本，提高执行效果，降低负面效应，避免引发新的矛盾和纠纷。依法优先采取网络查控、网络询价、网络司法拍卖、网络收发案款等在线执行措施，积极通过线上方式开展立案、询问谈话、执行和解、申诉信访、执行辅助等工作，充分满足人民群众的司法需求，确保疫情期间人民法院执行工作平稳有序运行。

1. 市场监管总局等7部门：印发《全国防疫物资产品质量和市场秩序 专项整治行动方案》 国市监竞争﹝2020﹞74号

各省、自治区、直辖市及新疆生产建设兵团市场监管局（厅、委）、发展改革委、工业和信息化厅、公安厅（局）、商务厅、药监局，各直属海关：

新冠肺炎疫情发生以来，按照党中央、国务院重要决策部署，各地各部门认真贯彻落实，采取一系列有力举措，大力加强防疫物资保障监管，取得积极成效。防疫物资生产大幅度提升，防疫物资质量不断提升，确保了国内抗疫需求，越来越多产品出口到海外，支持各国抗疫。

为进一步加强防疫物资产品质量监管，切实维护市场秩序，七部门决定联合开展全国防疫物资产品质量和市场秩序专项整治行动。现印发专项整治行动方案，请结合本地区实际情况，认真执行。

市场监管总局 国家发展改革委 工业和信息化部

公安部 商务部 海关总署

药监局

2020年5月11日

（此件公开发布）

全国防疫物资产品质量和市场秩序专项整治行动方案

党中央、国务院高度重视疫情防控工作，习近平总书记作出一系列重要讲话和重要指示批示。各地各部门认真贯彻落实党中央、国务院决策部署，采取有力举措，大力加强防疫物资保障监管，取得积极成效。随着我国复工复产、复市复业工作有序推进，特别是国际疫情的蔓延，对防疫物资需求大幅增加，防疫物资产品质量和市场秩序成为各方关注的焦点。为进一步提升产品质量，着力维护市场秩序，现制定全国防疫物资产品质量和市场秩序专项整治行动方案。

一、基本思路

防疫物资产品质量和市场秩序关系到经济社会发展全局。各地各部门要提高政治站位，从保障抗疫取得全面胜利的战略高度，从保护人民群众和医护人员健康安全的战略高度，从促进防疫物资产业高质量发展的战略高度，从维护国家形象和中国制造声誉的战略高度，充分认识开展此次专项整治行动的重要意义，增强紧迫感、责任感、使命感。通过专项整治行动，全面排查防疫物资产品质量和市场秩序中存在的隐患，全面整治生产、流通、消费、出口过程中存在的突出问题，建立横向协同、纵向联动的工作机制，深化防疫物资保障监管工作，为统筹推进疫情防控和经济社会发展提供有力支持。基本思路是：

突出全种类整治。着重对口罩、防护服、呼吸机、红外体温计（额温枪）、新型冠状病毒检测试剂等五类防疫物资及其重要原辅材料加强监管，保障防疫物资全产业链平稳运行。

突出全过程整治。贯穿防疫物资生产、流通、消费、出口全链条监管。强化生产源头控制，严格企业资质，明确产品标准，提升产品质量；强化流通环节监管，对不合格产品开展全流程追溯，依法追究生产经营者责任；强化消费市场监管，维护消费者利益；强化出口监管，严把出口防疫物资质量关。

突出全方位整治。统筹国内国际市场，既要全力保障国内防疫物资产品质量和供应，规范市场秩序；也要全力保障出口产品质量，规范出口秩序，有力支持国际抗疫大局。

突出综合整治。针对防疫物资生产、产品质量、标准认证、检验检测以及市场秩序等方面的突出问题，综合运用行政执法、刑事打击、信用惩戒、联合执法等多种手段，提升整治效能。

二、主要任务

（一）全面排查防疫物资生产企业。全面梳理本区域口罩、防护服、呼吸机、红外体温计（额温枪）、新型冠状病毒检测试剂等五类防疫物资及其重要原辅材料生产企业清单，认真深入开展排查。督促企业落实产品质量安全主体责任，加强自身原材料进货质量控制和生产工艺过程控制，规范产品标识标签和说明书。严格执行注册检验、临床试验、技术审评、体系核查、行政审批等各项要求，严格规范做好防疫物资审评审批工作，从源头上确保质量安全。对没有资质、资质不全、不符合生产条件的企业依法依规严肃处理，切实把好市场准入关。（市场监管、药监等部门按职责分工负责）

（二）全面加强防疫物资产品质量抽检。全面加强对生产销售的防疫物资和重要原辅材料的质量监管。以问题为导向，突出抽检工作的针对性和靶向性，加大对疫情发生以来新注册产品、新批准企业以及信息提示风险较高产品的抽检力度，做到关口前移，防止不符合质量标准的产品流入市场。全面排查已流入市场的不合格产品流向，依法处置，对违法生产经营主体依法查处并曝光。（市场监管、药监等部门按职责分工负责）

（三）全面加强出口防疫物资监管。严把五类防疫物资出口质量关，加大出口防疫物资查验力度。对不符合相关质量标准的出口产品，要追溯经销商、生产企业以及认证检验检测、价格收费各个环节。对投诉举报较多的出口企业进行全面核查，对抽检不合格，存在产品质量安全重大问题的，依法从严查处。（市场监管、海关、药监等部门按职责分工负责）

（四）全面规范认证检验检测行为。充分发挥认证检验检测机构在质量监管中的重要作用，明确并公布从事国内认证和主要国际辖区认证业务的机构名单。严厉打击伪造、冒用、非法买卖认证证书和认证标志，未经批准擅自在我国境内从事认证、检验检测活动的违法行为。（市场监管、海关等部门按职责分工负责）

（五）全面查处各类违法犯罪行为。严厉打击生产销售和出口不符合相关标准的产品，在产品中掺杂掺假、以假充真、以次充好或者以不合格产品冒充合格产品，生产销售过期失效产品，生产销售无生产日期、无厂名厂址、无产品质量合格证明等“三无”产品等质量违法行为。严厉打击囤积居奇、哄抬价格、违规收费等价格违法行为。严厉打击防疫物资领域制售假劣商品、非法经营等违法犯罪活动。深化协调配合，加强行刑衔接，建立线索通报、信息共享、案件移送快速通道和防疫物资证据快鉴快检通道，及时将相关违法违规行为纳入企业信用记录，实施联合惩戒，形成打击合力。（公安、海关、市场监管、药监等部门按职责分工负责）

（六）全面提升服务市场主体水平。积极主动靠前服务，深入了解企业生产经营面临的实际困难和政策诉求，不断完善相关政策法规，优化营商环境。强化防疫物资应急调配，保障有效供应。指导防疫物资生产企业加强全面质量管理，更好落实主体责任，推动相关标准信息指南落地应用，进一步指导企业规范生产和出口。督促商贸流通企业落实进销主体责任和购销台账制度。加强质量帮扶，强化国内外标准比对和推动互认，提升检验检测服务能力，及时更新防疫物资检验检测机构名录及相关信息，促进企业提升产品质量。加大企业商标、商业秘密等知识产权保护力度，维护企业正当权益。（发展改革、工业和信息化、商务、市场监管、药监等部门按职责分工负责）

三、工作要求

（一）提高政治站位，加强组织领导。各地各部门要强化政治意识，树立全局观念，把开展防疫物资产品质量和市场秩序专项整治行动作为一项重要政治任务抓紧抓实抓细。市场监管部门会同有关部门迅速建立联合工作机制，加强工作统筹，做好协调服务。各部门积极配合、主动作为，共同完成专项整治任务。市场监管部门在发挥好牵头作用的同时，建立内部高效协同机制，形成工作合力。

（二）强化协同配合，创新工作机制。建立健全信息报送机制，各地各部门要指定专人，每周定期向牵头单位报送整治进展情况，重大事项随时报告。建立全链条查处机制，各地各部门在整治过程中，对发现的重大问题和线索要及时移交相关部门，实行全流程追溯。建立综合整治机制，发挥各部门职能优势，注重横向配合、纵向联动，开展综合协同整治。

（三）突出整治重点，狠抓工作落实。各地结合实际情况，细化工作方案，严格按照时间节点，倒排工期，层层压实责任，确保专项整治各项目标任务落到实处。对重大问题、重大案件，各部门要加强研判、直接查办、挂牌督办，各地要建立问题清单台账制度，落实属地责任。5月中下旬，市场监管总局会同有关部门开展全国专项整治行动督导，推进工作进展，针对存在的问题加强分类指导。6月底进行全面工作总结，评估专项整治工作成效，提出完善长效机制的建议，对工作扎实、成绩显著的地方予以表扬激励。

（四）加强舆论引导，营造良好氛围。要坚持正面宣传引导，加强与宣传部门、新闻媒体的沟通协调，深入宣传解读政策措施，及时发布整治成果。对重大案件、典型案例加大曝光力度，形成有力震慑。积极推动社会共治、全民监督，充分发挥媒体监督作用，营造良好舆论氛围。

各省（区、市）市场监管（含药监）部门自收到本通知之日起，每周四17时前向市场监管总局报送本区域工作开展情况和统计表。

1. 民政部：做好疫情防控常态化形势下慈善捐赠工作 民函〔2020〕52号

各省、自治区、直辖市民政厅（局），各计划单列市民政局，新疆生产建设兵团民政局：

新冠肺炎疫情发生以来，在党中央坚强领导下，在各级民政部门倡导和动员下，广大爱心企业、爱心人士踊跃捐款捐物，全国各级慈善组织、红十字会广泛动员募捐、开展慈善活动，发扬一方有难、八方支援的优良传统，为湖北省武汉市等疫情严重地区提供支持，协助党和政府为全国疫情防控工作贡献了力量。当前我国疫情防控阶段性成效进一步巩固，复工复产取得重要进展，经济社会运行秩序加快恢复。各级民政部门要在巩固疫情防控慈善捐赠工作成果的基础上，继续指导督促慈善组织、红十字会在常态化疫情防控中做好慈善捐赠工作，有针对性地开展心理疏导等服务，更好发挥支持疫情防控和经济社会发展的作用。

一、做好疫情防控应急慈善捐赠后续工作

（一）加快分配使用，依法妥善安排结存捐赠款物。地方各级民政部门要指导督促慈善组织、红十字会对前期慈善捐赠做好收尾工作，充分、高效管好用好捐赠款物。对于前期重点支持疫情防控医疗和生活应急需求的公开募捐活动，要根据各地疫情风险等级的调整，相应调整募捐时限、适时予以终止；尚未向捐赠人开具有关证明的，要及时开具。对于前期疫情防控捐赠款物尚有结存的，根据境外输入疫情压力较大地方的需要，在征得捐赠人同意的前提下，及时向有需要的地区拨付；也可以根据疫情变化及时安排用于心理疏导和社会工作服务、受疫情影响重点群体抚慰和抗疫一线人员关爱等疫情防控相关用途；合理安排捐赠款物拨付进度，不允许长时间积压。

（二）严格监管责任，防范违法违规行为。地方各级民政部门要指导督促慈善组织、红十字会依法申领公开募捐资格证书、履行公开募捐方案备案，落实对捐赠款物的跟踪监督，防范违法违规行为。慈善组织、红十字会不得私分、挪用、截留、侵占或者倒卖捐赠款物。凡是开展捐赠活动所需工作经费已经由财政资金全额保障的慈善组织、红十字会，不得从捐赠资金中提取任何形式的工作经费。慈善组织、红十字会发现接收单位违背捐赠目的使用捐赠款物的，可以要求其改正；对拒不改正的依法要求其退还。对慈善组织、红十字会违反前述规定的，要依法予以严肃处理。

（三）加强财务管理，做到账实相符。地方各级民政部门要指导督促慈善组织、红十字会对捐赠款物及时登记入账。慈善组织、红十字会要将接收的捐赠款物全部纳入本单位财务集中管理、统一核算，做到手续完备、专项管理、账实相符、账目清楚；要依据会计制度规定对捐赠物资尽快计价，按规定开具捐赠票据。地方各级民政部门对慈善组织、红十字会接收分配使用捐赠款物的情况要及时汇总上报。

（四）落实信息公开，接受社会监督。地方各级民政部门要督促慈善组织、红十字会回应社会关切，主动接受社会监督。慈善组织、红十字会要按照逐级拨付逐级公布的要求，将接收单位的名称、接收款物的金额数量及时向社会公开。对于公开募捐和慈善项目实施周期超过六个月的，慈善组织、红十字会根据《慈善组织信息公开办法》规定，按照至少每三个月公开一次的要求，在全国慈善信息公开平台（“慈善中国”）上尽快完成对公开募捐和项目实施情况的第一次全面公布。湖北省民政厅要指导当地有关慈善组织、红十字会根据实际情况调整对捐赠款物接收分配情况的公布频率。地方各级民政部门要结合今年的年报年检工作，指导督促慈善组织、红十字会对疫情防控慈善捐赠情况进行专项检查，重点检查公开募捐和信息公开情况，并向社会公布结果。

二、根据疫情防控常态化需求精准开展慈善活动

（一）及时响应社会需求开展慈善活动。地方各级民政部门要指导督促慈善组织、红十字会继续广泛参与，精准把握、及时响应疫情防控常态化社会需求，紧密服务于疫情防控成果的巩固扩大，支持生产生活步入正轨，助力经济社会恢复，精准开展慈善募捐；同时也要根据外防输入、内防反弹防控工作的需要，有针对性地动员慈善捐赠予以支持。要动员引导慈善组织、红十字会把促进社会和谐稳定放在首位，面向基层社区开展慈善项目，充分发挥扶危济困、精神抚慰方面的优势，体现关心关爱和人文关怀，与各级政府的困难群众兜底保障和城乡社区服务形成合力。

（二）加强慈善、社会工作、志愿服务联动，协同做好心理疏导和社会工作服务。地方各级民政部门要指导督促慈善组织、红十字会在疫情善后工作中加强慈善捐赠与社会工作、志愿服务的联动，积极利用慈善捐赠，通过资助、购买服务等方式，支持社会工作服务机构及行业组织、志愿服务组织、心理援助机构对重点人群精准开展心理疏导、精神提振、融入融合等服务，要充分发挥慈善捐赠的社会化优势、社会工作的专业化优势和志愿服务的群众性优势，分层分类、一事一策地帮困解难，持续开展心理疏导及转介、家庭支持、资源链接、社会关系修复等服务，以具体行动推动恢复生产生活秩序，营造良好社会氛围，促进社会和谐稳定。

（三）关注重点人群，精准落实帮扶关爱。地方各级民政部门要指导督促慈善组织、红十字会面向重点人群精准服务，优先对下列群体开展关爱活动：一是新冠肺炎患者、逝者、隔离人员及受影响家庭；二是低保对象、特困人员、低收入家庭成员、建档立卡贫困人口，受疫情影响致贫人员和返贫的建档立卡贫困人口；三是孤寡老人、困境儿童、残疾人等特殊群体；四是在疫情防控一线奋战的医务工作者、人民解放军指战员、社区工作者、公安民警、基层干部、下沉干部、志愿者；五是受疫情影响无法外出务工、经营、就业导致生活困难的群众；六是在复工复产和恢复生产生活秩序中遇到困难的群众。

（四）聚焦湖北武汉等重点地区，继续动员慈善力量支援。各地要重点支持湖北省、武汉市等疫情严重地区的疫情善后与疫后恢复，特别是支持湖北省综合发挥慈善捐赠、社会工作、志愿服务的作用，有力组织开展心理疏导和社会工作服务。湖北省、武汉市民政部门要统筹安排好省市内外的慈善资源，指导慈善组织、红十字会建立专项基金，对确定用于心理疏导和社会工作服务的前期结存捐赠资金和新筹集捐赠资金统筹管理、专款专用。要精心组织好本地心理疏导和社会工作服务，推进以社区为阵地的线下服务与互联网线上服务密切结合，针对各类社区、家庭、群体的不同需求准确施策，高效运作。要按照规定有序开展向社会工作服务机构、志愿服务组织、心理援助机构等进行资助或者购买服务的工作，加强对资金使用效率和社会效果的评价。

请各省（区、市）民政厅（局）于5月20日前向民政部报送前一阶段动员慈善捐赠支援湖北省、武汉市疫情防控的情况和下一阶段工作计划。

民 政 部

2020年5月12日

1. 卫健委等4部门：《新冠肺炎出院患者主要功能障碍康复治疗方案》 国卫医函〔2020〕207号

各省、自治区、直辖市及新疆生产建设兵团卫生健康委、民政厅（局）、医保局、中医药管理局：

随着新冠肺炎出院患者人数的逐渐增加，出院患者多层次、多类型的康复医疗需求日益凸显。为进一步加强新冠肺炎出院患者主要功能障碍的康复治疗工作，落实全流程健康管理措施，促进患者全面康复，我们制定了《新冠肺炎出院患者主要功能障碍康复治疗方案》，经国务院应对新型冠状病毒肺炎疫情联防联控机制同意，现印发给你们，请认真贯彻落实。

国家卫生健康委 民 政 部

国家医疗保障局 国家中医药管理局

2020年5月13日

（信息公开形式：主动公开）

新冠肺炎出院患者主要功能障碍康复治疗方案

为改善新冠肺炎患者呼吸功能、心脏功能、躯体功能以及心理功能障碍，规范康复的操作技术及流程，最大限度减轻患者负担，促进全面康复，特制定本方案。

一、呼吸功能障碍

（一）主要表现。可表现为呼吸困难、活动后气短，喘息、胸闷，咳嗽咳痰无力，以限制性通气功能障碍、弥散量降低伴低氧血症或呼吸衰竭为主要表现。

（二）功能评估。1.症状评估：即改良的医学研究理事会呼吸困难量表（mMRC）评价；2.活动耐力评估：即6分钟步行试验（6MWT）和心肺运动负荷试验（CPET）评估；3.静态肺功能评估：即肺通气功能和弥散功能；4.动脉血气或无创脉氧饱和度评价：即动脉氧分压和氧饱和度等评价患者的缺氧程度。

（三）康复治疗。 1.呼吸训练：包括呼吸功能训练（主动循环呼吸技术ACBT，含呼吸控制、胸廓扩张运动和用力呼气技术）、呼吸模式训练（包括调整呼吸节奏、腹式呼吸训练、缩唇呼吸训练等）、呼吸肌力量训练、呼吸康复操（卧位、坐位及站立位系列运动）。

2.有氧运动：方式有行走、慢跑、骑自行车、游泳、健身操，以及在器械上完成的行走、踏车、划船等。建议从低强度开始，结合Borg自觉疲劳量表评分13-16分和改良Borg自觉疲劳量表气促评分≤5-6分，根据病情和患者耐受程度，每次运动20-60分钟，每周3-7次，循序渐进，逐步增大运动强度和时间。

3.氧疗：（1）静息状态下，动脉血氧分压≤55mmHg或经皮血氧饱和度（SpO2）≤88%，应给予氧疗；（2）如合并充血性心力衰竭、肺动脉高压等基础疾病者，氧疗指征为动脉血氧分压≤60mmHg或SpO2≤90%；（3）如运动中出现低氧血症或 SpO2≤88%，应给予补充氧疗，以保证运动中SpO2维持在95%。

4.ADL训练、康复宣教（如生活方式指导等）。

二、心脏功能障碍

（一）主要表现。心悸、胸闷、活动后气促、劳力呼吸困难，还可出现心前区不适及心绞痛，多与活动有关。心率增快或减慢，可出现多种心律失常。导致心功能障碍的原因与新型冠状病毒对心脏的直接损伤有关，也可继发于新冠肺炎导致的肺功能障碍，以及重型、危重型患者长期卧床、制动所致的废用性功能减退。此外，还可与合并基础疾病，如高血压、冠心病、糖尿病等有关。

（二）功能评估。根据患者病情和医院自身的条件，有条件者可采用CPET评估心脏功能，如无相应条件，可采用6MWT、台阶试验、代谢当量活动问卷等进行评估。同时应结合患者疾病临床情况，如原发病、基础疾病，心电图、心脏彩超、心肌酶谱等进行综合评定。

（三）康复治疗。基于心肺功能评估制定运动处方。

1.有氧运动：同呼吸功能障碍康复治疗有氧运动内容。

2.肌力及肌耐力训练：方式有抗阻运动器械、哑铃、引体向上、俯卧撑及弹力带和弹力管等。根据患者的能力，以重复10-15次的负荷重量（10-15RM），Borg评分13-14分和改良Borg气促评分≤5-6分为宜。根据病情和患者耐受程度，每次训练8-16组肌群，每个肌群2-3组，重复10-15次/组。建议隔天一次，每周训练2-3次。

3.柔韧性训练：有氧运动或抗阻训练后进行。每个肌群15-60秒，2-4次，以有明显拉伸感、无明显疼痛为宜。

4.平衡功能和协调性训练：视情况进行。

运动中应密切观察心电、血压、血氧饱和度等。必要时在氧疗的同时进行运动治疗。病毒性心肌炎活动期适当调整运动处方。运动治疗的同时，不宜忽视患者基础病的药物治疗以及饮食、睡眠、心理指导等。

三、躯体功能障碍

（一）主要表现。表现为全身乏力、易疲劳、肌肉酸痛，部分可伴有肌肉萎缩、肌力下降等。多见于危重、重症型新冠肺炎出院患者，由于长期卧床、制动所引起的继发性躯体功能障碍。

（二）功能评估。采用Borg自觉疲劳量表、徒手肌力检查、徒手平衡功能评定等进行评估。

（三）康复治疗。轻度、中度呼吸功能障碍患者可以选择有氧运动、肌力及肌耐力训练。重度呼吸功能障碍及体能极度下降的患者，需要从床上运动、转移、平衡功能、步行功能、及上下楼梯等开始训练。

1.有氧运动：同呼吸功能障碍康复治疗有氧运动内容。

2.肌力及肌耐力训练：同心脏功能障碍康复治疗肌力及肌耐力训练内容。

3.平衡功能和协调性训练：视情况进行。

4.氧疗：参考呼吸功能障碍康复治疗氧疗内容。

四、心理功能障碍

（一）主要表现。 1.情绪反应：焦虑担心害怕、情绪不稳定、抑郁悲伤、无助与愤怒。

2.认知改变：一些患者会有感觉失真、无法集中注意力、犹豫不决、自责等。

3.行为障碍：失眠、回避行为、过度进食、过量饮酒、自伤甚至自杀行为。

4.生理反应：可能会出现因情绪而引起的心慌、头痛、肌肉酸痛、消化不良，胃胀，反胃，食欲下降等心身反应。

（二）功能评估。 1.抑郁症筛查量表9项（PHQ-9）：由9个项目组成，采用0-3分的4级评分法。总分在0-4分为无抑郁症状，5-9分为轻度抑郁，10-14分为中度抑郁，15分以上为重度抑郁。

2.广泛性焦虑量表7项（GAD-7）：由7个项目组成，采用0-3分的4级评分法。总分在0-4分为无焦虑症状，5-9分为轻度焦虑，10-14分为中度焦虑，15分以上为重度焦虑。

3.匹兹堡睡眠问卷：为自评量表，用于评定近一个月睡眠质量，按照0-3等级计分，总分范围0-21分，得分越高，表示睡眠质量越差。

4.创伤后应激障碍症状清单（PCL-C）：是我国《创伤后应激障碍防治指南》推荐的版本，为自评量表，包括17个项目，分为1-5级评定，分数越高，代表PTSD发生的可能性越大。

（三）康复干预。 1.广泛开展科普宣教。面向社区居民广泛开展科普宣教。及时通过权威媒体发布信息。通过张贴宣传海报、宣传页，给社区居民发放肺炎知识宣传手册及心理健康服务手册等方式，在社区开展针对性科普宣传和活动。

2.新冠肺炎科普宣传。开展新冠肺炎相关科普知识的宣传，引导大众正确了解新冠肺炎的特点，减少对新冠肺炎康复患者及家属的歧视和排挤，保证康复患者复工权益。

3.心理健康科普宣传。为康复患者发放心理健康服务宣传页，内容包括心理健康知识、心理自我调适常识，以及心理支持平台二维码、心理热线电话等支持性资源。

4.做好针对性心理疏导和社会工作服务。根据受疫情影响情况，在有条件的社区卫生服务中心设置心理专干岗位。在街道（乡镇）设置社会工作站，配备专兼职社会工作者。有条件的社区可建立由社区工作者、社会工作者、志愿者、心理咨询师、心理治疗师、精神科医生等组成的社区心理疏导和社会工作服务队。建立心理健康服务档案，按需求、分层次提供多种形式的心理健康服务，包括个体咨询、夫妻咨询、家庭咨询和团体心理辅导或线上心理咨询服务。发现具有自伤、自杀、冲动伤人风险的出院患者及家属，社区工作人员、社会工作者、心理专干等人员要增加走访密度，留下紧急联系电话或心理热线。由心理咨询师、精神卫生社会工作者等介入进行危机干预，必要时向精神卫生医疗机构转诊。

5.要加强对新冠肺炎出院患者及其家属的人文关怀，帮助患者恢复正常生活，鼓励大众互帮互助，消除歧视。

五、日常生活活动能力障碍

（一）主要表现。部分病情较重、合并基础疾病的患者，可能无法独立完成穿脱衣、如厕、洗澡等。

（二）评估方法。采用改良巴氏指数评定表等进行评估。

（三）康复训练。对患者进行日常生活活动指导。主要是节能技术指导，将穿脱衣、如厕、洗澡等日常生活活动动作分解成小节间歇进行，随着体力恢复再连贯完成，逐步恢复至正常。对不能胜任工作岗位的出院患者进行有关功能训练和职业康复。

六、中医康复

（一）主要表现。表现为乏力、气短、咳嗽、胸闷、心悸、失眠、纳差、呕恶等。

（二）中医康复治疗。 1.基础方剂：黄芪15g、党参15g、炒白术15g、南北沙参各9g、麦冬15g、陈皮15g、茯苓15g、法半夏9g、知母12g、丹参15g、浙贝母15g、赤芍15g、桔梗9g、防风9g、甘草6g、炒三仙各9g、山药15g。

服法：每日1剂，水煎400ml，分2次服用，早晚各1次。

2.中医适宜技术。

（1）艾灸疗法：选取中脘、气海、天枢（双侧）、内关（双侧）、足三里（双侧）等穴位。

（2）穴位按摩：选取内关（双侧）、孔最（双侧）、膻中、足三里（双侧）等穴位。

（3）拔罐疗法：选取肺俞（双侧）、膈俞（双侧）、脾俞（双侧）、风门（双侧）等穴位。

（4）耳穴按摩和压豆：摩擦耳轮、提拉耳尖、下拉耳垂、鸣天鼓。耳穴压豆常用支气管、肺、内分泌、神门、枕、脾、胃、大肠、交感等。

3.辨证论治。

（1）正虚邪恋证：发热已退，口苦，咽干，胸胁苦满，烦躁，焦虑，眠差，咳嗽，或有黄痰，恶心纳差。舌红，苔白腻或黄腻，脉濡数或弦数。

推荐处方：柴胡9g、黄芩15g、桂枝9g、赤芍15g、白芍15g、炙甘草6g、煅龙骨15g（先煎）、煅牡蛎15g（先煎）、西洋参9g、北沙参15g、清半夏9g、陈皮9g、六神曲9g、茯苓15g。

服法：每日1剂，水煎400ml，分2次服用，早晚各1次。

推荐中成药：和解清热类。

（2）痰瘀阻络证：胸闷，胸痛，动则气短，乏力，咳嗽。舌紫暗或有瘀斑、瘀点，苔薄白，脉涩弱。适用于重型、危重型恢复期患者，肺功能损伤或肺部CT有纤维化表现。

推荐处方：黄芪15g、党参9g、麸炒白术9g、南沙参9g、北沙参9g、麦冬15g、陈皮9g、茯苓15g、法半夏6g、丹参9g、浙贝母3g、水蛭3g、土鳖虫3g、甘草6g、炒山楂3g、炒六神曲3g、炒麦芽3g、山药9g。

服法：每日1剂，水煎400ml，分2次服用，早晚各1次。

推荐中成药：益气活血类。

（3）肺脾气虚证、气阴两虚证推荐方药见《新型冠状病毒肺炎诊疗方案（试行第七版）》。

七、组织保障

（一）明确康复治疗原则。以重症、危重症患者为重点康复人群，对不同病情、不同功能障碍的患者采取个体化康复治疗措施。开展康复治疗前全面科学评估患者的健康状况和康复治疗承受能力，加强康复期间生命体征、耐受情况监测，确保患者安全。康复治疗要早期介入，做好临床治疗阶段和出院后康复治疗的衔接，要重视患者多器官、多系统功能和心理功能的综合康复。

（二）明确康复机构。各地要根据实际出台具体的新冠肺炎出院患者康复管理方案，根据患者病情级别、功能障碍类型和医疗机构的服务能力，分级分类明确康复机构，开展新冠肺炎出院患者康复治疗工作。

（三）加强康复力量。各地要开展对医务人员（包括医师、护士、康复治疗师、医务社会工作者等）不同层次的专业培训，不断提升康复意识，增强康复医疗处置能力。尤其要注重加强基层医疗机构社区康复能力建设，有针对性地开展康复医疗基本知识、基本技能的培训，提升社区康复能力和水平，为新冠肺炎出院患者提供方便可及的服务。

（四）加强康复医疗保障。各地要做好新冠肺炎出院患者主要功能障碍医疗康复的医疗保障工作，切实将《关于将部分医疗康复项目纳入基本医疗保障范围的通知（卫农卫发﹝2010﹞80号）》和《关于新增部分医疗康复项目纳入基本医疗保障支付范围的通知》（人社部发﹝2016﹞23号）规定的29项医疗康复项目医保政策落实到位，并可根据基金承受能力，将符合条件的心理治疗按规定纳入医保支付范围，同步加强康复医疗行为监管。新冠肺炎出院患者符合规定的门诊康复医疗费用纳入门诊慢特病管理。医保部门要加强对康复类医疗服务价格政策落实的指导和督导。

（五）加强基本生活救助。各地民政部门要对符合救助条件的需开展康复治疗的新冠肺炎出院患者及其家庭，按规定及时纳入低保、特困救助供养范围。对低保、特困救助供养暂时无法覆盖的困难群众，通过临时救助做到凡困必帮、有难必救。加强社会救助服务热线值守，保障热线畅通，确保需开展康复治疗的新冠肺炎出院患者求助有门。

1. 文旅部：印发《剧院等演出场所恢复开放疫情防控措施指南》

各省、自治区、直辖市文化和旅游厅（局），新疆生产建设兵团文化体育广电和旅游局：

　　现将《剧院等演出场所恢复开放疫情防控措施指南》《互联网上网服务营业场所恢复开放疫情防控措施指南》《娱乐场所恢复开放疫情防控措施指南》印发给你们，请结合实际抓好贯彻落实。

　　特此通知。

附件1

剧院等演出场所恢复开放疫情防控措施指南

为贯彻落实习近平总书记关于统筹推进新冠肺炎疫情防控和经济社会发展工作的重要指示精神及《国务院应对新型冠状病毒感染肺炎疫情联防联控机制关于做好新冠肺炎疫情常态化防控工作的指导意见》（国发明电〔2020〕14号）要求，有序推进演出场所恢复开放，制定本指南。

一、总体要求

（一）坚持常态防控。各地文化和旅游行政部门应当按照“属地原则”，严格遵守当地疫情防控指挥部门的要求，结合本地区实际情况，毫不放松抓紧抓实抓细各项防控工作，制定包含演出场所在内的疫情防控方案和应急预案，并及时进行动态调整，确保安全。

（二）坚持有序开放。在充分做好防疫措施的情况下，在低风险地区，经当地党委政府同意，可以举办营业性演出活动，但暂缓举办中大型营业性演出活动，暂缓新批涉外、涉港澳台营业性演出活动（演职人员已在境内的除外）。在中、高风险地区，暂缓举办营业性演出活动。

（三）坚持预约限流。恢复开放的演出场所应当严格执行人员预约限流措施。剧院等演出场所观众人数不得超过剧场座位数的30%，要间隔就坐，保持1米以上距离。演员之间要保持一定距离。含有多个剧场的综合性演出场所，同时只能开一个剧场。在歌舞娱乐场所、旅游景区、主题公园、游乐园、宾馆、饭店、酒吧、餐饮场所、现场音乐厅（LiveHouse）等场所举办营业性演出，演出主办方要安排工作人员在现场做好人员疏导。

二、场所防控管理

（四）落实防控主体责任。演出场所要严格执行疫情防控规定，制定本场所防控工作制度和应急预案，把防控责任落实到具体个人。

（五）做好日常消毒通风。演出场所要对舞台区、观众区、化妆间、通道、出入口、行政办公场地等公共区域，每日定时进行两次消毒。建立《场馆清洁消毒记录表》，记录消毒时间、责任人等信息。演出场所要保持通风状态，在条件允许情况下首选自然通风。

（六）配备防护用品。演出场所要配备口罩、手套等防护物资，并在洗手间配备洗手液及消毒用品，为员工和观众提供必要防护保障。

（七）加强防疫宣传。演出场所应通过设置提示牌、摆放宣传品、电子显示屏等多种方式，加强疫情防控知识科普宣传。

（八）排查安全隐患。演出场所恢复开放前应当积极配合有关部门，全面排查火灾等安全隐患，并按照《文化部关于落实安全生产责任加强文化市场安全生产工作的通知》（文市发〔2017〕5号）有关规定，加强场所日常检查，不符合安全条件的立整立改。

（九）鼓励线上服务。鼓励推广在线实名制购票及电子票，鼓励使用在线支付，尽量减少直接接触。对剧院等专业演出场所，实行实名制购票和实名制入场。

三、演员和观众管理

（十）做好演职人员管理。演出主办方应当与参演单位和个人签订安全协议或健康承诺书，提前做好对演职人员（含行政、后勤等工作人员）的体温监测等防控措施。每场演出尽量压缩不必要的演职人员，注意保持一定距离。演员人均化妆间面积不低于5平米。

（十一）做好入场检测登记。演出场所应当配备测量体温设施设备，并安排专人值守。观众进入演出场所必须佩戴口罩，测量体温，出示健康码。观众拒绝佩戴口罩或者体温异常的，应当拒绝其进入。

（十二）加强现场巡查。演出场所应当安排专人做好演出现场管理，提醒观众正确佩戴口罩，对号入座，保持安全距离。取消演出前后的现场互动环节。

四、员工健康管理

（十三）做好员工健康监测。按照当地要求做好员工健康管理，掌握员工出行轨迹等情况。员工上岗前要检测体温，出现发热、呼吸道等症状时，要暂停工作并及时报告。

（十四）指导员工做好个人防护。员工应当减少不必要外出，避免去人群聚集场所。组织对一线工作员工进行岗前培训，了解疫情防控相关知识，掌握新型冠状病毒个人防护知识、卫生健康习惯及疫情防控应急处置方法。

（十五）减少员工聚集。根据实际采取错时上下班、弹性工作制或居家办公方式。加强员工用餐管理，实行错峰就餐，有条件时使用餐盒、分散用餐。

五、异常情况处置

（十六）建立沟通机制。演出场所及演出主办方应当建立疫情应急沟通机制，畅通疫情上报通道，发现疫情情况应当及时向政府有关部门报告。

（十七）做好发现疫情时的应对处置。在演出现场如出现疑似疫情，演出场所应当立即启动应急预案，做好现场管理，避免恐慌，配合卫生健康部门采取隔离措施，做好清洁消毒等工作并暂时关闭场所。

六、保障措施

（十八）加强组织领导。各地文化和旅游行政部门要严格落实属地管理责任，提升防控和应急处置能力，加强与当地卫生健康部门之间的联动，确保恢复开放工作平稳有序。

（十九）加强监督检查。各地文化和旅游行政部门要加强对恢复开放的演出场所的巡查和监管力度，依法依规查处违法经营行为，维护市场经营秩序。

（二十）加强应急管理。各地文化和旅游行政部门要按照应急预案，明确疫情防控、安全突发事件应急措施和处置流程，开展排查、评估和宣传培训工作，及时发现隐患苗头，及时处置，发生异常情况及时上报并暂时关闭场所。

附件2

互联网上网服务营业场所恢复开放疫情防控措施指南

为贯彻落实习近平总书记关于统筹推进新冠肺炎疫情防控和经济社会发展工作的重要指示精神和《国务院应对新型冠状病毒感染肺炎疫情联防联控机制关于做好新冠肺炎疫情常态化防控工作的指导意见》（国发明电〔2020〕14号）要求，有序推进互联网上网服务营业场所（以下简称“上网服务场所”）恢复开放，制定本指南。

一、总体要求

（一）坚持常态防控。各地文化和旅游行政部门应当按照“属地原则”，严格遵守当地疫情防控指挥部门的要求，结合本地区实际情况，毫不放松抓紧抓实抓细各项防控工作，制定包含上网服务场所在内的疫情防控方案和应急预案，并及时进行动态调整，确保安全。

（二）坚持有序开放。在充分做好防疫措施的情况下，各地应当严格按照属地党委、政府统一部署，以县域为单位，分区分级，确定上网服务场所恢复开放时间和具体要求。疫情中高风险地区、疫情风险具有较大不确定性地区和境外输入压力较大地区，应当从严、审慎把控恢复开放时间。

（三）坚持预约限流。恢复开放的上网服务场所应当严格执行人员限流限量措施，要采取预约消费、错峰入场、间隔就坐等措施，并对场所内消费者数量实行动态管理，防范聚集性风险。

二、消费者保护

（四）落实戴口罩、检测登记制度。上网服务场所应当配备测量体温设施设备，并安排专人值守。消费者进入场所必须佩戴口罩、测量体温、出示健康码，严格落实实名登记制度。消费者不戴口罩或者体温异常的，场所应当拒绝其进入。

（五）实施预约限流措施。上网服务场所应当采取预约消费、错峰入场、间隔就坐等措施，限制上网人流。上网服务场所接纳消费者人数不得超过核定人数的50%，每个包间也不得超过核定人数的50%，严格执行限流限量要求。

（六）建立值守制度。上网服务场所应当安排专人监督进入场所的员工和消费者遵守相关防疫要求，及时对消费者进行疏导、分流，避免人群聚集，保持安全社交距离。

三、场所防控管理

（七）落实防控主体责任。上网服务场所应当落实防控主体责任，严格执行疫情防控规定，制定本场所防控工作制度和应急预案，把防控责任落实到具体个人。

（八）加强消毒通风。上网服务场所每日营业前，应当对场所内外公共部位（门厅、前台、独用楼道、独用电梯、楼梯、场所内卫生间、门把手等）进行全面清洁消毒。营业期间，及时对消费者使用过的显示器、键盘、耳机、鼠标等进行消杀，做到“一客一消毒”。场所应当保证充足的新风输入，定期对送风口、回风口进行消毒。

（九）配备防护物资。上网服务场所应当配备口罩、手套、洗手液、医用酒精、免洗手消毒剂、消毒湿巾等防护物资，在公共休息区、洗手间等区域配备洗手液、医用酒精等消毒物品，便于消费者和员工随时消毒清洁。

（十）加强疫情防控知识宣传。上网服务场所要利用官方网站、微信公众号、提示牌、电子显示屏等多种渠道，及时宣传疫情期间场所运营规则及疫情防控知识，提升员工及消费者的疫情防控意识。

（十一）排查消防隐患。上网服务场所恢复营业前应当积极配合有关部门，全面排查火灾等消防安全隐患，并按照《文化部关于落实安全生产责任加强文化市场安全生产工作的通知》（文市发〔2017〕5号）有关规定，加强场所日常检查，不符合安全条件的立整立改。

四、员工健康管理

（十二）做好员工健康监测。加强员工健康管理，掌握员工出行轨迹等情况。员工上班应当佩戴口罩，进行体温检测和健康询问，出现发热、呼吸道等症状，须暂停工作并立即就医。

（十三）减少员工聚集。加强员工用餐管理，鼓励实行错峰就餐、分散用餐；工作会议要进行科学管理；员工应当减少不必要外出，尽量避开密集人群，避免在公共场所长时间停留。

（十四）做好员工个人防护。组织对员工进行岗前培训，掌握常态化防控下的防护知识。养成勤洗手、戴口罩、公筷制等卫生习惯和健康生活方式。

五、异常情况处置

（十五）建立沟通机制。上网服务场所应当建立疫情应急沟通机制，畅通疫情上报通道，发现疫情应当及时向政府有关部门报告。

（十六）做好应急处置。上网服务场所如出现疑似疫情，应当立即启动应急预案，做好现场管理，避免恐慌，配合卫生健康部门采取隔离措施，做好清洁消毒等工作并暂时关闭场所。

六、保障措施

（十七）加强组织领导。各地文化和旅游行政部门要严格落实属地管理责任，提升防控和应急处置能力，加强与当地卫生健康部门之间的联动，确保恢复开放工作平稳有序。

（十八）加强监督检查。各地文化和旅游行政部门要加强对恢复开放的上网服务场所的巡查和监管力度，依法依规查处违法经营行为，切实保障消费者合法权益，维护市场经营秩序。

（十九）加强应急管理。各地文化和旅游行政部门要按照应急预案，明确疫情防控、安全突发事件应急措施和处置流程，开展排查、评估和宣传培训工作，及时发现隐患苗头，及时处置，发生异常情况及时上报并暂时关闭场所。

附件3

娱乐场所恢复开放疫情防控措施指南

为贯彻落实习近平总书记关于统筹推进新冠肺炎疫情防控和经济社会发展工作的重要指示精神和《国务院应对新型冠状病毒感染肺炎疫情联防联控机制关于做好新冠肺炎疫情常态化防控工作的指导意见》（国发明电〔2020〕14号）要求，有序推进娱乐场所恢复开放，制定本指南。

一、总体要求

（一）坚持常态防控。各地文化和旅游行政部门应当按照“属地原则”，严格遵守当地疫情防控指挥部门的要求，结合本地区实际情况，毫不放松抓紧抓实抓细各项防控工作，制定包含娱乐场所在内的疫情防控方案和应急预案，并及时进行动态调整，确保安全。

（二）坚持有序开放。在充分做好防疫措施的情况下，各地应当严格按照属地党委、政府统一部署，以县域为单位，分区分级，确定娱乐场所恢复开放时间和具体要求。疫情中高风险地区、疫情风险具有较大不确定性地区和境外输入压力较大地区，应当从严、审慎把控恢复开放时间。

（三）坚持预约限流。恢复开放的娱乐场所应当严格执行人员限流限量措施，实行预约消费、错峰入场等措施，并对场所内消费者数量实行动态监控，防范聚集性风险。

二、消费者保护

（四）落实戴口罩、检测登记制度。娱乐场所应当配备测量体温设施设备，并安排专人值守。消费者进入场所必须佩戴口罩、测量体温、出示健康码，并进行实名登记。消费者不戴口罩或者体温异常的，场所应当拒绝其进入。消费者在场所公共区域内应当科学佩戴口罩。

（五）实施预约限流措施。娱乐场所应当采取预约消费、错峰入场等措施，对消费者进行限流限量。歌舞娱乐场所接纳消费者人数不得超过核定人数的50%，每个包间接纳消费者人数也不得超过核定人数的50%；游艺娱乐场所内接纳消费者人数不得超过核定人数的50%。

（六）建立值守制度。娱乐场所应当安排专人监督进入场所的员工和消费者遵守相关防疫要求，及时对消费者进行疏导、分流，避免人群聚集，保持安全社交距离。

三、场所防控管理

（七）落实防控主体责任。娱乐场所应当落实防控主体责任，严格执行疫情防控规定，制定本场所防控工作制度和应急预案，把防控责任落实到具体个人。

（八）加强消毒通风。娱乐场所每日营业前，应当对场所内外公共部位进行全面消毒清洁。歌舞娱乐场所应当及时对消费者使用过的麦克风、点歌按钮、屏幕、座位桌台等设备消毒或更换；游艺娱乐场所应当及时对消费者使用过的设备按键、摇杆、代币及相关附属设备进行消毒或更换，做到“使用一次消毒一次”。场所应当经常开窗通风，保证空气流通。

（九）配备防护物资。娱乐场所应当配备口罩、手套、洗手液、医用酒精、免洗手消毒剂、消毒湿巾等防护物资，在公共休息区、洗手间等区域配备洗手液、医用酒精等消毒物品，便于消费者和员工随时消毒清洁。

（十）加强疫情防控知识宣传。娱乐场所要利用官方网站、微信公众号、提示牌、电子显示屏等多种渠道，及时宣传疫情期间场所运营规则及防控知识，提升员工及消费者的疫情防控意识。

（十一）排查消防隐患。娱乐场所恢复营业前应当积极配合有关部门，全面排查火灾等消防安全隐患，并按照《文化部关于落实安全生产责任加强文化市场安全生产工作的通知》（文市发〔2017〕5号）有关规定，加强场所日常检查，不符合安全条件的立整立改。

四、员工健康管理

（十二）做好员工健康监测。做好员工健康管理，掌握员工出行轨迹等情况。员工上班须佩戴口罩，进行体温检测和健康询问，出现发热、呼吸道症状时，应及时报告并立即就医。

（十三）减少员工聚集。加强员工用餐管理，鼓励实行错峰就餐、分散用餐；科学管理工作会议；员工应当减少不必要外出，尽量避开密集人群，避免在公共场所长时间停留。

（十四）指导员工做好个人防护。组织对员工进行岗前培训，掌握常态化防控下的防护知识。养成勤洗手、戴口罩、公筷制等卫生习惯和健康生活方式。

五、异常情况处置

（十五）建立沟通机制。娱乐场所应当建立疫情应急沟通机制，畅通疫情上报通道，发现疫情应当及时向政府有关部门报告。

（十六）做好发现疫情时的应对处置。娱乐场所如出现疫情疑似症状，应当立即启动应急预案，做好现场管理，避免恐慌，配合卫生健康部门采取隔离措施，做好清洁消毒等工作并暂时关闭场所。

六、保障措施

（十七）加强组织领导。各地文化和旅游行政部门要严格落实属地管理责任，提升防控和应急处置能力，加强与当地卫生健康部门之间的联动，确保恢复开放工作平稳有序。

（十八）加强监督检查。各地文化和旅游行政部门要加强对恢复开放的娱乐场所的巡查和监管力度，依法依规查处违法经营行为，切实保障消费者合法权益，维护市场经营秩序。

（十九）加强应急管理。各地文化和旅游行政部门要按照应急预案，明确疫情防控、安全突发事件应急措施和处置流程，开展排查、评估和宣传培训工作，及时发现隐患苗头，及时处置，发生异常情况及时上报并暂时关闭场所。

文化和旅游部市场管理司

2020年5月12日

1. 人社部、财政部：实施企业稳岗扩岗专项支持计划

各省、自治区、直辖市及新疆生产建设兵团人力资源社会保障厅（局）、财政厅（局）：

　　为有效应对国内外疫情形势和经济下行压力对就业的影响，做好常态化疫情防控中的稳就业工作，人力资源社会保障部、财政部决定启动实施企业稳岗扩岗专项支持计划。有关事项通知如下：

　　一、总体要求

　　坚决贯彻中央统筹推进疫情防控和经济社会发展工作部署，强化底线思维，注重精准施策，坚持援企、稳岗、扩就业、保民生并举，用足用好失业保险基金、职业技能提升行动专账资金，大力实施稳岗返还、以工代训，支持企业稳定岗位，鼓励企业吸纳就业，保障劳动者基本生活，努力保持就业局势和社会大局稳定。

　　二、政策举措

　　（一）加大稳岗返还力度。加快落实失业保险稳岗返还政策，支持参保企业不裁员、少裁员。其中，对中小微企业，2020年12月31日前返还标准最高可提至企业及其职工上年度缴纳失业保险费的100%。提高返还标准后，各级失业保险经办机构要尽快补发2020年度返还资金。对面临暂时性生产经营困难且恢复有望、坚持不裁员或少裁员的参保企业，返还标准可按不超过6个月的当地月人均失业保险金和参保职工人数确定，或按不超过3个月的企业及其职工应缴纳社会保险费确定。实施企业稳岗返还的统筹地区上年失业保险基金滚存结余应具备12个月以上备付能力，实施困难企业稳岗返还的统筹地区备付能力应达到24个月以上，对于备付能力不足的统筹地区，要充分发挥省级调剂金作用，帮助当地实施困难企业稳岗返还政策，尽可能让符合条件的企业都能享受政策支持。

　　（二）拓宽以工代训范围。

　　支持企业面向新吸纳劳动者开展以工代训，扩岗位、扩就业。对中小微企业吸纳就业困难人员、零就业家庭成员、离校两年内高校毕业生、登记失业人员就业，并开展以工代训的，可根据吸纳人数给予企业职业培训补贴。

　　支持困难企业开展以工代训，稳岗位、保生活。对受疫情影响出现生产经营暂时困难导致停工停业的中小微企业，组织职工开展以工代训的，可根据组织以工代训人数给予企业职业培训补贴。

　　各地可结合实际情况，将受疫情影响较大的外贸、住宿餐饮、文化旅游、交通运输、批发零售等行业补贴范围扩展到各类企业。补贴资金主要用于开展以工代训、职工生活补助等支出。符合条件的企业申请以工代训职业培训补贴，应向当地人力资源社会保障部门提供以工代训人员花名册、当月发放工资银行对账单（其中停工停产企业需提供上一季度发放工资银行对账单）。经当地人力资源社会保障部门审核通过后，按规定每月将补贴资金支付到企业在银行开立的基本账户。补贴标准由省级人力资源社会保障、财政部门确定，补贴期限最长不超过6个月，所需资金从职业技能提升行动专账资金中列支。以工代训职业培训补贴政策受理期限截止到2020年12月31日，原有以工代训政策执行期限不变。

　　三、工作要求

　　（一）优化经办服务。各地要梳理发布专项支持计划的政策清单、申办流程、补贴标准、服务机构及联系方式、监督投诉电话等，结合本地区重点关注企业清单宣介政策、了解困难、做好帮扶。要精简申领证明材料，对同一企业同时申请多项政策的推行“打包”办理，不得要求重复提交材料。对稳岗返还政策，稳岗返还金额可依据征缴机构提供的上年度缴费记录按规定确定，不再要求提供职工花名册、工资表等；“生产经营活动应符合国家及所在区域产业结构调整和环保政策”的审核可采用企业承诺方式。要提高审核发放效率，做到随报随审，不得设定集中申报期和资金拨付期。要加快建立补贴资金网上申领渠道，推广“不见面”服务，努力实现补贴受理审核发放全程网办。

　　（二）强化资金保障监管。各地要加大以工代训职业培训补贴资金保障力度，确保政策落实。要按月监测失业保险基金运行状况，加强情况预判和适时调控，确保基金收支平衡和安全可持续。建立健全失业保险基金、职业技能提升行动专账资金监管机制，向社会公示享受政策的单位、额度等情况，加强监督检查和专项审计，对违规使用、骗取套取资金的要依法依规严惩，涉嫌犯罪的及时移交司法机关处理。

（三）加强组织领导。实施稳岗扩岗专项支持计划是当前稳就业、促就业、防失业的重要抓手，各地人力资源社会保障、财政部门要高度重视、精心组织。要制定实施方案，结合本地区就业工作需要，细化实化稳岗返还、以工代训政策，合理确定政策享受对象，明确操作流程和补贴标准。要依托现有信息系统，将以工代训政策实施情况纳入技能提升行动统计范围，按月上报政策落实情况。要强化宣传引导，充分运用各类媒体，及时向社会宣传专项支持计划政策举措、进展成效。专项支持计划实施情况将作为本年度稳就业工作相关督查、技能提升行动落实情况考核的重要内容。

各地实施专项支持计划的有关情况及工作中发现的困难问题，要及时报送人力资源社会保障部、财政部。

人力资源社会保障部 财政部

2020年5月9日

1. 发改委等10部门：应对疫情统筹做好支持台资企业发展和推进台资项目有关工作 发改厅〔2020〕755号

各省、自治区、直辖市、新疆生产建设兵团发展改革委、台办、工业和信息化主管部门、财政厅、人力资源社会保障厅、自然资源主管部门、商务厅、银保监局、证监局，中国人民银行上海总部，各分行、营业管理部，省会（首府）城市中心支行：

为贯彻落实党中央、国务院关于统筹推进新冠肺炎疫情防控和经济社会发展工作的决策部署，进一步落细落实《关于促进两岸经济文化交流合作的若干措施》和《关于进一步促进两岸经济文化交流合作的若干措施》，现就相关事项通知如下。

一、持续帮扶台资企业复工复产。根据地方统筹做好疫情防控和复工复产总体安排，协助解决台资企业在生产经营过程中遇到的供应链协同、达产等方面困难，确保台资企业同等享有中央和地方出台的各类援企稳岗政策。

二、统筹协调推进重大台资项目。参照重大外资项目有关机制协调推进重大台资项目，密切跟踪在谈项目进展，充分发挥各类涉台产业园区等发展平台优势，出台具有竞争力和针对性的招商引资政策，加强各级联动和部门协同，建立项目绿色通道，做好工程建设保障、审批事项衔接，开展全流程对接服务，促进台资项目加快落地见效，为台资企业参与本地重大项目提供同等待遇。

三、积极支持台资企业增资扩产。根据地方实际，在法定权限内研究出台用地、用能、用工等方面具体措施，为台资企业增资扩产提供政策支持。全面落实境外投资者以分配利润直接投资暂不征收预提所得税政策规定，支持台资企业以分配利润进行再投资。支持台资企业参与海南自由贸易港建设、粤港澳大湾区建设、长三角一体化发展等区域发展战略和各地自贸试验区建设。支持有产业转移需求的东部地区台资企业优先向中西部和东北地区转移。

四、促进台资企业参与新型和传统基础设施建设。支持台资企业发挥自身优势，与大陆企业共同研发、共建标准、共创品牌、共拓市场，以多种形式参与大陆5G、工业互联网、人工智能、物联网等新型基础设施的研发、生产和建设。对台资企业和台湾高端人才从事新型基础设施相关的集成电路、工业软件、信息系统等，提供与大陆企业和同胞同等待遇。继续支持台资企业参与交通、能源、水利等传统基础设施建设。

五、支持台资企业稳外贸。鼓励台资企业发展跨境电商，开展线上供采对接，扩大出口业务。指导台资企业充分利用中欧班列开展进出口贸易。落实相关纾困政策，支持台资加工贸易企业统筹内外贸发展。进一步扩大出口信用保险对台资企业的覆盖面。

六、有效引导台资企业拓展内销市场。支持台资企业适应大陆“互联网+”发展和消费升级趋势，借助大陆电商平台开展线上市场营销推广，拓宽对接内需市场的渠道，充分挖掘大陆市场潜力。

七、全面落实税费减免政策。落实好阶段性减免企业社会保险费政策，对符合条件的台资企业按规定免征或减半征收社会保险单位缴费部分。有条件的地方可研究出台减免物业租金、降低生产要素成本、加大企业职工技能培训补贴等支持政策，符合条件的台资企业可同等申请享受。

八、强化金融支持台资企业疫情防控和复工复产。落实金融支持防控疫情相关政策，为受疫情影响较大的台资企业提供优惠的金融服务。发挥国有控股小额贷款公司等地方金融组织的作用，加大信贷支持力度，满足台资企业差异化金融需求。鼓励符合条件的台资企业在大陆上市融资，为符合条件的科创型台资企业在科创板上市提供支持。鼓励台湾金融机构把握大陆金融领域自主开放新机遇，参与两岸金融合作。

九、充分保障台资项目合理用地需求。对于台资企业复工复产、重大投资项目，坚持“要素跟着项目走”，合理安排用地计划指标，按照《国务院关于授权和委托用地审批权的决定》（国发〔2020〕4号）等政策文件和“放管服”要求依法依规做好用地保障服务。鼓励探索推行“标准地”供应改革，通过区域评价统一化、开发标准公开化、权利义务合同化、履约监管闭环化等方式，加快台资项目落地。

十、有力支持台资中小企业发展。充分发挥各级中小企业公共服务示范平台作用，通过线上培训等形式，为台资中小企业提供政策、技术、管理等方面服务。积极帮助台资中小企业解决受疫情影响造成的合同履行、劳动关系等法律问题。鼓励台资中小企业利用好本级相关资金等支持政策。

十一、主动做好台资企业服务工作。对台资企业一视同仁，着力为台资企业办实事、做好事、解难事。加强与本地台资企业协会、重点台资企业等沟通交流，宣传解读有关政策法规，通报疫情防控和复工复产有关工作要求，认真听取台资企业意见建议，积极回应台资企业关切诉求，妥善化解涉台纠纷，切实维护台资企业合法权益。

各地发展改革委、台办要会同工业和信息化主管部门、财政厅、人力资源社会保障厅、自然资源主管部门、商务厅、人民银行、银保监局、证监局等共同落实好上述工作部署，支持台资企业应对疫情、复工复产和投资发展，防范化解潜在风险，持续促进两岸经济交流合作，深化两岸融合发展。

国家发展改革委 国务院台办

工业和信息化部 财 政 部

人力资源社会保障部 自然资源部

商 务 部 人民银行

银 保 监 会 证 监 会

2020年5月15日

1. 最高人民法院 司法部 文化和旅游部 关于依法妥善处理涉疫情旅游合同纠纷有关问题的通知 法〔2020〕182号

各省、自治区、直辖市高级人民法院，解放军军事法院，新疆维吾尔自治区高级人民法院生产建设兵团分院：

各省、自治区、直辖市高级人民法院、司法厅（局）、文化和旅游厅（局），解放军军事法院，新疆维吾尔自治区高级人民法院生产建设兵团分院、新疆生产建设兵团司法局、新疆生产建设兵团文化体育广电和旅游局：

　　为贯彻落实党中央关于统筹推进疫情防控和经济社会发展工作部署，扎实做好“六稳”工作，落实“六保”任务，依法妥善化解涉疫情旅游合同纠纷，切实保障在常态化疫情防控中加快推进生产生活秩序全面恢复，抓紧解决复工复产面临的困难和问题，力争把疫情造成的损失降到最低限度，保障人民群众生命安全和身体健康，现将有关事项通知如下。

　　一、处理涉疫情旅游合同纠纷的基本要求

　　1.增强大局意识。旅游业是国民经济的重要支柱产业，推动旅游业平稳健康发展，对于促进经济平稳增长、持续改善民生具有重大意义。新冠肺炎疫情给旅游行业造成巨大冲击，由此导致旅游合同纠纷数量激增。文化和旅游部门、司法行政部门、人民法院要充分认识妥善处理旅游合同纠纷的重要意义，增强责任意识，发挥好行政机关与审判机关化解纠纷的职能作用，协同处理涉疫情旅游合同纠纷，为促进旅游业与经济社会持续发展、维护社会稳定提供服务和保障。

　　2.妥善化解纠纷。文化和旅游部门、司法行政部门、人民法院应当始终以法律为准绳，客观、全面、公平认定疫情在具体案件中对旅游经营者、旅游者造成的影响，在明确法律关系性质和合同双方争议焦点的基础上，平衡各方利益，兼顾旅游者权益保护与文化旅游产业发展，积极、正面引导旅游经营者和旅游者协商和解、互谅互让、共担风险、共渡难关，妥善化解纠纷，争取让绝大多数涉疫情旅游合同纠纷以非诉讼方式解决，维护良好的旅游市场秩序。

　　二、建立健全多元化解和联动机制

　　3.建立旅游合同纠纷多元化解机制。文化和旅游部门、司法行政部门、人民法院应当充分发挥矛盾纠纷多元化解机制作用，坚持把非诉讼纠纷解决机制挺在前面，强化诉源治理、综合治理，形成人民调解、行政调解、司法调解优势互补、对接顺畅的调解联动工作机制。文化和旅游部门、人民调解组织应当充分发挥调解职能作用，及时组织调解。司法行政部门应当组织律师积极参与旅游合同纠纷调解，充分发挥律师调解专业优势。当事人起诉的，人民法院可以征得当事人同意后，通过人民法院调解平台，委派或者委托特邀调解组织、特邀调解员进行调解。对调解不成的简易案件，人民法院应当速裁快审，努力做到能调则调，当判则判，及时定分止争。

　　4.畅通矛盾纠纷化解的协作对接渠道。文化和旅游部门、司法行政部门、人民法院应当发挥主观能动性，在兼顾法、理、情的基础上主动服务、创新服务。各部门、各单位之间主动加强沟通协调，共享信息，相互支持配合，形成工作合力。文化和旅游部门、司法行政部门对投诉、调解中反映出的新问题应及时与人民法院沟通。人民法院与当地文化和旅游部门、司法行政部门共同研判纠纷化解思路，确保纠纷处理的社会效果和法律效果统一。

　　5.充分发挥非诉讼纠纷化解机制作用。文化和旅游部门指导旅游经营者通过网络、电话、面谈等多种沟通方式加速涉疫情旅游合同纠纷的处理，简化流程、缩短时间；指导旅游经营者对员工进行培训，有效提升处理投诉人员业务水平，做好解释和安抚工作；做好涉疫情旅游合同纠纷的投诉处理工作，引导投诉人与被投诉人达成和解。人民调解组织可引导当事人选择人民调解调处矛盾纠纷并安排业务精通的调解员进行调解；律师调解工作室（中心）接到人民法院委派、委托调解或者接到当事人调解申请后，积极组织具有相应专业特长的律师调解员进行调解。当事人达成调解协议后，能够即时履行的即时履行，不能即时履行的明确履行时间，并引导当事人对调解协议申请司法确认。人民法院通过司法审查、司法确认等方式为非诉纠纷解决提供支持。

　　6.提供便捷高效的诉讼服务。人民法院开辟旅游合同纠纷诉讼绿色通道。有条件的地方可以充分发挥“旅游巡回法庭”在基层一线的作用，及时调处旅游合同纠纷。充分运用在线诉讼平台，开展线上调解、线上审判活动，切实将“智慧法院”用于解决群众实际困难。充分发挥小额速裁程序优势，通过快捷高效的法律服务，实现涉疫情旅游合同案件的快立、快审、快结。

　　三、依法妥善处理涉疫情旅游合同纠纷

　　7.严格执行法律政策。依据民法总则、合同法、旅游法，最高人民法院关于审理旅游纠纷案件适用法律若干问题的规定、关于依法妥善审理涉新冠肺炎疫情民事案件若干问题的指导意见（一），以及文化和旅游部办公厅印发的关于全力做好新型冠状病毒感染的肺炎疫情防控工作暂停旅游企业经营活动的紧急通知等相关法律、司法解释、政策，妥善处理涉疫情旅游合同的解除、费用负担等纠纷。

　　8.积极引导变更旅游合同。结合纠纷产生的实际情况，准确把握疫情或者疫情防控措施与旅游合同不能履行之间的因果关系，积极引导当事人在合理范围内调整合同中约定的权利义务关系，包括延期履行合同、替换为其他旅游产品，或者将旅游合同中的权利义务转让给第三人等合同变更和转让行为，助力旅游企业复工复产。旅游经营者与旅游者均同意变更旅游合同的，除双方对旅游费用分担协商一致的以外，因合同变更增加的费用由旅游者承担，减少的费用退还给旅游者。

　　9.慎重解除旅游合同。疫情或者疫情防控措施直接导致合同不能履行的，旅游经营者、旅游者应尽可能协商变更旅游合同。旅游经营者、旅游者未就旅游合同变更达成一致且请求解除旅游合同的，请求解除旅游合同的一方当事人应当举证证明疫情或者疫情防控措施对其履行合同造成的障碍，并已在合同约定的或合理的期间内通知合同相对人。旅游合同对解除条件另有约定的遵循合同约定。

　　10.妥善处理合同解除后的费用退还。因疫情或者疫情防控措施导致旅游合同解除的，旅游经营者与旅游者应就旅游费用的退还进行协商。若双方不能协商一致，旅游经营者应当在扣除已向地接社或者履行辅助人支付且不可退还的费用后，将余款退还旅游者。旅游经营者应协调地接社和履行辅助人退费，并提供其已支付相关费用且不能退回的证据，尽力减少旅游者因疫情或者疫情防控措施受到的损失。旅游经营者主张旅游者承担其他经营成本或者经营利润的，不予支持。旅游经营者应及时安排退费，因客观原因导致不能及时退费的，应当及时向旅游者作出说明并出具退款期限书面承诺。

　　11.妥善处理安全措施和安置费用的负担。因疫情影响旅游者人身安全，旅游经营者应当采取相应的安全措施，因此支出的费用，由旅游经营者与旅游者分担。因疫情或者疫情防控措施造成旅游者滞留的，旅游经营者应当采取相应的合理安置措施，因此增加的食宿费用由旅游者承担，增加的返程费用由旅游经营者与旅游者分担。

　　12.妥善认定减损和通知义务。旅游经营者、履行辅助人与旅游者均应当采取措施减轻疫情或疫情防控措施对合同当事人造成的损失，为防止扩大损失而支出的合理费用，可依公平原则予以分担。旅游经营者和旅游者应将受疫情或者疫情防控措施影响不能履行合同的情况及时通知对方，以减轻对方的损失。旅游经营者或旅游者未履行或未及时履行减损和通知义务的，应承担相应责任。

　　四、做好法律政策宣传工作

　　13.主动宣传法律、政策和典型案例。文化和旅游部门、司法行政部门、人民法院应当加大对涉疫情法律法规、政策文件等的解释和宣传力度，通过报纸、电视台、电台及各类新媒体解答涉疫情旅游合同纠纷热点问题，增强民众依法处理纠纷的自觉性，倡导旅游者理性维权。不断总结经验，宣传典型案例，提升涉疫情旅游合同矛盾纠纷多元化解机制在全社会的影响力和公信力。

　　14.共同维护社会稳定。涉疫情旅游合同纠纷牵涉面广、群体效应强，文化和旅游部门、司法行政部门、人民法院应密切关注各类媒体报道及投诉过程中的特殊情况，预防发生负面舆情和群体性事件，努力为统筹推进疫情防控和经济社会发展工作提供更加有力的服务和保障。

　　最高人民法院 司法部 文化和旅游部

2020年7月13日

1. 市场监管总局关于支持疫情防控和复工复产反垄断执法的公告 市场监管总局公告2020年第13号

公平竞争的市场环境是企业复工复产、全面恢复生产生活秩序、保持经济平稳运行的重要基础。为进一步贯彻落实党中央、国务院关于统筹推进疫情防控和经济社会发展工作的决策部署，市场监管总局将强化使命担当、积极主动作为，更好发挥反垄断监管职能，全力营造公平竞争的市场环境，支持疫情防控和复工复产。现就有关事项公告如下：

一、依法加快审查涉疫情防控和复工复产的经营者集中案件

疫情防控期间，经营者集中申报继续采取非现场方式进行。申报人可以将申报材料及补充问题回复电子版发送至市场监管总局（反垄断局）电子邮箱，不方便网上办理的可以邮寄；受理通知、补充文件清单、立案通知及审查决定将通过电子邮箱或传真送达，保障经营者集中反垄断审查工作正常进行。

市场监管总局已建立绿色审查通道，对医药制造、医疗仪器设备及器械制造、食品制造、交通运输、批发零售等与疫情防控和基本民生密切相关领域，受疫情影响较重的餐饮、住宿、旅游等行业，以及为复工复产实施的经营者集中，进行快速审查。通过电话会议、电子邮件等非现场形式，主动与经营者加强对接、密切沟通，为经营者提交完备申报文件、资料提供全程指导。在基本文件资料齐全后第一时间立案审查，依法加快审查工作，切实提高审查效率，为经营者节约交易时间，降低交易成本，支持疫情防控和复工复产。

二、依法豁免涉疫情防控和复工复产的经营者合作协议

鼓励经营者积极参与疫情防控，加快复工复产。经营者为疫情防控和复工复产达成的有利于技术进步、增进效率、实现社会公共利益和保护消费者利益的协议，如为在药品疫苗、检测技术、医疗器械、防护设备等领域改进技术、研究开发新产品；为提高防控物资产品质量、降低成本、增进效率而统一产品规格、标准或者实行专业化分工；为实现救灾救助等社会公共利益；为提高中小经营者经营效率、增强中小经营者竞争力等，符合《反垄断法》规定的，市场监管总局将依法给予豁免。

三、依法从严从重从快查处妨碍疫情防控和复工复产的垄断行为

经营者要严格依法经营。各省、自治区、直辖市市场监管部门要依法从严从重从快查处妨碍疫情防控和复工复产、损害消费者利益的垄断行为，重点查处口罩、药品、医疗器械、消杀用品等防控物资及原辅材料，供水、供电、供气等公用事业以及与其他民生密切相关行业和领域的经营者达成、实施的协同涨价、限制产量、分割市场、联合抵制、固定或限定转售价格等垄断协议，以及不公平高价、拒绝交易、限定交易、搭售或附加不合理交易条件、差别待遇等滥用市场支配地位行为，为疫情防控和复工复产营造公平竞争市场环境，切实保护消费者利益。典型案例及时予以公开曝光。

四、加强公平竞争审查政策支持

加大对有利于疫情防控和复工复产政策措施的支持力度，指导政策制定机关科学开展公平竞争审查。既要防止出台含有指定交易、固定价格、分割市场等排除、限制竞争内容的政策措施；也要简化审查流程，快速审查通过有利于保障重要防控物资生产供应，帮扶受疫情影响严重的行业和地区经营者恢复生产、渡过难关，扩大消费、稳定就业的政策措施。对排除和限制市场竞争不严重，且有助于实现扶贫开发、救灾救助等社会保障目的和社会公共利益的政策措施，应明确实施期限。

各级市场监管部门要加强与政策制定机关沟通交流，及时答复政策制定机关的意见征询，支持政策制定机关切实提高审查效率，做好公平竞争审查工作，更好服务疫情防控和复工复产。

五、积极做好对经营者的反垄断合规指导

加强经营者反垄断合规指导，支持经营者根据自身业务状况、规模大小、行业特性等，建立健全反垄断法律合规制度，全面、有效开展反垄断合规管理工作。各省、自治区、直辖市市场监管部门要主动靠前服务、积极担当作为，认真做好反垄断合规指引，引导经营者自觉遵守反垄断法律法规，避免与其他经营者达成垄断协议，防止从事滥用市场支配地位行为，依法实施经营者集中，公平参与市场竞争。

支持行业协会加强行业自律，积极引导本行业的经营者依法竞争，主动维护市场竞争秩序。

六、及时响应疫情防控和复工复产反垄断诉求

疫情防控期间，任何单位和个人发现经营者从事垄断协议、滥用市场支配地位等涉嫌垄断行为，或者行政机关和法律、法规授权的具有管理公共事务职能的组织滥用行政权力，排除、限制竞争，或者违反公平竞争审查标准出台政策措施的，可以向市场监管总局投诉和举报。

经营者和消费者可以通过电话、电子邮件、传真、邮寄、网站公众留言等多种方式，向市场监管总局进行与疫情防控和复工复产相关的反垄断业务咨询、豁免申请和投诉举报。市场监管总局将在2个工作日内与联系人进行联系，积极回应经营者和消费者诉求，全力保护市场公平竞争，维护消费者利益，为疫情防控和复工复产提供有力支持。

市场监管总局（反垄断局）联系电话：010-88650571，88650592；传真：010-68060820；电子邮箱：fldj@samr.gov.cn；邮寄地址：北京市西城区三里河东路8号，邮编100820；公众留言网址：http://www.samr.gov.cn。

各级市场监管部门要切实加强组织领导，强化责任担当，紧密结合各地实际，将各项政策措施落实落细落到位，维护公平竞争市场环境，更好服务疫情防控，支持企业复工复产。

市场监管总局

2020年4月4日

1. 慈善组织、红十字会依法规范开展疫情防控慈善募捐等活动指引

新冠肺炎疫情发生以来，广大慈善组织、红十字会积极响应中央号召，有序参与疫情防控工作，成为抗击疫情的重要力量。为进一步依法规范慈善组织、红十字会为支持疫情防控工作开展的慈善募捐（以下简称募捐）等活动，现就有关事项发布指引如下：

一、慈善组织、红十字会应当严格遵守《中华人民共和国慈善法》、《中华人民共和国红十字会法》、《中华人民共和国公益事业捐赠法》、《中华人民共和国突发事件应对法》、《慈善组织公开募捐管理办法》、《慈善组织信息公开办法》等法律法规，依法规范开展募捐等活动。

二、依法取得公开募捐资格的慈善组织、红十字会可以面向社会公众开展募捐活动。不具有公开募捐资格的组织和个人可以与具有公开募捐资格的慈善组织、红十字会合作开展公开募捐活动，由该具有公开募捐资格的慈善组织、红十字会管理募得款物。开展募捐活动要坚持自愿原则，不得摊派或变相摊派。

三、慈善组织、红十字会应当在开展公开募捐活动的十日前将募捐方案报送民政部门备案。因情况紧急，未能在开展公开募捐活动前办理募捐方案备案的，应当在公开募捐活动开始后十日内补办备案手续。

四、慈善组织、红十字会通过互联网开展公开募捐活动的，应当在民政部指定的互联网公开募捐信息平台发布公开募捐信息，并可以同时在以本组织名义开通的门户网站、官方微博、官方微信、移动客户端等网络平台发布公开募捐信息。

五、慈善组织、红十字会应当按照疫情防控要坚持全国一盘棋的要求，在各地联防联控机制统一领导和部署下，根据疫情防控工作的实际需要，充分尊重和体现捐赠人的意愿，合理分配使用捐赠款物。除定向捐赠外，重点支持湖北省特别是武汉市等疫情严重地区。

六、慈善组织、红十字会要加快工作节奏，建立快速便捷的分配通道，按照各地联防联控机制的要求，与疫情防控定点诊疗医院等接收单位有效对接，做到“快进快出、物走账清”，第一时间将捐赠款物送到疫情防控工作一线，防止捐赠款物积压。

七、慈善组织、红十字会接收的定向捐赠物资，按捐赠人意愿委托送达或由捐赠人直接送达最终使用单位。对于可以直接送达最终使用单位的非定向捐赠物资，由慈善组织、红十字会协调捐赠人直接送达最终使用单位或指定地点。

八、慈善组织、红十字会要做好捐赠款物收支情况的数据汇总和信息公开工作，及时向社会公布捐赠款物的接收分配情况，特别是重点疫情防控物资的接收分配明细，主动接受社会监督。

九、慈善组织、红十字会要采取审核捐赠协议、查验相关证明等方式，对受赠的药品、医疗器械等物资严格把关，确保来源合法、符合标准、安全有效、精准发放。不允许捐赠人以定向捐赠的名义指定其利害关系人作为受益人。

社会公众可通过全国慈善信息公开平台（中文域名：“慈善中国”，网址：cishan.chinanpo.gov.cn）或相关民政部门官方网站，了解募捐主体的公开募捐资格、公开募捐活动备案等情况，合理选择参与慈善项目。如发现慈善组织、红十字会存在违法违规情形的，可向各级民政部门投诉、举报。民政部门将加强监督指导，对违法违规行为严肃处理。

民政部社会组织管理局

民政部慈善事业促进和社会工作司

2020年2月14日

1. 财政部、税务总局：印发《关于电影等行业税费支持政策的公告》 财政部 税务总局公告2020年第25号

为支持电影等行业发展，现将有关税费政策公告如下：

一、自2020年1月1日至2020年12月31日，对纳税人提供电影放映服务取得的收入免征增值税。

本公告所称电影放映服务，是指持有《电影放映经营许可证》的单位利用专业的电影院放映设备，为观众提供的电影视听服务。

二、对电影行业企业2020年度发生的亏损，最长结转年限由5年延长至8年。

电影行业企业限于电影制作、发行和放映等企业，不包括通过互联网、电信网、广播电视网等信息网络传播电影的企业。

三、自2020年1月1日至2020年12月31日，免征文化事业建设费。

四、本公告发布之日前，已征的按照本公告规定应予免征的税费，可抵减纳税人和缴费人以后月份应缴纳的税费或予以退还。

财政部 税务总局

2020年5月13日

1. 文化和旅游部办公厅关于暂退部分旅游服务质量保证金支持旅行社应对经营困难的通知

各省、自治区、直辖市文化和旅游厅（局），新疆生产建设兵团文化体育广电和旅游局：

为贯彻落实习近平总书记关于新型冠状病毒感染的肺炎疫情防控工作的重要指示精神，进一步做好文化和旅游系统疫情防控工作，支持旅行社积极应对当前经营困难，履行社会责任，文化和旅游部决定向旅行社暂退部分旅游服务质量保证金（以下简称保证金）。现就有关事项通知如下：

一、范围和标准

暂退范围为全国所有已依法交纳保证金、领取旅行社业务经营许可证的旅行社，暂退标准为现有交纳数额的80%。被法院冻结的保证金不在此次暂退范围之内。

二、交还期限

自通知印发之日起两年内，接受暂退保证金的各旅行社应在2022年2月5日前将本次暂退的保证金如数交还。

三、有关要求

各地文化和旅游行政部门要依据有关规定抓紧组织实施，自本通知印发之日起，一个月之内完成暂退保证金工作；要建立工作台账，指导和督促相关旅行社企业在全国旅游监管服务平台及时完成保证金信息变更和备案工作；要加强监管，对未按期交还保证金的旅行社要依法依规查处，并记入企业信用档案。各地于2020年3月15日前将保证金退还情况报送文化和旅游部市场管理司。

特此通知。

文化和旅游部办公厅

2020年2月5日

1. 发改委：应对新冠肺炎疫情进一步帮扶服务业小微企业和个体工商户缓解房屋租金压力 发改投资规〔2020〕734号

各省、自治区、直辖市人民政府，新疆生产建设兵团：

  服务业小微企业和个体工商户在繁荣市场、促进就业、维护稳定方面发挥着重要作用。为落实党中央、国务院决策部署，统筹推进新冠肺炎疫情防控和经济社会发展工作，进一步帮扶服务业小微企业和个体工商户缓解房屋租金压力，经国务院同意，现提出以下意见：

  一、总体要求

  受新冠肺炎疫情影响，服务业小微企业和个体工商户房屋租金成本支出压力大，国有房屋租赁相关主体要带头履行社会责任、主动帮扶小微企业，鼓励非国有房屋租赁相关主体在平等协商的基础上合理分担疫情带来的损失。各级政府要加大政策支持力度，金融机构视需要给予适当支持。房屋租金减免和延期支付政策主要支持经营困难的服务业小微企业和个体工商户，优先帮扶受疫情影响严重、经营困难的餐饮、住宿、旅游、教育培训、家政、影院剧场、美容美发等行业。

  二、实施房屋租金减免

  （一）推动对承租国有房屋（包括国有企业和政府部门、高校、研究院所等行政事业单位房屋，下同）用于经营、出现困难的服务业小微企业和个体工商户，免除上半年3个月房屋租金。转租、分租国有房屋的，要确保免租惠及最终承租人。

  （二）中央所属国有房屋（包括有关部门、中央企业、中央高校和研究院所等所属国有房屋）出租的，执行房屋所在地对出现经营困难的服务业小微企业和个体工商户的房屋租金支持政策。因减免租金影响国有企事业单位业绩的，在考核中根据实际情况予以认可。

  （三）对承租非国有房屋用于经营、出现困难的服务业小微企业和个体工商户，鼓励出租人考虑承租人实际困难，在双方平等协商的基础上，减免或延期收取房屋租金。

  三、完善财税优惠政策

  （四）地方政府要统筹各类财政资金（包括中央转移支付、地方自有财力等），根据当地实际情况对承租非国有房屋的服务业小微企业和个体工商户给予适当帮扶。

  （五）对服务业小微企业和个体工商户减免租金的出租人，可按现行规定减免当年房产税、城镇土地使用税。落实好增值税小规模纳税人税收优惠政策。

  四、加大金融支持力度

  （六）引导国有银行业金融机构对服务业小微企业和个体工商户，年内增加优惠利率小额贷款投放，专门用于支付房屋租金。银行业金融机构要加大对服务业小微企业和个体工商户的信贷投放力度，用好普惠金融定向降准、再贷款等政策，以优惠利率给予资金支持。

  （七）对实际减免服务业小微企业和个体工商户房屋租金的出租人，引导国有银行业金融机构视需要年内给予基于房屋租金收入的优惠利率质押贷款支持。鼓励银行业金融机构开发推广基于房屋租金收入的质押贷款产品。

  （八）对服务业小微企业和个体工商户以及实际减免房屋租金的出租人的生产经营性贷款，受疫情影响严重、年内到期还款困难的，银行业金融机构与客户协商，视需要通过展期、续贷等方式，给予临时性还本付息安排。

  五、稳定房屋租赁市场

  （九）鼓励将国有房屋直接租赁给服务业小微企业和个体工商户。对确需转租、分租的，不得在转租、分租环节哄抬租金。

  （十）在受疫情影响缴纳租金确有困难时，承租人与出租人在遵守合同协议的前提下，本着平等协商的原则稳定租赁关系，出租人可适当减免或延期收取租金。

  （十一）地方政府及相关部门建立健全房屋租赁纠纷调处机制，引导租赁双方协商解决因疫情引发的租赁纠纷。帮扶服务业小微企业和个体工商户渡过难关，尽快复工复产、复商复市，推动服务业小微企业和个体工商户恢复正常生产秩序。

  （十二）有关部门按照职责分工，抓好相关政策措施落实工作，并加强督促指导。各地区要进一步细化完善实施方案，抓紧把相关政策措施落到实处，可根据实际情况进一步加大支持力度，帮扶服务业小微企业和个体工商户缓解房屋租金压力。

  各地区、各有关部门要充分认识应对新冠肺炎疫情进一步帮扶服务业小微企业和个体工商户缓解房屋租金压力的重要意义，进一步把思想认识行动统一到党中央、国务院决策部署上来，明确职责分工，完善工作机制，落实工作责任，加大工作力度，确保各项要求尽快落到实处。

国家发展改革委

住房城乡建设部

财  政  部

商  务  部

人 民 银 行

国  资  委

税 务 总 局

市场监管总局

2020年5月9日

1. 卫健委：印发中小学校和托幼机构新冠肺炎疫情防控技术方案

各省、自治区、直辖市及新疆生产建设兵团卫生健康委、教育厅（教委、教育局）：

　　为科学指导中小学校、托幼机构做好开学复课疫情防控工作，有序推进复学复课，针对中小学校、托幼机构学习生活环境状况、学生不同年龄段特征等情况，我们组织制定了《中小学校新冠肺炎疫情防控技术方案（修订版）》和《托幼机构新冠肺炎疫情防控技术方案（修订版）》。现印发给你们，请参照执行。

　　附件：1.中小学校新冠肺炎疫情防控技术方案（修订版）

　　　　　2.托幼机构新冠肺炎疫情防控技术方案（修订版）

国家卫生健康委办公厅            教育部办公厅

2020年5月7日

　　（信息公开形式：主动公开）

附件1

中小学校新冠肺炎疫情防控技术方案

（修订版）

　　一、组织保障和制度要求

　　1.加强组织领导。学校成立新冠肺炎疫情防控工作领导小组，全面领导学校疫情防控工作，确保各项措施落实。各岗位职责明确，任务到人。多校址办学的中小学校，每校址必须指定明确的疫情防控工作责任和工作联络人。

　　2.加强联防联控。教育部门和学校加强与卫生健康部门、疾控机构、就近定点医疗机构、社区卫生服务中心的沟通协调，配合属地街道（乡镇）、社区（村）等有关部门积极开展联防联控，卫生健康部门加强专业指导和人员培训，形成教育、卫生、学校、家庭与医疗机构、疾控机构“点对点”协作机制。

　　3.落实学校主体责任。学校应围绕关键环节和重点措施，制订专门的疫情防控工作方案、应急处置预案和工作制度，包括学校传染病疫情报告制度、晨午检制度、因病缺勤追踪登记制度、复课证明查验制度、健康管理制度、传染病防控健康教育制度、通风消毒制度、环境卫生检查制度和免疫预防接种查验制度等。做好应急演练，与当地医疗卫生机构做好沟通衔接。

　　4.做好保障物资储备。根据学校规模、学生及教职工数量，结合应急方案储备足够数量的疫情防控物资，包括消毒设备、消毒用品、口罩、手套、非接触式温度计、洗手液等。

　　5.校园内清洁消毒。开学前对学校环境和空调系统进行彻底清洁，并开展预防性消毒，教室、食堂、宿舍、图书馆等公共场所开窗通风。

　　二、人员管控要求

　　1.登记排查入校全体人员。提前掌握教职工（包括教师，以及食堂、保洁、保安等后勤服务人员）和学生健康状况，建立健康状况台账，做好健康观察。要求所有师生员工做好入校前至少14天的自我健康监测和行踪报告，并如实上报学校。对有发热、咳嗽、乏力、鼻塞、流涕、咽痛、腹泻等症状的人员，应督促其及时就医，暂缓返校，严禁带病上课、工作。

　　2.开展每日健康监测。加强对教职工和学生的晨午检，住宿学生增加晚检，实行“日报告”“零报告”制度。重点监测学生和教职工有无发热、咳嗽、乏力、鼻塞、流涕、咽痛、腹泻等症状，对因病缺勤的教职工和学生要密切跟踪其就诊结果和病情进展。有条件的学校可使用信息化手段进行报告、监测。

　　3.相关人员风险排查。建立学生及其共同生活居住的家庭成员及相关人员健康状况和风险接触信息报告制度，每日由家长向班主任报告。学生或共同生活人员出现发热等可疑症状时，要及时、如实报告学校，并送医就诊。

　　4.加强巡查。学校应每日开展校门口、食堂、厕所、教室、宿舍等重点区域、重点岗位、重点环节的巡查，发现潜在风险并及时通报和督促整改。

　　5.控制校内人员密度。学校实行相对封闭的管理措施，错时安排校内各班级作息，在入校离校、课间休息、用餐、如厕、进出宿舍等环节加强对学生的组织管理，人与人之间保持安全距离，所有可能引起人员排队聚集的场所均设置1米线，引导学生不追逐打闹、不握手、不拥抱。

　　6.严格控制聚集性活动。可通过错峰开会、网络视频或提前录制会议材料等方式召开学生会议，适当开展网络教育课程或线上展示交流活动，确需开展现场活动的，需按规定向学校相关部门申请。

　　7.开展健康教育与技能培训。将新冠肺炎及传染病防控知识与技能等纳入开学第一课内容，让学生和教职工掌握相应知识和技能，养成良好卫生习惯，做好自我防护。通过微信、校园网、校讯通等多种途径将相关知识技能信息推送给师生和家长，提高师生、家长对传染病的预防控制意识和应对能力。

　　8.加强师生员工心理疏导。关注师生员工的心理状况，通过开展心理健康知识培训，开设心理咨询、公布心理求助热线等方式给予适当心理援助。对未能及时开学的师生员工，更要做好心理疏导。

　　9.加强家校联动。提醒家长加强自我防护，避免不必要外出活动，做好亲子沟通。学生在校外出现发热等可疑症状，家长要及时、如实报告学校，并送医就诊。在学校正式通知返校前，不得提前返校，安心居家，做好线上教学或学习、返校前物资准备。

　　10.勤洗手。随时保持手卫生，餐前、便前便后、接触垃圾后、外出归来、使用体育器材、电脑等公用物品后、接触动物后、触摸眼睛等“易感”部位前、接触可疑污染物品后，均要洗手。采用正确洗手方法用流动水和洗手液（肥皂）洗手，也可用速干手消毒剂揉搓双手。

　　11.科学佩戴口罩。学生应随身备用符合一次性使用医用口罩标准或相当防护级别的口罩；低风险地区校园内学生不需佩戴口罩。口罩佩戴应遵循国务院应对新型冠状病毒肺炎疫情联防联控机制印发《公众科学戴口罩指引》原则。

　　12.加强近视防控。疫情期间，学生户外活动减少，电子产品使用过多，增加近视发生和进展的风险，师生家长要引导学生注意做好近视的防控。适当科学运动，平衡营养膳食，安排好作息，提高机体免疫力。

　　13.途中防护要求。上、下学途中要坚持家庭、学校“两点一线”，避免不必要外出活动。最好采取步行、自行车、私家车方式上下学，乘坐公共交通或校车时应注意个人防护，不与他人交谈，与他人保持合理间距，途中尽量避免用手触摸公共交通工具上的物品。上学到校或下学到家应及时洗手。

　　14.对住校生要求。在疫情防控期间不得出校，如必须出校，须严格履行请假程序，并告知家长，规划出行路线和出行方式。外出时，按相关规定做好个人防护和手卫生。

　　15.注意工作人员防护。老师授课时不需戴口罩，校门值守人员、清洁人员及食堂工作人员等应当佩戴口罩。食堂工作人员应穿工作服，并保持工作服清洁和定期洗涤、消毒。妥善保管消毒剂，标识明确，避免误食或灼伤。清洁消毒人员在配制和使用化学消毒剂时，应做好个人防护。

　　16.鼓励具备条件的中小学校教职工开学前自愿接受核酸检测。

　　三、重点区域防控要求

　　1.校园门口。实行校园相对封闭式管理，全面梳理所有进校通道，校外无关人员一律不准进校，师生进校门一律核验身份和检测体温。通过实行错时上下学、划定1米等候线等方式，避免人员聚集。校园封闭管理要做到专人负责、区域划分合理、人员登记排查记录齐全。

　　2.临时等候区。在校门口就近设置临时等候区，入校排查时若出现发热等可疑症状，应由专人带至临时等候区，复测体温，及时联系家长，做好基础防护后，按规定流程送发热门诊。定期常规消毒，若有可疑病例或发热人员进入，需在专业部门指导下进行消毒处理。

　　3.教室。有条件的学校应保证学生一人一桌，每名学生前后左右间距保持1米，对学生人数较多的班级可分班教学或错时上学，调整教学时间和学生行进路线，避免人员聚集，做到学习、生活空间相对固定，接触人员清楚。

　　4.食堂。实行学生错峰就餐，开餐前半小时完成就餐区域桌椅、地面及空气消毒，并通风换气，就餐排队时与他人保持1米距离，应遵循分时、错峰、单向就餐的原则，避免扎堆就餐、面对面就餐，避免交谈。餐前餐后必须洗手。加强餐（饮）具的清洁消毒，重复使用的餐（饮）具应当“一人一用一消毒”，就餐人员要做好餐余垃圾的清理、分类和投放。做好食品留样，专人管理，严格执行消毒时间、程序，制定就餐、消毒等管理台账。

　　5.饮用水设备与洗手设施。饮水设施应每天进行必要的清洁工作，每天对出水龙头至少消毒一次。要确保操场、厕所、食堂、宿舍等场所或附近洗手设施运行正常，原则上中小学校每40-45人设一个洗手盆或0.6m长盥洗槽，并备有足够数量的洗手液、肥皂等，也可配备速干手消毒剂或感应式手消毒设施。

　　6.学生及教职工宿舍。寄宿制学校学生宿舍不应设在地下室或半地下室，每个宿舍居住人数原则上不超过6人，人均宿舍面积不少于3平方米；学生宿舍应根据当地的气候条件设置通风设施。加强对教职工和学生宿舍的清洁通风，一般每天开窗通风不少于3次，每次不少于30分钟。每天对宿舍地面、墙壁、门把手、床具、课桌椅等物体表面进行预防性消毒，消毒后要保持宿舍内外的环境卫生保洁，每天专人巡查清扫并进行登记。寄宿制学校应建立学生宿舍专人负责制，严格学生宿舍楼门管理，实行凭证出入和体温排查。

　　7.厕所。学校厕所由专人管理，设置符合标准的便器。落实厕所保洁措施，保持空气流通，及时清洗地面，做好包括水龙头、门把手等重点部位的消毒，增加冲洗和消毒频率。厕所的洗手设施应完备，宜配洗手液，有条件的使用感应式水龙头、擦手纸或干手机。

　　四、环境卫生要求

　　1.开展校园环境整治。对学校进行彻底清洁，加强校园内教室、食堂、宿舍等学生重要聚集场所和洗手间、洗漱间的保洁和消毒，彻底清理卫生死角。认真做好学校室内外的环境卫生。正常情况下，以清洁为主，日常预防性消毒为辅。

　　2.加强通风换气。各类学习、工作、生活场所要加强通风换气，每次通风时间不少于30分钟，每日不少于3次。除特殊天气情况外，教室、办公室应保持全天开窗通风。如使用空调，应当保证空调系统供风安全，保证充足的新风输入，所有排风直接排到室外。

　　3.做好清洁消毒。加强物体表面清洁消毒。应当保持教室、宿舍、图书馆、学生实验室、体育活动场所、餐厅等场所环境卫生整洁，每日定期消毒并记录。对门把手、课桌椅、讲台、电脑键盘、鼠标、水龙头、楼梯扶手、宿舍床围栏、室内健身器材、电梯间按钮等高频接触表面，可用有效氯250-500mg/L的含氯消毒剂进行喷洒或擦拭，也可采用消毒湿巾进行擦拭。应当加强学校食堂、浴室及宿舍等重点场所地面的清洁，定期消毒并记录。可使用有效氯500mg/L的含氯消毒液擦拭消毒。

　　4.加强垃圾分类管理。校园垃圾做到日产日清，分类收集并及时清运各类垃圾。日常使用废弃口罩按生活垃圾处理。做好垃圾盛装容器的清洁和消毒工作，可用有效氯500mg/L的含氯消毒剂定期对其进行消毒处理。

　　五、出现疑似感染症状应急处置

　　1.教职工或学生每日入校前如出现发热、咳嗽、乏力、鼻塞、流涕、咽痛、腹泻等症状，应及时向学校报告，采取居家观察或就医排查等措施。

　　2.教职工或学生中如出现新冠肺炎疑似病例或确诊病例，学校应立即启动应急处置机制，在专业机构指导下采取相应疫情防控处置措施，并配合相关部门做好密切接触者的排查管理。对共同生活、学习的一般接触者要及时进行风险告知，如出现发热、干咳等症状时要及时就医。

　　3.在校期间，教职工或学生如出现发热、咳嗽、乏力、鼻塞、流涕、咽痛、腹泻等症状，应当立即上报学校疫情防控工作领导小组，第一时间采取隔离，严格按照“点对点”协作机制有关规定及时去定点医院就医。尽量避免乘坐公交、地铁等公共交通工具前往医院，并全程佩戴口罩。

　　4.学校要安排专人负责与接受隔离的教职工或学生家长进行联系沟通，掌握其健康状况。教职工和学生病愈后，返校要查验复课证明。

　　六、境外师生返校要求

　　1.境外师生未接到学校通知一律不返校，新生不报到。

　　2.境外师生返校前确保身体状况良好，返校途中做好个人防护和自我健康监测。

　　3.入境后严格执行当地规定进行核酸检测和隔离医学观察，每日健康监测并填报健康卡，解除隔离后且身体健康方可返校学习和工作。

附件2

托幼机构新冠肺炎疫情防控技术方案

（修订版）

　　一、开园前

　　（一）组织保障和制度要求。

　　1.各地根据当地疫情防控风险级别和疫情应急响应级别调整情况作出科学开园的决定，周密准备、有序推进托幼机构开园。严格落实属地责任、部门责任、单位责任和个人家庭责任，扎实做好托幼机构疫情防控各项工作安排。

　　2.落实主体责任，园长为本单位疫情防控第一责任人，全面负责托幼机构疫情防控工作的组织领导、责任分解、任务落实和督促检查。细化各项防控措施，制度明确，责任到人，确保每个细节、每个关键步骤落实到位，组织教职工进行培训、演练操作。

　　3.建立完善疫情防控联合工作机制。托幼机构应加强与属地卫生健康部门、疾控机构、就近定点医疗机构、辖区妇幼保健机构、社区卫生服务机构的沟通协调，配合属地街道（乡镇）、社区（村）等有关部门积极开展联防联控。形成教育与卫生健康部门合力，建立托幼机构与医疗机构、疾控机构“点对点”协作机制。确保开园前托幼机构疫情防控业务指导、巡查和培训全覆盖。

　　4.制定防控方案。托幼机构应围绕关键环节和重点措施，制定专门的疫情防控工作方案、应急处置预案和疫情防控相关工作流程和制度。如传染病疫情报告制度、晨午检制度、因病缺勤追踪登记制度、返园证明查验制度等。

　　5.保障物资储备。做好洗手液、速干手消毒剂、消毒剂、婴幼儿口罩、手套、体温计、呕吐包、紫外线消毒灯等防疫物资的储备，洗手处配备足量的洗手用品。建立环境卫生和清洁消毒管理制度，由专人负责托幼机构全面清洁消毒工作，包括清洁消毒用品管理、组织实施、工作监督等。

　　（二）场所和人员要求。

　　1.开园前对托幼机构环境和空调系统进行彻底清洁，对物体表面（如户外大型玩具、门把手等）进行预防性消毒处理，对各类生活、学习、工作场所（如活动室、睡眠室、盥洗室、教师办公室、音乐室、洗手间等）等所有场所开窗通风。

　　2.在托幼机构内设立（临时）隔离室，位置相对独立，设立提醒标识，应有单独使用的卫生间，配备专人负责，以备人员出现发热等症状时立即进行暂时隔离。

　　3.托幼机构教师做好婴幼儿每日健康状况统计，与家长密切联系，向卫生保健人员进行“日报告”与“零报告”。托幼机构卫生保健人员每日掌握教职工和婴幼儿健康情况，做好“日报告”、“零报告”的收集与管理，并上报主管部门；对全体教职工等开展防控制度、个人防护与消毒等知识和技能宣教。

　　4.家长每日做好婴幼儿健康监测和行踪报告，并如实上报托幼机构，确保开园前身体状况良好。开园前做好婴幼儿看护和防护物资准备，注意增减衣物防止婴幼儿感冒，减少前往人员密集场所；如必须外出，做好婴幼儿防护。

　　二、开园后

　　（一）人员管控。

　　1.登记排查入园。提前掌握教职工（包括食堂、保洁、保安等后勤服务人员）和婴幼儿健康状况，建立健康状况台账，做好健康观察。要求所有教职工和婴幼儿做好入园前至少14天的自我健康监测和行踪报告，并如实上报园方。对有发热、咳嗽、乏力、鼻塞、流涕、咽痛、腹泻等相关症状的人员，应督促其及时就医，暂缓返园，不要带病到园、工作。原则上外来人员不得入园，严格执行家长接送婴幼儿不入园制度。

　　2.各班级错峰、错时入园和离园，防止人员聚集。教职工和婴幼儿每天入园时测体温，不发热方可入园，严格落实婴幼儿晨午晚检和全日观察制度。晨午晚检时工作人员应佩戴口罩和一次性手套。

　　3.严格日常管理。坚持早、中、晚“一日三报告”制度和点名制度，每日掌握教职工和婴幼儿动态、健康情况，加强对教职工和婴幼儿的晨、午检工作，实行“日报告”、“零报告”制度，并向主管部门报告。做好缺勤、早退、病假记录，发现因病缺勤的教职工和婴幼儿及时进行追访、登记和上报。

　　4.建立婴幼儿及其共同生活居住的家庭成员及相关人员健康状况和风险接触信息报告制度，每日由家长向主班老师报告。

　　5.从严控制、审核、组织举办各类涉及婴幼儿聚集性的活动，不组织大型集体活动。

　　6.做好婴幼儿手卫生，尽量避免婴幼儿直接触摸门把手、电梯按钮等公共设施，接触后及时洗手或用速干手消毒剂揉搓双手。注意婴幼儿个人卫生，避免用手接触口眼鼻，注意咳嗽礼仪。入园后、进食前、如厕前后、从户外进入室内、玩耍前后、接触污渍后、擤鼻涕后、打喷嚏用手遮掩口鼻后、手弄脏后，均要洗手。洗手时应当使用洗手液或肥皂，在流动水下按照正确洗手法彻底洗净双手，也可使用速干手消毒剂揉搓双手。

　　7.尽量选择楼梯步行或扶梯，并与他人保持1米以上距离，避免与他人正面相对；若乘坐厢式电梯，与同乘者尽量保持距离，分散乘梯，避免同梯人过多。

　　8.严格落实托幼机构工作人员个人防护措施。婴幼儿应在充分保障健康安全的前提下离家到托幼机构，因婴幼儿特殊生理特征，不建议戴口罩；托幼机构教师、值守人员、清洁人员及食堂工作人员等应当佩戴口罩，做好手卫生。食堂工作人员还应穿工作服，并保持工作服清洁，清洁消毒人员在配制和使用化学消毒剂时，应做好个人防护。

　　9.通过多种形式面向教职工、婴幼儿和家长开展预防新冠肺炎的宣传教育。教会婴幼儿正确的洗手方法，培养婴幼儿养成良好卫生习惯，咳嗽、打喷嚏时用纸巾等遮挡口鼻。指导家长在疫情防控期间不带婴幼儿去人员密集和空间密闭场所。

　　10.境外师生未接到托幼机构通知一律不返园，新生不报到。返园前确保身体状况良好，返园途中做好个人防护和健康监测。入境后严格执行当地规定，进行核酸检测和隔离医学观察，每日健康监测并填报健康卡，解除隔离后且身体健康方可返园。

　　11.鼓励具备条件的托幼机构教职工开园前自愿接受核酸检测。

　　（二）重点场所防控。

　　1.加强物体表面清洁消毒。保持各类生活、学习、工作场所（如活动室、睡眠室、盥洗室、教师办公室、音乐室、洗手间等）等场所环境卫生整洁，每日定时消毒并记录。对门把手、水龙头、楼梯扶手、床围栏等高频接触表面，可用有效氯250-500mg/L的含氯消毒剂进行擦拭。

　　2.各类生活、学习、工作场所（如活动室、睡眠室、盥洗室、教师办公室、音乐室、洗手间等）等场所加强通风换气。每日通风不少于3次，每次不少于30分钟，也可采用机械排风。如使用空调，应当保证空调系统供风安全，保证充足的新风输入，全空气系统应关闭回风。

　　3.应当加强对各类生活、学习、工作场所（如活动室、睡眠室、盥洗室、教师办公室、音乐室、洗手间等）地面和公共区域设施等场所的清洁，定期消毒并记录。可使用有效氯500mg/L的含氯消毒液擦拭消毒。

　　4.加强饮食卫生。做好餐车、餐（饮）具的清洁消毒，餐（饮）具应当贴名，一人一具一消毒。餐（饮）具去残渣、清洗后，煮沸或流通蒸汽消毒15分钟；或采用热力消毒柜等消毒方式；或采用有效氯250mg/L的含氯消毒剂浸泡30分钟，消毒后应当将残留消毒剂冲洗干净。食堂工作人员的工作服应当定期洗涤、消毒，可煮沸消毒30分钟，或先用有效氯500mg/L的含氯消毒液浸泡30分钟，然后常规清洗。

　　5.加强饮水卫生和手卫生。饮水设施应每天进行必要的清洁工作，每天对出水龙头至少消毒一次。要确保操场、厕所、食堂、宿舍等场所或附近洗手设施运行正常。

　　6.加强婴幼儿个人用品消毒，包括玩具、毛巾等，用有效氯250mg/L的含氯消毒液浸泡30分钟，再用清水冲洗干净，放在通风处晾干。

　　7.加强托幼机构内垃圾分类管理。垃圾日产日清。日常使用废弃口罩按生活垃圾处理。做好垃圾盛装容器的清洁和消毒工作，可用有效氯500mg/L的含氯消毒剂定期对其进行消毒处理。

　　三、出现疑似感染症状应急处置

　　1.教职工或婴幼儿每日入园前如出现发热、干咳、乏力、呼吸急促、精神弱、呕吐、腹泻等症状，应及时向托幼机构报告，并按有关规定采取居家观察或就医排查等措施。

　　2.在园期间，教职工或婴幼儿如出现发热、干咳、乏力、呼吸急促、精神弱、呕吐、腹泻等可疑症状，应当立即上报本单位疫情防控第一责任人，第一时间采取隔离，严格按照“点对点”协作机制有关规定及时去定点医院就医。尽量避免乘坐公交、地铁等公共交通工具前往医院，应全程佩戴口罩，做好个人防护。

　　3.教职工或婴幼儿中如出现新冠肺炎疑似病例或确诊病例，托幼机构应立即启动应急处置机制，在疾控机构指导下采取相应疫情防控处置措施，并配合相关部门做好密切接触者的排查管理。对共同生活、学习的一般接触者要及时进行风险告知，如出现发热、干咳等症状时要及时就医。

　　4.托幼机构要安排专人负责与接受隔离的教职工或婴幼儿家长进行联系沟通，掌握其健康状况。教职工和婴幼儿病愈后，托幼机构根据卫生健康部门要求查验应提交的返园健康证明材料。

1. 商务部等8部门：进一步做好供应链创新与应用试点工作

新冠肺炎疫情发生以来，供应链创新与应用试点企业响应党和政府号召，迎难而上、积极作为，充分发挥供应链资源整合和高效协同优势，全力支持疫情防控、保障市场供应、推动复工复产，把试点创新成果应用到抗击疫情、推动经济发展的第一线。

为深入贯彻落实习近平总书记关于统筹推进新冠肺炎疫情防控和经济社会发展的系列重要讲话精神，发挥供应链创新与应用试点工作在推动复工复产、稳定全球供应链、助力脱贫攻坚等方面的重要作用，进一步充实试点内容，加快工作进度，2020年4月10日，商务部等8部门联合印发《关于进一步做好供应链创新与应用试点工作的通知》。

通知要求，试点城市要落实分区分级精准防控和精准复工复产要求，加快推动和帮助供应链龙头企业和在全球供应链中有重要影响的企业复工复产；试点企业要勇担社会责任，充分发挥龙头带动作用，加强与供应链上下游企业协同，协助配套企业解决技术、设备、资金、原辅料等实际困难。

通知指出，为认真贯彻落实打赢脱贫攻坚战、全面建成小康社会重大战略部署和坚持在常态化疫情防控中加快推进生产生活秩序全面恢复相关要求，针对供应链安全性和协同性方面存在的短板弱项，积极应对市场新需求、新业态、新模式加快发展给供应链创新与应用工作提出的新要求，要在原有试点任务基础上，重点做好加强供应链安全建设、加快推进供应链数字化和智能化发展、促进稳定全球供应链、助力决战决胜脱贫攻坚和充分利用供应链金融服务实体企业等五个方面工作。

通知强调，试点城市和试点企业要扎实推进试点工作，结合试点中期评估反馈意见和今年重点工作方向，制定针对性的整改落实措施，进一步完善工作思路和具体实施方案，加快试点工作进度，确保试点工作各项任务目标按期高质量完成；各部门要加强业务协同指导，精准扎实有序推动供应链全面复工复产，加强日常检查监督，指导各地加强供应链领域“政产研学用”有机融合；各地要立足本地实际，加快复制推广典型经验。

1. 教育部：将2020年上半年中小学教师资格考试推迟至下半年一并组织实施

根据国家疫情防控工作决策部署，切实保障广大考生和涉考工作人员的生命安全和身体健康，经研究决定，2020年上半年中小学教师资格考试推迟至下半年一并组织实施。上半年已报名缴费的考生可申请转至2020年下半年考试或申请退费，前期已通过的笔试科目成绩和合格证明有效期延长一年，具体规定请关注中国教育考试网（www.neea.edu.cn）。上半年教师资格认定工作，由各省（区、市）根据本省疫情防控形势确定具体开展时间。

为减轻对考生的影响，促进高校毕业生就业，经国务院同意，根据人力资源社会保障部、教育部等七部门印发的《关于应对新冠肺炎疫情影响实施部分职业资格“先上岗、再考证”阶段性措施的通知》（人社部发〔2020〕24号）规定，中小学、幼儿园、中等职业学校教师资格实施“先上岗、再考证”阶段性措施，用人单位2020年12月31日前招聘高校毕业生的，不得将教师资格作为限制性条件。请广大考生及时关注相关政策。

感谢大家的理解支持。

教育部

2020年4月24日

1. 人社部等7部门：应对新冠肺炎疫情影响实施部分职业资格“先上岗、再考证”阶段性措施 人社部发〔2020〕24号

各省、自治区、直辖市及新疆生产建设兵团人力资源社会保障厅（局）、教育厅（教委、教育局）、司法厅（局）、农业农村（农牧、畜牧兽医、渔业）厅（局、委）、文化和旅游厅（局）、卫生健康委、知识产权局（知识产权管理部门）：

为深入贯彻落实习近平总书记关于统筹推进新冠肺炎疫情防控和经济社会发展工作的重要指示精神，落实国务院常务会议部署要求，进一步强化稳就业举措，促进高校毕业生就业，经国务院同意，对《国家职业资格目录》中部分职业资格实施“先上岗、再考证”阶段性措施。现就有关事项通知如下：

一、对中小学、幼儿园、中等职业学校教师资格实施“先上岗、再考证”阶段性措施，凡符合教师资格考试报名条件和教师资格认定关于思想政治素质、普通话水平、身体条件等要求的高校毕业生，可以先上岗从事教育教学相关工作，再参加考试并取得教师资格。对护士执业资格、渔业船员资格、执业兽医资格、演出经纪人员资格、专利代理师资格等5项准入类职业资格实施“先上岗、再考证”阶段性措施，凡符合该5项职业资格考试报名条件的高校毕业生，可以先上岗从事相关工作，再参加考试并取得职业资格。用人单位在2020年12月31日前招聘高校毕业生的，不得将取得上述职业资格作为限制性条件。

二、对实施“先上岗、再考证”的准入类职业资格，高校毕业生在取得职业资格之前主要从事辅助性工作。其中，尚未取得教师资格的高校毕业生先上岗的，不宜独立承担一门课的讲授任务或幼儿园带班任务。尚未取得护士执业资格的护理、助产专业高校毕业生先上岗的，可以先从事医疗护理员工作，但不得从事诊疗技术规范规定的护理活动。尚未取得执业兽医资格的兽医相关专业高校毕业生先上岗的，不得独立从事动物诊疗活动、开具兽药处方。

三、尚未取得法律职业资格证书的高校毕业生，符合国家统一法律职业资格考试报名条件和申请律师执业实习其他条件的，可先申请实习登记，在律师事务所实习。实习期满经律师协会考核合格并取得法律职业资格证书的，或者自收到考核合格通知之日起一年内取得法律职业资格证书的，可以按规定申请律师执业。

四、各地区统筹安排技能培训补贴时，要将先上岗的高校毕业生纳入补贴范围。用人单位要按规定对先上岗的高校毕业生加强岗位培训，提高其实际工作能力。

五、各地区、各有关部门要落实好先上岗的高校毕业生各项待遇保障，按照规定为其计算工龄，依法缴纳社会保险费等，切实维护其合法权益。对实施“先上岗、再考证”阶段性措施的准入类职业资格，高校毕业生参加事业单位公开招聘被聘用从事相关工作的，事业单位与先上岗的高校毕业生签订聘用合同时，应当按规定约定1年试用期；先上岗的高校毕业生在试用期内未取得相应职业资格的，应当依法解除聘用合同。高校毕业生与用人单位签订劳动合同的，按照有关规定管理。

六、实施“先上岗、再考证”阶段性措施是当前形势下稳定高校毕业生就业的重要举措。各地区、各有关部门要高度重视，提高政治站位，加强监督指导，精心组织实施，做好政策宣传和舆论引导，让广大高校毕业生和社会公众充分了解政策，让用人单位精准把握政策，确保政策落实落地。要根据新冠肺炎疫情防控形势，合理安排职业资格考试，为高校毕业生参加考试提供便利，统筹做好高校毕业生就业和职业资格有关工作。

人力资源社会保障部

教育部

司法部

农业农村部

文化和旅游部

国家卫生健康委

国家知识产权局

2020年4月21日

1. 交通部等6部门：精准做好国际航行船舶船员疫情防控工作 交海明电〔2020〕142号

各省、自治区、直辖市、新疆生产建设兵团应对新冠肺炎疫情联防联控机制，各省、自治区、直辖市、新疆生产建设兵团交通运输厅（局、委）、外事办、卫生健康委，海关总署广东分署、各直属海关，各出入境边防检查总站，民航各地区管理局，长江航务管理局，各直属海事局：

为深入贯彻习近平总书记等中央领导同志重要指示批示精神，推动落实党中央、国务院关于统筹推进疫情防控和经济社会发展工作的决策部署，精准做好国际航行船舶船员疫情防控工作，有序保障中国籍船员换班、合同到期回国等，依法保障船员合法权益，按照国务院应对新冠肺炎疫情联防联控机制部署要求，现就精准做好国际航行船舶船员疫情防控工作通知如下：

一、严格落实新冠肺炎疫情防控责任

（一）严格落实企业主体责任。航运企业、海员外派机构是船员疫情防控第一责任人，要进一步完善公司管理体系和制度，严格落实船舶、船员疫情防控各项要求，遵守检疫检测规定，切实加强对在船船员的管理，开展船员疫情防控知识培训，指导船舶督促船员做好在船和生产作业期间的自我防护，配足配齐船上各类防护用品，保障船舶物料、船员膳食和生活必需品供给。

（二）严格落实行业监管责任。交通运输主管部门、直属海事局要按照职责分工加强对航运企业、海员外派机构的依法监管，督促指导建立健全并严格执行疫情防控工作各项制度，积极配合地方政府和各口岸查验单位落实防范境外疫情输入工作。各地交通运输、口岸查验和卫生健康单位要根据自身职责，进一步优化相关联防联控措施，做好国际航行船舶船员出入境管理。

（三）严格落实地方政府属地责任。港口所在地人民政府要严格落实属地责任，严格执行中国籍船员换班的规定，满足船员合理的离船请求；要组织交通运输、公安、卫生健康等相关部门，安排专门力量，切实加强对入境船员的管理，有效控制潜在的疫情传播风险。

二、对国际航行船舶船员实施严格封闭管理

（四）国际航行船舶在航行、停泊期间，要参照《船舶船员新冠肺炎疫情防控操作指南》做好疫情防控，实行在船封闭管理。要落实船长负责制，加强对在船船员健康状况的监测，每日不少于两次对船员体温进行测量，并进行连续记录；发现异常情况，要及时向航运企业、海员外派机构报告。

（五）船舶靠港后，除正常的中国籍船员换班、对伤病船员紧急救助必需等之外，不安排船员上岸活动；船舶应当加强舷梯口值班，严禁无关人员上下船，确需下船人员需做好个人防护。

三、优化中国籍船员换班出入境通关防控措施

（六）对于境内港口换班上船的中国籍船员，按照国家相关规定到达换班港口，满足当地人民政府疫情防控要求的，经海关检疫无异常后，可以换班上船。

（七）国际航行船舶入境后，计划换班下船的中国籍船员经海关检疫无异常且核酸检测阴性后，自船舶驶离上一港口满14天、健康记录显示连续14天及以上正常的，在办理换班入境手续后，港口所在地人民政府应当给予便利。不满足上述条件的，在办理换班入境手续后，对于海关检疫有异常或核酸检测阳性的船员，由港口所在地人民政府按照联防联控机制相关要求处置；对于海关检疫无异常且核酸检测阴性的船员，要严格遵守港口所在地关于境外返回非“四类人员”疫情防控相关规定和要求。

（八）船员在换班全过程应当做好个人防护外，在隔离期间必须严格遵守隔离规定，并定时按规定向航运企业、海员外派机构报告每日健康状况。

四、稳妥做好境外中国籍船员的回国安排

（九）针对境外中国籍船员在疫情期间面临的合同到期、签证失效等突出问题，我驻外使领馆应当畅通信息报告的接收渠道，积极做好领事保护工作。

（十）对于身处困境并强烈请求安排回国的中国籍船员，可研究以适当方式稳妥安排。相关交通运输、外事、民航部门、口岸查验和卫生健康等单位要在地方人民政府和上级主管部门领导下，积极协调航运企业、海员外派机构等做好船员权益保障和回国入境管理工作。

五、加强内外贸兼营船舶由国际航线转国内航线的管理

（十一）实施精准管理。内外贸兼营散装液体危险货物运输船舶由国际航线转国内航线（仅限一个国内航次）时，所有船员未出现任何症状且核酸检测结果为阴性的，由海关办理改营手续，可不再要求隔离14天，但须由航运企业和船舶向口岸所在地交通运输主管部门、海事管理机构分别作出自离开境外最后一个港口14天内除伤病船员紧急救助外不在我国港口下船、不进行船员换班、非执行公务人员不上船的书面承诺。

（十二）加强信息通报和监督。内外贸兼营散装液体危险货物运输船舶由国际航线转国内航线（仅限一个国内航次）时，自船舶离开境外最后一个港口不足14天的，进出国内港口要向海事管理机构如实报告国际航线转国内航线情况，以及船舶离开境外最后一个港口信息、船员及健康信息、拟靠泊内贸港口信息、14天内人员上下船信息等，并提前将上述相关信息及时通报国内航线有关港口企业。港口企业发现船舶在港期间违反承诺的，及时向有关管理部门报告。对不遵守承诺的航运企业，由口岸所在地交通运输主管部门纳入水路运输领域信用信息管理，并向口岸查验单位进行通报，疫情期间海关不再为其所属船舶办理由国际航线转国内航线的改营手续。对于造成疫情传播扩散的，依法追究相关企业和个人责任。

请各省、自治区、直辖市、新疆生产建设兵团应对新冠肺炎疫情联防联控机制将本通知精神传达到开通国际航运口岸城市人民政府应对新冠肺炎疫情联防联控机制，督促抓好贯彻落实。

交 通 运 输 部

外交部

国家卫生健康委

海 关 总 署

国家移民管理局

中国民用航空局

2020年4月22日

1. 卫健委等7部门：印发《入境人员心理疏导和社会工作服务方案》 国卫办疾控函〔2020〕319号

各省、自治区、直辖市及新疆生产建设兵团卫生健康委、民政厅（局）、交通运输厅（局、委）、海关总署广东分署，各直属海关，各出入境边防检查总站，民航各地区管理局、各铁路局集团公司：

　　为加强新冠肺炎疫情防控，做好入境人员心理疏导和社会工作服务，帮助入境人员适应隔离环境，提升入境管理和服务水平，我们联合制定了《入境人员心理疏导和社会工作服务方案》。现将文件印发给你们，请认真贯彻落实。

国家卫生健康委办公厅

民政部办公厅

交通运输部办公厅

海关总署办公厅

国家移民管理局综合司

民航局综合司

国铁集团办公厅

2020年4月21日

（信息公开形式：主动公开）

入境人员心理疏导和社会工作服务方案

出入境口岸是新冠肺炎“外防输入”的重要关口。在防控新冠肺炎境外输入工作中，为做好入境人员心理疏导和社会工作服务，帮助入境人员适应隔离环境，提升入境管理和服务水平，特制定本方案。

　　一、工作目标

　　（一）开展新冠肺炎防控知识、与疫情相关心理健康知识、国家输入性疫情防控措施等宣传，对国家采取的人员入境后隔离观察等措施进行政策解读，减轻入境人员因认知不足、环境不适所致的恐惧、焦虑等负性情绪。

　　（二）利用互联网心理疏导和社会工作服务资源、心理援助和社会工作服务热线等平台，为入境人员提供线上心理疏导和社会工作服务，帮助有需要者进行自我心理调适，尽快渡过适应期。

　　（三）在入境人员临时转运区、隔离点、定点医院等场所，开展线下心理疏导和社会工作服务，减少入境人员对隔离观察措施的排斥行为，营造积极健康的社会环境。为有需要者提供心理辅导、情绪支持、社会资源链接、矛盾纠纷调解等服务，建立危机干预及转介机制，及时妥善处理突发事件。

　　二、组织实施

　　（一）加强领导。口岸所在地的卫生健康、民政、交通运输、海关、移民边检、民航、铁路等部门成立入境人员心理疏导和社会工作服务领导小组进行统筹协调，各部门依照职责分工，对专业人员缺乏的重要边境口岸，省级相关部门要组织动员有关单位、组织和人员对口支援，也可通过政府购买服务或志愿服务等方式在机场、铁路、港口、公路等临时转运区、集中隔离点、定点医院、入境人员家属接待点等区域和场所，开展疫情防控知识宣传、政策解读、心理疏导和社会工作服务。

　　（二）组建服务队伍。口岸所在地的卫生健康、民政协同海关、移民边检、机场等部门、铁路、公路和水运等运输企业建立心理疏导和社会工作服务队，设立专人统筹协调入境人员心理疏导和社会工作服务，配备1-2名心理工作专业人员或社会工作者在入境管控的主要环节和区域开展疫情防控科普知识宣传、疫情防控政策咨询和解读工作，向入境人员提供心理疏导和社会工作服务资源网络信息，为有需要者提供心理辅导、情绪支持和有关转介服务。各隔离点所在地的卫生健康、民政部门建立心理疏导和社会工作服务队，配备1-2名心理工作专业人员或社会工作者。

　　（三）开展对口技术支持。口岸所在地的卫生健康、民政部门根据入境人员数量和需求情况，依托口岸所在地精神卫生医疗机构和社会工作行业组织组建心理疏导和社会工作专家组，吸收当地具有心理疏导和危机干预经验的心理卫生、社会工作服务等专业人员参加，为口岸、机场、临时转运区、隔离点开展入境人员心理疏导和社会工作服务提供技术支持和指导，提供精神医学干预服务。

　　（四）培训工作人员协助提供心理和社会支持。对承担口岸、机场、临时转运区、隔离点工作的相关部门和单位工作人员开展培训，指导其在工作过程中注意观察入境人员情绪，识别常见心理问题，使用简单心理支持技巧，将有疑似心理和精神问题人员及时反馈给精神卫生专业人员。隔离点应在每日工作例会和交接班中汇报重点人员情况，建立重点人员档案并持续提供心理疏导和社会工作服务。

　　三、工作措施

　　（一）在全流程开展宣传、提供心理疏导和社会工作服务资源。卫生健康、民政部门制定相关科普宣传素材，并提供给承担入境人员运输的铁路、陆路、水运等运输企业，为入境人员提供新冠肺炎防控知识、国家入境隔离观察政策和心理健康知识等科普宣传材料，提供心理疏导和社会工作援助热线电话、网络心理服务资源链接及二维码等资源。口岸、临时转运区等在醒目位置摆放易拉宝或电子大屏滚动播出心理健康科普信息，显示心理疏导和社会工作服务资源链接或二维码，提高入境人员防控意识技能，增强心理承受力，为有需求者提供求助渠道。

　　（二）做好入境过程中的心理疏导和社会工作服务。口岸、机场、临时转运区的心理疏导和社会工作服务队要建立值班巡查制度，制订心理危机应急预案。工作人员发现可能存在心理或精神问题的入境人员，及时报告心理疏导和社会工作服务队，或视其严重程度报告公安机关等，由其与对口精神卫生医疗机构联系或作出相应处置。经专业评估，发现心理或精神问题较为严重的人员，及时送往精神卫生医疗机构。对有自杀、自伤或伤害他人风险行为冲动的人员，口岸、机场、临时转运区应立即报告所在地相关部门进行处置。

　　（三）做好集中隔离期间入境人员的心理疏导和社会工作服务。入境人员隔离点要创建有利于维护心理健康的环境，按照隔离规定设置房间，尽量提供方便生活的设备和网络，鼓励隔离人员正常作息、通过电话网络等与家人、朋友保持沟通，缓解隔离带来的孤独感；鼓励隔离人员主动学习疫情防控及心理健康知识，提高疫情防控技能；鼓励隔离人员探索有益的兴趣爱好，丰富精神文化生活。提供疫情防控和心理健康科普、心理自评工具等资源。主动关注隔离人员的情绪变化、睡眠情况及行为表现，及时发现需要接受心理疏导者和心理危机高危人员。

　　（四）做好重点入境人员的心理疏导和社会工作服务。针对单独隔离的未成年人，要有指定的成人监护，保证环境的安全。协助未成年人保持与家人的联系,及时向家人反馈未成年人的环境适应状况和日常生活情况。对儿童要善于运用活泼、生动的形式，以他们能听懂的语言解释为什么要隔离等情况，可根据儿童年龄结合游戏辅导，采取绘本、视频等进行解释告知。如有儿童出现睡眠困难、不愿意进食、焦虑担心等情况，隔离点医护人员要及时告知心理疏导和社会工作服务人员，必要时请精神科医生会诊。对有特殊需要的隔离人员（患有原发躯体疾病、特殊药物治疗、家庭情况复杂、残障人士等），开展心理和社会工作服务需求评估，整合社会资源为特殊服务对象开展有针对性的社会工作帮扶计划，提供全方位的关爱保护和照护服务。

　　（五）做好入境人员家属的社会心理支持服务。入境人员家属所在街道（乡镇）或社区应当引导心理疏导和社会工作服务资源，为入境人员家属提供社会心理支持服务。建立志愿服务网络和邻里支持体系，搭建自助、互助和群助的平台，倡导家属之间互相帮助、邻里之间相互支持，增强隔离人员家属的社会支持网络。针对公众关心的热点话题，通过权威媒体开展新冠肺炎防控宣传和国家入境隔离政策，提高家属防控知识和能力，加强心理支持，减轻担忧。

附件

入境人员自我心理保健要点

一、及时获悉健康知识，规范自我防护行为

　　及时通过权威渠道获悉新冠肺炎相关知识以及自我防护的措施和办法。对不理解的信息可向专业人员咨询，获得澄清与解释。不信谣、不传谣。规范防护行动，避免过度焦虑和盲目恐慌。

　　二、树立人人有责态度，积极配合防范措施

　　树立对自身和他人的健康负责任的态度，正确理解传染病防范理念，积极配合相关部门和人员，做好统一管理、隔离、转运。确保自身健康得到充分、及时保障。遇到困难，与工作人员沟通交流，获得理解，共同解决。

　　三、保持生活作息规律，形成良好生活习惯

　　逐步调整时差，恢复正常作息时间，熟悉国内生活环境，规律饮食、营养均衡，不喝酒、不吸烟，保证每日睡眠7-9小时。坚持每日适量运动。保持心情愉悦。提升自身机体免疫力。

　　四、适应工作学习变化，把握休闲娱乐节奏

　　尽快适应在特殊阶段的工作、学习环境和条件的变化，制定合理计划，有效利用时间，注意劳逸结合。找到或培养适宜、健康的休闲娱乐方式，做好时间的控制和节奏的把握。保持放松、平和的心身状态。

　　五、保持自我积极心态，及时获取社会支持

　　客观、全面、理性地看待这次疫情所造成的影响，合理关注自身、他人以及周围环境积极的一面；保持自信，发挥主观能动性，采取有效方法和技巧调整情绪；愿意与家人、朋友、同事等分享内心的感受和想法，获得帮助和理解。

　　六、敏锐觉察心理状况，主动寻求专业帮助

　　如发现自己的想法、情绪和行为偏离常态，且此状况持续2周以上，请先通过拨打热线电话或者借助网络平台获得远程专业心理疏导服务。如果效果不佳，请及时告知家人或朋友，在他们的陪同下，前往精神卫生专科医院就诊。

1. 交通部等7部门：更好服务稳外贸工作

各省、自治区、直辖市、新疆生产建设兵团交通运输厅(局、委)、商务厅(局、委)、邮政管理局，各直属海关，各直属海事局，各地区铁路监督管理局，各民航地区管理局，各铁路局集团公司：

为深入贯彻落实习近平总书记重要指示批示精神和党中央、国务院决策部署，在国务院复工复产推进工作机制、外贸外资协调机制下，统筹做好新冠肺炎疫情防控和经济社会发展工作，发挥交通运输“先行官”作用，保障国际国内运输通道畅通便利，优化运输市场环境，提高运输服务效率，更好地服务稳外贸工作，现将有关事项通知如下：

一、畅通外贸运输通道

1、确保国际海运保障有力。利用港口生产统计、国际集装箱航线航班周报等手段，加强干线港口和班轮公司运行监测，根据外贸运输需求，引导班轮公司及时恢复前期因疫情影响削减的航线航班。密切跟踪国外疫情发展，发挥骨干航运企业作用，确保主要贸易航线不中断，为外贸运输提供有力保障。

2、加强航空货运运力配置。充分发挥国际航空货运审批“绿色通道”作用，支持航空公司增开全货运航线航班、使用客机执行货运航班，并在航权、时刻方面给予倾斜。落实对国际货运航线的支持政策，鼓励航空企业尽快扩大货机运力规模，快速提升覆盖全球主要国家和地区的国际货运能力，缓解运力短缺矛盾。支持以货运为主的枢纽机场开放日间繁忙时段的货运航班时刻配置，全力推动航空公司复航或增加货运航班。

3、推动中欧班列高质量发展。发挥中欧班列在疫情期间的重要通道作用，组织铁路企业增加中欧班列班次密度，扩大覆盖面，对中欧班列承运、装车和挂运给予重点保障，确保应运尽运。加强与中欧班列运营平台公司和相关企业的工作对接，推动国际邮件快件、跨境电商产品通过中欧班列实现常态化运输。优化班列运输组织，统筹考虑货源组织和运输组织的衔接，打通中欧班列运输“微循环”。鼓励港航企业与铁路企业加强合作，促进集装箱海运与铁路相互调运。

4、畅通国际邮件快件寄递渠道。推动邮政、快递企业利用自有全货机、包机、租赁飞机等多种方式增加国际航空货运运力，积极支持邮政、快递企业与国际航空、铁路、海运、道路运输企业深化合作，积极利用国际航班、中欧班列、国际快船以及周边国家陆路运输等多种渠道，加快缓解当前国际邮件快件积压问题。充分利用我国交通运输和物流企业境外业务网点、海外仓和地面运输系统，形成畅通有序的国际物流供应链。

5、确保国际道路货运畅通。加强与口岸管理相关单位的工作对接，重点保障防疫物资、重点建设项目和生产生活物资运输车辆的出入境便利化，为其优先办理相关手续，优先查验放行。及时协调解决口岸通关、车辆及人员查验、货物装卸等环节遇到的问题，在落实防疫措施前提下为运输车辆和驾驶员往来提供便利。

6、积极发展集装箱铁水联运。以集装箱干线港为重点，推进集装箱铁水联运发展，加快苏州太仓港疏港铁路工程、南京港龙潭铁路专用线工程、广州南沙港疏港铁路工程等项目的建设进度。统筹以港口为起点的中欧班列与其他班列线路联动发展，完善内陆无水港布局，加强国际海运、港口、铁路货运场站之间的衔接。大力组织开行铁水联运集装箱班列，提高铁路集疏港比例。加快多式联运公共信息平台建设，实现各种运输方式信息交换共享。

二、促进外贸运输便利化

7、深化国际贸易“单一窗口”建设。推动实现船舶联合登临检查，进一步简化进出口环节监管手续，优化海事监管、引航服务和通关流程，建立更加集约、高效、运行顺畅的船舶便利通关查验新模式，加快推进“单一窗口”功能覆盖海运和贸易全链条。

8、推动港口直装直提作业模式试点。推动上海港、天津港等开展进口货物“船边直提”和出口货物“抵港直装”等作业模式试点，在总结经验基础上复制推广到集装箱干线港，加快港口货物周转，提升物流效率。

9、提升港口能力和效率。推动上海港、天津港加快实施冷藏箱专用堆场扩能改造，2020年底前冷藏箱堆存能力较2019年提高1倍。加快推进广州南沙四期2号泊位、江苏太仓四期等新建自动化集装箱码头建设以及唐山港、天津港等已建集装箱码头的自动化改造，争取年底前主体工程完工。

10、提升国际道路运输便利化水平。加快推进国际道路运输管理与服务信息系统建设，推进对国际道路运输车辆的全程动态监管，为国际道路运输市场全域开放提供有力支撑。深入开展加入《危险货物国际道路运输公约》(ADR公约)的政策分析评估和法规标准对接等工作，加快国际便利化运输公约接轨步伐。研究制定跨里海国际联运走廊工作方案，加快开辟国际运输网络辐射新空间。

三、降低进出口环节物流成本

11、降低进出口环节收费。全面落实阶段性免征进出口货物港口建设费，减征货物港务费、港口设施保安费以及船舶油污损害赔偿基金等降费政策。会同价格主管部门研究进一步减并港口收费项目，降低政府定价的港口经营服务性项目收费标准。支持外贸企业提高海运运费议价能力，引导我企业出口选择到岸价格(CIF)结算，进口选择离岸价格(FOB)结算。引导班轮公司合理调整海运收费价格结构，降低海运附加费占总运费比重，督促国际班轮公司传导港口等降费政策效果。

12、加强港口航运市场监管。督促港口企业和相关单位认真落实口岸经营服务性收费目录清单和公示制度，依法对违规行为进行调查，并配合市场监管等部门进行查处，进一步规范港口收费行为。规范班轮公司海运附加费收费行为，依法加强对国际班轮公司运价备案检查。对于违反诚实信用原则巧设名目，就无实质服务内容的事项收取费用，以及在成本没有发生明显增长的情况下、推动附加费价格过快过高上涨的行为，会同市场监管部门依法实施查处。

13、鼓励港航企业与进出口企业深化互助合作。指导中国港口协会、中国船东协会发挥桥梁作用，加强与进出口相关企业协会的沟通协调，鼓励港航企业与进出口企业建立长期稳定、互利共赢的合作机制，同舟共济、共克时艰。引导大型港口企业、国际班轮公司继续给予进出口企业库场使用费、滞箱费等优惠。

四、营造良好外部环境

14、保障航运正常生产秩序。交通运输、海关部门要加强与移民边检、卫生健康等部门协调，统筹做好防范境外疫情输入和保障航运生产秩序工作，推动解决国际航行船舶中国籍船员境内港口正常换班、物资供应、船舶维修、船舶检验等工作。督促航运公司和海员外派机构切实落实主体责任，指导船舶做好疫情防控各项工作。

15、加强国际沟通合作。秉持人类命运共同体理念，加强与贸易伙伴国和世界贸易组织、国际海事组织、国际劳工组织、国际民航组织、铁路合作组织、万国邮联等国际组织的沟通协调，共同维护正常国际运输秩序，保障国际物流供应链正常运转，促进国际贸易健康平稳发展。

五、强化机制保障

16、做好疫情期间运行协调调度。加强综合交通运输各种方式之间的衔接，发挥各自的比较优势和组合效率。加强与相关部门的沟通协调，形成共同促进国际物流链畅通的合力。充分发挥国务院复工复产推进工作机制国际物流工作专班的作用，加强与工业和信息化等部门合作，实现外贸运输供需信息及时有效对接、运力及时有序调度；加强与主要制造企业、外贸企业等重点保障企业的联系，建立对接工作机制，统筹运力资源，协调解决医疗物资外援、商业出口和外贸物资国际运输问题。

交通运输部

商务部

海关总署

国家铁路局

中国民用航空局

国家邮政局

中国国家铁路集团有限公司

2020年4月20日

1. 人社部、住建部：落实新冠肺炎疫情防控期间暂缓缴存农民工工资保证金政策 人社厅发﹝2020﹞40号

各省、自治区人力资源社会保障厅、住房和城乡建设厅，直辖市人力资源社会保障局、住房和城乡建设（管）委，新疆生产建设兵团人力资源社会保障局、住房和城乡建设局：

为深入贯彻习近平总书记关于统筹推进新冠肺炎疫情防控和经济社会发展工作的重要指示精神，更好实施就业优先政策，加快落实阶段性、有针对性的减负措施，推动建筑企业复产复工，现就房屋和市政基础设施工程建设项目暂缓缴存农民工工资保证金等有关事项通知如下：

一、按照《国务院办公厅关于应对新冠肺炎疫情影响强化稳就业举措的实施意见》（国办发〔2020〕6号）要求，尽快制定本地区具体落实办法，确保自实施意见发布之日起至2020年6月底前，暂缓缴存农民工工资保证金政策不折不扣落实落地，政策实施期内新缴存的农民工工资保证金要尽快返还。

二、严格落实农民工工资保证金差异化存储办法，对一定时期内未发生拖欠工资的施工企业，依法依规实行减免措施，切实减轻工资支付记录良好企业的资金压力。

三、加快推行金融机构保函，鼓励使用银行类金融机构出具的银行保函替代现金农民工工资保证金，有条件的地区可以积极引入工程担保公司保函或工程保证保险。

四、加快推行建筑工人实名制管理制度，确保开复工的房屋建筑和市政基础设施工程项目建筑工人实名制管理全覆盖。对严格落实建筑工人实名制管理制度、规范管理农民工工资专户的建筑企业可按本地区规定享受农民工工资保证金差异化缴存政策。

各地区人力资源社会保障部门、住房和城乡建设主管部门要加强政策落实情况的督促检查，强化部门协调配合，按照职责分工共同做好现阶段暂缓缴存农民工工资保证金等相关工作。

请于2020年7月31日前，将本地区暂缓缴存农民工工资保证金政策落实情况及缓缴金额报人力资源社会保障部劳动监察局、住房和城乡建设部建筑市场监管司。

人力资源和社会保障部办公厅

中华人民共和国住房和城乡建设部办公厅

2020年4月9日

1. 最高法：依法妥善审理涉新冠肺炎疫情民事案件若干问题 法发〔2020〕12号

各省、自治区、直辖市高级人民法院，解放军军事法院，新疆维吾尔自治区高级人民法院生产建设兵团分院：

现将《最高人民法院关于依法妥善审理涉新冠肺炎疫情民事案件若干问题的指导意见（一）》印发给你们，请认真贯彻执行。

最高人民法院

2020年4月16日

最高人民法院关于依法妥善审理涉新冠肺炎疫情民事案件若干问题的指导意见（一）

为贯彻落实党中央关于统筹推进新冠肺炎疫情防控和经济社会发展工作部署会议精神，依法妥善审理涉新冠肺炎疫情民事案件，维护人民群众合法权益，维护社会和经济秩序，维护社会公平正义，依照法律、司法解释相关规定，结合审判实践经验，提出如下指导意见。

一、充分发挥司法服务保障作用。各级人民法院要充分认识此次疫情对经济社会产生的重大影响，立足统筹推进疫情防控和经济社会发展工作大局，充分发挥司法调节社会关系的作用，积极参与诉源治理，坚持把非诉讼纠纷解决机制挺在前面，坚持调解优先，积极引导当事人协商和解、共担风险、共渡难关，切实把矛盾解决在萌芽状态、化解在基层。在涉疫情民事案件审理过程中，根据案件实际情况，准确适用法律，平衡各方利益，保护当事人合法权益，服务经济社会发展，实现法律效果与社会效果的统一。

二、依法准确适用不可抗力规则。人民法院审理涉疫情民事案件，要准确适用不可抗力的具体规定，严格把握适用条件。对于受疫情或者疫情防控措施直接影响而产生的民事纠纷，符合不可抗力法定要件的，适用《中华人民共和国民法总则》第一百八十条、《中华人民共和国合同法》第一百一十七条和第一百一十八条等规定妥善处理；其他法律、行政法规另有规定的，依照其规定。当事人主张适用不可抗力部分或者全部免责的，应当就不可抗力直接导致民事义务部分或者全部不能履行的事实承担举证责任。

三、依法妥善审理合同纠纷案件。受疫情或者疫情防控措施直接影响而产生的合同纠纷案件，除当事人另有约定外，在适用法律时，应当综合考量疫情对不同地区、不同行业、不同案件的影响，准确把握疫情或者疫情防控措施与合同不能履行之间的因果关系和原因力大小，按照以下规则处理：

（一）疫情或者疫情防控措施直接导致合同不能履行的，依法适用不可抗力的规定，根据疫情或者疫情防控措施的影响程度部分或者全部免除责任。当事人对于合同不能履行或者损失扩大有可归责事由的，应当依法承担相应责任。因疫情或者疫情防控措施不能履行合同义务，当事人主张其尽到及时通知义务的，应当承担相应举证责任。

（二）疫情或者疫情防控措施仅导致合同履行困难的，当事人可以重新协商；能够继续履行的，人民法院应当切实加强调解工作，积极引导当事人继续履行。当事人以合同履行困难为由请求解除合同的，人民法院不予支持。继续履行合同对于一方当事人明显不公平，其请求变更合同履行期限、履行方式、价款数额等的，人民法院应当结合案件实际情况决定是否予以支持。合同依法变更后，当事人仍然主张部分或者全部免除责任的，人民法院不予支持。因疫情或者疫情防控措施导致合同目的不能实现，当事人请求解除合同的，人民法院应予支持。

（三）当事人存在因疫情或者疫情防控措施得到政府部门补贴资助、税费减免或者他人资助、债务减免等情形的，人民法院可以作为认定合同能否继续履行等案件事实的参考因素。

四、依法处理劳动争议案件。加强与政府及有关部门的协调，支持用人单位在疫情防控期间依法依规采用灵活工作方式。审理涉疫情劳动争议案件时，要准确适用《中华人民共和国劳动法》第二十六条、《中华人民共和国劳动合同法》第四十条等规定。用人单位仅以劳动者是新冠肺炎确诊患者、疑似新冠肺炎患者、无症状感染者、被依法隔离人员或者劳动者来自疫情相对严重的地区为由主张解除劳动关系的，人民法院不予支持。就相关劳动争议案件的处理，应当正确理解和参照适用国务院有关行政主管部门以及省级人民政府等制定的在疫情防控期间妥善处理劳动关系的政策文件。

五、依法适用惩罚性赔偿。经营者在经营口罩、护目镜、防护服、消毒液等防疫物品以及食品、药品时，存在《中华人民共和国消费者权益保护法》第五十五条、《中华人民共和国食品安全法》第一百四十八条第二款、《中华人民共和国药品管理法》第一百四十四条第三款、《最高人民法院关于审理食品药品纠纷案件适用法律若干问题的规定》第十五条规定情形，消费者主张依法适用惩罚性赔偿的，人民法院应予支持。

六、依法中止诉讼时效。在诉讼时效期间的最后六个月内，因疫情或者疫情防控措施不能行使请求权，权利人依据《中华人民共和国民法总则》第一百九十四条第一款第一项规定主张诉讼时效中止的，人民法院应予支持。

七、依法顺延诉讼期间。因疫情或者疫情防控措施耽误法律规定或者人民法院指定的诉讼期限，当事人根据《中华人民共和国民事诉讼法》第八十三条规定申请顺延期限的，人民法院应当根据疫情形势以及当事人提供的证据情况综合考虑是否准许，依法保护当事人诉讼权利。当事人系新冠肺炎确诊患者、疑似新冠肺炎患者、无症状感染者以及相关密切接触者，在被依法隔离期间诉讼期限届满，根据该条规定申请顺延期限的，人民法院应予准许。

八、加大司法救助力度。对于受疫情影响经济上确有困难的当事人申请免交、减交或者缓交诉讼费用的，人民法院应当依法审查并及时作出相应决定。对于确实需要进行司法救助的诉讼参加人，要依据其申请，及时采取救助措施。

九、灵活采取保全措施。对于受疫情影响陷入困境的企业特别是中小微企业、个体工商户，可以采取灵活的诉讼财产保全措施或者财产保全担保方式，切实减轻企业负担，助力企业复工复产。

十、切实保障法律适用统一。各级人民法院要加强涉疫情民事案件审判工作的指导和监督，充分发挥专业法官会议、审判委员会的作用，涉及重大、疑难、复杂案件的法律适用问题，应当及时提交审判委员会讨论决定。上级人民法院应当通过发布典型案例等方式加强对下级人民法院的指导，确保裁判标准统一。

1. 民政部、卫健委：印发《新冠肺炎疫情社区防控与服务工作精准化精细化指导方案》 民发〔2020〕38号

各省、自治区、直辖市民政厅（局）、卫生健康委，各计划单列市民政局、卫生健康委，新疆生产建设兵团民政局、卫生健康委：

为贯彻落实党中央、国务院关于新冠肺炎疫情防控工作决策部署，根据疫情防控形势变化进一步完善应急处置和常态化防控相结合的机制与措施，提高社区防控与服务工作精准化精细化水平，助力全面推进复工复产，我们编制了《新冠肺炎疫情社区防控与服务工作精准化精细化指导方案》，现印发给你们，请结合实际参照执行。

附件：新冠肺炎疫情社区防控与服务工作精准化精细化指导方案

民政部 国家卫生健康委

2020年4月14日

附件

新冠肺炎疫情社区防控与服务工作精准化精细化指导方案

为指导城乡社区根据区域风险等级和疫情实际精准精细开展社区防控与服务工作，在常态化疫情防控中加快推进生产生活秩序全面恢复，制定如下指导方案：

一、总体要求

全面贯彻落实习近平总书记重要指示批示精神和党中央、国务院决策部署，遵循“外防输入、内防反弹”的总体防控策略和以县域为单位实施差异化防控的基本要求，根据县（市、区、旗）疫情风险等级和社区疫情划分，科学精准制定实施社区疫情防控措施，做好新冠肺炎治愈患者和解除医学观察人员回归融入社区相关工作，助力全面推进复工复产，努力减少疫情防控对社区居民正常生活的影响。

二、防控策略

统筹推进社区疫情防控和服务群众工作，完善城乡社区联防联控工作机制和群防群控组织体系，建立健全社区党组织领导，社区自治组织、社区卫生服务机构负责，社区物业服务企业、社区经济社会组织积极协同的社区防控组织机制，建立健全社区工作者、社区医务人员为主体，派驻下沉人员、基层民警、其它社区工作人员、入境人员工作（接待）单位代表和入住酒店工作人员、社会工作者、社区志愿者、社区居民广泛参与的社区防控工作队伍，压实属地、部门、单位、家庭和个人责任，坚持早发现、早报告、早隔离、早治疗，强化社区防控网格化管理和社区服务精细化供给，充分联系群众、组织群众、发动群众，引导和激励群众加强自我防护和自我服务，夯实疫情防控和经济社会发展的基层基础。

（一）低风险地区和未发现病例社区。

1.社区防控：实施“外防输入”策略，根据联防联控机制印发的社区（乡镇、村）新冠肺炎防控技术方案，做好组织动员、健康教育、信息告知、高风险地区返回人员管理、环境卫生治理、物资准备等工作。对于入境人员，要会同其家庭、工作（接待）单位、入住酒店对其进行主动追踪登记，纳入网格化管理，并根据当地疫情防控要求依法做好其本人及其相关人员的集中（居家）隔离医学观察、健康监测工作。对于高风险地区返回人员，如采取集中隔离医学观察的，应协助做好人员接送工作；如采取居家隔离医学观察的，应组织相关社区服务机构和志愿者做好生活物资保障、健康监测工作，并协助法定机构送达解除隔离医学观察通知书。对于非高风险地区返回人员，如已持有健康证明（居家隔离医学观察期满证明，以及防疫健康信息码、健康通行码绿码等），且乘坐“点对点”特定交通工具到达的，可不再实施隔离医学观察。注意发现和报告无症状感染者。

2.社区服务：聚焦恢复秩序提供服务。解除社区封闭式管理措施，允许外来人员和车辆进入，继续实施人员和车辆出入登记、体温监测，有条件的地方可建立快递员、配送员准入机制。恢复社区综合服务设施和服务站点开放，优先提供政务事项办理、就业信息咨询、图书外借归还等服务项目，并做好卫生防护和人员限流措施，加强对社区室内外活动场所和器材设施的日常消毒。支持与居民生活密切相关的社区超市（便利店、菜店）和美容美发、洗染、维修等社区生活性服务网点恢复营业，鼓励采取电话或网络预约等方式减少人员聚集。社区居民委员会要协助辖区企业复工复产，村民委员会要组织开展农业生产。

3.群众参与：倡导普遍参与，组织社区居民参与社区防控和社区服务，共同开展高风险地区人员管理、矛盾纠纷调解等相关工作，积极参与环境卫生整治，并加强对社区防控物资管理的监督。

4.信息化建设应用：推广运用社区二维码、社区居民二维码（防疫健康信息码）支持出入管理、高风险地区人员管理，推动社区居民二维码（防疫健康信息码）跨区域互认；依托出入境管理、交通运输领域大数据，实现入境人员、高风险地区返回人员管理的无缝衔接；依托社区防控产品移动客户端和社区公众号、微信群、QQ群等开展健康教育、信息宣传和活动组织等工作，探索建立社区矛盾纠纷线上调解机制。

（二）中风险地区和出现病例或暴发疫情社区。

1.社区防控：实施“外防输入、内防扩散”策略，根据社区（乡镇、村）新冠肺炎防控技术方案，在疾控等专业公共卫生机构指导下加强密切接触者管理和消毒工作。合理确定防控管理场所和人员，实施针对性防控措施。对于新冠肺炎确诊病例的密切接触者，应充分发挥基层医疗卫生机构医务人员和社区工作者网格化管理作用，做好排查和集中（居家）隔离医学观察工作。完善对返回社区的新冠肺炎治愈出院患者管理，会同专业机构落实隔离管理和健康状况监测。

2.社区服务：聚焦疫情防控和恢复秩序提供服务。完善社区封闭式管理措施，在对相关场所和人员采取防控措施的同时，完善社区人员和车辆分类机制，确保复工复产人员和车辆正常出入。暂停社区综合服务设施和服务站点开放，对于确有需要的可采取电话或网络预约方式上门服务。设立社区生活物资集中销售（配送）点，协调有资质供应商、大型连锁超市或社区周边超市，做好米面粮油、肉禽蛋奶和水果蔬菜等基本生活物资集中销售（配送）服务，确保社区居民生活物资充足供应。设立社区快递（外卖）集中配送区域，采取按居住地址分区放置等方式，避免社区居民小规模聚集；有条件的地方可提供代收服务，并组织专人分批配送。建立社区居民心理慰藉疏导机制，引导其适应社区封闭式管理生活。完善社区孤寡独居老人、监护缺失儿童、残疾人和一线医务工作者家属关爱照料措施，组织社区服务机构和志愿者做好走访探视、物资配送和生活照料。社区居民委员会要督促辖区企业落实疫情防控责任。

3.群众参与：倡导志愿参与，组织社区志愿者参与社区防控和社区服务，共同开展包括密切接触者管理、社区生活物资配送在内的相关工作。

4.信息化建设应用：推广运用社区二维码、社区居民二维码（防疫健康信息码）支持密切接触者管理。依托社区防控产品移动客户端，实施社区志愿者招募、项目对接和监督，对接专业服务资源提供心理慰藉疏导服务，积极拓展生活物资在线订购和配送服务功能。

（三）高风险地区和传播疫情社区。

1.社区防控：实施“内防扩散、外防输出、严格管控”策略，根据社区（乡镇、村）新冠肺炎防控技术方案，严格落实社区管控措施并限制人员聚集。

2.社区服务：聚焦疫情防控提供服务。严格社区封闭式管理要求，根据疫情防控要求，依法限制或禁止社区人员和车辆出入，对于发热呼吸道症状者患者、其他各类急症患者、孕（产）妇等，协调应急车辆并做好消毒工作。根据物资保障安排，做好社区居民生活物资集中采购和供应工作，在条件允许的情况下应尽量满足社区居民多样化需求，组织社区服务机构或志愿者进行统一配送。强化社区居民心理慰藉疏导工作。重点做好社区孤寡独居老人、监护缺失儿童、残疾人和一线医务工作者家属关爱照料，全力防止出现冲击道德底线问题。统筹加强对慢性病患者的健康服务和药品保障。

3.群众参与：倡导专业参与，组织有一定志愿服务经验的社区志愿者参与社区生活物资配送等必要工作，并落实社区志愿者防护措施。

4.信息化建设应用：推广运用社区二维码、社区居民二维码（防疫健康信息码）支持社区管控措施；运用公共安全视频联网监控等支持人员聚集限制措施；依托社区防控产品移动客户端做好生活物资订购、心理慰藉疏导工作。

三、防控保障

（一）加强组织领导。加强对社区防控工作的组织领导，完善各级应对疫情工作领导小组和联防联控机制领导下的社区防控工作机制，压实“四方”责任，落实“四早”措施，根据分区分级差异化防控原则，进一步细化实化社区防控各项措施，守牢守严疫情防控的社区防线。要科学研判疫情波及范围、严重程度、社会影响等因素，根据各地应急相应级别和县域疫情风险等级动态变化，及时调整完善社区防控策略，在工作力量配备、防控物资调配、应急机制完善等方面予以充分保障。根据各地重大突发公共卫生事件应急响应要求启动或终止社区防控工作。及时总结社区防控工作成效经验和短板不足，切实提高社区治理和服务能力，逐步完善社区应急管理体系。

（二）夯实防控基础。牢固树立社区防控阵地意识，强化社区防控网格化管理，推动防控数据向社区推送、防控资源和力量向社区下沉、防控关口向社区前移。全面落实疫情防控一线城乡社区工作者和医务人员关心关爱措施，切实保障补助和工伤待遇落实到位。坚决为城乡社区工作人员减压减负，除社区疫情防控需要依法出具的居住证明和解除隔离医学观察通知书外，不得以疫情防控为由要求城乡社区组织出具其他证明。建立健全社区防控工作力量统筹机制，将各级企事业单位下沉人员纳入社区防控工作队伍，由城乡社区组织根据疫情防控需要统筹使用。要抓紧完善社区防控物资保障机制，将社区防控物资纳入各级联防联控工作机制重点保障范围，做到统一调拨、统一管理、统一发放，提高社区防控设施设备特别是体温测量设施设备精度。

（三）保障群众利益。推进社区防控工作依法有序进行，探索建立过度防控行为警示通报机制，不得提出超出分区分级社区防控工作要求的防控措施；不得采取对社区管理“一封了之”、对居民出行“一禁了之”、对外来人口“一拒了之”等简单化防控手段；不得以疫情防控为名侵害群众人身权利、财产权利和合法权益。加强社区防控工作人文关怀，对于高风险地区、疫情严重国家返回人员，及时做好对接工作，帮助其在解除集中（居家）隔离医学观察后回归社区，并做好其共同生活的单元住户和社区居民思想工作；对于新冠肺炎确诊病例、疑似病例、无症状感染者、密切接触者和出现发热呼吸道症状者，及时给予关心慰藉，帮助其在治愈出院或排除新冠肺炎感染后融入社区，及时制止和引导消除针对上述人群的歧视性言行。扩大社区防控工作群众参与，推动社区防控、服务和信息化建设应用各项工作与群众需求精准对接，构筑群防群控的人民防线；做好面向广大社区居民的政策宣传、解释工作，增进广大社区居民对社区防控工作的理解认同。

1. 财政部等3部门：进一步加大创业担保贷款贴息力度 全力支持重点群体创业就业 财金〔2020〕21号

各省、自治区、直辖市、计划单列市财政厅（局）、人力资源社会保障厅（局），新疆生产建设兵团财政局、人力资源社会保障局，中国人民银行上海总部、各分行、营业管理部、省会（首府）城市中心支行、各副省级城市中心支行：

　　当前新冠肺炎疫情对就业创业造成一定影响。为深入贯彻落实习近平总书记在统筹推进新冠肺炎疫情防控和经济社会发展工作部署会议上的重要讲话精神，全面强化稳就业举措，更好发挥创业担保贷款贴息资金引导作用，加强资金保障，全力支持复工复产和创业就业，推动经济社会有序稳定发展，现通知如下：

　　一、扩大覆盖范围

　　（一）增加支持群体。自通知印发之日至2020年12月31日新发放贷款，应将下列群体纳入支持范围：一是受疫情影响较大的批发零售、住宿餐饮、物流运输、文化旅游等行业暂时失去收入来源的个体工商户；二是贷款购车专门用于出租运营的个人；三是贷款购车加入网络约车平台的专职司机（需平台提供专职司机“双证”等证明材料）；四是符合条件的出租车、网约车企业或其子公司；五是对已享受创业担保贷款贴息政策且已按时还清贷款的个人，在疫情期间出现经营困难的，可再次申请创业担保贷款。

　　（二）降低申请门槛。小微企业当年新招用符合条件创业担保贷款申请条件的人数与企业现有在职职工人数的占比，由20%下降为15%，超过100人的企业下降为8%。

　　二、适当提高额度

　　符合条件的个人最高可申请创业担保贷款额度由15万元提高至20万元。对符合条件的个人创业担保贷款借款人合伙创业的，可根据合伙创业人数适当提高贷款额度，最高不超过符合条件个人贷款总额度的10%。

　　三、允许合理展期

　　对流动性遇到暂时困难的小微企业和个人（含个体工商户，下同）创业担保贷款，可给予展期，最长可展期至2020年6月30日，展期期间财政给予正常贴息。对已发放的个人创业担保贷款，借款人患新冠肺炎的，展期期限原则上不超过1年。

　　四、降低利率水平

　　金融机构新发放创业担保贷款利率应适当下降，具体标准为：贫困地区（含国家扶贫开发工作重点县、全国14个集中连片特殊困难地区）贷款利率上限由不超过LPR+300BP下降为LPR+250BP，中、西部地区由不超过LPR+200BP下降为LPR+150BP，东部地区由不超过LPR+100BP下降为不超过LPR+50BP。具体贷款利率由经办银行根据借款人和借款企业的经营状况、信用情况等与借款人和借款企业协商确定。本通知印发之日前已发放和已签订合同但未发放的贷款，仍按原规定执行。

　　五、合理分担利息

　　自2021年1月1日起，新发放的个人和小微企业创业担保贷款利息，LPR-150BP以下部分，由借款人和借款企业承担，剩余部分财政给予贴息。

　　六、简化审批程序

　　推行电子化审批，逐步实行全程线上办理。各地人力资源社会保障部门（以下简称人社部门）可通过所在地社区、村委会、群团组织、金融机构、担保机构等推荐方式拓展创业担保贷款申请渠道，推广依托社会保障卡搭载创业担保贷款申请、审核和拨付功能。逐步推行“一站式”服务，实行人社部门审核借款人资格、担保机构尽职调查、金融机构贷前调查“多审合一”，避免重复提交材料。人社部门资格审核原则上应压缩在7个工作日内，担保机构尽职调查压缩在3个工作日内，金融机构贷款受理至发放原则上压缩在5个工作日内，确需办理反担保、抵押等手续的可适当延长。对不符合条件的，应在5个工作日内通知申请人并说明原因，一次性告知需补充完善的手续和资料。鼓励各地自主整合担保基金与经办金融机构办理流程，进一步提升服务效率。

　　七、免除反担保要求

　　自通知印发之日起，新发放的10万元及以下的个人创业担保贷款，以及全国创业孵化示范基地或信用社区（乡村）推荐的创业项目，获得设区的市级以上荣誉称号的创业人员、创业项目、创业企业，经金融机构评估认定的信用小微企业、商户、农户，经营稳定守信的二次创业者等特定群体，免除反担保要求。鼓励有条件的地方对其他创业担保贷款逐步降低或免除反担保要求。

　　八、提升担保基金效能

　　各地相关部门要简化担保条件和手续，制定担保基金尽职免责和激励约束办法，合理提升担保基金代偿比例和效率。实行担保基金放大倍数与贷款还款率挂钩机制，创业担保贷款上年到期还款率（上年累计到期贷款实际回收金额/上年累计到期贷款应回收金额）达到90%以上的，本年可适当提高放大倍数至担保基金存款余额的10倍。

　　九、鼓励地方加大支持力度

　　各地可适当放宽创业担保贷款借款人条件、提高贷款额度上限，由此额外产生的贴息资金支出由地方财政承担。

　　十、强化统筹协调与激励约束

　　财政、人民银行、人社部门要完善协作机制，加快健全完善创业担保贷款分类统计制度，加强部门间信息共享，充分整合资格审核、贴息、贷款发放等数据。人社部门负责做好资格审核工作。各担保基金运营管理机构和经办金融机构按季向当地人民银行分支机构、财政、人社部门报告担保基金和创业担保贷款发放使用情况。人民银行分支机构强化普惠金融定向降准考核、专项金融债发行等外部激励约束，引导经办金融机构提升服务质效；财政部门负责做好担保基金、财政贴息和奖补资金的管理工作，明确对担保基金来源和补偿机制，强化考核和监督检查，发挥好奖补资金激励作用，确保贴息、奖补资金及时拨付到位。

　　十一、政策衔接

　　本通知印发前已生效的创业担保贷款合同，仍按原合同约定执行。本通知无明确规定的，仍按照《中国人民银行 财政部 人力资源社会保障部关于实施创业担保贷款支持创业就业工作的通知》（银发〔2016〕202号）、《财政部 人力资源社会保障部 中国人民银行关于进一步做好创业担保贷款财政贴息工作的通知》（财金〔2018〕22号）、《财政部关于修订发布<普惠金融发展专项资金管理办法>的通知》（财金〔2019〕96号）等原有相关规定执行。

　　财政部

　　人力资源社会保障部

　　中国人民银行

　　2020年4月15日

1. 财政部等3部门：扩大内销选择性征收关税政策试点 财政部 海关总署 税务总局公告2020年第20号

为统筹内外贸发展，积极应对新冠肺炎疫情影响，现将有关事项公告如下：

　　自2020年4月15日起，将《财政部 海关总署 国家税务总局关于扩大内销选择性征收关税政策试点的通知》（财关税〔2016〕40号）规定的内销选择性征收关税政策试点，扩大到所有综合保税区。

　　特此公告。

　　财政部 海关总署 税务总局

　　2020年4月14日

1. 医保局等4部门：印发《关于外籍新冠肺炎患者医疗费用支付有关问题的通知》 医保发〔2020〕14号

各省、自治区、直辖市及新疆生产建设兵团医疗保障局，外事办公室，财政厅（局），卫生健康委：

根据当前新冠肺炎疫情防控形势，为认真落实“外防输入、内防反弹”的总体防控策略，妥善做好外籍新冠肺炎患者医疗费用支付等工作，现就有关事项通知如下：

一、各地有关部门要在当地应对疫情工作领导小组（指挥部）的领导下，密切配合，实时掌握外籍新冠肺炎患者有关信息，按规定做好救治工作和医疗费用结算。

二、外籍新冠肺炎确诊和疑似患者未参加我国基本医保的，医疗机构应当先救治后收费，确保应收尽收；医疗费用由患者个人负担。参加商业健康保险的，由商业保险公司按合同及时支付。

三、外籍新冠肺炎确诊和疑似患者参加我国基本医保的，基本医保、大病保险应按规定支付，其余费用由患者个人负担。

四、参加我国基本医保的外籍人员，留院观察期间发生的医疗费用，基本医保按规定支付。未参加我国基本医保的，由个人负担。

五、外籍人员集中隔离产生的费用，原则上由个人负担。

六、各地有关部门要妥善做好外籍新冠肺炎患者的救治和费用结算、监测等工作。遇有重大问题和情况，及时向国家医疗保障局、外交部、财政部和国家卫生健康委等部门报告。

国家医保局 外交部

财政部 国家卫生健康委

2020年4月3日

1. 教育部：做好2020年全国硕士研究生复试工作 教学厅〔2020〕4号

各省、自治区、直辖市高等学校招生委员会、教育厅(教委)、教育招生考试机构，新疆生产建设兵团教育局，有关部门（单位）教育司（局），各研究生招生单位：

　　研究生考试招生是国家选拔高层次专门人才的重要途径，复试是研究生招生考试的重要组成部分。当前我国疫情防控阶段性成效进一步巩固，但国际疫情持续蔓延，我国防范疫情输入压力不断加大。各地各招生单位要深入贯彻落实习近平总书记关于统筹推进新冠肺炎疫情防控和经济社会发展工作的重要讲话和重要指示批示精神，在确保安全性、公平性和科学性的基础上，统筹兼顾、精准施策、严格管理，稳妥做好2020年全国硕士研究生复试工作。

　　一、科学制定复试工作方案

　　1.精准划定专业分数线。各招生单位要认真执行《2020年全国硕士研究生招生工作管理规定》（以下简称《招生管理规定》）和《2020年全国硕士研究生招生考试考生进入复试的初试成绩基本要求》（以下简称国家分数线），在国家分数线基础上，自主确定并公布本单位各专业考生进入复试的初试成绩要求（以下简称专业分数线）和其他学术要求，不得出台歧视性或其他有违公平的规定。招生单位要综合考虑生源情况、招生计划、学科专业特点和复试考核工作需要等，精准划定各专业分数线。

　　2.自主确定复试办法。各招生单位要在省级高校招生委员会的统一领导下，统筹考虑当地疫情防控要求和学校实际情况，因地因校制宜，自主确定复试办法。复试时间由各招生单位统筹考虑当地疫情形势、应急响应级别以及复试工作量等，按照分区分级、精准防控、错时错峰、防止聚集的要求，综合研判自主确定。复试启动时间原则上不早于4月30日。复试方式由各招生单位根据学科特点和专业要求，在确保公平和可操作的前提下自主确定，可采取现场复试、网络远程复试、异地现场复试以及委托其他高校复试等。各招生单位要结合本地本单位情况，对拟采取的复试方式进行充分评估，确保复试安排的安全性、公平性和科学性。采取现场复试的，要提前制定复试工作流程、防控措施和应急预案，尽量减少不必要的环节，防止人员聚集。同时，落实好各项防护措施，做好突发情况下的应急处置准备。采取远程复试的，要对软件平台的适用性、安全性、可靠性和稳定性等功能进行充分评估，确保满足远程复试要求。招生单位复试办法须报省级教育行政部门疫情防控工作领导小组审核同意后向社会公布并组织实施。

　　3.科学设计复试内容。各招生单位要针对不同的复试方式、学科专业特点和办学特色，精心设计复试内容，确保复试考核科学有效、公平公正。要通过考生大学学习成绩单、毕业论文、科研成果、专家推荐信等补充材料，加强对考生既往学业、一贯表现、科研能力、综合素质和思想品德等情况的全面考查。采取远程复试的，应尽可能采用综合性、开放性的能力型试题。

　　二、严格复试组织管理

　　4.严格考生资格审查。各招生单位要严把复试入口关。在复试前要对考生的居民身份证、学生证、学历学位证书、学历学籍核验结果等进行严格审查核验，对不符合规定者，不予复试。采取远程复试的，要会同技术平台提供方，积极运用“人脸识别”“人证识别”等技术，并通过综合比对“报考库”“学籍学历库”“人口信息库”“考生考试诚信档案库”等措施，加强对考生身份的审查核验，严防复试“替考”。

　　5.严格复试过程管理。各招生单位要采取切实有效措施，加强复试过程规范管理。要建立健全“随机确定考生复试次序”“随机确定导师组组成人员”“随机抽取复试试题”的“三随机”工作机制。要发挥和规范导师在复试选拔中的作用，加强导师遴选和培训，强化保密意识、责任意识和法治意识，并提高导师运用新技术、新手段科学规范选拔人才的能力。要与考生逐一签订《诚信复试承诺书》，确保提交材料真实和复试过程诚信。要采取灵活有效的方式，加强复试过程监管，严防复试弄虚作假、徇私舞弊，高校对复试过程要全程录音录像。采取远程复试的，要选用统一的软件平台，强化技术支持和安全保障，提前组织模拟演练，确保复试过程安全、顺畅、稳定。依照《招生管理规定》，招生单位认为有必要时，可对相关考生再次复试。

　　6.严肃考风考纪。各招生单位要严格按照相关规定，严肃查处违规违纪行为。对在复试过程中有违规行为的考生，一经查实，即按照《国家教育考试违规处理办法》《普通高等学校招生违规行为处理暂行办法》等规定严肃处理，取消录取资格，记入《考生考试诚信档案》。入学后3个月内，招生单位要按照《普通高等学校学生管理规定》有关要求，对所有考生进行全面复查。复查不合格的，取消学籍；情节严重的，移交有关部门调查处理。

　　三、精心开展咨询指导

　　7.加强政策宣传解读。各地各招生单位要结合本地本单位复试工作安排，综合运用各类媒体特别是新媒体，做好相关政策的宣传解读。要及时、准确解读本地区本单位复试工作方案的内容和相关要求，特别是复试时间、方式、流程等安排，让社会和考生充分知晓，确保考生复试组织工作顺畅有序。

　　8.加强考生咨询服务。各地各招生单位要畅通考生联系咨询通道，安排专人受理考生咨询，及时为考生答疑解惑。采取远程复试的，要加强对考生参加远程复试工作的指导，向考生详细介绍有关软件平台使用办法、复试流程和相关要求等。省级教育招生考试机构要及时做好有关信息发布工作，方便考生查询阅知。4月26日至30日，教育部将在“中国研究生招生信息网”组织举办“2020年研考招生复试网上咨询”活动，各招生单位要安排专人在线解答考生咨询。

　　9.做好兜底支持保障。各地各招生单位要强化人性化关怀和个性化安排，积极采取有效措施，加强对贫困地区考生、残疾考生等特殊群体的关爱帮扶。对于不具备远程复试条件的考生，招生单位要进行技术兜底保障，根据考生申请积极协调生源所在地省级教育招生考试机构提供必要合理的支持和帮助。

　　四、切实加强组织领导

　　10.强化属地管理责任。根据疫情防控需要，2020年全国硕士研究生复试工作实行属地化管理。各省级教育行政部门、教育招生考试机构要会同卫生健康、公安等部门，加强对本地招生单位复试工作的统筹管理，指导招生单位科学制定复试工作方案并严格审核把关，统筹协调在本地设立考点进行异地复试的省外招生单位制定具体复试方案。各省级教育行政部门要会同教育招生考试机构制定本地区复试组织工作方案，并报省级疫情防控工作领导小组审定后组织实施。

　　11.落实招生主体责任。招生单位要切实落实研究生复试工作主体责任。主要负责同志是第一责任人，对本单位研究生复试工作要亲自把关、亲自协调、亲自督查。要提高政治站位，加强组织领导，统筹做好疫情防控与研考复试工作。各招生单位要充分考虑各方面情况和因素，认真研究确定复试办法和各院系实施细则。组织现场复试的，要按照疫情防控要求，加强考场、人员安排和现场秩序管理。组织远程复试的，要细化工作流程、考务调度、监督管理机制等。招生单位复试工作方案须分别报属地和拟设考点所在地的省级教育行政部门疫情防控领导小组审核同意。

　　12.强化疫情防控措施。各地各招生单位要把广大师生生命安全和身体健康放在第一位，坚持安全第一、生命至上，严格落实疫情防控工作要求，切实做好复试过程中的疫情防控工作。要采取有效措施，降低人员密度，防止人员聚集。要做好人员排查、场地安排、卫生消毒等工作。要遵循错时错峰工作要求，不同学院、不同专业要分时、分批有序安排复试。要制定复试期间突发事件应急预案，提前做好应对准备。

教育部办公厅

2020年4月13日

1. 卫健委、教育部：印发《大专院校新冠肺炎疫情防控技术方案》 国卫办疾控函〔2020〕304号

各省、自治区、直辖市及新疆生产建设兵团卫生健康委、教育厅（教委、教育局）：

　　为科学指导大专院校有效落实疫情防控措施，有序推进复学复课，针对高校学生学习生活环境状况、学生构成等不同特点，我们组织制定了《大专院校新冠肺炎疫情防控技术方案》。现印发给你们，请参照执行。

国家卫生健康委办公厅

教育部办公厅

2020年4月13日

（信息公开形式：主动公开）

大专院校新冠肺炎疫情防控技术方案

　　一、开学前

　　（一）学校的准备。

　　1.各地确保疫情得到有效控制，学校具备基本防控条件，师生和校园公共卫生安全得到切实保障后，作出错时错峰开学的决定，周密准备，分学校、分批次、分生源、分时段通知学生返校报到，有序推进学校开学复课。

　　2.严格落实属地责任、部门责任、单位责任和个人家庭责任，扎实做好学校疫情防控各项工作安排。

　　3.建立完善疫情防控联合工作机制。统筹调度开学复课疫情防控重点工作，密切沟通，加强协作，建立监测督查机制、应急快速反应机制、任务包干包片机制。形成教育、卫生、学校与医疗机构、疾控机构“点对点”协作机制。确保开学前学校疫情防控业务指导、巡查和培训全覆盖。

　　4.根据本地区疫情防控形势和学生来源特点，制定具体防控方案和应急预案，做好应急演练，提前熟悉掌握当地医疗服务预案。

　　5.落实学校主体责任，校长是本单位疫情防控第一责任人。做好人员、物资、场地、监测等防控条件准备，细化各项防控措施，制度明确，责任到人，确保每个细节、每个关键步骤落实到位，并进行培训、演练操作。

　　6.做好消毒剂、口罩、手套等防疫物资的储备，建立环境卫生和清洁消毒管理制度，由专人全面负责学校清洁消毒工作，包括消毒产品的管理、组织实施、工作监督等。

　　7.开学前对学校环境和空调系统进行彻底清洁，对物体表面进行预防性消毒处理，教室、食堂、宿舍、图书馆等所有场所开窗通风。

　　8.在学校内设立（临时）隔离室，位置相对独立，以备人员出现发热等症状时立即进行暂时隔离。

　　9.学校每日掌握教职员工及学生健康情况，实行“日报告”、“零报告”制度，并向主管部门报告；对全体教职员工开展防控制度、个人防护与消毒等知识和技能培训。

　　（二）教职工的准备。

　　1.每日做好自我健康监测和行踪报告，并如实上报学校，确保开学前身体状况良好。

　　2.按照学校要求，认真学习各项防控制度，并掌握个人防护与消毒等知识和技能。

　　3.符合返校条件的教职工可经校内相关部门、学院备案审批分批返校，做好开学准备和各项教学科研、管理服务工作。

　　（三）学生的准备。

　　1.每日做好自我健康监测和行踪报告，并如实上报学校，确保开学前身体状况良好。

　　2.在学校正式确定和通知返校时间前，遵守有关规定，不得提前返校。

　　3.返校前安心居家，做好在线学习，学习和掌握个人防护知识，并做好返校前物资准备。

　　二、返校途中

　　1.返校前确保身体状况良好，准备口罩等个人防护用品，有条件时可随身携带速干手消毒剂。

　　2.乘坐火车、飞机等公共交通工具时，需全程佩戴口罩，安检时短暂取下口罩，面部识别结束后立即戴上口罩，尽快通过安检通道。

　　3.做好手卫生，尽量避免直接触摸门把手、电梯按钮等公共设施，接触后及时洗手或用速干手消毒剂揉搓双手。注意个人卫生，避免用手接触口眼鼻，注意咳嗽礼仪。

　　4.尽量选择楼梯步行或扶梯，并与他人保持1米以上距离，避免与他人正面相对；若乘坐厢式电梯，与同乘者尽量保持距离，分散乘梯，避免同梯人过多。

　　三、开学后

　　（一）学校管理要求。

　　1.严格日常管理。坚持点名制度，每日掌握教职员工及学生动态、健康情况，加强对学生及教职员工的晨、午检工作，实行“日报告”、“零报告”制度，并向主管部门报告。

　　2.从严控制、审核各类涉及学生聚集性的活动，不组织大型集体活动。在封闭、人员密集或与他人近距离接触（小于等于1米）时教职员工和学生应佩戴口罩。

　　3.学生返校后不召开聚集性会议，可通过错峰开会、网络视频或提前录制会议材料等方式召开学生会议；鼓励开展网络教育课程或线上展示交流活动；确需开展现场活动的，需按规定向学校相关部门申请。

　　4.学校食堂采取错峰用餐，用餐桌椅同向单人单座并保持间隔1.5米；学生宿舍床位重新分配，减少人员并拉开距离；图书馆和实验室等公共场所实行人员限流。

　　5.加强物体表面清洁消毒。应当保持教室、宿舍、图书馆、学生实验室、体育活动场所、餐厅等场所环境卫生整洁，每日定期消毒并记录。对门把手、课桌椅、讲台、电脑键盘、鼠标、水龙头、楼梯扶手、宿舍床围栏、室内健身器材、电梯间按钮等高频接触表面，可用有效氯250-500mg/L的含氯消毒剂进行喷洒或擦拭，也可采用消毒湿巾进行擦拭。

　　6.加强重点场所地面清洁消毒。应当加强学校食堂、浴室及宿舍地面的清洁，定期消毒并记录。可使用有效氯500mg/L的含氯消毒液擦拭消毒。

　　7.各类生活、学习、工作场所（如教室、宿舍、图书馆、学生实验室、体育活动场所、餐厅、教师办公室、洗手间等）加强通风换气。每日通风不少于3次，每次不少于30分钟。课间尽量开窗通风，也可采用机械排风。如使用空调，应当保证空调系统供风安全，保证充足的新风输入，所有排风直接排到室外。

　　8.加强餐（饮）具的清洁消毒，餐（饮）具应当一人一具一用一消毒。餐（饮）具去残渣、清洗后，煮沸或流通蒸汽消毒15分钟；或采用热力消毒柜等消毒方式；或采用有效氯250mg/L的含氯消毒剂浸泡30分钟，消毒后应当将残留消毒剂冲净。

　　9.加强校园内、宿舍内垃圾分类管理。及时收集清运，并做好垃圾盛装容器的清洁，可用有效氯500mg/L的含氯消毒剂定期对其进行消毒处理。

　　10.严格落实学校工作人员的个人防护措施。校门值守人员、清洁人员及食堂工作人员等应当佩戴口罩。食堂工作人员还应当穿工作服，并保持工作服清洁，工作服应当定期洗涤、消毒。可煮沸消毒30分钟，或先用有效氯500mg/L的含氯消毒液浸泡30分钟，然后常规清洗。清洁消毒人员在配制和使用化学消毒剂时，还应当做好个人防护。

　　11.加强因病缺勤管理。学校做好缺勤、早退、请假记录，对因病缺勤的教职员工和学生及时追访和上报。

　　12.加强健康宣教课堂，由专人定期对学校内的教职员工和学生进行个人防护与消毒等防控知识宣传和指导。加强心理健康服务管理，为师生提供心理健康咨询服务和热线指导平台。

　　（二）学生管理要求。

　　1.学生到校时，应当按学校相关规定有序报到，入校前接受体温检测，合格后方可入校；无特殊情况，尽量避免家长进入校区。

　　2.在校期间，自觉按照学校规定进行健康监测，每天保持适量运动，选择人员较为稀疏的空旷开放空间进行室外运动。

　　3.学生在疫情防控期间不得出校，避免到人群聚集尤其是空气流动性差的场所。如必须出校，须严格履行请假程序，规划出行路线和出行方式。外出时，做好个人防护和手卫生，去人口较为密集的公共场所，乘坐公共交通工具、厢式电梯等必须正确佩戴口罩。

　　4.做好手卫生措施。餐前、便前便后、接触垃圾、外出归来、使用体育器材、学校电脑等公用物品后、接触动物后、触摸眼睛等“易感”部位之前、接触污染物品之后，均要洗手。洗手时应当采用洗手液或肥皂，在流动水下按照正确洗手法彻底洗净双手，也可使用速干手消毒剂揉搓双手。

　　5.宿舍定期清洁，并做好个人卫生。被褥及个人衣物要定期晾晒、定期洗涤。如需消毒处理，可煮沸消毒30分钟，或先用有效氯500mg/L的含氯消毒液浸泡30分钟后，再常规清洗。

　　四、出现疑似感染症状应急处置

　　1.教职员工或学生如出现发热、干咳、乏力、鼻塞、流涕、咽痛、腹泻等症状，应当立即上报学校负责人，并及时按规定去定点医院就医。尽量避免乘坐公交、地铁等公共交通工具，前往医院路上和医院内应当全程佩戴口罩。

　　2.教职员工或学生中如出现新冠肺炎疑似病例，应当立即向辖区疾病预防控制部门报告，并配合相关部门做好密切接触者的管理。

　　3.对共同生活、学习的一般接触者进行风险告知，如出现发热、干咳等呼吸道症状以及腹泻、结膜充血等症状时要及时就医。

　　4.专人负责与接受隔离的教职员工或学生的家长进行联系，掌握其健康状况。

　　五、境外师生返校的要求

　　1.境外师生未接到学校通知一律不返校，新生不报到。

　　2.境外师生返校前确保身体状况良好，返校途中做好个人防护和健康监测。

　　3.入境后严格执行当地规定，进行隔离医学观察，每日健康监测并填报健康卡，解除隔离后且身体健康方可返校学习和工作。

1. 文旅部、卫健委：做好旅游景区疫情防控和安全有序开放工作 文旅发电〔2020〕71号

各省、自治区、直辖市人民政府：

　　近日，一些旅游景区在恢复开放期间出现大量游客聚集拥挤现象，增加了疫情传播风险。各地要引以为戒，举一反三，加强疫情防控。为严格规范解禁后旅游景区管理，确保旅游景区安全有序开放，经国务院应对新型冠状病毒感染肺炎疫情联防联控机制同意，现将有关事项通知如下。

　　一、坚持防控为先，实行限量开放。各地在做好旅游景区疫情防控工作的前提下，坚持分区分级原则，严格落实《旅游景区恢复开放疫情防控措施指南》要求，做到限量、有序开放，严防无序开放。疫情防控期间，旅游景区只开放室外区域，室内场所暂不开放；旅游景区接待游客量不得超过核定最大承载量的30%。收费景区在实施临时性优惠政策前要慎重做好评估，防止客流量超限。

　　二、强化流量管理，严防人员聚集。旅游景区要建立完善预约制度，通过即时通讯工具、手机客户端、景区官网、电话预约等多种渠道，推行分时段游览预约，引导游客间隔入园、错峰旅游。严格限制现场领票、购票游客数量。要做好游客信息登记工作，通过预约或现场领票、购票游览的游客都应提供身份证号、联系方式等必要信息，有关身份信息应依法依规使用、避免泄露。旅行社和旅游客运经营者要严格落实有关防控指南要求，适当控制旅游车辆载客量。有条件的地区要充分发挥本地“互联网+旅游”服务平台的作用，并采取大数据分析等多种新技术手段，推动智慧旅游，科学分流、疏导游客，做到旅游景区流量管理关口前置，严控客流。

　　三、细化管理措施，规范游览秩序。旅游景区要配备必要人员设备，加强清洁消毒，严格落实体温筛检等防控措施，结合实际，配套使用“健康码”核验等手段。发现可疑人员应当劝阻其进入，进行暂时隔离，并立即通知当地卫生健康部门及时处置。要优化设置游览线路，防止线路规划不合理导致游客扎堆拥挤现象。要加强巡视巡查，指导游客做好安全防护，保持购票、游览、休息、餐饮等场所人员间距。要加强各类旅游设备设施和消防装备器材安全隐患排查治理，强化野外火源管控。要在旅游景区出入口、重要参观点等容易形成人员聚集的区域设置专人，加强疏导，避免拥堵，确保防控到位。

　　四、做好宣传引导，倡导文明旅游。各地要通过官方网站、第三方平台、提示牌、广播、电子显示屏等方式，发布旅游景区恢复开放管理措施、疫情防控指南和森林防火知识、灾害天气预警信息，引导游客遵守旅游活动中的安全警示规定，帮助游客增强防护意识、掌握防护知识，引导游客自觉佩戴口罩，遵守公共秩序，积极配合防控工作，推进文明旅游。

　　五、加强组织领导，落实责任分工。各地要对旅游景区开放负主体责任，建立督导机制，必要时向重点旅游景区派出督导组，切实加强旅游景区开放和旅游安全检查工作。要指导旅游景区健全应急机制，分类完善应急预案，明确疫情防控应急措施和处置流程，落实落细防控责任，严防恢复运营引发各类安全事故。各地要健全部门联动机制，提高应急处置能力，遇到突发情况，要及时妥善处理，确保各项措施执行到位。

文化和旅游部 国家卫生健康委

2020年4月13日

1. 教育部：新冠肺炎疫情期间暂停恢复大型体育活动和聚集性活动

各省、自治区、直辖市教育厅（教委），新疆生产建设兵团教育局，部属各高等学校、部省合建各高等学校：

　　当前全国本土疫情传播已基本阻断，但境外疫情呈加速扩散蔓延态势，我国疫情输入压力持续加大。大型体育活动和聚集性活动人员集散量大、流动量大，疫情传播风险较大。为切实保障广大师生生命安全和身体健康，现就新冠肺炎疫情期间暂停恢复大型体育活动和聚集性活动有关事宜通知如下。

　　一、扎实做好学校疫情防控工作

　　各地各校要坚决贯彻落实习近平总书记关于坚决打赢疫情防控阻击战的重要指示精神，切实增强“四个意识”、坚定“四个自信”、做到“两个维护”，坚持实施“外防输入、内防反弹”的防控策略，针对当前境外输入型病例与风险增加的严峻形势，继续保持高度警惕，坚决防止麻痹侥幸心理，压实防控责任，落实防控措施。

　　二、暂停恢复大型体育活动和聚集性活动

　　为减少人员聚集和流动给疫情防控带来的风险，新冠肺炎疫情期间暂停恢复学校体育单项赛事、综合性运动会等大型体育活动和聚集性活动。各地各校要坚持审慎原则，完善有关组织和工作机制，提前谋划相关活动预案，做好活动赛期等调整工作。

　　三、创新形式推进学校体育教学改革

　　各地各校要针对疫情防控要求，统筹调整年度体育教育教学、运动训练与学生锻炼活动计划，合理制定教学组织实施方案。要充分利用网络平台，深入推进学校体育教学改革，聚焦“教会、勤练、常赛”目标，注重“教健康知识、传运动技能、练身体素质、育品德意志”，不断丰富学校体育教学、训练、竞赛等组织形式，减少人员聚集，帮助学生通过体育锻炼“享受乐趣、增强体质、健全人格、锤炼意志”。

　　各地各校要高度重视，切实加强组织领导，相关工作进展及时向教育部应对新冠肺炎疫情工作领导小组办公室报告。教育部将根据疫情防控形势及时对有关活动安排作出调整。

教育部应对新冠肺炎疫情工作领导小组办公室

（教育部办公厅代章）

2020年4月1日

1. 商务部办公厅关于创新展会服务模式 培育展览业发展新动能有关工作的通知

创新展会服务模式是党中央、国务院的重大部署，是在新冠肺炎疫情防控常态化条件下推动行业加快恢复和发展的重要举措。为深入贯彻习近平总书记关于统筹推进新冠肺炎疫情防控和经济社会发展工作等重要讲话和指示批示精神，推进展会服务创新、管理创新、业态模式创新，加快培育行业发展新动能，发挥展览业在扩大对外开放、增加社会就业、拉动消费增长等方面的重要作用，助力稳住外贸外资基本盘，现就有关事项通知如下：

一、统筹做好疫情常态化防控和展览业复工复产工作

（一）根据中央疫情防控有关精神和国务院工作部署，在做好疫情精准防控的同时，支持展览企业有序复工复产。在当前线下展会暂不开展的情况下，积极支持企业线上办展、线上参展。各地对线上展会活动要加强工作指导，做好政策引导和规范管理，提供服务便利。

（二）根据疫情形势变化，各地要按照国家和当地疫情防控相关要求，结合实际，动态调整展会活动安排。按照属地化管理原则，科学制定好复工复产防控措施，建立健全防控工作责任制和管理制度，落实主体责任。做好常态化疫情防控，提高应急处置能力。

二、加快推进展览业转型升级和创新发展

（三）积极打造线上展会新平台。推进展会业态创新，积极引导、动员和扶持企业举办线上展会，充分运用5G、VR/AR、大数据等现代信息技术手段，举办“云展览”，开展“云展示”、“云对接”、“云洽谈”、“云签约”，提升展示、宣传、洽谈等效果。

（四）促进线上线下办展融合发展。大力推动传统展会项目数字化转型，整合现有展会资源，打造网络展会集群，鼓励政府主办的线下展会率先线上开展，支持专业展会主办机构将线下品牌展会项目开通线上展览，探索线上线下同步互动、有机融合的办展新模式。

（五）培育线上展会龙头企业和品牌展会。支持开展跨城市、跨区域办展合作，着力培育一批具有先进办展理念、运营规范、模式创新成效显著的龙头在线展览企业，支持打造市场竞争力强、带动作用大的线上品牌展会，有效发挥示范、带动和辐射作用。

三、积极利用展会平台开拓国际市场

（六）助力企业抓订单、保客户。积极利用商务部和地方境外办展项目，在深耕传统市场的同时，加大“一带一路”沿线国家及新兴市场国家开拓力度，支持企业抓订单、促出口。引导企业充分利用各种技术手段，依托网络平台做好展前对接、线上推介、现场直播、远程洽谈签约等，努力保住老客户，吸引新客户。

（七）创新展会国际营销模式。整合政府、办展机构、行业组织、我驻外经商机构以及跨境电商平台等多方资源，推动展会信息互通，统筹线上线下渠道，强化展会国际营销和对外宣传推广，提升重点品牌展会国际影响力和知名度。推动优势展会资源整合，建立合作共享展会协同发展机制，形成开拓国际市场合力。

（八）深化展会国际合作。积极开展贸易促进服务，加强与境外专业展览机构和成熟展会项目合作，优化境外办展结构，鼓励自办展会与国际展会品牌对接合作，打造区域性国际展会品牌。

四、多措并举做好政策支持和保障

（九）推动政策落地见效。落实既有政策，指导展览企业用足用好国家和地方出台的财政税收、金融保险、复工复产保障等各项惠企政策措施，降低疫情造成的损失。加强政策实施效果跟踪评估，及时反映政策落实中存在的问题。

（十）用足用好财政资金。在外经贸发展专项资金规定支持范围内，支持各地因地制宜制定细化线上线下国际性展会扶持举措。鼓励各地积极出台展览业专项支持政策，发挥地方财政资金和相关产业引导基金作用，支持展览业尽快复苏。

（十一）持续推进简政放权。落实好《商务部办公厅关于进一步优化涉外经济技术展行政服务事项的通知》（商办服贸函〔2020〕52号），整合完善政务平台，依托商务部“展览业信息管理应用”系统，优化展会在线审批和备案管理流程，全面推行在线办理，推动实现“一网通办”。统筹监管资源，夯实属地监管，做好展会事中事后监管。

（十二）优化公共服务。各地要依托展览业重点联系企业制度，督促属地展馆统筹做好展会排期、信息公布及预警疏导。积极协调公安、市场监管、海关、卫生防疫等部门，对因疫情影响再次申请审批和备案的展会提供服务便利，做好因疫情未办展会相关善后工作。发挥中介组织作用，支持行业商会、协会与展览企业联合办展，及时为企业传递信息，反映诉求，提供法律支持和咨询等服务。

（十三）加强展会知识产权保护。切实提升线上线下展会各参与方知识产权保护意识，完善展会知识产权纠纷协调处理机制，有效保护参展商等各参与方合法权益。

各级商务主管部门要以习近平新时代中国特色社会主义思想为指导，增强“四个意识”，坚定“四个自信”，做到“两个维护”，坚决贯彻党中央、国务院决策部署。面对新形势新变化，各地区要加强形势研判预判，建立健全工作机制，在实践中不断创新管理和服务模式，密切关注展览业生产经营中遇到的难点、堵点，及时梳理存在的问题和企业诉求，加强工作对接，协调推动解决，认真总结创新展会服务模式的好经验、好做法，做好提炼推广，开展互鉴交流。有关工作进展情况及时上报。

商务部办公厅

2020年4月13日

1. 交通运输部等5部门：疫情防控期间针对伤病船员开展紧急救助处置工作 交海明电〔2020〕127号

各省、自治区、直辖市、新疆生产建设兵团交通运输厅（局、委）、外事办、卫生健康委，海关总署广东分署，各直属海关，各出入境边防检查总站，长江航务管理局，各直属海事局，各省级海上搜救中心：

　　为深入贯彻习近平总书记关于新冠肺炎疫情防控的重要指示批示精神，全面落实党中央、国务院关于统筹推进疫情防控和经济社会发展的决策部署，指导疫情防控期间伤病船员紧急救助处置工作有效开展，切实履行国际公约规定的责任和义务，现提出如下意见。

　　一、中国籍船舶在境外期间，在船船员伤病情况处置

　　（一）中国籍船舶第一时间向航运公司报告，航运公司接报后应当及时报告我海事管理机构，船舶同时可以按程序和相关国际指南申请远程医疗指导。

　　（二）船员需要送岸紧急救助的，船舶可向我驻外相关使领馆报告求助，请使领馆根据船上求助需求、船员伤病情况和当地医疗条件，协调当地政府及医疗部门履行《2006年海事劳工公约》《国际卫生条例》等规定的国际义务救助伤病船员。航运公司作为责任主体应当提供必要的资源保障。

　　（三）我海事管理机构要督促航运公司按照应急事件处置程序，为伤病船员提供紧急救助所必需的保障。

　　（四）若船舶发现确诊或疑似病例，且沿岸国或港口国拒绝提供紧急救助情况下，由交通运输部及时通报外交部，通过外交渠道敦促外方就近安排伤病船员实施紧急救助；船员可根据《2006年海事劳工公约》有关规定向当地港口当局求助。

　　二、外国籍船舶在境外期间，在船中国籍船员伤病情况处置

　　（一）海员外派机构接报后，按照“谁派出，谁负责”的原则，应先行向我驻外相关使领馆报告求助，并及时将有关情况向我海事管理机构报告。

　　（二）我海事管理机构应当督促海员外派机构按照应急事件处置程序，为伤病船员提供紧急救助所必需的保障，并及时将有关情况向交通运输部报告。

　　（三）交通运输部在必要时可联系船旗国主管机关协调处理。

　　三、船员在我国境内港口出现伤病情况处置

　　（一）船舶请求救助时，对在我国境内港口出现伤病的船员，无论是否属于“四类人员”，均应采取救治措施，必要时转运至口岸所在地相关医疗机构就诊，保障船员及时得到救助。在接到船舶救助请求后，所属辖区海上搜救中心应及时掌握船上需救助船员的信息，以及所有在船船员健康状况。相关海事管理机构按程序向海关、边检、地方人民政府（包括卫健部门）报告，配合有关部门做好船上是否存在疫情的确认工作，以便在存在疫情情况下，对“四类人员”采取合理的救助处置方式。

　　（二）海上搜救中心根据相关应急处置程序做好救助行动的相关协调处理，并配合相关部门按照港口所在地地方人民政府关于疫情防控期间的相关规定进行处置，及时、稳妥对伤病船员进行救助。

　　（三）必要时，海事管理机构应当对船员转运和救助过程中的水上交通秩序进行重点维护，配合相关部门开展救助工作。

　　（四）地方相关部门根据有关情况做好对外国驻华使领馆通报工作。

交通运输部 外交部 国家卫生健康委 海关总署 国家移民管理局

2020年4月12日

1. 交通运输部等6部门：精准做好国际航空货运机组人员疫情防控工作 交运明电〔2020〕128号

各省、自治区、直辖市、新疆生产建设兵团应对新冠肺炎疫情联防联控机制，各省、自治区、直辖市、新疆生产建设兵团交通运输厅（局、委）、卫生健康委、邮政管理局，海关总署广东分署、各直属海关，各出入境边防检查总站，各民航地区管理局：

　　当前，境外新冠肺炎疫情加速扩散蔓延，外防输入形势严峻复杂。同时，受疫情冲击国际航空货运能力大幅下降，对我国外贸进出口和国际供应链带来严重影响。为深入贯彻习近平总书记等中央领导同志重要指示批示精神，推动落实党中央、国务院关于统筹推进疫情防控和经济社会发展工作的决策部署，努力实现坚决阻断病毒通过交通运输工具境外输入传播渠道，保障国际物流运输通道不断，切实维护国际供应链稳定，按照国务院应对新冠肺炎疫情联防联控机制部署要求，现就精准做好国际航空货运机组人员疫情防控工作通知如下：

　　一、对国际航空货运机组人员实施严格封闭管理

　　（一）对机组人员实行封闭管理。国际航空货运机组人员下机休息后继续执飞国际航线的，海关登临检疫无异常后，按照不入境、不检测、指定地点集中休息的方式由所在城市人民政府负责实行封闭管理。对于具备相关条件的机场，在机场设立专门区域用于国际航空机组人员休息，要配备生活必需的相关设施设备，为机组人员休息创造良好条件。对于不具备相关条件的，机场所在地城市人民政府要在综合考虑机场、航空公司意见基础上，在机场附近指定地点（酒店、宾馆）用于国际航空货运机组人员集中封闭休息，从机场到集中封闭休息地点实行点对点专车接送。定点专车司乘人员、集中封闭区域工作人员应做好个人防护。

　　（二）严格管理机组人员活动范围。机组人员要加强自律，不得擅自离开集中封闭区域，不得到机场、指定酒店和宾馆的公共场所活动。要通过安排专人值守、视频监控等方式，对机组人员实行严格管理。

　　（三）加强入境后执飞国内航线机组人员防控管理。国际航空货运机组人员入境后继续执飞国内航线的，应在第一入境口岸接受核酸检测，检测结果确认前由当地人民政府实施管控，检测结果为阴性的，方可继续执飞。

　　二、优化国际航空货运机组人员出入境通关防控措施

　　（四）建立机组人员白名单制度。 允许国际航空货运相关企业对于符合健康标准的在飞国际机组人员建立白名单制度，相关口岸应为已报备的机组人员提供出入境通关便利。

　　（五）实施集中检疫通关。各地海关要对国际航空货运下机机组人员实行集中办理、快速通关的政策，根据海关检疫通关总体流量情况，临时设置专用通道，优先安排货运机组检疫通关，避免机组与旅客聚集接触，减少交叉感染风险。

　　（六）入境机组人员实施分类精准管理。对于中国航空企业国际货运机组，执行任务期间仅在国外机场短暂停留且严格按照要求做好防护而未入境的，由机组人员和航空公司分别作出未下机承诺并承担相应的责任担保，返航入境时免于核酸检测和集中隔离，体温检测符合规定的可继续执行货运飞行任务；执行任务期间在国外入境短期停留、实施封闭管理的，返回国内入境时，需按要求进行核酸检测，检测结果为阴性的免于集中隔离。

　　（七）简化固定航线机组人员管控措施。对执行固定往返航线的国际航空货运机组人员，体温检测符合规定的，不需采取异地作业隔离14天的管控措施。

　　（八）做好核酸检测阳性等外籍货运机组人员出境工作。执飞我国货运航班的外籍机组人员，属于有症状者、密切接触者或核酸检测阳性者，申请返回本国隔离治疗并由本人作出承担一切责任承诺的，经地方联防联控机制同意，由承运人实施严格防护后，可允许返回。

　　三、严格落实新冠肺炎疫情防控责任

　　（九）严格落实企业主体责任。国际航空货运企业是机组人员防疫防控第一责任人，要严格落实疫情防控各项要求，遵守检疫检测规定，细化停港作业、自我防护、集中休息等各项防控措施，切实加强对货运机组的管理。要细化落实机长负责制，切实加强对机组人员健康状况的定期检测，督促按照防疫指南的要求做好个人防护。要配足配齐机组人员各类防护用品，保障机组人员饮食和相关生活必需品供给，避免机组人员不必要的外出。

　　（十）严格落实行业监管责任。各民航地区管理局、各地邮政管理局要落实行业监管责任，按照职责分工加强对航空货运企业、邮政快递企业的监管，督促指导建立健全并严格执行疫情防控工作各项制度，积极配合地方政府落实机组人员封闭管理制度。各地交通运输、卫生健康和海关、出入境边防检查等部门要根据自身职责，进一步优化相关管控措施，做好国际航空货运机组检疫相关工作。

　　（十一）严格落实地方政府属地责任。相关地方人民政府要严格落实属地责任，尽快确定航空货运机组人员集中封闭休息地点。要组织卫生健康、交通运输、公安等相关部门，安排专门力量，切实加强对国际航空货运机组的管理，有效控制其活动范围和疫情传播风险。北京地区应按首都严格进京管理联防联控协调机制相关规定执行。

　　四、加强国际合作，推进全球航空货运疫情联防联控

　　（十二）推进联防联控。继续推动货运通航国家和地区与我强化联防联控，对国际航空货运机组加强出境检疫，避免机组人员“带病”执飞。

　　请各省、自治区、直辖市、新疆生产建设兵团应对新冠肺炎疫情联防联控机制将本通知精神传达到开通国际航空货运航线的城市人民政府应对新冠肺炎疫情联防联控机制，督促抓好贯彻落实。

交通运输部

国家卫生健康委

海关总署

国家移民管理局

中国民用航空局

国家邮政局

2020年4月13日

1. 商务部、扶贫办：切实做好扶贫农畜牧产品滞销应对工作

各省、自治区、直辖市、计划单列市和新疆生产建设兵团商务、扶贫主管部门：

今年以来，受新冠肺炎疫情等因素影响，部分扶贫农畜牧产品出现滞销。党中央、国务院高度重视。为贯彻落实习近平总书记重要指示和党中央、国务院决策部署，有效应对扶贫农畜牧产品滞销，现就有关事项通知如下：

一、着力解决扶贫农产品滞销问题

（一）组织采购对接。组织农产品流通企业、批发市场商户，与 贫困地区生产基地、龙头企业、农民专业合作社开展对接，优先支 持湖北省农畜牧产品销售，优先安排贫困地区尤其是尚未脱贫摘 帽国家级贫困县的扶贫农畜牧产品销售。通过补贴、贴息、储备轮换等方式，支持流通企业增加商业库存，就地就近解决农畜牧产品 滞销。推进地、市、县区域内''点对点”精准帮扶对接，支持设立农牧民直销点，鼓励大型农产品批发市场和公益性农产品示范市场减免费用、设立专区，优先销售贫困地区的滞销农畜牧产品。

（二）开展消费扶贫。落实国务院扶贫办等7部门《关于开展消费扶贫行动的通知》（国开办发〔2020〕4号）要求，通过政府采购、东西部扶贫协作、经营主体和社会组织参与等模式，扩大列入 《全国扶贫产品目录》的扶贫产品（以下简称扶贫产品）销售。按照 《财政部国务院扶贫办关于运用政府采购政策支持脱贫攻坚的通知》（财库〔2019〕27号）要求，迅速组织采购扶贫产品。指导东部发达省份和中西部省份大中城市为扶贫产品提供销售平台和渠道，落实采购任务指标。

（三）保障物流畅通。协调公安、交通运输等部门，畅通省内省 际扶贫农畜牧产品运输，确保车辆应享尽享“三不一优先"（不停 车、不检查、不收费、优先通行）等鲜活农产品“绿色通道"政策。组织农产品运输企业、物流集散中心与批发市场、生产基地、龙头企业、农民专业合作社加强合作，优先配送滞销农畜牧产品（包括扶贫产品）。引导物流企业针对贫困地区，采取物流配送费用优惠政策。

（四）抓好复工复产。有序组织农产品批发市场、农贸市场、超市等商业网点复工开业，保障扶贫农畜牧产品销售渠道畅通。支持农畜牧产品经销主体进村采购，维护正常产销秩序。积极引导 餐饮企业恢复营业，扩大消费规模。鼓励消费者增加本地应季农畜牧产品消费，降低滞销风险。

（五）落实支持政策。及时将与疫情防控重点物资保障相关的 农产品批发市场纳入国家专项再贷款和贴息政策支持范围。落实《商务部办公厅财政部办公厅关于疫情防控期间进一步做好农商 互联完善农产品供应链体系的紧急通知》（商办建函〔2020〕53号） 要求，积极支持疫情期间承担农产品保供任务的流通企业。

二、抓紧建立农产品滞销应对机制

（六）做好预测预警和应急防控。会同农业农村等部门，掌握在田农产品生产和上市情况，建立健全主产县农畜牧产品产销信 息共享机制，及时发布农畜牧产品供应、需求、价格等信息。综合研判重点农产品种植养殖规模、市场购销价格、主销区市场需求等 情况，做好预测预警和信息引导。建立农产品滞销应急响应制度, 提前制定应急预案。建立解决农畜牧产品滞销的骨干流通企业队伍，提前落实销售渠道。

（七）巩固产销合作机制。鼓励大型农产品批发市场建立滞销农畜牧产品（包括扶贫产品）“绿色通道''，推动大型连锁超市设立 滞销农畜牧产品（包括扶贫产品）销售专档、专区、专柜，简化采购程序，降低准入门槛。鼓励扩大生产基地直采规模，发展产供直 销，采取订单帮扶模式开展定向帮扶。引导滞销农畜牧产品产区 所在地、市、州及省内其他城市农贸市场、菜市场等零售终端设立直销专区，允许农民免费进场销售滞销农畜牧产品。鼓励开展农 社合作，引导生产扶贫产品的农民专业合作社运输车辆进入社区直接销售。

（八）举办产销对接活动。举办对接会、洽谈会、展示会、采购 会等各类产销对接活动，推广扶贫产品，重点组织国家级贫困县参与活动，为带贫企业和农民专业合作社做好服务。积极向贫困户 提供农资、农技、金融、品牌营销等专业跟踪服务，推动带贫生产经营主体与了解市场需求的采购商建立长期联系。

（九）开展电商助农行动。有关地方商务主管部门要组织2018年.2019年国务院激励的20个农村电商典型县市，分别与1—2个未脱贫县建立对接帮扶机制，在商品互通、市场共享、人员培训、工作指导等方面开展精准帮扶。引导电商企业开通滞销地 区农产品线上销售绿色通道，提供账号、流量等支持。动员社交电商、社区团购、小视频等新型电商平台，通过专区专栏、直播带货、专题活动等形式销售湖北特色农产品、贫困地区扶贫产品、滞销农畜牧产品。支持贫困户和农民专业合作社开办网上商店，与农民专业合作社、扶贫产品生产企业、种养大户建立直采直供关系。鼓励电商平台培训帮扶贫困地区商户，减免流量费用，提供协助运营、店铺诊断、广告投放等服务。

三、工作要求

（十）强化横向协作和纵向联动。各地商务、扶贫主管部门要 加强与农业农村等部门的沟通协调，推动“菜篮子"市长负责制落 地落实。搭建不同层次的滞销扶贫农畜牧产品对接平台，推动学校、医院、机关食堂和交易市场从贫困地区直接采购农畜牧产品。 促进交通、供销、邮政及电商、快递资源共享衔接，鼓励多站合一、服务同网。贫困地区商务主管部门要做好生产调度和货源组织。有东西部扶贫协作和对口支援任务的省级商务主管部门要定向定 点承包贫困地区农畜牧产品销售，把扶贫产品采购与机关工会福利挂钩，扩大采购规模。

各地商务主管部门要确定农畜牧产品滞销应对工作的分管负责人和处室联系人（联系方式于4月15日前报商务部），并按季度报送本地区农畜牧产品滞销应对工作情况。如出现大范围农畜牧产品滞销情况，请及时报告商务部，并通过有关信息平台发布滞销信息。各地扶贫部门要按照关于扶贫产品销售数据统计监测的有 关要求，会同商务主管部门，及时汇总有关平台和渠道采购、销售扶贫产品的相关数据，定期报送国务院扶贫办。

2020.4.13

1. 发改委、网信办：推进“上云用数赋智”行动 培育新经济发展 发改高技〔2020〕552号

各省、自治区、直辖市发展改革委、网信办：

  为深入贯彻落实习近平总书记关于统筹推进疫情防控和经济社会发展工作的重要指示批示精神，按照党中央、国务院决策部署，充分发挥技术创新和赋能作用抗击疫情影响、做好“六稳”工作，进一步加快产业数字化转型，培育新经济发展，助力构建现代化产业体系，实现经济高质量发展，国家发展改革委、中央网信办研究制定了《关于推进“上云用数赋智”行动 培育新经济发展实施方案》。现印发你们，请认真组织实施，推进中遇到的问题、形成的好做法请及时报国家发展改革委、中央网信办。国家数字经济创新发展试验区要积极行动，大胆探索，推进各项任务加快实施。

国家发展改革委

中央网信办

2020年4月7日

关于推进“上云用数赋智”行动

培育新经济发展实施方案

为深入实施数字经济战略，加快数字产业化和产业数字化，培育新经济发展，扎实推进国家数字经济创新发展试验区建设，构建新动能主导经济发展的新格局，助力构建现代化产业体系，实现经济高质量发展，特制定本实施方案。

  一、发展目标

  在已有工作基础上，大力培育数字经济新业态，深入推进企业数字化转型，打造数据供应链，以数据流引领物资流、人才流、技术流、资金流，形成产业链上下游和跨行业融合的数字化生态体系，构建设备数字化-生产线数字化-车间数字化-工厂数字化-企业数字化-产业链数字化-数字化生态的典型范式。

  打造数字化企业。在企业“上云”等工作基础上，促进企业研发设计、生产加工、经营管理、销售服务等业务数字化转型。支持平台企业帮助中小微企业渡过难关，提供多层次、多样化服务，减成本、降门槛、缩周期，提高转型成功率，提升企业发展活力。

  构建数字化产业链。打通产业链上下游企业数据通道，促进全渠道、全链路供需调配和精准对接，以数据供应链引领物资链，促进产业链高效协同，有力支撑产业基础高级化和产业链现代化。

  培育数字化生态。打破传统商业模式，通过产业与金融、物流、交易市场、社交网络等生产性服务业的跨界融合，着力推进农业、工业服务型创新，培育新业态。以数字化平台为依托，构建“生产服务+商业模式+金融服务”数字化生态，形成数字经济新实体，充分发掘新内需。

  二、主要方向

  （一）筑基础，夯实数字化转型技术支撑。

  加快数字化转型共性技术、关键技术研发应用。支持在具备条件的行业领域和企业范围探索大数据、人工智能、云计算、数字孪生、5G、物联网和区块链等新一代数字技术应用和集成创新。加大对共性开发平台、开源社区、共性解决方案、基础软硬件支持力度，鼓励相关代码、标准、平台开源发展。

  （二）搭平台，构建多层联动的产业互联网平台。

  培育企业技术中心、产业创新中心和创新服务综合体。加快完善数字基础设施，推进企业级数字基础设施开放，促进产业数据中台应用，向中小微企业分享中台业务资源。推进企业核心资源开放。支持平台免费提供基础业务服务，从增值服务中按使用效果适当收取租金以补偿基础业务投入。鼓励拥有核心技术的企业开放软件源代码、硬件设计和应用服务。引导平台企业、行业龙头企业整合开放资源，鼓励以区域、行业、园区为整体，共建数字化技术及解决方案社区，构建产业互联网平台，为中小微企业数字化转型赋能。

  （三）促转型，加快企业“上云用数赋智”。

  深化数字化转型服务，推动云服务基础上的轻重资产分离合作。鼓励平台企业开展研发设计、经营管理、生产加工、物流售后等核心业务环节数字化转型。鼓励互联网平台企业依托自身优势，为中小微企业提供最终用户智能数据分析服务。促进中小微企业数字化转型，鼓励平台企业创新“轻量应用”“微服务”，对中小微企业开展低成本、低门槛、快部署服务，加快培育一批细分领域的瞪羚企业和隐形冠军。培育重点行业应用场景，加快网络化制造、个性化定制、服务化生产发展，推进数字乡村、数字农场、智能家居、智慧物流等应用，打造“互联网+”升级版。

  （四）建生态，建立跨界融合的数字化生态。

  协同推进供应链要素数据化和数据要素供应链化，支持打造“研发+生产+供应链”的数字化产业链，支持产业以数字供应链打造生态圈。鼓励传统企业与互联网平台企业、行业性平台企业、金融机构等开展联合创新，共享技术、通用性资产、数据、人才、市场、渠道、设施、中台等资源，探索培育传统行业服务型经济。加快数字化转型与业务流程重塑、组织结构优化、商业模式变革有机结合，构建“生产服务+商业模式+金融服务”跨界融合的数字化生态。

  （五）兴业态，拓展经济发展新空间。

  大力发展共享经济、数字贸易、零工经济，支持新零售、在线消费、无接触配送、互联网医疗、线上教育、一站式出行、共享员工、远程办公、“宅经济”等新业态，疏通政策障碍和难点堵点。引导云服务拓展至生产制造领域和中小微企业。鼓励发展共享员工等灵活就业新模式，充分发挥数字经济蓄水池作用。

  （六）强服务，加大数字化转型支撑保障。

  鼓励各类平台、开源社区、第三方机构面向广大中小微企业提供数字化转型所需的开发工具及公共性服务。支持数字化转型服务咨询机构和区域数字化服务载体建设，丰富各类园区、特色小镇的数字化服务功能。创新订单融资、供应链金融、信用担保等金融产品和服务。拓展数字化转型多层次人才和专业型技能培训服务。以政府购买服务、专项补助等方式，鼓励平台面向中小微企业和灵活就业者提供免费或优惠服务。

  三、近期工作举措

  （一）服务赋能：推进数字化转型伙伴行动。

  1.发布数字化转型伙伴倡议。

  搭建平台企业（转型服务供给方）与中小微企业（转型服务需求方）对接机制，引导中小微企业提出数字化转型应用需求，鼓励平台企业开发更适合中小微企业需求的数字化转型工具、产品、服务，形成数字化转型的市场能动性。

  2.开展数字化转型促进中心建设。

  支持在产业集群、园区等建立公共型数字化转型促进中心，强化平台、服务商、专家、人才、金融等数字化转型公共服务。支持企业建立开放型数字化转型促进中心，面向产业链上下游企业和行业内中小微企业提供需求撮合、转型咨询、解决方案等服务。

  3.支持创建数字化转型开源社区。

  支持构建数字化转型开源生态，推动基础软件、通用软件、算法开源，加强专业知识经验、数字技术产品、数字化解决方案的整合封装，推动形成公共、开放、中立的开源创新生态，提升传统行业对新技术、工具的获取能力。

  （二）示范赋能：组织数字化转型示范工程。

  1.树立一批数字化转型企业标杆和典型应用场景。

  结合行业领域特征，树立一批具有行业代表性的数字化转型标杆企业，组织平台企业和中小微企业用户联合打造典型应用场景，开展远程办公服务示范，引导电信运营商提供新型基础设施服务，总结提炼转型模式和经验，示范带动全行业数字化转型。

  2.推动产业链协同试点建设。

  支持行业龙头企业、互联网企业建立共享平台，推动企业间订单、产能、渠道等方面共享，促进资源的有效协同。支持具有产业链带动能力的核心企业搭建网络化协同平台，带动上下游企业加快数字化转型，促进产业链向更高层级跃升。

  3.支持产业生态融合发展示范。

  支持行业龙头企业、互联网企业、金融服务企业等跨行业联合，建立转型服务平台体，跨领域技术攻关、产业化合作、融资对接，打造传统产业服务化创新、市场化与专业化结合、线上与线下互动、孵化与创新衔接的新生态。

  （三）业态赋能：开展数字经济新业态培育行动。

  1.组织数字经济新业态发展政策试点。

  以国家数字经济创新发展试验区为载体，在卫生健康领域探索推进互联网医疗医保首诊制和预约分诊制，开展互联网医疗的医保结算、支付标准、药品网售、分级诊疗、远程会诊、多点执业、家庭医生、线上生态圈接诊等改革试点、实践探索和应用推广。在教育领域推进在线教育政策试点，将符合条件的视频授课服务、网络课程、社会化教育培训产品纳入学校课程体系与学分体系、支持学校培育在线辅导等线上线下融合的学习模式。

  2.开展新业态成长计划。

  结合国家数字经济创新发展试验区建设和疫情防控中发挥积极作用的重点保障企业名单，面向数字经济新型场景应用、数据标注等新兴领域，探索建立新业态成长型企业名录制度，实行动态管理，加强了解企业面临的政策堵点和政策诉求，及时推动解决。

  3.实施灵活就业激励计划。

  结合国家双创示范基地、国家数字经济创新发展试验区建设，鼓励数字化生产资料共享，降低灵活就业门槛，激发多样性红利。支持互联网企业、共享经济平台建立各类增值应用开发平台、共享用工平台、灵活就业保障平台。支持企业通过开放共享资源，为中小微企业主、创客提供企业内创业机会。广泛开辟工资外收入机会，鼓励对创造性劳动给予合理分成，促进一次分配公平，进一步激活内需。面向自由设计师、网约车司机、自由行管家、外卖骑手、线上红娘、线上健身教练、自由摄影师、内容创作者等各类灵活就业者，提供职业培训、供需对接等多样化就业服务和社保服务、商业保险等多层次劳动保障。

  （四）创新赋能：突破数字化转型关键核心技术。

  1.组织关键技术揭榜挂帅。

  聚焦数字化转型关键技术和产品支撑，制定揭榜任务、攻坚周期和预期目标，征集并遴选具备较强技术基础、创新能力的单位或企业集中攻关。

  2.征集优秀解决方案。

  发挥市场在资源配置中的重要作用，整合行业专家、投资机构、应用企业等多方力量，从技术、需求、产业发展等角度多方评估，突破一批创新能力突出、应用效果好、市场前景广阔的数字化转型共性解决方案，夯实数字化转型技术基础。

  3.开展数字孪生创新计划。

  鼓励研究机构、产业联盟举办形式多样的创新活动，围绕解决企业数字化转型所面临数字基础设施、通用软件和应用场景等难题，聚焦数字孪生体专业化分工中的难点和痛点，引导各方参与提出数字孪生的解决方案。

  （五）机制赋能：强化数字化转型金融供给。

  1.推行普惠性“上云用数赋智”服务。

  结合国家数字经济创新发展试验区建设，探索建立政府-金融机构-平台-中小微企业联动机制，以专项资金、金融扶持形式鼓励平台为中小微企业提供云计算、大数据、人工智能等技术，以及虚拟数字化生产资料等服务，加强数字化生产资料共享，通过平台一次性固定资产投资、中小微企业多次复用的形式，降低中小微企业运行成本。对于获得国家政策支持的试点平台、服务机构、示范项目等，原则上应面向中小微企业提供至少一年期的减免费服务。对于获得地方政策支持的，应参照提出服务减免措施。

  2.探索“云量贷”服务。

  结合国家数字经济创新发展试验区建设，鼓励试验区联合金融机构，探索根据云服务使用量、智能化设备和数字化改造的投入，认定为可抵押资产和研发投入，对经营稳定、信誉良好的中小微企业提供低息或贴息贷款，鼓励探索税收减免和返还措施。

  3.鼓励发展供应链金融。

  结合数字经济创新发展试验区建设，探索完善产融信息对接工作机制，丰富重点企业和项目的融资信息对接目录，鼓励产业链龙头企业联合金融机构建设产融合作平台，创新面向上下游企业的信用贷款、融资租赁、质押担保、“上云”保险等金融服务，促进产业和金融协调发展、互利共赢。

  各地发展改革、网信部门要高度重视，国家数字经济创新发展试验区要积极行动，大胆探索，结合推进疫情防控和经济社会发展工作，拿出硬招、实招、新招，积极推进传统产业数字化转型，培育以数字经济为代表的新经济发展，及时总结和宣传推广一批好经验好做法。后续，国家发展改革委将进一步商相关部门，统筹组织实施试点示范、专项工程等工作。

1. 工信部：开展2020年中小企业公共服务体系助力复工复产重点服务活动 工信厅企业函〔2020〕72号

各省、自治区、直辖市及计划单列市、新疆生产建设兵团中小企业主管部门，有关单位：

　　为深入贯彻落实党中央、国务院关于统筹推进新冠肺炎疫情防控和经济社会发展工作决策部署，推动中小企业健康发展，现就开展中小企业公共服务体系助力复工复产重点服务活动通知如下：

一、总体要求

全面加强中小企业公共服务体系建设，紧紧围绕中小企业复工复产和高质量发展开展重点服务活动，解难点、除痛点、疏堵点、补盲点，为中小企业恢复生产经营和可持续发展切实提供支撑和保障。

二、重点服务活动

（一）政策宣贯服务。通过开设网上政策服务专栏、编发政策指引等方式，广泛宣传国家和地方出台的系列惠企政策。重点宣讲解读直接关系中小企业权益的财税支持、金融支持、社保减免、劳动用工等政策，汇集发布申报渠道和流程，帮助企业用好用足政策，打通政策落地“最后一公里”。

（二）数字化赋能服务。推动实施《中小企业数字化赋能专项行动》。聚焦线上办公、远程协作等方面，引导数字化服务商提供解决方案、工具包、工业APP等数字化服务产品。强化智能制造服务，帮助企业加快数字化改造，支持中小企业设备上云和业务系统向云端迁移。举办“创新中国行”数字化应用推广等活动。（三）创业创新服务。开展研发成果转化等创业服务，举办技术难题揭榜、诊断咨询等活动，优化创业创新环境。举办大中小企业融通对接、双创示范基地“融通创新”主题日等活动，推广“龙头+孵化”等融通发展模式。组织企业参加“创客中国”中小企业创新创业大赛和全国“双创”活动周，推动项目落地和投融资对接。搭建产业链供需对接平台，开展生产要素供需对接服务，助力产业链固链、补链、强链。

（四）“专精特新”企业培育服务。建立完善“专精特新”中小企业培育库，为入库企业提供技术创新支持、知识产权托管维权、品牌宣传推广等专项服务，促进其成长为专精特新“小巨人”企业、制造业单项冠军企业。开展“专精特新——腾计划”等活动，助力企业借助电子商务升级转型。

（五）融资服务。推动金融惠企政策落实，梳理摸排中小企业融资需求，加强与金融机构联系合作，推动其为中小企业提供信用贷款以及应收账款、订单、仓单和存货质押融资等金融服务。发挥政府性担保、再担保机构融资增信分险作用，助力中小企业复工复产。开展优质中小企业上市培育，促进投融资服务对接，提高中小企业直接融资比重。

（六）市场开拓服务。搭建线上产销对接平台，组织企业开展网上洽谈、在线签约等灵活多样的营销和招商活动。指导企业建立网上直播间、网上会客厅、新媒体营销平台，构建企业与电商平台对接桥梁，助力企业快速拓展销售渠道。支持企业运用招标采购平台和中小企业自采平台，实现网络化招标采购。

（七）其他专业化服务。举办中小企业线上人才招聘等活动，助力补足复工复产用工缺口。开展“企业微课”等线上培训活动，邀请知名专家、企业家在线授课，提升中小企业经营管理水平。加强法律援助和法律咨询公益服务，帮助企业解决受疫情影响造成的合同履行、劳资关系等法律问题。开展志愿服务，建立专家志愿服务团，充分调动社会力量服务中小企业。

三、保障措施

（一）加强组织领导。各地中小企业主管部门要加强对服务体系建设的组织领导，加大资金支持力度，结合本地实际制定具体实施方案，细化工作措施，因地制宜开展特色服务活动，全面助力中小企业复工复产。

（二）提升服务能力。强化中小企业公共服务平台网络、中小企业公共服务示范平台、小型微型企业创业创新示范基地和创新创业特色载体的带动作用，推动服务机构加强能力建设，促进资源共享和服务协同，完善评价机制，提高服务质量。发挥全国中小企业服务联盟作用，通过举办能力竞赛等活动，推动提升服务实效。

（三）创新服务方式。充分运用大数据、云计算、人工智能、5G等新一代信息技术，创新服务方式、拓宽服务渠道，有针对性地推出复工复产服务包、租金减免优惠包等专项服务产品，通过“互联网+”服务等形式，精准满足中小企业需求。

（四）及时总结宣传。各地中小企业主管部门要注重梳理总结经验做法和典型案例，加强分享借鉴和宣传推广，并及时将相关信息发送至cxfwc@miit.gov.cn。请填写重点服务活动实施情况统计表，形成重点服务活动工作总结，于年底前报送部（中小企业局）。

工业和信息化部办公厅

2020年4月9日

1. 国务院联防联控机制：印发新冠病毒无症状感染者管理规范 国办发明电〔2020〕13号

各省、自治区、直辖市人民政府，国务院各部委、各直属机构：

《新冠病毒无症状感染者管理规范》已经中央应对新型冠状病毒感染肺炎疫情工作领导小组同意，现印发给你们，请认真贯彻落实。

国务院应对新型冠状病毒感染肺炎疫情联防联控机制

2020年4月6日

新冠病毒无症状感染者管理规范

第一条　为加强对新冠病毒无症状感染者的发现、报告、管理工作，依据《中华人民共和国传染病防治法》、《中华人民共和国国境卫生检疫法》，制定本规范。

第二条　新冠病毒无症状感染者（以下简称无症状感染者）是指无相关临床表现，如发热、咳嗽、咽痛等可自我感知或可临床识别的症状与体征，但呼吸道等标本新冠病毒病原学检测呈阳性者。无症状感染者有两种情形：一是经14天的隔离医学观察，均无任何可自我感知或可临床识别的症状与体征；二是处于潜伏期的“无症状感染”状态。

第三条　无症状感染者具有传染性，存在着传播风险。

第四条　加强对无症状感染者的监测和发现：一是对新冠肺炎病例的密切接触者医学观察期间的主动检测；二是在聚集性疫情调查中的主动检测；三是在新冠肺炎病例的传染源追踪过程中对暴露人群的主动检测；四是对部分有境内外新冠肺炎病例持续传播地区旅居史人员的主动检测；五是在流行病学调查和机会性筛查中发现的相关人员。

第五条　规范无症状感染者的报告。各级各类医疗卫生机构发现无症状感染者，应当于2小时内进行网络直报。县级疾控机构接到发现无症状感染者报告后，24小时内完成个案调查，并及时进行密切接触者登记，将个案调查表或调查报告及时通过传染病报告信息管理系统进行上报。无症状感染者解除集中医学观察后，医疗卫生机构应当及时在传染病报告信息管理系统中填写解除医学观察日期。

第六条　强化信息公开。国务院卫生健康行政部门每天公布无症状感染者报告、转归和管理情况。各省（区、市）公布本行政区域的情况，本土传播和境外输入情况分别统计报告。

第七条　加强对无症状感染者的管理。无症状感染者应当集中医学观察14天。期间出现新冠肺炎相关临床症状和体征者转为确诊病例。集中医学观察满14天且连续两次标本核酸检测呈阴性者（采样时间至少间隔24小时）可解除集中医学观察，核酸检测仍为阳性且无临床症状者需继续集中医学观察。

第八条　无症状感染者在集中医学观察期间如出现临床表现，应当立即转运至定点医疗机构进行规范治疗，确诊后及时订正。

第九条　对无症状感染者的密切接触者，应当集中医学观察14天。

第十条　组织专家组对集中医学观察的无症状感染者进行巡诊，及时发现可能的确诊病例。

第十一条　对解除集中医学观察的无症状感染者，应当继续进行14天的医学观察、随访。解除集中医学观察后第2周和第4周要到定点医院随访复诊，及时了解其健康状况。

第十二条　有针对性加大筛查力度，将检测范围扩大至已发现病例和无症状感染者的密切接触者。做好对重点地区、重点人群、重点场所的强化监测，一旦发现无症状感染者应当集中隔离医学观察。

第十三条　无症状感染者具有传播隐匿性、症状主观性、发现局限性等特点，国家支持开展无症状感染者传染性、传播力、流行病学等科学研究。

第十四条　加强与世界卫生组织等有关国家和国际组织的信息沟通、交流合作，适时调整诊疗方案和防控方案。

第十五条　各地要加大新冠病毒知识科普宣传力度，指导公众科学防护，广泛开展培训，提高基层医疗卫生人员和社区工作人员等的防控能力和水平。

1. 国务院联防联控机制：进一步做好重点场所重点单位重点人群新冠肺炎疫情防控相关工作 国办发明电〔2020〕16号

各省、自治区、直辖市人民政府，国务院各部委、各直属机构：

当前，新冠肺炎疫情防控取得阶段性重要成效，经济社会秩序加快恢复。同时，境外疫情呈加速扩散蔓延态势，我国疫情输入压力持续加大，要在疫情防控常态化条件下加快恢复生产生活秩序，坚持实施“外防输入、内防反弹”的防控策略，进一步完善应急和常态化防控结合的措施与机制，在做好境内疫情精准防控的同时，积极有序推动复工复产。结合当前疫情防控形势，为有效防止聚集性疫情的发生，落实分区分级防控要求，推进生产生活秩序逐步恢复，进一步做好重点场所、重点单位、重点人群的疫情防控工作，将疫情风险降到最低，经中央应对新型冠状病毒感染肺炎疫情工作领导小组同意，现将有关事项通知如下：

一、指导原则

各地要根据疫情形势的发展，科学确定复工复产复学时间，严格落实属地责任、部门责任、单位责任和个人家庭责任，从严控制、审核、组织举办各类涉及人群聚集性的活动，有序推动恢复正常生产生活秩序。各行业在复工复产前要根据行业特点制定具体防控方案。各单位要落实主体责任，细化各项防控措施，确保每一个细节、每一个关键步骤落实到位。

（一）精准实施分区分级差异化的办公场所和公共场所防控措施。根据动态调整的应急响应级别，因地制宜实施办公场所和公共场所差异化防控。低风险地区坚持“逐步、适当放开”原则，凡是涉及人群聚集性的活动，应当在科学研判疫情形势的基础上审慎开放，减少集中聚集风险。对于工作生活必须的场所、开放式活动场所，有序逐步放开；对于娱乐、休闲等集中密闭场所，审慎开放。中、高风险地区坚持“安全、稳步”原则，原则上不组织涉及人群聚集性的活动。对于工作生活必须的场所、开放式活动场所，采取分类适度限制措施；对于娱乐、休闲等集中密闭场所，建议采取临时禁止开业措施，防范聚集性疫情风险，具体要求由各地依据本地疫情形势研究确定。

（二）强化特殊单位防控和人员防护措施。低风险地区加强养老机构、儿童福利院、监狱、精神卫生医疗机构等特殊单位风险防范，做好人员防护、消毒等日常防控工作。中、高风险地区继续采取强化措施，严格落实特殊单位的防控措施监管，制定应急预案，提高应急处置能力。

（三）加强重点场所和重点人群的防护指导。筑牢织密“外防输入、内防反弹”防线，盯紧航空运输、口岸检疫等各个环节，严格实施闭环管理措施，实现集中接送、检测、隔离等全流程高效无缝运转。要加强应急处置准备，严密落实“早发现、早报告、早隔离、早治疗”措施，最大限度减少传播风险。重点指导老年人、儿童、孕产妇、学生、医务人员等重点人群做好个人防护，教育引导群众养成良好的卫生习惯和生活方式，减少和避免人员聚集，严防聚集性疫情发生。

二、防控建议

（一）生活服务类场所。建议低风险地区在做好室内通风、环境清洁消毒、人员健康监测的前提下正常营业；在中、高风险地区应当限制人员数量，减少人群聚集。

（二）开放式活动场所。建议低风险地区逐步恢复正常营业；中、高风险地区在做好环境清洁消毒、人员健康监测的前提下正常营业，并采取措施限制人员数量，减少人群聚集。大型聚集性体育活动如马拉松长跑、聚集性宗教活动、各类展览及会展等暂不开展。

（三）密闭式娱乐、休闲场所。建议低、中、高风险地区均暂不开业，具体要求由各地依据本地疫情形势研究确定。

（四）客运场站和公共交通工具。如飞机、旅客列车、候车室等，要严格做好通风、环境清洁消毒、人员健康监测等日常监管，可通过采取控制乘客数量、分散就坐等措施，减少人员聚集。严格境外回国航空运输、口岸检疫、目的地专车接送等防控措施及监管。

（五）特殊单位场所。对于养老机构、儿童福利院、监狱、精神卫生医疗机构等特殊单位，低风险地区要做好风险防范，加强人员防护、消毒等日常防控工作；中、高风险地区要制定应急预案，严格落实防控措施监管，有条件的组织开展全面排查和核酸筛查。

（六）企事业单位。低风险地区做好室内通风、环境清洁消毒、人员健康监测等日常卫生管理；建议中、高风险地区鼓励采取错时上下班、弹性工作制或居家办公方式，减少人员聚集。

三、加强领导，强化责任落实

地方各级政府和有关部门要进一步提高政治站位，增强大局意识和底线思维，守土有责、守土尽责，统筹抓好疫情防控和经济社会发展重点工作，确保人员到位、信息畅通，严格执行“四早”措施和报告制度。落实属地责任，地方各级政府主要负责人对属地防控工作负总责。落实部门和行业责任，强化行业、系统管理，制定好复工复产防控措施并落实到位。各有关部门要按照各自职责，切实做好本行业、本系统的防控工作。落实单位主体责任，各单位要建立健全防控工作责任制和管理制度，配备必要的防护物品、设施，开展宣传教育。

上述要求将视疫情变化进行调整。有关重点场所、重点单位、重点人群防控技术指南由国务院联防联控机制综合组另行发布。

国务院应对新型冠状病毒感染肺炎疫情联防联控机制

2020年4月6日

1. 国务院联防联控机制：印发新冠肺炎出院患者复诊复检工作方案（试行） 国办发明电〔2020〕15号

各省、自治区、直辖市人民政府，国务院各部委、各直属机构：

《新冠肺炎出院患者复诊复检工作方案（试行）》已经中央应对新型冠状病毒感染肺炎疫情工作领导小组同意，现印发给你们，请认真贯彻落实。

国务院应对新型冠状病毒感染肺炎疫情联防联控机制

2020年4月6日

新冠肺炎出院患者复诊复检工作方案

（试行）

为进一步做好新冠肺炎患者治愈出院后的隔离观察、复诊复检、健康监测和康复管理等相关工作，实现全流程管理，促进患者全面康复，特制定本方案。

一、职责分工

（一）各级卫生健康部门要加强对新冠肺炎患者出院后隔离观察和管理的统筹协调，指导定点医院、隔离场所、康复医疗机构、基层医疗机构密切配合，加强信息沟通，协同做好新冠肺炎患者出院后的隔离观察、复诊复检、健康监测和康复管理等工作。

（二）定点医院要做好出院患者随访、定期复诊复检及健康指导工作。基层医疗机构要做好患者出院后居家隔离健康监测和健康管理等工作。集中隔离点要做好患者健康监测和生活照护工作。

二、隔离管理

（三）新冠肺炎患者治愈出院后，应当继续隔离医学观察14天。隔离期间每日做好体温、体征等身体状况监测，观察有无发热，以及咳嗽、气喘等呼吸道症状。患者出院后可采取居家隔离或隔离点集中隔离。各级卫生健康部门要指导相关医疗机构做好出院患者及家属隔离管理和健康监测。定点医院要及时将出院患者信息推送至患者辖区或居住地居（村）委会和基层医疗机构。设有集中隔离点的地区，卫生健康部门要指导定点医院与集中隔离点做好衔接，做好患者的隔离观察、基本康复、心理干预、健康监测等工作。

（四）居家隔离的出院患者应当换上新带衣物，与家属佩戴口罩返回，避免使用公共交通工具，回家后彻底清洁消毒住院时衣物。隔离期间尽可能居住在通风良好的单人房间，要在基层医疗机构指导下进行自我健康状况监测，佩戴口罩，隔离居室要经常开窗保持空气流通，同时做好保暖。要尽可能减少照护人员数量，做到分餐饮食，避免与家人的密切接触，做好手卫生和日常清洁，避免外出活动。隔离期间的日常生活用品应当单独清洗消毒。

（五）集中隔离点房间应当具有良好的独立通风条件，具有独立的卫生间，隔离对象原则上不得离开房间活动。应当配备适当的急诊急救物资、医护人员与其他工作人员，保证出院患者的生活和安全。

三、复诊复检管理

（六）定点医院要严格执行新版新型冠状病毒肺炎诊疗方案出院标准和出院后注意事项。患者出院前要组织专家结合其临床症状与体征、实验室与影像学检查结果等综合评估，明确后续跟踪随访事项。要建立专门的随访登记制度、手册，与出院患者签订出院告知书，详细告知出院后注意事项。

（七）定点医院要为出院患者安排好2—4周的复诊复检计划，重点复查血常规、生化、氧饱和度，复查新型冠状病毒病原学检测，优先选择可靠性较高的痰标本。有肺炎的患者，进行胸部CT影像学检查。

（八）对重型、危重型出院患者进行呼吸功能检查。根据出院患者肺部炎症吸收情况、肺纤维化和肺功能损害、肢体功能、心理功能情况进行康复指导，按照《新冠肺炎出院患者康复方案（试行）》要求，开展康复评估，根据评估结果有针对性地制订出院患者康复医疗计划并予以康复训练和心理干预。

（九）出院患者要按照复诊计划在定点医院进行复诊。各有关医疗机构和集中隔离点要密切关注出院患者健康状况，对老年人和有基础疾病的出院患者要特别加强健康状况监测。对在省级、设区的市级定点医院出院患者，原则上在属地定点医院复诊。

四、核酸复检阳性人员管理

（十）出院患者核酸复检呈阳性，并出现发热、咳嗽等临床表现，CT影像学显示肺部病变加重，属确诊病例，应当尽快将其转至定点医院进一步治疗。核酸检测呈阳性但无临床表现和影像学进展的，属确诊的康复期患者，应当继续隔离观察，按照本方案做好个人防护等相关工作。各级卫生健康部门应当做好科普宣传，定期向社会公布相关信息，营造积极的社会氛围，消除公众对患者歧视。

1. 民政部：及时调整完善疫情防控策略 有序恢复养老机构服务秩序 民电〔2020〕52 号

各省、自治区、直辖市民政厅（局），各计划单列市民政局，新疆生产建设兵团民政局：

为深入学习贯彻习近平总书记关于统筹推进新冠肺炎疫情防控和经济社会发展系列重要讲话精神，适应疫情防控新形势，及时调整完善疫情防控策略，在疫情防控常态化条件下有序恢复养老服务秩序，现提出以下意见：

一、及时调整完善疫情防控策略，有序恢复养老服务秩序。各省级民政部门要结合区域实际，根据“外防输入、内防反弹”总体防控策略及区域联防联控部署，将应急措施和常态化防控相结合，在严格做好疫情防控的前提下，及时制定有针对性的政策举措，有力有序推动恢复养老机构服务秩序，积极破解难点堵点，加强特殊困难老年人关爱服务。要明确拟恢复对外服务的养老机构防控条件，在保证入住老年人生命安全和健康前提下，优先安排老年人返院、刚需老年人入住和工作人员返岗复工。低风险地区要恢复养老服务秩序，保障养老服务工作正常开展，确保养老服务机构的正常运营。加大对无症状感染者管理工作力度，做好对返院、新入住老年人以及复工人员核酸检测，对发现的无症状感染者，要及时采取集中隔离措施，配合卫生疾控部门开展流行病学调查。

二、因地制宜调整养老机构人员进出管理措施。要坚守底线，继续严防外部感染源输入。老年人或工作人员有下列情形之一的，禁止进入养老机构：（1）15天内曾接触入境回国人员；（2）与已确诊或疑似病例有密切接触；（3）有发热、咳嗽、流涕、腹泻等疑似症状。（4）属于无症状感染者或无症状感染者的密切接触者。确需到养老机构探视到访的家属，应按照“限定时间（预约等方式）、限定人数、限定路线、限定区域（不进入生活区）”等要求进行。

低风险地区养老机构接收本区域内老年人和工作人员的，可不再要求14天医学隔离观察，但要采取“先预约，再入住”、加强健康和旅行信息排查等管理措施。中高风险地区符合条件的养老机构接收本区域内老年人和工作人员的，应经14天医学隔离观察和相关医学检查正常；点对点接收低风险地区老年人和工作人员的，可不要求14天医学隔离观察。

三、继续执行养老机构内部防控要求。出入管理防控措施由各省份根据实际情况作出调整外，养老机构内部防控仍按《养老机构新型冠状病毒感染的肺炎疫情防控指南（第二版）》要求严格落实。要积极做好在院老年人和工作人员精神慰藉，鼓励开展适宜的户外活动，继续提倡家属同老年人之间利用视频、电话等方式进行心理关爱。

四、严格落实部门监管责任和养老机构主体责任。各地民政部门要充分认识到疫情防控工作的长期性和复杂性，在有序恢复养老服务秩序的同时，仍要全面压实养老机构疫情防控责任，坚决防止因老年人返院入住、工作人员返岗入职带来新的传播风险和隐患。要总结前期经验，完善疫情应急响应和处置预案，一旦出现疫情立即启动应急处置程序，确保防控措施落实到位。要按照“谁用工、谁管理、谁负责”原则，加强对新进养老机构工作人员的管理，让工作人员知晓最新防控规定和要求。遇有突发情况和重大事件及时向当地党委和政府及上级民政部门报告。

民政部办公厅

2020年4月3日

1. 卫健委：印发新冠肺炎患者、隔离人员及家属心理疏导和社会工作服务方案 联防联控机制发〔2020〕39号

各省、自治区、直辖市及新疆生产建设兵团应对新型冠状病毒肺炎疫情联防联控机制（领导小组、指挥部）：

为做好新冠肺炎患者、隔离人员及家属心理疏导和社会工作服务，促进患者身体与心理同步康复，维护隔离人员、家属心理健康，营造相互关怀的社会环境，国家卫生健康委、民政部制定了《新冠肺炎患者、隔离人员及家属心理疏导和社会工作服务方案》。现印发给你们，请认真贯彻落实。

国务院应对新型冠状病毒肺炎疫情联防联控机制

（代章）

2020年4月7日

（信息公开形式：主动公开）

新冠肺炎患者、隔离人员及家属心理疏导和社会工作服务方案

为做好新冠肺炎患者、隔离人员及家属心理疏导和社会工作服务，促进患者身体与心理同步康复，回归正常生活和工作，维护隔离人员、家属心理健康，营造相互关怀的社会环境，特制定本方案。

一、工作目标

（一）开展新冠肺炎防控宣传和健康教育，提升群众防控知识水平，增强防护技能，减轻因认知不足所致的恐惧，减少、消除对患者、隔离人员及家属的歧视与排斥等行为。

（二）以互联网络平台为基础，推进互联网+社会心理服务，为患者、隔离人员及家属提供线上心理支持和服务，增强自我心理调适能力，帮助有需求的服务对象渡过困难期，恢复社会功能。

（三）以社区为主要阵地，建立心理疏导和社会工作服务网络，提供情绪引导、心理辅导、资源链接、困难纾解、社会支持网络修复等服务，改善社区环境，恢复社区秩序，推动基层社区治理，营造健康向上的社区氛围，促进社会稳定。

二、组织实施

（一）加强领导组建队伍。受疫情影响较大的地方，各区县防疫指挥部要成立新冠肺炎患者、隔离人员及家属心理疏导和社会工作服务领导小组，卫生健康、民政等部门分工负责，各街道（乡镇）明确1名领导负责辖区心理疏导和社会工作，指定专人统筹协调心理疏导和社会工作服务。各街道（乡镇）根据受疫情影响情况，在社区设置心理咨询室或社会工作室，配备1-2名专职或兼职心理咨询师或社会工作者；在社区卫生服务中心（乡镇卫生院）设置心理专干；在街道(乡镇)设置社会工作站，配备1-2名专职或兼职社会工作者，负责辖区群众社会心理健康工作。有条件的，可建立由社区工作人员、社会工作者、志愿者、心理咨询师、心理治疗师、精神科医师等组成的社区心理疏导和社会工作服务队，采取社区、社会工作者、社区志愿者、社区社会组织、社区公益慈善资源、心理服务专业力量联动服务方式开展工作。

（二）建立对口指导机制。各地按照已有精神卫生服务技术指导机制，由各社区卫生服务中心（乡镇卫生院）对口联系的上级精神卫生医疗机构派精神科医师、心理治疗师，提供技术指导和帮助。

（三）提供线下和转介服务。根据出院患者及家属、隔离人员家属需求，社区工作人员、心理专干可联系社会工作者、心理咨询师、心理治疗师进社区提供心理疏导和社会工作服务。发现出院患者及家属、隔离人员家属可能有精神卫生问题时，心理咨询师、精神卫生社会工作者等应当向对口精神卫生医疗机构转介，由精神科医生提供精神医学诊疗服务。

（四）开展人员培训和督导。上级精神卫生医疗机构和社会工作行业组织应当派具备心理援助技术能力的精神科医师、心理治疗师、社会工作师对社区工作人员、志愿者、心理专干等人员开展精神卫生知识培训，分别对街道和社区工作提供技术指导和专业督导。

三、工作原则

（一）统一领导、团队协作、分工负责。所有工作须在本专项工作领导小组的统一领导下组织进行，各类人员实行团队协作，明确成员分工与配合。未经允许个人不得擅自开展任何形式的心理疏导和社会工作服务活动。

（二）严守心理卫生和社会工作服务伦理原则、保密要求。对患者、隔离人员及家属的个人和家庭隐私包括个人信息、生活轨迹、家庭情况等严格保密；设专人负责服务对象档案管理并签署保密协定。

（三）分类分级。结合各社区实际状况，采用分类、分级处理。对不同社区、不同年龄段、不同人群、具有不同需求的服务对象进行分类干预。

四、具体工作措施

（一）广泛开展科普宣教。针对公众关心的热点话题，通过权威媒体开展新冠肺炎防控宣传，提升公众知识水平，增强防控能力。发放心理健康服务宣传材料，提供心理健康专业知识、心理热线电话号码、心理支持网络平台二维码等支持性资源，帮助患者、隔离人员及家属自我心理调适。组织专家通过媒体、视频、网络授课等形式，对社区居民开展心理健康讲座，介绍温馨家庭氛围、团结社区关系对个体渡过难关的帮助和意义，鼓励大众互帮互助，消除歧视。

（二）在医院和隔离点开展心理评估和疏导。患者、隔离人员在定点医院/康复驿站/隔离点期间，定点医院/康复驿站/隔离点主动向其推荐心理自评工具（如，湖北省精神卫生中心牵头研发的“强肺心理支持系统”），鼓励有需要者寻求心理疏导及心理支持。有条件的定点医院/康复驿站/隔离点对患者、隔离人员开展集体心理疏导。对评估后发现心理问题风险较高的，协调心理咨询师、心理治疗师、社会工作者等专业人员提供心理干预。

（三）保证心理疏导与治疗连续性。对在定点医院已经使用精神科药物干预的患者，如评估需要继续给予精神科药物治疗的，定点医院应当将患者相关治疗信息记入出院信息单中，交康复驿站或隔离点继续维持治疗。对评估需要继续治疗的，康复驿站或隔离点在其离开时，应当将相关治疗信息交社区卫生服务中心（乡镇卫生院）继续予以治疗。

（四）为有需求的居家人员提供社会和心理支持。社区应当鼓励出院患者及家属、隔离人员家属主动寻求社会和心理支持，由专业人员进行心理与社会工作服务需求评估。对评估后发现心理问题风险较高的，在征得本人同意后，由社区卫生服务中心（乡镇卫生院）、社会工作服务机构协调心理咨询师、心理治疗师、社会工作者等提供心理和社会工作服务，建立和激活服务对象的社会支持系统，引导其恢复生活信心。

（五）开展困难人员生活扶助与支持。对有具体生活困难的患者、隔离人员及家属，社区工作人员、社会工作者、心理专干等，应当主动向街道(乡镇)和社区汇报，协助申请困难救助，链接援助资源，同时寻求社会组织和社会公益慈善资源的帮助或支持。

（六）加强病亡者家属关爱和心理支持。对病亡者家属，心理专干、社会工作者要主动为其提供哀伤辅导、心理疏导、社会支持等服务，加强关心关爱，引导其宣泄哀伤情绪，帮助顺利渡过哀伤期，恢复正常生活。必要时，联系心理和精神卫生专业人员提供专业支持。

（七）及时发现处置高危风险。对发现有自伤、自杀、冲动伤人风险的患者、隔离人员及家属，相关专业人员要在第一时间向街道（乡镇）负责领导报告并通报社区。社区工作人员、社会工作者、心理专干等人员要增加走访密度，告知其家人紧急联系电话。由心理咨询师、精神卫生社会工作者等介入进行危机干预，持续提供心理疏导和社会工作服务，必要时向精神卫生医疗机构转诊。

附件：患者、隔离人员及家属心理疏导和社会工作服务要点

附件

患者、隔离人员及家属心理疏导和社会工作服务要点

一、及时向新冠肺炎患者、隔离人员及家属澄清和解释最新的新冠肺炎相关知识和防护策略，提高他们对疾病的认知程度。

二、建议新冠肺炎出院患者、隔离人员坚持保持作息规律、营养均衡、劳逸结合、睡眠充足、适度锻炼，以提高机体对疾病的免疫力。

三、引导新冠肺炎出院患者、隔离人员及家属建立理性的生活方式，排除有可能存在的歧视及外在干扰带来的心理影响，调适焦虑、无助、恐惧等心理，将注意力转移到自己的生活和工作中来，并为患者、隔离人员及家属营造关爱、包容、尊重和接纳的社会环境。

四、在心理疏导和社会工作服务过程中，注重倾听、共情、正常化、鼓励、积极建议等基本咨询技巧的使用。多采取稳定情绪的心理干预策略。多采取整合视角的社会工作干预策略，综合运用社会工作专业方法为患者、隔离人员及其家庭提供全方位社会支持体系。

五、对于患者所提出的实际问题，例如对复工的担忧，工作人员应当鼓励其认真学习和理解政府制定的相关政策，积极寻求家人、同事、朋友或社会工作者、社区工作人员的帮助，理性解决实际问题。

六、及时追踪并充分获悉来自多渠道的病亡者家属的心理状况信息，尊重病亡者家属的求助意愿，在准确心理评估的前提下，开展有针对性的心理疏导和危机干预。

1. 国家税务总局、银保监会：发挥“银税互动”作用助力小微企业复工复产

国家税务总局各省、自治区、直辖市和计划单列市税务局，国家税务总局驻各地特派员办事处，各银保监局，各大型银行、股份制银行：

为深入贯彻落实党中央、国务院关于统筹抓好新冠肺炎疫情防控和经济社会发展的决策部署，进一步纾解小微企业困难，现就进一步发挥“银税互动”作用，助力小微企业复工复产有关事项通知如下：

一、实施重点帮扶

各省税务机关加强与银保监部门和银行业金融机构的协作，及时梳理受疫情影响较大的批发零售、住宿餐饮、物流运输、文化旅游等行业的小微企业名单，按照国家社会信用体系建设要求依法推送相关企业名称、注册地址、经营地址、联系方式、法定代表人、纳税信用评价结果信息；在依法合规、企业授权的前提下，可向银行业金融机构提供企业纳税信息。各地税务、银保监部门充分利用“银税互动”联席会议机制和“百行进万企”等平台，帮助银行业金融机构主动对接企业需求、精准提供金融服务。

二、创新信贷产品

根据小微企业贷款需求急、金额小、周转快的特点，银行业金融机构要创新“银税互动”信贷产品，及时推出适合小微企业特点的信用信贷产品。进一步优化信贷审批流程，提高贷款需求响应速度，适当增加信用贷款额度，延长贷款期限，加大对此前在银行业金融机构无贷款记录的“首贷户”的信贷投放力度。认真落实《关于对中小微企业贷款实施临时性延期还本付息的通知》（银保监发〔2020〕6号）要求，帮助小微企业缓解资金困难尽快复工复产。

三、落实扩围要求

税务、银保监部门和银行业金融机构紧密合作，认真落实《国家税务总局 中国银行保险监督管理委员会关于深化和规范“银税互动”工作的通知》（税总发〔2019〕113号）关于扩大“银税互动”受惠企业范围至纳税信用M级的要求，对湖北等受疫情影响严重的地区，银行业金融机构结合自身风险防控要求，可逐步将申请“银税互动”贷款的企业范围扩大至纳税信用C级企业;纳入各省税务机关纳税信用评价试点的个体工商户可参照实行。

四、提高服务质效

税务部门和合作的银行业金融机构要积极发挥网上渠道优势，提供安全便捷的“非接触式”服务，确保疫情防控期间“银税互动”平台运行、信息推送、申请受理业务不中断，并在2020年9月底前实现“银税互动”数据直连工作模式。

国家税务总局办公厅

中国银行保险监督管理委员会办公厅

2020年4月7日

1. 民航局、海关总署：中国籍旅客乘坐航班回国前填报防疫健康信息

为减少疫情跨境传播，从附件所列国家已购买回国机票的中国籍旅客需要提前填报防疫健康信息。具体要求如下：

一、自2020年4月8日起，已购票旅客在登机前需要提前通过防疫健康码国际版微信小程序，逐日填报个人资料、健康状况、近期出行情况等信息。特殊情况可由他人代为填报。

二、2020年4月8日至4月22日为过渡期。过渡期内乘坐航班的中国籍旅客，应于4月8日起连续逐日填报。过渡期后乘坐航班的，应于登机前第14天起连续逐日填报。

三、未按上述要求填报的，将无法登机。旅客填报虚假信息，将导致行程受阻，并须承担相应法律责任。

特此公告。

中国民用航空局中华人民共和国海关总署

2020年4月7日

1. 财政部：充分发挥政府性融资担保作用 为小微企业和“三农”主体融资增信 财金〔2020〕19号

各省、自治区、直辖市、计划单列市财政厅（局），新疆生产建设兵团财政局，国家融资担保基金，各级政府性融资担保、再担保机构：

　　为充分发挥政府性融资担保作用，更加积极支持小微企业和“三农”主体融资增信，帮助企业复工复产、渡过难关，现将有关事项通知如下：

　　一、当前形势下政府控股的融资担保、再担保机构要积极为小微企业和“三农”主体融资增信，努力扩大业务规模，提升服务效率，及时履行代偿责任，依法核销代偿损失，协调金融机构尽快放贷，不抽贷、不压贷、不断贷，着力缓解小微企业融资难、融资贵。

　　二、各级政府性融资担保、再担保机构要严格落实《国务院办公厅关于有效发挥政府性融资担保基金作用切实支持小微企业和“三农”发展的指导意见》（国办发〔2019〕6号）要求，不得偏离主业盲目扩大经营范围，不得向非融资担保机构进行股权投资，不得新开展政府融资平台融资担保业务。

　　三、坚持下沉一线、更好发挥放大效应的原则，推动国家融资担保基金加快开展股权投资，力争2020年投资10家支小支农成效明显的地市级政府性融资担保机构。推动国家融资担保基金与银行业金融机构开展批量担保贷款合作，力争实现2020年新增再担保业务规模4000亿元目标。国家融资担保基金对合作机构单户100万元及以下担保业务免收再担保费，2020年全年对单户100万元以上担保业务减半收取再担保费。

　　四、地方各级政府性融资担保、再担保机构2020年全年对小微企业减半收取融资担保、再担保费，力争将小微企业综合融资担保费率降至1%以下。进一步提高支小支农业务占比，确保2020年新增小微企业和“三农”融资担保金额和户数占比不低于80%，其中新增单户500万元以下小微企业和“三农”融资担保金额占比不低于50%。

　　五、2020年中央财政继续实施小微企业融资担保降费奖补政策，依据《财政部 工信部关于对小微企业融资担保业务实施降费奖补政策的通知》（财建〔2018〕547号）要求，对扩大小微企业融资担保业务规模、降低小微企业融资担保费率等成效明显的地方予以奖补激励。各地要加强相关政策衔接，加大代偿补偿力度，切实保障降费效果。符合条件的担保、再担保机构可按规定享受担保赔偿准备和未到期责任准备在企业所得税税前扣除等税收优惠政策。

　　六、地方各级财政部门要会同有关方面调整对政府性融资担保、再担保机构的盈利考核要求，重点考核在实现保本微利的前提下支农支小成效（包括当年新增支小支农担保户数、当年新增支小支农担保金额、当年新增500万元以下支小支农担保金额占比、当年平均综合融资费率等），落实尽职免责要求，建立绩效评价结果与资本金补充、风险补偿、薪酬待遇等直接挂钩的激励约束机制。对于支小支农成效明显但代偿压力较大的机构，地方各级财政部门要根据绩效考核结果及时给予适当支持，推动实现可持续经营。

　　七、对严重偏离支小支农主业、擅自扩大业务范围、违规开展股权投资和政府融资平台融资担保业务的地方各级政府性融资担保、再担保机构，要予以公开通报，不得享受各级财税支持政策，不得纳入国家融资担保基金合作范围。

　　国家融资担保基金、地方各级财政部门和政府性融资担保、再担保机构要切实增强“四个意识”，坚定“四个自信”，做到“两个维护”，强化责任担当，认真抓好组织实施，尽快完善配套措施，推动政策落实落细落地。

　　财 政 部

　　2020年3月27日

1. 民政部、教育部：统筹推进儿童福利领域疫情防控与复工复产复学相关工作 民办发〔2020〕14号

各省、自治区、直辖市民政厅（局）、教育厅（教委），各计划单列市民政局、教育局，新疆生产建设兵团民政局、教育局：

为深入贯彻习近平总书记关于统筹推进新冠肺炎疫情防控和经济社会发展系列重要指示精神，全面落实国务院应对新冠肺炎疫情联防联控机制有关工作部署，现就儿童福利领域疫情防控与复工复产复学相关工作要求通知如下：

一、中、高风险地区机构继续严格执行封闭管理要求。中、高风险地区的儿童福利机构、未成年人救助保护机构要继续严格管理。对于回机构、返岗的工作人员隔离观察至少14天，对于采取居家隔离的，要求居家休息不外出，不接触其他人员，每日测量体温并报告情况，身体状况无异常的方可安排上岗。对于新接收的儿童和新招录的工作人员进行核酸检测并隔离观察14天无异常后，经医疗卫生部门同意再进入儿童生活区域，确有需要的还应进行CT检查。对于养育儿童的综合性社会福利机构要确保儿童生活区域保持独立，除服务儿童的工作人员外，其他人员均不得进入，服务儿童的工作人员应为专职人员，不得交叉服务其他民政服务对象。

二、低风险地区机构实施差异化精准防控。低风险地区机构按照当地联防联控指挥机构统一部署和防控要求实施差异化精准防控，对于因就学而确需回机构居住的儿童，应当提前安排进入机构隔离区隔离观察至少14天，无异常后再进入机构独立的生活区域。所在地区中小学已全部开学的低风险地区机构，可以在确保疫情防控到位的前提下，对于15天内未到过中高风险地区、未密切接触来自中高风险地区人员且无相关疑似症状的轮岗工作人员和因就学而确需回机构居住的儿童，按照当地联防联控指挥机构要求，不再进行14天的隔离观察，但工作人员轮休期间应当居家休息不外出。所在地区所有大中小学、幼儿园等已全面开学开园的低风险地区机构，可以按照当地联防联控指挥机构要求，自行决定取消封闭管理的时间。在全国疫情全面解除前，低风险地区机构对于新接收的儿童和新招录工作人员仍应继续执行隔离观察等相关规定。

三、进一步提升家庭寄养儿童防控工作水平。要建立每日沟通报告制度，通过电话、视频等方式与寄养家庭保持密切联系，详细了解寄养儿童及共同生活成员的身体健康状况，宣传普及疫情防控知识，督促指导寄养家庭继续实施严格的防控措施。寄养家庭成员应当尽量减少外出，不与非共同生活的其他亲属、朋友等近距离来往。要建立台账，精准详细掌握寄养家庭共同生活成员外出复工复产情况。为保证寄养儿童的安全，按照当地联防联控指挥机构统一部署，中、高风险地区在人员密集场所工作的寄养家庭共同生活成员，尽量采取网上办公方式或者延期复工；低风险地区复工复产的家庭共同生活成员要确保防控到位，尽量保持企业到家庭上下班两点一线模式。推动具备条件的地区为寄养家庭配备防疫物资。

四、加强对其他儿童群体的救助保护工作。各地要认真贯彻落实国务院应对新冠肺炎疫情联防联控机制印发的《因新冠肺炎疫情影响造成监护缺失儿童救助保护工作方案》，及时发现、报告和分类处置因新冠肺炎疫情影响造成监护缺失儿童，确保这类儿童群体监护人在住院、隔离期间基本生活和照料照护得到基本保障。要指导各地乡镇（街道）儿童督导员、村（居）儿童主任加强对农村留守儿童和困境儿童的关爱力度，重点关注复工复产后农村留守儿童的关爱保护需求，督促监护人或受委托照护人依法履行监护责任，着重做好复学前农村留守儿童关爱保护工作；要在做好疫情期间特殊儿童群体心理疏导工作基础上，继续发挥儿童临时救助或心理援助热线作用，做好农村留守儿童、困境儿童救助保护衔接、心理抚慰等工作，推进未成年人保护工作常态化、制度化。

五、统筹做好开学复课各项工作。各地民政部门和教育部门要保持信息畅通，密切关注开学信息，加强与学校的沟通协作，做好开学前各项准备工作。对于所在地区已发布开学计划的低风险地区的儿童福利机构，要提前为就学儿童划分独立的生活区域，确保就学儿童不与机构内其他儿童接触，并为其配备专职人员提供服务。就学儿童一般应居住在相对独立、通风良好、有独立厕所的单人房间。要为就学儿童进出机构提前规划路线，避免与其他人员交叉接触。要落实好就学儿童上下学途中的各项防护措施，原则上采取机构到学校点对点接送的方式，儿童和工作人员在途中要全程佩戴口罩，提供校车的还应做好校车的安全检查和日常消毒工作。就学儿童返回机构时，要一律进行体温测量，用消毒洗手液洗手、消毒，更换口罩后，才能进入生活区域。密切关注就学儿童的健康状况，发现异常状况的，要及时启动应急预案，做好处置工作。各地要加强跟进指导，切实做好寄养儿童、散居孤儿、农村留守儿童等群体上下学途中的个人防护和开学复课相关工作。

六、扎实推进在线教育等工作。各地民政部门和教育部门要积极解决就学儿童线上学习条件问题，为就学儿童在线学习提供必要的设施设备和指导服务，确保就学儿童“停课不停学”。要统筹用好本地资源和国家平台，充分利用国家中小学网络云平台和中国教育电视台4频道空中课堂等免费学习资源，保障就学儿童的学习需要。要注重加强爱国主义教育、防疫知识教育、生命教育、心理健康教育、家庭教育等。要关心就学儿童的身心健康，培养就学儿童良好的个人卫生习惯，加强体育锻炼，正常作息，规律生活。

七、加大监督检查力度。各地要狠抓工作落实，创新工作方法，利用电话、微信、视频连线等信息化手段，加大对儿童福利领域有关工作的监督检查，确保各项措施落实到位。要采取“四不两直”方式督导检查疫情防控工作，筑牢儿童福利领域安全防线。

民政部办公厅 教育部办公厅

2020年3月24日

1. 农业农村部、人社部：扩大返乡留乡农民工就地就近就业规模 农办产〔2020〕2号

各省、自治区、直辖市及计划单列市农业农村（农牧）厅（局、委）、人力资源社会保障厅（局），新疆生产建设兵团农业农村局、人力资源社会保障局：

　　为贯彻落实中央统筹推进新冠肺炎疫情防控和经济社会发展工作部署，按照国务院关于应对新冠肺炎疫情影响强化稳就业举措的要求，农业农村部、人力资源社会保障部制定了《扩大返乡留乡农民工就地就近就业规模实施方案》，现印发给你们，请认真贯彻执行。

　　各级农业农村部门和人力资源社会保障部门要着眼大局，主动作为，结合本地实际，细化实施方案，实化政策措施，强化责任落实，有力促进农民工就地就近就业。要密切配合、同向发力，明确任务分工，加强指导服务，搞好监测调度，及时解决返乡留乡农民工就业中的实际困难，努力实现农民工就地就近就业目标。

　　 农业农村部办公厅 人力资源和社会保障部办公厅

　　2020年3月26日

扩大返乡留乡农民工就地就近就业规模实施方案

　　今年是全面建成小康社会目标实现之年，是全面打赢脱贫攻坚战收官之年，促进农民就业增收任务艰巨。突发的新冠肺炎疫情，对农民工外出务工造成较大冲击，给返乡留乡农民工就业增收带来较大困难。为贯彻落实中央统筹推进新冠肺炎疫情防控和经济社会发展工作部署，按照国务院关于应对新冠肺炎疫情影响强化稳就业举措的要求，在推动农民工有序返城返岗就业的同时，要加强政策扶持，强化指导服务，扩大返乡留乡农民工就地就近就业规模，努力实现全年促进农民工就业目标。

　　一、总体要求

　　贯彻落实习近平总书记在统筹推进新冠肺炎疫情防控和经济社会发展工作部署会议上的重要讲话精神和国务院的部署，以实施乡村振兴战略为总抓手，以促进农民就业增收为目标，集成政策措施，集聚资源要素，集合公共服务，促进返乡留乡农民工就地就近就业创业，形成就业促增收、致富奔小康的良好局面，为全面小康和脱贫攻坚提供支撑。

　　二、目标任务

　　（一）回归农业稳定一批。引导返乡留乡农民工投入农业生产，领办合办农民合作社、农机服务社，开办家庭农场，兴办特色种植业和规模养殖业，推进农产品产业链、物流体系建设，扩大农业生产和服务领域的就业机会。

　　（二）工程项目吸纳一批。抓住补齐“三农”短板的机遇，以实施农村基础设施项目为带动，引导返乡留乡农民工参与农田水利、村庄道路、人居环境整治、乡村绿化等工程项目建设，吸纳更多返乡留乡农民工就业。

　　（三）创新业态培育一批。依托农业农村资源，发掘农业多种功能和乡村多重价值，催生加工流通、休闲旅游、健康养生、农事体验、电子商务、直播直销等新产业新业态，丰富产业形态，增加返乡留乡农民工就业。

　　（四）扶持创业带动一批。实施返乡留乡农民工创业推进行动，强化政策引导，优化创业环境，引导返乡留乡农民工积极发展乡村车间、家庭工场、手工作坊、创意农业等，带动更多返乡留乡农民工就业。

　　（五）公益岗位安置一批。对通过市场渠道难以就业的农民工，整合各类资源，积极拓宽渠道，开发乡村保洁员、水管员、护路员、生态护林员等公益性岗位，托底安置返乡留乡农民工就业。

　　三、重点措施

　　（一）落实就业扶持政策。落实吸纳农民工就业的财政、税收、信贷等援企稳岗政策。按照稳就业和返乡入乡创业工作要求，对首次创业、正常经营1年以上的返乡留乡创业农民工，给予一次性创业补贴。按照普惠金融发展专项资金管理办法，对符合条件的返乡留乡农民工创业担保贷款予以贴息。

　　（二）引导企业扩大岗位。支持企业特别是农业产业化龙头企业通过临时性、季节性、弹性用工等形式，吸引返乡留乡农民工灵活就业。鼓励企业间开展用工调剂，采取借调代岗形式，增加就业机会，实现返乡留乡农民工共享就业。支持企业延伸产业链和服务外包，吸引返乡留乡农民工在加工、包装、运输等环节交替上岗，实现返乡留乡农民工临时兼业。有序推进乡村劳动密集型制造业、服务业企业和中小微企业复工复产，实现返乡留乡农民工到岗就业。

　　（三）开发更多新型业态。积极发展生产性服务业，吸引返乡留乡农民工在农资供应、统防统治、代耕代种、农机维修等农业前端行业就业。积极发展农产品初加工，吸引农民工在农产品储藏保鲜、分等分级、清洗包装等后端行业就业。跨界配置农业和现代产业要素，吸引返乡留乡农民工在休闲旅游、健康养生、共享农庄、农村电商等新业态就业。鼓励返乡留乡农民工发展乡村养老育幼、家政服务、资源回收等生活性服务业。

　　（四）加强基础设施建设。加快实施高标准农田建设、水利设施建设、乡村道路改造、小流域治理、农村危房改造、人居环境整治等工程，吸纳返乡留乡农民工就业。通过现代农业产业园、农业产业强镇、优势特色产业集群等农业项目的实施，优先安排返乡留乡农民工就业。引导社会资本下乡，建设原料基地，下沉加工产能，兴办商贸物流，增加返乡留乡农民工就业岗位。

　　（五）优化就业创业服务。及时向社会公布政策清单、申办流程、补贴标准，确保政策和服务落实到位。组织开展线上线下多元化就业指导，健全岗位信息公共发布平台，实现岗位信息在线发布和跨地区共享。加强返乡留乡农民工定期联系和分级分类服务。在县乡行政服务大厅设立服务窗口，为返乡留乡农民工就业创业提供“一站式”服务。

　　（六）开展职业技能培训。实施返乡留乡职业技能提升行动，运用互联网+职业技能培训模式，按照就业意向、区域特点和产业需求，开发一批特色专业和示范培训线上培训课程资源。加强返乡留乡农民工专项培训，开展返乡留乡农民工技能提升培训或转岗转业培训。加强就业见习实习、创业孵化实训基地建设，鼓励培训机构与企业联合开展定向、定岗、订单式就业创业技能培训。组建创业导师队伍和专家顾问团，建立专业化、规模化、制度化培养机制。

　　四、组织保障

　　（一）强化协调保障。各级农业农村部门和人力资源社会保障部门要按照农业农村优先、就业优先的要求，落实支持农业农村发展、促进就业创业等扶持政策，加强工作协调，明确任务、压实责任，推进措施落实。鼓励地方加大财政、信贷资金投入，支持返乡留乡农民工稳定就业、顺畅创业。

　　（二）强化宣传引导。利用各类媒体，加强宣传引导和政策解读。发掘促进返乡留乡农民工就地就业创业工作典型经验，选树返乡留乡农民工在中西部地区、重点贫困地区就业创业的先进典型，讲好就业故事，发挥典型引路作用，营造支持返乡留乡农民工就业创业良好氛围。

　　（三）强化监测调度。对返乡留乡农民工就业创业情况进行摸底调查，加强返乡留乡农民工就业和创业动态监测，与有关部门加强沟通协作，开展大数据对比分析，做好就业形势研判。及时协调解决返乡留乡农民工就业创业存在的突出问题，努力实现全年工作目标。

1. 民航局：疫情防控期间继续调减国际客运航班量

各运输航空公司：

　　为坚决遏制境外新冠肺炎疫情输入风险高发态势，根据国务院疫情联防联控工作要求，现决定进一步调减国际客运航班运行数量。具体要求如下：

　　一、以民航局3月12日官网发布的“国际航班信息发布（第5期） ”为基准，国内每家航空公司经营至任一国家的航线只能保留1条，且每条航线每周运营班次不得超过1班；外国每家航空公司经营至我国的航线只能保留1条，且每周运营班次不得超过1班。

　　二、请各航空公司根据上述要求，提前向民航局运行监控中心申请预先飞行计划。

　　三、各航空公司按照本通知的要求调减航班涉及的航线经营许可和起降时刻等予以保留。

　　四、各航空公司要严格执行民航防控工作领导小组办公室印发的最新版《运输航空公司疫情防控技术指南》。在抵离中国的航班上采取严格的防控措施，确保客座率不高于75%。

　　五、根据疫情防控需要，我局可能出台进一步收紧国际客运航班总量的政策，请各航空公司密切关注、提前研判，做好已售机票的延期、退票等处置工作。

　　六、各航空公司可利用客机执行全货运航班，不计入客运航班总量。

　　七、各航空公司根据本通知第一条调整的航班计划自2020年3月29日起执行。

　　八、本通知自发布之日起生效，截止日期另行通知。自本通知生效之日起，民航发〔2020〕11号通知失效。

　　中国民用航空局

　　2020年3月26日

1. 工信部：开展产业链固链行动 推动产业链协同复工复产的通知 工信厅政法函〔2020〕54号

各省、自治区、直辖市及新疆生产建设兵团工业和信息化主管部门，部机关有关司局：

为贯彻落实国务院常务会议部署，以龙头企业带动上下游配套企业协同复工复产，决定开展产业链固链行动。有关事项通知如下：

一、总体要求

深入学习领会习近平总书记关于统筹做好疫情防控和经济社会发展工作的重要指示精神，贯彻落实党中央、国务院决策部署，按照国务院常务会议关于推动产业链协同复工复产工作要求，在分区分级精准防控的同时，有序推动全产业链加快复工复产。坚持以大带小、上下联动、内外贸协同，聚焦重点产业链，以龙头企业带动上下游配套中小企业，特别是“专精特新”中小企业，增强协同复工复产动能。加强统筹指导和协调服务，打通产业链、供应链堵点，落实各项支持政策，协调解决企业实际困难，畅通产业链、资金链循环，维护产业链稳定。

二、主要任务

（一）梳理解决企业实际困难。围绕防疫物资、民生保障、春耕备耕、国际供应链产品、劳动密集型产业等重点领域，梳理龙头企业及其产业链上下游未复工达产的核心配套企业名单。建立“一对一”联系机制，采用视频连线、电话会议、微信群等方式建立监测会商制度，发挥行业协会作用，分类梳理企业困难，积极协调推动解决。

（二）落实援企稳企惠企政策。加强政策宣传，指导企业用好用足已出台政策。推动落实支农支小再贷款再贴现政策，阶段性减免企业社会保险费和企业缓缴住房公积金政策，失业保险稳岗返还政策，对中小微企业贷款实施临时性还本付息等各类支持政策。深化产融合作，加强产融平台信息对接，提高金融资源配置效率。加大清理拖欠民营企业中小企业账款力度。加强企业防疫物资保障。

（三）激发市场活力，拉动产业链协同复工复产。发挥“互联网+”作用，拉动轻纺、家电、汽车等传统消费，培育智慧健康养老、绿色产品等消费热点。支持发展远程医疗、在线教育、数字科普、在线办公、协同作业等新业态新模式，拓展数字网络等信息消费。实施中小企业数字化赋能专项行动。加快5G网络、物联网、大数据、人工智能、工业互联网、智慧城市等新基础设施建设，加快制造业智能化改造。加快工业和通信业重点项目开工建设，跟踪抓好重大外资项目落地，推动在建项目尽快投产达产。

（四）开展国际疫情研判，做好政策储备。加强全球疫情对重点产业链影响的分析研判，提出应对预案和政策储备。梳理产业链关键领域和薄弱环节，研究出台支持政策，引导企业稳固供应链，提升产业链水平。保障在全球产业链中有重要影响的龙头企业和关键环节平稳生产，维护国际供应链稳定。

三、工作要求

（一）切实发挥推动产业链协同复工复产工作专班作用，促进产业链上下游、大中小企业协同复工复产，实现良性互动。加强统筹协调，采取“一事一议”“一企一策”方式，推动解决龙头企业及其核心配套企业复工复产中的跨部门跨地区问题，帮助企业尽快复工复产。

（二）各地工业和信息化主管部门要结合实际建立跨部门工作专班。加强对行业龙头企业及核心配套企业的跟踪服务，结合本地产业特色，梳理形成一批龙头企业及核心配套企业名单，开展精准对接。加大对中小型关联企业的融资支持。加强对企业反映问题的分类梳理，能够属地解决的要及时解决，对需要国家层面协调解决的跨部门跨地区问题要及时向工业和信息化部反映。

（三）部机关有关司局要分批梳理行业龙头企业及其产业链上下游未复工达产的核心配套企业名单。加强与各地及重点企业的沟通联系，建立日调度制度，加强研究分析和跟踪服务，逐项解决地方和企业反映的问题。对跨部门跨地区问题要及时提交推动产业链协同复工复产工作专班研究。部复工复产联络员工作组要深入基层，及时掌握第一手情况，积极协调解决问题，推动政策落实。

特此通知。

工业和信息化部办公厅

2020年3月23日

1. 交通运输部等4部门：做好有关人员进出湖北省交通运输保障工作

各省、自治区、直辖市、新疆生产建设兵团交通运输厅(局、委)、公安厅(局)、人力资源社会保障厅(局)、卫生健康委：

为深入贯彻习近平总书记关于做好“点对点、一站式”输送返岗、外地滞留在鄂人员返乡等工作的重要指示精神，认真落实中央应对新冠肺炎疫情工作领导小组关于进一步做好疫情防控和后续相关工作推进全面复工复产的决策部署，根据中央批准解除离鄂离汉通道管控的部署安排，现就做好有关人员进出湖北省交通运输保障工作有关事项通知如下：

一、精心组织“点对点”返岗返乡包车

(一)摸清返岗、返乡需求底数。湖北省人力资源社会保障部门要会同有关部门，通过线上线下多种渠道，利用好农民工返岗复工“点对点”线上服务系统和用工对接平台，组织市县摸排湖北省复工复产企业用工人员返岗和本地务工人员外出返岗需求，汇集返岗人员姓名、联系方式、返岗时间、来源地、用工企业及联系人等信息。湖北省以外各地人力资源社会保障部门要组织摸排入鄂返岗务工人员出行需求信息。输出地与输入地人力资源社会保障部门要加强对接，及时将有关信息与交通运输、卫生健康等部门共享。各地交通运输部门要及时获取外地滞留在汉在鄂人员、湖北籍在外滞留人员返乡需求信息。

(二)开展行前服务。输出地、输入地交通运输部门要根据人力资源社会保障部门提供的返岗需求信息、当地疫情联防联控机制提供的滞留人员返乡需求信息，按照“一车一方案”的原则，提前制定包车运输组织方案，为目的地集中人员提供“点对点”直达运输服务。输出地卫生健康部门要在当地人民政府的统一领导下，指导做好相关人员的防疫健康教育和体温检测工作。输出地、输入地交通运输部门要在行前主动对接包车相关信息，输入地各有关部门要在当地人民政府统一领导下，提前做好人员接收准备。

(三)有序组织包车运输。输出地或输入地交通运输部门要做好进出湖北省除武汉市外其他地区的省际“点对点”包车运输组织。3月25日零时起，可组织开行进出武汉市的省际“点对点”包车。在做好健康管理、落实防控措施的前提下，对持有湖北健康码“绿码”的武汉外出务工人员，经核酸检测合格后，采取“点对点、一站式”的方式集中精准输送，确保安全有序返岗。如湖北省运力不足，对开省份和周边省份交通运输部门要积极予以支持。包车承运单位可按照《交通运输部关于疫情防控期间免收农民工返岗包车公路通行费的通知》(交公路明电〔2020〕52号)要求格式自制车辆通行证，随车携带，享受“不停车、不检查、不收费、优先便捷通行”政策。沿线高速公路经营管理单位要做好相关服务保障工作。各地公安机关要组织做好“点对点”包车的交通安全管理工作。输入地各有关部门要在当地人民政府统一领导下，做好包车抵达后的人员交接工作。

二、有序恢复进出湖北道路客运服务

(四)分步实施省际道路客运恢复工作。3月25日零时起，可恢复运行进出湖北省除武汉市外其他地区的省际道路客运;4月8日零时起，可恢复运行进出武汉市的省际道路客运。

(五)提供安全便捷的运输服务。各地交通运输部门要督促指导道路客运经营者，抓紧开展客车技术状况全面检查，及时开展维修维护保养，确保车辆技术状况良好。要加强对相关从业人员的教育培训，提升安全、防疫、应急处置能力。要及时向社会公告恢复客运服务情况，并通过联网售票、电子客票、联程运输等方式，服务乘客便捷出行。

三、强化公路通行保障

(六)分步开展有关人员自驾离鄂离汉。3月25日零时起，湖北省除武汉市以外其他地区、持有湖北健康码“绿码”的人员可通过自驾离鄂;4月8日零时起，持有湖北健康码“绿码”的人员可通过自驾离汉。湖北及周边省份公安机关要会同交通运输部门严格按照时间节点要求，有序解除离鄂离汉通道管控。

(七)切实做好公路疏堵保畅。各省级交通运输部门特别是湖北省及周边省份交通运输部门要加强公路网运行监测，科学研判流量变化情况，利用公路沿线情报板、电视、广播、微信、微博、导航软件等多种方式，及时发布公路路况和交通管制等信息，引导公众合理选择行驶路线或错峰出行。及时增加高速公路收费站工作人员，积极配合公安机关做好现场交通疏导及远端分流等工作。

四、全面加强防疫和安全管理

(八)落实分区分级防控要求。各地交通运输部门要指导道路客运经营者、客运站做好乘客行前体温检测，坚决防止有发热等症状人员带病出行，要组织乘客和司乘人员全程佩戴口罩。要督促指导道路客运经营者提前制定应急运输预案，严格落实《交通运输部关于分区分级科学做好客运场站和交通运输工具新冠肺炎疫情防控工作的通知》(交运明电〔2020〕84号)等要求，做好客车消毒通风、卫生清洁、人员防护、车内留观区设置、乘客途中测温、发热乘客移交等防疫工作。出入中、高风险区的省际、市际客运班车和包车要严格将客座率控制在50%以内。

(九)强化道路客运安全管理。各地交通运输部门要加强信息共享和源头监管，督促道路客运企业落实安全主体责任，选用合格车辆、合格驾驶人进行运输，加强道路客运车辆动态监管，严格执行长途客运车辆凌晨2时至5时停车休息或者接驳运输规定，切实消除安全隐患。公安机关要依法从严查处超速、疲劳驾驶等严重违法行为，维护交通安全。

五、严格落实进京管理要求

(十)严格按部署组织进京运输。北京滞留在鄂人员返京和务工人员进京“点对点”包车运输，按照首都严格进京管理联防联控协调机制部署执行。进出北京的省际道路客运暂不恢复运营，恢复时间由首都严格进京管理联防联控协调机制确定。

(十一)依法从严打击进京非法营运。京津冀地区交通运输部门要会同公安机关加强信息共享和执法联动，加大非法营运车辆查处力度，坚决防止疫情通过非法营运车辆输入北京。

(十二)服务北京滞留在鄂人员安全有序自驾返京。北京市、湖北省交通运输部门要积极对接相关部门，及时获取经批准可自驾返京人员及车辆信息，并告知河北、河南省交通运输部门;通过“京心相助”小程序，定点推送相关信息，引导自驾回京人员尽量选择指定通道返回，中途需停车休息的，进入指定服务区停车休息(详见附件)。河北、河南省交通运输部门要及时将获取的自驾离鄂返京人员及车辆信息通知沿途指定服务区，并指导服务区经营管理单位切实做好车辆停放、体温检测、服务保障等相关工作;要会同卫生健康、公安等部门，加强服务区防疫力量部署,强化服务区安全管控，共同做好疫情防控和服务保障工作。

进出湖北省的铁路、民航运输保障工作要求，由国家铁路局、中国民用航空局负责部署。

附件：北京滞留在鄂人员自驾返京公路通道及服务区

交通运输部

公安部

人力资源社会保障部

国家卫生健康委

2020年3月24日

1. 卫健委 财政部：做好中央派遣支援湖北省新冠肺炎疫情防控工作医务人员生活保障 国卫办财务发〔2020〕3号

各省、自治区、直辖市及新疆生产建设兵团卫生健康委、财政厅（局）：

为进一步做好中央派遣支援湖北省新冠肺炎疫情防控工作医务人员（包括国家组织派往湖北省的医务人员、按照国家统一要求派出的省际对口支援湖北省各市州的医务人员，均包含疾控人员，以下统称援鄂医务人员）的生活保障，现将有关工作通知如下：

一、援鄂医务人员往返湖北省的交通费用，由组派单位同级财政承担。

二、受援地负责安排好援鄂医务人员的衣食住行等生活保障，所需费用由受援地承担。

三、受援地负责统计援鄂医务人员的临时性工作补助有关工作量，由派出省份单列报送国家卫生健康委。补助经费由派出地财政部门按照规定垫付，中央财政据实结算。此前已由受援地发放部分，派出地不再重复发放。

四、地方自行出台的其他有关援鄂医务人员待遇政策，由出台政策的地方负责落实并负担费用。

国家卫生健康委办公厅  财政部办公厅

2020年3月17日

1. 住建部：进一步做好城市环境卫生工作

各省、自治区住房和城乡建设厅，直辖市城市管理委（城市管理局、绿化市容局）：

新冠肺炎疫情发生以来，各地环境卫生主管部门和环卫作业单位坚决贯彻落实习近平总书记系列重要指示精神，按照党中央国务院决策部署，全面投入疫情防控的人民战争、总体战、阻击战，全力保障城市整洁、守护公众安全，取得了重要成绩。特别是广大一线环卫工人，发扬“宁愿一人脏，换来万家净”的精神，沐风栉雨、尽职尽责、勇于担当、不辱使命，成为疫情中的逆行者和城市中的暖人风景线。为进一步巩固疫情防控期间城市环卫各项工作成果，保障各地复工复产，弘扬城市环卫精神，推进城市环卫工作健康发展，现将有关事项通知如下：

一、切实关心关爱一线环卫工作者

（一）继续做好安全防护。各级环卫行业主管部门要结合当地实际，加快完善同疫情防控相适应的城市公共区域清扫保洁、生活垃圾收运处理、公厕管理和粪便收运处理等工作的流程规范，指导督促环卫作业单位不折不扣执行。要在属地卫生健康、疾病控制等部门的指导下，完善环卫作业人员防护措施，督促环卫作业单位切实履责，指导环卫职工增强自我保护意识，坚决防止松懈、麻痹大意思想，保护环卫工人的生命安全和身体健康。继续做好对环卫作业工具、作业场所、工间休息场所，以及环卫职工宿舍、食堂、浴室等区域的消毒灭菌工作。

（二）适时启动休息调整。要督促指导环卫作业单位根据环卫作业量的变化，加强力量统筹、做好生产调度，采取轮休、补休等方式，保证长期在一线作业的环卫职工得到必要休整。对长时间高负荷一线作业的环卫职工，要加强人文关怀，组织开展心理疏导。千方百计做好一线环卫工人的饮食调剂和休息保障工作。开展必要的走访慰问活动，帮助一线环卫职工解决家庭实际困难。

二、全力巩固城市环卫疫情防控成果

（三）继续做好清扫保洁和消毒杀菌工作。各级环卫行业主管部门要根据疫情防控形势变化和当地部署，按照标准规范要求，指导环卫作业单位做好城市道路等清扫保洁工作，强化机械化保洁作业方式，科学设置人工普扫频次。结合地区实际，继续做好医院、商超市场等重点区域及其周边的清扫保洁，并在当地卫生健康部门的指导下，对必要点位进行消毒灭菌作业。根据当地复工复产后防疫工作预案，积极有效做好企业园区、公交站点、交通枢纽等复工后人流密集区域周边的保洁和消毒杀菌工作。

（四）严格生活垃圾收集运输管理。根据复工复产后生活垃圾产生量的变化，合理调配作业力量，加强生活垃圾全过程监管，及时收集、清运、处理，确保生活垃圾日产日清，继续严格防止医疗废物混入生活垃圾。严格落实《国家卫生健康委办公厅关于做好新型冠状病毒感染的肺炎疫情期间医疗机构医疗废物管理工作的通知》（国卫办医函〔2020〕81号）《国家卫生健康委办公厅关于加强新冠肺炎首诊隔离点医疗管理工作通知》（国卫办医函〔2020〕120号）等要求，医疗机构和首诊隔离点在诊疗新冠肺炎活动中产生的口罩等废弃物，继续按照医疗废物进行管理。继续对废弃口罩收集、清运实行分类分区域管理。居民日常使用产生的口罩，作为生活垃圾管理，要严格实施无害化处理。

（五）规范生活垃圾处理设施运行管理。要督促指导生活垃圾处理设施运营单位严格执行相关标准规范，做好生活垃圾转运站、填埋场、焚烧厂等运行管理工作，保证生活垃圾得到无害化处理。要进一步规范进入处理设施各类生活垃圾的检验、称重计量和数据统计，严格禁止医疗废物等进入处理设施。当地党委和政府另有规定的可从其规定。

（六）做好公厕运行管理和粪便收运处理。要督促指导各责任单位继续做好公厕和化粪池的日常维护。全面落实公厕保洁、消毒、运行维护措施，加强化粪池巡查监管。做好对粪便收运车辆设备、处理设施、作业场所的日常维护和消毒杀菌。根据实际情况，适当调整粪便处理设施工艺参数，保证粪便无害化处理。

三、全面推进环卫各项工作

（七）扎实推进生活垃圾分类工作。46个重点城市要增强紧迫感，按既定方案加快建立分类投放、分类收集、分类运输、分类处理系统，积极有序推进厨余垃圾等分类处理设施建设，努力把疫情造成的损失降到最低限度，确保如期完成生活垃圾分类目标任务。武汉、北京等疫情防控重点地区，要在落实防疫措施前提下推进生活垃圾分类工作。各省、自治区住房和城乡建设厅要督促指导其他各地级城市，进一步细化实施方案，确定生活垃圾分类标准，明确目标任务、重点项目、配套政策、具体措施，扎实推进生活垃圾分类工作。

（八）扎实推进在建新建项目复工开工。各级环卫行业主管部门要进一步摸排本地区各类环卫设施存在的短板，特别是梳理疫情防控期间暴露出的设施短板。要抓住机遇，有针对性加快各类环卫设施建设，包括生活垃圾分类转运和焚烧、填埋、生物处理设施，垃圾渗滤液处理设施，建筑垃圾处置和资源化利用设施，填埋场封场治理项目等。要对全部在建和新建环卫设施项目，建立项目台账，细化项目规模、工艺、投资、开工时间等内容，配合有关部门多渠道落实建设资金，确保项目及早复工、开工，扎实有序加快建设，为积极扩大有效需求多做贡献。

（九）加强建筑垃圾治理工作。总结推广建筑垃圾治理试点经验，加强建筑垃圾全过程管理。建立渣土堆放场所常态化监测机制，消除安全隐患。加快建筑垃圾填埋消纳设施建设，规范作业管理。加快建筑垃圾回收和再利用体系建设，推动建筑垃圾资源化利用，因地制宜推进再生产品应用。35个建筑垃圾治理试点城市要建立长效机制，巩固试点成果，充分发挥示范带头作用。

（十）持续加大宣传力度。各地要建立健全长效宣传机制，全面展示环卫人任劳任怨、无私奉献的风采。要及时总结疫情防控期间，环卫行业攻坚克难、履职尽责、慎终如始，圆满完成所承担的日常及应急任务的经验做法，挖掘先进人物、先进集体、先进事迹，大力开展正面宣传。要深入分析疫情对环卫行业的影响，明确相关文件、标准规范、操作指南、技术规程的适用范围和时间，研究提出下一步推进环卫工作的建议。

有关情况请及时反馈至城市建设司环卫处。

联系人：简正 王开

电 话：010-58934756 传真：010-58933434

中华人民共和国住房和城乡建设部办公厅

2020年3月20日

1. 商务部等：应对新冠肺炎疫情支持边境（跨境）经济合作区建设促进边境贸易创新发展有关工作

内蒙古自治区、辽宁省、吉林省、黑龙江省、广西壮族自治区、云南省、新疆维吾尔自治区商务主管部门，中国进出口银行各相关分行：

边境经济合作区、跨境经济合作区（以下简称边境（跨境）经济合作区）是沿边地区贸易投资的重要平台、经济社会发展的重要支撑。为深入学习贯彻习近平总书记关于统筹推进新冠肺炎疫情防控和经济社会发展工作重要讲话精神，认真落实党中央、国务院决策部署，加强对边境（跨境）经济合作区的金融支持，促进边境贸易创新发展，稳住外贸外资基本盘，现将有关事项通知如下：

一、发挥金融职能作用，做好疫情应对工作

进出口银行作为支持中国对外经济贸易投资发展与国际经济合作的重要银行机构，在服务企业、服务地方、服务开放型经济发展等方面具有专业优势，发挥着重要作用。各地商务主管部门要发挥政策引导作用，统筹疫情应对和稳住外贸外资基本盘需要，支持进出口银行为边境（跨境）经济合作区及区内企业提供优质服务，促进解决复工复产面临的资金周转和扩大融资等迫切问题，保障边境贸易产业链、供应链畅通运转，将疫情带来的影响降到最低。进出口银行各相关分行要立足职能定位，加大金融支持力度，创新完善金融支持方式，合理配置信贷资源，优化投向结构，支持园区和企业应对疫情、复工复产、拓展业务，为稳边安边兴边做出积极贡献。

二、加大对重点领域的信贷支持力度，培育开放发展新动能

（一）推动边境（跨境）经济合作区“小组团”滚动开发。按照《商务部 自然资源部关于推动边境经济合作区探索“小组团”滚动开发的通知》（商资函〔2019〕410号）要求，围绕“小组团”滚动开发，积极参与边境（跨境）经济合作区产业规划、投融资计划、招商运营计划等方案编制及项目实施，支持重点组团培育，做好融资融智服务，促进边境（跨境）经济合作区建设及区内企业发展。

（二）支持边境地区承接产业转移。立足边境地区特点，进一步挖掘发展潜力，以边境（跨境）经济合作区为主要平台载体，以支持加工制造业转型升级为重点，完善对园区及区内企业的信贷融资、项目对接、业务咨询等服务，促进东部地区产业有序转移，提升边境地区承接产业转移能力，培育沿边特色优势产业。

（三）完善边境贸易产业链。支持边境（跨境）经济合作区及相关边贸商品市场的基础设施、物流仓储等配套设施建设，为小额贸易企业和进出口加工业企业等提供流动资金、厂房建设、设施设备购置等贷款，促进边境贸易产品落地加工，发展跨境电商等新业态，将边境（跨境）经济合作区建成集边境贸易、加工制造、生产服务、物流采购于一体的高水平沿边开放平台。

（四）推动市场多元化。支持边境（跨境）经济合作区主动融入共建“一带一路”，深化与周边经贸合作，积极拓展农产品及关键设备技术进口，扩大对新兴市场国家的出口。

（五）支持“引进来”和“走出去”。鼓励招商引资，吸引企业到边境（跨境）经济合作区投资经营。支持边境（跨境）经济合作区内企业开展符合境外投资方向的境外投资。

三、建立合作和政策保障机制，将工作落实落细

（一）建立对口联系人机制。商务部（外资司）和进出口银行（战略规划部）建立日常联系和会晤机制，加强信息沟通和情况交流，可通过举办业务对接会、政策研讨、调研、培训等形式，推动各地商务主管部门、边境（跨境）经济合作区和进出口银行各相关分行深入合作。各地商务主管部门、边境（跨境）经济合作区及进出口银行各相关分行要主动对接、密切协同，建立专人联系机制，积极推进相关合作事宜。

（二）建立企业和项目推荐机制。各地商务主管部门和进出口银行各相关分行建立优质企业和项目推荐机制，发挥各自优势共同筛选拟支持对象，并以多种形式加大宣传指导，推动有关支持政策措施落实落地。

（三）加强政策保障。商务部加强统筹指导，支持进出口银行开展相关业务。进出口银行各相关分行在合规和风险可控的前提下，加大信贷资源倾斜，提高审批效率，切实加强对边境（跨境）经济合作区及相关边境贸易工作的金融支持。重点加大对边境地区制造业中长期贷款投放力度，扶持小微和民营企业发展。

（四）加大服务力度。各地商务主管部门与进出口银行各相关分行应通过实地走访、调研、银（政）企座谈会等多种形式加强与边境（跨境）经济合作区及相关边境贸易企业的联系，立足当地情况、产业特色、企业实际融资需求等，制定有针对性的工作方案，灵活、便利、准确实施。进出口银行各相关分行应积极发挥金融的支持和带动作用，通过开展银团贷款、小微企业银行转贷款、投贷结合、银保合作、银担合作等方式，联合商业银行、保险公司、融资担保基金等金融机构共同解决企业融资难题，更好满足边境贸易加快发展的金融服务需求。

（五）注重风险管控。各地商务主管部门与进出口银行各相关分行在开展相关工作时，要客观、全面评估当地财政状况、产业规划、企业经营状况、项目可行性和发展前景、潜在风险等要素，切实做好相关风险预防和管控。

商务部办公厅 中国进出口银行办公室

2020年3月23日

1. 国办：发布应对新冠肺炎疫情影响强化稳就业举措的实施意见 国办发〔2020〕6号

各省、自治区、直辖市人民政府，国务院各部委、各直属机构：

为深入贯彻习近平总书记关于统筹推进新冠肺炎疫情防控和经济社会发展工作的重要指示精神，加快恢复和稳定就业，经国务院同意，现提出如下意见：

一、更好实施就业优先政策

（一）推动企业复工复产。坚持分区分级精准防控，提高复工复产服务便利度，取消不合理审批，坚决纠正限制劳动者返岗的不合理规定。加快重大工程项目、出口重点企业开复工，以制造业、建筑业、物流业、公共服务业和农业生产等为突破口，全力以赴推动重点行业和低风险地区就业，循序渐进带动其他行业和地区就业。协调解决复工复产企业日常防护物资需求，督促其落实工作场所、食堂宿舍等防控措施。（发展改革委、工业和信息化部、交通运输部、卫生健康委按职责分工负责）

（二）加大减负稳岗力度。加快实施阶段性、有针对性的减税降费政策。加大失业保险稳岗返还，对不裁员或少裁员的中小微企业，返还标准最高可提至企业及其职工上年度缴纳失业保险费的100%，湖北省可放宽到所有企业；对暂时生产经营困难且恢复有望、坚持不裁员或少裁员的参保企业，适当放宽其稳岗返还政策认定标准，重点向受疫情影响企业倾斜，返还标准可按不超过6个月的当地月人均失业保险金和参保职工人数确定，或按不超过3个月的企业及其职工应缴纳社会保险费确定。2020年6月底前，允许工程建设项目暂缓缴存农民工工资保证金，支付记录良好的企业可免缴。切实落实企业吸纳重点群体就业的定额税收减免、担保贷款及贴息、就业补贴等政策。加快实施阶段性减免、缓缴社会保险费政策，减免期间企业吸纳就业困难人员的社会保险补贴期限可顺延。（财政部、人力资源社会保障部、住房城乡建设部、交通运输部、水利部、人民银行、税务总局按职责分工负责）

（三）提升投资和产业带动就业能力。实施重大产业就业影响评估，明确重要产业规划带动就业目标，优先投资就业带动能力强、有利于农村劳动力就地就近就业和高校毕业生就业的产业。加快制定和完善引导相关产业向中西部地区转移的政策措施。对部分带动就业能力强、环境影响可控的项目，制定环评审批正面清单，加大环评“放管服”改革力度，审慎采取查封扣押、限产停产等措施。（发展改革委、人力资源社会保障部、生态环境部、商务部按职责分工负责）

（四）优化自主创业环境。深化“证照分离”改革，推进“照后减证”和简化审批，简化住所（经营场所）登记手续，申请人提交场所合法使用证明即可登记。充分发挥创业投资促进“双创”和增加就业的独特作用，对带动就业能力强的创业投资企业予以引导基金扶持、政府项目对接等政策支持。加大创业担保贷款支持力度，扩大政策覆盖范围，优先支持受疫情影响的重点群体，对优质创业项目免除反担保要求。政府投资开发的孵化基地等创业载体应安排一定比例场地，免费向高校毕业生、农民工等重点群体提供。各类城市创优评先项目应将带动就业能力强的“小店经济”、步行街发展状况作为重要条件。（发展改革委、工业和信息化部、财政部、人力资源社会保障部、商务部、人民银行、市场监管总局、银保监会、全国妇联按职责分工负责）

（五）支持多渠道灵活就业。合理设定无固定经营场所摊贩管理模式，预留自由市场、摊点群等经营网点。支持劳动者依托平台就业，平台就业人员购置生产经营必需工具的，可申请创业担保贷款及贴息；引导平台企业放宽入驻条件、降低管理服务费，与平台就业人员就劳动报酬、工作时间、劳动保护等建立制度化、常态化沟通协调机制。取消灵活就业人员参加企业职工基本养老保险的省内城乡户籍限制，对就业困难人员、离校2年内未就业高校毕业生灵活就业后缴纳社会保险费的，按规定给予一定的社会保险补贴。（财政部、人力资源社会保障部、自然资源部、人民银行、市场监管总局按职责分工负责）

二、引导农民工安全有序转移就业

（六）引导有序外出就业。强化重点企业用工调度保障、农民工“点对点、一站式”返岗复工服务，推广健康信息互认等机制，提升对成规模集中返岗劳动者的输送保障能力。引导劳动者有序求职就业，及时收集发布用工信息，加强输出地和输入地信息对接，鼓励低风险地区农民工尽快返岗复工。对组织集中返岗、劳务输出涉及的交通运输、卫生防疫等给予支持。对人力资源服务机构、劳务经纪人开展跨区域有组织劳务输出的，给予就业创业服务补助。（公安部、财政部、人力资源社会保障部、交通运输部、卫生健康委按职责分工负责）

（七）支持就地就近就业。抓好春季农业生产，大力发展新型农业经营主体，组织暂时无法外出的农民工投入春耕备耕，从事特色养殖、精深加工、生态旅游等行业。在县城和中心镇建设一批城镇基础设施、公共服务设施，加强农业基础设施建设，实施农村人居环境改善工程，开展以工代赈工程建设，优先吸纳农村贫困劳动力和低收入群体就业。（发展改革委、人力资源社会保障部、交通运输部、农业农村部、卫生健康委按职责分工负责）

（八）优先支持贫困劳动力就业。企业复工复产、重大项目开工、物流体系建设等优先组织和使用贫困劳动力，鼓励企业更多招用贫困劳动力。支持扶贫龙头企业、扶贫车间尽快复工。利用公益性岗位提供更多就地就近就业机会，优先对贫困劳动力托底安置。加大对“三区三州”等深度贫困地区、52个未摘帽贫困县、易地扶贫搬迁大型安置区的支持力度。对吸纳贫困劳动力就业规模大的，各地可通过财政专项扶贫资金给予一次性奖励。（发展改革委、财政部、人力资源社会保障部、农业农村部、扶贫办按职责分工负责）

三、拓宽高校毕业生就业渠道

（九）扩大企业吸纳规模。对中小微企业招用毕业年度高校毕业生并签订1年以上劳动合同的，给予一次性吸纳就业补贴。国有企业今明两年连续扩大高校毕业生招聘规模，不得随意毁约，不得将本单位实习期限作为招聘入职的前提条件。（财政部、人力资源社会保障部、国资委、烟草局、邮政局等部门和企业按职责分工负责）

（十）扩大基层就业规模。各级事业单位空缺岗位今明两年提高专项招聘高校毕业生的比例。开发城乡社区等基层公共管理和社会服务岗位。扩大“三支一扶”计划等基层服务项目招募规模。出台改革措施，允许部分专业高校毕业生免试取得相关职业资格证书。畅通民营企业专业技术职称评审渠道。（教育部、民政部、财政部、人力资源社会保障部、农业农村部按职责分工负责）

（十一）扩大招生入伍规模。扩大2020年硕士研究生招生和普通高校专升本招生规模。扩大大学生应征入伍规模，健全参军入伍激励政策，大力提高应届毕业生征集比例。（发展改革委、教育部、财政部、退役军人部、中央军委政治工作部、中央军委国防动员部按职责分工负责）

（十二）扩大就业见习规模。支持企业、政府投资项目、科研项目设立见习岗位。对因疫情影响见习暂时中断的，相应延长见习单位补贴期限。对见习期未满与高校毕业生签订劳动合同的，给予见习单位剩余期限见习补贴。（财政部、人力资源社会保障部、商务部、国资委、共青团中央按职责分工负责）

（十三）适当延迟录用接收。引导用人单位推迟面试体检和签约录取时间。对延迟离校的应届毕业生，相应延长报到接收、档案转递、落户办理时限。离校未就业毕业生可根据本人意愿，将户口、档案在学校保留2年或转入生源地公共就业人才服务机构，以应届毕业生身份参加用人单位考试、录用，落实工作单位后参照应届毕业生办理相关手续。（教育部、人力资源社会保障部、国资委按职责分工负责）

四、加强困难人员兜底保障

（十四）保障失业人员基本生活。畅通失业保险金申领渠道，放宽失业保险申领期限，2020年4月底前实现线上申领失业保险金。对领取失业保险金期满仍未就业的失业人员、不符合领取失业保险金条件的参保失业人员，发放6个月的失业补助金，标准不高于当地失业保险金的80%。对生活困难的失业人员及家庭，按规定及时纳入最低生活保障、临时救助等社会救助范围。（民政部、财政部、人力资源社会保障部按职责分工负责）

（十五）强化困难人员就业援助。动态调整就业困难人员认定标准，及时将受疫情影响人员纳入就业援助范围，确保零就业家庭动态清零。对通过市场渠道确实难以就业的，利用公益性岗位托底安置。开发一批消杀防疫、保洁环卫等临时性公益岗位，根据工作任务和工作时间，给予一定的岗位补贴和社会保险补贴，补贴期限最长不超过6个月，所需资金可从就业补助资金中列支。（财政部、人力资源社会保障部、中国残联按职责分工负责）

（十六）加大对湖北等疫情严重地区就业支持。建立农资点对点保障运输绿色通道，支持湖北省组织农业生产。对湖北高校及湖北籍2020届高校毕业生给予一次性求职创业补贴，湖北省各级事业单位可面向湖北高校及湖北籍高校毕业生开展专项招聘，高校毕业生基层服务项目向湖北省倾斜。做好湖北省疫情解除后的就业工作，加大资金、政策、项目倾斜，开展专场招聘和专项帮扶。维护就业公平，坚决纠正针对疫情严重地区劳动者的就业歧视。（发展改革委、教育部、工业和信息化部、财政部、人力资源社会保障部、农业农村部按职责分工负责）

五、完善职业培训和就业服务

（十七）大规模开展职业技能培训。加大失业人员、农民工等职业技能培训力度，实施农民工等重点群体专项培训，适当延长培训时间。对企业组织职工参加线上线下培训，组织新招用农民工、高校毕业生参加岗前培训的，给予职业培训补贴。动态发布新职业，组织制定急需紧缺职业技能标准。（财政部、人力资源社会保障部按职责分工负责）

（十八）优化就业服务。2020年3月底前开放线上失业登记。推进在线办理就业服务和补贴申领。持续开展线上招聘服务，发挥公共就业服务机构、高校就业指导机构、经营性人力资源服务机构作用，加大岗位信息、职业指导、网上面试等服务供给。对大龄和低技能劳动者，通过电话、短信等方式推送岗位信息，提供求职、应聘等专门服务。低风险地区可有序开展小型专项供需对接活动。优化用工指导服务，鼓励困难企业与职工协商采取调整薪酬、轮岗轮休、灵活安排工作时间等方式稳定岗位，依法规范裁员行为。（教育部、财政部、人力资源社会保障部、全国总工会、全国工商联按职责分工负责）

六、压实就业工作责任

（十九）强化组织领导。各地区各有关部门要在确保疫情防控到位的前提下，毫不放松抓紧抓实抓细稳就业各项工作。县级以上地方政府要加快建立由政府负责人牵头的就业工作领导机制，压实工作责任，细化实化扶持政策。各有关部门要同向发力，围绕稳就业需要，落实完善政策措施，形成工作合力。要健全公共就业服务体系，加强基层公共就业服务能力建设，提升基本公共就业服务水平。（各有关部门和单位、各省级人民政府按职责分工负责）

（二十）加强资金保障。加大就业补助资金和稳岗补贴投入力度。支持市县政府根据稳就业工作推进和政策实施需要，统筹用好就业创业、职业培训、风险储备等方面资金。失业保险基金结余大的地区，要加速稳岗返还、保生活政策落地见效。（财政部、人力资源社会保障部、各省级人民政府按职责分工负责）

（二十一）强化表扬激励。持续开展就业工作表扬激励，完善激励办法，对落实稳就业政策措施工作力度大、促进重点群体就业创业等任务完成较好的地方，及时予以资金支持等方面的表扬激励。（人力资源社会保障部、财政部牵头，各有关部门和单位、各省级人民政府按职责分工负责）

（二十二）加强督促落实。细化分解目标任务，在相关督查工作中将稳就业作为重要内容，重点督促政策服务落地及重点群体就业、资金保障落实等。对不履行促进就业职责，产生严重后果或造成恶劣社会影响的，依法依规严肃问责。完善劳动力调查，研究建立省级调查失业率按月统计发布制度，启动就业岗位调查，做好化解失业风险的政策储备和应对预案。（人力资源社会保障部、统计局牵头，各有关部门和单位、各省级人民政府按职责分工负责）

上述新增补贴政策，受理截止期限为2020年12月31日。各地区各有关部门要抓紧政策实施，发挥政策最大效应，工作中遇到的重要情况和重大问题及时报告国务院。

国务院办公厅

2020年3月18日

1. 民航局：疫情防控期间控制国际客运航班量 民航发〔2020〕11号

各运输航空公司：

当前，防境外疫情输入面临严峻复杂形势。根据国务院疫情联防联控工作要求，为精准施策，加强境外疫情输入风险防范工作，现决定对国际客运航班运行数量进行总控。具体安排如下：

一、以民航局3月12日官网发布的“国际航班信息发布（第5期） ”为基准，每家航空公司在每条航线上的航班量只减不增。

二、各公司航班计划如有变更（包括调减航班计划和更改航班号、班期、机型等），请至迟于实际执行前一周的周三15:00时，报送至邮箱（国内航空公司报送至guojichu@caac.gov.cn、外航报送至 intl@caac.gov.cn），届时未报送，视为无变化。

三、各航空公司国际客运航班计划的批复信息于每周四在民航局官网更新发布。

四、请各航空公司根据上述第三条发布的信息，向民航局运行监控中心申请预先飞行计划。

五、本通知自发布之日起生效，截止日期另行通知。

民航局

2020年3月19日

1. 科技部：印发《关于科技创新支撑复工复产和经济平稳运行的若干措施》 国科发区〔2020〕67号

各省、自治区、直辖市及计划单列市、副省级城市科技厅（委、局），新疆生产建设兵团科技局：

为深入贯彻落实习近平总书记关于统筹推进新冠肺炎疫情防控和经济社会发展工作系列重要讲话精神，按照党中央、国务院一手抓疫情防控、一手抓经济社会发展的统一部署，充分发挥科技创新对当前复工复产和经济平稳运行的支撑保障作用，科技部研究制定了《关于科技创新支撑复工复产和经济平稳运行的若干措施》。现印发给你们，请结合各自实际，抓好贯彻落实。有关进展情况请及时报送科技部。

科技部

2020年3月21日

（此件主动公开）

关于科技创新支撑复工复产和经济平稳运行的若干措施

为深入贯彻落实习近平总书记关于统筹推进新冠肺炎疫情防控和经济社会发展工作系列重要讲话精神，按照党中央、国务院一手抓疫情防控、一手抓经济社会发展的统一部署，充分发挥科技创新对当前复工复产和经济平稳运行的支撑保障作用，现提出以下若干措施。

一、总体要求

科技创新是推动复工复产、保障经济平稳运行、做好“六稳”工作的重要支撑保障。要以习近平总书记有关统筹推进新冠肺炎疫情防控和经济社会发展以及科技创新系列重要讲话精神为根本遵循，紧紧围绕党中央、国务院决策部署，立足科技工作职能定位，坚持底线思维，发挥创新驱动、科技引领作用，形成体系化工作安排。要突出科技工作着力点，聚焦高新区、科技型中小企业和高新技术企业、高新技术产业等科技创新主阵地，以及疫情影响严重地区的发展需求，依靠科技创新解决复工复产、经济平稳运行中的痛点难点堵点。要强化目标导向，以近中期能否尽快取得实效作为根本标准，采取更加精准、可操作的工作举措，确保年内能够取得成效，有力有效对冲疫情影响。要注重发挥好政府与市场两方面作用，深化科技工作“放管服”改革，强化政策引导，激发市场创新活力。要注重调动各方面力量，充分发挥各级科技管理部门、科技人员和各类创新主体等重要作用，形成全国科技系统一盘棋推动复工复产和经济平稳运行的工作局面，为实现全年经济社会发展目标、推动创新驱动高质量发展作出应有贡献。

二、重点举措

（一）启动实施“科技助力经济2020”重点专项。

1.按照“周期短、见效快、程序简捷规范”的原则，通过重点研发计划快速启动实施一批技术创新项目，特别是短期内能见到实效、带动效果明显的技术成果转化落地项目，实施周期两年以内，支持一批优秀科技型企业克服疫情带来的短期困难，对疫情严重地区予以适当倾斜。各地方要结合自身实际加大科技投入，支持科技型企业有序复工复产和经济平稳运行，实现创新发展。

（二）充分发挥国家高新区在推动复工复产中的重要载体作用。

2.各地方要针对低、中、高不同风险等级地区以及不同企业和产业特点，制定差异化、精准化的复工复产举措，指导推动国家高新区分级有序复工复产。推广应用健康监测、智慧物流、远程办公、数字政务等新技术新产品，利用科技手段支撑企业复工复产。建立健全国家高新区复工复产统计监测体系，发挥一站式信息服务平台作用，实现信息共享。

3.加快推动新布局一批国家高新区，优化国家高新区空间布局，通过以升促建，完善科技服务体系，促进高新区高质量发展。进一步完善国家高新区高质量发展评价指标体系和考核办法，建立有进有出的动态调整机制，强化动态管理。

（三）实施科技型中小企业创新发展行动。

4.大力推动科技创新创业，加快壮大科技型中小企业规模，促进高质量就业。加强对“双创”服务机构的考核评估，引导科技企业孵化器、众创空间、大学科技园等打造市场化、专业化、全链条服务平台，提高对科技型中小企业的服务能力。支持龙头骨干企业发挥创新资源、市场渠道、供应链等优势，通过建立专业化众创空间、协同创新共同体等方式带动产业链上下游中小企业同步复工、协同创新。推动重点区域和有条件的地方开展科技创新券跨区域“通用通兑”，根据“双创”服务机构对科技型中小企业的服务绩效予以后补助支持。

5.加大对科技型中小企业的支持力度。利用各种线上线下平台组织开展政策宣讲、在线培训、在线答疑等，面向科技型中小企业宣传解读支持企业复工复产相关援企稳岗、减税免费、社保减免、金融支持等重点政策。会同有关部门研究制定政策措施，加大中央财政对科技型中小企业，特别是科技型小微企业研发活动的绩效奖励，建立部省联合资助机制。协调推动提高科技型中小企业研发费用加计扣除比例。

6.加大国家科技成果转化引导基金对科技型中小企业的融资支持。推动设立支持新药、医疗装备、检测、疫苗等领域的子基金，加快抗疫攻关科研成果的转化和产业化。引导已设子基金加大对疫情重点地区科技型中小企业的支持。会同有关部门研究推动科技成果转化贷款风险补偿试点，引导地方政府和商业银行积极支持科技型中小微企业发展。

（四）加大对高新技术企业的激励引导。

7.研究完善高新技术企业认定管理办法和便利化措施，加强高新技术企业创新能力评价，进一步落实高新技术企业所得税优惠政策，推动更多领域和地区符合条件的企业享受激励政策。

8.开展高新技术企业上市培育行动，加强对高新技术企业的对接服务与培训指导，与证监会、上交所、深交所等加强合作，畅通高新技术企业上市融资渠道。

（五）实施先进技术推广应用“百城百园”行动。

9.围绕“一城一主题、一园一产业”，组织遴选100个左右创新型城市（县市）和100个左右国家高新区、国家农业高新技术产业示范区、国家农业科技园区等，结合地方需求及其优势快速推广应用一批先进技术和科技创新产品。

10.加快国家技术转移体系建设。会同有关部门制定发布关于高校专业化技术转移机构建设发展的实施意见，遴选一批高校开展国家技术转移中心建设试点，面向社会开展技术转移服务。鼓励和推动社会化、市场化技术转移机构发展。建设科技成果信息共享服务平台，完善科技成果动态征集、评估、发布机制。

（六）培育壮大新产业新业态新模式。

11.大力推动关键核心技术攻关，加大5G、人工智能、量子通信、脑科学、工业互联网、重大传染病防治、重大新药、高端医疗器械、新能源、新材料等重大科技项目的实施和支持力度，突破关键核心技术，促进科技成果的转化应用和产业化，培育一批创新型企业和高科技产业，增强经济发展新动能。

12.编制面向智慧医疗、智慧农业、公共卫生、智慧城市、现代食品、生态修复、清洁生产等应用场景的技术目录，在国家高新区、国家新一代人工智能创新发展试验区、国家农业高新技术产业示范区、国家农业科技园区等打造示范应用场景，推动实施一批医疗健康、智能制造、无人配送、在线教育等新兴产业技术项目，引导消费和投资方向。

（七）开展科技人员服务企业专项行动。

13.重点支持拥有创新成果的科技人才加快成果转化应用，推动国家科技人才计划入选人才等率先服务企业，引导地方组织科技人员服务企业。组织创新人才培养示范基地选派“科技专员”，为企业开展科技创新和科学普及服务。搭建人才与企业技术需求信息交互服务平台，推动科技人员与企业精准对接服务，建立人才与企业需求双向互动交流机制。优化外国人来华服务管理，提供出入境便利。加快组织实施疫情防控有关的高端外国专家项目，探索离岸创新、远程合作等智力引进新模式。

14.落实科技人员创新创业各项政策，将科技人员服务企业情况作为职称评审、岗位竞聘等重要内容，对于成效突出的优先推荐申报国家科技人才计划。抓紧落实赋予科研人员职务科技成果所有权或长期使用权试点。

（八）推动科技特派员助力保障春耕生产和扶贫攻坚。

15.精准选派一批科技特派员深入生产一线，重点围绕春耕生产、重大病虫害和动物疫病防治等技术需求加强技术服务。加快推广一批符合实际需求的先进技术成果，编制印发科技手册，组织科技特派员向农民定向推送农业生产政策、春耕备耕技术，指导企业、专业合作社等参与农技推广。

16.开展科技助力脱贫攻坚，统筹推动对建档立卡贫困村科技服务和就业带动全覆盖工作。实施就业行动计划，扎实开展产业扶贫，深入推进消费扶贫。

（九）扩大高校毕业生就业渠道。

17.在国家科技计划支持的项目中，推动高校、科研院所设立科研助理或辅助人员岗位，扩大博士后岗位规模，其劳务费用和有关社保补助按规定从项目经费中列支，支持高校毕业生短期就业。

18.依托国家高新区设立大学生就业实训基地，开展创业培训，建立见习岗位等，吸纳高校毕业生就业。

三、组织实施

（一）切实提高政治站位。各级科技管理部门要从增强“四个意识”、做到“两个维护”的高度，切实把思想、认识和行动统一到党中央、国务院决策部署上来，充分认识科技创新支撑复工复产和经济平稳运行的重要性紧迫性，强化责任担当，积极为高校、科研院所恢复正常科研秩序以及科技园区、科技企业复工复产创造条件、提供保障。

（二）健全协同联动的落实机制。加强中央和地方联动，强化跨部门协同，充分调动科技界、各类创新主体等积极性主动性，形成共同推动复工复产、保障经济平稳运行的工作合力。各地方要结合各自实际，进一步细化实化政策举措，明确责任分工和时间节点，加强协调，加快部署启动，确保年内取得成效。

（三）统筹当前和长远任务部署。立足当前形势和任务要求，及时调整优化重点科技工作部署，加大资源配置、政策措施等重点向支撑复工复产、经济平稳运行倾斜。面向长远，加快推进已经部署的科技项目、平台基地、科技规划、体制改革等重点任务，夯实科技创新能力，引导企业和产业提升创新能力与核心竞争力。

（四）强化政策宣传落实。各级科技管理部门、科技园区要把落实政策作为工作重点，大力开展援企稳岗、复工复产、人才激励等政策的宣传落实，确保各项政策应落实尽落实，充分激发各类创新主体和科技人员创新创造活力。加强与财税、金融、产业、人力资源等相关部门的政策协同，强化各类政策对科技创新的引导支持，争取有新的政策突破。

（五）加大资金投入保障。注重用好存量资金，争取增量资金，通过各级科技计划（专项、基金）等为科技支撑复工复产、保障经济平稳运行提供保障。加大对湖北等疫情影响较大地区的支持力度，加大科技创新资源援助力度。拓展各类资金投入渠道，引导社会资本、金融投资等加大投入。

（六）注重奖惩并重。对于在支撑复工复产、保障经济平稳运行中作出突出贡献的单位和个人，在各级科技计划项目申报、表彰奖励、平台建设等方面给予积极支持。总结梳理一批在复工复产、支撑经济发展方面成效显著的地方、园区和企业典型案例，做好宣传推广。对于敷衍塞责、不担当不作为的按有关规定予以惩戒。

1. 商务部 财政部：用好内外贸专项资金支持稳外贸稳外资促消费工作 商办财函〔2020〕98号

为深入贯彻习近平总书记关于统筹推进新冠肺炎疫情防控和经济社会发展重要指示精神，落实党中央、国务院决策部署，统筹推进疫情防控和商务发展相关工作，充分发挥中央财政资金效益，把各地商务发展的巨大潜力和强大动能充分释放出来，更好支持应对疫情、做好稳外贸稳外资促消费工作，现就有关事项通知如下：

一、用好外经贸发展专项资金，全力支持稳住外贸外资基本盘

对受疫情影响较大的外经贸领域予以倾斜，帮助企业应对疫情影响，促进外经贸高质量发展。一是全力稳住外贸基本盘。对有订单有市场的企业确因疫情增加的相关费用给予适当支持。在深耕细作传统市场的基础上，加快培育新的增长点，支持企业开拓多元化市场。鼓励服务贸易创新发展。在符合相关规定前提下，引导加大信贷保险支持，以政银保合作等方式加大贸易融资支持。加大对中小外贸企业的扶持力度，在同等条件下，适度向中小外贸企业，特别是向受疫情影响较大的中小外贸企业倾斜。二是全力稳住外资基本盘。充分发挥对外开放平台引资作用，支持国家级经开区、自贸试验区、边境经济合作区和跨境经济合作区建设，打造国际合作新载体，推进边合区、跨合区“小组团”滚动开发。健全外商投资促进公共服务体系，建立重点企业联系制度，创新企业服务方式。三是稳住产业链供应链。引导企业有序开展对外投资合作。支持中西部和东北地区承接加工贸易，优化国内产业布局。

二、用好服务业发展资金，加大支持促进国内消费

一是用好电子商务进农村综合示范资金，重点支持农产品进城，兼顾工业品下乡，对承担疫情防控相关重要物资保供任务，且工作突出的电商、物流、商贸流通等企业，在同等条件下予以适当倾斜。二是用好流通领域供应链体系建设资金，强化生活必需消费品供应链保障功能，对结余两年以上的专项资金统筹使用，可用于支持疫情防控相关生活必需消费品的保供支出，在同等条件下向相关项目适度倾斜。三是用好农产品供应链体系建设资金，安排一定比例资金用于支持农产品保供工作，包括农产品流通企业承担保供任务时发生的运费、租金、保供储备、冷链、防疫以及供应链中断恢复过程中发生的相关费用补贴。具体按照《商务部办公厅财政部办公厅关于疫情防控期间进一步做好农商互联工作的紧急通知》（商办建函[2020]53号）相关要求执行。

三、严格规范资金管理，不断提升资金使用绩效

各地财政、商务主管部门要进一步加强沟通配合，积极推进内外贸专项资金预算执行。一要抓紧安排已经下达的2020年专项资金，认真对照专项资金管理办法和相关工作通知，结合本地疫情防控和经济社会发展具体情况，制定修订资金使用细则，用好地方配套资金，带动社会资本，加大力度支持稳外贸稳外资促消费工作。二要统筹盘活结余资金，根据《财政部关于推进地方盘活财政存量资金有关事项的通知》（财预〔2015〕15号）要求，在原定支持事项规定的使用范围内，结合稳外贸稳外资促消费工作需要，因地制宜确定具体支持方向，建立完善项目储备库，做好资金与项目的衔接，加快结余资金执行进度。三要加强监督管理，有效防范风险，资金安排应避免与其它政策平台、资金渠道等交叉重复。

各地财政、商务主管部门要以习近平新时代中国特色社会主义思想为指导，增强“四个意识”、坚定“四个自信”、做到“两个维护”，坚决贯彻落实党中央、国务院决策部署，提高站位，统筹协作，强化担当，完善组织领导，创新工作方法，用足用好中央财政内外贸专项资金政策，全力支持稳住外贸外资基本盘，促进国内消费，为实现全年经济社会发展目标任务作出积极贡献。

商务部办公厅 财政部办公厅

2020年3月6日

1. 公安部推出新冠肺炎疫情防控期间治安管理便民利企15项措施

为认真贯彻落实习近平总书记重要指示精神，根据中央关于统筹推进疫情防控和经济社会发展的总体部署，公安部推出了新冠肺炎疫情防控期间治安管理便民利企15项措施，分别是：

一、为复工复产群众办理居民身份证、居住证、居民户口簿开通“绿色通道”，优先受理、优先制发。

二、群众所持居民身份证有效期满或丢失的，可在现居住地公安派出所就近换领、补领。

三、对于申请跨省办理居民身份证的，群众经书面承诺，可免提交在当地就业、就学、居住的证明材料，公安机关核准办证人员身份后，即予以办理。

四、对在疫情防控期间到期的户口迁移证件，有效期限延长至疫情结束后30日。

五、对申领居住证数量较多的企事业单位，公安派出所上门采集信息、集中受理制作。

六、对在疫情防控期间到期的居住证，有效期限延长至疫情结束后30日，30日内补办签注手续的居住时限连续计算。

七、对急需办理户政业务的群众，公安户政部门和派出所通过网上服务平台、自助服务设备、电话等方式，提供政策咨询、信息查询和预约办理服务。

八、运用公安基础业务资源，协助企业核实员工信息，为疫情防控、人员隔离观察提供信息支撑，为企业及员工安全复工复产提供精准指导。

九、推动落实重点企业“一企一警”“一企一策”措施，深入了解掌握企业需求，组织摸排企业受困情况，帮助解决复工复产难题。

十、突出重点企业、重点园区内部及周边，指导督促落实内部安全防范措施，协调加大周边巡防力度，做到涉企警情快速处置、涉企案件快速侦办，积极为企业复工复产营造良好环境。

十一、保安从业单位在疫情期间招聘保安员，可先行招录上岗，同时在30日内组织培训申领保安员证。

十二、保安员考试推行网上报名、网上审核和预约考试，公安机关核发保安员证通过邮寄送达保安从业单位。

十三、指导保安企业加强医院、金融单位、企业、重要基础设施等重点单位、场所保安服务，积极服务疫情防控和单位复工复产。

十四、推行新开办企业公章刻制备案多证合一、一网通办，公章刻制经营单位网上承办、1个工作日内完成刻制，可通过邮寄等方式交付公章。

十五、有条件的地方推行旅馆业、公章刻制业等行业网上审批、备案，提高审批备案效率，为企业提供便捷服务。

下一步，公安部将细化工作部署、加强督导检查、开展评估问效，确保15项便民利企措施落实落地、取得实效，更好地为企业、群众复工复产创造便利条件，更好地服务经济社会发展，为坚决打赢疫情防控的人民战争、总体战、阻击战创造良好的社会治安环境。

2020.3.23

1. 住建部 扶贫办：统筹做好疫情防控和脱贫攻坚保障贫困户住房安全相关工作 建办村〔2020〕6号

各省、自治区、直辖市住房和城乡建设厅（住房和城乡建设委，住房和城乡建设管委）、扶贫办（局），新疆生产建设兵团住房和城乡建设局、扶贫办：

为贯彻落实习近平总书记在决战决胜脱贫攻坚座谈会及统筹推进新冠肺炎疫情防控和经济社会发展工作部署会上的重要讲话精神，深入落实党中央、国务院脱贫攻坚决策部署，确保如期实现贫困户住房安全有保障目标任务，现就统筹抓好新冠肺炎疫情防控和脱贫攻坚农村危房改造相关工作通知如下。

一、克服新冠肺炎疫情影响，加快推进农村危房改造任务扫尾

（一）明确农村危房改造任务及完成时限。地方各级住房和城乡建设部门要进一步加强与扶贫部门协作，对2019年已下达尚未竣工的存量任务以及“回头看”排查出的新增任务等农村危房改造扫尾任务逐户建立台账，统筹用好提前下达的2020年中央财政农村危房改造补助资金，倒排工期，压实责任，确保所有建档立卡贫困户需改造的危房2020年6月底前全部竣工。

（二）分区分级推进农村危房改造工程复工。要按照分区分级精准复工复产的工作要求，根据不同地区的新冠肺炎疫情防控工作情况，指导所辖地区有序推进农村危房改造复工。疫情严重的地区，在重点抓好疫情防控的同时，做好人工、建筑材料等准备，根据疫情防控形势逐步实施改造。没有疫情或疫情较轻的地区，要集中精力加快实施改造。相关省份要加强对“三区三州”等深度贫困地区和受疫情影响严重的贫困地区工作支持指导力度，加大资金投入，定期调研督导。

（三）做好农村危房改造过程新冠肺炎疫情防控。要做好农村危房改造施工人员的防护知识普及，增强自我保护意识。根据本地区疫情防控形势督促农村危房改造施工人员使用必要的防护用品，保障身体健康安全。加强对农村危房改造施工人员的健康状况监测，发现疫情应及时采取应对措施并向当地有关部门报告。

二、对标脱贫攻坚普查，完善档案信息管理

（四）完善信息录入与管理。在国务院扶贫办提供建档立卡贫困户信息的基础上，住房和城乡建设部对脱贫攻坚三年行动农户档案信息检索系统（以下简称信息检索系统）进行升级。省级住房和城乡建设部门要指导县级住房和城乡建设部门对照信息检索系统中的建档立卡贫困户信息，逐户梳理其住房安全保障方式和住房安全有保障的认定结果，并于2020年6月底前完成相关信息录入工作。同时，县级住房和城乡建设部门要加快农村危房改造农户档案的信息录入工作，对已录入信息认真校核完善，确保农村危房改造农户档案信息真实、完整。

（五）加强信息互通与共享。地方各级住房和城乡建设部门和扶贫部门要加强信息系统数据的互联互通，确保所掌握的建档立卡贫困户住房安全保障情况口径一致。地方各级住房和城乡建设部门要定期将本地区农村危房改造进展情况及时提供给本级扶贫部门，扶贫部门要及时更新完善扶贫开发信息系统有关数据，并将更新完善后的数据及时反馈住房和城乡建设部门。

三、抓好问题整改，巩固脱贫成效

（六）抓好问题整改。要对照中央脱贫攻坚专项巡视“回头看”、2019年扶贫成效考核以及各地脱贫攻坚大排查等发现的问题，逐项建立台账，明确整改方向，落实整改责任，按照“立行立改、边查边改”的原则，持续深入抓好危房改造质量不高、补助资金拨付缓慢、项目管理不规范等问题整改工作。有关省份要通过实地走访、入户暗访、个别抽查等方式，加大督导力度，确保问题整改到位。

（七）巩固脱贫成效。要结合问题整改，深入分析农村危房改造工作责任落实、监督管理、制度执行等方面存在的突出问题，举一反三，完善相关政策和工作机制，推动建立长效机制。用好漠视侵害群众利益专项整治工作成果，把着力解决群众最关心最直接最现实的利益问题作为一项长期工作常抓不懈。对贫困户住房安全保障情况实施动态监测，及时跟踪并解决出现的新问题，根据当地实际研究建立防止返贫的工作机制。

从2020年4月起，省级住房和城乡建设部门要联合扶贫部门每月6日前向住房和城乡建设部村镇建设司和国务院扶贫办规划财务司报送上月末建档立卡贫困户危房改造工程进度情况。

中华人民共和国住房和城乡建设部办公厅

国务院扶贫开发领导小组办公室综合司

2020年3月16日

1. 农业农村部：印发《2020年国家产地水产品兽药残留监控计划》等3个计划 农渔发〔2020〕4号

有关省、自治区、直辖市及计划单列市农业农村（农牧）厅（局、委），新疆生产建设兵团农业农村局，福建省海洋与渔业局、青岛市海洋发展局，中国水产科学研究院、全国水产技术推广总站，有关水产品质检机构：

为落实党中央、国务院关于应对新冠肺炎疫情、做好农产品稳产保供、抓好“菜篮子”产品生产等工作部署，加强水产养殖用兽药及其他投入品使用的监督管理，提升养殖水产品质量安全水平，加快推进水产养殖业绿色发展，根据《中华人民共和国渔业法》《中华人民共和国农产品质量安全法》《兽药管理条例》等法律法规规定，我部制定了《2020年国家产地水产品兽药残留监控计划》《2020年海水贝类产品卫生监测和生产区域划型计划》《2020年水产养殖用兽药及其他投入品安全隐患排查计划》。现印发给你们，请遵照执行。

农业农村部

2020年3月11日

1. 民政部：印发《志愿服务组织和志愿者参与疫情防控指引》 民办发〔2020〕11号

各省、自治区、直辖市民政厅（局），新疆生产建设兵团民政局：

当前，新冠肺炎疫情防控形势依然严峻复杂，任务艰巨繁重。各级民政部门要认真贯彻落实习近平总书记系列重要讲话和指示精神，保持头脑清醒，慎终如始，再接再厉，善作善成，继续动员志愿服务组织和志愿者积极有序参与疫情防控工作。

现将《志愿服务组织和志愿者参与疫情防控指引》印发给你们，请结合实际引导广大志愿服务组织和志愿者继续投身疫情防控，不麻痹、不厌战、不松劲，为全面打赢新冠肺炎疫情防控的人民战争、总体战、阻击战贡献力量。

民政部办公厅

2020年3月17日

志愿服务组织和志愿者参与疫情防控指引

新冠肺炎疫情发生以来，广大志愿服务组织和志愿者积极响应中央号召，踊跃参与疫情防控工作，成为抗击疫情的重要力量。根据当前疫情防控新形势、新要求，对志愿服务组织和志愿者参与疫情防控有关工作提出如下指引。

一、做好个人防护

1.及时关注疫情发展趋势，了解掌握最新防控政策措施，提高防控意识。

2.注意学习新冠肺炎病毒潜伏时间、传播特点、感染症状、防护措施等知识，保护自己，服务他人。

3.注意作息饮食科学，避免过度疲劳，提高免疫力，在保证自身安全的情况下参与志愿服务。

4.外出佩戴医用口罩，勤洗手，保持良好卫生习惯，参加疫情防控有关工作时采取符合要求的防护措施。

5.出现发热、干咳、乏力等症状及时自我隔离、就医。

6.志愿服务组织要加强对志愿者的服务，合理设置志愿服务岗位和服务时长，加强对志愿者的心理支持，不让志愿者带病上岗、长时间高负荷服务，确保志愿者身心健康。

7.志愿服务组织安排志愿者参与可能发生人身危险的志愿服务活动前，应当为志愿者购买相应的人身意外伤害保险。

二、有序参与服务

8.遵循志愿服务宗旨和自愿、无偿、平等、诚信、合法的原则，规范开展疫情防控志愿服务。

9.服从当地党委和政府的统一部署和调度，合理调配资源和力量。

10.坚持组织化原则，鼓励有意参与疫情防控的志愿者与志愿服务组织或所在单位、社区联系并报名，有序参与志愿服务。

11.坚持文明服务，遵守社会公德，尊重个人隐私，反对歧视行为，促进社会融合。

12.能够通过线上提供服务的，优先选择线上服务，降低安全风险。需要开展线下服务的，志愿服务组织应当向志愿者提示可能发生的风险，并提供防护措施，加强安全教育。

13.坚持就近就便服务，合理确定服务区域。鼓励志愿者优先参与所在街道、社区的疫情防控。在疫情应急响应终止前，不跨区域开展非必要的线下服务，不开展人员聚集性志愿服务活动。

三、坚持需求导向

14.密切关注群众生产生活中迫切需要解决的实际问题，明确阶段性工作重点，结合自身能力确定服务对象和服务内容。

15.注重关心一线医务人员、社区工作者、病亡者家属、有特殊困难人员、流动人口等重点群体。

16.重点开展社区疫情防控、医疗救治辅助、生活用品配送、一线医务人员及家属关爱、确诊患者及家属情绪疏导、老人及儿童陪伴呵护、困难群众帮扶、心理援助、复工复产防疫宣传等志愿服务。

17.志愿服务组织根据需要积极招募具备医护知识、应急救援能力以及心理咨询、社会工作、文化教育、网络技术、交通运输等方面专长的志愿者，开展专业志愿服务。

四、协助医疗救治

18.根据需要在新冠肺炎定点医院和集中隔离点开展专业医护志愿服务，做好帮助患者适应医疗环境、提供生活照顾、协助进行患者管理、辅助开展护理服务等工作。志愿者应当具有相关职业资格或者具备相关专业知识。

19.开展医务人员支持保障，为定点医院和集中隔离点的医务人员提供生活照护和个性化支持，包括上下班护送、送餐、代购代送、代修理等。

20.开展病患健康指导和心理服务，为新冠肺炎患者、集中隔离医学观察人员、居家观察人员提供医学咨询、心理疏导、情绪支持、压力舒缓等服务。

21.健康指导和心理服务由医务人员、心理工作者、社会工作者等专业志愿者提供。优先采用网络、电话等线上服务、远程服务的方式开展。

五、参与社区防控

22.协助社区开展疫情排查工作，包括电话调查、入户访问、测量体温等。

23.协助社区落实人员管控措施，配合做好返回人员的信息登记、居家隔离观察等防控工作，对人员出入进行检查等。

24.协助社区工作者、社会工作者、心理工作者为居民提供个别疏导、心态调适，消除其负面情绪。

25.协助做好社区消毒杀菌、卫生清洁等工作。

26.服务中如果遇到不配合人员，应第一时间与社区联系并说明情况，由社区工作者协调解决。

六、提供便民服务

27.为抗疫一线医务人员、社区工作者家庭提供关爱服务，为家中老人、孩子提供接送就医、生活照料、心理疏导、课业辅导、物品代购等服务。

28.为社区隔离人员提供关爱服务，协助社区收集评估隔离人员需求，提供健康咨询、情绪支持、物品代购代送、生活垃圾处理等服务。

29.为出院患者及其家属、结束留观人员及其家属、居家隔离人员及其家属、来自疫情严重地区人员等开展与社区其他居民之间关系调适服务，倡导不排斥、不歧视的社区氛围。

七、关爱特殊群体

30.协助社区对受疫情影响的特殊困难群体进行摸排，并及时提供关爱服务。

31.将因疫情防控在家隔离的孤寡老人、因家人被隔离或者收治而无人照料的老年人和未成年人、困难儿童、特困人员、残疾人等特殊群体作为重点服务对象。

32.采取电话询问、网络联系、上门走访慰问等多种方式，详细掌握特殊困难群体的生活状况和服务需求，发现有生活困难的，第一时间向社区报告。

33.协助链接政府、社会组织等多方面资源，为困难群体提供必要救助或相关服务，保障困难群众生活，防止冲击社会道德底线的事件发生。

八、参与慈善捐赠

34.协助慈善组织、红十字会做好慈善捐赠政策解读、捐赠引导等工作。

35.协助慈善组织、红十字会做好捐赠物资的接收、清点、整理、分发、转运等工作。

九、做好防疫宣传

36.开展疫情防治健康知识宣传。充分利用多种手段，有针对性地开展新冠肺炎防控知识宣传，使群众充分了解疫情变化、防控知识，引导群众提高文明素养和防护意识，配合党委和政府的防控工作。

37.向服务对象提供党委和政府疫情防控要求、疫情防控动态、公共政策调整等信息服务，引导群众关注权威发布，不信谣、不传谣，减轻群众忧虑和恐慌心理，营造积极乐观、守望相助的良好氛围，增强战胜疫情信心。

十、加强协作配合

38.志愿服务组织和志愿者应当强化组织性、协同性，注重与各类单位和组织，如村（居）民委员会、医疗机构、物业公司、有关生产销售企业、新闻媒体、社会工作服务机构以及其他参与疫情防控的社会组织等保持密切联系，加强协作配合，形成疫情防控合力。

39.充分运用好网络新媒体，发挥线上服务人身安全风险低、信息传播速度快等优势，积极开展需求评估、资源链接、心理咨询、在线培训指导、防疫知识宣传等线上志愿服务；也可根据线下的实际需要，在确保安全的前提下，围绕医疗救治、社区防控、生活帮扶、重点区域等开展线下志愿服务，构建立体化疫情防控志愿服务网络。

40.注意开展经验交流，在实践中学习，在交流中改进，及时总结提炼，形成服务范本，为其他志愿服务组织和志愿者提供借鉴。

1. 卫健委：印发公众科学戴口罩指引 联防联控机制发〔2020〕33号

各省、自治区、直辖市及新疆生产建设兵团应对新型冠状病毒肺炎疫情联防联控机制（领导小组、指挥部）：

根据当前防控形势和全面有序恢复正常生产生活秩序需要，我们组织编制了《公众科学戴口罩指引》。本指引从普通公众、特定场所人员、重点人员以及职业暴露人员进行分类，并对不同场景下戴口罩提出科学建议。现印发给你们，请参照执行。

国务院应对新型冠状病毒肺炎疫情联防联控机制

（代章）

2020年3月17日

（信息公开形式：主动公开）

公众科学戴口罩指引

科学戴口罩，对于新冠肺炎、流感等呼吸道传染病具有预防作用，既保护自己，又有益于公众健康。目前，在抗击新冠肺炎疫情形势下，为引导公众科学戴口罩，有效防控疫情，保护公众健康，特提出以下指引。

一、普通公众

（一）居家、户外，无人员聚集、通风良好。

防护建议：不戴口罩。

（二）处于人员密集场所，如办公、购物、餐厅、会议室、车间等；或乘坐厢式电梯、公共交通工具等。

防护建议：在中、低风险地区，应随身备用口罩（一次性使用医用口罩或医用外科口罩），在与其他人近距离接触（小于等于1米）时戴口罩。在高风险地区，戴一次性使用医用口罩。

（三）对于咳嗽或打喷嚏等感冒症状者。

防护建议：戴一次性使用医用口罩或医用外科口罩。

（四）对于与居家隔离、出院康复人员共同生活的人员。

防护建议：戴一次性使用医用口罩或医用外科口罩。

二、特定场所人员

（一）处于人员密集的医院、汽车站、火车站、地铁站、机场、超市、餐馆、公共交通工具以及社区和单位进出口等场所。

防护建议：在中、低风险地区，工作人员戴一次性使用医用口罩或医用外科口罩。在高风险地区，工作人员戴医用外科口罩或符合KN95/N95及以上级别的防护口罩。

（二）在监狱、养老院、福利院、精神卫生医疗机构，以及学校的教室、工地宿舍等人员密集场所。

防护建议：在中、低风险地区，日常应随身备用口罩（一次性使用医用口罩或医用外科口罩），在人员聚集或与其他人近距离接触（小于等于1米）时戴口罩。在高风险地区，工作人员戴医用外科口罩或符合KN95/N95及以上级别的防护口罩；其他人员戴一次性使用医用口罩。

三、重点人员

新型冠状病毒肺炎疑似病例、确诊病例和无症状感染者；新型冠状病毒肺炎密切接触者；入境人员(从入境开始到隔离结束)。

防护建议：戴医用外科口罩或无呼气阀符合KN95/N95及以上级别的防护口罩。

四、职业暴露人员

（一）普通门诊、病房等医务人员；低风险地区医疗机构急诊医务人员；从事疫情防控相关的行政管理人员、警察、保安、保洁等。

防护建议：戴医用外科口罩。

（二）在新型冠状病毒肺炎确诊病例、疑似病例患者的病房、ICU工作的人员；指定医疗机构发热门诊的医务人员；中、高风险地区医疗机构急诊科的医务人员；流行病学调查、实验室检测、环境消毒人员；转运确诊和疑似病例人员。

防护建议：戴医用防护口罩。

（三）从事呼吸道标本采集的操作人员；进行新型冠状病毒肺炎患者气管切开、气管插管、气管镜检查、吸痰、心肺复苏操作，或肺移植手术、病理解剖的工作人员。

防护建议：头罩式（或全面型）动力送风过滤式呼吸防护器，或半面型动力送风过滤式呼吸防护器加戴护目镜或全面屏；两种呼吸防护器均需选用P100防颗粒物过滤元件，过滤元件不可重复使用，防护器具消毒后使用。

五、使用注意事项

（一）呼吸防护用品包括口罩和面具，佩戴前、脱除后应洗手。

（二）佩戴口罩时注意正反和上下，口罩应遮盖口鼻，调整鼻夹至贴合面部。

（三）佩戴过程中避免用手触摸口罩内外侧，应通过摘取两端线绳脱去口罩。

（四）佩戴多个口罩不能有效增加防护效果，反而增加呼吸阻力，并可能破坏密合性。

（五）各种对口罩的清洗、消毒等措施均无证据证明其有效性。

（六）一次性使用医用口罩和医用外科口罩均为限次使用，累计使用不超过8小时。职业暴露人员使用口罩不超过4小时，不可重复使用。

1. 卫健委：印发新冠肺炎疫情心理疏导工作方案 联防联控机制发〔2020〕34号

各省、自治区、直辖市及新疆生产建设兵团应对新型冠状病毒肺炎疫情联防联控机制（领导小组、指挥部）：

目前，我国新冠肺炎疫情防控形势发生积极向好变化，不同人群心理状况也随之变化。为贯彻落实中央领导同志指示精神，进一步加强重点人群心理疏导和心理干预，现将《新冠肺炎疫情心理疏导工作方案》印发给你们，请认真贯彻落实。

国务院应对新型冠状病毒

肺炎疫情联防联控机制

（代章）

2020年3月18日

（信息公开形式：主动公开）

新冠肺炎疫情心理疏导工作方案

新冠肺炎疫情发生后，为指导各地做好不同人群心理危机干预工作，1月26日联防联控工作机制印发《新型冠状病毒感染的肺炎疫情紧急心理危机干预指导原则》。目前，我国新冠肺炎疫情防控形势发生积极向好变化，不同人群心理状况也随之变化。为进一步加强重点人群心理疏导和心理干预，制定本方案。

一、工作目标

针对患者及其家属、病亡者家属、一线工作人员等重点人群，开展心理疏导、心理干预等心理服务，维护公众心理健康，促进社会和谐稳定。

二、工作措施

（一）加强患者及家属的心理疏导。

各地医疗卫生机构要关注患者的心理健康状况，由心理治疗师、社会工作者等提供心理疏导服务。对于心理健康状况较差的患者，及时进行评估干预，必要时请精神科医师会诊。在患者出院时，将使用精神科药物干预患者的有关资料转交到隔离点或患者所在地区的基层医疗卫生机构，确保治疗的延续性。（卫生健康部门负责）

湖北武汉等受疫情影响严重地区的区县新冠肺炎防控指挥部要组建由精神卫生和心理健康专业人员、社会工作者等组成的服务队，对治愈隔离患者提供心理服务，重点为焦虑抑郁、失眠、创伤后应激障碍等患者提供心理疏导，及时识别自伤、自杀、攻击或其他精神病性症状人群，由精神科医生会诊或转入精神卫生医疗机构治疗。（卫生健康、民政部门负责）

城乡社区工作者要加强对患者家属及治愈归家患者的人文关怀，帮助患者恢复正常生活。引导社区居民正确对待患者及家属，避免歧视。通过组织动员社会工作者和专业志愿者等力量，为城乡社区有心理问题的治愈患者及家属提供精神慰籍、心理抚慰、社会融入等服务，及时识别有严重心理行为问题的个体，并向精神卫生医疗机构转介。（民政部门牵头，卫生健康部门配合）

（二）做好病亡者家属关心关爱及心理疏导。各地民政、卫生健康、工会、共青团、妇联、残联等部门要加强对病亡者家属的关心、关爱，组织社会工作服务机构、精神卫生医疗机构等为其提供社会支持、心理干预服务，引导其宣泄哀伤情绪，帮助其顺利度过哀伤期，恢复正常生活。病亡者所在单位、社区等应当建立关爱帮扶小组，为有需求的病亡者家属提供关爱帮扶和心理支持。对出现严重心理问题的家属，协助其到当地精神卫生机构就诊。湖北省、武汉市等疫情严重地区要做好骨灰分批交接安葬的工作预案，强化人文关怀，指导通过网上等方式寄托哀思，避免人群集聚。（民政、卫生健康、工会、共青团、妇联、残联等部门负责）

（三）强化低保对象、特困人员、特殊困难老年人、困境儿童、流浪乞讨人员、残疾人等心理支持。民政、妇联、残联等部门要动员和引导慈善组织、社会工作服务机构、志愿服务组织等社会力量，为低保对象、特困人员、特殊困难老年人、困境儿童、流浪乞讨人员、残疾人等群体提供生活救助和关爱帮扶。湖北省、武汉市民政、妇联、残联等部门要摸清低保对象、特困人员、孤寡老人、孤儿、困境儿童、残疾人等群体的具体情况，把日常生活服务和保障与心理服务相结合，了解工作对象心理特点，针对性地提供心理支持或协助寻求心理专业人员帮助。（民政、妇联、残联等部门负责）

（四）做好疫情防控医务工作者心理服务。各地卫生健康部门要充分利用当地精神卫生、心理健康及社会工作服务资源，为医务工作者提供心理服务。对一线医务人员加强关心关爱，在轮休期间由精神卫生专业人员组织开展放松训练等活动。对出现明显应激反应的医务人员，要进行针对性的个体心理治疗或适当的药物干预。湖北省、武汉市要充分发挥当地精神卫生医疗机构和援鄂心理救援队的作用，通过讲座、团体辅导、个体咨询、网络平台、心理热线等方式，为医务人员提供心理服务。（卫生健康部门负责）

（五）加强公安民警等一线工作人员心理疏导。公安、司法行政、民政、工会等部门要做好公安民警（辅警）、司法行政干警、社区工作者、基层工作人员、下沉干部等值班、轮班安排，利用本系统资源或社会资源，对一线工作人员提供心理服务，对有严重心理行为问题的个体进行主动干预。为因疫情防控殉职的民警（辅警）、社区工作者、基层工作人员等的家属加强心理疏导。湖北省、武汉市要发挥各类志愿者作用，注意识别、发现有心理需求或可能有严重心理问题的个体，及时通过社区干部联系心理服务专业人员和社会工作者进行评估、干预。（政法委、公安、司法行政、民政、工会等部门负责）

（六）加强特殊人群管理服务。各地要建立健全基层综合管理小组，加强公安监所被监管人员、服刑人员、社区矫正对象、刑满释放人员、强制隔离戒毒人员、强制隔离戒毒解戒人员、社区戒毒社区康复人员、参加戒毒药物维持治疗人员和自愿戒毒人员、易肇事肇祸严重精神障碍患者等特殊人群管理服务。信访部门要畅通诉求表达渠道，引导利益受损群众理性反映诉求，做好思想疏导工作。湖北省、武汉市要做好滞留在鄂、在汉人员特别是生活困难、经济损失较大人群的心理服务。对有典型心理行为问题的利益受损群体，引入心理服务工作人员配合进行矛盾处理或纠纷化解，预防极端事件发生。（政法委、公安、民政、司法行政、卫生健康、信访部门负责）

（七）积极开展广大群众心理疏导。各地宣传、广电部门要发挥各类媒体作用，做好心理健康知识普及和国家政策解读，及时疏导广大群众因长期隔离带来的负面情绪，营造强信心、暖人心、聚民心的社会氛围。教育、工会、共青团、妇联等部门要利用各类线上、线下心理服务资源，加强对学生、机关企事业单位员工、妇女儿童等不同人群的心理疏导。湖北省、武汉市要将心理服务融入网格化管理工作，对仍有确诊病例的小区，安排心理服务专业人员、社会工作者或志愿者，及时疏导居民长期封闭管理产生的负面情绪。（宣传、广电、教育、工会、共青团、妇联负责）

三、保障措施

（一）各地要将新冠肺炎疫情心理服务纳入疫情防控整体工作部署，建立党政领导、部门协作、社会动员的工作机制。

（二）各地结合实际情况，对心理服务提供资金支持。支持精神卫生医疗机构、社会工作服务机构、社会心理服务机构等参与心理服务工作。

（三）各地要对心理服务工作者、社会工作者、专业志愿者等开展培训，提升服务水平，恪守职业道德，保护服务对象个人隐私。

（四）各地卫生健康部门要对不同人群心理健康状况进行评估，及时调整心理服务工作重点。通过委托第三方等方式对有关机构心理服务情况及效果进行评价。

1. 商务部：推广疫情防控时期保障生活必需品供应典型做法

各省、自治区、直辖市、计划单列市及新疆生产建设兵团商务主管部门：

新冠肺炎疫情发生以来，各地采取封闭管理、隔离管控、限流限行等防控措施，给生活必需品供应带来了新的挑战。针对新情况、新问题，各地商务主管部门认真贯彻落实习近平总书记重要讲话精神和党中央、国务院决策部署要求，主动协调相关部门、积极组织流通企业、联系对接街道社区，千方百计保障居民生活必需品供给，在保供网络和服务模式上做了一些行之有效的创新探索，取得了较好的实践效果。我们梳理总结了一些具有典型意义的做法（见附件），供各地学习借鉴。

当前疫情防控到了最吃劲的关键阶段，做好生活必需品供应保障工作，是打赢疫情防控阻击战的支撑。各地商务主管部门要认真贯彻落实中央决策部署要求，统筹做好疫情防控和生活必需品供应保障工作。要立足本地实际，创新思路，分类施策，狠抓落实，进一步提高保障供应的能力和水平，更好地满足特殊时期人民群众基本生活需要。各地在实践中积累的好经验、好做法，要及时报商务部（市场建设司），我们将予以积极宣传推广。

附件：疫情防控时期保障生活必需品供应典型做法

商务部办公厅

2020年2月25日

1. 卫健委：统筹做好新冠肺炎疫情防控全面有序开展预防接种工作 国卫办疾控函〔2020〕235号

各省、自治区、直辖市及新疆生产建设兵团卫生健康委：

为指导各地根据疫情形势变化，安全、及时地开展预防接种，科学、有效做好疫苗针对传染病防控工作，现就当前形势下全面有序开展预防接种工作提出如下要求：

一、科学统筹新冠肺炎疫情防控和预防接种工作。前期，部分地区根据新冠肺炎疫情防控需要，暂停或调整了当地预防接种工作安排。当前我国新冠肺炎疫情防控呈现积极向好态势，各地复工复产正逐步有序展开。各级卫生健康部门要结合实际，综合分析本地区疫苗针对传染病防控形势，统筹做好新冠肺炎疫情防控和预防接种工作。湖北以外地区要周密安排，全面有序恢复正常的预防接种秩序。湖北省要根据新冠肺炎疫情防控工作的统一要求，科学合理安排预防接种有关工作。各地要认真做好因新冠肺炎疫情影响造成监护缺失适龄儿童的预防接种工作。

二、湖北以外地区要全面有序开展预防接种工作。各省级卫生健康行政部门要认真落实国务院联防联控机制《关于科学防治精准施策分区分级做好新冠肺炎疫情防控工作的指导意见》要求，根据本省域内划分的新冠肺炎疫情风险等级，指导相关县区预防接种门诊科学合理制订并落实有关防控措施，全面有序开展预防接种工作。各地疾控机构要指导预防接种门诊创新服务方式，通过网络或电话开展预约接种，优先安排因疫情防控未及时接种的国家免疫规划疫苗补种及其他疫苗后续剂次接种。接种门诊要科学确定每日接种量，合理安排接种时段，尽可能减少接种前等待时间，避免接种后留观人员聚集。要加强接种场所内部环境消毒，开窗通风，引导受种者及陪护人员做好个人防护。对于新冠肺炎疫情风险评估等级较高的地区，预防接种门诊要严格落实环境卫生消毒、出入人员测温、健康线设置等措施，预防接种工作人员要做好个人防护，严防机构内感染事件发生。

三、湖北省要在做好疫情防控的基础上做好预防接种相关工作。湖北省卫生健康委要根据疫情防控形势变化动态调整本地区预防接种工作计划，确保社会生活恢复后各类预防接种门诊能迅速恢复正常的预防接种服务。各级医疗机构要在严格落实当地新冠肺炎疫情防控各项措施的前提下，按照国家免疫规划程序优先安排好新生儿首针乙肝疫苗和卡介苗接种。每个县（市、区）要确保有定点狂犬病暴露预防处置门诊开放，用于开展狂犬病主动及被动免疫、外伤处置、破伤风疫苗接种等服务。

国家卫生健康委办公厅

2020年3月15日

1. 中组部 人社部：应对新冠肺炎疫情影响做好事业单位公开招聘高校毕业生工作 人社厅发〔2020〕27号

各省、自治区、直辖市及新疆生产建设兵团党委组织部、政府人力资源社会保障厅（局），中央和国家机关各部委、各人民团体组织人事部门：

习近平总书记在统筹推进新冠肺炎疫情防控和经济社会发展工作部署会议上强调，“要注重高校毕业生就业工作，统筹做好毕业、招聘、考录等相关工作，让他们顺利毕业、尽早就业”。为切实贯彻落实习近平总书记的重要讲话精神，现就应对新冠肺炎疫情影响做好事业单位公开招聘高校毕业生工作有关事项通知如下。

一、各地各部门要落实分区分级精准防控要求，在切实做好疫情防控工作的同时，安全有序开展事业单位公开招聘工作。要创新招聘方式，尽量采用电话、视频、网络等形式组织线上报名、笔试、面试。对疫情低风险地区，可在落实卫生防疫要求、控制规模、确保安全的前提下，开展现场笔试、面试等工作。对疫情高、中风险地区高校毕业生报名参加招聘的，暂不组织现场笔试、面试，以适当方式开展线上笔试、面试。

二、要加大事业单位面向高校毕业生的公开招聘力度，今明两年事业单位空缺岗位主要用于专项招聘高校毕业生（含择业期内未落实工作单位的高校毕业生）。其中，湖北省事业单位可以面向湖北省高校的毕业生或湖北籍高校毕业生开展专项招聘。

三、要组织指导事业单位及早发布招聘公告，公布岗位数量和岗位条件，及时为高校毕业生应聘提供岗位信息，增加就业机会。招聘公告须在事业单位人事综合管理部门公开招聘服务平台、主管部门网站上发布；有条件的地区，可在省级事业单位人事综合管理部门公开招聘服务平台上集中发布；要有针对性地通过报纸、电视、广播、网络等媒介广泛推介招聘信息，扩大信息发布范围和社会知晓度。

四、要积极鼓励和引导高校毕业生到艰苦边远地区基层事业单位工作，在打赢脱贫攻坚战、决胜全面建成小康社会中建功立业。艰苦边远地区乡镇事业单位招聘本科以上高校毕业生、县级事业单位招聘硕士以上高校毕业生，以及招聘行业、岗位、脱贫攻坚急需紧缺专业高校毕业生，可以结合实际情况，采取面试、直接考察的方式公开招聘；可以根据应聘人员报名、专业分布等情况适当降低开考比例，或不设开考比例，划定成绩合格线。

五、要积极鼓励和引导高校毕业生参加“三支一扶”基层服务项目计划。对高校毕业生参加“三支一扶”计划服务期满且考核合格的，可以按照有关规定进行专项招聘，并增加工作实绩在组织考察中的权重。对今明两年经省级人力资源社会保障部门统一组织招募参加“三支一扶”计划，服务期满且考核合格的人员，所在基层事业单位有岗位空缺的可以直接聘用，并不再约定试用期。

六、各地各部门要根据疫情防控要求，制定事业单位公开招聘活动防疫指南，落实落细各项防疫措施。事业单位人事综合管理部门、主管部门和事业单位要统筹毕业和招聘工作的衔接，优化做好高校毕业生特别是疫情严重地区高校毕业生公开招聘报名、考试、考察、体检、聘用报到等工作，保障疫情高风险地区高校毕业生公平参加招聘的权益，切实把党中央、国务院对高校毕业生的关心关爱落实到位。

中共中央组织部办公厅 人力资源社会保障部办公厅

2020年3月11日

1. 教育部 财政部：做好2020年中小学幼儿园教师国家级培训计划组织实施工作 教师厅〔2020〕1号

各省、自治区、直辖市教育厅（教委）、财政厅（局），新疆生产建设兵团教育局、财政局，有关单位：

　　为深入学习贯彻习近平新时代中国特色社会主义思想和党的十九大精神，全面落实《中共中央 国务院关于全面深化新时代教师队伍建设改革的意见》，切实提高精准培训的组织能力，努力形成“学习强师”的可持续发展局面，不断增强教师立德树人的本领，建设适应教育现代化要求的高素质专业化创新型教师队伍，坚决打赢疫情防控阻击战，现就做好2020年中小学幼儿园教师国家级培训计划（以下简称“国培计划”）组织实施工作有关事宜通知如下。

　　一、落实中央部署，按需做好疫情防控期间线上培训

　　助力打赢疫情阻击战。疫情防控期间，各地各校一律不得组织教师线下集中面授培训。各地要根据当地“停课不停学”工作部署，合理调整培训项目设置和经费预算安排，灵活安排、有效开展教师线上培训，支持教师立足教育教学岗位助力打赢疫情阻击战。

　　因地制宜开展在线教学培训。实施教师在线教学能力提升行动，已经或计划开展在线教学的省（区、市），要通过线上培训帮助教师掌握所选用平台的使用方法，加强教师在线教学方法和信息技术应用研修。设置心理疏导调适、疫情防控知识、健康卫生教育、信息安全、学生视力保护等培训专题。将家校合作纳入教师培训内容，推进生命教育、感恩教育、责任教育融入家庭教育。

　　加强疫情严重地区教师培训帮扶。各级教师培训资源向疫情严重省份予以倾斜，组织“国培计划”远程培训机构开放教师培训网络资源。发挥“国培计划”承担机构院校、名师名校长等作用，向疫情严重地区的学校主动输送优质线上课程资源。组织有关高校、教师发展机构等开放资源，为当地教师提供应对疫情急需的信息素养提升、心理疏导、卫生防疫等支援服务。

　　二、落实立德树人，服务基础教育改革发展需要

　　深入推进师德养成教育。认真学习党的十九届四中全会、全国教育大会精神，开展习近平总书记关于教育的重要论述专题学习。设置师德师风培训课程内容，试行师德修养、班级管理等培训课程指导标准，践行新时代教师职业行为准则，分享展现时代风貌的优秀影视作品，讲好身边最美教师的教育故事，加强党史、新中国史、改革开放史教育，弘扬民族精神和时代精神，推动理想信念教育常态化、制度化，引导教师以德立身、以德立学、以德施教、以德育德，争做新时代“四有”好老师。

　　服务基础教育改革发展大局。以培养德智体美劳全面发展的社会主义建设者和接班人为目标，贯彻落实《中共中央 国务院关于学前教育深化改革规范发展的若干意见》《中共中央 国务院关于深化教育教学改革 全面提高义务教育质量的意见》《国务院办公厅关于新时代推进普通高中育人方式改革的指导意见》等决策部署，明确学前教育、义务教育、高中阶段教育教师培训重点，落实“五育并举”，切实提升教师立德树人的能力。

　　三、落实扶贫攻坚，提升贫困地区乡村教师素质

　　精准实施乡村教师培训扶贫攻坚行动。以“三区三州”等深度贫困地区、集中连片贫困地区为重点，实现贫困地区乡村教师、校园长培训全覆盖。加强民族地区教师的国家通用语言文字应用能力培训。各省（市、区）贫困地区乡村教师校长培训全覆盖任务完成情况将纳入“国培计划”项目绩效评估指标体系。

　　实施“一对一”对口帮扶培训项目。跨区域遴选高等学校、优质学校和研训机构，组建名师名校长引领团队，重点支持深度贫困地区学校，实施底部攻坚，精准帮扶，形成国家示范、地方跟进、以点带面的精准扶贫机制。

　　探索集成建设教师智能研修平台。联合移动互联网企业，打造教师移动学习终端。通过名师专题课堂、双师教学、远程协同教研等形式，推送优质教学资源，重点帮助乡村教师提高教育教学水平。

　　四、落实分层分类，改进教师培训内容与方式

　　规范分层分类培训。聚焦不同发展阶段教师和乡村校园长应具备的核心素养与关键能力，示范引领各地按照培训指南要求，分层分类开展新教师入职培训、青年教师助力培训、骨干教师提升培训及教师培训者团队研修和校园长任职培训、提高培训、高级研修及专题培训。

　　完善培训内容方式。服务学生成长，突出育人主线，依据教师校长培训课程指导标准优化项目内容设计。重点围绕学科育德、学科知识体系、课堂学习指导与有效教学、作业与考试命题设计、家庭教育指导能力等方面完善培训课程设置。增加网络研修比重，推进教师校长自主选学，实施混合式研修。实行任务驱动教学，突出教师参与，强化教师实践，注重成果产出。

　　加强信息技术应用培训。按照《教育部关于实施全国中小学教师信息技术应用能力提升工程2.0的意见》要求，依据整校推进实施指南，基于学校信息化设施设备基础条件和应用需要，构建“以校为本、基于课堂、应用驱动、注重创新、精准测评”教师信息素养发展新机制。分类设置管理者、培训团队、学科骨干教师项目，示范引领中小学校开展信息化教学校本研修。

　　建立骨干教师引领辐射机制。重视骨干教师在培训中的重要作用，发挥好名师名校长和国家级骨干教师带动区域教育教学改革的示范作用、骨干培训者推进培训专业化的引领作用。

　　五、强化能力建设，提升培训体系专业化水平

　　完善教师培训体系。继续加强责任明确、分工合理、分层递进的国家、省、市、县、校五级培训体系建设。面向乡村，重点提升县级教师发展支持体系的专业能力，有效带动学校校本研修和教师自主研修。

　　培育高水平教师培训团队。采取需求导向、任务驱动、实践取向、成果引领等方式，突出思想政治素质和师德要求，提升培训设计、实施、评价、研究等关键能力，培养省级专业培训团队，示范带动县级教师培训团队建设。

　　打造培训品牌基地。以项目县为载体，建设优质市县教师发展机构。高等学校、教师培训机构要以建设培训品牌基地为目标，实现项目实施与培训研究、成果推广有机融合，建设精品培训项目，生成优质培训成果，在体系建设中发挥龙头作用。

　　六、强化规范管理，形成有活力可持续的发展机制

　　推进培训项目有效执行。各地要结合当地疫情防控情况，进一步优化组织实施流程，提高工作效率，推进项目执行进度。当年，各地收到中央财政提前下达下一年度“国培计划”补助资金预算后，应立即研究制订下一年度项目实施方案，同时按规定及时分解下达资金，确保培训组织实施工作顺利开展。下一年度，各地应根据获得的“国培计划”补助资金全年预算，结合实际及时补充完善年度实施项目，加快培训实施进程。完善后的年度项目实施方案应及时报教育部备案。教育部将委托有关专家工作组开展年度项目实施方案诊断指导，定期进行督促检查和项目视导。

　　严格规范管理培训项目。健全培训管理制度，设立或委托专门管理机构，组建省级专家工作组，对项目实施和资金使用等进行全过程全方位监管，做到对培训机构与项目区县的指导与评估的全覆盖，并通报评估结果。完善资金使用细则，加强资金使用监管，保证资金安全。健全教师培训管理信息化平台，规范、精细管理教师培训学分，深化培训学分应用，激发教师参训动力。

　　精准学员选派和严格过程管理。健全教师参训数据库和储备库，根据项目条件精准遴选参训学员，对不符合参训条件的学员进行劝退。开展学员遴选“双向选择”试点，教育行政部门公布培训项目计划后，教师自愿报名参加培训，项目承担单位择优录取学员，教育行政部门审核公示。合理规定周期内教师参加“国培计划”的限制次数，杜绝重复培训。严格过程管理，开展学员培训成果应用考核，及时向学员选派单位通报参训情况。将教师参培情况纳入培训诚信档案，无特殊情况未参训、被劝退、未结业的学员在三年内不得再参加“国培计划”。加强学员健康管理和安全教育，建立突发事件应急机制，改善培训条件，引导学员培训期间购买人身意外伤害保险。

　　激发多方参与“国培计划”的活力。鼓励教育部直属事业单位和高等学校发挥资源优势、专业优势和学科优势，积极参与“国培计划”项目。高等学校要采取综合措施激励教师参与培训工作，将教师参与授课、指导、管理等纳入工作量考核范围。实行首席专家负责制，聘请高水平专家担任项目首席专家，遴选专业能力强的培训者担任班主任。

　　开展“国培十年”总结工作。组织专门力量，重点围绕支持服务体系、管理体制机制、培训模式创新、培训内容建设、培训质量监管、培训绩效评估等方面，开展“国培计划”典型案例、精品项目评选、先进事迹宣传、优秀成果交流展示等系列活动。开展国家级优秀培训团队和优质培训资源遴选创建工作，着力打造国培精品项目和国培品牌基地。对“国培计划”实施十年以来的主要经验和成效进行全面总结，重点提炼“国培计划”的价值、特色与创新点，形成“国培计划”质量报告，继往开来，示范引领全国教师培训工作。

　　附件：1.“国培计划”示范项目实施方案

　　　　　2.“国培计划”中西部项目实施方案

　　　　　3.“国培计划”幼师国培项目实施方案

教育部办公厅 财政部办公厅

2020年3月4日

1. 最高人民法院等5部门：进一步加强国境卫生检疫工作 依法惩治妨害国境卫生检疫违法犯罪 署法发〔2020〕50号

各省、自治区、直辖市高级人民法院、人民检察院、公安厅（局）、司法厅（局），解放军军事法院、军事检察院，新疆维吾尔自治区高级人民法院生产建设兵团分院、新疆生产建设兵团人民检察院、公安局、司法局，海关总署广东分署，海关总署驻天津、上海特派办，各直属海关：

　　为贯彻落实《中央全面依法治国委员会关于依法防控新型冠状病毒感染肺炎疫情、切实保障人民群众生命健康安全的意见》，保证国境卫生检疫所涉行政执法和刑事司法的有效衔接、相关法律法规的准确适用，为防控疫病疫情跨境传播、维护公共卫生安全和社会安定有序提供有力的法治保障，最高人民法院、最高人民检察院、公安部、司法部、海关总署联合制定了《关于进一步加强国境卫生检疫工作 依法惩治妨害国境卫生检疫违法犯罪的意见》。现予以印发，请结合实际认真贯彻执行。在执行中遇到有关情况和问题，请分别及时报告最高人民法院、最高人民检察院、公安部、司法部、海关总署。

　　特此通知。

　　附件： 最高人民法院 最高人民检察院 公安部 司法部 海关总署关于进一步加强国境卫生检疫工作 依法惩治妨害国境卫生检疫违法犯罪的意见

最高人民法院 最高人民检察院 公安部

司法部 海关总署

　　2020年3月13日

最高人民法院 最高人民检察院 公安部

司法部 海关总署关于进一步加强国境

卫生检疫工作 依法惩治妨害国境

卫生检疫违法犯罪的意见

　　为进一步加强国境卫生检疫工作，依法惩治妨害国境卫生检疫违法犯罪行为，维护公共卫生安全，保障人民群众生命安全和身体健康，根据有关法律、司法解释的规定，制定本意见。

　　一、充分认识国境卫生检疫对于维护公共卫生安全的重要意义

　　国境卫生检疫对防止传染病传入传出国境，保障人民群众生命安全和身体健康，维护公共卫生安全和社会安定有序发挥着重要作用。党中央、国务院高度重视国境卫生检疫工作，特别是新冠肺炎疫情发生以来，习近平总书记对强化公共卫生法治保障、改革完善疾病预防控制体系、健全防治结合、联防联控、群防群治工作机制作出一系列重要指示批示。各级人民法院、人民检察院、公安机关、司法行政机关、海关要切实提高政治站位，把思想和行动统一到习近平总书记重要指示批示精神上来，坚决贯彻落实党中央决策部署，增强“四个意识”、坚定“四个自信”、做到“两个维护”；从贯彻落实总体国家安全观、推动构建人类命运共同体的高度，始终将人民群众的生命安全和身体健康放在第一位，切实提升国境卫生检疫行政执法和司法办案水平。特别是面对当前新冠肺炎疫情在境外呈现扩散态势、通过口岸向境内蔓延扩散风险加剧的严峻形势，要依法及时、从严惩治妨害国境卫生检疫的各类违法犯罪行为，切实筑牢国境卫生检疫防线，坚决遏制疫情通过口岸传播扩散，为维护公共卫生安全提供有力的法治保障。

　　二、依法惩治妨害国境卫生检疫的违法犯罪行为

　　为加强国境卫生检疫工作，防止传染病传入传出国境，保护人民群众健康安全，刑法、国境卫生检疫法对妨害国境卫生检疫违法犯罪行为及其处罚作出规定。人民法院、人民检察院、公安机关、海关在办理妨害国境卫生检疫案件时，应当准确理解和严格适用刑法、国境卫生检疫法等有关规定，依法惩治相关违法犯罪行为。

　　（一）进一步加强国境卫生检疫行政执法。海关要在各口岸加强国境卫生检疫工作宣传，引导出入境人员以及接受检疫监管的单位和人员严格遵守国境卫生检疫法等法律法规的规定，配合和接受海关国境卫生检疫。同时，要加大国境卫生检疫行政执法力度，对于违反国境卫生检疫法及其实施细则，尚不构成犯罪的行为，依法给予行政处罚。

　　（二）依法惩治妨害国境卫生检疫犯罪。根据刑法第三百三十二条规定，违反国境卫生检疫规定，实施下列行为之一的，属于妨害国境卫生检疫行为：

　　1. 检疫传染病染疫人或者染疫嫌疑人拒绝执行海关依照国境卫生检疫法等法律法规提出的健康申报、体温监测、医学巡查、流行病学调查、医学排查、采样等卫生检疫措施，或者隔离、留验、就地诊验、转诊等卫生处理措施的；

　　2. 检疫传染病染疫人或者染疫嫌疑人采取不如实填报健康申明卡等方式隐瞒疫情，或者伪造、涂改检疫单、证等方式伪造情节的；

　　3. 知道或者应当知道实施审批管理的微生物、人体组织、生物制品、血液及其制品等特殊物品可能造成检疫传染病传播，未经审批仍逃避检疫，携运、寄递出入境的；

　　4. 出入境交通工具上发现有检疫传染病染疫人或者染疫嫌疑人，交通工具负责人拒绝接受卫生检疫或者拒不接受卫生处理的；

　　5. 来自检疫传染病流行国家、地区的出入境交通工具上出现非意外伤害死亡且死因不明的人员，交通工具负责人故意隐瞒情况的；

　　6. 其他拒绝执行海关依照国境卫生检疫法等法律法规提出的检疫措施的。

　　实施上述行为，引起鼠疫、霍乱、黄热病以及新冠肺炎等国务院确定和公布的其他检疫传染病传播或者有传播严重危险的，依照刑法第三百三十二条的规定，以妨害国境卫生检疫罪定罪处罚。

　　对于单位实施妨害国境卫生检疫行为，引起鼠疫、霍乱、黄热病以及新冠肺炎等国务院确定和公布的其他检疫传染病传播或者有传播严重危险的，应当对单位判处罚金，并对其直接负责的主管人员和其他直接责任人员定罪处罚。

　　三、健全完善工作机制，保障依法科学有序防控

　　（一）做好行刑衔接。海关要严把口岸疫情防控第一关，严厉追究违反国境卫生检疫规定的行政法律责任，完善执法办案流程，坚持严格执法和依法办案。做好行政执法和刑事司法的衔接，对符合国境卫生检疫监管领域刑事案件立案追诉标准的案件，要依照有关规定，及时办理移送公安机关的相关手续，不得以行政处罚代替刑事处罚。

　　（二）加快案件侦办。公安机关对于妨害国境卫生检疫犯罪案件，要依法及时立案查处，全面收集固定证据。对新冠肺炎疫情防控期间发生的妨害国境卫生检疫犯罪，要快侦快破，并及时予以曝光，形成强大震慑。

　　（三）强化检察职能。人民检察院要加强对妨害国境卫生检疫犯罪案件的立案监督，发现应当立案而不立案的，应当要求公安机关说明理由，认为理由不成立的，应当依法通知公安机关立案。对于妨害国境卫生检疫犯罪案件，人民检察院可以对案件性质、收集证据和适用法律等向公安机关提出意见建议。对于符合逮捕、起诉条件的涉嫌妨害国境卫生检疫罪的犯罪嫌疑人，应当及时批准逮捕、提起公诉。发挥检察建议的作用，促进疫情防控体系化治理。

　　（四）加强沟通协调。人民法院、人民检察院、公安机关、司法行政机关、海关要加强沟通协调，畅通联系渠道，建立常态化合作机制。既要严格履行法定职责，各司其职，各负其责，又要相互配合，相互协作，实现资源共享和优势互补，形成依法惩治妨害国境卫生检疫违法犯罪的合力。对社会影响大、舆论关注度高的重大案件，要按照依法处置、舆论引导、社会面管控“三同步”要求，及时澄清事实真相，做好舆论引导和舆情应对工作。

　　（五）坚持过罚相当。进一步规范国境卫生检疫执法活动，切实做到严格规范公正文明执法。注重把握宽严相济政策：对于行政违法行为，要根据违法行为的危害程度和悔过态度，综合确定处罚种类和幅度。对于涉嫌犯罪的，要重点打击情节恶劣、后果严重的犯罪行为；对于情节轻微且真诚悔改的，依法予以从宽处理。

　　（六）维护公平正义。人民法院、人民检察院、公安机关要依法保障犯罪嫌疑人、被告人的各项诉讼权利特别是辩护权，切实维护当事人合法权益，维护法律正确实施。司法行政机关要加强对律师辩护代理工作的指导监督，促进律师依法依规执业。人民法院、人民检察院、公安机关、司法行政机关、海关要认真落实“谁执法谁普法”责任制，选取典型案例，开展以案释法，加大警示教育，震慑不法分子，释放正能量，为疫情防控营造良好的法治和社会环境。

1. 国务院应对新型冠状病毒感染肺炎疫情联防联控机制：印发《因新冠肺炎疫情影响造成监护缺失儿童救助保护工作方案》 国发明电〔2020〕11号

各省、自治区、直辖市人民政府，国务院各部委、各直属机构：

《因新冠肺炎疫情影响造成监护缺失儿童救助保护工作方案》已经中央应对新型冠状病毒感染肺炎疫情工作领导小组同意，现予印发，请各地各有关部门结合实际，采取有效措施，切实解决有关问题，兜住安全底线。

国务院应对新型冠状病毒感染

肺炎疫情联防联控机制

2020年3月14日

因新冠肺炎疫情影响造成监护缺失儿童

救助保护工作方案

儿童是祖国的未来、民族的希望。为深入贯彻落实中央应对新冠肺炎疫情工作领导小组《关于进一步做好疫情防控期间困难群众兜底保障工作的通知》（国发明电〔2020〕9号）和国务院联防联控机制《关于进一步做好民政服务机构疫情防控工作的通知》（国发明电〔2020〕6号）要求，进一步做好因新冠肺炎疫情影响造成监护缺失儿童（以下简称监护缺失儿童）救助保护工作，制定本方案。

本方案所称监护缺失儿童，按照《民政部办公厅关于做好因新冠肺炎疫情影响造成监护缺失的儿童救助保护工作的通知》（民电〔2020〕19号）界定，包括其父母或其他监护人确认感染、疑似感染或需隔离观察，其父母或其他监护人因防疫抗疫工作需要以及其他因疫情影响不能完全履行抚养义务和监护职责的儿童。

一、及时发现报告

1.各地要指导乡镇人民政府（街道办事处）统筹儿童督导员、儿童主任、基层妇联执委、社区工作者、社会工作者等工作力量，结合疫情防控排查，对各村（社区）儿童监护情况进行全面摸底；相关人员发现儿童监护缺失的，要及时向村（居）民委员会、乡镇人民政府（街道办事处）报告。

2.卫生健康部门、医疗卫生机构对确诊收治或需集中隔离医学观察的对象，首先询问其监护对象情况，对存在儿童监护缺失情形的，要及时向其所在村（居）民委员会、工作单位或同级民政部门通报。村（居）民委员会要主动了解本辖区确诊收治或需集中隔离医学观察对象家庭的儿童监护情况。

3.父母或其他监护人因疫情影响暂时不能履行抚养义务和监护职责的，要第一时间主动向所在村（居）民委员会或者本人工作单位报告家中儿童监护状况及联系方式。

4.各地要开通儿童救助保护热线，通过手机短信、手机客户端、广播电视等形式加大宣传力度，在车站码头、村（社区）主干道、小区门口等醒目位置公布号码，结合疫情宣传、走访排查等告知儿童家庭，扩大社会知晓度。发挥“12338”妇女维权服务热线的发现、报告和转介作用。鼓励居民发现儿童脱离监护的情况并及时拨打热线进行报告。

5.提供上门服务的医务工作者、学校教师、儿童主任、社区工作者、社会工作者、志愿者和相关公益慈善组织，发现儿童监护缺失的，要及时向儿童实际居住地村（居）民委员会、乡镇人民政府（街道办事处）报告情况。

二、落实监护照料

6.儿童的父母或其他监护人对因疫情影响不能完全履行抚养义务和监护职责的，村（居）民委员会要督促其委托其他具有监护能力的人代为照料。对没有依法具有监护资格的人的，由村（居）民委员会临时照料；对确有困难的，由县级民政部门承担临时监护责任。

7.监护缺失儿童有重点疫区接触史、重点疫区人员接触史、新冠肺炎确诊病例或疑似病例接触史或者身体状况不明的，民政部门、乡镇人民政府（街道办事处）、村（居）民委员会要协调卫生健康部门将儿童安置到当地定点医疗卫生机构检测。对经检测未感染的儿童，乡镇人民政府（街道办事处）、村（居）民委员会要确认其监护责任的落实情况；对定性为确诊病例或疑似病例的，要优先安置到定点医疗卫生机构就诊救治。

8.各地要加强跟进指导，对委托监护、指定由专人照料的儿童，要指定儿童督导员、儿童主任、基层妇联执委、社区工作者、社会工作者等实行包干到人，对儿童照料情况进行家访或电话跟踪，每周不少于两次。

9.父母或其他监护人治愈出院或结束隔离后，要在提供当地医疗卫生机构出具的出院证明或相关健康医学证明的前提下，及时将儿童接回照料。

三、加强救助帮扶

10.在疫情防控期间，对父母因疫情影响不能完全履行抚养和监护责任的儿童，符合事实无人抚养儿童认定情形的，要及时将其纳入事实无人抚养儿童保障范围；符合孤儿认定情形的，要及时纳入孤儿保障范围；对民政部门负责临时监护的，由未成年人救助保护机构等妥善安置照料。

11.对因疫情影响导致生活陷入困难的儿童及家庭，要及时按规定给予临时救助。符合条件的，要及时落实低保等社会救助政策，确保基本生活得到有效保障。

12.卫生健康部门要指导医疗卫生机构开通儿童医疗救治绿色通道，确保确诊或疑似感染儿童第一时间送定点医疗卫生机构接受治疗。对疫情期间有其他疾病的监护缺失儿童，村（居）民委员会、受委托监护人要密切关注其身体状况，协助做好就医就诊。

13.各地要对有需求的儿童提供生活照料，对监护缺失儿童给予重点关怀，支持社会工作者、法律工作者等专业人员针对不同年龄段的儿童提供心理疏导、精神关爱、亲情陪伴等服务。教育部门和学校要加强对监护缺失适龄儿童居家学习的指导与服务，确保“停课不停学”。

14.未成年人救助保护机构要积极主动参与监护缺失儿童的线索响应、临时照料、服务转介、个案跟踪、资源链接等服务。

四、强化工作保障

15.各地要把监护缺失儿童救助保护工作纳入重要工作内容，完善工作方案，细化制度措施，加强组织保障，扎实做好各项工作，确保不发生冲击社会道德底线的事件。各级民政部门、妇联组织、妇儿工委办公室要发挥牵头作用。

16.各地要强化资金保障，各级民政部门要统筹使用困难群众救助资金、用于社会福利事业的彩票公益金等各类资金，多方筹措其他社会资金，切实加强监护缺失儿童救助保护工作。

17.加大政府购买服务力度，引进社会工作、心理咨询服务等机构，为监护缺失儿童提供各类专业服务，提高精准关爱水平。

18.加强监督指导，各地要加大跟踪检查力度，对因工作不到位发生极端问题的，要依法依规严肃追究相关责任。

1. 税务总局：发布支持疫情防控和经济社会发展税费优惠政策指引

一、支持防护救治

1.取得政府规定标准的疫情防治临时性工作补助和奖金免征个人所得税

【享受主体】

参加疫情防治工作的医务人员和防疫工作者

【优惠内容】

自2020年1月1日起，对参加疫情防治工作的医务人员和防疫工作者按照政府规定标准取得的临时性工作补助和奖金，免征个人所得税。政府规定标准包括各级政府规定的补助和奖金标准。

对省级及省级以上人民政府规定的对参与疫情防控人员的临时性工作补助和奖金，比照执行。

上述优惠政策适用的截止日期将视疫情情况另行公告。

【政策依据】

《财政部 税务总局关于支持新型冠状病毒感染的肺炎疫情防控有关个人所得税政策的公告》（2020年第10号）

2.个人取得单位发放的预防新型冠状病毒感染肺炎的医药防护用品等免征个人所得税

【享受主体】

取得单位发放的用于预防新型冠状病毒感染的肺炎的药品、医疗用品和防护用品等实物（不包括现金）的个人

【优惠内容】

自2020年1月1日起，单位发给个人用于预防新型冠状病毒感染的肺炎的药品、医疗用品和防护用品等实物（不包括现金），不计入工资、薪金收入，免征个人所得税。

上述优惠政策适用的截止日期将视疫情情况另行公告。

【政策依据】

《财政部 税务总局关于支持新型冠状病毒感染的肺炎疫情防控有关个人所得税政策的公告》（2020年第10号）

二、支持物资供应

3.对疫情防控重点保障物资生产企业全额退还增值税增量留抵税额

【享受主体】

疫情防控重点保障物资生产企业

【优惠内容】

自2020年1月1日起，疫情防控重点保障物资生产企业可以按月向主管税务机关申请全额退还增值税增量留抵税额。增量留抵税额，是指与2019年12月底相比新增加的期末留抵税额。

企业名单由省级及省级以上发展改革部门、工业和信息化部门确定。

上述优惠政策适用的截止日期将视疫情情况另行公告。

疫情防控重点保障物资生产企业适用增值税增量留抵退税政策的，应当在增值税纳税申报期内，完成本期增值税纳税申报后，向主管税务机关申请退还增量留抵税额。

【政策依据】

（1）《财政部 税务总局关于支持新型冠状病毒感染的肺炎疫情防控有关税收政策的公告》（2020年第8号）

（2）《国家税务总局关于支持新型冠状病毒感染的肺炎疫情防控有关税收征收管理事项的公告》（2020年第4号）

4.纳税人提供疫情防控重点保障物资运输收入免征增值税

【享受主体】

提供疫情防控重点保障物资运输服务的纳税人

【优惠内容】

自2020年1月1日起，对纳税人运输疫情防控重点保障物资取得的收入，免征增值税。优惠政策适用的截止日期将视疫情情况另行公告。

疫情防控重点保障物资的具体范围，由国家发展改革委、工业和信息化部确定，具体内容如下：

国家发展改革委疫情防控重点保障物资清单

税务总局：发布支持疫情防控和经济社会发展税费优惠政策指引1.jpg

工业和信息化部疫情防控重点保障物资（医疗应急）清单

税务总局：发布支持疫情防控和经济社会发展税费优惠政策指引2.jpg

纳税人运输疫情防控重点保障物资取得的收入免征增值税的，免征城市维护建设税、教育费附加、地方教育附加。

纳税人按规定享受免征增值税优惠的，可自主进行免税申报，无需办理有关免税备案手续，但应将相关证明材料留存备查。在办理增值税纳税申报时，应当填写增值税纳税申报表及《增值税减免税申报明细表》相应栏次。

纳税人按规定适用免征增值税政策的，不得开具增值税专用发票；已开具增值税专用发票的，应当开具对应红字发票或者作废原发票，再按规定适用免征增值税政策并开具普通发票。纳税人在疫情防控期间已经开具增值税专用发票，按规定应当开具对应红字发票而未及时开具的，可以先适用免征增值税政策，对应红字发票应当于相关免征增值税政策执行到期后1个月内完成开具。

纳税人已将适用免税政策的销售额、销售数量，按照征税销售额、销售数量进行增值税申报的，可以选择更正当期申报或者在下期申报时调整。已征应予免征的增值税税款，可以予以退还或者抵减纳税人以后应缴纳的增值税税款。

【政策依据】

（1）《财政部 税务总局关于支持新型冠状病毒感染的肺炎疫情防控有关税收政策的公告》（2020年第8号）

（2）《国家税务总局关于支持新型冠状病毒感染的肺炎疫情防控有关税收征收管理事项的公告》（2020年第4号）

5.纳税人提供公共交通运输服务、生活服务及居民必需生活物资快递收派服务收入免征增值税

【享受主体】

提供公共交通运输服务、生活服务，以及为居民提供必需生活物资快递收派服务的纳税人

【优惠内容】

自2020年1月1日起，对纳税人提供公共交通运输服务、生活服务，以及为居民提供必需生活物资快递收派服务取得的收入，免征增值税。

公共交通运输服务的具体范围，按照《营业税改征增值税试点有关事项的规定》（财税〔2016〕36号印发）执行。

生活服务、快递收派服务的具体范围，按照《销售服务、无形资产、不动产注释》（财税〔2016〕36号印发）执行。

税务总局：发布支持疫情防控和经济社会发展税费优惠政策指引3.jpg

上述优惠政策适用的截止日期将视疫情情况另行公告。

纳税人提供公共交通运输服务、生活服务，以及为居民提供必需生活物资快递收派服务取得的收入免征增值税的，免征城市维护建设税、教育费附加、地方教育附加。

纳税人按规定享受免征增值税优惠的，可自主进行免税申报，无需办理有关免税备案手续，但应将相关证明材料留存备查。在办理增值税纳税申报时，应当填写增值税纳税申报表及《增值税减免税申报明细表》相应栏次。

纳税人按规定适用免征增值税政策的，不得开具增值税专用发票；已开具增值税专用发票的，应当开具对应红字发票或者作废原发票，再按规定适用免征增值税政策并开具普通发票。纳税人在疫情防控期间已经开具增值税专用发票，按规定应当开具对应红字发票而未及时开具的，可以先适用免征增值税政策，对应红字发票应当于相关免征增值税政策执行到期后1个月内完成开具。

纳税人已将适用免税政策的销售额、销售数量，按照征税销售额、销售数量进行增值税申报的，可以选择更正当期申报或者在下期申报时调整。已征应予免征的增值税税款，可以予以退还或者抵减纳税人以后应缴纳的增值税税款。

【政策依据】

（1）《财政部 税务总局关于支持新型冠状病毒感染的肺炎疫情防控有关税收政策的公告》（2020年第8号）

（2）《财政部 税务总局关于全面推开营业税改征增值税试点的通知》（财税〔2016〕36号）

（3）《国家税务总局关于支持新型冠状病毒感染的肺炎疫情防控有关税收征收管理事项的公告》（2020年第4号）

6.对疫情防控重点保障物资生产企业扩大产能购置设备允许企业所得税税前一次性扣除

【享受主体】

疫情防控重点保障物资生产企业

【优惠内容】

自2020年1月1日起，对疫情防控重点保障物资生产企业为扩大产能新购置的相关设备，允许一次性计入当期成本费用在企业所得税税前扣除。

企业名单由省级及省级以上发展改革部门、工业和信息化部门确定。

上述优惠政策适用的截止日期将视疫情情况另行公告。

疫情防控重点保障物资生产企业适用一次性企业所得税税前扣除政策的，在优惠政策管理等方面参照《国家税务总局关于设备器具扣除有关企业所得税政策执行问题的公告》（2018年第46号）的规定执行。企业在纳税申报时将相关情况填入企业所得税纳税申报表"固定资产一次性扣除"行次。

【政策依据】

（1）《财政部 税务总局关于支持新型冠状病毒感染的肺炎疫情防控有关税收政策的公告》（2020年第8号）

（2）《国家税务总局关于支持新型冠状病毒感染的肺炎疫情防控有关税收征收管理事项的公告》（2020年第4号）

7.对卫生健康主管部门组织进口的直接用于防控疫情物资免征关税

【享受主体】

卫生健康主管部门组织进口的直接用于防控疫情物资

【优惠内容】

自2020年1月1日至2020年3月31日，对卫生健康主管部门组织进口的直接用于防控疫情物资免征关税。

免税进口物资，可按照或比照海关总署公告2020年第17号，先登记放行，再按规定补办相关手续。

【政策依据】

《财政部 海关总署 税务总局关于防控新型冠状病毒感染的肺炎疫情进口物资免税政策的公告》（2020年第6号）

三、鼓励公益捐赠

8.通过公益性社会组织或县级以上人民政府及其部门等国家机关捐赠应对疫情的现金和物品允许企业所得税或个人所得税税前全额扣除

【享受主体】

通过公益性社会组织或者县级以上人民政府及其部门等国家机关对应对新型冠状病毒感染的肺炎疫情进行捐赠的企业和个人

【优惠内容】

自2020年1月1日起，企业和个人通过公益性社会组织或者县级以上人民政府及其部门等国家机关，捐赠用于应对新型冠状病毒感染的肺炎疫情的现金和物品，允许在计算企业所得税或个人所得税应纳税所得额时全额扣除。

国家机关、公益性社会组织接受的捐赠，应专项用于应对新型冠状病毒感染的肺炎疫情工作，不得挪作他用。

上述优惠政策适用的截止日期将视疫情情况另行公告。

"公益性社会组织"是指依法取得公益性捐赠税前扣除资格的社会组织。企业享受规定的全额税前扣除政策的，采取"自行判别、申报享受、相关资料留存备查"的方式，并将捐赠全额扣除情况填入企业所得税纳税申报表相应行次。个人享受规定的全额税前扣除政策的，按照《财政部 税务总局关于公益慈善事业捐赠个人所得税政策的公告》（2019年第99号）有关规定执行。

【政策依据】

（1）《财政部 税务总局关于支持新型冠状病毒感染的肺炎疫情防控有关捐赠税收政策的公告》（2020年第9号）

（2）《国家税务总局关于支持新型冠状病毒感染的肺炎疫情防控有关税收征收管理事项的公告》（2020年第4号）

9.直接向承担疫情防治任务的医院捐赠应对疫情物品允许企业所得税或个人所得税税前全额扣除

【享受主体】

直接向承担疫情防治任务的医院捐赠用于应对新型冠状病毒感染的肺炎疫情物品的企业和个人

【优惠内容】

自2020年1月1日起，企业和个人直接向承担疫情防治任务的医院捐赠用于应对新型冠状病毒感染的肺炎疫情的物品，允许在计算企业所得税或个人所得税应纳税所得额时全额扣除。

捐赠人凭承担疫情防治任务的医院开具的捐赠接收函办理税前扣除事宜。

承担疫情防治任务的医院接受的捐赠，应专项用于应对新型冠状病毒感染的肺炎疫情工作，不得挪作他用。

上述优惠政策适用的截止日期将视疫情情况另行公告。

企业享受规定的全额税前扣除政策的，采取"自行判别、申报享受、相关资料留存备查"的方式，并将捐赠全额扣除情况填入企业所得税纳税申报表相应行次。个人享受规定的全额税前扣除政策的，按照《财政部 税务总局关于公益慈善事业捐赠个人所得税政策的公告》（2019年第99号）有关规定执行；在办理个人所得税税前扣除、填写《个人所得税公益慈善事业捐赠扣除明细表》时，应当在备注栏注明"直接捐赠"。

企业和个人取得承担疫情防治任务的医院开具的捐赠接收函，作为税前扣除依据自行留存备查。

【政策依据】

（1）《财政部 税务总局关于支持新型冠状病毒感染的肺炎疫情防控有关捐赠税收政策的公告》（2020年第9号）

（2）《国家税务总局关于支持新型冠状病毒感染的肺炎疫情防控有关税收征收管理事项的公告》（2020年第4号）

10.无偿捐赠应对疫情的货物免征增值税、消费税、城市维护建设税、教育费附加、地方教育附加

【享受主体】

无偿捐赠应对疫情货物的单位和个体工商户

【优惠内容】

自2020年1月1日起，单位和个体工商户将自产、委托加工或购买的货物，通过公益性社会组织和县级以上人民政府及其部门等国家机关，或者直接向承担疫情防治任务的医院，无偿捐赠用于应对新型冠状病毒感染的肺炎疫情的，免征增值税、消费税、城市维护建设税、教育费附加、地方教育附加。

上述优惠政策适用的截止日期将视疫情情况另行公告。

纳税人按规定享受免征增值税、消费税优惠的，可自主进行免税申报，无需办理有关免税备案手续，但应将相关证明材料留存备查。在办理增值税纳税申报时，应当填写增值税纳税申报表及《增值税减免税申报明细表》相应栏次；在办理消费税纳税申报时，应当填写消费税纳税申报表及《本期减（免）税额明细表》相应栏次。

纳税人按规定适用免征增值税政策的，不得开具增值税专用发票；已开具增值税专用发票的，应当开具对应红字发票或者作废原发票，再按规定适用免征增值税政策并开具普通发票。纳税人在疫情防控期间已经开具增值税专用发票，按规定应当开具对应红字发票而未及时开具的，可以先适用免征增值税政策，对应红字发票应当于相关免征增值税政策执行到期后1个月内完成开具。

纳税人已将适用免税政策的销售额、销售数量，按照征税销售额、销售数量进行增值税、消费税纳税申报的，可以选择更正当期申报或者在下期申报时调整。已征应予免征的增值税、消费税税款，可以予以退还或者分别抵减纳税人以后应缴纳的增值税、消费税税款。

【政策依据】

（1）《财政部 税务总局关于支持新型冠状病毒感染的肺炎疫情防控有关捐赠税收政策的公告》（2020年第9号）

（2）《国家税务总局关于支持新型冠状病毒感染的肺炎疫情防控有关税收征收管理事项的公告》（2020年第4号）

11.扩大捐赠免税进口物资范围

【享受主体】

防控疫情捐赠进口物资

【优惠内容】

自2020年1月1日至2020年3月31日，适度扩大《慈善捐赠物资免征进口税收暂行办法》规定的免税进口范围，对捐赠用于疫情防控的进口物资，免征进口关税和进口环节增值税、消费税。

（1）进口物资增加试剂，消毒物品，防护用品，救护车、防疫车、消毒用车、应急指挥车。

（2）免税范围增加国内有关政府部门、企事业单位、社会团体、个人以及来华或在华的外国公民从境外或海关特殊监管区域进口并直接捐赠；境内加工贸易企业捐赠。捐赠物资应直接用于防控疫情且符合前述第（1）项或《慈善捐赠物资免征进口税收暂行办法》规定。

（3）受赠人增加省级民政部门或其指定的单位。省级民政部门将指定的单位名单函告所在地直属海关及省级税务部门。

财政部、海关总署、税务总局公告2020年第6号项下免税进口物资，已征收的应免税款予以退还。其中，已征税进口且尚未申报增值税进项税额抵扣的，可凭主管税务机关出具的《防控新型冠状病毒感染的肺炎疫情进口物资增值税进项税额未抵扣证明》，向海关申请办理退还已征进口关税和进口环节增值税、消费税手续；已申报增值税进项税额抵扣的，仅向海关申请办理退还已征进口关税和进口环节消费税手续。有关进口单位应在2020年9月30日前向海关办理退税手续。

免税进口物资，可按照或比照海关总署公告2020年第17号，先登记放行，再按规定补办相关手续。

【政策依据】

（1）《慈善捐赠物资免征进口税收暂行办法》（财政部 海关总署 税务总局公告2015年第102号发布）

（2）《财政部 海关总署 税务总局关于防控新型冠状病毒感染的肺炎疫情进口物资免税政策的公告》（2020年第6号）

四、支持复工复产

12.受疫情影响较大的困难行业企业2020年度发生的亏损最长结转年限延长至8年

【享受主体】

受疫情影响较大的困难行业企业

【优惠内容】

自2020年1月1日起，受疫情影响较大的困难行业企业2020年度发生的亏损，最长结转年限由5年延长至8年。

困难行业企业，包括交通运输、餐饮、住宿、旅游（指旅行社及相关服务、游览景区管理两类）四大类，具体判断标准按照现行《国民经济行业分类》执行。困难行业企业2020年度主营业务收入须占收入总额（剔除不征税收入和投资收益）的50%以上。

受疫情影响较大的困难行业企业按规定适用延长亏损结转年限政策的，应当在2020年度企业所得税汇算清缴时，通过电子税务局提交《适用延长亏损结转年限政策声明》。

【政策依据】

（1）《财政部 税务总局关于支持新型冠状病毒感染的肺炎疫情防控有关税收政策的公告》（2020年第8号）

（2）《国家税务总局关于支持新型冠状病毒感染的肺炎疫情防控有关税收征收管理事项的公告》（2020年第4号）

13.阶段性减免增值税小规模纳税人增值税

【享受主体】

增值税小规模纳税人

【优惠内容】

自2020年3月1日至5月31日，对湖北省增值税小规模纳税人，适用3%征收率的应税销售收入，免征增值税；适用3%预征率的预缴增值税项目，暂停预缴增值税。

自2020年3月1日至5月31日，除湖北省外，其他省、自治区、直辖市的增值税小规模纳税人，适用3%征收率的应税销售收入，减按1%征收率征收增值税，按以下公式计算销售额：销售额=含税销售额/（1+1%）；适用3%预征率的预缴增值税项目，减按1%预征率预缴增值税。

增值税小规模纳税人在办理增值税纳税申报时，按照上述规定，免征增值税的销售额等项目应当填写在《增值税纳税申报表（小规模纳税人适用）》及《增值税减免税申报明细表》免税项目相应栏次；减按1%征收率征收增值税的销售额应当填写在《增值税纳税申报表（小规模纳税人适用）》"应征增值税不含税销售额（3%征收率）"相应栏次，对应减征的增值税应纳税额按销售额的2%计算填写在《增值税纳税申报表（小规模纳税人适用）》"本期应纳税额减征额"及《增值税减免税申报明细表》减税项目相应栏次。

《增值税纳税申报表（小规模纳税人适用）附列资料》第8栏"不含税销售额"计算公式调整为：第8栏=第7栏÷（1+征收率)。

【政策依据】

（1）《财政部 税务总局关于支持个体工商户复工复业增值税政策的公告》（2020年第13号）

（2）《国家税务总局关于支持个体工商户复工复业等税收征收管理事项的公告》（2020年第5号）

14.阶段性减免企业养老、失业、工伤保险单位缴费

【享受主体】

除机关事业单位外的基本养老保险、失业保险、工伤保险（以下简称三项社会保险）参保单位

【优惠内容】

自2020年2月起，湖北省可免征各类参保单位（不含机关事业单位）三项社会保险单位缴费部分，免征期限不超过5个月。

自2020年2月起，各省、自治区、直辖市（除湖北省外）及新疆生产建设兵团（以下统称省）可根据受疫情影响情况和基金承受能力，免征中小微企业三项社会保险单位缴费部分，免征期限不超过5个月；对大型企业等其他参保单位（不含机关事业单位）三项社会保险单位缴费部分可减半征收，减征期限不超过3个月。

受疫情影响生产经营出现严重困难的企业，可申请缓缴社会保险费，缓缴期限原则上不超过6个月，缓缴期间免收滞纳金。

各省根据工业和信息化部、统计局、发展改革委、财政部《关于印发中小企业划型标准规定的通知》（工信部联企业〔2011〕300号）等有关规定，结合本省实际确定减免企业对象，并加强部门间信息共享，不增加企业事务性负担。

各省税务局要对2020年2月份已经征收的社保费进行分类，确定应退（抵）的企业和金额。要按照人力资源社会保障部、财政部、税务总局共同明确的处理原则，优化流程，提高效率，及时为应该退费的参保单位依职权办理退费，切实缓解企业特别是中小微企业经营困难。对采取以2月份已缴费款冲抵以后月份应缴费款的参保单位，要明确冲抵流程和操作办法，有序办理费款冲抵业务。

各级税务机关要会同有关部门落实好缓缴社保费政策，结合本地实际，进一步优化业务流程，从快办理缓缴相关业务。要严格落实缓缴期限原则上不超过6个月、缓缴期间免收滞纳金等政策要求，确保缴费人应享尽享。

【政策依据】

（1）《人力资源社会保障部 财政部 税务总局关于阶段性减免企业社会保险费的通知》（人社部发〔2020〕11号）

（2）《国家税务总局关于贯彻落实阶段性减免企业社会保险费政策的通知》（税总函〔2020〕33号）

15.阶段性减免以单位方式参保的个体工商户职工养老、失业、工伤保险

【享受主体】

以单位方式参保养老保险、失业保险、工伤保险的个体工商户

【优惠内容】

自2020年2月起，免征以单位方式参保的个体工商户三项社会保险单位缴费部分，免征期限不超过5个月。

【政策依据】

《人力资源社会保障部 财政部 税务总局关于阶段性减免企业社会保险费的通知》（人社部发〔2020〕11号）

16.阶段性减征职工基本医疗保险单位缴费

【享受主体】

基本医疗保险参保单位

【优惠内容】

自2020年2月起，各省、自治区、直辖市及新疆生产建设兵团（以下统称省）可指导统筹地区根据基金运行情况和实际工作需要，在确保基金收支中长期平衡的前提下，对职工医保单位缴费部分实行减半征收，减征期限不超过5个月。

原则上，统筹基金累计结存可支付月数大于6个月的统筹地区，可实施减征；可支付月数小于6个月但确有必要减征的统筹地区，由各省指导统筹考虑安排。缓缴政策可继续执行，缓缴期限原则上不超过6个月，缓缴期间免收滞纳金。

各省税务局要对2020年2月份已经征收的社保费进行分类，确定应退（抵）的企业和金额。要按照税务总局、国家医保局共同明确的处理原则，优化流程，提高效率，及时为应该退费的参保单位依职权办理退费，切实缓解企业特别是中小微企业经营困难。对采取以2月份已缴费款冲抵以后月份应缴费款的参保单位，要明确冲抵流程和操作办法，有序办理费款冲抵业务。

各级税务机关要会同有关部门落实好缓缴社保费政策，结合本地实际，进一步优化业务流程，从快办理缓缴相关业务。要严格落实缓缴期限原则上不超过6个月、缓缴期间免收滞纳金等政策要求，确保缴费人应享尽享。

【政策依据】

（1）《国家医保局 财政部 税务总局关于阶段性减征职工基本医疗保险费的指导意见》（医保发〔2020〕6号）

（2）《国家税务总局关于贯彻落实阶段性减免企业社会保险费政策的通知》（税总函〔2020〕33号）

17.鼓励各地通过减免城镇土地使用税等方式支持出租方为个体工商户减免物业租金

详见地方文件。

2020.3.16

1. 卫健委：印发新冠肺炎出院患者健康管理方案（试行） 国卫办医函〔2020〕225号

各省、自治区、直辖市及新疆生产建设兵团卫生健康委：

为进一步规范和加强新冠肺炎患者出院后的隔离管理、复诊复检、健康监测、康复医疗等工作，实现全流程管理，促进出院患者全面康复，我们组织制定了《新冠肺炎出院患者健康管理方案（试行）》。现印发给你们，请参照执行。

国家卫生健康委办公厅

2020年3月13日

（信息公开形式：主动公开）

新冠肺炎出院患者健康管理方案（试行）

为进一步做好新冠肺炎患者治愈出院后的隔离管理、随访复诊、健康监测、康复医疗等工作，实现全流程管理，促进出院患者全面康复，特制定本方案。

一、职责分工

各地卫生健康行政部门要加强对新冠肺炎患者出院后健康管理的统筹协调，定点医院、隔离场所、康复医疗机构、基层医疗机构密切配合，加强信息沟通，协同做好新冠肺炎患者出院后隔离管理、随访复诊、健康监测、康复医疗等工作。新冠肺炎患者治愈出院后，应当继续隔离进行14天医学观察和健康管理。

二、出院前准备

定点医院要严格执行《新型冠状病毒肺炎诊疗方案（第七版）》出院标准和出院后注意事项。患者出院前要对其临床症状、体征、实验室与影像学检查结果等综合评估，明确后续跟踪随访事项。要为出院患者安排好2～4周的随访复诊计划。

三、出院交接

出院后以居家隔离为主。定点医院要及时将出院患者信息推送至患者辖区或居住地居委会和基层医疗机构，基层医疗机构要指导出院患者及家属按要求做好隔离管理和自我健康监测。湖北省武汉市等设有集中隔离点的地区，卫生健康行政部门要指导定点医院与集中隔离点、基层医疗机构做好衔接。

四、隔离管理

出院患者应进行严格居家隔离，尽可能居住在通风良好的单人房间，并减少与家人的密切接触。做到分餐饮食，做好手卫生和日常清洁，避免外出活动。湖北省武汉市等设有出院患者集中隔离点的地区，隔离期间要做好出院患者医学观察、康复、照护等服务。

五、出院后随访复诊和健康监测

出院患者要按照复诊计划在定点医院进行复诊，一般在患者出院后第2周、第4周进行。各有关医疗机构和集中隔离点要密切关注出院患者健康状况，对老年人和有基础疾病的出院患者要特别加强健康状况监测，一旦发现出院患者出现发热、咳嗽等临床表现，应尽快将其转至定点医院进一步治疗。

六、康复管理

各地要结合本地实际，按照《新冠肺炎出院患者康复方案（试行）》要求提供康复医疗服务。在患者较为集中的地区要安排基层医疗机构承担社区康复任务。

七、应用信息技术和平台

各地要依托区域卫生信息平台，努力做到居民健康档案、电子病历、出院健康监测等信息共享和业务协同，实现新冠肺炎患者临床诊治与健康管理的无缝衔接。全科医生和乡村医生要通过家庭医生签约APP、有线电视网络等多种手段与辖区内管理的出院患者开展信息互动，通过“互联网+”等形式，为出院患者提供健康管理服务。

1. 广电总局：发布统筹疫情防控和推动广播电视行业平稳发展有关政策措施 广电发〔2020〕17号

各省、自治区、直辖市广播电视局，新疆生产建设兵团文化体育广电和旅游局，总局机关各部门、直属各单位，中央广播电视总台办公厅，中国教育电视台，电影卫星频道节目制作中心：

为深入贯彻落实习近平总书记在统筹推进新冠肺炎疫情防控和经济社会发展工作部署会议上的重要讲话精神以及党中央、国务院各项决策部署，支持广播电视和网络视听行业积极开展疫情防控、有序做好复工复产、实现行业平稳发展，现就有关政策措施通知如下：

一、加强疫情防控和复工复产的宣传舆论引导支持。加强顶层设计和科学策划，统筹全国广播电视和网络视听媒体，用好全国广播电视宣传工作例会、舆情会商、议题设置、宣传调控等机制，深度聚焦主题，通过新闻报道、专题节目、纪录片、短视频、公益广告和文艺作品等多种形式，综合运用多媒体、多元素、新手段、新技术，全方位、全过程宣传阐释习近平总书记重要讲话和指示批示精神，宣传解读党中央、国务院统筹推进疫情防控和经济社会发展重大决策部署、各地疫情防控举措、复工复产进展、科学防疫知识、抗疫一线工作者感人事迹等。有步骤、有重点地开展系列宣传报道，递进式深化、立体式推动，打总体战、出组合拳，做亮主题宣传，做活典型宣传，做深成就宣传，为夺取疫情防控和实现经济社会发展目标双胜利提供有力支持、营造良好氛围。

二、加大对内容创作生产传播的支持力度。围绕统筹推进疫情防控和经济社会发展重大决策部署，指导创作生产一批优秀的电视剧、纪录片、动画片、网络影视剧、电视节目、公益广告、MV、短视频等，优先列入重点选题和资金扶持范围，并通过现有资金渠道，调整优化结构，统筹存量资金，加大资金扶持力度。加强对外宣传，讲好中国抗疫故事，通过“丝绸之路影视桥工程”“中国当代作品翻译工程”等项目，将抗击疫情的优秀作品纳入对外译制推广的支持范畴。加大扶持力度，继续做好优秀电视剧、动画片、纪录片、广播剧、少儿节目等的捐赠工作。

三、优化业务审批流程和方式。进一步推进“互联网+政务服务”，加快推进“不见面审批”，促进“线下办”为主向“网上办”“掌上办”为主转变，逐步实现重点电视剧、网络影视剧等通过“线上+邮寄”办理备案审批。研究推进广播电视节目制作机构、电视剧制作机构审批改革，更好地引导、管理和服务制作机构。进一步加强节目制作统筹管理，研究对突发事件等特殊题材电视剧审批的绿色通道。做好广播电视设备器材入网认定审批，进一步推行网上办理，优化办理流程，缩短审批时限，对在疫情防控期间广播电视设备器材入网认定证书到期的，有效期顺延至申请企业所在地疫情解除。探索推行“告知承诺”“容缺受理”等审批方式，推进简化审批程序、压缩审批时限、延长许可证有效期等优化审批服务的举措。强化事中事后管理，创新监管手段，建立健全信用监管制度。充分运用数字版权保护、区块链、人工智能等技术，提升内容审核效率，实现在线安全智能审核。

四、进一步提升政务服务效率和水平。通过网上办公、视频会议等方式，做好电视剧、纪录片、动画片、网络视听节目等的主题策划、创作研讨、专家评议、专题培训等工作。加快各类行政审批服务平台的统一和规范化，加快政务大厅办理平台与行政审批平台的对接。推进广播电视编辑记者播音员主持人资格考试合格证、播音员主持人资格证对接广电总局电子证照平台工作，开通电子证照网上查询下载功能，取消纸质资格考试合格证。建设“飞天奖”“星光奖”评奖评审应用系统，组织开展线上申报评奖工作。探索和推进“广电智慧学院”建设，充分利用“互联网+”组织开展培训，丰富和拓展网络培训资源和精品课程。

五、统筹提升广播电视应急能力。及时对接国家应急管理、公共卫生防疫、生物安全、野生动物保护等法律法规的修订，将此次疫情防控中广电领域相关经验和举措体现在国家层面立法之中。在《广播电视管理条例》和《信息网络传播视听节目管理条例》制修订中，增加完善应急管理相关内容。充分利用广电总局高清电视电话会议系统、安全播出指挥调度平台等网络化、信息化手段，拓展指挥调度平台应用场景，加强在全国广播电视安全保障工作季度例会、重大事件事故调查、安全播出操作备案等日常安全播出工作中的使用力度。加强一线安全播出场所的疫情防控措施，完善应急预案。

六、加快完善应急广播体系建设。研究制订《应急广播管理暂行办法》。加快深度贫困县应急广播体系建设，克服疫情影响，尽早建成发挥效益。进一步完善国家应急广播调度控制平台，尽快实现应急广播平台与应急发布部门之间、国家级和省级平台之间的对接和联通。积极争取中央财政扩大基层应急广播建设补助范围。积极推动地方各级广电部门建设完善本级应急广播平台和传输覆盖系统，满足各级党委政府和行业部门管理和服务需要，助力提升基层治理体系和治理能力。

七、加快推动全国有线电视网络整合和广电5G建设一体化发展。贯彻落实《全国有线电视网络整合发展实施方案》，加快推动全国有线电视网络整合和广电5G建设一体化发展，抓紧组建“全国一网”股份公司，加快推进广电5G核心网、承载网、城市试验网建设和商用步伐，支持利用5G技术对有线电视网络进行改造升级，实现居民家庭有线无线交互，大屏小屏互动，加快形成富有广电特色的市场应用场景和可持续的盈利模式，形成行业发展新增长点。贯彻落实加快5G网络、数据中心等新型基础设施建设决策部署，积极争取国家政策支持。根据疫情防控和复工复产助学需要，指导中国广电优先在疫情严重和相关重点地区布局推动广电5G建设。

八、强化“智慧广电”新产品、新业态、新模式支持。紧紧抓住疫情防控带来的数字文化、在线消费、智慧管理等方面的迭代升级、变革重构机遇，深入实施“智慧广电”战略，以加快推进全国有线电视网络整合和广电5G建设一体化发展、建设新一代战略文化基础设施为突破口，充分利用物联网、大数据、云计算、人工智能等技术，促进高新视听业务、内容、平台、网络、终端的共融共通，全面提升综合信息服务能力。加快发展超高清视频、虚拟现实、可穿戴设备等新型信息产品，推动居民家庭文化消费升级。鼓励各级广电部门更好发挥政府作用，支持引导广电企业在“雪亮工程”、空中课堂、智慧城管、智慧养老、电视会议、远程医疗等业务场景应用中，更多更好链接融合政府资源、社会资源、生产资源、生活资源，拓展政用、民用、商用领域的服务形态，在疫情防控和复工复产中多渠道发挥作用。

九、加强产业政策支持引导。鼓励广电总局认定命名的湖北等地国家级产业基地（园区）在疫情期间为入驻企业提供减免租金、困难帮扶等优惠措施，对正在向广电总局申报且符合条件的产业基地（园区）在认定管理中予以优先支持。鼓励支持疫情防控中发挥特殊作用的新业态、新模式相关产业项目申报国家广播电视和网络视听产业发展项目库，采取多种方式推动项目合作和资金对接，为优质项目建立多元投融资渠道。指导有关展会活动主办方组委会做好应对疫情的工作预案，在举办时间、场馆条件、招商招展、配套活动、宣传引导等方面积极协调支持。向国家有关部门积极反映行业制作机构、播出机构、传输机构等在疫情防控中发挥的重要作用、面临的实际困难，积极争取专项扶持政策。

十、加强对公共服务体系建设和脱贫攻坚的支持。积极争取中央预算内投资支持，推动智慧广电公共服务设施提档升级，提升广播电视惠民工程实施效果。加强对各类广播电视设施设备的检查、维护、维修、监测，确保安全播出和正常运转，确保广播电视基本公共服务有效提供。鼓励采取政府购买服务、联合采购等多种形式，支持各级广播电视和网络视听服务机构在一定期限内通过增加节目、开放权限、节目联播等方式增加公共服务供给，满足疫情防控期间人民群众显著增长的精神文化需求。充分发挥广播电视和网络视听优势，充分利用“公益广告、节目+扶贫”“短视频、直播+扶贫”开拓产业扶贫、消费扶贫渠道，帮助贫困地区解决生产发展和产品积压难题。

十一、用足用好中央和地方减税降费、金融扶持、社会保障等扶持政策。积极贯彻落实、用足用好中央和地方出台的各类扶持政策，指导各类广电企事业单位协调落实有关减免缓缴税款、社会保险费、住房公积金、房租等各项优惠帮扶政策措施，积极做好有关政策宣传、沟通对接，主动做好服务，进一步减轻企事业单位负担。落实好有关延期分期还款、贷款展期、减免小微企业贷款利息等政策措施，纾解企业资金压力。落实国有企业承担政府疫情防控保障任务的工资分配激励、经营业绩考核政策，充分调动和发挥国有企业积极性和作用。支持各级各类行业协会充分发挥专业优势和会员单位资源优势，加强政策宣传、搭建沟通渠道、提供咨询服务，充分调动各个渠道力量投入疫情防控和复工复产工作。

十二、鼓励激励党员干部担当作为。充分发挥基层党组织战斗堡垒作用和党员先锋模范作用，带头做好舆论引导，带头落实防控措施，带头服务职工群众，带头严格遵守纪律，让党旗在疫情防控斗争第一线高高飘扬。推动实施广播电视和网络视听行业领军人才和青年创新人才工程，在推荐选拔中充分考察人选在疫情防控期间工作表现，优先推荐在疫情防控一线做出突出贡献的人选。

国家广播电视总局

2020年3月12日

1. 民政部业务主管社会组织：进一步在统筹推进疫情防控和经济社会发展工作中积极作为 民社管函〔2020〕21号

民政部业务主管社会组织：

新冠肺炎疫情发生以来，民政部业务主管社会组织（以下简称“部管社会组织”）认真贯彻落实党中央、国务院决策部署和民政部党组工作要求，主动参与疫情防控工作，充分发挥各自优势和特长，在一线和后方奋勇战“疫”，发挥出了部管社会组织的示范引领作用,彰显了社会组织的强大力量。经过全国上下艰苦努力，当前已初步呈现疫情防控形势持续向好、生产生活秩序加快恢复的态势。为深入贯彻落实习近平总书记关于统筹推进新冠肺炎疫情防控和经济社会发展工作的指示精神，进一步推动部管社会组织在疫情防控和经济社会发展中积极作为，持续发挥示范引领作用，通知如下。

一、坚决贯彻落实重要讲话精神

为统筹推进新冠肺炎疫情防控和经济社会发展工作，党中央、国务院多次召开工作会议，研究部署当前疫情防控工作和稳定经济社会运行重点工作。习近平总书记在2月23日的统筹推进新冠肺炎疫情防控和经济社会发展工作部署会、3月4日的中央政治局常务委员会会议、3月6日的决战决胜脱贫攻坚座谈会上作了重要讲话，为我们统筹做好当前疫情防控和经济社会发展各项工作指明了努力方向、提供了根本遵循、增加了前进动力。各部管社会组织务必认真学习、深刻领会、迅速贯彻，切实为巩固和拓展来之不易的疫情防控良好势头，力争为全国经济社会发展早日全面步入正常轨道，为实现决胜全面建成小康社会、决战脱贫攻坚目标任务作出积极贡献。

二、坚决抓实抓细疫情防控工作

疫情防控工作虽然取得了阶段性成果，但是疫情防控任务依旧艰巨繁重，部管社会组织要保持头脑清醒，慎终如始，再接再厉，善作善成，继续把疫情防控作为当前头等大事和最重要的工作，不麻痹、不厌战、不松劲、毫不放松抓紧抓实抓细各项防控工作。严格执行当地党委政府及有关行业主管部门的疫情防控规定，全面压实防控主体责任，及时调整完善本单位防控措施，持续抓好本单位的疫情防控工作。落实民政部党组关于加强基本民生保障工作的要求，结合自身业务特点，积极为孤寡老人、困难儿童、重病重残人员等困难群体提供援助、生活救助等服务，做好困难群众的防护工作。主动协助城乡社区做好联防联控，构筑群防群治的严密防线。

三、坚决推进有序复工复产工作

在当前已初步呈现疫情防控形势持续向好、生产生活秩序加快恢复的态势下，部管社会组织要从实际出发，在抓住主要矛盾和矛盾的主要方面基础上“弹好钢琴”，统筹疫情防控和事业发展，稳妥做好本单位复工复产，积极助力其他部门复工复产，为经济社会发展贡献力量。行业协会、商会要指导和帮助企业等会员单位科学精准防疫、有序复工复产。志愿服务组织要发挥社会工作的专业优势，支持广大社工、义工和志愿者开展心理疏导、情绪支持、保障支持等服务。慈善组织要高效运转，增强透明度，主动接受监督，让每一份爱心善意都及时得到落实。

四、坚决助力决战决胜脱贫攻坚

2020年是脱贫攻坚的全面收官之年，突发新冠肺炎疫情又带来新的挑战，部管社会组织要进一步提高政治站位，努力克服疫情影响，把党中央和民政部党组关于脱贫攻坚的各项决策部署贯彻落实好，为确保如期打赢脱贫攻坚战作出应有贡献。一是要发挥专业技术优势，参与落实贫困地区特色产业发展规划，实现精准对接，为贫困地区特色产业发展提供智力和技术支持。二是要发挥动员资源优势，灵活开展扶贫公益活动，动员更多慈善力量投身脱贫攻坚，加快慈善项目落地。三是要发挥志愿服务优势，为贫困人口提供生活帮扶、能力提升、权益保障、生计发展等社工专业服务，帮助贫困群众转变思想观念、树立脱贫信心、拓宽致富道路。四是要规范有序参与，严禁打着扶贫旗号从事与扶贫无关的活动，配合做好相关工作检查和信息统计，接受社会各方监督。

民政部社会组织管理局 民政部社会组织服务中心党委

2020年3月13日

1. 卫健委：进一步加强疫情期间医疗机构感染防控工作 国卫办医函〔2020〕226号

各省、自治区、直辖市及新疆生产建设兵团卫生健康委：

为指导各地在有序开展医疗服务的基础上，进一步强化医疗机构感染防控工作，最大限度减少交叉感染，现就疫情期间医疗机构感染防控工作有关要求通知如下：

一、结合分区分级管理，加强区域部署和指导落实

各级卫生健康行政部门要高度重视疫情期间医疗机构感染防控工作，把做好预防和控制院内感染作为疫情防控工作的重中之重，以及开展日常医疗服务的基础和前提。要根据辖区内新冠肺炎的流行程度和区域风险等级，加强区域内协调部署，毫不放松落实感染防控各项要求。要加强对辖区内医疗机构感染防控工作的现场指导，及时发现问题、纠正错误，既避免防控不足，也避免防控过度。对存在问题较多的医疗机构，要建立整改台账，实行限时销号管理。

二、严格落实标准预防，采取相应防护措施

医疗机构要严格落实标准预防，进入医疗机构的各类人员均应当正确选择和佩戴口罩、正确进行手卫生。医疗机构做好环境通风管理，落实分区管理要求，合理划分清洁区、潜在污染区和污染区，区别医务人员通道和患者通道。医务人员防护按照《新冠肺炎疫情期间医务人员防护技术指南（试行）》（国卫办医函〔2020〕155号）执行，正确合理使用防护用品。在标准预防的基础上，根据诊疗操作的风险高低进行额外防护。可能接触到患者血液、体液、分泌物或实施产生气溶胶操作时，选择佩戴护目镜/防护面屏、隔离衣、医用防护口罩等防护用品。

三、加强门急诊预检分诊，落实“四早”要求

医疗机构要落实《医疗机构传染病预检分诊管理办法》《互联网诊疗管理办法》等要求，做好患者的分流和风险管控。通过互联网在线咨询，重点询问是否存在发热、咳嗽等呼吸道疾病症状体征，以及流行病学史，初步判断就诊科室，为患者提供分时段预约诊疗，减少现场挂号就诊。在门急诊规范设置预检分诊点，对患者进行体温筛查，对发热患者转移到发热门诊就诊。发热门诊医生对患者进一步询问疫情高风险国家或地区旅行或居住史，检查新冠肺炎相关症状体征，对可疑新冠病毒感染者进行影像学和实验室检测，做到早发现、早报告、早隔离、早治疗。医疗机构的急诊可设立缓冲区域，对需要急诊急救治疗且不能排除新冠肺炎的患者进行隔离收治。

四、加强患者收入院管理和住院患者管理

医疗机构要根据本区域的风险等级，严格执行本地人民政府关于“四类人员”（确诊病例、疑似病例、发热症状患者、密切接触者）相关管理要求，制定本机构疫情期间患者入院筛查流程。对有流行病学史、存在新冠肺炎感染风险的入院患者，可通过影像学、病原学和血清学检测方法作进一步鉴别诊断。鼓励中、高风险地区有条件的医疗机构设置过渡病房，对新收入院的患者进行单间收治，待排除新冠病毒感染后再转至常规病房进一步住院治疗，降低潜在院内交叉感染风险。患者住院期间要加强陪护人员、探视人员的管理，根据本地区的疫情流行情况制定陪护、探视的管理制度。

五、做好院内感染监测和健康监测

医疗机构要开展感染防控的主动监测，切实做好急诊患者、血液透析、肿瘤放疗化疗患者等重点人群的感染监测工作，对手术治疗、内镜操作等侵入性操作环节实现监测全覆盖。通过主动监测，及时发现散发感染病例、聚集性感染病例和疑似新冠肺炎患者的情况，采取相应防控和调查措施。医疗机构要建立健康状况强制报告制度，要求全体医务人员、其他工作人员（包括保洁、配送、保安、护工等）、患者和陪护人员每天或定期报告个人健康状况，及时发现发热及有呼吸道症状的人员并采取相应措施。

六、细化重点人群医疗服务流程

对于孕产妇、急诊手术患者、急性心脑血管疾病患者、血液透析患者、恶性肿瘤患者等人群，各级各类医疗机构要重点保障其医疗需求，因地制宜制定针对性的诊疗流程和应急预案。对于急诊患者且不能排除新型冠状病毒感染时，可按照疑似患者收治和防护，保证患者和医务人员安全。对于门诊慢性病患者，除视病情延长处方用量外，鼓励互联网医院开展线上复诊，鼓励医疗机构开展线上咨询和就医指导，并做好药品的供应保障。

七、做好日常的医疗机构感染防控

各级卫生健康行政部门要将感染防控工作作为考核医疗机构的标准之一，加强日常监督管理。医疗机构要认真落实《医疗机构感染预防与控制基本制度（试行）》（国卫办医函〔2019〕480号），加强感控专兼职人员的配备，加大感控经费投入，落实各级感控管理组织的责任。严格开展全员岗前感染防控培训工作，未经培训合格不得上岗。按照相关技术标准和规范，使重点部门的建筑布局和工作流程符合要求，并配备必要的设备设施，为医务人员提供方便的洗澡等清洁条件。将标准预防理念贯穿到日常各项工作，做好消毒、隔离、预防职业暴露、医疗废物管理等工作，降低院内感染发生风险。

国家卫生健康委办公厅

2020年3月13日

1. 农业农村部：进一步优化审批服务推动农业企业加快复工复产 农办法〔2020〕2号

各省、自治区、直辖市农业农村（农牧）厅（局、委），部行政审批业务司局、直属有关单位：

为深入贯彻落实《国务院办公厅关于进一步精简审批优化服务精准稳妥推进企业复工复产的通知》（国办发明电〔2020〕6号）要求，深化农业“放管服”改革，为农业企业复工复产提供优质高效的审批服务，切实保障农资有效供应和农业安全生产，现就有关事项通知如下。

一、充分认识优化审批服务推动农业企业复工复产的重要意义

近日，习近平总书记就全国春季农业生产工作作出重要指示，强调要在严格落实分区分级差异化疫情防控措施的同时，全力组织春耕生产，确保不误农时，保障夏粮丰收。李克强总理也作出批示指出，要推动农资企业加快复工复产，抓好蔬菜、畜禽等生产，确保农业生产平稳发展，为打赢疫情防控阻击战，实现今年经济社会发展目标任务提供有力支撑。各级农业农村部门要切实把思想和行动统一到党中央、国务院决策部署上来，在坚决做好疫情防控工作的同时，依法保障和积极推动农资、养殖业、农业龙头企业等尽快复工复产，努力夺取疫情防控和农业农村经济发展“双胜利”。要进一步深化农业“放管服”改革，转变政府职能，优化营商环境，持续推进许可事项取消下放、优化整合，不断规范审批事项和行为，及时协调解决复工复产中审批工作存在的问题和困难，为农业企业复工复产提供便利服务。

二、简化审批手续，优化办事流程

各级农业农村部门要压实属地管理责任，按照分区分级原则，采取差异化审批。低风险地区不得采取审批、备案等方式延缓开工；中、高风险地区，对列入省政府清单管理的审批和证明事项，要明确办理程序、材料和时限，清单之外一律不得实施审批或索要证明，防止出现层层加码、互为前置审批、循环证明等现象。疫情防控期间，农业农村部负责的换发兽药产品批准文号的审批事项申请，临时免除现场检查、抽样和复核检验程序；进口饲料和饲料添加剂登记申请，临时免除样品复核检验程序；境外贸易商申请农业转基因生物安全证书（进口）的审批事项，专家评审环节临时取消现场评审；从国外引进农作物种子、苗木检疫审批事项，省级意见可后期补交。地方农业农村部门要建立协调机制，加强与有关部门协作配合，为具备复工复产条件的农业企业开辟绿色通道，提供方便、创造条件。为加快种子、农药、化肥、兽药等农资企业复工复产，地方农业农村部门要巩固“证照分离”改革成果，通过备案、告知承诺、精简不必要的证明等措施，简化审批手续，最大限度优化办事流程。

三、压缩审批时间，提高审批效率

各级农业农村部门要进一步压缩审批时限。审批过程需要组织现场勘验、技术检验、专家评审的，应积极统筹行政资源，广泛采取企业提供现场核查视频资料、网上并行技术审查、视频论证会等方式，尽量缩短审批过程，为农业企业复工复产争取时间。分级分类采取“特事特办”“急事急办”，创新审批“绿色通道”，对兽用消毒剂类产品批准文号申请实行优先审批，即报即办，确保满足畜禽养殖场防疫急需。农业农村部负责的农作物种子生产经营许可证变更申请、进口饲料和饲料添加剂变更登记事项办理时限进一步压缩1/3以上。地方农业农村部门要加快种子、农药、饲料、兽药等生产经营许可证的审批发放，对只需进行书面资料审查的，原则上应当场作出决定；对相关许可证延续、变更等申请，应明确期限尽快办理；对有关初审事项，应做到审批结果即审即报，初审意见通过网上审批系统“先行一步”，减少运转时间，提高审批时效。

四、推进网上办事，创新服务方式

各级农业农村部门要积极推动行政许可事项特别是复工复产重点事项网上办、掌上办，实现“不见面”审批。及时梳理相关惠农利企政策措施和审批服务事项，主动将疫情防控、复工复产等专题服务接入一体化国家政务服务平台，为企业和群众获取疫情防控信息、办理复工复产等提供便利。引导企业和群众优先选择网上办事、网上咨询等方式，减少实体政务服务大厅人员聚集。通过网上专家评审会、网上提交电子材料等信息化手段作出审批决定，提高审批效率。在兽药产品批准文号和说明书审批事项全程电子化的基础上，推动研制新兽药使用一类病原微生物审批、从国外引进农业种子苗木检疫审批事项实现全程电子化。推动发布渔业捕捞许可证和执业兽医师资格证书电子证照标准，充分运用统一身份认证、电子证照、电子签章等新型电子政务手段，加快实现权威高效的数据共享，提高审批效能。

五、加强审批监管，维护企业权益

各级农业农村部门要聚焦农业企业复工复产过程中面临的实际困难，有针对性地靠前服务，建立健全复工复产诉求响应机制。严格禁止在法律法规外增加许可事项、增设许可条件，防止设置过高门槛限制和影响复工复产。农业农村部负责的农药、肥料、饲料和饲料添加剂进口登记证书到期但因疫情影响无法按时提交续展申请的，可延期提交；农药、肥料、饲料和饲料添加剂进口登记证书到期但因疫情影响无法按时提交续展申请材料（包括样品）或完成续展登记的，由申请人在网上提交续展申请表和相关材料（样品）合格承诺书，经审查后公告允许延长相关登记证的有效期限。地方农业农村部门应围绕许可证书到期延续、变更、换发等具体情形，有针对性地提出容缺受理、延期办理、承诺制办理等有效措施，促进复工复产企业依法依规开展生产。

农业农村部办公厅

2020年3月12日

1. 人社部 扶贫办：进一步做好贫困劳动力返岗复工“点对点”服务 人社厅明电[2020]17号

各省、自治区、直辖市及新疆生产建设兵团人力资源社会保障厅（局）、扶贫办（局）：

为深入贯彻落实习近平总书记在统筹推进新冠肺炎疫情防控和经济社会发展工作部署会议和决战决胜脱贫攻坚座谈会上的重要讲话精神，有针对性地做好贫困劳动力返岗复工“点对点”服务，确保贫困劳动力优先返岗复工，稳定贫困家庭收入，促进脱贫攻坚战决战决胜，现将有关事项通知如下：

一、实施有序返岗。人社部门要认真履行返岗复工“点对点”服务保障职能，输出地扶贫部门要向人社部门提供贫困劳动力信息摸查成果，实现信息共享；输入地人社部门要详细了解企业复产复工的用工需求，提供岗位；输出地、输入地要做好劳务对接工作。采取多种途径推广使用农民工返岗复工点对点出行服务小程序和农民工返岗复工点对点用工对接服务平台（http://wgfw.mohrss.gov.cn），支持企业和农民工自行填报需求，有针对性地开展劳务输出。

二、打通“最先一公里”。要想方设法解决贫困劳动力“出村难”问题，统一安排，组织专门交通工具，从各村镇等劳动力分散的居住点接送外出务工人员到县城或火车站、机场集中，实现从家门到贫困劳动力返岗复工“点对点”专车、专列、包机和工厂大门的无缝衔接，确保安全运送。

三、互认健康信息。要积极配合卫生健康部门做好贫困劳动力的健康检查和新冠肺炎防疫宣传，推广使用全国一体化政务服务平台防疫健康信息码，加强输出地与输入地健康信息的互认，简化健康证明材料，做到既应隔尽隔、平安返岗，又不重复隔离、增加企业和个人负担，尽可能缩短返岗复工时间。

四、保障防护物品。积极帮助复产复工企业制定和完善贫困劳动力上岗防护措施，对企业购置防护物品确有困难的，要主动协调做好口罩、消毒液等防疫物资的保障工作，严格做好员工吃、住、行和车间管理等环节防疫工作，增强企业防护保障能力，确保员工安全健康的生产生活环境。

五、突出工作重点。各地要将“三区三州”等深度贫困地区、易地扶贫搬迁安置点以及挂牌督战的52个贫困县作为重点，优先为贫困劳动力提供“点对点”返岗复工服务。对贫困劳动力通过有组织输出到户籍所在县以外就业的，给予一次性求职创业补贴，可从就业补助资金中列支。适当安排财政扶贫专项资金用于组织稳定贫困人口就业岗位。对无法外出的贫困劳动力，通过鼓励扶贫车间等带贫主体吸纳等渠道促进其就地就近就业增收。充分发挥贫困村创业致富带头人的作用，加大培训力度，提高吸纳贫困劳动力就业能力。

六、推进部门联动。各地要清醒认识当前疫情防控和经济社会发展形势的复杂性，统筹做好疫情防控和贫困劳动力返岗复工“点对点”服务保障，积极会同公安、交通运输、卫生健康等部门，密切协同配合，形成工作合力，努力减轻疫情对贫困劳动力就业增收的影响，为实现决战脱贫攻坚目标任务奠定基础。

人力资源社会保障部办公厅

国务院扶贫办综合司

2020年3月12日

1. 交通运输部：精准有序恢复运输服务扎实推动复工复产 交运明电〔2020〕95号

各省、自治区、直辖市、新疆生产建设兵团交通运输厅（局、委）：

为贯彻落实国务院关于统筹新冠肺炎疫情防控和经济社会发展精准有序扎实推动复工复产工作部署，精准有序恢复运输服务，有力支撑复工复产、经济社会发展和生产生活秩序恢复。现就有关事项通知如下：

一、加强运输服务需求动态研判

（一）认真研判运输服务动态需求。随着全国新冠肺炎疫情防控形势积极向好，企业复工复产将快速推进，由此带来的人员流动和物资运输需求将快速增长。各地交通运输主管部门要组织专门力量，加强运输需求变化趋势动态研判，指导运输企业制定专门工作方案，提前做好运力准备工作，扎实做好农民工运输、生产物资和原材料运输、农业生产资料运输，确保不因运输环节影响企业复工复产。

（二）精准对接重点群体运输需求。各地交通运输主管部门要针对将来一段时期全国范围内援鄂医疗队返回、大学生返校、农民工返城等重点运输任务，积极主动与当地卫健、人社、教育、文旅等部门，以及高等院校、重点用工企业沟通协调，及早对接运输需求，精细做好运输服务保障。

二、分区分级精准恢复运输服务

（三）坚持分区分级恢复运输保障。各地交通运输主管部门要在当地疫情防控机制领导下，严格落实《交通运输部关于分区分级科学做好客运场站和交通运输工具新冠肺炎疫情防控工作的通知》（交运明电〔2020〕84号）要求，分区分级精准恢复道路客运和城市公共交通服务。低风险地区，要全面恢复城乡道路运输服务。中风险地区，要在做好疫情防控工作前提下，尽快恢复城乡道路运输服务。高风险地区，要采取有效措施保障医护人员、公共事业运行一线人员等重点群体必要出行，稳妥有序恢复交通运输服务。随着返城人员增多，中、高风险地区，要结合疫情防控形势变化情况，有序保障高铁站、机场、公路客运站等枢纽接驳运输服务。

（四）保障重点地区人员安全有序流动。各地交通运输主管部门要按照中央统一部署，继续实施严格的离汉离鄂通道管控和进京管理。对于武汉市以外的湖北其他地区，在做好健康管理、落实防控措施的前提下，采取“点对点、一站式”运输方式，集中精准、安全可控地做好运输保障，帮助外地滞留在鄂人员逐步有序返回。

三、科学合理控制客座率

（五）分区分级控制客座率。各地交通运输主管部门要督促指导运输企业按照《客运场站和交通运输工具新冠肺炎疫情分区分级防控指南》相关要求，科学合理控制客座率。出入中、高风险地区（不含途经）的省际、市际客运班车、包车，继续将客座率控制在50%以内。高风险地区城市公共汽电车每平方米不得超过4人，中风险地区每平方米不得超过6人。高风险地区城市轨道交通满载率要控制在50%以内，中风险地区要控制在70%以内。低风险地区之间的“点对点”运输任务，可逐步有序放开客座率限制要求。

四、加强入境人员交通运输防控

（六）严格入境人员交通运输防控。各地交通运输主管部门要主动对接当地外事、海关、移民管理、民航、口岸等部门，加强部门协同联动和信息共享，建立应急疏运保障车队，指导运输企业科学合理制定运输方案，根据不同规模、不同目的地旅客转运需求，灵活组织运力，分类做好境外返回人员疏运保障工作。要按照高风险地区的疫情防控标准，严格落实车辆消毒、通风、运输组织、人员防护等措施，进一步细化疫情防控措施，坚决遏制入境人员境内转运过程中通过交通运输工具交叉感染风险，严防疫情境外输入。

五、加快推动物流园区复工复产

（七）分区分级推动物流园区复产。各地交通运输主管部门要按照国务院统一部署，切实把物流园区复工复产提高到更高优先级。低风险地区物流园区要全面复工复产，鼓励引导尚未返城的农民工在就近物流园区择业。中、高风险地区内，对于影响全国范围内物资集散和不同运输方式转运的主要物流枢纽和重点物流园区，要在加强园区从业人员防护、采取通风消毒等防疫措施的基础上，逐步有序恢复生产，确保不因枢纽结点衔接不畅影响物流运输总体效率。

六、加快推动维修驾培行业复工复产

（八）推动机动车维修行业复工复产。各地交通运输主管部门要对执行疫情防控、应急运输等车辆开通维修救援“绿色通道”，优先予以重点保障。低风险地区要全面恢复维修作业，有效满足人民群众修车需求。中、高风险地区要充分发挥全国汽车维修电子健康档案系统作用，鼓励维修企业和互联网配件销售平台融合发展，采取电话、网络预约等便民措施提前预约，减少车主在维修点聚集和等待时间。鼓励开展线上车辆故障诊断咨询、车辆技术维护提示等非接触式维修。

（九）推动驾培培训行业复工复产。各地交通运输主管部门要结合本地区疫情形势变化，认真研判、精准施策，分区分级分类有序推动驾培机构复工复产。低风险地区要根据本地疫情防控要求，在做好卫生防疫防护措施的前提下，全面恢复所有科目驾驶培训。中、高风险地区要科学合理制定复工复产计划，做好人员信息登记、体温检测、教练员和学员防护、教学车辆和教学设施设备消毒等工作，逐步有序恢复部分科目培训。鼓励推行理论课程远程网络教学和实际操作技能“一人一车”的培训教学服务。

七、创新推出便利经营措施

（十）积极推广网上办理。各地交通运输主管部门要积极推广运输服务业务网上办理，充分发挥全国互联网道路运输便民政务服务系统作用，开展道路运输业务网上办理，优化办理流程，对普通货运车辆年审、道路运输证换发、从业资格证审验换证等相关运政业务一网通办，切实做到便民利民。

（十一）提升车辆技术管理便民服务水平。鼓励汽车生产企业和检测机构通过网上平台申报达标车型。技术支持单位要集中力量加快技术审查，主动协调解决问题，做好技术服务工作，确保达标车型申报一个、审查一个、发布一个。统筹考虑疫情影响、汽车生产企业和检测机构复工复产现状，《营运货车安全技术条件 第1部分：载货汽车》第二阶段实施条款、《营运货车安全技术条件 第2部分：牵引车辆与挂车》第一阶段实施条款，从2020年5月1日顺延至2020年9月1日起实施。

八、推动落实运输企业扶持政策

（十二）推动落实财政金融优惠政策。各地交通运输主管部门要按照《交通运输部关于做好交通运输企业财税金融优惠政策落实工作的通知》（交财审明电〔2020〕79号）要求，会同相关部门将各项财政税收金融支持政策落到实处，帮助运输企业渡过难关。要指导货运企业和出租汽车企业及时与金融机构和融资租赁公司对接，落实货车司机、出租车司机等特殊群体个人经营性质贷款，可参照个体工商户实施临时性延期还本付息政策，以及融资租赁公司等机构根据自身经营实力和风险承受能力对企业和个人客户实施临时性延付租金等安排。

（十三）积极落实保险优惠政策。各地交通运输主管部门要会同银保监部门按照《关于做好新冠肺炎疫情防控期间车险服务有关工作的通知》（财险部函〔2020〕36号）、《中国保险行业协会关于落实新冠肺炎疫情防控期间机动车车险服务有关工作的通知》（中保协函〔2020〕37号）相关要求，指导运输企业主动对接保险行业协会和保险公司，通过双方或者多方协商的方式，明确受疫情影响较大的营运车辆，延长机动车交强险和商业险车险保单保障期限。鼓励社会化卫星定位系统监控平台服务商，降低或减免道路运输车辆卫星定位服务运维费用。

九、加大政策宣传引导力度

（十四）加大已出台政策宣传力度。各地交通运输主管部门要全面梳理交通运输疫情防控、复工复产和扶持政策，通过新闻媒体、行业协会、网络平台等渠道，加大政策宣传力度。中国通信信息中心以及交通运输新业态平台企业，要通过重点营运车辆联网联控系统、全国道路公共货运平台、交通运输新业态平台等信息化系统，及时将相关扶持政策传递到企业和驾驶员，引导企业和驾驶员按规定程序申请申报。鼓励中国交通运输协会、中国道路运输协会、中国物流与采购联合会、中国汽车工业协会、中国汽车流通协会、中国维修协会等行业协会，充分发挥与会员企业紧密联系优势，大力宣传国家出台的对交通运输的扶持政策以及在疫情防控、应急物资运输和复工复产中表现突出的会员单位。

十、确保行业安全稳定

（十五）加强行业安全监管。各地交通运输主管部门要督促道路运输经营者严格落实安全生产主体责任，强化对车辆和司乘人员的统一管理和动态监控，长途客运班车和包车严格执行凌晨2时至5时停车休息或接驳运输制度。要会同公安机关加大联合监管执法和信息共享力度，依法从严查处超载超速、疲劳驾驶、非法营运、违规异地经营等违法违规行为，切实消除安全生产隐患，坚决遏制重特大事故发生。

（十六）维护行业稳定。各地交通运输主管部门要深入了解运输服务企业困难，切实做好小微企业、货运司机、出租汽车司机等重点群体帮扶工作。引导出租汽车企业、行业协会和驾驶员、工会加强沟通协商，阶段性减免出租车“份子钱”，维护行业稳定。加强对货运物流行业监测分析，引导物流企业、货车司机有序复工复产，合理引导运输价格预期，促进行业健康稳定发展。

交通运输部

2020年3月13日

1. 民政部：发布《社区“三社联动”线上抗疫模式工作导引（第二版）》

为贯彻落实习近平总书记在统筹推进新冠肺炎疫情防控和经济社会发展工作部署会上“要推动防控资源和力量下沉，把社区这道防线守严守牢”、“要发挥社会工作的专业优势，支持广大社工、义工和志愿者开展心理疏导、情绪支持、保障支持等服务”的重要指示精神，充分发挥社区、社会组织、社会工作“三社联动”机制在社区防控中的协同作用，民政部基层政权建设和社区治理司、慈善事业促进和社会工作司、社会组织管理局联合指导中国社会工作联合会在《社区“三社联动”线上抗疫模式工作导引（第一版）》（以下简称《工作导引》第一版）的基础上，总结地方实践经验做法，修订完善形成了《社区“三社联动”线上抗疫模式工作导引（第二版）》（以下简称《工作导引》第二版）。

随着疫情防控工作的深入开展，加强居家生活保障、做好困难人群帮扶、强化心理疏导等工作成为社区防控中的重点。针对上述工作实际，《工作导引》第二版总结了地方实践中建立核心服务群和防控支持群的有益经验，既保留了《工作导引》第一版“发挥社会工作服务机构和社会工作者的专业优势，链接全国范围内的优秀心理服务专家等志愿者，通过线上抗疫平台，为重点对象提供心理疏导、情绪支持、危机干预、资源链接等服务”的内容；又重点细化了线上抗疫模式的各级各类微信群建立与运营机制，通过建立并完善三级核心服务群、四类线上防控支持群，发动社区居民参与，建立社区防控互助体系，引导包括社区居民在内的各方力量共同做好居家生活保障、困难人群帮扶等工作。同时，《工作导引》第二版回应了各地咨询提出的操作性问题，增加了群管理和群沟通技巧等内容。

现公布《工作导引》第二版，供各地在工作中参考使用。在使用过程中，中国社会工作联合会将提供公益性专业咨询支持。

2020.3.13

1. 商务部：统筹做好新冠肺炎疫情防控和商务扶贫工作

为深入贯彻习近平总书记关于新冠肺炎疫情防控和脱贫攻坚的重要讲话精神，认真落实党中央、国务院决策部署，近日，商务部印发《关于统筹做好新冠肺炎疫情防控和商务扶贫工作的通知》，要求各地商务主管部门、部属各单位认真学习贯彻习近平总书记重要讲话精神，切实把统筹疫情防控和商务扶贫各项工作抓实抓细抓落地，为坚决打赢疫情防控战和脱贫攻坚战贡献力量。

一、密切跟踪分析疫情对商务扶贫工作的影响，进一步贯彻落实精准扶贫精准脱贫基本方略，充分发挥商务扶贫优势和特色，指导推动贫困地区创新工作方法，因地制宜采取更有针对性的措施。

二、进一步强化电商扶贫、产销对接等举措落实，开展品牌推介洽谈活动、电商实操培训，加大力度动员和组织电商企业、大型农产品批发市场、公益性农产品示范市场、物流企业等与贫困地区积极对接，切实解决扶贫农畜牧产品滞销问题，多渠道解决农产品卖难问题。

三、加强对家政企业、外派劳务企业指导，强化与就业、交通等主管部门协作，了解贫困地区劳动力的基本情况，做好与贫困县劳务人员的对接，采取科学有效措施组织贫困地区劳务人员有序返岗，对暂不能返岗的，视情安排线上培训等。

四、继续运用广交会等展会平台支持贫困县企业拓展国际市场。推进国际合作扶贫项目。加强贫困县商务人才培训。

五、协调推动各项边贸政策落地，促进边境贸易创新发展。继续做好东部地区国家级经开区对口帮扶边合区、跨合区的对口帮扶工作。

六、落实好国务院扶贫办、商务部等部门《关于开展消费扶贫行动的通知》（国开办发〔2020〕4号）要求，深入开展消费扶贫。

七、加强对定点扶贫地区工作指导，加快推进定点扶贫项目，协助解决面临的困难和问题，督促履行脱贫攻坚主体责任，做好因疫致贫返贫人口的帮扶，巩固脱贫成果。

八、指导商务领域扶贫企业用足用好财税、金融、就业等方面的支持政策，有序复工复产，积极拓展国内外市场。

九、细化完善工作措施，特别要结合疫情应对，及时调整改进相关工作安排，努力将疫情对商务扶贫工作的不利影响降到最低。

十、加强调研指导，采取灵活多样的方式，密切与基层商务主管部门沟通联系，听取意见建议，及时发现商务扶贫工作落实中存在的困难和问题，积极推动解决。

十一、强化作风建设，巩固拓展“不忘初心、牢记使命”主题教育成果，持续深化商务扶贫专项巡视整改，坚决反对形式主义、官僚主义，切实减轻基层负担，提高工作实效。

十二、做好商务扶贫总结宣传，及时发掘提炼商务扶贫的实践经验、创新举措、典型做法，积极宣传商务扶贫先进事迹，关心关爱扶贫干部，营造更加积极向上的工作氛围。

2020.3.11

1. 国务院应对新型冠状病毒感染肺炎疫情联防联控机制：聚焦一线贯彻落实保护关心爱护医务人员措施 国发明电〔2020〕10号

各省、自治区、直辖市人民政府，国务院各部委、各直属机构：

近日，中央应对新型冠状病毒感染肺炎疫情工作领导小组印发《关于全面落实进一步保护关心爱护医务人员若干措施的通知》（国发明电〔2020〕5号），国务院办公厅转发卫生健康委、人力资源社会保障部、财政部《关于改善一线医务人员工作条件切实关心医务人员身心健康的若干措施》（国办发〔2020〕4号），国务院应对新型冠状病毒感染肺炎疫情联防联控机制多次研究部署，就疫情防控期间强化一线医务人员保护关心爱护措施提出明确要求，为广大一线医务人员全身心投入疫情防控工作提供了坚强保障、注入了强大动力、给予了关怀激励。但一些地方在执行和落实中，存在对象不精准、执行不严格、工作不规范、落实不到位等问题。为确保党中央、国务院保护关心爱护一线医务人员政策措施落到实处，现就有关事项通知如下：

一、关于保护关心爱护措施向一线医务人员倾斜。一线医务人员是指疫情防控期间按照政府统一部署、卫生健康部门调派或医疗卫生机构要求，直接参与新冠肺炎防疫和救治一线工作，且与确诊或疑似病例直接接触的接诊、筛查、检查、检测、转运、治疗、护理、流行病学调查、医学观察，以及直接进行病例标本采集、病原检测、病理检查、病理解剖的医疗卫生专业技术人员。一线医务人员以实际参加现场调查处置、患者救治等工作情况为准，不受编制、身份等限制。临时性工作补助、一次性慰问补助、卫生防疫津贴等要及时发放，要向一线医务人员特别是救治重症患者的医务人员倾斜，不得按行政级别确定发放标准。

二、关于临时性工作补助的计算和发放。按照人力资源社会保障部、财政部《关于建立传染病疫情防治人员临时性工作补助的通知》（人社部规〔2016〕4号）规定，一线医务人员领取临时性工作补助的天数，按其直接接触确诊或疑似病例、标本（含尸体解剖）的天数计算。对在重症危重症患者病区工作的一线医务人员，按实际工作天数的1.5倍计算应发工作天数。对在集中隔离观察点工作的一线医务人员，按发现确诊病例的当日计算工作天数。执行一档标准的一线医务人员，包括在卫生健康部门确定的定点医院、方舱医院的隔离区或其他收治确诊病例的医疗卫生机构的隔离区直接参与患者救治的医务人员，直接进行病例标本采集、病原检测和病理检查的医疗卫生专业技术人员，以及在湖北省内发热门诊工作的一线医务人员。其他一线医务人员执行二档标准。

三、关于及时发放卫生防疫津贴。卫生防疫津贴执行标准和发放办法，按照人力资源社会保障部、财政部《关于调整卫生防疫津贴标准的通知》（人社部发〔2020〕13号）规定执行。卫生防疫津贴执行范围，在疫情防控期间扩大到全体一线医务人员，在疫情结束后恢复至文件规定范围。

四、关于轮休、隔离不影响正常工资福利待遇。严格落实人力资源社会保障部、财政部《关于新型冠状病毒肺炎疫情防控期间事业单位人员有关工资待遇问题的通知》（人社部发〔2020〕9号）规定，一线医务人员在轮休、隔离期间，工资福利待遇按出勤对待，原正常发放的基本工资、岗位津贴（含卫生防疫津贴）、绩效工资继续执行。不得将一线医务人员轮休、隔离天数计入本人带薪年休假假期，不得向轮休的一线医务人员安排工作任务。

五、关于做好饮食营养等保障。对轮休、隔离的一线医务人员，各地卫生健康部门要牵头做好生活保障，为其配送必要的饮食、营养品和生活必需品。对过于劳累导致患病的一线医务人员，要开通就医绿色通道，保证其及时就医。加强对一线医务人员的人文关怀，尽量不安排双职工的医务人员同时到一线工作。做好援鄂医疗队及其他一线医务人员家属关心慰问工作，及时帮助解决生活、就医、照料等方面实际困难，切实解决他们的后顾之忧。

六、关于压实贯彻落实责任和强化监督检查。各地医疗卫生机构是贯彻落实保护关心爱护一线医务人员措施的责任主体，主要负责人是抓好落实的第一责任人，要健全规章制度，认真建立工作台账，据实按日记录、按月汇总上报应当领取临时性工作补助人员的名单、档次和天数，同时要纠正不必要的填表报数，减轻一线医务人员负担。各地卫生健康、人力资源社会保障、财政部门要按照职责分工，加强协同配合和工作指导，强化资金监管和督促落实。各地要坚持问题导向和结果导向，及时回应群众关切，协调解决政策措施贯彻落实中的苗头性、倾向性问题，坚决杜绝形式主义、官僚主义；发放临时性工作补助、卫生防疫津贴要严格进行登记、审核、报批，及时公示公开，主动接受监督，确保发放工作公平公正有序开展；要加大监督检查力度，不得扩大泛化一线医务人员范围，对有章不循、巧立名目、截留侵占、虚报冒领、违规发放、挤占挪用的，依纪依法严肃追究相关人员责任。

国务院应对新型冠状病毒感染

肺炎疫情联防联控机制

2020年3月11日

1. 全国粮食和物资储备系统全力做好疫情防控粮油供应和应急救灾物资保障工作

新冠肺炎疫情发生以来，国家粮食和物资储备局深入学习贯彻落实习近平总书记重要指示精神，认真落实党中央、国务院决策部署，按照国家发展改革委党组统筹安排，认真履行联防联控机制生活物资保障组成员单位职责，切实抓好粮油保供稳价、保障疫情防控物资急需，助力坚决打赢疫情防控人民战争、总体战、阻击战。

一、建立机制，压实责任，坚决把党中央、国务院决策部署贯彻落实到位

（一）成立领导小组，及时传达贯彻中央精神。1月27日成立应对新型冠状病毒感染肺炎疫情工作领导小组，张务锋同志担任组长，局负责同志担任副组长，各司局和直属联系单位主要负责同志为成员，下设应急保供组、局内防控组。截至目前，已先后组织召开8次局应对疫情工作领导小组会议和多次党组会、专题会，第一时间传达学习贯彻中央有关会议精神，落实生活保障组要求，研究部署疫情防控和粮油市场供应保障工作。应急保供组、局内防控组分别召开11次、9次工作例会，及时部署抓好落实。

（二）立即部署安排，做好粮油保供稳价。1月底即召开全国粮食和物资储备系统应对疫情做好粮油市场保供稳价工作视频会议，印发专门通知，要求各地压紧压实主体责任，集中力量保重点，强化调度支持，充分发挥粮食应急保障体系在保供稳市中的作用，确保粮油市场总体平稳。

（三）紧盯市场供应，采取有效措施稳定社会预期。建立粮食应急工作日报告制度，要求各省级粮食部门每天下午3点前报告是否启动粮食应急预案、粮油应急保供和市场情况。针对前期个别网点出现的群众集中购粮现象，会同地方政府和粮食部门及时采取有效措施，维护粮食流通秩序，加强供应保障。截至目前，各地粮油市场供应正常、价格平稳。

二、多措并举，强化调度，确保粮油市场供应充足、运行平稳

（一）建立健全监测报告制度，积极组织粮油加工企业复工复产。落实全国粮油市场日监测日报告制度。印发关于加快推进全国粮油企业复工复产工作的通知，稳妥有序推进企业复工复产。截至3月7日，37家保供协作机制重点加工企业全部开工，小麦日加工量1.39万吨，稻谷日加工量0.67万吨。全国粮食应急加工企业共计5388家，已开工4264家，占比为79.1%。其中，小麦日加工能力已达到44.2万吨，稻谷日加工能力已达到45.4万吨。

（二）建立五省联动机制，重点保障湖北省和武汉市粮油供应。制定湖北省和武汉市粮油供应工作方案，建立联动保障机制，加强安徽、江西、湖南、河南等毗邻省份和在地央企的协同联动，确保湖北和武汉粮油供应充足。通过建立成品临时储备、原粮储备紧急加工为成品粮、补贴扩大加工、支持企业增加商品库存、外省购入等方式，增强湖北省和武汉市成品粮油保障能力。

（三）及时组织投放政策性粮食，保障粮油、饲料、医用酒精等加工需求。为满足南方地区饲料用粮需求，组织公开竞价销售296万吨玉米，成交132万吨，其中湖北省23家企业购买7.7万吨；合理安排小麦和早籼稻竞价销售，2月10日起每周增加投放湖北省中晚稻80万吨，今年以来累计成交小麦、稻谷109万吨。

（四）因地制宜持续抓好秋粮收购。为满足农民售粮需要，会同有关部门研究确定将东北地区中晚稻最低收购价预案执行截止日期由2月29日延长至3月31日，切实发挥最低收购价政策托底作用。要求各地认真落实粮食安全省长责任制，严格落实分区分级精准防控要求，稳妥有序组织复工复产，抓好市场化收购，缓解本地售粮压力，保障南方销区省份用粮需求。

三、协同联动，利用储备资源，救灾物资调运及时高效、有序有力

（一）高效调运中央救灾物资。各中央救灾物资储备库特别是湖北及周边仓库，认真落实粮食和储备局部署，加强应急值守，克服疫情防控期人手不足、物流运输紧张等困难，合理安排人力、物力，确保物资调运及时高效。自1月28日以来，按照应急管理部调用指令，累计调运7批次、23.85万件中央救灾物资，用于支援湖北、浙江、河南、江西、贵州等五省做好转移人员安置、交通管制卡点人员值守、疑似人员隔离观察、医院新增床位补给等疫情防控工作。其中，调运19.8万件中央救灾物资，保障湖北、武汉疫情防控急需。

（二）积极组织做好物资中转。各垂管局充分利用储备资源，为当地疫情防控提供物资中转保障。湖北局及时为湖北省提供应急物流保障服务，截至目前，所属11个储备仓库累计接发中转应急保供物资263.56万箱/件和22.02万吨。其中，防疫救灾物资23.1万件，医护用品418吨、240.31万件，粮油食品1634吨、1440箱，医用原材料6061吨，农资化肥8.14万吨、成品油11.47万吨、雷神山用防渗透膜162吨、其他物资1.58万吨。

（三）协助解决石油企业困难，临时代储成品油。新冠肺炎疫情发生后，国内成品油消费量明显下滑，石油企业成品油库存快速上涨、压力剧增，影响正常生产。2月22日，印发《关于在新冠肺炎疫情期间支持相关企业开展成品油临时周转有关工作的通知》，利用国家储备油库资源，积极协调帮助石油企业解决库存压力

2020.3.12

1. 农业农村部：进一步加强远洋渔船新冠肺炎疫情防控 农办渔 [2020] 7号

有关省、自治区、直辖市农业农村厅（局），福建省海洋与渔业局，计划单列市渔业主管局，中国远洋渔业协会，中国农业发展集团有限公司，各远洋渔业企业：

当前，国内新冠肺炎疫情防控进入关键时期，境外疫情持续蔓延，特别是与我远洋渔业关系密切的一些国家和地区防控形势日益严峻。为进一步加强远洋渔船新冠肺炎疫情防控，防范输入性疫情，统筹抓好疫情防控和远洋渔业生产工作，现就有关事项通知如下。

一、高度重视远洋渔船疫情防控工作

我远洋渔船作业分布广泛，涉及国家多，船员来源广，船上人员密集，与外方接触频繁，疫情防控任务艰巨复杂。近期，境外疫情持续蔓延，对我远洋渔船的生产作业和船员健康造成较大威胁。各地渔业主管部门和远洋渔业企业要充分认识疫情防控的复杂性艰巨性，高度重视远洋渔船疫情防控工作，统筹抓好境内外疫情防控和远洋渔业生产，确保在外广大远洋船员的身体健康和生命安全。

二、严格落实远洋渔船疫情防控措施

各地渔业主管部门要按照疫情防控总体要求，督促指导远洋渔业企业和远洋渔船疫情防控方案制定到位、防疫物资配备到位、防控措施落实到位。远洋渔业企业要因地制宜制定疫情防控方案，落实疫情防控主体责任，做好所属渔船疫情防控工作。远洋渔船船长作为第一责任人，要切实落实船员个人防护、健康检查和记录、船上饮食安全、舱室通风和清洁消毒等防控措施，按规定做好疑似病例的排查、监测、隔离和报告。

三、切实防范远洋渔船发生输入性疫情

针对近期部分国家和地区疫情变化情况，远洋渔业企业要合理安排、尽量减少或暂停在疫情较重的国家或地区靠港进行外籍船员招募、船员轮换、加油补给等，切实做好相关防控措施，坚决防止远洋渔船发生输入性疫情。确有必要招募或轮换船员的，要按规定做好健康检查和隔离；确有必要进行海上加油或补给的，要充分做好防护应对措施。要尽量避免与外部人员直接接触，确保不发生感染疫情风险。

四、统筹做好疫情防控和远洋渔业生产

根据远洋渔业企业实际情况，我部已部署进一步加快远洋渔业行政审批进度，对因疫情影响造成的证件办理不及时等问题给予特殊安排，便利企业申请。各地渔业主管部门、中国远洋渔业协会及相关技术支撑单位要充分利用远洋渔业管理信息系统、远洋渔船船位监测系统、远洋渔业管理微信群等信息化手段，开展远洋渔业项目审批确认、国际组织注册、数据报送、船位监测、线上服务等工作，更好地为远洋渔业企业提供服务。各远洋渔业企业要密切与在外远洋渔船的沟通联络，随时掌握渔船作业和船员健康情况，统筹做好疫情防控和远洋渔业生产经营各项工作。各单位要做好信息收集和调度，有关情况和问题请及时报我部渔业渔政管理局。

联系人：渔业渔政管理局远洋渔业处 赵晓奇、朱江峰

电 话：010-59192952，59192969

农业农村部办公厅

2020年3月10日

1. 发改委：应对疫情进一步深化改革做好外资项目有关工作 发改外资〔2020〕343号

各省、自治区、直辖市及计划单列市、新疆生产建设兵团发展改革委：

为贯彻落实党中央、国务院关于统筹推进新冠肺炎疫情防控和经济社会发展的决策部署，深入实施《外商投资法》及配套法规，加大改革力度，做好当前和今后一段时期稳外资工作，促进外资基本盘稳定，现就有关事项通知如下。

一、积极帮扶外资项目和外资企业复工复产。各级发展改革委要统筹好疫情防控和复工复产，把外资项目和外资企业复工复产作为当前重要工作。对于已投产项目，要帮助协调返岗、物流、物资等问题，尽快恢复产能。对于在建项目，要做好工程建设保障、审批事项衔接，力争整体项目进度不受影响。对于前期项目，要创新工作方法，支持项目方远程办理各项手续，尽快进入实施阶段。要主动联系外资企业，协调解决复工复产障碍，各项援企政策统一适用于外资企业。对于重点外资企业要采取“一对一”精准帮扶，对于外资企业反映的产业链协同复工问题要积极响应，涉及跨省协调事项及时报告我委。

二、加强重大外资项目跟踪储备。各级发展改革委要根据职能，积极做好投资促进工作，加强与开发区、招商部门对接，完善重大外资项目跟踪储备机制。其中，对于制造业、高技术服务业总投资10亿美元以上在谈、签约、在建外资项目，如涉及国家部委协调事项的，省级发展改革委要及时向国家发展改革委申报，符合条件的将按程序纳入重大外资项目工作专班协调。技术先进、医疗防疫、关键领域或中西部地区、东北地区的项目，可适度放宽规模。

三、做好重大外资项目协调推进。纳入重大外资项目工作专班协调的项目，工作专班及有关部门将给予政策支持，会同项目所在地政府统筹推进。各级发展改革委要积极协调推进地方层面的重大外资项目，确保要素跟着项目走。利用外资重点省市发展改革委要推动建立健全地方层面的重大外资项目专班机制，制定项目清单和工作方案，加强各级联动、部门协同，建立绿色通道，推动项目尽快落地。通过重大外资项目工作以点带面，全方位提升外资项目服务水平。

四、全面落实外资准入负面清单管理制度。对于外资准入负面清单（以下简称负面清单）之外的外资项目，不得设置单独针对外资的准入限制，除《政府核准的投资项目目录》规定内外资项目均需核准的，其他外资项目由地方发展改革委实行属地化备案管理。对于负面清单之内涉及固定资产投资的外资新建项目或并购项目，根据股比、高管要求等规定，办理核准手续。其中，总投资3亿美元及以上项目由国家发展改革委核准，3亿美元以下项目由省级发展改革委核准。落户自贸试验区的外资项目，按照自贸试验区负面清单执行。

五、提高外资项目备案便利化程度。所有备案的外资项目一律实行告知性备案管理。备案机关通过全国投资项目在线审批监管平台（以下简称在线平台），在项目实施前收到项目单位提供的项目基本信息即完成备案。项目基本信息包括项目单位、项目名称、项目地点、项目内容、投资规模、投资方及国别、出资额及出资比例、符合负面清单及产业政策声明。备案证明可通过在线平台由项目单位自行打印。

六、简化外资项目核准手续。项目单位提交项目申请报告，除规定内容外，无需附企业财务报表、资金信用证明、环境影响评价审批文件、节能审查意见、国有资产出资确认文件。除法律、行政法规另有规定外，外资项目核准手续可与其他许可手续并行办理。全面推进远程办理，有条件的地方要加快完善在线平台收件、出件功能，实现无纸化办理，暂不具备条件的地方可通过邮寄方式收件、出件，实现不见面办理。疫情期间申报材料有关附件存在办理困难的项目，核准机关可实行容缺受理、先办后补。

七、优化鼓励类外资项目进口设备免税确认流程。对于属于《鼓励外商投资产业目录》范围的鼓励类外资项目，在投资总额内进口自用设备继续实行免征关税政策。限额以上的鼓励类外资项目，取消省级以下转报环节，项目单位直接向省级发展改革委提出免税申请，由省级发展改革委初审后报我委。疫情期间，对于因供应链问题暂不能确定进口设备清单的项目可容缺受理，相关材料可通过纵向网报送我委，先行启动办理程序。

八、保护外资项目合法权益。外资项目备案、核准机关应当遵循便民、高效原则，公开外资项目备案和核准的办理流程、办理条件、办理时限，不得擅自增加审核要求，不得超出办理时限，不得非法干预外商投资自主权。外资项目备案、核准机关及其工作人员不得利用行政手段强制或变相强制转让技术。对于履职过程中知悉的商业秘密，应当依法予以保密。各项支持企业和项目的政策，应当平等适用于内外资企业、内外资项目。

九、加强对外资企业的走访服务。各级发展改革委要加强与外资企业和外资企业商会沟通交流，宣传解读政策法规，听取外资企业和商会对改善本地区投资环境的建议，主动回应企业关切。要经常性走访外资企业和外资项目，深入了解生产经营和招商引资情况，加强形势分析，协调解决问题，推动完善本地区投资促进和便利化等稳外资政策措施。制定与外资有关的政策，应当以座谈会、书面等形式征求外资企业意见，充分考虑跨国投资实际情况，切实体现内外资一视同仁、平等对待。

十、完善外资项目事中事后监管。外资项目备案、核准机关要加强与同级政府有关部门的协同配合，按照谁审批谁监管、谁主管谁监管的原则，采取“双随机一公开”等方式完善项目监管，监督检查应当尽可能纳入跨部门联合抽查范围，以避免重复检查增加企业负担。省级发展改革委要通过在线方式抽查备案外资项目，加强对负面清单执行情况的监督检查，发现违反负面清单规定的情形及时上报，并依法予以处理。

十一、进一步扩大鼓励外商投资范围。近期我委将会同商务部等部门修订《鼓励外商投资产业目录》。本次修订拟进一步扩大鼓励范围，促增量和稳存量并举，聚焦促进制造业高质量发展、发挥中西部地区和东北地区承接产业转移潜力。请省级发展改革委结合本地区实际，根据招商引资情况、外资项目和外资企业诉求、产业发展方向等，会同商务等有关部门抓紧研究提出需增加的《鼓励外商投资产业目录》条目建议，于3月17日前报我委。

各级发展改革委要充分认识当前和今后一段时期稳外资的重要性，在抓好疫情防控的同时，勇于担当、善于作为，通过改革完善外资项目有关工作，努力做好稳外资工作。落实本通知的经验、问题和建议，请及时向我委报告。

国家发展改革委

2020年3月9日

1. 科技部：做好创业孵化机构科学防疫推进创业企业有序复工复产保持创新创业活力 国科火字〔2020〕66号

各省、自治区、直辖市及计划单列市科技厅（委、局），新疆生产建设兵团科技局，各科技企业孵化器、大学科技园、众创空间：

为深入贯彻落实习近平总书记在统筹推进新冠肺炎疫情防控和经济社会发展工作部署会议上的重要讲话精神，在切实做好疫情防控的同时，积极推动创业孵化机构和创业企业有序复工复产，继续保持创新创业活力，力争实现逆势发展、更高质量发展，现就有关事项通知如下。

一、坚决贯彻党中央疫情防控各项要求

当前正处于疫情防控关键期，科技企业孵化器、大学科技园、众创空间等创业孵化机构要坚决贯彻习近平总书记关于疫情防控系列重要讲话精神，深刻认识疫情的严峻形势，坚决落实防控各项要求，把疫情防控作为当前头等大事。建立科学有效的排查机制，与所在地的疫情联防联控机构保持密切联系，严格执行国务院联防联控机制印发的《企事业单位复工复产疫情防控措施指南》，切实加强孵化场地防控，督导创业企业员工做好个人防护，建立疫情防控应急措施和处置流程，避免发生集聚性疫情，坚决打赢疫情防控阻击战。

二、高度重视并稳步推进创业孵化机构和创业企业有序复工复产

创业孵化机构是我国实施创新驱动发展战略的“基础设施”，创业企业是我国经济创新发展的重要“火种”。各级主管部门要根据疫情防控实际，把帮助创业孵化机构和企业有序复工复产摆在重要位置，为其逐步恢复正常生产经营秩序创造有利条件，努力实现疫情防控和创新创业发展两手抓、两不误，切实为疫情消退后持续推进我国经济新旧动能转换、高质量发展蓄积有生力量。各地科技主管部门要加强与创业孵化机构的联系，对所在地区的创业孵化机构复工复产情况进行摸底，了解困难和政策需求，及时将情况上报科技部火炬中心。请各科技企业孵化器、大学科技园、众创空间登录科技部火炬中心信息服务平台（http://www.chinatorch.gov.cn/fhq），点击“创业孵化机构复工复产情况监测”菜单栏，分别于3月17日、24日、31日完成填报工作。

三、确保各项引导支持政策精准及时落地

进一步落实科技企业孵化器、大学科技园、众创空间在房产税、土地使用税和增值税等方面的引导支持政策，及时做好新升级国家级科技企业孵化器和国家备案众创空间的免税申报入库工作。各地科技主管部门应加大对创业孵化机构的支持，对为在孵企业减免办公场地、科研和生产用房租金以及服务费的创业孵化机构，给予一定奖励或补助。各创业孵化机构要及时协助创业企业享受税收减免、财政补贴、企业贷款、职工社保、稳定就业等政策，帮助其更好开展高新技术企业申报、科技型中小企业评价、知识产权申报，吸纳高校毕业生创业就业等政策服务。

四、以疫情防控为契机推动创业孵化服务升级和新业态培育

鼓励支持创业孵化机构推出疫情期间针对企业融资、供应链物流、防疫物资配套、员工返岗等方面的专项服务，携手创业企业共克时艰；积极开展线上服务，利用线上平台开展创业辅导、项目路演、导师分享、资源对接等各类创新创业活动，持续保持创新创业活力；探索利用互联网、大数据、区块链、人工智能等新技术新方式，提升孵化服务智能化水平。鼓励创业孵化机构把握疫情防控需求，发挥科技创业企业研发快、转型快、成长快的特点和优势，主动在新型检测试剂、智能医疗和检测设备、互联网教育、远程办公等领域，发掘优秀创业项目，为创业团队和企业提供研发合作、市场开拓、供应链对接等服务，催生相关新产品新企业，培育新业态新产业。

五、因时因地创新管理服务方式

各级科技主管部门要进一步提升科技治理水平，优化管理方式，简化办事流程，对疫情防控期间表现突出的科技企业孵化器、大学科技园、众创空间，在项目申报、年度考核评价中予以加分奖励。科技部火炬中心将继续完善网上信息服务，推动科技企业孵化器、大学科技园、众创空间的统计监测、税收减免、认定备案工作实现网上办理，2019年度科技企业孵化器统计数据填报工作截止日期延长至2020年3月15日。

各地科技主管部门要以疫情防控为契机，研究出台新形势下推进创业孵化服务升级发展的引导措施，着力提升孵化服务的深度和集聚水平，完善创业孵化生态。各地科技主管部门、创业孵化机构要及时总结科学防疫与保持双创活力统筹兼顾的先进经验和做法，做好相关宣传报道，并报送科技部火炬中心。

科技部火炬中心

2020年3月4日

1. 民政部：印发《民政精神卫生福利机构疫情防控工作指南（第二版）》

各省、自治区、直辖市民政厅（局），各计划单列市民政局，新疆生产建设兵团民政局：

为贯彻习近平总书记关于新冠肺炎疫情防控工作系列重要讲话和指示精神，落实《国务院应对新型冠状病毒感染肺炎疫情联防联控机制关于进一步做好民政服务机构疫情防控工作的通知》（国发明电〔2020〕6号）要求，结合各地疫情防控工作实践，我部对《民政精神卫生福利机构疫情防控工作指南（试行）》进行了修订，形成了《民政精神卫生福利机构疫情防控工作指南（第二版）》，现印发给你们，请结合实际，认真贯彻执行。执行中遇到的重大情况以及工作建议，请及时报送部社会事务司。

附件：民政精神卫生福利机构疫情防控工作指南（第二版）

民政部办公厅

2020年3月6日

附件

民政精神卫生福利机构疫情防控工作指南

（第二版）

为指导做好新冠肺炎疫情防控工作，切实保障民政精神卫生福利机构服务对象和工作人员身体健康和生命安全，依据《中华人民共和国传染病防治法》和国务院应对新型冠状病毒感染肺炎疫情联防联控机制《关于依法科学精准做好新冠肺炎疫情防控工作的通知》、《关于进一步做好民政服务机构疫情防控工作的通知》等文件精神，现制定《民政精神卫生福利机构疫情防控工作指南（第二版）》。

一、建立防控工作机制。建立健全工作方案和工作流程，成立以党政领导为组长的疫情防控工作领导小组，全面负责疫情防控工作的领导、指挥、协调和突发事件的处置工作。制定实施疫情防控工作预案，明确组织机构、工作原则、工作制度、应急规程和工作要求等，做到人员到位、分工明确、责任清晰。定期召开会议，传达贯彻上级要求，学习贯彻政策文件，研究分析防控形势，部署疫情防控工作。同时担负医疗救治、流浪精神病人收治职责的精神卫生福利机构，应当按照其他规范要求统筹建立疫情防控工作机制。

二、分级实施封闭管理。推迟、减少会议和大型活动，取消所有外出及机构内人员集中用餐和其他各类人员聚集等集中性活动。湖北省以及疫情严重地区的机构实施封闭式管理，暂停接收新的社会精神障碍患者入院，暂停探访、咨询、接待活动，做好暂不接收患者家属的安抚和政策解释工作。疫情低中度风险地区可按照当地疫情防控统一部署，在严格落实各项防护措施的前提下，逐步恢复接收新的社会精神障碍患者入院，有序开展相关活动和服务。

三、强化人员出入管理。所有人员进入机构前需测量体温并做好登记，如出现异常迅速离岗，按要求进行报告并隔离观察或就医。严格控制外来人员，与疫情防控无关的外来人员及车辆未经批准一律不得入内。对经批准出入机构的车辆做好登记工作，乘车人员须测量体温并佩戴口罩，消毒后方可进入机构。

四、专门设立隔离区域。科学设立隔离区，规范设置临时留观室。新入院和外出治疗返回服务对象、新入职和返岗工作人员，须经过14天的严格医学隔离观察，确定身体状况无异常后方可进入居住区或安排上岗，提前把控风险，杜绝交叉感染。隔离区内的医护人员必须按要求穿戴好手套、口罩、护目镜、帽子、防护服等防护装备。

五、严格执行报告制度。认真做好疫情监测报告工作，落实疫情监测报告责任，安排专人负责，加强应急值守，确保监测报告及时、准确、到位，做好疫情应急响应和处置。配合当地卫生健康部门和疾控机构做好疫情报告工作，及时上报给所属民政部门、卫生健康部门。

六、加强日常疫情监测。建立晨晚检制度，对服务对象和工作人员每日进行体温检测并进行健康登记，对发热、咳嗽的服务对象，重点观察且进行必要隔离。湖北省以及疫情严重地区的机构须对服务对象和工作人员进行全员核酸检测排查，确保不漏一人。如发现确诊或疑似病例，立即向当地卫生健康部门和疾控机构报告，并按要求及时转运到定点收治医院、隔离点或轻症治疗点接受治疗或观察。确诊或疑似病例且密切接触服务对象较多的机构，须按程序提请当地根据实际需要，按照集中隔离点的防控标准，配备必要的医务人员和设备，纳入辖区集中隔离点范围进行规范管理。

七、严格执行防护流程。落实消毒流程，所有工作人员进入服务对象居住区前须采取消毒、更换工作服等措施。组织工作人员和服务对象勤洗手，做好手部卫生，佩戴口罩。所有工作区、居住区每日定时通风、定时消毒，提高防护能力。换下的口罩、手套等用品，集中消毒后统一处理。

八、做好院内感染防控。加强对重点部门、重点部位的消毒防护工作，包括各种医疗物品的消毒灭菌、医疗废弃物的处理工作，确诊或疑似患者遗体的处置工作，办公场所、公共场所、住院区、就餐区等区域的卫生管理和消毒工作，必要场所配备洗手设备和消毒剂，防止发生医源性感染。加强对食堂的食品安全和卫生安全管理，严格控制食品来源，强化规范操作，加大食堂设施设备的消毒力度。

九、加强预防培训宣传。做好工作人员培训及个人防护工作，机构的一线工作人员以及其他密切接触服务对象的工作人员，严格执行戴口罩上岗和其他必要的防护制度。通过手机信息、宣传栏、板报、广播等多种形式，加强传染病防治法律法规及预防知识的宣传普及，增强防范意识、提高防护技能、严防疫情扩散。

十、做好物资保障工作。购置储备必要的防护服、口罩、护目镜、一次性乳胶手套、一次性鞋套、测温仪、酒精、消毒液等防护物资，湖北省以及疫情严重地区的机构，须按程序提请当地按照三级防护标准配备、下发防控物资，确保机构每日防护需要；其他地区要在保障日常防护需要的同时，按照不少于三天需求量做好储备，确保应急使用。储备服务对象及工作人员生活物资，按程序提请当地按要求采取统一配送方式，保障机构所需米面油、肉蛋菜等生活物资供应，将机构纳入社会捐赠物资调配范围，确保生活正常有序，保障急救车辆的正常运行。

十一、开展关心关爱活动。加强机构内服务对象心理疏导，开展协助拨打亲情电话等精神慰藉活动。关心爱护工作人员，做好防护保障，合理安排轮休，提供心理支持，保障他们的身心健康。

十二、强化应急保障工作。密切关注本地区疫情情况，按照工作要求加强应急值守，保持信息畅通，提前与当地政府、有关部门及社区等沟通协调，预备应急车辆，做好疫情应急响应和处置工作。

1. 交通运输部 发改委：阶段性降低港口收费标准等事项

各省、自治区、直辖市交通运输厅（局、委）、发展改革委、物价局，交通运输部长江航务管理局、珠江航务管理局，交通运输部各直属海事局：

为深入贯彻党中央、国务院决策部署，统筹做好新冠肺炎疫情防控和经济社会发展工作，决定阶段性降低港口政府定价经营服务性收费标准，促进口岸营商环境优化，推动物流业、产业链协同复工复产。现将有关事项通知如下：

一、降低港口政府定价经营服务性收费

2020年3月1日至6月30日，将实行政府定价的货物港务费、港口设施保安费两项港口经营服务性收费标准分别降低20%；取消非油轮货船强制应急响应服务及收费。鼓励各地结合本地实际，疫情防控期间加大收费优惠力度。港口经营人要严格执行政府定价管理规定，根据本通知及时调整对外公示的收费项目名称和收费标准。

二、加强上下游合作

按照法治化原则，加强港航企业与货主之间的对接和协作，建立降费传导机制，形成利益共同体，积极应对疫情影响。鼓励港口经营人对受疫情影响提货困难的企业，特别是小微企业，继续给予减免库场使用费等优惠。引导班轮公司合理调整海运收费价格结构，鼓励采用包干方式收取费用。

三、维护良好市场秩序

各级交通运输（港口、海事）管理部门要加强国际海运、港口市场监管，依法对违规行为进行调查，并配合有关部门进行查处，进一步规范海运、港口收费行为，巩固落实降费措施成效。地方各级交通运输（港口）管理部门和价格主管部门要加强政策宣传，将本通知及时、准确传达到相关经营人和单位，督促港航企业和相关单位认真落实口岸经营服务性收费目录清单和公示制度。

交通运输部 发展改革委

2020年3月6日

1. 民航局：积极应对新冠肺炎疫情有关支持政策 民航函〔2020〕145号

民航各地区管理局，各运输（通用）航空公司，各航空油料公司，中国民航信息网络股份有限公司，各机场公司，局属各单位：

为贯彻落实党中央、国务院的决策部署，按照“保安全运行、保应急运输、保风险可控、保精细施策”的工作要求，积极应对新冠肺炎疫情对民航业的影响，切实为民航企业发展纾困，精准有序推动复工复产，现将有关促进民航业稳定发展的支持政策通知如下：

一、 实施积极财经政策

（一）落实免征航空公司应缴纳的民航发展基金等优惠政策，确保惠企政策在行业内落地。

（二）落实疫情防控期间，中央财政对国际定期客运航班以及按照国务院联防联控机制部署执行重大运输飞行任务给予资金支持的政策。

（三）充分利用现行补贴政策，对执行疫情防控任务的通用航空企业给予支持。

二、积极推进降费减负

（四）机场管理机构免收按照国务院联防联控机制部署执行重大运输飞行任务的航空性业务收费和地面服务收费，空管单位免收进近指挥费和航路费。

（五）降低境内、港澳台地区及外国航空公司机场、空管收费标准。一类、二类机场起降费收费标准基准价降低10%，免收停场费；航路费（飞越飞行除外）收费标准降低10%。境内航空公司境内航班航空煤油进销差价基准价降低8%。上述降费政策自2020年1月23日起实施，截止日期视疫情情况另行通知。

（六）鼓励信息、局属企事业等单位适当降低现行收费标准。

三、加大基础设施投资

（七）各机场管理机构、相关运行保障单位和民航医学研究单位紧急购置或开发应对疫情的专用固定资产设施设备均可申请民航发展基金补助，具体由民航局或各地区管理局核定后审批资金补助申请。

（八）支持重大建设项目科学有序开工复工。网上受理项目申请，加快推进项目前期工作，协调解决开工复工重点难点问题，支持重大、紧急建设项目利用已开放的交易中心加快项目招投标工作。抓紧新开工复工枢纽机场、脱贫攻坚、蓝天保卫战等一批重点基础设施项目，力争全年完成固定资产投资1000亿元。

（九）落实现行民航发展基金政策，更好支持航空公司提升安全能力。加大航空公司安全、安保、飞行品质监控、卫星导航及其他新技术应用项目投资。支持航空公司机载设备安全升级改装，全额安排民航发展基金用于B737NG飞机数据帧扩展项目建设。

（十）支持空管、信息、供油等关系国家、行业和公众利益战略性基础设施规划建设，加大政府资金支持力度。

四、促进航空运输发展

（十一）实施灵活的运力引进政策，支持引导航空公司优化运力，简化运力引进程序。

（十二）优化航线航班许可管理，支持航空公司根据市场需求灵活调整航班计划、国际航线结构和航权额度，简化航线航班审批程序、时刻协调程序等，缩短国际航线开航（复航）审批时限。

（十三）顺延执行2019年冬航季航班计划至5月2日，暂停考核航空公司航班计划执行率、时刻执行率和航班正常率。积极出台适应航空公司恢复生产需求的换季航班时刻政策，对2020年夏航季国内航班时刻执行率豁免考核，2020年夏航季国内、国际和地区时刻池、库内的航班时刻，可按照市场需求变化进行有序流动。

（十四）积极支持并协助航空公司就疫情期间的特殊运营需求和恢复国际航线过程中遇到的航权、时刻等问题，与相关国家民航主管部门进行积极沟通协调。

五、优化政务服务工作

（十五）调整行政审批工作方式，采用网上办理、非现场办理等形式，优化审批流程，推行告知承诺制，提高审批效率。

（十六）转变监管方式，提升监管效能，减少现场监管方式，充分运用非现场监管等新型监管方式。

民航各地区管理局要加强监督检查，确保上述政策措施落实落地。各航空公司要强化主体责任，加强内部管理，开源节流、挖潜增效，确保行业安全稳定运行。各保障单位要提高站位、服务大局，为行业安全稳定运行提供保障。

中国民用航空局

2020年3月9日

1. 林草局：积极应对新冠肺炎疫情有序推进2020年国土绿化工作 林生发〔2020〕25号

各省、自治区、直辖市林业和草原主管部门，内蒙古、大兴安岭森工（林业）集团公司，新疆生产建设兵团林业和草原主管部门，国家林业和草原局各司局、各派出机构、各直属单位：

为深入贯彻习近平总书记重要指示精神，认真落实中央关于统筹推进新冠肺炎疫情防控和经济社会发展工作的决策部署，一手抓疫情防控，一手抓2020年国土绿化谋划推进，现就有关事项通知如下：

一、分区施策推进春季造林

随着天气转暖，春季造林已由南向北渐次展开。各地要在确保疫情防控和满足复工复产相关要求的前提下，以县级为单位，因地制宜，有序开展春季造林绿化。疫情低风险地区要在安全可控前提下，抢抓当前造林黄金时节，全力推进造林绿化进度；中风险地区要坚持疫情防控优先，安全有序开展春季造林绿化，同时要提前谋划，待疫情结束后，加快推进造林绿化；高风险地区要把疫情防控作为最重要最紧迫的任务，及时调整工作节奏，尽可能地做好造林绿化各项前期谋划和准备工作，为疫情结束后及时开展造林绿化争取主动。各地要优先推进国家重点生态工程建设，确保年度计划任务保质保量完成。

二、全力做好雨季秋季造林准备

因疫情影响错过春季造林季节的南方地区，疫情结束后要及时组织开展整地、备苗等准备工作，加大秋季、冬季造林力度，尽可能降低疫情对造林绿化带来的影响。北方地区在大力推进春季造林的同时，要密切关注天气趋势预测，提前谋划，提前准备，加大雨季、秋季造林力度。各地要开展苗木调查摸底，加大容器苗生产培育力度，有条件的地方，可将苗龄小的裸根苗转为容器苗培育，以满足其他季节造林绿化用苗需求。要充分发挥机械设备在造林绿化生产中的重要作用，有条件的地方，要动员和组织有关专业化企业和经营主体，采用挖坑机等机具开展整地作业服务，提高劳动效率，加快造林进度。

三、积极做好内业准备工作

各地要抓紧部署指导造林绿化项目作业设计编制、审查、批复等工作。尚未完成作业设计编制的地方要加快工作进度。已经编制作业设计的地方，要按照疫情防控要求，创新作业设计审批形式，采用函评、视频会议、网上审批等非人员聚集方式进行评审批复，尽可能缩短项目审批时间，确保疫情防控期间项目推进不停摆。对于需要招投标的造林绿化项目，要积极协调相关部门，尽量简化流程、简化环节、压缩时限，采取简易招标方式，加快造林绿化项目实施进度。

四、有序组织造林绿化用工

各地要考虑受疫情管控影响，专业造林绿化企业复工慢、用工难、用工贵等问题，组织动员当地农民，特别是深度贫困地区建档立卡贫困人员，以及因疫情影响难以返城务工人员，采取线上培训、视频培训、编制简易技术手册等形式进行技术培训，就地就近承担造林绿化任务，缓解用工短缺难题，加快春季造林进度，同时为群众创造就业机会，增加劳务收入，助力脱贫攻坚。组织农民群众造林绿化，要注意合理有序安排，避免人员集聚，做好人员防护措施。

五、统筹安排造林绿化任务

各地要根据疫情，及时做好2020年度造林绿化任务时空安排、结构调整。尚未分解下达2020年造林计划任务的省（区、市），要充分考虑疫情影响，统筹安排好春季、雨季、秋冬季造林计划任务，与基层做好对接，及时分解落实到各市县区。已经分解下达造林计划任务的省（区、市），可根据疫情程度等实际情况，重新研究调整本地区造林计划任务安排，将受疫情影响难以完成的春季造林任务调整到雨季、秋冬季造林。充分考虑疫情造成用工短缺、人工造林受限等因素，及时调整造林结构，适当加大封山育林、飞播造林力度。各地林业和草原主管部门要与相关部门加强沟通协调，积极落实造林绿化用地，为全面完成造林绿化任务提供用地保障。

六、切实保障造林物资运输畅通

受疫情管控影响，一些地方道路还不通畅，对造林物资运输影响较大。各级林业和草原部门要统筹做好春季造林物资运输需求研判，加强与交通运输部门沟通协调，重点解决跨地区调运难、进村难等问题，切实保障苗木、种子、化肥、农药等造林物资运输畅通。

七、适时抓好松材线虫除治工作

各地要及时掌握春季疫木除治进度，分区施策、分类管理，加强指导，加快进度，力争按规定时限完成疫木除治任务，并严格按照有关要求进行疫木无害化处理。对确实无法在媒介昆虫羽化前完成疫木除治任务的，要及时调整除治方案，采取有针对性的补救措施，重点是全面加强疫区检疫封锁，防止疫木流失导致疫情传播；要加大媒介昆虫的防治力度，科学应用飞机防治和地面防治等多种方式，降低虫口密度。

八、积极推进草原保护修复

各地要认真组织实施退牧还草工程、退化草原人工种草生态修复试点项目，提前做好任务分解、实施方案编制等准备工作，加快工程实施进度。加快编制草原生态修复工程规划，加强草原禁牧、草畜平衡的监管，持续推进草原生态保护和修复。及时分析预判草原蝗灾等生物灾害发生趋势，提前做好灾害防控应急预案、物资储备等工作。

九、有效建立并完善国土绿化调度会商机制

各地要加强国土绿化进展情况调度，及时跟踪掌握面上造林绿化及天然林资源保护、退耕还林、京津风沙源治理、石漠化综合治理、三北等重点地区防护林体系建设、国家储备林、退牧还草、退化草原人工种草等重点生态工程进展，分析研判疫情影响，及时发现问题，研究对策，督促指导。实行调度工作月报制度，各省级林业和草原主管部门应于每月3日前将上月各项工程进展情况报送我局各相关司局、各重点工程管理办公室；我局各相关司局、各重点工程管理办公室应于每月5日前将各自管理的重点工程进展情况提交生态司汇总。

中国林科院组织有关专家针对疫情影响提出了若干主要树种造林技术措施建议（见附件），现一并下发，供各地参考。

特此通知。

附件：中国林科院组织有关专家针对疫情影响提出的若干主要树种造林技术措施建议

国家林业和草原局

2020年2月28日

附件

中国林科院组织有关专家针对疫情影响提出的若干

主要树种造林技术措施建议

一、疫情对速生用材林培育经营影响及应对技术措施建议

1.杉木

疫情影响：杉木通常宜在冬末春初新芽未萌动前造林，受疫情影响栽植时间被迫延后，影响杉木的培育和经营工作。

应对技术措施：（1）实行大规格穴状整地，提倡容器苗造林。大规格穴状整地可以缩短工期，减少土壤干扰，避免雨季造成的养分流失，保障造林成活率；容器苗造林可以缩短缓苗时间，同时保证了苗木成活率。（2）尽早尽快栽植，控制造林密度。建议在3月底完成栽植，并在栽植穴施用基肥，保证苗木早期生长；栽植密度167~296株，可提高单位面积保存率，同时降低抚育成本。（3）注意除萌补植，做好幼林抚育管理。造林后及时切除萌条，培厚土压萌，同时注意成活情况，及时补植；做好除杂草、追肥等幼苗抚育工作。（4）提倡机械化作业，减低人员密集度，提高作业安全度与效率，同时省时、省力。

2.杨树

疫情影响：春季是杨树苗木繁育和生产的最佳时间，受到疫情影响，交通运输、劳动力等受到限制，导致杨树错过最佳扦插和抚育时间。

应对技术措施：（1）尽快尽早制作插穗，提倡错时抚育。插穗要用黑色、可通气的覆膜保水贮藏，减小修枝强度，也可延后抚育时间。（2）提倡机械化作用，保证造林时效。利用机械快速起苗，假植于阴凉处，疫情缓解后可采用扦插、截干等方式保证造林时效。

3.落叶松

疫情影响：落叶松采穗嫁接和造林时间一般在春季，受到疫情影响，良种种质资源采穗、苗木调拨等工作受到限制。

应对技术措施：（1）尽早采穗，做好育苗准备工作。建议在落叶松树液流动前完成采穗工作，在生长季开始前完成育苗准备工作。（2）提倡容器苗造林。落叶松采用容器苗造林可推迟到雨季进行，如果不能容器苗造林，建议大田裸根育苗。

4.马尾松

疫情影响：马尾松适宜的造林时间一般为1~3月，由于疫情的发生，马尾松的造林时间要推迟至3~4月。

应对技术措施：（1）选用容器苗造林，推迟苗木萌动发芽时间。利用容器苗造林时，使用遮阳物覆盖延迟马尾松容器苗发芽和抽梢时间，并延后施肥时间。（2）做好马尾松授粉、病虫害防治工作。建议加强马尾松雌雄球花观测，及时开展马尾松花粉采集、雌球花套袋和杂交授粉工作；可采用无人机进行病虫害的防治，也可利用无人机来实现马尾松种子园的人工辅助授粉。

5.桉树

疫情影响：由于疫情的发生，桉树的造林时间可能要推迟到5~6月份开始进行。同时3月份是桉树病虫害高发期，疫情影响防治工作。

应对技术措施：（1）减缓苗木的出圃时间。当气温不断升高，不揭开荫棚或用稻草等覆盖以延迟桉树组培苗生长，同时注意苗木贮藏。（2）提前防治病虫害。可利用无人机进行农药喷洒，推迟造林的建议进行多次抚育。

二、疫情对经济林培育和经营的影响及应对技术措施建议

1.油茶

疫情影响：受到疫情的影响，油茶播种催芽时间需推迟到3月中下旬以后才能进行，造林计划被迫推迟。

应对技术措施：（1）加快油茶种子播种催芽。油茶种子催芽要50天以上，建议采用大棚内加小拱棚的2层塑料薄膜加温催芽，如果遇到倒春寒，可在大棚内设置加温设备，保证油茶芽苗在5月上旬可生长到符合嫁接的要求。（2）采用油茶容器苗造林。尽早使用容器苗造林，不用裸根苗造林，加快造林速度，保证成活率。（3）加强油茶林施肥培土。在春稍萌发前施肥保证油茶春稍的生长，越早越好，切忌春稍萌发后大量施肥。（4）及时开展整形修剪促进高产。尽早安排修剪，按照先剪病虫害枝，再剪枯死枝、下垂枝、重叠枝的先后顺序。

2.薄壳山核桃

疫情影响：受到疫情的影响，薄壳山核桃造林时间推迟。

应对技术措施：（1）采用容器苗种植。在2月中下旬及3月初可以选择裸根苗和容器苗种植，但在3月中下旬宜选择容器苗；造林时间宜早不宜迟，避免气温回升，影响苗木栽植成活率。（2）尽快开展造林。薄壳山核桃造林宜早不宜迟，在树液流动前、阴天和雨后造林，保证成活率。

3.杜仲

疫情影响：受疫情影响，苗木运输、劳动力和生产受到严重限制，杜仲造林受到影响。

应对技术措施：（1）做好播种前种子沙藏等工作，造林宜选用容器苗和裸根苗。（2）种子、苗木运输采用就近原则，力争就近调运种子、苗木，减少中间运输环节。（3）做好假植工作。如果不能开展造林任务导致苗木开始发芽，后期种植时注意对苗木保护，适当剪去上面萌发的部分枝条，种植后多浇水。

4.油桐

疫情影响：油桐大田裸根苗的适宜造林时间为每年的12月至翌年的3月，疫情发生后，部分地区油桐的造林时间要推迟至3~4月份进行。

应对技术措施：（1）延迟苗木萌动发芽时间。油桐苗3月底尚未萌芽并抽梢，可用黄泥浆浸透根部，用薄膜包裹保湿，移栽择降雨后或者浇水造林，避免长距离运输，3月底前完成造林，可适当提高造林密度。（2）提倡开展杂交授粉工作。对于花期错开的品系，尽量采用低温快递花粉的方式开展杂交工作。（3）推迟施肥时间。对于当年生油桐幼林，栽培时用有机肥作为基肥，可推迟至3月底施肥；对于2~3年生幼林，第一次幼抚和施肥也可推迟1个月进行。

5.无患子

疫情影响：无患子实生育苗一般在1~2月份进行，集中防治病虫害时间在1~4月份。受到疫情影响，劳动力等受到限制，播种和造林时间将推迟至3月中旬以后，病虫害防治工作难度加大。

应对技术措施：（1）提前预处理种子，保证幼苗生长。提前对种子进行水选，去除不饱满及有虫害的种子。（2）尽早将接穗采下保存。建议尽早将接穗采下，分别品系捆扎并做好标记，放入贮藏坑或置于室内阴凉处，用湿沙埋好，上盖湿稻草或塑料薄膜保湿。（3）及时清除受害苗木。建议进行林地清理，清除受病害影响的苗木，同时挖好栽植穴并回填表土。

三、疫情对珍贵用材林培育经营的影响及应对技术措施建议

1.北方珍贵树种

疫情影响：2~3月份是我国北方珍贵树种良种储备和繁育的重要季节，因疫情劳动力、交通运输等受到限制，影响了北方珍贵树种的造林工作。

应对技术措施：（1）适当推迟繁育时间，做好良种繁育。对于已完成良种接穗收集的，可以沙藏接穗，也可置入冷库冷藏，延长储存时间，推迟嫁接时间至3月底到4月中旬；未完成良种接穗收集的，做好6月的夏芽嫁接计划。（2）推迟造林，并加强抚育。建议将栽培造林推迟到3月底至4月上旬进行，对于两根一干或三根两干的苗木，可在造林后平茬管理，提高造林成活率，4月份开展大苗移栽。

2.南方珍贵树种

疫情影响：我国南方地区珍贵树种，如降香黄檀、柚木、红锥等多在3~4月份开始大面积造林，受到疫情的影响，造林时间预计推迟1个月以上，错过最佳造林时间。

应对技术措施：（1）采用容器苗造林，优化繁育措施。开展容器苗造林，在苗圃集中炼苗，加强水肥管理，尽早进行修枝剪叶，尽快进行种苗移栽。（2）利用无人机进行病虫害防治。在病虫害高发期，利用无人机高空喷洒农药，减少人员聚集。

“疫情就是命令，防控就是责任”，中国林科院将深入贯彻习近平总书记关于疫情防控的重要指示精神，把疫情防控工作作为当前最重要的工作来抓，绝不松懈。在筑牢防控疫情的基础上，充分发挥中国林科院在林草科技行业的带头作用，坚定信心、凝心聚力、协同发力，积极组织技术专家为快速有效恢复生产提供有效服务，为打赢这场疫情防控阻击战提供科技支撑。

1. 交通运输部：做好疫情防控期间公路水运工程复工开工质量安全工作 交安监明电〔2020〕91号

各省、自治区、直辖市、新疆生产建设兵团交通运输厅（局、委），长江航务管理局：

为贯彻落实党中央、国务院关于复工复产工作部署，采取切实有效措施，确保新冠肺炎疫情防控期间公路水运工程质量安全形势稳定，现就有关事项通知如下：

一、全面强化复工开工质量安全意识

（一）严格落实质量安全责任。要深入学习领会、坚决贯彻落实习近平总书记在统筹推进新冠肺炎疫情防控和经济社会发展工作部署会议上的重要讲话精神，清醒认识做好复工开工项目质量安全工作对经济社会发展的重要性，提高政治站位，认真研究部署，落实质量安全责任，统筹做好复工开工项目疫情防控和质量安全工作。

（二）坚守质量安全底线。当前正值公路水运工程复工开工和疫情防控的关键时期，质量安全工作面临更加复杂严峻的形势。各级交通运输主管部门要深入贯彻落实党中央、国务院关于疫情防控和复工复产决策部署，在做好疫情防控的前提下，精准、稳妥推进公路水运工程复工开工，坚决守住质量安全底线，切实减少一般事故，严防重特大质量安全事故发生。

二、切实加强复工开工质量安全保障

（三）做好政策措施保障。地方各级交通运输主管部门要排查摸清辖区内复工开工项目情况，综合研究疫情带来的质量安全风险，分析风险因素、预判风险程度、制定防控措施。指导有关单位落实工期保障、费用补偿等与质量安全相关举措，按照疫情风险等级，分区精准施策。加强与相关部门的协调联动，优化复工开工质量安全监管流程，提高监管效率。

（四）做好施工组织保障。督促工程项目单位统筹考虑复工开工材料、人员、设备、防疫等因素，精准把握工程质量安全特点、工程施工阶段风险隐患特征，及时调整施工组织，在严格防控疫情、保证质量安全的前提下科学有序复工开工。

（五）做好从业人员保障。督促指导从业单位全面掌握人员动态，关键技术人员疫情防控要精准到人，在严格防控疫情前提下，及时组织人员有序返岗。因疫情防控，管理技术人员不能及时到岗的，要制定临时顶岗方案，做好审核，暂时调整为其他符合条件的人员。对即将到期的施工企业主要负责人和安全生产管理人员考核合格证的复核工作可适当简化，确保人员在岗履职。

（六）做好安全生产条件保障。地方各级交通运输主管部门要加大对复工开工项目的安全生产条件检查力度，督促建设、监理、施工等单位落实安全生产条件相关保障措施。组织开展安全风险辨识和隐患排查，确保复工开工准备工作充分、防范措施完善、隐患排查治理彻底。

三、有效加强复工开工质量安全监管

（七）加强工程质量管控。压实建设单位首要责任和施工企业主体责任，严格执行施工质量管控程序，切实把好材料设备进场关、工序质量验收关、工程质量评定关，确保质量标准不降低。发挥试验检测的保障作用，做到严格遵守规程、数据真实可靠、资料完整齐全。严肃查处不按设计、不按方案施工以及偷工减料、数据造假等违法违规行为。

（八）加强施工安全监管。压实企业主体责任，加强专项施工方案管理，保证方案编制质量，严格审核和审查程序，有效指导工程施工。加强安全生产经费管理，严格提取比例、完善管理制度、做到专款专用。督促从业单位根据安全和技术特点，配备施工经验、专业能力等与项目规模、技术难度相匹配的技术和管理人员；增强复工开工进场人员安全教育、岗前培训和安全技术交底的针对性。持续推进“平安工地”建设常态化。

（九）严格“红线问题”查处。按照“坚守公路水运工程质量安全红线”专项行动要求，制定方案、建立台账、强化整改、动态清零。要加强对高墩索塔、不良地质隧道、路堑高边坡、深基坑、临边临水作业、沉箱浮运、船闸闸室、工程围堰等关键部位的安全隐患排查治理。全面排查关键部位临时结构的设计验算、设置部位、安装形式、维护更换、作业环境等方面的问题和隐患。

（十）加强隧道施工安全管理。加强隧道施工超前地质预报和监控量测管理，严格方案审批，狠抓方案实施。按照设计和施工方案严格控制开挖、初支、二衬施工步距，加强火工品安全管理。加强数据分析研判，充分利用监测成果指导施工，有效防控安全风险，严防坍塌、瓦斯爆炸（突出）、火药爆炸、突泥涌水等事故。

（十一）加强特种设备安全管理。督促施工单位使用质量合格的架桥机、龙门吊等特种设备，建立设备、操作人员等安全技术档案；聘请有资质的单位进行设备安装、拆卸，严格按规定进行设备验收、报检。督促施工单位、生产厂家、监理单位严格做好挂篮、爬模等非标专用设备的联合验收工作。严禁未经检验的特种设备、未经联合验收的非标专用设备，或者检验、验收不合格的设备进场。

（十二）加强现场施工安全防护。督促施工单位按规定配备安全防护用品、搭设安全作业平台、设置安全防护设施，严禁无防护作业。严查违规和冒险作业行为，坚决杜绝“三违”现象。

四、严格做好疫情防控和应急工作

（十三）认真落实疫情防控工作要求。要按照属地疫情防控要求，严格落实疫情防控责任，完善疫情防控措施和检查、报告制度，将疫情防控工作纳入日常管理，严防疫情发生。

（十四）落实预警预案等应急工作措施。要加强对疫情、水文、气象、地质等信息的收集分析和预判，及时发布预警信息。督促从业单位完善应急管理制度，加强安全生产值班值守和信息报送工作，加强应急预案管理和实施工作，确保应急救援及时有效。

交通运输部

2020年3月6日

1. 民政部：开展“携手同心助战疫关爱保障行动” 民办函〔2020〕25号

各省、自治区、直辖市民政厅（局），新疆生产建设兵团民政局：

为切实加强对参加新冠肺炎疫情防控工作的志愿者、社区工作者、社会工作者和湖北省养老机构工作者的安全保障，民政部决定与腾讯公益慈善基金会联合开展“携手同心助战疫关爱保障行动”。现就做好有关工作通知如下：

一、充分认识开展关爱保障行动的重要意义

新冠肺炎疫情发生以来，广大志愿者、社区工作者、社会工作者迅速响应党中央、国务院号召，自觉服从地方党委和政府统一部署和各地疫情联防联控机制统筹调配，积极投身疫情防控工作，成为抗击疫情的重要力量。习近平总书记在统筹推进新冠肺炎疫情防控和经济社会发展工作部署会议上发表重要讲话时指出，社区工作人员等坚守岗位、日夜值守，广大志愿者等真诚奉献、不辞辛劳，为疫情防控作出了重大贡献；要支持广大社工、义工和志愿者开展心理疏导、情绪支持、保障支持等服务。中央应对新冠肺炎疫情工作领导小组《关于全面落实疫情防控一线城乡社区工作者关心关爱措施的通知》要求切实做好城乡社区工作者职业伤害保障，加强城乡社区工作者关爱慰问。开展好关爱保障行动是贯彻落实习近平总书记重要讲话精神和党中央决策部署的重要举措，是化解参加疫情防控工作的志愿者、社区工作者、社会工作者和湖北省养老机构工作者后顾之忧的重要途径，是致敬这些不顾安危、不畏艰难、平凡而伟大的抗疫“逆行者”的重要体现。各地民政部门要充分认识开展关爱保障行动的重要意义，进一步提高政治站位，组织实施好有关工作。

二、准确把握关爱保障行动的基本要求

此次关爱保障行动采用“专属保险+关爱补助”的方式。专属保险对象是投保前未患新冠肺炎或疑似病症，年龄介于18-70周岁，参加到疫情防控工作中的志愿者、社区工作者、社会工作者和湖北省养老机构工作者。志愿者还应当在全国志愿服务信息系统实名注册。腾讯公益慈善基金会通过采购太平洋财产保险股份公司商业保险，由保险公司负责甄别鉴定理赔资料和发放赔付款项。专属保险的保险对象、保险范围、申请流程等详细要求见附件1。关爱补助对象是2020年3月15日之前已确诊的感染重型、危重型新冠肺炎或者身故的参加疫情防控工作的志愿者、社区工作者、社会工作者和湖北省养老机构工作者。符合条件的申请人向当地地市级或者县级民政部门提出申请，由省级民政部门统一向中国慈善联合会递交申请。中国慈善联合会审核同意后将补助款项直接拨付到申请人指定账户。关爱补助的申请材料、办理流程等详细要求见附件2。

三、认真做好关爱保障行动的组织实施

各地民政部门要高度重视关爱保障行动的实施，以认真负责的态度、务实有效的举措，确保行动快速落地、有效执行。任务重的地区要成立工作专班，指定专人负责，确保行动顺利实施。要通过多种方式，将此次行动的重要意义和保障内容及时传达到有关社区、志愿服务组织、社会工作服务机构和湖北省养老机构，让大家都感受到来自党和政府、社会各界的关怀和温暖。要引导符合条件人员尽量在3月10日前领取专属保险，避免出现专属保险与关爱补助之间的空档期。要引导关爱保障对象如实提交申请材料，全面、客观、准确表达诉求，坚持诚实守信，不伪造、虚构事实。

四、不断强化疫情防控的自身保护

各地民政部门要认真贯彻落实中央应对新冠肺炎疫情工作领导小组、国务院应对新冠肺炎疫情联防联控机制以及民政部印发的系列文件要求，指导志愿服务组织和基层群众性自治组织、社会工作服务机构和养老机构等强化安全责任意识，落实安全防护措施，加强安全防护培训，引导志愿者、社区工作者、社会工作者和养老机构工作者在疫情防控工作中注意做好自身保护。不具备安全防护条件的，不得组织有关人员参与疫情防控工作。

五、积极开展感人事迹的社会宣传

广大志愿者、社区工作者、社会工作者以及湖北省养老机构工作者等积极投身疫情防控一线，用自己的实际行动践行社会责任和家国情怀，落实联防联控、群防群治要求，为打赢抗疫阻击战出力尽责，是此次疫情防控中的最美“逆行者”。各地民政部门要结合实施关爱保障行动，积极选树先进典型，充分运用广播电视、报刊杂志、微信微博和互联网等各类媒体和平台，生动讲述他们的感人故事，广泛传播他们主动担当、无私奉献的可贵品质，激励和引导更多的志愿者、社区工作者、社会工作者和养老机构工作者等为打赢疫情防控的人民战争、总体战、阻击战贡献力量。

附件：1.专属保险方案

2.关爱补助方案

民政部办公厅

2020年3月8日

1. 国务院应对新型冠状病毒肺炎疫情联防联控机制：开展新冠肺炎防控科普宣教活动 联防联控机制综发〔2020〕100号

各省、自治区、直辖市及新疆生产建设兵团应对新型冠状病毒肺炎疫情联防联控机制（领导小组、指挥部）：

为做好新冠肺炎疫情防控工作，将科普知识向城乡社区、农村地区、重点场所下沉，科普技能向重点人群和公众普及，指导各地充分发挥科普宣传主阵地的作用，开展新冠肺炎疫情防控科普宣传活动，助力打赢疫情防控阻击战，拟在全国范围组织推广使用《新冠肺炎防控手册（漫画版）》（城镇版、农村版）科普宣教活动。现将有关事宜通知如下：

一、活动范围

（一）重点地区、重点场所、重点单位、重点人群做到重点强化。特别是在湖北省、武汉市等疫情严重地区，监管场所、养老机构、精神卫生医疗机构、福利院等重点场所和重点人群，在城乡社区和农村地区，要突出加强宣传教育。

（二）做到发放范围最大化，争取重点人群人手一册，城乡居民一户一册。同时，发挥疾控、社区等人员的作用，开展解读与培训，将健康知识宣传出去，做到家喻户晓，让广大群众在抗击疫情过程中，潜移默化形成健康行为。

二、组织方式

本着快速传播、经济便捷的原则，结合各地疫情防控需要，采取属地管理、分片印发的方式，由中国人口出版社按照国家有关规定将《新冠肺炎防控手册（漫画版）》（城镇版、农村版）开放版权，并为地方出版社办理授权手续。各省份可与当地出版社协商印刷发行事宜。

三、时间和要求

（一）各省份接到通知后于3日内确定当地出版社和印发数量，由当地出版社与中国人口出版社联系人协商出版发行等事宜，填写附件1二维码对应表格并传真至中国人口出版社。

（二）各地要及时总结工作进展，于每周一将工作进展传真到国家卫生健康委疾控局，直到疫情结束。

（三）请各省份于3月9日13:00前，将工作联系人和联系方式（见附件2）传真至国家卫生健康委疾控局。

国家卫生健康委疾控局 吕冰琪、严俊

电话：010-68791182，2358，传真：010-68792342

中国人口出版社联系人：

郭弘崴 电话：18600030017

周炳然 电话：13701354530

姜淑芳 电话：13701390754

传  真：010-83519400

附件：1.《新冠肺炎防控手册（漫画版）》需求填报二维码

2.新冠肺炎防控科普宣教活动联系人反馈表

国务院应对新型冠状病毒肺炎

疫情联防联控机制综合组

2020年3月7日

1. 人社部 卫健委：做好农民工返岗复工“点对点”出行健康服务工作

各省、自治区、直辖市及新疆生产建设兵团人力资源社会保障厅（局）、卫生健康委：

为贯彻落实统筹推进新冠肺炎疫情防控和经济社会发展工作部署会议精神，按照国务院有关工作部署和《人力资源社会保障部 公安部 交通运输部 国家卫生健康委 国家铁路集团关于做好农民工返岗复工“点对点”服务保障工作的通知》（人社部明电〔2020〕4号）要求，进一步做好农民工返岗复工“点对点”出行健康服务工作，现就有关事项通知如下：

一、认真开展农民工出行健康核验。各地要按照分区分级差异化防控要求，开展农民工“点对点”出行健康服务检查，通过国家全民健康信息平台和全国一体化政务服务平台，调用全国疫情防控风险数据服务接口，查询新冠肺炎确诊病例和疑似病例、同乘同户同住密切接触者、过去14天到访地区风险等级等信息。各级人力资源社会保障部门要积极通过农民工返岗复工“点对点”服务系统、电子社保卡等渠道，引导农民工开展健康信息核验。卫生健康部门要指导做好农民工的防疫健康教育和行前体温检测工作，加大对地方健康码工作的指导，不断优化完善各项管理服务措施。

二、扎实推进农民工健康信息互认。已经使用本地健康码或健康证明的地区，要主动对接国家全民健康信息平台和全国一体化政务服务平台，积极推进省内、省际互认。已经签订省际健康互认合作备忘录的地区要加快推进落实，并逐步扩大互认范围，为人员跨省有序流动提供便利条件。除疫情防控重点地区、高风险地区外，各地在组织农民工返岗复工“点对点”出行时，要减少设置其他条件。

三、加强农民工个人信息保护。各级人力资源社会保障部门、卫生健康部门要按照中央网络安全和信息化委员会办公室关于做好个人信息保护利用大数据支撑联防联控工作通知要求，采取科学有效的技术防护措施，加强农民工返岗复工“点对点”出行服务中信息填报、查询、核验等环节的管理，确保农民工个人信息安全。

四、压紧压实农民工出行健康服务工作责任。做好农民工出行健康服务工作，是加强农民工返岗复工“点对点”服务保障、统筹推进新冠肺炎疫情防控和经济社会发展的重要环节。各级人力资源社会保障、卫生健康部门要提高政治站位，认真履职尽责，加强沟通衔接，密切配合协作，切实把农民工返岗复工“点对点”出行健康服务工作抓实抓细抓到位。

人力资源社会保障部办公厅

国家卫生健康委办公厅

2020年3月7日

1. 民政部：贯彻落实中央应对新型冠状病毒感染肺炎疫情工作领导小组《关于全面落实疫情防控一线城乡社区工作者关心关爱措施的通知》

各省、自治区、直辖市民政厅（局），新疆生产建设兵团民政局：

近日，中央应对新型冠状病毒感染肺炎疫情工作领导小组印发了《关于全面落实疫情防控一线城乡社区工作者关心关爱措施的通知》（国发明电﹝2020﹞8号，以下简称《通知》），对关心关爱疫情防控一线城乡社区工作者作出全面部署。为切实把《通知》的各项政策措施落实到位，现就有关要求通知如下：

一、切实履行牵头协调职责。各地民政部门要根据《通知》要求，在地方各级疫情防控工作领导小组领导下，牵头协调组织、宣传、发展改革、工业和信息化、财政、人力资源社会保障、卫生健康、退役军人等部门，按照职责落实好《通知》确定的八个方面关心关爱一线城乡社区工作者政策措施，确保政策措施落实到位、所需资金保障到位。

二、抓紧制定配套政策措施。各省级民政部门要会同有关部门抓紧制定报批以省级疫情防控工作领导小组名义印发的贯彻落实《通知》的政策文件或方案，做好与本地区已有政策措施的衔接。要重点推动明确由省级人民政府根据疫情防控任务指导市县制定工作补助标准，确保疫情防控一线城乡社区工作者工作补助发放到位。要协调做好城乡社区工作者职业伤害保障，特别是要确保疫情防控期间因公殉职的城乡社区工作者按照法规政策规定享受工伤保险相关待遇。

三、解决好农村社区工作者保障待遇。农村社区工作者保障待遇是城乡社区工作者队伍建设的薄弱环节。各省级民政部门要加强同本级组织、财政、人力资源社会保障等部门沟通协调，研究保障农村社区工作者工作补助、享受工伤保险待遇和社区防控经费等方面的资金来源渠道。要指导县级民政部门在当地疫情防控工作领导小组领导下，实现以县（市、区、旗）为单位，统一城乡社区工作者职业伤害保障政策措施，统筹改善工作防护条件。要科学测算农村社区防控工作经费，推动加大财政支持力度，通过增加村级组织运转经费、安排奖补资金等形式，给予适当补助。

四、加强《通知》落实宣传督导。各地民政部门要广泛宣传《通知》出台的重要意义和关心关爱政策措施，及时发现疫情防控一线城乡社区工作者先进典型，总结经验、整理事迹、大力宣传报道，激励广大城乡社区工作者继续发扬不怕吃苦、顽强拼搏、连续作战精神，当好打赢疫情防控人民战争、总体战、阻击战的排头兵。民政部将进一步加大对各地贯彻落实《通知》的督导力度。各地民政部门要在当地疫情防控工作领导小组统筹部署下加强《通知》贯彻落实情况的督促指导，确保《通知》有效落实落地。

各省（区、市）民政厅（局）和新疆生产建设兵团民政局贯彻落实《通知》工作进展情况和重要事项要及时报告民政部。

民 政 部

2020年3月6日

1. 教育部：应对新冠肺炎疫情做好2020届全国普通高等学校毕业生就业创业工作 教学〔2020〕2号

各省、自治区、直辖市教育厅（教委），有关省、自治区人力资源社会保障厅，部属各高等学校、部省合建各高等学校：

2020届全国普通高校毕业生规模达874万人。当前正值高校毕业生求职择业的关键时期，受经济下行压力和新冠肺炎疫情叠加影响，高校毕业生求职困难增多，就业形势复杂严峻。党中央、国务院高度重视高校毕业生就业工作，及时作出一系列重要决策部署。各地各高校既要充分认识当前做好高校毕业生就业工作的重要性、紧迫性，切实增强责任感和使命感；又要看到我国经济长期向好的基本面和国家出台一系列政策大力促进就业等有利因素，进一步增强和坚定做好毕业生就业工作的信心。为贯彻落实习近平总书记在统筹推进新冠肺炎疫情防控和经济社会发展工作部署会议上的重要讲话以及系列重要指示批示精神，落实国务院常务会议部署要求，多措并举做好高校毕业生就业工作，现就有关事项通知如下。

一、强化担当，加强对高校毕业生就业工作的组织领导

（一）强化统筹部署。各地各高校要强化组织领导，把促进高校毕业生就业摆上领导班子重要议事日程。各省级教育部门要深入研判本地高校毕业生就业形势，抓紧制定本地促进高校毕业生就业工作方案，加强工作部署和对高校的督导检查，确保本地高校毕业生就业局势稳定。

（二）强化部门协同。要充分发挥各省（区、市）就业工作领导小组的统筹协调作用，教育部门要主动协调并会同人力资源社会保障、发展改革、卫生健康、公安、财政等部门，加强工作协调和信息沟通，把高校毕业生作为公共就业服务的重点群体，充分用好公共就业人才服务资源，共同制定稳定高校毕业生就业的政策措施，合力促进毕业生就业创业。

（三）强化高校责任。各高校要把做好毕业生就业工作作为当前一项紧迫的政治任务，认真落实“一把手”工程，主要负责同志要亲自部署，分管领导要靠前指挥，院系领导要落实责任，进一步健全校内相关机构分工负责、协同推进、院系联动、全员参与的工作机制。要主动作为，细化本校就业工作安排，精心组织就业活动。及时掌握毕业生求职心态和就业进展，帮助学生解决就业过程中面临的困难和问题，充分体现对毕业生的关心关爱。

二、创新方式，提升网上就业服务能力

（四）组织网上就业大市场。教育系统在疫情没有得到有效缓解之前，要暂停举办各类高校毕业生现场招聘活动。要充分利用部、省、校三级联通的就业网络体系以及社会招聘网站，联合举办“2020届高校毕业生全国网络联合招聘——24365校园招聘服务”活动（24小时365天招聘活动），各地各高校要组织毕业生积极参加上述网上招聘活动。要建立严格的信息审核机制，确保招聘单位及岗位信息真实准确。各高校要及时发布毕业生学科专业及生源信息，多渠道主动联系用人单位，充分发挥学术资源、校友资源作用，调动辅导员、班主任、专业教师、研究生导师等，举全校之力为毕业生提供就业信息和服务。

（五）优化网上就业服务。各地各高校要加快建设“互联网+就业”智慧平台，丰富和完善线上业务办理相关功能，加快与人力资源社会保障部门招聘网站链接与信息共享，鼓励毕业生和用人单位通过网络进行供需对接。有条件的地区和高校要根据毕业生求职意愿和用人单位需求，实现人岗信息智能匹配、精准推送。积极推动实行网上面试、网上签约。要利用网络为留学回国毕业生提供便捷的学历学位认证服务，做好相关就业信息服务。

（六）强化线上就业创业指导。充分利用各类国家、省和高校教育资源，开发、共享一批线上就业创业精品课程和就业创业讲座视频，方便毕业生点播观看。汇总发布各地各高校毕业生就业创业政策汇编及就业创业网站等信息，方便毕业生查阅使用。

三、拓宽渠道，促进毕业生就业并增加升学深造机会

（七）促进毕业生多渠道就业。各地各高校要积极配合有关部门组织好“特岗计划”“大学生村官”“三支一扶”“西部计划”等基层项目以及事业单位、国有企业招聘，并及时发布调整后的笔试面试时间等信息。聚力服务脱贫攻坚和乡村振兴战略，引导毕业生到中西部地区、东北地区、艰苦边远地区基层，到现代农业、社会公共服务等领域就业创业。落实好基层就业学费补偿贷款代偿、考研加分等优惠政策。建立校企合作对接平台，在重点区域、重大工程、重大项目、重要领域中加强人才供需对接。深入挖掘互联网、大数据、人工智能和实体经济深度融合创造的就业机会，充分利用平台经济、众包经济、共享经济等新经济形态平台，支持毕业生以新就业形态、灵活多样方式实现多元化就业。会同有关部门落实大学生创业优惠政策，加强创业平台建设，举办中国“互联网+”大学生创新创业大赛，鼓励和支持更多毕业生自主创业。

（八）积极引导大学毕业生参军入伍。各地各高校要深入贯彻落实习近平总书记给南开大学新入伍大学生回信精神，配合兵役机关落实好国务院、中央军委关于今年征兵工作部署，针对毕业生群体开展精准宣传动员和重点征集。

（九）加大高校毕业生补充教师队伍力度。各地教育部门要积极会同有关部门，通过挖潜创新、统筹调剂等多种方式加强编制配备，招录更多高校毕业生到中小学、幼儿园特别是到急需教师的高中和幼儿园任教，落实应届公费师范生全部入编入岗，补齐缺口满足发展需要。

（十）持续推送大学生到国际组织实习任职。要加大政策支持力度，及时收集发布国际组织招聘信息，组织开展专家讲座、训练营、国际交流等活动，进一步拓宽实习任职渠道。

（十一）增加毕业生升学深造机会。扩大今年硕士研究生招生规模，主要向国家战略和民生领域急需的临床医学、公共卫生与预防医学、集成电路、软件、新材料、先进制造、人工智能等相关学科和专业学位类别倾斜，向中西部和东北地区高校倾斜。扩大今年普通高等学校专升本规模，主要由职业教育本科和应用型本科高校向产业升级和改善民生急需的专业招生，向电子信息类、计算机类、生物医学工程类和预防医学、健康服务与管理、应急管理、养老服务管理、护理等专业倾斜。

四、关心关爱，做好重点群体就业帮扶

（十二）加强思想教育和就业心理辅导。针对当前就业形势和疫情影响，各地各高校要及时了解掌握毕业生思想动态和心理状况，有针对性地开展教育引导工作。有条件的地区和高校要开通就业心理咨询和就业帮扶热线，疏导毕业生就业焦虑情绪，缓解就业心理压力。

（十三）强化湖北等重点地区和重点群体就业帮扶。扩大农村义务教育阶段学校教师特设岗位计划在湖北高校招募规模。配合有关部门，增加中央基层项目在湖北高校的招募计划。更大力度扩大湖北省普通高校专升本招生计划。举办面向湖北高校以及湖北籍学生的专场网上招聘活动，高校要协调用人单位适当延长招聘时间、推迟体检时间、推迟签约录取。同时，高校要全面掌握建档立卡贫困家庭、身体残疾等毕业生情况，实行分类帮扶和“一人一策”动态管理，优先推荐岗位。

五、规范管理，提升就业工作服务水平

（十四）维护毕业生就业权益。各地各高校要坚决反对任何形式的就业歧视，在教育系统招聘活动中，不得发布拒绝招录疫情严重地区高校毕业生的招聘信息，严禁设置性别、民族等歧视性条件和院校、培养方式（全日制和非全日制）等限制性条件。加强对学生的就业安全教育，严密防范招聘陷阱、就业欺诈、“培训贷”等不法行为，并配合有关部门予以打击。

（十五）改革完善就业统计制度。加强高校毕业生就业状况统计监测，启动高校毕业生就业状况布点监测工作。各高校要严格遵守就业签约工作“四不准”要求（不准以任何方式强迫毕业生签订就业协议和劳动合同，不准将毕业证书、学位证书发放与毕业生签约挂钩，不准以户档托管为由劝说毕业生签订虚假就业协议，不准将毕业生顶岗实习、见习证明材料作为就业证明材料），确保数据真实准确。我部将委托第三方对就业信息进行核查，各地也要建立就业状况核查机制，对发现的弄虚作假情况，要依法依规对相关责任人员严肃问责。

（十六）健全就业状况反馈机制。启动毕业生和用人单位大规模线上跟踪调查，并及时将调查结果反馈高校招生、学科专业设置和人才培养工作，促进高校专业结构调整和人才培养模式改革。

（十七）适当延长毕业生择业时间。各地各高校可视情况适当延长就业签约时间，及时为已落实工作单位的毕业生办理就业手续。要配合有关部门引导用人单位推迟面试和录取时间，对延迟离校应届毕业生推迟报到、落户等时限。要与人力资源社会保障部门做好离校未就业毕业生信息衔接和服务接续工作，为离校未就业毕业生持续提供就业服务。对离校时未落实工作单位的高校毕业生，可按规定将户口、档案在学校保留两年，并为落实单位的毕业生按应届毕业生身份及时办理就业手续。

教育部

2020年3月4日

1. 卫健委 民政部：加强应对新冠肺炎疫情工作中心理援助与社会工作服务 国卫办疾控函〔2020〕194号

各省、自治区、直辖市卫生健康委、民政厅（局），各计划单列市及新疆生产建设兵团卫生健康委、民政局：

为贯彻落实党中央、国务院关于坚决打赢新冠肺炎疫情防控阻击战的决策部署，支持和帮助湖北省武汉市等受疫情影响严重地区，减轻疫情所致的群众心理伤害和社会影响，在新冠肺炎疫情防控中加强心理援助与社会工作服务工作，现将有关事项通知如下。

一、加强新冠肺炎感染者心理援助与社会工作服务

湖北省武汉市等受疫情影响严重地区，要将心理援助与社会工作服务纳入疫情防控的整体部署，统筹安排，统一管理，在定点医院、方舱医院组织开展心理援助与社会工作服务。地方卫生健康行政部门要根据本地区原有精神卫生服务资源、医务社会工作者数量和分布，结合对口支援的精神卫生和当地医务社会工作专业力量，按照相关工作方案（附件1、2），对新冠肺炎感染者提供心理支持和心理危机干预。对有精神行为问题的感染者，及时组织精神科会诊，提供危机干预和精神科药物治疗。湖北省可结合工作实际，依托当地社会工作行业组织，遵循有关医务社会工作支援服务原则，开展方舱医院医务社会工作服务试点。

二、开展被隔离人员心理援助与社会工作服务

受疫情影响严重地区的区县应当建立卫生健康、民政等多部门协调工作机制，对接精神卫生、社会工作、志愿服务等服务资源，组建由精神卫生、心理健康专业人员、社会工作者、相关专业志愿者等组成的服务队，按照工作方案（附件3），为在集中隔离点隔离的人员提供疫情认知、健康指导和心理情绪辅导，评估心理危机，开展家庭支援、社会关系修复、政策咨询及转介等服务。应当重点关注儿童、老人、残障人士、有原发疾病等特殊需要的人员和因公殉职者家属、病亡者家属，做好困难救助、心理疏导、哀伤辅导、社会支持网络修复等社会工作服务，开展针对性评估和危机干预、转介服务。

三、强化一线工作人员心理支持与社会工作服务

受疫情影响严重地区的卫生健康行政部门和民政部门要组织当地精神卫生、心理健康和社会工作服务专业队伍，采取“线上+线下”的方式，对在疫情防控一线工作的医护人员、疾控人员等医务人员和坚守工作岗位的公安、交通等人员和基层社区工作人员提供心理援助。各级民政部门应当动员引导广大社会工作者和志愿者，对一线工作人员及家属提供心理疏导、情绪支持、保障支持等服务。定点医院、方舱医院和集中隔离点应当创建适宜的环境和条件，建立适当的心理援助与社会工作服务渠道，帮助一线工作人员进行自我心理调适。

四、严守心理援助伦理规范，做好服务管理和效果评估

参与心理援助与社会工作服务的人员应当在党委和政府的统一领导下开展工作，遵守心理工作、社会工作服务的伦理规范、恪守职业道德，承诺严格保护受助者个人隐私、严格遵守信息发布规则。各地卫生健康行政部门、民政部门要加强对心理援助与社会工作服务的管理，建立心理援助工作报告制度，及时组织开展效果评估。

五、加强组织保障

各地卫生健康行政部门、民政部门要充分认识做好心理援助与社会工作服务的重要意义，将心理援助与社会工作服务纳入疫情防控整体工作中进行部署，加强对心理援助与社会工作服务工作的组织领导和工作指导。要为参与工作的人员提供必要的防护物资及工作场地。要将工作开展中涌现的先进事迹、优秀人物、典型经验与好的做法进行广泛宣传，对具有突出贡献的团队和个人进行奖励嘉许。

附件：1.新冠肺炎治疗定点医院心理援助与医务社会工作服务工作方案

2.方舱医院心理援助与社会工作服务工作方案

3.新冠肺炎防控集中隔离点心理援助与社会工作服务工作方案

国家卫生健康委办公厅  民政部办公厅

2020年3月5日

1. 人社部：做好农民工返岗复工“点对点”出行线上服务工作 人社厅明电〔2020〕14号

各省、自治区、直辖市及新疆生产建设兵团人力资源社会保障厅（局）：

为坚决贯彻党中央、国务院关于做好疫情防控和复产复工决策部署，落实《人力资源社会保障部 公安部 交通运输部 国家卫生健康委 国家铁路集团关于做好农民工返岗复工“点对点”服务保障工作的通知》（人社部明电〔2020〕4号）要求，提高农民工返岗复工“点对点”服务保障工作效率，保障成规模、成批次外出的农民工安全有序返岗复工，现就有关事项通知如下：

一、启用农民工返岗复工“点对点”服务系统。我部于2020年3月5日开通全国统一的农民工返岗复工“点对点”服务系统。该系统支持农民工通过电子社保卡、小程序等方式，在线填报出行需求信息；支持农民工出行健康信息核验；支持各级人力资源社会保障部门分类提取农民工出行需求信息。各地在开展农民工返岗复工“点对点”服务保障工作时，要积极通过该系统采集农民工返岗复工出行需求信息，开展各项服务。对于已经开通线上信息采集系统的地区，已有信息采集指标或调整后信息采集指标能够满足部农民工返岗复工“点对点”服务系统采集需求的，可继续使用原本地系统。对于未开通线上信息采集系统的，原则上要求使用全国统一的农民工返岗复工“点对点”服务系统。

二、加强出行需求信息运用。各级人力资源社会保障部门要指定专人负责，通过我部网址（http://wgfw.mohrss.gov.cn），查询下载农民工填报的出行需求信息。各级人力资源社会保障部门接收下载的人员信息已经系统比对全国新冠肺炎疫情防控风险数据，其中健康状况均为“未见异常”。各地要按照出行时间相近、务工目的地相近、是否已有工作岗位等条件做好数据分类应用，及时联系农民工本人和企业，做好组织出行。对于系统中的建档立卡贫困人口，在农民工返岗复工“点对点”服务保障工作中要重点帮扶。对于有意愿外出务工但无工作岗位的农民工要加大就业服务力度。

三、做好出行服务衔接。各级农民工返岗复工“点对点”服务协作机制联络员，负责每日汇总本地农民工“点对点”出行信息。输出地和输入地的人力资源社会保障部门要加强工作对接，及时共享农民工返岗复工“点对点”出行需求信息，并做好与交通运输、卫生健康、铁路集团公司等部门和单位的信息交换与衔接。省级农民工返岗复工“点对点”服务协作机制要加强调度和指导。

四、加大宣传推广力度。各级人力资源社会保障部门要运用官方网站、官方公众号、就业信息平台等渠道，通过制发、张贴小程序二维码等方式，广泛宣传农民工返岗复工“点对点”服务系统，动员广大农民工通过该系统填报出行需求信息，提高“点对点”工作服务效率。各级农民工返岗复工“点对点”服务协作机制要充分发挥统筹协调作用，推动有关成员单位加大对农民工返岗复工“点对点”服务系统的宣传力度。

人力资源社会保障部办公厅

2020年3月6日

1. 人社部：做好新冠肺炎疫情防控一线专业技术人员职称工作 人社厅发〔2020〕23号

各省、自治区、直辖市及新疆生产建设兵团人力资源社会保障厅（局），国务院有关部门人事机构：

为贯彻落实习近平总书记关于疫情防控系列重要讲话精神，进一步突出品德、能力、业绩导向，更好地发挥职称评价“指挥棒”作用，鼓励和引导专业技术人员积极投身疫情防控一线，根据中央应对疫情工作领导小组《关于全面落实进一步保护关心爱护医务人员若干措施的通知》要求，现就做好新冠肺炎疫情防控一线卫生专业技术人员和科研攻关人员等（以下简称一线专业技术人员）职称工作有关事项通知如下。

一、优先申报评审。参加疫情防控的一线专业技术人员参加职称评审时，用人单位不受本单位岗位结构比例限制，优先推荐申报，优先评审。职称评审同等条件下向疫情防控一线专业技术人员倾斜。

二、开辟绿色通道。参加疫情防控的一线专业技术人员，可提前一年申报评审高一级职称或参加专业技术资格考试。做出突出贡献，获得省部级以上表彰奖励的，开辟职称评审绿色通道，结合业务能力水平评价，可直接申报参加高一级职称评审或考试。

三、突出抗疫表现。将抗疫表现作为职称评审的重要内容，鼓励一线专业技术人员救死扶伤、严谨求实、顽强拼搏、无私奉献。将一线专业技术人员参加疫情防控的工作成果和贡献列入年度职称评价指标，作为加分项。破除“唯论文”倾向，疫情防控一线专业技术人员参加职称评审，对论文不作硬性要求，疫情防控中的临床救治情况、病案病例、诊疗方案、关键核心技术研发成果、流行病学报告、病理报告、药物疫苗研发情况、试剂检测设备产品研发应用情况、工作总结、心理治疗和疏导案例等均可作为成果申报参评。对职称外语、计算机应用能力不作要求，免于参加专业实践能力考核。一线专业技术人员参加疫情防控经历可视同为一年基层工作经历，视同完成当年继续教育学时学分。要通过职称评审，鼓励广大专业技术人员紧紧围绕疫情防控，维护人民群众生命健康，突破关键核心技术，加强疫病防控和公共卫生攻关体系建设，把论文写在临床一线，把研究成果应用到疫情防控一线。

四、优先岗位聘用。参加疫情防控的一线专业技术人员晋升岗位等级不受本单位岗位结构比例限制。评审通过的，用人单位将其直接聘任到相应专业技术岗位。

五、优化评审服务。各地要视疫情防控工作情况合理安排职称评审，给一线专业技术人员适当休整时间，不搞突击性申报、评审。职称申报要简化材料、优化程序，不给一线专业技术人员增加额外负担，使一线专业技术人员心无旁骛投身工作。评审通过后，要督促做好后续岗位聘用、兑现工资待遇等有关工作。

本通知自印发之日起施行，适用于在新冠肺炎疫情防控一线工作的卫生专业技术人员，以及参加新型检测试剂、抗体药物、疫苗、诊疗方案、病毒病原学和流行病学研究等疫情防控科研攻关一线人员等，具体范围由各地结合实际研究确定。通过绿色通道享受提前申报、优先晋升岗位等级的人员，原则上只享受一次政策优惠。

做好职称工作是关心关爱一线专业技术人员的重要举措。各地人力资源社会保障部门要以高度的政治责任感抓好落实，认真倾听一线专业技术人员所急所盼，与卫生健康、科研等有关部门和单位加强沟通，密切合作，共同把疫情防控一线专业技术人员的职称工作抓实抓细抓落地，为坚决打赢疫情防控阻击战提供坚强有力的人才保障。

人力资源社会保障部办公厅

2020年3月4日

1. 知识产权局：严厉打击与新冠肺炎疫情相关非正常商标申请代理行为 国知办函运字〔2020〕149号

各省、自治区、直辖市及新疆生产建设兵团知识产权局（知识产权管理部门），四川省知识产权服务促进中心：

近日，一些代理机构受申请人委托，违背社会道德和人类良知，将与新冠肺炎疫情防控相关、作为商标使用易造成社会不良影响的标志申请注册商标，引发社会各界极大关切。为深入贯彻习近平总书记重要讲话和指示批示精神，落实党中央、国务院关于统筹抓好疫情防控和经济社会发展的决策部署，打击恶意抢注行为，按照国家知识产权局党组关于在疫情期间强化行业监管的要求，现就疫情期间严厉打击代理非正常商标申请行为通知如下：

一、加大监控排查力度

目前，我局相关部门已对代理有关非正常商标注册申请业务的代理机构进行筛查，并已转送所在省（区、市）知识产权局查办。今后还将持续加大对代理与疫情防控相关非正常商标申请行为的监控排查力度，及时转送查办。

二、依法加强监管查处

各省（区、市）知识产权局要根据我局转办的代理非正常商标申请行为线索，立即组织立案调查，对不当行为责令其立即整改；对存在违法违规行为的，报请我局依法停止受理其办理商标代理业务；对触犯刑法等相关法律的，及时移送相关部门处理。

三、指导加强行业自律

全国性相关行业协会要充分发挥行业自律作用，各省（区、市）知识产权局要指导当地相关行业协会加强行业自律，强化业内自律监督，对存在严重违法违规行为的会员单位及时予以惩戒，对非会员单位依法依职责予以通报曝光、公开谴责。

四、切实加强信用联合惩戒

各省（区、市）知识产权局要加快建立健全商标代理信用记录档案，将严重违法违规等行为记入代理机构和个人档案，及时报送我局并向社会公布，同时依照规定与相关部门开展联合惩戒，并在有关知识产权扶持激励政策实施、品牌机构培育、人才选拔等工作中予以严格限制或取消资格。

各省（区、市）知识产权局要高度重视，将此项工作作为知识产权系统认真落实党中央、国务院关于统筹抓好疫情防控和经济社会发展决策部署的一项重要任务，处理好严格监管和促进发展的关系，切实加大力度，抓实抓细，抓好落实，并按照有关要求及时将调查整改、依法查处等落实情况报送我局运用促进司。

特此通知。

国家知识产权局办公室

2020年3月4日

1. 中央应对新型冠状病毒感染肺炎疫情工作领导小组：进一步做好疫情防控期间困难群众兜底保障工作 国发明电〔2020〕9号

各省、自治区、直辖市党委和人民政府，新疆生产建设兵团，中央和国家机关有关部门：

为更好解决疫情防控期间部分群众面临的突发性、紧迫性、临时性生活困难，以及保障特殊困难人员基本照料服务需求，切实做好兜底保障工作，织密织牢社会安全网，坚决打赢疫情防控人民战争、总体战、阻击战，现就有关要求通知如下：

一、保障好疫情防控期间困难群众基本生活

坚持应保尽保、保障到位。各地要统筹使用中央财政困难群众救助等补助资金和地方各级财政安排资金，及时足额发放低保金、特困供养金、孤儿基本生活费，以及困难残疾人生活补贴和重度残疾人护理补贴，切实保障好困难群众基本生活。疫情比较严重的地区，可适当增加困难群众生活补助，所需资金由地方财政负担。对于受疫情影响无法外出务工、经营、就业，收入下降导致基本生活困难的城乡居民，符合条件的要及时纳入最低生活保障范围。对外出务工、返岗复工的低保对象，在计算家庭收入时适当扣减务工成本。

及时足额发放价格临时补贴。密切关注物价变动情况，物价涨幅达到规定条件时，及时启动实施社会救助和保障标准与物价上涨挂钩联动机制，按时足额向低保对象、特困人员等困难群众发放价格临时补贴，有条件的地方可以适当提高补贴标准。

做好贫困人口救助帮扶。对符合条件的建档立卡贫困人口，要及时纳入低保、特困供养、临时救助范围。对受疫情影响致贫的其他人员和返贫的建档立卡贫困人口，要及时落实临时救助等社会救助政策，确保其基本生活不受影响。

加大新冠肺炎患者及受影响家庭救助力度。对确诊病例中的低保对象、特困人员、低收入家庭成员以及建档立卡贫困人口，按规定及时给予临时救助，可一事一议加大救助力度。对因家庭成员被隔离收治导致基本生活出现暂时困难的家庭，由当地街道（乡镇）或县级民政部门实施临时救助。对生活困难的患者及其家庭，按规定及时纳入低保、特困供养或临时救助范围；对其中的病亡人员家庭，加大临时救助力度。

各地对基本生活受疫情影响，其他社会救助制度暂时无法覆盖的困难群众，要通过临时救助做到凡困必帮、有难必救。湖北省和武汉市以及其他疫情严重地区，可委托社区（村）实施“先行救助”，根据急难情形提供物质帮助或服务，发现困难立即救助。

二、保障好陷入临时困境外来人员基本生活

做好临时滞留人员帮扶工作。对疫情防控期间，因交通管控等原因暂时滞留，在住宿、饮食等方面遭遇临时困难的人员，各地要根据其基本生活需要，及时提供临时住宿、饮食、御寒衣物等帮扶。

做好其他外来人员救助工作。对受疫情影响，找不到工作又得不到家庭支持，基本生活出现暂时困难的外来务工人员，各地要按规定给予临时救助。符合生活无着的流浪乞讨人员救助条件的由救助管理机构实施救助。

做好发现引导帮扶救助工作。湖北省和武汉市以及其他疫情严重地区，公安、城管、疾控和城乡社区工作人员在巡查、排查时，发现上述外来人员有发热、干咳等症状的，要立即安排其接受集中隔离医学观察，对其他外来人员要引导或协助其向救助管理机构求助。救助管理机构要按要求做好帮扶和救助工作。救助管理机构收住能力饱和、不能满足求助人员临时住宿需求的，要开辟临时庇护场所，增强收住能力，切实做到应救尽救。救助管理机构要切实加强防控措施和内部管理，防止出现聚集性感染。

三、保障好特殊困难人员基本照料服务需求

妥善照顾由被隔离收治人员负责监护或照料的对象。各地承担隔离收治确诊患者、疑似患者、发热患者、密切接触者任务的机构和工作人员，要主动向上述人员了解由其负责监护或照料的对象的情况，对由其监护或照料的对象中生活不能自理的老年人、残疾人、未成年人，特别是分散供养特困人员，要及时通知所在社区（村）上门探视和联系安排相关人员或机构提供监护或照料。

主动探视和及时帮助居家隔离特殊困难人员。各地对居家隔离的孤寡老人、社会散居孤儿、留守儿童、留守老年人以及重病重残等特殊困难人员，要保持经常联系，加强走访探视，及时提供帮助。

四、确保困难群众求助有门、受助及时

各地要公布并畅通求助热线，简化工作流程，健全转介机制，明确主体责任，确保及时受理和回应困难群众求助。要运用“互联网+”、大数据等技术手段，积极推行社会救助全流程线上办理，加快办理速度。疫情防控期间，为减少人群聚集和感染风险，各地要充分利用社会救助家庭经济状况核对机制，可采用非接触、远距离等灵活方式开展入户调查，按规定及时公布有关社会救助事项经办结果，主动接受社会监督。为增强低保对象抵御风险经济能力，各地可根据疫情形势决定暂停开展低保对象退出工作。

五、切实加强组织领导

强化属地责任。各地党委和政府要按照《国务院应对新型冠状病毒感染肺炎疫情联防联控机制关于进一步做好民政服务机构疫情防控工作的通知》（国发明电〔2020〕6号）和本通知要求，进一步深化细化实化相关政策措施，统筹研究，同步推进，担当履责，协同配合，坚决抓好贯彻落实，扎实做好重点单位、重点场所、重点人群的疫情防控和民生保障工作，确保不发生冲击社会道德底线的事件。

强化资金保障。地方财政要加大困难群众基本生活保障资金投入，中央财政继续通过困难群众救助补助资金支持地方开展社会救助工作，对湖北省予以重点倾斜。各地要加快资金拨付进度，按要求尽快将中央及省级财政安排的救助补助资金拨付到位，为做好受疫情影响困难群众基本生活保障提供有力支撑。

强化监督检查。各地要严肃纪律，依纪依法坚决迅速查处工作落实中作风漂浮、敷衍塞责、推诿刁难、弄虚作假、不作为等问题。进一步加强资金监管，及时查处和曝光虚报冒领、截留私分、贪污挪用等问题。要激励党员干部、一线工作人员担当作为，对非主观故意将不符合条件人员纳入救助帮扶范围的，可免予追究相关责任。

强化社会支持。要加强舆论引导，及时宣传党和政府保障基本民生的惠民政策和有力举措，进一步强信心、暖人心、聚民心。要动员引导有关社会组织、心理工作者、志愿者积极参与，通过热线电话、微信等方式，为被隔离收治人员及其家属、病亡人员家属提供心理援助、情绪疏导等服务。

中央应对新型冠状病毒感染

肺炎疫情工作领导小组

2020年3月6日

1. 交通运输部：进一步做好公路水运工程疫情防控有序精准推动复工开工

各省、自治区、直辖市、新疆生产建设兵团交通运输厅（局、委）：

　　为进一步落实党中央、国务院部署，在严格防控新冠肺炎疫情的前提下，积极有序推动公路水运工程复工开工，为保持经济平稳运行作出积极贡献，现就有关事项通知如下：

　　一、精准复工复产

　　（一）精准分区推进。

　　在当地党委政府和疫情防控机制领导下，在卫生健康部门指导下，按照分区分级的要求，分类指导各地区有关单位制定完善疫情防控措施、预案及复工安排。低风险地区尽快将防控策略调整为外防输入，推动全面复工；中风险地区要依据防控形势有序复工；高风险地区继续集中精力抓好疫情防控工作。天气寒冷地区暂不具备条件的，要做好备工备料等复工准备，积极推进受天气影响较小的隧道等工程施工。

　　（二）精准分工负责。

　　除疫情防控任务较重省份外，其他省级交通运输主管部门要结合本地区实际情况和项目具体情况，在严格防控疫情的前提下积极有序推进复工复产，加强监督管理，督促指导相关单位切实履行疫情防控主体责任，协调地方政府和有关部门保障有利建设环境。建设单位要组织参建单位统筹做好疫情防控和复工复产，落实人员返岗、材料进场计划并协调保障建设环境等，在疫情防控措施到位的前提下加快推进工程建设，加快工程款计量支付。施工单位及其他相关参建单位要积极组织人员返岗，落实疫情防控责任和要求，加快施工进度，确保工程质量安全。

　　（三）精准督导项目。

　　按照项目属性、特点和各地区实际情况，在严格防控疫情的前提下分类推进项目复工，抢抓春季施工。除疫情防控重点地区和天气寒冷地区外，高速公路和重点水运工程建设项目要加大复工力度，加快施工进度；普通国省干线公路和其他水运工程建设项目要因地制宜加快推进，未复工项目力争3月15日前复工；跨年度的农村公路项目，具备条件的可有序组织复工。贯彻落实国家重大区域发展战略，助力打赢脱贫攻坚战，全力以赴保障重大项目和脱贫攻坚兜底性项目，注重发挥跨江跨海通道、山区高速公路、航电枢纽、干线高等级航道等重大工程示范带动作用。

　　（四）精准推进建设。

　　跨地区的项目，要加强地区间协作协调，根据各地区疫情风险等级和施工单位、劳务班组具体情况，差别化制定复工方案，精准落实分标段复工安排，在严格防控疫情的前提下尽早实现全面复工。加快实施相对易于封闭的桥梁、隧道、疏浚、清礁等工程。抓紧实施度汛工程关键环节，确保安全度汛和后续工程顺利实施。因疫情防控难以及时到位的少数施工单位和班组，要根据施工组织需要，加强标段之间、班组之间统筹，鼓励相互支持，确保完成投资任务。要避免在建项目长时间停工造成损失浪费，防止形成“半拉子”工程。

　　（五）精准落实人员。

　　建设单位和施工、监理、检测、设计等参建单位要梳理参建人员情况，确保参建人员尽快就位履职。农民工输出、输入地交通运输主管部门可配合当地有关部门，积极衔接沟通，对目的地集中、具备一定出行规模的农民工群体，可组织采用“点对点”运输方式，确保顺利返岗。根据人员到位状况，合理优化施工组织和管理措施，确保管理体系健全、运转有效、保障有力，避免因人员不足导致生产脱节、管理脱节。因疫情管控不能及时到岗的管理技术人员，允许安排具备相应条件的其他人员暂时顶岗，或临时通过网络办公、视频会商等方式开展工作，不作为人员履约问题。

　　（六）精准制定政策。

　　依照有关规定，疫情属不可抗力因素。疫情防控有关投入纳入工程建设成本，防控费用的具体计列结算方式以及有关合同工期调整原则等，由省级交通运输主管部门加强指导，参建单位依据合同约定和相关规定进行处理。针对疫情防控期间新情况、新要求，及时了解工程项目和参建单位面临的实际困难，积极加强指导、协调和服务，改进监管方式，及时传达国家有关支持政策，更好地推动复工复产。

　　二、加快投资建设

　　（七）加快启动新项目。

　　组织实施“十三五”规划备选项目。提前启动一批支撑国家战略实施、符合“十四五”规划方向的建设项目，优先实施国家高速公路待贯通路段建设、交通繁忙路段扩容改造、内河高等级航道和沿海重要港区公共基础设施建设，推进普通国省道提质改造、剩余待建路段贯通以及城镇过境段改造。结合实施脱贫攻坚与乡村振兴战略，推动老旧县乡道提级改造、较大人口规模自然村通硬化路建设。积极推动重点货类专业化码头建设。

　　（八）加快前期工作。

　　根据疫情防控要求和工作安排，改进工作方式，分区分级精准统筹推进项目前期工作。抓紧开展现场勘察，充分利用无人机、卫星遥感和BIM技术等，加强工程现场图片和视频资料收集、分析，加快工程可行性研究报告、勘察设计文件编制等工作。在符合保密要求前提下，采用视频会商、网络办公、资料邮寄等方式，做好内业设计及文件内审等工作。加大与自然资源、生态环境、财政等部门的沟通协调力度，加快完善审批要件。

　　（九）加快审查审批。

　　按照国家有关规定，优化审批流程。对于需报部审查审批的项目，部将调整、改进疫情防控期间审批方式，及时安排咨询评估，符合条件的项目将及时出具资金安排意见，及时批复初步设计。在符合保密要求前提下，充分利用在线审批平台，或采用邮寄等方式报送文件资料；通过图文资料、视频信息相结合等方式核实了解工程现场情况，弥补难以现场踏勘的不足；采取视频会商等方式开展技术评审，加快批复文件办理。特殊复杂工程通过以上方式难以支撑技术决策的，可增加技术设计或专题论证，重要问题后置。疫情过后，可酌情增加设计回访或现场调研，更好地指导工程建设。

　　（十）加快招标工作。

　　积极对接当地公共资源交易中心，采取有效措施积极应对疫情防控对招标投标各环节的影响。充分利用信息化手段，积极推进招标投标备案、评标专家抽取等全程网上办理。鼓励优先采用电子招标，推行远程异地开标。暂未实现电子招标的省份，要积极探索邮寄或线上获取招标投标文件、采用远程视频开标等方式，实现“不见面开标”，确保招标投标顺利进行。

　　（十一）加快开工建设。

　　对符合条件的项目，加快办理施工许可。用足用好相关政策，积极协调有关部门加快办理各项审批备案手续，尽快做好施工准备，在严格防控疫情的前提下尽早进场施工。按照《交通运输部财政部对交通建设领域真抓实干成效明显地方进一步加大激励支持力度的实施方案》（交规划发〔2019〕40号），部将继续对推进有力的地区实施相关激励。

　　三、加强疫情防控和质量安全监督管理

　　（十二）落实复工开工条件。

　　根据疫情防控总体要求和属地统一部署，强化质量安全、卫生防疫等岗前交底培训。结合项目特点，加强疫情防控措施核查、复工开工安全生产条件审查和隐患排查治理，做到准备工作充分、防范措施落实、隐患治理彻底。

　　（十三）严格工地疫情防控。

　　督促和指导项目单位及各参建单位，按照“谁用工、谁管理、谁负责”原则，落实属地要求，履行疫情防控主体责任，结合项目实际情况严格做好疫情防控工作。在卫生健康部门指导下，完善疫情防控制度和预案，落实防控经费、物资和人员，加强对重点场所消毒、通风，做好人员排查登记和体温检测，结合疫情防控需要科学合理组织施工，在严格防控疫情的前提下有序复工。

　　（十四）严格质量安全管理。

　　指导有关单位做好风险研判，督促参建企业制定质量安全措施和工作方案，落实质量安全责任，严格按图纸和规范施工，严禁抢赶工期、冒险作业等行为。充分考虑疫情影响，协助企业解决实际困难，把好材料设备进场关、工序验收关、质量评定关，确保工程质量标准不降低。

　　（十五）加强监督管理。

　　鼓励通过信息化方式办理质量监督手续及相关监管事项，采用远程监控等方式及时了解工程现场质量安全状况。可适当简化即将到期的施工企业主要负责人和安全生产管理人员考核合格证书的复核工作。采取有效措施，加强对建设单位和各参建单位疫情防控工作的监督检查。

　　（十六）做好应急保障。

　　加强对疫情、水文、气象、地质等信息的收集分析和预判，及时发布预警信息，落实监控措施。加强安全生产、疫情防控值班值守和信息报送工作，完善应急预案，提前做好物资、设备和人力储备与检查，落实落细各项应急准备工作，确保应急救援抢险、疫情处置等工作及时有效。

　　四、夯实工作基础

　　（十七）加强组织领导。

　　各地交通运输主管部门和各有关单位要提高政治站位，强化责任意识，充分认识加快复工开工建设、加大交通投资力度的重要意义，统筹考虑疫情防控要求和建设项目实际情况，加强组织领导，积极有序推动企业复工复产和项目开工建设。对重点地区、重点企业、重点项目，给予重点指导和支持，强化激励保障措施。建立项目复工台账，分类督促指导。有关情况及典型经验、问题及时报部。

　　（十八）积极促进就业。

　　工程建设中，注重吸纳劳动力就业，优先保障贫困地区、贫困劳动力就业。农村公路建设、养护可因地制宜，优先吸纳当地农民工就业。切实保障农民工生产生活条件，确保疫情防护措施到位，做好农民工工资支付有关工作。各有关单位要运用多种方式加大宣传力度，解除农民工后顾之忧。

　　（十九）拉动有效需求。

　　充分发挥交通基础设施建设基础性、先导性作用，以有效投资拉动有效需求，带动相关产业尽快复工复产。鼓励在保证质量的前提下，提前备工备料。指导建设单位、施工单位积极对接水泥、沥青、钢材、油料、砂石料、机电设施、工程机械等材料设备供应单位，衔接生产供应计划，及时主动发布招标计划和相关需求，有序恢复生产供应。

　　 交通运输部

　　 2020年3月4日

（155） 生态环境部：统筹做好疫情防控和经济社会发展生态环保工作	环综合〔2020〕13号

各省、自治区、直辖市、新疆生产建设兵团、副省级城市生态环境厅（局）：

新冠肺炎疫情发生后，党中央、国务院高度重视。习近平总书记多次作出重要指示批示、召开会议研究部署、进行现场调研指导。中央应对疫情工作领导小组及时部署推进。全国生态环境系统狠抓落实，不断强化相关环境监管和服务措施，全力支撑保障打赢疫情防控的人民战争、总体战、阻击战。当前疫情防控工作正处于最吃劲的关键阶段，企业复工复产正在有序推进。为深入贯彻习近平总书记重要指示批示精神和党中央、国务院部署要求，统筹推进疫情防控、经济社会发展和生态环境保护，确保完成全面建成小康社会、“十三五”规划以及污染防治攻坚战阶段性目标任务，提出如下意见。

一、落实“两个100%”，抓紧抓实抓细疫情防控生态环境保护工作

以全国所有医疗机构及设施环境监管和服务100%全覆盖，医疗废物、废水及时有效收集转运和处理处置100%全落实为主要目标，全力以赴做好疫情防控相关环保工作。

（一）严格做到医疗废物废水应收尽收和应处尽处

以定点医疗场所为重点，全面摸清各地疫情医疗废物、医疗废水产生、收集、转运、贮存和处理处置情况，确保应收尽收和应处尽处。

以湖北省为重点，武汉市为重中之重，加快补齐医疗废物、危险废物收集转运和处理处置设施短板，做好区域协同、医疗废物与危险废物协同、固定设施处置与移动设备处置协同，科学调配应急处置能力。全力做好北京疫情防控环保工作。推动医疗机构源头分类收集疫情医疗废物，督促处置单位优先收运和处置疫情医疗废物，保障集中处置设施稳定运行。

持续加强医疗废水和城镇污水收集、处理、消毒等关键环节的监督管理，严禁排放未经消毒处理的医疗废水。

（二）强化环境应急管理

全力做好疫情地区大气、地表水等环境质量监测，重点强化饮用水水源地水质监测，增加余氯特征指标，及时公开监测信息，加快提升应急监测能力。

加强企业复工复产突发环境事件应急准备，抓实抓细环境应急预案修订，对环境应急物资、装备和技术查漏补缺。坚持24小时应急值守。

（三）严密内部疫情防控

把人员生命安全和身体健康放在第一位，按照属地管理原则，严格遵守地方党委、政府疫情防控有关规定，认真执行严要求、严报告、严隔离、严管控、严返岗等“五严”措施，切实做好内部疫情防控工作。

二、建立“两个清单”，积极支持相关行业企业复工复产

建立和实施环评审批正面清单和监督执法正面清单，着力提高工作效能，积极支持相关行业企业复工复产，更加有力支撑保障疫情防控和促进经济社会平稳健康发展，落实精准治污、科学治污、依法治污，加快推进生态环境治理体系和治理能力现代化建设。两个清单实行时间原则上截至2020年9月底，根据形势需要可适当延长。对行之有效、广泛认可的措施，可固化形成长效机制。鼓励地方因地制宜，创造性地加以贯彻落实。

（四）制定实施环评审批正面清单，便利项目开工建设

豁免部分项目环评手续办理。继续落实好已出台的新冠肺炎疫情防控期间急需的医疗卫生、物资生产、研究试验等三类建设项目环评应急服务保障政策。加强与排污许可制衔接，对照《建设项目环境影响评价分类管理名录》（以下简称《名录》），对关系民生且纳入《固定污染源排污许可分类管理名录（2019年版）》实施排污许可登记管理的相关行业，以及社会事业与服务业，不涉及有毒、有害及危险品的仓储、物流配送业等10大类30小类行业的项目，不再填报环境影响登记表。

拓展环评告知承诺制审批改革试点。将环境影响总体可控、受疫情影响较大、就业密集型等民生相关的部分行业纳入环评告知承诺制审批改革试点，包括工程建设、社会事业与服务业、制造业、畜牧业、交通运输业等多个领域，共涉及《名录》中17大类44小类行业。

强化项目环评审批服务。动态更新国家层面、地方层面和利用外资层面重大项目环评审批服务“三本台账”，提前介入，开辟绿色通道，提高审批效率。对复工复产重点项目、生猪规模化养殖等项目，采取拉条挂账方式，主动做好环评审批服务。创新环评管理方式，公开环境基础数据，优化管理流程，实现“不见面”审批。

（五）制定实施监督执法正面清单，发挥激励导向作用

免除部分企业现场执法检查。对与疫情防控物资生产和民生保障密切相关的，污染排放量小、吸纳就业能力强的，涉及重大工程和重点领域的管理规范、环境绩效水平高的企业，不进行现场执法检查。

推行非现场监管方式。充分利用遥感、无人机巡查、在线监控、视频监控、用能监控、大数据分析等科技手段开展非现场检查，及时提醒复工复产企业正常运行治污设施。

落实“双随机、一公开”监管。规范行使行政处罚自由裁量权，严格依照法定权限和程序执法，审慎采取查封、扣押和限制生产、停产整治措施。对因受疫情防控直接影响，环境违法行为轻微并及时纠正且未造成环境危害后果的，可以不予处罚，督促尽快整改。对因受疫情防控直接影响而未能按期完成整改的，可以酌情延长整改期限。

对偷排偷放、恶意排污、监测数据弄虚作假，以及涉疫情医疗废物、医疗废水，侵害群众健康、群众反映强烈、严重污染环境的环境违法犯罪行为，要坚决依法查处。

（六）加大技术帮扶，协助企业解决治污难题

利用国家生态环境科技成果转化综合服务平台，做好环境污染治理方案和技术的需求方和供给方对接，为企业免费提供技术咨询服务。引导鼓励工业园区和企业推进第三方治理，推广环境医院、环保管家、环境顾问等服务模式。做好“结对定点帮扶”“送政策送技术送服务”等活动。

（七）积极推动和配合落实相关政策，缓解企业资金困难

充分发挥国家绿色发展基金作用，鼓励有条件的地方、金融机构和社会资本设立区域性绿色发展基金。加强污染防治资金项目管理，向受疫情影响较重的地区和疫情防控重点工作倾斜。对受疫情影响严重且符合污染治理条件的复工复产企业，依法核准延期缴纳环境保护税。推动金融机构加大绿色信贷实施力度。

（八）实施差异化监管，精准服务复工复产

根据疫情防控形势和分区分级精准复工复产有关要求，采取差异化生态环境监管措施并实行动态调整，为统筹推进疫情防控和经济社会发展提供支撑保障。

三、突出“三个治污”，确保生态环境质量持续改善和环境安全

突出精准治污、科学治污、依法治污，聚焦解决突出问题，以打赢蓝天保卫战为重中之重，持续改善生态环境质量，保障生态环境安全。已经完成目标任务的，要巩固提升；尚未完成的要加强督导，确保完成。

（九）强化精准治污，提升污染治理的针对性

集中力量打赢蓝天保卫战，注重统筹细颗粒物（PM2.5）和臭氧（O3）协同防治、统筹秋冬季和春夏季、统筹重点地区和城市群地区，着力抓好清洁取暖散煤替代、钢铁等行业超低排放改造、“公转铁”等工程建设，加快推进工业窑炉、重点行业挥发性有机物（VOCs）治理，确保完成优良天数比例等约束性指标任务。

以长江、黄河流域为重点抓好碧水保卫战，加强水源保护区划定保护、城镇污水收集处理设施建设、黑臭水体治理。扎实推进净土保卫战，提升危险废物环境监管、处置利用和风险防范能力。做好医疗设备辐射安全监管。加快提升生物安全管理水平。

（十）实施科学治污，不断满足污染治理实际需求

加大大气重污染成因与治理攻关及成果应用，构建重污染天气应对技术体系，实现提前研究、提前告知、提前预警。深化细化大气污染源排放清单研究，强化污染治理和应急减排效果评估，持续推进“一市一策”驻点跟踪研究。

深入组织实施水专项和长江生态环境保护修复联合研究项目。开展疫情防控相关生态环保技术研发与应用，提升废物废水处理处置、应急监测等环境保护技术支撑水平。

运用好第二次污染源普查成果，建立完善风险预警模型，推行热点网格预警机制。

（十一）坚持依法治污，营造知法守法氛围

进一步完善水、大气污染物和固体废弃物排放标准和规范，充分听取企业和行业协会商会意见，征求意见过程向全社会公开。

加强合法性审核，政策标准的制定、实施要统筹考虑疫情防控和经济社会发展需要，为企业预留足够时间，提高可预期性。在出台标准规范的同时，发布指导企业达标排放的相关规范及指南。

将执法与普法、执法与服务相结合，大力开展“送法入企”活动，做好企业复产复工的环境法规宣贯。

四、扛起主体责任，不折不扣抓好落实

各级生态环境部门要认真落实党中央关于统筹推进新冠肺炎疫情防控和经济社会发展的决策部署，增强“四个意识”，坚定“四个自信”，做到“两个维护”，扛起主体责任，抓好贯彻落实，实现疫情防控、企业复工复产和生态环境保护共赢。

（十二）加强组织领导

各级生态环境部门主要负责同志是本部门疫情防控第一责任人，要亲自谋划、亲自部署、亲自推动。要加强党的领导，充分发挥各级党组织战斗堡垒和党员干部先锋模范作用，在实践中加快打造生态环境保护铁军。强化宣传报道，弘扬正能量，及时答疑解惑。

（十三）创新工作方法

加快推进生态环境系统互联网+政务系统建设。推行“不见面”环保审批，实行线上受理。通过信息化手段有效辅助日常办公。应用远程监控、卫星遥感等新技术，加强重点行业、重点企业和人民群众反映强烈的污染排放监管。

（十四）建立调度机制

实时了解各地主要工作举措和任务进展，及时协调解决遇到的困难和问题，总结推广各地好做法、好经验、好案例。

（十五）严明纪律作风

坚决克服形式主义、官僚主义、只说不做、工作不深入不落实等问题。将做好疫情防控、服务经济社会发展、加强生态环境保护作为考验各级单位和干部的考场，在斗争一线锤炼、考察、识别和使用干部，对不作为慢作为的失职行为严肃追责问责，对表现突出的予以表扬表彰。

附件：1.环境影响评价审批正面清单

2.监督执法正面清单

生态环境部

2020年3月3日

（156） 卫健委：印发新冠肺炎出院患者康复方案（试行）	国卫办医函〔2020〕189号

各省、自治区、直辖市及新疆生产建设兵团卫生健康委：

为改善新冠肺炎出院患者的呼吸功能、躯体功能、心理功能、日常生活活动能力及社会参与能力，规范康复操作技术及流程，进一步促进其全程康复，我们组织专家制定了《新冠肺炎出院患者康复方案（试行）》。现印发给你们，请参照执行。

国家卫生健康委办公厅

2020年3月4日

新冠肺炎出院患者康复方案

（试行）

为改善新冠肺炎患者呼吸功能、躯体功能以及心理功能障碍，规范康复的操作技术及流程，特制定本方案。

一、目标

改善新冠肺炎出院患者呼吸困难症状和功能障碍，减少并发症，缓解焦虑抑郁情绪，降低致残率，最大程度恢复日常生活活动能力、提高生活质量。

二、适用人群及场所

（一）人群。新冠肺炎出院患者。

（二）场所。指定的出院后患者康复医疗机构、隔离场所、养老院、社区、家庭。

三、主要内容

（一）需开展康复治疗的功能障碍。

呼吸功能障碍。表现为咳嗽、咳痰、呼吸困难、活动后气短，可伴有呼吸肌无力及肺功能受损等。

躯体功能障碍。表现为全身乏力、易疲劳、肌肉酸痛，部分可伴有肌肉萎缩、肌力下降等。

心理功能障碍。有恐惧、愤怒、焦虑、抑郁等情绪问题。

日常生活活动能力及社会参与能力障碍。无法独立完成穿脱衣、如厕、洗澡等。无法实现正常的人际交往和无法重返工作岗位。

（二）康复功能评估。

呼吸功能评估。采用呼吸困难指数量表（mMRC）等进行评估，有条件地区或机构建议行肺功能检查。

躯体功能评估。采用Borg自觉疲劳量表、徒手肌力检查等进行评估。

心理功能评估。采用抑郁自评量表（SDS）、焦虑自评量表（SAS）、匹兹堡睡眠问卷等进行评估。

日常生活活动能力评估。采用改良巴氏指数评定表等进行评估。

六分钟步行试验。要求患者在平直走廊里尽可能快的行走，测定六分钟的步行距离，最小折返距离≥30米。

（三）康复治疗方法。

1.呼吸功能训练

主动循环呼吸技术（ACBT）：一个循环周期由呼吸控制、胸廓扩张运动和用力呼气技术三个部分组成。呼吸控制阶段指导患者用放松的方法以正常的潮气量进行呼吸，鼓励肩部及上胸部保持放松，下胸部及腹部主动收缩，以膈肌呼吸模式完成呼吸，该阶段持续时间应与患者对放松的需求相适应。胸廓扩张阶段强调吸气，指导患者深吸气到吸气储备量，屏息1-2秒，然后被动而轻松的呼气。用力呼气阶段为穿插呼吸控制及呵气。呵气是一种快速但不用最大努力的呼气，过程中声门应保持开放。利用呵气技巧进行排痰，代替咳嗽降低呼吸肌做功。注意在呵气过程中用口罩遮挡。

呼吸模式训练：包括调整呼吸节奏（吸:呼=1:2）、腹式呼吸训练、缩唇呼吸训练等。

呼吸康复操：根据患者体力情况进行卧位、坐位及站立位的颈屈伸、扩胸、转身、旋腰、侧躯、蹲起、抬腿、开腿、踝泵等系列运动。

2.躯体功能训练

有氧运动：针对患者合并的基础疾病和遗留功能障碍问题制订有氧运动处方。包括踏步、慢走、快走、慢跑、游泳、太极拳、八段锦等运动形式。以运动后第二天不出现疲劳的运动强度为宜，从低强度开始，循序渐进，每次20-30分钟，每周3-5次。对于容易疲劳的患者可采取间歇运动形式进行。餐后1小时后开始。

力量训练：使用沙袋、哑铃、弹力带或瓶装水等进行渐进抗阻训练，每组15-20个动作，每天1-2组，每周3-5天。

3.心理康复干预

设计可产生愉悦效应及转移注意力的作业疗法，达成调整情绪，疏解压力的目的。通过专业心理学培训的护理人员和康复治疗师也可以开展专业的心理咨询，包括正念放松治疗和认知行为治疗。注意慎用让患者重复叙述创伤经历的方法，以免造成重复伤害。如出现精神障碍，建议精神专科介入。

4.日常生活活动能力训练

对患者进行日常生活活动指导。主要是节能技术指导，将穿脱衣、如厕、洗澡等日常生活活动动作分解成小节间歇进行，随着体力恢复再连贯完成，逐步恢复至正常。

四、有关注意事项

（一）禁忌证。如患者出现以下情况之一，不建议开展上述康复治疗。

1.静态心率＞100次/分。

2.血压＜90/60mmHg、＞140/90 mmHg或血压波动超过基线20mmHg，并伴有明显头晕、头痛等不适症状。

3.血氧饱和度≤95%。

4.合并其他不适合运动的疾病。

（二）当患者在治疗过程中出现以下情况，应立即停止上述康复治疗，重新评估并调整治疗方案。

1.出现明显疲劳，休息后不能缓解。

2.出现胸闷、胸痛、呼吸困难、剧烈咳嗽、头晕、头痛、视物不清、心悸、大汗、站立不稳等。

（三）当患者合并有肺动脉高压、充血性心力衰竭、深静脉血栓、不稳定的骨折等疾病则应与专科医生咨询相关注意事项后再开始呼吸康复治疗。

（四）高龄患者常伴有多种基础疾病，体质较差，对康复训练的耐受能力较差，康复治疗前应进行综合评估，康复训练应从小剂量开始，循序渐进，避免出现训练损伤及其他严重并发症。

（五）重型、危重型患者出院后，视当地康复医疗工作实际，可在指定的康复医疗机构、基层医疗卫生机构进行出院后康复。轻型、普通型患者出院后，社区及居家应适当休息、适当运动，尽最大可能恢复体能、体质和免疫能力。

（157） 卫建委等2部门：印发新冠肺炎康复者恢复期血浆临床治疗方案（试行第二版）	国卫办医函〔2020〕185号

各省、自治区、直辖市及新疆生产建设兵团卫生健康委，军队各大单位：

为进一步做好新冠肺炎康复者恢复期血浆临床治疗工作，提高治愈率，我们组织专家在总结、分析前期治疗工作的基础上，对治疗方案进行修订，形成了《新冠肺炎康复者恢复期血浆临床治疗方案（试行第二版）》。现印发给你们，请参照执行。各有关单位要认真落实工作要求，保障捐献血浆者和患者安全，科学合理输注，将康复者血浆纳入综合治疗的内容，充分发挥其中和病毒的有效作用，配合其他治疗措施，确保临床治疗取得良好效果。

附件：新冠肺炎康复者恢复期血浆临床治疗方案（试行第二版）

国家卫生健康委办公厅  中央军委后勤保障部卫生局

2020年3月4日

（158） 北京：做好新型冠状病毒肺炎疫情防控期间稳定劳动关系支持企业复工复产	京人社劳字[2020]23号

各区人力资源和社会保障局、总工会、企业联合会/企业家协会、工商联：

　　为深入贯彻落实习近平总书记在统筹推进新冠肺炎疫情防控和经济社会发展工作部署会议的重要讲话精神、在北京市调研指导新型冠状病毒肺炎疫情防控工作时的重要讲话精神和一系列重要指示精神，按照党中央、国务院关于新型冠状病毒肺炎疫情防控工作决策部署和市委、市政府工作要求，积极发挥广大企业和职工在疫情防控中的重要作用，全力支持企业复工复产稳定劳动关系，动员广大职工凝心聚力共克时艰，根据人力资源社会保障部、全国总工会、中国企业联合会/企业家协会、全国工商联《关于做好新型冠状病毒感染肺炎疫情防控期间稳定劳动关系支持企业复工复产的意见》(人社部发〔2020〕8号)，现就我市做好疫情防控期间稳定劳动关系支持企业复工复产提出以下实施意见：

　　一、高度重视疫情对劳动关系领域带来的新挑战

　　近期，受新型冠状病毒肺炎疫情影响，劳动关系领域面临新情况新问题。部分行业企业面临较大的生产经营压力，劳动者面临待岗、失业、收入减少等风险，劳动关系不稳定性增加，劳动关系矛盾逐步凸显。当前，新型冠状病毒肺炎疫情防控正处于关键阶段，全市各级协调劳动关系三方要认真学习习近平总书记关于疫情防控工作的一系列重要指示精神，坚决贯彻落实党中央、国务院决策部署和市委、市政府的工作要求，高度重视当前特殊时期劳动关系运行中出现的突出问题，加强劳动关系风险监测和研判，引导企业与职工共担责任共渡难关。要充分发挥三方机制在保企业、保就业、保稳定中的独特作用，深入分析当前劳动关系形势，结合实际帮助企业制定复工复产的措施，联合各方力量共同行动，加大对特殊时期企业劳动关系处理的指导服务，防止出现企业大规模裁员，确保劳动关系总体和谐稳定。

　　二、灵活处理疫情防控期间的劳动用工问题

　　(一)鼓励协商解决复工前的用工问题。对因受疫情影响职工不能按期到岗或企业不能开工生产的，要指导企业主动与职工沟通，有条件的企业可安排职工通过电话、网络等灵活工作方式在家上班完成工作任务；对不具备远程办公条件的企业，可与职工协商优先使用带薪年休假、企业自设福利假等各类假。要指导企业工会积极动员职工与企业同舟共济，在兼顾企业和劳动者双方合法权益的基础上，帮助企业尽可能减少受疫情影响带来的损失。

　　(二)鼓励灵活安排工作时间。在疫情防控期间，为减少人员聚集，要鼓励符合规定的复工企业实施灵活用工措施，与职工协商采取错时上下班、弹性上下班等方式灵活安排工作时间。鼓励通过协商等方式，安排孕期女职工从事符合条件的工作岗位，允许哺乳期女职工自行选择集中使用哺乳时间。对承担政府疫情防控保障任务需要紧急加班的企业，在保障劳动者身体健康和劳动安全的前提下，指导企业与工会和职工协商，可适当延长工作时间应对紧急生产任务，依法不受延长工作时间的限制。

　　(三)指导规范用工管理。在疫情防控期间，要指导企业全面了解职工被实施隔离措施或政府采取的紧急措施情况，要求企业不得在此期间解除受相关措施影响不能提供正常劳动职工的劳动合同或退回被派遣劳动者。对符合规定的复工企业，要指导企业提供必要的防疫保护和劳动保护措施，积极动员职工返岗。对不愿复工的职工，要指导企业工会及时宣讲疫情防控政策要求和企业复工的重要性，主动劝导职工及时返岗。对经劝导无效或以其他非正当理由拒绝返岗的，指导企业依法予以处理。鼓励企业积极探索稳定劳动关系的途径和方法，对采取相应措施后仍需要裁员的企业，要指导企业制定裁员方案，依法履行相关程序，妥善处理劳动关系，维护企业正常生产经营秩序。

　　三、协商处理疫情防控期间的工资待遇问题

　　(四)支持协商未返岗期间的工资待遇。在受疫情影响的延迟复工或未返岗期间，对用完各类休假仍不能提供正常劳动或其他不能提供正常劳动的职工，指导企业参照国家和本市关于停工、停产期间工资支付相关规定与职工协商，在一个工资支付周期内的，按照劳动合同约定的标准支付工资；超过一个工资支付周期的，按照本市有关规定支付职工基本生活费。

　　(五)支持困难企业协商工资待遇。对受疫情影响导致企业生产经营困难的，鼓励企业通过协商民主程序与职工协商采取调整薪酬、轮岗轮休、缩短工时等方式稳定工作岗位；对暂无工资支付能力的，要引导企业与工会或职工代表协商延期支付，各级工会组织要积极给予协助，帮助企业减轻资金周转压力。

　　(六)保障职工工资待遇权益。对因依法被隔离导致不能提供正常劳动的职工，要指导企业按正常劳动支付其工资；隔离期结束后，对仍需停止工作进行治疗的职工，按医疗期有关规定支付工资。对在春节假期延长假期间因疫情防控不能休假的职工，指导企业应先安排补休，对不能安排补休的，依法支付加班工资。

　　四、采取多种措施减轻企业负担

　　(七)帮助企业减少招聘成本。要加大线上招聘服务工作力度，打造线上春风行动，大力推广远程面试，提高招聘企业与劳动者“点对点”直接对接率。鼓励经营性人力资源服务机构参与公共就业服务。为重点企业提供职业介绍服务的经营性人力资源服务机构，在疫情防控期间给予一次性就业创业服务补助。补助标准为:推荐成功上岗人数为50人(含)以上的，补助2.4万元；100人(含)以上的，补助4.5万元；200人(含)以上的，补助9万元。规范人力资源服务收费，坚决打击恶意哄抬劳动力价格行为。对受疫情影响缺工较大的企业或者承担政府保障任务企业，鼓励人力资源服务机构减免费用提供招聘服务。

　　(八)合理分担企业稳岗成本。用好失业保险援企稳岗返还政策，对受疫情影响较大，面临暂时性生产经营困难且恢复有望、坚持不裁员或少裁员的中小微参保企业，可按6个月的上年度本市月人均失业保险金标准和参保职工人数，返还上年度失业保险费。用好国家关于阶段性减免企业基本养老保险、失业保险、工伤保险费政策。用好小微企业工会经费支持政策，对受疫情影响符合条件的小微企业工会经费全额返还。用好企业组织会费，对受疫情影响符合条件的困难企业实行一定比例的企业会费返还。用好工会防疫专项资金，加大对防疫一线职工的慰问，充分调动职工参与防控疫情的积极性。

　　(九)提供在线免费培训。指导企业积极组织开展职工在线免费培训，支持帮助受疫情影响企业特别是中小微企业开展职工技能培训。在疫情防控期间公布一批免费向公众开放的在线培训机构目录清单，为企业职工提供方便、快捷的在线技能培训课程，提升职工技能素质。

　　五、统筹各方力量加大指导服务力度

　　(十)加强劳动用工指导服务。各级人力资源社会保障部门要及时研究和解决疫情防控期间劳动关系领域中的重大问题，主动回应社会关切，制定有针对性政策，准确解读我市援企稳岗的各项政策，帮助企业解决发展中的困难。要做好协调劳动关系三方牵头工作，加强政策宣传和组织协调，发挥各方优势，形成工作合力，指导企业通过与工会和广大职工协商薪酬、采用灵活用工、综合调剂使用年度内休息日、支持企业间通过“共享用工”的方式解决暂时性用工缺口问题，保障企业正常运转、稳定就业岗位，尽量不裁员或少裁员。各级工会要教育引导职工积极参与疫情防控工作，加强个人防护，向职工宣传解读劳动用工、工资支付等相关劳动法律法规，引导职工依法理性表达诉求，积极做好职工心理疏导工作，为困难职工提供必要的帮扶救助。要团结、动员广大职工大力发扬劳动精神、劳模精神、工匠精神，为企业生产经营献计献策，保障企业正常运行。企业工会要主动发挥作用，与企业通过协商解决因疫情带来的复工复产期间劳动关系问题，促进企业健康发展。各级企联和工商联组织要指导企业在当地党委和政府的统一安排下有序组织复工复产，梳理评估企业的实际困难并积极向有关部门提出针对性帮扶支持政策建议和指导服务，要鼓励企业承担社会责任，通过技术创新等提高竞争力。要引导受疫情影响导致生产经营困难的企业，完善企业内部协商民主机制，畅通与职工对话渠道，通过多种方式稳定劳动关系和工作岗位。要引导企业关心关爱职工健康，帮助解决职工实际困难，切实保障职工权益。引导企业用好政府相关租金补贴政策，减轻企业经营负担，引导同行业或上下游企业互帮互助，抱团取暖。

　　(十一)主动化解劳动关系矛盾。加强疫情防控期间劳动关系监测和形势分析研判，有效应对和妥善处置劳动关系领域出现的苗头性、倾向性问题，力争把风险隐患化解在萌芽状态，着力提升基层预防化解调处劳动争议能力，推动企业建立健全内部劳动争议协商解决机制。发挥多元化解机制作用，充分运用互联网、微信视频等多种方式开展调解仲裁工作，强化专业性劳动争议调解，加强案件处理指导，依法公正处理争议案件。进一步畅通举报投诉渠道，加大劳动保障监察执法力度，依法查处违法行为。

　　(十二)做好表彰先进典型工作。各级协调劳动关系三方要深入开展和谐劳动关系创建活动，主动宣传在防控疫情中真正实现有事好商量、遇事多商量、有难题共同解决的企业，要在和谐劳动关系创建活动评比达标、劳动模范评选、五一劳动奖章、奖状、优秀企业家评选等荣誉授予中优先考虑疫情防控期间对稳定劳动关系做出突出贡献的企业和个人，激励引导广大企业家和职工在疫情防控工作中主动履职，担当作为。

　　全市各级协调劳动关系三方要切实增强“四个意识”，坚定“四个自信”，做到“两个维护”，深入贯彻落实党中央、国务院关于应对新型冠状病毒肺炎疫情工作的决策部署和市委市政府的工作要求，把稳定劳动关系支持企业与职工共渡难关作为当前重要工作来抓，统筹处理好促进企业发展和维护职工权益的关系，充分发挥中国特色和谐劳动关系的制度优势，坚定信心、积极作为，为打赢疫情防控的人民战争、总体战、阻击战做出积极贡献。

北京市人力资源和社会保障局

北京市总工会

北京企业联合会/北京市企业家协会

北京市工商业联合会

2020年2月19日

（159） 中央应对新型冠状病毒感染肺炎疫情工作领导小组：全面落实疫情防控一线城乡社区工作者关心关爱措施	国发明电〔2020〕8号

各省、自治区、直辖市党委和人民政府，新疆生产建设兵团，中央和国家机关有关部门：

新冠肺炎疫情发生以来，广大城乡社区工作者贯彻落实党中央、国务院决策部署，坚守岗位、日夜值守，英勇奋战在疫情防控一线，为遏制疫情扩散蔓延作出了重要贡献。习近平总书记多次作出重要指示，强调要关心关爱城乡社区工作者。李克强总理作出批示，要求对社区工作者防控工作给予保障。为激励广大城乡社区工作者不畏艰险、勇往直前，坚决打赢疫情防控人民战争、总体战、阻击战，现提出以下措施：

一、对城乡社区工作者适当发放工作补助。各地要采取措施，关心关爱参加疫情防控的社区（村）“两委”成员、社区（村）专职工作人员等一线城乡社区工作者。在落实好城乡社区工作者现有报酬保障政策基础上，在疫情防控期间地方可对一线城乡社区工作者给予适当工作补助，补助标准由省级人民政府根据疫情防控任务指导市县制定，所需资金由地方财政承担。

二、切实做好城乡社区工作者职业伤害保障。城乡社区工作者在疫情防控期间发生事故伤害或患病，符合《工伤保险条例》认定情形的，应依法认定为工伤，已经参加工伤保险的，发生的相关费用按照《工伤保险条例》相关规定支付；未参加工伤保险的，由用人单位按照法定标准支付，财政补助单位因此发生的费用，由同级财政予以补助。对于因履行职责感染新冠肺炎或病亡的城乡社区工作者，符合《人力资源社会保障部 财政部 国家卫生健康委关于因履行工作职责感染新型冠状病毒肺炎的医护及相关工作人员有关保障问题的通知》（人社部函〔2020〕11号）规定的，可按规定享受相关待遇。

三、改善城乡社区工作者防护条件。各地要按规定落实社区防控工作经费，改善一线城乡社区工作者防护条件，落实属地原则强化社区防控工作物资保障，根据当地疫情防控需要，合理配发口罩、防护服、消毒水、非接触式体温计等卫生防护器材和防控器具。组织开展面向城乡社区工作者的医疗卫生知识、疫情防控知识培训，提高其做好社区防控工作的能力。

四、切实为城乡社区工作者减负减压。严肃查处疫情防控中的形式主义、官僚主义问题，坚决制止多头重复向基层派任务、要表格，除党中央、国务院已有明确要求的外，原则上不得向社区摊派工作任务，确因疫情防控工作特殊需要的，须经省级人民政府批准。除因社区疫情防控需要出具的居住证明和居家医学观察期满证明外，不得以疫情防控为由要求城乡社区组织出具其他证明。进一步充实城乡社区疫情防控力量，抽调机关和企事业单位党员干部下沉支援社区疫情防控一线，组织和发挥好社区服务机构、志愿者作用。加快构建社区防控工作信息化支撑体系，运用现代信息技术提高社区防控工作效能，减轻城乡社区工作者工作压力。

五、保障城乡社区工作者身心健康。各地要协调安排好疫情防控期间一线城乡社区工作者就餐、休息场所等生活保障。在保证社区防控人员力量的前提下，合理调度疫情防控一线城乡社区工作者，采取轮休、补休等方式，保证城乡社区工作者及时得到必要休整。对长时间高负荷工作人员安排强制休息，防止带病上岗。疫情结束后，要及时安排健康体检和心理减压；有条件的地方，可安排休假疗养。

六、加强城乡社区工作者关爱慰问。做好疫情防控一线城乡社区工作者特别是感染新冠肺炎、因公受伤、突发疾病城乡社区工作者和因公死亡城乡社区工作者的家属的走访慰问工作，最大限度帮助其解决实际困难。对奋战一线、无法照顾家庭的城乡社区工作者，要组织社区服务机构和志愿者对其家属给予更多帮助关爱。

七、开展城乡社区工作者表彰褒扬。注重在疫情防控一线考察识别城乡社区工作者，对表现突出的要大力褒奖、大胆使用，在招录（聘）公务员或事业单位工作人员、选拔街道（乡镇）干部时在同等条件下优先考虑。根据国家有关规定，对涌现出的先进典型进行表彰。对疫情防控期间因公殉职的城乡社区工作者追授“全国优秀城乡社区工作者”。依照有关法规做好因疫情防控牺牲殉职城乡社区工作者的烈士评定和褒扬工作，全面做好抚恤优待。获得表彰以及被认定为烈士的城乡社区工作者的子女，在入学升学方面按规定享受相关待遇。

八、加大城乡社区工作者先进典型宣传力度。充分发挥报刊、广播、电视等新闻媒体和网络新媒体作用，大力宣传报道在社区防控工作中表现突出的城乡社区工作者的感人事迹，营造全社会关心、支持、参与城乡社区疫情防控的正确舆论导向和良好社会氛围。疫情结束后，依托“最美城乡社区工作者”宣传推选活动，发现、选树和宣传一批城乡社区工作者先进典型。

各地、各部门要按照党中央、国务院决策部署，坚决落实好上述政策措施，以高度负责的态度、务实有效的举措，切实关心关爱城乡社区工作者，为守严守牢疫情联防联控、群防群控的社区防线提供有力保障。地方各级疫情防控工作领导小组要加强调度和督查，确保各项关心关爱政策措施落实到位；民政部门要牵头协调落实好关心关爱一线城乡社区工作者的政策措施，组织、宣传、发展改革、工业和信息化、财政、人力资源社会保障、卫生健康、退役军人等部门要按照职责做好相关政策措施和资金保障落实工作。

中央应对新型冠状病毒感染肺炎

疫情工作领导小组

2020年3月3日

（160） 民政部等4部门：印发《新冠肺炎疫情社区防控工作信息化建设和应用指引》	民办发〔2020〕5号

各省、自治区、直辖市民政厅（局）、网络安全和信息化委员会办公室、工业和信息化主管部门、卫生健康委，各计划单列市民政局、网络安全和信息化委员会办公室、工业和信息化主管部门、卫生健康委，新疆生产建设兵团民政局、网络安全和信息化委员会办公室、工业和信息化主管部门、卫生健康委：

为贯彻落实习近平总书记关于统筹推进疫情防控和经济社会发展工作的重要讲话精神和党中央、国务院决策部署，切实发挥信息化助力社区防控工作的积极作用，坚决守住社区防控这道疫情防控的有效防线，我们编制了《社区防控工作信息化建设和应用指引》（第一版，以下简称《指引》），同时根据《指引》有关要求，指导相关行业协会等社会组织推荐部分社区防控信息化产品（服务），各地可结合本地社区防控工作实际自愿选择使用（具体清单另行发布）。

各地应用《指引》的有关情况和案例，请及时报民政部、中央网信办、工业和信息化部、国家卫生健康委。

附件：新冠肺炎疫情社区防控工作信息化建设和应用指引（第一版）

民政部办公厅

中央网信办秘书局

工业和信息化部办公厅

国家卫生健康委办公厅

2020年3月2日

附件

新冠肺炎疫情社区防控工作信息化建设和应用指引

（第一版）

为贯彻落实习近平总书记关于统筹推进疫情防控和经济社会发展工作的重要讲话精神和党中央、国务院决策部署，促进社区防控工作与现代信息技术深度融合，强化外防输入、内防扩散的技术支撑，提高社区防控的信息化、智能化水平，发挥社区防控在疫情防控中的阻击作用，现就社区防控信息化建设和应用提供如下指引：

一、建设和应用目标

按照疫情防控总体部署和社区防控工作要求，坚持适用性、便捷性、安全性和前瞻性相统一，发挥互联网、大数据、人工智能等信息技术优势，依托各类现有信息平台特别是社区信息平台，开发适用于社区防控工作全流程和各环节的功能应用，有效支撑社区疫情监测、信息报送、宣传教育、环境整治、困难帮扶等防控任务，统筹发挥城乡社区组织、社区工作者的动员优势和信息化、智能化手段的技术优势，有效支撑省、市、县、乡四级数据联通，构筑起人防、物防、技防、智防相结合的社区防线，形成立体式社区防控数据链路和闭环，提升城乡社区疫情防控工作成效。

二、建设和应用内容

（一）疫情监测和重点人群管理。

1.信息摸排。充分利用现有信息技术手段，采用云服务+大数据+网格化方式，构建社区防控数据平台，生成社区防控小程序和二维码，社区二维码应包含基层群众性自治组织统一社会信用代码信息。支持社区居民通过在线注册或扫描社区二维码方式，完成基础信息和健康信息登记，并生成社区居民二维码（或应用依托国家统一身份认证系统、统一电子证照系统生成的防疫健康信息码，下同），实现一次登记、多端共享。支持社区工作人员通过扫描社区居民二维码方式核验或补录社区居民基础信息和健康信息。社区居民基础信息和健康信息填报模板应符合标准化要求，对孕产妇等重点人群信息进行标注，并可根据各地情况进行统一参数化设置；有条件的社区，应与基础电信企业提供的“到访地查询服务”有效衔接，并实现社区内房屋和住户的精准关联。

2.病例监测。支持根据相关要求实施居家医学观察的社区居民在线填报每日健康信息、症状监测信息，与基层医疗卫生机构工作人员或家庭医生开展在线互动，获得健康指导或就医指南。对接新冠肺炎患者救治医院和基层医疗卫生机构，随访监测出院患者健康状况。有条件的社区，可通过查询公共安全视频监控联网信息，协助确定其是否落实居家医学隔离要求。为结束居家医学观察的社区居民在线生成证明。

3.体温检测。有条件的社区，可设置红外体温检测设备，自动对社区出入人员进行体温检测，并对超过设定值人员进行提示。

（二）出入管理。

1.社区居民出入管理。支持本社区居民通过扫描二维码进入社区（楼栋），同时实现社区居民出入记录登记；对于实施出入次数管理的社区，可对超过设定值的人员进行提示。有条件的社区，在社区居民授权同意的情况下，可通过社区物联设备智能互联提供人脸识别通行服务。

2.社区外来人员出入管理。加快推动相关部门数据共享，引导互联网企业共享疫情相关数据。对于实现社区居民基础信息和健康信息在街道（乡镇）及以上层面共享互认的，支持外来人员通过扫描二维码并经社区工作人员确认后进入社区（楼栋）。对于尚未实现信息共享的，支持本社区居民线上提交外来人员信息，经社区工作人员确认后生成外来人员二维码，外来人员通过扫描二维码进入社区，并自动登记外来人员出入信息。

3.车辆出入管理。支持通过车辆牌照自动识别本社区居民登记车辆，并经本社区居民在线提交和社区工作人员确认，实现外来车辆进出社区。有条件的社区，可增加停车指引和车库监控。

（三）信息报送。

1.社区防控工作人员管理。建设社区防控人员工作平台，生成社区防控小程序管理端。支持依托互动功能提供技术支持、依托视频会议功能实现实时工作指导。

2.社区防控信息管理。根据联防联控工作需要，通过对接相关政府部门疫情防控信息平台、政务信息平台，建立分层分级访客登记分析、任务分发、信息报送组织体系。支持根据社区居民自主填报、社区工作人员主动采集的信息自动汇总数据、生成并报送社区疫情信息报表。

3.社区防控信息应用。运用大数据对社区防控情况进行分析。有条件的地方，可依托GIS技术实现社区疫情信息可视化，展示社区网格化防控工作动态图、重点区域防控预警图、确诊和疑似病例行为轨迹地图等。有条件的地方，可推动社区防控、企业防控信息共享互认。

（四）宣传教育。

建设社区居民互动平台。通过对接政府相关部门疫情发布平台，宣传党中央、国务院应对疫情的重要工作部署和政策，发布和更新当地疫情防控的政策措施，实现疫情信息动态更新。支持社区工作者发布社区疫情防控信息和防控工作通知，介绍疫情防控知识、医疗卫生科普知识、居家隔离保护措施、个人预防基本知识，及时发布社区周边疫情动态，实现实时通知状态反馈。支持社区居民线上参与社区防控工作重大事项协商。

（五）环境整治。

支持社区居民线上提供问题线索，对接政府相关部门政务信息平台，加强对社区公共道路、公共空间、公共设施和公用器材日常消毒情况和集贸（农贸）市场、便民摊点群等环境整治情况的监督。

（六）困难帮扶。

建设社区综合服务平台。开展“互联网+医疗健康”服务，为患有慢性疾病或其他有需要的社区居民提供在线健康教育，有条件的地方，可探索运用新一代人工智能技术提供健康监测和咨询服务。为接受居家医学观察或其他有需要的社区居民提供在线心理健康服务。为社区老年人，包括春节期间回家的养老院入住老人提供健康监测和紧急呼叫服务；为社区儿童提供远程监护和在线教育服务。

（七）社区服务。

1.社区公共服务。有条件的社区，可加强与社区公共服务综合信息平台衔接，为社区党员提供在线政策宣传和学习服务。丰富社区居民网络文化生活。为社区居民提供在线缴纳公共事业费用等“无接触”服务。

2.社区志愿服务。有条件的社区，可加强与全国志愿服务信息系统衔接，实行社区志愿者在线招募和管理，完善志愿者基本信息和服务信息数据；实行社区志愿服务项目在线发布，促进社区志愿服务供给和需求精准对接。

3.社区生活服务。有条件的社区，在线对接大型连锁企业和社区周边小型企业资源，为社区居民提供日常生活用品在线购买和配送服务，满足居民日常生活需求。设有快递、外卖配送区域的社区，可提供智能快递柜、外卖送达在线提示、自主取货等服务方式，减少人员流动、聚集。

三、相关支撑环境

（一）部署条件。社区防控信息化产品（服务）原则上应在区（县、市、旗）及以上层面集中部署应用，确有需要的也可在街道（乡镇）和城乡社区层面部署，优先使用已有的政务或公共服务平台，同时做好与卫生健康、公安、民政等部门相关疫情防控信息平台、公民身份信息认证平台、社区治理信息管理系统等功能衔接。鼓励疫情防控结束后，通过二次开发、系统接入等方式，支持拓展城乡社区治理和服务功能。

（二）系统安全。社区防控信息化产品（服务）应当依照法律、行政法规的规定和国家标准的强制性要求，采取技术措施和其他必要措施，保障网络安全、稳定运行，有效应对网络安全事件；具有数据加密、脱敏和防爬取能力，保障数据安全。社区防控信息化产品（服务）提供者应参照等级保护三级以上要求进行安全防护，应当为其产品（服务）持续提供安全维护，在规定或与产品（服务）使用者约定的期限内，不得终止提供安全维护。

（三）隐私保护。社区防控信息化产品（服务）因疫情防控工作要求，需要收集社区居民信息的，应向社区居民明确提示并取得同意，明确用于此次疫情防控，对于用于其他目的的，必须重新征得社区居民本人同意；同时遵守《中华人民共和国网络安全法》、《中华人民共和国居民身份证法》和有关法律、行政法规关于个人信息保护的规定，落实中央网信办《关于做好个人信息保护利用大数据支撑联防联控工作的通知》要求。

（四）公益原则。社区防控信息化产品（服务）的开发、应用、维护应坚持公益属性，鼓励疫情防控期间不向使用者收取费用。列入社区防控信息化产品（服务）推荐清单的，产品（服务）提供者还需签署《承诺书》。社区防控信息化产品（服务）中涉及非公益性服务的，应向使用者明确提示。

四、有关依据

1.国务院应对新冠肺炎疫情联防联控机制《关于加强新型冠状病毒感染的肺炎疫情社区防控工作的通知》（肺炎机制发〔2020〕5号）。

2.国务院应对新冠肺炎疫情联防联控机制《关于做好儿童和孕产妇新型冠状病毒感染的肺炎疫情防控工作的通知》（肺炎机制发〔2020〕17号）。

3.中央网信办《关于做好个人信息保护 利用大数据支撑联防联控工作的通知》（中网办发电〔2020〕5号）。

4.民政部、国家卫生健康委《关于进一步动员城乡社区组织做好新型冠状病毒感染的肺炎疫情防控工作的紧急通知》（民发〔2020〕9号）。

5.民政部、国家卫生健康委《关于深入学习贯彻习近平总书记重要指示精神 进一步做好城乡社区疫情防控工作的通知》（民发〔2020〕13号）。

6.民政部办公厅《关于做好基层群众性自治组织特别法人统一社会信用代码赋码工作的通知》（民办函〔2017〕285号）。

7.国家卫生健康委办公厅《关于加强信息化支撑新型冠状病毒感染的肺炎疫情防控工作的通知》（国卫办规划函〔2020〕100号）。

8.国家卫生健康委办公厅《关于做好新型冠状病毒肺炎出院患者跟踪随访工作的通知》（国卫办医函〔2020〕142号）。

（161） 民政部：分区分级精准做好养老服务机构疫情防控与恢复服务秩序工作	民办发〔2020〕6号

各省、自治区、直辖市民政厅（局），各计划单列市民政局，新疆生产建设兵团民政局：

为贯彻落实习近平总书记在统筹推进新冠肺炎疫情防控和经济社会发展工作部署会议上的重要讲话精神，落实国务院关于科学防治精准施策分区分级做好新冠肺炎疫情防控工作以及进一步做好民政服务机构疫情防控工作的部署要求，现就新冠肺炎疫情期间分区分级精准做好养老服务机构疫情防控与恢复服务秩序工作提出如下意见：

一、总体要求

坚持分区、分级、分类、稳妥、有序原则，在当地党委和政府领导下，将养老服务机构疫情防控纳入当地疫情防控联防联控机制，在确保疫情防控到位的前提下，稳妥推进疫情防控期间养老服务机构恢复服务工作，确保老年人生命健康安全，更好服务老年人需求。各地民政部门要根据各省（区、市）人民政府确定的风险地区名单，以县（市、区、旗）为单位，按要求落实相关防控措施。

二、压实疫情防控工作责任

各地民政部门要切实履行主管职责，把疫情防控措施抓实抓细抓落地，不搞“一刀切”，重点防控输入性疫情和内部疾病传播，坚决切断传染源，阻断传播途径。

养老服务机构要承担疫情防控主体责任，严格落实疫情防控各项措施和工作要求，制定符合自身特点的实施细则和应急预案，明确每个部门、岗位和服务环节的职责任务，发现异常情况，及时启动预案，做好处置工作。

三、分区分级精准防控，稳妥有序开放服务

全国无确诊病例县（区）的养老机构，坚持“预防为主、外防输入”策略，经当地党委和政府同意后，在确保疫情防控到位的前提下，可有序恢复开放。对15天内未到过中高风险区域、未密切接触来自中高风险区域人员且无相关疑似症状的本区域老年人和工作人员，可按照当地新冠肺炎疫情联防联控机制要求办理返院和新入住手续。可招聘本区域15天内未到过中高风险区域、未密切接触来自中高风险区域人员且身体健康的工作人员。上述人员可不再要求14天的医学隔离观察。对15天内到过中高风险区域或密切接触过来自中高风险区域人员的本区域老年人，招聘来自中高风险区域的工作人员或密切接触过来自中高风险区域人员的本区域工作人员，需要在符合标准的集中隔离点进行不少于14天的医学隔离观察，经专业医疗卫生机构诊断无新冠病毒感染的，可入住或上岗。社区养老服务机构可在确保疫情防控到位的前提下，向周边老年人提供非聚集性居家和社区养老服务。

其他低风险区域和中风险区域的养老机构，坚持“外防输入、内防扩散”的策略，按照当地疫情联防联控机制统一部署，在严格执行民政部印发的《养老机构新型冠状病毒感染的肺炎疫情防控指南（第二版）》前提下，可逐步恢复开放。对本区域拟返院和新入住的老年人，以及返岗和拟招聘的机构工作人员，如无相关疑似症状且15天内未到过高风险区域、未密切接触来自高风险区域人员，无《养老机构新型冠状病毒感染的肺炎疫情防控指南（第二版）》禁止进入的情形，经医学隔离观察14天，并经专业医疗卫生机构诊断无新冠病毒感染后，可收住入院或上岗工作。优先满足本区域生活不能自理且有集中供养意愿的特困老年人，因子女参加抗击疫情、因疫情接受隔离治疗等原因导致家庭无力照顾、确需返院的和新入住的生活不能自理老年人机构养老服务需求。社区养老服务机构原则上继续暂停集中式服务和聚集性活动。

高风险区域的养老机构和出现疫情的养老机构，要按照“严格管控、外防输出、内防扩散”策略，落实当地疫情联防联控工作部署，严格执行民政部印发的《新冠肺炎疫情高风险地区及被感染养老机构防控指南》，继续实施严格封闭管理。对本区域生活不能自理且有集中供养意愿的特困老年人，因子女参加抗击疫情、因疫情接受隔离治疗等原因导致家庭无力照顾、确需返院和拟新入住的生活不能自理老年人，以及因机构管理服务需要确需返院或新招聘的工作人员，要严格按照《新冠肺炎疫情高风险地区及被感染养老机构防控指南》要求，进行14天医学隔离观察，经专业医疗卫生机构诊断无新冠病毒感染的可收住入院或上岗工作。

各地要在疾控机构指导下切实做好新入机构人员的医学隔离工作，可结合实际设置医学隔离观察点。具备条件的养老机构，可内部设立隔离观察点。针对不少养老机构不具备内部隔离条件的现实困难，各地可以市或县域为单位，选择一个当前入住老年人少、具备医学隔离条件的养老机构，作为拟返院和新入住老年人、拟返院和新招聘员工的集中隔离观察点。隔离观察点设置，必须符合老年人的特殊需求，按照当地疫情联防联控机制有关标准与要求，进行管理与服务。也可利用当地已有的集中隔离场所，或专门设立集中隔离场所。

四、采取有效措施支持养老服务机构渡过难关

地方各级民政部门要提早着手研究分析疫情对本区域养老服务发展的影响，主动向当地党委和政府汇报，将养老服务机构特别是社会服务机构类型的养老服务机构纳入扶持范围，推动落实好阶段性减免企业社保费、医保费和缓缴住房公积金，临时降低用气用水用电价格等国家及本地区出台的优惠政策。鼓励各地结合实际情况出台养老服务场地租金减免和临时补贴政策。有条件的地方可联合相关部门做好资金调度提前拨付补助资金，采取阶段性减免公建民营养老服务机构管理费、提高民办养老服务机构运营补贴标准，提高政府购买养老服务补贴标准，给予养老护理员临时岗位补贴等扶持措施，帮助养老服务机构渡过难关。

附件：1.养老服务机构分区分级精准防控工作要点

2.新冠肺炎疫情期间养老服务机构扶持政策要点

民政部办公厅

2020年3月4日

附件1

养老服务机构分区分级精准防控工作要点

一、全国无确诊病例县（市、区、旗）的养老服务机构

1.对15天内未到过中高风险区域、未密切接触来自中高风险区域人员且无相关疑似症状的本区域老年人和工作人员，可以按照当地新冠肺炎疫情联防联控机制要求办理返院和新入住手续，不再要求14天的医学隔离观察。

2.可以招聘本区域15天内未到过中高风险区域、未密切接触来自中高风险区域人员且身体健康的工作人员，不再要求14天的医学隔离观察。

3.对15天内到过中高风险区域或密切接触过来自中高风险区域人员的本区域老年人，招聘来自中高风险区域的工作人员或密切接触过来自中高风险区域人员的本区域工作人员，需要在符合标准的集中隔离点进行不少于14天的医学隔离观察，并经专业医疗卫生机构诊断无新冠病毒感染的，方可入住或上岗。

4.社区养老服务机构在确保疫情防控到位的前提下，可以向周边老年人提供非聚集性居家和社区养老服务。

二、其他低风险区域和中风险区域的养老服务机构

5.按照当地疫情联防联控机制统一部署，在严格执行民政部印发的《养老机构新型冠状病毒感染的肺炎疫情防控指南（第二版）》前提下，可以逐步恢复开放。

6.对本区域拟返院和新入住的老年人，以及返岗和拟招聘的机构工作人员，如无疑似症状且15天内未到过高风险区域、未密切接触来自高风险区域人员，无《养老机构新型冠状病毒感染的肺炎疫情防控指南（第二版）》禁止进入的情形，经医学隔离观察14天，并经专业医疗卫生机构诊断无新冠病毒感染后，可以收住入院或上岗工作。

7.优先满足本区域生活不能自理且有集中供养意愿的特困老人，因子女参加抗击疫情、因疫情接受隔离治疗等原因导致家庭无力照顾、确需返院的和新入住的生活不能自理老年人机构养老服务需求。

8.社区养老服务机构原则上继续暂停集中式服务和聚集性活动。

三、高风险区域的养老服务机构和出现疫情的养老服务机构

9.严格执行民政部印发的《新冠肺炎疫情高风险地区及被感染养老机构防控指南》，继续实施严格封闭管理。

10.对本区域生活不能自理且有集中供养意愿的特困老年人，因子女参加抗击疫情、因疫情接受隔离治疗等原因导致家庭无力照顾、确需返院和拟新入住的生活不能自理老年人，以及因机构管理服务需要确需返岗或新招聘的工作人员，要严格按照《新冠肺炎疫情高风险地区及被感染养老机构防控指南》要求，进行14天医学隔离观察，并经专业医疗卫生机构诊断无新冠病毒感染的，方可收住入院或上岗工作。

四、养老机构设置隔离点要求

11.切实做好新入机构人员的医学隔离工作，结合实际设置医学隔离观察点。具备条件的养老机构，可以内部设立隔离观察点。针对不少养老机构不具备内部隔离条件的现实困难，各地可以市或县域为单位，选择一个当前入住老年人少、具备医学隔离条件的养老机构，作为拟返院和新入住老年人、拟返院和新招聘员工的集中隔离观察点。

12.隔离观察点设置，必须符合老年人的特殊需求，按照当地疫情联防联控机制有关标准与要求，进行管理与服务。也可以利用当地已有的集中隔离场所，或专门设立集中隔离场所。

附件2

新冠肺炎疫情期间养老服务机构扶持政策要点

一、地方各级民政部门要积极争取将养老服务机构纳入扶持范围，享受以下相应优惠政策

1.《人力资源社会保障部 财政部 税务总局关于阶段性减免企业社会保险费的通知》（人社部发〔2020〕11号）。

2.《财政部 税务总局关于支持新型冠状病毒感染的肺炎疫情防控有关税收政策的公告》（财政部 税务总局公告2020年第8号）。

3.《住房和城乡建设部 财政部 人民银行关于妥善应对新冠肺炎疫情实施住房公积金阶段性支持政策的通知》（建金〔2020〕23号）。

4.《国家医保局 财政部 税务总局关于阶段性减征职工基本医疗保险费的指导意见》（医保发〔2020〕6号）。

5.《发展改革委关于阶段性降低企业用电成本支持企业复工复产的通知》（发改价格〔2020〕258号）。

6.《市场监管总局 发展改革委 财政部 人力资源社会保障部 商务部 人民银行关于应对疫情影响 加大对个体工商户扶持力度的指导意见》（国市监注〔2020〕38号）。

7.《银保监会关于加强银行业保险业金融服务配合做好新型冠状病毒感染的肺炎疫情防控工作的通知》（银保监办发〔2020〕10号）。

8.《发展改革委办公厅 民政部办公厅关于积极发挥行业协会商会作用支持民营中小企业复工复产的通知》（发改办体改〔2020〕175号）。

二、鼓励各地结合实际对养老服务机构出台针对性扶持政策

鼓励各地民政部门联合有关部门做好资金调度提前拨付养老服务体系建设补助资金。联合有关部门提高民办养老服务机构运营补贴标准、政府购买养老服务补贴标准，给予在岗工作人员临时岗位补贴。鼓励减免养老服务机构场地租金或者给予临时补贴。对疫情期间为养老服务机构减免租金的设施场地出租方，当年缴纳房产税、城镇土地使用税确有困难的，可申请困难减免。阶段性减免公建民营养老服务机构管理费。对参保综合责任保险的养老服务机构，协调保险公司加强与养老服务机构对接，适当延长保险期限、优惠或者缓缴保险费。

（162） 财政部：有效应对新冠肺炎疫情影响切实加强地方财政“三保”工作	财预〔2020〕12号

关于有效应对新冠肺炎疫情影响切实加强地方财政“三保”工作的通知

财预〔2020〕12号

各省、自治区、直辖市、计划单列市财政厅（局）：

支持基层政府保基本民生、保工资、保运转，是保障群众切身利益的基本要求，也是推动政府履职和各项政策实施的基础条件。为深入贯彻落实习近平总书记关于统筹推进新冠肺炎疫情防控和经济社会发展工作的重要指示精神，按照党中央、国务院决策部署，有效应对疫情影响，进一步加强地方财政“三保”工作，现就有关事项通知如下：

一、压实“三保”保障责任

严格按照县级为主、省级兜底的原则，将“三保”保障责任落到实处。县级要全面落实好保障责任，牢固树立“三保”与疫情防控及经济恢复并重的政策导向，充分认识巩固基层“三保”在确保社会稳定、维护市场环境、提振市场信心方面的关键作用，坚持“三保”支出在财政支出中的优先顺序，切实保障基本民生、工资发放和机构运转。省级要切实承担起主体责任，进一步加大对基层特别是财政困难县区的财力倾斜，市级要对所属区加大保障支持力度。各地要组织逐县摸排，对“三保”支出预算安排存在缺口的，要及时调整或调剂预算补足。中央财政健全激励约束机制，完善县级基本财力保障机制奖补资金、均衡性转移支付等资金分配办法，对省级调控不力的，按规定扣减后直接下达到县。有关部门要统筹本系统内各项收入和存量资金，按规定确保部门行政事业单位工资发放和正常运转。

二、阶段性提高地方财政资金留用比例

2020年3月1日至6月底，在已核定的各地当年留用比例基础上统一提高5个百分点。期间各地因提高留用比例增加的现金流，应全部通过提高县级财政资金留用比例或增加日常资金调度的方式留给县级使用，不得滞留在省级财政。

三、加快下达转移支付预算

中央财政抓紧下达对地方各项转移支付预算，增强地方基本民生保障能力。地方财政部门要加快开展省以下转移支付资金分配、拨付和使用等工作。按因素法分配的，要根据当期或上年度基础因素计算预拨；按项目法分配的，要切实加快项目审批和资金下达，或采取先预拨后清算的方式下达。承担相关支出责任的地方各级财政接到上级转移支付预算后，要尽快安排形成实际支出和实际工作量。地方各级财政尤其是县级基层财政要用好各项转移支付资金，加大统筹力度，优先用于疫情防控和“三保”支出并确保按时足额兑付，特别是要切实保障基本民生，强化对困难群众的兜底保障。

四、强化地方库款运行监测督导

各级财政部门商同级人民银行扩大分级库款余额等信息共享范围，进一步提高库款监测的时效性。财政部及时向省级财政部门发送关于库款保障水平偏低市县情况的提示函，支持疫情较重地区加强基层财政库款保障能力，确保“三保”支出需要。完善中央财政对重点县区工资保障监测预警机制，扩大监测覆盖面，同时组织各地强化工资支付保障风险评估，健全并落实好应急预案。省级财政部门要加强预研预判，综合施策，强化对县区库款运行监测，重点关注库款持续紧张的县区情况，督促困难县区合理安排支出顺序，指导并支持困难地区做好“三保”工作，坚决兜牢“三保”底线。

五、切实加大财政支出结构调整力度

各级财政部门要认真清理用好结转结余资金，超过年限的以及受疫情影响支出进度的，要收回用于急需领域。允许疫情防控任务较重、“三保”压力较大地区制定更加严格的盘活存量资金措施，报经本级政府同意后，对2019年及以前年度结转结余资金收回集中用于疫情防控和“三保”方面急需支出，待具备条件时另行安排预算。加大暂存暂付性款项清理收回力度，严控新增暂付性支出。各级财政全年“三保”支出预算未按政策足额安排的，除应急救灾外一律要调减其他项目支出。省、市两级财政部门要切实加强对县级财政调整支出结构的监督、指导、督促工作。

六、进一步大力压减一般性支出

各级财政要更加牢固树立“过紧日子”的思想，厉行勤俭节约，大力压减一般性支出和非急需、非刚性支出。可开可不开的会坚决不开、可办可不办的培训坚决不办。可暂缓实施的支出项目资金，要及时缴回财政。可统筹、整合的预算资金，要及时调整支出用途，保障“三保”支出需要，支持疫情防控和复工复产。除疫情防控需要外，各地原则上不再研究出台新的增支政策，必须出台的要充分考虑基层财政可承受能力，合理把握节奏和力度。

财政部将加强疫情防控期间地方“三保”工作的督促指导。各地财政部门要按照本通知要求切实采取措施，抓紧抓实抓细各项贯彻落实工作，牢牢兜住“三保”风险底线。财政部各地监管局要加强疫情期间本地区“三保”工作的监管，及时发现问题及风险隐患并督促整改。

财政部

2020年3月4日

（163） 财政部：加快拨付贴息资金 强化疫情防控重点保障企业资金支持	财办金〔2020〕13号

各省、自治区、直辖市、计划单列市财政厅（局），新疆生产建设兵团财政局：

为更好落实《关于打赢疫情防控阻击战 强化疫情防控重点保障企业资金支持的紧急通知》（财金〔2020〕5号）和全国强化疫情防控重点保障企业资金支持电视电话会议上关于“各地要按照战时标准和要求，迅速行动起来，落实好各项政策措施。各地财政部门要从快拨付贴息资金”的要求，现补充通知如下：

一、省级财政部门应会同相关部门严格把关，精准认定，避免将未承担疫情防控应急保障物资生产和调配任务、贷款资金未用于扩能增产的企业纳入支持范围，确保“好钢用在刀刃上”，切实提高财政贴息资金使用效益。要防止层层加码叠加贴息支持，避免出现贷款利率过低甚至负利率带来企业套利、行业攀比、管理混乱等问题。

二、为加快贴息资金拨付进度，地方各级财政部门应与本级人民银行分支机构、贷款银行加强沟通，实时掌握优惠贷款发放进度，主动上门对接服务，宣传贴息政策，可采取“先拨后结”方式，先行安排贴息资金，及时拨付至符合条件的企业，全力支持相关企业扩大产能。

三、省级财政部门应每月汇总辖区内贴息资金拨付和支持企业的有关情况，通过官网等渠道予以公示，加强正面舆论引导，宣传政策实施效果。

四、省级财政部门应于2020年5月31日前汇总一并向财政部申请贴息资金结算。

五、省级财政部门应于每周五中午前汇总贴息资金拨付数额、支持优惠贷款规模、支持企业总数等情况反馈我部金融司。

财政部办公厅

2020年3月2日

（164） 工信部：进一步做好新冠肺炎疫情防控期间宽带网络助教助学工作	工信厅通信函〔2020〕34号

各省、自治区、直辖市通信管理局，中国电信集团有限公司、中国移动通信集团有限公司、中国联合网络通信集团有限公司、中国铁塔股份有限公司：

近期，根据新冠肺炎疫情导致学校延迟开学的情况，地方通信管理局与教育主管部门加强工作协同，基础电信企业积极响应，因地制宜推出了系列支持举措，助力网上教育教学工作平稳有序开展。针对当前部分学生用户反映个别农村偏远地区信号弱、贫困家庭用网支出压力大等问题，为持续强化宽带网络助教助学能力，有效帮扶建档立卡贫困家庭学生，现就进一步做好疫情防控期间宽带网络助教助学工作通知如下：

一、提高政治站位。各地通信管理局、各基础电信企业要深入学习贯彻习近平总书记关于疫情防控工作的重要讲话和指示批示精神，按照各地“停课不停学”有关工作安排，突出重点、瞄准痛点、疏通堵点，切实抓好宽带网络助教助学工作。特别是要进一步突出重点人群、重点环节，切实把好事办好，为疫情防控学校延迟开学期间网上教育教学提供有力支撑。

二、加强网络覆盖。各地通信管理局要组织基础电信企业持续加大宽带网络和4G/5G基站建设力度，不断提升学校网络带宽条件，为各级各类学校开展在线教学提供网络支撑。要结合电信普遍服务试点项目，加快农村偏远地区网络覆盖，着力解决网速慢、信号弱等问题。要积极配合教育主管部门，按照当地教育教学工作安排，及时做好疫情重点地区、临时教育教学场所等区域的网络保障。

三、提升平台能力。各地通信管理局要主动对接教育主管部门，督促所选用的在线教育服务提供商不断优化提升平台能力，通过加强平台建设改造、按需及时扩容带宽、增加内容分发节点等方式，切实提升广大师生访问体验。工业和信息化部将会同有关部门对各级各类在线教育平台开展文件下载速率、视频卡顿率等关键性能监测，并适时向社会公布。

四、提供资费优惠。鼓励基础电信企业重点面向建档立卡贫困家庭学生推出特惠流量包等精准帮扶举措，减轻困难学生用网资费压力。各地通信管理局要加强组织协调，与当地扶贫办（局）和教育主管部门做好受帮扶对象数据对接，确保相关帮扶举措精准落实到位。

五、做好网络维护。基础电信企业要加强对宽带网络运行状态的监测，及时发现并排除故障隐患；要建立快速处理机制，成立客服专班，畅通业务咨询、服务和投诉渠道，及时解决师生反映的网络使用相关问题，支撑疫情防控学校延迟开学期间网上教育教学工作顺利实施。

特此通知。

工业和信息化部办公厅

2020年3月2日

（165） 交通运输部：分区分级科学做好客运场站和交通运输工具新冠肺炎疫情防控工作	交运明电〔2020〕84号

各省、自治区、直辖市、新疆生产建设兵团交通运输厅（局、委）：

为深入贯彻习近平总书记关于统筹推进新冠肺炎疫情防控和经济社会发展的重要指示精神，落实中央应对新冠肺炎疫情工作领导小组决策部署，按照国务院应对新冠肺炎疫情联防联控机制关于进一步落实分区分级差异化防控策略的要求，科学做好客运场站和交通运输工具疫情防控工作，部制定了《客运场站和交通运输工具新冠肺炎疫情分区分级防控指南》（见附件，以下简称《指南》），请结合实际贯彻执行，有关事项通知如下：

一、分区分级差异化施策，科学推进客运场站和交通运输工具疫情防控工作

客运场站和交通运输工具是人员密集场所，疫情传播风险较大。各地交通运输主管部门要深刻认识客运场站和交通运输工具疫情防控工作的重要性、紧迫性，结合复工复产和交通运输秩序恢复，按照分区分级差异化防控策略，科学推进客运场站和交通运输工具疫情防控工作。要督促指导客运场站和客运企业严格落实《指南》规定，全面做好道路客运、城市公共汽电车、轨道交通、出租汽车、水路客运领域场站设施和交通运输工具的消毒、通风、运输组织、人员防护和宣传工作，坚决遏制疫情通过客运场站和交通运输工具传播。要指导客运场站和客运企业加强从业人员，特别是一线工作人员卫生防护和疫情防控相关的知识技能培训，切实提升防控能力。要积极争取当地疫情防控领导机构和有关部门的支持，全力保障客运场站和交通运输工具防疫物资供给，切实提高交通运输领域疫情防控能力和水平。

二、落实企业主体责任，切实抓好《指南》的贯彻落实

各地交通运输主管部门要督促指导客运场站运营单位按照《指南》要求，科学做好公共区域、乘客接触设施设备等的清洁、消毒、通风、换气等工作，保证空气流通和环境整洁；根据需要科学采取限流措施，减少站内人员聚集，并劝导乘客佩戴口罩，做好乘客体温测量、留观区设置等工作。要督促客运企业做好车辆内部、行李舱等部位和设施设备的清洁消毒，加强车辆通风换气；根据区域疫情风险等级，差异化控制客座率，并落实乘客体温测量、留观区设置、消毒剂配备等要求。要督促客运场站和客运企业通过车站电子屏、站内广播、车载视频、海报等多种形式，积极开展卫生防护和疫情防控知识宣传，增强从业人员和旅客科学防护的意识和能力。

三、引导公众广泛参与，推动形成群防群治的工作局面

各地交通运输主管部门要充分利用电视、广播、门户网站、微信公众号等多种途径，向社会各界广泛宣传客运场站和交通运输工具疫情防控技术要求和《指南》内容，引导广大旅客按疫情防控要求有序乘坐交通运输工具，共同维护良好交通运输秩序。要组织客运场站和客运企业及时向社会公示监督电话，畅通公众诉求渠道，主动接受社会监督；鼓励社会公众积极参与交通运输疫情防控工作，及时发现和纠正疫情防控漏洞，第一时间消除疫情风险和安全隐患，推动形成群防群治的工作局面。

铁路、民航、邮政领域场站及交通运输工具分区分级疫情防控要求，由国家铁路局、中国民用航空局、国家邮政局分别负责制定。

附件：客运场站和交通运输工具新冠肺炎疫情分区分级防控指南.doc

交通运输部

2020年3月1日

（166） 中央应对新型冠状病毒感染肺炎疫情工作领导小组：印发当前春耕生产工作指南	国发明电〔2020〕7号

各省、自治区、直辖市党委和人民政府，新疆生产建设兵团，中央和国家机关有关部门：

现将《当前春耕生产工作指南》印发给你们，请结合实际抓好贯彻落实。

中央应对新型冠状病毒感染肺炎疫情

工作领导小组

2020年3月2日

当前春耕生产工作指南

为认真贯彻习近平总书记在统筹推进新冠肺炎疫情防控和经济社会发展工作部署会议上的重要讲话精神，落实李克强总理在国务院常务会议上的部署要求，统筹抓好新冠肺炎疫情防控和春耕生产工作，分区分级恢复生产秩序，不失时机抓好春季农业生产，夺取粮食和农业丰收，为实现全年经济社会发展目标任务提供有力支撑，特制定本指南。

一、压实责任确保春播粮食面积

（一）压实粮食安全省长责任制。将做好春耕生产工作作为粮食安全省长责任制考核的重要内容，年底通报考核结果，各省（自治区、直辖市）粮食播种面积和产量要稳定在上年水平。各地要提高政治站位，主产区要努力发挥优势，产销平衡区和主销区要保持应有的自给率，共同承担起维护国家粮食安全的责任。抓紧将任务分解落实到市到县，层层压实责任。

（二）调动农民种粮积极性。各地要落实好小麦、稻谷最低收购价政策，统筹实施好玉米、大豆生产者补贴和稻谷补贴政策，推进小麦、稻谷、玉米完全成本保险和收入保险试点，稳定农民收益预期，保护和调动农民种粮积极性。加强政策解读和舆论引导，给农民吃上“定心丸”。

（三）稳定春播粮食面积。引导农民合理安排种植结构。鼓励有条件的地区恢复双季稻面积，稳定早稻面积并力争有所扩大。适当调整轮作休耕试点，扩大轮作、减少休耕，轮作以种植粮食作物为主。粮改饲政策主要支持北方农牧交错带。要通过代耕代种等方式帮助缺乏劳动力的农户种足种满，杜绝耕地撂荒。

二、分级分类尽快恢复春耕生产秩序

（四）细化农村疫情防控方案。落实分类细化农村疫情防控科学指导的要求，从农村实际出发，以县域为单位，抓紧制定差异化防控措施，划小管控单元，科学防控、精准施策，纠正偏颇和极端做法，不搞“一刀切”。打通农资供应、农机作业、农民下田等堵点，尽可能减少疫情防控对春耕生产的影响。

（五）低风险地区全面恢复生产秩序。低风险地区实施“外防输入”策略，尽快全面恢复农业生产秩序。保障城乡交通运输正常运转，不得封路封村，确保农民正常下田、农机顺利上路、农资顺畅流通。抓紧动员农民下田，开展施肥打药、育秧泡田、深松整地、除草修剪等农事活动，推动春耕生产全面展开。

（六）中风险地区有序恢复生产秩序。中风险地区实施“外防输入、内防扩散”策略，尽快有序恢复春耕生产秩序。在采取必要防控措施的同时，保证农资、农产品正常流通，根据疫情防控实际，合理安排春耕生产，组织农民错时下田、错峰作业，做到疫情防控与春耕生产统筹推进。

（七）高风险地区稳妥恢复农业生产。高风险地区在继续集中精力抓好疫情防控工作的同时，根据疫情态势逐步恢复春耕生产。田间野外作业、人员密度低的地区，因地制宜组织农民开展农业生产。作业空间密闭、人员较为密集的地区，组织农民错时错峰下田，避免人员聚集。疫情最重的湖北和疫情较重的省份，也要根据实际情况在做好防控前提下组织农民开展春耕生产。

三、分区按时抓好春管春耕

（八）南方早稻产区抓好育秧栽插。华南稻区2月下旬开始育秧，长江流域3月中旬育秧。指导农民选择高产优质早稻品种，及早备足种子。适时浸种泡田，抢晴播种育秧，大力推广工厂化育秧、集中育秧。加强秧田管理，科学调控肥水，培育壮秧。加快栽插进度，扩大机插秧面积，确保种在适播期。

（九）夏粮主产区做好麦田管理。2月下旬江淮、黄淮南部冬小麦进入返青起身期，3月上中旬大部分冬小麦返青起身，春管进入高峰期。因苗施策，分类管理，合理调控肥水，落实促弱控旺措施。防范“倒春寒”等灾害，适时灌水、追肥。及早准备药剂药械，防控重大病虫害，组织开展统防统治。

（十）长江流域中稻产区做好育秧准备。4月下旬至5月上旬中稻开始浸种育秧，5月下旬至6月上旬开始移栽。根据季节安排、茬口衔接、品种生育期，适时播种育秧。推广集中育秧、旱育秧或湿润育秧，确保苗齐苗匀苗壮。及时泡田整地，适时移栽，合理确定密度，提高栽插质量。

（十一）东北西北地区做好春耕备耕。4月上中旬东北水稻开始浸种育秧，4月中旬至5月上旬东北西北旱地作物开始播种。科学确定主推品种，指导农民选用适期品种，防止越区种植。及时下摆种子、化肥、农药、农膜等农资。适时开展春整地、春覆膜，加强墒情监测，检修调试机械，组织农机抢早整地。

（十二）做好春灌用水调度。根据农作物需水要求，做好春灌用水计划，加强灌溉用水调配。充分发挥农民用水合作组织作用，维护好灌溉秩序。利用灌溉间隙，加快完善灌排设施，保障春灌用水。

四、保障春耕生产农资供应

（十三）推动农资企业复工复产。组织指导种子、化肥、农药、农膜等关系春耕生产的农资生产企业做好工作场所疫情防控、员工个人防护，尽快全面复工复产，提高开工负荷，切实增加市场供应。因缺乏劳动力影响正常复工复产的，及时采取用工替代等措施，加强地区间劳务供需对接。

（十四）推进农资到村到户。突出解决农资运销“最后一公里”受阻问题，建立农资“点对点”保供以及化肥生产原辅料和产品运输绿色通道，禁止违法设卡拦截、断路封路等行为，畅通农资运输。调度农资供需情况，建立乡村应急配送机制，保障即时配送，尽早有序恢复门店营业。

（十五）加强农资市场监管。瞄准农资经营集散地、春耕春管重点地区，围绕重点农资品种，依法依规加强监督执法，开展质量监督抽查，查找问题隐患，严厉打击制售假劣农资、哄抬价格等违法违规行为，严肃查处假劣农资案件。

五、抓好科学防灾减灾

（十六）加强农田水利建设。着力解决砂石原料、机械、人员等运输难进场难问题，推动春季农田水利工程和高标准农田尽快复工建设，确保完成年度建设任务。完善工程建设验收监督检查机制，建一块成一块，提升抗御自然灾害能力。

（十七）防范自然灾害。今年区域性、阶段性旱涝灾害趋势较为明显，极端天气气候事件可能增多，要重点做好防寒抗冻和防涝抗旱。加强监测预警，及早制修订灾害防范预案，做好物资和技术准备。强化技术服务，指导农民落实抗灾救灾和灾后恢复生产措施。

（十八）防控重大病虫害。加强西南、汉水流域、黄淮海地区小麦条锈病和江淮、黄淮地区小麦赤霉病监测防治。特别要做好草地贪夜蛾分区防控，实施防线前移、划区布防，开展统防统治和联防联控，坚决遏制病虫害扩散蔓延。加强沙漠蝗入侵监测预警，做好药剂药械准备，确保一旦发生立即消灭。

六、创新方法开展指导服务

（十九）搞好技术指导培训。充分利用广播电视、网络微信、“12316”平台等手段，组织专家开展在线培训、在线指导、在线答疑。组织农技人员在做好个人防护的前提下，开展必要的实地指导，帮助农民解决春耕生产实际困难。

（二十）做好规模主体帮扶。支持农业规模经营主体特别是设施农业生产主体，积极组织返乡农民工和闲置劳动力等，解决生产用工难题。有序组织农民工返岗复工，采取“点对点、一站式”直达运输服务。帮助粮食生产规模经营主体搞好土地流转合同签订、续签等。各地在组织农民工返岗复工和指导春耕生产中，要把完成脱贫攻坚任务作为工作重点，对贫困人口予以优先考虑。

（二十一）开展农机作业服务。制定农机跨区作业预案，合理调剂调配机具，保障农机作业畅通。组织好跨区机耕机播，高质量高标准完成春播任务。组织动员有关生产主体和企业，做好人员培训、机具准备和检修、维修配件供应等，确保农机具和农机手能够及时投入生产。

（167）交通运输部关于分区分级科学做好疫情防控期间城乡道路运输服务保障工作的通知	交运明电〔2020〕80号

各省、自治区、直辖市、新疆生产建设兵团交通运输厅（局、委）：

为贯彻落实国务院应对新型冠状病毒感染肺炎疫情联防联控机制关于分区分级做好新冠肺炎疫情防控工作的决策部署，科学有序恢复城乡道路运输服务，有力支撑企业复工复产和经济社会平稳运行，现将有关事项通知如下：

一、分区分级恢复城乡道路运输服务

各省级交通运输主管部门要在当地疫情防控工作机制领导下，根据辖区内低风险、中风险、高风险县（市、区、旗）名单，落实分区分级管控要求，有序恢复道路客运、城市公共交通（含城市轨道交通）和出租汽车（含网约车）等城乡道路运输服务。低风险地区要按照“外防输入”原则，立即全面恢复城乡道路运输服务。中风险地区要按照“外防输入、内防扩散”原则，在做好疫情防控工作前提下，尽快有序恢复城乡道路运输服务。高风险地区要按照“内防扩散、外防输出、严格管控”的原则，采取有效措施保障医护人员、公共事业运行一线人员等重点群体的必要出行，并结合疫情防控形势变化情况，稳妥有序恢复城乡道路运输服务。湖北省要继续实行严格的离汉离鄂通道管控措施。北京市的省际道路客运服务，按照首都进京人员防控机制的决策执行。除湖北省和北京市以外，其他暂未恢复省际道路客运服务的地区，不得阻碍其他非高风险县（市、区、旗）发出的客运车辆进入或者途经本地。

二、严格落实交通运输疫情防控措施

各地交通运输主管部门要督促指导道路运输经营者和从业人员按照《交通运输部等部门关于统筹做好春节后错峰返程疫情防控和交通运输保障工作的通知》（交运明电〔2020〕44号）等部署要求，严格执行客运场站和交通工具消毒通风、乘客测温和信息登记、发热乘客移交、从业人员和乘客防护等疫情防控措施。对中、高风险地区客运场站和出入中、高风险地区的交通工具，要在落实上述疫情防控措施的基础上，适当增加消毒通风频次。疫情防控期间，三类以上班线客车和包车要在客车后两排设置留观区域；出入高、中风险地区（不含途经）的省际、市际客运班车、包车，要将客座率控制在50%以内。严禁客运班车、包车在途经的高风险地区上下客。

三、切实加强道路运输安全监管

各地交通运输主管部门要牢固树立安全发展理念，在做好疫情防控的同时，切实保障道路运输安全运营，坚决遏制重特大事故发生。要督促经营者严格落实安全生产管理制度，加强车辆动态监控，严格执行长途客运车辆凌晨2时至5时停车休息或者接驳运输规定，强化气象信息预报预警，提升道路运输安全风险防控能力。要会同公安机关加大联合监管执法和信息共享力度，依法从严查处“三超一疲劳”、非法营运、客运车辆超范围经营、违规异地经营等违法违规行为。对农民工返岗包车，要强化针对性的安全措施，加强行驶路线规划、车辆检测维护、司乘人员培训、运输过程监控等全流程管理，切实消除安全隐患，确保运营安全。

交通运输部

2020年2月27日

（168）文化和旅游部：积极应对疫情影响保持导游队伍稳定相关工作

文化和旅游部办公厅关于积极应对疫情影响保持导游队伍稳定相关工作事项的通知

各省、自治区、直辖市文化和旅游厅（局），新疆生产建设兵团文化体育广电和旅游局：

导游是促进旅游业发展的重要力量，是提升旅游服务质量的关键因素。为落实中央关于统筹推进新冠肺炎疫情防控和经济社会发展工作的整体部署，保持疫情期间导游队伍稳定，为旅游业恢复发展蓄力储能，现将有关事项通知如下：

一、切实提高思想认识

保持疫情期间导游队伍稳定是贯彻落实中央关于统筹推进新冠肺炎疫情防控和经济社会发展工作整体部署、全面强化稳就业举措的必然要求，是旅游市场恢复经营的重要前提，是提升旅游服务质量、推动旅游业高质量发展的关键因素。各地要进一步提高政治站位，从统筹推进新冠肺炎疫情防控和经济社会发展的大局出发，深刻认识疫情期间保持导游队伍稳定的必要性，把此项工作放在重要位置，结合本地实际，采取有效措施，保持导游队伍稳定。

二、强化劳动权益保护

各地要加强疫情期间导游人员权益保护工作,依法落实导游人员权益保护相关规定。要指导和支持行业组织切实履行维护导游合法权益等相关责任，加强对旅行社依法履行劳动合同义务的监督，在疫情期间非因法定事由不得提前解除与导游签订的劳动合同。要指导行业组织、旅行社落实好有关部门、当地政府出台的疫情期间稳定劳动关系的相关政策。

三、开展线上免费培训

各地要充分利用疫情待岗这一时机，着眼导游从业需求，指导支持行业组织、旅行社运用在线培训平台，开展形式多样的线上免费培训，帮助导游提升专业素养。要从全国导游大赛获奖选手、“金牌导游”、导游大师工作室、旅游院校专家和一线优秀导游中精选师资，突出线上培训特点，开设互动环节，增强培训工作实效。

四、完善综合服务保障

各地要加强资金保障，对于疫情期间积极开展线上免费培训的行业组织、旅行社及导游，可按相关规定给予适当补助；对表现突出的导游，可在“金牌导游”遴选和工作室创办等方面予以相应支持。要加强服务保障，充分发挥行业组织、旅行社的积极作用，及时回应导游关切。各地要积极协调和支持本地区行业组织免除注册导游2020年全年会费。针对目前导游关注的“受疫情影响，到期导游证无法按时换发，影响后续执业”的情况，换证手续可延期半年办理。

五、不断加强关爱引导

各地要建立导游人员关爱机制，加强政策宣传和组织协调，及时了解当地导游人员思想动态，有针对性地做好心理疏导工作。对确实存在突出困难的导游提供必要帮扶救助，解决实际困难。

特此通知。

文化和旅游部办公厅

2020年2月27日

（169）发改委：阶段性减免部分征信服务收费	发改价格〔2020〕291号

人民银行：

为深入贯彻落实党中央、国务院决策部署，统筹推进新冠肺炎疫情防控和经济社会发展，助力企业复工复产、共渡难关，现就你行征信中心阶段性减免部分征信服务收费有关事项通知如下。

一、免收企业应收账款质押登记、变更登记、异议登记费。

二、免收农村商业银行、农村合作银行、农村信用社、村镇银行、小额贷款公司、消费金融公司、融资租赁公司、融资性担保公司、民营银行、独立法人直销银行等10类金融机构征信查询服务费，包括查询企业信用报告和个人信用报告服务费。

三、上述措施，自2020年3月1日至2020年6月30日执行。

国家发展改革委

2020年2月28日

（170）卫健委：基层医疗卫生机构在新冠肺炎疫情防控中分类精准做好工作	国卫办基层函﹝2020﹞177号

各省、自治区、直辖市及新疆生产建设兵团卫生健康委：

为贯彻落实国务院联防联控机制《关于科学防治精准施策分区分级做好新冠肺炎疫情防控工作的指导意见》，指导各地基层医疗卫生机构根据当前疫情防控形势发展的趋势变化，突出重点、统筹兼顾、分类施策，在基层新冠肺炎疫情防控中差异化、精准化推进工作，有效落实落细相关工作措施，进一步把基层卫生健康服务的“网底”兜实、兜牢，现就有关事项通知如下：

一、进一步增强统筹做好疫情防控和日常工作的责任感

新冠肺炎疫情发生以来，各地基层医疗卫生机构坚决贯彻党中央、国务院决策部署，落实国家卫生健康委和地方各级卫生健康行政部门工作要求，履职尽责，全力参与疫情防控工作，为有效遏制疫情在城乡社区的扩散和蔓延，发挥了重要的基础性作用。目前，全国疫情防控形势积极向好的态势正在拓展，各地按照统筹做好疫情防控和经济社会发展的工作部署，正在分区分类、逐步有序恢复生产生活秩序。各地社区卫生服务中心（站）、乡镇卫生院、村卫生室等基层医疗卫生机构要结合区域疫情防控态势，进一步强化工作责任，统筹做好疫情防控和日常诊疗、慢性病管理、健康指导等工作，确保城乡居民及时、就近获得基本卫生健康服务。

二、分区分类科学精准开展工作

各地要落实以县域为单位实施差异化防控的总体要求，社区卫生服务中心（站）和乡镇卫生院、村卫生室在地方党委政府和卫生健康行政部门的统一领导下，根据区域风险级别,配合城乡社区组织在基层落实针对性疫情防控措施和做好基本医疗卫生服务工作。

（一）疫情防控低风险县（市、区）的基层医疗卫生机构，一方面要贯彻落实区域“外防输入”策略，加强门诊预检分诊筛查，做好对发热患者的监测、发现、报告和转诊。协助落实对重点地区和高风险地区返回人员管理措施。加强公众健康宣教，引导做好个人防护。另一方面要全面恢复正常医疗卫生服务秩序，为居民提供基本诊疗、基本公共卫生服务、健康管理等服务。要合理安排门诊时间，积极推行预约服务，减少就诊居民排队聚集。要注意加强内部消毒、环境卫生工作，严防机构内感染事件发生。

（二）疫情防控中风险县（市、区）的基层医疗卫生机构，要贯彻落实区域“外防输入、内防扩散”策略，在采取低风险地区各项防控措施基础上，做好人力、物资、隔离观察场所等方面的准备。会同城乡社区组织落实“四早”措施，实施网格化、地毯式管理，协助落实对辖区病例密切接触者的追踪排查和隔离医学观察措施，配合疾控机构开展流行病学调查。根据《国家卫生健康委办公厅关于做好新型冠状病毒肺炎出院患者跟踪随访工作的通知》（国卫办医函〔2020〕142号）要求，协助落实对出院患者的随访管理。加强疫情防控宣传教育，强化家庭防护措施，防范家庭聚集病例。协助做好病例家庭、楼栋单元等疫点的消毒。结合区域疫情防控趋势逐步有序恢复医疗卫生服务秩序，保证基本卫生健康服务提供。

（三）疫情防控高风险县（市、区）的基层医疗卫生机构，要协助落实好辖区“内防扩散、外防输出、严格管控”策略，在采取中风险地区各项防控措施基础上，全力参与做好城乡社区综合防控工作，及时协助落实社区管控和限制人员聚集等措施。要合理调配人力，重点关注辖区老年人、孕产妇、慢性病患者等人群基本卫生健康和用药需求。

三、有序开展家庭医生签约和基本公共卫生服务

疫情防控期间，鼓励基层医疗卫生机构在实施家庭医生签约服务和基本公共卫生服务项目中创新服务模式，优化服务流程，积极利用互联网手段，提高服务效率。

根据区域疫情防控分级情况，低风险县（市、区）持续开展家庭医生签约和基本公共卫生服务。中、高风险县（市、区）要加强分层分类管理，基层医疗卫生机构暂缓安排老年人健康体检等服务，合理调整儿童保健门诊和预防接种门诊，暂缓面对面新生儿访视与儿童健康体检，针对孕产妇、儿童、老年人和高血压、糖尿病等慢性病患者的健康管理，可通过电话、微信、短信、视频、智能语音、区域健康云APP等多种途径开展随访服务，相关随访记录应及时录入居民健康档案。鼓励依托区域卫生健康云平台实现随访数据的联通与共享。家庭医生服务团队要主动关心签约居民，推送针对性、个性化的健康教育和疫情防控信息，提供健康宣教服务，指导签约居民开展自我健康管理与个人防护，进一步提升签约居民对家庭医生签约服务的满意度与获得感。对于诊断明确、病情稳定的慢性病患者，基层医疗卫生机构应当按照规定落实慢性病患者长期处方、延伸处方等政策，减少患者不必要的就诊频次。

各地要确保2020年基本公共卫生服务经费及时足额到位，人均经费补助标准中新增部分全部落实到乡村和城市社区，统筹用于基层医疗卫生机构开展疫情防控的人员经费、公用经费等支出，创新方式完善项目实施情况绩效评价。

四、充分发挥县域医共体支持作用

各地要充分发挥县域医共体推进资源下沉的协同作用。疫情防控低风险县（市、区）要抓紧推进紧密型县域医共体建设，进一步增强疫情防控和基本医疗卫生服务能力。疫情防控中、高风险县（市、区）要强化牵头医院对基层医疗卫生机构的培训和指导工作，对防控力量薄弱的基层医疗卫生机构及时安排支持、支援人员，充实基层防控力量，切实提高区域综合疫情防控能力。依托区域远程医疗促进优质资源向基层辐射下沉。可通过县域医共体加强对基层机构慢性病、特殊疾病用药的配备，满足居民就近用药需求。鼓励在基层疫情防控中推广行之有效的中医药防治方案，充分发挥中医药的独特优势和作用。

五、进一步关心爱护基层医务人员

各地要继续加大对基层医疗卫生机构消毒、防护物资调配力度，保障参与疫情防控基层医务人员防护用品。加强对基层医务人员的关心支持，保证合理作息时间，主动了解并协调解决他们的实际困难。加大对在疫情防控工作中表现突出的基层医疗卫生机构和基层医务人员的表扬力度。大力宣传基层医疗卫生机构和基层医务人员先进事迹，积极营造良好工作氛围。

各省级卫生健康行政部门要加强组织领导，推动各地基层医疗卫生机构切实增强大局意识、责任意识，在城乡社区防控工作中有效发挥专业支撑作用，并结合区域风险等级动态调整逐步推动由全面防控向精准防控、重点防控转变，统筹抓好疫情防控和日常医疗卫生服务两项工作，关注重点人群、重点环节，为打赢疫情防控的人民战争、总体战、阻击战打下坚实工作基础。

国家卫生健康委办公厅

2020年3月1日

（171）医保局 卫健委：推进新冠肺炎疫情防控期间开展“互联网+”医保服务

各省、自治区、直辖市、新疆生产建设兵团医保局、卫生健康委：

为坚决贯彻落实党中央、国务院关于加强新型冠状病毒肺炎疫情防控工作的决策部署，方便广大参保人员就医购药，减少人群聚集和交叉感染风险，按照《国务院办公厅关于促进“互联网+医疗健康”发展的意见》（国办发〔2018〕26号）等文件精神，现就疫情期间开展“互联网+”医保服务提出如下指导意见：

一、将符合条件的“互联网+”医疗服务费用纳入医保支付范围

经卫生健康行政部门批准设置互联网医院或批准开展互联网诊疗活动的医疗保障定点医疗机构，按照自愿原则，与统筹地区医保经办机构签订补充协议后，其为参保人员提供的常见病、慢性病“互联网+”复诊服务可纳入医保基金支付范围。

按照《国家医疗保障局关于完善“互联网+”医疗服务价格和医保支付政策的指导意见》（医保发〔2019〕47号）规定，落实相关价格和支付政策。定点非公立医疗机构提供的“互联网+”复诊服务，参照定点公立医疗机构的价格和支付政策进行结算。

二、鼓励定点医药机构提供“不见面”购药服务

落实“长处方”的医保报销政策。积极推进城乡居民高血压、糖尿病门诊用药保障机制落地。参保人员凭定点医疗机构在线开具的处方，可以在本医疗机构或定点零售药店配药。探索推进定点零售药店配药直接结算，按照统筹地区规定的医保政策和标准，分别由个人和医保基金进行结算，助力疫情防控。鼓励定点医药机构在保障患者用药安全的前提下，创新配送方式，减少人群聚集和交叉感染风险。

三、完善经办服务

统筹地区医保经办机构与提供“互联网+”医疗服务的定点医疗机构签订补充协议时，应明确纳入医保支付的“互联网+”医疗服务范围、条件、收费和结算标准、支付方式、总额指标管理以及医疗行为监管、处方审核标准等，原则上对线上线下医疗服务实行统一管理。医保经办机构要与定点医药机构密切配合、做好对接，对符合规定的“互联网+”医疗服务、在线处方药费等实现在线医保结算。

四、不断提升信息化水平

使用医保电子凭证实现互联网医保服务无卡办理。前期已经开通医保电子凭证实现互联网医保服务的省份，继续做好推广应用工作。未开通医保电子凭证的省份，按照《国家医疗保障局办公室关于全面推广应用医保电子凭证的通知》（医保办〔2020〕10号）要求开展工作；未开通省份暂不具备开通条件的，保持现有信息系统稳定，避免重复建设、分散建设。同步做好互联网医保服务有关数据的网络安全工作，防止数据泄露。

五、加强医保基金监管

根据“互联网+”医疗服务特点，落实线上实名制就医，配套建立在线处方审核制度、医疗服务行为监管机制，保障诊疗、用药合理性，防止虚构医疗服务，确保医保基金安全。定点医药机构应当为患者建立和妥善保存电子病历、在线电子处方、购药记录等信息，做到诊疗、处方、交易、配送全程可追溯，实现信息流、资金流、物流全程可监控，满足患者可以在线查询检查检验结果、诊断治疗方案、处方和医嘱等病历资料。

六、确保工作平稳有序开展

要做好“互联网+”医保服务政策宣传，坚持正确舆论导向，准确解读相关政策。要提供必要的电话和网上咨询服务，及时为群众解答有关问题。要做好疫情期间系统上线、完善应用、情况上报、评估总结等工作，及时总结推广成功经验做法，逐步向有条件的定点医药机构和地区推广，更好为广大参保群众提供优质的服务。

国家医保局 国家卫生健康委

2020年2月28日

（172）卫健委：进一步落实科学防治精准施策分区分级要求做好疫情期间血液保障工作	联防联控机制发〔2020〕31号

各省、自治区、直辖市及新疆生产建设兵团应对新型冠状病毒肺炎疫情联防联控机制（领导小组、指挥部）：

新冠肺炎疫情发生以来，各地高度重视血液安全供应工作，建立血液库存监测预警制度，加强无偿献血宣传动员，启动血液调配机制，血液供应平稳有序，有力保障临床医疗救治。近期，随着正常的医疗服务秩序逐步恢复，临床用血需求大幅增加，部分地区出现血液紧张的问题。为进一步做好血液供应工作，满足临床用血需求。现将有关要求通知如下：

一、落实分区分级管理，完善血液保障策略

各省级应对新型冠状病毒肺炎疫情联防联控机制（领导小组、指挥部）应当认真落实分区分级差异化管理要求，制定低风险、中风险和高风险地区的血液保障策略，将血液保障供应与正常医疗服务恢复同部署、同落实。

地方各级人民政府应当进一步落实《献血法》，充分发挥无偿献血领导责任，提前谋划，狠抓落实。地方各有关部门应当在联防联控机制（领导小组、指挥部）领导下，加强无偿献血宣传、教育、组织、动员等工作。各级地方卫生健康行政部门应当会同有关部门，制定并实施血液供应保障预案，实现血站与医疗机构有效联动、无偿献血与临床用血同步发展，切实确保临床用血供需平衡。

二、加强宣传动员工作，提供优质献血服务

各地要结合实际情况，加强无偿献血宣传工作，重点宣传疫情防控期间血液保障的重要意义，弘扬无私奉献和献血救人的人道主义精神，普及无偿献血和疫情防控知识，消除人民群众疫情期间献血的顾虑。利用传统媒体和新媒体，通过发布倡议书等形式，做好无偿献血社会动员工作。各地应当组织动员各类党政机关、企事业单位等组织参加团体无偿献血，充分发挥团体无偿献血的作用。乡镇、街道、社区应当做好辖区内动员工作。

各地各部门应当为无偿献血提供支持和便利条件，协调解决流动采血车停放问题，允许采血车进乡镇、社区和单位。各级卫生健康行政部门应当按照分区分级管理要求，指导血站调整无偿献血招募模式，鼓励利用电话、互联网开展献血者招募，献血模式应当由传统的街头随机献血向预约献血转变，开展流动献血车进单位、进社区、进乡镇活动。各血站应当会同有关部门进一步优化献血流程，改善献血环境，做好清洁消毒，保障献血者权益及献血安全。

三、健全血液预警机制，加强科学合理用血

各级卫生健康行政部门应当建立血液库存和血液安全预警机制。要密切监测辖区内血液采集、供应和库存水平，对于血液库存低于5天使用量的，应当启动血液应急保障预案，并向同级人民政府报告，充分进行社会动员，启动调剂机制，不断提升血液库存水平。各省级卫生健康行政部门应当结合当地实际情况确定血液筛查策略，根据风险级别开展新冠肺炎病毒血清学或核酸检测，保证血液安全。各级卫生健康行政部门应当指导医疗机构与血站加强信息沟通，共同应对、妥善处理血液保障工作中面临的问题，保障临床急救用血和日常医疗用血。

各级卫生健康行政部门应当将临床合理用血工作作为保障医疗质量安全的重要内容，指导医疗机构加强临床用血管理，规范用血标准，不断健全临床合理用血评价制度。各医疗机构应当推广合理用血新理念，严格用血指征，实现临床用血精准化，有效节约血液资源。

国务院应对新型冠状病毒肺炎疫情联防联控机制

（代章）

2020年3月1日

（173）林草局：贯彻落实《全国人民代表大会常务委员会关于全面禁止非法野生动物交易、革除滥食野生动物陋习、切实保障人民群众生命健康安全的决定》	林护发〔2020〕22号

各省、自治区、直辖市林业和草原主管部门，内蒙古、大兴安岭森工（林业）集团公司，新疆生产建设兵团林业和草原主管部门，国家林业和草原局各司局、各派出机构、各直属单位：

2020年2月24日，十三届全国人大常委会第十六次会议审议通过了《全国人民代表大会常务委员会关于全面禁止非法野生动物交易、革除滥食野生动物陋习、切实保障人民群众生命健康安全的决定》（以下简称《决定》）。为贯彻执行《决定》精神，全面禁止食用野生动物，现就有关要求通知如下：

一、深入学习领会《决定》精神，统一思想认识，提高政治站位

非法野生动物交易、滥食野生动物不仅危害野生动物种群安全和国家生态安全，而且对公共卫生安全和人民群众身体健康构成重大隐患。党中央对此高度重视，习近平总书记多次作出重要指示批示。在当前新冠肺炎疫情防控的关键时期，全国人大常委会聚焦滥食野生动物这一突出问题出台专门决定，不仅为全面禁食野生动物，严厉打击非法野生动物交易，从源头上防范和控制公共卫生安全风险提供了有力的法制保障，而且对提高全社会的生态保护和公共卫生安全意识，加强生态文明建设，促进人与自然和谐共生，具有重要意义。各级林业和草原主管部门要立即组织专题学习，以习近平新时代中国特色社会主义思想为指导，进一步增强“四个意识”，坚定“四个自信”，做到“两个维护”，深入领会出台《决定》的必要性和紧迫性，准确把握《决定》精神，提高政治站位，强化责任担当，加强组织领导，周密部署，综合施策，坚决贯彻执行《决定》各项规定。

二、严厉打击乱捕滥猎、非法交易野生动物等活动，坚决取缔非法野生动物市场

各级林业和草原主管部门要抓住《决定》公布施行的有利时机，集中调配力量，在野生动物重要分布区、栖息地，实行分区划片，明确保护责任，全面加强野生动物保护。特别是在候鸟大规模集群的越冬地、繁殖地、迁飞通道，要加密保护哨站、巡护线路的设置，进一步加大野外巡护看守力度，全面清除鸟网、猎套等非法猎捕工具，切实维护候鸟等野生动物种群安全。要主动配合市场监管、网络监管、交通运输等部门，对市场上摆卖食用野生动物的摊位、门店、野味餐馆等场所一律依法予以关闭、查封，对网络发布的食用野生动物交易信息和其他非法野生动物交易广告一律依法予以清除，进一步净化市场环境和网络空间；对违法运输、寄递野生动物及制品的，加大查验追踪力度，依《决定》加重处罚，切实有效阻断非法交易链条。要建立有奖举报机制，广泛收集违法犯罪线索，并配合公安机关对大案要案一追到底，摧毁犯罪团伙，从严加重惩处，形成强大的震慑声势，坚决遏制乱捕滥猎、非法交易野生动物等违法犯罪活动的势头。

三、全面整顿野生动物人工繁育和经营利用从业机构，依法清理许可证件及文书

各级林业和草原主管部门要对本区域所有以食用为目的从事野生动物人工繁育、经营利用的机构，开展一次彻底的检查整顿。对违法从事野生动物人工繁育、经营利用活动的机构，依法加重处罚；对取得人工繁育许可证，以食用为目的从事陆生野生动物人工繁育的，须撤回并注销所核发的人工繁育许可证件或文书，并一律停止为食用目的出售、运输野生动物等活动；对从业机构人工繁育种类列入《畜禽遗传资源目录》、林业和草原主管部门已核发人工繁育许可证件或文书的，须一律撤回并注销所核发的许可证件或文书，其人工繁育种群管理适用《畜牧法》的规定；对从业机构人工繁育种类列入《人工繁育国家重点保护水生野生动物名录》或《国家重点保护经济水生动植物资源名录》，林业和草原主管部门已核发人工繁育许可证件或文书的，也一律撤回并注销所核发的许可证件或文书，今后按水生野生动物管理；对以食用为目的从事野生动物经营利用的机构，已取得以食用为目的的经营利用许可证件和文书一律予以撤回并注销或申明作废，停止一切以食用为目的的经营利用陆生野生动物活动。

四、一律停止受理以食用为目的猎捕、经营野生动物等活动的申请，严格规范对非食用性利用野生动物活动的审批行为

自《决定》公布之日起，各级林业和草原主管部门一律停止受理以食用为目的猎捕、出售、购买、进口野生动物等活动的行政许可申请。因科研、药用、展示等特殊情况，需要对野生动物进行非食用性利用的，依法负责审批的林业和草原主管部门要制定严格的审批条件和程序，列明需提交的证明材料，建立科学评估机制，经审核确认来源合法、符合法定条件的，方可予以批准，并须在批准文件中明确要求申请人依法接受检验检疫，有效防范疫源疫病的传播和扩散。同时，要加强对非食用性利用野生动物活动的后续监管，防止滥用、虐待等不当方式，坚决杜绝假借合法渠道从事非法交易活动和食用的行为。要加强对审批工作人员的政治思想教育、政策学习和专业培训，进一步规范行政审批行为，提高审批质量，对行政许可工作中的徇私舞弊、玩忽职守、滥用职权等不依法履行职责的，一律依法依规从严查处、决不姑息。

五、完善配套法规和制度，夯实依法保护基础

林业和草原主管部门要按照《决定》精神，对有关法规、规章体系进行一次全面梳理，对配套法规、制度不健全或与《决定》不相符的，积极争取立法机关、人民政府及早进行修改和完善。重点要加快《国家重点保护野生动物名录》、《国家保护的有重要生态、科学、社会价值的陆生野生动物名录》和地方重点保护野生动物名录的调整进度，积极推进《陆生野生动物保护实施条例》修订，抓紧制定野生动物人工繁育、专用标识等配套管理办法和技术标准。各省、自治区、直辖市林业和草原主管部门要积极争取地方立法机关及时研究修订地方保护法规，适时开展《野生动物保护法》执行情况调研，坚持以问题为导向，研究探讨解决问题的策略，为进一步修改完善法律法规提供科学依据。

六、周密部署安排，科学妥善处置后续事宜

在推进《决定》贯彻执行过程中，各级林业和草原主管部门要对可能面临的野生动物收容安置和一定补偿等问题预先进行研判，及时向当地人民政府报告。要依据物种习性和本地实际情况，科学制定工作方案，在当地人民政府的领导下组织实施，并对从业机构和人员做好解释说明，加强野生动物处置工作的指导和监督，确保科学稳妥实施。重点地区还要提前做好应急预案，及时化解隐患，严防出现违法出售野生动物用于食用等行为。

七、广泛开展宣传教育，引导全社会树立生态文明新风尚

各级林业和草原主管部门要多渠道、多形式、多层次加强野生动物保护宣传和公众教育，会同宣传、教育等有关部门，大力宣传出台《决定》的重大意义，普及野生动物保护知识和禁止食用野生动物的规定。通过举办专题宣传、专家讲解、进校园、进乡村等活动，科学解读滥食野生动物对生物安全、生态安全和公共卫生安全的危害，倡导绿色、健康、文明的饮食理念。健全社会监督机制，密切关注网络舆情动态，主动回应社会关切，广泛争取理解和支持。积极发挥各类野生动物保护社会团体、公益组织和广大志愿者的桥梁纽带作用，鼓励和引导社会公众自觉抵制、主动举报滥食野生动物行为，形成政府主导、全社会参与、共同革除滥食野生动物陋习的新局面。

贯彻落实《决定》的有关情况请及时上报我局。

特此通知。

国家林业和草原局

2020年2月27日

（174）发改委：做好疫情防控重点保障物资生产企业名单管理有关工作 发改办财金〔2020〕176号

国资委办公厅，各省、自治区、直辖市及计划单列市、新疆生产建设兵团发展改革委，有关中央企业：

根据《关于支持新型冠状病毒感染的肺炎疫情防控有关税收政策的公告》（财政部、税务总局2020年第8号，以下简称8号公告）要求，为做好疫情防控重点保障物资生产企业名单管理工作，现就有关事项通知如下。

一、纳入名单管理的企业可以享受的税收政策

（一）根据8号公告，对疫情防控重点保障物资生产企业为扩大产能新购置的相关设备，允许一次性计入当期成本费用在企业所得税税前扣除；疫情防控重点保障物资生产企业可以按月向主管税务机关申请全额退还增值税增量留抵税额，其中，增量留抵税额是指与2019年12月底相比新增加的期末留抵税额。

（二）企业生产的疫情防控重点保障物资范围，按照《国家发展改革委办公厅关于提供疫情防控重点保障物资具体范围的函》（发改办财金〔2020〕145号）执行。根据疫情防控需要，国家发展改革委可予以动态调整。

二、企业申报要求

申请纳入名单的疫情防控重点保障物资生产企业须符合以下条件：

（一）在疫情防控期间，从事第一条第二款规定的疫情防控重点保障物资生产，且合法合规经营的；

（二）对于从事多种经营的集团公司及其所属企业，只有集团内生产第一条第二款规定范围内产品的纳税人才能纳入名单；

（三）如需享受企业所得税税前扣除相关政策，须说明已扩大或拟扩大产能新购置设备等情况。

三、企业申报流程

（一）国家发展改革委负责确定符合条件的中央企业（含下属法人企业）名单。其中，属于国资委管理的中央企业由国资委统一审核后报送国家发展改革委，其他中央企业可直接向国家发展改革委申报（申请表见附件）。国家发展改革委审核汇总后报送财政部、税务总局。

（二）各省级发展改革部门负责审核确定省级及以下符合条件的企业名单。其中，属于省级国资委管理的国有企业由各省级国资委统一审核后报送各省级发展改革部门，其他企业按照属地管理原则依程序向省级发展改革部门申报。各省级发展改革部门审核汇总后报送同级财政、税务部门。

四、加强名单管理

（一）各级发展改革部门要严格加强名单管理，重点审核企业生产产品是否符合本通知支持范围，是否发挥疫情防控生产保障作用。一旦发现企业有弄虚作假、骗取税收优惠等违法违规行为，应及时调整出名单，通知同级财政、税务部门取消享受相关税收政策资格，并按照有关规定依法追究相应责任。

（二）各省级发展改革部门要加强与同级财政、税务、国资等部门工作衔接配合，及时了解疫情防控重点保障物资生产企业情况，加强对企服务，发现问题要及时协调解决，重大问题要及时上报国家发展改革委等有关部门。

五、有关工作要求

各省级发展改革部门要会同本级财政、税务部门，参照本通知相关要求，抓紧制定本地区名单管理办法，明确申报流程和具体要求，及时主动向社会公开。

本通知自印发之日起实施，截止日期视疫情情况另行通知。

附件：疫情防控重点保障物资生产企业名单（中央企业）申请表

国家发展改革委办公厅

2020年2月28日

（175）林草局：统筹推进新冠肺炎疫情防控和经济社会发展做好建设项目使用林地工作	林资规〔2020〕1号

各省、自治区、直辖市林业和草原主管部门，内蒙古、大兴安岭森工（林业）集团公司，新疆生产建设兵团林业和草原局：

为深入贯彻落实党中央统筹推进新冠肺炎疫情防控和经济社会发展工作部署，做好建设项目使用林地支持保障工作，现将有关事项通知如下：

一、确保疫情防控建设项目使用林地

因疫情防控急需使用林地的建设项目，可以根据需要先行使用林地，并在疫情结束后6个月内补办使用林地审核手续。属于临时占用林地的，疫情结束后应当恢复林业生产条件，交还原林地使用者，不再办理使用林地审批手续。

二、支持重点建设项目使用林地

为支持经济社会发展，确保国家、省（自治区、直辖市）重点建设项目顺利实施，对列入国家和省（自治区、直辖市）重点建设项目目录的公路、铁路等基础设施项目、民生项目，因疫情防控原因无法及时办理使用林地手续的，允许先行使用林地，项目在开工前应当向省级林业和草原主管部门报备。

三、保障脱贫攻坚项目使用林地

2020年是全面建成小康社会和“十三五”规划收官之年，为确保脱贫目标如期实现，对脱贫攻坚项目，可以根据需要先行使用林地。其中，因脱贫攻坚需要，在国家级自然保护区内实施的道路、桥梁维修改造项目，不新增占地的，不需要办理在国家级自然保护区修筑设施的行政许可，自然保护区管理机构应当加强指导和监督。

四、做好服务保障和监管工作

各级林业和草原主管部门要对疫情防控建设项目、重点建设项目、脱贫攻坚项目加强监管，坚持集约节约使用林地，严禁违规搭车。重点建设项目、脱贫攻坚项目，应当在开工后6个月内及时办理使用林地审核审批手续。

各级林业和草原主管部门要对上述项目靠前服务，及时解决问题，加快项目办理进度，切实保障林地定额，省级林地定额不足的，可以申请国家备用定额。疫情防控期间，在国家级自然保护区内建设的项目，我局不组织专家进行现场考察和评审。

本通知自发布之日起施行，有效期至2020年12月31日。

特此通知。

国家林业和草原局

2020年2月28日

（176）银保监会：加强银行业保险业金融服务配合做好新型冠状病毒感染的肺炎疫情防控工作	银保监办发〔2020〕10号

各银保监局，各政策性银行、大型银行、股份制银行，外资银行，金融资产管理公司，各保险集团（控股）公司、保险公司、保险资产管理公司，各会管单位：

为坚决贯彻落实党中央、国务院决策部署，全力配合做好新型冠状病毒感染的肺炎疫情防控工作，现就加强银行业保险业金融服务有关事项通知如下：

一、落实疫情防控要求。各银行保险机构要全力协助有关部门和地方政府做好新型冠状病毒感染的肺炎疫情防控，坚决落实各项工作要求。加大员工疫情排查力度，做好员工防疫安排。定期对柜台、自助服务机具等设施进行清洁消毒，强化营业网点、办公场所的卫生防疫管理。适当调整工作计划和考核要求，减少人员聚集和客户集中拜访。

二、保障金融服务顺畅。各银行保险机构要根据疫情防控工作需要，合理安排营业网点及时间，保障基本金融服务和关键基础设施稳定运行。对受疫情影响临时停业或调整营业时间的网点，要主动做好解释说明，提供替代解决方案。鼓励积极运用技术手段，在全国范围特别是疫情较为严重的地区，加强线上业务服务，提升服务便捷性和可得性。

三、开辟金融服务绿色通道。各银行保险机构要进一步加大对疫区的支持，减免手续费，简化业务流程，开辟快速通道。要充分发挥银行信贷、保险保障、融资担保等多方合力，加强对社会民生重点领域金融支持。对受疫情影响暂时失去收入来源的人群，要在信贷政策上予以适当倾斜，灵活调整住房按揭、信用卡等个人信贷还款安排，合理延后还款期限。对感染新型冠状病毒的出险理赔客户要优先处理，适当扩展责任范围，应赔尽赔。

四、强化疫情防控金融支持。各银行保险机构要主动加强与有关医院、医疗科研单位及企业的服务对接，积极满足卫生防疫、医药产品制造及采购、公共卫生基础设施建设、科研攻关等方面的合理融资需求。要按照特事特办、急事急办原则，切实提高业务办理效率，提供优质高效的疫情防控综合金融服务。鼓励向疫情防控一线的相关单位和工作者提供更加优惠的金融服务。不得借疫情渲染炒作金融产品。

五、做好受困企业金融服务。各银行保险机构要通过调整区域融资政策、内部资金转移定价、绩效考核办法等措施，提升受疫情影响严重地区的金融供给能力。对于受疫情影响较大的批发零售、住宿餐饮、物流运输、文化旅游等行业，以及有发展前景但暂时受困的企业，不得盲目抽贷、断贷、压贷。鼓励通过适当下调贷款利率、完善续贷政策安排、增加信用贷款和中长期贷款等方式，支持相关企业战胜疫情灾害影响。

2020年1月26日

（177）商务部：积极应对新冠肺炎疫情加强外资企业服务和招商引资工作

各省、自治区、直辖市、计划单列市及新疆生产建设兵团商务主管部门：

为贯彻落实党中央、国务院关于做好新型冠状病毒感染肺炎疫情防控工作的决策部署，在切实抓好疫情防控工作的前提下，千方百计做好为外资企业服务和招商引资工作，现就有关事项通知如下：

（一）积极帮助外资企业有序恢复正常生产经营。在做好防控工作的前提下，按照当地政府统一安排，协助做好复工前各项准备工作，大力支持外资企业有序复工复产。对于生产医用防护服、口罩、护目镜等防护用品类外资企业，要按照统一部署特事特办，迅速组织复工达产、全力满足需求。积极协助企业采购必要防护物资；指导企业结合实际情况，编制疫情应对工作方案，严格做好各项内部防控工作。深入了解企业用工需求，充分利用网络平台等方式促进劳动力供需对接，加大企业复产用工保障力度。协调帮助解决外资企业复产存在的困难，妥善处理外资企业投诉。切实关心企业在华工作的外籍员工及家属的生活，帮助做好防护工作。

（二）加强外资大项目服务保障。密切跟踪在谈外资大项目进展，及时了解外商决策进展，加大沟通力度，积极促成投资合作，推动项目尽早签约落地。推动在建外资大项目建设，优化网上政务服务，建立政务服务快速通道，协调相关部门在用地、用工、水电等方面加大保障力度，保障企业投资按计划进行，把疫情影响降到最低。建立健全重点外资大项目联系机制，不添乱、多帮忙，及时掌握项目进展情况，开展全流程对接服务，积极协调解决项目建设中遇到的突出困难。

（三）创新和优化招商引资方式。针对当前人员流动受阻等难题，发挥互联网平台优势，整合各类招商资源，积极通过网上洽谈、视频会议、在线签约等方式推进网上招商，持续不断推进投资促进和招商工作。进一步完善招商引资储备项目库，筛选一批有吸引力、有发展潜力的项目上网发布，与外商做好前期对接。充分利用国家和各地海外招商机构平台，加大投资环境和合作项目宣传推介力度；加强与境外各类商协会等中介组织合作，积极开展委托招商、以商招商，组织灵活多样的招商活动，争取一批新项目签约落地。

（四）因地制宜精准帮扶。与各地市场监管部门加强沟通协调，落实好外商投资信息报告制度，确保信息报告及时、真实、准确、完整。优化和加强外资统计工作；充分运用信息化手段，加强研判和预警，及时掌握各行业、各领域外资企业受疫情影响情况；强化与各地外资企业协会和外国商会的联系机制，及时了解会员企业生产经营状况和投资动向，有针对性地加强服务和指导。聚焦疫情对各类外资企业的不同影响，分类施策，一企一策，会同相关部门采取精准帮扶措施，帮助企业渡过难关。

（五）持续优化营商环境。全面落实《外商投资法》及其实施条例和《国务院关于进一步做好利用外资工作的意见》（国发〔2019〕23号）等法规政策，加强宣传解读，营造更加稳定公平透明、可预期的投资环境。梳理国家和各地近期出台的各类援企稳岗政策，指导外资企业用好用足财政、金融、税收、社保、就业以及政府采购等各类帮扶解困政策，做到内外资企业平等对待、一视同仁，最大限度减少疫情影响。各地要尽快编制和公布外商投资指引，发布办事指南、项目信息和相关数据信息等，为外商投资提供服务和便利，不断优化营商环境。

各地商务主管部门要统一思想，提高站位，建立健全疫情应对工作机制，加强组织领导，明确责任分工，创新工作方法，真正把精力用到协调解决外资领域应对疫情遇到的紧迫问题上；加强横向协作、纵向联动，高度重视协调配合和信息共享，疫情防控期间要及时报送稳外资工作情况，在疫情应对、外资大项目跟踪服务、外资企业经营动态、统计信息、形势研判等方面保持密切沟通。对外资企业当前复工或经营中的困难进行摸底，整理分析后与其它有关情况一并反馈商务部（外资司）。

商务部办公厅

2020年2月7日

（178）药监局：加快医用防护服注册审批和生产许可	药监综械管函﹝2020﹞71号

各省、自治区、直辖市药品监督管理局，新疆生产建设兵团药品监督管理局：

根据国务院联防联控工作机制物资保障组有关工作安排，为进一步提升医用防护服产能，切实做好新型冠状病毒疫情的防控工作，现就加快医用防护服注册审批和生产许可事项通知如下：

一、支持和鼓励医用防护服生产企业扩大产能。对医用防护服生产企业新增生产场地的，所在地省级药品监管部门按照《医疗器械注册管理办法》第四十九条和《医疗器械生产监督管理办法》第十五条的规定，快速办理。

二、支持和鼓励其他生产企业转产医用防护服。对生产出口符合美国、欧盟、日本等国家（地区）相关标准的防护服企业和有一定生产能力的工业防护服生产企业，通过完善生产条件，健全生产质量管理体系，其产品能够符合《医用一次性防护服技术要求》（GB19082-2009），所在地省级药品监管部门按照《医疗器械注册管理办法》第五章和《医疗器械生产监督管理办法》第八条的规定，加快办理产品注册和生产许可。

三、优化医用防护服产品注册和生产许可程序。各地在办理医用防护服注册申请时，可参照《关于印发医疗器械应急审批程序的通知》（国食药监械﹝2009﹞565号），开展应急审批。

办理医用防护服生产许可时，生产许可现场检查可与注册现场核查合并进行，根据医疗器械生产质量管理规范相关要求检查，重点关注人员和设施管理、生产过程控制、成品检验和出厂放行等环节。

对符合条件的，同步发放产品注册证和生产许可证。产品注册证和生产许可证有效期原则上均不超过一年。

四、深入企业服务指导。各省级药品监管部门应当根据当地企业的实际情况，派员深入企业加强指导，帮助企业尽快达到相关要求。同时，要切实加强产品质量安全监管，监督企业持续符合法规标准要求。

以上措施属于此次疫情防控期间的临时应急措施，疫情结束后自行解除。

国家药监局综合司

2020年2月7日

（179）国务院联防联控机制：进一步指导各地落实分区分级差异化防控策略

2月28日，国务院联防联控机制印发《关于进一步落实分区分级差异化防控策略的通知》（以下简称《通知》），就进一步指导各地准确分析把握疫情和经济社会发展形势，落实分区分级差异化防控策略，统筹推进疫情防控和经济社会发展工作，作出部署安排。

湖北省和武汉市要全面加强疫情防控，继续实施“严格管控、外防输出、内防扩散”策略，紧紧扭住城乡社区防控和患者救治两个关键，继续实行严格的离汉离鄂通道管控措施，坚决遏制疫情扩散输出。加强力量薄弱地区防控，坚决做到应检尽检、应收尽收、应治尽治，切实提高收治率和治愈率、降低感染率和病亡率。

北京市要按照“外防输入、内防扩散”策略，全力做好疫情防控工作。要充分发挥严格进京管理协调机制作用，加强京津冀地区联防联控，尽最大可能切断传染源。广泛动员各方面力量参与协同防控，全面落实疫情防控期间严格进京管理若干措施，引导外地人员逐步有序返京。完善联防联控体系，强化社区防控网格化管理，坚决切断输入型传染源，全力遏制疫情扩散，确保首都安全稳定。

湖北省周边省份及浙江省、广东省、黑龙江省等重点省份要按照“外防输入、内防扩散和局部暴发、分类指导”策略，坚决遏制疫情上升势头，阻断疫情蔓延。湖北省周边省份要主动与湖北省建立省际联防联控机制，既要防止湖北省疫情向周边省份蔓延，也要做好对湖北省物资、人员援助及交通保障。

全国其他地区要以县域为单位，实施“预防为主、外防输入、内防局部扩散”策略，继续落实“四早”措施，精准检测、追踪到人、随访到户，确保重点对象登记在册，异常情况及时处置，防止疫情反弹。疫情风险较低的地方要在有防护保障情况下尽快复工复产，恢复人民群众正常生活，努力促进经济社会健康发展。其他地方要结合疫情防控分区分级有序推进复工复产。

《通知》强调，各地区要根据当前疫情防控形势和要求，突出重点、统筹兼顾、分类指导、分区施策，进一步细化实化防控措施，加强重点人群、重点场所、重点单位管控，落实各项防控技术方案。要充分发挥基层作为联防联控、群防群治关键防线作用，加强对社区防控工作的组织领导，强化基层防控人员和物资保障，夯实社区排查和防控基础，推动科学精准的防控手段向城乡社区继续下沉，健康防护知识向公众广泛普及，不断提高疫情防控的科学性、精准性、针对性。（新华社北京2月29日电）

（180）国务院应对新型冠状病毒感染肺炎疫情联防联控机制：进一步做好民政服务机构疫情防控工作	国发明电〔2020〕6号

各省、自治区、直辖市人民政府，国务院各部委、各直属机构：

为进一步做好养老机构（含公办、民办）、儿童福利机构、未成年人救助保护机构、精神卫生福利机构、流浪乞讨人员救助管理机构（含托养机构）和殡葬服务机构等民政服务机构新冠肺炎疫情防控和服务保障工作，根据中央应对疫情工作领导小组部署，现提出以下要求：

一、严防民政服务机构发生聚集性感染

各地民政部门要切实履行主管职责，督促指导各类民政服务机构严格按照《关于依法科学精准做好新冠肺炎疫情防控工作的通知》要求，重点防控输入性疫情和内部疾病传播，坚决切断传染源，阻断传播途径。

强化出入管理。湖北省和武汉市及其他地区发生了疫情感染的养老机构要坚决执行民政部印发的《新冠肺炎疫情高风险地区及被感染养老机构防控指南》，落实当地防控工作部署，按照封闭管理要求，严格管控服务对象和工作人员进出；其他地区未发生疫情感染的养老机构，要继续落实好《养老机构新型冠状病毒感染的肺炎疫情防控指南（第二版）》要求。儿童福利机构、未成年人救助保护机构要按照防控工作要求，妥善安排收住人员生活，切实履行监护职责，待疫情结束后，再有序开展安置等相关工作。精神卫生福利机构要严格落实各项防控措施，继续做好特困人员、流浪乞讨人员中的精神障碍患者及公安部门移交的肇事肇祸精神障碍患者的精神康复服务，按照当地疫情防控统一部署，有序开展精神障碍社区康复等服务。流浪乞讨人员救助管理机构要按照“应救尽救”要求，对于有临时住宿需求的求助人员，及时收住并安排在独立区域隔离观察不少于14天；对于观察期未满要求离站的受助人员，要及时安排核酸检测，确定其未感染后按规定办理离站。各地对于救助管理机构收住能力已饱和、不能满足求助人员临时住宿需求的，要开辟临时庇护场所，提供临时救助。殡葬服务机构在保障遗体接运、火化和骨灰暂存等基本殡葬服务外，可暂停开展守灵、告别、祭奠等人员聚集活动；对于新冠肺炎患者遗体，要按照《中华人民共和国传染病防治法》规定，及时进行卫生处理，就近安排火化。取消民政服务机构内人员集中用餐和其他各类人员聚集活动。加强机构内服务对象心理疏导，开展协助拨打亲情电话等精神慰藉活动。

强化人员配置。各地要尽快增派医疗和护理力量，统筹解决民政服务机构防疫力量薄弱、人手不足等问题。民政服务机构受疫情影响工作人员严重紧缺的，民政、人力资源社会保障部门要加大招录力度，确保民政服务机构正常运转。返岗和新招录的工作人员应当隔离观察至少14天，身体状况无异常的方可安排上岗。民政服务机构要保护关心爱护工作人员，做好防护保障，合理安排轮休，保障他们的身心健康。

强化物资保障。湖北省和武汉市以及疫情严重地区，要按照三级防护标准配备、下发防控物资，确保民政服务机构每日防护需要；其他地区要在保障日常防护需要的同时，按照不少于三天需求量做好储备，确保应急使用。各地要采取统一配送方式，保障民政服务机构所需米面油、肉蛋菜等生活物资供应，将民政服务机构纳入社会捐赠物资调配范围。要区分民政服务机构性质，根据防控工作需要，通过财政资金、社会捐赠资金或机构自筹等方式，按现行经费渠道统筹解决疫情防控所需资金。

强化防护能力。民政服务机构要通过手机信息、宣传栏、板报、广播等多种形式，深入宣传疫情防护知识。按照科学预防、积极防治的要求，组织工作人员和服务对象勤洗手、戴口罩，做好个人卫生防护，做好定期监测体温和消毒、通风等工作，提高防护能力。

各地不得将收住有服务对象的民政服务机构征用为疑似病例隔离点。各地医养结合机构在疫情期间不得擅自对外开展发热病人诊疗排查活动。

二、坚决落实“四早”要求及时收治感染患者

坚决落实“早发现、早报告、早隔离、早治疗”要求，做到应检尽检、应收尽收、应治尽治，切实保障老年人、儿童、精神障碍患者、流浪乞讨人员等特殊人群的生命安全和身体健康。

早发现。湖北省和武汉市以及疫情严重地区，要及时对民政服务机构收住的服务对象和工作人员进行全员核酸检测排查，确保不漏一人。卫生健康部门要派出流动医疗小组到民政服务机构上门开展医学排查和治疗指导。疾控机构要指导民政服务机构做好消毒和密切接触者追踪管理等工作。

早报告。各地要落实疫情监测报告责任，安排专人负责，加强应急值守，确保民政服务机构疫情监测报告及时、准确、到位，做好疫情应急响应和处置。

早隔离。对于民政服务机构内出现的疑似病例，各地要及时转运到隔离点进行医学观察，最大限度缩短疑似病例收治时间。对于已发现确诊或疑似病例且密切接触服务对象较多的民政服务机构，各地要根据实际需要，按照集中隔离点的防控标准，配备必要的医务人员和设备，纳入辖区集中隔离点范围进行规范管理。

早治疗。各地对于民政服务机构内出现的确诊患者和疑似病例，要及时转运到定点收治医院、隔离点或轻症治疗点接受治疗或观察。要组织医疗卫生机构到民政服务机构开展上门巡诊，避免收住的服务对象因外出就医造成感染。

三、做好特殊群体兜底保障

各地对于受疫情影响在家隔离的孤寡老人、因家人被隔离收治而无人照料的老年人和未成年人，以及社会散居孤儿、留守儿童、留守老年人等特殊群体，要组织开展走访探视，及时提供帮助，防止发生冲击社会道德底线的事件。各地要建立社区干部联系帮扶一线医务人员家庭制度，帮助解决老幼照护等实际困难，解除一线医务人员后顾之忧。

各地要强化落实属地责任，按照本通知要求，加强统一领导和督促指导，将民政服务机构的疫情排查、转移隔离、集中救治、物资保障等作为防控工作重点，确保各项政策措施落实到位，保障好民政服务对象的生活和健康。

国务院应对新型冠状病毒感染

肺炎疫情联防联控机制

2020年2月28日

（181）国务院应对新型冠状病毒肺炎疫情联防联控机制：进一步规范和加强新冠肺炎流行期间消毒工作	联防联控机制综发〔2020〕89号

各省、自治区、直辖市及新疆生产建设兵团应对新冠肺炎疫情联防联控机制（领导小组、指挥部）：

为指导各地科学消毒、精准消毒，打好疫情防控阻击战，现就进一步规范和加强新冠肺炎流行期间的消毒工作提出如下要求：

一、依法依规开展消毒工作

各地要以《中华人民共和国传染病防治法》为依据，按照《新型冠状病毒肺炎防控方案（第五版）》、《疫源地消毒总则》（GB19193-2015）、《疫源地消毒剂卫生要求》（GB27953-2011）等方案和标准开展医疗机构、病例居住过的场所、转运车辆等特定场所的消毒。工作场所、公共场所、社区和学校等场所的预防性消毒方法参照《关于依法科学精准做好新冠肺炎疫情防控工作的通知》（联防联控机制发〔2020〕28号）和《消毒剂使用指南》（国卫办监督函〔2020〕147号）进行。

二、采取科学消毒措施

各地要精准施策、科学消毒，真正做到切断传播途径，控制传染病流行。无明确传染源时，做好预防性消毒，增加医院、机场、车站等人员密集场所的物体表面消毒频次，加强高频接触的门把手、电梯按钮等清洁消毒，做好垃圾、粪便和污水的收集和无害化处理，做好个人手卫生。有明确传染源时，加强隔离病区、病例居住过的场所和转运车辆等的随时消毒和终末消毒。

三、防止过度消毒

各地要防止过度消毒，不对室外环境开展大规模的消毒，雨雪天气不开展外环境消毒；不对外环境进行空气消毒；不直接使用消毒剂（粉）对人员全身进行喷洒消毒；不对水塘、水库、人工湖等环境中投加消毒剂（粉）；不在有人条件下对室内空气使用化学消毒剂消毒。

四、做好质量控制和效果评价

各地要确保消毒效果，做好消毒质量控制。所用消毒产品要合法有效，并严格遵循产品说明书使用。要根据消毒对象的特点，选择可靠的消毒方法及消毒剂量，采取必要的检测手段，确保消毒效果。对消毒范围广、持续时间长的预防性消毒和影响大的终末消毒，各级疾控部门要做好消毒效果评价。

国务院应对新型冠状病毒肺炎

疫情联防联控机制综合组

（代  章）

2020年2月29日

（182）科技部：做好国家高新区科学防疫推动企业有序复工复产

各国家高新区管委会：

为深入贯彻落实习近平总书记在统筹推进新冠肺炎疫情防控和经济社会发展工作部署会议上的重要讲话精神，按照中央应对疫情工作领导小组部署和国务院联防联控机制工作要求，在切实做好疫情防控的前提下，有序推动国家高新区企业复工复产，现将有关要求通知如下。

一、落实主体责任，坚决把党中央决策部署抓实抓细抓落地。国家高新区是我国实施创新驱动发展战略的核心载体，是实现经济高质量发展的前沿阵地。各国家高新区管委会要增强“四个意识”、坚定“四个自信”、做到“两个维护”，切实提高政治站位，充分认识做好疫情防控和复工复产的重要性和紧迫性。要在地方党委和政府的统一领导下，发挥主体责任作用，严格贯彻落实国务院联防联控机制印发的《企事业单位复工复产疫情防控措施指南》，根据本地疫情防控要求和园区企业实际情况，制定园区复工复产疫情防控实施方案，明确具体措施、健全工作机制、压实落实责任，努力实现疫情防控和经济社会发展两手抓、两不误。

二、发挥科技优势，为企业疫情防控和复工复产提供精准支撑。针对不同产业、不同类型企业实际需求，组织推荐一批疫情防控的新技术新成果，开展精准对接和转化应用。支持和引导制造业企业系统云化部署，采用云制造平台、云服务平台等工业互联网产品、解决方案和工具包。鼓励服务业企业通过线上服务、使用智能机器人等手段，实现无接触服务。采取“复工复产通用管理系统”“健康二维码”等疫情防控科技手段，做好返岗复工人员精准健康监测。支持有条件的企业充分利用网上办公、视频会议、远程协作和数字化管理等方式，营造“少见面”“少接触”的办公环境，着力降低企业复工复产带来的聚集性传染风险。

三、主动对接需求，为企业有序复工复产提供细致服务。要加强政策宣传、解读，强化政策落地，使企业充分享受到国家支持企业发展的财税、金融、社保等有关政策。要主动询问企业困难，对接企业需求，做好统筹协调，着力解决好企业复工复产过程中可能出现的员工返岗难、企业融资难、供应链协同难、物流配送难、防疫物资不足等关键制约问题。要强化系统观念，逐个环节打通产业链、供应链“堵点”，推动产业链各环节协同复工复产。

四、加大对科技型中小企业的支持，帮助企业渡难关强后劲。要制定政策和措施，采取多种方式，加强对科技型中小企业研究开发活动的支持，提升企业创新能力，增强企业发展后劲。要引导和动员科技人员进入企业，开展技术咨询、成果转化、人才培养等创新服务，帮助企业特别是科技型中小企业破解发展难题，增强核心竞争力。要引导社会资本加大投入，不断壮大天使投资、创业投资规模，引导银行等金融机构加大对科技型中小企业支持力度，缓解科技型中小企业融资难融资贵问题。

五、支持双创载体建设，提升专业化服务能力。鼓励科技企业孵化器、专业化众创空间、技术转移机构、检验检测平台等服务载体搭建在线服务平台，强化创新资源共享，为企业特别是科技型中小企业提供全周期全链条服务。利用创新券、政府购买服务等多种方式，对各类双创服务载体进行奖励或后补助支持。鼓励有条件的科技企业孵化器、众创空间、大学科技园等对在孵企业适当减免或缓征租金等，支持初创小微企业有序复工复产、健康发展。

六、培育壮大新业态新模式，推动园区产业升级。加快科技成果转化应用及产业化，支持企业围绕数字化、智能化等方向，加快重组产业领域和要素资源，加速推进人工智能、大数据与传统产业深度融合，推动产业价值链的延伸或突破。加快在线消费、医疗健康、互联网教育等领域新技术、新产品、新服务的商业化和落地实施。要适应新经济、新业态的制度变革需求，积极研究出台相应的新经济行业规则，提供以场景创新、包容审慎监管为核心的新经济制度环境。

七、进一步提升科技治理水平，为企业提供便利化服务。要充分利用移动互联网、5G、云计算等数字化手段，积极推进数字政务服务和数字治理服务，实现线上办公、线上招商、线上招投标等。要优化管理方式，简化优化办事流程，合理安排科技计划申报、科技型中小企业评价、高新技术企业认定、技术合同登记、外国人来华工作许可办理等工作，倡导实行网上受理。疫情比较严重或严重地区的国家高新区，可延长高新技术企业认定和科技型中小企业评价时间。因疫情受影响的企业，在2020年6月30日前，不能正常提交高新技术企业认定和科技型中小企业评价有关材料的，不作逾期处理。

八、建立“一站式”信息服务平台，加强沟通和服务力度。科技部建立服务于高新区疫情防控和复工复产的信息平台（网址：https://gxq.chinatorch.org.cn），及时发布各类支持园区和企业复工复产的政策信息和疫情防控政策、规范指南，为园区提供各类支持企业复工复产和疫情防控的技术工具，畅通科技部与各国家高新区及国家高新区之间的信息沟通与交流，分享复工抗疫作法经验，共享各类技术、产品和工具，促进国家高新区之间互帮互助，共同做好企业复工复产和疫情防控工作。

有关疫情防控和企业复工复产情况、存在的问题和需求，以及实践中探索的好经验、好做法，请及时报送科技部成果转化与区域创新司。

科技部办公厅

2020年2月28日

（183）市场监管总局等6部门：应对疫情影响 加大对个体工商户扶持力度	国市监注〔2020〕38号

各省、自治区、直辖市人民政府，新疆生产建设兵团：

个体工商户在繁荣市场经济、扩大社会就业、方便群众生活、维护社会和谐稳定等方面发挥着重要作用。为认真贯彻落实习近平总书记关于统筹推进新冠肺炎疫情防控和经济社会发展工作的重要指示精神，按照党中央、国务院决策部署，帮助个体工商户应对疫情影响、尽快有序复工复产、稳定扩大就业，经国务院同意，现提出如下指导意见：

一、帮助个体工商户尽快有序复工复产

（一）分类有序推动复工复产。各地要严格落实分区分级精准复工复产要求，分业态分形式有序推动个体工商户复工复产。对于实体批发零售类、餐饮类、居民服务类、交通运输类等涉及群众基本生活保障行业的个体工商户，要结合本地疫情防控实际，有序解除复工复产禁止性规定。涉及人员聚集的文化娱乐、教育培训等行业，应结合实际，适时明确复工复产时间。符合各地复工复产规定的个体工商户，无需批准即可依法依规开展经营活动。

（二）保障用工和物流需求。各地要认真落实国务院应对新型冠状病毒感染肺炎疫情联防联控机制印发的《企事业单位复工复产疫情防控措施指南》，保证符合复工复产防疫安全标准规定的人员及时上岗。要采取措施，尽快完善灵活就业政策，促进快递等行业尽快复工复产，稳定快递末端网点，保障物流畅通。要发挥电子商务类平台企业作用，为线上线下个体工商户特别是生鲜类经营者提供供需对接信息资源服务。

二、降低个体工商户经营成本

（三）加大资金支持力度。各地要加强与金融机构的对接，对受疫情影响严重、到期还款困难以及暂时失去收入来源的个体工商户，灵活调整还款安排，合理延长贷款期限，不得盲目抽贷、断贷、压贷。引导金融机构增加3000亿元低息贷款，定向支持个体工商户。

（四）减免社保费用。有雇工的个体工商户以单位方式参加企业职工养老保险、失业保险、工伤保险的，参照《人力资源社会保障部 财政部 税务总局关于阶段性减免企业社会保险费的通知》（人社部发〔2020〕11号）中的企业办法享受单位缴费减免和缓缴政策。个体工商户以个人身份自愿参加企业职工基本养老保险或居民养老保险的，可在年内按规定自主选择缴费基数（档次）和缴费时间。对受疫情影响无法按时办理参保登记的个体工商户，允许其在疫情结束后补办登记，不影响参保人员待遇。

（五）实行税费减免。在继续执行公共交通运输服务、生活服务以及为居民提供必需生活物资快递收派服务收入免征增值税政策的同时，自2020年3月1日至5月31日，免征湖北省境内增值税小规模纳税人（含个体工商户和小微企业，下同）增值税，其他地区小规模纳税人征收率由3%降为1%。对疫情期间为个体工商户减免租金的大型商务楼宇、商场、市场和产业园区等出租方，当年缴纳房产税、城镇土地使用税确有困难的，可申请困难减免。政府机关所属事业单位、国有企业法人性质的产品质量检验检测机构、认证认可机构，减免个体工商户疫情期间的相关检验检测和认证认可费用。

（六）减免个体工商户房租。对承租行政事业单位房屋资产、政府创办创业园、孵化园、商品交易市场、创业基地和国有企业出租的经营用房的个体工商户，鼓励各地结合实际情况进行租金减免。承租其他经营用房或摊位的，各地可以结合实际出台相关优惠、奖励和补贴政策，鼓励业主为租户减免租金。

三、方便个体工商户进入市场

（七）为个体工商户提供便捷高效的服务。全面推广个体工商户全程网上办理登记服务，简化登记流程。对于从事餐饮、零售等行业的个体工商户，要做好营业执照登记与许可审批的衔接，帮助经营者尽快开展经营活动。个体工商户可将年报时间延长至2020年年底前。

（八）进一步释放经营场所资源。各地要统筹考虑城乡综合管理需要和个体工商户创业就业的现实需求，尽快建立完善个体工商户经营场所负面清单管理制度，及时向社会公布本地区禁止登记经营的场所区域和限制性条件清单。

（九）依法对个体经营者豁免登记。对销售农副产品、日常生活用品或者个人利用自己的技能从事依法无须取得许可的便民劳务活动的个体经营者，特别是在疫情期间从事群众基本生活保障的零售业个体经营者，各地要进一步拓宽其活动的场所和时间，依法予以豁免登记。

四、加大对个体工商户的服务力度

（十）保障个体工商户电气供应。2020年上半年，对受疫情影响无力足额缴纳电、气费用的个体工商户，实行“欠费不停供”措施。商贸流通、餐饮食品、旅游住宿、交通运输等行业个体工商户用电、用气价格按照相关部门出台的阶段性降低用电、用气成本的政策执行。

（十一）发挥工商联以及个体劳动者协会等社团组织作用。充分发挥工商联以及个体劳动者协会等各类社团组织的桥梁纽带作用，通过开展维权保障、宣传教育、培训学习、经贸交流、困难帮扶、公益活动等举措为个体工商户提供服务、排忧解难。

（十二）鼓励互联网平台发挥作用。鼓励互联网平台对个体工商户放宽入驻条件、降低平台服务费、支持线上经营。帮助个体工商户运用移动支付、应用软件等服务，拓展运营新模式。发挥平台机构信用信息优势作用，联合互联网银行、中小银行，帮助个体工商户拓展融资渠道，提供定期免息或低息贷款。地方政府可对帮扶效果好的电子商务类平台企业予以财政资金支持。

市场监管总局 发展改革委 财政部

人力资源社会保障部 商务部 人民银行

2020年2月28日

（184）银保监会等5部门：对中小微企业贷款实施临时性延期还本付息	银保监发〔2020〕6号

各银保监局，中国人民银行上海总部，各分行、营业管理部，各省会（首府）城市中心支行，各副省级城市中心支行，各省（自治区、直辖市及计划单列市）发展改革委，新疆生产建设兵团发展改革委，各省（自治区、直辖市及计划单列市）中小企业主管部门，新疆生产建设兵团中小企业主管部门，各省（自治区、直辖市）财政厅（局），新疆生产建设兵团财政局，各政策性银行、大型银行、股份制银行，外资银行：

为深入贯彻落实党中央、国务院关于新冠肺炎疫情防控和应对工作的决策部署，进一步纾解中小微企业困难，推动企业有序复工复产，提高金融服务的针对性、有效性，有关部门作了深入研究，确定按照市场化、法治化原则，对符合条件、流动性遇到暂时困难的中小微企业贷款，给予临时性延期还本付息安排。经国务院同意，现就有关事宜通知如下：

一、关于贷款到期本金安排

对于2020年1月25日以来到期的困难中小微企业（含小微企业主、个体工商户）贷款本金，银行业金融机构应根据企业延期还本申请，结合企业受疫情影响情况和经营状况，通过贷款展期、续贷等方式，给予企业一定期限的临时性延期还本安排。还本日期最长可延至2020年6月30日。对于少数受疫情影响严重、恢复周期较长且发展前景良好的中小微企业，银行业金融机构可根据实际情况与企业协商确定另外的延期安排。上述贷款涉及担保的，银行业金融机构应与企业、担保人等协商处理。

二、关于贷款利息支付安排

对于2020年1月25日至6月30日中小微企业需支付的贷款利息，银行业金融机构应根据企业延期付息申请，结合其受疫情影响的实际情况，给予企业一定期限的延期付息安排。贷款付息日期最长可延至2020年6月30日，免收罚息。延期利息的具体偿还计划，由银行业金融机构与企业双方自主协商、合理确定。

三、关于湖北地区特殊安排

湖北地区各类企业适用上述政策。银行业金融机构应为湖北地区配备专项信贷规模，实施内部资金转移定价优惠，力争2020年普惠型小微企业综合融资成本较上年平均水平降低1个百分点以上。

四、关于企业新增融资安排

银行业金融机构应积极对接中小微企业融资需求，建立绿色通道，简化贷款审批流程，适度下放审批权限，应贷尽贷快贷。要改进绩效考评、尽职免责等内部资源配置和政策安排，努力提高小微企业信用贷款、中长期贷款占比和“首贷率”。地方法人银行应主动申请人民银行的新增再贷款、再贴现额度，积极配合政策性银行的新增信贷计划，以优惠利率向民营、中小微企业发放贷款。

五、落实要求

做好需求对接。银行业金融机构应积极对接企业贷款延期还本付息需求，开通线下和线上（网上银行、手机银行、电话银行）等多种渠道，为企业延期还本付息申请提供便利。要及时受理企业申请，限时回复办理。

明确支持重点。银行业金融机构应合理评估企业状况，调整完善企业还本付息安排，重点支持前期经营正常、受疫情影响遇到暂时困难、发展前景良好的中小微企业。对于受疫情影响特别严重、遇到特殊困难的行业，例如交通运输、批发零售、文化娱乐、住宿餐饮等，银行业金融机构应根据实际情况，给予适当倾斜。

有效防范风险。银行业金融机构应对临时性延期还本付息贷款进行专门统计、密切监测，对于贷款期间企业经营出现实质性变化的，及时予以相应处置。同时，应完善反欺诈模型运用，推进信息共享联防。一旦发现弄虚作假等违法违规行为，应立即停止融资支持，并通过上报征信、诉讼等惩戒措施，有效防控道德风险。

六、有关配套政策

对于上述临时性延期还本付息的贷款，银行业金融机构应坚持实质性风险判断，不因疫情因素下调贷款风险分类，不影响企业征信记录。

人民银行综合运用公开市场操作、中期借贷便利、常备借贷便利、再贷款、再贴现等货币政策工具，保持银行体系流动性合理充裕，维持银行负债端平稳接续。

各级财政部门对于国有控股和参股的银行业金融机构2020年的经营考核，应充分考虑应对疫情、服务中小微企业的特殊因素，给予合理评价。

银保监会 人民银行

发展改革委 工业和信息化部 财政部

2020年3月1日

（185）国务院应对新型冠状病毒肺炎疫情联防联控机制：切实做好新冠肺炎聚集性疫情防控工作	联防联控机制综发﹝2020﹞81号

各省、自治区、直辖市及新疆生产建设兵团应对新型冠状病毒肺炎疫情联防联控机制（领导小组、指挥部）：

　　目前，全国疫情防控形势积极向好的态势正在拓展，企事业单位逐步复工复产，但是在复工复产过程中出现了一些聚集性疫情，严重影响了疫情防控和经济社会发展。为贯彻落实习近平总书记在统筹做好疫情防控和经济社会发展工作部署会议上的重要讲话精神，现就做好聚集性疫情防控工作紧急通知如下：

　　一、落实分区分级精准防控，压实各方责任

　　各地要坚决贯彻落实习近平总书记重要指示精神和中央决策部署，统筹做好疫情防控和经济社会秩序恢复两项工作。压实新冠肺炎疫情防控的属地、部门、单位和个人责任，克服麻痹思想、厌战情绪和侥幸心理，充分认识复工复产后人员流动、人群聚集等可能带来的疫情传播风险，指导城乡社区、机关、企事业单位等树立新冠肺炎疫情防控第一责任人的意识，落实落细各项防控措施。社区和基层医疗卫生机构要按照社区防控工作方案要求，实施网格化、地毯式管理，做好返城人员登记和排查工作。各企事业单位要全面承担起疫情防控的主体责任，抓好工作和休息时段员工管理及各项防控措施的落实，尽最大努力减少聚集性疫情发生。

　　二、落实“四早”措施，及时有效处置聚集性疫情

　　聚集性疫情的处置关键是抓好早发现、早报告、早隔离、早治疗“四早”措施的落实，确保疫情防控措施落实是企事业单位复工复产的前提条件，专业防控只能加强不能削弱。各地要严格按照新冠肺炎防控方案的要求，加强对重点地区、重点场所、重点人群的疫情监测评估，做好防范和应对准备。各单位、各部门发现新冠肺炎疑似情况，要立即采取强有力的措施并及时主动报告。一旦发生疑似疫情，辖区卫生健康部门要迅速组织开展流行病学调查、疑似病例救治、密接接触者管理，落实区域消毒等各项措施，有效处置聚集性疫情，防止疫情进一步扩散蔓延。

　　三、强化工作指导，监督责任落实

　　做好聚集性疫情防控处置直接关系到各省乃至全国防控工作的成败和经济社会的发展，直接关系到复工复产的全面推进，各地要充分发挥联防联控工作机制作用，加强对重点县域的指导，聚焦重点人群、重点场所、重点时段、重点环节，持续加大督查指导力度，及时发现问题、解决问题。要动态评估防控进展，及时总结，通报批评防控不力的地区和机构。对因思想松懈、工作不力导致聚集性疫情发生的，要严格督查问责。

国务院应对新型冠状病毒肺炎

疫情联防联控机制综合组

（代章）

2020年2月25日

（186）文化和旅游部：印发《旅游景区恢复开放疫情防控措施指南》

各省、自治区、直辖市文化和旅游厅（局），新疆生产建设兵团文化体育广电和旅游局：

　　现将《旅游景区恢复开放疫情防控措施指南》印发给你们，请结合实际抓好贯彻落实。

　　特此通知。

　　　　 文化和旅游部资源开发司

　　　　 2020年2月25日

旅游景区恢复开放疫情防控措施指南

　　为落实中央关于统筹推进新冠肺炎疫情防控和经济社会发展工作的有关精神，指导全国旅游景区继续实施疫情防控、稳步做好恢复开放相关工作，根据国务院应对新型冠状病毒感染肺炎疫情防控机制印发的《企事业单位复工复产疫情防控措施指南》，结合旅游景区人员聚集性强、流动性大的特点，制定本工作指南。

　　一、景区开放总体要求

　　（一）坚持分区分级原则，不搞 “一刀切”。按照外防输入、内防扩散的要求，对旅游景区开放条件和必要性进行全面评估。疫情高风险地区旅游景区暂缓开放，疫情中风险和低风险地区旅游景区开放工作由当地党委政府决定。

　　（二）健全疫情防控应急机制。要继续把疫情防控放在首位，毫不放松抓紧抓实抓细各项防控工作。景区恢复开放前应提前做好《旅游景区新冠肺炎防控应急预案》，明确疫情防控应急措施和处置流程。旅游景区主要负责人是景区疫情防控的第一责任人，要把防控责任落实到部门和个人，确保各项措施执行到位。

　　（三）主动做好宣传引导。要从防控大局出发，主动做好公众宣传引导，提升公众疫情防控意识，避免因景区开放造成大范围跨区域人员流动增加疫情防控风险，或引发公众对疫情防控形势的误判。

　　二、加强景区员工健康监测和管理

　　（四）做好员工健康监测和报告。应按照当地要求做好员工健康管理，掌握员工出行轨迹等情况，对来自疫情严重地区的人员要实行居家或集中隔离医学观察。要实行“一进一测一登记”制度，进入景区前须进行体温检测，出现异常情况要及时报告。要关心关爱员工身心健康，及时做好疏解疏导。

　　（五）强化疫情防控培训。应对员工开展传染病预防知识、突发事件应急处置等事项的培训，确保员工上岗前具备必须的防控和处置知识与能力。

　　（六）严格上岗工作规范。应严格落实“戴口罩、勤洗手、保距离”要求，做好个人防护。减少人员聚集，加强员工用餐管理，实行错峰就餐；减少召开会议数量，必须召开的会议应缩短时间、控制规模。

　　三、做好景区公共卫生和场馆防控

　　（七）加强清洁消毒。应及时对景区密闭建筑、公共场所、卫生设施、游乐设备、餐饮场所等进行通风换气、清洁消毒。景区内洗手、喷淋等设施应保持正常运行。做好景区垃圾分类处理。

　　（八）做好医务服务。有条件的景区要准备必要的药物和防护物资，不具备条件的应当与医疗机构建立联系。

　　（九）实施分区分项开放。各景区应根据实际，分区域分项目逐步恢复开放。对不符合开放条件的场所及容易形成人员聚集的项目，可先不开放或延后开放。

　　（十）确保设备安全。应对景区交通和设施设备进行安全检查，确保符合恢复运营条件和安全管理要求。

　　四、强化景区游览管理

　　（十一）严控游客流量。景区科学合理设置承载量。要有效采取门票预约、智慧引导等手段，科学分流疏导游客，做好游客流量关口前置管控。

　　（十二）防止人员聚集。应采取分时段、间隔性办法安排游客入园。景区出入口、重要参观点等容易出现人员聚集位置要配备管理人员，加强游客秩序管理。优化设置游览线路。

　　（十三）落实实名登记。应实行实名制购票，采取适当方式完整记录入园游客联络方式、来往交通等信息，做到可查询可追踪。鼓励景区积极利用大数据和智慧手段，做好游客信息动态监测。

　　（十四）加强游客防护。鼓励景区采取互联网售票、二维码验票等方式有效减少人员接触。游客在测量体温、佩戴口罩后方可入园。保持交通、购票、游览、休息、餐饮等场所人员间距。餐饮服务单位应加强循环使用餐具清洁消毒或使用一次性餐具。

　　（十五）加强现场巡查。应增配人员，加大景区巡查力度，对随意摘除口罩、随地吐痰等不符合疫情防控要求、不文明旅游等行为进行及时劝诫，切实维护好景区游览秩序。

　　（十六）加强防控知识宣传。应通过官方网站、微信公众号、游客服务中心、提示牌、广播、电子显示屏等平台，及时发布景区恢复开放管理措施和疫情防控知识，帮助游客掌握防护要点、增强防护意识、配合防控工作。

　　五、及时有效处置异常情况

　　（十七）加强沟通联动。加强与当地卫生防疫和文化旅游等部门联动，发生异常情况应及时上报。

　　（十八）做好应对处置。发现疑似症状员工或病例的景区，要立即采取隔离措施，加强密切接触者追踪、疫点消毒工作，暂时关闭景区，待情况得到控制并按程序报批后再恢复开放。

（187）市监管总局等3部门：发布支持复工复产十条 国市监综〔2020〕30号

各省、自治区、直辖市及计划单列市、副省级城市、新疆生产建设兵团市场监管局（厅、委）、药监局、知识产权局：

为贯彻落实习近平总书记重要讲话和指示批示精神，按照党中央、国务院关于切实加强疫情科学防控、有序做好企业复工复产工作的部署，现就充分发挥职能作用，解决企业复工复产中面临的实际问题，提出十条政策措施。

一、登记网上办理

充分依托“网上办、掌上办、寄递办、预约办”等有效手段，进一步压减登记注册环节、时间和成本，对生产防疫用品的企业登记注册实行特事特办。对于疫情期间出现的新产业新业态，及时调整经营范围标准。

二、实行告知承诺

对凡涉及生产许可证、强制性认证的复产转产企业产品，快捷办理，压缩审批时限。对具备生产条件但因办理耗时长、暂不能提交相应材料的企业实行告知承诺制，由企业承诺在相应时限内补充提交相关材料后当场给予办结。

在保障食品安全的前提下，简化食品生产、经营许可流程，推进网上办理，推广食品经营许可电子证书的发放。对于申请许可的新办企业、申请许可变更的企业，需要现场核查的，由省级市场监管部门依据本地区食品安全风险分级情况，对低风险食品试点开展告知承诺，对符合条件的实施“先证后查”。

检验检测机构资质认定，全面实行网上办理、邮寄办理，采取延期评审、告知承诺、远程监控评审、专家文审等方式进行。

便利企业并购交易，经营者集中反垄断审查实行网上申报，提高简易案件审查效率，保障企业并购交易顺利进行。优化经营者集中反垄断审查工作机制，加强企业竞争合规指导和服务。

三、建立行政许可应急绿色通道

对生产企业转产生产口罩、防护服等应急物资的，简化生产资质审批程序，合并产品注册及生产许可证检查流程，启动加急检验检测程序，同时认可企业的部分自检报告。对符合许可条件的企业现场确认后，立即办理产品注册和发给生产许可证。对于转产生产医疗器械的企业，实行应急审批，依法办理医疗器械注册证和生产许可证。

对疫情防控所需药品的注册申请，在确保安全性和有效性的基础上，加快审评审批。对疫情防控所需的药品，各省级药品监管部门要指导企业合理安排生产，充分释放产能，全力保障临床供应。对医用口罩、防护服等医疗器械产品注册、生产许可和检验检测等实施特别措施，合并审批流程。

对涉及防治新冠肺炎的专利申请、商标注册，依请求予以优先审查办理。支持企业开展知识产权质押融资，建立知识产权质押登记绿色通道，支持企业快速融资和续贷，缓解资金困难。

四、延长行政许可期限

对在疫情防控期间营业执照登记事项发生变化又不能及时办理变更登记的，延期至疫情解除之后一个月内办理。

对因疫情影响未能按时办理工业产品生产许可证换证导致许可证书过期的生产企业，可办理许可证延期，待疫情解除后再行提交延期申请。

对食品生产、经营许可证到期的，有效期可顺延至当地疫情解除。

对因疫情影响不能按时完成换证的特种设备生产单位及检验检测机构，可办理许可证延期。鼓励符合条件的特种设备生产单位，采用自我声明承诺的方式免评审换证。

对受疫情影响无法按期申请办理检验检测机构复查换证的，可以延期至疫情解除后办理，证书有效期延长至疫情解除后三个月。

对复工复产企业办理专利、商标、集成电路布图设计等事务，因受疫情影响超出相关期限的，依法给予期限中止、顺延，以及请求恢复权利等便利化救济政策措施。

五、加快标准转换应用

支持外贸出口企业复产，加快国际标准与国内标准的转换，推动出口产品依据标准和国内标准的衔接。对依据国际标准生产，且相关国际标准主要技术指标达到强制性国家标准安全要求的，允许在国内生产销售。鼓励社会团体组织制定相关团体标准，增加企业复工复产所需标准的有效供给。对于生产国外标准口罩用于出口、有能力生产国内标准口罩未取得相关资质的企业，加快办理。

六、审慎异常名录管理

对生产、经营疫情防控相关物资的企业，已被列入经营异常名录的，经企业申请，可以简化流程、尽快移出。对因受疫情影响暂时失联的企业，可以暂不列入经营异常名录。

七、严查乱收费乱涨价

加强价格监管，严查各类涉及企业的乱收费行为，减轻企业复工复产负担。支持企业增加产能，严厉打击口罩等防控物资生产领域哄抬价格等违法行为，维护防控物资生产所需机器设备、原辅材料市场价格秩序稳定。

八、加强质量技术服务帮扶

实行计量型式评价专人受理，缩短试验时间。对到期的有关计量标准器具，经所在单位自行核查满足相关技术要求，可适当延长有效期。

鼓励标准化技术组织和机构，围绕企业复工复产提供标准化咨询等标准技术服务。帮助企业尽快建立可操作的防疫质量控制流程和规范，实现防疫控制与企业复工的精细化管理。启动物品编码业务24小时快速响应机制。

引导认证机构加强对复工复产企业的技术服务，开辟线上咨询、受理、评价等综合服务通道，采取线上认证申请受理、延期现场审核检查、优先安排3C认证等方式进行办理。

九、减免技术服务收费

疫情防控期间，市场监管总局所属的计量检定机构、产品质量检验检测机构、特种设备检验检测机构，对复工复产企业计量器具的检定校准收费、产品质量检验检测项目收费、特种设备检验项目收费减少50%，对湖北省企业免除各类检定校准和检验检测费用。市场监管总局所属的标准化技术机构，对中外标准信息咨询服务、标准时效性确认和标准翻译费用予以免收。

十、鼓励企业参加“三保”行动

持续深入开展“保价格、保质量、保供应”行动，不断提升活动覆盖面，为企业搭建与民生期望互通互信的平台。鼓励引导更多企业参加“三保”行动，承诺加快恢复产能，保证产品质量，保持价格稳定，保障市场供应，凝聚疫情防控合力。

各级市场监管、药监、知识产权部门要加强组织领导，强化责任担当，紧密结合各地实际，把十条政策措施进一步细化，落实落细落到位，更好服务于企业复工复产工作，坚决打赢疫情防控阻击战。

市场监管总局 国家药监局 国家知识产权局

2020年2月15日

（188）国务院应对新型冠状病毒肺炎疫情联防联控机制：开展线上服务进一步加强湖北疫情防控工作	联防联控机制综发〔2020〕85号

各省、自治区、直辖市及新疆生产建设兵团应对新冠肺炎疫情联防联控机制（领导小组、指挥部）：

当前疫情防控工作正处在最吃劲的关键阶段，湖北省特别是武汉市依然面临新冠肺炎患者集中、医疗卫生资源紧张、一线医护人员工作强度大等突出问题，亟需拓展线上服务空间，缓解线下诊疗压力，构建线上线下一体化服务模式。为贯彻落实党中央、国务院关于新冠肺炎疫情防控工作的总体部署，充分发挥线上服务远程、高效、智能、便捷等优势，统筹多方力量支撑湖北疫情防控工作，现就有关事项通知如下：

一、加强远程医疗服务。依托国家远程医疗与互联网医学中心（中日友好医院），联合全国部分医院远程会诊平台，搭建重点面向湖北新冠肺炎重症、危重症患者的国家级远程会诊平台，组织呼吸科、感染科、急诊医学科、重症医学科、精神（心理）科、中医科等临床专家资源，对接湖北省级统一平台，义务为全省新冠肺炎医疗救治定点机构提供远程视频会诊、远程影像诊断、远程查房等服务，同时提供针对其他疑难病症的远程医疗服务，满足疫情救治和群众医疗急需。

二、推进人工智能服务。依托中国医师协会等学术团体，组织抗击新冠肺炎远程医疗和人工智能专家组，集聚多方专家资源，通过健康湖北“问诊一点通”在线平台，为患者义务开展线上医疗咨询服务。通过中国医师协会智慧医疗专委会，牵头组织为湖北提供针对新冠肺炎防治的智能自测评估、智能预检分诊、智能辅助诊断等智能医疗应用，义务开展针对新冠肺炎的远程多学科会诊、远程影像联合判读、协同治疗等服务，构建新冠肺炎社区疫情智能监测防控体系。

三、提升中医诊疗服务。依托中国中医科学院、北京中医药大学等中医药专家资源，利用服务于一线救治的中医临床信息管理平台和面向全国开放的大众版“新冠肺炎疫情防控中医药服务平台”，重点为湖北提供针对新冠肺炎的中医药防治知识、自我症状风险评估、线上中医药咨询等服务。鼓励中医医院面向湖北基层医疗机构提供方便实用的中医远程会诊、远程查房等服务，并通过中医馆健康信息平台，结合国家发布的诊疗方案，运用中医理论进行辨证论治，指导基层医生制定中医治疗方案。

四、开展心理援助服务。依托中国疾病预防控制中心精神卫生中心（北京大学第六医院）线上咨询平台，联合国家精神心理疾病临床研究中心网络成员单位、中国科学院心理研究所等高水平专业机构，组织精神卫生、心理治疗、心理危机干预等专家，借助“互联网 ”技术手段，重点为湖北患者、医护人员和疫情防控一线工作人员开展网络心理服务和专业培训指导。充分发挥“健康中国”、省级全民健康信息平台、“12320”等现有网络平台和心理服务热线作用，向受疫情影响的人群有针对性地提供7×24小时心理援助服务。鼓励符合条件的网络心理服务平台，针对疫情防控提供相关公益服务。

五、规范网上诊疗服务。依托国家卫生健康委网站等官方渠道，集中汇聚并发布已经注册审批的互联网医院、互联网诊疗平台及相关医院网站的服务链接。鼓励各地充分发挥互联网医院、互联网诊疗的独特优势，重点面向湖北患者在线开展部分常见病、慢性病复诊及药品配送服务，及时回应和满足当地群众基本就医需求。鼓励优质互联网医疗平台对接湖北省需求，协调在固定社区门口集中投放药品，解决群众居家观察期间买药不便问题。

六、拓展对口支援服务。依托各对口支援省份现有的远程医疗平台，构建实地支援和远程支援相结合、线上线下服务一体化的帮扶体系，延伸为湖北省内对口帮扶市（州、林区）提供远程会诊、远程影像诊断、防治指导等服务，借助信息技术下沉专家资源，提升远程对口支援能力。鼓励各对口支援省份和全国有条件的地区，运用互联网诊疗咨询平台开辟湖北绿色通道，开展针对新冠肺炎的线上免费咨询、健康评估、健康指导、健康宣教、就诊指导、心理疏导等服务，弥补湖北线上医疗健康服务力量的不足，通过线上线下衔接配合，有序分流实体医院发热门诊压力。

七、强化技术保障服务。依托中国互联网协会等行业组织，倡导互联网企业等社会力量为湖北疫情防控提供人力、技术、物资等多方援助。鼓励符合条件的互联网企业充分发挥技术和平台优势，为湖北省域内医疗机构开展医疗健康服务提供技术支持，特别是对各类重点医疗机构和新建方舱医院提供网络技术安全环境支撑。通过中国信息通信研究院搭建数字健康资源供给对接平台，整合国内5G智慧医疗、远程医疗网络、移动健康APP、网络安全等资源，协调相关企业义务为湖北新冠肺炎医疗救治定点医院、方舱医院及其他防控机构提供信息技术解决方案、高速网络环境、信息技术产品以及网络安全保障，并建立与湖北的防控物资对接渠道，支撑主管部门防护物资调配。

各地各单位要坚持需求导向，加强协调配合，精准对接湖北省和武汉市疫情防控所急所盼，借助线上服务提升援助效率，切合实际提供有效帮扶，全力支援湖北早日打赢疫情防控阻击战。

国家卫生健康委规划司联系电话：010—68791424，68792930

国家中医药局规财司联系电话：010—59957792，59957661

湖北省卫生健康委联系电话：027—87821870，87710138

综合技术协调保障联系电话：010—62302915，62300170

国务院应对新型冠状病毒肺炎疫情

联防联控机制综合组

2020年2月26日

（189）国务院应对新型冠状病毒肺炎疫情联防联控机制：进一步做好农村地区新型冠状病毒感染的肺炎疫情防控工作	肺炎机制发〔2020〕14号

各省、自治区、直辖市及新疆生产建设兵团应对新型冠状病毒感染的肺炎疫情联防联控机制（领导小组、指挥部）：

新型冠状病毒感染的肺炎疫情发生以来，党中央、国务院高度重视，习近平总书记先后作出一系列重要指示批示。当前，疫情防控正处于关键期，农村防疫工作是当前防控工作重点之一，迫切需要采取更加有力有序、科学周密举措，防止疫情在农村地区扩散。为贯彻落实中央政治局常委会会议、中央应对新型冠状病毒感染的肺炎疫情工作领导小组会议和国务院有关会议部署安排，进一步做好农村地区新型冠状病毒感染的肺炎疫情防控工作，现就有关事项通知如下：

一、充分认识做好农村地区疫情防控工作的重要性紧迫性。习近平总书记高度重视疫情防控工作，强调要把人民群众生命安全和身体健康放在第一位，把疫情防控工作作为当前最重要的工作来抓。李克强总理指出，要高度重视农村疫情防控。我国农村地区医疗卫生条件薄弱，农民工大批返乡可能增加疫情发生的风险。各级党委政府特别是县、乡党委政府，要认真学习领会习近平总书记重要指示精神，贯彻落实中央决策部署，增强“四个意识”、坚定“四个自信”、做到“两个维护”，充分认识农村地区疫情防控的重要性、复杂性和严峻性，提高政治站位，强化责任担当，积极履职尽责，全力以赴推动各项防控措施在农村落地落实，坚决打赢农村疫情防控阻击战。

二、压实县乡党委政府和村“两委”责任。各地党委政府要在应对疫情工作领导小组和联防联控工作机制下，成立农村疫情防控专班，强化统一指挥调度，坚持农村和城市防控一体部署、一体推进，切实保障农村疫情防控工作物资、资金和人员需要。县、乡党委政府要把主要精力放在抓农村疫情防控上，充分发挥基层党组织、村民自治组织作用，引导动员广大农民群众积极参与群防群控，形成防控合力。

三、充分发挥农村基层医疗卫生机构和村医作用。在县级卫生健康行政部门领导和疾控等专业机构指导下，乡镇卫生院、村卫生室人员要做好农村地区的筛查、医学追踪、预检分诊和转诊，实行日报告制度。发现发热患者和来自疫情发生地区的疑似感染者要及时转诊至上级医院发热门诊，分级分类做好防护和信息登记并按时上报。协助追踪、督促来自疫情发生地区和确诊病例、疑似病例的密切接触者，落实居家医学观察措施。基层党委政府要抓好农村地区消毒用品、口罩和防护服等防护用品、体温计等的调拨储备，确保农村基层医疗卫生机构物资供应。通过视频培训、发放技术指南等方式，加大对县乡村医疗卫生人员培训力度，提升疾病识别、初步处理、感染控制和防护能力。对人力不足的卫生院、卫生室要及时安排支持、支援人员，确保工作任务落实到位。

四、强化返乡人员、流动人口健康管理。乡镇卫生院、村医协助做好对返乡人员、流动人口的追踪、筛查，加强健康管理。加强对武汉等重点疫情发生地区返回人员排查、登记、随访，做好健康状况监测，了解是否有发热、咳嗽等不适症状，做好登记，落实居家观察等措施，发现异常情况及时报告处置。督促休闲农业、农村旅游经营主体落实防控责任和防控措施，确保游客和工作人员健康安全。

五、加强防控工作宣传引导。通过循环播放广播、编印宣传资料、印发倡议书和公开信、电话微信通知等多种方式，加强农村地区防控信息通报和防疫知识宣传，增强农民群众健康意识、防病意识，提高防病能力。结合村庄清洁行动，教育引导农民群众养成戴口罩、勤洗手等健康生活方式。积极倡导节庆文明新风，停办庙会集市、游园节庆等公众活动，劝说农民群众不大办婚丧嫁娶，尽量少摆席、少串门、少走动，避免人群聚集，降低疫情传播风险。

国务院应对新型冠状病毒感染的

肺炎疫情联防联控机制

2020年1月30日

（190）国务院应对新型冠状病毒肺炎疫情联防联控机制：全力做好一线医务人员及其家属保障工作	肺炎机制发〔2020〕23号

各省、自治区、直辖市及新疆生产建设兵团应对新型冠状病毒肺炎疫情联防联控工作机制（领导小组、指挥部）：

新型冠状病毒肺炎疫情发生以来，广大医务工作者积极响应党中央号召，牢记“一切为了人民健康”的初心使命，坚决贯彻习近平总书记重要指示精神，增强“四个意识”、坚定“四个自信”、做到“两个维护”，舍小家，为大家，不顾个人安危，生动诠释了敬佑生命、救死扶伤、甘于奉献、大爱无疆的崇高精神。习近平总书记在1月25日和2月3日两次中央政治局常委会会议上，都对关心关怀奋战在一线的医务人员作出重要指示。当前，疫情防控进入关键期，坚守一线的医务人员及其家属在家庭生活等方面遇到困难，各级党委政府必须采取强有力措施，及时有效为一线医务人员解除后顾之忧，为打赢疫情阻击战提供坚强保证。重点从以下五个方面做好保障：

一、生活保障

各级党委政府要动员社会力量，组织发动志愿者或安排专门人员，对一线医务人员及其家属展开慰问，定期了解他们的需求和困难，建立台账，积极协调解决，要为一线医务人员提供必要的隔离场所，确保医务人员充分的睡眠和饮食。对一线医务人员及其家属的基本生活用品要保证供给。

二、心理保障

各级党委政府要认真落实《关于印发新型冠状病毒感染的肺炎疫情紧急心理危机干预指导原则的通知》（肺炎机制发〔2020〕8号），将一线医务人员及其家属作为重点心理干预人群，主动关心，通过不同形式进行谈心疏导，减少他们的心理焦虑，给予积极的精神关怀。

三、人文保障

各级党委政府要为一线医务人员和家属建立沟通联络渠道，让一线医务人员和家属互相支持鼓励，构筑战胜病毒的信心决心。尽量不安排双职工的医务工作者同时到一线工作，特别是家有老人和孩子需要照顾的医务人员，要尽可能使其兼顾家庭。要安排志愿者或专门人员对有家庭困难的一线医务人员家属进行对口帮助。

四、安全保障

各级党委政府要做好对一线医务人员及其家属口罩等防护用品的配给。对有交通困难的医护人员及其家属提供必要帮助。对发现有歧视孤立一线医务人员及其家属行为的，要及时进行批评教育，情节严重的应给予相应处理处罚。

五、其他保障

各级党委政府要认真落实参与防治工作医务人员的临时性工作补助，落实加班费、误餐补助等福利措施。加大典型人物、事迹的发掘和宣传力度，及时表扬表彰，弘扬正气，传播正能量，增强一线医务人员及其家属荣誉感。

各级党委政府要组建专人专班，以高度负责的态度落实一线医务人员及其家属保障工作，当好他们的贴心人和主心骨，采取务实、贴心、到位的举措，帮助他们解决实际困难，做好他们的坚强后盾。建立台账和相关落实情况要及时报送联防联控机制。

国务院应对新型冠状病毒肺炎

疫  情  联  防  联  控  机 制

（代 章）

2020年2月5日

（191）自然资源部：做好疫情防控期间地质灾害防范工作	自然资办函〔2020〕308号

各省、自治区、直辖市自然资源主管部门，新疆生产建设兵团自然资源主管部门：

2020年2月以来，全国多地遭遇强降雨、强降雪等极端天气。据气象部门预测，2月底至3月上旬，南方局部地区将出现持续降雨或多次降雨过程，北方地区随气温逐步升高，冰雪消融、冻土开化，部分地区地质灾害风险开始显现。为做好疫情防控期间地质灾害防范工作，各地要结合实际情况，及早部署，积极应对，尽最大努力保障人民群众生命财产安全。

一是高度重视，及早部署。各地自然资源主管部门要密切联系相关部门，时刻关注强降雨、洪涝、台风、地震等可能引发次生地质灾害的不利因素。东北、华北地区要做好“桃花汛”引发地质灾害的防范工作；西北地区要防范融雪和冻融作用引发地质灾害的防范工作；西南山区要防范强降雨和地震引发地质灾害的防范工作；东南沿海地区要做好强对流天气期间局地短时强降雨引发地质灾害的防范工作。

二是认真排查，积极应对。各地要结合疫情防控实际，提早启动监测预警和专家巡查指导制度。要采取灵活有效的工作方式，组织力量加强排查，在避免人员密切接触前提下，重点针对人口居住区、城乡结合部、各类移民搬迁场所、山区丘陵区高陡边坡、公路边坡、建房切坡等加大排查力度，排查出来的隐患点要全部纳入群测群防和预警体系，切实做到时时有人管、处处有人防。

三是及时预警，果断避险。各地要加强群测群防和监测预警，切实做好应急预案，一旦出现险情灾情，果断撤离危险区内所有人员，并做好临时避难场所的人员隔离防护措施。

四是加强宣传，增强意识。要进一步加强宣传教育，采取互联网、手机、广播、电视等方式普及防疫特殊时期防灾减灾和临灾避险知识，增强基层人民群众地质灾害防范意识。

五是积极稳妥推进治理工程。各地要坚决贯彻落实习近平总书记关于分区分级精准复工复产的重要指示精神，结合本地实际情况，按照复工复产有关规定，稳妥推进地质灾害治理工程复工，努力保障原计划于今年汛期前完成的重大治理工程加快实施，尽快发挥防灾减灾效益。

自然资源部办公厅

2020年2月26日

（192）卫健委：进一步落实科学防治精准施策分区分级要求做好疫情期间医疗服务管理工作	国卫办医函〔2020〕162号

各省、自治区、直辖市及新疆生产建设兵团卫生健康委：

为进一步落实国务院联防联控机制《关于科学防治精准施策分区分级做好新冠肺炎疫情防控工作的指导意见》，指导各地在分区分级精准防控的基础上，有序做好医疗服务管理工作，满足患者就医需求。现将有关要求通知如下：

一、落实分区分级差异化管理要求

地方各级卫生健康行政部门要认真贯彻落实党中央关于突出重点、统筹兼顾，分类指导、分区施策的要求，根据本省域划分的低风险、中风险、高风险地区，制订针对性强、切实有效的医疗服务管理措施。要指导辖区内各级各类医疗机构有序推动和加强日常医疗服务管理，恢复和保障正常的医疗服务，满足人民群众就医需求。

二、低风险地区全面提供正常医疗服务

低风险地区要严格落实“外防输入”策略、全面恢复正常生产生活秩序的要求，全面向人民群众提供日常医疗服务。要指导辖区内医疗机构合理调配医疗资源，保障门急诊、住院、手术、检验检查等服务顺利开展。要加强医疗机构发热门诊病例监测、发现和报告，进一步强化医疗机构内感染预防与控制，加强医疗管理，保障医疗质量和医疗安全。除派出医务人员驰援湖北导致人员数量不足等特殊原因外，医疗机构不得以疫情防控为由，影响提供日常医疗服务。

三、中风险地区尽快有序提供正常医疗服务

中风险地区要严格落实“外防输入、内防扩散”策略、尽快有序恢复正常生产生活秩序的要求，逐步加强提供正常医疗服务。要指导辖区内医疗机构在做好疫情防控相关工作的基础上，逐步将工作重点转移到日常医疗服务保障上。除必要的疫情防控人员安排外，其他人员应当尽快返回各自工作岗位，恢复正常工作状态，及时为人民群众提供日常医疗服务。要毫不放松，落实落细疫情防控工作，科学合理调配疫情防控人员等医疗资源，并根据区域风险等级进行动态调整。

四、高风险地区做好重点患者医疗服务保障

高风险地区要严格落实“内防扩散、外防输出、严格管控”策略、根据疫情态势逐步恢复生产生活秩序的要求，在重点抓好疫情防控工作的同时，加强重点患者的医疗服务保障。要指导医疗机构根据不同患者的医疗需求进行分类救治，满足患者基本的、必须的就医需求。要一并做好普通肺炎、重症流感以及其他感染性疾病患者的医疗救治；要发挥基层医疗机构作用，对需长期用药治疗的冠心病、高血压、糖尿病、精神病等慢性非传染性疾病以及艾滋病、结核、肝炎等慢性传染性疾病患者加强管理，保障其用药需求；要采取有效措施，切实满足肿瘤放化疗、血液透析等需长期治疗的重大疾病患者的医疗需求；要落实有关要求，满足孕产妇、儿童、老年人等弱势群体的医疗服务需求；要持续开放院前急救和院内急诊，保障急危重伤病患者能够得到及时救治。高风险地区也要随风险等级调整，逐步恢复全面提供正常医疗服务。

五、加强互联网诊疗咨询服务工作

各地区要充分利用“互联网+医疗”的优势作用，在疫情防控中积极做好互联网诊疗咨询服务。大力开展预约挂号、预约检查，合理分布预约时段，减少人群聚集，降低交叉感染风险。积极提供线上健康评估、健康指导、健康宣教、就诊指导、慢病复诊、心理疏导等服务，精准指导患者有序地去实体医院就诊。卫生健康行政部门要加强对互联网诊疗服务的监管，确保诊疗服务规范、科学、合理开展。

国家卫生健康委办公厅

2020年2月26日

（193）交通运输部 农业农村部：认真贯彻落实习近平总书记重要指示精神 全力做好春季农业生产物资运输服务保障	交运明电〔2020〕77号

各省、自治区、直辖市交通运输厅（局、委）、农业农村（农牧）厅（局、委），新疆生产建设兵团交通运输局、农业农村局：

近日，习近平总书记对全国春季农业生产工作作出重要指示强调，越是面对风险挑战，越要稳住农业，越要确保粮食和重要副食品安全；各级党委要把“三农”工作摆到重中之重的位置，统筹抓好决胜全面建成小康社会、决战脱贫攻坚的重点任务，把农业基础打得更牢，把“三农”领域短板补得更实，为打赢疫情防控阻击战、实现全年经济社会发展目标任务提供有力支撑。为深入贯彻落实习近平总书记重要指示精神和党中央、国务院决策部署，统筹做好2020年春季农业生产物资（种子种苗、农药、肥料、农膜、饲料、兽药、疫苗、仔畜雏禽、转场蜜蜂、水产苗种、农机具及零配件、相关物资包装等）运输保障工作，全力支撑春季农业生产，确保粮食和重要副食品安全，现将有关事项通知如下：

一、主动对接需求，联合制定春季农业生产物资运输保障方案

各地交通运输、农业农村部门要主动对接当地发展改革等部门，详细了解当前春季农业生产物资运输需求，摸清重点物资的布局、数量、运输路线，认真梳理运输现状及存在问题，坚持问题导向、精准服务，全力做好农业生产物资运输服务保障工作。要依托交通运输、农资产品行业协会，组织相关骨干农业生产物资重点生产流通企业、大型运输企业进行运输供需对接，联合制定春季农业生产物资运输保障方案。对于湖北等疫情严重地区相关部门和单位提出的春季农业生产物资运输需求，交通运输部门要采取针对性保障措施，建立对接机制，实施“点对点”支持，一事一处理，确保农业生产物资不因运输问题而影响供给。

二、有序恢复正常交通运输秩序，保证春季农业生产物资运输通畅

各地交通运输部门要全力配合公安机关，严格贯彻《国务院办公厅关于做好公路交通保通保畅工作确保人员车辆正常通行的通知》及公安部等七部门《关于做好恢复正常交通运输秩序保障道路交通畅通工作的意见》要求，有序恢复正常交通运输秩序，有力保障公路路网顺畅运行，切实解决春季农业生产物资运输车辆“进村难”、高速公路“上得来、下不去”以及农机具跨区域作业转运难等问题。各地农业农村部门要向春季农业生产物资重点生产流通经营主体提供各地交通运输部门公布的24小时应急运输电话，交通运输部门要及时受理和解决春季农业生产物资等运输车辆通行中相关问题，保障车辆顺畅高效通行。

三、落实绿色通道政策，保障春季农业生产物资运输车辆优先便捷通行

各地交通运输部门要全面落实疫情防控期间免收车辆通行费政策，将春季农业生产物资和农机具转运纳入应急运输绿色通道政策范围，承运企业自行打印全国统一的“疫情防控应急物资及人员运输车辆通行证”（网址：http://www.gov.cn/zhengce/zhengceku/2020-02/02/content_5473801.htm），确保春季农业生产物资和农机具运输车辆不停车、不检查、不收费，优先通行。各地农业农村部门要会同交通运输部门向当地农资产品行业协会、春季农业生产物资重点生产流通经营主体宣传有关政策，主动做好协调服务，确保春季农业生产物资和农机具及时入村进场。对短期内向疫情重点地区运送物资的驾驶人、装卸工等提供保障的人员，按照当地疫情防控要求实施科学防护，体温检测符合规定并采取佩戴口罩等防护措施的，无需采取14天隔离措施。

交通运输部

农业农村部

2020年2月26日

（194）交通运输部：规范交通运输行政执法服务统筹推进疫情防控和经济社会发展工作	交法明电〔2020〕78号

各省、自治区、直辖市、新疆生产建设兵团交通运输厅（局、委），部长江航务管理局、珠江航务管理局，部直属海事局：

为深入贯彻落实习近平总书记重要讲话精神和党中央、国务院关于统筹推进疫情防控和经济社会发展工作部署，坚持运用法治思维和法治方式开展工作，现就规范交通运输行政执法，切实做好统筹推进疫情防控和经济社会发展执法保障工作通知如下：

一、提高政治站位，切实把思想和行动统一到习近平总书记重要讲话精神上来

各级交通运输主管部门和交通执法机构要深入学习认真落实习近平总书记关于统筹推进新冠肺炎疫情防控和经济社会发展工作重要讲话精神，切实增强“四个意识”、坚定“四个自信”、做到“两个维护”，把思想和行动统一到党中央、国务院决策部署上来，越是到最吃劲的时候，越要坚持依法防控，在法治轨道上统筹推进各项工作。坚持执法为民，坚持严格规范公正文明执法，切实做好服务保障统筹推进疫情防控和经济社会发展有关工作。

二、找准定位，为统筹推进疫情防控和经济社会发展工作提供法治保障

当前疫情形势依然严峻复杂，防控正处在最吃劲的关键阶段。交通运输是经济社会发展的“先行官”，在疫情防控和经济社会发展中发挥着重要作用。交通运输执法队伍作为疫情防控的重要力量，要坚持依法行政，严格依照法定权限和程序执法，依法应对、妥善处理疫情防控中出现的各类矛盾和问题，全面提高依法防控、依法治理能力，确保维护道路航道通畅、运输秩序平稳和人员物资运输安全等在法治轨道上统筹推进。各级交通运输主管部门和交通执法机构党组织、党员领导干部要发挥战斗堡垒作用和先锋模范作用，主动担当、积极作为、坚守岗位、靠前指挥，抓好工作落地落实，在实战中增强工作本领。要防止和克服麻痹思想、侥幸心理、松劲心态，抓好执法工作，助力打通“大动脉”、畅通“微循环”，全力保障交通网络畅通高效。

三、规范执法行为，抓好行政执法各项工作落地落实

各级交通运输主管部门和交通执法机构要以保畅通、保运输、保安全、保重点、保服务为目标，坚持问题导向、坚守法治底线，坚决防止和杜绝不作为、乱作为、粗暴执法等行为，最大限度减少对群众利益影响，保障人民生命健康安全。要按照当地党委、政府关于疫情防控统一部署，遵循“分区分级”要求，在执法工作中推动落实好“三不一优先”“一断三不断”等交通运输各项防控要求，打通客流、物流堵点，推进客运货运和城市交通、邮政快递等恢复正常运营，为经济社会发展和人民群众生产生活提供坚实支撑。

要认真贯彻执行《中华人民共和国传染病防治法》《全国人大常委会关于全面禁止非法野生动物交易、革除滥食野生动物陋习、切实保障人民群众生命健康安全的决定》以及交通运输相关法律法规，紧盯重点环节、关键部位，将执法关口前移，重点加强对客运、货运场站和港口码头以及运输企业的监督检查，督促相关企业落实经营主体责任。依法打击破坏疫情防控的交通运输违法行为。对非法营运、客运班车不按规定线路行驶和站点停靠、客运包车招揽包车合同以外的旅客乘车等违法违规行为，要依法严肃予以查处。对发现非法运输野生动物违法行为、需要采取防疫应急措施的人员和交通工具，要及时移交并配合卫生健康、公安机关等相关部门依法处置。对擅自设卡、拦截、断路、暂停港口作业等阻断交通的违法行为，根据疫情形势变化和需要，在当地党委、政府领导下，积极配合相关部门依法恢复正常交通秩序。在执法过程中，要注意及时发现运输行业中影响疫情防控和经济社会发展的苗头性、倾向性问题，做好预警工作。对轻微违法行为，可采取提醒、劝告等非强制性方法进行执法。

四、抓好执法监督，促进严格规范文明公正执法

各级交通运输主管部门和交通执法机构要认真履行行政执法监督职责，增强行政执法监督效能，加强对执法工作的监督指导。对涉及疫情防控有关交通运输行政执法活动的投诉举报要及时复核、及时回复。对群众和企业反映强烈的问题，及时依法查处。要严格落实行政执法三项制度，规范行政处罚等执法程序，加强涉案财物管理，依法保障和维护行政相对人的权益。要严格落实执法责任制，推动建立健全“执法有依据、行为有规范、权力有制约、过程有监督、违法有追究”的行政执法责任制度体系。要落实责任倒查机制和追究机制，对疫情防控执法工作中作风漂浮、落实不力、影响疫情防治工作的失职渎职执法人员，依法从严追究责任。对出现的乱执法、乱罚款等违法违规案件，需要追究党纪政纪责任的，要依法移送纪检监察机关处理。涉嫌犯罪的，要依法移送司法机关处理。

五、围绕疫情防控，加强交通运输普法宣传工作

认真落实“谁执法谁普法”责任制，对照普法责任清单，结合疫情防控和复工复产期间交通运输工作特点，强化普法宣传教育，加强《中华人共和国传染病防治法》《突发公共卫生事件应急条例》等法律法规宣传，在公共运输工具、客货场站、高速公路服务区等以多种方式开展普法宣传。特别是结合全国人大常委会刚刚通过的关于禁止非法野生动物交易的有关决定，将违法运输野生动物等案件作为交通运输疫情防控普法的重点，以案释法，充分发挥典型案例的引导、规范、预防与教育功能，营造知法守法，防控疫情、法治同行、社会群众理解并支持交通运输执法工作的浓厚氛围。

六、加强关心爱护，提升执法人员疫情防控保障水平

要加强对执法人员的关心关怀，科学合理做好执法执勤工作安排，保障一线执法人员合理休息时间和及时轮换。精准细致做好执法人员思想政治工作，做好自身防护，及时进行心理疏导和慰问。做好执法站所环境清洁、通风、消毒等工作，为一线执法人员配备防护服、口罩等必要的防护装备，提供必要的户外执勤生活保障。对执法人员中确诊患者、疑似病例、密切接触者，要协调做好就医、隔离、留观、检查等工作，确保执法队伍战斗力。对在疫情防控工作中表现突出的，要给予表彰奖励。

各级交通运输主管部门要认真梳理、分析疫情防控期间执法工作中存在的问题，提出意见建议，为后续相关法律法规制度立改废释提供依据。要反思交通运输法治政府部门建设存在的短板和薄弱环节，总结经验、完善机制，不断深化交通运输法治政府部门建设。

国家铁路局、中国民用航空局、国家邮政局要参照本通知有关要求，结合系统工作实际对行政执法工作进行部署和安排，更好地服务统筹推进疫情防控和经济社会发展工作。

交通运输部

2020年2月26日

（195）商务部关于应对新冠肺炎疫情做好稳外贸稳外资促消费工作的通知	商综发〔2020〕30号

各省、自治区、直辖市、计划单列市及新疆生产建设兵团商务主管部门：

为深入贯彻习近平总书记关于疫情防控工作重要讲话和指示批示精神，落实党中央、国务院决策部署，坚决打赢疫情防控的人民战争、总体战、阻击战，统筹做好疫情防控和经济社会发展，积极做好稳外贸稳外资促消费工作，把疫情对商务发展的影响降到最低，现就有关事项通知如下：

一、支持企业有序复工复产。按照当地统一安排和规定条件，协调复工审核部门加快办理手续，支持外贸、外资、商贸流通和电子商务企业有序复工复产，稳妥有序推进共建“一带一路”重大项目。引导生产防护用品类的外贸外资企业扩大生产。鼓励外贸企业增加国内紧缺的医用物资和农产品进口。积极协调有关部门，将承担生活必需品保供任务的重点商贸流通企业和电子商务企业纳入防护物资优先保障范围，优先配备口罩、消毒液、防护手套、护目镜等必要的防护物资。

二、简化对外经贸管理程序。引导企业无纸化申领进出口许可证件。全面实现最终用户和最终用途证明申办系统无纸化，加速处理与疫情防控相关物项的办理申请。开展两用物项和技术进出口许可跨部门电子会签。推进技术进出口合同登记申请无纸化，抓紧启动电子证照工作。在疫情解除之前，依据企业网络传输资料，在线审核服务外包合同。加快实行对外投资备案（核准）无纸化管理，实现全流程网上办理。

三、强化法律服务帮助企业降低风险。支持贸促会、商会等为外贸企业和境外项目实施主体无偿出具因疫情导致未能按时履约的不可抗力事实性证明。协调国内外组展机构，帮助因疫情无法出国参展的企业妥善处理已付费用等问题。协调中介机构为受影响的走出去企业提供法律等咨询服务。发挥各级应对贸易摩擦工作站作用，提供法律服务，帮助企业解决疫情对贸易救济案件应对造成的困难。

四、支持外贸新业态新模式发展。指导跨境电商综试区提供海外仓信息服务，帮助企业利用海外仓扩大出口。支持市场采购贸易与跨境电商融合发展，探索试点市场闭市期间成交新渠道。积极推进二手车出口，为受疫情影响的企业提供便利服务。积极与周边国家沟通协调，疫情解除后尽快恢复开展边民互市贸易，支持边境贸易发展。

五、加强出口信用保险支持。加强与中信保公司驻各地营业机构协作，充分发挥出口信用保险作用，进一步扩大出口信用保险覆盖面，合理降低短期险费率，加大对受疫情影响企业支持力度，帮助企业应对订单取消、出运拒收等风险。开辟理赔服务绿色通道，在贸易真实的情况下适当放宽理赔条件，做到应赔尽赔、能赔快赔。积极开展政府+银行+保险合作，扩大保单融资规模。

六、积极应对境外贸易限制措施。充分发挥贸易救济预警体系和法律服务机制作用，及时发布各国针对疫情出台的贸易限制措施，畅通企业反映问题渠道。关注本地区出口产品在国外遇到不合理限制情况，及时上报，配合驻外使（领）馆经商机构加大双边交涉力度，配合在世界贸易组织发声、做相关方工作，敦促各成员遵守世界贸易组织规则、尊重世界卫生组织权威和专业意见，反对有关国家（地区）过度反应和施加不必要的贸易限制。

七、深化服务贸易创新发展。发挥服务贸易创新发展引导基金导向作用，支持符合条件的服务贸易企业创新发展。落实好非商业性境外办展政策，对受疫情影响较大的地区和企业适当予以政策倾斜。对于已在商务部审批或备案的涉外经济技术展览会，因疫情影响已确定取消或推迟的，申办单位可通过商务部统一平台取消办展或调整办展时间。对商务部实施的两种涉外经济技术展行政许可事项全面推行在线审批。

八、用好自贸协定优惠政策。利用中国自贸区服务网等平台，做好我国与有关国家和地区已签署自贸协定的宣传推广，鼓励企业用好用足自贸协定中的关税减免、投资促进等优惠政策。

九、稳定外资企业信心。实施好外商投资法及实施条例等法律法规。指导外资企业用足用好应对疫情各项支持政策措施，做到内外资企业同等对待、一视同仁，发挥外资企业投诉机制作用，保障外资企业同等适用支持政策。根据本地实际情况，在法定权限内制定支持外资企业应对疫情影响、加大稳外资力度专项政策，相关政策措施和做法及时报商务部复制推广。

十、加强外资大项目跟踪服务。密切跟踪在谈外资大项目，分类施策、一企一策，精准研究支持政策，及时协调解决项目推进过程中的困难和问题。对于在建外资大项目，开展“点对点”服务保障，协调解决用地、用工、水电、物流等问题，保障企业投资按计划进行。对于大项目推进过程中涉及中央事权的共性政策问题，及时上报。

十一、创新和优化招商引资方式。通过网上洽谈、视频会议、在线签约等方式，整合各类招商资源，持续推进投资促进和招商工作。充分利用各种招商机构和平台，加大投资环境和合作项目宣传推介力度。加强与境外各类商协会等中介组织合作，积极开展委托招商、以商招商，组织灵活多样的招商活动，争取一批新项目签约落地。

十二、指导开放平台抓防控促发展。支持自由贸易试验区、国家级经济技术开发区加强用工、供应等跨区域对接，缓解疫情期间企业用工、配套困难。指导自由贸易试验区加快改革开放创新试点，围绕试点领域涵养客商资源，培育壮大外向型经济。加快北京市服务业扩大开放综合试点措施落地，抓紧开展试点经验总结评估及复制推广。

十三、加强外资企业动态监测。完善外商投资信息报告制度，充分运用信息化手段，加强研判和预警，及时掌握各行业、各领域外资企业受疫情影响情况。强化与外资企业协会和外国商会的联系机制，及时了解会员企业生产经营状况和投资动向，有针对性地加强服务和指导，特别是对龙头企业和产业链关键环节企业的服务保障。

十四、设立海外疫情应对快速反应机制。发挥境外企业和对外投资联络服务平台作用，加强运行监测、风险提示、应对指导和服务保障，指导企业有效防范风险，推进高质量共建“一带一路”。充分发挥境外中资企业商（协）会作用，在境外中资企业商会联席会议机制办公室设立海外疫情应对快速反应机制，及时搜集企业遇到的实际困难和问题，做好协调服务。

十五、支持商贸流通企业创新经营服务模式。鼓励开展商务领域经营互助，引导零售企业与餐饮、住宿等生活服务企业通过共享员工等方式，缓解零售企业用工短缺困难，着力稳定相关行业工作岗位。积极协调有关部门和小区物业，在符合各地相关规定和保证食品卫生安全的前提下，支持企业采取店铺外摆、露天市场、团购、无店铺等方式进行销售，便利社区居民生活。

十六、加快步行街改造提升。加快推出一批全国示范步行街，启动第二批步行街改造提升试点。已经确定的改造施工项目，可积极创造条件尽早启动。指导步行街管理机构在认真做好疫情防控的前提下，统筹协调各类商户有序复工营业。超前谋划、合理安排，在疫情得到有效控制后，组织形式多样的促消费活动，充分释放被疫情抑制的消费潜力。

十七、推动服务消费提质扩容。落实生活服务业疫情防控指南，指导企业规范服务流程，切实保障服务人员和消费者身体健康。开展社区生活服务业发展试点，引导餐饮等生活服务企业在做好疫情防控的前提下积极下沉社区，为受疫情影响的社区居民提供安全的生活服务。

十八、释放新兴消费潜力。发展网络消费，引导电子商务企业以数据为纽带，精准匹配网络消费新需求，打造“小而美”的网络新品牌。提高农产品可电商化水平，扩大农产品网络消费。开展网络促销活动，促进网上品牌品质消费。鼓励电商、快递等企业与实体店、商务楼宇和小区物业等合作，开展末端配送服务合作。鼓励探索无直接接触配送服务，推广定点收寄、定点投递等方式。鼓励生活服务企业拓展线上销售，采用“中央厨房+线下配送”等新发展模式经营。

十九、发展便利店和菜市场。优化便利店网点、菜市场布局，充分发挥经营灵活、贴近社区居民的优势，满足居民生活必需品需求，打造“一刻钟便民生活服务圈”。引导商贸流通企业和电子商务平台经营者，为小店提供集采集配、供应链融资、先供货后付款等服务。鼓励小店开展网络营销拓展业务，发挥小店在促进就业、扩大消费、提升经济活力、服务改善民生等方面作用。

二十、发挥好财政资金作用。商财政部门抓紧安排使用提前下达的2020年中央财政专项资金，加大结存资金统筹使用力度，用好地方配套资金，带动社会资本，全力支持稳外贸稳外资促消费。外经贸发展专项资金可向受疫情影响较大的领域倾斜，重点支持企业开拓国际市场、以政银保合作方式加大贸易融资支持、外商投资促进服务、开放平台吸引外资、“一带一路”投资合作等。服务业发展资金中，电商进农村综合示范资金重点支持农产品进城、工业品下乡，促进疫情防控期间的农产品销售，农商互联农产品供应链资金增加支持农产品保供方向，流通领域供应链体系建设资金要强化生活必需品供应链功能作用，未安排的结余资金可统筹用于支持应对疫情的商贸流通相关工作。上述资金安排应避免与其它政策平台、资金渠道等交叉重复。

各级商务主管部门要以习近平新时代中国特色社会主义思想为指导，增强“四个意识”、坚定“四个自信”、做到“两个维护”，坚决贯彻党中央、国务院决策部署，牢记初心使命，强化责任担当，积极主动作为，加强横向协作、纵向联动，统筹疫情防控和商务发展，依法推进各项工作，全力以赴稳外贸稳外资促消费，努力完成全年目标任务，更好地服务经济社会发展大局，为全面建成小康社会多作贡献。

商务部

2020年2月18日

（196）国务院应对新冠肺炎疫情联防联控机制科研攻关组：规范医疗机构开展新型冠状病毒肺炎药物治疗临床研究

各省、自治区、直辖市及计划单列市科技厅（委、局）、卫生健康委、药品监督管理局，新疆生产建设兵团科技局、卫生健康委、药品监督管理局，各有关单位：

为进一步做好新型冠状病毒肺炎（以下简称新冠肺炎）疫情防控工作，促进有潜在抗病毒或对症治疗作用的药品及早进入临床应用，根据《传染病防治法》、《药品管理法》、《医疗机构管理条例》、《涉及人的生物医学研究伦理审查办法》和《医疗卫生机构开展临床研究项目管理办法》，就国家启动公共卫生应急机制期间，有关“老药新用”开展抗新冠肺炎治疗临床研究，提出以下要求：

一、支持符合条件的医院开展相关药品临床研究

相关临床研究原则上应当在县级以上地方卫生行政部门确定的新冠肺炎救治定点医院（包括方舱医院等）进行。所使用的药品应为已上市药品。相关药品应在体外实验中对新冠病毒具有明确的抑制作用，或动物实验结果支持开展新冠肺炎治疗临床研究。在临床研究中给药方法不超过现有药品说明书的用法用量，预期人体内药物浓度可以达到体外实验换算到人体的有效浓度。医疗机构是临床研究的责任主体。临床研究活动应由副高及以上专业职称的执业医师负责，针对轻、中、重症患者分层制定完善的临床研究方案，对可能出现的风险制定预案和管控措施。

二、提高相关药品临床研究的整体效率

各级卫生和科技行政部门要及时收集辖区内医疗机构和科研机构所提出的拟开展临床研究的药品，及时通过省级卫生和科技行政部门上报国务院应对新冠肺炎疫情联防联控机制（以下简称联防联控机制）科研攻关组组长单位科技部的办公厅，科研攻关组下设的药物研发专班要尽快组织专家组研讨并提出初步意见；推荐进入临床研究的，由科研攻关组及时将推荐意见转至科研攻关组副组长单位国家卫生健康委的科教司，由国家卫生健康委科教司统一协调医疗机构承接临床研究任务。开展相关药品临床研究的医院应当按照《医疗机构开展临床研究项目管理办法》的要求进行伦理审查、立项，按要求备案，并在医学研究登记备案信息系统（网址：http://114.255.48.20）上传有关信息。医院要提供条件保障伦理委员会紧急独立开展伦理审查。伦理委员会要提高审查效率，在保障伦理审查质量的前提下，加强指导和支持，简化文档要求。各级卫生和科技行政部门应当加强统筹协调，促进数据整合，提高研究效率。

三、促进相关药品临床研究规范开展

开展相关药品临床研究，应当坚持治疗优先、疫情防控优先，坚决防止因研究影响患者治疗、影响整体疫情防控工作开展。对创新性强以及风险较高的项目，要加强科学性审查和风险评估。开展相关临床研究活动应有适当经费保障，要参考《药物临床试验质量管理规范》开展全过程质量控制和风险管控。医院根据需要可聘请独立于药品供应方、参与临床研究工作的医务人员和患者的数据安全监查委员会，委员会可以在临床研究结束之前定期对研究进展情况进行评判，如发现试验组有明显的毒副作用，或者无明确的治疗效果，立即向医疗机构报告，医疗机构提前终止研究，并及时上报联防联控机制科研攻关组；对疗效明确的则应促进药品尽快推广使用，以尽快使更多患者受益。对未设独立的数据安全监查委员会的，医疗机构要随时关注药物可能的毒副作用，如有明显毒副作用或无明确治疗效果，应立即终止临床研究，切实保障受试者的权益。

四、推动临床研究成果的应用

各级卫生和科技行政部门要及时收集辖区内医疗机构相关药品临床研究工作进展情况，汇总临床研究结果，上报联防联控机制科研攻关组。联防联控机制科研攻关组组织药物研发专班统一汇总相关研究信息，初步审查后，将效果较好的药品推荐至联防联控机制医疗救治组（国家卫生健康委医政医管局），由医疗救治组组织专家研究决定是否扩大使用范围或纳入诊疗方案。疫情防控期间，由科研攻关组建立药品临床研究信息的统一发布机制。

请各级卫生和科技行政部门及时将此通知转至辖区内相关医疗机构和科研机构遵照执行，并将已开展的相关药品的临床研究，按本通知要求履行相关报备程序。经药品监管部门批准的新冠肺炎适应症的药物临床试验，需要依法依规开展，不在本通知范围内。

国务院应对新型冠状病毒感染的肺炎

疫情联防联控机制科研攻关组

（科技部办公厅代章）

2020年2月24日

（197）卫健委：印发基层医疗卫生机构在新冠肺炎疫情防控期间为老年人 慢性病患者提供医疗卫生服务指南（试行）	国卫基层家医便函〔2020〕2号

各省、自治区、直辖市及新疆生产建设兵团卫生健康委基层处：

为指导基层医疗卫生机构在新冠肺炎疫情防控期间为老年人、慢性病患者更好地提供医疗卫生服务，结合《国家基本公共卫生服务规范（第三版）》和国家卫生健康委有关疫情防控的政策措施，根据疫情防控工作需要，制定了《基层医疗卫生机构在新冠肺炎疫情防控期间为老年人 慢性病患者提供医疗卫生服务指南（试行）》。现印发给你们，请根据实际情况，参考使用。

国家卫生健康委基层司

2020年2月24日

（信息公开形式：主动公开）

基层医疗卫生机构在新冠肺炎疫情防控期间为老年人 慢性病患者提供医疗卫生服务指南（试行）

一、规范诊疗服务

（一）合理布局候诊区域，增加候诊椅配置，控制候诊患者间隔1米以上距离，做好患者预检分诊，加强通风换气和内部清洁消毒，防止院内感染。

（二）合理安排门诊时间，保证正常诊疗秩序，积极推行网上预约、电话预约等方式，实行错峰诊疗，减少就诊患者排队聚集。

（三）优化挂号、诊疗、检查、取药等服务流程，减少患者待诊时间及患者间接触风险。

（四）开展长期处方服务，对诊断明确、病情稳定的慢性病患者，经家庭医生评估后，根据需要制定长期药物治疗方案，给予长期处方服务。

（五）有条件的基层医疗卫生机构可对确有实际困难的高龄、失能老年人及行动不便的慢性病患者提供上门巡诊、家属代取药等服务。

二、优化健康管理服务

（六）在“网格化”健康管理基础上，充分运用微信、手机APP等信息化手段与老年人、慢性病患者或其家属（照护人员）建立有效的互动沟通渠道，开展随访服务，及时掌握老年人、慢性病患者健康状况，督促其加强血压、血糖自我监测，并进行针对性指导。

（七）指导老年人、慢性病患者保证充足睡眠，均衡营养，居家适度活动，根据自身条件及环境选择广播体操、八段锦等运动。

（八）加强老年人、慢性病患者心理疏导，引导其树立既要高度重视，又不过分恐慌的防控观念，提高自我防范意识。

（九）暂缓安排老年人、慢性病患者的年度体检工作，待疫情防控结束后，视情况逐步恢复相关工作。

三、加强疫情防控宣传教育

（十）鼓励老年人、慢性病患者及其家属利用多种媒介，了解新冠肺炎防控知识技能、疫情动态与相关政策，全面、正确看待疫情发展。

（十一）教育老年人、慢性病患者尽量居家减少外出，做好室内消毒通风，避免走亲访友等参加聚集性活动。确需外出时，要规范佩戴口罩，加强手卫生，做好个人防护，并避免乘用人员密集交通工具。

（十二）告知老年人、慢性病患者出现发热、咳嗽、鼻塞、头痛、乏力、气促、结膜充血或消化道症状时，立即按规定报告并做好隔离控制，及时转送发热门诊就诊。

（198）民政部：印发《新冠肺炎疫情高风险地区及被感染养老机构防控指南》

各省、自治区、直辖市民政厅（局），各计划单列市民政局，新疆生产建设兵团民政局:

为切实加强养老机构防控新冠肺炎疫情的分类管理工作，现印发《新冠肺炎疫情高风险地区及被感染养老机构防控指南》。本指南适用于两类养老机构：一是湖北省武汉市等疫情高风险地区的养老机构；二是非高风险地区已出现感染病例的养老机构。非高风险地区未出现感染病例的养老机构继续实施《养老机构新冠肺炎疫情防控指南》（第二版），也可以根据本指南加强相关措施。

联系人：唐潇鹤 010-58123155

附件：新冠肺炎疫情高风险地区及被感染养老机构防控指南

民政部办公厅

2020年2月25日

（199）文化和旅游部：印发《公共图书馆、文化馆（站）恢复开放工作指南》	公共函〔2020〕1号

各省、自治区、直辖市文化和旅游厅（局），新疆生产建设兵团文化体育广电和旅游局：

现将《公共图书馆、文化馆（站）恢复开放工作指南》印发给你们，请结合实际抓好贯彻落实。

特此通知。

文化和旅游部公共服务司

2020年2月25日

公共图书馆、文化馆（站）恢复开放工作指南

为落实中央关于统筹推进新冠肺炎疫情防控和经济社会发展工作的有关精神，指导全国公共图书馆、文化馆（站）继续实施疫情防控、稳步做好恢复开放相关工作，根据国务院应对新型冠状病毒感染肺炎疫情联防联控机制印发的《企事业单位复工复产疫情防控措施指南》，特制定本工作指南。

一、恢复开放的总体要求

（一）坚持分区分级，不搞“一刀切”。疫情高风险地区的公共图书馆、文化馆（站）继续闭馆。疫情中风险和低风险地区的公共图书馆、文化馆（站）恢复开放工作，由当地党委政府决定。

（二）坚持防疫优先，做好恢复开放的预案和准备工作。各级公共图书馆、文化馆（站）要继续把疫情防控放在首位，毫不放松地抓好防控工作。拟恢复开放的场馆，要提前做好预案，制定科学可行的具体措施，并根据当地防控部门最新指导意见及时进行动态调整，确保安全。场馆及邻近区域出现确诊或疑似病例的暂不恢复开放。

二、加强员工健康监测和管理

（三）做好员工健康监测和报告。按照当地要求做好员工健康管理，掌握员工出行轨迹等情况，对去过疫情严重地区的人员实行居家隔离观察。员工入馆（站）前要检测体温，出现发热、呼吸道症状时，要及时报告。

（四）指导员工做好个人防护。员工应减少不必要外出，避免去人群聚集场所；在人员密集的地方，应按照《不同人群预防新型冠状病毒感染口罩选择和使用技术指引》要求，正确佩戴口罩等防护用品。

（五）减少员工聚集。根据实际采取错时上下班、弹性工作制或居家办公方式。加强员工用餐管理，实行错峰就餐，有条件时使用餐盒、分散用餐。减少召开会议，必须召开的会议要缩短时间、控制规模，参会人员佩戴口罩并保持安全距离。

三、做好场馆防控工作

（六）分区域分项目恢复开放服务。各场馆要根据实际，分区域分项目逐步恢复开放，对不符合开放条件的场地以及容易形成人员聚集的项目等，应暂不开放。

（七）配备防护物资。各场馆要配备口罩、手套、消毒液、洗手液等防护物资，为员工和到馆群众提供必要防护保障。

（八）保持场馆通风。各场馆要保持通风状态，在条件允许情况下首选自然通风。如使用空调，应当确保供风安全充足。

（九）加强清洁消毒。各场馆的阅览室、文化活动室、展览厅、会议室、报告厅、餐厅、卫生间等区域以及图书期刊、自助借还机、演出器材、展览设备等，要进行定期消毒。

（十）加强疫情防控知识宣传。各场馆通过设置提示牌、LED显示屏播放等多种方式，加强疫情防治知识科普宣传，使员工和到馆群众充分了解掌握防治知识、支持配合防控工作。

（十一）持续做好线上服务。各级公共图书馆、文化馆（站）在逐步恢复场馆服务的同时，要继续通过公共文化云等数字平台，加强内容更新，为群众提供优质公共数字文化服务。

四、加强对群众进出场馆的管理和服务

（十二）加强人员进出管理。各场馆要安排专人对所有进出通道严格管理，对进入人员进行登记；确保进馆人员佩戴口罩，并在入口处接受体温检测，体温正常方可进入；体温超过正常温度的，应禁止其入内并及时上报疫情防控部门；对不配合或干扰防疫工作的，要依法依规报告相关部门处置。

（十三）采取人员限流措施。各场馆可视情况实行实名预约服务，结合防控工作需要，对每日入馆人数进行总量控制。

（十四）避免形成人员聚集。各场馆要对到馆群众进行疏导、分流，阅览室、文化活动室、展厅等公共空间，人与人之间要保持安全距离。疫情防控期间，原则上不举办线下讲座、培训、演出、比赛等人员聚集活动。

五、做好异常情况处置

（十五）明确场馆防控责任。各场馆主要负责人是疫情防控第一责任人，要建立完善内部疫情防控组织体系，明确疫情防控应急措施和处置流程，把防控责任落实到部门和个人。

（十六）做好发现疫情的应对处置。发现疑似或确诊病例的场馆，要立即配合疾控部门采取隔离措施，加强密切接触者追踪、疫点消毒等工作，暂时关闭场馆。

（200）交通运输部：加强中欧班列运行保障工作	交规划明电〔2020〕74号

各省、自治区、直辖市、新疆生产建设兵团交通运输厅（局、委）：

为深入贯彻落实党中央、国务院决策部署，统筹做好新冠肺炎疫情防控和经济社会发展工作，发挥交通运输“先行官”作用，打通“大动脉”，畅通“微循环”，推动中欧班列在疫情期间发挥更好的战略通道作用，促进中欧班列高质量发展，现就有关事项通知如下：

一、在疫情防控期间，将中欧班列集装箱运输车辆纳入应急运输“绿色通道”，根据《交通运输部 国家卫生健康委关于切实简化疫情防控应急运输车辆通行证办理流程及落实对应急运输保障人员不实行隔离措施通知》（交运明电〔2020〕57号）等文件规定，按照统一通行证式样，由承运单位或驾驶人员自行打印、自行填写、随车携带，保障中欧班列集装箱运输车辆不停车、不检查、不收费，优先便捷通行。

二、按照《国务院办公厅关于做好公路交通保通保畅工作确保人员车辆正常通行的通知》等要求，各地要组织集卡车司机和相关人员尽快返岗，对短期向疫情重点区域运送物资的中欧班列集装箱运输车辆司机、装卸工等提供保障的人员，经过体温检测符合规定的，在采取戴口罩等必要防护措施的前提下，原则上不需采用隔离14天的措施。

三、地方交通运输主管部门加强与中欧班列运营平台公司和相关企业的工作对接，做好运输组织保障，促进道路运输、水运与铁路有效衔接，推动港航与铁路间信息交换共享。鼓励港航企业与铁路企业加强合作，促进集装箱海运与铁路相互调运。

四、按照国务院应对新型冠状病毒感染肺炎疫情联防联控机制关于科学防治精准施策分区分级做好新冠肺炎疫情防控工作的指导意见，分区分级精准防控，有序推动物流园区等物流枢纽节点复工复产，特别是涉及中欧班列的重要物流枢纽节点，优先推动复工复产。

五、加强部门协作，提升中欧班列运输便利化水平，减少中转换装，降低损耗，提高中欧班列货物运输服务水平和运营效率。

六、按照“远近结合、标本兼治、提质增效”的工作思路，梳理中欧班列安全稳定运行中涉及枢纽节点、“最后一公里”等的问题，结合“十四五”综合交通运输发展规划编制，谋划推动一批大型枢纽站点集结中心（物流园区）、铁路专用线项目等规划建设，优化中欧班列开行总体布局。加强西部陆海新通道、中欧陆海快线与中欧班列的衔接，促进形成联动发展格局。

七、铁路局要加强与铁路合作组织的沟通联系，并充分发挥与俄罗斯、哈萨克斯坦等国政府铁路主管部门合作机制的作用，保障中欧班列国际运输正常化。邮政局要推动做好中欧班列运邮工作。

交通运输部

2020年2月24日

（201）工信部：印发《关于有序推动工业通信业企业复工复产的指导意见》	工信部政法〔2020〕29号

各省、自治区、直辖市及计划单列市、新疆生产建设兵团工业和信息化主管部门：

为深入贯彻习近平总书记关于统筹推进新冠肺炎疫情防控和经济社会发展工作的重要指示精神，落实国务院联防联控机制《关于切实加强疫情科学防控有序做好企业复工复产工作的通知》要求，在确保疫情防控到位的前提下，推动非疫情防控重点地区企业复工复产，努力实现今年工业通信业发展目标任务，现提出如下意见。

一、总体要求

以习近平新时代中国特色社会主义思想为指导，增强“四个意识”，坚定“四个自信”，做到“两个维护”，把党中央、国务院各项决策部署抓实抓细抓落地，统筹做好疫情防控和经济社会发展工作。坚持突出重点、统筹兼顾，分类指导、分区施策，加强产业链协调，充分运用新一代信息技术，全面支持疫情科学防控和企业复工复产。坚决杜绝疫情防控和企业复工复产工作中的形式主义、官僚主义做法，主动担当，积极作为，深入基层，服务企业，抓紧抓实抓细各项措施落实，为基层解决实际困难，切实帮助企业做好复工复产工作。

二、全力保障医用防护物资供给

要把疫情防控物资保障作为当前工作的重中之重，重点开展医疗救治急需的呼吸机、心电监护仪等医疗设备和治疗药品的组织生产和及时供应，全面提升医用防护服、口罩等防护物资的生产保供能力，统筹协调医用物资全产业链企业同步复工复产，增强企业生产柔性，科学谋划产能，确保产品质量，强化物资保障协调机制，为打赢湖北和武汉保卫战，以及全国疫情防控全面胜利提供坚强有力的物资保障。

三、切实帮助企业做好疫情防控工作

指导企业落实《企事业单位复工复产疫情防控措施指南》，督促落实疫情防控措施，做好通风、消毒、体温检测等防控工作，建立必备的卫生设施，加强员工健康监测，强化日常防控管理，防止发生聚集性疫情。运用大数据、人工智能等新一代信息技术，做好复工复产企业跟踪监测，对疫情防控实施零报告制度。要杜绝“填表抗疫”“作秀留痕”等形式主义做法，切实解决企业实际问题。

四、加大中小企业扶持力度

按照《工业和信息化部关于应对新型冠状病毒肺炎疫情帮助中小企业复工复产共渡难关有关工作的通知》（工信明电〔2020〕14号）要求，指导企业用好用足现有财税、金融、社保等优惠政策。继续研究出台阶段性、有针对性的减税降费政策，帮助中小微企业渡过难关。鼓励中央企业、大型国企等龙头企业发挥表率作用，帮助中小企业开展应收账款融资，带动产业链上下游中小企业复工复产，协同开展疫情防控和生产恢复。针对中小企业现金流不足的突出问题，落实金融支持政策，帮助企业缓解融资困难。继续加大力度推动清理拖欠民营企业中小企业账款工作，缓解企业资金压力。

五、加紧推动民生必需品生产企业复工复产

加快化肥、农膜、农机装备整机及零部件等涉农企业复工复产，做好春耕备耕物资供应，确保农业生产不误农时。推动食品、日用品等生活必需品企业复工复产，保障民生物资供给稳定。充分利用电商、信息服务等平台，促进产业链供需线上对接，鼓励开展农机等设备的在线维护。

六、推动重点行业企业复工复产

优先支持汽车、电子、船舶、航空、电力装备、机床等产业链长、带动能力强的产业。继续支持智能光伏、锂离子电池等产业以及制造业单项冠军企业，巩固产业链竞争优势。重点支持5G、工业互联网、集成电路、工业机器人、增材制造、智能制造、新型显示、新能源汽车、节能环保等战略性新兴产业。大力提升食品包装材料、汽车零部件、核心元器件、关键电子材料等配套产业的支撑能力。

七、推进重大项目开工复工

积极扩大国内有效需求，发挥重大项目、重点工程建设示范带动作用，加快在建和新开工项目建设进度，带动工程机械、原材料等企业复工复产及人员返岗就业。结合本地区实际，围绕民生就业、产业基础能力、未来产业竞争制高点等重点方向，启动一批投资规模大、带动能力强的重大项目和重点工程。协调解决重大外资项目复工复产遇到的问题，推动重大外资项目落地。

八、大力促进市场消费提质扩容

支持新业态新模式，丰富5G+、超高清视频、增强现实/虚拟现实等应用场景，推动发展远程医疗、在线教育、数字科普、在线办公、协同作业、服务机器人等，带动智能终端消费。积极稳定汽车等传统大宗消费，鼓励汽车限购地区适当增加汽车号牌配额，带动汽车及相关产品消费。加大生物医药、智能健康管理设备、高端医疗器械、医疗机器人、公共卫生智能监测检测系统等大健康产业投入力度，满足人民群众的健康需求。

九、打通人流、物流堵点

落实分区分级精准防控策略，保障人员有序流动和物流畅通，推动产业链各环节协同复工复产。指导企业用好用足援企稳岗政策，发挥信息化手段优势，积极开展精准对接，协调打通企业员工返岗通道，提高员工返岗率。主动对接有关部门，将重点行业重点企业生产原料、零部件、产品纳入绿色物流通道，保障运输畅通。搭建跨区域人员、物资对接平台，进行精准服务，统筹解决园区、产业集群内企业人员返岗、生产物资运输等问题，推动企业抱团恢复生产。

十、加强分类指导

对低风险地区，落实好相关防控措施，简化程序，强化服务，指导企业复工复产，全面恢复正常生产生活秩序；对中风险地区，在做好疫情防控前提下，合理安排企业复工复产，尽快有序恢复正常生产生活秩序；对高风险地区，要继续集中精力抓好疫情防控工作，根据疫情态势逐步恢复生产生活秩序。要选派优秀干部下沉一线，对重点企业跟踪服务，指导企业复工复产。

工业和信息化部

2020年2月24日

（202）司法部：推动严格规范公正文明执法为疫情防控工作提供有力法治保障

为深入贯彻落实习近平总书记关于新冠肺炎疫情防控工作重要讲话和重要指示批示精神，贯彻落实党中央、国务院关于疫情防控工作决策部署，充分发挥行政执法协调监督职能，大力推动严格规范公正文明执法，维护经济社会发展秩序，保护人民群众的生命安全和身体健康，保障疫情防控工作顺利开展，制定本意见。

一、提高政治站位。各级司法行政机关要认真学习贯彻习近平总书记关于新冠肺炎疫情防控工作重要讲话和重要指示批示精神，把思想和行动统一到习近平总书记重要讲话和重要指示批示精神，统一到党中央、国务院关于疫情防控工作决策部署上来，切实增强“四个意识”、坚定“四个自信”、做到“两个维护”，把疫情防控工作作为当前工作的重中之重，抓紧抓实，用足用好法律法规规定，坚持执法为民，坚持严格规范公正文明执法，加强行政执法监督，为坚决打赢疫情防控阻击战提供有力法治保障。

二、坚持严格执法。组织推动各级行政执法机关依法惩处各类抗拒疫情防控措施、暴力伤医、野生动物非法交易、扰乱市场秩序和社会秩序等违法行为，做到有法必依、执法必严、违法必究。坚持重典治乱，对阻碍和破坏强制隔离、封闭场所、交通检疫等法定防控措施实施，殴打伤害医务人员、扰乱医疗救治秩序，已感染新型冠状病毒肺炎人员故意传播病毒，利用疫情囤积居奇、哄抬物价、牟取暴利、欺骗消费者，非法捕杀、收购、运输、出售野生动物及其制品，生产、销售假冒伪劣疫情防控药品、伪劣口罩、手套、消毒水等防治、防护产品和物资，造谣传谣制造混乱，假借疫情非法募捐、非法挪用、截留或者侵占捐赠款物，擅自改变捐赠物资用途等违法行为，要根据疫情防控特殊需要，加大行政执法力度，严厉打击，该严的要严、该重的要重、该快的要快，涉嫌构成犯罪的，要及时移送司法机关处理，切实维护正常经济社会秩序，为疫情防控工作提供强有力法治支撑。

三、坚持规范执法。全面提高依法防控、依法治理能力，推动各级行政执法机关严格依照法定权限和程序执法，对疫情防控中出现的各类矛盾和问题，依法应对、妥善处理，确保各项疫情防控工作在法治轨道上统筹推进、顺利开展。要强化执法指引，指导各相关部门认真梳理疫情防控工作涉及的行政执法法律法规及为应对疫情制定的各种制度措施，为行政执法机关严格执法提供准确的执法依据、标准、程序，做到依法履职、执法有据。坚守法治底线，在采取强制隔离、封闭场所、交通检疫等防控措施和应急处理措施时，要严格依照传染病防治法及其实施办法、突发事件应对法、野生动物保护法、动物防疫法、突发公共卫生事件应急条例等法律法规，以及地方和部门针对疫情防控工作需要制定的地方性法规、规章、行政规范性文件的有关规定要求，依法审慎作出决策。疫情防控措施要与疫情可能造成的社会危害的性质、程度和范围相适应，避免过度执法，尽量最小程度地减损公民、法人和其他组织的权益。未经批准，不得擅自采取设卡、断路、阻断交通等行为，不得“一刀切”，采取一律封闭管理、强制劝返等极端措施。要依法有序推动企业复工复产，对符合复工复产要求的，不得限制和影响企业正常生产经营。在行政征用时，要遵循依法、必需、合理的原则，履行法定审批程序，并依法及时给予被征用人相应补偿;征用相关场地、设备、物资的，要登记造册、尽到保护义务，使用完毕后及时返还。要严格执法程序，认真落实行政执法公示制度、全过程记录制度、重大执法决定法制审核制度，确保执法行为于法有据、有据可查，行政执法决定合法有效。

四、坚持公正执法。把公正执法理念贯穿行政执法全过程，做到执法规范、裁量公正、处罚公平，让人民群众在具体执法案件中感受到公平正义。坚持法治统一，严格按照法律法规规章规定的行政处罚种类、幅度进行裁量，杜绝执法随意、执法不公。坚持过罚相当，综合考虑违法行为的性质、情节、社会危害程度以及执法相对人的悔过态度等情形，依法给予相应处罚，防止和避免同事不同罚、处罚畸重畸轻、显失公平公正、过度执法等情形，不得在行政执法中对外地企业和公民区别对待。坚持教育与惩戒相结合的原则，在疫情防控时期，对违法情节显著轻微及社会危害程度不大的违法行为，可以考虑先不予处罚或者暂缓执行处罚，以教育劝导为主。

五、坚持文明执法。推动、监督各级行政执法机关积极践行执法为民理念，尊重行政相对人的基本权利和合法权益，确保行政执法法律效果和社会效果的统一。进一步改进办案方式方法，注重语言规范、行为规范，杜绝粗暴执法、生硬执法，避免激化矛盾，引发对立冲突。严禁以执法名义打击报复行政相对人，严禁乱收费、乱罚款、乱摊派。要善于释法说理，做到以法为据、以理服人、以情感人，减少执法阻力，赢得群众对执法工作的理解和支持。要充分考虑疫情防控时期的公众情绪，避免引发社会舆情。要服务大局，从有利于疫情防控和经济社会发展的角度出发开展行政执法活动，决不能就办案而办案、机械办案，严重影响和破坏疫情防控工作秩序和经济社会发展大局。

六、创新执法方式。推动行政执法机关牢固树立服务意识，根据疫情防控工作和有序推进复工复产需要，创新工作方法，及时调整面向单位和个人的政务服务方式，做到网上办、不见面、保安全、能办事，确保工作高效与合法相统一。具备网上在线申办条件的，要坚持网上在线办公，通过政府官网、微信公众号、掌上政务办事客户端等方式公布防控期间的办理指南和各业务窗口咨询联系方式，引导企业和群众采取网上申报的方式办理，尽量不去政务服务中心现场。对行政许可涉及的申报、受理、送达等行为，鼓励优先采用网上申报、网上审批、证照快递送达等“不现场接触”的方式办理。加快推动行政执法信息化建设，从执法程序的启动到执法决定的送达，凡是能通过电子方式办理的，一律通过电子方式办理;如有紧急特殊事项，确需现场办理的，可以拨打相关电话或通过网站进行预约，并在指定时间和指定场所办理，即办即走，最大限度减少滞留时间，尽量做到“不接触”。要简化工作流程，对与疫情防控和有序推进复工复产工作有关的紧急事项，要按照法定程序急事急办;对因疫情防控，行政许可事项不能在法定期限作出的，可以依法延长办理期限。对行政许可到期重新申请的，在审核符合有关条件的基础上，可以先自动延期，疫情结束后，再补办相关手续。无法直接提交有关样品和报告的，可以先就有关事项提交承诺书，疫情结束后，补交相关样品和报告。事后发现提交虚假材料的，将撤销相关许可。行政决定不能在法定期限作出或者送达的，可以依法延期作出决定或者送达。对在疫情防控期间发现的可进一步取消的行政事项或者优化的行政程序，可以提出相关意见建议。对要求企业、群众开具有关证明事项的，也要参照上述原则办理。

七、加强执法协调。建立健全行政执法协作机制，组织推动各相关行政执法机关认真履职、相互配合、密切协作，形成工作合力，防止执法不力、相互推诿，贻误疫情防治工作顺利开展。疫情防控期间，县级以上地方人民政府及其部门对本行政区域内疫情防控的有关执法工作负责;涉及两个以上行政区域执法的，由有关行政区域共同的上一级人民政府负责，或者由各有关行政区域的上一级人民政府共同负责。要整合基层执法力量，必要时可以跨地区、跨部门、跨层级统筹使用，各执法部门要服从统一指挥，做好联动响应、信息共享、协同监管工作，做到违法线索互告、执法监管协同、执法结果互通。组织推动公安、卫生健康、农业、市场监管、林业草原、交通运输等部门加强联合执法，提高执法效率，避免重复执法。进一步完善将行政执法中查办的涉嫌犯罪案件移送司法机关处理的工作机制，对涉嫌犯罪的，要及时按照《行政执法机关移送涉嫌犯罪案件的规定》等规定，移送公安机关立案查处，坚决防止以罚代刑、有罪不究、降格处理等现象发生。

八、强化执法监督。各级司法行政机关要认真履行本级政府赋予的行政执法监督职责，建立完善行政执法争议的协调和违法执法行为联合处理机制，增强行政执法监督效能。要加强对本地疫情防控相关执法工作的监督指导，督促指导行政执法机关依法做好各项疫情防控工作，及时协调研究解决执法中产生的法律争议和问题。高度重视社会舆情，健全投诉举报处理机制，充分运用现有行政执法监督平台，对群众和企业反映强烈的不作为、乱作为、简单粗暴以及过度执法等问题，接到举报后，要及时转送有关执法机关进行处理，做到行政执法活动可投诉、可追溯、可监督。各行政执法单位均应对外公布行政执法监督渠道和联系方式，并安排专人负责，对涉及重大疫情防控有关行政执法活动的投诉举报要及时复核、及时回复。要强化对基层群众自治组织、企事业单位等非行政执法机关及其疫情防控人员的指导、培训和监督，防止其实施过度疫情防控措施。执法监督中发现相关案件在事实认定、法律适用、处罚裁量等方面存在错误或者明显不当的，要及时提出监督意见，督促有关行政执法机关及时予以纠正，探索建立行政执法案例指导制度。

九、严格执法责任。要推动各级行政执法机关严格落实执法责任制，确保党中央、国务院以及各地方各部门制定的疫情防控措施落地见效。要严格界定执法权限，明确相应责任，推动建立健全“执法有依据、行为有规范、权力有制约、过程有监督、违法有追究”的行政执法责任制度体系。建立健全责任倒查机制和追究机制，对疫情防控执法过程中产生的执法不力、执法不公甚至是执法腐败等问题，该追责的严格依法追责。对作风漂浮、落实不力、隐瞒真相、弄虚作假，甚至干扰、妨碍、抗拒有关疫情防治工作的失职渎职人员，要依法从严追究责任。需要追究党纪政纪责任的，要依法移送纪检监察机关处理。涉嫌犯罪的，要依法移送司法机关处理。

十、提升执法保障。严格按照行政执法工作有关要求，协调有关部门服务保障疫情防控执法一线工作需要，配足配齐行政执法设备和防护装备，加强口罩、测温仪、消毒用品等物资筹措和调配，以及就餐、值夜、交通等联防联控的后勤保障，确保行政执法活动正常规范有序开展。要合理调度执法一线疫情防控力量，加强对执法人员的健康检查、业务指导、上岗培训和安全保护，坚决防止带病上岗。要保障行政执法人员人身安全，对妨碍公务、抗拒执法等违法犯罪行为要依法打击，切实维护执法权威。要关心爱护执法人员，对执法人员的合法权益要给予充分的保障，对在疫情防控工作中表现突出的，要给予表彰奖励。

各级司法行政机关要充分发挥在本地疫情防控法治工作中的组织推动、执法协调监督和参谋助手作用，对疫情防治工作中出现的行政执法共性法律问题加强研究，对涉及疫情防控的重大行政决策和重大行政执法决定等事务做好合法性审查，确保出台的有关决策措施合法有效。

2020.2.25

（203）国务院应对新型冠状病毒肺炎疫情联防联控机制：依法科学精准做好新冠肺炎疫情防控工作	联防联控机制发〔2020〕28号

各省、自治区、直辖市及新疆生产建设兵团应对新型冠状病毒肺炎疫情联防联控机制（领导小组、指挥部）：

为贯彻落实国务院应对新型冠状病毒肺炎疫情联防联控机制关于科学防治精准施策分区分级做好新冠肺炎疫情防控工作的指导意见，进一步提高新冠肺炎疫情防控工作的科学性、精准性，依据《中华人民共和国传染病防治法》、《突发公共卫生事件应急条例》等法律法规，现就做好防控工作有关事项通知如下：

一、 总体要求

根据当前疫情防控形势发展趋势变化，突出重点、统筹兼顾、分类指导、分区施策，坚持依法防控、科学防治、精准施策，加强重点人群、重点场所管控，着力抓实抓细各项措施，提高疫情防控的科学性、精准性和针对性。

二、 具体措施

（一）加强人员社会管控，严防输入和扩散风险。

1.实行人员分类管理。根据居民近期旅行史或居住史、目前健康状况、病例密切接触史等判断其传播疾病风险，将居民划分为高风险、中风险、低风险人员，采取针对性的管控措施。高风险人员在定点医疗机构、定点医学观察机构或居家实施严格的隔离治疗或医学观察，相关机构和社区对其进行严格管控。中风险人员严格落实居家隔离医学观察要求，自觉接受社区管理。低风险人员体温检测正常可出行和复工。对疫情特别严重的湖北省继续采取最严格的防控措施，已实施交通管控的武汉市和湖北省其他地市，严控人员输出；未实施交通管控的地市，人员抵达目的地后一律集中隔离14天。

2.有效落实“四早”措施。有关部门、医疗卫生机构要认真落实传染病早发现、早报告、早隔离、早治疗的“四早”措施，切断传播途径，防止扩散。要做好新冠肺炎病例、聚集性疫情、社区疫情的监测和报告，鼓励单位和个人发现、报告相关病例和疫情。要强化实验室检测和诊断，切实提升检测质量和诊断时效。要综合运用流行病学调查和大数据分析方法，及时发现可疑病例、密切接触者并进行追踪管理。各地要指定发热门诊、定点收治医院开展发热病人筛查，及时诊断并隔离治疗新冠肺炎病例，做到“应检尽检”、“应收尽收”、“应治尽治”，防止漏诊、误诊，防止轻症转重症。

3.鼓励实行动态健康认证。鼓励有条件的地区推广个人健康码等信息平台，不具备信息化条件的地区可采用个人健康申报等方式，居民通过网络平台申领电子健康码或通过社区申领纸质版健康码（健康通行卡），获得出行、复工等资格。政府有关部门、用人单位、社区等综合判断个人健康风险等级，实现特殊时期动态健康认证。

（二）做好重点场所防控，严防扩散风险。

1.落实社区防控责任。充分发挥社区动员能力，实行网格化、地毯式管理，责任到人，联系到户，确保各项防控措施切实落实、不留死角。针对未发现病例的社区，实行“外防输入”的策略，做好组织动员、健康教育、信息告知、重点地区和高风险地区返回人员管理、环境卫生治理、物资准备等工作。针对出现病例或暴发疫情的社区，实行“内防扩散、外防输出”的策略，在采取上述措施基础上，还应当做好密切接触者管理和消毒等工作。针对出现疫情传播的社区，实行“内防蔓延、外防输出”的策略，进一步实行疫区封锁、限制人员聚集等措施。

2.落实用工单位防控责任。用工单位严格落实复工复产疫情防控要求，做好返岗员工登记报备并建立员工健康台账。对于需要接受隔离医学观察但无相关症状的员工，经检测筛查排除感染，可适当缩短隔离时间，在做好防护措施的情况下提前返岗。做好办公场所、工区及公共区域、职工宿舍的通风消毒、环境清理等工作，为员工配备必要的个人防护用品。实行“进出检”制度，做好员工日常体温检测和健康监测。实施分区作业、分散错峰就餐，控制会议频次和规模，尽量减少人员聚集。鼓励具备条件的企事业单位采取错时上下班、弹性工作制或居家办公方式。单位应当设立隔离观察区域，员工出现可疑症状时应当及时隔离并安排就近就医，配合当地疾控部门做好病例报告、流行病学调查、相关区域封闭消毒等工作。

3.落实院校防控责任。各地根据疫情发展情况确定开学时间，严禁学生提前返校。院校开学前做好预案和监测设备准备、隔离空间预备、环境卫生改善等工作。开学后学校医务室加强监测，对来自疫情防控重点地区、和确诊病人有过接触以及有相应症状的学生，采取单独隔离措施。开展“晨午晚检”，实行“日报告”、“零报告”制度，加强因病缺勤管理，对因病缺勤学生和教职员工及时追访和上报。校园实行封闭管理，禁止校外人员进入，不组织大型集体活动。学生和教职员工如出现发热、乏力、干咳等可疑症状，应当及时隔离并安排就近就医，发现病例的院校，要及时向辖区疾控机构或医疗机构上报，积极配合做好流行病学调查，以班级为单位，确定防控管理场所，排查甄别密接人员，严格采取消毒隔离等针对性防控措施。

4.加强公共服务类场所防控。对农贸市场、商场、超市等生活必需类场所及酒店、宾馆等生活服务类场所，在精准有序推动开业的同时，严格落实环境卫生整治、消毒、通风、“进出检”、限流等措施，商超物品尽量提前包装标价，推荐顾客自助购物、自助结算，缩短排队等候时间。提供住宿服务的经营单位要如实登记旅客信息，对来自疫情防控重点地区的旅客进行排查并及时报告当地疾控机构，按照疾控机构的指导采取相应措施。对公共交通工具和机场、车站、码头等人员密集场所，按要求设立留验站，配备必要人员设备，严格落实体温筛检等防控措施，发现可疑人员应当劝阻其登乘，进行暂时隔离，并立即通知检疫部门或当地卫生健康部门及时处置。

5.加强特殊场所疫情防控。对监管场所、养老机构、福利院、精神卫生医疗机构等特殊场所，重点防控输入性疫情和内部疾病传播。要开展预防性卫生措施，全面排查入监干警职工、养老机构、福利院、精神卫生医疗机构工作人员等，落实体温检测和健康监测制度，禁止有可疑症状的人员上岗。要密切关注服刑人员、机构老年人、儿童、精神障碍患者的健康状况，出现新冠肺炎可疑症状，应当立即隔离观察并及时送医排查。要做好防控物资配备，加强日常消毒和环境卫生，加强个人卫生防护。出现确诊、疑似病例，应对其可能活动场所开展全面消杀，规范处置个人物品，对其密切接触者按要求进行集中隔离医学观察。

6.加强农村疫情防控。充分发挥农村基层党组织、村民自治组织以及乡镇卫生院、村卫生室作用，组织动员农民群众开展群防群控。减少集市等人群聚集活动，做好出外打工人员防疫常识教育。对乡镇（涉农街道）和村组实行网格化管理，对发现病例的县，对疫点进行终末消毒和环境卫生清理，除有病例村组外，允许其他村组村民有序出行。具备条件且防控措施到位的乡村旅游场所，可逐步有序对外开放，经营主体落实防控责任，确保游客和工作人员健康安全。

三、加强组织领导

（一）强化责任落实。各地各级要增强大局意识，统筹抓好疫情防控和经济社会发展，压紧压实属地责任、部门责任、单位责任、家庭责任、个人责任，依法依规落实科学防控、精准施策总要求，推动由全面防控向精准防控、重点防控转变。

（二）强化信息通报。各省、自治区、直辖市人民政府要尽快公布当前本省低风险、中风险、高风险县（市、区、旗）名单，落实分区分级管控要求，并将动态调整的风险地区名单作为疫情防控工作措施日报告内容及时报送国家卫生健康委。

（三）强化宣传引导。切实加强传染病防治法等法律法规宣传，引导全社会在法治轨道上统筹推进各项防控措施。及时回应媒体关切，引导群众切身感受疫情发展的向好趋势。全面做好政策解读，进一步凝聚民心、坚定信心、稳定人心。

（四）强化监测评估。动态评估防控成效，及时调整防控策略，全面提高建章立制规范性、风险识别合理性、措施落实精准性。

国务院应对新型冠状病毒肺炎疫情

联防联控机制

2020年2月24日

（204）人社部 扶贫办：应对新冠肺炎疫情进一步做好就业扶贫工作

各省、自治区、直辖市及新疆生产建设兵团人力资源社会保障厅（局）、扶贫办（局）：

为深入贯彻党中央、国务院决策部署，在确保做好防疫工作的前提下，进一步做好就业扶贫工作，现就有关事项通知如下：

一、切实提高思想认识。各地要把思想和行动切实统一到习近平总书记重要讲话精神和党中央决策部署上来，在确保疫情防控安全的前提下，抓好涉及决胜全面建成小康社会、决战脱贫攻坚的重点任务，切实做好就业扶贫工作，不能有缓一缓、等一等的思想。努力减轻疫情对贫困劳动力就业增收的影响，助力全面建成小康社会和打赢脱贫攻坚战。

二、鼓励重点企业优先招录。根据本地区防疫物资、生产生活必需品生产以及上下游配套产业等重点企业用工需求，梳理适合贫困劳动力的就业岗位，实施远程精准推送。鼓励重点企业优先招用符合条件的贫困劳动力，给予一次性吸纳就业补贴，有条件地区可适当提高补贴标准。

三、安全有序组织外出返岗务工。主动了解贫困劳动力外出返岗务工需求和意愿，加强与输入地信息对接，对具备返岗条件的，优先开展“点对点”集中运送到岗，提供口罩等出行、上岗物资。鼓励公共就业服务机构、人力资源服务机构、劳务经纪人开展贫困劳动力有组织劳务输出，给予就业创业服务补助。

四、促进就地就近转移就业。在确保防疫安全前提下，协调推动疫情较轻地区的企业、就业扶贫车间等经营主体和乡村农田水利等项目尽早复工复产，推荐符合条件的贫困劳动力优先上岗。临时增设的保洁环卫、防疫消杀、卡点值守等岗位吸纳贫困劳动力的，参照当地乡村公益性岗位补贴标准给予岗位补贴。

五、重点做好深度贫困地区就业帮扶。将“三区三州”等深度贫困地区、易地扶贫搬迁安置点所在地区以及挂牌督战的52个贫困县作为工作重点，组织定向投放就业岗位，加大有组织劳务输出力度，适当扩大乡村公益性岗位规模，进一步给予资金倾斜支持，提高政策补贴标准。

六、推动务农就业增收。结合春季农业生产部署，引导无法外出贫困劳动力投入春耕备耕，从事农资运输、农产品产销等实现就业增收。加强与电子商务平台合作，推动爱心助农等活动惠及贫困劳动力。对吸纳贫困劳动力就业的各类农资企业、农业经营主体，参照重点企业给予一次性吸纳就业补贴。

七、加强关心关爱。组织第一书记、驻村工作队、帮扶责任人等人员力量，定期联络对口帮扶贫困劳动力，关心他们的健康、生活和务工情况。对贫困劳动力反映的卫生防疫、生活保障等方面问题，及时转交相关部门帮助解决。对受疫情影响失业的参保贫困劳动力，按照不高于当地失业保险金标准发放失业补助金。

八、优化线上服务线上培训。及时向贫困劳动力筛选推送一批有针对性的岗位信息，帮助贫困劳动力掌握居家线上求职应聘操作流程，有条件的地区可在确保防疫安全前提下，组织不具备线上求职条件的贫困劳动力到公共就业服务机构参加视频面试。鼓励支持贫困劳动力积极参加线上培训，在培训期间按规定纳入职业培训补贴范围，并给予一定的生活费补贴。

九、强化组织领导。各地要进一步压实工作责任，积极主动作为，向当地疫情防控领导机构建言献策，加强部门协同，创造有利于贫困劳动力的良好环境。动态掌握贫困劳动力就业情况，做到就业状态清、就业需求清、技能水平清、就业困难清，及时提供有针对性的就业帮扶，切实防止因疫失业返贫。

人力资源社会保障部 国务院扶贫办

2020年2月21日

（205）住建部等3部门：妥善应对新冠肺炎疫情实施住房公积金阶段性支持政策	建金〔2020〕23号

各省、自治区、直辖市人民政府，新疆生产建设兵团：

为贯彻落实习近平总书记关于新冠肺炎疫情防控和应对工作的重要指示精神，按照党中央、国务院关于出台阶段性、有针对性的政策措施，纾解企业困难的决策部署，经国务院常务会议审议通过，现就实施住房公积金阶段性支持政策有关事项通知如下：

一、受新冠肺炎疫情影响的企业，可按规定申请在2020年6月30日前缓缴住房公积金，缓缴期间缴存时间连续计算，不影响职工正常提取和申请住房公积金贷款。

二、受新冠肺炎疫情影响的职工，2020年6月30日前住房公积金贷款不能正常还款的，不作逾期处理，不作为逾期记录报送征信部门，已报送的予以调整。对支付房租压力较大的职工，可合理提高租房提取额度、灵活安排提取时间。

三、经认定的新冠肺炎疫情严重和较严重地区，企业在与职工充分协商的前提下，可在2020年6月30日前自愿缴存住房公积金。继续缴存的，自主确定缴存比例；停缴的，停缴期间缴存时间连续计算，不影响职工正常提取住房公积金和申请住房公积金贷款。

各地区要按照本通知要求，高度重视，周密部署，加强对工作落实情况的指导监督。各设区城市人民政府要结合本地实际，抓紧提出实施住房公积金阶段性支持政策的具体办法，做好落实工作。

中华人民共和国住房和城乡建设部

中华人民共和国财政部

中国人民银行

2020年2月21日

（206）卫健委 中医药局：印发新型冠状病毒肺炎恢复期中医康复指导建议（试行）	肺炎机制医疗发〔2020〕108号

各省、自治区、直辖市卫生健康委、中医药管理局，新疆生产建设兵团卫生健康委：

为充分发挥中医药独特优势，加快新冠肺炎恢复期康复，在总结新冠肺炎中医康复经验基础上，国务院应对新型冠状病毒肺炎疫情联防联控机制医疗救治组组织专家制定了《新型冠状病毒肺炎恢复期中医康复指导建议(试行)》。现印发给你们，供工作中参考。

国家卫生健康委员会办公厅　　国家中医药管理局办公室

2020年2月22日

新型冠状病毒肺炎恢复期

中医康复指导建议

(试行)

本建议适用于符合国家卫生健康委和国家中医药局联合印发的新型冠状病毒肺炎诊疗方案解除隔离和出院标准的恢复期人群。临床医师可参考本指导建议，根据患者个体情况给予相关治疗或康复指导，患者出院后亦可根据本建议采用适宜的自我干预方法。

一、推荐中药

(一)肺脾气虚证

临床表现：气短，倦怠乏力，纳差呕恶，痞满，大便无力，便溏不爽。舌淡胖，苔白腻。

推荐处方：法半夏9g、陈皮10g、党参15g、炙黄芪30g、炒白术10g、茯苓15g、藿香10g、砂仁6g(后下)、甘草6g。

服法：每日1剂，水煎400ml，分2次服用，早晚各1次。

推荐中成药：补气健脾类。

(二)气阴两虚证

临床表现：乏力，气短，口干，口渴，心悸，汗多，纳差，低热或不热，干咳少痰。舌干少津，脉细或虚无力。

推荐处方：南北沙参各10g、麦冬15g、西洋参6g、五味子6g、生石膏15g(先煎)、淡竹叶10g、桑叶10g、芦根15g、丹参15g、生甘草6g。

服法：每日1剂，水煎400ml，分2次服用，早晚各1次。

推荐中成药：益气养阴类。

二、中医适宜技术

(一)艾灸疗法

常用选穴：大椎、肺俞、上脘、中脘、膈俞、足三里、孔最等。

(二)经穴推拿

穴位按摩：太渊、膻中、中府、肺俞、肾俞、大肠俞、列缺、中脘、足三里等，咳嗽、咽痒、干咳者，可加少商、尺泽等。

经络推拿：手太阴肺经、手阳明大肠经、足阳明胃经、足太阴脾经、任脉、督脉等。

(三)耳穴压豆

常用耳穴：支气管、肺、内分泌、神门、枕、脾、胃、大肠、交感等。

(四)刮痧

手太阴肺经、手阳明大肠经、足太阳膀胱经等。

(五)拔罐

背腧穴为主，如肺俞、膏肓、脾俞、肾俞、大椎等。

(六)针刺疗法

常用选穴：太渊、曲池、肺俞、足三里、阴陵泉、关元等。

随症配穴：乏力、怕冷、舌淡者，可加膈俞、肾俞、大肠俞;食欲差、大便稀溏、舌淡者，可加中脘、天枢;咳嗽、咳痰、舌淡者，可加大椎或定喘、膏肓等。膏肓、肺俞、膈俞等穴局部肌肉薄，注意规范操作，避免引起气胸。

注：中药和中医适宜技术应在医师指导下使用。

三、膳食指导

总体建议：膳食平衡、食物多样、注重饮水、通利二便，并注重开胃、利肺、安神、通便。

根据食物属性和患者情况，进行分类指导：

(一)有怕冷、胃凉等症状的，推荐生姜、葱、芥菜、芫荽等;

(二)有咽干、口干、心烦等症状的，推荐绿茶、豆豉、杨桃等;

(三)有咳嗽、咯痰等症状的，推荐梨、百合、落花生、杏仁、白果、乌梅、小白菜、橘皮、紫苏等;

(四)有食欲不振、腹胀等症状的，推荐山楂、山药、白扁豆、茯苓、葛根、莱菔子、砂仁等;

(五)有便秘等症状的，推荐蜂蜜、香蕉、火麻仁等;

(六)有失眠等症状的，推荐酸枣仁、柏子仁等。

四、情志疗法

(一)五行音乐疗法

聆听五音与五脏、五志配合的乐曲，鼓动血脉、调畅情志。

(二)移情易性法

改变生活环境和方式，转移或分散患者某种思维的集中点，免于不良刺激，摆脱不良情绪。

五、传统功法

新冠肺炎轻型及普通型患者出院后，可采取多种功法;重型或危重型患者出院后，根据自身恢复情况选择适当的传统功法。

(一)八段锦

练习时间10～15 分钟左右，建议每天1~2次，按照个人体质状况，以能承受为宜。

(二)太极拳

推荐每日1次，每次30～50分钟为宜。

六、其他方法

(一)呼吸六字诀

“嘘(xu)、呵(he)、呼(hu)、呬(si)、吹(chui)、嘻(xi)”，依次每个字6秒，反复6遍，腹式呼吸方式，建议每天1~2组，根据个人具体情况调整当天运动方式及总量。

(二)呼吸疗愈法

主动进行缓慢深长的腹式呼吸训练，可采用鼻子吸气，嘴巴呼气，或鼻吸鼻呼，释放和疗愈身心。

(三)“三一二”经络锻炼法

“三”指合谷、内关、足三里三个穴的按摩，“一”是意守丹田、腹式呼吸，“二”是两下肢下蹲为主的体育锻炼。建议每天1~2次，按照个人体质状况，以能承受为宜。

（207）中央应对新型冠状病毒感染肺炎疫情工作领导小组：发布全面落实进一步保护关心爱护医务人员若干措施	国发明电〔2020〕5号

各省、自治区、直辖市人民政府，国务院各部委、各直属机构：

新冠肺炎疫情发生以来，全国广大医务人员义无反顾冲上疫情防控第一线，同时间赛跑，与病魔较量，顽强拼搏、日夜奋战，以对党和人民高度负责的精神，为保护人民生命健康作出重大贡献。医务人员是战胜疫情的中坚力量，当前全国疫情防控进入关键时期，医务人员工作任务重、感染风险高、工作压力大，各地各有关部门务必高度重视对他们的保护、关心、爱护，加强各方面支持保障，解除他们的后顾之忧，使他们始终保持强大战斗力、昂扬斗志、旺盛精力，持续健康、心无旁骛投入战胜疫情斗争。现提出以下措施：

一、提高疫情防治人员薪酬待遇。各地要按规定向参与疫情防治的医务人员发放临时性工作补助、核增一次性绩效工资总量、对卫生防疫人员落实卫生防疫津贴政策。在此基础上，疫情防控期间，将湖北省（含援鄂医疗队，下同）一线医务人员临时性工作补助相应标准提高1倍，并确保发放到位，中央财政对湖北省全额补助；及时核增医疗卫生机构一次性绩效工资总量，将湖北省一线医务人员薪酬水平提高2倍；扩大卫生防疫津贴发放范围，确保覆盖全体一线医务人员，所需经费按现行渠道解决。

二、做好工伤认定和待遇保障。各地要做好因履行工作职责感染新冠肺炎医务人员的工伤认定，开辟绿色通道、简化理赔程序，保障医务人员及时享受工伤保险待遇。

三、实施职称评聘倾斜措施。参加疫情防治的一线医务人员在职称评聘中优先申报、优先参评、优先聘任。医务人员参加疫情防治经历可视同为一年基层工作经历。参加疫情防治的一线医务人员晋升职称、晋升岗位等级不受本单位岗位结构比例限制。

四、落实一线医务人员生活保障。加强生活服务和后勤保障，为一线医务人员提供基础性疾病药物、卫生用品以及干净、营养、便捷的就餐服务。征用医院周边有条件的宾馆、招待所等固定场所，为一线医务人员提供舒适的生活休息环境和与家人隔离的必要条件。采取专车接送解决定点医院一线医务人员通勤问题。

五、加强医务人员个人防护。全力救治受感染的医务人员。加强对疫情防护物资的统筹调配，医用防护用品要重点向疫情防控一线投放使用，特别是要全力保障定点救治医院和发热门诊、集中隔离观察点等一线医务人员防护物资需求，最大限度减少院内感染。

六、确保轮换休整到位。合理安排一线医务人员轮休，做好轮休医务人员隔离保障，对长时间高负荷工作人员安排强制休息。提前做好一线医务人员后备力量储备，及时排查轮换因身体、心理等原因不适合继续在一线的医务人员。疫情结束后，及时组织一次免费健康体检和疗养休养，并适当增加休息和带薪休假时间。

七、及时做好心理调适疏导。加强对一线医务人员的心理干预和疏导，开展心理健康评估，强化心理援助措施，减轻医务人员心理压力。一线医务人员所在单位党组织要通过谈心谈话、关怀问候等方式，密切关注医务人员思想动态、情绪变化，做到心理问题早发现、早干预、早疏导。

八、切实落实有困难家庭的照顾帮扶。深入了解一线医务人员特别是援鄂医疗队队员家庭实际困难，建立台账，分类施策，切实解决一线医务人员后顾之忧。开通一线医务人员家属就医绿色通道，建立社区干部联系帮扶一线医务人员家庭制度，帮助解决老幼照护等实际困难。对于一线医务人员子女教育给予更多帮助关爱。

九、创造更加安全的执业环境。严格落实各项安全防范措施，加大力量投入，完善问责机制，维护医疗秩序，保障医务人员合法权益。对于伤害医务人员的，坚决依法严肃查处。

十、开展烈士褒扬和先进表彰。依法做好因疫情防控牺牲殉职人员的烈士评定和褒扬工作，全面做好抚恤优待。利用多种形式、渠道加大对医务人员职业精神的宣传力度。根据国家有关规定，开展医务人员及时奖励，对涌现出的先进典型进行及时表彰。对于获得表彰以及被认定为烈士的医务人员的子女，在入学升学方面按规定享受相关待遇。

地方各级党委和政府要按照党中央、国务院决策部署，坚决落实好以上政策措施，以高度负责的态度、务实到位的举措，切实保护关心爱护医务人员，为坚决打赢疫情防控阻击战提供有力保障。各级疫情防控工作领导小组要加强调度和督查，确保各项关心关爱政策落实到位。各级卫生健康部门要牵头逐一落实一线医务人员关心关爱政策措施，协调落实医务人员有关政策措施的资金保障，统筹用好党费、专项资金等各种渠道。各级人力资源社会保障部门要牵头进行政策统筹，分类研究提出具体建议，就相关政策形成具体化、易操作、可落地的措施和方案。各级财政部门要做好资金保障。各地医疗卫生机构要强化主体责任，结合实际做好相关保障工作，准确提供一线医务人员信息，确保精准、无遗漏。

中央应对新型冠状病毒感染肺炎

疫情工作领导小组

2020年2月22日

（208）国务院应对新型冠状病毒感染肺炎疫情联防联控机制：印发企事业单位复工复产疫情防控措施指南	国发明电〔2020〕4号

各省、自治区、直辖市人民政府，国务院各部委、各直属机构：

《企事业单位复工复产疫情防控措施指南》已经中央应对新型冠状病毒感染肺炎疫情工作领导小组同意，现印发给你们，请结合实际抓好贯彻落实。

国务院应对新型冠状病毒感染肺炎

疫情联防联控机制

2020年2月21日

企事业单位复工复产疫情防控措施指南

为指导落实好新冠肺炎疫情防控各项工作要求，推动企事业单位稳步有序复工复产，特制定本指南。

一、加强员工健康监测

（一）做好员工健康管理。各单位要切实掌握员工流动情况，按照当地要求分区分类进行健康管理，对来自疫情严重地区的人员实行居家或集中隔离医学观察。对处在隔离期间和入住集体宿舍的员工，应每日进行2次体温检测。及时掌握缺勤人员健康状况。

（二）实行健康状况报告。各单位要设立可疑症状报告电话，员工出现发热、呼吸道症状时，要及时向本单位如实报告。要每天汇总员工健康状况，向当地疾控部门报告，发现异常情况及时报告并采取相应的防控措施。

二、做好工作场所防控

（三）加强进出人员登记管理。各单位要指派专人对进出单位和宿舍的所有通道进行严格管理。使用指纹考勤机的单位应暂时停用，改用其他方式对进出人员进行登记。员工每次进入单位或厂区时，应在入口处检测体温，体温正常方可进入。要尽量减少非本单位人员进入，确因工作需要的，应检测体温，并询问来源地、工作单位、接触疫情发生地区人员等情况，符合要求方可进入。

（四）保持工作场所通风换气。各单位在条件允许情况下首选自然通风，如室温因通风有所降低，应提醒工作人员适当加衣保暖。如使用空调，应当确保供风安全充足，所有排风直接排到室外，不使用空调时应当关闭回风通道。

（五）保障洗手等设施正常运行。工作场所应设置洗手设备，洗手、喷淋设施应保持正常运行。如无洗手设备，应配备免洗消毒用品。

（六）做好工作和生活场所清洁消毒。工作场所、食堂、电梯、卫生间、洗手池、通勤工具等公共区域及相关物品，应由专人负责定期消毒。电梯按钮、门把手等频繁接触部位应适当增加消毒次数。

（七）减少员工聚集和集体活动。引导员工在使用通道、电梯、楼梯、吸烟区时有序排队，保持适当间距，吸烟时不与他人交谈。减少召开会议，需要开的会议要缩短时间、控制规模，保持会议室空气流通，提倡召开视频或电话会议。员工集体宿舍原则上每间不超过6人，人均不少于2.5平方米。根据实际情况可采取错时上下班、弹性工作制或居家办公方式。

（八）加强员工集体用餐管理。适当延长食堂供餐时间，实行错峰就餐，有条件时使用餐盒、分散用餐。要加强循环使用餐具清洁消毒，不具备消毒条件的要使用一次性餐具。员工用餐时应避免面对面就坐，不与他人交谈。

（九）做好医务服务。设立医务室的单位要调配必要的药物和防护物资，配合疾控部门规范开展隔离观察与追踪管理。未设立医务室的单位应当就近与医疗机构建立联系，确保员工及时得到救治或医疗服务。关心关爱员工心理健康，及时疏解心理压力。

（十）规范垃圾收集处理。在公共区域设置口罩专用回收箱，加强垃圾箱清洁，定期进行消毒处理。加强垃圾分类管理，及时收集并清运。

三、指导员工个人防护

（十一）强化防控宣传教育。采用多种形式加强复工复产后疫情防治知识科普宣传，使员工充分了解防治知识、掌握防护要点、增强防护意识、支持配合防控工作。

（十二）落实个人防护要求。员工要减少不必要外出，避免去人群聚集尤其是空气流动性差的场所。在人员密集场所应按照《不同人群预防新型冠状病毒感染口罩选择和使用技术指引》要求，正确佩戴口罩等防护用品。养成勤洗手习惯，打喷嚏或咳嗽时要用纸巾、手绢、衣袖等遮挡，倡导合理膳食、适量运动、规律作息等健康生活方式。

四、做好异常情况处置

（十三）明确单位防控责任。各单位主要负责人是疫情防控第一责任人，要建立单位内部疫情防控组织体系，明确疫情防控应急措施和处置流程，把防控责任落实到部门和个人。

（十四）设立隔离观察区域。当员工出现可疑症状时，及时到该区域进行暂时隔离，并报告当地疾控部门，按照相关规范要求安排员工就近就医。

（十五）封闭相关区域并进行消毒。发现可疑症状员工后，立即隔离其工作岗位和宿舍，并根据医学观察情况进一步封闭其所在的办公室、车间等办公单元以及员工宿舍楼等生活场所，严禁无关人员进入，同时在专业人员指导下对其活动场所及使用物品进行消毒。配合有关方面做好密切接触者防控措施。

（十六）做好发现病例后的应对处置。已发现病例的单位，要实施内防扩散、外防输出的防控策略，加强病例流行病学调查、密切接触者追踪管理、疫点消毒等工作。疫情播散的单位，要实施内防蔓延、外防输出的防控策略，根据疫情严重程度，暂时关闭工作场所，待疫情得到控制后再恢复生产。

（209）商务部：在做好防疫工作的前提下推动商务领域企业有序复工复产

各省、自治区、直辖市、计划单列市及新疆生产建设兵团商务主管部门：

为深入贯彻习近平总书记关于新冠肺炎疫情防控工作的重要讲话和指示批示精神，落实统筹推进新冠肺炎疫情防控和经济社会发展工作部署会议的要求，按照中央应对疫情工作领导小组的部署和国务院联防联控机制工作要求，在切实做好疫情防控的前提下，推动商务领域企业有序复工复产，做好稳外贸稳外资促消费工作，努力实现全年商务改革发展目标任务。现就有关事项通知如下：

一、压实责任，严防聚集性感染

各地要按照“突出重点、统筹兼顾，分类指导、分区施策”的要求，依法做好分区分级差异化防控，严格落实国务院联防联控机制印发的《企事业单位复工复产疫情防控措施指南》，建立最严格的岗位责任制，密切跟踪企业复工复产过程中疫情防控动态，及时上报有关信息，发现问题果断处理，严防复工复产过程中发生聚集性感染事件。要积极协调有关部门，采取措施加强一线员工防护用品配备，主动向企业通报疫情防控最新政策和工作进展，及时发出防控预警，提出防控建议。要压实企业疫情防控主体责任，从严从细落实好各项疫情防控措施。

二、做好各领域疫情防控工作

各地要加强分类指导，结合内贸、外贸、外资企业实际情况，明确不同领域复工复产过程中疫情防控的重点，完善分区分级精准防控策略。要指导企业按照已经印发的相关领域、行业疫情防控指南，制定本企业疫情防控方案并组织实施。

（一）零售、餐饮、住宿等生活服务领域。要指导企业按照零售、餐饮、住宿、洗染、美容美发、摄影、家电服务、家政等行业防疫指南要求，切实做好经营场所、商品管理等方面的卫生防疫措施，营造健康安全的消费环境。同时，指导企业创新经营服务方式，鼓励运用线上模式开展业务，组织动员餐饮企业提供“移动食堂”或餐饮外卖服务。

（二）外贸、外资领域。要指导外贸、外资企业科学安排生产经营，制定疫情应对方案和应急预案，严格做好厂区（办公区）、宿舍、食堂等人群聚集场所的防控工作。密切联系重点外贸、外资企业，做好疫情防控实时跟踪和上报等工作。

（三）电子商务领域。要鼓励电商企业充分利用信息化技术，实行远程居家办公。督促电商企业落实员工健康台账，掌握一线员工返岗前14天内活动轨迹，严禁“带病上岗”。严格落实入库商品、工作场所和设备、交通工具等定时消毒制度，记录每位配送人员配送清单、配送轨迹，并妥善保存备查。

三、推动企业有序复工复产

各地要在当地党委和政府的统一领导下，坚持科学防治、精准施策的原则，推动内贸、外贸、外资企业有序复工复产。

（一）生活服务领域。对于承担居民生活必需品市场供应保障的批发市场、超市、便利店、社区菜店等流通企业，在做好一线员工防疫物资配备的前提下，要支持企业尽快复工复产，全力满足民生消费需求。指导与休闲娱乐等升级消费需求关系密切的其他流通企业，根据当地疫情形势变化及防控要求，进行前瞻研判，适时复工复产，逐步恢复消费信心和市场活力。

（二）外贸领域。突出重点，克服困难，用足用好出口退税、出口信用保险等合规的外贸政策工具，优先保障供应链集中的重点地区以及外贸领域龙头企业、关键环节复工复产，保障外贸产业链、供应链畅通运转。要建立重点外贸企业复工清单，综合考虑进出口规模、产业特征、订单紧急程度、本地用工情况、地区防疫情况等，建立省市县三级重点外贸企业复工清单，及时更新清单信息，摸清复工复产详实情况。通过开设政策兑现绿色通道，加快有关部门和地方政府出台的各项帮扶政策落地。

（三）外资领域。继续优化营商环境，增强外商长期投资经营的信心。优先保障在全球供应链中有重要影响的外资龙头企业和配套企业复产复工，保障全球供应链稳定性。协调推动电子、汽车等领域外资企业和上下游企业同步复工。抓好重大外资项目落地，加大服务保障力度，推动加快项目建设和投资进度。发挥好国家级经开区、自贸试验区引资平台作用，符合条件的要加快推动外资企业复工复产。

（四）电子商务领域。要采取有效措施为电商复工复产提供必要的便利条件，着力解决好电商企业人员返岗的食宿出行及企业在仓储、分拣、配送中遇到的实际问题。鼓励电商平台企业充分发挥信息汇集、资源调配、异地协同、远程服务等方面优势，为平台商户复工提供支持，适当降低平台商户的佣金费率，提供小额信用贷款。鼓励电商企业推出社区拼购、多天套餐、无人售货机、无接触配送等生活必需品销售新模式，减少订购配送频次，降低直接接触风险，安全便利地保障消费需求。

四、做好跟踪指导工作

各地要密切跟踪企业疫情防控和复工复产情况，在本地党委政府统一领导下，积极会同相关部门协调解决企业在复工复产中的困难和问题。打通人流、物流堵点，放开货运物流限制，推动产业链各环节协同复工复产。要大力简化工作程序，对已具备条件的企业，尽快组织复工复产；对因疫情5防控需要被关闭的企业，要尽快安排复核，符合防控要求的要组织有序复工复产。要切实落实已经出台的稳外贸稳外资促消费政策措施，缓解企业经营困难，努力降低疫情对商务工作的影响。

各地要坚决贯彻落实党中央、国务院决策部署，敢于担当、主动作为，统筹做好疫情防控和复工复产工作，努力完成商务工作全年目标任务。有关工作进展情况及时上报。

商务部

2020年2月23日

（210）商务部：统筹做好生活必需品供应保障有关工作

各省、自治区、直辖市、计划单列市及新疆生产建设兵团、副省级省会城市商务主管部门：

为深入贯彻落实习近平总书记重要指示批示和中央政治局常委会会议精神，按照中央应对疫情工作领导小组统一部署和国务院联防联控机制印发的《关于科学防治精准施策分区分级做好新冠肺炎疫情防控工作的指导意见》要求，在确保疫情防控安全的前提下，有序有力组织商贸企业复工复产，进一步做好生活必需品供应保障工作，现将有关工作通知如下：

一、提高站位，不断加强组织领导。要深刻领会党中央、国务院对疫情形势的新判断和防控工作的新部署，进一步提高政治站位，根据本地党委、政府对分区分级精准防控作出的动态调整，顺应形势，厘清思路，及时纠正防控中偏颇和极端做法。一把手亲自抓防疫的同时，要尽快组织精干力量，制定完善工作方案，有序推进商贸企业复工营业。要及时发现防疫前线与复工一线存在的问题，果断协调处理，进一步理顺疫情防控与复工复产的关系，切实做到防疫和复工两手抓、两手硬，尽可能减少防控对群众生产生活的影响。

二、完善机制，强化重点地区保障。武汉稳，则湖北稳；湖北稳，则全国稳。相关地区商务主管部门要切实按照《商务部办公厅关于新冠肺炎疫情防控期间进一步完善九省联保联供协作机制通知》《关于疫情防控期间做好九省联保联供协作机制工作强化有关事项的通知》等要求，全力配合进一步做实做强九省联保联供协作机制，强化湖北武汉生活必需品保障供应的成效。各地商务主管部门要重点聚焦蔬菜、肉类、蛋类、奶类、大米、面粉、食用油和方便食品等8类生活必需品，做到总量充裕。加强市场监测预警，掌握库存和货源动态，积极开拓进口渠道；抓紧做好保供骨干企业的对接协调，切实加强供需双方按需调运，指导企业加大补货频次和力度，防止大中城市出现货源不足、集中采购、商品空架等现象。

三、精准施策，有序推进复工复产。要按照“科学评估、突出重点、分级管控、有序扩围”的原则，结合本地疫情形势和经济社会发展实际需要，按照地方党委、政府要求做好分区分级差异化防控，有序安排商贸企业复工营业。湖北省和武汉市在实施最严防控措施基础上，指导有条件的商贸企业，创新开展“社区代购+集中配送”“线上下单+无接触配送”等便民保供新模式。北京、上海、广东、河南、浙江、湖南、安徽、江西等疫情防控重点地区，要准确研判疫情风险，科学划区、分区防控。高风险区域在严密管控的同时，优先组织生活必需品经营企业复工；中低风险区域要以外防输入为主，兼顾内防扩散，抓紧有序动员商贸流通企业复工营业，不断扩大业态经营范围。其他非疫情防控重点地区，要在确保防疫安全的基础上，千方百计全面组织动员商贸企业复工开业，严禁一关了之、一停了之。

四、问题导向，优化服务保障举措。要在本地生活物资保障组统一协调下，积极会同相关部门，抓紧破解复工复产难点问题。大力简化复工审批流程，对因防控需要被勒令关闭的企业，要尽快安排复核，符合防控要求的要有序批准复工；对已具备复工条件的企业，要加大动员力度，尽快组织开门营业；要着力保障复工一线防疫用品急需，加强防疫安全指导；要抓紧落实或协调出台一批政策措施，满足企业用工需求，缓解企业运营困难。坚决打通交通运输堵点问题。会同公安、交通运输、卫健等部门，严格落实“五个严禁”“一断三不断”“五不得一支持”等要求，及时制止擅自层层设卡、封村封路、阻断交通等行为，不断优化交通防疫检查设置，打通运输主动脉的梗阻，确保物流微循环的畅通，加快生活必需品调运，切实提高补货保供的效率。

五、统筹兼顾，推动商务事业发展。要在全力做好生活必需品保供的同时，认真落实中央经济工作会议部署，统筹推进全国商务工作会议布置的各项工作。要对疫情影响、市场走势及时分析，进行前瞻研判，提出可行预案。要因地制宜，抓紧出台务实管用的“一促两稳”政策，将疫情对商务领域的影响降到最低，坚决完成商务全年目标任务。

各地商务主管部门要坚决贯彻落实党中央、国务院决策部署，统一思想，提高站位，认清形势，主动作为，统筹做好生活必需品保供各项工作，更好满足人民群众基本生活需要。有关情况请按要求及时上报商务部。

联系方式：市场运行司 010-85093851/3861(传真)

tiaok_scyx@mofcom.gov.cn

商务部

2020年2月21日

（211）卫健委：做好新冠肺炎疫情防控一线医务人员老年亲属关爱服务工作	肺炎机制综发〔2020〕73号

各省、自治区、直辖市及新疆生产建设兵团应对新型冠状病毒肺炎疫情联防联控机制（领导小组、指挥部）：

为认真贯彻落实习近平总书记关于“务必高度重视对医务人员的保护关心爱护”重要指示精神，按照国务院应对新型冠状病毒肺炎疫情联防联控机制《关于全力做好一线医务人员及其家属保障工作的通知》（肺炎机制发〔2020〕23号）和国务院办公厅转发国家卫生健康委、人力资源社会保障部、财政部《关于改善一线医务人员工作条件切实关心医务人员身心健康若干措施》的通知（国办发〔2020〕4号）要求，加强对奋战在新冠肺炎疫情防控一线医务人员的关心关爱，着力做好因子女参与疫情防控工作而家中无人照料老年亲属的服务工作，现提出以下要求：

一、了解实际需求

各地要高度重视防控一线医务人员老年亲属的关爱服务工作，指定专人负责，建立工作台账，全面掌握参与新冠肺炎疫情防控一线医务人员家中老年亲属生活及照料情况，及时了解他们的需求和困难，积极动员各方力量帮助解决困难和问题。尽量不安排双职工的医务人员同时到防控一线工作，特别是家中有老年人需要照顾的医务人员，要尽可能使其兼顾家庭。

二、提供健康服务

各地要将新冠肺炎疫情防控一线医务人员家中无人照料的老年亲属作为重点关爱服务对象，通过电话、微信等方式，定期了解其健康状况，特别是对患有高血压、糖尿病等慢性病或行动不便、高龄的老年人，要帮助其做好健康管理。对于有用药、就医需求的老年人，在做好防护措施的基础上，要安排专门工作人员或志愿者送药上门，陪同就医。需要住院服务的，应当予以全力保障，并安排陪护人员。

三、提供托养服务

各地要在充分了解需求并尊重个人意愿的基础上，积极组织有条件的养老机构、医养结合机构（指同时具备医疗卫生资质和养老服务能力的医疗卫生机构或养老机构）为一线医务人员家中无人照料的老年亲属提供临时托养服务，安排好老年人的住宿、饮食及护理，让前方医务人员放心、安心，让后方老年亲属暖心、舒心。临时托养机构应当具备防护隔离条件，并严格执行各项防控措施，切实保障入住老年人生命安全和身体健康。

四、提供生活服务

各地要积极动员社会力量、志愿者或安排专门工作人员，为一线医务人员家中无人照料的老年亲属提供“一对一”的生活照料服务。根据老年人需求，为其提供必要的防护用品、代买配送生活必需品以及助餐、助洁等服务。

五、加强组织保障

各地要按照党中央、国务院决策部署，切实加强组织领导，加大保障力度，制定具体关爱服务办法及相关服务费用减免政策，全力做好一线医务人员老年亲属的关爱服务工作，为一线医务人员解除后顾之忧，为坚决打赢疫情防控阻击战提供坚强保障。

国务院应对新型冠状病毒肺炎疫情联防联控机制综合组

2020年2月21日

（212）医保局等3部门：阶段性减征职工基本医疗保险费	医保发〔2020〕6号

各省、自治区、直辖市人民政府，新疆生产建设兵团：

为贯彻落实习近平总书记关于新冠肺炎疫情防控工作的重要指示精神，切实减轻企业负担，支持企业复工复产，根据社会保险法有关规定，经国务院同意，现就阶段性减征职工基本医疗保险（以下简称职工医保）单位缴费有关工作提出如下指导意见：

一、自2020年2月起，各省、自治区、直辖市及新疆生产建设兵团（以下统称省）可指导统筹地区根据基金运行情况和实际工作需要，在确保基金收支中长期平衡的前提下，对职工医保单位缴费部分实行减半征收，减征期限不超过5个月。

二、原则上，统筹基金累计结存可支付月数大于6个月的统筹地区，可实施减征；可支付月数小于6个月但确有必要减征的统筹地区，由各省指导统筹考虑安排。缓缴政策可继续执行，缓缴期限原则上不超过6个月，缓缴期间免收滞纳金。

三、各省要指导统筹地区持续完善经办管理服务，确保待遇支付，实施减征和缓缴不能影响参保人享受当期待遇。参保单位应依法履行代扣代缴个人缴费的义务，医保经办机构要做好个人权益记录，确保个人权益不受影响。优化办事流程，不增加参保单位事务性负担。

四、各省要指导统筹地区切实加强基金管理，做好统计监测，跟踪分析基金运行情况，采取切实管用的措施，管控制度运行风险，确保基金收支中长期平衡。减征产生的统筹基金收支缺口由统筹地区自行解决。各省可根据减征情况，合理调整2020年基金预算。

五、已经实施阶段性降低单位费率等援企政策的省可继续执行，也可按照本指导意见精神指导统筹地区调整政策。已实施阶段性降低职工医保单位费率的统筹地区，不得同时执行减半征收措施。

各省要提高思想认识，加强组织领导，分类指导统筹地区做好相关工作。决定实施减征政策的省，印发的具体实施方案于3月5日前报医保局、财政部、税务总局备案。各级医疗保障、财政、税务等部门要加强协同，切实履职，全力做好疫情防控期间的医疗保障各项工作，确保政策落实到位，重要情况及时报告。

国家医保局 财政部 税务总局

2020年2月21日

（213）发改委：阶段性降低非居民用气成本支持企业复工复产	发改价格〔2020〕257号

各省、自治区、直辖市及计划单列市、新疆生产建设兵团发展改革委，中国石油天然气集团有限公司、中国石油化工集团有限公司、中国海洋石油集团有限公司：

为贯彻落实习近平总书记关于坚决打赢疫情防控阻击战的重要指示精神，按照党中央、国务院决策部署，统筹疫情防控与经济社会发展，降低企业用气成本，支持企业复工复产、共渡难关，现就阶段性降低非居民用气成本有关事项通知如下。

一、非居民用气门站价格提前执行淡季价格政策。天然气生产经营企业要提前执行淡季价格，自本通知印发之日起，对执行政府指导价的非居民用气，要以基准门站价格为基础适当下浮，尽可能降低价格水平；对价格已放开的非居民用气，鼓励天然气生产经营企业根据市场形势与下游用气企业充分协商沟通，降低价格水平。

二、对化肥等受新冠肺炎疫情影响大的行业给予更大价格优惠。鼓励天然气生产经营企业充分考虑疫情对不同行业的影响，对化肥等涉农生产且受疫情影响大的行业给予更加优惠的供气价格。

三、及时降低天然气终端销售价格。各地价格主管部门要高度重视，抓紧工作，根据上游企业降价情况，及时降低非居民用气终端销售价格，切实将门站环节降价空间全部传导至终端用户。加强跟踪调查，及时发现并解决政策落实中出现的具体问题，确保平稳实施。鼓励各地通过加强省内管道运输和配气价格监管等方式，进一步降低天然气终端销售价格，释放更多降价红利。

四、切实维护天然气市场稳定。有关部门和天然气生产经营企业要加强生产组织和供需衔接，保障下游企业用气需要，保持市场平稳运行。天然气生产经营企业要充分考虑疫情对下游企业生产运营的影响，按照实际供气量进行结算。

五、加强宣传解释。各地要采取多种方式积极宣传，准确解读阶段性降低企业用气成本政策，及时回应社会关切，营造良好舆论氛围，增强企业信心。

六、实施时间。上述措施有效期至2020年6月30日。

国家发展改革委

2020年2月22日

（214）发改委：阶段性降低企业用电成本支持企业复工复产	发改价格〔2020〕258号

各省、自治区、直辖市及计划单列市、新疆生产建设兵团发展改革委，国家电网有限公司、南方电网有限责任公司、内蒙古电力（集团）有限责任公司：

为贯彻落实党中央、国务院决策部署，统筹疫情防控与经济社会发展，支持企业复工复产、共渡难关，现就阶段性降低企业用电成本有关事项通知如下。

一、降价范围

此次降电价范围为除高耗能行业用户外的，现执行一般工商业及其它电价、大工业电价的电力用户。

二、降价措施

自2020年2月1日起至6月30日止，电网企业在计收上述电力用户（含已参与市场交易用户）电费时，统一按原到户电价水平的95%结算。

三、进一步明确支持性两部制电价政策执行时间

2020年2月7日，我委出台的《关于疫情防控期间采取支持性两部制电价政策 降低企业用电成本的通知》（发改办价格〔2020〕110号），进一步明确执行至2020年6月30日。

四、工作要求

（一）各地价格主管部门要高度重视，结合当地情况，指导电网企业切实抓好政策落实，加强跟踪调度，及时发现解决政策落实中出现的具体问题，确保政策平稳实施。同时，要采取多种形式积极宣传、准确解读阶段性降低企业用电成本政策，增强企业信心。

（二）各地价格主管部门要积极配合当地市场监管部门，切实加强商业综合体、产业园区、写字楼等转供电环节收费行为监管，确保降电价红利及时足额传导到终端用户，增加企业获得感。

（三）电网企业要积极主动向用户做好政策宣传告知，明确降价范围对应的用户，妥善做好政策执行时间追溯，尽快将政策执行到位。

国家发展改革委

2020年2月22日

（215）卫健委：修订新型冠状病毒肺炎英文命名	国卫医函〔2020〕70号

各省、自治区、直辖市人民政府，新疆生产建设兵团，国务院应对新型冠状病毒肺炎疫情联防联控机制成员：

现决定将“新型冠状病毒肺炎”英文名称修订为“COVID-19”，与世界卫生组织命名保持一致，中文名称保持不变。

国家卫生健康委

2020年2月21日

（216）卫健委：印发新型冠状病毒肺炎防控方案（第五版）	国卫办疾控函〔2020〕156号

各省、自治区、直辖市及新疆生产建设兵团卫生健康委，中国疾病预防控制中心：

为进一步指导各地做好新型冠状病毒肺炎防控工作，我委组织更新了新型冠状病毒肺炎防控方案。现印发给你们，请参照执行。各地在执行过程中如有相关建议，请及时反馈我委。

附件：新型冠状病毒肺炎防控方案（第五版）

国家卫生健康委办公厅

2020年2月21日

（217）财政部 生态环境部：加强污染防治资金管理 支持打赢疫情防控阻击战	财资环〔2020〕3号

各省、自治区、直辖市财政厅（局）、生态环境厅（局），新疆生产建设兵团财政局、生态环境局：

为深入贯彻落实习近平总书记重要指示精神和党中央、国务院决策部署，支持打赢新冠肺炎疫情防控阻击战，现就积极应对疫情影响，做好污染防治资金管理工作通知如下：

一、环保支出要向受疫情影响较重的市县倾斜。各省份要继续保障财政污染防治资金投入，结合本地区疫情防控的实际，分配污染防治资金时要向受疫情影响较重的市县倾斜，给予积极支持，切实保障好这些地区污染防治资金需要，防止疫情次生灾害对生态环境和人民群众健康造成不良影响，支持打赢疫情防控阻击战。

二、资金使用要聚焦重点支出方向。根据疫情防控需要，水污染防治资金要支持开展应急监测和处置、加强饮用水水源地环境保护、垃圾填埋场地下水环境监管等，保障群众用水安全；土壤污染防治资金可支持废物应急处置，确保安全处置效果；农村环境整治资金要结合农村公共卫生体系建设，加强村庄生活污水和垃圾处理，切实提高卫生水平和环境质量。

三、优化资金安排程序。对已经安排下达的污染防治资金，可根据疫情实际需求，在现有规定范围内做适当调整，切实保障疫情防控经费需求。对疫情防控重点环保项目，应本着急事急办、特事特办的原则，可适当简化项目立项、入库审批程序，待疫情结束后统一备案。

四、抓紧推动项目实施。具备开工条件的生态环保项目，要在保证安全基础上，及时开工建设；暂时不能开工的，要提前谋划、采取措施，积极开展项目设计、评审等项目实施前期准备工作,一旦具备条件立即推动项目实施；确定不能按时执行的，可予以合理顺延。

五、加强资金监督管理。各级财政部门、生态环境部门切实加强相关污染防治资金管理，避免出现不按规定调整资金使用问题。各省份要加强对市县的指导，突出绩效导向，实行全过程绩效管理。

各地要充分认识当前疫情防控形势下做好污染防治工作的重要性，积极应对、认真履职、主动作为，加强部门间协同配合，切实把各项工作抓实抓细，发挥财政支持打好污染防治攻坚战重要作用。

财政部 生态环境部

2020年2月20日

（218）人社部等3部门：阶段性减免企业社会保险费	人社部发﹝2020﹞11号

各省、自治区、直辖市人民政府，新疆生产建设兵团：

为贯彻落实习近平总书记关于新冠肺炎疫情防控工作的重要指示精神，纾解企业困难，推动企业有序复工复产，支持稳定和扩大就业，根据社会保险法有关规定，经国务院同意，现就阶段性减免企业基本养老保险、失业保险、工伤保险（以下简称三项社会保险）单位缴费部分有关问题通知如下：

一、自2020年2月起，各省、自治区、直辖市（除湖北省外）及新疆生产建设兵团（以下统称省）可根据受疫情影响情况和基金承受能力，免征中小微企业三项社会保险单位缴费部分，免征期限不超过5个月；对大型企业等其他参保单位（不含机关事业单位）三项社会保险单位缴费部分可减半征收，减征期限不超过3个月。

二、自2020年2月起，湖北省可免征各类参保单位（不含机关事业单位）三项社会保险单位缴费部分，免征期限不超过5个月。

三、受疫情影响生产经营出现严重困难的企业，可申请缓缴社会保险费，缓缴期限原则上不超过6个月，缓缴期间免收滞纳金。

四、各省根据工业和信息化部、统计局、发展改革委、财政部《关于印发中小企业划型标准规定的通知》（工信部联企业〔2011〕300号）等有关规定，结合本省实际确定减免企业对象，并加强部门间信息共享，不增加企业事务性负担。

五、要确保参保人员社会保险权益不受影响，企业要依法履行好代扣代缴职工个人缴费的义务，社保经办机构要做好个人权益记录工作。

六、各省级政府要切实承担主体责任，确保各项社会保险待遇按时足额支付。加快推进养老保险省级统筹，确保年底前实现基金省级统收统支。2020年企业职工基本养老保险基金中央调剂比例提高到4%，加大对困难地区的支持力度。

七、各省要结合当地实际，按照本通知规定的减免范围和减免时限执行，规范和加强基金管理，不得自行出台其他减收增支政策。各省可根据减免情况，合理调整2020年基金收入预算。

各省要提高认识，切实加强组织领导，统筹做好疫情防控和经济社会发展工作，抓紧制定具体实施办法，尽快兑现减免政策。各省印发的具体实施办法于3月5日前报人力资源社会保障部、财政部、税务总局备案。各级人力资源社会保障、财政、税务部门要会同相关部门，切实履行职责，加强沟通配合，全力做好疫情防控期间企业社会保险工作，确保企业社会保险费减免等各项政策措施落实到位。

人力资源社会保障部 财政部 税务总局

2020年2月20日

（219）发改委：提供疫情防控重点保障物资具体范围	发改办财金[2020]145号

财政部、国家税务总局办公厅：

根据《关于支持新型冠状病毒感染的肺炎疫情防控有关税收政策的公告》（财政部、税务总局公告2020年第8号）相关要求，按照国务院联防联控机制工作安排，我委确定了疫情防控重点保障物资中生活物资和部分医疗应急物资的具体范围清单（见附件）。该清单将视疫情防控需要予以动态调整。

特此函告。

附件：疫情防控重点保障物资清单

疫情防控重点保障物资清单

一、医疗应急物资

1、应对疫情使用的医用防护服、隔离服、隔离面罩、医用

及具有防护作用的民用口罩、医用护目镜、新型冠状病毒检测试

剂盒、负压救护车、消毒机、消杀用品、红外测温仪、智能监测

检测系统、相关医疗器械、酒精和药品等重要医用物资。

2、生产上述物资所需的重要原辅材料、重要设备和相关配

套设备。

3、为应对疫情提供相关信息的通信设备。

二、生活物资

1、帐篷、棉被、棉大衣、折叠床等救灾物资。

2、疫情防控期间市场需要重点保供的粮食、食用油、食盐、

糖，以及蔬菜、肉蛋奶、水产品等“菜篮子”产品，方便和速冻食

品等重要生活必需品。

3、蔬菜种苗、仔畜雏禽及种畜禽、水产种苗、饲料、化肥、

种子、农药等农用物资。

国家发展改革委办公厅

2020年2月18日

（220）发改委：加强投资项目远程审批服务 保障新型冠状病毒感染肺炎疫情防控期间项目办理工作	发改电〔2020〕66号

各省、自治区、直辖市及计划单列市、新疆生产建设兵团发展改革委：

为深入贯彻党中央、国务院关于新型冠状病毒感染肺炎的疫情防控工作部署，在加强疫情防控的同时，围绕做好“六稳”工作，积极推进投资项目储备，保障投资项目申报、受理、审批和前期工作稳妥有序开展，保持新开工项目后劲，现就依托全国投资项目在线审批监管平台加强项目远程审批服务有关事项通知如下：

一、严格执行在线告知性备案制度。对实行备案制的投资项目，按照《企业投资项目核准和备案管理条例》《企业投资项目核准和备案管理办法》，一律通过全国投资项目在线审批监管平台（以下简称在线平台）实行网上备案。项目单位需要备案证明文件的，备案机关要指导项目单位通过在线平台打印；项目单位不具备在其办公场所自行远程打印条件、不能到备案机关或实体大厅领取的，备案机关要及时通过邮寄方式送达项目单位。

二、多措并举，加快推行网上审批和核准。相关地方根据疫情防控需要暂停实体大厅服务的，对实行审批制、核准制的投资项目及时启动“不见面”在线办理。相关省级发展改革委要完善、畅通在线平台互联网端项目申报、进度查询功能，项目审批、核准机关要指导项目单位上传电子材料、实行远程审批；具备条件的地方，应尽快完善网上出件功能。对投资项目办理过程中确需纸质材料的，以及项目单位在其办公场所无法实现批准文件网上出件的，应通过邮件方式寄送。其他地方要对标“不见面”审批模式，尽快完善在线平台“一网通办”功能，提升远程审批服务水平。

三、强化咨询服务和技术保障。各地审批、核准、备案机关要通过在线平台公布投资项目办理咨询电话，开通在线咨询服务专区，加强咨询服务值守，为项目单位远程提供政策解答、流程指引、释疑解惑等优质便捷服务，指导规范项目单位办理网上审批。在线平台运行维护单位要加强技术保障，及时响应、解决在线审批的技术问题。

四、加强跟踪指导。各省级发展改革委要及时掌握暂停实体大厅服务的市、县，逐一指导督促其审批、核准、备案机关尽快应用在线平台开展网上受理、远程审批（核准、备案）工作。在线平台采取省级统一建设模式的，各省级发展改革委要安排技术人员加强值守，对相关地方提出的技术需求和疑难问题，要及时予以协调解决。

各地要从贯彻中央精神、努力实现今年经济社会发展目标的高度，充分认识应用在线平台加强投资项目远程审批服务工作的紧迫性和重要性，采取有力措施提高在线审批服务水平，确保在疫情防控期间保障项目办理畅通，有效稳定社会投资意愿，积极稳妥做好项目储备工作，为稳定投资运行提供坚实基础，坚决防止出现项目申报无门、审批服务出现断档问题。各省级发展改革委于2月10日前，将本地加强在线平台应用、推行远程审批服务情况，通过纵向网报送我委投资司制度法规处。

国家发展改革委办公厅

2020年2月4日

（221）人社部：切实做好新冠肺炎疫情防控期间人力资源服务有关工作	人社厅明电〔2020〕11号

各省、自治区、直辖市及新疆生产建设兵团人力资源社会保障厅（局）：

为深入贯彻习近平总书记关于做好新冠肺炎疫情防控工作的重要指示精神，坚决落实党中央、国务院关于打赢疫情防控阻击战的决策部署，结合当前复工复产工作形势需要，指导鼓励各地各类人力资源服务机构紧贴抗击疫情、促进就业、服务发展、维护稳定等工作，依法科学有力有序地做好人力资源服务有关工作，为打赢疫情防控人民战争提供坚实人力资源服务支撑，现将有关事项通知如下：

一、加强重点单位用工服务。各地要激发人力资源服务机构积极性，指导公共服务机构切实发挥带头示范作用，引导经营性服务机构积极履行社会责任。聚焦疫情防控、公共事业运行、群众生活必需等涉及国计民生的重点单位，特别是与疫情防控工作直接相关的生产运输单位的用工需求，鼓励人力资源服务机构发挥信息汇聚、联通各方、专业服务的优势，综合运用线上对接、远程协作、线上专场招聘等方式，提供精准有力的供需对接服务。各地对提供职业介绍的人力资源服务机构，要根据有关规定落实就业创业服务补助政策。

二、强化线上求职招聘。各地要指导鼓励人力资源服务机构，大力拓展各类线上求职招聘服务模式，进一步创新挖潜、整合资源，通过开发全新功能，拓展服务方式，满足包括高校毕业生、农民工等重点群体在内的各类求职者的就业择业需求，为受疫情影响暂停或推迟的现场、校园等招聘活动，打造更优、更便捷的线上招聘服务平台。鼓励人力资源服务机构为受疫情影响较大或担负疫情防控重点任务的单位，适当减免招聘服务费用。

三、拓展线上培训。针对疫情防控期间用人单位和劳动者的就业创业、技能提升等多方面培训需求，鼓励人力资源服务机构及时跟进，积极开发和优化在线学习、直播课堂、热线辅导等服务项目和产品，提供各类实用型、多样化线上培训服务。鼓励有条件的人力资源服务机构，向疫情防控重点单位提供各类人力资源培训服务，对符合职业技能培训相关条件的，各地应按规定纳入补贴类培训范围。

四、加强人力资源管理咨询服务。各地要鼓励人力资源服务机构，主动对接用人单位在疫情防控期间存在的人员招聘、职位发布、简历筛选、人选沟通、聘用签约、绩效考核等人力资源管理工作需求，积极提供人力资源管理咨询、解决方案拟定等服务，助力用人单位经济高效、规范可靠地招到用好人力资源。

五、做好疫情防控人社政策宣讲。各地要指导支持人力资源服务机构及行业协会，通过各类宣传媒介，尤其是在开展人力资源服务过程中广泛运用的网络传媒、微信公众号等新媒体平台，对近期人社部门制定出台的疫情防控期间稳定就业、劳动关系、社会保障、人事人才等政策措施，进行全面深入地宣讲报道，周知人社政策，助力惠企惠民。

六、切实关心关爱职工。各地要指导鼓励人力资源服务机构及行业协会，切实关心关爱本单位职工和人力资源服务对象单位职工，尤其是滞留疫情严重地区和受疫情影响无法及时返岗职工的身心健康，通过发布公告、在线沟通等方式，积极向大家宣讲疫情防控政策措施和科学防疫健康知识，及时掌握职工动态情况，提供有针对性的职业发展咨询、心理健康援助等服务。

七、强化产业园区协同服务。各地人力资源服务产业园在做好自身防疫工作的同时，要统筹把握、稳妥有序安排园区内人力资源服务机构返岗复工，积极发挥园区产业集聚、服务齐全、功能完备的独特优势，协同开展统筹对接需求、线上专场招聘、全链条服务等活动，努力形成为疫情防控提供人力资源服务的工作合力。各地应结合实际，对在疫情防控期间发挥作用突出的人力资源服务机构，研究制定减免场地租金、给予奖励补贴等方面的措施办法。

八、加强人力资源服务需求监测。各地要加大对疫情防控期间人力资源服务需求的监测力度，深入研判疫情对人力资源服务行业发展的影响，特别要高度关注高校毕业生、农民工等重点就业群体，紧盯劳动者返岗复工、疫情防控进展等重要时间节点，充分发挥各类人力资源服务机构的专业优势，采取设点监测、线上调研、数据对比等方式，通过发布监测信息、开展预测分析、编制需求目录等手段，为做好疫情防控、服务就业大局、促进稳定发展，提供重要参考。

各地要切实提高政治站位，落实主体责任，勇于担当作为，依法合规做好疫情防控期间人力资源服务工作。同时，及时发现和宣传在疫情防控及复工复产工作中涌现出的人力资源服务先进典型，深入总结经验做法，不断提升人力资源服务能力水平。工作中遇到重要情况，请及时报告我部人力资源流动管理司。

联系人：杨 波，联系电话：010-84207252/84208244（传真）

人力资源社会保障部办公厅

2020年2月18日

（222）卫健委等2部门：做好新冠肺炎康复者捐献恢复期血浆招募动员服务工作	国卫办医函〔2020〕148号

各省、自治区、直辖市及新疆生产建设兵团卫生健康委、红十字会：

近期，为满足临床救治工作需要，各地陆续开展了采集新冠肺炎康复者恢复期血浆治疗重症、危重症患者工作。为保障捐献血浆者权益，鼓励更多的新冠肺炎康复者捐献血浆，现就做好捐献恢复期血浆相关招募动员服务工作提出以下要求：

一、做好重症、危重症患者救治、降低病死率是当前新冠肺炎疫情防控工作的突出任务，各级卫生健康行政部门、红十字会、医疗机构和血站要充分认识做好新冠肺炎康复者捐献恢复期血浆（以下简称捐献恢复期血浆）招募动员服务工作的重要性、紧迫性，加强组织领导、明确工作方案，建立工作协调机制，形成工作合力。

二、地方各级卫生健康行政部门、红十字会要在当地联防联控机制（领导小组、指挥部）指导下，进一步强化社会动员，利用多种渠道大力宣传捐献恢复期血浆的先进人物和感人事迹，弘扬无私奉献和大爱无疆的人道主义精神，营造积极的社会氛围，倡导、动员符合条件的新冠肺炎康复者踊跃捐献血浆，挽救重症、危重症患者生命。

三、地方各级卫生健康行政部门、红十字会要在当地联防联控机制(领导小组、指挥部)指导下，按照《献血法》等法律法规，协调相关部门制订出台捐献恢复期血浆的激励制度，褒扬捐献血浆、挽救他人的高尚行为。捐献恢复期血浆的新冠肺炎康复者享受无偿献血者荣誉和待遇，捐献血浆200毫升视为捐献一次全血。献血浆后，发放《献血证》并将献血浆者信息录入全国血液管理信息系统，按规定参与无偿献血表彰活动。

四、承担血浆捐献工作任务的医疗机构和血站，应当遵照《新冠肺炎康复者恢复期血浆临床治疗方案（试行第一版）》要求，严格按照临床医师对捐献者的评估意见制订血浆采集计划，不得超采、频采；组织做好捐献血浆前、中、后各项服务工作，以捐献者为中心，切实维护捐献者健康权益，保障献浆安全。地方各级卫生健康行政部门要依据《献血法》等对血站加强指导，维护正常献血秩序；地方各级红十字会应当组织志愿者积极参与咨询、服务、随访等工作。

国家卫生健康委办公厅     中国红十字会总会办公室

2020年2月18日

（223）卫健委：做好新型冠状病毒肺炎出院患者跟踪随访工作 国卫办医函〔2020〕142号

各省、自治区、直辖市及新疆生产建设兵团卫生健康委：

为做好新型冠状病毒肺炎患者治愈出院后的跟踪随访工作，实现全流程管理，更好地促进患者恢复健康，现就有关事项通知如下：

一、切实强化责任，加强部门协作

收治新型冠状病毒肺炎定点医院、康复医疗机构、疾病预防控制等专业公共卫生机构、基层医疗机构要密切配合，加强信息沟通，按职责分工共同做好新型冠状病毒肺炎患者解除隔离和治愈出院后的跟踪随访工作。定点医院、康复医疗机构和基层医疗机构要指定专人负责患者出院后的跟踪随访、康复和健康管理。疾病预防控制等专业公共卫生机构根据随访工作和健康管理需要提供技术支持。

二、细化跟踪随访工作流程

各定点医院要严格执行我委新型冠状病毒肺炎诊疗方案中关于解除隔离和出院的标准，综合临床症状、体征、实验室检查与影像学结果，组织专家综合评估，明确后续跟踪随访事项，建立专门的随访登记制度、手册和出院告知书。定点医院要做好与患者居住地基层医疗机构间的联系，共享病历资料，及时将出院患者信息推送至患者辖区或居住地居委会和基层医疗机构，同时要给出院患者开具康复指导处方，指导其赴相关医疗机构开展康复。康复医疗机构要为重型、危重型出院患者提供肺功能损害评估，根据肺功能损害程度制定合理呼吸康复计划并予以康复治疗。基层医疗机构在收到出院患者信息后，要及时对出院患者开展健康指导和健康状况监测，发生异常情况及时向定点医院报告，根据需要为轻型、普通型出院患者提供社区康复服务或居家康复指导。

三、科学有序落实跟踪随访措施

各定点医院要做好出院患者的复诊安排，在患者出院时为其安排好出院2-4周后的复诊计划，并预约好第一次复诊时间。复诊时重点复查血常规、生化、氧饱和度，必要时复查新冠病毒病原学检测。有肺炎的患者，复查胸部CT影像学检查，了解肺部炎症吸收情况。基层医疗机构要加强对出院患者的健康管理，及时了解患者体温、呼吸道症状，重点加强对老年人和患有高血压、糖尿病等慢性基础疾病的出院患者的监测。一旦发现发热、咽痛、胸闷等不适症状，指导患者尽快到设有发热门诊的上级医院就医。

四、充分运用信息手段开展随访

各地要依托区域全民健康信息平台，努力做到居民健康档案、电子病历、出院随访档案等信息共享和业务协同，实现新型冠状病毒肺炎患者临床诊治与健康管理的全闭环。指导社区家庭医生和乡村医生通过家庭医生签约APP、有线电视网络、电话、微信、智能语音提醒等多种手段与辖区内管理的出院患者开展信息互动。

五、加强出院患者健康指导

各定点医院要指导出院患者做好自我健康管理，引导出院患者主动、及时向居住地居委会、基层医疗机构报告个人信息和健康状况。患者出院后，应当继续进行自我健康状况监测，佩戴口罩，有条件的居住在通风良好的单人房间，并减少与家人的密切接触，分餐饮食，做好手卫生，减少外出活动。

国家卫生健康委办公厅

2020年2月17日

（224）工信部：做好宽带网络建设维护助力企业复工复产有关工作	工信厅通信函〔2020〕25号

各省、自治区、直辖市通信管理局，中国电信集团有限公司、中国移动通信集团有限公司、中国联合网络通信集团有限公司、中国铁塔股份有限公司：

为认真贯彻落实习近平总书记关于新冠肺炎疫情防控工作重要指示精神和党中央、国务院决策部署，有序推进疫情防控和企业复工复产，现就做好宽带网络建设维护，助力企业复工复产有关工作通知如下：

一、切实提高政治站位。固定和移动宽带网络已成为广大人民群众生产生活的必需品，做好当前宽带网络建设维护，对于疫情防控和企业复工复产具有重要支撑作用。各单位要深入学习贯彻习近平总书记关于疫情防控工作的重要指示精神，增强“四个意识”、坚定“四个自信”、做到“两个维护”，切实扛起责任、主动作为，坚持疫情防控和经济发展两手抓两不误，抓实抓细宽带网络建设维护工作，不断提升服务用户和企业能力，助力企业加快复工复产。

二、建立协调推动机制。各通信管理局要与当地电信企业、铁塔公司建立助力企业复工复产的工作机制，坚持问题导向，明确工作职责，细化工作方案，因地制宜，协调联动，共同做好宽带网络建设维护工作。各通信管理局要加强与地方政府部门的沟通，做好与当地疫情防控和企业复工复产工作的衔接，推动解决宽带网络建设维护中的防护物资紧缺、现场无法进入、人员复工复岗难等问题。各企业集团公司和总部要落实建设维护工作主体责任，加强对各级公司的指导，充分利用信息化手段，提升企业快速响应能力，主动发现和及时响应客服、媒体、互联网等各个途径反映的问题，全力做好建设维护工作。工业和信息化部与各地通信管理局、各企业集团公司和总部建立部省、部企信息化工作机制，统筹协调指导各地开展工作。

三、积极对接宽带网络需求。各企业要强化责任意识和服务意识，高度重视宽带网络需求。疫情防控期间，要进一步强化线上服务、电话服务等非接触式服务能力和远程指导能力，提升服务成效。要结合各地实际，有序组织人员复工，统筹调度人员，提高工作效率。对包含企业宽带和专线用户在内的宽带网络业务咨询、受理、变更、投诉等需求，要尽量提供非接触式服务方式。对故障处理等需求，在与用户协商一致情况下，优先远程指导用户修复故障，降低感染风险。对确需上门处理的固定宽带网络安装调测、移动宽带网络建设优化及故障处理等需求，分区域分情况对待。具备上门条件的，要加强对工作人员管理，落实防疫措施和责任，及时响应用户需求。因疫情防控而不具备上门条件的，要及时耐心与用户沟通，研究其他解决方案，获得用户理解；确实无法解决的，待具备上门条件后第一时间予以解决。

四、主动提升网络服务能力。各企业要加强对宽带网络运行状态的监测和巡检，提前发现并排除故障隐患。要及时处理已受理的宽带网络业务需求，做好宽带网络优化和升级，畅通业务咨询、服务和投诉渠道，按要求为用户提供服务。同时，要对因疫情隔离导致人员不能外出的网络覆盖区域采取针对性保障措施，为群众生活和企业复工复产提供安全稳定的网络环境。

五、保障人员安全。各企业要加强对建设维护一线人员及相关合作单位的管理，落实好检测筛查、通勤保障、个体防护等疫情防控措施。要优先保障上门建设维护所需口罩等防护物资需要，合理安排作息时间，确保人员身心健康。

工业和信息化部办公厅

2020年2月19日

（225）人社部等5部门：做好农民工返岗复工“点对点”服务保障工作	人社部明电〔2020〕4号

各省、自治区、直辖市及新疆生产建设兵团人力资源社会保障厅（局）、公安厅（局）、交通运输厅（局、委）、卫生健康委，各铁路局集团公司：

为坚决贯彻落实习近平总书记关于做好新冠肺炎疫情防控工作的重要指示精神，认真落实国务院联防联控机制关于切实加强疫情科学防控、有序做好企业复工复产工作的决策部署，对于已有工作岗位和新确定工作岗位拟进城务工的农民工，组织开展对用工集中地区和集中企业“点对点”的农民工专车（专列）运输服务，保障成规模、成批次外出的农民工安全有序返岗复工，现就有关事项通知如下：

一、摸清出行需求。人力资源社会保障、交通运输部门通过电视广播、官方网站、微信、就业信息平台、交通运输出行平台等渠道，广泛宣传农民工返岗复工“点对点”服务保障的信息，开通线上线下报名渠道。县级人力资源社会保障部门要全面摸排有意愿外出农民工的信息，鼓励已确定工作岗位的农民工积极报名，汇集农民工出行人数、时间、地点、用人单位等信息，动员时间段相近、务工目的地相近的农民工集中时间出行，就省内就业、跨省出行、集中地区、集中企业等进行分类，并逐级上报至省级人力资源社会保障部门，形成外出农民工基础数据备核。

二、开展行前服务。县级人力资源社会保障部门将有出行需求的农民工人员名单、集中返岗出行时间、地点等相关信息提供给交通运输部门和卫生健康部门。交通运输部门根据农民工集中返岗需求，制定专门包车运输方案，为目的地集中、具有一定规模的农民工提供“点对点”直达服务。对省际客运需求及时报省级交通运输部门统筹做好省际间协调和沟通。卫生健康部门要指导做好农民工的防疫健康教育和行前体温检测工作，发现异常情况及时报告处置。充分利用国家政务服务平台同行密接自查服务、中国政府网新冠肺炎患者同乘接触者查询系统，加强排查识别，阻断风险人员外出。对于跨省外出农民工，省级人力资源社会保障部门要指导输出地县级人力资源社会保障部门在行前主动与输入地人力资源社会保障部门对接出行人数、到达时间和地点、用人单位等信息，输入地人力资源社会保障部门要与用人单位确认相关信息，对接当地有关部门，做好人员接收准备。

三、组织返岗运输。各地交通运输部门制定运输方案时要优先保障成规模、成批次、目的地集中的农民工出行，提前集中登记信息，按照“一车一方案”的原则，开展“点对点”直达运输。督促指导农民工返岗包车运营单位按照《交通运输部关于全力做好农民工返岗运输服务保障工作的通知》（交运明电〔2020〕56号）要求，全面做好运输组织、落实各项疫情防控措施和乘客信息登记、转交工作，确保运输过程安全、顺畅、有序。积极争取农民工输入、输出地方政府加大农民工返岗包车的支持，通过政府购买服务或者补贴部分包车费用的形式，降低农民工出行成本。鼓励出台相关优惠政策，引导有条件的用工企业主动承担农民工返岗包车费用。各地人力资源社会保障部门与铁路运输部门根据农民工返岗需求，开行火车专列或安排外出农民工包车厢运输，交通运输部门组织做好道路客运班线和城市公交等与铁路班次运力的衔接，打通农民工返岗“最先和最后一公里”。各地公安部门要加大在火车站、汽车站等重点部位的巡逻防控，做好“点对点”包车的道路通行保障和交通安全管理，对突发事件及时进行处置。

四、做好抵达地交接。接收地各相关部门要在当地人民政府的统一领导下，做好专车抵达后的交接工作。人力资源社会保障部门要及时对接有关企业组织接收农民工入厂。企业要为返岗复工农民工建立个人健康档案，随访健康状况，做到全覆盖，确保达到防疫要求、体温测量正常后方可上岗。对于行前14天内和在途没有相关症状的，要尽快复工。对于出现发热、乏力、咳嗽等不适症状的，应立即进行隔离观察，及时送诊排查。

五、加强上岗防护保障。在地方人民政府及疫情防控领导小组领导下，各有关部门要指导督促企业严格落实疫情防控主体责任，完善相应设施设备，提供卫生和隔离观察场所，开展环境卫生整治和重点场所消毒，开展员工健康状况监测，为职工配发口罩等防护用品，确保疫情防控措施落实落地，坚决防止因复工造成聚集感染。

六、加强组织领导。各地各有关部门要认真落实党中央、国务院的决策部署，把引导农民工安全有序返岗作为做好复工复产的重点工作任务，实现防疫防控与稳定农民工就业两不误。各地要在疫情防控领导小组领导下，建立人力资源社会保障部门牵头，公安、交通运输、卫生健康等部门和铁路集团公司参加的农民工返岗复工“点对点”服务协作机制，明确工作责任，加强沟通衔接，加大信息共享力度，形成工作合力，确保输出有组织、健康有检测、承运有防护、到达有交接、全程可追溯。要加大对农民工返岗复工有序出行专项行动典型经验和做法的宣传推广力度，广泛动员和鼓励社会力量为农民工返岗复工做好疫情防控提供帮助，营造良好的社会氛围。

人力资源社会保障部

公安部

交通运输部

国家卫生健康委

国家铁路集团

2020年2月19日

（226）财政部：加强新冠肺炎疫情防控财税政策落实和财政资金监管工作	财办〔2020〕11号

各省、自治区、直辖市、计划单列市财政厅（局），新疆生产建设兵团财政局，财政部各地监管局：

为打赢新冠肺炎疫情防控阻击战，支持企业复工复产、保障经济运行，中央财政和地方陆续出台相关财税政策，不断加大财政资金支持力度。现就加强相关财税政策落实和财政资金监管有关事项通知如下：

一、加强督促指导，确保疫情防控财税政策落到实处

各级财政部门要认真学习针对疫情防控出台的有关经费保障、财政补助、税收优惠、金融支持、政府采购等相关政策措施，理解领会吃透政策精神。加强政策宣传解释和辅导，确保患者、参与疫情防控的医务及相关人员、有关医疗卫生机构“应享尽享”救助补助或经费保障政策，确保疫情防控重点保障企业和个人尽享财税支持政策，及时获得资金支持。密切跟踪相关财税政策落地实施情况，及时了解政策实施中遇到的困难和问题，加强疫情对当地经济发展和财政收支影响的分析，研究提出相关政策调整和完善建议。

省级财政部门要加强对市县级财政部门的业务指导，认真落实《财政部关于进一步做好新型冠状病毒感染肺炎疫情防控经费保障工作的通知》（财办〔2020〕7号）等文件精神，指导当地相关部门和基层财政部门落实好各项财税政策。

财政部各地监管局要积极协助地方财政部门细化出台相关实施细则或操作指引，明确患者费用补助、临时性工作补助、税收优惠、财政贴息、物资采购政策等实施范围、使用方向、补助对象、补助标准、申报流程等，确保疫情防控的各项财税政策落实落细。

二、强化财政监管，确保疫情防控财政资金用在实处

各级财政部门要切实加强疫情防控财政资金监管，重点关注与疫情防控有关的患者救助费用补助、临时性工作补助、财政贴息、物资采购等有关财政资金的审核、拨付、管理使用等情况，督促指导相关部门规范疫情防控资金财务管理和会计核算，切实保障资金使用安全合规有效。疫情防控期间以非现场监管为主，灵活采用线上报送、线上跟踪等“非现场、不见面”的“互联网+监管”方式，利用视频会议、电话沟通、微信联系等手段，有效有序开展监管工作。对于疫情防控财税政策落实不到位，财政资金分配、资金补助不合理等问题，要及时予以纠正。要充分发挥舆论和群众监督的作用，及时向社会公开相关财税政策和资金分配等信息，通过网络、电话等多种形式，及时受理群众举报，发现问题严肃处理。疫情结束后结合实际适时开展现场监管，严肃财经纪律，查处违法违规行为。涉及重大违法违纪问题，要及时移交纪检监察或司法机关处理。

地方各级财政部门要加强中央转移支付以及地方一般公共预算、政府性基金预算、社会捐赠等各项资金的统筹，按照“特事特办、急事急办”原则，加快预算执行，及时、足额拨付各项疫情防控资金。

财政部各地监管局要及时掌握中央财政补助资金下达情况，建立疫情防控资金台账。督促地方财政部门统筹做好资金调度、垫付，优先保障和拨付疫情防控资金，切实保障基层疫情防控及“三保”支出需求。对资金使用中存在的困难和问题，要及时反映并提出改进建议。发现地方财政部门将中央专项转移支付用于平衡预算，以及资金分配、资金补助不合理等问题，要及时指出并督促纠正；对擅自截留、挤占、挪用疫情防控资金，购置与疫情防控无关物资等违法违规行为，要督促地方严肃查处。

三、做好绩效评价，提高疫情防控财税政策和财政资金绩效

疫情防控工作结束后，各地财政部门和财政部各地监管局要及时对相关财税政策落实和财政资金使用效果开展绩效评价。要结合出台的财税政策和财政补助资金确定的工作目标和要求，科学设计绩效评价指标，全面收集各项数据信息，进行必要的现场调研。围绕政策落实和资金管理使用的时效性、公平性、有效性开展评价，重点关注政策和资金是否及时、精准到位，资金补助标准是否科学合理，资金分配是否公开透明、公平公正，防控政策和资金是否达到预期效果等。根据评价情况，系统总结政策落实和资金使用中好的做法和经验，分析存在的不足和问题，有针对性的提出意见建议，作为调整完善财税政策、加强资金管理的重要参考和依据。

财政部各地监管局的重点绩效评价和绩效自评抽审工作由财政部统一组织实施，地方财政部门绩效评价工作按照财政部的有关要求自行组织开展。

四、改进工作作风，切实提高监管工作实效

（一）提高政治站位，强化责任担当。加强新冠肺炎疫情防控财税政策落实和财政资金监管，是保障相关财税政策取得实效和财政资金安全规范使用的重要举措。各级财政部门要认真落实习近平总书记“切实把各项工作抓实、抓细、抓落地，坚决打赢疫情防控的人民战争、总体战、阻击战，努力实现今年经济社会发展目标任务”的要求，进一步提高政治站位，树牢“四个意识”，做到“两个维护”，认真履职，勇于担当，切实做到守土有责、守土担责、守土尽责。

（二）加强沟通协调，形成监管合力。财政部各地监管局和地方财政部门要加强沟通协调，既要各尽其责，又要相互支持，强化数据共享、信息互通。各级财政部门要注重同当地人行、银保监、税务、发改以及纪检监察、审计等部门的协调配合，推动形成监管合力。

（三）坚持实事求是，改进工作作风。各级财政部门既要严格执行工作程序和内部控制要求，又要充分考虑疫情防控非常时期的特殊情况，坚持具体情况具体分析，注意听取多方意见建议，全面、客观、审慎看待发现的问题，实事求是作出判断。要减少基层财政数据统计、报表填报、文件报送等工作，坚决杜绝形式主义、官僚主义，做到高效有序监管。

财 政 部

2020年2月17日

（227）发改委等16部门：新冠肺炎疫情防控下做好2020年春耕化肥生产供应工作	发改电〔2020〕394号

各省、自治区、直辖市、新疆生产建设兵团发展改革委、工业和信息化厅（局）、公安厅（局）、财政厅（局）、人力资源社会保障厅（局）、交通运输厅（局）、农业农村厅（局）、商务厅（局）、卫生健康委、人民银行各分支机构、海关总署各直属海关、市场监督管理局、能源局，供销合作社，各铁路局集团公司，农业发展银行各分支机构，各有关行业协会，各有关企业：

2020年春耕即将自南向北陆续展开，而近期国内化肥生产流通因新冠肺炎疫情受到一定影响。为不误农时抓好春耕备肥，切实保障化肥等农资生产供应和价格基本稳定，现就有关事项通知如下。

一、加快化肥生产企业复工复产

化肥行业是涉及国计民生的重要领域，各地要切实贯彻落实国务院应对新型冠状病毒感染肺炎疫情联防联控机制关于切实加强疫情科学防控有序做好企业复工复产工作的通知精神，在做好疫情防控的基础上，有力有序有效推动化肥及其原辅料生产企业复工复产，重点支持磷复肥生产企业复工复产，努力增加春耕前化肥产量。国家能源集团、中煤集团、陕煤集团等煤炭生产企业要在块煤、烟煤等原料供应方面，向煤制化肥生产企业倾斜。中石油、中石化、中海油等天然气生产企业要在保障民生用气前提下，重点保障气制化肥生产企业用气需求。鼓励供煤供气企业与化肥生产企业签订中长期协议，严禁以各种理由不履行协议，严禁在协议约定以外收取不合理费用。对化肥生产企业复工复产中的用工短缺问题，要积极协助技术人员返工返岗。

二、保障化肥等农资及原辅料运输畅通

我国化肥产销具有生产集中、使用分散的特点，在当前疫情防控情况下，化肥生产原辅料、产成品运输及配送面临较大困难。各地交通运输、公安交管部门要切实保障农业生产急需的化肥、农药、种子等农资，以及生产化肥所需煤炭、磷矿石、硫磺、包装袋等原辅料运输通畅，确保优先便捷通行。铁路部门要组织安排运力，优先保障山西、新疆、内蒙古等氮肥主产地，云南、贵州等磷肥主产地，青海、新疆等钾肥主产地化肥，以及煤炭等化肥生产所需原料外运需要，必要时启动重点物资运输保障机制。对于已到站但无法及时卸载的化肥等农资，协商减免延时费、滞纳金、仓储费等相关费用。

三、做好储备和进出口调节

发挥好储备吞吐调节作用，对国家已安排的化肥淡季商业储备，各承储企业要提前将储备化肥调运至销区基层网点，春耕用肥旺季到来后要及时销售，努力满足春耕集中用肥需要。各省级发展改革委要会同有关方面加强对本地区国家化肥淡储任务的督促落实，指导各承储企业及时足量投放。已建立地方化肥淡季商业储备的地区，要切实发挥好储备保春耕作用。中农集团、中化集团要发挥好钾肥储备的作用，根据化肥市场形势及时投放。各地海关要严格按照相关标准对进出口化肥质量进行检验，各化肥行业协会要引导化肥生产流通企业将国产化肥集中供应国内市场，相关化肥贸易企业要根据国内市场肥种余缺情况组织好化肥进口，保障春耕用肥需要。

四、发挥农资流通主渠道作用

在做好疫情防控的基础上，各地要支持化肥流通企业及基层农资经营网点复工营业。针对农资流通环节备肥日益减少、农民随用随买的现状，供销合作社系统要充分发挥农资流通主渠道作用，抓紧对接化肥、农药、种子等农资生产企业货源，重点地区要高于往年一定数量购肥备肥，并利用农资销售网络点多、面广的优势，尽快将货物运送到基层零售网点，满足春耕期间农民就近购买农资的需要。各地发展改革部门要会同有关部门指导农资流通骨干企业加大购肥备肥力度，帮助巩固并拓展农资销售网络，对接基层农资零售网点，加快充实乡村一级农资库存；指导大型农资电商平台建立健全线下农资配送网络，帮助传统农资经销商向线上线下融合发展的现代服务商转型，在方便农民购买农资的同时，提供农业生产综合服务。各地供销、邮政和骨干农资企业，要建立乡村农资应急配送机制，保障农资即时配送，打通农资流通“最后一公里”。

五、加大金融和农化服务支持

人民银行运用再贷款、再贴现工具，支持金融机构对包括化肥生产流通企业在内的涉农行业扩大信贷投放。商业银行要加大信贷支持力度，提高审贷效率，改善金融服务。农业发展银行要结合职能定位，发挥政策性金融机构作用，对国家化肥淡储承储企业择优提供信贷支持。各级农业农村部门要加强技术指导服务，向农民普及科学施肥知识，提供选肥、用肥指导，倡导使用环保、高效新型肥料，普及推广测土配方施肥技术，大力发展专业农化服务组织，引导农民合理用肥、高效施肥，降低用肥成本。

六、加强农资市场监测监管

各地发展改革、农业农村等部门要加强对化肥等农资市场供应和价格情况的监测分析，对价格异动要及时预警，各有关方面要通力协作，及时采取措施。各地农业农村、市场监管等部门要结合春季农资打假工作，依法严肃查处掺杂使假、偷换养分、虚标含量以及价格欺诈、哄抬价格、串通涨价等违法违规行为，并通过新闻媒体公开曝光，维护正常流通秩序。充分发挥好12315等投诉举报电话作用，畅通消费者维权渠道，鼓励社会各界积极参与农资打假工作，切实维护企业和农民合法权益。

2020年是全面建成小康社会目标实现之年，是全面打赢脱贫攻坚战收官之年。保障春耕期间化肥供应和价格稳定关系到全年农业生产稳定发展和农民稳定增收，对实现今年经济社会发展目标意义重大。地方政府和有关部门要高度重视，一手抓新冠肺炎疫情防控，一手抓春耕农业生产，两手抓、两手都要硬，按照本通知要求，认真落实国务院联防联控机制的有关部署和要求，及时协调解决化肥生产供应中的重大问题，扎扎实实做好春耕化肥等农资市场保供稳价工作。湖北省要集中精力抓好新冠肺炎疫情防控工作，后期可根据疫情防控工作进展，稳妥有序做好春耕化肥等农资生产供应工作。

国家发展改革委

工业和信息化部

公  安  部

财  政  部

人力资源社会保障部

交 通 运 输 部

农 业 农 村 部

商  务  部

卫 生 健 康 委

人 民 银 行

海 关 总 署

市场监管总局

国 家 能 源 局

供销合作总社

国 铁 集 团

农业发展银行

2020年2月17日

（228）工信部：运用新一代信息技术支撑服务疫情防控和复工复产工作	工信厅信发〔2020〕4号

各省、自治区、直辖市及计划单列市、新疆生产建设兵团工业和信息化主管部门，各省、自治区、直辖市通信管理局，各有关单位：

为贯彻落实习近平总书记关于“鼓励运用大数据、人工智能、云计算等数字技术，在疫情监测分析、病毒溯源、防控救治、资源调配等方面更好发挥支撑作用”的重要指示精神和党中央国务院关于切实加强疫情科学防控、有序做好企业复工复产工作的决策部署，充分运用新一代信息技术支撑服务疫情防控和企业复工复产工作，现就有关事项通知如下。

一、运用新一代信息技术全面支持疫情科学防控

（一）支持运用互联网、大数据、云计算、人工智能等新技术服务疫情监测分析、病毒溯源、患者追踪、人员流动和社区管理，对疫情开展科学精准防控。

（二）依托互联网平台开展医疗防疫物资的供需精准对接、高效生产、统筹调配及回收管理。

（三）组织信息技术企业与医疗科研机构联合攻关，利用人工智能、大数据、5G等技术，加快病毒检测诊断、疫苗新药研发、防控救治等速度，提高抗疫效率。

（四）引导企业加强互联网应用能力，充分运用网上疫情防控资源和信息化工具，建立线上线下、联防联控的管理体系。

（五）支持完善疫情期间网络零售服务和物流配送体系，加强电子图书、影视、游戏等领域数字文化产品和服务的开发，形成丰富多样的“零接触”购物和娱乐模式，确保百姓生活必需品和精神营养品供应。

二、运用新一代信息技术加快企业复工复产

（六）针对疫情对企业造成的停工停产问题，指导企业用好信息技术手段和信息化工具，增强软件应用能力，创新思路和方法，用两化融合提升生产管理水平，助力企业尽快复工复产。

（七）推动制造企业与信息技术企业合作，深化工业互联网、工业软件（工业APP）、 人工智能、增强现实/虚拟现实等新技术应用，推广协同研发、无人生产、远程运营、在线服务等新模式新业态，加快恢复制造业产能。

（八）发挥大型平台企业和行业龙头企业的作用，通过工业互联网平台保障供应链的完整，做好生产协同和风险预警。对于可能停产断供的关键环节，提前组织柔性转产和产能共享，以信息化手段管控好供应链安全。

（九）面对疫情对中小企业复工复产的严重影响，支持运用云计算大力推动企业上云，重点推行远程办公、居家办公、视频会议、网上培训、协同研发和电子商务等在线工作方式。

（十）支持互联网交通、物流、快递等生产性服务企业率先复工复产。支持工业电子商务企业和物流企业高效协同，运用互联网、大数据、区块链等技术完善智慧物流体系，打通生产生活物资流通堵点，保障生产资料和生活用品有效供给。

（十一）大力推动产融结合。加快发展基于生产运营数据的企业征信和线上快速借贷，推广应用供应链金融、知识产权质押等融资方式，保障企业复工复产的资金需求，防止出现资金链断裂。

三、强化服务保障

（十二）助力各项政策落地落实。利用互联网技术支持疫情防控和复工复产政策措施的快速部署、快速落地，基于大数据技术开展政策实施跟踪和成效精准分析，通过人工智能等技术做好相关咨询服务，围绕疫情防控要求切实做到“数据多跑路，企业少跑腿”。

（十三）强化提升企业两化融合能力。针对企业在疫情防控期间暴露出的应急响应、供应链管控、产业链协同等方面的问题，通过标准宣贯、展示推广、系统培训和精准引导，加快推动企业应用新一代信息技术构建新型能力体系，提升企业应急响应速度和平稳快速复工复产能力。

（十四）强化数字基础设施保障。协同推动网络和计算等数字资源的有力保障，优先保障基础软硬件企业复工复产，满足全面恢复生产对数字基础设施扩容增量等需求。

（十五）搭建数据融通平台，对企业复工率、到岗率、开工率等进行全方位实时监测，面向企业提供政策咨询、复工管理、防疫培训、招工用工、财税支持等精准服务。

（十六）强化统筹协调。各地工业和信息化主管部门、通信管理局要密切配合，及时总结和宣传推广一批好经验好做法。有关情况请及时报送工业和信息化部（信息技术发展司）。

工业和信息化部办公厅

2020年2月18日

（229）商务部：应对新冠肺炎疫情做好稳外贸稳外资促消费工作	商综发〔2020〕30号

各省、自治区、直辖市及计划单列市、新疆生产建设兵团商务主管部门：

为深入贯彻习近平总书记关于疫情防控工作重要讲话和指示批示精神，落实党中央、国务院决策部署，坚决打赢疫情防控的人民战争、总体战、阻击战，统筹做好疫情防控和经济社会发展，积极做好稳外贸稳外资促消费工作，把疫情对商务发展的影响降到最低，现就有关事项通知如下：

一、支持企业有序复工复产。按照当地统一安排和规定条件，协调复工审核部门加快办理手续，支持外贸、外资、商贸流通和电子商务企业有序复工复产，稳妥有序推进共建“一带一路”重大项目。引导生产防护用品类的外贸外资企业扩大生产。鼓励外贸企业增加国内紧缺的医用物资和农产品进口。积极协调有关部门，将承担生活必需品保供任务的重点商贸流通企业和电子商务企业纳入防护物资优先保障范围，优先配备口罩、消毒液、防护手套、护目镜等必要的防护物资。

二、简化对外经贸管理程序。引导企业无纸化申领进出口许可证件。全面实现最终用户和最终用途证明申办系统无纸化，加速处理与疫情防控相关物项的办理申请。开展两用物项和技术进出口许可跨部门电子会签。推进技术进出口合同登记申请无纸化，抓紧启动电子证照工作。在疫情解除之前，依据企业网络传输资料，在线审核服务外包合同。加快实行对外投资备案（核准）无纸化管理，实现全流程网上办理。

三、强化法律服务帮助企业降低风险。支持贸促会、商会等为外贸企业和境外项目实施主体无偿出具因疫情导致未能按时履约的不可抗力事实性证明。协调国内外组展机构，帮助因疫情无法出国参展的企业妥善处理已付费用等问题。协调中介机构为受影响的走出去企业提供法律等咨询服务。发挥各级应对贸易摩擦工作站作用，提供法律服务，帮助企业解决疫情对贸易救济案件应对造成的困难。

四、支持外贸新业态新模式发展。指导跨境电商综试区提供海外仓信息服务，帮助企业利用海外仓扩大出口。支持市场采购贸易与跨境电商融合发展，探索试点市场闭市期间成交新渠道。积极推进二手车出口，为受疫情影响的企业提供便利服务。积极与周边国家沟通协调，疫情解除后尽快恢复开展边民互市贸易，支持边境贸易发展。

五、加强出口信用保险支持。加强与中信保公司驻各地营业机构协作，充分发挥出口信用保险作用，进一步扩大出口信用保险覆盖面，合理降低短期险费率，加大对受疫情影响企业支持力度，帮助企业应对订单取消、出运拒收等风险。开辟理赔服务绿色通道，在贸易真实的情况下适当放宽理赔条件，做到应赔尽赔、能赔快赔。积极开展政府+银行+保险合作，扩大保单融资规模。

六、积极应对境外贸易限制措施。充分发挥贸易救济预警体系和法律服务机制作用，及时发布各国针对疫情出台的贸易限制措施，畅通企业反映问题渠道。关注本地区出口产品在国外遇到不合理限制情况，及时上报，配合驻外使（领）馆经商机构加大双边交涉力度，配合在世界贸易组织发声、做相关方工作，敦促各成员遵守世界贸易组织规则、尊重世界卫生组织权威和专业意见，反对有关国家（地区）过度反应和施加不必要的贸易限制。

七、深化服务贸易创新发展。发挥服务贸易创新发展引导基金导向作用，支持符合条件的服务贸易企业创新发展。落实好非商业性境外办展政策，对受疫情影响较大的地区和企业适当予以政策倾斜。对于已在商务部审批或备案的涉外经济技术展览会，因疫情影响已确定取消或推迟的，申办单位可通过商务部统一平台取消办展或调整办展时间。对商务部实施的两种涉外经济技术展行政许可事项全面推行在线审批。

八、用好自贸协定优惠政策。利用中国自贸区服务网等平台，做好我国与有关国家和地区已签署自贸协定的宣传推广，鼓励企业用好用足自贸协定中的关税减免、投资促进等优惠政策。

九、稳定外资企业信心。实施好外商投资法及实施条例等法律法规。指导外资企业用足用好应对疫情各项支持政策措施，做到内外资企业同等对待、一视同仁，发挥外资企业投诉机制作用，保障外资企业同等适用支持政策。根据本地实际情况，在法定权限内制定支持外资企业应对疫情影响、加大稳外资力度专项政策，相关政策措施和做法及时报商务部复制推广。

十、加强外资大项目跟踪服务。密切跟踪在谈外资大项目，分类施策、一企一策，精准研究支持政策，及时协调解决项目推进过程中的困难和问题。对于在建外资大项目，开展“点对点”服务保障，协调解决用地、用工、水电、物流等问题，保障企业投资按计划进行。对于大项目推进过程中涉及中央事权的共性政策问题，及时上报。

十一、创新和优化招商引资方式。通过网上洽谈、视频会议、在线签约等方式，整合各类招商资源，持续推进投资促进和招商工作。充分利用各种招商机构和平台，加大投资环境和合作项目宣传推介力度。加强与境外各类商协会等中介组织合作，积极开展委托招商、以商招商，组织灵活多样的招商活动，争取一批新项目签约落地。

十二、指导开放平台抓防控促发展。支持自由贸易试验区、国家级经济技术开发区加强用工、供应等跨区域对接，缓解疫情期间企业用工、配套困难。指导自由贸易试验区加快改革开放创新试点，围绕试点领域涵养客商资源，培育壮大外向型经济。加快北京市服务业扩大开放综合试点措施落地，抓紧开展试点经验总结评估及复制推广。

十三、加强外资企业动态监测。完善外商投资信息报告制度，充分运用信息化手段，加强研判和预警，及时掌握各行业、各领域外资企业受疫情影响情况。强化与外资企业协会和外国商会的联系机制，及时了解会员企业生产经营状况和投资动向，有针对性地加强服务和指导，特别是对龙头企业和产业链关键环节企业的服务保障。

十四、设立海外疫情应对快速反应机制。发挥境外企业和对外投资联络服务平台作用，加强运行监测、风险提示、应对指导和服务保障，指导企业有效防范风险，推进高质量共建“一带一路”。充分发挥境外中资企业商（协）会作用，在境外中资企业商会联席会议机制办公室设立海外疫情应对快速反应机制，及时搜集企业遇到的实际困难和问题，做好协调服务。

十五、支持商贸流通企业创新经营服务模式。鼓励开展商务领域经营互助，引导零售企业与餐饮、住宿等生活服务企业通过共享员工等方式，缓解零售企业用工短缺困难，着力稳定相关行业工作岗位。积极协调有关部门和小区物业，在符合各地相关规定和保证食品卫生安全的前提下，支持企业采取店铺外摆、露天市场、团购、无店铺等方式进行销售，便利社区居民生活。

十六、加快步行街改造提升。加快推出一批全国示范步行街，启动第二批步行街改造提升试点。已经确定的改造施工项目，可积极创造条件尽早启动。指导步行街管理机构在认真做好疫情防控的前提下，统筹协调各类商户有序复工营业。超前谋划、合理安排，在疫情得到有效控制后，组织形式多样的促消费活动，充分释放被疫情抑制的消费潜力。

十七、推动服务消费提质扩容。落实生活服务业疫情防控指南，指导企业规范服务流程，切实保障服务人员和消费者身体健康。开展社区生活服务业发展试点，引导餐饮等生活服务企业在做好疫情防控的前提下积极下沉社区，为受疫情影响的社区居民提供安全的生活服务。

十八、释放新兴消费潜力。发展网络消费，引导电子商务企业以数据为纽带，精准匹配网络消费新需求，打造“小而美”的网络新品牌。提高农产品可电商化水平，扩大农产品网络消费。开展网络促销活动，促进网上品牌品质消费。鼓励电商、快递等企业与实体店、商务楼宇和小区物业等合作，开展末端配送服务合作。鼓励探索无直接接触配送服务，推广定点收寄、定点投递等方式。鼓励生活服务企业拓展线上销售，采用“中央厨房+线下配送”等新发展模式经营。

十九、发展便利店和菜市场。优化便利店网点、菜市场布局，充分发挥经营灵活、贴近社区居民的优势，满足居民生活必需品需求，打造“一刻钟便民生活服务圈”。引导商贸流通企业和电子商务平台经营者，为小店提供集采集配、供应链融资、先供货后付款等服务。鼓励小店开展网络营销拓展业务，发挥小店在促进就业、扩大消费、提升经济活力、服务改善民生等方面作用。

二十、发挥好财政资金作用。商财政部门抓紧安排使用提前下达的2020年中央财政专项资金，加大结存资金统筹使用力度，用好地方配套资金，带动社会资本，全力支持稳外贸稳外资促消费。外经贸发展专项资金可向受疫情影响较大的领域倾斜，重点支持企业开拓国际市场、以政银保合作方式加大贸易融资支持、外商投资促进服务、开放平台吸引外资、“一带一路”投资合作等。服务业发展资金中，电商进农村综合示范资金重点支持农产品进城、工业品下乡，促进疫情防控期间的农产品销售，农商互联农产品供应链资金增加支持农产品保供方向，流通领域供应链体系建设资金要强化生活必需品供应链功能作用，未安排的结余资金可统筹用于支持应对疫情的商贸流通相关工作。上述资金安排应避免与其它政策平台、资金渠道等交叉重复。

各级商务主管部门要以习近平新时代中国特色社会主义思想为指导，增强“四个意识”、坚定“四个自信”、做到“两个维护”，坚决贯彻党中央、国务院决策部署，牢记初心使命，强化责任担当，积极主动作为，加强横向协作、纵向联动，统筹疫情防控和商务发展，依法推进各项工作，全力以赴稳外贸稳外资促消费，努力完成全年目标任务，更好地服务经济社会发展大局，为全面建成小康社会多作贡献。

商 务 部

2020年2月18日

（230）卫健委：加强新冠肺炎疫情期间严重精神障碍患者治疗管理工作	肺炎机制综发〔2020〕70号

各省、自治区、直辖市及新疆生产建设兵团应对新型冠状病毒肺炎疫情联防联控机制（领导小组、指挥部）：

在新冠肺炎疫情中，严重精神障碍患者也不可避免地受到疫情影响。据统计，目前已有323名严重精神障碍患者被确诊患新冠肺炎，有43名为疑似患新冠肺炎，范围涉及全国17个省份，病例分布以武汉市为主，孝感市、黄冈市也有部分病例，其他地区以散发为主。为落实国务院应对新冠肺炎疫情联防联控机制的总体部署，各地应当做好疫情期间严重精神障碍患者管理治疗工作，加强住院和居家的严重精神障碍患者管理治疗和社区照护，防范院内感染，降低患者肇事肇祸风险。现将有关事项通知如下：

一、做好精神卫生医疗机构疫情防控，防范院内感染

精神卫生医疗机构要做好新冠肺炎疫情防控工作，制定院内感染应对预案，储备防护物资，与当地具有新冠肺炎诊疗能力的综合性医疗机构建立联络会诊机制；开展全员培训，加强新冠肺炎症状和发热监测；疫情期间，落实院内感染各项防范措施，减少探视预防感染，做好病区清洁和消毒管理。有条件的机构，设立发热病区，改造医务人员和患者通道，降低交叉感染风险。

对新入院患者应当进行门诊筛查，详细询问新冠肺炎流行病学接触史，做好相关检查。对住院的严重精神障碍患者发现有疑似或者确诊新冠肺炎的，当应立即采取隔离措施，将患者转诊到定点医院治疗，并及时向当地卫生健康行政部门报告。对暂时无法转出到定点医院的确诊患者，精神卫生医疗机构应当立即设置发热病区，请具有新冠肺炎诊疗能力的综合性医疗机构派员会诊。同时，精神卫生医疗机构应当立即采取措施，隔离密切接触的医务人员和患者医学观察14天，并彻底消毒病房。

二、做好严重精神障碍患者罹患新冠肺炎后的治疗照护

在新冠肺炎疫情期间，乡镇（街道）精神卫生综合管理小组（以下简称综合管理小组）要安排人员，加强居家严重精神障碍患者的定期访视。对出现发热或者肺炎疑似症状的患者，综合管理小组应当及时协助将其送往就近的发热门诊就诊，如确诊新冠肺炎或者疑似感染入院治疗的，应当告知相关医疗机构患者既往精神疾病史和目前治疗情况。

收治患新冠肺炎的严重精神障碍患者的医疗机构，如不具备精神科疾病诊疗能力，应当及时请精神科医师联络会诊。卫生健康行政部门可指定辖区内精神卫生医疗机构承担对应的联络会诊工作。

三、做好居家严重精神障碍患者的管理治疗和社区照护

综合管理小组要密切关注居家的严重精神障碍患者服药情况，可采取网络视频、电话沟通等方式与患者和其家属进行联络，主动做好社区管护和服务。对在封闭管理区居住的患者，要主动了解患者服药需求，协助采取精神卫生医疗机构邮寄药品，或将药品送至基层医疗卫生机构、综合管理小组代取药、送药上门等方式，帮助患者持续药物治疗。

要切实落实精神科医师与社区精防人员点对点技术指导，对于出现明显精神症状、情绪暴躁、或行为冲动等病情不稳定患者，综合管理小组可联系上级精神卫生医疗机构，通过电话、网络咨询等方式，由精神科医师给予居家患者提供远程医疗服务。对于需要紧急处置的患者，综合管理小组应当协助送至精神卫生医疗机构就医。

国务院应对新型冠状病毒肺炎

疫情联防联控机制综合组

（代章）

2020年2月17日

（231）商务部：新冠肺炎疫情防控期间进一步完善九省联保联供协作机制

湖北、安徽、江西、山东、河南、湖南、广西、云南、重庆商务主管部门：

新冠肺炎疫情发生后，商务部党组高度重视，为保障疫情防控期间重点地区生活必需品市场供应，第一时间部署建立了九省联保联供协作机制，各有关省市团结协作，无私奉献，为确保湖北武汉及周边地市生活必需品供应作出积极贡献。当前，新冠肺炎疫情仍在发展，湖北省封闭区域有所扩大，疫情防控工作到了最吃劲的关键阶段。为树牢底线思维，进一步完善机制运作模式，加强协调配合，保障湖北武汉市场供应稳定，现通知如下：

一、强化组织领导。各地区商务主管部门要严格贯彻落实党中央、国务院部署要求，做好打持久战的准备，从讲政治的高度把保湖北武汉市场供应作为当前工作的重中之重，湖北稳，则全国稳。要进一步健全组织机制，明确目标责任，湖北要成立由一把手直接负责的工作专班，与其他各省积极对接；其他各省要指派主管工作的负责同志亲自抓落实，指定相关业务骨干作为联络员，把工作做实做细。通过机制微信群，各地要及时通报情况，反映问题，通力协作，会商解决。

二、明确任务目标。充分调动各方面积极性，保障湖北武汉生活物资供应基本平稳有序，防止持续性脱销断档和恐慌性抢购等情况发生。重点保障蔬菜、肉类、蛋类、奶类、大米、面粉、食用油以及方便食品8类生活必需品供应。各地要继续发扬“一方有难、八方支援”精神，加强对接和服务保障，确保湖北武汉生活必需品供应充足稳定。

三、确保调运畅通。湖北省商务厅要积极协调省疫情防控指挥部和有关部门，严格落实有关保畅保通通知要求，强化省内物流运输保障，会同省内有关部门制定工作方案，确保省内生活必需品物资调运畅通。其他各地区要加强沟通，通力配合，及时解决出现的问题，协调有关部门共同保障向湖北调运生活物资通道畅通。

四、抓紧组织复工。有步骤地合理安排和动员商贸流通企业复工营业，是保障生活必需品供应的主要措施之一。各地在做好疫情防控要求的前提下，要采取灵活措施统筹安排人员上岗，保障本地区生活必需品物资供应。对于疫情得到有效控制的地区，可以适当扩大复工规模，增加调出能力，做好支援重点地区准备。

五、畅通销售渠道。针对疫情防控期间的特殊形势，各地区，特别是湖北武汉在封闭管理的小区要组织动员企业创新便民保供服务模式，利用网络新技术、新业态、新方法解决生活必需品配送供应问题，创新“无接触”“少接触”购销方式。组织企业和志愿者开展蔬菜套餐配送，安排流动菜车进社区，设立果蔬露天销售点等。有条件地方要稳步恢复农贸市场、马路市场经营，持续畅通终端销售渠道，增加生活必需品供应网点。各地要及时将保障生活必需品市场供应的好做法、好经验等情况总结上报商务部（市场运行司）。

联系人：李宝星、刘虎成

电 话：010-85093851、85093840

传 真：010-85093861

商务部办公厅

2020年2月17日

（232）卫健委：进一步做好医养结合机构新冠肺炎疫情防控工作	肺炎机制综发〔2020〕67号

各省、自治区、直辖市及新疆生产建设兵团应对新型冠状病毒肺炎疫情联防联控机制（领导小组、指挥部）：

新冠肺炎疫情发生以来，各类医养结合机构（指同时具备医疗卫生资质和养老服务能力的医疗卫生机构或养老机构）主动融入防控工作大局，充分发挥积极作用，助力疫情防控阻击战。当前，疫情形势仍然严峻复杂，防控工作进入关键阶段，为做好医养结合机构疫情防控工作，有效预防医养结合机构内的传播风险，提出以下要求：

一、完善防控工作机制

各级卫生健康行政部门要会同民政部门指导医养结合机构做好疫情防控各项工作。各医养结合机构要把老年人生命安全和身体健康放在第一位，切实落实主体责任，完善疫情防控工作机制，根据前期防控情况及时调整防控策略和应急预案，明确工作流程，加强应急值守，按要求及时上报信息，提高防控效果。

二、服从当地联防联控统一指挥

各级卫生健康行政部门要按照疫情防控工作总体部署和要求，周密安排，发挥医养结合机构在疫情防控工作中的应有作用。各类医养结合机构不分举办主体、经营性质、类别规模，要遵从属地管理原则，在当地党委、政府的统一领导下，在卫生健康行政部门、民政部门和疾控等专业机构的指导下，规范、安全开展疫情防控工作。坚持分类管控，快速反应，认真查找薄弱环节，做到“早发现、早报告、早隔离、早治疗”。

三、切实做好疫情防控工作

（一）认真开展宣传教育与防护。医养结合机构要针对老年人和管理人员、医务人员、护理员等工作人员进行分类培训与指导，使老年人和工作人员充分了解新冠肺炎防控相关科学知识，掌握防护要点。规范清洁消毒，分类分级指导相关人员做好科学合理防护，确保机构内全体人员防护到位。

（二）加大机构内疫情防控力度。医养结合机构要严格按照《医疗机构内新型冠状病毒感染预防与控制技术指南》和《养老机构新型冠状病毒感染的肺炎疫情防控指南》，做好新冠肺炎相关预防与控制工作。针对节后返院返岗等情况，加强人员出入管理，因特殊情况确需返回养老区域的老年人、拟新入住养老区域的老年人及返岗的工作人员，都要按照规定严格隔离、密切观察，无异常后方可收住或上岗工作。不具备隔离观察条件的，可在本级联防联控机制统筹安排下进行集中隔离观察。疫情期间，鼓励利用互联网等信息化手段为老年人提供亲情关怀服务。发现机构内发热或疑似新冠肺炎老年人或工作人员，应当立即报告，并按要求做好相应处置工作。不具备救治能力的，应当及时将患者转诊到具备救治能力的医疗机构。

（三）切实消除机构内交叉感染风险隐患。除按照上级和当地联防联控机制统一部署确定为定点救治医疗机构、设置发热门诊、开展排查工作的医疗机构外，各地医养结合机构在疫情期间不得擅自对外开展发热病人的诊疗排查活动，切实消除机构内交叉感染风险隐患。养老机构内部设置的医务室、护理站等医疗机构，应当按照原国家卫生计生委办公厅《关于印发<养老机构医务室基本标准（试行）>和<养老机构护理站基本标准（试行）>的通知》（国卫办医发〔2014〕57号）要求，为入住养老机构的老年患者提供相关医疗卫生服务，不得超出许可服务范围对外服务。

（四）妥善安排对内对外服务。按统一部署作为定点救治医疗机构、设置发热门诊或开展排查工作的医养结合机构，要妥善安排好对内、对外服务。根据防控需要，合理调配人力资源，科学安排工作班次，保障医务人员获得必要休息。负责对外诊断、救治、排查工作的医务人员不再负责对内服务，由其他医务人员提供养老区域内老年人的医疗卫生服务。要严格按照《国家卫生健康委办公厅关于加强重点地区重点医院发热门诊管理及医疗机构内感染防控工作的通知》（国卫办医函〔2020〕102号）要求，加强门急诊预检分诊、发热门诊和普通病区管理，强化感染防控，严格实行分区管理，消除新型冠状病毒在机构内的传播风险。

四、强化责任落实

各级卫生健康行政部门要会同民政部门切实加强对医疗卫生机构和养老机构的行业监管和防控指导，对发现的问题及时督促整改。对在疫情防控工作中瞒报漏报迟报、防控不力、失职渎职等导致疫情扩散严重后果的要严肃追责。医养结合机构要严格执行疫情防控相关部署及工作规范、指南，按照防控预案和应急预案，明确责任分工，压实工作责任，切实保障入住老年人的生命健康安全。

国务院应对新型冠状病毒肺炎

疫情联防联控机制综合组

2020年2月17日

（233）工信部：做好疫情防控期间信息通信行业网络安全保障工作	工信厅网安函〔2020〕22号

各省、自治区、直辖市通信管理局，中国电信集团有限公司、中国移动通信集团有限公司、中国联合网络通信集团有限公司、中国铁塔股份有限公司、中国广播电视网络有限公司，中国信息通信研究院、中国软件评测心、国家工业信息安全发展研究中心、中国工业互联网研究院，国家计算机网络与信息安全管理中心、中国互联网络信息中心，相关域名注册管理和服务机构、互联网企业、移动通信转售企业、网络安全企业：

为深入贯彻落实习近平总书记关于新型冠状病毒肺炎疫情防控工作的重要指示精神，切实做好疫情防控和经济社会运行的网络安全支撑保障工作，确保疫情防控期间网络基础设施安全，防止发生重大网络安全事件，现就有关事项通知如下：

一、全力保障重点地区重点用户网络系统安全

（一）加强重点用户网络安全技术支撑。充分发挥信息通信行业网络、技术和队伍优势，组织力量为党政机关、医疗机构、公共应急、教育教学等疫情联防联控单位以及重点工业互联网企业等用户提供网络安全技术支撑，主动沟通对接，及时为重点用户相关网站和信息系统提供网络链路保障、防拒绝服务攻击和防域名劫持等应急处置支撑。

（二）加强重点地区网络基础设施安全防护。加强涉疫情重点保障地区网络基础设施、重要域名系统等安全防护，利用远程检测等技术手段，强化对重点区域的网络安全风险评估和隐患排查，为疫情防控指挥调度、医疗救助、远程办公和人民群众生产生活提供安全可靠的基础网络服务。

（三）加强涉疫情网络安全威胁监测处置。按照公共互联网网络安全威胁与处置工作机制，对伪装成疫情信息传播网络病毒或相关钓鱼网站、恶意邮件、恶意程序，以及医疗机构、疫情防控物资生产企业所属网络系统存在受控、漏洞等情况加大监测力度，利用网络安全威胁信息共享平台及时通报有关情况，发布风险提示，协助相关单位采取有效处置措施，从源头上降低网络安全风险，维护疫情防控期间的网络秩序和公共利益。

（四）加强疫情期间电话用户入网服务保障。鼓励基础电信企业、移动通信转售企业通过网络渠道为用户办理电话入网实名登记手续，引导广大用户利用电信企业门户网站、手机APP、掌上营业厅等线上方式办理电话入网手续及手机卡。基础电信企业、移动通信转售企业应加大对网络渠道销售手机卡业务及办理流程的宣传，做好相关业务系统和平台的保障，切实为广大用户提供便利。

二、加强信息安全和网络数据保护

（五）加强涉疫情电信网络诈骗防范。充分发挥电信网、互联网诈骗技术防范系统等技术平台作用，切实强化对涉疫情诈骗电话、短信的精准分析和依法快速处置；针对涉防疫医疗物资购买、航班行程退改签等诈骗新手法新套路，及时研判预警，对相关涉诈网站、域名、APP、账户依法快速处置。进一步加强与公安机关工作配合和信息沟通，及时通报涉疫情电信网络诈骗信息线索。充分利用微信、微博、短彩信、APP等平台开展涉疫情电信网络诈骗的宣传引导和风险提示。

（六）加强网上涉疫情相关信息监测处置。严格落实维护网上信息安全主体责任，依托全国互联网信息安全管理系统等技术手段，配合相关部门及时做好网上涉疫情相关风险隐患信息动态监测、违法不实信息应急处置等支撑保障工作，及时发现、积极化解、稳妥处置因疫情引发的网上不安定不稳定因素。

（七）加强个人信息和数据安全保护。落实《中央网络安全和信息化委员会办公室关于做好个人信息保护利用大数据支撑联防联控工作的通知》等要求，在积极利用行业数据、平台等支撑联防联控工作中，切实处理好数据使用与数据保护的关系，进一步强化个人信息收集、使用等各环节的规范管理，将个人信息和数据安全保护各项要求落到实处。

三、进一步强化责任落实和工作协同

（八）加强网络安全责任落实。各地通信管理局要加强统一领导和统筹协调，做到守土有责、守土担责、守土尽责，指导督促地方信息通信行业做好疫情防控网络安全保障工作。各基础电信企业、域名机构、互联网企业、网络安全专业机构、网络安全企业要切实承担起网络安全主体责任，充分发挥网络安全技术支撑保障作用，为打赢疫情防控阻击战作出积极贡献。

（九）加强网络安全信息报送。湖北省通信管理局、基础电信企业集团公司在疫情防控期间每日向部（网络安全管理局）报送疫情防控重点用户网络安全保障情况、涉疫情网络安全监测预警信息、针对疫情防控开展的网络安全防护工作情况和其他重大事项。各单位发生或发现重大网络安全事件要第一时间报部（网络安全管理局）。

特此通知。

工业和信息化部办公厅

2020年2月14日

（234）人社部：在新冠肺炎疫情防控期间免费开放中国职业培训在线等培训平台提供线上培训与教育服务	人社厅函〔2020〕24号

各省、自治区、直辖市及新疆生产建设兵团人力资源社会保障厅（局）：

为进一步贯彻落实《人力资源社会保障部关于进一步做好新型冠状病毒感染的肺炎疫情防控工作的通知》和《国家发展和改革委员会办公厅人力资源社会和保障部办公厅工业和信息化部办公厅全国总工会办公厅关于应对新型冠状病毒感染肺炎疫情支持鼓励劳动者参与线上职业技能培训的通知》精神，推动各地开展职业技能提升行动，我部决定对部属有关单位开发运营的培训在线平台免费向社会开放，支持各地开展职业技能提升线上培训工作。现就有关事项通知如下：

一、在疫情防控期间“中国职业培训在线”（http://px.class.com.cn）、“就业创业和职业培训在线”（http://jc.mohrss.gov.cn）、“新职业在线学习平台”（http://xzy.mohrss.gov.cn）、“中国国家人事人才培训网”（http://www.chinanet.gov.cn）、“人力资源和社会保障部教育培训网”（http://edu.mohrss.gov.cn）等培训平台，将对劳动者个人、职业培训机构、技工院校师生开放，免费提供线上培训资源以及视频课程、电子书、课件、动画等职业培训教学资源。

二、各级人力资源社会保障部门要组织各类职业培训机构用好这些培训平台的学习培训功能，开展线上线下融合教学；组织培训学员利用这些平台提供的教学资源，完成线上培训课程，实现停训不停学；引导各类企业组织在岗和待岗返企的职工加强线上学习。

三、各级人力资源社会保障部门按照《人力资源社会保障部办公厅财政部办公厅关于做好职业技能提升行动专账资金使用管理工作的通知》（人社厅发〔2019〕117号）、《人力资源社会保障部教育部财政部交通运输部国家卫生健康委关于做好疫情防控期间有关就业工作的通知》（人社部明电〔2020〕2号），探索对线上培训给予相应职业培训补贴。同时，要严格监管线上培训过程，提高管理效率与服务水平，杜绝套取培训补贴资金情况的发生。

四、请各级人力资源社会保障厅（局）及时做好本通知的落实工作，工作中遇到的问题请及时向我部反馈。

（一）技术咨询

1.人力资源社会保障部中国人力资源社会保障出版集团“中国职业培训在线”平台

联系人：兰洁 联系电话：010-64962031，13488809182

2.中国就业培训技术指导中心“就业创业和职业培训在线平台”、“新职业在线学习平台”

联系人：姜郁 联系电话：010-84661161，13910770983

3.人力资源社会保障部中国公务员高级培训中心“中国国家人事人才培训网”

联系人：史军燕 联系电话：010-64829607，13910066209

4.人力资源社会保障部教育培训中心“人力资源和社会保障部教育培训网”

联系人：任大为 联系电话：010-64842152，17600306916

（二）政策咨询

中国就业培训技术指导中心

联系人：王鹏 联系电话：010-84661061，13910770983

人力资源社会保障部办公厅

2020年2月12日

（235）卫健委：印发新型冠状病毒肺炎疫情防控期间养老机构老年人就医指南	肺炎机制综发〔2020〕65号

各省、自治区、直辖市及新疆生产建设兵团应对新型冠状病毒肺炎疫情联防联控机制（领导小组、指挥部）：

为做好疫情防控期间养老机构入住老年人就医工作，现将《新型冠状病毒肺炎疫情防控期间养老机构老年人就医指南》印发给你们，请参照执行。

国务院应对新型冠状病毒肺炎疫情联防联控机制综合组

2020年2月15日

（信息公开形式：主动公开）

新型冠状病毒肺炎疫情防控期间养老机构老年人就医指南

鉴于养老机构在疫情防控期间实行封闭式管理，为做好疫情防控期间养老机构入住老年人就医服务工作，制定如下指南：

一、密切关注老年人健康状况。

1.测量体温。每天早晚各为老年人测量1次体温（接触式体温计要做到个人专用，若不能做到个人专用，则需彻底消毒后再用。非接触式体温枪应当按照使用要求定期消毒），随时询问老年人身体情况。

2.慢性疾病老年人管理。提醒慢性病长期服药老年人，要规律服药，不轻易自行换药或停药，有身体不适要及时告知护理人员。有条件的养老机构应当通过检测血压、血糖、呼吸状况、体重等方式，观察慢性病老年人身体状况，注意有无用药不足或过量的表现，以及药物不良反应（特别是体位性低血压、低血糖），预防跌倒。

3.加强新冠肺炎知识宣教。告知老年人，目前针对新冠肺炎，没有确认有效的抗病毒治疗方法，切勿擅自预防性服药。

二、老年人身体出现不适或疾病发作，养老机构应当及时与老年人和家属沟通商量，达成一致后，通过机构内医务人员处置、电话求助医疗机构、请医疗机构医生出诊、拨打120急救电话就医或由家属送医。有条件的或根据入住服务协议，由养老机构工作人员随同协助就医。

1.慢性基础疾病（高血压、糖尿病等）、皮肤病、一般过敏、轻微扭伤擦伤、普通牙科治疗、常规康复等，可采取上门诊视等方式保守治疗，不建议外出就医。老年人常用药物由家属、机构通过委托取药、代购等方式解决。

2.出现咳嗽、咳痰、咽痛、头痛等症状且无加重，没有流行病学史的老年人（14天内没有接触过新冠肺炎确诊患者或疑似患者、没有出入有确诊或疑似患者社区或活动场所），可在机构内按一般感冒治疗，暂不外出就医；有慢性呼吸道疾病（慢性阻塞性肺疾病、支气管哮喘等），病情稳定的老年人可在医生指导下常规用药，若病情加重危及生命则须立即联系医疗机构进行转诊。

3.急性发热，如确无流行病学史，可先在机构内隔离观察，按一般感冒发热进行治疗；如机构内有条件，可完善血常规、肝肾功能、CRP等常规检查（或抽血送附近医疗机构检验）。同时咨询相关医疗机构，有必要再送医。

4.急危重症患者应当及时就医。老年人一旦出现慢性病急性加重或突发急病，养老机构应当立即实施力所能及的抢救措施，同时拨打120急救电话送医，并及时通知老年人家属。

急危重症包括但不限于急性心衰、卒中、心肌梗塞、心跳呼吸骤停、急性损伤创伤、急性中毒、急性胸痛腹痛、消化道出血、肠梗阻、重症肺炎、严重腹泻脱水等疾病，以及神经、心脏、呼吸、消化、泌尿等系统的危急重情况。

三、老年人出现新冠肺炎可疑症状（包括发热、干咳、乏力、鼻塞、流涕、咽痛、腹泻等），不排除有流行病学史的，应当立即执行隔离观察，并及时送医疗机构排查。

四、被确诊为疑似病例或确诊病例的，应当立即送定点医疗机构就诊；养老机构须及时向相关部门报告，在当地卫生健康、民政部门指导下对密切接触者（接触的其他老年人及工作人员等）开展排查，实施14天隔离观察；机构开展全面消杀、规范处置个人物品等相关工作。

五、在医疗机构就诊后返回养老机构的老年人和陪同工作人员，应当隔离观察14天，无异常后方可入住和工作。新冠肺炎老年人治愈后需返回养老机构的，应当隔离观察14天，无异常后方可入住。

（236）国务院扶贫办 财政部：积极应对新冠肺炎疫情影响 加强财政专项扶贫资金项目管理工作 确保全面如期完成脱贫攻坚目标任务 国开办发〔2020〕5号

各省、自治区、直辖市扶贫办（局）、财政厅（局）：

为深入贯彻习近平总书记关于坚决打赢疫情防控阻击战的重要指示精神，认真落实党中央、国务院的统一部署，积极应对新冠肺炎疫情对脱贫攻坚的影响，确保脱贫攻坚全面胜利、圆满收官，现就疫情防控期间加强财政专项扶贫资金项目管理工作通知如下：

一、加快资金分配拨付。2020年中央财政将继续较大幅度增加专项扶贫资金规模，新增资金分配测算时向受疫情影响较重地区适当倾斜。各省（自治区、直辖市，以下简称省）要继续保障财政专项扶贫资金投入，在分配资金时结合实际对受疫情影响较重的市县给予倾斜支持，切实保障好这些地区脱贫攻坚资金需要，尽可能减少疫情对脱贫攻坚工作的影响。省级扶贫、财政部门要督促指导市县将提前下达的2020年中央财政专项扶贫资金和地方各级安排的扶贫资金及时分解落实到项目上，优先支持疫情防控相关的影响脱贫攻坚任务完成的扶贫项目。

二、调整和优化资金使用。各省可结合实际，按照精准施策的要求，研究制定针对受疫情影响较重地区脱贫攻坚的支持政策，在现有资金管理制度框架内，参考以下要求，允许县级因地制宜调整和优化资金使用要求。具体政策措施由省级扶贫、财政部门研究提出报省扶贫开发领导小组审定后实施。

一是重点向产业项目倾斜，结合实际加大对受疫情影响较大的产业扶贫项目生产、储存、运输、销售等环节的支持，解决“卖难”问题。支持贫困户恢复生产，开展生产自救，加大奖补力度。创造条件鼓励贫困劳动力就业创业。对在疫情防控期间，努力克服疫情影响积极带动贫困户发展的扶贫龙头企业和合作社等带贫主体，可给予一次性生产补贴和贷款贴息支持。对在疫情防控中做出突出贡献、社会效益好的涉农企业和其他涉农组织，可优先支持其参与符合条件的脱贫攻坚项目，降低资金使用门槛，其在疫情防控期间的捐赠等投入视作减贫带贫效益。

二是强化就业支持，适当安排财政专项扶贫资金用于组织稳定贫困人口就业岗位。对结合疫情防控需要，新增的保洁环卫、防疫消杀、巡查值守等临时岗位，优先安置贫困劳动力就业。落实好现行公益性岗位相关政策。对疫情防控期间复工复产的扶贫车间和当地企业、参与东西劳务协作的扶贫企业，依据吸纳贫困劳动力规模，按规定落实相关政策，有条件地区可加大支持力度。疫情防控期间外出务工的贫困劳动力按规定给予交通和生活费补助，有条件地区可加大奖补力度。

三是全力保障贫困群众基本生活。对罹患新冠肺炎、集中或居家隔离、无法外出务工、无法开展基本生产、收入受到重大影响等生活陷入困境的建档立卡贫困群众和因疫致贫返贫农民群众，应按现有支持渠道及时落实好针对性帮扶措施，确保基本生活不受影响。

三、完善县级脱贫攻坚项目库建设。针对疫情影响，做好项目库动态调整，及时优化年度项目实施计划，对因疫情致贫急需实施的项目，对符合疫情防控需要和脱贫攻坚政策的项目，对有利于增加贫困户收入的项目，优先入库，优先安排资金支持。根据防控工作需要，适当优化项目入库、公告公示流程或具体组织实施方式。防控期间加强线上工作，做好项目库清理规范和信息完善补录等工作，加强对市县项目库的分析应用，提高项目库建设质量。

四、保障年度扶贫项目实施。抗击疫情期间，具备开工条件的扶贫项目要及时开工建设。暂时不能开工的，要积极采取措施，运用现代化技术手段开展扶贫项目设计、评审等项目实施前期各项准备工作。属于政府采购的项目，进一步优化采购流程，加快采购进度，确保项目早开工实施见效。

五、强化资金监督管理。认真对照财政专项扶贫资金相关管理制度从严管理资金，确保资金精准使用，体现脱贫成效。省级要指导市县加强资金使用管理，明确流程、标准和监督等要求，突出绩效导向，实行全过程绩效管理。落实资金项目公开公示制度要求，主动接受各方面监督。

各省要充分认识当前疫情防控形势下做好脱贫攻坚工作的紧迫性，着力抓好工作衔接和组织协调，切实把各项工作抓实抓细，进一步提高财政专项扶贫资金安排使用和项目管理水平，助力受疫情影响贫困群众实现稳定脱贫，确保如期打赢脱贫攻坚战。

国务院扶贫办 财政部

2020年2月17日

（237）教育部 工信部：发布中小学延期开学期间“停课不停学”有关工作安排	教基厅函〔2020〕3号

各省、自治区、直辖市教育厅（教委）、通信管理局，新疆生产建设兵团教育局：

为认真贯彻落实习近平总书记重要指示精神，按照党中央、国务院关于防控新冠肺炎疫情的决策部署，现就做好中小学延期开学期间“停课不停学”工作通知如下。

一、总体要求

“停课不停学”要坚持把做好疫情防控工作放在首位，维护广大师生健康安全，坚决防止疫情向校园蔓延。要坚持省级统一部署与各地各校因地制宜实施相结合，为支持帮助学生学习，教育部整合国家、有关省市和学校优质教学资源，在延期开学期间开通国家中小学网络云平台和电视空中课堂，免费提供有关学习资源，供各地自主选择使用，各地要结合本地学习资源，统筹安排，针对不同情况，实事求是，避免“一刀切”，特别要防止各地各校不顾条件都组织教师录课，增加教师不必要的负担。要坚持国家课程学习与疫情防控知识学习相结合，注重加强爱国主义教育、生命教育和心理健康教育，鼓励学生锻炼身体、开展课外阅读。要坚持学校教师线上指导帮助与学生居家自主学习相结合，学校教师要指导帮助学生选择适宜的学习资源，限时限量合理安排学习，促进学生全面发展、身心健康。

二、主要资源

1.开通国家中小学网络云平台。在统筹整合有关资源基础上，依托中央电化教育馆，于2月17日开通国家中小学网络云平台（网址：ykt.eduyun.cn，电话：400-8980-910），教学资源包括小学一年级至普通高中三年级（使用说明及课程安排等可在国家中小学网络云课堂主页上查看），供各地选择使用。

2.开通专用电视频道。为拓宽学习资源渠道，2月17日起，将有关课程和资源通过中国教育电视台4频道陆续推送（播出时间表由中国教育电视台提前公告），覆盖全国偏远农村有线电视未通达地区，供各地选择收看。因电视频道播出时间有限，播出内容以小学阶段为主，同时兼顾初中和高中，特别是参加中考、高考学生的需要。

3.开放部分省市和中小学校网络学习平台。为丰富学习资源，组织部分省级教育部门及中小学免费向全国开放网络学习平台或网校（相关链接见国家中小学网络云课堂主页）。

4.其他资源。免费提供有关教材电子版（下载地址见教育部门户网站和国家中小学网络云课堂主页）；人民教育出版社向社会免费开放“人教点读”数字教学资源库（下载方式见国家中小学网络云课堂主页）。

三、组织实施

5.加强组织协调。教育部、工业和信息化部建立部际协调工作机制，强化组织实施，统筹协调基础电信企业、相关接入服务企业做好平台运行保障。各地通信管理局要会同教育主管部门，建立相应协调机制，指导属地基础电信企业、接入服务企业做好相关工作。各地教育部门要切实加强“停课不停学”工作的组织领导，切实负起责任，认真制定实施方案，注重实施效果，防止形式主义，防止由校外培训机构代替学校实施系统化学科教学；要加强宣传解释，积极推广好经验好做法，营造良好社会氛围。

6.强化居家学习指导。各地各校要高度重视、精心组织，指导学生以多种方式居家学习。加强学生网上学习资源选择的指导，鼓励有条件的地方和学校利用好本地本校优质资源；要针对线上学习特点和学科特点，认真研究明确适合线上学习的课程。学校要防止照搬套用正常课堂教学方式、时长和教学安排，应依据居家网上学习的特点，采取多种途径加强学习指导，帮助学生科学制定居家学习计划，注重培养学生自主学习能力，特别要加强对防疫阻击战一线人员子女和农村留守儿童的学习指导和关爱。对上网学习的要求，可借鉴一些地方好的做法，对小学低年级上网学习不作统一硬性要求，由家长和学生自愿选择，对其他学段学生作出限时限量的具体规定。要注重疫情防护知识普及，加强生命教育、公共安全教育和心理健康教育；认真学习防疫阻击战中涌现的先进事迹，弘扬社会美德，增强学生爱党爱国爱人民爱社会主义的思想情感。

7.做好开学后教学与居家学习的衔接。要防止以居家学习完全代替学校课堂教学，正式开学恢复课堂教学后，各地各校要精准分析学情，认真对学生居家学习情况进行摸底，对学习质量进行诊断评估，有针对性地制订教学计划。对已经居家在网上学习过的课程，有重点地对已学内容进行讲解和复习，加大对学习困难学生的帮扶力度，确保每名学生较好地掌握已学知识内容，然后再进行新的课程教学；对小学低年级没有参加网上课程学习的，要从头开始实施教学。

8.确保不增加学生负担。各地教育部门、学校和教师要防止多重选择网上学习资源，依据不同年龄段学生的特点，指导学生合理确定网上学习时间，统筹做好各科学习资源用量用时，避免学生网上学习时间过长，坚决防止超前过快学习。不得强行要求学生每天上网“打卡”、上传学习视频等，防止增加学生不必要的负担。要指导学生合理安排作息时间，通过增大休息间隔、做视力保健操、强化体育锻炼等方式，保护学生视力，增强学生体魄，保障学生身心健康。学校、教师要指导学生根据自身现有条件，选择网络学习或电视空中课堂学习，防止再购置新的设备而增加家庭经济负担。

9.保障网络稳定顺畅运行。国家中小学网络云平台服务开通前，各地要根据当地网络情况、服务能力、学生分布等提前做好分析预判，预先排除风险，做好充分准备；服务开通后，各地要做好网络运行分析检测，畅通反馈渠道，确保发现问题及时解决。教育部门要指导各地各校“错峰”登录国家中小学网络云平台；原定开学时间晚于2月17日的，要引导在原定正常开学时间再登录。

教育部办公厅 工业和信息化部办公厅

2020年2月12日

（238）民政部 卫健委：深入学习贯彻习近平总书记重要指示精神进一步做好城乡社区疫情防控工作	民发〔2020〕13号

各省、自治区、直辖市民政厅（局）、卫生健康委，各计划单列市民政局、卫生健康委，新疆生产建设兵团民政局、卫生健康委：

社区防控是疫情防控的关键环节，是打赢疫情防控的人民战争、总体战、阻击战的重要基础。新冠肺炎疫情发生以来，党中央高度重视，始终把人民群众生命安全和身体健康放在第一位，就做好社区防控工作提出一系列明确要求。习近平总书记2月10日在北京市调研疫情防控工作时，对做好社区防控工作作出重要指示，并亲切慰问了参与社区防控工作人员。各地民政、卫生健康部门要深入学习贯彻习近平总书记重要指示精神和党中央、国务院决策部署，切实做好社区防控各项工作，使所有社区都成为疫情防控的坚强堡垒。现就有关事项通知如下：

一、进一步提高政治站位

习近平总书记关于社区防控工作的重要指示，强调社区是疫情联防联控的第一线，也是外防输入、内防扩散最有效的防线，把社区这道防线守住，就能有效切断疫情蔓延扩散的渠道；强调充分发挥社区在疫情防控中的阻击作用，把防控力量向社区下沉，加强社区各项防控措施的落实，使所有社区成为疫情防控的坚强堡垒；强调以疫情防控工作成效来检验和拓展“不忘初心、牢记使命”主题教育成果，发挥基层党组织政治引领作用和党员先锋模范作用，把社区居民发动起来，构筑起疫情防控的人民防线等，明确了社区防控工作的基础地位、方法路径和政治保证，体现了对广大社区工作者的亲切关怀，为做好社区防控工作提供了根本遵循。各地民政、卫生健康部门要认真学习贯彻习近平总书记重要指示精神，严格落实早发现、早报告、早隔离、早治疗措施，扎实做好城乡社区疫情防控工作，巩固好在社区防控中来之不易的工作成效，筑牢疫情防控的人民防线。

二、进一步落实防控措施

当前，疫情防控工作到了最吃劲的关键阶段，要毫不放松做好疫情防控重点工作。各地民政、卫生健康部门要认真贯彻落实国务院联防联控机制《关于加强新型冠状病毒感染的肺炎疫情社区防控工作的通知》和民政部、国家卫生健康委《关于进一步动员城乡社区组织开展新型冠状病毒感染的肺炎疫情防控工作的紧急通知》精神，在当地党委和政府统一领导下，指导强化社区网格化防控体系，发挥城乡社区组织、社区工作者凝聚作用和社区、社会组织、社会工作、社区志愿者联动机制协同作用，会同基层医疗卫生机构、基层医务人员，动员广大社区居民参与，切实做好疫情监测、信息报送、宣传教育、环境整治、困难帮扶等各项工作，切实把社区防控的“网底”兜住、兜实。

各地要根据当前疫情防控和复工复产要求，科学防控、精准施策，分区分级落实相应疫情防控措施。要广泛动员各方面力量参与协同防控，加强对辖区企业、学校等人员密集单位的指导，强化社区防控网格化管理，坚决切断输入型传染源。各地特别是人口流入大省、大市要重点做好针对返程人员的疫情监测工作，实现入户排查和重点群体监控“两个全覆盖”；重点做好封闭式管理社区的人员疏导、医疗救治、生活保障、心理慰藉，引导救护、救助、消防等应急车辆按需进出，将各项防控措施抓早抓小、落细落实。

要统筹防控疫情和服务群众工作，避免在社区防控工作中“简单化”、“一刀切”，在加强疫情防控的同时尽可能方便社区居民生产生活。加大对社区各类民政服务对象的照护力度，重点做好对分散供养特困人员，生活不能自理且子女亲属已接受隔离治疗，或者子女亲属参加疫情防控一线工作无暇照顾的空巢（独居）老年人的生活照料，对因新冠肺炎疫情影响造成监护缺失的儿童的救助保护，对生活无着流浪人员的劝导救助。

要推动社区防控工作依法有序开展，组织开展《中华人民共和国传染病防治法》和卫生防疫基本知识宣传教育，统筹社区工作者、社区民警和基层法律服务工作者等力量，针对疫情防控中的突出问题和群众重点关切，采取群众喜闻乐见的方式，对相关法律法规进行同步宣传解读，引导广大社区居民增强法治意识和防护意识，依法支持和配合疫情防控工作。充分发挥城乡社区人民调解组织作用，及时有效化解疫情防控期间的矛盾纠纷。

三、进一步关心基层工作人员

各地民政、卫生健康部门要积极向各地应对疫情工作领导小组和联防联控机制汇报，完善社区防控工作统筹协调机制，规范社区防控工作数据收集，坚决杜绝形式主义，最大限度压减不必要的材料报表，让社区工作者、基层医务人员将更多精力投入到做好社区防控工作上。协调改善社区工作者防护条件，强化社区防控工作物资保障，重点配齐口罩、防护服、消毒水、非接触式体温计等各类卫生防护器材和防控器具。组织开展面向社区工作者的医疗卫生知识、疫情防控知识简易培训，提高其做好社区防控工作的能力。支持开发适应社区防控工作要求的技术应用，强化人员排查、居家观察、出入管理、志愿服务、宣传教育等防控环节的信息化支撑，减轻社区工作者工作压力。

各地要推动各类防控力量下沉，防控任务重、社区人手不足的地方，要组织机关事业单位工作人员到社区帮助工作；优化社区防控人力配置，合理安排参与社区防控工作人员轮休。争取各地党委和政府重视支持，为参与社区防控工作的专职城乡社区工作者适当发放临时性工作补助，并为参与疫情防控工作的社区志愿者适当发放补贴。有条件的地方，可为参与社区防控工作的社区工作者、基层医务人员办理专属保险。帮助解决社区工作者、基层医务人员家庭实际困难，探索开展社区工作者心理健康评估。加大对表现突出的城乡社区组织、基层医疗卫生机构和社区工作者、基层医务人员表扬力度。做好因社区防控工作牺牲的社区工作者、基层医务人员家属慰问关怀工作，相关情况要第一时间报当地党委、政府，并报上级民政、卫生健康部门。

四、进一步加强组织领导

各地民政、卫生健康部门要充分认识做好社区防控工作的重要意义，完善系统内部疫情防控工作领导协调机制，配齐配强基层政权建设和社区治理业务工作力量，加强对社区防控工作的组织领导和工作指导。承担社区防控工作牵头职责的，要加强与各成员单位的沟通协调，科学研判社区防控工作形势任务，及时提出加强社区防控工作的意见建议；未承担社区防控工作牵头职责的，要主动与牵头部门协调配合，积极反映、解决城乡社区组织和社区工作者参与疫情防控工作面临的困难和问题。要在社区防控工作中查找社区治理和服务工作中存在的不足和短板并及时解决，解决不了的要及时向当地党委和政府报告并争取支持。在总结社区防控工作经验基础上，探索建立社区应急管理体系。要把社区防控工作成效作为检验本系统“不忘初心、牢记使命”主题教育成效和治理能力的重要依据，切实发挥基层党组织战斗堡垒作用和共产党员先锋模范作用，鼓励民政、卫生健康系统共产党员到社区防控工作一线支持帮助工作，为发挥社区在疫情防控中的阻击作用贡献智慧和力量。

五、进一步强化宣传引导

各地民政、卫生健康部门要运用党委和政府新闻发布平台，充分解读加强社区防控工作的法律法规和政策措施，主动回应社区居民普遍关心的共性问题，引导社区居民客观理性认识疫情，避免并化解恐慌情绪。要依托各类媒体、网站，大力宣传社区防控工作取得的成效经验，宣传优秀城乡社区组织、基层医疗卫生机构和社区工作者、基层医务人员、社会工作者、志愿者先进事迹，为做好社区防控工作营造良好社会氛围。

各地民政、卫生健康部门指导做好社区防控工作有关情况要及时报民政部、国家卫生健康委。

民政部 国家卫生健康委

2020年2月16日

（239）财政部：做好新冠肺炎疫情防控资产保障工作	财资〔2020〕4号

党中央有关部门，国务院各部委、各直属机构，全国人大常委会办公厅，全国政协办公厅，最高人民法院，最高人民检察院，各民主党派中央，有关人民团体，各省、自治区、直辖市、计划单列市财政厅（局），新疆生产建设兵团财政局，有关中央管理企业：

　　为深入贯彻落实习近平总书记关于坚决打赢新冠肺炎疫情防控阻击战重要指示精神和党中央、国务院决策部署，积极应对疫情，保障人民生命健康，现就行政事业单位疫情防控资产管理工作通知如下：

　　一、简化配置程序。疫情防控期间，行政事业单位根据疫情防控工作需要紧急配置救治器械、医药防护用品等疫情防控资产，各部门、各地方本着急事急办、特事特办的原则，可简化资产配置审批程序，在履行必要的内部程序后，快速高效配置。

　　二、统筹调配使用。疫情防控期间，行政事业单位根据工作需要统筹调配、捐赠疫情防控资产，按照审批权限需报财政部门审批的，可按主管部门要求履行相关程序后实施，待疫情结束后统一报财政部门备案。

　　三、完善内部管理。各部门、各地方要指导行政事业单位建立疫情防控资产登记台账，完善疫情防控资产的验收、保管、领用、报损制度，明确责任人，确保资产安全完整、流向可查。加强资产管理部门与采购管理、预算管理、财务管理等部门的协调对接，按规定入账。

　　四、规范资产处置。疫情结束后，行政事业单位应回收可重复使用的疫情防控资产，由财政部门和主管部门按权限统筹调剂使用、公开拍卖或捐赠；报废、报损的疫情防控资产，按照行政事业单位资产处置审批权限审批或备案。已建立“公物仓”等国有资产集中处置管理平台的地方，鼓励对行政事业单位闲置的疫情防控资产进行集中统一处置。

　　五、加强监督检查。各级财政部门会同主管部门加强对行政事业单位疫情防控资产管理的监督检查，严禁“搭便车”购置与疫情防控工作无关的资产，避免国有资产损失和浪费。对于违规违纪违法操作，造成不良影响和国有资产流失的，由相关部门按照有关规定追究责任。

　　财 政 部

　　2020年2月14日

（240）教育部：在疫情防控期间有针对性地做好教师工作	教师厅函〔2020〕2号

各省、自治区、直辖市教育厅（教委），新疆生产建设兵团教育局，部属各高等学校、部省合建各高等学校：

　　为贯彻落实习近平总书记关于坚决打赢疫情防控阻击战的重要指示精神和党中央、国务院决策部署，根据《教育部应对疫情工作领导小组工作方案（试行）》要求，现就指导教师积极有效开展应对工作通知如下。

　　一、加强对教师群体的疫情防控工作。一线教师与学生群体接触密切，做好自身防护才能更好地维护学生健康。当前，疫情防控正处在关键时期，各地教育部门和学校要严防死守，始终把师生生命安全和身体健康放在第一位，未经学校批准学生一律不准返校，校外无关人员一律不准进校门，师生进入校门一律核验身份和检测体温，对发烧咳嗽者一律实行医学隔离观察，不服从管理者一律严肃处理。疫情防控期间，不得组织教师参加线下集中面授培训、集中职称评审、大型会议等集聚性活动，要按照当地防控要求从严从紧做好学校疫情防控需要返校教师的妥善安排，确保教师立足教育教学岗位助力打赢疫情防控阻击战。

　　二、做好“停课不停教、不停学”组织部署工作。各地教育部门和学校要因地制宜组织教师开展在线教学，明确授课内容、课程安排、授课组织形式，教学过程中要注意青少年身心健康，把握好教学内容的适量和教学时长的适当。不得违反相关规定安排教师超前超限超纲在线教学，不得要求教师在正常休息时间进行授课。要结合当地线上教学平台和各校实际，整合优质教育资源，确保各级各类教师有序开展教育教学。充分利用国家网络云课堂、国家教育资源公共服务平台、国家开放大学数字化学习资源中心、中国教育干部网络学院、中国教育电视台频道节目、“人教点读”APP、人教网、高等教育出版社爱课程等免费平台，指导学生在线学习或收听收看。教师参加在线教学或网络远程培训，按照考核认定的学时数计入教师培训学时（学分）。教师承担在线教育教学、在线辅导答疑、作业批改等计入工作量，纳入绩效管理。

　　三、做好教师信息技术能力提升和师训资源开放共享工作。依托国培计划、省培计划等培训项目，适时组织开展教师远程教学及信息技术能力在线专题培训。充分发挥部属高校特别是部属师范大学，教育部幼儿园园长、小学校长、中学校长培训中心，国家级培训项目管理办公室和名师名校长领航工程项目培养基地等机构的师训功能，向社会免费开设咨询和线上指导。各地教育部门要指导本地师训、教研、信息化等部门以及地方高校特别是地方师范院校，加大对教师信息化能力的培训力度，落实相关配套措施，一校一策，为教师科学高效开展线上教育教学提供支撑和保障。

　　四、做好心理疏导和教育引导工作。各地教育部门和学校要指导教师做好自我调适，理性应对疫情，全面科学掌握疫情防控要领，多渠道向学生宣传防控知识，对学生深入进行健康理念和自我保护教育。引导教师加强家校沟通，推进将生命教育、感恩教育、责任教育融入家庭教育。充分发挥班主任、思政课教师、心理教师以及学科教师的育人作用，做好对学生的心理疏导，促进身心健康发展，引导学生树立科学观念，不信谣、不传谣。

　　五、加大对在防疫一线作出突出贡献教师的激励表彰力度。支持高校改革创新医药卫生等相关学科领域教师科研评价办法，建立重实绩、重贡献的激励机制，鼓励专家团队和领军人才集智攻关，尽快取得实际应用成果，为战胜疫情提供科技支撑。对作出突出贡献的优秀教师特别是高校医学院和附属医院中的优秀教师典型，各级教育部门和学校要给予大力宣传和表彰奖励，在职称评审、评优评先、绩效分配等方面予以政策倾斜，鼓励社会团体、企事业单位和民间组织设立关爱基金或出资奖励。

　　六、做好对防疫一线医护人员子女的关怀工作。各地教育部门和中小学、幼儿园要及时掌握了解防疫一线医护人员子女的学习生活困难，组织本地本校教师有针对性地进行关心和辅导。注重发挥模范教师、优秀教师、教学名师以及名师名校长领航班学员的示范带头作用。鼓励教师志愿服务组织因地制宜对防疫一线人员特别是一线医护人员子女进行看护和教育，帮助他们在疫情防控期间学有所获、健康成长，为一线人员解除后顾之忧。

　　七、做好对湖北省等疫情严重地区教师的支援帮扶工作。国培计划、特岗计划、教师表彰奖励名额等向疫情严重省份倾斜支持。组织国培远程培训机构开放教师培训网络资源。发挥国培承担机构院校、名师名校长等作用，向疫情严重地区的学校主动输送优质线上课程资源。协同有关高校、教师发展机构等全力开放资源，为当地教师提供应对疫情急需的信息素养提升、心理疏导、卫生防疫等支援服务。

　　各地各校在抗击疫情过程中涌现出的优秀教师典型和感人事迹，请及时报送教育部教师工作司。

　　联系人：刘扬 高顺利

　　电 话：010-66096771 66020522（传真）

　　邮 箱：gaoshunli@moe.edu.cn

教育部应对新型冠状病毒肺炎疫情工作

领导小组办公室（代章）

2020年2月10日

（241）交通运输部：新冠肺炎疫情防控期间免收收费公路车辆通行费

各省、自治区、直辖市人民政府，国家发展改革委、财政部、中国人民银行、国资委、银保监会、证监会：

　　经国务院同意，决定在新冠肺炎疫情防控期间，免收全国收费公路车辆通行费。现将有关事宜通知如下：

　　一、实施范围

　　（一）免收通行费的时间范围从2020年2月17日0时起，至疫情防控工作结束。具体截止时间另行通知(开放式收费高速公路和普通公路以车辆通过收费站收费车道的时间为准，联网收费高速公路以车辆驶离出口收费车道的时间为准)。

　　（二）免收通行费的车辆范围为依法通行收费公路的所有车辆。

　　（三）免收通行费的收费公路范围为符合《中华人民共和国公路法》和《收费公路管理条例》规定，经依法批准设置的收费公路(含收费桥梁和隧道)。

　　二、保障措施

　　（一）加强组织领导。新冠肺炎疫情防控期间免收收费公路车辆通行费的具体工作，由各省(区、市)人民政府负责统一组织实施。省级交通运输主管部门要在省级人民政府统一领导下，会同有关部门制定方案，落实责任，细化措施，加强协作，共同做好实施工作。

　　（二）加强公路保通保畅。结合疫情防控工作需要以及交通量等相关状况，配合有关部门，科学实施防疫检测和交通管理，强化保通保畅，保护人民群众生命安全和身体健康，保障疫情防控和生产生活物资运输，支持企业复工复产，为稳定经济社会大局提供有力支撑。

　　（三）相关配套保障政策将另行出台。

　　交通运输部

　　2020年2月15日

（242）进一步做好疫情防控金融服务	银保监办发〔2020〕15号

各银保监局，各政策性银行、大型银行、股份制银行，外资银行，金融资产管理公司，各保险集团（控股）公司、保险公司、保险资产管理公司，各会管单位：

为深入贯彻落实党中央、国务院决策部署，推动银行业保险业做好新冠肺炎疫情防控金融服务，助力打赢疫情防控的人民战争、总体战、阻击战，更好地支持实体经济发展，现就近期重点工作事项通知如下：

一、做好金融服务，支持企业复工复产

（一）全力支持疫情防控企业扩大产能。紧紧围绕疫情防控需求，全力做好治疗药物、疫苗研发等卫生医疗重点领域，以及重要物资生产、运输物流等相关企业的融资支持。用足用好中央政策，专设机制、充分授权、主动对接，降低融资成本，提供优惠利率和优质金融服务，支持企业恢复产能和扩大生产。鼓励保险机构结合自身情况，为身处疫情防控一线的工作人员提供意外、健康、养老、医疗等优惠保险服务。

（二）全面服务受疫情影响企业复工复产。各银行机构要提早谋划、及时掌握企业信息，优化信贷流程，合理延长贷款期限，有效减费降息，支持受影响企业有序高效恢复生产经营。鼓励保险机构通过减费让利、适度延后保费缴纳时间等方式，支持受疫情影响较重企业渡过暂时难关。鼓励银行保险机构积极拓展服务领域，在支付结算、融资规划、产销支持等更多领域，发挥机构自身优势，提供特色产品、专业咨询、财务管理、信息科技支持等增值服务。围绕国家重大战略，精准支持对宏观经济和区域发展具有重要带动作用的项目工程。加大制造业贷款投放力度，加强供应链金融服务。

（三）积极帮扶遇困小微企业、个体工商户。做好辖内小微企业和个体工商户的服务对接和需求调查，对受疫情影响暂时遇到困难、仍有良好发展前景的小微客户，积极通过调整还款付息安排、适度降低贷款利率、完善展期续贷衔接等措施进行纾困帮扶。加大对普惠金融领域的内部资源倾斜，提高小微企业“首贷率”和信用贷款占比，进一步降低小微企业综合融资成本。加大企业财产保险、安全生产责任保险、出口信用保险等业务拓展力度，为小微企业生产经营提供更多保障。

（四）加大春耕春种金融支持。针对农村地区疫情防控特点，积极通过线上线下多种方式有效满足农村地区基础金融服务需求。加大涉农贷款投放力度，全力保障农副产品生产和春耕备耕农资供应信贷资金需求。鼓励地方银行机构建立农产品应急生产资金需求快速响应机制，支持疫情期间农产品保供稳价。支持保险机构稳步拓展农业保险品种，扩大农业保险覆盖面，稳定农业种养殖户和农民生产经营预期。

二、加强科技应用，创新金融服务方式

（五）提高线上金融服务效率。各银行保险机构要积极推广线上业务，强化网络银行、手机银行、小程序等电子渠道服务管理和保障，优化丰富“非接触式服务”渠道，提供安全便捷的“在家”金融服务。在有效防控风险的前提下，探索运用视频连线、远程认证等科技手段，探索发展非现场核查、核保、核签等方式，切实做到应贷尽贷快贷、应赔尽赔快赔。

（六）加强线下配套服务和宣传引导。对于不习惯使用线上业务客户，要针对性做好金融服务，提供定期存款自动到期续存、错峰办理养老金支取等便民服务。完善自助机具服务功能，放大字体、简化页面、加强引导，减少非必要的柜面业务办理。对客户已熟悉使用的各类自助机具等线下服务方式，银行机构要合理保留，已经撤销的，要有效恢复或提供替代服务方式。

三、增强使命意识，切实履行社会责任

（七）加强社会责任承担。主动承担环境、社会和公司治理责任，倡导绿色、环保、健康投融资理念，更积极有效地将企业和个人保护生态环境、履行社会责任、践行社会公德、诚信行为表现等情况纳入信贷审批、产品定价和风险管理流程。积极投身公益事业，服务公众、回报社会，根据自身实际情况和能力，组织做好向疫情严重地区的捐赠支持。利用海外分支机构资源优势，协助国内有关机构采购紧缺医疗防疫物资，提高跨境支付结算效率。

四、完善制度机制，落实疫情防控要求

（八）严格做好金融服务卫生防疫。各银行保险机构要全力配合地方政府做好疫情防控，出现问题的要严肃追责问责。全面强化办公区域和营业场所的卫生防疫，切实做好安全防护、设施清洁消毒、防护物品配备、提示公告发布等工作。以做好防疫管理为前提，合理安排营业网点及时间，做好柜面人员的统筹调配，保障客户服务安全有序开展。强化营业网点疫情防控要求，不得开展聚集性营销活动。

（九）加强员工防疫关心关爱。切实关爱员工健康，关心员工家庭，加强员工疫情感染排查，动态掌握本单位人员防疫隔离情况，有效防范管控疫情风险。对于因疫情遇到困难的员工家庭，要积极通过工会支持、职工互助等方式，及时给予帮扶。对于直系亲属参加一线防疫工作的员工，要视情在工作安排和待遇上给予倾斜支持。

五、改进工作作风，提升金融监管服务质效

（十）强化政治担当。银保监系统要坚决服从党中央统一指挥、统一协调、统一调度，做到疫情防控全国一盘棋。党员干部尤其是领导干部要率先带头，身体力行，及时了解疫情防控变化，勇于承担社会责任。对不服从统一指挥和调度、本位主义严重的，以及不敢担当、作风漂浮、推诿扯皮的，要依纪依法严肃惩处。

（十一）力戒形式主义和官僚主义。在疫情防控期间，除各级疫情防控指挥部统一部署外，不提不符合实际的要求，不搞标新立异的载体创新，不开不必要的动员会、座谈会、汇报会等，让基层把更多精力投入到疫情防控第一线。上级部门要掌握基层部门实际情况，主动为基层部门服务。

（十二）加强业务学习。充分利用防疫期间专门工作机制，抓好重点工作，做好学习规划。加强业务学习和知识储备，提高专业素养，做到学以致用，增强解决突发复杂问题的专业本领和能力。

2020年2月14日

（243）农业农村部等3部门：解决当前实际困难加快养殖业复工复产

各省、自治区、直辖市农业农村（农牧）、畜牧兽医、海洋与渔业厅（局、委），发展改革委，交通运输厅（局、委），新疆生产建设兵团农业农村局、发展改革委、交通运输局：

　　肉蛋奶和水产品是居民生活必需品。新冠肺炎疫情发生以来，养殖业发展遇到新的困难，饲料供不上、物资运不进、产品销不出、用工回不来等问题普遍存在，如不迅速妥善解决，不仅会影响产业稳定发展，而且很快会影响城乡居民基本生活和新冠肺炎疫情防控大局。各地各部门要坚决贯彻习近平总书记重要指示批示精神和党中央、国务院决策部署，针对养殖业面临的特殊困难，采取特别的支持帮扶措施解决问题堵点，推动养殖业立即复工复产。现将有关事项通知如下。

　　一、加快饲料企业和畜禽屠宰加工企业复工复产

　　除武汉等疫情严重的城市外，各地都要允许饲料和畜禽屠宰加工企业复工，不得设置审查核准等限制条件。优先保障相关企业的人员防护物资需求和水、电、气等供应。优先安排相关企业用工返程返岗，地方政府统一组织的返岗包车纳入疫情防控应急运输绿色通道政策范围，免收高速公路通行费。建立饲料原料供需调度机制，推动当地粮库与饲料企业紧密对接，增加政策性玉米投放供应，适时调配投放库存稻谷、小麦等替代原料。加快豆粕等上游原料生产企业开工，充分调动相关行业协会力量，促进豆粕等饲料原料产销对接。各地对湖北等疫情严重地区提出的饲料原料采购需求，要采取特别保障措施实施“点对点”支持。

　　二、确保物资和产品运输畅通

　　将饲料产品及玉米、豆粕等饲料原料、种畜禽、仔猪禽苗、水产苗种、出栏畜禽、生鲜乳、乳制品、鲜活水产品、冷鲜猪肉、转场蜜蜂等纳入生活必需品应急运输保障范围，切实落实绿色通道政策，除必要的对司机快速体温检测外，确保“三不一优先”，便捷快速通行。尽快打通养殖业所需物资下乡和产品进城进厂的运输通道，不得拦截仔畜、雏禽及种畜禽、饲料原料及产品、畜禽水产品运输车辆。对一些县、乡、村封村封路、一概劝返等不恰当做法，要坚决予以纠正。各地有关部门要公布联系电话，安排专人接听群众来电，及时解决所反映的具体问题。

　　三、千方百计推进当前养殖业解困

　　家禽业要突出解决好养殖缺饲料、禽苗运不出和禽肉禽蛋卖难问题，帮助重点养殖场（户）和饲料企业、屠宰企业建立对接机制，协调金融机构解决家禽养殖企业流动资金贷款，落实活禽交易市场分类管理、不搞一刀切，确保饲料不断供、产品不滞压、资金不断流，必要时地方政府要采取禽肉、禽蛋产品临时收储措施。生猪要合力抓好生产恢复发展，“点对点”协调解决好建筑用工问题，加大建筑材料供应和保障力度，确保各地新建和改扩建生猪养殖项目尽快开工复工、投产达产。奶业要防止出现生鲜乳限收拒收特别是倒奶现象，督促收购生鲜乳的乳品加工企业用好用足产能，严格履行生鲜乳购销合同，敞开收购生鲜乳，支持采取生鲜乳喷粉等应急措施。水产养殖要解决好对虾、罗非鱼、小龙虾等产品压塘严重问题，协调主产区和大中城市构建销售流通对接关系，充分发挥电商等渠道优势，帮助重点水产加工企业尽快复工复产。

　　四、促进畜禽水产品产销衔接

　　加强畜禽水产品产销调度分析，充分发挥行业协会等作用，建立产销衔接平台，收集发布产销信息，引导及时精准对接，解决产品“卖不出”和“买不到”的问题。重点解决贫困地区特色养殖产品销路问题，组织开展多种形式的产销对接活动，确保贫困群众的养殖产品有销路、卖得出。加强对家禽产品消费的科学宣传和正确引导，促进市场消费恢复正常。

　　五、把复工复产支持政策落实到企业

　　各地要组织饲料、养殖、屠宰加工和水产品加工等骨干企业按要求积极申报，纳入疫情防控重点保障企业名单，组织开展银企对接，将国家专项再贷款和贴息政策落实到位，争取享受税收减免等政策。疫情防控期间，对相关企业用电、用水、用气实施阶段性缓缴费用。对因疫情影响导致生产经营困难的企业给予稳岗补贴，实施“五险一金”费用缓缴和阶段性减免。

各地要高度重视养殖业当前面临的困难，接到本通知后立即落实政策措施。相关部门要各司其职，强化协作，形成工作合力。省级农业农村部门要会同有关部门建立重点企业特别是龙头企业联系平台，县级农业农村部门要对辖区内所有规模生猪和家禽养殖场、饲料、屠宰加工和水产品加工企业指定联络员，及时掌握生产经营情况，协调解决面临的困难，推动本通知各项支持政策落实落地，确保养殖业基础生产能力不受损失，确保养殖业稳定发展，确保肉蛋奶和水产品有效供给，为打赢新冠肺炎疫情防控的总体战、阻击战作出贡献。

农业农村部办公厅	国家发展改革委办公厅　　交通运输部办公厅

2020年2月15日

（244）人社部 财政部：发布《关于新型冠状病毒肺炎疫情防控期间事业单位人员有关工资待遇问题的通知》	人社部发〔2020〕9号

各省、自治区、直辖市及新疆生产建设兵团人力资源社会保障厅（局）、财政厅（局），中央和国家机关各部委、各直属机构人事、财务部门：

为贯彻落实习近平总书记关于新型冠状病毒肺炎疫情防控工作的重要指示精神，保障打赢疫情防控阻击战，现就疫情防控期间事业单位人员有关工资待遇问题通知如下：

一、在疫情防控期间，各级人力资源社会保障、财政部门要在当地党委、政府领导下，根据承担新型冠状病毒肺炎疫情防治工作任务情况，因地制宜向承担防控任务重、风险程度高的医疗卫生机构核增一次性绩效工资总量，不作为绩效工资总量基数，所需经费通过现行渠道安排，疫情结束后不再执行。要及时指导有关单位在内部分配时，向敢于担当、勇挑重担、加班加点参加疫情防控的一线工作人员特别是作出突出成绩的人员倾斜。

二、为促进有效实施隔离和医学观察等预防控制措施，根据《中华人民共和国传染病防治法》有关规定，对新型冠状病毒肺炎患者、疑似病人、密切接触者在其隔离治疗期间或医学观察期间以及因政府实施隔离措施或采取其他紧急措施导致不能提供正常劳动的事业单位工作人员，在此期间的工资、福利待遇由其所属单位按出勤对待。

三、各级人力资源社会保障、财政部门要高度重视疫情防控期间事业单位工作人员尤其是医疗卫生工作人员的工资待遇问题，加大对有关地区、部门和单位的指导督促力度，并及时做好政策解释。相关单位要认真执行政策，确保落实到位，强化政策效果，全力支持疫情防控，促进打赢这场疫情防控阻击战。

人力资源社会保障部 财政部

2020年2月11日

（245）卫健委 中医药局：进一步加强社会办医管理做好新冠肺炎疫情防控工作	国卫医函〔2020〕63号

各省、自治区、直辖市及新疆生产建设兵团卫生健康委、中医药局：

新冠肺炎疫情发生以来，全国各级各类医疗机构坚决服从党中央统一指挥调度，主动融入防控工作大局，全力以赴抗击疫情。社会办医疗机构（以下简称社会办医）作为我国医疗卫生服务体系中的重要组成部分，在防控工作中承担着相当重要的任务。当前，疫情形势仍然严峻复杂，防控工作进入关键阶段，为进一步加强社会办医管理，做好新冠肺炎疫情防控工作，现就有关事项通知如下：

一、切实做好患者接诊救治工作。社会办医要严格落实门急诊预检分诊制度，开设发热门诊的要规范设置管理并确保有效运行。接诊要注意询问患者流行病学史，发热患者要做好隔离处置工作，疑似和确诊患者要按照“四早”“四集中”原则及时处置或转运救治，并按有关规定上报。纳入定点医院的社会办医，要选派技术骨干力量参与医疗救治工作，对于重症、危重病例，本单位不能满足医疗救治要求的，应尽快转运到其他救治力量更强的定点医院进行救治，不宜转运的，应请上级卫生健康行政部门协调专家力量给予指导支持。要切实做好日常诊疗管理，重点加强门诊预约、网络/电话咨询、手术择期、严格住院患者及家属管理等工作，保证医疗质量和医疗安全。

二、加强医疗机构内感染预防与控制。社会办医要进一步做好新型冠状病毒感染预防与控制工作，根据病原学特点，结合传染源、传播途径、易感人群和诊疗条件等，制定工作预案，建立预警机制和工作流程。在严格落实标准预防的基础上，强化接触传播、飞沫传播和空气传播的感染防控，严格做好诊疗环境、医疗器械、患者用物等的清洁消毒和医疗废物处置。要加强门诊和病房管理，有关建筑布局和工作流程应当符合医院隔离技术规范，并采取有效措施疏导分流患者，避免人员聚集。要加强医务人员调配和医用物资储备，正确使用医用防护用品，确保医务人员个人防护到位。

三、强化开展疫情防控知识技能培训。有关社会办医学协会要充分发挥行业组织作用，通过APP、第三方平台等形式，围绕新冠病毒相关基础知识、病例发现与报告、诊疗方案、医疗机构内感染和个人防护、病例转运、患者费用保障政策等内容，对社会办医的医务人员进行业务培训。对于医疗服务能力有限的社会办医，可组织专家进行定点指导。社会办医要在对医务人员进行全方位培训的基础上，重点强化医疗机构内新型冠状病毒感染预防与控制技术指南、常见医用防护用品使用范围指引等技术规范和工作要求的培训。

四、全面组织动员社会办医力量。各级卫生健康行政部门（含中医药主管部门）要深刻认识疫情防控的重要性和紧迫性，全面动员、全面部署、全面加强防控工作，统筹调度辖区内各级各类医疗资源。有关社会办医学协会要加强宣传倡导工作，鼓励社会办医同心同德、群策群力，持续不断为疫情防控贡献力量。社会办医不分举办主体、经营性质、类别规模，要统一服从本地区疫情防控工作部署调度，制定具体工作方案，加强医务人员和医用物资调配储备，做好患者接诊救治、医疗机构内感染防控等工作，坚决做到守土有责、守土担责、守土尽责。

国家卫生健康委  国家中医药局

2020年2月15日

（246）铁路部门强化八项措施 全力做好复工返程运输疫情防控

铁路部门进一步强化八项措施，全力做好复工返程运输疫情防控工作。

一是科学安排运力。密切关注各地复工复产安排，充分利用大数据，及时分析客流变化和货运需求。精准安排错峰返程运力，实行“一日一图”，动态增减列车；加强售票管理，实行分散售票策略，禁售无座车票，适当控制旅客列车客座率。精准对接企业复工复产货运需求，“一企一策”制定运输方案，聚焦重点区域，深挖货源潜力，统筹运力安排，加强装卸车组织，提高运输效率。

二是最大限度避免旅客聚集。对车站候车、购票和退票的旅客采取分散措施，规范人脸识别进站检票操作，保持检票有效距离；引导客流较密集车厢的旅客到同等席别较空车厢分散就坐；餐车不再接待旅客就餐和购物，改为列车工作人员定时送盒饭和商品至旅客座席；客运乘务人员对车厢端门加强看管，劝阻旅客尽量不在车厢走动；加强出站组织，保证通道畅通有序，避免人员聚集。

三是加强站车通风消毒保洁。严格落实站车通风、消毒、卫生保洁等防疫措施。优化动车组列车供风，每隔5至10分钟完成一次新风换气；加强空调列车滤网清洗消毒；对非空调列车勤开车窗、及时通风换气；加大各类旅客列车预防性消毒、随时消毒、终末消毒和全程卫生保洁力度，对厕所门把手、洗手台水龙头、废物箱投放处等重点部位加密消毒频次。

四是组织疫情防控公益宣传。运用车站、列车视频和广播，面向旅客和职工，大力宣传国家权威部门机构推出的健康防护知识，循环播放防疫知识公益宣传片，在站车场所发放通俗易懂宣传手册、漫画等，编发铁路职工岗位防疫知识手册。

五是依法科学做好发热旅客防控。配合地方政府防疫部门，在全国铁路所有办理客运业务的车站对进出站旅客测量体温，在列车运行途中对旅客测量体温进行抽检；根据不同车型、席别，在车辆端部预留一定席位，作为发热旅客隔离场所；在旅客列车上发现发热旅客，立即采取移至隔离席位、复测体温和安排消毒等措施，及时下交前方车站；对列车下交或进出站排查发现的发热旅客，及时送留验站或移交地方卫生健康部门。

六是及时协查车上密切接触者。运用12306售票大数据资源，第一时间配合地方政府和卫生健康部门，协查确诊病例乘坐火车时车上密切接触者；落实好购票人须提供乘车人手机号措施，提高协查准确率。

七是及时高效运送医护人员和防控物资。建立高效运输保障机制，坚持特事特办，开辟绿色通道，全力保障湖北及其他重点地区医护人员和防控物资、生活物资的运输，确保以最快的速度运达。

八是加强铁路职工防护工作。要求职工在工作岗位必须佩戴口罩，认真落实勤洗手、常通风、不聚集等防护要求，全面实行出退乘、出入单位测体温制度；对调度指挥等重点岗位人员实行集中管理、严格管理，划小工作单元，严控与外界接触；对客运服务、机辆乘务等一线工作人员，强化个人防护；加强职工心理疏导，纾解焦虑紧张情绪。

2020.2.14

（247）财政部：发布国有金融企业积极做好疫情防控捐赠有关事项	财金函〔2020〕7号

各中央金融企业，各省、自治区、直辖市、计划单列市财政厅（局），新疆生产建设兵团财政局：

新冠肺炎疫情发生以来，国有金融企业（以下简称金融企业）认真贯彻习近平总书记关于加强疫情防控工作的重要指示批示和中央政治局常委会会议精神，按照中央应对疫情工作领导小组会议和国务院常务会议部署，立足自身职能，强化金融服务，践行社会责任，及时向受疫情影响严重地区捐赠，大力组织境外机构开展防疫物资采购，传递正能量，对打赢疫情防控阻击战发挥了积极作用。为进一步做好相关工作，现就金融企业疫情防控捐赠有关事项通知如下：

一、金融企业应积极履行社会责任，在做好疫情防控金融服务的基础上，综合考虑自身职能定位、经营情况、财务状况，结合疫情防控需要，确定疫情防控捐赠事项，包括捐赠金额、捐赠对象、使用方向等。

二、鼓励金融企业实施精准捐赠，切实将疫情防控捐赠资金使在刀刃上，用在关键处。金融企业通过公益性社会组织或县级以上人民政府及其部门等国家机关捐赠时，可在捐赠时明确具体要求，包括资金使用对象、使用条件、使用方式等。

三、鼓励金融企业根据疫情防控需要，采取多种方式实施捐赠。鼓励大型金融企业强化工作统筹，充分发挥境外机构作用，组织采购境内短缺的防疫物资，向公益性社会组织或县级以上人民政府及其部门等国家机关，以及承担疫情防治任务的医院捐赠。鼓励保险机构立足主业，发挥风险保障功能，向一线医务人员、疫情防控工作人员捐赠保险产品。

四、金融企业应当合理调配捐赠资金，疫情防控捐赠在金融企业年度对外捐赠额度中优先安排。

五、金融企业疫情防控捐赠应依法履行公司治理程序。因疫情防控需要实施紧急捐赠的，金融企业可按照“特事特办、急事先办”的原则，采取电话、视频等多种方式，履行相关程序。

六、金融企业疫情防控捐赠符合《关于支持新型冠状病毒感染的肺炎疫情防控有关捐赠税收政策的公告》（财政部 税务总局公告2020年第9号）有关规定的，可按规定享受相应的税收优惠政策。

财政部

2020年2月13日

（248）邮政局：推进邮政业落实支持疫情防控相关财税金融政策

为深入贯彻习近平总书记重要指示批示精神，根据党中央、国务院决策部署，近期国家有关部门精准施策，陆续出台了一系列支持疫情防控的财税金融政策，为邮政业更好发挥在疫情防控中的重要作用，科学有序恢复生产提供了重要保障。2月12日，国家邮政局办公室下发通知，要求切实推进邮政业落实相关财税金融政策，全力做好疫情防控和有序复产工作。

通知指出，要充分认识邮政业疫情防控工作面临的形势和落实好财税金融政策的重要意义。邮政业是国家战略性基础设施和社会组织系统之一，是疫情防控工作的重要一环，在支援医用物资运输、保障群众基本生活、维护正常经济社会秩序方面发挥着重要作用。当前，邮政业最重要的任务是一手抓疫情有效防控，一手抓科学有序复产，做到“四个确保”“三个优先”和“一个梯度推进”，着力为疫情防控贡献行业力量，全力以赴完成行业年度发展的目标任务。各级邮政管理部门、行业协会要充分认识财税金融政策对减轻企业负担、稳定末端网点、提振市场信心的重要意义，全力推动财税金融政策在邮政业落到实处，切实增强企业防控保供能力，为打赢疫情防控阻击战做出贡献。

通知强调，要全面准确把握财税金融政策内容，大力开展相关政策宣传，组织推进政策落地实施。各级邮政管理部门要加强与有关部门的沟通协调，推动将邮政业所属企业列为疫情期间保障城市运行、提供群众生活必需品的重要国计民生相关企业，促进国家财税金融政策以及本地区出台的财政补贴、融资支持、减轻社保负担、减免房屋租金、开展援企稳岗等方面支持政策在行业内落地。要通过多种渠道方式向邮政快递企业宣传讲解政策，加强调研指导，全面掌握企业享受政策情况，推动解决政策落实中的困难和问题。要与推进既有减税降费政策落实工作紧密结合起来，形成政策合力，充分发挥政策工具对于支持企业科学有序复产、保障医用物资运递、保障生产生活的作用。

附：与邮政业密切相关的支持疫情防控财税金融政策

（一）自2020年1月1日起，对纳税人运输疫情防控重点保障物资取得的收入，免征增值税（财政部 税务总局公告2020年第8号）。

（二）自2020年1月1日起，对纳税人提供的为居民提供必需生活物资快递收派服务取得的收入，免征增值税（财政部 税务总局公告2020年第8号）。快递收派服务，是指接受寄件人委托，在承诺的时限内完成函件和包裹的收件、分拣、派送服务的业务活动（财税〔2016〕36号）；中国邮政集团公司及其所属邮政企业提供的邮政普遍服务和邮政特殊服务，免征增值税（财税〔2016〕39号）。

（三）自2020年1月1日起，企业和个人通过公益性社会组织或者县级以上人民政府及其部门等国家机关，捐赠用于应对新型冠状病毒感染的肺炎疫情的现金和物品，允许在计算应纳税所得额时全额扣除。

企业和个人直接向承担疫情防治任务的医院捐赠用于应对新型冠状病毒感染的肺炎疫情的物品，允许在计算应纳税所得额时全额扣除。

单位和个体工商户将自产、委托加工或购买的货物，通过公益性社会组织和县级以上人民政府及其部门等国家机关，或者直接向承担疫情防治任务的医院，无偿捐赠用于应对新型冠状病毒感染的肺炎疫情的，免征增值税、消费税、城市维护建设税、教育费附加、地方教育附加（财政部 税务总局公告2020年第9号）。

（四）适度扩大《慈善捐赠物资免征进口税收暂行办法》规定的免税进口范围，对捐赠用于疫情防控的进口物资，免征进口关税和进口环节增值税、消费税（财政部 海关总署 税务总局公告2020年第6号）。

（五）国家发展改革委、工业和信息化部对以下疫情防控重点保障企业实施名单制管理：……为应对疫情……承担上述物资运输、销售任务的企业。

人民银行向相关全国性银行和疫情防控重点地区地方法人银行发放专项再贷款，支持其向名单内企业提供优惠贷款。金融机构向相关企业提供优惠利率的信贷支持，贷款利率上限为贷款发放时最近一次公布的一年期LPR减100基点。中央财政按企业实际获得贷款利率的50%进行贴息（财金〔2020〕5号）。

（六）对受疫情影响较大的批发零售、住宿餐饮、物流运输、文化旅游等行业，以及有发展前景但受疫情影响暂遇困难的企业，特别是小微企业，不得盲目抽贷、断贷、压贷。对受疫情影响严重的企业到期还款困难的，可予以展期或续贷。通过适当下调贷款利率、增加信用贷款和中长期贷款等方式，支持相关企业战胜疫情灾害影响（银发〔2020〕29号）。

（七）对春节期间（截至2020年2月9日）开工生产、配送疫情防控急需物资的企业，符合条件的可给予一次性吸纳就业补贴。统筹使用工业企业结构调整专项奖补资金，用于支持符合条件的受疫情影响企业稳定岗位、保障基本生活等支出（人社部明电〔2020〕2号）。

（八）对按月申报的纳税人、扣缴义务人，在全国范围内将2020年2月份的法定申报纳税期限延长至2月24日；湖北等疫情严重地区可以视情况再适当延长，具体时间由省税务局确定并报税务总局备案；纳税人、扣缴义务人受疫情影响，在2020年2月份申报纳税期限延长后，办理仍有困难的，还可依法申请进一步延期（税总函〔2020〕19号）。

2020.2.14

（249）交通运输部：做好疫情期间道路运输车辆技术保障工作	交运明电〔2020〕59号

各省、自治区、直辖市、新疆生产建设兵团交通运输厅（局、委）：

为深入贯彻落实习近平总书记重要指示批示精神和党中央、国务院决策部署，坚决打赢疫情防控阻击战，在抓好疫情防控工作的同时统筹抓好道路运输安全稳定发展工作，切实做好疫情期间道路运输车辆技术保障和车辆年审工作，为疫情防控和经济社会发展提供充足的道路运输运力保障，经交通运输部同意，现就有关事项通知如下：

一、对道路运输车辆年审采取临时延期措施

道路运输车辆年审制度是保障道路运输车辆技术管理的重要基础性制度。当前，为全力服务疫情防控工作，在疫情期间各地交通运输部门可结合当地疫情防控工作和道路运输保障需要，采取以下临时政策措施：对道路运输车辆年审时间到期但因疫情原因暂时无法进行车辆审验或不具备相关审验条件的，允许其年审周期自动向后延续至疫情结束之后45天。疫情期间，各地交通运输部门不对未开展技术等级评定、车辆年审逾期或到期未审验的道路运输车辆进行处罚。疫情结束后，各地交通运输部门要督促有关道路客货运输经营者及车辆及时延续办理车辆年审手续；道路运输车辆年审制度恢复到原政策制度执行。

二、做好道路运输车辆性能维护工作

疫情期间，对于未开展车辆年审的道路运输车辆，各地交通运输部门要督促相关道路运输经营者按照规定及时做好车辆性能维护，特别要做好涉及车辆灯光、转向、制动、轮胎等安全系统和部件的性能维护，并做好车辆维护记录，确保车辆技术状况良好和车辆行驶安全。

各地如有紧急情况，应第一时间向部报告。

交通运输部

2020年2月12日

（250）财政部办公厅 教育部办公厅关于切实做好学校疫情防控经费保障工作的通知	财办教〔2020〕11号

　　各省、自治区、直辖市、计划单列市财政厅（局）、教育厅（局、教委），新疆生产建设兵团财政局、教育局：

为深入贯彻落实习近平总书记重要指示批示精神和党中央、国务院决策部署，全力支持打赢疫情防控阻击战，按照《财政部关于进一步做好新型冠状病毒感染肺炎疫情防控经费保障工作的通知》（财办〔2020〕7号）和《教育部关于切实做好新型冠状病毒感染的肺炎疫情防控工作的通知》（教电〔2020〕29号）有关要求，现就做好学校疫情防控经费保障工作通知如下：

一、研究制定经费保障政策措施

地方各级财政、教育部门要认真贯彻落实习近平总书记重要指示批示精神，按照党中央、国务院决策部署，高度重视学校疫情防控工作，切实增强做好学校疫情防控工作的政治责任感、使命感和紧迫感，全力做好学校疫情防控经费保障工作。地方各级财政部门要积极主动与教育部门沟通协商，密切关注疫情对学校教育教学和学习生活成本等的影响，及时研究制定学校疫情防控经费保障政策措施，支持学校确保不因资金问题而影响疫情防控。

二、统筹安排教育经费预算

地方各级财政、教育部门要统筹安排上级教育转移支付和自有财力，进一步优化教育支出结构，加大对疫情防控重点地区学校的支持力度，提升学校防控能力，保障学校正常运转。中央财政已提前下达的支持学前教育发展资金、城乡义务教育补助经费（公用经费补助部分）、改善普通高中学校办学条件补助资金、现代职业教育质量提升计划资金、支持地方高校改革发展资金等教育转移支付资金，由地方财政、教育部门及学校根据相关资金管理办法和学校疫情防控需要，安排用于学校疫情防控物资、设备采购等支出。省级财政部门在分配下达2020年中央和省级相关教育转移支付时，要向疫情防控重点市县倾斜。

三、加快财政教育资金拨付使用

各地财政部门要有序规范组织资金调度，按照“急事急办、特事特办”的原则，加快资金拨付使用，确保学校疫情防控资金及时到位，并会同教育部门强化资金使用监管。要按照《关于疫情防控采购便利化的通知》（财办库〔2020〕23号）要求，建立采购“绿色通道”，根据学校疫情防控需要提高采购时效，保证采购质量。

四、指导学校加强疫情防控经费保障

地方各级教育部门要指导学校结合实际研究制定疫情防控工作经费保障方案，研判资金需求，细化经费保障措施，统筹用好财政资金、学校自有资金和社会捐赠等各渠道资金，确保学校疫情防控工作顺利开展。

五、加强政策跟踪和分析研判

各级财政、教育部门要及时跟踪有关财政经费保障政策的落实情况，发现问题及时研究解决。省级财政、教育部门要密切关注疫情发展态势，分析研判延期开学对各级各类学校学生返校、教育教学、学生资助政策落实等工作的影响，及时制定预案。

经费保障工作中的重要情况和问题，应及时向财政部、教育部报告。

联系人及联系电话：财政部科教和文化司 高瑛泽 010-68551959；教育部财务司 王俊 010-66097557

财政部办公厅 教育部办公厅

2020年2月7日

（251）关于做好新型冠状病毒感染肺炎疫情防控期间学生资助工作的通知

教财司函〔2020〕30号

各省、自治区、直辖市教育厅（教委）、财政厅（局），各计划单列市教育局、财政局，新疆生产建设兵团教育局、财政局，各中央部门所属高等学校：

近期正值新型冠状病毒感染肺炎疫情防控的关键时期，为深入贯彻习近平总书记重要指示精神，落实中央应对疫情工作领导小组会议部署，切实保障家庭经济困难学生基本学习生活需求，全面助力打赢疫情防控阻击战，现就做好疫情防控期间学生资助工作有关要求通知如下：

一、高度重视疫情防控期间学生资助工作。做好疫情防控期间学生资助工作，保障家庭经济困难学生的基本学习生活需求，是学生资助战线全面参与打赢疫情防控阻击战的重要内容，是全面贯彻中央有关决策部署的具体体现。各地各校学生资助管理部门要高度重视，既要按照统一要求，落实好疫情防控相关职责，也要加强组织领导，制定周密方案，全面谋划和部署好新学期学生资助的各项工作。

二、积极资助患病家庭经济困难学生。各地各校学生资助管理部门要根据有关部门及学校掌握的疫情信息，及时了解本地本校家庭经济困难学生的身体和生活等情况。要重点聚焦疫情严重地区、贫困地区、农村地区、边远地区，重点关注建档立卡、低保、特困救助供养、残疾等特殊困难学生群体。对受到疫情影响的家庭经济困难学生，应及时对其予以资助。在本人或家人感染疫病的家庭经济困难学生入校后，各校要根据其家庭经济困难程度，在国家资助的基础上，使用学校按事业收入或学费收入一定比例提取的学生资助基金，采取减免学费或临时生活补助等方式，保障他们的正常学习生活。

三、全面落实好各项学生资助政策。疫情防控期间，各地各校要妥善安排好寒假留校家庭经济困难学生的生活和学习。各地各校应充分考虑疫情期间家庭经济困难学生的实际需求，及时足额发放国家助学金等资助资金，帮助他们顺利度过疫情难关。对于已经毕业且处于还款期的国家助学贷款借款学生，在疫情防控期间因受疫情影响不能正常还本付息的，按《中国人民银行 财政部 银保监会 证监会 外汇局关于进一步强化金融支持防控新型冠状病毒感染肺炎疫情的通知》（银发〔2020〕29号）精神，可合理延后还款期限；如因患新型冠状病毒感染肺炎失去还款能力的，要协调经办银行启动救助机制，为其办理代偿应还本息相关手续，减轻他们的还款压力。

教育部财务司 财政部科教和文化司

2020年2月6日

（252）国务院扶贫开发领导小组：做好新冠肺炎疫情防控期间脱贫攻坚工作	国开发〔2020〕3号

各省、自治区、直辖市和新疆生产建设兵团扶贫开发领导小组，国务院扶贫开发领导小组成员单位：

为贯彻落实习近平总书记重要指示精神和李克强总理要求，按照党中央、国务院关于新冠肺炎疫情防控和脱贫攻坚的决策部署，现就做好疫情防控期间脱贫攻坚工作有关事项通知如下。

一、坚决打赢疫情防控阻击战和脱贫攻坚战

2020年是决胜全面建成小康社会、决战脱贫攻坚之年。脱贫攻坚是必须完成的硬任务，还有不少硬仗要打，突如其来的新冠肺炎疫情，带来了新的困难和挑战。疫情防控阻击战和脱贫攻坚战都是重大政治任务，要认真贯彻落实习近平总书记重要指示精神，切实增强“四个意识”、坚定“四个自信”、做到“两个维护”，一手抓疫情防控，一手抓脱贫攻坚。要坚定信心决心，坚持如期全面完成脱贫攻坚任务不动摇，工作总体安排部署不能变，决不能有缓一缓、等一等的思想。要统筹兼顾，切实把各项工作抓实、抓细、抓落地，坚决打赢疫情防控阻击战和脱贫攻坚战。

二、努力克服疫情对脱贫攻坚的影响

要密切跟踪分析疫情对脱贫攻坚的影响，进一步贯彻落实精准扶贫精准脱贫基本方略，实施分类施策，采取更有针对性的措施。疫情较重地区在符合疫情防控要求下，转变工作方式，充分利用信息化手段，积极稳妥推进脱贫攻坚各项重点工作。疫情较轻或没有疫情的地区，要将脱贫攻坚作为重要工作任务，在做好疫情防控前提下，抓紧向前推进。

（一）解决贫困群众外出务工难题。抓住重大项目建设和企业复工复产的时机，摸清贫困劳动力的基本情况，根据联防联控机制的统一部署，按照“分批有序错峰”的要求，优先组织贫困劳动力返程返岗和外出务工，特别是做好挂牌督战县贫困劳动力就业工作。利用东西部扶贫协作等工作机制，加强输出地和输入地有效对接。利用扶贫车间、公益岗位等，促进贫困人口就近就地就业。结合规范村级光伏扶贫电站的收益分配开发公益岗位，妥善安置因疫情暂时无法外出务工的贫困劳动力。

（二）解决生产发展和产品积压难题。做好春耕备耕工作，组织做好种苗、农资供应，确保农业生产不受影响。认真落实国务院关于公路交通保通保畅要求，强化贫困地区农产品产销对接，充分利用“互联网+”拓宽销售渠道，积极开展消费扶贫专项行动，切实防止农产品积压。支持贫困户发展产业的扶贫小额信贷要及时发放，保持利率、期限不变，适当延长受疫情影响还款困难贫困户的还款期限。

（三）解决扶贫项目开工复工难题。在符合疫情防控条件下，支持和组织推动脱贫攻坚项目建设尽早开工复工，一时还达不到开工复工要求的，要提前备工备料，为开工复工创造条件。采取以工代赈方式，组织贫困劳动力参与扶贫项目建设。提高工作效率，加快建设进度，小微扶贫项目可以按规定程序实行“一事一议”。

三、扎实推进脱贫攻坚重点工作

今年的脱贫攻坚工作已经作出全面部署，疫情防控期间，要结合高质量打赢脱贫攻坚战要求，抓紧推进各项重点工作落实，落实贫困县“四个不摘”，巩固脱贫成果，确保经得起历史和人民的检验。

（一）做好脱贫攻坚挂牌督战。贯彻落实好《国务院扶贫开发领导小组关于开展挂牌督战工作的指导意见》，制定县、村挂牌督战实施方案，抓紧开展督战工作，及时解决制约完成脱贫攻坚任务的突出问题，确保深度贫困地区按期脱贫摘帽。

（二）查漏补缺补齐短板。紧盯重点地区和重点问题，尽快补齐短板，抓紧完成剩余脱贫任务，不留死角。抓好脱贫攻坚问题排查发现问题整改工作，不等不拖。脱贫攻坚专项巡视“回头看”、成效考核发现问题反馈后，要一体整改、一体推进，以问题整改促进脱贫攻坚提质增效。

（三）建立机制防止返贫。建立防止返贫监测和帮扶机制，对存在返贫致贫风险的脱贫人口和边缘人口进行动态监测，及时将返贫人口和新发生贫困人口纳入帮扶范围，采取措施防止返贫和新的致贫。

（四）加强易地扶贫搬迁后续帮扶工作。要高度重视易地扶贫搬迁集中安置点的后续帮扶工作，加大产业发展的支持力度，广泛开展技能培训和就业指导，提供基本公共服务，做好社区治理和社会融入等工作，确保搬得出、稳得住、有就业、逐步能致富。

四、加强疫情防控和脱贫攻坚宣传引导工作

要加大疫情防控知识宣传力度，教育引导贫困户树立健康理念、养成良好卫生习惯，摒弃滥食野生动物陋习，做到科学防控，提高自我防护能力。要充分发挥驻村工作队在疫情防控和脱贫攻坚中的重要作用，把工作落实到村组和家庭。要做好脱贫攻坚政策解读工作，做好舆情应对，及时回应社会关切。要创新宣传方式，大力宣传疫情防控期间的脱贫攻坚生动事例和感人事迹，形成脱贫攻坚强大正能量。

五、转变作风关心贫困群众和扶贫干部

要发扬斗争精神，越是困难越向前，敢于担当、求真务实、一抓到底，在疫情防控和脱贫攻坚中检验干部、锻炼干部。要克服形式主义官僚主义问题，努力为基层减负，集中精力抓疫情防控和脱贫攻坚。要关注贫困群众生产生活和疫情防控，加强心理干预和疏导，有针对性做好人文关怀。要关心基层扶贫干部的工作生活和身心健康，做好疫情防护保障安全，帮助他们解决实际困难。

各地贯彻落实本通知情况，对本地疫情影响分析和采取的应对举措及相关建议，于2月20日前报国务院扶贫办。

国务院扶贫开发领导小组

2020年2月12日

（253）财政部：进一步做好新型冠状病毒感染肺炎疫情防控经费保障工作	财办〔2020〕7号

各省、自治区、直辖市、计划单列市财政厅（局），新疆生产建设兵团财政局：

近期，湖北省武汉市等地发生新型冠状病毒感染肺炎疫情，防控形势十分严峻。各级财政部门认真贯彻落实习近平总书记的重要指示精神，按照党中央、国务院的统一部署，及时响应、果断应对，疫情防控经费保障工作总体有序有力。但也要看到，这次疫情防控时间紧、任务重，又逢春节假期，防控形势依然复杂严峻。各级财政部门要切实把思想和行动统一到习近平总书记重要指示精神上来，统一到党中央、国务院决策部署上来，立足财政部门职责，加强统筹谋划，采取更加有力的举措，全力支持疫情防控工作，坚决打赢疫情防控阻击战。现就进一步做好疫情防控经费保障工作通知如下：

一、明确经费保障目标

各级财政部门要按照习近平总书记重要指示精神，坚决贯彻党中央、国务院的决策部署，按照财政部《关于认真学习贯彻习近平总书记重要指示精神 在打赢疫情防控阻击战中积极有效发挥财政职能作用的通知》（财办〔2020〕5号）的要求，进一步增强支持做好疫情防控工作的政治责任感、使命感和紧迫感，切实加强组织领导，努力做到工作部署更细致、政策考虑更周全、资金安排更科学，全力做好疫情防控经费保障工作。确保人民群众不因担心费用问题而不敢就诊，确保各地不因资金问题而影响医疗救治和疫情防控。

二、统筹安排经费预算

近期，按照党中央、国务院统一部署，财政部会同有关部门已经明确了患者救治费用补助、参加防治工作的医务工作者临时性工作补助以及防护、诊断和治疗专用设备、快速诊断试剂采购经费补助等政策，并预拨了部分资金。地方各级财政部门要用好中央财政转移支付资金，同时千方百计筹措资金，切实履行好经费保障职能。年初卫生防疫经费预算安排不足的，要及时调整增加预算安排。要统筹一般公共预算、政府性基金预算等财政资金以及社会捐赠资金，进一步优化财政支出结构，大力盘活财政存量资金，加大医疗卫生投入力度，优先保障疫情防控经费需求。充分保障相关部门疫情防控工作经费，加大对疫情严重、防控资金缺口较大地区的转移支付力度。各地不得将上级财政安排的疫情防控相关转移支付资金用于平衡预算。对应由地方财政负担的支出，地方各级财政部门要足额安排资金；对应由中央财政负担的支出，要求地方先行垫付的，地方各级财政部门要积极筹措资金，足额安排垫付资金，并做好建立台账、票据审核等资金结算基础工作。同时，对基层财政“三保”经费的保障力度也要切实加强。

三、加快调度拨付资金

地方各级财政部门要加强库款形势分析研判，有序规范组织资金调度，必要时可采取预拨、垫付等措施，优先保障疫情防控资金拨付。按照“急事急办、特事特办”的原则，协调人民银行、代理国库集中支付业务的商业银行、经办财政专户业务的商业银行等，开通资金支付绿色通道，及时足额支付预算安排的疫情防控资金。省级财政部门要密切关注各市县库款情况，督导疫情防控任务重、库款保障水平偏低的市县科学调度资金，并适时予以专项调度支持，切实保障基层疫情防控支出需要。

四、细化相关政策措施

地方各级财政部门要积极主动与相关业务主管部门协商，研究细化疫情防控经费保障政策措施。要配合人力资源社会保障、卫生健康等部门细化疫情防控医疗卫生工作人员待遇保障政策，按规定抓紧落实；配合医疗保障部门细化确诊和疑似参保患者、异地就医参保患者医疗费用个人负担部分财政补助政策，科学合理确定补助标准,明确医保支付、财政补助及个人负担费用的具体结算流程；配合卫生健康、医疗保障等部门明确未参保患者医疗费用补助政策；配合生态环境、卫生健康等部门做好医疗废物应急处置工作。在当地党委政府的统一领导下，地方各级财政部门可结合本地实际，及时研究出台其他相关保障政策，全面做好疫情经费保障工作。

五、支持做好物资保障

地方各级财政部门要积极配合有关部门做好疫情防控物资保障和采购工作。一是积极配合相关部门做好医药物资采购储备调拨和资金保障工作。二是根据肺炎疫情科研攻关需要，加大资金支持，全力配合相关部门及时拨付资金，切实保障研制快速简易确诊试剂、疫苗、有效药物等科研攻关的资金需求。全力配合相关部门加大对疫苗研究的支持力度，争取尽早投入使用。三是密切关注农副产品市场供应，配合相关部门做好动用政府储备粮油相关工作，确保供应充足、物价稳定。四是支持开展饮用水源等应急环境加密监测，强化应急监测物资保障，切实保障人民群众饮水安全，避免疾病传播和环境污染。五是要按照《财政部办公厅关于疫情防控采购便利化的通知》（财办库〔2020〕23号）要求，做好疫情防控物资采购工作。

六、强化资金使用监管

地方各级财政部门要及时制定疫情防控资金使用管理具体办法，明确经费列支渠道和相关预算调剂程序，既要优先保障疫情防控资金需求，又要确保有限的资金用在“刀刃”上。要加强对资金使用的事前、事中和事后全流程监管，规范资金审批程序，确保资金专款专用，任何地方不得擅自截留、挤占、挪用或改变资金用途。要“关口前移”，督促指导相关部门建立健全资金使用明细账和各项物资入库登记、领用发放及使用管理等规章制度，确保手续完备、凭证齐全。严禁使用财政补助资金超标准装修改造或购置与疫情防控工作无关的设备、器材、交通工具等。财政部各地监管局要做好资金的追踪问效，发现问题应及时向财政部报告。

七、切实做好信息上报和新闻宣传工作

地方各级财政部门要进一步畅通信息沟通渠道，提升信息报送的时效性和准确性。及时跟踪有关财政经费保障政策的落实情况，发现问题要及时研究应对预案，重大问题要及时向财政部报告。及时总结各地有关疫情防控情况，特别是要总结推广经费调度、物资采购等工作中好的经验和做法。及时上报各地疫情防控财政投入情况和出台的经费保障政策，严格执行财政部疫情防控财政工作日报制度。及时报送工作信息，认真审核信息内容，确保信息的真实性和准确性。要加强新闻宣传和舆论引导，主动回应社会关切，及时公开财政支持保障信息，增强社会信心。

各级财政部门要按照本通知要求，进一步提高政治站位，加大工作力度，确保疫情防控经费保障政策落实到位、工作部署到位、预算安排到位、资金拨付到位、监督管理到位，全力支持做好疫情防控工作，坚决打赢疫情防控阻击战。

财 政 部

2020年1月31日

（254）交通运输部 卫健委：切实简化疫情防控应急运输车辆通行证办理流程及落实对应急运输保障人员不实行隔离措施	交运明电〔2020〕57号

各省、自治区、直辖市、新疆生产建设兵团交通运输厅（局、委）、卫生健康委：

为深入贯彻落实习近平总书记关于新冠肺炎疫情防控和维护正常经济社会秩序的重要指示精神，进一步做好交通运输保障工作，有力支撑疫情防控和企业复工复产，现就有关事项通知如下：

一、切实简化疫情防控应急运输车辆通行证办理流程。地方各级交通运输部门要认真落实《交通运输部关于切实保障疫情防控应急物资运输车辆顺畅通行的紧急通知》（交运明电〔2020〕37号）和《交通运输部关于疫情防控期间免收农民工返岗包车公路通行费的通知》（交公路明电〔2020〕52号）要求，切实简化“新型冠状病毒感染肺炎疫情防控应急物资及人员运输车辆通行证”（以下简称“车辆通行证”）、“新型冠状病毒感染肺炎疫情防控期间农民工返岗包车通行证”（以下简称“包车通行证”）办理流程，通过政府网站、微信、电子邮件等便捷方式提供“车辆通行证”“包车通行证”统一式样，供承运单位或驾驶人在本地自行打印、自行填写，严禁要求承运单位或驾驶人到任何交通运输部门履行审批程序，严禁要求在“车辆通行证”“包车通行证”上盖章。对持有上述通行证的车辆，要严格执行应急运输绿色通道政策，确保应急运输车辆不停车、不检查、不收费，优先便捷通行。

二、落实对应急运输保障人员不实行隔离措施。《中央应对新型冠状病毒感染肺炎疫情工作领导小组关于做好春节后错峰返程加强疫情防控工作的通知》（国发明电〔2020〕1号）、《国务院办公厅关于做好公路交通保通保畅工作确保人员车辆正常通行的通知》要求对于短期向疫情重点区域运送物资的司机、装卸工等提供保障的人员，原则上不需采用隔离14天的措施。但在具体实施过程中，仍有一些地方不按要求执行，对进出湖北省及其他疫情重点区域的保障人员采取“一刀切”的隔离措施，造成公路应急运力和人员短缺，严重影响了交通运输保障工作的正常开展。

为切实做好疫情防控期间的交通运输保障工作，地方各级交通运输部门要会同卫生健康部门，认真落实党中央、国务院有关决策部署，对短期向湖北省等疫情重点区域运送物资的司机、装卸工（包括邮政快递车辆司机、装卸工）等提供保障的人员，以及从事道路水路客运但未进入武汉市的司乘人员，经过体温检测符合规定的，在采取戴口罩等必要防护措施的前提下，不得采取隔离措施，坚决避免地方“一刀切”采取隔离措施影响交通运输保障工作。

附件：

1.新型冠状病毒感染肺炎疫情防控应急物资及人员运输车辆通行证

2.新型冠状病毒感染肺炎疫情防控期间农民工返岗包车通行证

交通运输部

国家卫生健康委

2020年2月12日

（255）国务院应对新型冠状病毒感染肺炎疫情联防联控机制：印发新冠肺炎流行期间办公场所和公共场所空调通风系统运行管理指南	肺炎机制综发〔2020〕50号

各省、自治区、直辖市及新疆生产建设兵团应对新型冠状病毒肺炎疫情联防联控机制（领导小组、指挥部）：

为指导办公场所和公共场所安全合理使用空调通风系统，阻止疫情蔓延和扩散，现将《新冠肺炎流行期间办公场所和公共场所空调通风系统运行管理指南》印发给你们，请参照执行。

国务院应对新型冠状病毒肺炎

疫情联防联控机制综合组

（代  章）

2020年2月12日

（信息公开形式：主动公开）

新冠肺炎流行期间办公场所和公共场所

空调通风系统运行管理指南

一、目的

为保证新冠肺炎流行期间，办公场所和公共场所空调通风系统的安全合理使用，防止因空调通风系统开启而导致新冠肺炎疫情的传播和蔓延，最大限度地保护使用者，特制定本指南。

二、运行要求

（一）当空调通风系统为全空气系统时，应当关闭回风阀，采用全新风方式运行。

（二）当空调通风系统为风机盘管加新风系统时，应当满足下列条件：

1.应当确保新风直接取自室外，禁止从机房、楼道和天棚吊顶内取风；

2.保证排风系统正常运行；

3.对于大进深房间，应当采取措施保证内部区域的通风换气；

4.新风系统宜全天运行。

（三）当空调通风系统为无新风的风机盘管系统（类似于家庭分体式空调）时，应当开门或开窗，加强空气流通。

三、管理要求

（一）新风采气口及其周围环境必须清洁，确保新风不被污染。

（二）对于人员流动较大的商场、写字楼等场所，不论空调系统使用运行与否，均应当保证室内全面通风换气；并且，每天下班后，新风与排风系统应当继续运行1小时，进行全面通风换气，以保证室内空气清新。

（三）人员密集的场所应当通过开门或开窗的方式增加通风量，同时工作人员应当佩戴口罩。

（四）建议关闭空调通风系统的加湿功能。

（五）加强对风机盘管的凝结水盘、冷却水的清洁消毒。

（六）下水管道、空气处理装置水封、卫生间地漏以及空调机组凝结水排水管等的U型管应当定时检查，缺水时及时补水，避免不同楼层间空气掺混。

（七）当场所出现下列情况时应当停止使用空调通风系统：

1.发现疑似、确诊新型冠状病毒感染的肺炎病例;

2.集中空调通风系统的类型、供风范围等情况不清楚。

（八）空调通风系统的清洗消毒应当符合下列要求：

1.空调通风系统的常规清洗消毒应当符合《公共场所集中空调通风系统清洗消毒规范》（WS/T 396-2012）的要求。可使用250mg/L～500mg/L含氯（溴）或二氧化氯消毒液，进行喷洒、浸泡或擦拭，作用10min～30min。对需要消毒的金属部件建议优先选择季铵盐类消毒剂。

2.当发现新冠肺炎确诊病例和疑似病例时，在疾病预防控制中心的指导下，对空调通风系统进行消毒和清洗处理，经卫生学评价合格后方可重新启用。

（256）扶贫办 银保监会：积极应对新冠肺炎疫情影响切实做好扶贫小额信贷工作

为努力化解新冠肺炎疫情影响，促进扶贫小额信贷健康发展，2月10日，国务院扶贫办、中国银保监会联合印发了《关于积极应对新冠肺炎疫情影响切实做好扶贫小额信贷工作的通知》。

《通知》从６个方面对积极应对新冠肺炎疫情影响，切实做好扶贫小额信贷工作提出明确要求。一是适当延长受疫情影响出现还款困难的贫困户扶贫小额信贷还款期限。二是在疫情期间，对新发放贷款、续贷和展期需求，要加快审批进度，简化业务流程，提高办理效率。三是要摸清贫困户生产经营受疫情影响情况，提前做好预案，对贫困群众春季生产和后期恢复生产资金需求，符合申贷、续贷、追加贷款等条件的，及时予以支持。四是要充分发挥村两委、驻村帮扶工作队等基层力量作用，做好扶贫小额信贷政策宣传和贷款使用跟踪指导。五是要强化监测防范风险，特别要加强对受疫情影响严重、还款压力较大地区的业务指导，切实防范信用风险。六是各级扶贫部门和银行保险监管部门要加强组织领导，做好工作衔接，充分发挥扶贫小额信贷作用，帮助受疫情影响贫困户尽快恢复生产、实现稳定脱贫，助力高质量打赢脱贫攻坚战。

附件：

国务院扶贫办 中国银保监会

关于积极应对新冠肺炎疫情影响

切实做好扶贫小额信贷工作的通知

各省、自治区、直辖市扶贫办（局）、各银保监局：

2020年是全面建成小康社会目标实现之年，是脱贫攻坚收官之年。当前，新冠肺炎疫情对脱贫攻坚造成一定影响，部分地区扶贫小额信贷工作面临困难。为深入贯彻落实习近平总书记重要指示精神，努力化解疫情影响，促进扶贫小额信贷健康发展，现就做好近期工作通知如下：

一、适当延长还款期限

各级扶贫部门和银行保险监管部门要尽快摸排本地区受疫情影响情况，指导承办银行机构结合本地实际，适当延长到期日在2020年1月1日后（含续贷、展期）、受疫情影响出现还款困难的贫困户扶贫小额信贷还款期限。延期最长不超过6个月，期间继续执行原合同条款，各项政策保持不变。鼓励承办银行机构适当降低延期期间贷款利率。

二、简化业务流程手续

指导承办银行机构在疫情期间，对新发放贷款、续贷和展期需求，加快审批进度，简化业务流程，提高业务办理效率。引导贫困户通过电话银行、手机银行、网络银行等线上方式和村村通金融服务点申请贷款和自助还款。对受疫情影响的贫困户，可采取多种方式灵活办理业务，待疫情解除后，按程序补办相关手续，期间发生逾期的不纳入征信失信记录。

三、切实满足有效需求

摸清贫困户生产经营受疫情影响情况，充分考虑春季生产需要和后期恢复生产资金需求，提前做好预案，督促指导承办银行机构认真落实《中国银保监会 财政部 中国人民银行 国务院扶贫办关于进一步规范和完善扶贫小额信贷管理的通知》（银保监发〔2019〕24号）要求，对贫困群众的生产资金需求，符合申贷、续贷、追加贷款等条件的，及时予以支持。保持扶贫小额信贷政策稳定性，不抬高贷款门槛，不缩短贷款期限。

四、充分发挥基层作用

充分发挥村两委、驻村帮扶工作队等基层力量作用，通过电话、互联网等多种方式，加强与贫困户联系沟通，在坚决打赢疫情防控阻击战的同时，做好扶贫小额信贷政策宣传和贷款使用跟踪指导。学习借鉴湖南省宜章县扶贫小额信贷“四员”工作法等先进经验，通过设置信贷管户员、产业指导员、科技特派员、电商销售员等方式，帮助贫困户用好扶贫小额信贷。

五、强化监测防范风险

做好扶贫小额信贷数据录入管理，及时与承办银行机构开展数据信息共享比对，加强实时监测分析，全面掌握年内到期贷款金额比重、区域分布，对受疫情影响严重、还款压力较大的重点地区，要持续予以关注，加强业务指导，切实防范信用风险。

六、加强组织领导

各级扶贫部门和银行保险监管部门要增强“四个意识”，坚定“四个自信”，做到“两个维护”，切实把思想和行动统一到习近平总书记的重要指示精神上来，做好工作衔接和组织协调，充分发挥扶贫小额信贷作用，帮助受疫情影响贫困户尽快恢复生产、实现稳定脱贫，助力高质量打赢脱贫攻坚战。

国务院扶贫办 中国银保监会

2020年2月10日

（257）国务院应对新型冠状病毒感染肺炎疫情联防联控机制：压实“菜篮子”市长负责制 做好农产品稳产保供工作

各省、自治区、直辖市人民政府，国务院各部委、各直属机构：

近期，在防控新冠肺炎疫情中，一些地方农产品生产运输销售受阻，部分养殖企业饲料供应短缺，给居民生活必需品供应带来较大影响。党中央、国务院对此高度重视。习近平总书记多次作出重要指示批示，强调要切实维护正常经济社会秩序，确保蔬菜、肉蛋奶、粮食等居民生活必需品供应。李克强总理多次安排部署，要求确保疫情防控物资和生活必需品等稳定供应。为贯彻落实党中央、国务院决策部署，进一步压实“菜篮子”市长负责制，切实抓好农产品稳产保供，坚决打赢疫情防控阻击战，现将有关事项通知如下：

一、严格落实地方属地责任。要强化地方首责，把“菜篮子”产品稳产保供作为一项重要政治任务，严格落实“菜篮子”市长负责制，地方政府特别是市级政府要负责辖区内蔬菜、肉蛋奶、水产品等供应，统筹抓好生产发展、产销衔接、流通运输、市场调控、质量安全等各项工作。加强监测预警，完善应急预案，搞好调剂调运。产区要保证本区域“菜篮子”产品产得出、运得走、不积压、不卖难；销区要主动对接产区，保证“菜篮子”产品调得进、供得上、不脱销、不断档。公开热线电话等问题反映和受理渠道，及时解决农产品稳产保供中存在的突出问题，力戒形式主义、官僚主义。

二、抓好“菜篮子”产品生产。要积极引导种植大户、合作社等规模经营主体开展互助合作、错峰采收，解决蔬菜生产用工难、用工贵问题。抓好“南菜北运”基地和北方设施蔬菜产区蔬菜生产，扩大市场供应量。大力发展工厂化育苗，缩短蔬菜生长周期，大中城市周边适当发展速生叶菜、芽苗菜，加快成熟上市。加快恢复生猪生产，及时做好畜禽补栏，毫不放松抓好非洲猪瘟、禽流感等重大动物疫病防控。推进水产品绿色健康养殖，保障水产饲料和苗种供应。加大蔬菜农药、禽蛋和水产品兽药残留监测监管力度，切实守好“菜篮子”产品质量安全底线。

三、保障道路运输通畅。要落实好鲜活农产品运输“绿色通道”政策，维护正常市场流通秩序。把粮油、蔬菜、肉蛋奶、水产品等农产品纳入疫情防控期间生活必需品保障范围，除必要的对司机快速体温检测外，对运输车辆严格落实不停车、不检查、不收费等优先便捷通行措施，确保区域间快速调运，必要的地方可设立农产品运输“接驳区”。畅通农业生产资料物流通道，不得拦截蔬菜种苗、仔畜雏禽及种畜禽、水产种苗、饲料、化肥等农资运输车辆。对承运的企业和车主，地方财政可适当给予补助。

四、促进农产品流通销售。各地要支持有条件的加工销售企业和冷链物流企业扩大鲜活农产品收购，有序组织市场投放，防止出现卖难和断供。充分发挥流通企业、电商平台作用，推进批发市场、物流配送和销售终端互联互通，实现从批发到零售的有机衔接和高效运转。有序组织批发市场、商场超市、社区门店等商业网点复工开业，增加“菜篮子”产品货架种类、数量，保障市场供应和价格基本稳定。

五、加强活禽交易市场分类管理。各地要按照依法依规、分类管理、精准施策的要求，加强活禽交易市场管理，提高本地区禽肉产品供给保障能力。对正常开放的，明确开办者和销售者责任，完善经营设施条件，加强监管排查，严格落实按时清洗消毒、定期休市、过夜零存栏等管理措施。对因防范疫情暂停交易的，通过集中屠宰、产销对接等方式，建立“点对点”活禽销售通道，帮助养殖场户解决活禽屠宰上市问题。对有条件和已实行永久性关闭的，统筹谋划禽肉、猪牛羊肉及其他“菜篮子”产品供应，加强产加销对接，提升产业链质量，完善配送体系，推行冰鲜和冷冻肉类上市，保障居民消费。

六、加快养殖行业上下游企业复产。要着力解决养殖场户断粮、缺料、缺药等突出问题，推动饲料、屠宰等养殖行业上下游企业尽快复产复工，促进养殖业健康发展。加大玉米等储备粮投放，加快豆粕等生产企业开工，保障大宗饲料原料供给。将饲料、种畜禽、兽药、畜产品包装材料等生产企业列入复工复产重点企业名单，加快复工复产。不得关闭正常经营的屠宰场，支持合法合规的屠宰场尽快复工。

七、加快信贷支持政策落地。要及时将与疫情防控重点物资保障相关的饲料、种畜禽及种子（苗）、屠宰、奶业、“菜篮子”产品等骨干生产企业纳入国家专项再贷款和贴息政策支持范围，严格名单制管理，组织开展银企对接，细化实化具体措施，将专项再贷款和贴息资金尽快落实到位，确保专款专用。

八、强化部门协同配合。要充分发挥联防联控机制作用，落实“菜篮子”食品管理部际联席会议制度，切实提高政治站位，强化部门协作，形成工作合力。发展改革、公安、财政、交通运输、农业农村、商务、卫生健康、人民银行、市场监管、银保监等部门要各司其职、密切配合，把疫情防控和稳产保供各项政策举措不折不扣落到实处，确保居民生活必需品供应，维护正常经济社会秩序。

国务院应对新型冠状病毒感染肺炎疫情联防联控机制

2020年2月12日

（258）税务总局：发布支持新型冠状病毒感染的肺炎疫情防控有关税收征收管理事项

为支持新型冠状病毒感染的肺炎疫情防控工作，贯彻落实相关税收政策，现就税收征收管理有关事项公告如下：

一、疫情防控重点保障物资生产企业按照《财政部 税务总局关于支持新型冠状病毒感染的肺炎疫情防控有关税收政策的公告》（2020年第8号，以下简称“8号公告”）第二条规定，适用增值税增量留抵退税政策的，应当在增值税纳税申报期内，完成本期增值税纳税申报后，向主管税务机关申请退还增量留抵税额。

二、纳税人按照8号公告和《财政部 税务总局关于支持新型冠状病毒感染的肺炎疫情防控有关捐赠税收政策的公告》（2020年第9号，以下简称“9号公告”）有关规定享受免征增值税、消费税优惠的，可自主进行免税申报，无需办理有关免税备案手续，但应将相关证明材料留存备查。

适用免税政策的纳税人在办理增值税纳税申报时，应当填写增值税纳税申报表及《增值税减免税申报明细表》相应栏次；在办理消费税纳税申报时，应当填写消费税纳税申报表及《本期减（免）税额明细表》相应栏次。

三、纳税人按照8号公告和9号公告有关规定适用免征增值税政策的，不得开具增值税专用发票；已开具增值税专用发票的，应当开具对应红字发票或者作废原发票，再按规定适用免征增值税政策并开具普通发票。

纳税人在疫情防控期间已经开具增值税专用发票，按照本公告规定应当开具对应红字发票而未及时开具的，可以先适用免征增值税政策，对应红字发票应当于相关免征增值税政策执行到期后1个月内完成开具。

四、在本公告发布前，纳税人已将适用免税政策的销售额、销售数量，按照征税销售额、销售数量进行增值税、消费税纳税申报的，可以选择更正当期申报或者在下期申报时调整。已征应予免征的增值税、消费税税款，可以予以退还或者分别抵减纳税人以后应缴纳的增值税、消费税税款。

五、疫情防控期间，纳税人通过电子税务局或者标准版国际贸易“单一窗口”出口退税平台等（以下简称“网上”）提交电子数据后，即可申请办理出口退（免）税备案、备案变更和相关证明。税务机关受理上述退（免）税事项申请后，经核对电子数据无误的，即可办理备案、备案变更或者开具相关证明，并通过网上反馈方式及时将办理结果告知纳税人。纳税人需开具纸质证明的，税务机关可采取邮寄方式送达。确需到办税服务厅现场结清退（免）税款或者补缴税款的备案和证明事项，可通过预约办税等方式，分时分批前往税务机关办理。

六、疫情防控期间，纳税人的所有出口货物劳务、跨境应税行为，均可通过网上提交电子数据的方式申报出口退（免）税。税务机关受理申报后，经审核不存在涉嫌骗取出口退税等疑点的，即可办理出口退（免）税，并通过网上反馈方式及时将办理结果告知纳税人。

七、因疫情影响，纳税人未能在规定期限内申请开具相关证明或者申报出口退（免）税的，待收齐退（免）税凭证及相关电子信息后，即可向主管税务机关申请开具相关证明，或者申报办理退（免）税。

因疫情影响，纳税人无法在规定期限内收汇或办理不能收汇手续的，待收汇或办理不能收汇手续后，即可向主管税务机关申报办理退（免）税。

八、疫情防控结束后，纳税人应按照现行规定，向主管税务机关补报出口退（免）税应报送的纸质申报表、表单及相关资料。税务机关对补报的各项资料进行复核。

九、疫情防控重点保障物资生产企业按照8号公告第一条规定，适用一次性企业所得税税前扣除政策的，在优惠政策管理等方面参照《国家税务总局关于设备器具扣除有关企业所得税政策执行问题的公告》（2018年第46号）的规定执行。企业在纳税申报时将相关情况填入企业所得税纳税申报表“固定资产一次性扣除”行次。

十、受疫情影响较大的困难行业企业按照8号公告第四条规定，适用延长亏损结转年限政策的，应当在2020年度企业所得税汇算清缴时，通过电子税务局提交《适用延长亏损结转年限政策声明》（见附件）。

十一、纳税人适用8号公告有关规定享受免征增值税优惠的收入，相应免征城市维护建设税、教育费附加、地方教育附加。

十二、9号公告第一条所称“公益性社会组织”，是指依法取得公益性捐赠税前扣除资格的社会组织。

企业享受9号公告规定的全额税前扣除政策的，采取“自行判别、申报享受、相关资料留存备查”的方式，并将捐赠全额扣除情况填入企业所得税纳税申报表相应行次。个人享受9号公告规定的全额税前扣除政策的，按照《财政部 税务总局关于公益慈善事业捐赠个人所得税政策的公告》（2019年第99号）有关规定执行；其中，适用9号公告第二条规定的，在办理个人所得税税前扣除、填写《个人所得税公益慈善事业捐赠扣除明细表》时，应当在备注栏注明“直接捐赠”。

企业和个人取得承担疫情防治任务的医院开具的捐赠接收函，作为税前扣除依据自行留存备查。

十三、本公告自发布之日施行。

特此公告。

附件：适用延长亏损结转年限政策声明

国家税务总局

2020年2月10日

（259）卫生健康委：印发不同人群预防新型冠状病毒感染口罩选择与使用技术指引	肺炎机制发〔2020〕20号

各省、自治区、直辖市及新疆生产建设兵团应对新型冠状病毒感染的肺炎疫情联防联控机制(领导小组、指挥部)：

在新型冠状病毒感染的肺炎流行期间，为指导不同人群科学合理地选择和使用口罩，严防新型冠状病毒感染的肺炎疫情蔓延和扩散，制定了《不同人群预防新型冠状病毒感染口罩选择和使用技术指引》。现印发给你们，请参照执行。

国务院应对新型冠状病毒感染的

肺 炎 疫 情 联 防 联 控 机 制

(代  章)

2020年2月4日

(信息公开形式：主动公开)

不同人群预防新型冠状病毒感染口罩选择与使用技术指引

在新型冠状病毒感染的肺炎流行期间，建议选择合适的口罩类型，不过度防护。按防疫工作性质和风险等级提出以下指引：

一、高风险暴露人员

(一)人员类别：

1.在收治新型冠状病毒肺炎患者(确诊病例、疑似病例)的病房、ICU和留观室工作的所有工作人员，包括临床医师、护士、护工、清洁工、尸体处理人员等;

2.疫区指定医疗机构发热门诊的医生和护士;

3.对确诊病例、疑似病例进行流行病学调查的公共卫生医师。

(二)防护建议：

1.医用防护口罩;

2.在感染患者的急救和从事气管插管、气管镜检查时加戴护目镜或防护面屏;

3.医用防护口罩短缺时，可选用符合N95/KN95及以上标准颗粒物防护口罩替代，也可选用自吸过滤式呼吸器(全面型或半面型)配防颗粒物的滤棉，动力送风过滤式呼吸器的防护效果更佳。

二、较高风险暴露人员

(一)人员类别：

1.急诊科工作医护人员等;

2.对密切接触人员开展流行病学调查的公共卫生医师;

3.疫情相关的环境和生物样本检测人员。

(二)防护建议：

符合N95/KN95及以上标准的颗粒物防护口罩;

三、中等风险暴露人员

(一)人员类别：

1.普通门诊、病房工作医护人员等;

2.人员密集场所的工作人员，包括医院、机场、火车站、地铁、地面公交、飞机、火车、超市、餐厅等相对密闭场所的工作人员;

3.从事与疫情相关的行政管理、警察、保安、快递等从业人员;

4.居家隔离及与其共同生活人员。

(二)防护建议：

佩戴医用外科口罩。

四、较低风险暴露人员

(一)人员类别：

1. 超市、商场、交通工具、电梯等人员密集区的公众;

2. 室内办公环境;

3. 医疗机构就诊(除发热门诊)的患者;

4. 集中学习和活动的托幼机构儿童、在校学生等。

(二)防护建议。

佩戴一次性使用医用口罩(儿童选用性能相当产品)。

五、低风险暴露人员

(一)人员类别。

1.居家室内活动、散居居民;

2.户外活动者，包括空旷场所/场地的儿童、学生;

3.通风良好工作场所工作者。

(二)防护建议。

居家、通风良好和人员密度低的场所也可不佩戴口罩。非医用口罩，如棉纱、活性炭和海绵等口罩具有一定防护效果，也有降低咳嗽、喷嚏和说话等产生的飞沫播散的作用，可视情选用。

六、使用事项

在新型冠状病毒感染的肺炎流行期间，在保障公众健康的前提下，可适当延长口罩使用(使用时间、使用次数)。

(一)口罩更换。

1.医用标准的防护口罩均有使用期限，口罩专人专用，人员间不能交叉使用。高风险人员在结束工作、中途进餐(饮水)、入厕等脱下防护装置后，重新进入需更换;

2.口罩被患者血液、呼吸道/鼻腔分泌物，以及其他体液污染要立即更换;

3.较高风险人员在接诊高度疑似患者后需更换;

4.其他风险类别暴露人员佩戴的口罩可反复多次使用。口罩佩戴前按规程洗手，佩戴时避免接触口罩内侧。口罩脏污、变形、损坏、有异味时需及时更换。

(二)口罩保存、清洗和消毒。

1.如需再次使用的口罩，可悬挂在洁净、干燥通风处，或将其放置在清洁、透气的纸袋中。口罩需单独存放，避免彼此接触，并标识口罩使用人员;

2.医用标准防护口罩不能清洗，也不可使用消毒剂、加热等方法进行消毒;

3.自吸过滤式呼吸器(全面型或半面型)和动力送风过滤式呼吸器的清洗参照说明书进行;

4.棉纱口罩可清洗消毒，其他非医用口罩按说明书处理。

（260）国办：转发卫健委、人社部、财政部关于改善一线医务人员工作条件切实关心医务人员身心健康若干措施	国办发〔2020〕4号

各省、自治区、直辖市人民政府，国务院各部委、各直属机构：

国家卫生健康委、人力资源社会保障部、财政部《关于改善一线医务人员工作条件切实关心医务人员身心健康的若干措施》已经国务院同意，现转发给你们，请认真贯彻执行。

国务院办公厅

2020年2月10日

（此件公开发布）

关于改善一线医务人员工作条件

切实关心医务人员身心健康的若干措施

国家卫生健康委　人力资源社会保障部　财政部

新冠肺炎疫情发生以来，广大医务人员积极响应党中央号召，不顾个人安危，迎难而上，英勇奋战在抗击疫情的最前线，为保护人民生命健康作出了重大贡献，用实际行动践行了“敬佑生命、救死扶伤、甘于奉献、大爱无疆”的崇高精神。当前，全国疫情防控进入关键时期，医务人员面临着工作任务重、感染风险高、工作和休息条件有限、心理压力大等困难。保护关爱医务人员是打赢疫情防控阻击战的重要保障，为改善一线医务人员工作条件，切实关心医务人员身心健康，使他们更好地投入疫情防控工作，现提出以下工作措施：

一、改善医务人员工作和休息条件

加强医疗卫生机构硬件设施改造，加强医务人员职业暴露的防护设施建设和设备配置，使收治病人的医疗卫生机构满足传染病诊疗和防控要求。要重点改造医生办公室、值班室和休息室，营造有利于医务人员工作的良好环境。要为医务人员提供良好后勤服务，保障医务人员充足的睡眠和饮食。县级以上地方人民政府可依法征用医院周边酒店作为医务人员休息场所，以满足一线医务人员单人单间休息条件，并对基本生活用品保证供应。

二、维护医务人员身心健康

（一）要合理安排医务人员作息时间。根据疫情防控实际，科学测算医务人员工作负荷，合理配置医务人员，既满足医疗服务需求，又保障医务人员休息时间。对于因执行疫情防控不能休假的医务人员，在防控任务结束后，由所在医疗卫生机构优先安排补休。允许需要紧急补充医护人员等疫情防控工作人员的相关医疗卫生机构简化招聘程序。

（二）加强医务人员个人防护，最大限度减少院内感染。要尽一切可能配齐防护物资和防护设备，防护用品调配要向临床一线倾斜。

（三）组织做好一线医务人员健康体检，发现医务人员感染及时隔离，尽最大可能减少医务人员之间、医务人员与病人之间交叉感染。

（四）加强医务人员心理危机干预和心理疏导。开展医务人员心理健康评估，强化心理援助措施，有针对性地开展干预和心理疏导，减轻医务人员心理压力。

三、落实医务人员待遇

（一）各地要按照《人力资源社会保障部 财政部关于建立传染病疫情防治人员临时性工作补助的通知》（人社部规〔2016〕4号）和《财政部 国家卫生健康委关于新型冠状病毒感染肺炎疫情防控有关经费保障政策的通知》（财社〔2020〕2号）有关要求，统计疫情防控一线医务人员和防疫工作者工作情况，由同级卫生健康部门会同人力资源社会保障、财政部门按月审核，报经国家卫生健康委审核并报人力资源社会保障部、财政部审定后，由同级财政部门在次月垫付临时性工作补助经费，中央财政据实结算。

（二）各级人力资源社会保障、财政部门要会同卫生健康部门，在同级政府领导下，根据实际情况，因地制宜及时向防控任务重、风险程度高的医疗卫生机构核增不纳入基数的一次性绩效工资总量，并指导有关单位搞好内部分配，向加班加点特别是作出突出贡献的一线人员倾斜。

（三）各地要落实好《人力资源社会保障部 财政部 国家卫生健康委关于因履行工作职责感染新型冠状病毒肺炎的医护及相关工作人员有关保障问题的通知》（人社部函〔2020〕11号），开通工伤认定绿色通道，切实保障好医务人员合法权益。

四、提高卫生防疫津贴标准

为进一步保障新冠肺炎疫情防疫人员权益，根据《国务院办公厅关于加强传染病防治人员安全防护的意见》（国办发〔2015〕1号），出台提高卫生防疫津贴标准的政策。各地要按照政策规定及时抓好落实，特别是对参与新冠肺炎疫情防疫人员，要及时足额发放到位。

五、加强对医务人员的人文关怀

各地要动员组织社会力量，发动志愿者或专门人员，对一线医务人员展开慰问，定期了解他们的需求和困难，建立台账，积极协调解决。加大对参与疫情防控工作医务人员的关怀力度，为一线医务人员和家属建立沟通联络渠道，尽量不安排双职工的医务人员同时到一线工作，对家有老人和孩子需要照顾的医务人员要尽可能创造条件使其兼顾家庭。要安排志愿者或专门人员对有家庭困难的一线医务人员家属进行对口帮扶。

六、创造更加安全的执业环境

严格落实国家卫生健康委、最高人民法院、最高人民检察院、公安部《关于做好新型冠状病毒肺炎疫情防控期间保障医务人员安全维护良好医疗秩序的通知》（国卫医函〔2020〕43号）的各项安全防范措施，加大警力投入，完善问责机制，对发现有歧视孤立一线医务人员及其家属行为的，要及时进行批评教育，情节严重的依法予以处理。对在疫情防控工作中伤害医务人员的，要坚决依法严肃查处，维护正常医疗卫生秩序。

七、弘扬职业精神做好先进表彰工作

利用多种形式加大对医务人员职业精神的宣传力度，深入挖掘宣传在抗击疫情工作中作出突出贡献的医务团队和个人，共同营造尊医重卫的良好氛围。将医务人员在重大自然灾害或突发公共卫生事件中的表现作为职称评审中医德医风考核的重要内容。可根据《事业单位工作人员奖励规定》开展及时奖励，并结合疫情防控工作进展，做好对作出突出贡献的医务团队和个人的及时性表彰工作，为做好疫情防控工作增强信心、凝聚力量。

各地卫生健康、人力资源社会保障和财政等相关部门要按照党中央、国务院决策部署，密切配合，全力做好各项工作，以高度负责的态度、务实到位的举措，切实改善一线医务人员工作条件，关心医务人员身心健康，保障医务人员权益，为坚决打赢疫情防控阻击战提供有力保障。

（261）国家医保局 财政部 税务总局关于阶段性减征职工基本医疗保险费的指导意见 （医保发〔2020〕6号）

各省、自治区、直辖市人民政府，新疆生产建设兵团：

为贯彻落实习近平总书记关于新冠肺炎疫情防控工作的重要指示精神，切实减轻企业负担，支持企业复工复产，根据社会保险法有关规定，经国务院同意，现就阶段性减征职工基本医疗保险（以下简称职工医保）单位缴费有关工作提出如下指导意见：

一、自2020年2月起，各省、自治区、直辖市及新疆生产建设兵团（以下统称省）可指导统筹地区根据基金运行情况和实际工作需要，在确保基金收支中长期平衡的前提下，对职工医保单位缴费部分实行减半征收，减征期限不超过5个月。

二、原则上，统筹基金累计结存可支付月数大于6个月的统筹地区，可实施减征；可支付月数小于6个月但确有必要减征的统筹地区，由各省指导统筹考虑安排。缓缴政策可继续执行，缓缴期限原则上不超过6个月，缓缴期间免收滞纳金。

三、各省要指导统筹地区持续完善经办管理服务，确保待遇支付，实施减征和缓缴不能影响参保人享受当期待遇。参保单位应依法履行代扣代缴个人缴费的义务，医保经办机构要做好个人权益记录，确保个人权益不受影响。优化办事流程，不增加参保单位事务性负担。

四、各省要指导统筹地区切实加强基金管理，做好统计监测，跟踪分析基金运行情况，采取切实管用的措施，管控制度运行风险，确保基金收支中长期平衡。减征产生的统筹基金收支缺口由统筹地区自行解决。各省可根据减征情况，合理调整2020年基金预算。

五、已经实施阶段性降低单位费率等援企政策的省可继续执行，也可按照本指导意见精神指导统筹地区调整政策。已实施阶段性降低职工医保单位费率的统筹地区，不得同时执行减半征收措施。

各省要提高思想认识，加强组织领导，分类指导统筹地区做好相关工作。决定实施减征政策的省，印发的具体实施方案于3月5日前报医保局、财政部、税务总局备案。各级医疗保障、财政、税务等部门要加强协同，切实履职，全力做好疫情防控期间的医疗保障各项工作，确保政策落实到位，重要情况及时报告。

国家医保局 财政部 税务总局

2020年2月21日

（262）民政部：做好因新冠肺炎疫情影响造成监护缺失的儿童救助保护工作	民电〔2020〕19号

各省、自治区、直辖市民政厅（局），各计划单列市民政局，新疆生产建设兵团民政局：

为深入学习贯彻习近平总书记关于新型冠状病毒肺炎疫情防控工作的重要指示精神，全面贯彻落实党中央、国务院关于分类指导做好疫情防控工作决策部署，按照全国民政系统疫情防控工作电视电话会议要求，现就加强因新冠肺炎疫情影响造成监护缺失的儿童救助保护工作通知如下：

一是及时发现报告。要指导各地儿童督导员、儿童主任在乡镇（街道）、村（居）民委员会统一部署下，及时发现因新冠肺炎疫情影响造成监护缺失的儿童（包括父母或其他监护人确认感染、疑似感染或需隔离观察，父母或其他监护人因防疫抗疫工作需要以及其他因疫情影响不能完全履行抚养和监护责任的儿童），并第一时间向所在村（居）民委员会、乡镇（街道）报告相关信息，及时协调解决问题或报告反映困难。

二是分类做好临时照料。要根据因疫情影响造成监护缺失的儿童自身和家庭状况，区分不同情形，分类做好临时照料服务。对与患者有密切接触史、身体状况不明的儿童，在由指定地点进行隔离医学观察或诊断期间，要跟进了解相关情况。对确认其非感染或已过观察期而监护责任未落实或一时难以落实的儿童，当地民政部门要协调乡镇（街道）、村（居）民委员会落实临时照料、提供关爱服务，或由民政部门承担临时监护责任。

三是加强救助保障。加强对因疫情影响造成监护缺失的儿童救助保障工作，指导各地及时将其纳入救助保障范围，对于符合条件的，及时落实临时救助等社会救助政策和困境儿童保障政策，确保生活兜底到位。疫情防控期间符合事实无人抚养儿童、孤儿或其他困境儿童申请条件的，要优化简化审核审批程序，探索通过远程方式提供相关证明，也可采取个人承诺方式先行保障，其他手续可待疫情结束后补办，并做到当月审批、当月发放。

四是开通救助保护热线。各地要依托地市级未成年人救助保护机构等方式开通因疫情影响造成监护缺失的儿童救助保护热线并向社会公布，及时响应有关线索，做好临时照料咨询、转介服务、个案跟踪、资源链接等工作。地市级未成年人救助保护机构要加强与县级未成年人救助保护机构的联动，延伸服务触角，提升服务水平，切实做好救助保护工作。

地方各级民政部门要依靠当地联防联控机制，充分发挥农村留守儿童关爱保护和困境儿童保障工作领导协调机制作用，加强部门间的沟通协作，保证信息通畅，确保紧急问题在第一时间得到处理，帮助因疫情影响造成监护缺失的儿童及其家庭顺利渡过难关。

民政部办公厅

2020年2月11日

（263）卫健委 住建部：印发《新型冠状病毒肺炎应急救治设施设计导则（试行）》	国卫办规划函﹝2020﹞111号

各省、自治区、直辖市及新疆生产建设兵团卫生健康委、住房城乡建设厅（委、局）：

为加强对收治新型冠状病毒肺炎患者救治设施的改造、新建工作的指导，现将《新型冠状病毒肺炎应急救治设施设计导则（试行）》印发你们，供参考。请各地结合工作实际，做好统筹规划，精准调配医疗资源，调整现有医疗设施功能，通过院区功能调整、设施改造等多种方式，全力保障疫情救治需要。

附件：新型冠状病毒肺炎应急救治设施设计导则（试行）

国家卫生健康委办公厅 住房和城乡建设部办公厅

2020年2月8日

（264）人社部：切实做好新型冠状病毒感染肺炎疫情防控期间技能人才评价有关工作	人社厅函〔2020〕22号

各省、自治区、直辖市及新疆生产建设兵团人力资源社会保障厅（局），国务院有关部委、直属机构及有关中央企业人事劳动保障工作机构，有关社会培训评价组织：

为深入贯彻落实习近平总书记关于坚决打赢新型冠状病毒感染肺炎疫情防控阻击战的决策部署，按照我部《关于进一步做好新型冠状病毒感染的肺炎疫情防控工作的通知》等有关要求，现就做好疫情防控期间技能人才评价工作有关事项通知如下：

一、各地人力资源社会保障部门要坚持把人民群众生命安全和身体健康放在第一位，把疫情防控工作作为当前最重要的工作来抓，坚决扛起疫情防控政治责任，加强对本行政区域内技能人才评价工作（含职业资格鉴定、职业技能等级认定、专项职业能力考核和职业技能竞赛）的组织协调和监督管理。

二、疫情防控期间，暂停开展集中考核评价活动，暂缓举办考务管理人员、考评人员、督导员等人员集中培训活动，并做好宣传解释工作。开展技能人才自主评价的企业，按照当地党委政府关于疫情防控有关要求，结合企业生产经营实际，采取线上理论考试、生产过程考核、工作业绩考评等方式，统筹组织开展；对在疫情防控一线表现突出的技能人员，可晋升职业技能等级。

三、严格落实疫情防控措施，减少非必须的现场办理。对考试报名及资格审核、评价结果查询、证书（含职业资格证书、职业技能等级证书、专项职业能力证书）补（换）发、证书数据信息更正等服务事项，通过“网上办”“电话办”“邮寄办”等方式，推行“不见面”服务，帮助群众足不出户办理业务。

四、各地人力资源社会保障部门要统筹做好技能人才评价工作。加大“放管服”改革力度，深化职业资格制度改革，全面推行职业技能等级制度。疫情防控期间，推广在线进行职业技能等级评价机构特别是自主评价用人单位备案工作，推动各类企业全面开展职业技能等级自主评价，指导社会培训评价组织有序开展职业技能等级认定。

人力资源社会保障部办公厅

2020年2月6日

（265）商务部：做好重点城市生活物资保供工作	商办建函[2020]32号

为贯彻落实党中央、国务院关于做好新型冠状病毒感染肺炎疫情防控工作的有关决策部署，在做好疫情防控工作的同时，科学有效保障人口密集、返城人员集中、疫情防控和保供压力大的重点城市生活物资供给，2月10日，商务部印发《关于做好重点城市生活物资保障工作的通知》（以下简称《通知》）。

《通知》要求，各地商务主管部门在当地党委、政府统一领导和部署下，坚持属地原则与联保联供并重、市场原则与政府保障并重、疫情防控与保障供应并重、抓重点与顾全面并重，通过摸清底数情况、强化预警监测、加强物资储备、强化调运配送、做好应急保障、维护市场秩序、动员各方力量等措施，围绕大米、面粉、食用油、肉类、鸡蛋、奶类、蔬菜、方便食品等重要生活物资，完成好重点城市保供任务。

2020年2月10日

（266）税务总局：充分发挥税收职能作用 助力打赢疫情防控阻击战	税总发〔2020〕14号

国家税务总局各省、自治区、直辖市和计划单列市税务局，局内各单位:

为深入贯彻习近平总书记关于新冠肺炎疫情防控工作的一系列重要指示批示精神，全面落实党中央、国务院决策部署，充分发挥税收职能作用，助力打赢疫情防控阻击战，促进经济社会平稳健康发展，现提出如下措施：

一、认真落实税收优惠政策，助力疫情防控和企业复产扩能

（一）不折不扣落实支持疫情防控的税收优惠政策。坚决扛牢落实支持疫情防控税收政策的政治责任，对2020年2月1日和2月6日新出台涉及“六税”“两费”的十二项政策以及地方在法定权限范围内出台的政策，及时优化调整信息系统，加大内部培训力度，简化办理操作程序，尽量采取网上线上方式向纳税人、缴费人开展政策宣传辅导，积极加强与发改、工信等部门沟通，确保政策简明易行好操作，让纳税人、缴费人及时全面懂政策、会申报，实现应享尽享、应享快享。对其他税收优惠政策特别是国家实施的更大规模减税降费政策措施也要进一步落实落细，巩固和拓展政策实施成效。

（二）编制支持疫情防控的税收优惠政策指引。税务总局编制发布《新冠肺炎疫情防控税收优惠政策指引》，便利纳税人、缴费人更好地了解掌握相关政策和征管规定。各级税务机关要对照政策指引逐项加大落实力度，确保全面精准落地。

（三）切实加强税收政策执行情况的监督评估。通过绩效考评和专项督查等方式，加强对支持疫情防控税收优惠政策执行情况的督促检查，严明纪律要求，确保政策执行不打折扣。加强政策运行情况的统计核算和跟踪分析，积极研究提出改进完善的意见建议。

二、深入拓展“非接触式”办税缴费，切实降低疫情传播风险

（四）明确网上办税缴费事项。税务总局梳理和发布涉税事项网上办理清单。各地税务机关要积极告知纳税人、缴费人凡是清单之内的事项均可足不出户、网上办理，不得自行要求纳税人、缴费人到办税服务厅或政务服务大厅办理清单列明的相关业务。

（五）拓展网上办税缴费范围。各地税务机关要按照“尽可能网上办”的原则，在税务总局发布清单的基础上，结合本地实际，积极拓展丰富网上办税缴费事项，实现更多业务从办税服务厅向网上转移，进一步提高网上办理率。

（六）优化网上办税缴费平台。加强电子税务局、手机APP等办税缴费平台的运行维护和应用管理，确保系统安全稳定。优化电子税务局与增值税发票综合服务平台对接的相关应用功能，进一步方便纳税人网上办理发票业务。拓展通过电子税务局移动端利用第三方支付渠道缴纳税费业务，为纳税人、缴费人提供更多的“掌上办税”便利。

（七）强化线上税费咨询服务。增强12366纳税服务热线咨询力量配备，确保接线通畅、解答准确、服务优质。制作疫情防控税收热点问题答疑，及时向纳税人、缴费人推送。积极借助12366纳税服务平台、主流直播平台等，通过视频、语音、文字等形式与纳税人、缴费人进行实时互动交流，及时回应社会关切。

（八）丰富多元化非接触办理方式。各地税务机关在拓展网上线上办税缴费服务的同时，要积极为纳税人、缴费人提供其他非接触式办税缴费渠道。不断拓宽“网上申领、邮寄配送”发票、无纸化方式申报出口退（免）税以及通过传真、邮寄、电子方式送达资料等业务范围，扩大非接触办税缴费覆盖面。

三、大力优化现场办税缴费服务，营造安全高效便捷的办理环境

（九）确保安全办理。严格做好办税缴费服务场所（包括自助办税终端区域）的体温检测、室内通风、卫生防疫、清洁消毒等工作，在做好一线工作人员安全防护的同时，主动为纳税人、缴费人提供纸巾、洗手液等基本防护用品。科学规划办税服务厅进出路线和功能区域设置，保持人员之间安全距离。积极争取当地卫生防疫部门的支持，出现紧急情况及时妥善处理。对办税缴费服务场所的安全防护措施，以适当方式明确告知纳税人、缴费人，确保安心放心办税缴费。

（十）加强引导办理。增强办税服务厅导税和咨询力量配置，严格落实首问责任制，进一步做好对纳税人、缴费人办税缴费的引导服务，最大限度提高办理效率、压缩办理时间，确保“放心进大厅、事情快捷办”。

（十一）开辟直通办理。对生产、销售和运输疫情防控重点保障物资的纳税人、缴费人，提供办税缴费绿色通道服务，第一时间为其办理税费事宜，全力支持疫情防控重点物资稳产保供。

（十二）拓展预约办理。全面梳理分析辖区内纳税人、缴费人办税缴费情况，主动问需，主动对接。对确需到办税服务厅办理业务的，主动提供预约服务，合理安排办理时间。办税服务厅每天要根据人员流量情况和业务紧急程度，及时加强与纳税人、缴费人的电话、微信联系沟通，提示其错峰办理，千方百计减少人员集聚。

（十三）推行容缺办理。对纳税人、缴费人到办税服务厅办理涉税事宜，提供的相关资料不齐全但不影响实质性审核的，经纳税人、缴费人作出书面补正承诺后，可暂缓提交纸质资料，按正常程序为其办理。

四、积极调整税收管理措施，帮助受疫情影响的企业纾困解难

（十四）依法延长申报纳税期限。在延长2月份申报纳税期限的基础上，对受疫情影响办理申报仍有困难的纳税人，可依法申请进一步延期。疫情严重地区，对缴纳车辆购置税等按次申报纳税的纳税人、扣缴义务人，因疫情原因不能按规定期限办理纳税申报的，可以延期办理。

（十五）依法办理延期缴纳税款。对受疫情影响生产经营发生严重困难的企业特别是小微企业，税务机关要依法及时核准其延期缴纳税款申请，积极帮助企业缓解资金压力。

（十六）切实保障发票供应。对生产和销售医疗救治设备、检测仪器、防护用品、消杀制剂、药品等疫情防控重点保障物资以及对此类物资提供运输服务的纳税人，申请增值税发票“增版”“增量”的，可暂按需调整其发票领用数量和最高开票限额，不需事前实地查验。除发生税收违法行为等情形外，不得因疫情期间纳税人生产经营情况发生变化而降低其增值税发票领用数量和最高开票限额。

（十七）优化税务执法方式。进一步落实“无风险不检查、无批准不进户、无违法不停票”的要求，坚持以案头分析为主，充分发挥大数据优势，深入推进“互联网＋监管”。在疫情防控期间，减少或推迟直接入户检查，对需要到纳税人生产经营所在地进行现场调查核实的事项，可经本级税务机关负责人确认，延至疫情得到控制或结束后办理；对确需在办税服务厅实名办税的人员，通过核验登记证件、身份证件等方式进行验证，暂不要求进行“刷脸”验证；对借疫情防控之机骗取税收优惠或虚开骗税等涉税违法行为，要坚决依法查处。

（十八）依法加强权益保障。对受疫情影响逾期申报或逾期报送相关资料的纳税人，免予行政处罚，相关记录不纳入纳税信用评价；对逾期未申报的纳税人，暂不按现行规定认定非正常户。对行政复议申请人因受疫情影响耽误法定申请期限的，申请期限自影响消除之日起继续计算；对不能参加行政复议听证等情形，税务机关依法中止审理，待疫情影响消除后及时恢复。

各级税务机关要以高度的思想自觉、政治自觉和行动自觉，深入学习贯彻习近平总书记关于疫情防控工作的一系列重要指示批示精神，坚决落实党中央、国务院的决策部署，按照税务总局的要求和地方党委、政府的安排，在切实加强自身防控的同时，充分发挥税务部门职能作用，不折不扣落实各项税收优惠政策，积极主动优化办税缴费服务，为坚决打赢疫情防控阻击战贡献税务力量。上述一些临时性调整的措施实施期限视疫情情况另行通知。在此期间，要加强对各项措施执行情况的监督检查，对落实不力造成不良影响的，严肃追究有关单位和人员的责任。各地工作中的经验做法和意见建议，要及时向税务总局报告。

国家税务总局

2020年2月10日

（267）自然资源部：做好疫情防控建设项目用地保障工作	自然资办函〔2020〕215号

各省、自治区、直辖市自然资源主管部门、新疆生产建设兵团自然资源主管部门，中央军委后勤保障部军事设施建设局，国家林业和草原局，中国地质调查局及部其他直属单位，各派出机构，部机关各司局：

为认真贯彻党中央、国务院关于新型冠状病毒感染肺炎疫情防控工作部署，坚决打赢疫情防控阻击战，现就做好疫情防控应急建设项目用地保障工作通知如下：

一、支持疫情防控建设项目先行使用土地

对于疫情防控急需使用的土地，可根据需要先行使用；对选址有特殊要求，确需占用永久基本农田和生态保护红线的，视作重大项目允许占用。使用期满不需转为永久性建设用地的，使用结束后恢复原状，交还原土地使用者，不再补办用地手续。需要转为永久性建设用地的，待疫情结束后及时完善用地手续。同时，要做好被占地单位和群众的补偿安置。

二、坚持节约集约用地

科学合理安排用地，坚持节约集约用地。严禁与防控疫情无关的项目搭便车违规用地。

三、保障疫情防控建设项目用地计划指标

对疫情防控建设项目，新增建设用地计划指标应保尽保，计划指标挂账使用。地方计划指标不足的，疫情结束后由省级自然资源主管部门汇总形成项目用地批准清单，由国家统一核销计划指标。

四、创新工作方式

各级自然资源主管部门在正常用地审批中，要根据防控疫情需要，通过开展网上联审等，创新工作方式。可采取影像比对等方式，暂时取消永久基本农田占用和补划实地踏勘论证等户外要求。要围绕落实好“六稳”工作部署，抓紧做好交通、能源、水利等重大项目用地组卷报批工作，持续做好用地保障。

自然资源部办公厅

2020年2月11日

（268）税务总局发布《新冠肺炎疫情防控税收优惠政策指引》

面对新型冠状病毒感染肺炎疫情防控的严峻形势，税务部门深入贯彻落实习近平总书记一系列重要指示精神，坚决执行党中央、国务院决策部署，全力参与疫情防控工作。

近日，财税部门联合发布系列公告，明确自2020年1月1日起实施一系列聚焦疫情防控关键领域和重点行业的税收优惠政策，助力打赢疫情防控阻击战。疫情就是命令、防控就是责任，确保国家支持新冠肺炎疫情防控税收优惠政策落实到位，让纳税人实实在在享受到相关税收优惠，为纳税人提供高效便捷安全的办税服务，是税务部门当前的首要任务。

为更好发挥税收支持疫情防控的职能作用，帮助纳税人准确掌握和及时适用各项税收政策，税务总局对新出台的支持疫情防控税收优惠政策进行了梳理，形成了本指引，共涉及支持防护救治、支持物资供应、鼓励公益捐赠、支持复工复产四个方面12项政策。

一、支持防护救治

1.取得政府规定标准的疫情防治临时性工作补助和奖金免征个人所得税；

2.个人取得单位发放的预防新型冠状病毒感染肺炎的医药防护用品等免征个人所得税。

二、支持物资供应

3.对疫情防控重点保障物资生产企业全额退还增值税增量留抵税额；

4.纳税人提供疫情防控重点保障物资运输收入免征增值税；

5.纳税人提供公共交通运输服务、生活服务及居民必需生活物资快递收派服务收入免征增值税；

6.对疫情防控重点物资生产企业扩大产能购置设备允许企业所得税税前一次性扣除；

7.对卫生健康主管部门组织进口的直接用于防控疫情物资免征关税。

三、鼓励公益捐赠

8.通过公益性社会组织或县级以上人民政府及其部门等国家机关捐赠应对疫情的现金和物品允许企业所得税或个人所得税税前全额扣除；

9.直接向承担疫情防治任务的医院捐赠应对疫情物品允许企业所得税或个人所得税税前全额扣除；

10.无偿捐赠应对疫情的货物免征增值税、消费税、城市维护建设税、教育费附加、地方教育附加；

11.扩大捐赠免税进口范围。

四、支持复工复产

12.受疫情影响较大的困难行业企业2020年度发生的亏损最长结转年限延长至8年。

2020.2.12

（269）发改委等3部门：发挥政府储备作用支持应对疫情紧缺物资增产增供	发改运行﹝2020﹞184号

各省、自治区、直辖市发展改革委、财政厅、工业和信息化厅：

党中央、国务院高度重视新型冠状病毒感染肺炎疫情防控工作，把疫情防控工作作为当前最重要的工作来抓。为贯彻落实习近平总书记关于加强疫情防控工作的重要指示和中央政治局常委会会议精神，根据中央应对新型冠状病毒感染肺炎疫情工作领导小组会议精神和国务院常务会议有关部署，全力保障医疗防护紧缺物资供应，迅速提高疫情防控保障能力，坚决打赢疫情防控阻击战，现就发挥政府储备作用支持应对疫情紧缺物资增产增供有关事项通知如下：

一、鼓励企业多措并举扩大重点医疗防护物资生产供应

支持生产《政府兜底采购收储的产品目录（第一批）》（详见附件，以下简称《产品目录》）所列疫情防控重点医疗物资的企业抓紧释放已有产能，进一步增加产量，尽快实现满负荷复工复产，支持有关企业通过技术改造、增添生产线（设备）迅速扩大产能。支持有条件的企业尽快实现转产，重点生产应对疫情急需的紧缺医疗物资。积极帮助企业解决资金、资质、生产场地、设备购置和原材料采购等实际困难，促进上下游生产良性循环，提高全产业链生产能力。指导企业科学改进生产工艺，提高产品技术水平，保证产品质量。

二、实施疫情防控重点医疗物资政府兜底采购收储

充分发挥中央政府储备作用，疫情防控期间，将《产品目录》中重点医疗防护物资品种增列储备物资目录，国家相关部门根据疫情防控需要，按现有收储制度和办法，对《产品目录》中企业多生产的重点医疗防护物资，全部由政府兜底采购收储。各地区要组织企业尽快增加紧缺的重点医疗防护物资生产，加强质量管理，严格执行产品标准，及时交付使用。收储物资由国家统一管理、统一调拨。根据疫情防控形势逐步完善《产品目录》，对目录实施动态管理，进一步加强公共卫生应急物资保障能力建设。

三、加强地方应急物资政府收储

各地区要认真研究应对疫情过程中暴露出来的公共卫生和物资储备短板，有针对性地建立健全相关制度，着力补短板强弱项。要进一步加强应急物资储备建设，创新体制机制，扩大收储范围，增加储备规模，特别是要增加应对公共卫生事件重要物资的政府储备规模，在项目立项建设、资金补贴、用地、用能、融资等方面给予政策支持。地方政府储备要与中央政府储备有机结合、互为补充，加强衔接联动，确保形成合力。

四、支持企业对扩大的产能适时转产

疫情得到有效控制后，鼓励相关企业对扩大的产能根据疫情防控需要和市场需求进行转产，推动产品生产由《产品目录》类型向市场需求量大的其他类型、标准或型号转变，在满足重点医疗物资需要的同时向满足工业或消费需要转变，促进产品结构调整，实现企业转型升级。对转型生产企业保留适当产能，鼓励地方政府相关部门研究出台在一定期限内予以相应支持的政策措施。

五、完善重点支持企业名单管理制度

为规范疫情紧缺物资收储管理，各省级政府相关部门按照《产品目录》，根据疫情防控需要，对疫情防控重点物资生产企业的物资收储实施名单制管理，企业名单由各地区审核汇总后报国家相关部门。中央企业可由相关行业主管部门或直接向国家相关部门提出申请。企业需按照核定产能满负荷生产，并按照国家统一要求安排产品调拨。各地区根据疫情防控需要和企业生产经营情况，对重点支持企业名单实施动态调整。国家相关部门要加强名单信息共享，并做好与有关金融机构等沟通衔接，研究出台支持政策。

六、依法加强产品质量和市场监管

国家相关职能部门要指导企业落实全过程合规的主体责任和严格出厂检验与放行要求，确保相关物资符合国家有关标准，满足疫情防控工作需要。凡收储的物资，必须严格按照国家有关标准进行检测认证。不符合国家有关标准的物资或劣质物资，一律不得收储。进一步加大对疫情防控重点物资生产、销售企业的督促检查，坚决维护公平公正的市场秩序，加强履约情况监督，对违法生产不合格产品和严重违约的企业，依法严肃查处。

各地区各相关部门要进一步提高政治站位，增强“四个意识”，坚定“四个自信”，做到“两个维护”，把思想认识统一到党中央、国务院关于疫情防控的决策部署上来，按照国务院联防联控机制工作要求，千方百计调动企业生产积极性，消除企业生产经营后顾之忧，加强统筹协调，全力提高重点紧缺医疗物资产能产量，坚决打赢疫情防控阻击战。

附件：政府兜底采购收储的产品目录（第一批）

国家发展改革委

财  政  部

工业和信息化部

2020年2月7日

政府兜底采购收储的产品目录（第一批）

1、医用防护服 GB19082-2003

2、N95医用级防护口罩 GB19083

3、医用外科口罩 YY0469-2011

4、医用一次性使用口罩

5、生产医用防护服、医用口罩等重要原材料

6、医用护目镜/防护面屏/负压防护头罩

7、医用隔离衣

8、新型冠状病毒核酸检测试剂盒

9、全自动红外体温检测仪

10、经卫生健康、药监部门依程序确认治疗有效的药品和疫苗（具体品目另发）

（270）发改委：疫情防控期间做好企业债券工作	发改办财金〔2020〕111号

各省、自治区、直辖市及计划单列市、新疆生产建设兵团发展改革委，各有关单位:

为贯彻落实习近平总书记重要指示和《中共中央关于加强党的领导、为打赢疫情防控阻击战提供坚强政治保证的通知》精神，深入落实中央应对疫情工作领导小组和国务院联防联控机制有关要求，进一步强化金融对疫情防控工作的支持，现就疫情防控期间企业债券有关工作通知如下。

一、多措并举支持疫情地区和疫情防控企业的债券融资需求

（一）支持企业债券募集资金用于疫情防控相关医疗服务、科研攻关、医药产品制造以及疫情防控基础设施建设等项目，在偿债保障措施完善的情况下，可适当放宽该类项目收益覆盖要求。申报阶段，支持企业债券资金用于处于前期阶段的该类项目建设，但应全面详尽披露最新的项目合法合规信息。

（二）允许企业债券募集资金用于偿还或置换前期因疫情防控工作产生的项目贷款。

（三）鼓励信用优良企业发行小微企业增信集合债券，为受疫情影响的中小微企业提供流动性支持。允许债券发行人使用不超过40%的债券资金用于补充营运资金，同时将委托贷款集中度的要求放宽为“对单个委贷对象发放的委贷资金累计余额不得超过5000万元且不得超过小微债募集资金总规模的10%”。

（四）对于自身资产质量优良、募投项目运营良好，但受疫情影响严重的企业，允许申请发行新的企业债券专项用于偿还2020年内即将到期的企业债券本金及利息。

二、最大限度简便疫情防控期间企业债券业务办理

（一）设立申报“绿色通道”。受疫情影响较大的企业以及疫情防控重点保障企业新申请企业债券的，企业可直接向我委报送申报材料，实行“即报即审”，安排专人对接、专项审核，并比照我委“加快和简化审核类”债券审核程序办理。

（二）实行非现场业务办理。全面支持发行人、主承销商等中介机构通过非现场办理企业债券的发行申请、意见反馈、批文领取、信息披露、发行前备案等业务，可灵活选择线上系统、传真、电子邮件、邮寄等方式向我委政务服务大厅报送材料，纸质材料待疫情结束后进行补充提供或领取。

（三）适当延长批文有效期。企业债券批复文件在2020年2月至6月期间到期的，相关批文有效期统一自动延长至2020年6月30日，并豁免发行人履行延期申请程序。

（四）优化发行环节管理。一是近期获准通过但尚未领取企业债券批复文件的，可在发行材料中充分披露是否发生重大期后事项及其影响后，使用我委政务信息公开的电子批复文件启动发行。二是近期拟启动债券发行工作的发行人，应提前与中央国债登记结算有限责任公司联系，鼓励错峰预约簿记建档时间，并尽可能减少发行现场人员。三是对于已经启动债券发行程序，但因受疫情影响未能在发行有效期内完成发行的，可申请适当放宽债券发行时限，或者发行人可按照有关规定履行程序后，灵活选择择期发行。

三、切实做好债券存续期信息披露和本息兑付工作

（一）持续做好信息披露。发行人受疫情影响预计难以按期披露2019年年报和2020年一季度报告的，应按照交易场所有关规定提前披露延期公告，并在公告中说明受疫情影响导致无法披露的详细情况、预计可披露时间以及基本经营和财务数据等信息。期间，如发生重大期后事项，发行人应及时按照交易场所规定进行公告。

（二）稳妥做好本息兑付。疫情期间涉及债券还本付息的，发行人应尽早安排偿债资金，确保顺利完成本息兑付。确需召开债券持有人会议的，鼓励以视频或电话会议等非现场方式召开，并可合理简化召集召开程序。

各级发展改革部门和各有关单位应自觉加强党的领导、提高政治站位，增强“四个意识”、坚定“四个自信”、做到“两个维护”，深刻认识做好新型冠状病毒感染肺炎疫情防控的重要性和紧迫性，始终把人民群众生命安全和身体健康放在第一位，坚定不移把党中央、国务院各项决策部署落到实处，扎实做好企业债券对疫情防控的支持与保障工作，共同打赢疫情防控阻击战。

国家发展改革委办公厅

2020年2月8日

（271）卫生健康委 中医药局：印发新型冠状病毒感染的肺炎诊疗方案（试行第五版） 国卫办医函〔2020〕103号

各省、自治区、直辖市及新疆生产建设兵团卫生健康委、中医药管理局：

为进一步做好新型冠状病毒感染的肺炎病例诊断和医疗救治工作，我们组织专家在对前期医疗救治工作进行分析、研判、总结的基础上，对诊疗方案进行修订，形成了《新型冠状病毒感染的肺炎诊疗方案（试行第五版）》，现印发给你们，请参照执行。各有关医疗机构要在医疗救治工作中积极发挥中医药作用，加强中西医结合，建立中西医联合会诊制度，促进医疗救治取得良好效果。

附件：新型冠状病毒感染的肺炎诊疗方案（试行第五版）

国家卫生健康委办公厅  国家中医药管理局办公室

2020年2月4日

（272）人社部等4部门：做好新型冠状病毒感染肺炎疫情防控期间稳定劳动关系支持企业复工复产	人社部发〔2020〕8号

各省、自治区、直辖市及新疆生产建设兵团人力资源社会保障厅（局）、总工会、企业联合会/企业家协会、工商联：

为贯彻落实党中央关于新型冠状病毒感染肺炎疫情防控工作的决策部署，积极发挥广大企业和职工在疫情防控中的重要作用，全力支持企业复工复产稳定劳动关系，动员广大职工凝心聚力共克时艰，现就做好疫情防控期间稳定劳动关系支持企业复工复产提出以下意见。

一、高度重视疫情对劳动关系领域带来的新挑战

近期，受新型冠状病毒感染肺炎疫情影响，劳动关系领域面临新情况新问题。部分行业企业面临较大的生产经营压力，劳动者面临待岗、失业、收入减少等风险，劳动关系不稳定性增加，劳动关系矛盾逐步凸显。当前，新型冠状病毒感染肺炎疫情防控正处于关键阶段，各级协调劳动关系三方要认真学习贯彻习近平总书记关于疫情防控工作的一系列重要指示精神，坚决贯彻落实党中央决策部署，高度重视当前特殊时期劳动关系运行中出现的突出问题，加强劳动关系风险监测和研判，引导企业与职工共担责任共渡难关。要充分发挥三方机制在保企业、保就业、保稳定中的独特作用，深入分析当前劳动关系形势，结合实际帮助企业制定复工复产的措施，联合各方力量共同行动，加大对特殊时期企业劳动关系处理的指导服务，确保劳动关系总体和谐稳定。

二、灵活处理疫情防控期间的劳动用工问题

（一）鼓励协商解决复工前的用工问题。对因受疫情影响职工不能按期到岗或企业不能开工生产的，要指导企业主动与职工沟通，有条件的企业可安排职工通过电话、网络等灵活的工作方式在家上班完成工作任务；对不具备远程办公条件的企业，与职工协商优先使用带薪年休假、企业自设福利假等各类假。要指导企业工会积极动员职工与企业同舟共济，在兼顾企业和劳动者双方合法权益的基础上，帮助企业尽可能减少受疫情影响带来的损失。

（二）鼓励灵活安排工作时间。在疫情防控期间，为减少人员聚集，要鼓励符合规定的复工企业实施灵活用工措施，与职工协商采取错时上下班、弹性上下班等方式灵活安排工作时间。对承担政府疫情防控保障任务需要紧急加班的企业，在保障劳动者身体健康和劳动安全的前提下，指导企业与工会和职工协商，可适当延长工作时间应对紧急生产任务，依法不受延长工作时间的限制。

（三）指导规范用工管理。在疫情防控期间，要指导企业全面了解职工被实施隔离措施或政府采取的紧急措施情况，要求企业不得在此期间解除受相关措施影响不能提供正常劳动职工的劳动合同或退回被派遣劳动者。对符合规定的复工企业，要指导企业提供必要的防疫保护和劳动保护措施，积极动员职工返岗。对不愿复工的职工，要指导企业工会及时宣讲疫情防控政策要求和企业复工的重要性，主动劝导职工及时返岗。对经劝导无效或以其他非正当理由拒绝返岗的，指导企业依法予以处理。鼓励企业积极探索稳定劳动关系的途径和方法，对采取相应措施后仍需要裁员的企业，要指导企业制定裁员方案，依法履行相关程序，妥善处理劳动关系，维护企业正常生产经营秩序。

三、协商处理疫情防控期间的工资待遇问题

（四）支持协商未返岗期间的工资待遇。在受疫情影响的延迟复工或未返岗期间，对用完各类休假仍不能提供正常劳动或其他不能提供正常劳动的职工，指导企业参照国家关于停工、停产期间工资支付相关规定与职工协商，在一个工资支付周期内的按照劳动合同规定的标准支付工资；超过一个工资支付周期的按有关规定发放生活费。

（五）支持困难企业协商工资待遇。对受疫情影响导致企业生产经营困难的，鼓励企业通过协商民主程序与职工协商采取调整薪酬、轮岗轮休、缩短工时等方式稳定工作岗位；对暂无工资支付能力的，要引导企业与工会或职工代表协商延期支付，帮助企业减轻资金周转压力。

（六）保障职工工资待遇权益。对因依法被隔离导致不能提供正常劳动的职工，要指导企业按正常劳动支付其工资；隔离期结束后，对仍需停止工作进行治疗的职工，按医疗期有关规定支付工资。对在春节假期延长假期间因疫情防控不能休假的职工，指导企业应先安排补休，对不能安排补休的，依法支付加班工资。

四、采取多种措施减轻企业负担

（七）帮助企业减少招聘成本。要加大线上招聘服务工作力度，打造线上春风行动，大力推广远程面试，提高招聘企业与劳动者“点对点”直接对接率。规范人力资源服务收费，坚决打击恶意哄抬劳动力价格行为。对受疫情影响缺工较大的企业或者承担政府保障任务企业，鼓励人力资源服务机构减免费用提供招聘服务。

（八）合理分担企业稳岗成本。用好失业保险稳岗返还政策，对受疫情影响不裁员或少裁员的中小微企业，可放宽裁员率标准，让更多企业受益。用好培训费补贴政策，对受疫情影响的企业，在确保防疫安全情况下，在停工期、恢复期组织职工参加各类线上或线下职业培训的，可按规定纳入补贴类培训范围。用好小微企业工会经费支持政策，对受疫情影响符合条件的小微企业工会经费全额返还。用好企业组织会费，对受疫情影响符合条件的困难企业实行一定比例的企业会费返还。用好工会防疫专项资金，加大对防疫一线职工的慰问，充分调动职工参与防控疫情的积极性。

（九）提供在线免费培训。指导企业积极组织开展职工在线免费培训，支持帮助受疫情影响企业特别是中小微企业开展职工技能培训和困难企业职工转岗培训，开放“中国职业培训在线”平台全部功能，免费提供培训教学资源。

五、统筹各方力量加大指导服务力度

（十）加强劳动用工指导服务。各级人力资源社会保障部门要及时研究和解决疫情防控期间劳动关系领域中的重大问题，主动回应社会关切，制定有针对性政策，准确解读政策，帮助企业解决发展中的困难。要做好协调劳动关系三方牵头工作，加强政策宣传和组织协调，发挥各方优势，形成工作合力。各级工会要做好团结、动员广大职工工作，积极发挥企业工会作用，为困难职工提供必要的帮扶救助和心理危机干预疏导。要引导职工关心企业的生存与发展，依法理性表达诉求。动员职工大力发扬劳动精神、劳模精神、工匠精神，为企业长远发展献计献策、贡献力量。各级企联和工商联组织要梳理评估企业的实际困难并积极向有关部门提出针对性帮扶支持政策建议和指导服务，要鼓励企业承担社会责任，通过技术创新等提高竞争力。要引导受疫情影响导致生产经营困难的企业，完善企业内部协商民主机制，畅通与职工对话渠道，通过多种方式稳定劳动关系和工作岗位。要引导企业关心关爱职工健康，帮助解决职工实际困难，切实保障职工权益。要充分发挥行业协会积极作用，通过减免租金等形式减轻企业经营负担，引导同行业或上下游企业互帮互助，抱团取暖。

（十一）主动化解劳动关系矛盾。要力争把风险隐患化解在萌芽状态，着力提升基层预防化解劳动争议能力，推动企业建立健全内部劳动争议协商解决机制。大力加强专业性劳动争议调解工作，创新仲裁办案方式，加强争议处理指导监督，发挥多元机制合力，大力推广“互联网+调解仲裁”，切实提高争议处理效能。进一步畅通举报投诉渠道，加大劳动保障监察执法力度，依法查处违法行为。

（十二）做好表彰先进典型工作。各级协调劳动关系三方要深入开展和谐劳动关系创建活动，主动宣传在防控疫情中真正实现有事好商量、遇事多商量、有难题共同解决的企业，要在和谐劳动关系创建活动评比、劳动模范评选、五一劳动奖章、奖状等荣誉授予中优先考虑疫情防控期间对稳定劳动关系作出突出贡献的企业和个人，激励引导广大企业家和职工在疫情防控工作中主动履职，担当作为。

各级协调劳动关系三方要切实增强“四个意识”，坚定“四个自信”，做到“两个维护”，深入贯彻落实党中央应对新型冠状病毒感染肺炎疫情工作的决策部署，把稳定劳动关系支持企业与职工共渡难关作为当前重要工作来抓，统筹处理好促进企业发展和维护职工权益的关系，充分发挥中国特色和谐劳动关系的制度优势，坚定信心、积极作为，为打赢疫情防控阻击战作出积极贡献。

人力资源社会保障部 中华全国总工会

中国企业联合会/中国企业家协会 中华全国工商业联合会

2020年2月7日

（273）网信办：做好个人信息保护利用大数据支撑联防联控工作

各省、自治区、直辖市网络安全和信息化委员会，中央和国家机关有关部委：

为做好新型冠状病毒感染肺炎疫情联防联控中的个人信息保护，积极利用包括个人信息在内的大数据支撑联防联控工作，经中央网络安全和信息化委员会同意，现将有关事项通知如下：

1.各地方各部门要高度重视个人信息保护工作，除国务院卫生健康部门依据《中华人民共和国网络安全法》《中华人民共和国传染病防治法》《突发公共卫生事件应急条例》授权的机构外，其他任何单位和个人不得以疫情防控、疾病防治为由，未经被收集者同意收集使用个人信息。法律、行政法规另有规定的，按其规定执行。

2.收集联防联控所必需的个人信息应参照国家标准《个人信息安全规范》，坚持最小范围原则，收集对象原则上限于确诊者、疑似者、密切接触者等重点人群，一般不针对特定地区的所有人群，防止形成对特定地域人群的事实上歧视。

3.为疫情防控、疾病防治收集的个人信息，不得用于其他用途。任何单位和个人未经被收集者同意，不得公开姓名、年龄、身份证号码、电话号码、家庭住址等个人信息，因联防联控工作需要，且经过脱敏处理的除外。

4.收集或掌握个人信息的机构要对个人信息的安全保护负责，采取严格的管理和技术防护措施，防止被窃取、被泄露。

5.鼓励有能力的企业在有关部门的指导下，积极利用大数据，分析预测确诊者、疑似者、密切接触者等重点人群的流动情况，为联防联控工作提供大数据支持。

6.任何组织和个人发现违规违法收集、使用、公开个人信息的行为，可以及时向网信、公安部门举报。网信部门要依据《中华人民共和国网络安全法》和相关规定，及时处置违规违法收集、使用、公开个人信息的行为，以及造成个人信息大量泄露的事件；涉及犯罪的公安机关要依法严厉打击。

中央网络安全和信息化委员会办公室

2020年2月4日

（274）卫生健康委 中医药局：印发新型冠状病毒肺炎诊疗方案（试行第五版 修正版）	国卫办医函[2020]117号

各省、自治区、直辖市及新疆生产建设兵团卫生健康委、中医药管理局：

根据全国新型冠状病毒肺炎医疗救治专家组建议，经研究，我们对《新型冠状病毒感染的肺炎诊疗方案（试行第五版）》中利巴韦林的用法用量进行了修正。现将修正后的诊疗方案印发给你们，请参照执行。

附件：新型冠状病毒肺炎诊疗方案（试行第五版 修正版）

国家卫生健康委办公厅　国家中医药管理局办公室

2020年2月8日

（275）卫生健康委 中医药局：印发新型冠状病毒感染的肺炎诊疗方案（试行第三版）	国卫办医函〔2020〕66号

各省、自治区、直辖市及计划单列市、新疆生产建设兵团卫生健康委、中医药管理部门：

为进一步指导全国科学规范做好新型冠状病毒感染的肺炎病例诊断和医疗救治工作，我们组织专家对诊疗方案进行修订，形成了《新型冠状病毒感染的肺炎诊疗方案（试行第三版）》。现印发给你们，请参照执行。

国家卫生健康委医政医管局联系人：杜青阳

联系电话：010-68792989

传  真：010-68791823

国家中医药管理局医政司联系人：王瑾

联系电话：010-59957686

传  真：010-599576845

附件：新型冠状病毒感染的肺炎诊疗方案(试行第三版)

国家卫生健康委办公厅 国家中医药管理局办公室

2020年1月22日

（276）卫生健康委 中医药局：印发新型冠状病毒感染的肺炎诊疗方案（试行第四版）	国卫办医函〔2020〕77号

各省、自治区、直辖市及新疆生产建设兵团卫生健康委、中医药管理部门：

为进一步做好新型冠状病毒感染的肺炎病例诊断和医疗救治工作，我们组织专家对诊疗方案进行修订，形成了《新型冠状病毒感染的肺炎诊疗方案（试行第四版）》。现印发给你们，请参照执行。各有关医疗机构要在医疗救治工作中积极发挥中医药作用，加强中西医结合，建立中西医联合会诊制度，促进医疗救治取得良好效果。

附件：新型冠状病毒感染的肺炎诊疗方案（试行第四版）

国家卫生健康委办公厅 国家中医药管理局办公室

2020年1月27日

（277）发改委：疫情防控期间采取支持性两部制电价政策 降低企业用电成本	发改办价格〔2020〕110号

国家电网有限公司、南方电网公司、内蒙古电力（集团）有限责任公司：

为贯彻落实习近平总书记关于坚决打赢疫情防控阻击战的重要指示精神和党中央、国务院决策部署，在疫情防控期间降低企业用电成本，支持企业共渡难关，现就采取支持性两部制电价政策有关事项通知如下。

一、对疫情防控期间暂不能正常开工、复工的企业，放宽容（需）量电价计费方式变更周期和减容（暂停）期限，电力用户即日可申请减容、暂停、减容恢复、暂停恢复。申请变更的用户不受“暂停用电不得小于15天”等条件限制，减免收取容（需）量电费。对于疫情发生以来停工、停产的企业，可适当追溯减免时间。

二、对因满足疫情防控需要扩大产能的企业，原选择按合同最大需量方式缴纳容（需）量电费的，实际最大用量不受合同最大需量限制，超过部分按实计取。

三、全力保障为疫情防控直接服务的新建、扩建医疗等场所用电需求，采取免收高可靠性供电费等措施，降低运行成本。

请你公司细化落实相关电价政策，主动向用户宣传告知，做好用户申请受理、办理减免等工作。

国家发展改革委办公厅

2020年2月7日

（278）发改委：积极应对疫情创新做好招投标工作保障经济平稳运行	发改电〔2020〕170号

各省、自治区、直辖市、新疆生产建设兵团发展改革委、招投标指导协调工作牵头部门、公共资源交易平台整合牵头部门，中国招标投标协会、全国公共资源交易平台、中国招标投标公共服务平台：

新型冠状病毒感染肺炎疫情爆发以来，在党中央领导下，各地区、各有关部门和单位按照疫情防控工作部署要求，及时采取暂停招标项目现场交易活动、推行网上办理、减少到场要求等措施，有效减少人员聚集，阻断疫情传播，保障人民群众生命安全和身体健康。为在做好疫情防控工作的同时，做好“六稳”工作，有序开展招投标等公共资源交易活动，保障经济平稳运行，现就有关事项通知如下：

一、坚决服从疫情防控和经济社会发展大局。各地招投标和公共资源交易平台工作要认真落实本地区关于疫情防控工作的统一部署要求，切实把人民群众生命安全和身体健康放在第一位。要切实保障疫情防控相关采购项目顺利进行，对于疫情防控急需的应急医疗设施、隔离设施等建设项目，符合《招标投标法》第六十六条规定的，可以不进行招标，由业主采用非招标方式采购，或者在招标时酌情缩短有关时限要求。要根据疫情防控形势变化、项目紧急程度和市场主体需求，及时动态调整工作安排，在确保安全的同时最大程度减轻对招投标等公共资源交易活动的影响。

二、切实避免对招投标等交易活动“一刀切”。疫情防控期间，各级各类公共资源交易平台要立足公共服务职能定位，充分发挥软硬件优势，积极与当地疾控部门建立联动机制，在做好防控工作的前提下，全力支持和保障招投标等各类公共资源交易活动。特别对于涉及保障城市运行必需、疫情防控必需、涉及重要国计民生以及企业生产经营急需的项目，要建立“绿色通道”服务机制，创新方式方法，确保依法规范、及时有序进行。

三、保证招标项目竞争度和投标质量。针对节后复工企业可能出现在岗人员不足、工作协同不便、人员流动受限的实际，引导招标人依法、合理设定招标文件发售、投标文件提交等时限，以便投标人做好投标准备；需购买纸质招标文件的，提供邮寄方式，不要求投标人到指定地点购买；需提交纸质投标文件的，允许邮寄提交。

四、加快推进招投标全流程电子化。《国务院办公厅转发国家发展改革委关于深化公共资源交易平台整合共享指导意见的通知》（国办函〔2019〕41号）对2020年公共资源电子化交易全面实施提出了明确目标。各地要狠抓目标落实，加强部门协调，着力消除全流程电子化的盲点、断点、堵点，尽快在各行业领域全面推广电子招投标，实现发布招标公告公示、下载招标文件、提交投标文件、开标、评标、异议澄清补正、合同签订、文件归档等全流程电子化，扭转电子和纸质招投标双轨并行的局面。

五、全面推行在线投标、开标。为有效降低现场投标、开标带来的人员聚集风险，同时降低企业交易成本，各地要依托电子招投标交易平台，加快部署在线投标、开标系统，制定明确时间表，年内实现所有依法必须进行招标的项目在线投标、开标。

六、积极推广电子评标和远程异地评标。各地要加快建设完善电子评标系统，应用信息化工具辅助评标，提高评标效率；完善远程异地评标系统及技术规范，尽早实现省内常态化运行，并为跨省运行创造条件，有效减少评标人员聚集，实现有限专家资源充分共享；积极应对远程异地评标等对传统属地监管模式带来的挑战，建立跨区域协同监管机制。疫情防控期间，采取随机抽取方式难以组建评标委员会的，可依法由招标人自行确定专家；确有需求的地方，可以在监管到位的前提下选择适当的招标项目探索开展基于网络协同的专家分散评标。

七、改进投标担保方式。在全面推行投标保证金线上缴退的同时，大力推广使用保函特别是电子保函替代现金保证金，实现在线提交、在线查核。鼓励调整纸质保函提交方式，建议招标人在开标前不强制要求提交纸质原件，由中标候选人在中标候选人公示前提交并在网上公示。鼓励招标人对简单小额项目不要求提供投标担保，对中小企业投标人免除投标担保，减轻企业负担。

八、支持社会力量参与提升招投标服务供给。为保障在线开标、远程异地评标，以及满足避免人员过分集中对开标评标场所的需求，各地要注重发挥社会力量积极性，增加电子招投标交易平台和开标评标场所的服务供给，允许招标人选择依法规范并符合行政监督要求的市场主体建设运营的电子招投标交易平台开展招标，在监管到位前提下探索允许在社会化交易场所开标评标。

九、大力推进招投标行政监督电子化。各地要积极适应招投标全流程电子化的新形势，加快行政监督方式手段的电子化、智慧化。按照政务信息化要求，依托电子招投标交易平台，加快部署行政监督窗口和监督工具，实现对电子招投标交易全程事中事后监管。

十、保障投标人和潜在投标人合法权益。对于疫情防控期间暂停开标评标活动的招标项目，有关部门、单位和公共资源交易中心要指导招标人通过发布招标文件澄清或修改公告等适当方式另行通知招投标活动时间。对招投标活动实行减少人员聚集等相关措施时，确保对所有投标人公平公正。要保证异议、投诉渠道畅通，不得借疫情防控之名实施排斥限制投标人等违法违规行为。

十一、切实尽责履行政府职能。各级发展改革委或者本级政府确定的其他牵头部门应当加强工作统筹，敢于担当负责，会同有关招投标行政监督部门，按照本地区疫情防控统一部署要求，认真做好招投标指导协调工作。要加强政策宣传解读，畅通电话、网络等咨询渠道，及时为市场主体释疑解惑，保障招投标活动积极稳妥有序开展。要按照国家发展改革委《关于加强投资项目远程审批服务 保障新型冠状病毒感染肺炎疫情防控期间项目办理工作的通知》（发改电〔2020〕66号）要求，实行招标方案审批核准的电子化、便利化。各地公共资源交易平台整合牵头部门要切实加强对本地区公共资源交易工作的指导协调，确保在疫情防控期间公共资源交易市场不乱、监管不断。

十二、加强招投标活动服务保障。各地公共资源交易中心在暂停提供现场交易服务期间，要通过电话、网络等方式进行业务咨询及办理，保障项目办理不断档；在提供现场交易服务时，要严格按要求落实人员登记、健康监测、环境消毒等防控措施；要优化和简并办事流程，推行见证证明、场所预约等服务事项网上办理，利用在线核验、邮寄、告知承诺等方式取消或者减少人员到场要求；确需现场递交文件资料的，要做到即交即走，减少人员聚集程度及滞留时间。中国招标投标公共服务平台和各省级招标公告公示发布媒介要充分利用电子招投标交易平台及移动互联网技术，为市场主体提供稳定、便捷的在线发布服务；对疫情防控相关招标项目的业务办理予以专门优先保障。全国公共资源交易平台要加强动态监测，密切跟踪各地公共资源交易平台运行情况。电子招投标交易平台开发运营单位要加强值守，快速响应，做好技术服务保障；便利CA数字证书办理程序，尽可能通过网络或邮寄开展原件审核、介质发放等工作。相关单位要加快推进CA数字证书网络共享，运用手机扫码等技术实现免插介质完成身份验证、签名盖章、加密解密等交易流程。

十三、充分发挥行业协会作用。中国招标投标协会等行业组织要充分发挥政府与市场的桥梁纽带作用，面向市场主体加强疫情防控相关招投标政策的宣传解读，引导行业自律，及时了解和反映行业运行状况、市场主体诉求，并积极建言献策。

各地区、各有关部门和单位要从贯彻党中央、国务院决策部署，努力实现今年经济社会发展目标的高度，充分认识当前形势下创新开展招投标工作的紧迫性和重要性，立足当前、着眼长远，积极探索、精准施策，既要解决当前突出问题，更要注重建立长效机制，加快提升招投标工作服务经济社会发展大局的质量和水平。各地创新做好招投标工作的典型经验做法、遇到的困难问题、意见建议，请及时报送国家发展改革委（法规司）。

特此通知。

国家发展改革委办公厅

2020年2月8日

（279）财政部等5部门：发布关于打赢疫情防控阻击战强化疫情防控重点保障企业资金支持的紧急通知	财金〔2020〕5号

各省、自治区、直辖市、计划单列市财政厅（局）、发展改革委、工业和信息化主管部门、审计厅（局），人民银行上海总部，各分行、营业管理部、各省会（首府）城市中心支行、副省级城市中心支行，新疆生产建设兵团财政局、发展改革委、工业和信息化局、审计局，财政部各地监管局，审计署各特派员办事处：

党中央、国务院高度重视新型冠状病毒感染肺炎疫情防控工作，把疫情防控工作作为当前最重要的工作来抓。为认真贯彻习近平总书记关于加强疫情防控工作的重要指示和中央政治局常委会会议精神，根据李克强总理主持召开中央应对疫情工作领导小组会议精神和国务院常务会议部署，全力保障疫情防控重要医用物资和生活必需品供应，坚决遏制疫情蔓延势头，坚决打赢疫情防控阻击战，现就强化疫情防控重点保障企业资金支持有关事项通知如下：

一、规范疫情防控重点保障企业名单管理

（一）支持范围。发展改革委、工业和信息化部对以下疫情防控重点保障企业实施名单制管理：

1. 生产应对疫情使用的医用防护服、隔离服、医用及具有防护作用的民用口罩、医用护目镜、新型冠状病毒检测试剂盒、负压救护车、消毒机、消杀用品、红外测温仪、智能监测检测系统和相关药品等重要医用物资企业；

2. 生产上述物资所需的重要原辅材料生产企业、重要设备制造企业和相关配套企业；

3. 生产重要生活必需品的骨干企业；

4. 重要医用物资收储企业；

5. 为应对疫情提供相关信息通信设备和服务系统的企业以及承担上述物资运输、销售任务的企业。

（二）名单申报流程。各省级发展改革、工业和信息化部门负责审核汇总本地区疫情防控重点保障企业名单，报发展改革委、工业和信息化部。中央企业可由相关行业主管部门或直接向发展改革委、工业和信息化部提出申请。发展改革委、工业和信息化部根据疫情防控物资调拨需要，研究确定全国疫情防控重点保障企业名单（以下简称全国性名单）。

湖北省和浙江省、广东省、河南省、湖南省、安徽省、重庆市、江西省、北京市、上海市等省份，可根据疫情防控工作需要，自主建立本地区的疫情防控重点保障企业名单（以下简称地方性名单），由省级发展改革、工业和信息化部门报发展改革委、工业和信息化部备案。

上述地区对疫情防控物资保障有重要作用的重点医用物资、生活必需品生产企业，未纳入名单前可按照急事急办、特事特办原则，先向金融机构申请信贷支持，在金融机构审核的同时，及时向省级发展改革、工业和信息化部门申请纳入名单。

（三）严格名单管理。发展改革委、工业和信息化部应按照中央应对疫情工作领导小组和国务院联防联控机制部署要求，严格报送名单程序和筛选标准，指导做好疫情防控重点保障企业名单报送工作，根据疫情防控需要和企业规范生产经营情况，对名单实施动态调整。

（四）加强信息共享。发展改革委、工业和信息化部与财政部、人民银行、审计署实时共享全国性和地方性名单信息。财政部、人民银行应实时将名单内企业获得财政贴息和优惠贷款情况反馈发展改革委、工业和信息化部、审计署。

二、通过专项再贷款支持金融机构加大信贷支持力度

（一）发放对象。人民银行向相关全国性银行和疫情防控重点地区地方法人银行发放专项再贷款，支持其向名单内企业提供优惠贷款。发放对象包括开发银行、进出口银行、农业发展银行、工商银行、农业银行、中国银行、建设银行、交通银行、邮政储蓄银行等9家全国性银行，以及疫情防控重点地区的部分地方法人银行。全国性银行重点向全国性名单内的企业发放贷款，地方法人银行向本地区地方性名单内企业发放贷款。

（二）利率和期限。每月专项再贷款发放利率为上月一年期贷款市场报价利率（LPR）减250基点。再贷款期限为1年。金融机构向相关企业提供优惠利率的信贷支持，贷款利率上限为贷款发放时最近一次公布的一年期LPR减100基点。

（三）发放方式。专项再贷款采取“先贷后借”的报销制。金融机构按照风险自担原则对名单内企业自主决策发放优惠贷款，按日报告贷款进度，定期向人民银行申领专项再贷款资金。

三、中央财政安排贴息资金支持降低企业融资成本

（一）贴息范围。对享受人民银行专项再贷款支持的企业，中央财政给予贴息支持。

（二）贴息标准和期限。在人民银行专项再贷款支持金融机构提供优惠利率信贷支持的基础上，中央财政按企业实际获得贷款利率的50%进行贴息。贴息期限不超过1年。

（三）贴息资金申请程序。地方企业向所在地财政部门申请贴息支持，由省级财政部门汇总本地区贴息申请并报送财政部。中央企业直接向财政部申请。财政部审核后，向省级财政部门和中央企业尽快拨付贴息资金。省级财政部门应尽快将贴息资金直接拨付地方企业。

四、切实加强应急保障资金监督管理

（一）确保专款专用。疫情防控重点保障企业要将金融机构提供的优惠信贷支持，全部用于疫情防控相关的生产经营活动，积极扩大产能、抓紧增产增供，服从国家统一调配，保障疫情防控相关重要医用物资、生活必需品平稳有序供给。对于挪用优惠信贷资金用于偿还企业其他债务，或投资、理财等套利活动，未从事疫情防控相关生产经营活动，或对生产的物资不服从国家统一调配的企业，一经发现，取消享受优惠政策支持资格，追回中央财政贴息和优惠信贷资金，并按照有关规定追究相应责任。地方不配合国家对重要物资统一调配的，取消当地企业的相关政策支持。

（二）加强监督管理。各级有关部门和中央企业要严格按照程序和筛选标准报送企业名单和融资需求。金融机构要从严审批、从快发放贷款，加强贷后管理，确保资金第一时间用于疫情防控相关生产经营活动。发展改革委、工业和信息化部要跟踪监督重点保障企业生产的医用物资、生活必需品流向，确保物资用于疫情防控的重要地区和领域。人民银行要建立电子台账，跟踪监督再贷款资金使用情况。财政部门要加强对中央财政贴息资金安排的监管、监督。审计部门要加强对重点保障企业贴息贷款的审计监督，促进资金使用的公开、公平、公正。疫情防控重点保障企业和相关金融机构要自觉接受财政、审计部门的检查监督。

（三）提高资金使用效益。各级财政部门要及时拨付贴息资金，加强资金使用全流程监管，强化绩效管理要求，确保贴息资金使用安全、合规和有效，并根据工作需要适时组织开展绩效评价。任何单位和个人不得以任何理由、任何形式截留、挪用贴息资金。

（四）严格责任追究。各地区各相关部门工作人员存在违反本通知要求滥用职权、玩忽职守、徇私舞弊等违法违纪行为的，企业借机骗取套取财政和信贷资金的，要依据《中华人民共和国监察法》、《中华人民共和国预算法》、《财政违法行为处罚处分条例》等法律法规追究相应责任、坚决严惩不贷；涉嫌犯罪的，依法移送司法机关处理。

五、强化责任担当，狠抓贯彻落实

（一）提高站位，加强领导。各地区各相关部门要进一步提高政治站位，增强“四个意识”、坚定“四个自信”、做到“两个维护”，深刻认识打赢疫情防控阻击战的重要性和紧迫性，坚决服从中央统一指挥、统一协调、统一调度，对疫情防控重点保障企业资金需求应保尽保，切实加强组织领导，抓好贯彻落实。

（二）明确责任，强化协同。各级发展改革、工业和信息化部门要主动了解疫情防控重点保障企业生产经营需求，下沉服务、上门服务，及时帮助企业排忧解难。各人民银行分支机构要指导金融机构主动对接疫情防控重点保障企业融资需求、尽快放贷，保障企业生产经营需要。各级财政部门、人民银行分支机构要简化申报流程、提高办理效率，尽快发放专项再贷款、拨付贴息资金。各级审计部门要加强资金跟踪审计，发现问题及时推动整改。各部门要加强联动、信息共享，形成工作合力，重大问题及时报告。

（三）特事特办，及早见效。各部门要切实强化责任担当，坚持特事特办、急事急办，业务办理高效化、便利化，全力以赴支持疫情防控重点保障企业开展生产经营、扩大生产能力，确保政策尽快落地见效，真正惠及疫情防控重点保障企业以及受疫情影响的人群、企业和地区。

财政部

发展改革委

工业和信息化部

人民银行

审计署

2020年2月7日

（280）财政部 教育部：切实做好学校疫情防控经费保障工作	财办教〔2020〕11号

各省、自治区、直辖市、计划单列市财政厅（局）、教育厅（局、教委），新疆生产建设兵团财政局、教育局：

　　为深入贯彻落实习近平总书记重要指示批示精神和党中央、国务院决策部署，全力支持打赢疫情防控阻击战，按照《财政部关于进一步做好新型冠状病毒感染肺炎疫情防控经费保障工作的通知》（财办〔2020〕7号）和《教育部关于切实做好新型冠状病毒感染的肺炎疫情防控工作的通知》（教电〔2020〕29号）有关要求，现就做好学校疫情防控经费保障工作通知如下：

　　一、研究制定经费保障政策措施

　　地方各级财政、教育部门要认真贯彻落实习近平总书记重要指示批示精神，按照党中央、国务院决策部署，高度重视学校疫情防控工作，切实增强做好学校疫情防控工作的政治责任感、使命感和紧迫感，全力做好学校疫情防控经费保障工作。地方各级财政部门要积极主动与教育部门沟通协商，密切关注疫情对学校教育教学和学习生活成本等的影响，及时研究制定学校疫情防控经费保障政策措施，支持学校确保不因资金问题而影响疫情防控。

　　二、统筹安排教育经费预算

　　地方各级财政、教育部门要统筹安排上级教育转移支付和自有财力，进一步优化教育支出结构，加大对疫情防控重点地区学校的支持力度，提升学校防控能力，保障学校正常运转。中央财政已提前下达的支持学前教育发展资金、城乡义务教育补助经费（公用经费补助部分）、改善普通高中学校办学条件补助资金、现代职业教育质量提升计划资金、支持地方高校改革发展资金等教育转移支付资金，由地方财政、教育部门及学校根据相关资金管理办法和学校疫情防控需要，安排用于学校疫情防控物资、设备采购等支出。省级财政部门在分配下达2020年中央和省级相关教育转移支付时，要向疫情防控重点市县倾斜。

　　三、加快财政教育资金拨付使用

　　各地财政部门要有序规范组织资金调度，按照“急事急办、特事特办”的原则，加快资金拨付使用，确保学校疫情防控资金及时到位，并会同教育部门强化资金使用监管。要按照《关于疫情防控采购便利化的通知》（财办库〔2020〕23号）要求，建立采购“绿色通道”，根据学校疫情防控需要提高采购时效，保证采购质量。

　　四、指导学校加强疫情防控经费保障

　　地方各级教育部门要指导学校结合实际研究制定疫情防控工作经费保障方案，研判资金需求，细化经费保障措施，统筹用好财政资金、学校自有资金和社会捐赠等各渠道资金，确保学校疫情防控工作顺利开展。

　　五、加强政策跟踪和分析研判

　　各级财政、教育部门要及时跟踪有关财政经费保障政策的落实情况，发现问题及时研究解决。省级财政、教育部门要密切关注疫情发展态势，分析研判延期开学对各级各类学校学生返校、教育教学、学生资助政策落实等工作的影响，及时制定预案。

　　经费保障工作中的重要情况和问题，应及时向财政部、教育部报告。

　　联系人及联系电话：财政部科教和文化司 高瑛泽 010-68551959；教育部财务司 王俊 010-66097557

财政部办公厅 教育部办公厅

2020年2月7日

（281）卫健委：进一步加强疫情期间医用防护服严格分级分区使用管理	国卫办医函〔2020〕118号

各省、自治区、直辖市及新疆生产建设兵团卫生健康委：

当前，新型冠状病毒肺炎疫情防控形势不断变化，医用防护服供需矛盾日益突出。为充分利用有限资源，指导医务人员正确做好个人防护，维护医务人员队伍的身体健康，现将有关要求通知如下：

一、高度重视医用防护服的合理使用

医用防护服是战胜新型冠状病毒肺炎疫情的重要保障物资之一。科学合理使用医用防护服，既能有效保护人员健康，又能保障医疗救治工作顺利开展。过度无序使用既浪费有限的医疗资源，又容易造成防护不当增加感染风险。各地要高度重视医用防护服使用管理工作，将其作为当前防疫工作的大事来抓。严格落实《新型冠状病毒感染的肺炎防控中常见医用防护用品使用范围指引（试行）》（国卫办医函〔2020〕75号）和《国家卫生健康委办公厅关于加强疫情期间医用防护用品管理工作的通知》（国卫办医函〔2020〕98号）等文件要求，保障临床合理防护，杜绝浪费。要实行一把手负责制，亲自调度、合理分配，按照“优先保障高风险区域、高风险操作、高风险人员”的原则，严格分级分区使用，加强管理，夯实责任，确保医疗救治工作中医用防护服合理使用。

二、加强医用防护服的分级分区使用管理

当前，通过国家有关部门通力协作，医用防护服的供应保障能力虽然得到一定提高，但是全国医用防护服仍整体供给不足。要严格医用防护服的使用范围，在隔离留观病区（房）、隔离病区（房）和隔离重症监护病区（房）使用，其他区域和在其他区域的诊疗操作原则上不使用。

无特殊情况，符合国标（GB19082）的一次性无菌医用防护服，以及在境外上市符合日标、美标、欧标等标准的一次性无菌医用防护服（所需证明材料包括：境外医疗器械上市许可证明和检测报告、无菌证明、企业做出质量安全承诺等），仅用于隔离重症监护病区（房）等有严格微生物指标控制的场所；隔离留观病区（房）、隔离病区（房）仅使用在境外上市符合日标、美标、欧标等标准的医用防护服（所需证明材料包括：境外医疗器械上市许可证明和检测报告、企业做出质量安全承诺等），以及符合《国务院应对新型冠状病毒感染的肺炎疫情联防联控机制物资保障组关于疫情期间防护服生产使用有关问题的通知》（工信明电〔2020〕7号）中规定的“紧急医用物资防护服”。

三、加强管理，促进合理使用医用防护服

医疗机构应当将医用防护服纳入全院统一管理，建立台账，根据医务人员工作所在不同区域、开展的不同操作及管理患者的症状轻重程度，科学合理分配医用防护服。要根据收治患者的实际情况，合理安排医务人员在隔离区域工作的班次，发挥资源利用最大效益。

国家卫生健康委办公厅

2020年2月8日

（282）工信部：应对新型冠状病毒肺炎疫情帮助中小企业复工复产共渡难关	工信明电〔2020〕14号

各省、自治区、直辖市及计划单列市、新疆生产建设兵团中小企业主管部门：

为深入贯彻落实习近平总书记关于坚决打赢疫情防控阻击战的重要指示精神和党中央、国务院关于在做好疫情防控工作同时统筹抓好“六稳”工作的有关决策部署，帮助广大中小企业坚定信心，强化措施，实现有序复工复产，渡过难关，现就有关事项通知如下：

一、全力保障企业有序复工复产

1.加强分类指导。各级中小企业主管部门要按照当地疫情防控总体要求，结合实际情况分类施策，在全力保障疫情防控必需、公共事业运行必需、群众生活必需等重点企业尽快复工复产的同时，积极稳妥地推动其他生产性企业完成复工复产准备工作，在疫情防控达标后有序复工复产。

2.推动落实复工复产措施。指导企业制订复工复产方案和应急预案，落实疫情防控主体责任和各项措施，做到防控机制到位、检疫查验到位、设施物资到位、内部管理到位和宣传教育到位，确保生产生活平稳有序。

3.强化复工复产要素保障。会同有关部门帮助企业协调解决职工返岗、原材料供应、物资运输以及口罩、消杀用品、测温仪等防控物资保障等难题，指导企业开展生产自救。推动有关单位对疫情期间中小企业生产经营所需的用电、用水、用气，实施阶段性缓缴费用，缓缴期间实行“欠费不停供”措施。加大企业复产用工保障力度，精准摸查发布企业用工需求信息，推进线上供求匹配对接和远程招聘，加强本地供需对接，挖掘本地供给潜力，满足企业阶段性用工需求。

4.发挥中小企业服务疫情防控的作用。对纳入疫情防控重点保障企业名单的中小企业，要配合做好相关保障工作。对有条件、有意愿转产防疫物资的中小企业，要“一企一策”，全力帮助协调解决转产过程中的问题。

二、进一步加强对中小企业的财政扶持

5.推动落实国家对防疫重点企业财税支持政策。协助纳入中央疫情防控重点保障企业名单的本地中小企业按政策规定申请贴息支持和税收优惠。湖北、浙江、广东、河南、湖南、安徽、重庆、江西、北京、上海等省（市）中小企业主管部门对纳入本地区疫情防控重点保障企业名单中的中小企业加强政策落实和服务。鼓励在中央贷款贴息的基础上，地方财政再予以进一步支持。

6.鼓励地方政府出台相关财政扶持政策。充分发挥本级中小企业发展专项资金的作用，有条件的地方可以设立专项纾困资金，加大对受疫情影响严重中小企业的支持。鼓励各地结合本地中小企业受疫情影响实际情况，依法依规减免税款和行政事业性收费，推动出台减免物业租金、阶段性缓缴或适当返还社会保险费、延期缴纳税款、降低生产要素成本、加大企业职工技能培训补贴和稳岗奖励等财政支持政策，切实减轻中小企业成本负担。已出台相关政策的地区，要加强部门协调，推动尽快落地见效。

7.推动加大政府采购和清欠工作的力度。引导各级预算单位加大对中小企业的倾斜力度，提高面向中小企业采购的金额和比例。加大行政机关、事业单位和国有企业拖欠中小企业账款清理力度，加快完成清欠目标任务，不得形成新增逾期拖欠。

三、进一步加强对中小企业的金融扶持

8.加大信贷支持力度。各地要主动加强与金融机构的对接，推动金融机构对有发展前景但受疫情影响暂遇困难的中小微企业，适当下调贷款利率，增加信用贷款和中长期贷款，不得盲目抽贷、断贷、压贷，对到期还款困难的，可予以展期或续贷。推广基于多维度大数据分析的新型征信模式，解决银企信息不对称问题，提高优质中小企业的信用评分和贷款可得性。发挥应急转贷资金作用，降低应急转贷费率，为受疫情影响较大的企业提供应急转贷资金支持。鼓励有条件的地方建立贷款风险补偿资金，对疫情期间金融机构向小微企业发放的贷款不良部分给予适当补偿。

9.强化融资担保服务。引导各级政府性融资担保、再担保机构提高业务办理效率，取消反担保要求，降低担保和再担保费率。对于确无还款能力的小微企业，为其提供融资担保服务的各级政府性融资担保机构应及时履行代偿义务，视疫情影响情况适当延长追偿时限，符合核销条件的，按规定核销代偿损失。

10.创新融资产品和服务。积极推动运用供应链金融、商业保理、应收账款抵质押、知识产权质押等融资方式扩大对中小企业的融资供给。充分发挥互联网金融便利快捷的优势，尽快开发疫情期间适合中小微企业的融资产品，满足中小企业需要。发挥各地中小企业融资服务平台作用，积极开展线上政银企对接。协调银行、保险机构开放信贷、保险理赔绿色通道，加快放贷速度和理赔进度。

11.加快推进股权投资及服务。积极发挥国家和地方中小企业发展基金协同联动效应，带动社会资本扩大对中小企业的股权融资规模，鼓励加大对受疫情影响暂时出现困难的创新型、成长型中小企业投资力度，加快投资进度。引导各类基金发挥自身平台和资源优势，加大对受疫情影响较大的被投企业投后服务力度，协调融资、人才、管理、技术等各类资源，帮助企业渡过难关。

四、进一步加强对中小企业的创新支持

12.组织开展疫情防控相关技术与产品创新。鼓励“专精特新”小巨人企业和“专精特新”中小企业针对新冠肺炎防治，在检测技术、药物疫苗、医疗器械、防护装备等方面开展技术攻关和生产创新，对取得重大突破的“专精特新”中小企业，在申报“专精特新”小巨人企业时予以优先考虑。即时启动2020年“创客中国”中小企业创新创业大赛“疫情防控”类参赛项目征集。率先征集诊断试剂、医疗器械、装备生产、药物疫苗、防护装备等创新项目，并做好技术完善、认证检测、资质申请和推广应用等服务工作。

13.支持企业数字化转型。大力推广面向中小企业的互联网平台服务，积极推行网上办公、视频会议、远程协作和数字化管理，以此为基础全面提升中小企业管理信息化水平。帮助提供线下服务的企业创新商业模式，拓展线上服务。加快5G、工业互联网应用部署，推广一批适合中小企业的工业软件应用，支持中小企业提升敏捷制造和精益生产能力。支持产业集群内中小企业以网络化协作弥补单个企业资源和能力不足，通过协同制造平台整合分散的制造能力，实现技术、产能与订单共享。

14.支持企业提升智能制造水平。引导大企业及专业服务机构面向中小企业推出云制造平台和云服务平台，发展适合中小企业智能制造需求的产品、解决方案和工具包。推动中小企业业务系统云化部署，对接工业互联网平台，引导有基础、有条件的中小企业加快生产线智能化改造，推动低成本、模块化的智能制造设备和系统在中小企业部署应用。

15.促进大中小企业融通创新发展。加快落实促进大中小企业融通发展三年行动计划。充分发挥国有企业和行业龙头企业的作用，带动产业链中小企业协同开展疫情防控、生产恢复与技术创新。帮助中小企业与供应链上下游企业沟通合作、抱团取暖，营造共荣发展、共克时艰的融通生态。

五、进一步加强对中小企业的公共服务

16.发挥中小企业公共服务平台作用。充分发挥国家和省级中小企业公共服务示范平台以及各地中小企业公共服务平台网络作用，为中小企业提供优质高效的线上服务。引导各地中小企业公共服务平台网络通过开设专栏等形式及时梳理各项惠企支持政策，开展中小企业疫情防控支持政策咨询解读等专项服务。鼓励国家和省级小型微型企业创业创新示范基地、享受过财政支持政策的创新创业特色载体等在疫情期间适当减免或延期收取中小企业的租金、物业管理和其他费用，支持企业创新发展。

17.加强培训服务。通过开展线上培训等形式，给中小企业送政策、送技术、送管理，为企业恢复正常生产经营做好各项准备工作。指导受疫情影响的企业在确保防疫安全情况下，在停工期、恢复期组织职工参加职业培训的，按规定纳入补贴类培训范围。

18.加强涉疫情相关法律服务。积极为中小企业提供法律援助和法律咨询公益服务，帮助中小企业解决受疫情影响造成的合同履行、劳资关系等法律问题。协助因疫情导致外贸订单无法如期履行或不能履行的中小企业申领不可抗力事实性证明，减少企业损失。对确因疫情影响无法正常履行相关义务的企业，协调不记入信用记录。

六、进一步加强统筹协调

19.发挥各级促进中小企业发展工作协调机制作用，提请召开领导小组会议专题研究部署，结合实际采取精准有效措施，减轻企业负担、降低生产成本、稳定人员就业、保障要素供给，帮助广大中小企业树立信心、减少损失、渡过难关，有序复工复产，切实保障经济平稳运行。

20.各级中小企业主管部门要切实履行职责，加强中小企业生产经营监测分析，及时发现并推动解决企业复工复产过程中遇到的突出问题。加强舆论宣传工作，引导中小企业坚定信心，共克时艰。加强部门协调，形成工作合力，共同推动国家及本地政府各项惠企政策落地，指导中小企业用好用足相关政策，扩大惠企政策受益面，提升企业实实在在地获得感。

各地要将落实有关工作进展情况及时上报工业和信息化部中小企业局。

工业和信息化部

2020年2月9日

（283）商务部：做好疫情防控期间生活物资对接调运保供有关工作

各省、自治区、直辖市、计划单列市及新疆生产建设兵团商务主管部门：

当前，保障物流通畅是做好疫情防控期间生活物资保供工作的重要前提。2月4日，全国防控物资暨春运错峰返程运输保障电视电话会议明确要求，要全力做好生活物资保障工作，切实保障好人民群众的正常生活。为加快解决疫情防控期间生活物资对接调运保供工作中存在的问题，现就有关事项通知如下：

一、加强运输保障政策宣传。交通运输部于近日下发《关于切实保障疫情防控应急物资运输车辆顺畅通行的紧急通知》（交运明电〔2020〕37号），提出了进一步简化通行证办理流程、将重要生活物资纳入应急运输保障范围等措施。公安部下发通知要求保障疫情防控应急运输车辆优先通行，为疫情防控应急运输车辆提供通行便利。各地商务主管部门要通过行业协会、新闻媒体、互联网等多种渠道和方式加强政策宣传解读，确保相关流通企业熟知有关便利通行政策。

二、指导流通企业严格按要求开展运输。各地商务主管部门要指导相关流通企业按要求办理《新型冠状病毒感染的肺炎疫情防控物资及人员运输车辆通行证》等手续，充分利用免费通行、绿色通道、“三不一优先”等便利政策，严格遵守卫生检疫、交通管控等要求，高效、合规做好生活物资运输工作，切实提高对接调运保供质量和效率。

三、帮助流通企业解决实际困难和问题。各地商务主管部门要深入调查研究，及时了解掌握生活物资对接调运保供中遇到的困难和存在的问题，积极帮助企业协调卫生健康、交通运输、公安等部门落实好政策，对生活物资对接调运保供中存在的共性问题及其他重要问题，要第一时间向商务部报告。

联系人及联系方式：商务部市场建设司 李琛

电话：010-85093705 传真：010-85093680

商务部办公厅

2020年2月6日

（284）国务院联防联控机制知：加强新型冠状病毒肺炎疫情防控期间孕产妇疾病救治与安全助产工作	肺炎机制发〔2020〕25号

各省、自治区、直辖市及新疆生产建设兵团应对新型冠状病毒肺炎疫情联防联控机制（领导小组、指挥部）：

为切实做好新型冠状病毒肺炎流行期间孕产妇和新生儿防护工作，保障母婴安全，现就有关工作提出以下要求。

一、加强孕产妇和新生儿安全管理

（一）积极指导孕产妇做好防护和孕产期保健。

1.各地应当指导助产机构加强院感防控，根据医院条件，为产科门诊及病房尽可能创造独立进出通道,落实产房、产科、母婴同室院感防控要求。

2.助产机构应当通过微信、APP、电话、视频、线上孕妇学校等方式加强对孕产妇健康教育和咨询指导，帮助孕产妇做好自我监测和居家防护。

3.根据孕产妇具体情况，必要时可适当调整产检时间。对有妊娠合并症、并发症等高危孕产妇，指导其按时接受产前检查，出现异常情况及时就医。

4.指导孕产妇正确识别和应对临产征兆，及时前往助产机构住院分娩。对原建档机构为新型冠状病毒肺炎救治定点医院的孕产妇，要及早作出合理安排；对其中邻近预产期的，要协调落实产检及分娩机构，并及时通知到人，确保衔接。

（二）严格做好发热孕产妇就诊管理。

1.各地应当在有条件的助产机构设置发热门诊，并及时向社会公布机构名单。

2.助产机构应当建立预检分诊制度，及时识别可疑病例，对发热孕产妇要由专人指引到发热门诊就诊。

3.发热门诊应当对发热孕产妇进行排查。对于能够明确排除疑似感染的孕产妇，可转至普通门诊就诊。对于疑似或确诊孕产妇，按照规定尽快转诊至定点医院。严禁让疑似或确诊孕产妇自行转诊。

4.助产机构应当严格落实母婴安全五项制度，将确诊孕产妇纳入“紫色”妊娠风险管理，综合评估孕产妇健康状况，落实高危孕产妇专案管理。

（三）切实保障疑似和确诊孕产妇产检和安全助产服务。

1.各地应当根据本辖区孕产妇数量、助产技术服务资源，指定一批综合救治能力较强的助产机构作为疑似或确诊孕产妇产检和住院分娩定点医院（以下简称“孕产妇定点医院”），优先保障疑似和确诊孕产妇的收治。孕产妇定点医院名单应当及时向社会公布。

2.孕产妇定点医院要切实为疑似和确诊孕产妇提供产检和安全助产服务。定点医院产科应当设置隔离病区或隔离病房，床位数量根据实际情况确定并及时动态调整，保障设施设备和人员配备，确保满足疑似和确诊孕产妇服务需求。

3.孕产妇在转诊至定点医院前产程已发动或急需终止妊娠来不及转运的，所在医疗机构应当做好隔离防护措施，在产妇分娩后及时评估产妇健康状况，确认安全后转运至定点医院落实后续治疗措施。

4.各地应当组建由产科、儿科、呼吸科、感染科等多学科参与的专家指导组，指导做好辖区内危重感染孕产妇救治工作。

（四）加强疑似和感染孕产妇产时管理和新生儿救治。

1.加强产儿科合作，对于疑似或确诊感染产妇分娩，产科应当至少提前30分钟通知新生儿科医生到场。

2.产妇为疑似病例、确诊病例和确诊后未痊愈者，暂停母乳喂养。

3.疑似或确诊感染产妇分娩的新生儿，经新生儿科评估一般情况良好的，转入新生儿隔离观察病区。

疑似感染产妇连续2次核酸检测阴性的，新生儿可转出隔离观察病区，实施母婴同室或居家护理；如母亲核酸检测阳性，新生儿隔离观察至少应当达到14天。

确诊感染产妇分娩的新生儿，应当在隔离观察病区观察至少14天。

4.疑似或确诊感染产妇分娩的新生儿如出现反应欠佳、呼吸困难、发热或有其他重症临床表现的，应当及时转入新生儿救治能力强的定点医院。

5.疑似或确诊感染产妇分娩的新生儿转运时，要严格按照《国家卫生健康委办公厅关于印发新型冠状病毒感染的肺炎病例转运工作方案的通知》（国卫办医函〔2020〕76号）要求执行。

二、工作要求

（一）各地要提高政治站位，把人民群众生命安全和身体健康放在第一位，对孕产妇和儿童等重点人群优先予以保障。坚持底线思维，强化风险意识，坚守母婴安全底线。

（二）各级卫生健康行政部门要结合实际，细化实化工作方案，有针对性地解决孕产妇和儿童健康面临的突出问题，确保各项措施落到实处。要统筹医疗资源，确保机构、床位、人员和物资到位，做好院感防控。

（三）各级卫生健康行政部门要做好组织协调，保障助产机构、定点机构、危急重症救治中心之间流程畅通、信息联通、转运有序。各级妇幼保健机构要充分履行职责，做好宣传教育、信息报告、技术支持、统筹协调和辖区管理等工作。

国务院应对新型冠状病毒肺炎疫情联防联控机制

2020年2月8日

（285）国务院应对新型冠状病毒感染的肺炎疫情联防联控机制：设立应对疫情心理援助热线	肺炎机制发〔2020〕18号

各省、自治区、直辖市应对新型冠状病毒感染的肺炎疫情联防联控机制（领导小组、指挥部）：

当前，防控新型冠状病毒感染的肺炎疫情正处于关键时期，31个省份启动了重大突发公共卫生事件一级响应，展开了疫情防控阻击战。为做好防控疫情的社会心理服务工作，向公众提供心理支持、心理疏导等服务，预防与减轻疫情所致的心理困顿，防范心理压力引发的极端事件，各地要在原有心理援助热线的基础上设立应对疫情心理援助热线（以下简称“心理热线”），现将有关要求通知如下：

一、省级或者地市级卫生健康行政部门要切实负起责任，统一组织协调当地心理热线，组建热线技术专家组，提供技术支持。要尽快评估卫生健康、教育、民政等部门、学会、协会等社会组织已开通的心理热线的服务能力，依托有条件的热线设立专席，开通疫情应对心理援助专线。每条热线至少开通2个坐席，结合本地公众需求提供24小时免费心理服务。地方政府应当对热线主办机构给予适当经费补助。

二、各地要通过电视、官方网站等多种媒体及时向社会公布心理热线电话号码，让群众广泛了解。有条件的地方应向电信部门申请开通热线电话短号码，方便群众记忆和拨打。

三、各地卫生健康行政部门要指导、协调热线主办机构尽快组建、充实热线工作团队，鼓励有心理咨询和心理危机干预经验的精神卫生、心理学专业人员、符合条件的社会心理服务志愿者，共同参与热线服务。各地要按照已下发的《新型冠状病毒感染的肺炎疫情紧急心理危机干预指导原则》（肺炎机制发〔2020〕8号，可在国家卫生健康委官网下载），针对不同人群的心理危机干预要点，对热线工作人员进行针对性的培训并进行技术支持和督导。

四、各地要加强心理热线管理，使用规范的热线服务流程，遵循心理热线服务伦理原则，定时分析汇总来电咨询的信息，了解和掌握公众关注的热点和各类来电人员的心理状态，做好评估和预判。发现突出问题或可能发生应激事件时，要及时将相关信息报告当地卫生健康行政部门。

国务院应对新型冠状病毒感染的

肺炎疫情联防联控机制

2020年2月2日

（286）国务院关税税则委员会：发布防控新型冠状病毒感染的肺炎疫情进口物资不实施对美加征关税措施	税委会〔2020〕6号

海关总署：

为积极支持新型冠状病毒感染的肺炎疫情防控工作，对按照防控新型冠状病毒感染的肺炎疫情进口物资免税政策（详见财政部 海关总署 税务总局公告2020年第6号，以下称免税政策）进口且原产于美国的物资，不实施对美加征关税措施，即恢复我对美232措施所中止的关税减让义务、不加征我为反制美301措施所加征的关税；已加征税款予以退还。

上述不实施加征关税措施与免税政策实施时间保持一致。

特此通知。

国务院关税税则委员会

2020年2月1日

（287）市场监管总局：发布关于新型冠状病毒感染肺炎疫情防控期间查处哄抬价格违法行为的指导意见	国市监竞争﹝2020﹞21号

各省、自治区、直辖市及新疆生产建设兵团市场监督管理局（厅、委）：

为确保新型冠状病毒感染肺炎疫情（以下简称疫情）防控期间口罩、抗病毒药品、消毒杀菌用品、相关医疗器械等防疫用品以及与群众日常生活相关的粮油肉蛋菜奶等基本民生商品市场价格秩序稳定，强化和规范各级市场监管部门查处哄抬价格违法行为，依照《价格法》《价格违法行为行政处罚规定》等法律法规，现提出如下指导意见：

一、经营者不得捏造、散布防疫用品、民生商品涨价信息。经营者有捏造或者散布的任意一项行为，即可认定构成《价格违法行为行政处罚规定》第六条第（一）项所规定的哄抬价格违法行为。

二、经营者存在以下情形的，可以认定为捏造涨价信息。

（一）虚构购进成本的；

（二）虚构本地区货源紧张或者市场需求激增的；

（三）虚构其他经营者已经或者准备提价的；

（四）虚构可能推高防疫用品、民生商品价格预期的其他信息的。

三、经营者存在以下情形的，可以认定为散布涨价信息。

（一）散布捏造的涨价信息的；

（二）散布的信息虽不属于捏造信息，但使用“严重缺货”“即将全线提价”等紧迫性用语或者诱导性用语，推高价格预期的；

（三）散布言论，号召或者诱导其他经营者提高价格的；

（四）散布可能推高防疫用品、民生商品价格预期的其他信息的。

四、经营者有以下情形之一，可以认定构成《价格违法行为行政处罚规定》第六条第（二）项所规定的哄抬价格违法行为。

（一）生产防疫用品及防疫用品原材料的经营者，不及时将已生产的产品投放市场，经市场监管部门告诫仍继续囤积的；

（二）批发环节经营者，不及时将防疫用品、民生商品流转至消费终端，经市场监管部门告诫仍继续囤积的；

（三）零售环节经营者除为保持经营连续性保留必要库存外，不及时将相关商品对外销售，经市场监管部门告诫仍继续囤积的。

生产环节、批发环节经营者能够证明其出现本条第（一）项、第（二）项情形，属于按照政府或者政府有关部门要求，为防疫需要进行物资储备或者计划调拨的，不构成哄抬价格违法行为。

对于零售领域经营者，市场监管部门已经通过公告、发放提醒告诫书等形式，统一向经营者告诫不得非法囤积的，视为已依法履行告诫程序，可以不再进行告诫，直接认定具有囤积行为的经营者构成哄抬价格违法行为。

五、经营者出现下列情形之一，可以认定构成《价格违法行为行政处罚规定》第六条第（三）项所规定的哄抬价格违法行为。

（一）在销售防疫用品过程中，强制搭售其他商品，变相提高防疫用品价格的；

（二）未提高防疫用品或者民生商品价格，但大幅度提高配送费用或者收取其他费用的；

（三）经营者销售同品种商品，超过1月19日前（含当日，下同）最后一次实际交易的进销差价率的；

（四）疫情发生前未实际销售，或者1月19日前实际交易情况无法查证的，经营者在购进成本基础上大幅提高价格对外销售，经市场监管部门告诫，仍不立即改正的。

经营者有本条第（三）项情形，未造成实际危害后果，经市场监管部门告诫立即改正的，可以依法从轻、减轻或者免予处罚。

本条第（四）项“大幅度提高”，由市场监管部门综合考虑经营者的实际经营状况、主观恶性和违法行为社会危害程度等因素，在案件查办过程中结合实际具体认定。

六、出现下列情形，对于哄抬价格违法行为，市场监管部门可以按无违法所得论处。

（一）无合法销售或者收费票据的；

（二）隐匿、销毁销售或者收费票据的；

（三）隐瞒销售或收费票据数量、账簿与票据金额不符导致计算违法所得金额无依据的；

（四）实际成交金额过低但违法行为情节恶劣的；

（五）其他违法所得无法准确核定的情形。

七、出现下列情形，对于无违法所得或者视为无违法所得的哄抬价格违法行为，市场监管部门应当依据《价格违法行为行政处罚规定》第六条规定的情节较重或者情节严重的罚则进行处罚；经营者违法所得能够明确计算的，应当依法从重处罚。

（一）捏造或者散布疫情扩散、防治方面的虚假信息，引发群众恐慌，进而推高价格预期的；

（二）同时使用多种手段哄抬价格的；

（三）哄抬价格行为持续时间长、影响范围广的；

（四）哄抬价格之外还有其他价格违法行为的；

（五）疫情防控期间，有两次以上哄抬价格违法行为的；

（六）隐匿、毁损相关证据材料或者提供虚假资料的；

（七）拒不配合依法开展的价格监督检查的；

（八）其他应当被认定为情节较重或者情节严重的情形。

八、经营者违反省级人民政府依法实施的价格干预措施关于限定差价率、利润率或者限价相关规定的，构成不执行价格干预措施的违法行为，不按哄抬价格违法行为进行查处。

九、市场监管部门发现经营者哄抬价格违法行为构成犯罪的，应当依法移送公安机关。

十、各省 、自治区、直辖市市场监管部门可根据本意见，报经省级人民政府同意，出台认定哄抬价格违法行为的具体标准以及依法简化相关执法程序的细化措施，并向市场监管总局（价监竞争局）备案。在本意见出台前，省级市场监管部门或者其他有关部门经省级人民政府同意，已经就认定哄抬价格违法行为作出具体规定的，继续执行。

十一、国家有关部门宣布疫情结束之日起，本意见自动停止实施。

市场监管总局

2020年2月1日

（288）国办：做好公路交通保通保畅工作 确保人员车辆正常通行

日前，国务院办公厅印发《关于做好公路交通保通保畅工作 确保人员车辆正常通行的通知》（以下简称《通知》），就进一步做好公路交通保通保畅工作，确保人员车辆正常通行，切实维护经济社会正常秩序作出专门部署。

一要全力保障公路路网顺畅运行。各地区、各有关部门要按照“科学有序、依法依规”原则，调整优化公路交通管控措施，严禁擅自封闭高速公路出入口，严禁阻断国省干线公路，严禁硬隔离或挖断农村公路，严禁阻碍应急运输车辆通行，严禁擅自在高速公路服务区和收费站、省界和国省干线公路设置疫情防控检疫点或检测站，已违法违规设置的要坚决撤销。要加强交通疏导，防止发生长时间、长距离公路交通拥堵，确保应急车道畅通。

二要有序恢复公路运输服务。湖北省特别是武汉市在城市公共交通（含轨道交通）、城际公交暂停运营期间，要统筹调度公交车、出租车（含网络预约出租车）运力，开展“点对点”服务，为医生、公共服务人员、老弱病残孕幼等各类重点人员出行提供必要保障。湖北省以外地区要结合复工开学安排，有序恢复城际客运、城市公共交通、出租车等公路运输服务，采取有力措施保障运力供给和乘车安全，统筹做好错峰出行，满足群众正常出行需求。要积极创造条件，通过公路直达运输等服务方式，保障农民工、学生等群体出行。疫情防控期间，对地方政府组织的农民工返岗包车免收高速公路通行费。

三要切实做好应急物资运输。各地区、各有关部门要突出重点，优先保障湖北省特别是武汉市的各类应急运输，优先保障疫情防控物资、生活必需物资运输。对持有“疫情防控应急物资及人员运输车辆通行证”的车辆，要严格执行应急运输绿色通道政策。对于短期向疫情重点区域运送物资的司机、装卸工等提供保障的人员，原则上不需采取隔离14天的措施。

《通知》强调，各地区要严格落实属地责任，全面系统摸排辖区内公路交通基础设施、运输市场情况，采取有力有效措施，解决保通保畅工作中存在的问题，确保人员车辆正常通行和重点防疫物资运输畅通。对擅自阻断公路交通基础设施、干扰公路运输正常秩序的，要严肃追究有关部门和单位的责任。

（新华社北京2月8日电）

（289）商务部 发改委：保障流通企业防护用品需要 做好市场保供工作

各省、自治区、直辖市、计划单列市及新疆生产建设兵团商务主管部门、发展改革委：

中央高度重视应对新型冠状病毒感染肺炎疫情工作中生活必需品供应，多次作出重要部署。流通企业（主要包括农产品批发市场、商场、超市、菜市场、农产品仓储企业、物流运输企业等）是保障生活必需品供应、促进价格稳定的重要载体，但也存在口罩、消毒液等防护用品短缺问题，影响流通企业正常营业和保障市场供应。为做好流通企业防护用品保障，促进生活必需品供应，现将有关事项通知如下：

一、做好物资核定和保障工作。各地商务主管部门要确定本地区发挥保供作用的骨干流通企业名单，实行目录名单管理。要摸清一线员工人数，核定口罩、消毒液等防护用品需求数量，及时向当地政府报告，协调相关部门予以优先保障。

二、切实发挥防护用品作用。各地商务主管部门要督促流通企业把防护用品主要用在保障市场供应上，口罩优先分配给一线员工，保障一线员工防疫安全，消毒液主要用在对外经营场所，指导流通企业认真做好营业场所及设施设备的消毒和清洁卫生工作。

三、多渠道解决防护物资保障。在把重点企业防护物资列入优先保障范围的同时，各地商务主管部门要积极组织企业拓宽防护用品保障渠道，通过扩大进口、捐赠、调剂等各种方式，千方百计保障发挥保供作用的骨干流通企业防护物资需要。

四、压实企业主体责任。流通企业是经营场所疫情防范工作的主体，要按照当地疫情防控工作主管部门有关要求，切实承担起疫情防控责任，把防控各项措施做细、做实、做到位。

商务部办公厅 国家发展改革委办公厅

2020年2月6日

（290）教育部：在疫情防控期间做好普通高等学校在线教学组织与管理工作	教高厅〔2020〕2号

各省、自治区、直辖市教育厅（教委），新疆生产建设兵团教育局，有关部门（单位）教育司（局），部属各高等学校、部省合建各高等学校，各教育部高等学校教学指导委员会、有关在线课程平台单位：

　　为贯彻落实习近平总书记关于打赢疫情防控阻击战的重要指示精神，针对新型冠状病毒感染肺炎疫情对高校的正常开学和课堂教学造成的影响，根据《教育部应对疫情工作领导小组工作方案（试行）》要求，现就在疫情防控期间高等学校在线教学组织与管理提出如下指导意见。

　　一、总体要求

　　采取政府主导、高校主体、社会参与的方式，共同实施并保障高校在疫情防控期间的在线教学。各高校应充分利用上线的慕课和省、校两级优质在线课程教学资源，在慕课平台和实验资源平台服务支持带动下，依托各级各类在线课程平台、校内网络学习空间等，积极开展线上授课和线上学习等在线教学活动，保证疫情防控期间教学进度和教学质量，实现“停课不停教、停课不停学”。

　　二、工作任务

　　1.面向全国高校免费开放全部优质在线课程和虚拟仿真实验教学资源。截至2020年2月2日，教育部组织了22个在线课程平台制定了多样化在线教学解决方案，免费开放包括1291门国家精品在线开放课程和401门国家虚拟仿真实验课程在内的在线课程2.4万余门，覆盖了本科12个学科门类、专科高职18个专业大类，供高校选择使用（见附件）。

　　2.立即制定在线教学组织与实施方案。针对疫情防控需要，高校要合理调整、统筹安排春季学期与秋季学期课程教学计划。在当前疫情防控期间，要暂停所有寒假社会实践，原计划进行的寒假社会实践推移到下一年度或下一学期内进行，可视疫情发展情况酌情减免寒假社会实践学分。根据校情学情制定疫情防控期间在线教学实施方案，充分利用线上教学优势，以信息技术与教育教学深度融合的教与学改革创新，推进学习方式变革，提高教学效率、保证教学质量、完成教学任务。

　　3.保证在线学习与线下课堂教学质量实质等效。高校要以文件、校园网公告等方式公布课程资源质量要求、在线教学课堂纪律和考试纪律要求、学生学习评价措施等管理措施；要引导教师择优选用适合的慕课、专属在线课程（SPOC）以及校内在线课程资源，应用公共课程服务平台、校内智慧教学系统和网络学习空间以及数字化教学软件等方式，开展线上教学、组织线上讨论、答疑辅导等教学活动，布置在线作业，进行在线测验等学习考核；要与课程平台建立教学质量保障联动机制，充分利用学习行为分析数据，了解学生在线学习情况。鼓励和支持有条件的高校，充分发挥各专业“虚拟教研室”的组织载体作用，加快研发一批有特色、代表性强、数量充足的在线试题，服务学生在线学习，提高学生学习积极性和课程挑战性。

　　4.发挥“国家精品在线开放课程”示范引领作用。“国家精品在线开放课程”的课程负责人和团队要上线提供全程教学服务，发挥示范引领作用，带动全国慕课教师团队开展线上教学服务。

　　5.开放国家虚拟仿真实验教学项目共享平台服务。国家虚拟仿真实验教学项目共享平台（实验空间）全天候开放，免费提供2000余门虚拟仿真实验课程资源，并提供在线实验教学支撑和教学考核管理。

　　6.倡导社会力量举办的在线课程平台免费提供优质课程资源和技术支持服务。倡导课程平台以及更多在线教育机构面向全国高校和社会公众免费开放优质在线课程。在线课程平台要有组织地与地方教育行政部门、疫情严重地区高校乃至更大范围高校建立联系，了解高校情况和教学需求，结合平台课程资源特点和技术优势，为高校制定丰富多样的在线教学解决方案；要为教师提供教学平台及软件支持服务，支持教师利用慕课等在线教学资源自主开展在线教学；鼓励开展网上在线教学培训，帮助广大教师适应新型教学环境、掌握在线教学技能、提高在线教学效果。

　　7.加强对高校选择在线课程平台教学解决方案的支持服务。各级教育行政部门要继续组织在线教育机构研发多样化在线教学解决方案，及时向高校提供解决方案及联系方式，保障教学需求和技术服务支持对接畅通，为高校选择资源和技术服务提供便利。

　　8.发挥专家组织指导、整合、协调作用。各教育部高等学校教学指导委员会要充分发挥教学指导作用，整合名校名师名企力量，推动快速上线一批前期有工作基础的优质慕课和实验课程，丰富线上教学资源。慕课联盟联席会要充分发挥专业性、区域性、跨区域性纽带作用，团结慕课联盟单位，通过专家工作组指导等方式，与高校有组织地开展以慕课主讲教师为主的线上教学、“慕课主讲教师+慕课课程工作组”为主的跨校协同教学、“慕课主讲教师+本地教师”的协作式跨校教学、借助慕课作为参考课支持本校教师校内在线授课教学等多种形式协同教学，指导高校选好课、用好课、讲好课。

　　9.加强疫情防控知识宣传。鼓励慕课平台开设有关流行病学、传染病学的慕课专题，国家虚拟仿真实验教学项目共享平台（实验空间）开通公共卫生与预防医学虚拟仿真实验专题等针对性实验，供全国大学生及社会公众了解相关知识与政策，提高科学防控能力。

　　三、工作保障

　　1.加强组织领导。中央有关部门（单位）教育司（局）、省级教育行政部门结合所属高校实际，帮助高校制定在线教学组织与管理方案，整合公共服务平台和省级课程平台资源与服务信息，指导所属高校在线教学组织与管理。

　　2.强化政策激励与引导。高校要将慕课教师以及承担教学任务的所有任课教师线上教学计入教学工作量，激励广大教师积极投身教学改革与创新，积累实战经验，产生更多更好教学成果。贯彻落实有关政策要求，引导学生在疫情防控期间积极选修线上优质课程，增加学生自主学习时间，强化在线学习过程和多元考核评价的质量要求。制定在线课程学习学分互认与转化政策，保障学生学业不受疫情影响。

　　3.确保在线教学安全平稳运行。高校要与课程平台就在线教学组织进行充分沟通，择优选取符合本校实际、与网络环境条件相匹配的方案，保证在线教学平稳运行。要与课程平台密切配合、规范管理，强化对课程内容、教学过程和平台运行监管，采取安全有效手段，防范和制止有害信息传播，保障在线教学运行安全。

　　4.建立信息报送渠道。各级教育行政部门和高校要建立数据统计报告制度，及时将在线教学组织实施情况和意见建议报送教育部。教育部直属高校直接报送教育部，其他中央部门所属高校和部省合建高校抄送教育部，地方高校由省级教育行政部门汇总后报送教育部。联系单位：教育部高等教育司课程教材与实验室处，联系人：宋毅、李静、王繁，电话：010-66096925、66097392、66097199，18810032218，电子邮箱：gaojs_jxtj@moe.edu.cn。

教育部应对新型冠状病毒感染肺炎疫情工作

领导小组办公室（代章）

2020年2月4日

（291）中国人民银行等5部门：进一步强化金融支持防控新型冠状病毒感染肺炎疫情	银发〔2020〕29号

新型冠状病毒感染的肺炎疫情发生以来，党中央、国务院高度重视，习近平总书记作出一系列重要指示。金融系统认真贯彻落实党中央、国务院决策部署，主动作为，确保金融服务畅通，支持各地疫情防控，发挥了积极作用。当前，疫情防控正处于关键阶段。为切实贯彻落实中共中央《关于加强党的领导、为打赢疫情防控阻击战提供坚强政治保证的通知》精神和中央应对新型冠状病毒感染肺炎疫情工作领导小组工作部署，进一步强化金融对疫情防控工作的支持，现就有关事宜通知如下：

一、保持流动性合理充裕，加大货币信贷支持力度

（一）保持流动性合理充裕。人民银行继续强化预期引导，通过公开市场操作、常备借贷便利、再贷款、再贴现等多种货币政策工具，提供充足流动性，保持金融市场流动性合理充裕，维护货币市场利率平稳运行。人民银行分支机构对因春节假期调整受到影响的金融机构，根据实际情况适当提高2020年1月下旬存款准备金考核的容忍度。引导金融机构加大信贷投放支持实体经济，促进货币信贷合理增长。

（二）加大对疫情防控相关领域的信贷支持力度。在疫情防控期间，人民银行会同发展改革委、工业和信息化部对生产、运输和销售应对疫情使用的医用防护服、医用口罩、医用护目镜、新型冠状病毒检测试剂盒、负压救护车、消毒机、84消毒液、红外测温仪和相关药品等重要医用物资，以及重要生活物资的骨干企业实行名单制管理。人民银行通过专项再贷款向金融机构提供低成本资金，支持金融机构对名单内的企业提供优惠利率的信贷支持。中央财政对疫情防控重点保障企业给予贴息支持。金融机构要主动加强与有关医院、医疗科研单位和相关企业的服务对接，提供足额信贷资源，全力满足相关单位和企业卫生防疫、医药用品制造及采购、公共卫生基础设施建设、科研攻关、技术改造等方面的合理融资需求。

（三）为受疫情影响较大的地区、行业和企业提供差异化优惠的金融服务。金融机构要通过调整区域融资政策、内部资金转移定价、实施差异化的绩效考核办法等措施，提升受疫情影响严重地区的金融供给能力。对受疫情影响较大的批发零售、住宿餐饮、物流运输、文化旅游等行业，以及有发展前景但受疫情影响暂遇困难的企业，特别是小微企业，不得盲目抽贷、断贷、压贷。对受疫情影响严重的企业到期还款困难的，可予以展期或续贷。通过适当下调贷款利率、增加信用贷款和中长期贷款等方式，支持相关企业战胜疫情灾害影响。各级政府性融资担保再担保机构应取消反担保要求，降低担保和再担保费。对受疫情影响严重地区的融资担保再担保机构，国家融资担保基金减半收取再担保费。

（四）完善受疫情影响的社会民生领域的金融服务。对因感染新型肺炎住院治疗或隔离人员、疫情防控需要隔离观察人员、参加疫情防控工作人员以及受疫情影响暂时失去收入来源的人群，金融机构要在信贷政策上予以适当倾斜，灵活调整住房按揭、信用卡等个人信贷还款安排，合理延后还款期限。感染新型肺炎的个人创业担保贷款可展期一年，继续享受财政贴息支持。对感染新型肺炎或受疫情影响受损的出险理赔客户，金融机构要优先处理，适当扩展责任范围，应赔尽赔。

（五）提高疫情期间金融服务的效率。对受疫情影响较大领域和地区的融资需求，金融机构要建立、启动快速审批通道，简化业务流程，切实提高业务办理效率。在受到交通管制的地区，金融机构要创新工作方式，采取在就近网点办公、召开视频会议等方式尽快为企业办理审批放款等业务。

（六）支持开发性、政策性银行加大信贷支持力度。国家开发银行、进出口银行、农业发展银行要结合自身业务范围，加强统筹协调，合理调整信贷安排，加大对市场化融资有困难的防疫单位和企业的生产研发、医药用品进口采购，以及重要生活物资供应企业的生产、运输和销售的资金支持力度，合理满足疫情防控的需要。

（七）加强制造业、小微企业、民营企业等重点领域信贷支持。金融机构要围绕内部资源配置、激励考核安排等加强服务能力建设，继续加大对小微企业、民营企业支持力度，要保持贷款增速，切实落实综合融资成本压降要求。增加制造业中长期贷款投放。

（八）发挥金融租赁特色优势。对于在金融租赁公司办理疫情防控相关医疗设备的金融租赁业务，鼓励予以缓收或减收相关租金和利息，提供医疗设备租赁优惠金融服务。

二、合理调度金融资源，保障人民群众日常金融服务

（九）保障基本金融服务畅通。金融机构要根据疫情防控工作需要，合理安排营业网点及营业时间，切实做好营业场所的清洁消毒，保障基本金融服务畅通。金融机构要加强全国范围特别是疫情严重地区的线上服务，引导企业和居民通过互联网、手机APP等线上方式办理金融业务。

（十）加强流通中现金管理。合理调配现金资源，确保现金供应充足。加大对医院、居民社区以及应急建设项目等的现金供应，及时满足疫情物资采购相关单位和企业的大额现金需求。做好现金储存及业务办理场地的消毒工作。对外付出现金尽可能以新券为主，对收入的现金采取消毒措施后交存当地人民银行分支机构。

（十一）确保支付清算通畅运行。人民银行根据需要，放开小额支付系统业务限额，延长大额支付系统、中央银行会计核算数据集中系统运行时间，支持金融机构线上办理人民币交存款等业务。人民银行分支机构、清算机构及银行业金融机构要做好各类支付清算系统、中央银行会计核算数据集中系统的正常安全运营，开通疫情防控专用通道，保障境内外救援和捐赠资金及时划拨到位、社会资金流转高效顺畅。

（十二）建立银行账户防疫“绿色通道”。银行业金融机构要在风险可控的前提下，做好与防控疫情相关的银行账户服务工作，简化开户流程，加快业务办理。要积极开辟捐款“绿色通道”，确保疫情防控款项第一时间到达指定收款人账户。减免银行业金融机构通过人民银行支付系统办理防控疫情相关款项汇划费用。鼓励清算机构、银行业金融机构对向慈善机构账户或疫区专用账户的转账汇款业务、对疫区的取现业务减免服务手续费。

（十三）加大电子支付服务保障力度。支持银行业金融机构、非银行支付机构在疫情防控期间，采用远程视频、电话等方式办理商户准入审核和日常巡检，通过交易监测强化风险防控。鼓励清算机构、银行业金融机构和非银行支付机构对特定领域或区域特约商户实行支付服务手续费优惠。银行业金融机构、非银行支付机构要强化电子渠道服务保障，灵活调整相关业务限额，引导客户通过电子商业汇票系统、个人网上银行、企业网上银行、手机银行、支付服务APP等电子化渠道在线办理支付结算业务。

（十四）切实保障公众征信相关权益。人民银行分支机构和金融信用信息基础数据库接入机构要妥善安排征信查询服务，引导公众通过互联网、自助查询机进行征信查询。要合理调整逾期信用记录报送，对因感染新型肺炎住院治疗或隔离人员、疫情防控需要隔离观察人员和参加疫情防控工作人员，因疫情影响未能及时还款的，经接入机构认定，相关逾期贷款可以不作逾期记录报送，已经报送的予以调整。对受疫情影响暂时失去收入来源的个人和企业，可依调整后的还款安排，报送信用记录。

（十五）畅通国库紧急拨款通道。建立财库银协同工作机制，及时了解财政部门疫情防控资金拨付安排，随时做好资金拨付工作。加强对商业银行相关业务的指导，建立信息反馈机制，及时跟踪资金拨付情况。人民银行和商业银行确保资金汇划渠道畅通和国库业务相关系统运行安全稳定，构建疫情防控拨款“绿色通道”。各级国库部门要简化业务处理流程和手续，确保疫情防控资金及时、安全、准确拨付到位。

（十六）切实保障消费者合法权益。金融机构要树立负责任金融理念，对受疫情影响临时停业或调整营业时间的网点，要提前向社会公布并主动说明临近正常营业的网点。金融机构要充分利用线上等方式保持投诉渠道畅通，优化客户咨询、投诉处理流程，及时妥善处理疫情相关的金融咨询和投诉。金融机构要切实加强行业自律，维护市场秩序，不得利用疫情进行不当金融营销宣传。

三、保障金融基础设施安全，维护金融市场平稳有序运行

（十七）加强金融基础设施服务保障。金融市场基础设施要从工作机制、人员配备、办公场所、系统运维、技术支持等方面提升服务保障能力，确保发行、交易、清算、结算等业务正常运转，尽可能实施全流程、全链条线上操作。要制定应急预案，对突发事件快速响应、高效处理。要加强与主管部门、市场机构、其他金融基础设施的沟通，保持业务系统联通顺畅。对受疫情影响较大的地区，要开设“绿色通道”，必要时提供特别服务安排，并降低服务收费标准。

（十八）稳妥开展金融市场相关业务。金融机构要合理调配人员，稳妥开展金融市场相关交易、清算、结算、发行、承销等工作，加强流动性管理与风险应对。要合理引导投资者预期，确保金融市场各项业务平稳有序开展。对受疫情影响较大地区的金融机构，要保持正常业务往来，加大支持力度。

（十九）提高债券发行等服务效率。中国银行间市场交易商协会、上海证券交易所、深圳证券交易所等要优化公司信用类债券发行工作流程，鼓励金融机构线上提交公司信用类债券的发行申报材料，远程办理备案、注册等，减少疫情传播风险。对募集资金主要用于疫情防控以及疫情较重地区金融机构和企业发行的金融债券、资产支持证券、公司信用类债券建立注册发行“绿色通道”，证券市场自律组织对拟投资于防疫相关医疗设备、疫苗药品生产研发企业的私募股权投资基金，建立登记备案“绿色通道”，切实提高服务效率。

（二十）灵活妥善调整企业信息披露等监管事项。上市公司、挂牌公司、公司债券发行人受疫情影响，在法定期限内披露2019年年报或2020年第一季度季报有困难的，证监会、证券交易所、全国中小企业股份转让系统要依法妥善安排。上市公司受疫情影响，难以按期披露业绩预告或业绩快报的，可向证券交易所申请延期办理；难以在原预约日期披露2019年年报的，可向证券交易所申请延期至2020年4月30日前披露。湖北省证券基金经营机构可向当地证监局申请延期办理年度报告的审计、披露和报备。受疫情影响较大的证券基金经营机构管理的公募基金或其他资产管理产品，管理人可向当地证监局申请延期办理年报审计和披露。对疫情严重地区的证券基金期货经营机构，适当放宽相关风控指标监管标准。

（二十一）适当放宽资本市场相关业务办理时限。适当延长上市公司并购重组行政许可财务资料有效期和重组预案披露后发布召开股东大会通知的时限。如因受疫情影响确实不能按期更新财务资料或发出股东大会通知的，公司可在充分披露疫情对本次重组的具体影响后，申请财务资料有效期延长或股东大会通知时间延期1个月，最多可申请延期3次。疫情期间，对股票发行人的反馈意见回复时限、告知函回复时限、财务报告到期终止时限，以及已核发的再融资批文有效期，自本通知发布之日起暂缓计算。已取得债券发行许可，因疫情影响未能在许可有效期内完成发行的，可向证监会申请延期发行。

（二十二）减免疫情严重地区公司上市等部分费用。免收湖北省上市公司、挂牌公司应向证券交易所、全国中小企业股份转让系统缴纳的2020年度上市年费和挂牌年费。免除湖北省期货公司应向期货交易所缴纳的2020年度会费和席位费。

四、建立“绿色通道”，切实提高外汇及跨境人民币业务办理效率

（二十三）便利防疫物资进口。银行应当为疫情防控相关物资进口、捐赠等跨境人民币业务开辟“绿色通道”。对有关部门和地方政府所需的疫情防控物资进口，外汇局各分支机构要指导辖区内银行简化进口购付汇业务流程与材料。

（二十四）便捷资金入账和结汇。对于境内外因支援疫情防控汇入的外汇捐赠资金业务，银行可直接通过受赠单位已有的经常项目外汇结算账户办理，暂停实施需开立捐赠外汇账户的要求。

（二十五）支持企业跨境融资防控疫情。企业办理与疫情防控相关的资本项目收入结汇支付时，无需事前、逐笔提交单证材料，由银行加强对企业资金使用真实性的事后检查。对疫情防控确有需要的，可取消企业借用外债限额等，并可线上申请外债登记，便利企业开展跨境融资。

（二十六）支持个人和企业合理用汇需求。银行应当密切关注个人用汇需求，鼓励通过手机银行等线上渠道办理个人外汇业务。与疫情防控有关的其他特殊外汇及人民币跨境业务，银行可先行办理、事后检查，并分别向所在地外汇局分支机构、人民银行分支机构报备。

（二十七）简化疫情防控相关跨境人民币业务办理流程。支持银行在“展业三原则”基础上，凭企业提交的收付款指令，直接为其办理疫情防控相关进口跨境人民币结算业务以及资本项目下收入人民币资金在境内支付使用。

五、加强金融系统党的领导，为打赢疫情防控阻击战提供坚强政治保证

（二十八）强化疫情防控的组织保障。金融管理部门和金融机构要增强“四个意识”，坚定“四个自信”，做到“两个维护”，切实把思想和行动统一到习近平总书记的重要指示精神上来，把打赢疫情防控阻击战作为当前重大政治任务，全力以赴做好各项金融服务工作。

（二十九）做好自身的疫情防控工作。金融管理部门和金融机构要完善疫情应对工作机制，持续关注员工特别是从疫情较重地区返回员工的健康情况，建立日报制度，加大疫情排查力度，做好员工防疫安排，努力为员工提供必要的防疫用品。

（三十）配合地方政府加强应急管理。金融管理部门和金融机构要按照属地原则，配合当地政府做好组织协调，及时处理突发事件。服从当地政府防疫安排，对防控疫情需要征用的人员车辆、设备设施等，不得推诿拒绝。各单位要继续严格执行应急值守制度，确保政令畅通。

各单位在执行中遇到的情况请及时报告。

中国人民银行 财政部 银保监会 证监会 外汇局

2020年1月31日

（292）国务院应对新型冠状病毒感染的肺炎疫情联防联控机制发出通知：做好儿童和孕产妇新型冠状病毒感染的肺炎疫情防控工作	肺炎机制发〔2020〕17号

各省、自治区、直辖市及新疆生产建设兵团应对新型冠状病毒感染的肺炎疫情联防联控机制（领导小组、指挥部）：

儿童和孕产妇是新型冠状病毒感染的肺炎的易感人群。为贯彻落实习近平总书记关于新型冠状病毒感染的肺炎疫情防控工作重要指示，按照中央关于做好疫情防控工作有关要求，现就进一步做好儿童和孕产妇新型冠状病毒感染的肺炎疫情防控工作、助力打赢疫情防控阻击战提出以下要求。

一、做好居家儿童疫情防控工作

各地要开展多种形式的健康教育，普及疫情防控知识，指导家长做好居家儿童防控措施落实。儿童应尽量避免外出，不到人员密集和空间密闭的场所，不走亲访友，不与有呼吸道感染症状的人接触，确需外出的要正确佩戴口罩，做好防护措施。家长要加强居室通风，做好室内消毒，创造清洁生活环境，外出回家后洗手更衣再接触儿童。家长要教会儿童正确洗手方法，督促儿童勤洗手、不乱摸，适度运动，合理膳食，充足睡眠，帮助儿童养成良好的卫生习惯。母亲母乳喂养时要佩戴口罩、洗净手，保持局部卫生。

家长为密切接触者的家庭，家长需居家隔离的，应当与儿童分开居住。儿童如出现发热、咳嗽、流涕等呼吸道感染症状，应当及时就诊，遵从医务人员指导。

二、做好基层医疗卫生机构儿童疫情防控工作

根据《关于加强新型冠状病毒感染的肺炎疫情社区防控工作的通知》（肺炎机制发〔2020〕5号）和《民政部、国家卫生健康委关于进一步动员城乡社区组织开展新型冠状病毒感染的肺炎疫情防控工作的紧急通知》（民发〔2020〕9号），基层医疗卫生机构要做好社区儿童疫情防控工作。要全面掌握辖区内儿童信息，做好健康管理。在当地党委政府统一部署下，会同城乡社区组织按照“追踪到人、登记在册、社区管理、上门观察、规范运转、异常就医”的原则将来自疫情发生地区、外地返回居住地的儿童作为重点人群，加强发热和症状监测，进行有效管理。指导家长和儿童科学认识和预防疾病，增强防控意识，提高防护能力。基层医疗卫生机构在疫情期间合理调整儿童保健门诊和预防接种门诊，暂停面对面新生儿访视和儿童健康体检，通过微信、电话、视频等方式开展在线咨询和指导。

三、做好妇幼保健机构儿童疫情防控工作

各级妇幼保健机构要按照当地卫生健康行政部门的统一部署开展疫情防控工作，严格值班值守，规范分诊救治，落实报告制度，强化院感防控。针对家长、儿童和托幼机构教职员工做好健康宣教。在疫情期间合理调整儿童保健门诊和预防接种门诊，暂停亲子活动、家长学校等与儿童相关的集体性活动。

妇幼保健机构要分区域设置普通儿童就诊门诊和发热门诊（诊室）。要按照《医疗机构传染病预检分诊管理办法》有关规定严格落实预检分诊制度。执行发热病人接诊、筛查流程，加强发热病人预检分诊、登记、报告等工作，做好病例排查，及时识别可疑病例，认真落实发热病人登记报告制度。要按照新型冠状病毒感染的肺炎诊疗方案做好临床诊疗工作，一旦发现疑似病例，应当立即转诊到具备有效隔离条件和防护条件的定点医疗机构隔离治疗。要做好辖区内基层医疗卫生机构和托幼机构人员儿童疫情防控的指导和培训工作。

四、做好儿童医院和综合医院儿科疫情防控工作

儿童医院和综合医院儿科要按照国家卫生健康委发布的最新版新型冠状病毒感染的肺炎诊疗方案要求，做好发热患儿的筛查、疑似患儿和确诊患儿的处置。要分区域设置普通儿童就诊门诊和发热门诊（诊室）。各地新型冠状病毒感染的肺炎救治专家组成员中要有一定数量的儿科医生，参与患儿救治，并对基层防治工作进行指导。

五、做好托幼机构疫情防控工作

托幼机构根据当地政府部署延迟开园。托幼机构主要负责人是本单位新型冠状病毒感染的肺炎疫情防控第一责任人，要切实保障各项责任、措施落实到位。未开园期间，托幼机构应当每日了解教职员工及儿童健康情况，实行“日报告”、“零报告”制度，每天根据防控要求向主管部门报告具体情况。开园前要根据上级主管部门要求和最新版新型冠状病毒感染的肺炎防控方案对全体教职员工进行制度、知识和技能培训，并做好园区的预防性消毒工作。

开园后每天对园区进行日常消毒，开窗通风。教职员工每天入园前应测体温，严格落实儿童检测体温等晨午晚检制度和全日观察，发现异常者不得入园。严格落实教职员工和儿童手卫生措施。做好教职员工和儿童因病缺勤的追访工作。执行家长接送儿童不入园制度，指导家长培养儿童日常卫生习惯，在疫情完全解除前不带儿童去人员密集场所。

六、做好助产机构孕产妇疫情防控工作

助产机构要结合实际，尽可能为产科门诊及病房设置独立进出通道。要通过微信、APP、电话、视频等方式加强对孕产妇健康教育和咨询指导。根据孕产妇具体情况，必要时可适当调整产检时间。对有妊娠合并症/并发症等高危孕产妇，指导其按时接受产前检查，出现异常情况应及时就医，避免因担忧、恐惧而延误病情。对临近预产期且建档机构为新型冠状病毒感染的肺炎救治定点医院的孕产妇，要及早作出合理安排，并及时告知孕产妇，减轻其焦虑感。对出现发热、乏力、干咳等症状且有流行病学史的孕产妇，要指导其及时到发热门诊就诊。各地要按照相关要求在有条件的助产机构设置发热门诊，指定综合救治能力强的医疗机构作为定点医院，为疑似和确诊孕产妇提供疾病救治和安全助产服务，确保母婴安全。产妇为疑似病例、确诊病例和确诊后未痊愈者，暂停母乳喂养。

七、强化儿童和孕产妇健康服务相关医疗机构院感防控

各级妇幼保健机构、基层医疗卫生机构、儿童医院等相关医疗机构要加强院感控制管理，指导医务人员严格按照标准预防原则，根据医疗操作可能传播的风险，做好个人防护、手卫生、环境消毒和废弃物管理等医院感染控制工作，严防医务人员感染事件发生。要强化院内疾病人群与健康人群就诊区域隔离分流，加强妇科、产科、儿科等重点科室病室管理，减少家属探视，暂停新生儿病房探视和陪护，切实降低住院患者感染风险。

八、加强医务人员全员培训

各级妇幼保健机构、基层医疗卫生机构、儿童医院等相关医疗机构要对医务人员全面开展新型冠状病毒感染的肺炎病例的发现与报告、医疗救治、院感防控、密接管理、个人防护等内容的培训，提高防控和诊疗能力。对门诊、急诊、检验科等重点岗位医务人员要开展培训效果考核评估，确保掌握相关知识与技能。

九、利用信息化手段做好儿童和孕产妇疫情防控工作

各地要充分发挥信息化技术和新媒体作用，借助“互联网+医疗健康”优势，对儿童和孕产妇开展疫情防控健康教育和科普宣传。医疗卫生机构要利用短信、微信、微博、视频等新媒体，通过开设“网上问诊”、“发热门诊”等服务板块，开展儿童保健和孕产妇保健在线咨询和指导。社会力量举办机构开展与儿童和孕产妇相关的早教、亲子活动和保健服务的，鼓励以互联网形式提供，暂停线下活动。

国务院应对新型冠状病毒感染的肺炎

疫情联防联控机制

（代章）

2020年2月2日

（293）交通运输部：切实保障疫情防控应急物资运输车辆顺畅通行	交运明电〔2020〕37号

各省、自治区、直辖市、新疆生产建设兵团交通运输厅（局、委）：

为认真贯彻习近平总书记关于做好新型冠状病毒感染肺炎疫情防控工作的重要指示精神，落实党中央、国务院决策部署，切实做好应对疫情各类应急物资、生活物资、重点生产物资、医护及防控人员的运输保障工作，保障应急物资运输车辆的顺畅通行，现就做好应急运输车辆顺畅通行有关事项紧急通知如下：

一、向社会公开应急运输电话。为切实保障疫情防控应急运输车辆快速通行，部向社会公开全国各省（自治区、直辖市）交通运输厅（局、委）应急运输电话（见附件1），及时受理和解决应急物资运输车辆通行中的相关问题，切实保障车辆的顺畅高效通行。

各省交通运输主管部门要严格实施应急物资运输公开电话值班值守制度，保持应急运输电话24小时畅通，明确岗位职责和工作流程，细化各种情况的处理措施。要通过行业协会、新闻媒体、互联网等媒介手段对公开电话进行广泛宣传，确保广大道路货运经营者能够知晓和使用应急运输电话。要建立与各收费站点、各交通运输执法队伍的便捷沟通机制，督促各地交通运输部门严格落实防疫应急运输车辆绿色通道政策，保障防疫应急物资和人员运输车辆“不停车、不检查、不收费”，优先便捷通行。

二、进一步简化《通行证》办理流程。为进一步简化程序，方便应急物资运输车辆高效便捷通行，应急始发地或目的地交通运输主管部门根据相关部门提供的调拨和转运等相关信息，提供《新型冠状病毒感染的肺炎疫情防控物资及人员运输车辆通行证》式样（见附件2），由承运单位或驾驶人在本地自行打印,自行填写，随车携带，享受免费通行政策。各地交通运输主管部门督促沿途收费公路经营管理单位做好相关服务保障工作。

三、切实保障应急运输车辆顺畅通行。各省交通运输部门要严格落实“一断三不断”的要求，坚决阻断病毒传播渠道，保障公路交通网络不断、应急运输通道不断、必要的群众生产生活物资的运输通道不断。在疫情防控特殊时期，要将重要生活物资纳入应急运输保障范围，落实绿色通道政策，确保“三不一优先”。要积极协调卫生健康等部门，在高速公路服务区、收费站、省界主线站设置卫生检疫站的，进一步增加防疫监测力量，尽量采取复式检测，对应急运输车辆实行“绿色通道”，在保障检测质量的前提下，快速检测通行，减少车辆排队和拥堵。要积极协调公安部门，加强路面交通管控，及时增派警力进行疏导，确保高速公路右侧应急车道畅通，保障应急物资运输车辆和医患人员运输车辆快速通行。

四、进一步严格应急运输保障工作纪律。各省交通运输主管部门要按照“属地负责、特事特办、部省联动”的原则，第一时间协调解决通过公开电话所反映的应急运输车辆通行的问题，属于本省内应急物资运输车辆通行的问题，由各省及时协调解决，属于跨省运输车辆通行问题，及时与相关省份交通运输主管部门进行协调，解决不了的及时向部物流保障办公室报告。严禁各地无故推诿、搪塞、拖延不解决问题，严禁各地出台“土政策”阻碍从本省外运的应急物资运输，严禁各地违规设卡阻碍应急物资运输车辆通行，对于不及时协调解决问题阻碍应急物资运输车辆通行的，部将定期汇总相关情况后报国务院办公厅督查室。

附件：1.全国应急物资运输电话

2.《新型冠状病毒感染的肺炎疫情防控物资及人员运输车辆通行证》式样

交通运输部

2020年2月1日

（294）商务部：进一步强化生活必需品市场供应保障工作

各省、自治区、直辖市、计划单列市及新疆生产建设兵团，副省级省会城市商务主管部门：

新型冠状病毒感染肺炎疫情发生以来，各地认真贯彻落实党中央、国务院重要决策部署，快速采取行动。保障生活必需品市场供应，取得了积极成效。目前，受春节假期延长，人员陆续返城叠加影响，大中城市生活必需品需求不断增加，局部物流不畅，中小企业开门营业不足，生活必需品保供面临的局面更为复杂，任务更加艰巨。各地要深入贯彻习近平总书记重要讲话和指示批示精神，坚决把思想和行动统一到党中央、国务院决策部署上来，按照全国商务系统应对疫情电视电话会议和全国应对疫情生活物资保障工作视频会议要求，进一步落实好粮食安全省长负责制和“菜篮子”市长负责制，坚持底线思维，压实工作责任，千方百计保障生活必需品市场供应，切实满足人民群众基本生活需要。现将有关工作通知如下：

（一）压紧压实组织领导责任。各级商务主管部门要在当地党委、政府统一领导下，主要负责同志坚守岗位，靠前指挥，立即组织工作人员尽快到岗到位，调配精兵强将，充实防疫保供工作力量。要摸清一线市场最新最实的情况，发现问题及时组织解决。要根据防疫保供新形势新变化，调整工作方案，完善应急预案，采取有效举措，争分夺秒保障生活必需品市场供应跟得上、不脱销、不断档。

（二）强化细化联保联供机制。要发扬“一方有难，八方支援”精神，进一步加强9省区市联保联供协作机制和31个省区市商务主管部门疫情联防联控应急工作联络机制作用，筑牢保障“疫情严重区域”市场供应防线，主动作为、积极支持湖北特别是武汉做好生活物资保供工作。湖北商务厅和武汉商务局要做好市场保供与疫情防控政策衔接，研究落实二次物流方式在交通枢纽地带设置对结点，衔接各地调配物资。武汉市商务局要强化与公安、交通等部门的密切协作，迅速打通生活物资调配至零售终端的物流通道。周边及主产地区商务主管部门要加强与湖北、武汉统筹协调，组织骨干企业保障供应，做好跨区调运工作。要在物资调运前，与湖北省及武汉市做好沟通对接，安排生活物资高效有序落地，确保货源供应调得快、用得上。各地一旦出现区域性生活必需品断档脱销，要第一时间提出需求，商务部将立即组织对接，推动协调解决。

（三）动员企业及时开门营业。要继续发挥好大型商贸骨干企业市场保供示范带头作用，全面组织动员中小商贸流通和居民生活服务企业，在做好疫情防护的前提下，通过临时顶岗、合理排班、动态调岗等灵活措施，统筹安排人员上岗，持续提供购物、餐饮、卫生保洁等基本生活服务。要指导商超等流通企业增加补货频次，充实蔬菜等鲜活商品上架。既要细化分类保关键，增加蔬菜特别是土豆、白菜等耐储蔬菜的供应，又要集中力量保重点，增加粮油、肉类、方便面、瓶装水、口罩等生活必需品市场供应。

（四）不折不扣做好市场监测。要切实执行好“生活必需品市场”日报监测、应急商品数据库日报、生活必需品供求情况报送、31个省区市生活必需品市场异常情况零报告等重要制度，摸清蔬菜等12种重点生活必需品货源底数和对接方式；及时掌握和报送当地生活必需品市场供求变化。发现集中抢购、脱销断档等异常情况，要第一时间处置并上报，没有异常情况也要每日按时报告。对重大异常波动不报、缓报、漏报、瞒报的，要提请相关部门严肃追责，严肃处理。要加强趋势预测，对市场走势及早研判，提出预案，不断提高监测灵敏性、及时性、有效性和前瞻性。

（五）做实做细货源对接调运。要加强货源组织，尽快落实一批连锁集团公司等储备规模大、物流配送能力强的应急保供骨干企业，明确公布一批应急商品投放网点，指导督促商贸流通企业进一步拓宽货源，加大采购力度。要充分发挥农产品批发市场促进农产品集散、把关农产品质量、保障城市农产品供应的作用。要积极发挥大型电商平台筹措物资、对接产销的作用。要指导具有资质和能力的企业积极开拓进口渠道，及时组织进口。要协助超市、商场等零售企业，特别是中小企业与生活必需品批发市场高效对接。要抓紧解决部分地区物流配送“最后一公里”受阻等问题，在本地区生活物资保障组的统一协调下，加强与本地区各部门横向协作，共享信息、互通资源，集中协调解决流通企业在交通运输、物流配送等方面的问题，畅通生活必需品运输配送渠道。对于哄抬物价、囤积居奇等市场投机行为，要严厉打击，绝不姑息。

（六）加强宣传做好内部防控。要切实加强宣传引导，密切关注舆情变化，做好宣传方案，充分发挥新媒体传播快的优势，及时准确报道本地市场供应情况，实事求是地宣传保供稳价成效。要组织指导企业积极正面发声，采取安民告示等多种方式，切实提示顾客不要恐慌、理性购买，稳定社会预期，增强民众信心。要制定周密方案，采取严格措施，切实落实单位内部人员疫情防控和企业经营场所通风、消毒、人流疏导等措施，保障各级商务部门和企业人员安全健康，确保生活必需品供应持续不断、高效安全。

各地商务主管部门要统一思想，提高站位，充分认识做好生活必需品供应保障、稳定社会预期等工作的极端重要性，切实增强紧迫感和责任感，扎实做好各项工作，有关情况请按要求及时上报商务部（市场运行司）。

联系人：市场运行司调控处 010-85093851/3861(传真) tiaok_scyx@mofcom.gov.cn

市场运行司统计监测处 010-85093841/3096(传真) jiancechu@mofcom.gov.cn

商务部办公厅

2020年2月1日

（295）生态环境部：做好新型冠状病毒感染的肺炎疫情医疗污水和城镇污水监管工作	环办水体函〔2020〕52号

各省、自治区、直辖市生态环境厅（局），新疆生产建设兵团生态环境局：

为有效应对新型冠状病毒感染的肺炎疫情（以下简称疫情），进一步加强医疗污水和城镇污水监管工作，防止新型冠状病毒通过污水传播扩散，现将有关事项通知如下。

一、高度重视医疗污水和城镇污水监管工作，将其作为疫情防控工作的一项重要内容抓紧抓实。进一步加强医疗污水收集、污染治理设施运行、污染物排放等监督管理；主动加强与卫生健康、城镇排水等部门的协调配合，健全联动机制，形成工作合力。

二、已发生疫情的地方，当地生态环境部门要指导督促接收新型冠状病毒感染的肺炎患者或疑似患者诊疗的定点医疗机构（医院、卫生院等）、相关临时隔离场所及研究机构，严格执行《医疗机构水污染物排放标准》（GB 18466-2005），参照《医院污水处理技术指南》（环发〔2003〕197号）、《医院污水处理工程技术规范》（HJ 2029-2013）和《新型冠状病毒污染的医疗污水应急处理技术方案（试行）》（见附件）等有关要求，对污水和废弃物进行分类收集和处理，确保稳定达标排放。对没有医疗污水处理设施或污水处理能力未达到相关要求的医院，应督促其参照《医院污水处理工程技术规范》及《医院污水处理技术指南》，因地制宜建设临时性污水处理罐（箱），采取加氯、过氧乙酸等措施进行杀菌消毒。切实加强对医疗污水消毒情况的监督检查，严禁未经消毒处理或处理未达标的医疗污水排放。对隔离区要指导其对外排粪便和污水进行必要的杀菌消毒。

地方生态环境部门要督促城镇污水处理厂切实加强消毒工作，结合实际，采取投加消毒剂或臭氧、紫外线消毒等措施，确保出水粪大肠菌群数指标达到《城镇污水处理厂污染物排放标准》（GB 18918-2002）要求。

当前公共场所和家庭为防控疫情多采用含氯消毒剂进行消毒，排入城镇污水处理厂的污水余氯量可能偏高，影响生化处理单元正常运行。地方生态环境部门要督促各城镇污水处理厂密切关注进水水质余氯指标的变化情况，及时采取有针对性的应对措施，确保出水达标。

三、未发生疫情的地方，当地生态环境部门要密切关注疫情发展，指导督促本行政区域内医疗机构、接纳医疗污水的城镇污水处理机构等提前做好应对准备。

四、加大农村医疗污水处置的监管力度，指导督促卫生院（所）因地制宜采取加氯、过氧乙酸等措施进行专门的灭菌消毒，防止病毒通过医疗污水扩散。严格污水灌溉的环境管理，禁止向农田灌溉渠道排放医疗污水。

五、进一步加强饮用水水源地保护，做好水质监测，确保饮用水水源不受污染。加大对农贸市场、集贸市场、超市、车站、机场、码头等重点场所污水收集处理的现场监督检查力度，依法查处违法排污，严防发生污染事故。

六、在当地党委政府统一领导下，做好本行政区域内医疗污水和城镇污水处理、污染物排放信息发布工作。按照生态环境部调度安排，及时准确统计报送当地医疗污水和城镇污水监管情况。要加强与卫生健康、城镇排水、农业农村、公安等部门信息共享，强化联防联控，严防疫情扩散蔓延，合力打赢疫情防控阻击战。

特此通知。

附件：新型冠状病毒污染的医疗污水应急处理技术方案（试行）

生态环境部办公厅

2020年2月1日

（296）财政部：支持金融强化服务 做好新型冠状病毒感染肺炎疫情防控工作	财金〔2020〕3号

各省、自治区、直辖市、计划单列市财政厅（局），新疆生产建设兵团财政局，财政部各地监管局：

为坚决贯彻落实《中共中央关于加强党的领导、为打赢疫情防控阻击战提供坚强政治保证的通知》精神，发挥财政资金引导撬动作用，支持金融更好服务新型冠状病毒感染肺炎疫情防控（以下简称疫情防控）工作，现通知如下：

一、对疫情防控重点保障企业贷款给予财政贴息支持。对2020年新增的疫情防控重点保障企业贷款，在人民银行专项再贷款支持金融机构提供优惠利率信贷的基础上，中央财政按人民银行再贷款利率的50%给予贴息，贴息期限不超过1年，贴息资金从普惠金融发展专项资金中安排。

（一）经发展改革委、工业和信息化部等部门确定的疫情防控重点保障企业，可凭借2020年1月1日后疫情防控期内新生效的贷款合同，中央企业直接向财政部申请，地方企业向所在地财政部门申请贴息支持。对支持疫情防控工作作用突出的其他卫生防疫、医药产品、医用器材企业，经省级财政部门审核确认后，可一并申请贴息支持。

（二）各省级财政部门汇总编制本地区贴息资金申请表（见附件），于2020年5月31日前报送财政部。财政部审核后，向省级财政部门拨付贴息资金，由省级财政部门直接拨付给相关借款企业。5月31日后，再视情决定是否受理贴息资金申请。

（三）享受贴息支持的借款企业应将贷款专项用于疫情防控相关生产经营活动，保障疫情防控相关重要医用、生活物资平稳有序供应，不得将贷款资金用于投资、理财或其他套利活动，不得哄抬物价、干扰市场秩序。

二、加大对受疫情影响个人和企业的创业担保贷款贴息支持力度。对已发放的个人创业担保贷款，借款人患新型冠状病毒感染肺炎的，可向贷款银行申请展期还款，展期期限原则上不超过1年，财政部门继续给予贴息支持，不适用《普惠金融发展专项资金管理办法》（财金〔2019〕96号）关于“对展期、逾期的创业担保贷款，财政部门不予贴息”的规定。对受疫情影响暂时失去收入来源的个人和小微企业，地方各级财政部门要会同有关方面在其申请创业担保贷款时优先给予支持。

三、优化对受疫情影响企业的融资担保服务。鼓励金融机构对疫情防控重点保障企业和受疫情影响较大的小微企业提供信用贷款支持，各级政府性融资担保、再担保机构应当提高业务办理效率，取消反担保要求，降低担保和再担保费率，帮助企业与金融机构对接，争取尽快放贷、不抽贷、不压贷、不断贷。国家融资担保基金对于受疫情影响严重地区的政府性融资担保、再担保机构，减半收取再担保费。对于确无还款能力的小微企业，为其提供融资担保服务的各级政府性融资担保机构应及时履行代偿义务，视疫情影响情况适当延长追偿时限，符合核销条件的，按规定核销代偿损失。

四、加强资金使用绩效监督管理。各级财政部门应及时公开疫情防控重点保障企业获得贴息支持情况，并督促相关贷款银行加强贷后管理，确保贴息贷款专款专用。疫情防控重点保障企业贴息资金管理执行《普惠金融发展专项资金管理办法》，财政部各地监管局应加强贴息资金使用情况监管，强化跟踪问效，切实提高财政资金使用效益。对于未按规定用途使用贷款的企业，一经发现，要追回中央财政贴息资金。

五、认真抓好政策贯彻落实。地方各级财政部门要增强“四个意识”，做到“两个维护”，把打赢疫情防控阻击战作为当前重大政治任务，会同有关方面加强政策宣传、组织协调和监督管理工作，发现政策执行中的重大情况，及时向财政部报告，切实保障政策真正惠及疫情防控重点保障企业以及受疫情影响的人群、企业和地区。

附件：疫情防控重点保障企业贷款贴息资金申请表

财 政 部

2020年2月1日

（297）财政部 卫健委：发布新型冠状病毒感染肺炎疫情防控有关经费保障政策	财社〔2020〕2号

各省、自治区、直辖市、计划单列市财政厅（局）、卫生健康委，新疆生产建设兵团财政局、卫生健康委：

为贯彻落实习近平总书记对新型冠状病毒感染肺炎疫情防控工作作出的重要批示精神，按照党中央、国务院决策部署，支持各地更好地做好防控经费保障工作，坚决遏制疫情蔓延势头，现将有关经费保障问题通知如下：

一、落实患者救治费用补助政策。对于确诊患者发生的医疗费用，在基本医保、大病保险、医疗救助等按规定支付后，个人负担部分由财政给予补助。所需资金由地方财政先行支付，中央财政对地方财政按实际发生费用的60%予以补助。

二、对参加防治工作的医务人员和防疫工作者给予临时性工作补助。参照《人力资源社会保障部财政部关于建立传染病疫情防治人员临时性工作补助的通知》（人社部规〔2016〕4号）有关规定，按照一类补助标准，对于直接接触待排查病例或确诊病例，诊断、治疗、护理、医院感染控制、病例标本采集和病原检测等工作相关人员，中央财政按照每人每天300元予以补助；对于参加疫情防控的其他医务人员和防疫工作者，中央财政按照每人每天200元予以补助。补助资金由地方先行垫付，中央财政与地方据实结算。中央级医疗卫生机构按照属地化管理，中央财政补助资金拨付地方后由地方财政统一分配。

三、医疗卫生机构开展疫情防控工作所需的防护、诊断和治疗专用设备以及快速诊断试剂采购所需经费，由地方财政予以安排，中央财政视情给予补助。中央级医疗卫生机构按照属地化管理，中央财政补助资金拨付地方后由地方财政统一分配。

各级财政部门要会同卫生健康部门尽快按规定落实上述补助政策，务必做好防控经费保障工作，决不能因为费用问题延误救治和疫情防控。同时，要及时对相关支出进行严格审核，报国家卫生健康委和财政部，作为中央财政与地方财政资金结算的依据。

财政部 国家卫生健康委

2020年1月25日

（298）卫健委：印发新型冠状病毒感染不同风险人群防护指南和预防新型冠状病毒感染的肺炎口罩使用指南

各省、自治区、直辖市及新疆生产建设兵团卫生健康委，国务院应对新型冠状病毒感染的肺炎疫情联防联控机制成员单位：

　　为指导做好新型冠状病毒感染不同风险人群防护工作，降低因防护用品配备使用不当等引发的疾病感染风险，我们组织专家编制了《新型冠状病毒感染不同风险人群防护指南》和《预防新型冠状病毒感染的肺炎口罩使用指南》，现印发你们，供指导加强个人防护使用。

疫情防控组

国家卫生健康委疾控局（代章）

2020年1月30日

（信息公开形式：主动公开）

新型冠状病毒感染不同风险人群防护指南

为指导做好新型冠状病毒感染不同风险人群防护工作，特制订本指南。本指南适用于普通居家人员、出行人员、居家隔离人员、特定行业人员。

　　一、普通居家人员

　　（一）尽量减少外出活动。减少走亲访友和聚餐，尽量在家休息。减少到人员密集的公共场所活动，尤其是相对封闭、空气流动差的场所，例如公共浴池、温泉、影院、网吧、KTV、商场、车站、机场、码头和展览馆等。

　　（二）做好个人防护和手卫生。家庭置备体温计、口罩、家用消毒用品等物品。未接触过疑似或确诊患者且外观完好、无异味或脏污的口罩，回家后可放置于居室通风干燥处，以备下次使用。需要丢弃的口罩，按照生活垃圾分类的要求处理。随时保持手卫生，从公共场所返回、咳嗽手捂之后、饭前便后，用洗手液或香皂流水洗手，或者使用免洗洗手液。不确定手是否清洁时，避免用手接触口鼻眼。打喷嚏或咳嗽时，用手肘衣服遮住口鼻。

　　（三）保持良好的生活习惯。居室整洁，勤开窗，经常通风，定时消毒。平衡膳食，均衡营养，适度运动，充分休息。不随地吐痰，口鼻分泌物用纸巾包好，弃置于有盖垃圾箱内。

　　（四）主动做好个人与家庭成员的健康监测，自觉发热时要主动测量体温。家中有小孩的，要早晚摸小孩的额头，如有发热要为其测量体温。

　　（五）若出现发热、咳嗽、咽痛、胸闷、呼吸困难、乏力、恶心呕吐、腹泻、结膜炎、肌肉酸痛等可疑症状，应根据病情，及时到医疗机构就诊。

　　二、出行人员

　　（一）日常生活与工作出行人员，外出前往超市、餐馆等公共场所和乘坐公共交通工具时，要佩戴口罩，尽量减少与他人的近距离接触。个人独处、自己开车或独自到公园散步等感染风险较低时，不需要佩戴口罩。

　　（二）出现可疑症状需到医疗机构就诊时，应佩戴口罩，可选用医用外科口罩，尽量避免乘坐地铁、公交车等交通工具，避免前往人群密集的场所。就诊时应主动告知医务人员相关疾病流行地区的旅行居住史，以及与他人接触情况，配合医疗卫生机构开展相关调查。

　　（三）远距离出行人员，需事先了解目的地是否为疾病流行地区。如必须前往疾病流行地区，应事先配备口罩、便携式免洗洗手液、体温计等必要物品。旅行途中，尽量减少与他人的近距离接触，在人员密集的公共交通场所和乘坐交通工具时要佩戴KN95/N95及以上颗粒物防护口罩。口罩在变形、弄湿或弄脏导致防护性能降低时需及时更换。妥善保留赴流行地区时公共交通票据信息，以备查询。从疾病流行地区返回，应尽快到所在社区居民委员会、村民委员会进行登记并进行医学观察，医学观察期限为离开疾病流行地区后 14 天。医学观察期间进行体温、体征等状况监测，尽量做到单独居住或居住在通风良好的单人房间，减少与家人的密切接触。

　　三、居家隔离人员

　　（一）对新型冠状病毒感染的肺炎病例密切接触者，采取居家隔离医学观察。医学观察期限为自最后一次与病例、感染者发生无有效防护的接触后 14 天。居家隔离人员应相对独立居住，尽可能减少与共同居住人员的接触，做好医学观察场所的清洁与消毒工作，避免交叉感染。观察期间不得外出，如果必须外出，经医学观察管理人员批准后方可，并要佩戴医用外科口罩，避免去人群密集场所。

　　（二）居家隔离人员每日至少进行2次体温测定，谢绝探访。尽量减少与家人的密切接触，不得与家属共用任何可能导致间接接触感染的物品，包括牙刷、香烟、餐具、食物、饮料、毛巾、衣物及床上用品等。

　　（三）他人进入居家隔离人员居住空间时，应规范佩戴KN95/N95及以上颗粒物防护口罩，期间不要触碰和调整口罩。尽量避免与居家隔离人员直接接触，如发生任何直接接触，应及时做好清洁消毒。

　　四、特定行业人员

　　（一）对于公共交通工具司乘人员、出租车司机、公共场所服务人员、武警、交警、安保人员、媒体记者、快递人员等行业人员，因日常接触人员较多，存在感染风险，其所在单位应为其配置一次性使用医用口罩或医用外科口罩或KN95/N95及以上颗粒物防护口罩，以及手消毒液、消毒纸巾、体温计等物品，并做好工作环境的日常清洁与消毒。工作期间，应做好个人防护，规范佩戴口罩上岗。口罩在变形、弄湿或弄脏导致防护性能降低时需及时更换。注意保持手卫生，用洗手液或香皂流水洗手，或者使用免洗洗手液。每日至少2次测量体温。一般情况下，不必穿戴防护服、防护面罩等防护用品。如出现可疑症状（如发热、咳嗽、咽痛、胸闷、呼吸困难、乏力、恶心呕吐、腹泻、结膜炎、肌肉酸痛等），应立即停止工作，根据病情居家隔离或就医。

　　（二）对于隔离病区工作人员、医学观察场所工作人员、疑似和确诊病例转运人员，建议穿戴工作服、一次性工作帽、一次性手套、医用一次性防护服、医用防护口罩或动力送风过滤式呼吸器、防护面屏或护目镜、工作鞋或胶靴、防水靴套等。

　　（三）对于流行病学调查人员，开展密切接触者调查时，穿戴一次性工作帽、医用外科口罩、工作服、一次性手套，与被调查对象保持1米以上距离。开展疑似和确诊病例调查时，建议穿戴工作服、一次性工作帽、一次性手套、医用一次性防护服、KN95/N95及以上颗粒物防护口罩或医用防护口罩、防护面屏或护目镜、工作鞋或胶靴、防水靴套等，对疑似和确诊病例也可考虑采取电话或视频方式流调。

　　（四）对于标本采集人员、生物安全实验室工作人员，建议穿戴工作服、一次性工作帽、双层手套、医用一次性防护服、KN95/N95及以上颗粒物防护口罩或医用防护口罩或动力送风过滤式呼吸器、防护面屏、工作鞋或胶靴、防水靴套。必要时，可加穿防水围裙或防水隔离衣。

　　（五）对于环境清洁消毒人员、尸体处理人员，建议穿戴工作服、一次性工作帽、一次性手套和长袖加厚橡胶手套、医用一次性防护服、KN95/N95及以上颗粒物防护口罩或医用防护口罩、工作鞋或胶靴、防水靴套、防水围裙或防水隔离衣等。环境清洁消毒人员使用动力送风过滤式呼吸器时，根据消毒剂种类选配尘毒组合的滤毒盒或滤毒罐，做好消毒剂等化学品的防护。

预防新型冠状病毒感染的肺炎

口罩使用指南

口罩是预防呼吸道传染病的重要防线，可以降低新型冠状病毒感染风险。口罩不仅可以防止病人喷射飞沫，降低飞沫量和喷射速度，还可以阻挡含病毒的飞沫核，防止佩戴者吸入。根据目前对该疾病的认识，就如何正确使用口罩制定本指南。

　　一、佩戴原则

　　基本原则是科学合理佩戴，规范使用，有效防护。具体如下：

　　（一）在非疫区空旷且通风场所不需要佩戴口罩，进入人员密集或密闭公共场所需要佩戴口罩。

　　（二）在疫情高发地区空旷且通风场所建议佩戴一次性使用医用口罩；进入人员密集或密闭公共场所佩戴医用外科口罩或颗粒物防护口罩。

　　（三）有疑似症状到医院就诊时，需佩戴不含呼气阀的颗粒物防护口罩或医用防护口罩。

　　（四）有呼吸道基础疾病患者需在医生指导下使用防护口罩。年龄极小的婴幼儿不能戴口罩，易引起窒息。

　　（五）棉纱口罩、海绵口罩和活性炭口罩对预防病毒感染无保护作用。

　　二、推荐的口罩类型及使用对象

　　（一）一次性使用医用口罩：推荐公众在非人员密集的公共场所使用。

　　（二）医用外科口罩：防护效果优于一次性使用医用口罩，推荐疑似病例、公共交通司乘人员、出租车司机、环卫工人、公共场所服务人员等在岗期间佩戴。

　　（三）KN95/N95及以上颗粒物防护口罩：防护效果优于医用外科口罩、一次性使用医用口罩，推荐现场调查、采样和检测人员使用，公众在人员高度密集场所或密闭公共场所也可佩戴。

　　（四）医用防护口罩：推荐发热门诊、隔离病房医护人员及确诊患者转移时佩戴。

　　三、使用后口罩处理原则

　　（一）健康人群佩戴过的口罩，没有新型冠状病毒传播的风险，一般在口罩变形、弄湿或弄脏导致防护性能降低时更换。健康人群使用后的口罩，按照生活垃圾分类的要求处理即可。

　　（二）疑似病例或确诊患者佩戴的口罩，不可随意丢弃，应视作医疗废弃物，严格按照医疗废弃物有关流程处理，不得进入流通市场。

　　四、儿童佩戴口罩的标准与注意事项

　　建议儿童选用符合国家标准GB2626-2006 KN95，并标注儿童或青少年颗粒物防护口罩的产品。儿童使用口罩需注意以下事项：

　　（一）儿童在佩戴前，需在家长帮助下，认真阅读并正确理解使用说明，以掌握正确使用呼吸防护用品的方法；

　　（二）家长应随时关注儿童口罩佩戴情况，如儿童在佩戴口罩过程中感觉不适，应及时调整或停止使用；

　　（三）因儿童脸型较小，与成人口罩边缘无法充分密合，不建议儿童佩戴具有密合性要求的成人口罩。

（299）国办：发布组织做好疫情防控重点物资生产企业复工复产和调度安排工作的紧急通知	国办发明电〔2020〕2号

各省、自治区、直辖市人民政府，国务院各部委、各直属机构：

为做好新型冠状病毒感染肺炎疫情防控重点物资生产企业的复工复产和调度安排工作，经国务院同意，现就有关事项紧急通知如下：

一、各省（区、市）人民政府要切实履行主体责任，迅速组织本地区生产应对疫情使用的医用防护服、N95口罩、医用护目镜、负压救护车、相关药品等企业复工复产。要做好生产人员、技术人员和相关设备、原辅料、资金等各方面保障工作，帮助企业及时解决生产经营中遇到的困难和问题，并根据需要及时扩大相关产品产能。

二、国务院应对新型冠状病毒感染肺炎疫情联防联控机制物资保障组负责对上述重点医疗应急防控物资实施统一管理、统一调拨，地方各级人民政府不得以任何名义截留、调用。物资保障组将向重点企业选派驻企特派员，负责监督物资的统一调拨，帮助企业及时反映困难和问题，配合有关部门抓好产品质量监管。

三、生产重点医疗应急防控物资的有关企业，要按照国务院应对新型冠状病毒感染肺炎疫情联防联控机制物资保障组要求，抓紧组织原材料采购和产品生产，及时完成生产任务，并加强产品质量管理，确保物资符合相关安全标准。有关企业要根据物资保障组要求，及时上报产能产量、产品库存等数据。

四、为确保做好重点医疗应急防控物资的及时生产、调拨、运输和配用等方面协调工作，建立有关工作衔接机制，确保24小时联络畅通。各省级人民政府要确定一名厅（局）级负责同志牵头对接联系物资调拨工作。

各地区、各部门及有关企业要统一思想，提高站位，充分认识做好重点地区应急防控物资供应工作的重要性，切实增强紧迫感和责任感，按照本通知要求扎实做好各项工作。

国务院办公厅

2020年1月29日

（300）中央和国家机关工委通知强调：中央和国家机关各级基层党组织和党员、干部要在疫情防控斗争中充分发挥作用

1月28日，中央和国家机关工委发出通知，要求中央和国家机关各级基层党组织和党员、干部认真贯彻落实习近平总书记重要指示精神，在打赢疫情防控阻击战中充分发挥作用。

通知指出，新型冠状病毒感染的肺炎疫情发生后，党中央高度重视。习近平总书记主持召开中央政治局常委会会议并发表重要讲话，作出一系列重要指示，党中央印发了《关于加强党的领导、为打赢疫情防控阻击战提供坚强政治保证的通知》，为做好疫情防控工作提供了根本遵循。作为践行“两个维护”的第一方阵，中央和国家机关各级基层党组织和广大党员、干部必须迅速行动起来，带头贯彻落实，让党旗在疫情防控斗争中高高飘扬。

通知强调，各级基层党组织和广大党员、干部要深入学习习近平总书记重要讲话和一系列重要指示精神，从带头做到“两个维护”的政治高度深刻认识做好疫情防控工作的重要性、紧迫性，进一步增强政治责任感、使命感，把疫情防控作为当前最重要的工作来抓，以打赢疫情防控阻击战的实际行动，建设让党中央放心、让人民群众满意的模范机关。

通知强调，要勇于担当作为，教育引导广大党员、干部特别是党员领导干部牢记人民利益高于一切，不忘初心、牢记使命，坚守岗位、靠前指挥，切实加强党的领导，做到守土有责、守土担责、守土尽责，不折不扣地贯彻落实好党中央决策部署，在重大考验中践行初心使命、诠释对党忠诚。

通知强调，各级基层党组织和广大党员要走在前、作表率，在重大疫情防控斗争中，充分发挥战斗堡垒和先锋模范作用，确保每名党员都成为一面鲜红的旗帜，每个党支部都成为党旗高高飘扬的战斗堡垒。把机关及所属在京企事业单位的基层党组织和广大党员、干部全面动员起来，坚定站在疫情防控第一线，打头阵、作先锋，并广泛动员、组织、凝聚群众，全面落实防控措施，构筑群防群治的严密防线。

通知强调，要严肃纪律规矩，切实加强党员、干部教育管理，进一步强化政治机关意识，严肃党的政治纪律和政治规矩，确保党中央政令畅通、令行禁止。教育引导广大党员、干部保持政治定力，不信谣、不传谣，自觉同以习近平同志为核心的党中央保持高度一致。强化日常监督，对落实党中央决策部署不坚决不得力、不担当不作为甚至擅离职守、迟报漏报瞒报疫情以及造谣传谣的，依规依纪依法严肃处理。

通知强调，要关爱干部职工，密切关注干部职工思想动态，有针对性做好思想政治工作，确保干部职工思想稳定。全面贯彻“坚定信心、同舟共济、科学防治、精准施策”要求，认真做好疫情防控政策措施宣传解读，普及科学防护知识，提醒干部职工做好科学防护，真正把关心关爱干部职工的具体举措落到实处。要督促返京干部职工严格遵守北京市疫情防控有关规定，并及时提供必要的服务保障。

2020.1.18

（301）中共中央印发《关于加强党的领导、为打赢疫情防控阻击战提供坚强政治保证的通知》

近日，中共中央印发了《关于加强党的领导、为打赢疫情防控阻击战提供坚强政治保证的通知》。全文如下：

新型冠状病毒感染的肺炎疫情发生以来，习近平总书记高度重视，作出一系列重要指示，多次主持召开会议，对疫情防控工作进行研究部署，提出明确要求。1月27日，习近平总书记就各级党组织和广大党员、干部要在打赢疫情防控阻击战中发挥积极作用作出重要指示，强调各级党委（党组）、各级领导班子和领导干部、基层党组织和广大党员要不忘初心、牢记使命，挺身而出、英勇奋斗、扎实工作，团结带领广大人民群众坚定不移把党中央决策部署落到实处，坚决打赢疫情防控阻击战。各级党委（党组）要坚决贯彻落实习近平总书记重要指示精神，为打赢疫情防控阻击战提供坚强政治保证。现就有关事项通知如下。

疫情就是命令，防控就是责任。面对疫情加快蔓延的严重形势，各级党委（党组）要增强“四个意识”、坚定“四个自信”、做到“两个维护”，切实把思想和行动统一到习近平总书记重要指示精神上来，认清肩负的责任使命，按照坚定信心、同舟共济、科学防治、精准施策的要求切实做好工作，牢记人民利益高于一切，组织动员各级党组织和广大党员、干部把打赢疫情防控阻击战作为当前的重大政治任务，把投身防控疫情第一线作为践行初心使命、体现责任担当的试金石和磨刀石，把党的政治优势、组织优势、密切联系群众优势转化为疫情防控的强大政治优势，确保党中央重大决策部署贯彻落实，让党旗在防控疫情斗争第一线高高飘扬。

各级党委（党组）要激励引导广大党员、干部特别是领导干部在疫情防控斗争中挺身而出、英勇奋斗、扎实工作，经受住考验，切实做到守土有责、守土担责、守土尽责。要在疫情防控第一线考察、识别、评价、使用干部，把领导班子和领导干部在疫情防控斗争中的实际表现作为考察其政治素质、宗旨意识、全局观念、驾驭能力、担当精神的重要内容。对表现突出的，要表扬表彰、大胆使用；对不敢担当、作风飘浮、落实不力的，甚至弄虚作假、失职渎职的，要严肃问责。各级组织部门、纪检监察部门要在各级党委领导下，积极主动履职，有效发挥作用。

各级党委（党组）要充分发挥基层党组织战斗堡垒作用和共产党员先锋模范作用，把基层党组织和广大党员全面动员起来，发扬不畏艰险、无私奉献的精神，坚定站在疫情防控第一线，做到哪里任务险重哪里就有党组织坚强有力的工作、哪里就有党员当先锋作表率。要广泛组织基层党组织和党员落实联防联控措施，建立健全区县、街镇、城乡社区等防护网络，做好疫情监测、排查、预警、防控等工作，加强联防联控，严防死守、不留死角，构筑群防群治抵御疫情的严密防线。要坚持党建引领，把区域治理、部门治理、行业治理、基层治理、单位治理有机结合起来，切实提高疫情防控的科学性和有效性。机关、企事业单位以及社会组织党组织要按照统一安排，扎实做好本部门本单位本行业的预防和控制工作。要组织党员、干部做好群众工作，稳定情绪、增强信心，不信谣、不传谣，当好群众的贴心人和主心骨，紧紧依靠人民群众坚决打赢疫情防控阻击战。

各级党委（党组）要会同卫生健康等部门和单位，动员和选派专家和医护人员中的党员、干部勇挑重担、迎难而上，在医疗救护、科研攻关、基础预防等岗位发挥作用。要关心关爱奋战在疫情防控斗争一线的专家和医护人员，采取务实、贴心、到位的举措，帮助疫情防控斗争一线的专家和医护人员解决实际困难，解除后顾之忧。要及时总结宣传各级党组织和广大党员、干部在疫情防控斗争中涌现出的先进典型和感人事迹，凝聚起众志成城、全力以赴、共克时艰的强大正能量。

各级党委（党组）动员党组织和广大党员、干部在防控疫情斗争中发挥作用的情况，要及时报告党中央。

2020.1.28
