# Supplementary material for: Mining and quantitative evaluation of COVID-19 policy tools in China
Source: PLoS One. 2023 Apr 7;18(4):e0284143. doi: 10.1371/journal.pone.0284143 (PMC10081750; doi:10.1371/journal.pone.0284143)
Supplement: Attachment — Submitted filename: Response to Reviewers.docx [file pone.0284143.s003.docx]

Dear Reviewers,

Thank you very much for your comments and suggestions on the paper "**Mining and quantitative evaluation of COVID-19 policy tools in China**". I will reply to each reviewer's comments one by one.

Many thanks to reviewer 1 for pointing out detailed errors in the grammar and format of the article. I have corrected these errors. In addition, I have also improved the supplement of references. In response to your question "Is there Methodology section in this paper?" My answer is that there is no separate section in the paper to discuss the research method. The third section of the paper, "Construction of PMC-AE Index Model for COVID-19 Policy Quantification", introduces the PMC-AE method，and applied it directly.

Thanks to reviewer 2 for your recognition of the topic of the paper. I have corrected the problems you raised, especially the introduction and conclusion. I include a discussion section to discuss how China has used a mix of policy instruments, how this mix has changed over the course of the crisis, and to compare it with previous studies. The questions raised in the introduction section are answered. What's more, the theoretical and practical implications of the study and recommendations for future studies are added in the introduction and discussion sections, respectively.

Thanks to reviewer 3 for your comments, which were few but useful. With your suggestion, I have added the theoretical and practical significance to the introduction section, and added the limitations of the article and future research direction of the article in the newly added discussion section.

Finally, I would like to express my sincere thanks again. I hope that you can put forward valuable comments on my revised article again and look forward to your reply.

Sincerely,

Luming Cheng

School of Economics and Management, Tianjin Chengjian University, Tianjin, China

Chengluming@yeah.net
