# Supplementary material for: Mining and quantitative evaluation of COVID-19 policy tools in China
Source: PLoS One. 2023 Apr 7;18(4):e0284143. doi: 10.1371/journal.pone.0284143 (PMC10081750; doi:10.1371/journal.pone.0284143)
Supplement: Attachment — Submitted filename: Comments2.docx [file pone.0284143.s004.docx]

Comments

1. The Abstract introduces the concept of BMC-AE. Even though it is in the Abstract, what should be explained, what it means? One sentence should suffice!
2. The sentence “finally, the complicated intervention was carried out by issuing some specific and targeted epidemic prevention policies” in the Abstract should be its’ own sentence.
3. The word “Control” in the Introduction section should not be capitalized.
4. You said in the Introduction section that “Quantitative analysis of policy texts using policy tools is the mainstream policy analysis method at present, which has been widely used in e-commerce, agriculture, science and technology finance, pension, industrial Internet and other fields”. All references after that refer to studies done in China. Are there any international references that you could cite?
5. In the “1. Policy tool analysis” heading there should be a space after the period!
6. In Table 1 you could replace the “number” with “#” to save space.
7. You mention in the “1. Policy tool analysis” that text mining software was used. Which one?
8. In the section “Policy tools analysis” you said that “ should be eliminated”. I would recommend using an even stronger expression of “were eliminated” as this is what actually happened.
9. Please the exact expression “COVID-19” consistently throughout the paper.
10. In section “1.2 Policy release department analysis” the word “Joint” should not be capitalized.
11. In the section “1.2 Policy release department analysis”, should all the words in the expression “National health committee” be capitalized?
12. In Tables, I would recommend to left align the first column!
13. In the section “1.3 Policy tool analysis” the first sentence is confusing! Maybe it should be written as “…to clearly explore…”?
14. Should the column headings in the Tables be capitalized? Now, there is no consistency!
15. In Tables, should all the words in column 1 be capitalized? Same comment for the column headings!
16. After Table 3 to clearly explore you wrote “and taking effective measures to control and control the epidemic on the basis of understanding the development trend of the epidemic.” The use of the word “control” in the sentence causes confusion. Maybe you could rephrase it?
17. Before Table 4, you wrote, “Financially supported epidemic prevention tools are direct government assistance for small and medium-sized enterprises.” As you use the semicolon before, should the word “Financially” be de-capitalized?
18. In the next paragraph, you wrote “we divided policy tools into strategic layers, supply layers, demand layers and environmental layers”. Usually in academic papers, the use of words like “we”, and “I” should be avoided, and instead passive expression should be used consistently throughout the paper!
19. In section 2, you again introduce the expression PMC-AE. I think it would be a good idea to explain briefly what it in fact is!
20. The word “Secondly,” should be de-capitalized in the first paragraph of section 2.
21. Is there is Methodology section in this paper?
22. The words in the Tables are sometimes capitalized and sometimes not. There should be consistency!
23. In the last paragraph of section 2.1. you said “Compared with subjective expert scoring..” Should you rather say “Compared with subject expert scoring..”?
24. Should the figure headings include the complete word “Figure”?
25. In 2.3. you introduce the acronym Omnia Mobilis? There is no reference and there is no explanation. What is it?
26. In the same sentence you use the word “correlation”. Maybe a better word is relationship?
27. AE technology? Reference needed!
28. Figure 3 has been obviously copied from somewhere and therefore needs a reference. Also, it is better to draw the image again, as the copy is incomplete.
29. The first sentence after figure 3 needs a period at the end.
30. The argraph after Figure 3 needs references.
31. The footnote font in text after Table 7 is not consistent with the font in the rest of the text.
32. The images in Figure 4 are unclear.
33. In section 3.4. the sentence “Policies with scores between 9 and 10 are classified as grade Ⅲ policies with P4” needs a period before it.
34. In section 3.4. second paragraph what is “sasac”?
35. The sentence “The score of policy P1 is in the second place” should start a new paragraph!
36. In section 3.4. the sentence “The differences in the final scores of policy samples P2, P3 and P6 are not significant”. Are you referring to statistical significance? If not, maybe a different word should be used?
37. Alos, in the same sentence you use the word “they”. To what exactly are you referring to? The scores? If this is the case, it needs to be said specifically!
38. The sentence “Policy P8 "Giving Full play to tax Functions to Help Win the Battle of Epidemic Prevention and Control" ranks the third” needs to start a new paragraph!
39. Maybe the sentence “Tax payment has been deferred in accordance with the law..” can be written as “tax payment can be deferred in accordance with the law..”
40. What are smes? I think you mean SMEs, and if so, that is the specific acronym to be used.
41. The sentence “Policy, sample P7 COVID - 19 outbreak community prevention and control and the service work for..” needs to start a new paragraph.
42. The font used in the references section differs for the rest of the text.
