# Supplementary material for: Mining and quantitative evaluation of COVID-19 policy tools in China
Source: PLoS One. 2023 Apr 7;18(4):e0284143. doi: 10.1371/journal.pone.0284143 (PMC10081750; doi:10.1371/journal.pone.0284143)
Supplement: Attachment — Submitted filename: Response to Reviewers.docx [file pone.0284143.s005.docx]

Dear Reviewers,

Thank you very much for your comments and suggestions on the paper "**Mining and quantitative evaluation of COVID-19 policy tools in China**". I will reply to each reviewer's comments one by one.

Many thanks to reviewer 1 for pointing out detailed errors in format of tables. I have modified the tables. In addition, I added the methodology section and introduced the expression PMC-AE. With your comments in mind, the summary has also been revised.

Many thanks to reviewer 4 for your valuable suggestions. The manuscript has been refined for English grammatical structure and phraseology. The abstract has been reworked to highlight the main findings and conclusion. According to your suggestions, I have readjusted the structure of the article and clearly explained the data collection process, methodology and results, which makes the article more clearly expressed. The discussion section adds more references and also contains some comparisons of the results with the available evidence, which includes how China has used a mix of policy instruments, how this mix has changed over the course of the crisis, and to compare it with previous studies. Because the theoretical contributions and practical implications of the research have already been mentioned in the introduction section, they are not mentioned again in the discussion section. The conclusion section is moved after the discussion of the study.

Finally, I would like to express my sincere thanks again. I hope that you can put forward valuable comments on my revised article again and look forward to your reply.

Sincerely,

Luming Cheng

School of Economics and Management, Tianjin Chengjian University, Tianjin, China

chengluming@yeah.net
